# Author Correction: Genetics of circulating inflammatory proteins identifies drivers of immune-mediated disease risk and therapeutic targets

**DOI:** 10.1038/s41590-023-01635-6

**Published:** 2023-09-07

**Authors:** Jing Hua Zhao, David Stacey, Niclas Eriksson, Erin Macdonald-Dunlop, Åsa K. Hedman, Anette Kalnapenkis, Stefan Enroth, Domenico Cozzetto, Jonathan Digby-Bell, Jonathan Marten, Lasse Folkersen, Christian Herder, Lina Jonsson, Sarah E. Bergen, Christian Gieger, Elise J. Needham, Praveen Surendran, Estonian Biobank Research Team, Andres Metspalu, Lili Milani, Reedik Mägi, Mari Nelis, Georgi Hudjašov, Dirk S. Paul, Ozren Polasek, Barbara Thorand, Harald Grallert, Michael Roden, Urmo Võsa, Tonu Esko, Caroline Hayward, Åsa Johansson, Ulf Gyllensten, Nick Powell, Oskar Hansson, Niklas Mattsson-Carlgren, Peter K. Joshi, John Danesh, Leonid Padyukov, Lars Klareskog, Mikael Landén, James F. Wilson, Agneta Siegbahn, Lars Wallentin, Anders Mälarstig, Adam S. Butterworth, James E. Peters

**Affiliations:** 1 British Heart Foundation Cardiovascular Epidemiology Unit, Department of Public Health and Primary Care, University of Cambridge, Cambridge, UK; 2 Victor Phillip Dahdaleh Heart and Lung Research Institute, University of Cambridge, Cambridge, UK; 3 Australian Centre for Precision Health, Unit of Clinical and Health Sciences, University of South Australia, Adelaide, South Australia Australia; 4 South Australian Health and Medical Research Institute, Adelaide, South Australia Australia; 5 Uppsala Clinical Research Center, Uppsala University, Uppsala, Sweden; 6 Centre for Global Health Research, Usher Institute, University of Edinburgh, Edinburgh, UK; 7 Department of Medical Epidemiology and Biostatistics, Karolinska Institutet, Stockholm, Sweden; 8 Development and Medical, Pfizer Worldwide Research, Stockholm, Sweden; 9 Estonian Genome Center, Institute of Genomics, University of Tartu, Tartu, Estonia; 10 Department of Immunology, Genetics, and Pathology, Biomedical Center, SciLifeLab Uppsala, Uppsala University, Uppsala, Sweden; 11 Department of Metabolism, Digestion and Reproduction, Faculty of Medicine, Imperial College London, London, UK; 12 School of Immunology and Microbial Sciences, King’s College London, London, UK; 13 Nucleus Genomics ltd, New York, NY USA; 14 Institute for Clinical Diabetology, German Diabetes Center, Düsseldorf, Germany; 15 Department of Endocrinology and Diabetology, Medical Faculty and University Hospital Düsseldorf, Heinrich Heine University Düsseldorf, Düsseldorf, Germany; 16 German Center for Diabetes Research, Munich-Neuherberg, Germany; 17 Institute of Neuroscience and Physiology, University of Gothenburg, Gothenburg, Sweden; 18 Institute of Epidemiology, Helmholtz Zentrum München, German Research Center for Environmental Health, Neuherberg, Germany; 19 Research Unit of Molecular Epidemiology, Helmholtz Zentrum München, German Research Center for Environmental Health, Neuherberg, Germany; 20 British Heart Foundation Centre of Research Excellence, School of Clinical Medicine, Addenbrooke’s Hospital, University of Cambridge, Cambridge, UK; 21 Health Data Research UK, Wellcome Genome Campus and University of Cambridge, Hinxton, UK; 22 Centre for Genomics Research, Discovery Sciences, BioPharmaceuticals R&D, AstraZeneca, Cambridge, UK; 23 Medical School, University of Split, Split, Croatia; 24 MRC Human Genetics Unit, Institute of Genetics and Cancer, University of Edinburgh, Western General Hospital, Edinburgh, UK; 25 Clinical Memory Research Unit, Department of Clinical Sciences Malmö, Lund University, Lund, Sweden; 26 Skåne University Hospital, Malmö, Sweden; 27 Wallenberg Centre for Molecular Medicine, Lund University, Lund, Sweden; 28 Clinical Memory Research Unit, Faculty of Medicine, Lund University, Lund, Sweden; 29 Department of Neurology, Skåne University Hospital, Lund University, Lund, Sweden; 30 NIHR Blood and Transplant Research Unit in Donor Health and Behaviour, University of Cambridge, Cambridge, UK; 31 Department of Human Genetics, Wellcome Sanger Institute, Hinxton, UK; 32 Division of Rheumatology, Department of Medicine (Solna), Karolinska Institutet and Karolinska University Hospital, Stockholm, Sweden; 33 Center for Molecular Medicine, Karolinska Institutet, Stockholm, Sweden; 34 Department of Medical Sciences and Uppsala Clinical Research Center, Uppsala University, Uppsala, Sweden; 35 Department of Immunology and Inflammation, Imperial College London, London, UK

**Keywords:** Autoimmune diseases, Immunogenetics, Protein array analysis, Genotyping and haplotyping, Gene expression analysis

Correction to: *Nature Immunology* 10.1038/s41590-023-01588-w, published online 10 August 2023.

In the version of the article originally published, the middle panel in Fig. 3b was mistakenly a duplicate of the upper panel. The figure has now been corrected in the HTML and PDF versions of the article.

