# Supplementary material for: Genetics of circulating inflammatory proteins identifies drivers of immune-mediated disease risk and therapeutic targets
Source: Nat Immunol. 2023 Aug 10;24(9):1540–51. doi: 10.1038/s41590-023-01588-w (PMC10457199; doi:10.1038/s41590-023-01588-w)

nature immunology

Article https://doi.org/10.1038/s41590-023-01588-w

Genetics of circulating inflammatory proteins identifies drivers of immune-mediated disease risk and therapeutic targets

In the format provided by the authors and unedited

nature immunology

Article https://doi.org/10.1038/s41590-023-01588-w

Genetics of circulating inflammatory proteins identifies drivers of immune-mediated disease risk and therapeutic targets

In the format provided by the authors and unedited

Supplementary Material - Table of contents

i). Supplementary Notes

- Supplementary Note 1: Cohort details

- Supplementary Note 2: Comparison with pQTL study of Ferkingstad *et al.*

- Supplementary Note 3: Additional information on potential mechanisms underpinning *cis*-pQTLs

- Supplementary Note 4: TNFSF10 pQTL protein-protein network analysis

- Supplementary References

ii). Supplementary Figure and Item Titles

iii). Supplementary Figures

Supplementary Notes

Supplementary Note 1: Cohort details

INTERVAL. The INTERVAL study[1] is a prospective cohort study of blood donors. Volunteers were recruited and consented between 2012 and 2014 from 25 NHSBT (National Health Service Blood and Transplant) static donor centres across England. The INTERVAL study was approved by the Cambridge (East) Research Ethics Committee. A subset of 4,896 participants from INTERVAL with Olink inflammation panel data contributed to the present study. 2,907 were male and 1,989 female. These participants had an age range between 49 and 78 years (median 61, IQR=55-66). A complete list of the investigators and contributors to the INTERVAL trial is provided in reference[2].

Stabilization of Atherosclerotic Plaque by Initiation of Darapladib Therapy Trial (STABILITY). STABILITY is a randomized, controlled trial conducted in 15,828 patients with chronic coronary heart disease (CHD) comparing darapladib or placebo, in addition to standard of care (https://clinicaltrials.gov/ct2/show/NCT00799903)[3]. Data from 2,951 individuals with Olink inflammation panel and genotyping data contributed to this pQTL study. All participants gave written informed consent to the biomarker and genetic sub-studies and the study was approved by the National Ethics Committees in the participating countries.

Biomarkers For Identifying Neurodegenerative Disorders Early and Reliably (BioFINDER). The BioFINDER study[4] is run by the Clinical Memory Research Unit and The Biomedical center, at Lund University, Sweden. Participants were recruited from the Memory and Neurology clinics at Skåne University Hospital and the Memory Clinic at Ängelholm's Hospital. Study participants comprised 1,496 individuals with mild cognitive symptoms, dementia or parkinsonian symptoms, and cognitively healthy elderly. The Ethics Committee of Lund University approved this study. All study participants gave written consent for participation in the study, which was performed in accordance with the Helsinki Declaration.

Cooperative Health Research in the Region of Augsburg (KORA). KORA is a series of independent population-based studies from the general population living in the region of Augsburg, Southern Germany. The KORA F4 study was conducted from 2006-2008 as a follow-up study to KORA S4 (1999-2001)[5]. 1,064 participants from KORA F4 contributed data to this study.

Orkney Complex Disease Study (ORCADES). The ORCADES is a family-based study in the isolated Scottish archipelago of Orkney, part of the Viking Genes studies (ed.ac.uk/viking). A total of 981 individuals with Olink inflammation panel data contributed to this pQTL study. All participants gave written informed consent and the study was approved by Research Ethics Committees in Orkney, Aberdeen (North of Scotland REC), and South East Scotland REC, NHS Lothian (reference: 12/SS/0151).

Northern Sweden Population Health Study (NSPHS). NSPHS is a cross-sectional study conducted in the communities of Karesuando (samples gathered in 2006) and Soppero (2009) in the subarctic region of the County of Norrbotten, Sweden[6]. Ethical approval was given by the local ethics committee at the University of Uppsala (Regionala Etikprövningsnämnden, Uppsala, Dnr 2005:325) in compliance with the Declaration of Helsinki. All participants gave

their written informed consent to the study. A total of 866 individuals with Olink inflammation panel data contributed to the present study.

VIS. CROATIA-Vis is a family-based, cross-sectional study in the island of Vis, Croatia. 899 participants with Olink Inflammation panel and genotyping data contributed to this pQTL study. All participants gave written informed consent and the study was approved by Research Ethics Committees in Croatia (Institutional Ethics Committee of the University of Split School of Medicine [protocol code 2181-198-03-04/10-11-0008] and Scotland).

STANLEY (SWEBIC). The SWEBIC cohort comprises participants meeting the diagnostic criteria for Bipolar disorder as per the DSM-IV recruited at Swedish sites in affiliation with the Stanley Medical Research Institute. In total, SWEBIC contributed 644 samples (cohort A [swe6]: 300, cohort B [lahl]: 344) with Olink inflammation panel data to this pQTL study. Ethical approval for SWEBIC was granted by the Regional Ethical Review Board in Stockholm, Sweden (2008/2009-31/2).

Estonian Biobank (EstBB). The EstBB cohort is a volunteer-based sample of the Estonian resident adult population (aged ≥ 18 years)[7]. A total of 487 participants contributed to this study. Ethical approval was given by the Estonian Committee on Bioethics and Human Research (1.1-12/624) and by the Research Ethics Committee of the University of Tartu (application number 262/T-3, October 2016), data extraction nr K29.

RECOMBINE/EIRA. The RECOMBINE/EIRA biobank was generated from participants with active rheumatoid arthritis recruited between February 2011 and May 2013 at the Karolinska University Hospital, Stockholm, Sweden as part of the Epidemiologic Investigation of Rheumatoid Arthritis (EIRA) study[8]. The study was approved by the Ethical Review Board at Karolinska Institutet, Stockholm, Sweden (reference numbers 2007-0889-31, 2007-1443-32 and 2015/1844-31/2). All participants gave written informed consent. At the time of recruitment, all participants were undergoing treatment initiation or escalation. 540 patients with Olink inflammation panel data contributed to this pQTL study.

ARISTOTLE. The Apixaban for reduction in stroke and other ThromboemboLic events in atrial fibrillation (ARISTOTLE) trial was designed to investigate the safety and efficacy of the Factor Xa (FXa) inhibitor apixaban for secondary prevention of stroke in atrial fibrillation patients. Full details of the ARISTOTLE cohort are available at[9,10]. In total, ARISTOTLE contributed 1,585 samples with Olink inflammation panel data for comparison with the pQTL discovery meta-analysis findings. All participants gave written informed consent to the biomarker and genetic sub-studies and the study was approved by the National Ethics Committees in the participating countries.

Supplementary Note 2: Comparison with pQTL study of Ferkingstad *et al.*

In addition to testing the pQTLs from our discovery meta-analysis for replication in the ARISTOTLE study, we also compared our results to those from the study by Ferkingstad *et al*[11] from the deCODE group, hereafter referred to as the “deCODE study”. The deCODE study performed pQTL mapping in approximately 35,000 individuals from Iceland, with 4,907 proteins measured using the aptamer-based SomaScan V4 platform (whereas we used the

Olink Inflammation platform in our study).

72 of the 91 proteins in our study were measured in the deCODE study. Of the 180 significant locus-protein associations from our study, 158 were testable for replication in the deCODE summary statistics. Of these 158 variants, 75 were significant at $P<5x10^{-10}$, 78 at $P<5x10^{-8}$, and 96 at a more liberal *P*-value threshold of $2.8x10^{-4}$ (a Bonferroni-correction for the 180 pQTLs eligible for replication testing; *P*-values from linear regression). The numbers of pQTLs replicating in ARISTOTLE and in deCODE at varying *P*-value thresholds are shown in **Supplementary Note Table 1**. Overall, we replicated 126 (71%) of the 178 testable pQTLs in either ARISTOTLE or deCODE at $P<2.8x10^{-4}$.

Supplementary Note Table 1. The numbers of pQTLs replicating in ARISTOTLE and in deCODE at varying *P*-value thresholds (*P*-values from linear regression).

***P*-value threshold**	**No. pQTLs replicated in ARISTOTLE**	**No. pQTLs replicated in deCODE**	**No. pQTLs replicated in either ARISTOTLE or deCODE**
$5.0x10^{-10}$	32	75	87
$5.0x10^{-8}$	39	78	92
$2.78x10^{-4}$	72	96	126
**Number of testable pQTLs**	**174**	**158**	**178**

Of note, 7 pQTLs replicated in ARISTOTLE with $P<5x10^{-8}$ and yet did not replicate in the deCODE study despite the larger sample size of the latter (**Supplementary Note Table 2**). 4 of these 7 were *cis*-pQTLs. Of the three *trans*-pQTLs, two have been reported in previous studies that use single protein ELISAs; i) the association of rs385076 with IL-18 levels was reported by Johansson *et al*[12], and ii) rs2228145, a variant in *IL6R* (the gene encoding the interleukin-6 receptor), is well known to affect soluble IL6 levels[13,14].

The lack of signal in the deCODE data (generated using the Somascan platform), despite convincing evidence of a pQTL in these instances (in both Olink and ELISA-based studies), may reflect platform effects. Consistent with this, rs2228145 was not significantly associated with plasma IL6 levels in the study of Sun *et al*[15], which used an earlier version of the SomaScan platform. There are a number of possible explanations for the absence of detecting a pQTL using the SomaScan: i) the specific aptamer may lack sensitivity; ii) aptamers do not always measure their canonical target protein, and instead may target a cleavage product or the protein bound in complex to another protein; iii) aptamers are more vulnerable to off-target binding than the Olink platform used in our study. This is because the Olink platform uses a pair of antibodies, each with a complementary oligonucleotide tag, for each protein target. If,

and only if, both antibodies bind then the oligonucleotides hybridise and a qPCR read-out is generated.

Supplementary Note Table 2. Details of pQTLs which replicated at $P<5x10^{-8}$ in ARISTOTLE (n=1,585, Olink proteomics) but not in deCODE (n=35,556, SomaScan proteomics). *P*-values from linear regression.

**Protein**	**rsID**	**P-value in ARISTOTLE**	**P-value in deCODE**	***Cis* or *trans***
LTA	rs2229092	$5.8x10^{-81}$	0.56	*cis*
CCL3	rs8951	$2.4\ x10^{-25}$	0.0087	*cis*
GDNF	rs62360376	$1.6x10^{-13}$	0.48	*cis*
CCL13	rs3136676	$3.1x10^{-10}$	0.15	*cis*
IL18	rs385076	$5.1x10^{-17}$	0.42	*trans*
TNFSF10	rs28929474	$1.6x10^{-9}$	0.78	*trans*
IL6	rs2228145	$3.0x10^{-8}$	0.0022	*trans*

Supplementary Note 3: Additional information on potential mechanisms underpinning *cis*-pQTLs

Supplementary Note Table 3 provides information on whether the sentinel *cis*-pQTL variant was a protein-altering variant (PAV), or whether it was in high LD (r^2≥0.8) with a PAV, and whether there was a colocalising eQTL in at least one tissue or cell type.

Of the 11 *cis*-pQTLs with sentinel variants that are PAVs, only 2 had a corresponding colocalising (PP H4 >0.8) *cis*-eQTL. These were pQTLs for IL15RA and PLAU. The remaining 9 did not (*cis*-pQTLs for ADA, CCL8, CCL25, CXCL10, HGF, LTA, MMP10, SIRT2, TGFB1).

For a further 10 *cis*-pQTLs, the sentinel variants were not themselves PAVs but were in high LD (r^2≥0.8) with a PAV. However, 3 of these PAVs were not in the gene encoding the target protein but were instead in another nearby gene, and thus protein-altering effects could not account for the pQTL. Of the remaining 7 *cis*-pQTLs, 4 have a colocalising corresponding *cis*-eQTL (CD6, CCL11, CST5, CXCL5), whereas 3 do not (TNFSF14, CCL4, CASP8).

There were 27 *cis*-pQTLs without a corresponding colocalising *cis*-eQTL. 12 of the 27 *cis*-pQTLs without a colocalising eQTL are potentially explaining by PAVs, leaving 15 unexplained

by current available eQTL data.

Supplementary Note Table 3. Annotation of *cis*-pQTLs. The target protein is denoted by the non-italicised gene symbol of the encoding gene. PAV = protein-altering variant. LD = linkage disequilibrium. PP H4 = posterior probability of hypothesis 4 from coloc.

**Sentinel pQTL variant**	**Target protein**	**Sentinel pQTL is a PAV**	**Sentinel pQTL is in high LD (r^2≥0.8) with a PAV**	**Colocalising eQTL in at least 1 tissue (PP H4 ≥0.8)**
rs11555566	ADA	**Yes**	N/A	No
rs6328	NGF	No	No	**Yes**
rs56328050	CASP8	No	**Yes**	No
rs79722574	CCL11	No	**Yes**	**Yes**
rs11574915	CCL19	No	Yes but in a different gene to that encoding target protein (*FAM205A*)	No
rs10207134	CCL20	No	No	**Yes**
rs712048	CCL23	No	No	No
rs2032887	CCL25	**Yes**	N/A	No
rs8064426	CCL4	No	**Yes**	No
rs11265493	CD244	No	No	**Yes**
rs1883832	CD40	No	No	**Yes**
rs674379	CD5	No	No	**Yes**
rs2074227	CD6	No	**Yes**	**Yes**
rs2276862	CDCP1	No	No	**Yes**
rs17610659	CSF1	No	No	**Yes**
rs4815244	CST5	No	**Yes**	**Yes**
rs671623	CX3CL1	No	No	**Yes**
rs1366949	CXCL1	No	No	**Yes**
rs11548618	CXCL10	**Yes**	N/A	No
rs6827617	CXCL11	No	Yes but in a different gene to that encoding target protein	No

			(*SDAD1*)	
rs450373	CXCL5	No	**Yes**	**Yes**
rs16850073	CXCL6	No	No	**Yes**
rs4241577	CXCL9	No	Yes but in a different gene to that encoding target protein (*NAA*)	**Yes**
rs62193248	DNER	No	No	**Yes**
rs3014874	S100A12	No	No	**Yes**
rs838131	FGF21	No	No	No
rs12509595	FGF5	No	No	**Yes**
rs62360376	GDNF	No	No	No
rs5745687	HGF	**Yes**	N/A	No
rs12123181	IL10	No	No	No
rs2266590	IL10RB	No	No	**Yes**
rs10076557	IL12B	No	No	No
rs2228059	IL15RA	**Yes**	No	**Yes**
rs17700884	IL17C	No	No	No
rs5744249	IL18	No	No	**Yes**
rs2270297	IL18R1	No	No	**Yes**
rs112359206	IL7	No	No	**Yes**
rs6446951	IL8	No	No	No
rs1800472	TGFB1	**Yes**	N/A	No
rs1133763	CCL8	**Yes**	N/A	No
rs7213460	CCL7	No	No	No
rs3136676	CCL13	No	No	No
rs8951	CCL3	No	No	**Yes**
rs471994	MMP1	No	No	**Yes**
rs17860955	MMP10	**Yes**	N/A	No
rs2247769	TNFRSF11B	No	No	No
rs822335	CD274	No	No	**Yes**

rs144373891	SIRT2	**Yes**	N/A	No
rs60094514	SLAMF1	No	No	No
rs149278	SULT1A1	No	No	**Yes**
rs72912115	TGFA	No	No	**Yes**
rs2229092	LTA	**Yes**	N/A	No
rs1776354	TNFRSF9	No	No	**Yes**
rs344562	TNFSF14	No	**Yes**	No
rs574044675	TNFSF10	No	No	No
rs4512994	TNFSF11	No	No	**Yes**
rs34790908	TNFSF12	No	No	**Yes**
rs55744193	PLAU	**Yes**	No	**Yes**
rs6921438	VEGFA	No	No	No

Supplementary Note 4: Protein-protein network analysis for TNFSF10

Plasma levels of TNFSF10 were associated with 7 genetic loci. To help identify the mediating genes at each of these loci, we constructed a protein-protein interaction network based on the candidate mediating genes at each loci and TNFSF10 itself using STRINGdb. We observed a cluster of eight interacting proteins (**Extended Data Figure 7a**), representing proteins encoded by 5 of the 7 loci with pQTLs for TNFSF10. The genes encoding these proteins highlight the plasminogen-activating system as a common feature. The relation of the trans-pQTLs for TNFSF10 and these are as follows: (i) rs4760 is a missense variant in *PLAUR*, which encodes the plasminogen-activator urokinase receptor; (ii) rs5030044 is intronic in *KNG1*, encoding kininogen 1 which is involved in bradykinin formation, which is regulated by plasmin; (iii) rs28929474 is a missense variant in *SERPINA1*, which encodes alpha1-antitrypsin, an anti-protease which can bind plasmin; (iv) rs8178824 is intronic to *APOH*, which encodes a cofactor for plasminogen activation[16,17]; and (v) rs654488 is upstream of *MEP1B*, encoding meprin A, which is activated by the plasmin[18]. Together, these findings highlight the most likely mediating genes at 5 of the 7 regulatory loci and links TNFSF10 to the plasminogen-activating system.

Supplementary References

1. Astle, W.J. *et al.* The Allelic Landscape of Human Blood Cell Trait Variation and Links to Common Complex Disease. *Cell* **167**, 1415-1429 e1419 (2016).

2. Di Angelantonio, E. *et al.* Efficiency and safety of varying the frequency of whole blood donation (INTERVAL): a randomised trial of 45 000 donors. *Lancet* **390**, 2360-2371 (2017).

3. Wallentin, L. *et al.* Plasma proteins associated with cardiovascular death in patients with chronic coronary heart disease: A retrospective study. *PLoS Med* **18**, e1003513 (2021).

4. Hansson, O. *et al.* The genetic regulation of protein expression in cerebrospinal fluid. *EMBO Mol Med* **15**, e16359 (2023).

5. Herder, C. *et al.* A Systemic Inflammatory Signature Reflecting Cross Talk Between Innate and Adaptive Immunity Is Associated With Incident Polyneuropathy: KORA F4/FF4 Study. *Diabetes* **67**, 2434-2442 (2018).

6. Igl, W., Johansson, A. & Gyllensten, U. The Northern Swedish Population Health Study (NSPHS)--a paradigmatic study in a rural population combining community health and basic research. *Rural Remote Health* **10**, 1363 (2010).

7. Leitsalu, L. *et al.* Cohort Profile: Estonian Biobank of the Estonian Genome Center, University of Tartu. *Int J Epidemiol* **44**, 1137-1147 (2015).

8. Padyukov, L., Silva, C., Stolt, P., Alfredsson, L. & Klareskog, L. A gene-environment interaction between smoking and shared epitope genes in HLA-DR provides a high risk of seropositive rheumatoid arthritis. *Arthritis Rheum* **50**, 3085-3092 (2004).

9. Hijazi, Z. *et al.* Screening of Multiple Biomarkers Associated With Ischemic Stroke in Atrial Fibrillation. *J Am Heart Assoc* **9**, e018984 (2020).

10. Siegbahn, A. *et al.* Multiplex protein screening of biomarkers associated with major bleeding in patients with atrial fibrillation treated with oral anticoagulation. *J Thromb Haemost* **19**, 2726-2737 (2021).

11. Ferkingstad, E. *et al.* Large-scale integration of the plasma proteome with genetics and disease. *Nature Genetics* **53**, 1712-1721 (2021).

12. Johansson, Å. *et al.* NLRC4 Inflammasome Is an Important Regulator of Interleukin-18 Levels in Patients With Acute Coronary Syndromes: Genome-Wide Association Study in the PLATelet inhibition and patient Outcomes Trial (PLATO). *Circ Cardiovasc Genet* **8**, 498-506 (2015).

13. Sarwar, N. *et al.* Interleukin-6 receptor pathways in coronary heart disease: a collaborative meta-analysis of 82 studies. *Lancet* **379**, 1205-1213 (2012).

14. Swerdlow, D.I. *et al.* The interleukin-6 receptor as a target for prevention of coronary heart disease: a mendelian randomisation analysis. *Lancet* **379**, 1214-1224 (2012).

15. Sun, B.B. *et al.* Genomic atlas of the human plasma proteome. *Nature* **558**, 73-79 (2018).

16. Sakai, T., Balasubramanian, K., Maiti, S., Halder, J.B. & Schroit, A.J. Plasmin-cleaved beta-2-glycoprotein 1 is an inhibitor of angiogenesis. *Am J Pathol* **171**, 1659-1669 (2007).

17. Bu, C. *et al.* Beta2-glycoprotein i is a cofactor for tissue plasminogen activator-mediated plasminogen activation. *Arthritis Rheum* **60**, 559-568 (2009).

18. Rösmann, S. *et al.* Activation of human meprin-alpha in a cell culture model of colorectal cancer is triggered by the plasminogen-activating system. *J Biol Chem* **277**, 40650-40658 (2002).

Supplementary Figure Titles

Supplementary Figure 1. Manhattan plots for the 91 proteins.

Supplementary Figure 2. Forest plots and regional association plots for the 180 pQTLs.

Supplementary Figure 3. Regional association plots comparing plasma *cis*-pQTLs and blood *cis*-eQTLs for the gene encoding the corresponding protein.

Supplementary Figure 4. Regional association plots comparing plasma *cis*-pQTLs and disease associations.

Supplementary Item. **3-dimensional interactive genomic map of pQTLs**.
The html file (supplied separately) shows pQTL sentinel variant position in relation to the gene encoding the target protein and the strength of the statistical association (two-sided *P*-values are from meta-analysis of linear regression estimates). Hover over a point to see detailed information. The image can be rotated by holding at left clicking the mouse.

Supplementary Figure 1. Manhattan plots for the 91 proteins. Proteins are labelled with both the label provided by Olink, and with the encoding gene symbol in parentheses. Two-sided *P*-values are from meta-analysis of linear regression estimates. Horizontal dash blue line = P=5x10^{-10} (genome-wide significance after Bonferroni-correction for testing multiple proteins).

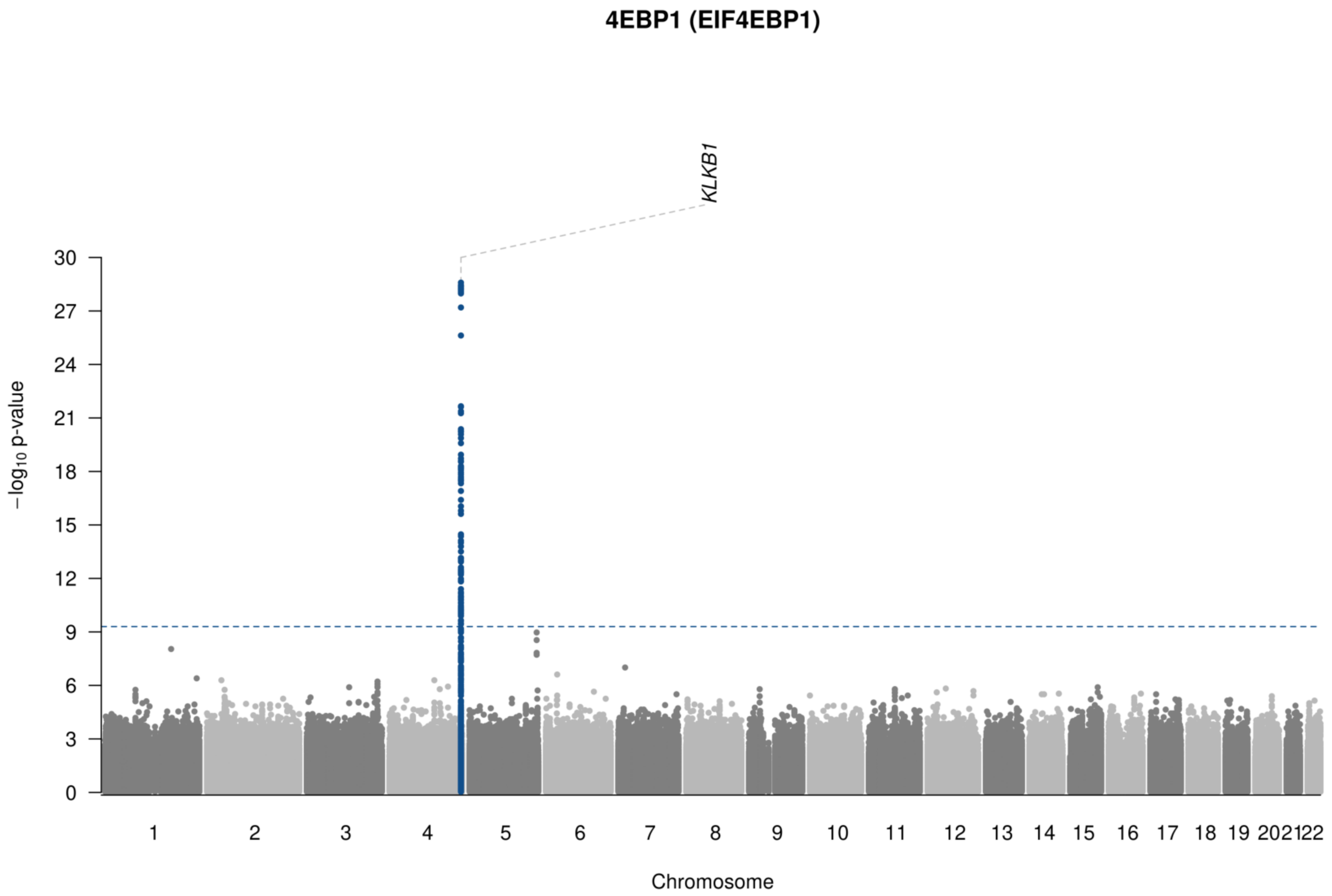
4EBP1 (EIF4EBP1)
KLKB1
30
27
24
21
18
15
12
9
6
3
0
−log10 p-value
1
2
3
4
5
6
7
8
9
10
11
12
13
14
15
16
17
18
19
202122
Chromosome

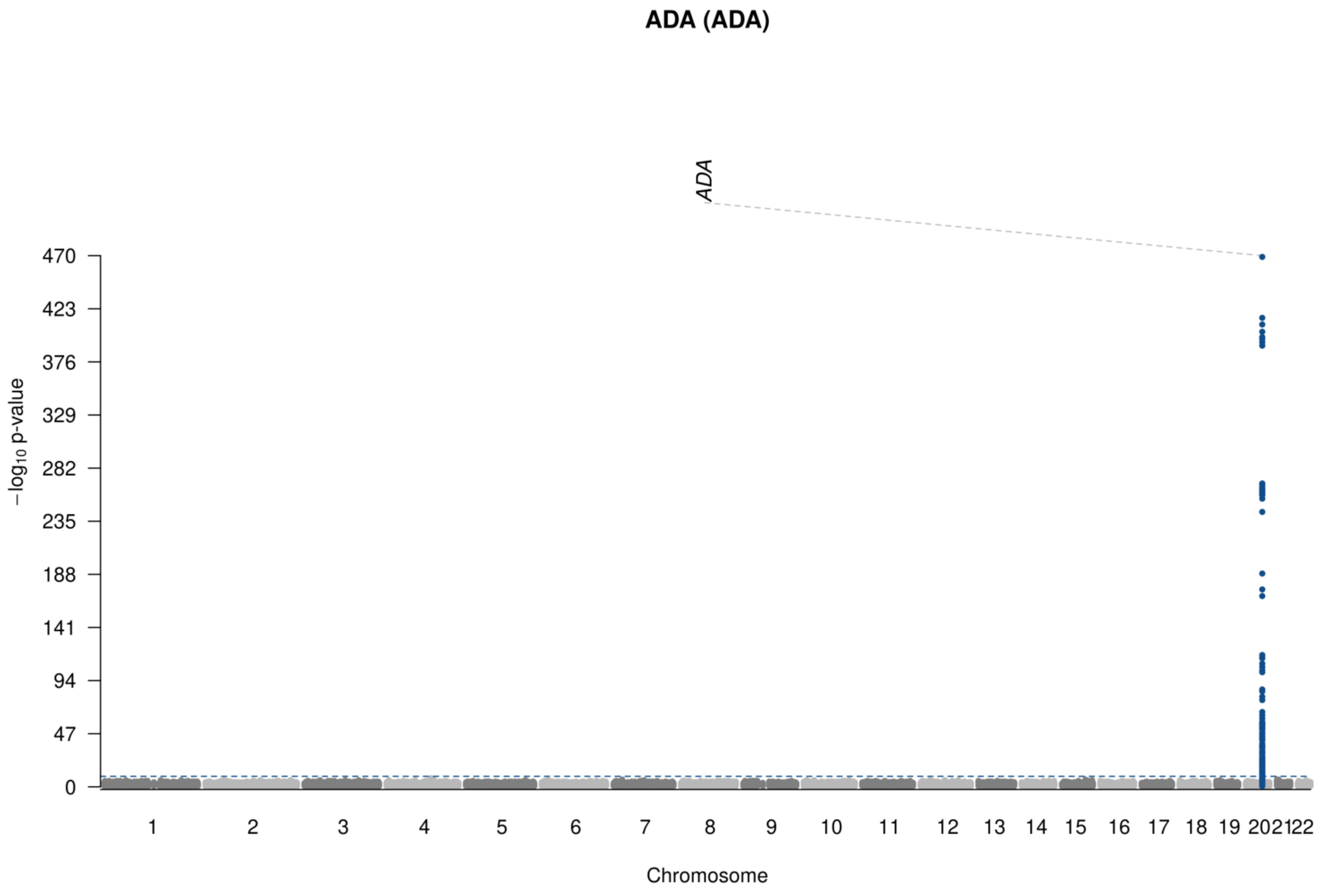
ADA (ADA)
ADA
470
423
376
329
282
235
188
141
94
47
0
−log10 p-value
1
2
3
4
5
6
7
8
9
10
11
12
13
14
15
16
17
18
19
20
21
22
Chromosome

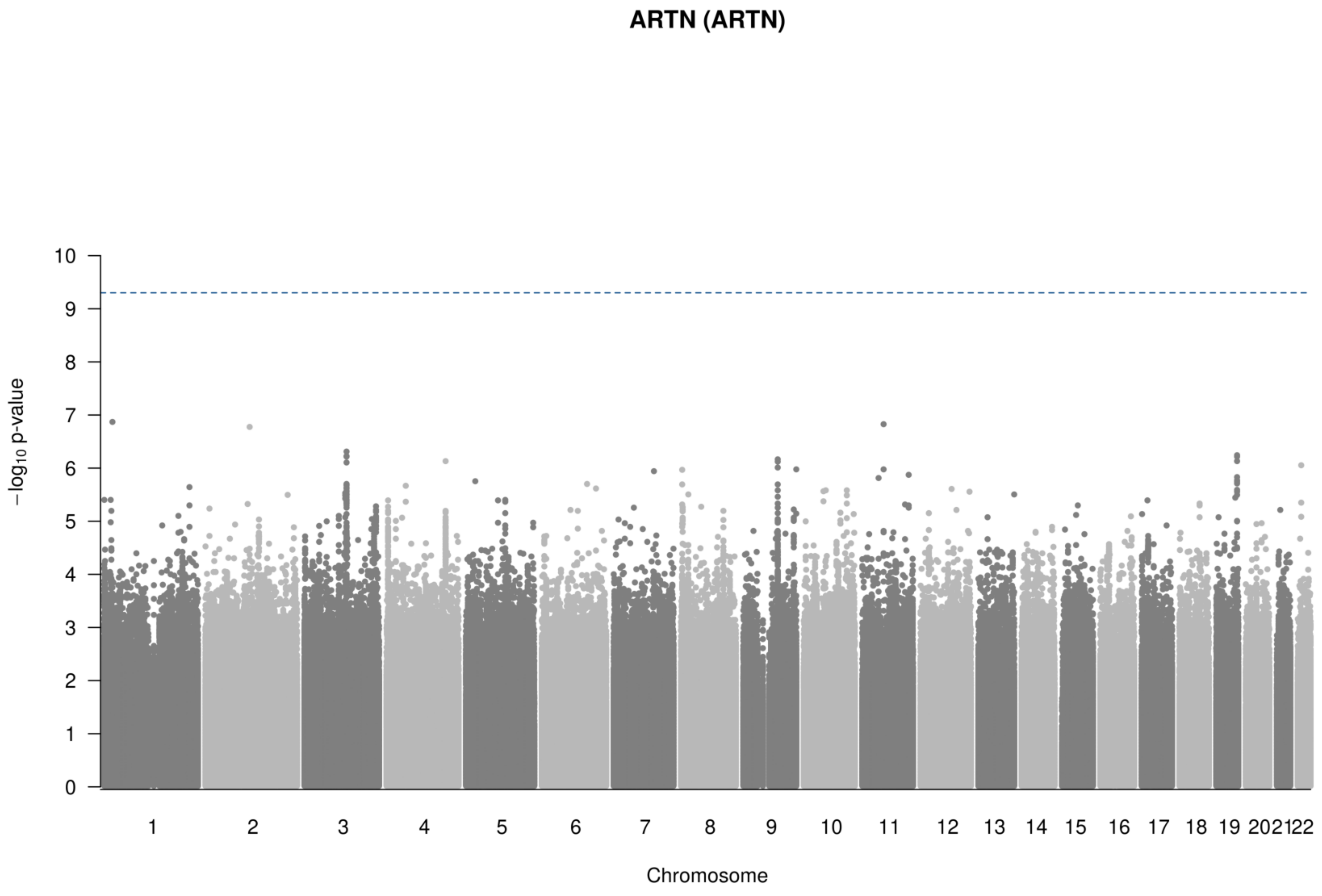
ARTN (ARTN)
−log10 p-value
0
1
2
3
4
5
6
7
8
9
10
1
2
3
4
5
6
7
8
9
10
11
12
13
14
15
16
17
18
19
20
21
22
Chromosome

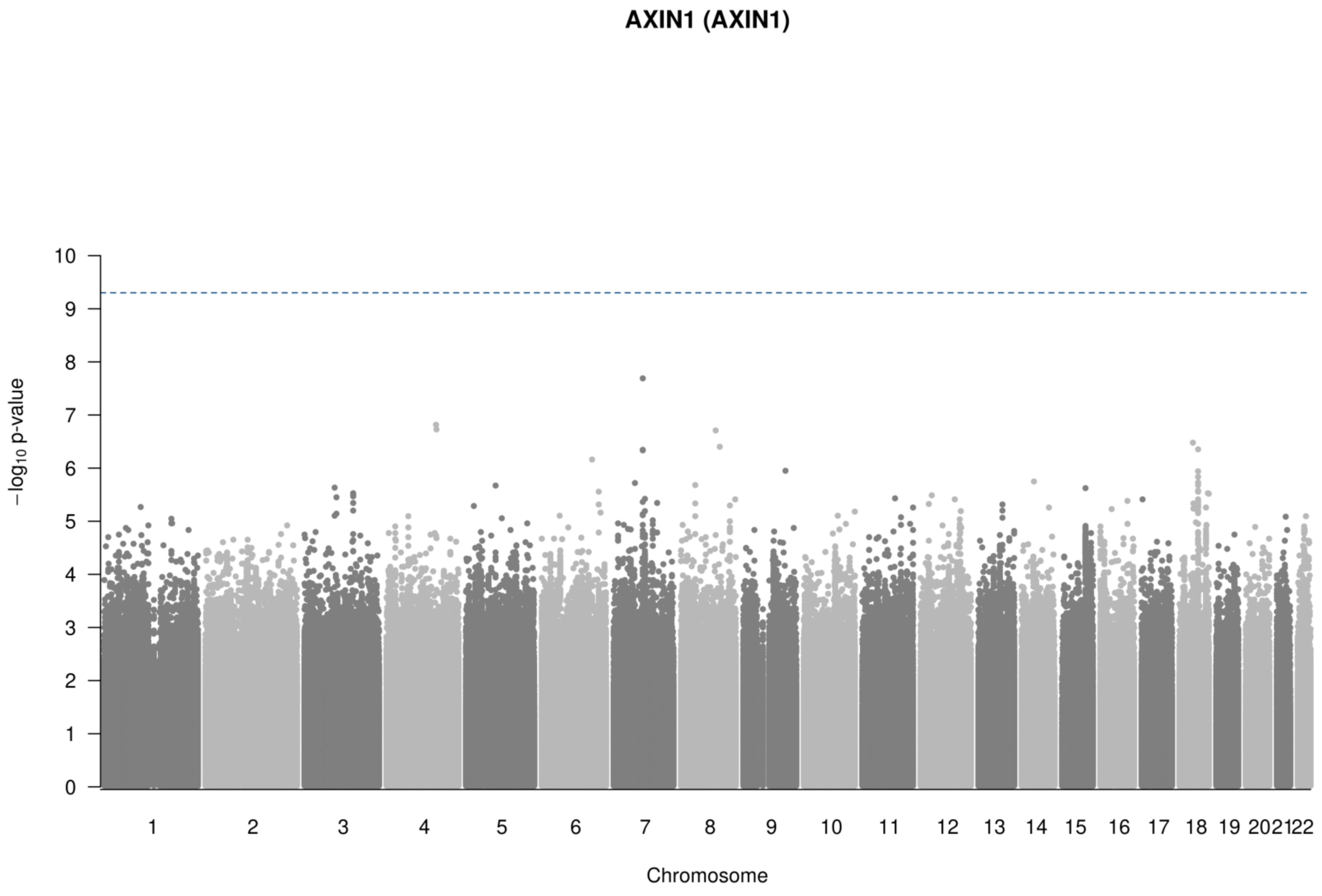
AXIN1 (AXIN1)
10
9
8
7
6
5
4
3
2
1
0
−log10 p-value
1
2
3
4
5
6
7
8
9
10
11
12
13
14
15
16
17
18
19
202122
Chromosome

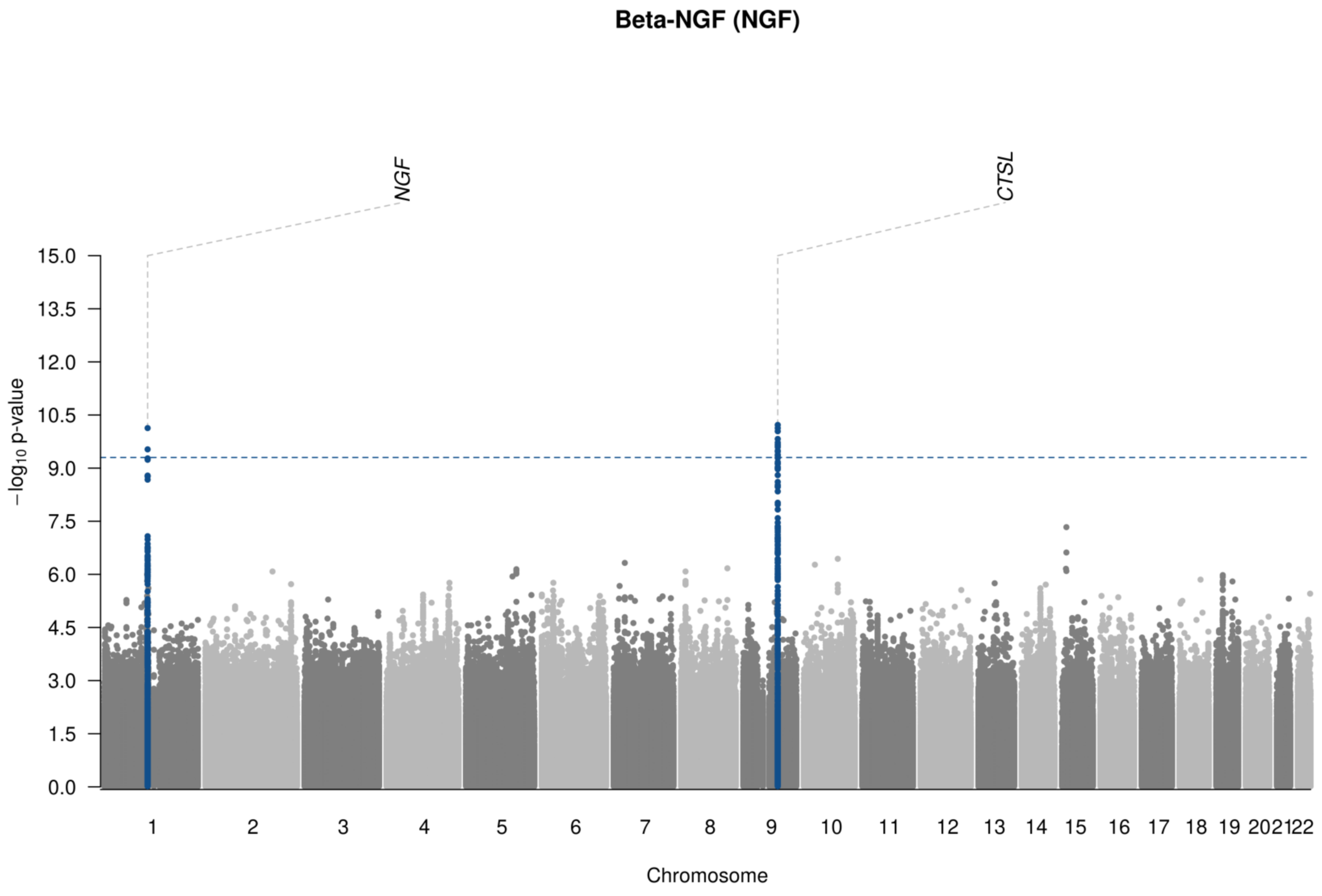

Beta-NGF (NGF)
NGF
CTSL
15.0
13.5
12.0
10.5
9.0
7.5
6.0
4.5
3.0
1.5
0.0
−log10 p-value
1
2
3
4
5
6
7
8
9
10
11
12
13
14
15
16
17
18
19
20
21
22
Chromosome

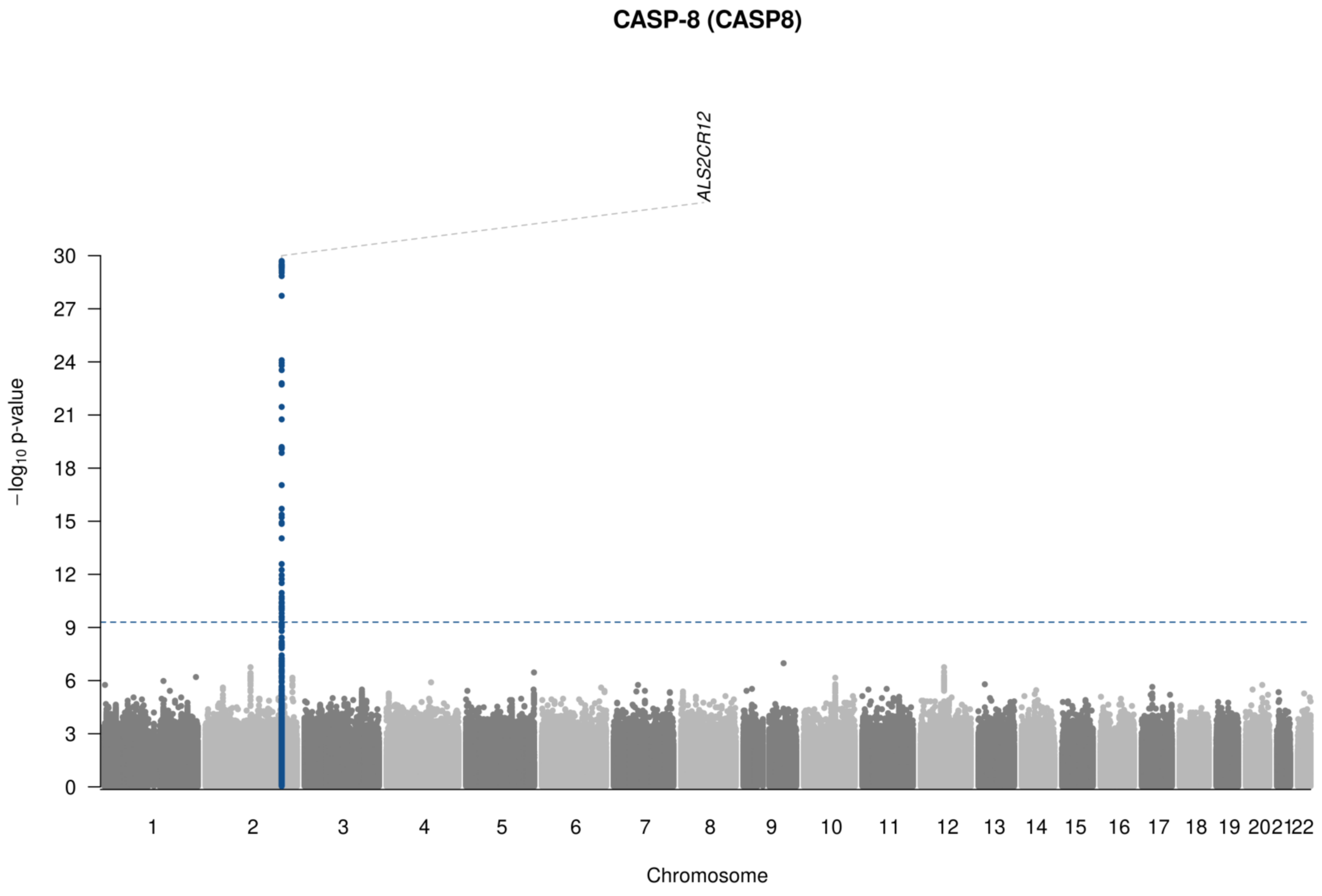

CASP-8 (CASP8)
ALS2CR12
−log10 p-value
0
3
6
9
12
15
18
21
24
27
30
1
2
3
4
5
6
7
8
9
10
11
12
13
14
15
16
17
18
19
20
21
22
Chromosome

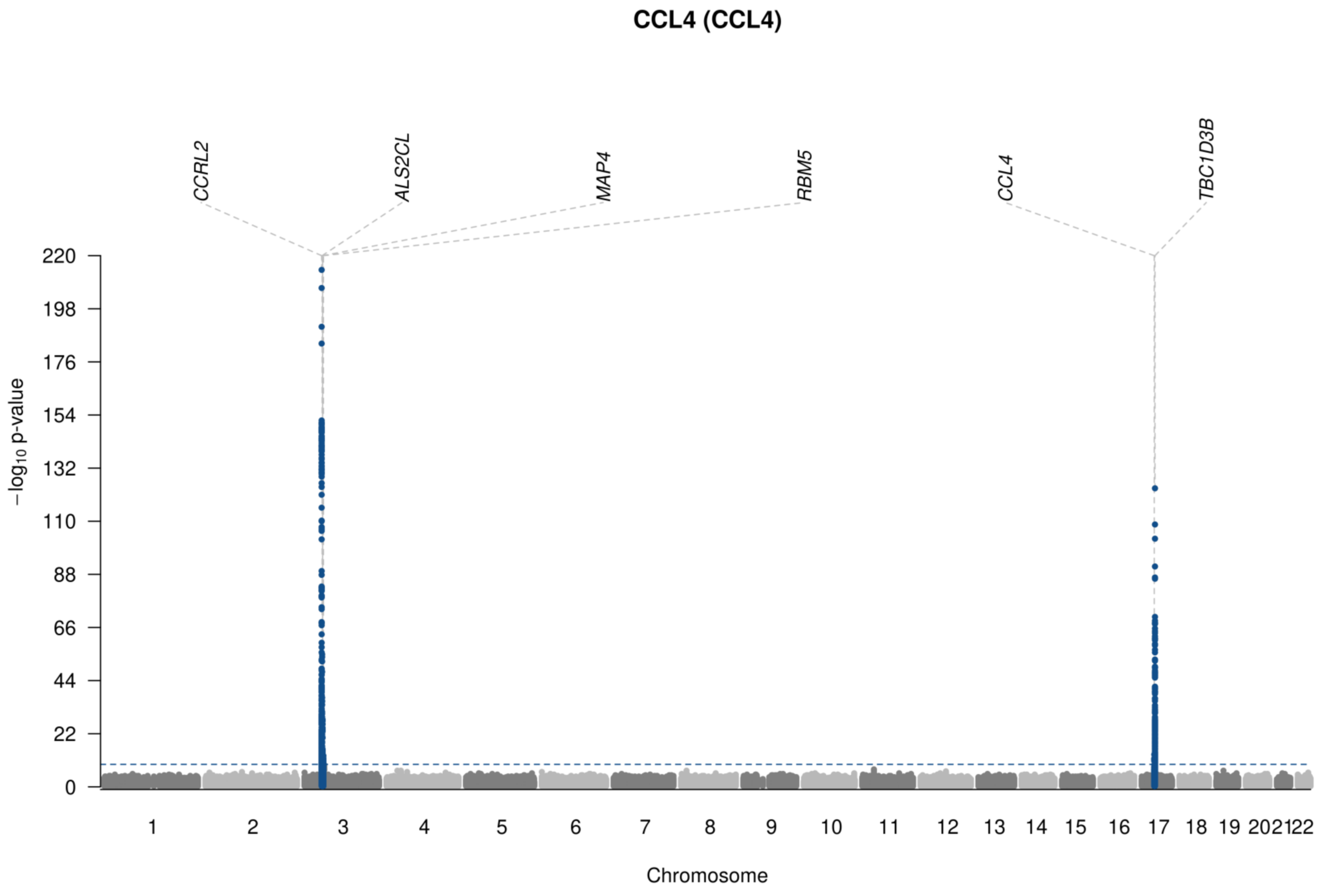

CCL4 (CCL4)
CCRL2
ALS2CL
MAP4
RBM5
CCL4
TBC1D3B
−log10 p-value
220
198
176
154
132
110
88
66
44
22
0
1
2
3
4
5
6
7
8
9
10
11
12
13
14
15
16
17
18
19
20
21
22
Chromosome

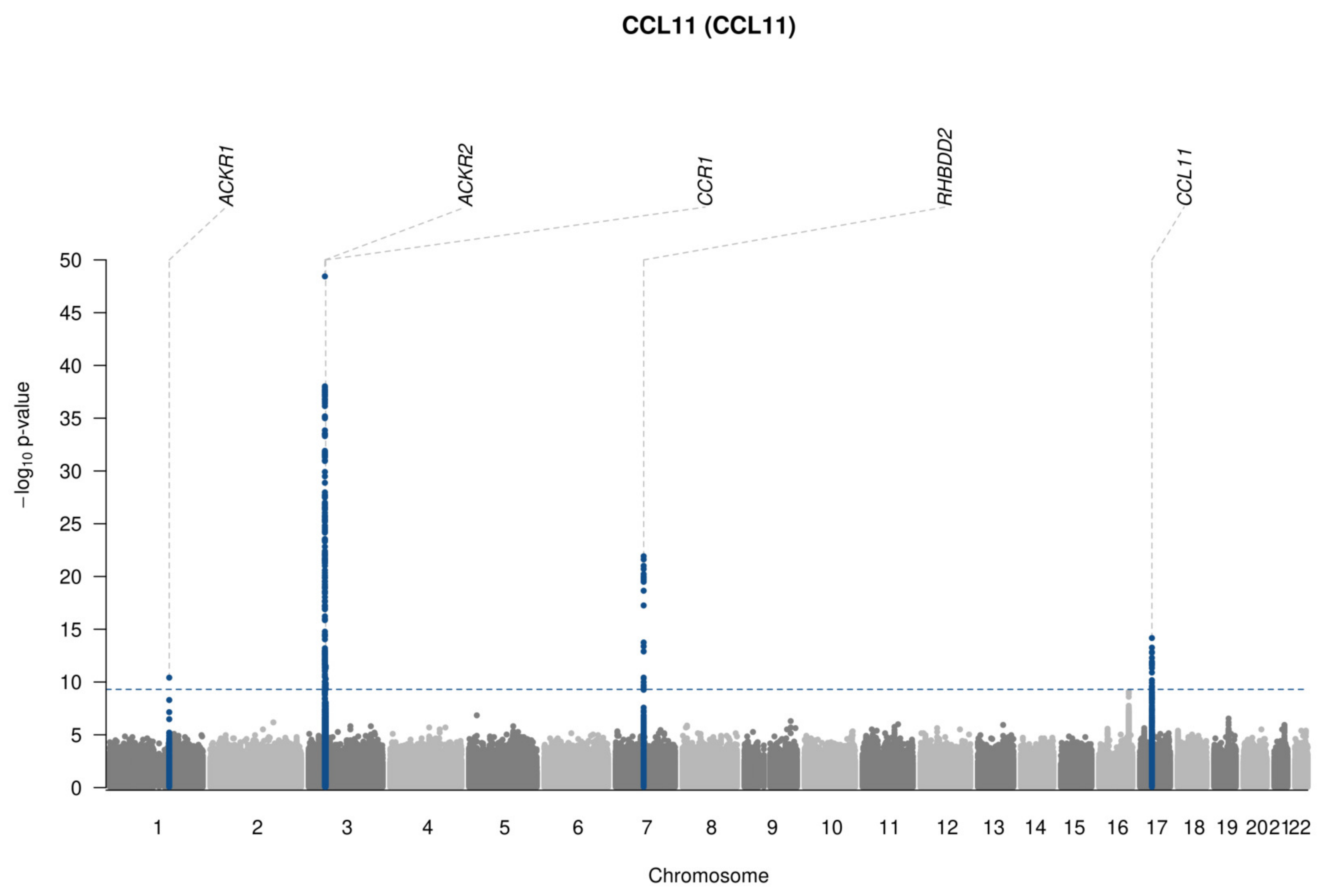

CCL11 (CCL11)
ACKR1
ACKR2
CCR1
RHBDD2
CCL11
−log10 p-value
0
5
10
15
20
25
30
35
40
45
50
1
2
3
4
5
6
7
8
9
10
11
12
13
14
15
16
17
18
19
20
21
22
Chromosome

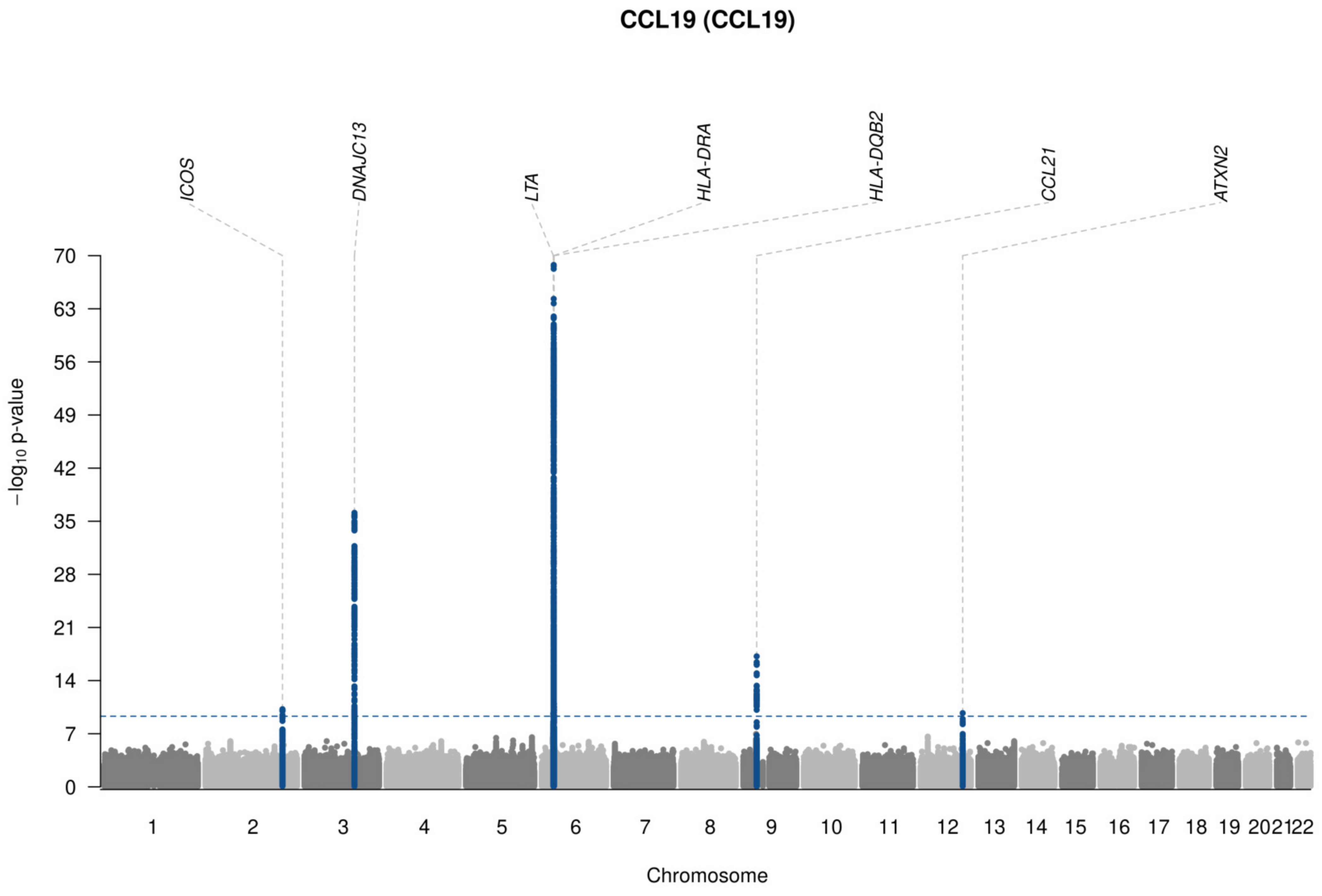
CCL19 (CCL19)
ICOS
DNAJC13
LTA
HLA-DRA
HLA-DQB2
CCL21
ATXN2
−log10 p-value
70
63
56
49
42
35
28
21
14
7
0
1
2
3
4
5
6
7
8
9
10
11
12
13
14
15
16
17
18
19
20
21
22
Chromosome

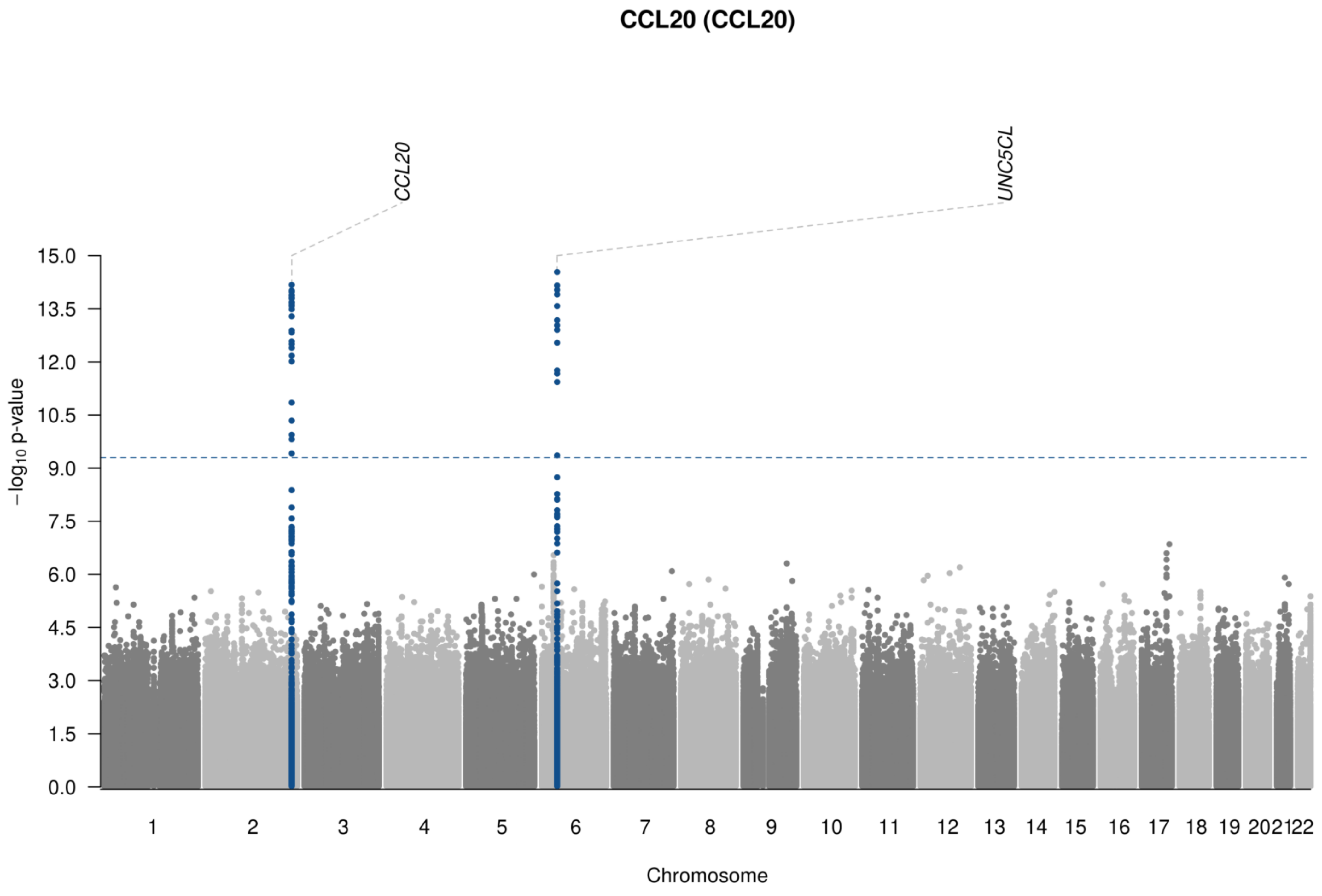
CCL20 (CCL20)
CCL20
UNC5CL
15.0
13.5
12.0
10.5
9.0
7.5
6.0
4.5
3.0
1.5
0.0
−log10 p-value
1
2
3
4
5
6
7
8
9
10
11
12
13
14
15
16
17
18
19
20
21
22
Chromosome

CCL23 (CCL23)

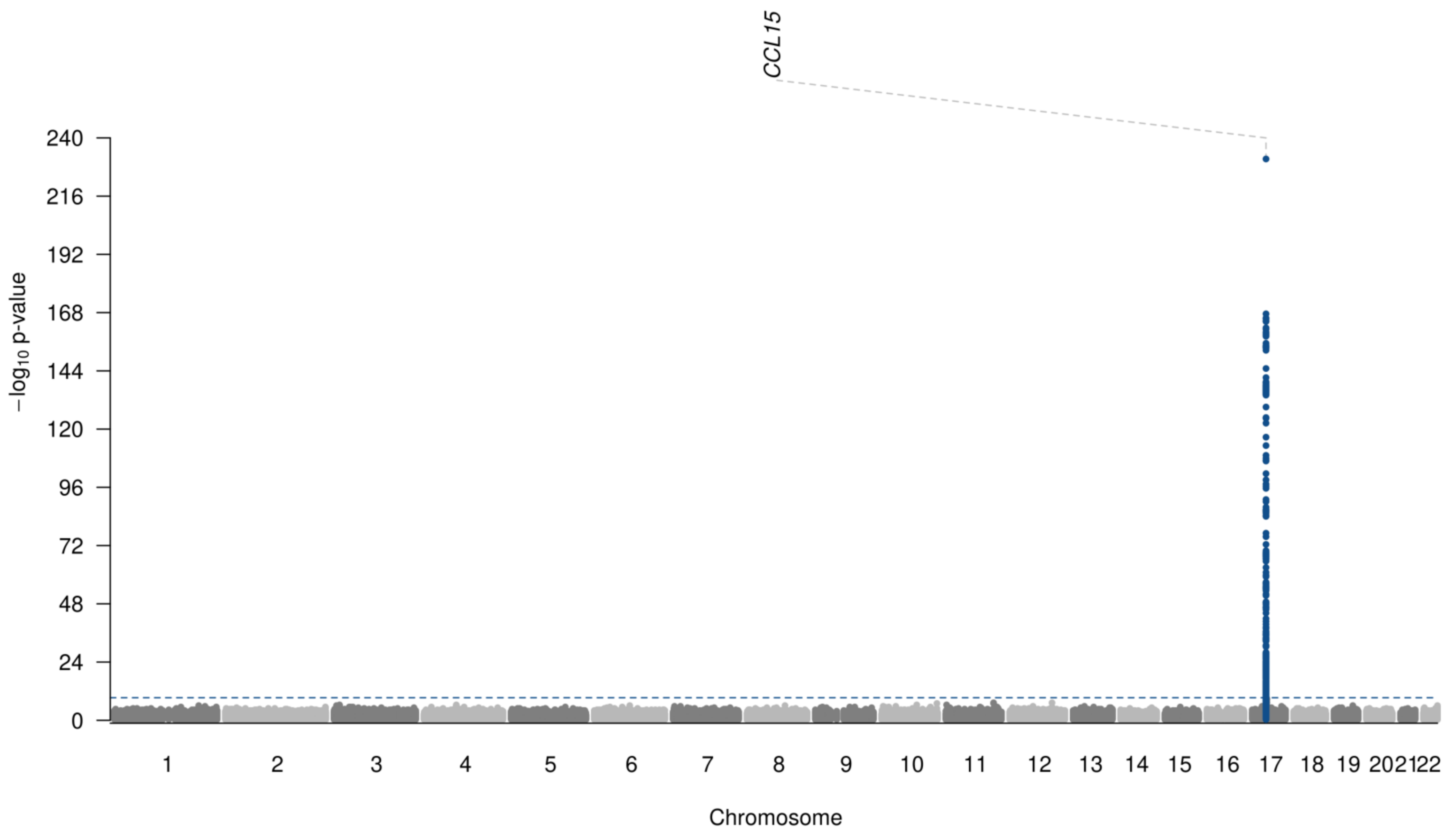

CCL15
−log10 p-value
240
216
192
168
144
120
96
72
48
24
0
1
2
3
4
5
6
7
8
9
10
11
12
13
14
15
16
17
18
19
20
21
22
Chromosome

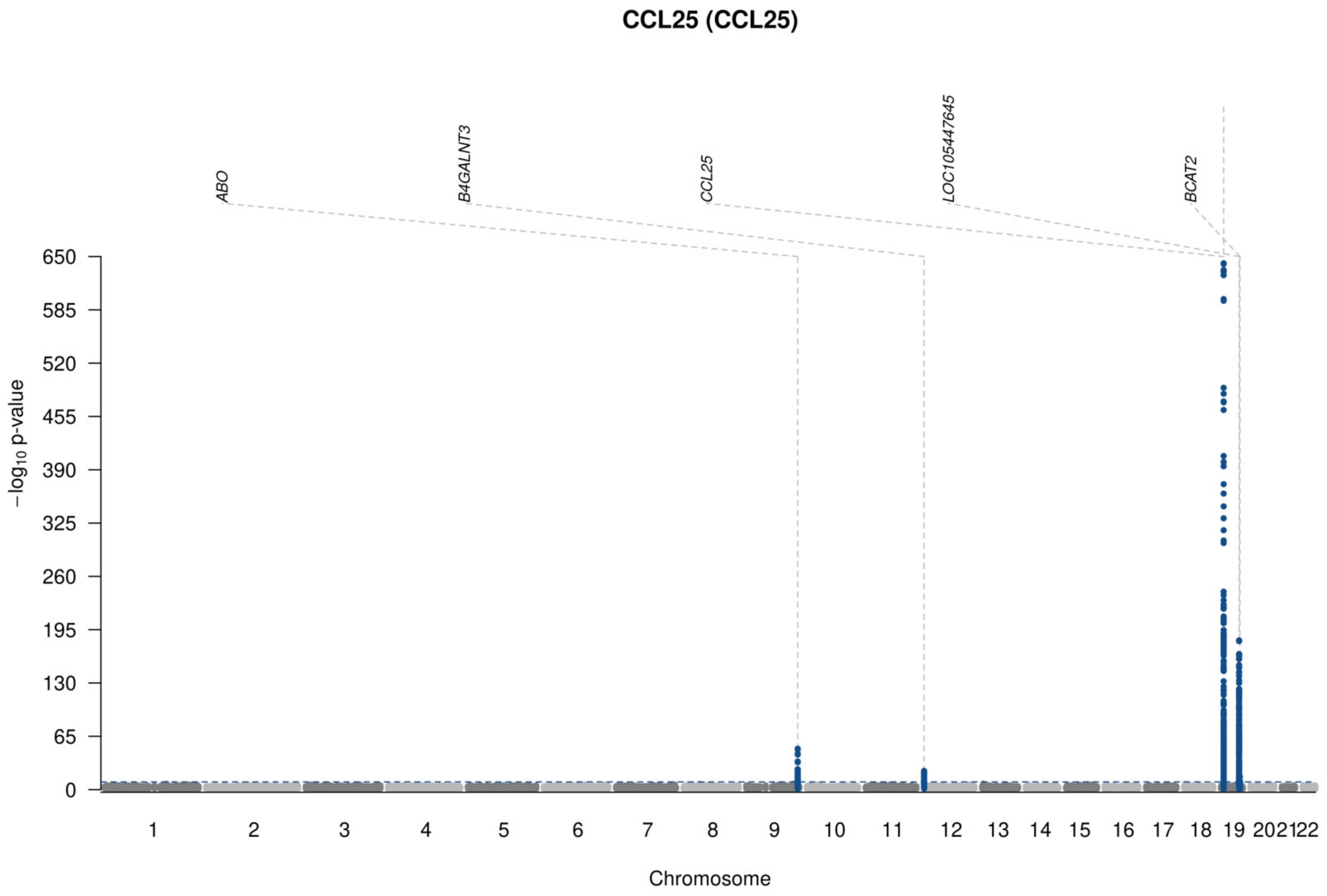

CCL25 (CCL25)
ABO
B4GALNT3
CCL25
LOC105447645
BCAT2
650
585
520
455
390
325
260
195
130
65
0
−log10 p-value
1
2
3
4
5
6
7
8
9
10
11
12
13
14
15
16
17
18
19
20
21
22
Chromosome

CCL28 (CCL28)

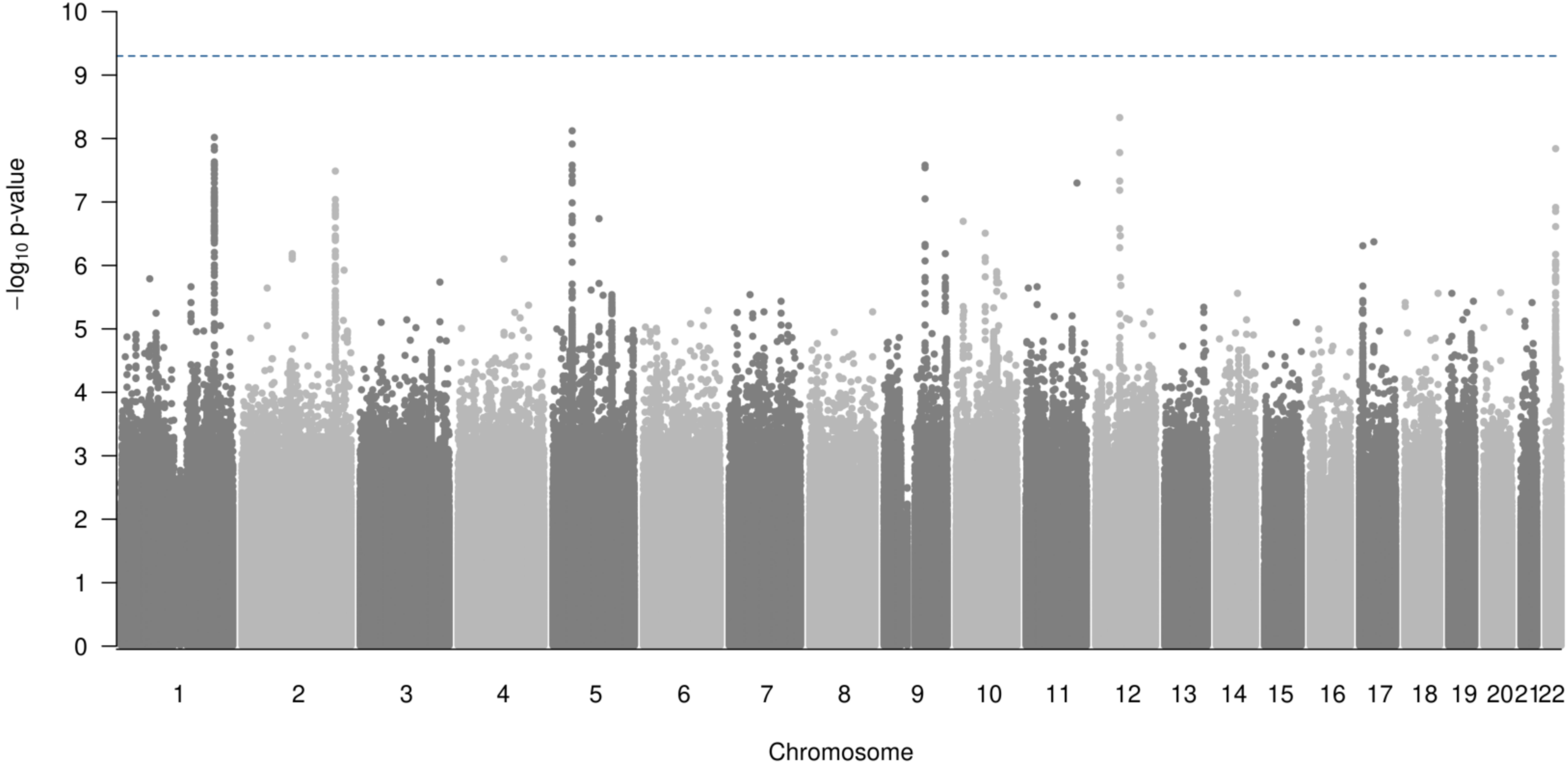

−log₁₀ p-value
0
1
2
3
4
5
6
7
8
9
10
1
2
3
4
5
6
7
8
9
10
11
12
13
14
15
16
17
18
19
20
21
22
Chromosome

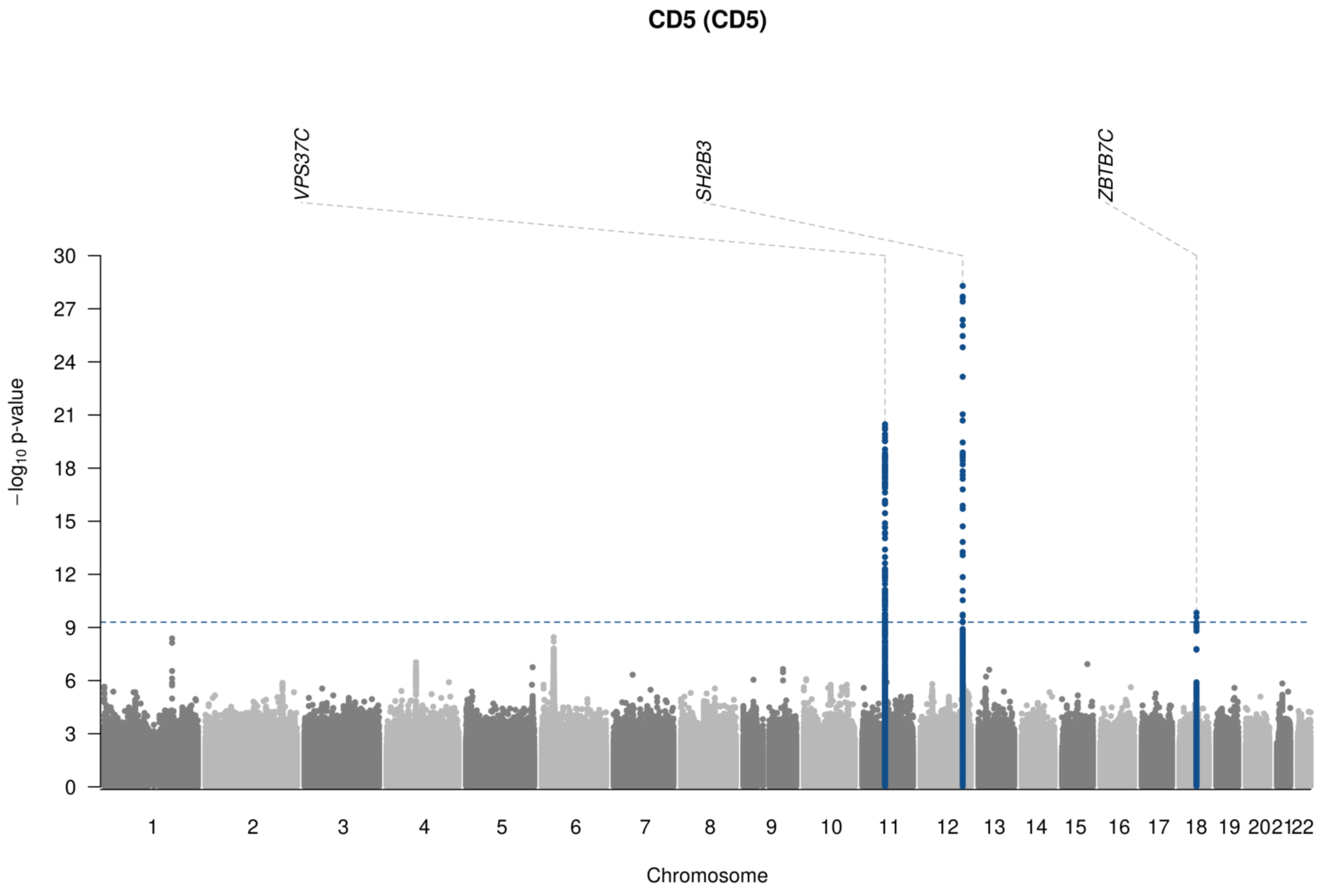
CD5 (CD5)
VPS37C
SH2B3
ZBTB7C
30
27
24
21
18
15
12
9
6
3
0
−log10 p-value
1
2
3
4
5
6
7
8
9
10
11
12
13
14
15
16
17
18
19
20
21
22
Chromosome

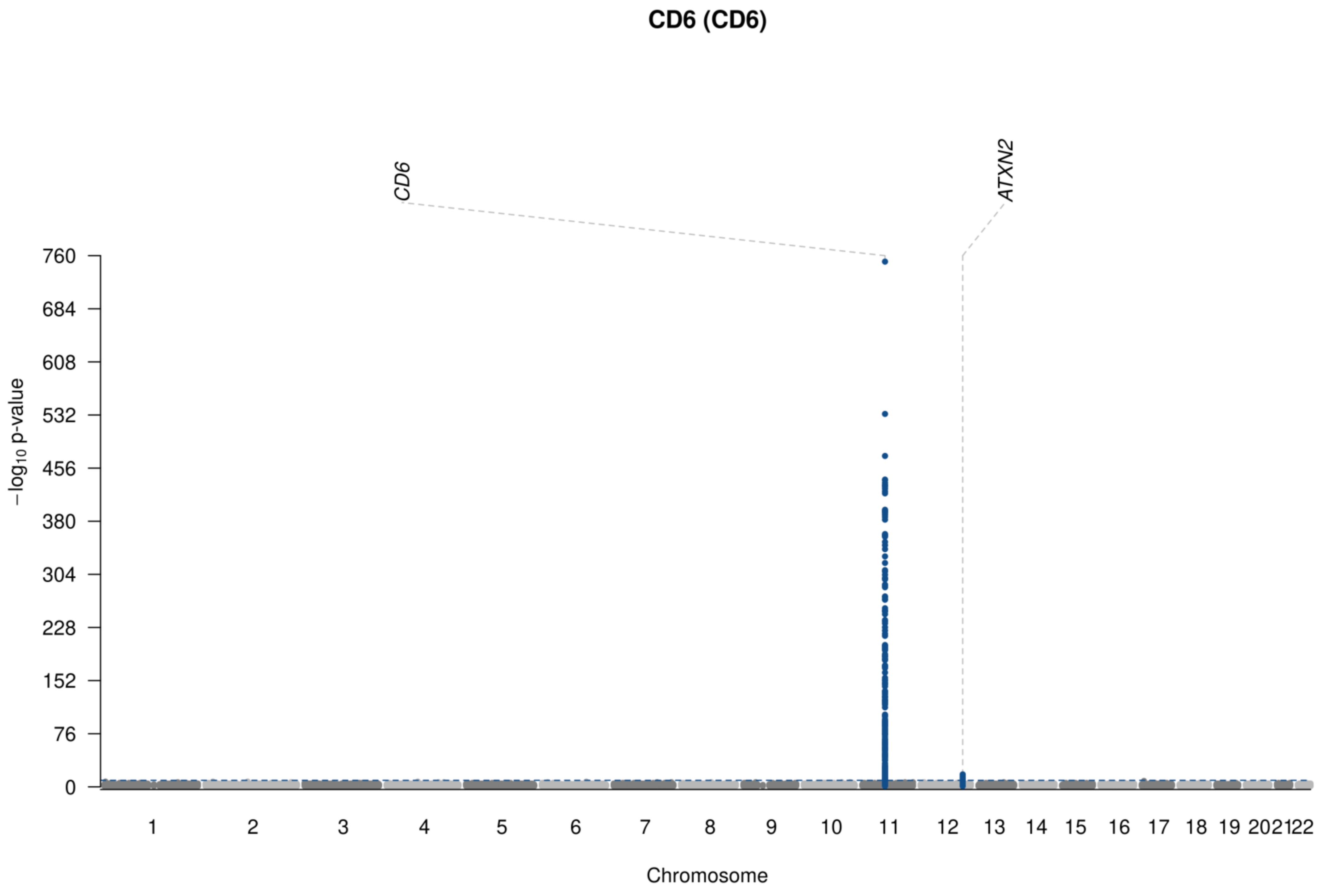

CD6 (CD6)
CD6
ATXN2
760
684
608
532
456
380
304
228
152
76
0
$-\log_{10}$ p-value
1
2
3
4
5
6
7
8
9
10
11
12
13
14
15
16
17
18
19
202122
Chromosome

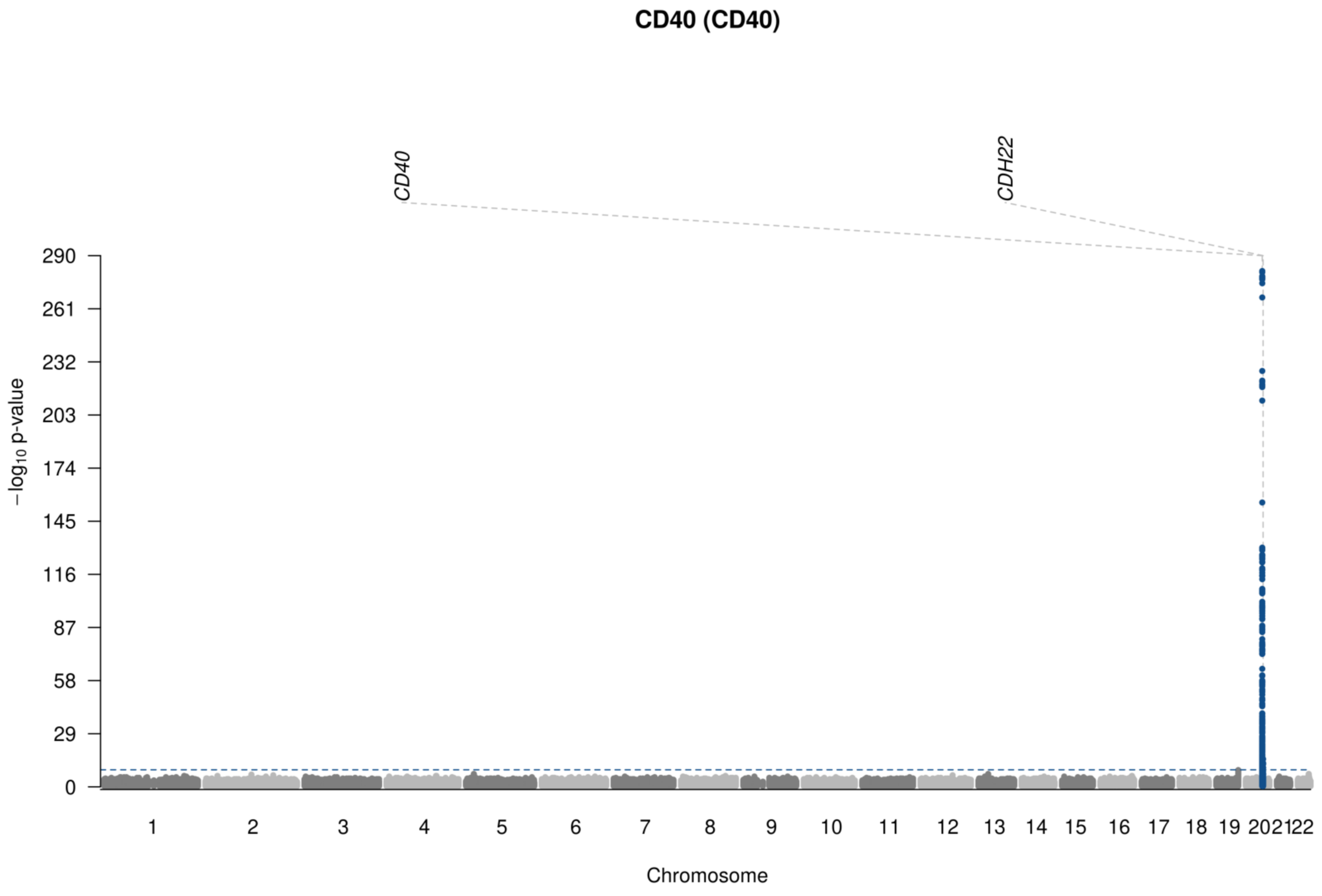

CD40 (CD40)
CD40
CDH22
290
261
232
203
174
145
116
87
58
29
0
$-\log_{10}$ p-value
1
2
3
4
5
6
7
8
9
10
11
12
13
14
15
16
17
18
19
20
21
22
Chromosome

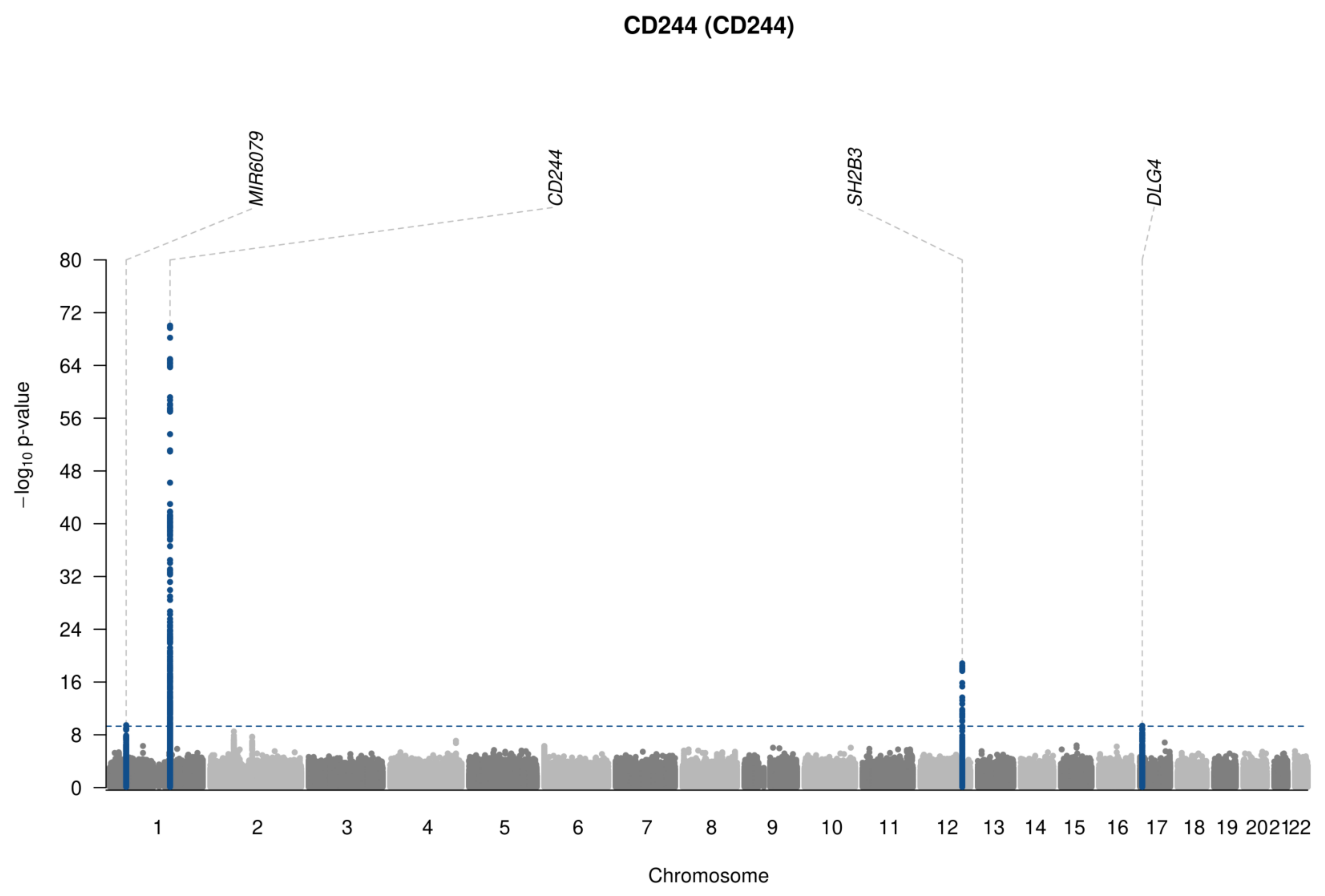

CD244 (CD244)
MIR6079
CD244
SH2B3
DLG4
80
72
64
56
48
40
32
24
16
8
0
$-\log_{10}$ p-value
1
2
3
4
5
6
7
8
9
10
11
12
13
14
15
16
17
18
19
20
21
22
Chromosome

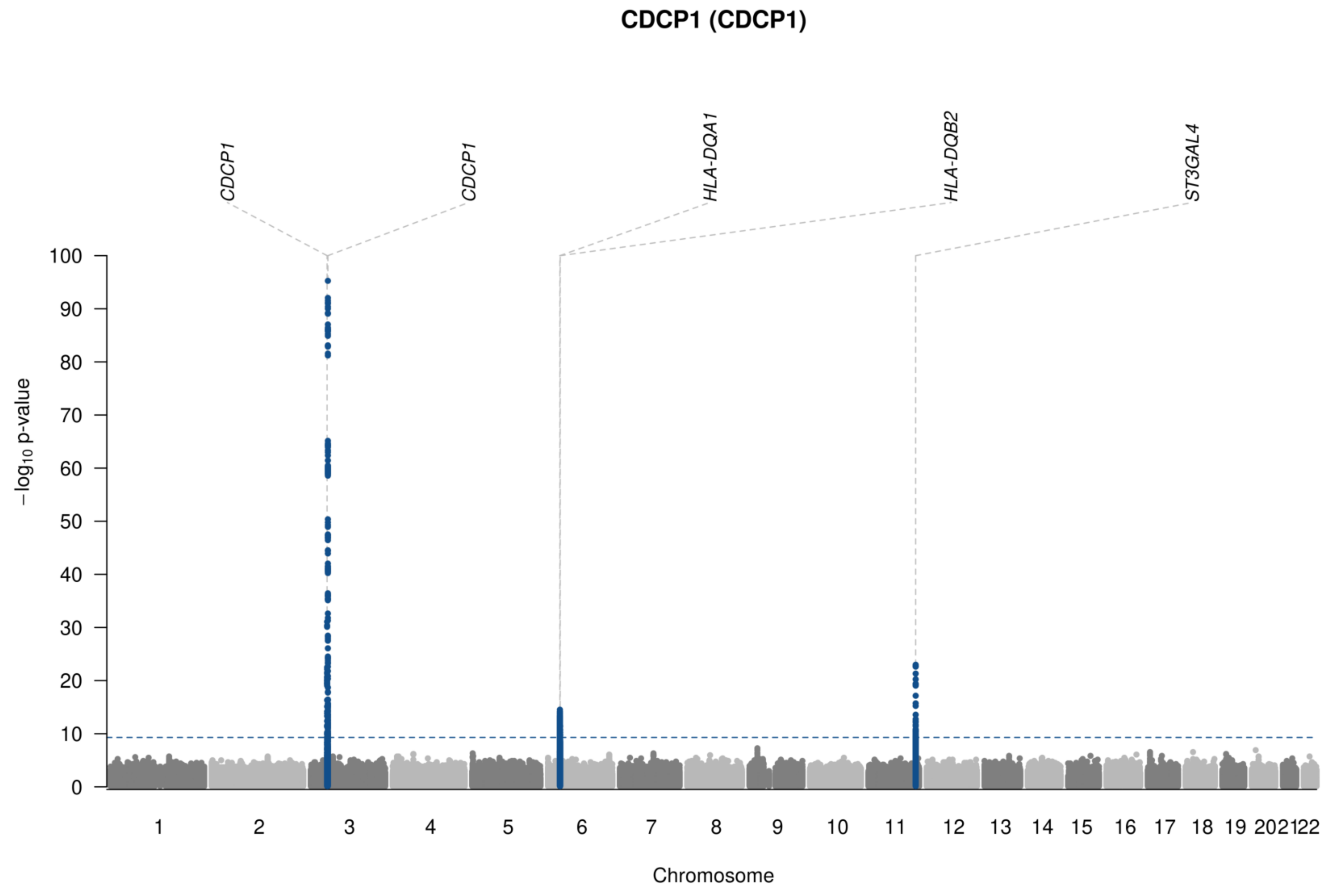

CDCP1 (CDCP1)
CDCP1
CDCP1
HLA-DQA1
HLA-DQB2
ST3GAL4
100
90
80
70
60
50
40
30
20
10
0
−log10 p-value
1
2
3
4
5
6
7
8
9
10
11
12
13
14
15
16
17
18
19
20
21
22
Chromosome

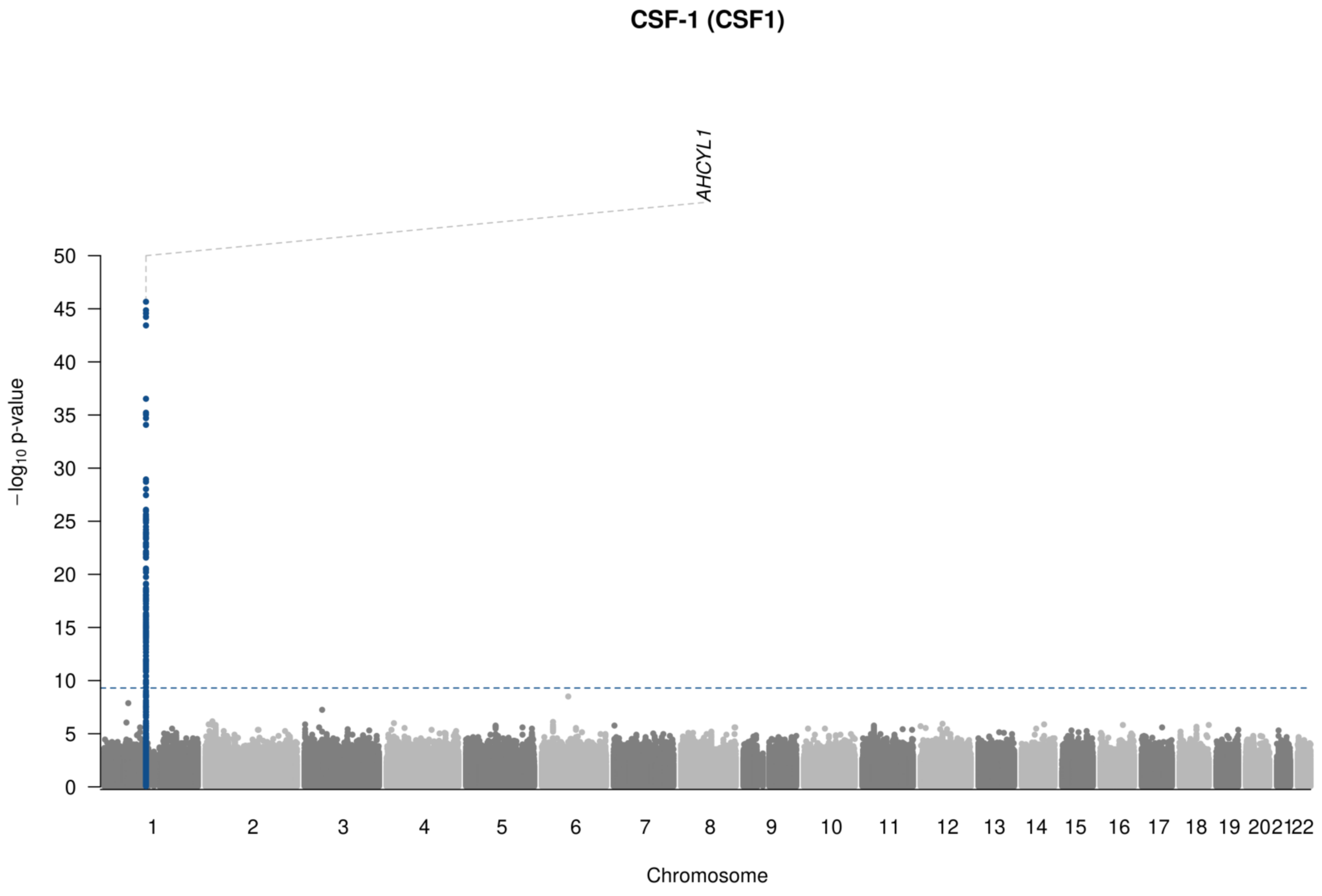

CSF-1 (CSF1)
AHCYL1
−log10 p-value
50
45
40
35
30
25
20
15
10
5
0
1
2
3
4
5
6
7
8
9
10
11
12
13
14
15
16
17
18
19
20
21
22
Chromosome

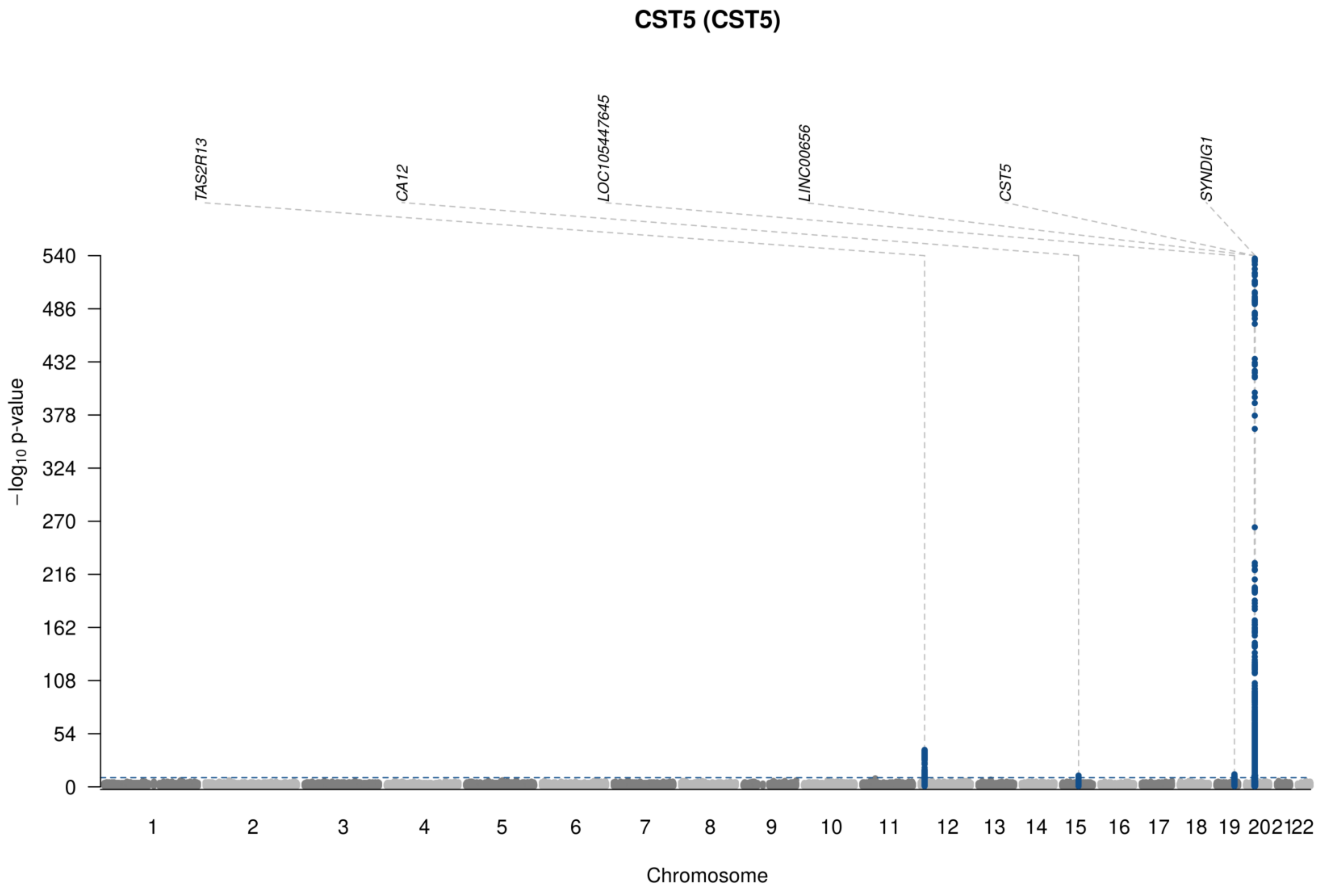

CST5 (CST5)
TAS2R13
CA12
LOC105447645
LINC00656
CST5
SYNDIG1
540
486
432
378
324
270
216
162
108
54
0
−log10 p-value
1
2
3
4
5
6
7
8
9
10
11
12
13
14
15
16
17
18
19
20
21
22
Chromosome

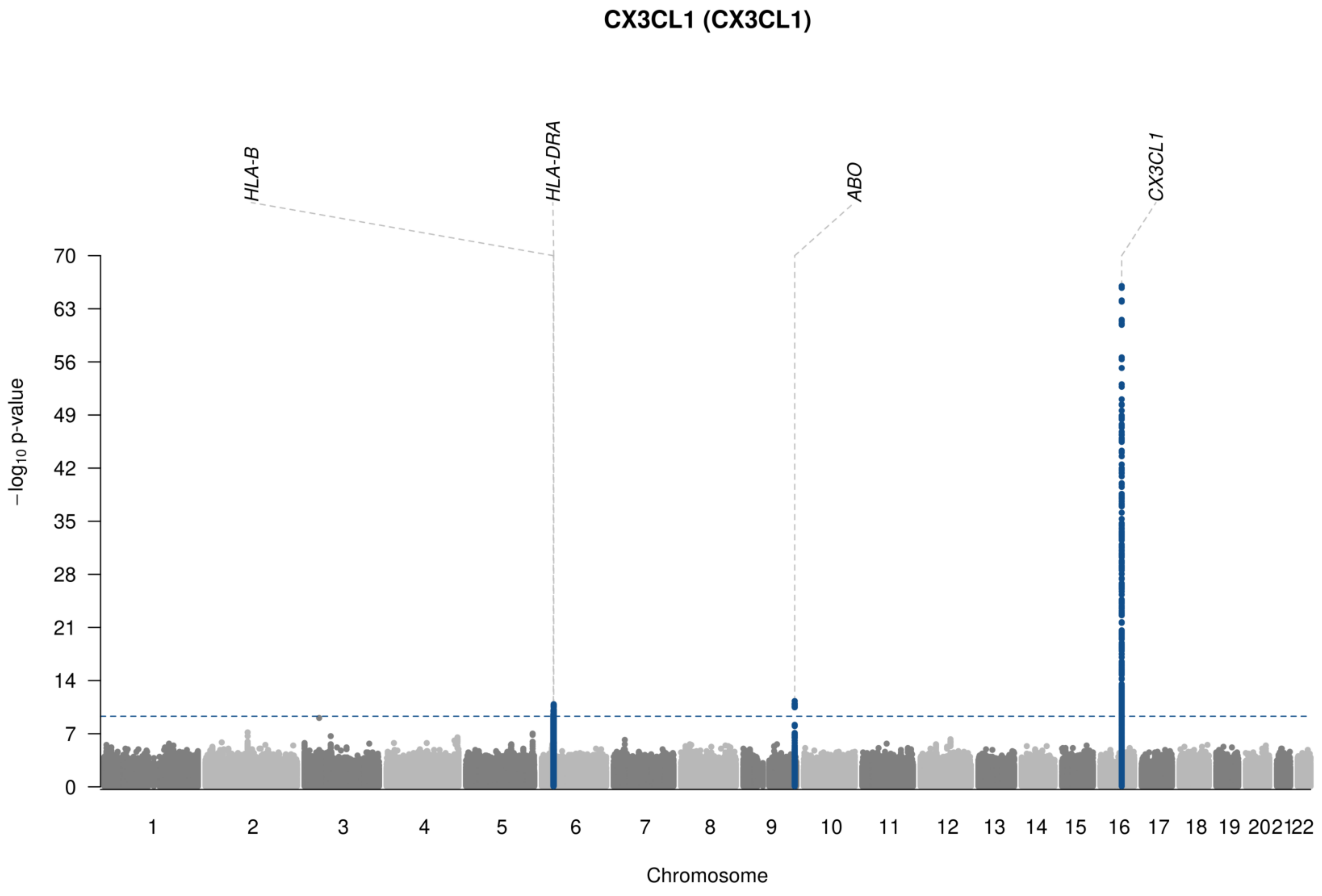

CX3CL1 (CX3CL1)
HLA-B
HLA-DRA
ABO
CX3CL1
−log10 p-value
70
63
56
49
42
35
28
21
14
7
0
1
2
3
4
5
6
7
8
9
10
11
12
13
14
15
16
17
18
19
20
21
22
Chromosome

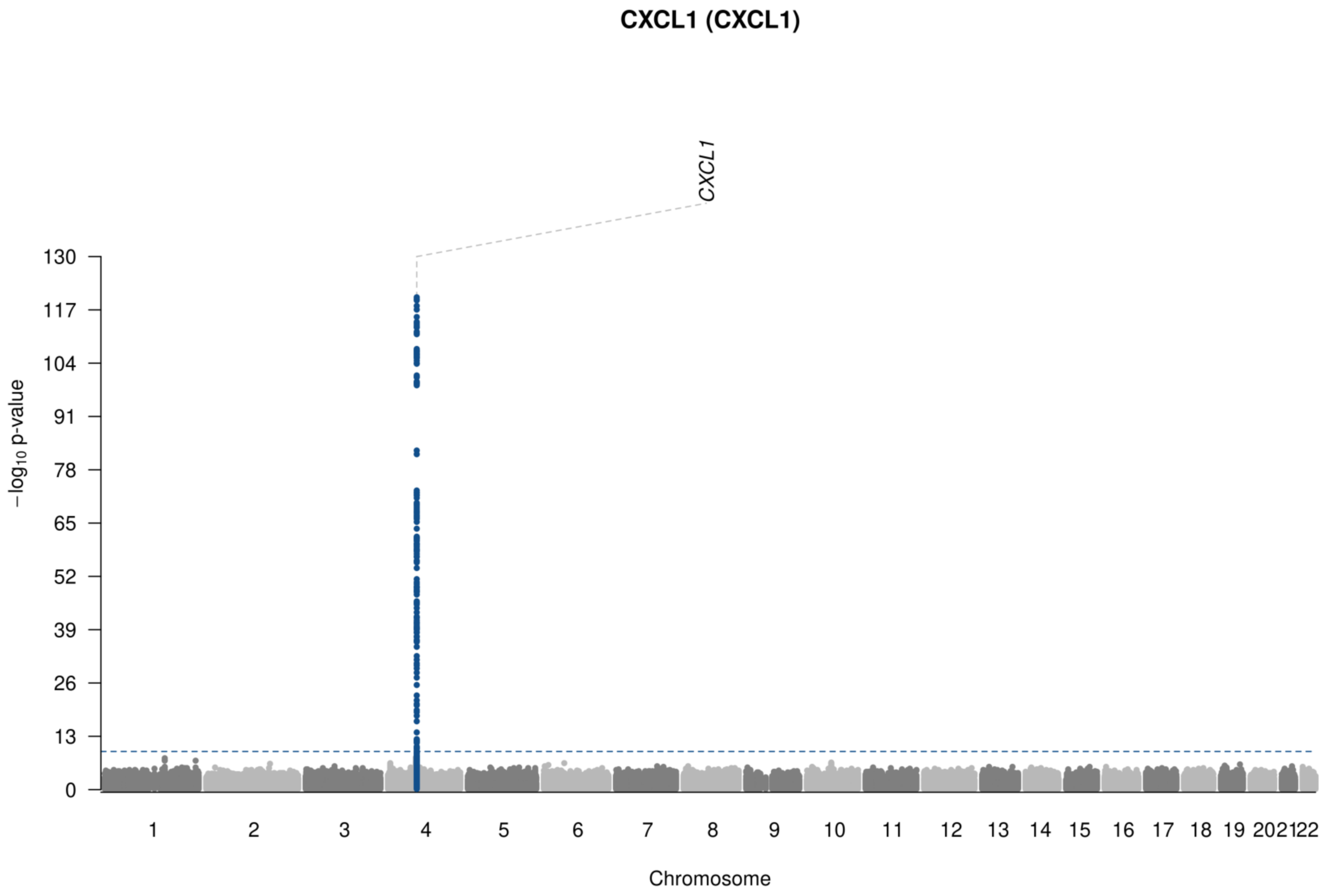

CXCL1 (CXCL1)
CXCL1
−log10 p-value
130
117
104
91
78
65
52
39
26
13
0
1
2
3
4
5
6
7
8
9
10
11
12
13
14
15
16
17
18
19
20
21
22
Chromosome

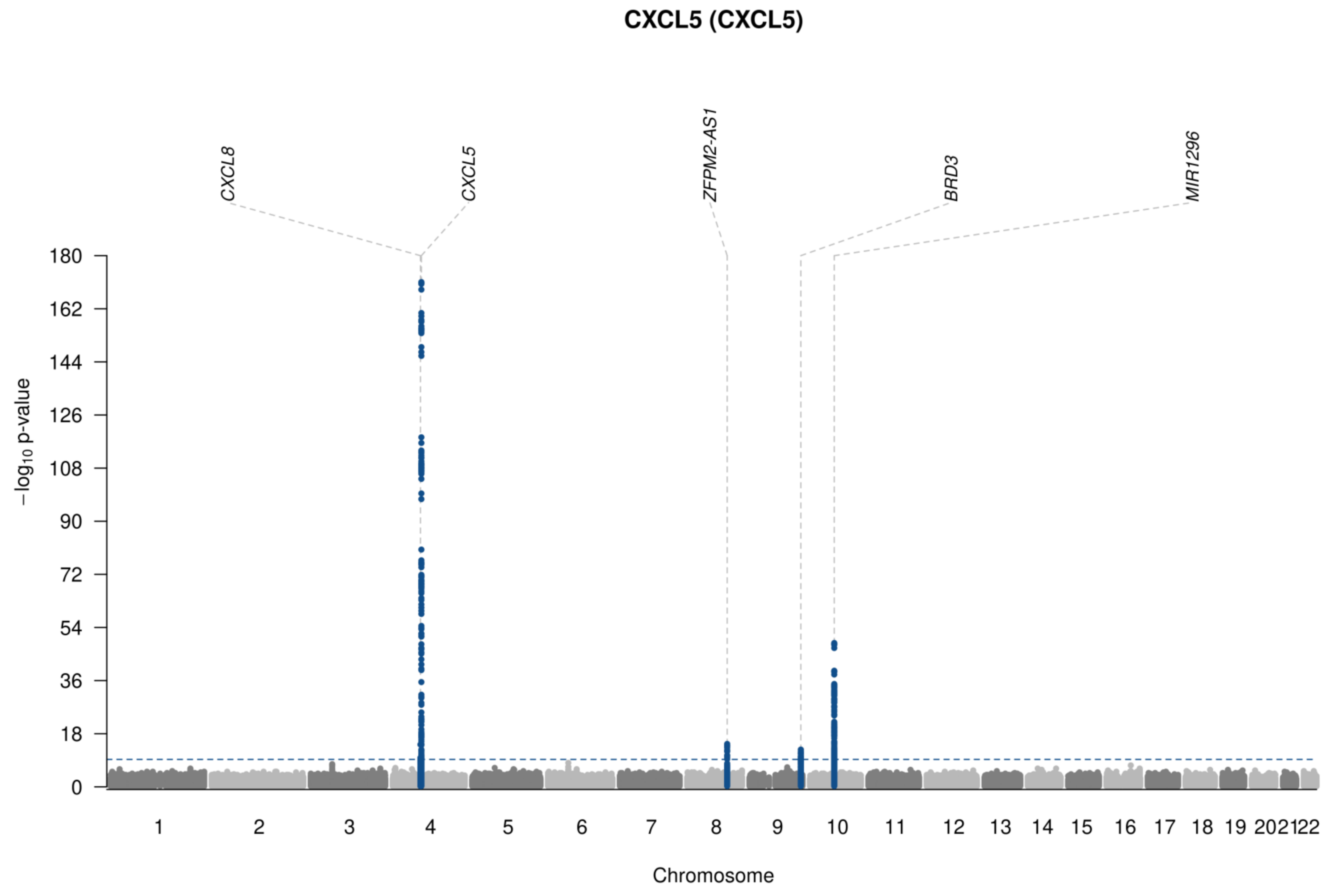

CXCL5 (CXCL5)
CXCL8
CXCL5
ZFPM2-AS1
BRD3
MIR1296
180
162
144
126
108
90
72
54
36
18
0
$-\log_{10}$ p-value
1
2
3
4
5
6
7
8
9
10
11
12
13
14
15
16
17
18
19
20
21
22
Chromosome

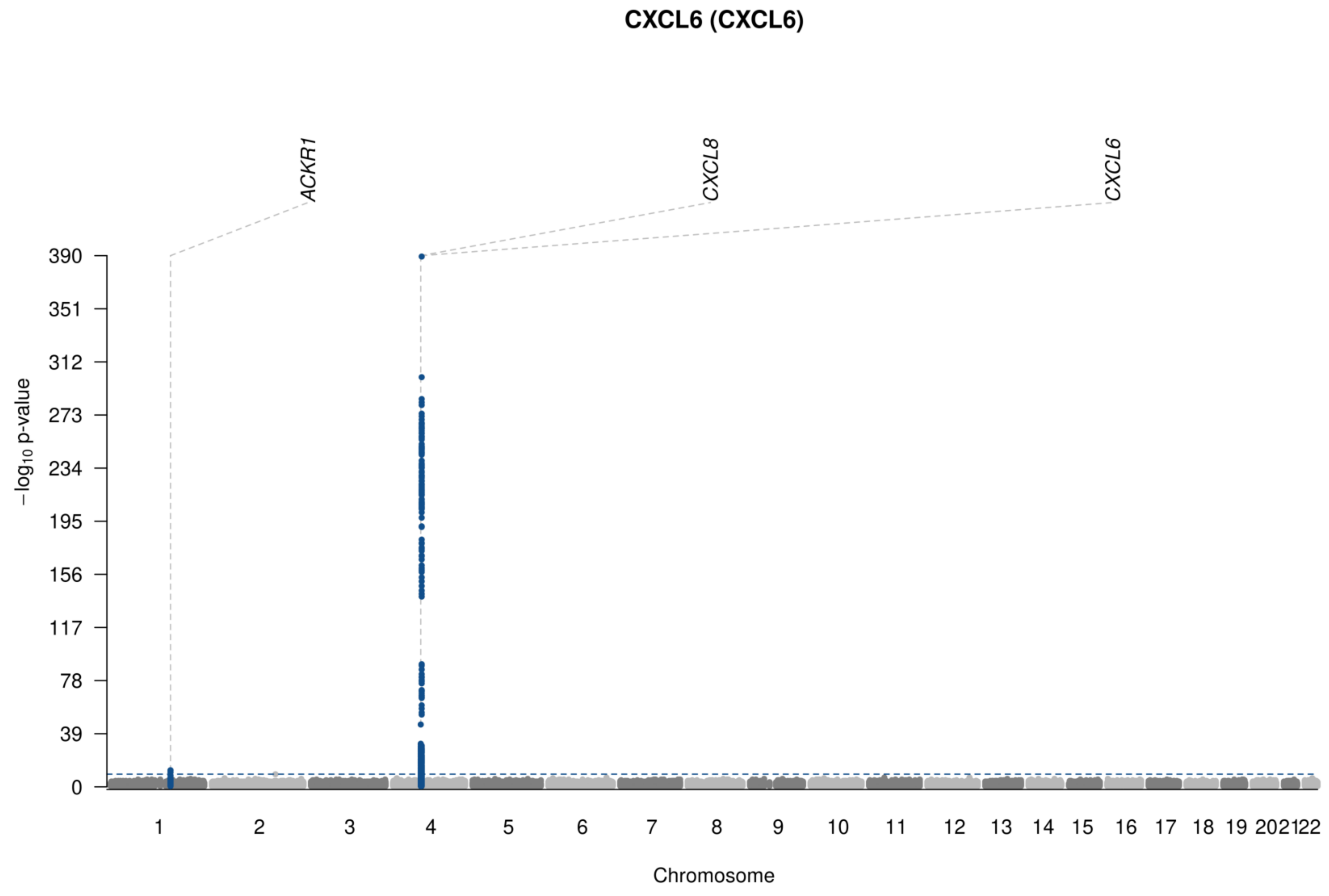

CXCL6 (CXCL6)
ACKR1
CXCL8
CXCL6
390
351
312
273
234
195
156
117
78
39
0
−log10 p-value
1
2
3
4
5
6
7
8
9
10
11
12
13
14
15
16
17
18
19
20
21
22
Chromosome

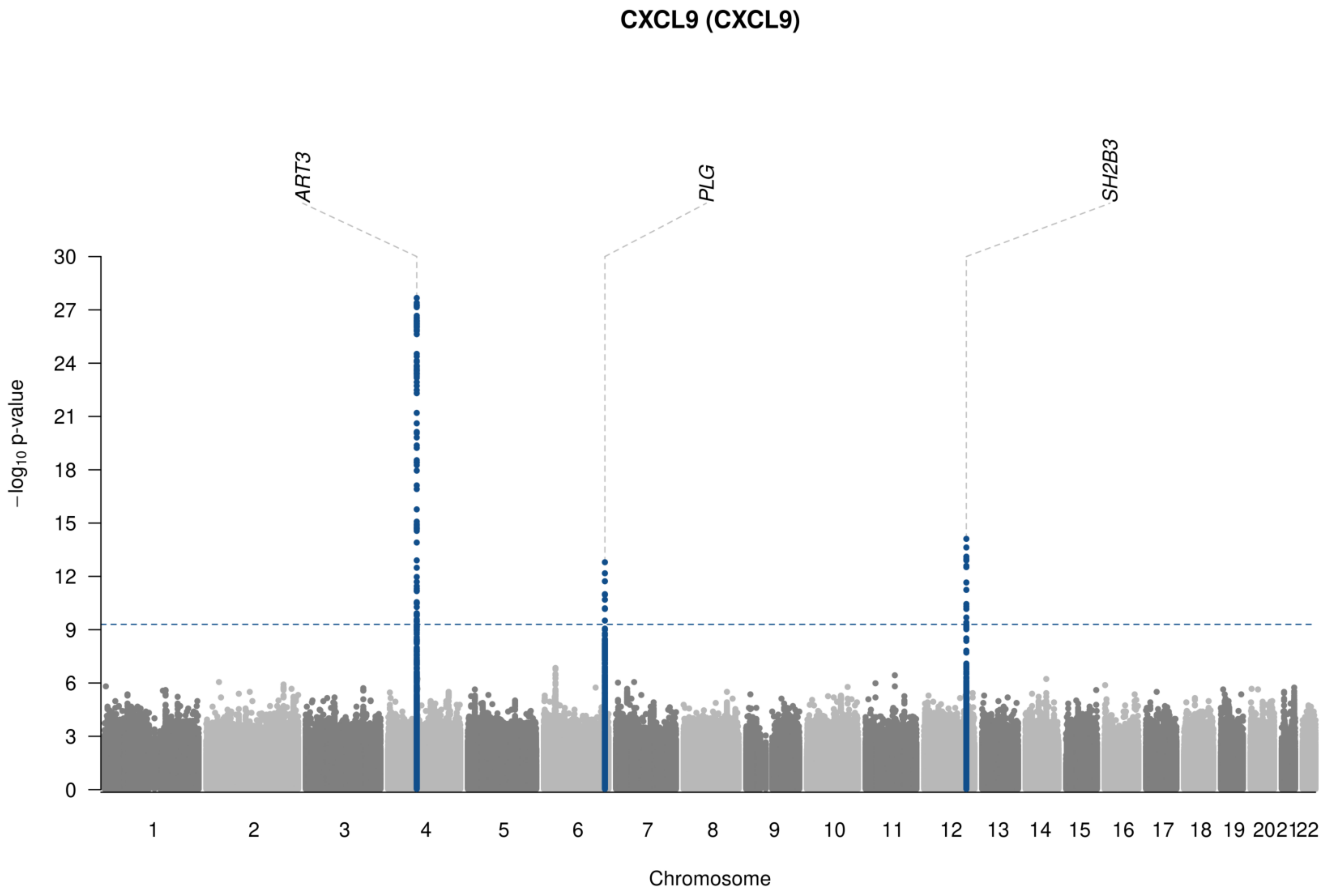
CXCL9 (CXCL9)
ART3
PLG
SH2B3
30
27
24
21
18
15
12
9
6
3
0
−log10 p-value
1
2
3
4
5
6
7
8
9
10
11
12
13
14
15
16
17
18
19
20
21
22
Chromosome

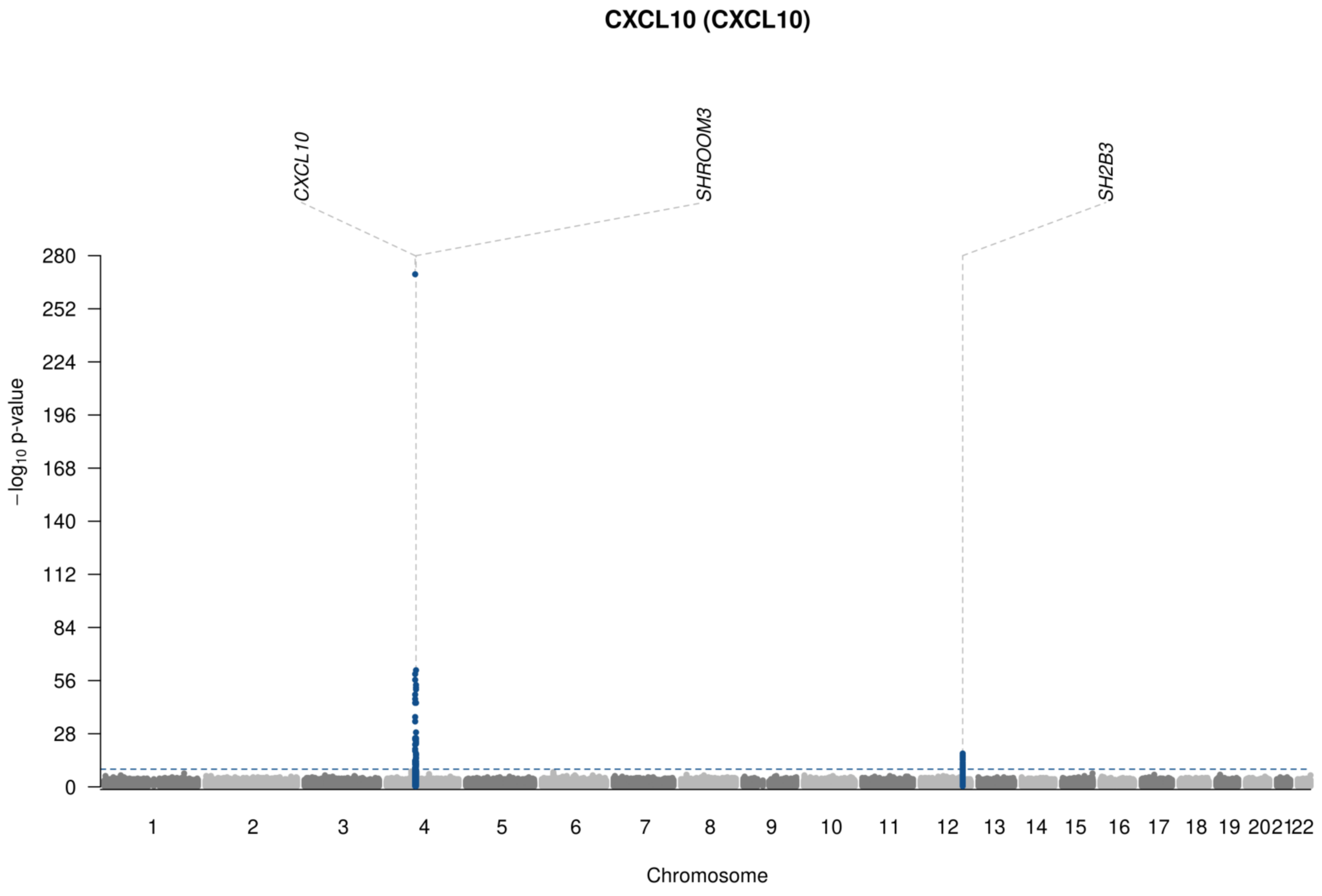

CXCL10 (CXCL10)
CXCL10
SHROOM3
SH2B3
280
252
224
196
168
140
112
84
56
28
0
−log10 p-value
1 2 3 4 5 6 7 8 9 10 11 12 13 14 15 16 17 18 19 20 21 22
Chromosome

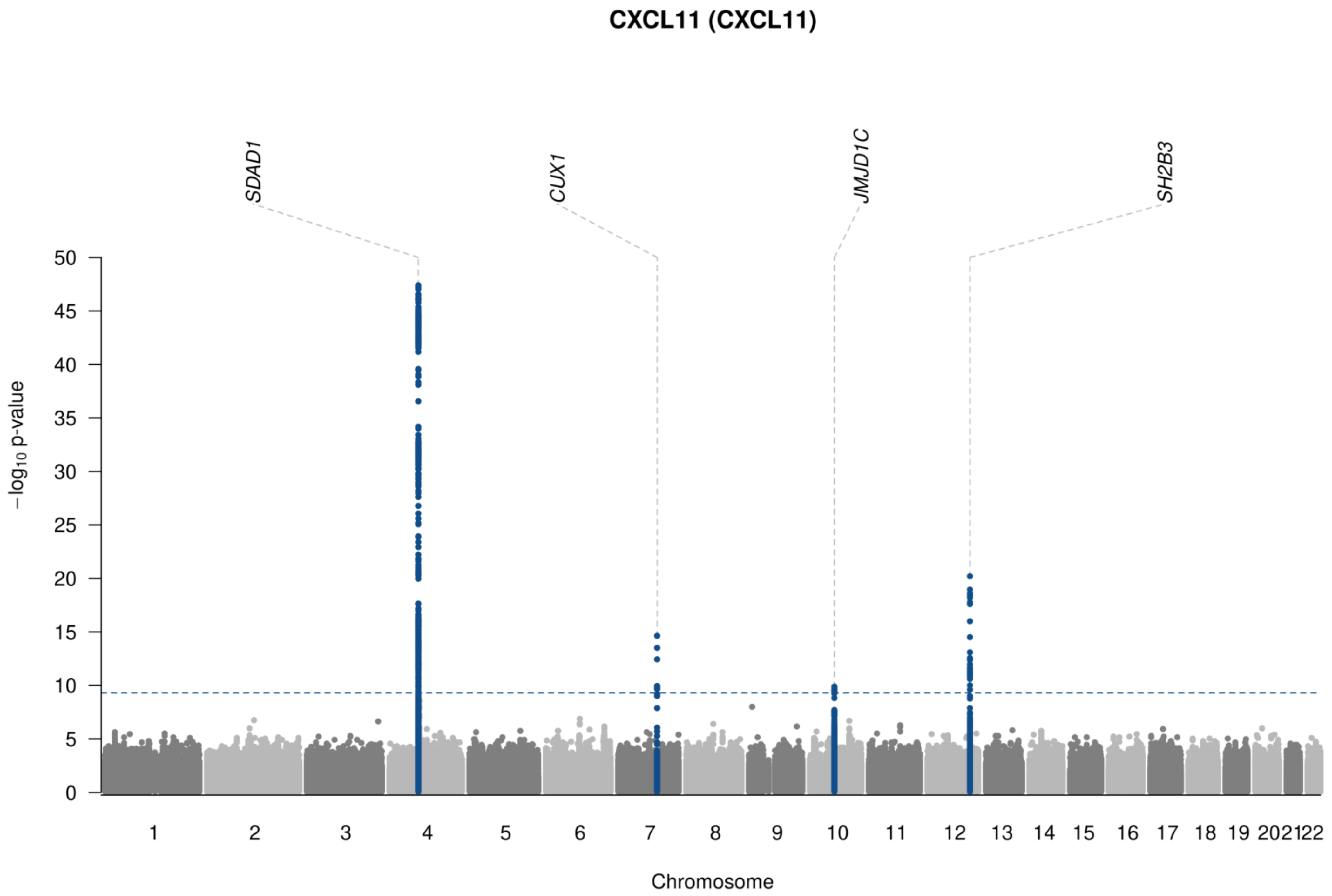

CXCL11 (CXCL11)
SDAD1
CUX1
JMJD1C
SH2B3
$-\log_{10}$ p-value
50
45
40
35
30
25
20
15
10
5
0
1
2
3
4
5
6
7
8
9
10
11
12
13
14
15
16
17
18
19
20
21
22
Chromosome

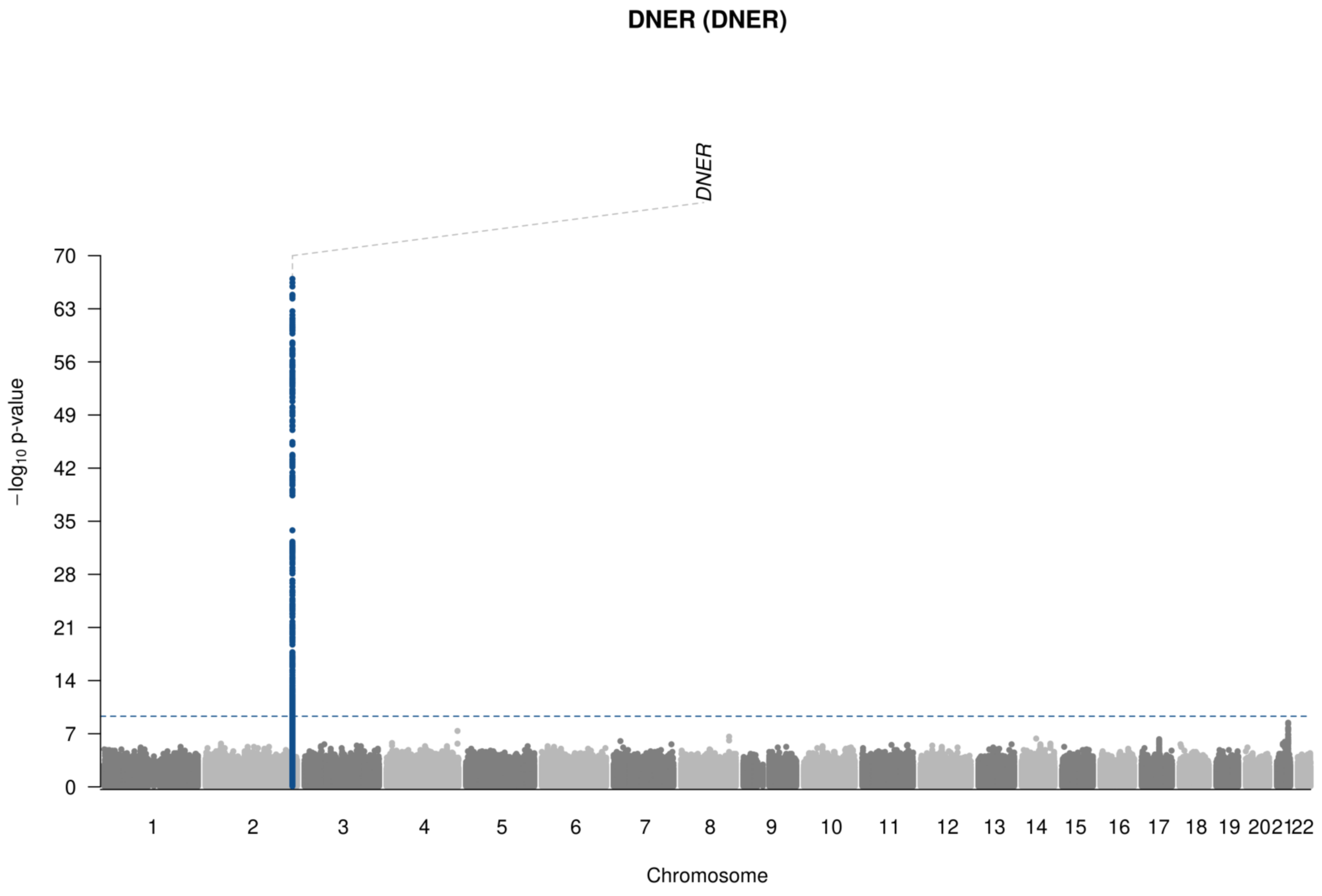

DNER (DNER)
DNER
70
63
56
49
42
35
28
21
14
7
0
−log10 p-value
1
2
3
4
5
6
7
8
9
10
11
12
13
14
15
16
17
18
19
20
21
22
Chromosome

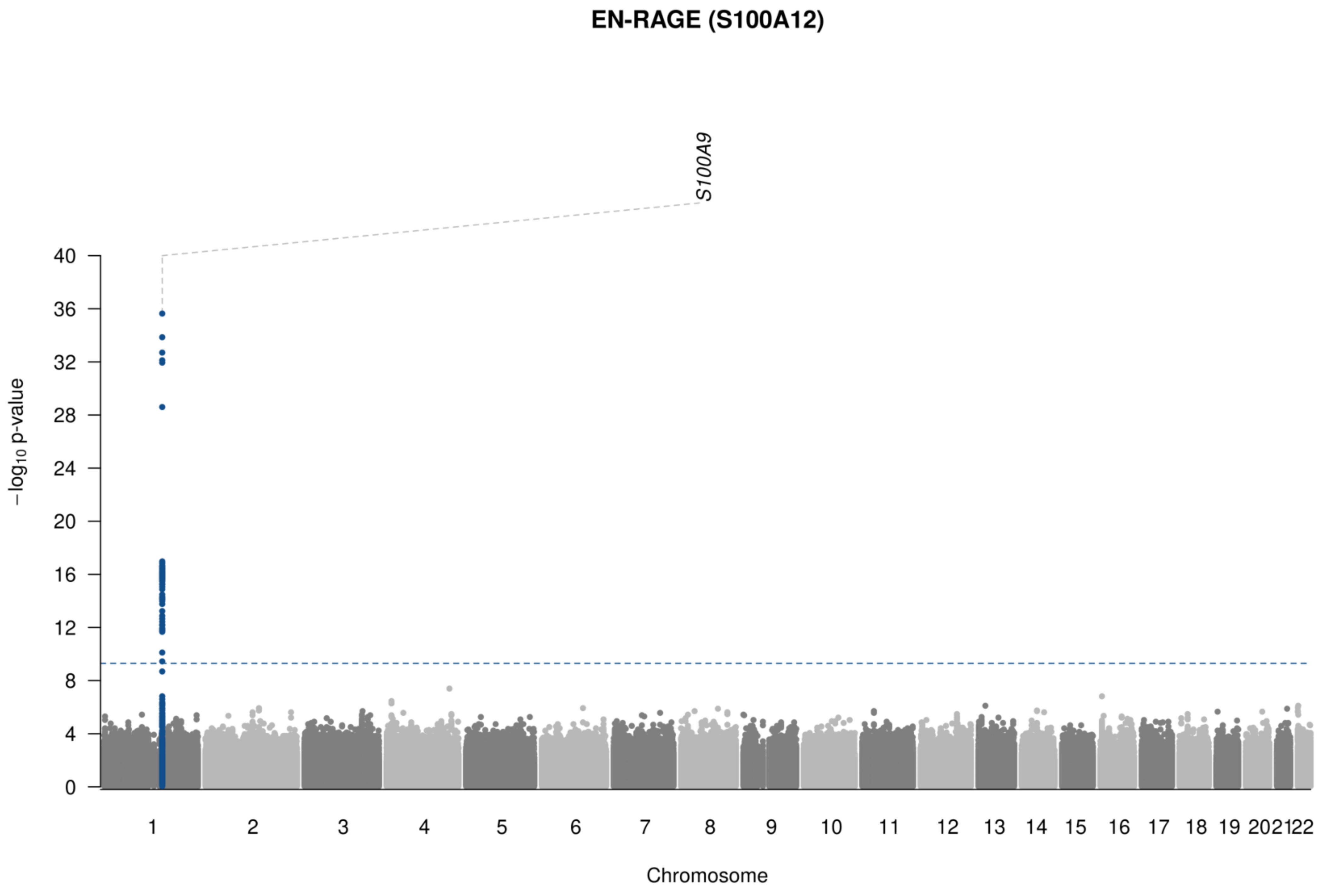

EN-RAGE (S100A12)
S100A9
−log10 p-value
0
4
8
12
16
20
24
28
32
36
40
1
2
3
4
5
6
7
8
9
10
11
12
13
14
15
16
17
18
19
20
21
22
Chromosome

FGF-5 (FGF5)

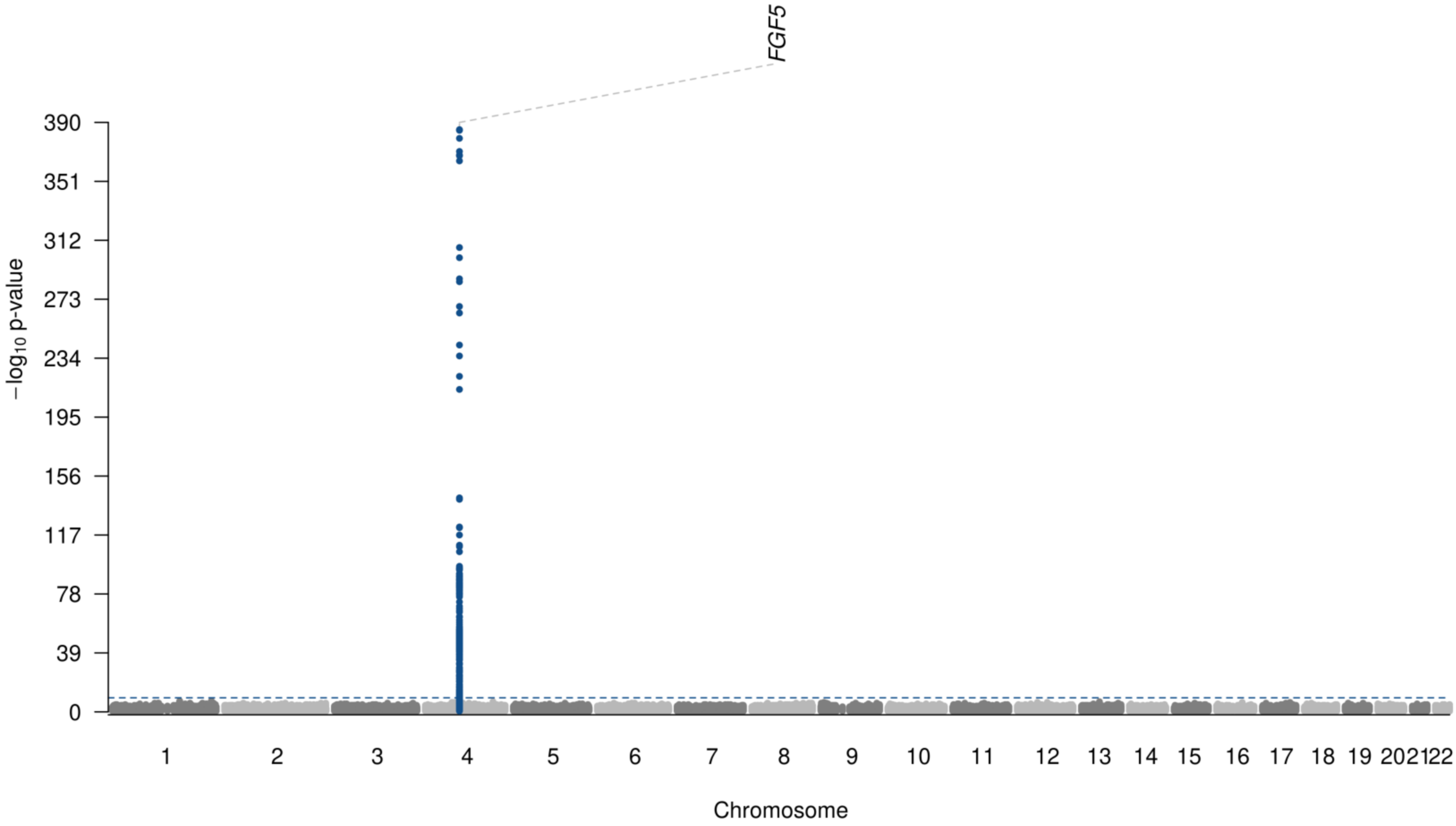

FGF5
390
351
312
273
234
195
156
117
78
39
0
−log10 p-value
1
2
3
4
5
6
7
8
9
10
11
12
13
14
15
16
17
18
19
202122
Chromosome

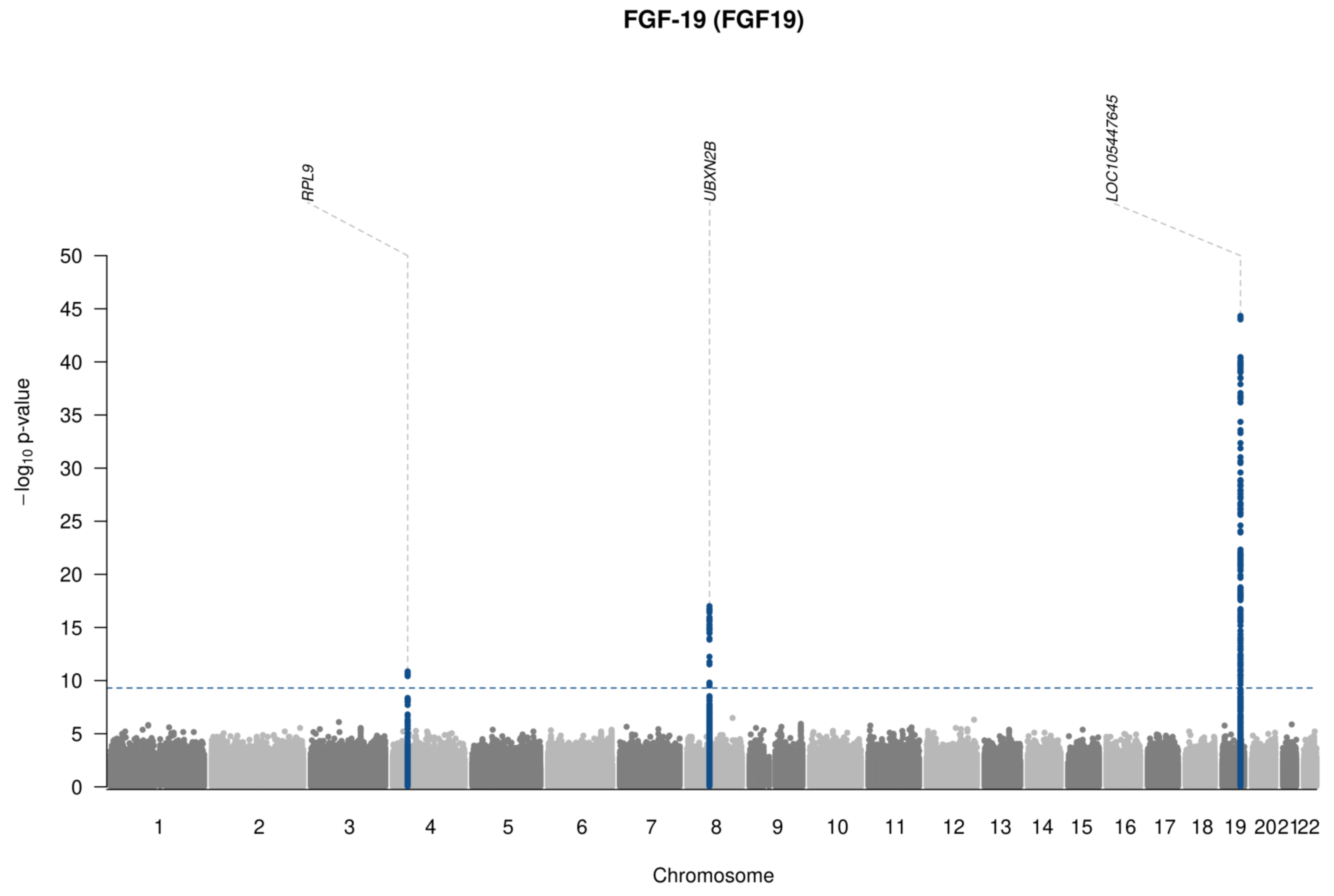
FGF-19 (FGF19)
RPL9
UBXN2B
LOC105447645
−log10 p-value
0
5
10
15
20
25
30
35
40
45
50
1
2
3
4
5
6
7
8
9
10
11
12
13
14
15
16
17
18
19
20
21
22
Chromosome

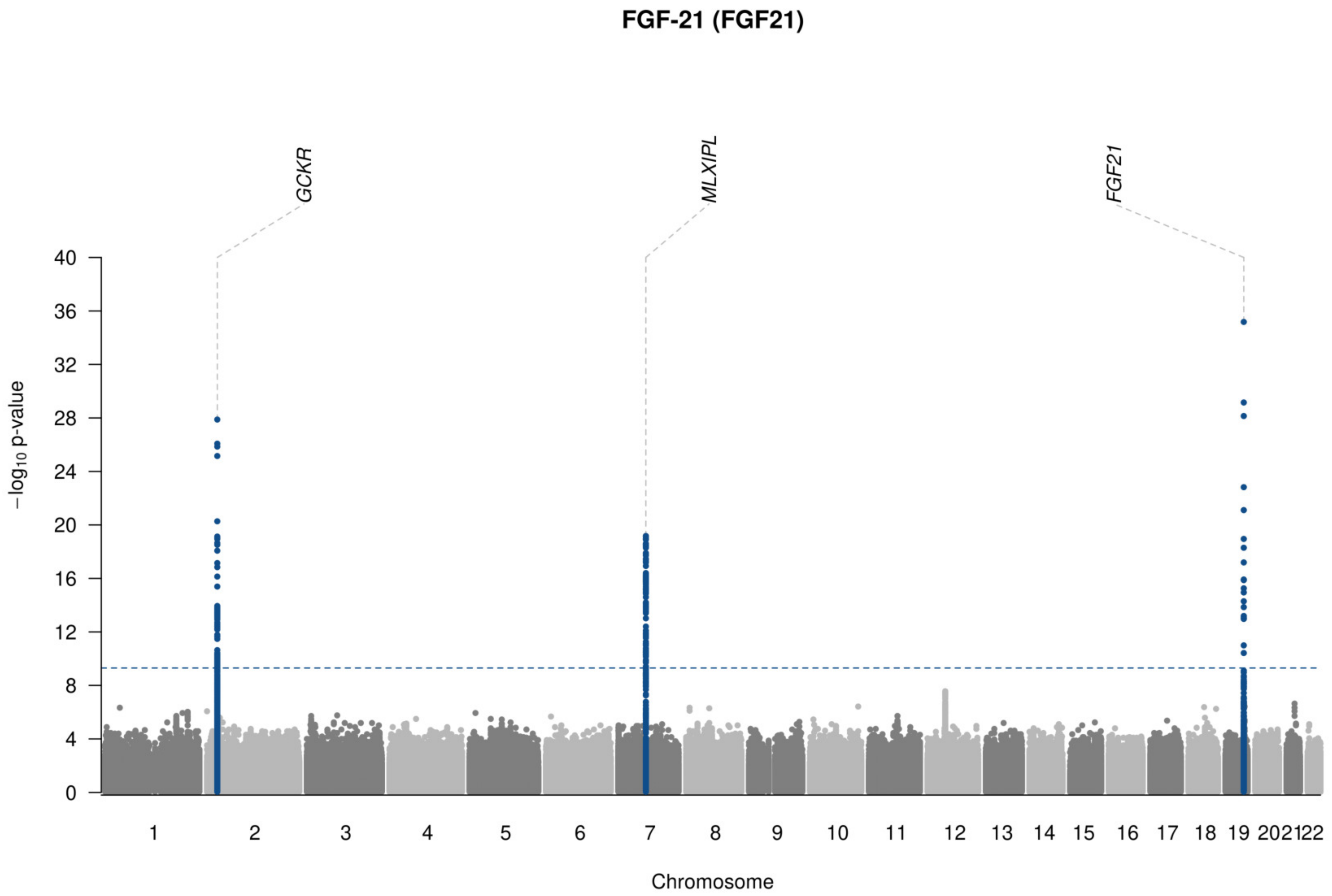

FGF-21 (FGF21)
GCKR
MLXIPL
FGF21
−log10 p-value
40
36
32
28
24
20
16
12
8
4
0
1
2
3
4
5
6
7
8
9
10
11
12
13
14
15
16
17
18
19
20
21
22
Chromosome

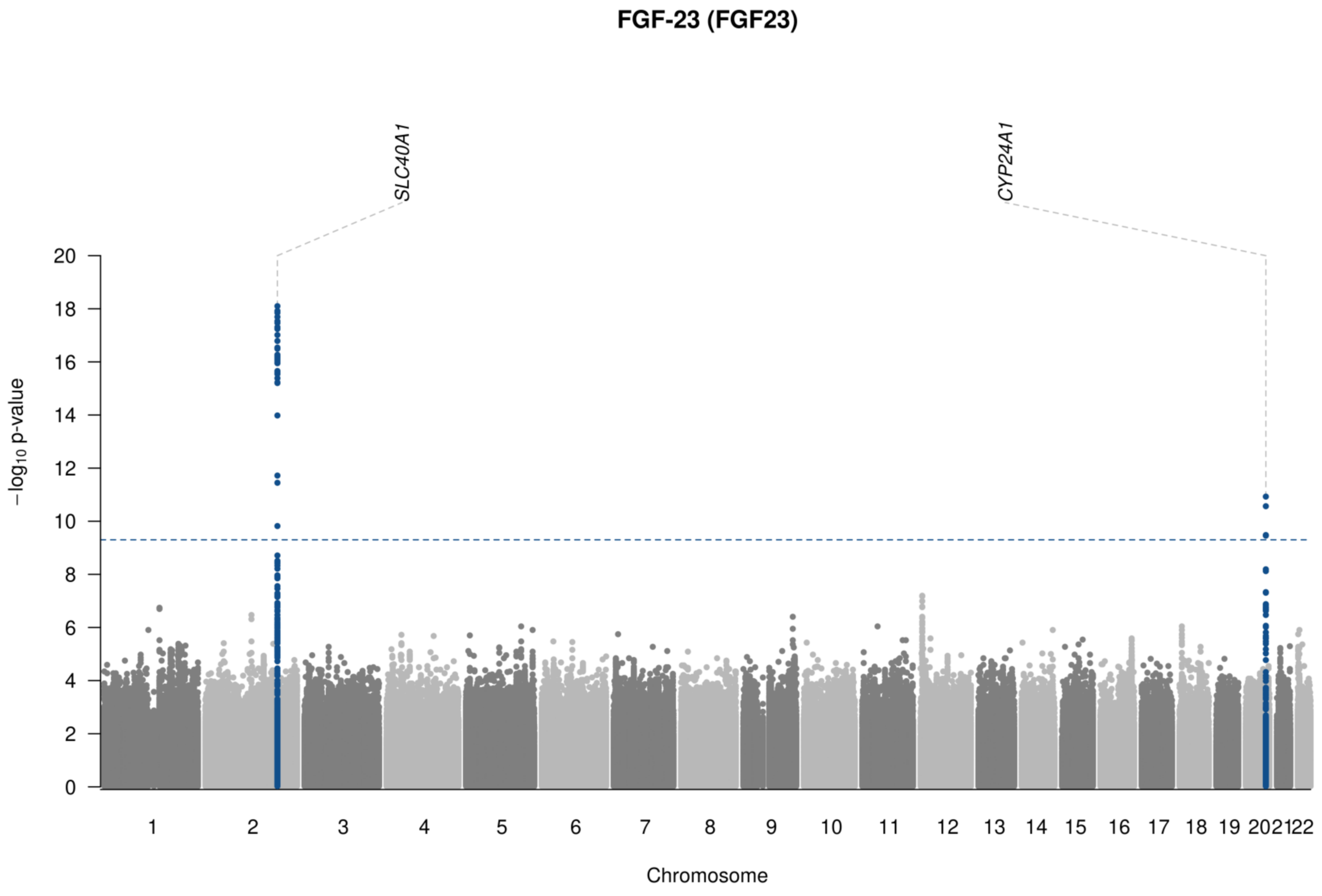
FGF-23 (FGF23)
SLC40A1
CYP24A1
−log10 p-value
20
18
16
14
12
10
8
6
4
2
0
1
2
3
4
5
6
7
8
9
10
11
12
13
14
15
16
17
18
19
20
21
22
Chromosome

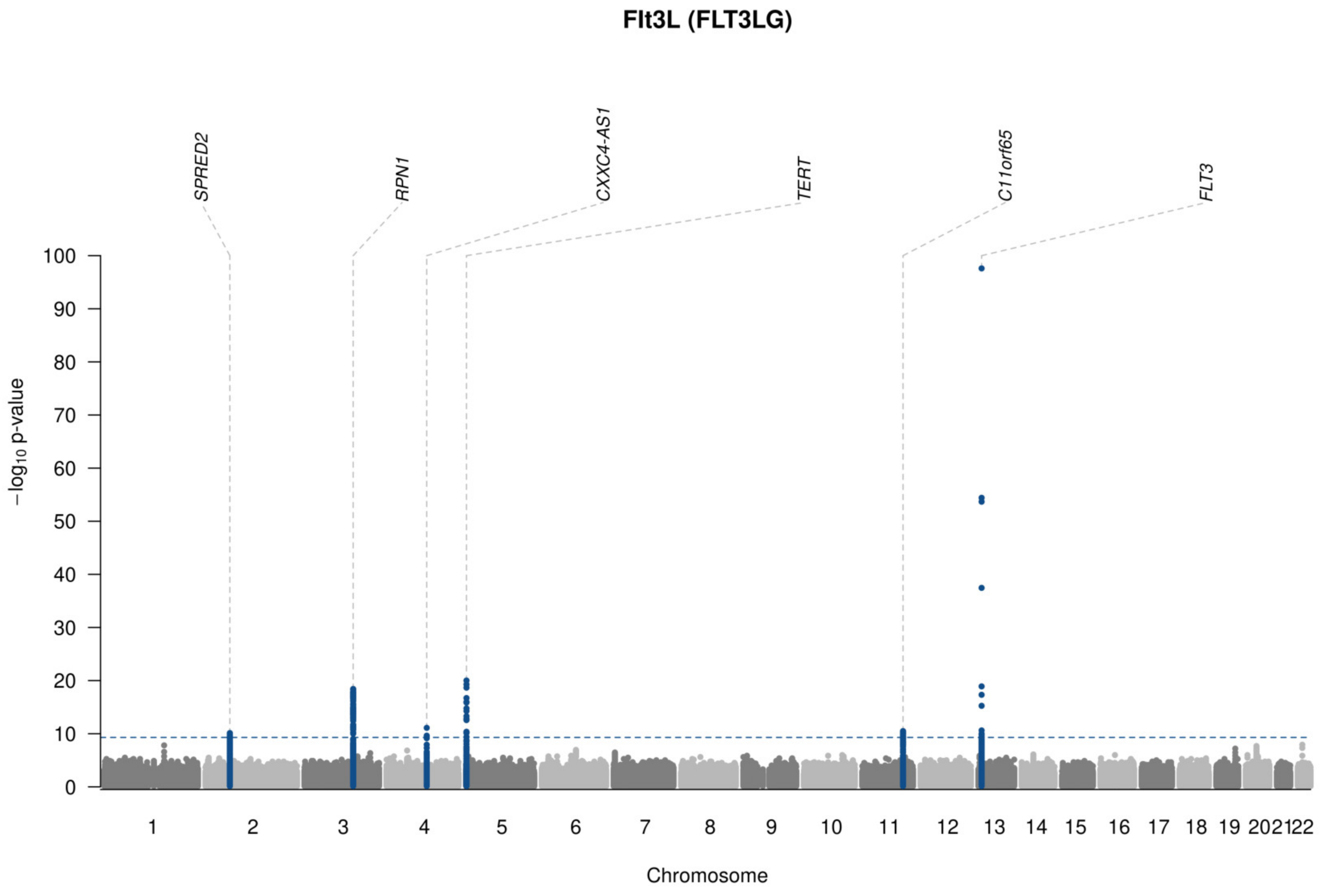

Flt3L (FLT3LG)
SPRED2
RPN1
CXXC4-AS1
TERT
C11orf65
FLT3
$-\log_{10}$ p-value
100
90
80
70
60
50
40
30
20
10
0
1
2
3
4
5
6
7
8
9
10
11
12
13
14
15
16
17
18
19
20
21
22
Chromosome

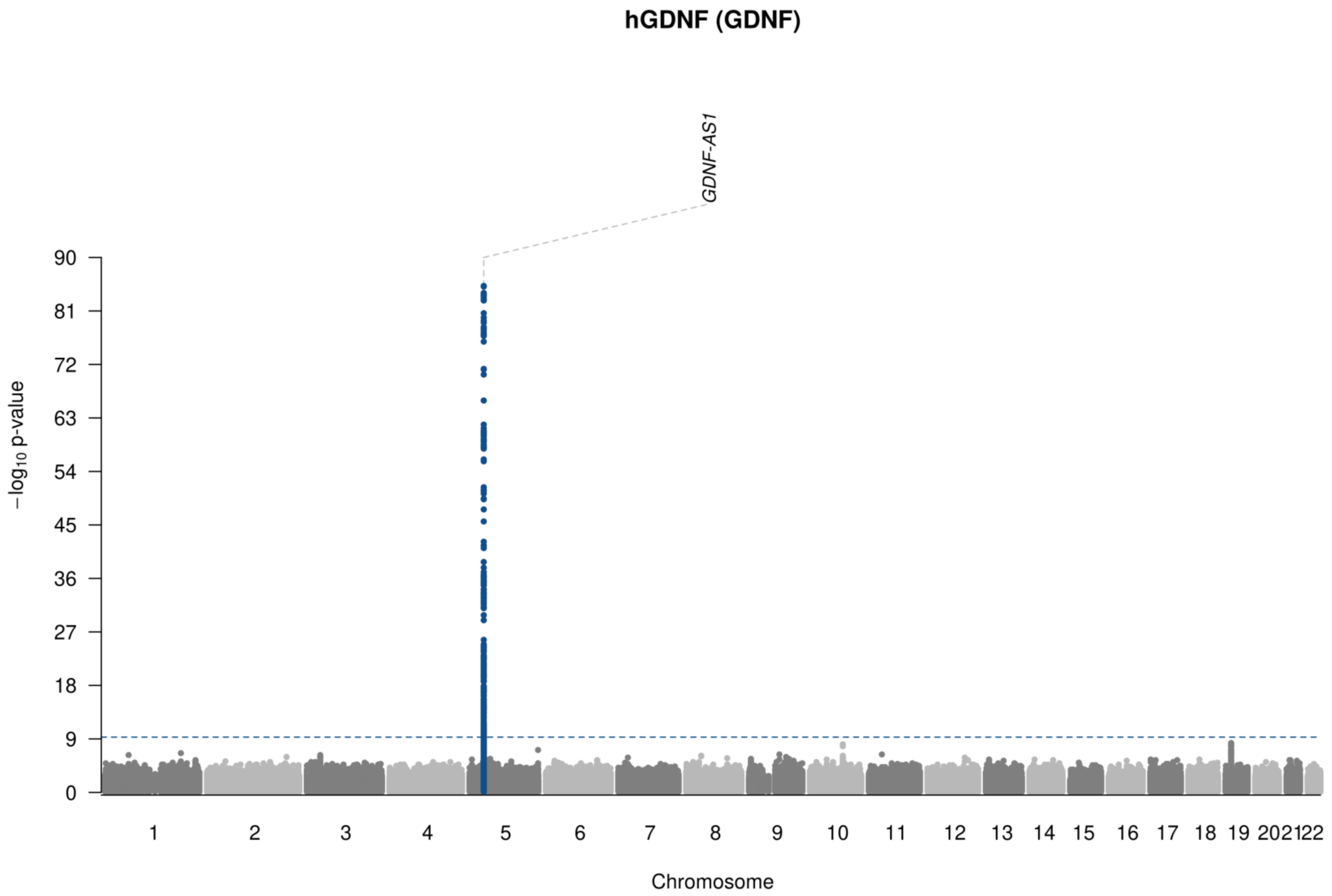
hGDNF (GDNF)
GDNF-AS1
90
81
72
63
54
45
36
27
18
9
0
−log10 p-value
1
2
3
4
5
6
7
8
9
10
11
12
13
14
15
16
17
18
19
20
21
22
Chromosome

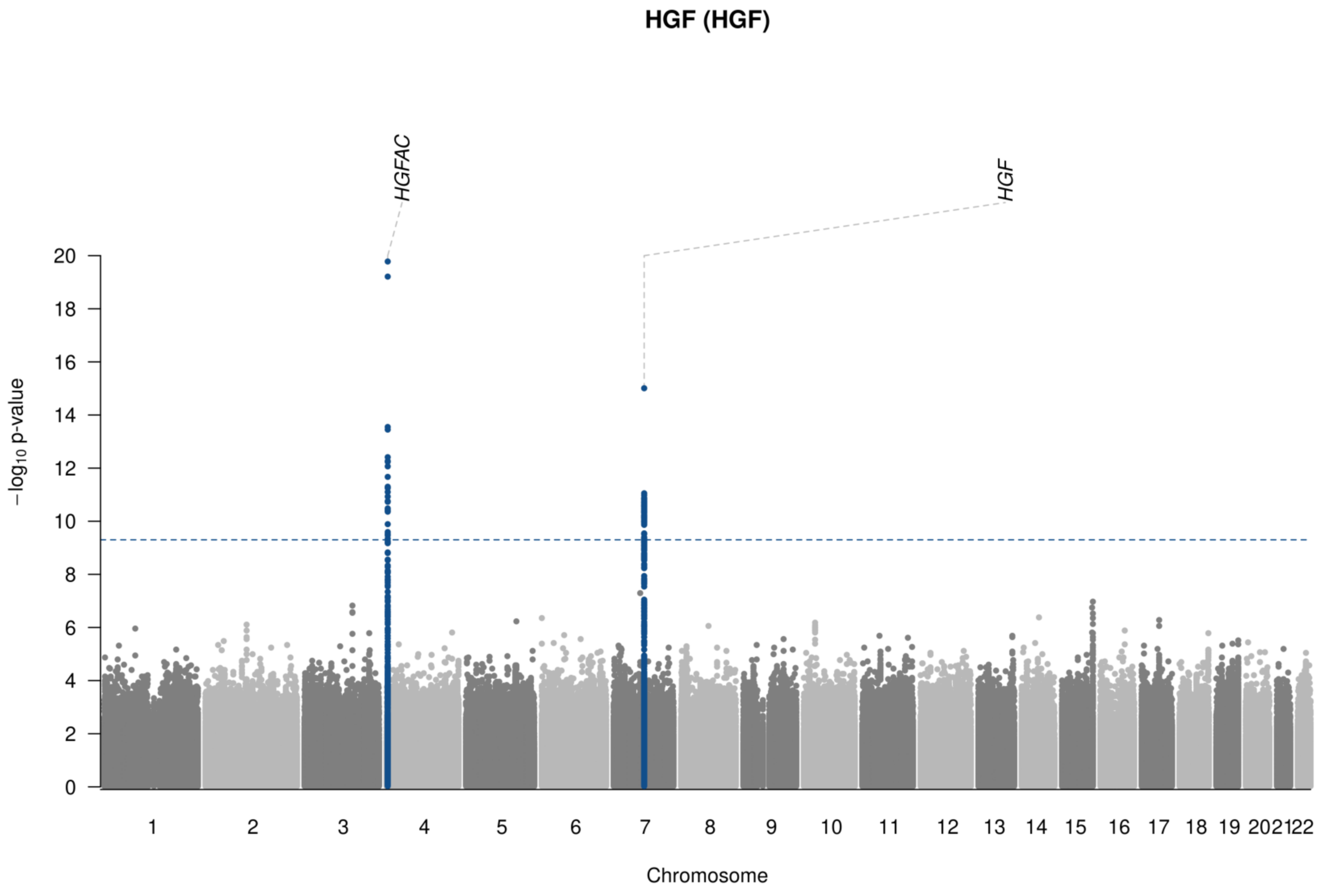

HGF (HGF)
HGFAC
HGF
−log10 p-value
20
18
16
14
12
10
8
6
4
2
0
1
2
3
4
5
6
7
8
9
10
11
12
13
14
15
16
17
18
19
20
21
22
Chromosome

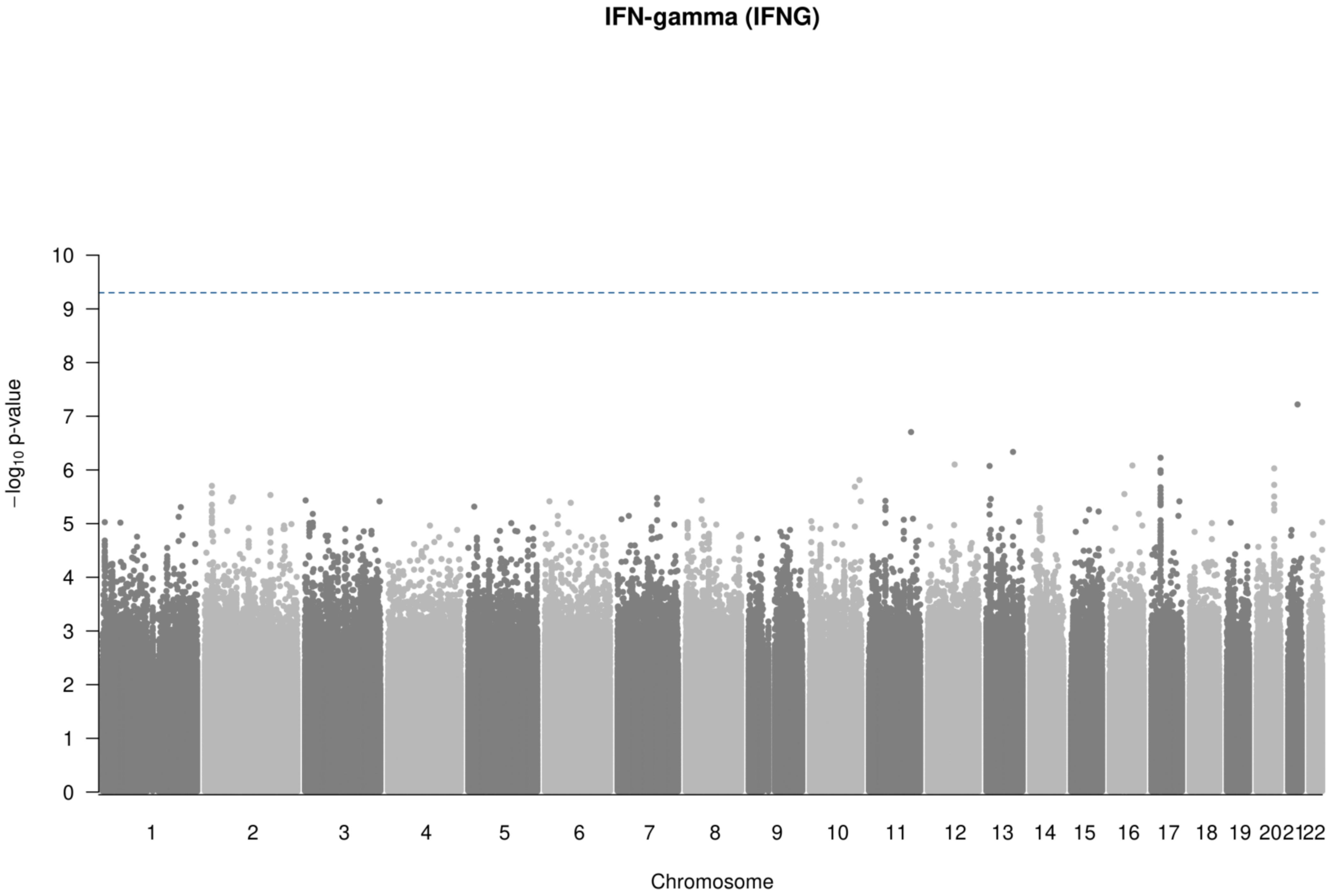
IFN-gamma (IFNG)
$-\log_{10}$ p-value
Chromosome
0 1 2 3 4 5 6 7 8 9 10
1 2 3 4 5 6 7 8 9 10 11 12 13 14 15 16 17 18 19 20 21 22

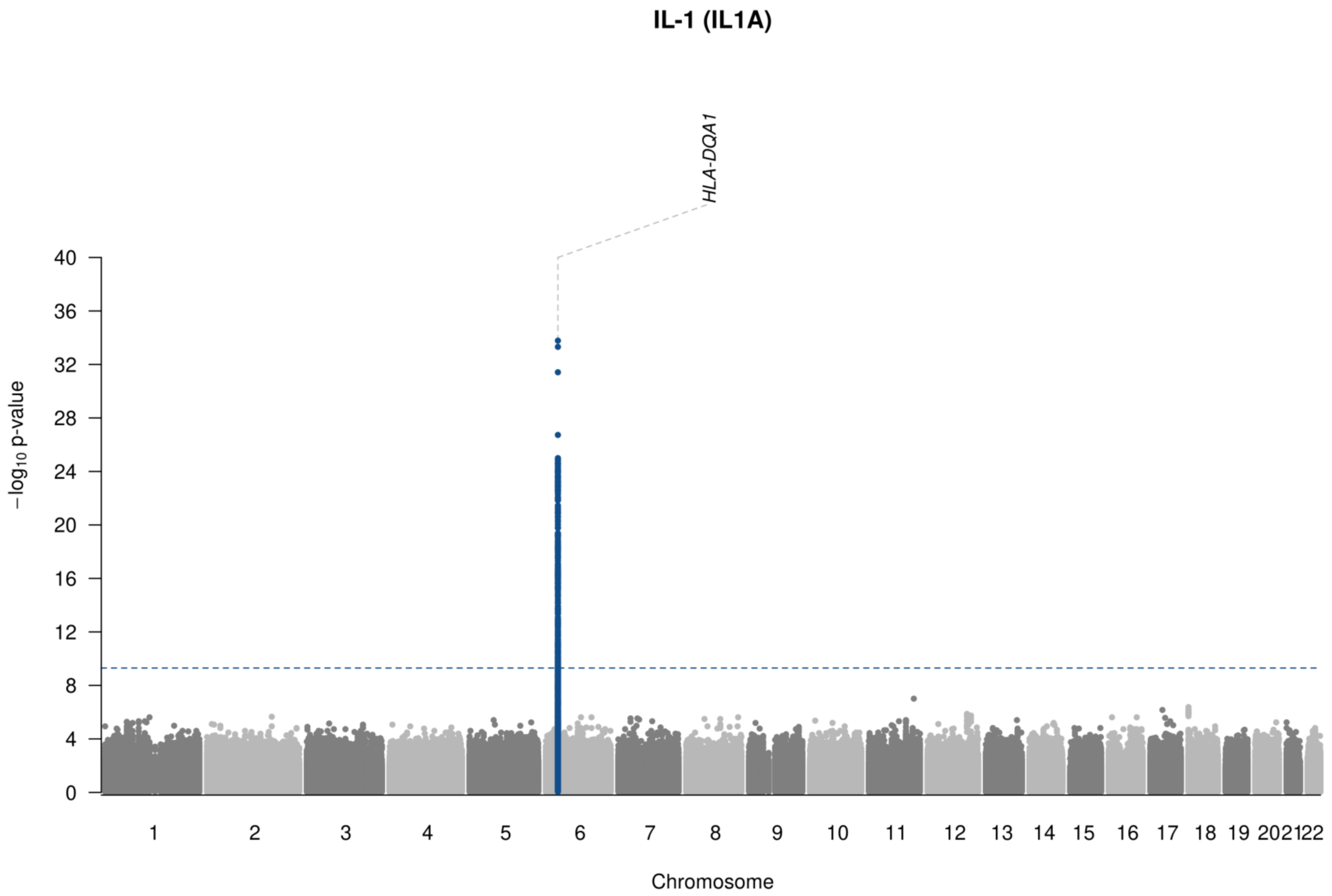

IL-1 (IL1A)
HLA-DQA1
−log10 p-value
0
4
8
12
16
20
24
28
32
36
40
1
2
3
4
5
6
7
8
9
10
11
12
13
14
15
16
17
18
19
20
21
22
Chromosome

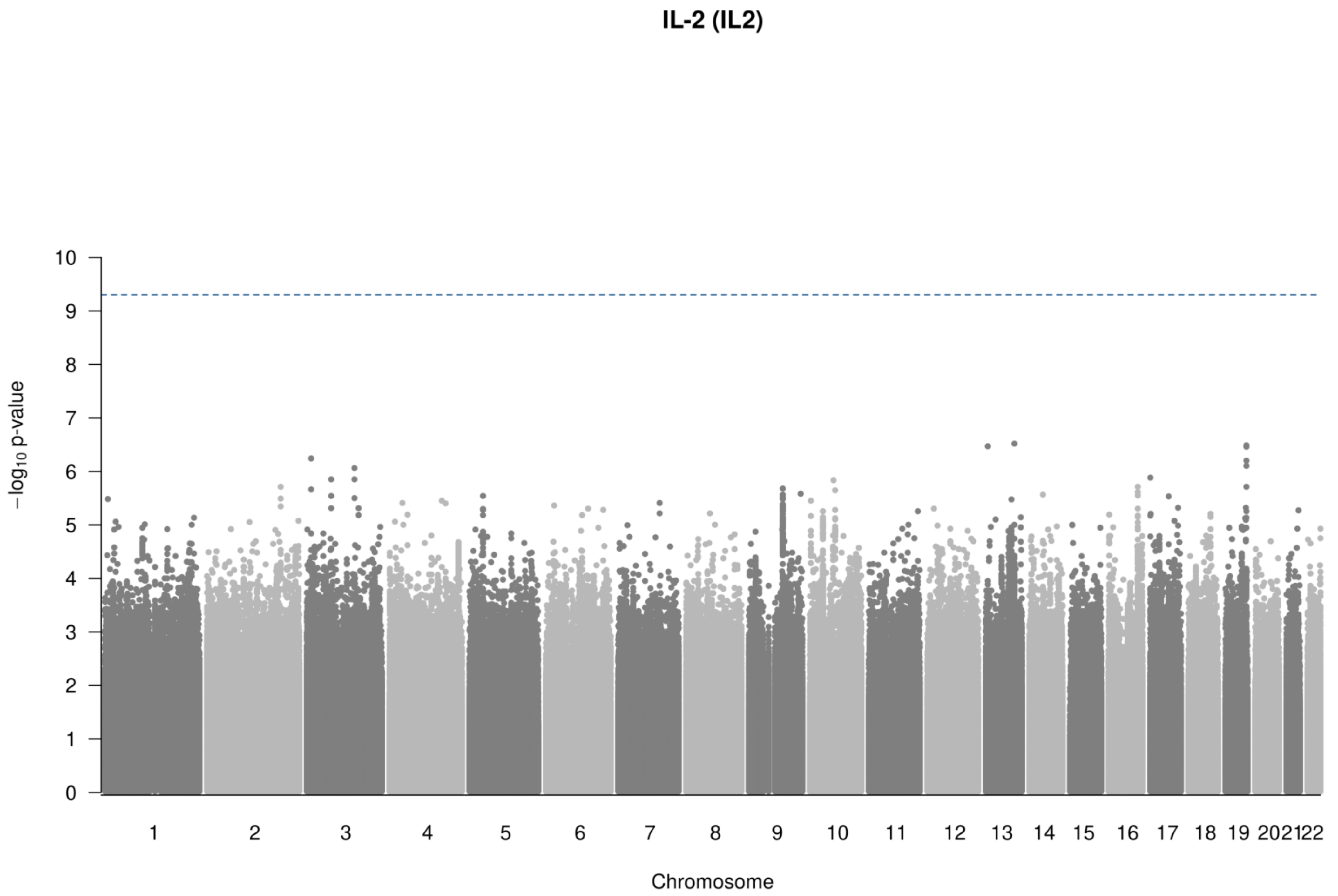
IL-2 (IL2)
−log10 p-value
0
1
2
3
4
5
6
7
8
9
10
1
2
3
4
5
6
7
8
9
10
11
12
13
14
15
16
17
18
19
20
21
22
Chromosome

IL-2RB (IL2RB)

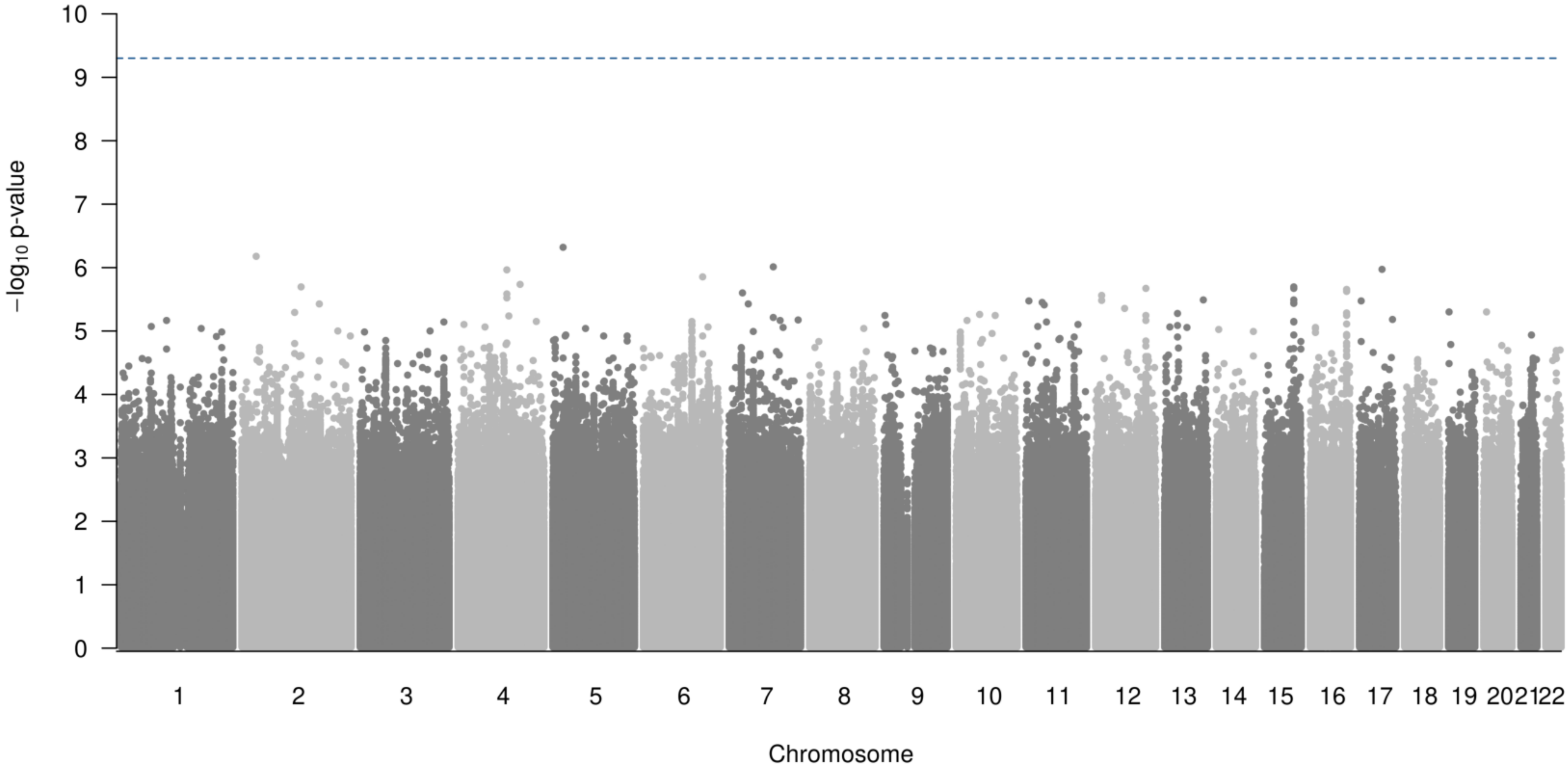

10
9
8
7
6
5
4
3
2
1
0
$-\log_{10}$ p-value
1
2
3
4
5
6
7
8
9
10
11
12
13
14
15
16
17
18
19
20
21
22
Chromosome

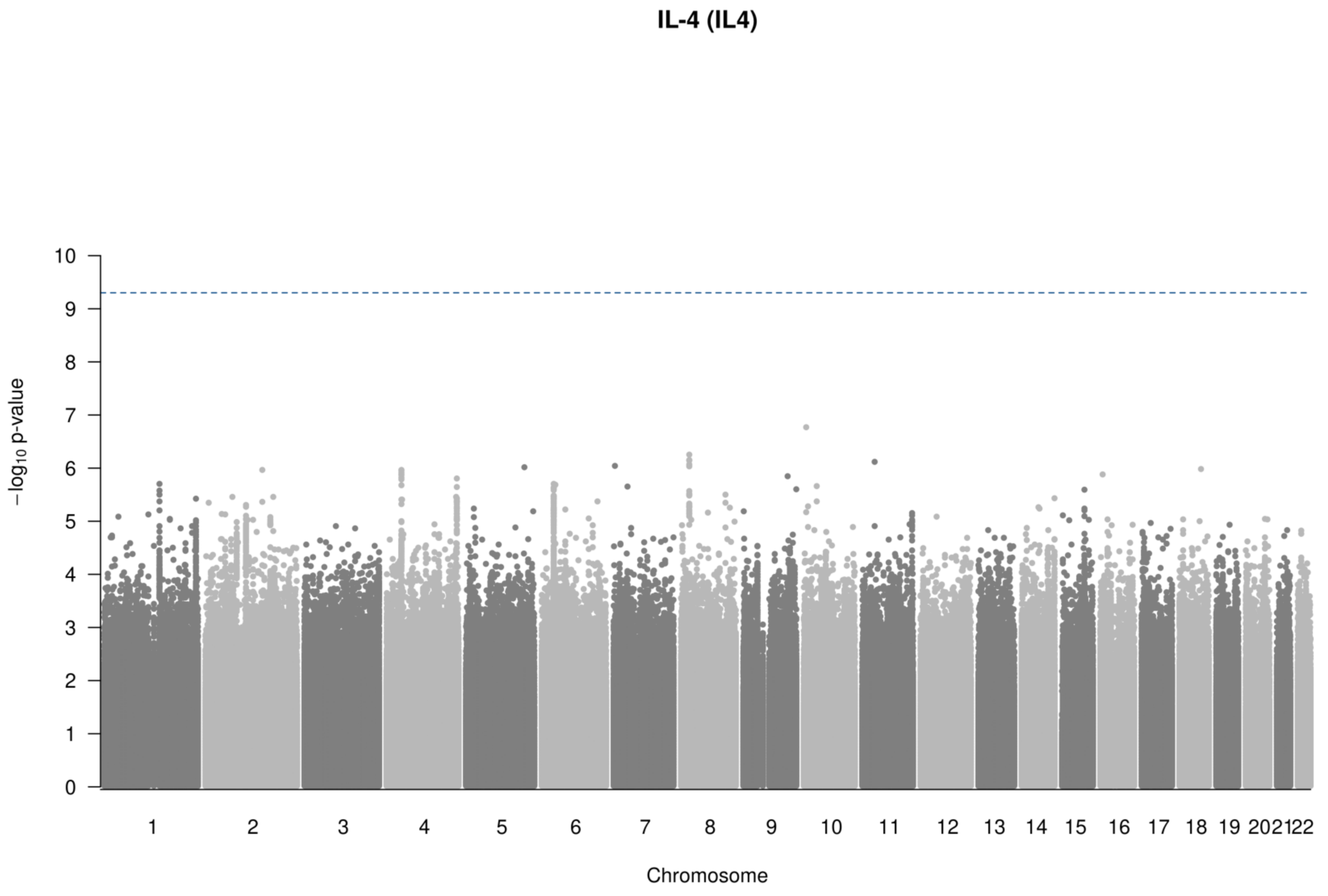

IL-4 (IL4)
10
9
8
7
6
5
4
3
2
1
0
−log10 p-value
1
2
3
4
5
6
7
8
9
10
11
12
13
14
15
16
17
18
19
20
21
22
Chromosome

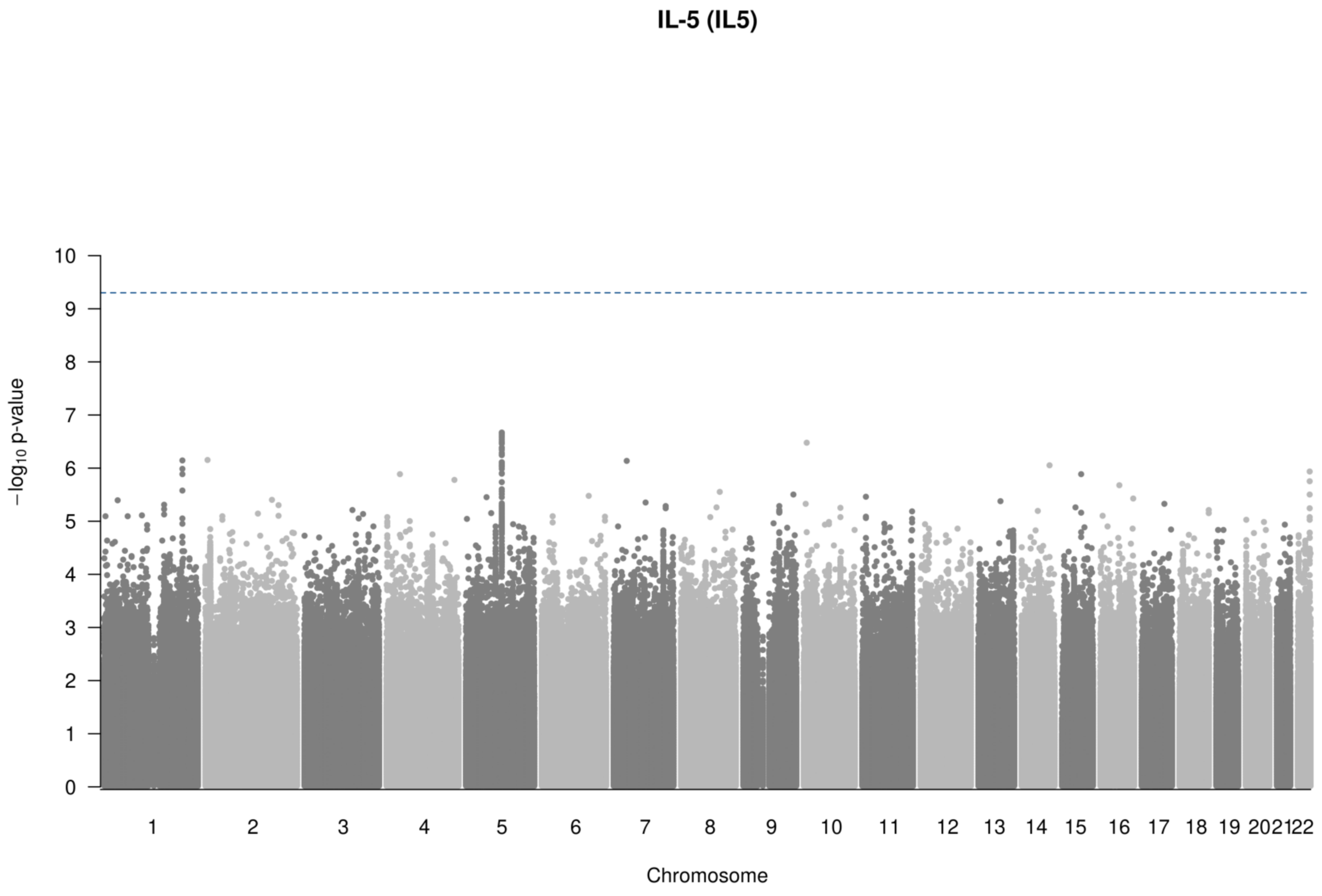
IL-5 (IL5)
$-\log_{10}$ p-value
0
1
2
3
4
5
6
7
8
9
10
1
2
3
4
5
6
7
8
9
10
11
12
13
14
15
16
17
18
19
20
21
22
Chromosome

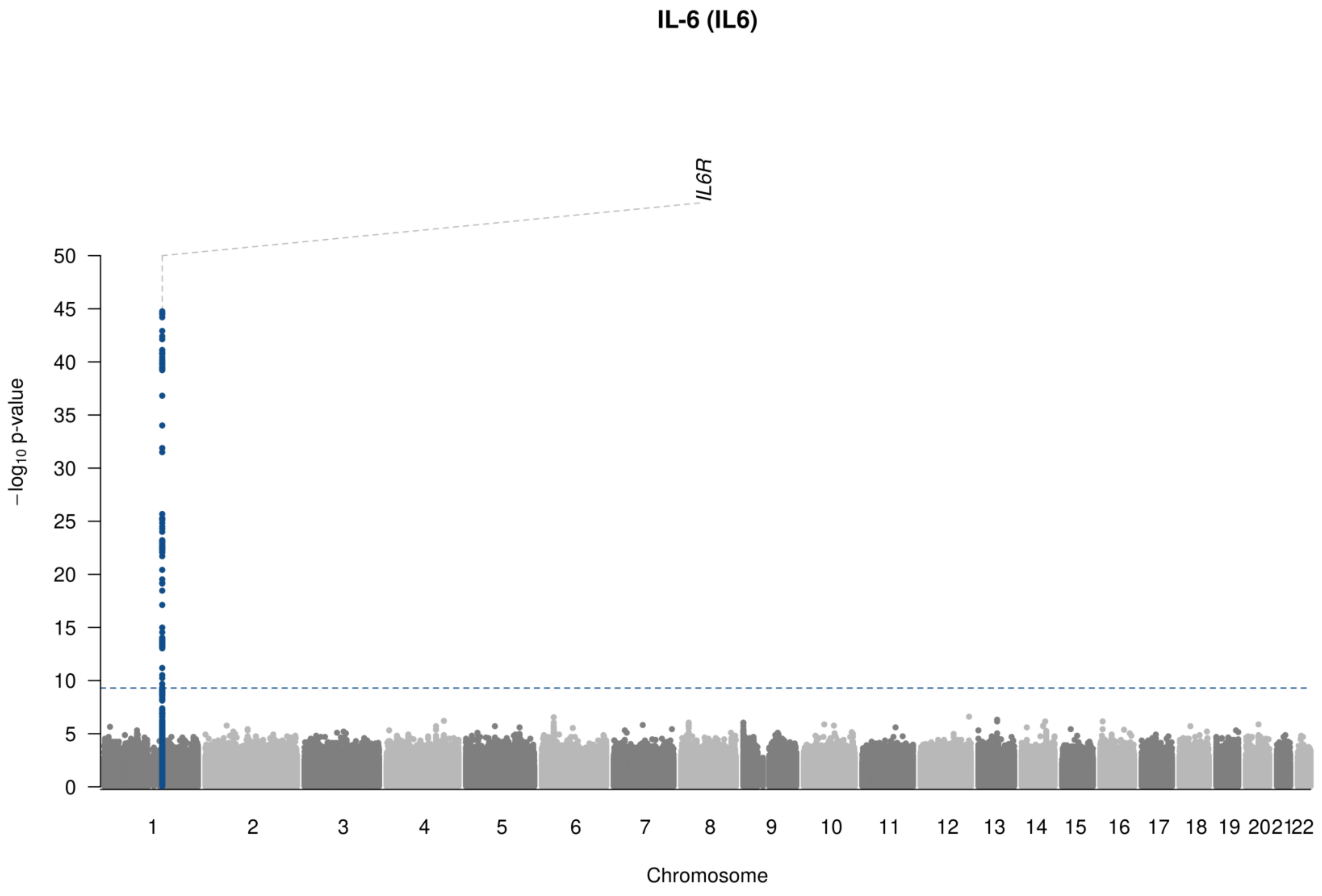

IL-6 (IL6)
IL6R
50
45
40
35
30
25
20
15
10
5
0
−log10 p-value
1
2
3
4
5
6
7
8
9
10
11
12
13
14
15
16
17
18
19
202122
Chromosome

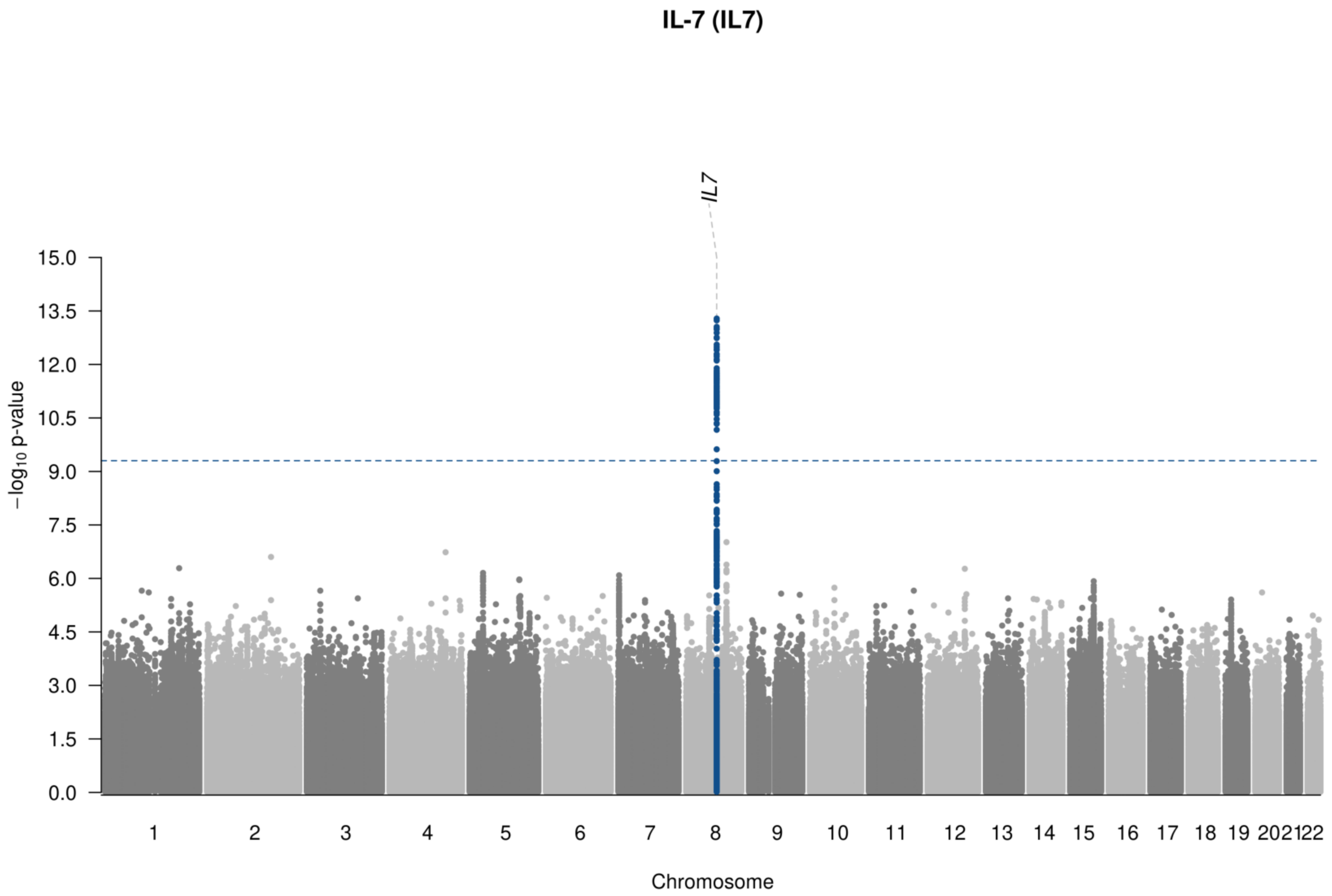
IL-7 (IL7)
IL7
−log10 p-value
15.0
13.5
12.0
10.5
9.0
7.5
6.0
4.5
3.0
1.5
0.0
1
2
3
4
5
6
7
8
9
10
11
12
13
14
15
16
17
18
19
20
21
22
Chromosome

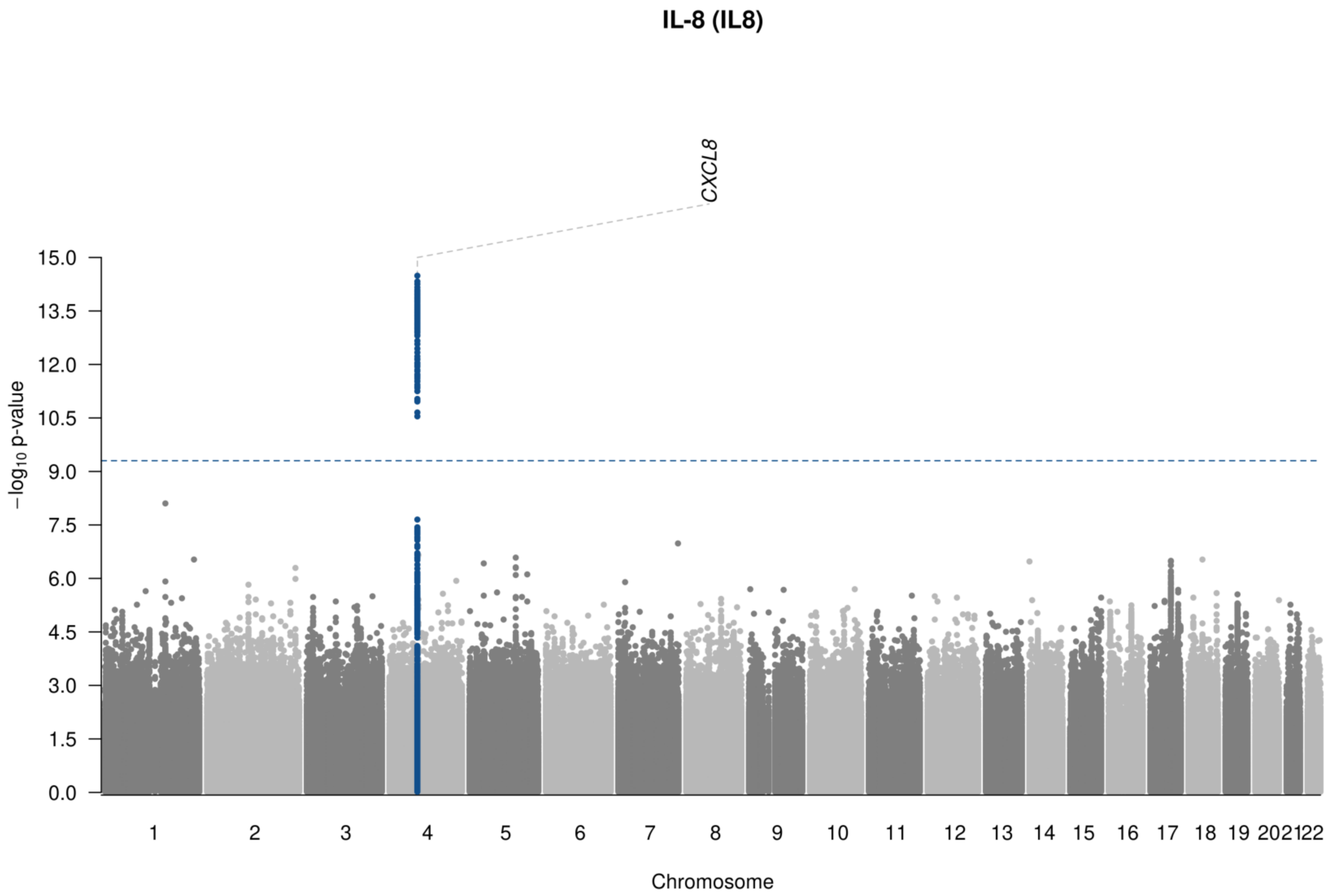
IL-8 (IL8)
CXCL8
−log10 p-value
15.0
13.5
12.0
10.5
9.0
7.5
6.0
4.5
3.0
1.5
0.0
1
2
3
4
5
6
7
8
9
10
11
12
13
14
15
16
17
18
19
20
21
22
Chromosome

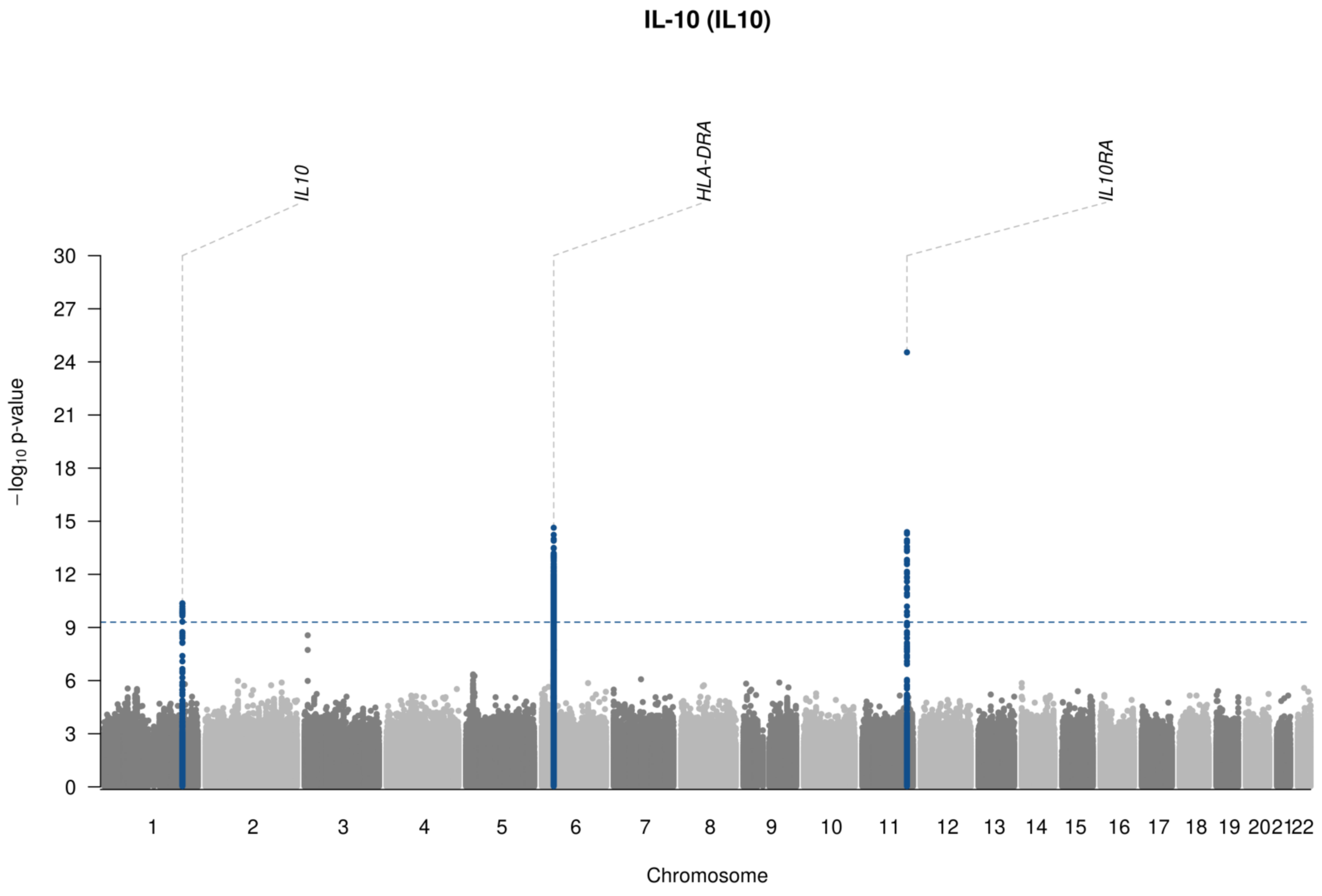

IL-10 (IL10)
IL10
HLA-DRA
IL10RA
30
27
24
21
18
15
12
9
6
3
0
−log10 p-value
1
2
3
4
5
6
7
8
9
10
11
12
13
14
15
16
17
18
19
20
21
22
Chromosome

IL-10RA (IL10RA)

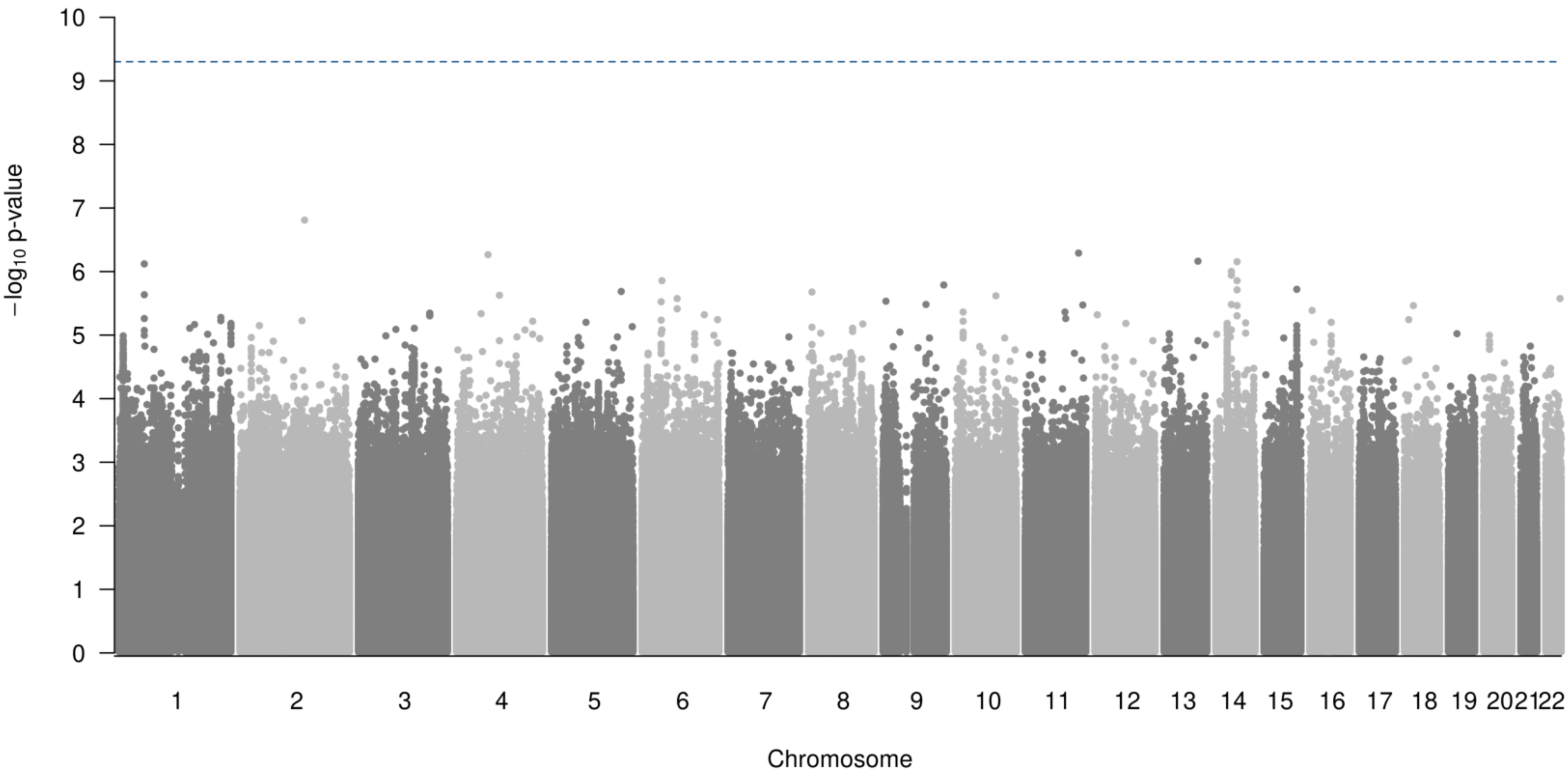

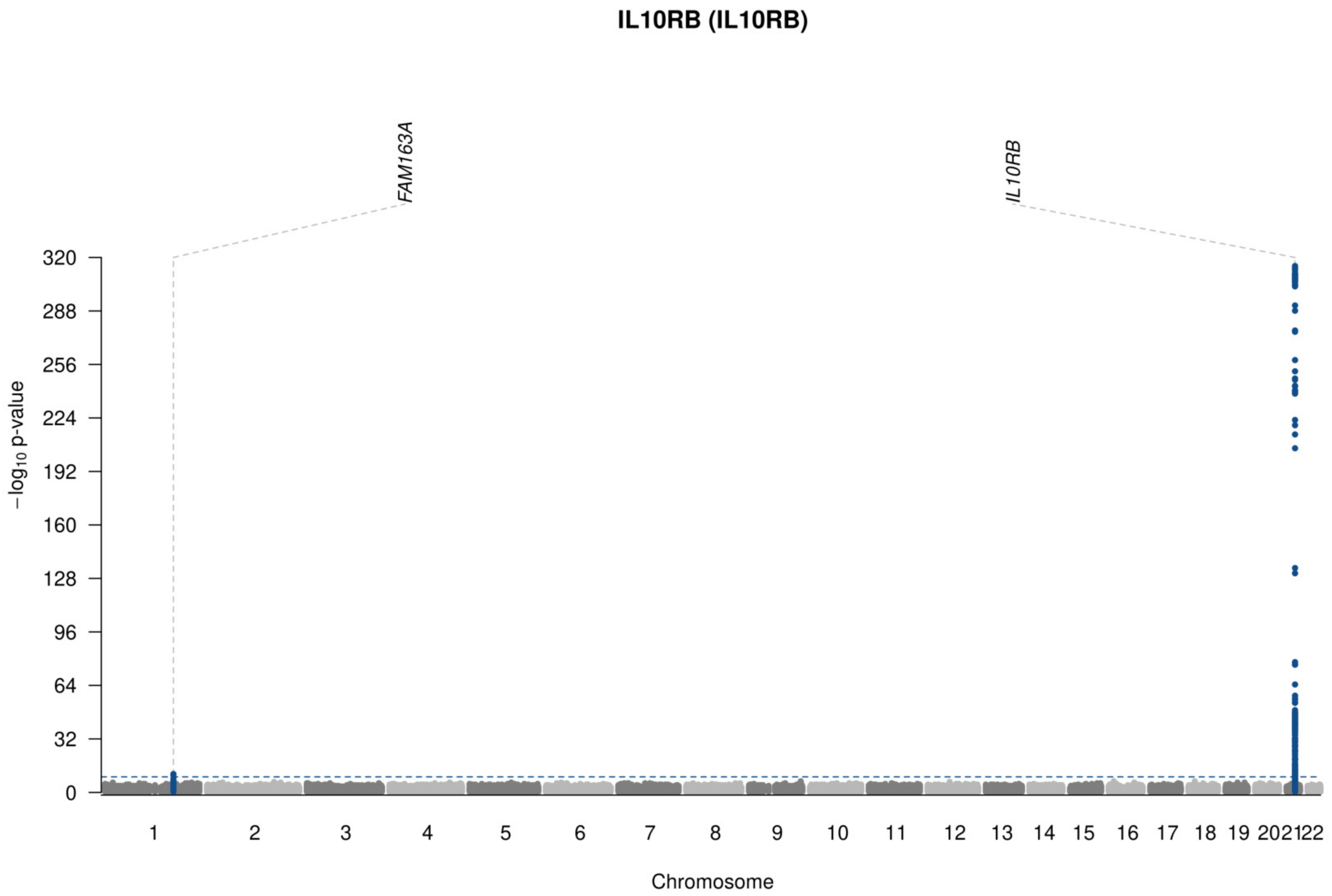

IL10RB (IL10RB)
FAM163A
IL10RB
−log10 p-value
320
288
256
224
192
160
128
96
64
32
0
1
2
3
4
5
6
7
8
9
10
11
12
13
14
15
16
17
18
19
20
21
22
Chromosome

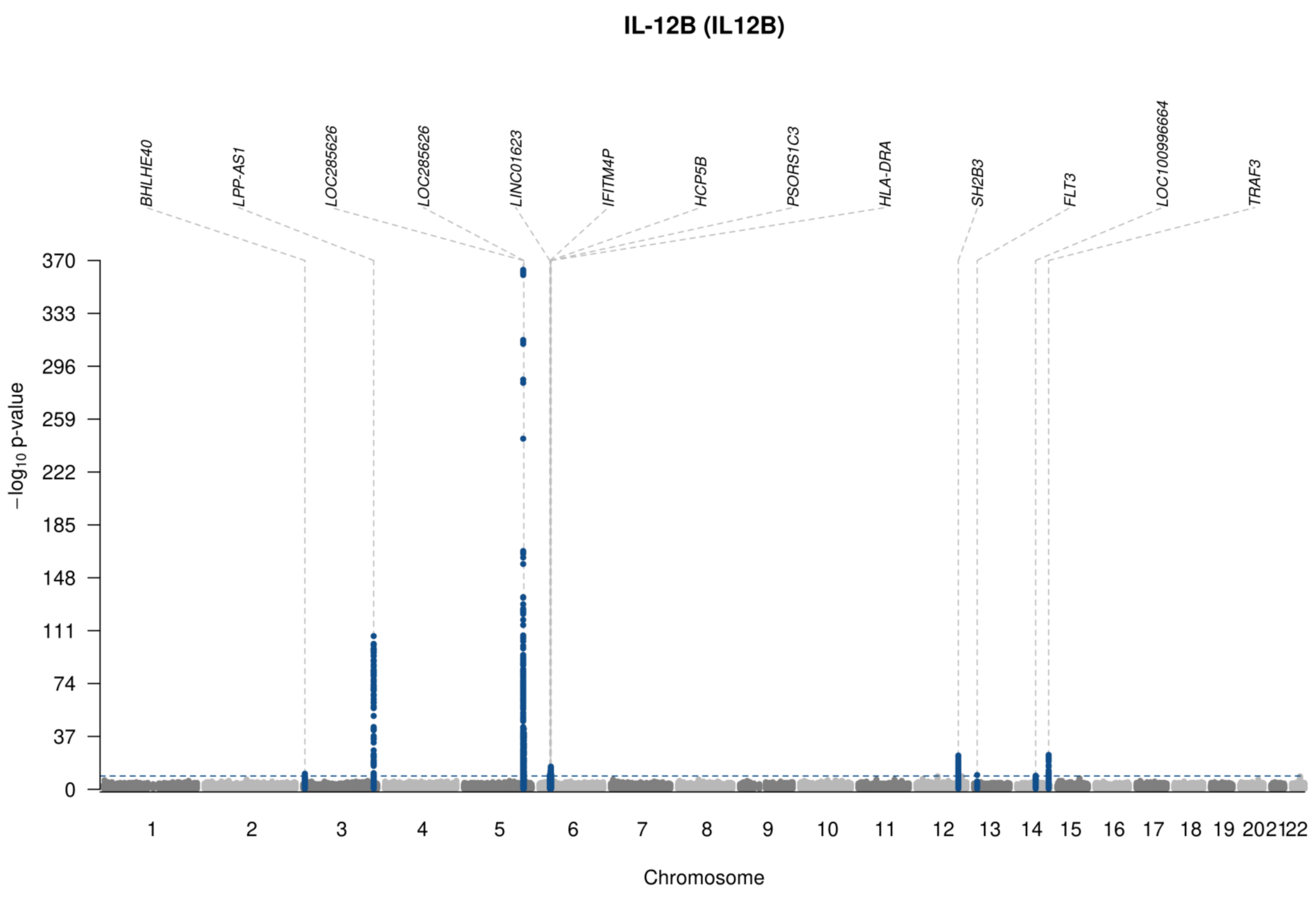

IL-12B (IL12B)
BHLHE40
LPP-AS1
LOC285626
LOC285626
LINC01623
IFITM4P
HCP5B
PSORS1C3
HLA-DRA
SH2B3
FLT3
LOC100996664
TRAF3
370
333
296
259
222
185
148
111
74
37
0
−log10 p-value
1 2 3 4 5 6 7 8 9 10 11 12 13 14 15 16 17 18 19 20 21 22
Chromosome

IL-13 (IL13)

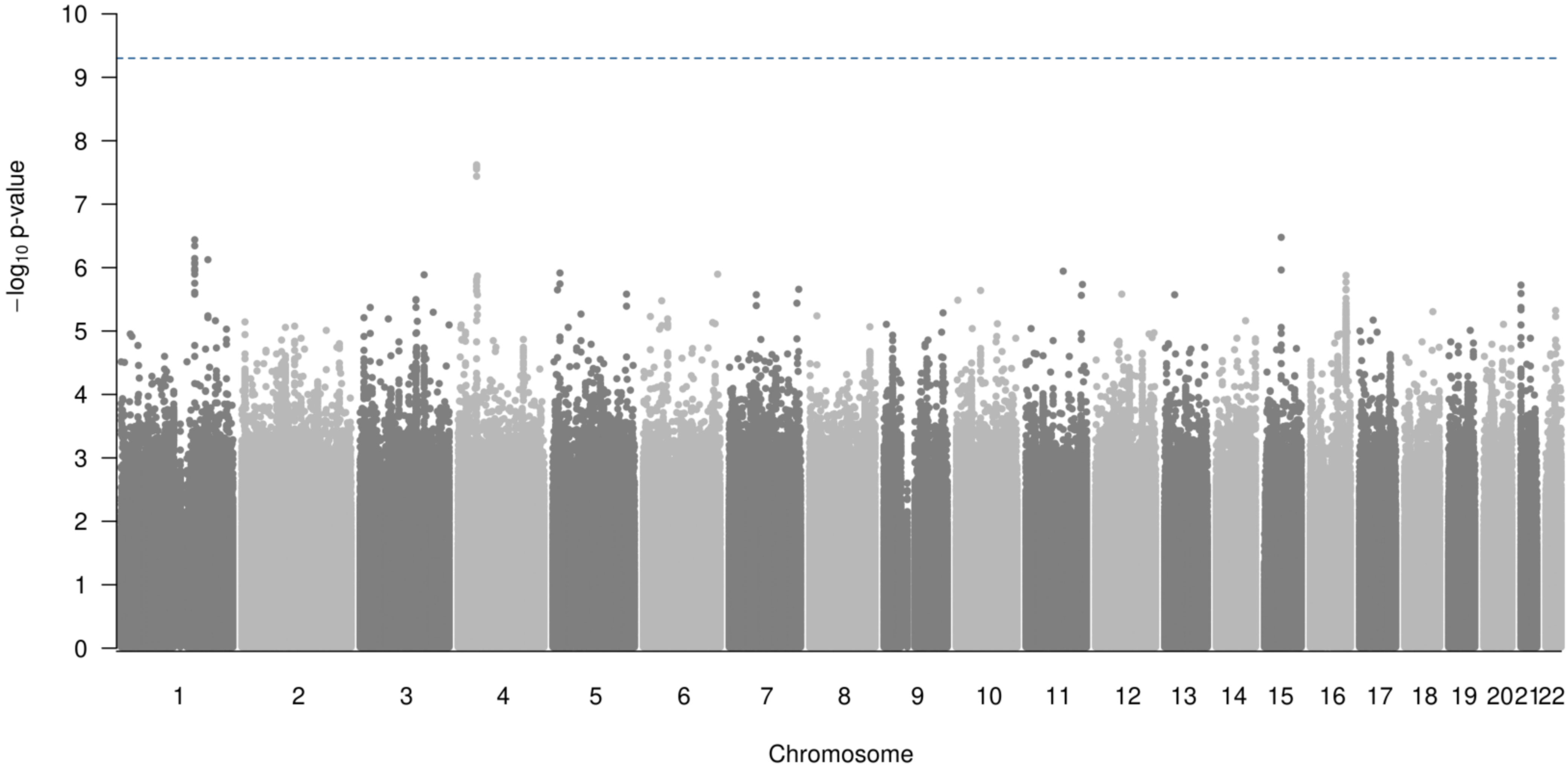

10
9
8
7
6
5
4
3
2
1
0
−log10 p-value
1
2
3
4
5
6
7
8
9
10
11
12
13
14
15
16
17
18
19
20
21
22
Chromosome

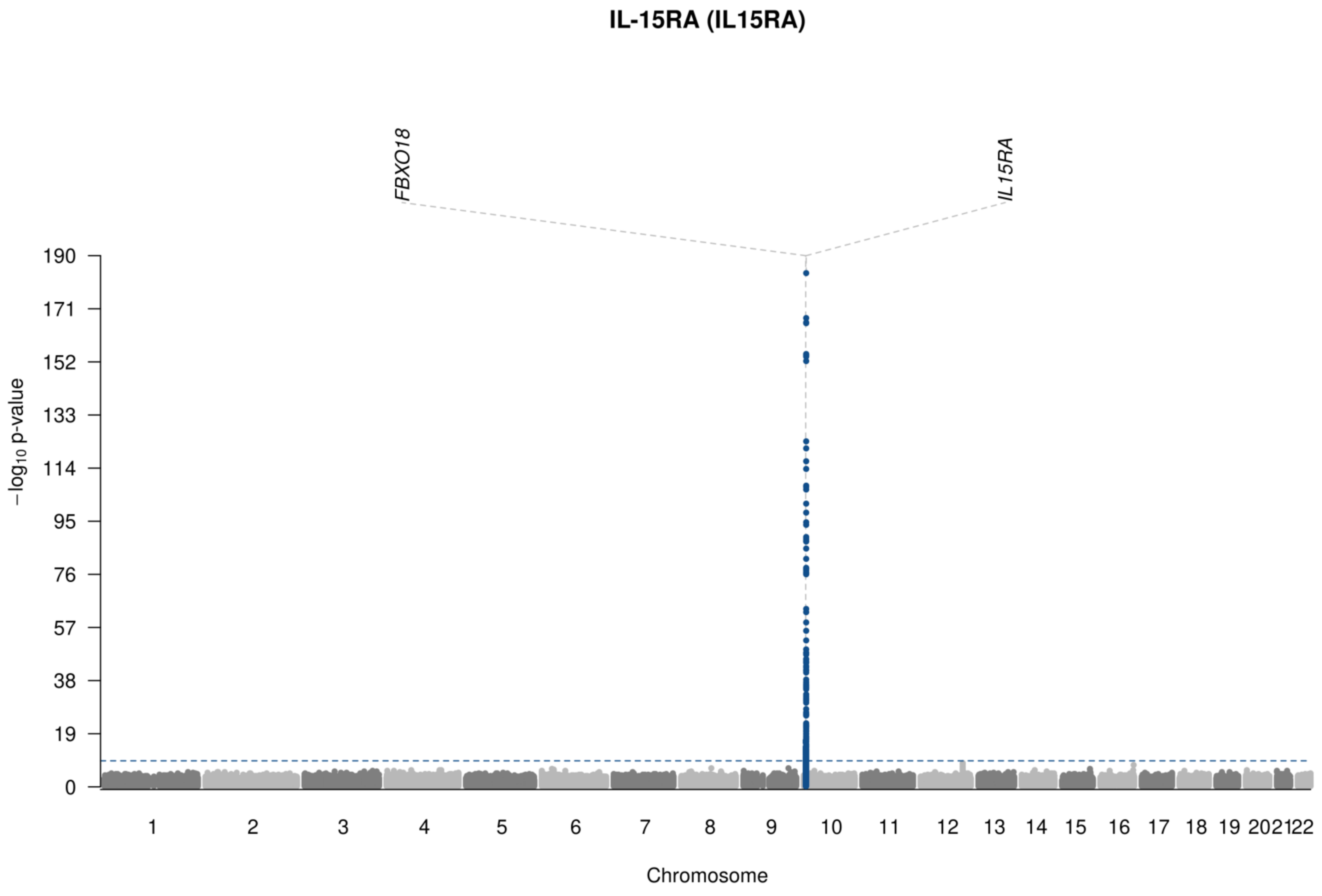

IL-15RA (IL15RA)
FBXO18
IL15RA
190
171
152
133
114
95
76
57
38
19
0
$-\log_{10}$ p-value
1
2
3
4
5
6
7
8
9
10
11
12
13
14
15
16
17
18
19
202122
Chromosome

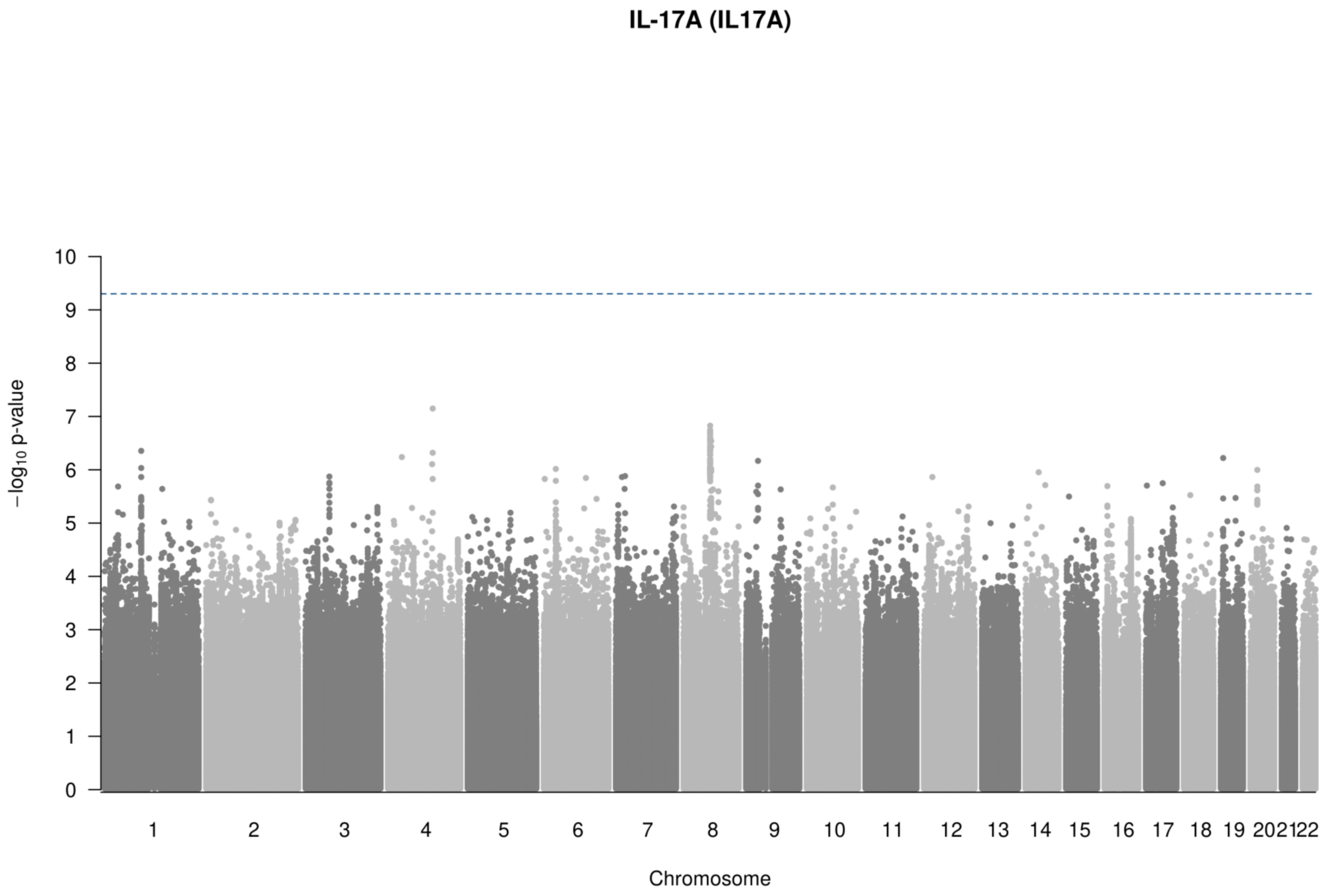
IL-17A (IL17A)
−log10 p-value
0
1
2
3
4
5
6
7
8
9
10
1
2
3
4
5
6
7
8
9
10
11
12
13
14
15
16
17
18
19
20
21
22
Chromosome

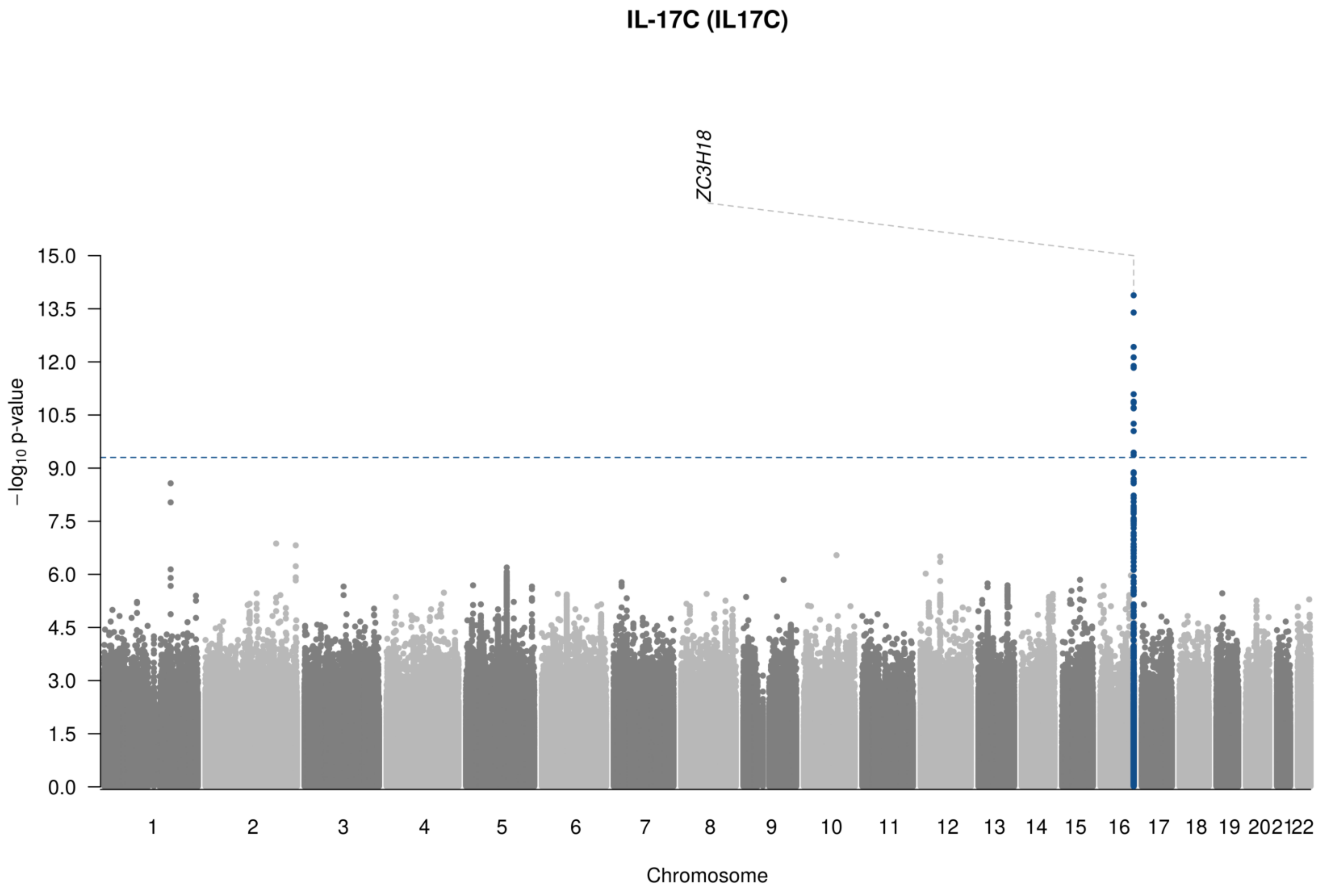
IL-17C (IL17C)
ZC3H18
15.0
13.5
12.0
10.5
9.0
7.5
6.0
4.5
3.0
1.5
0.0
$-\log_{10}$ p-value
1
2
3
4
5
6
7
8
9
10
11
12
13
14
15
16
17
18
19
20
21
22
Chromosome

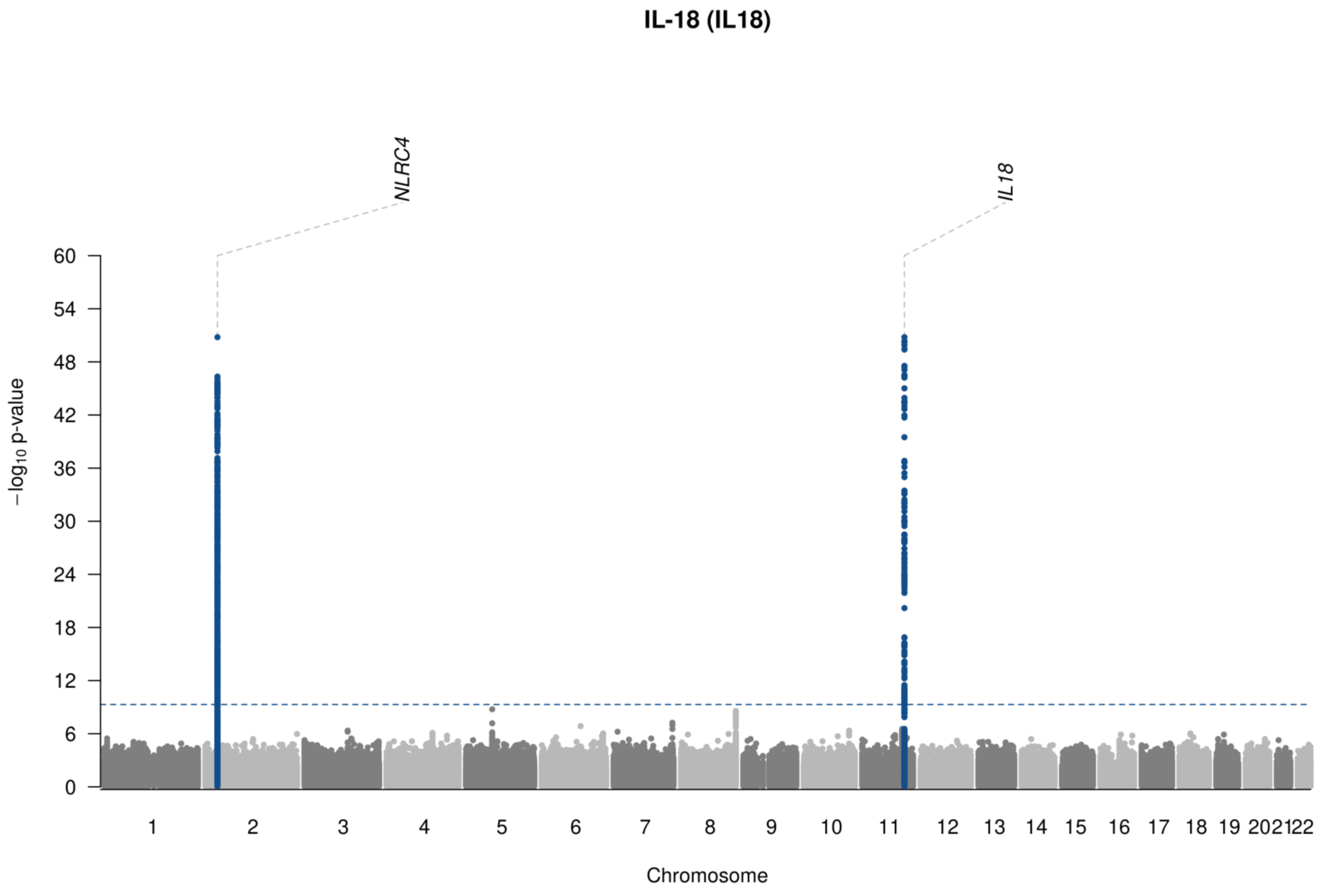
IL-18 (IL18)
NLRC4
IL18
−log10 p-value
60
54
48
42
36
30
24
18
12
6
0
1
2
3
4
5
6
7
8
9
10
11
12
13
14
15
16
17
18
19
20
21
22
Chromosome

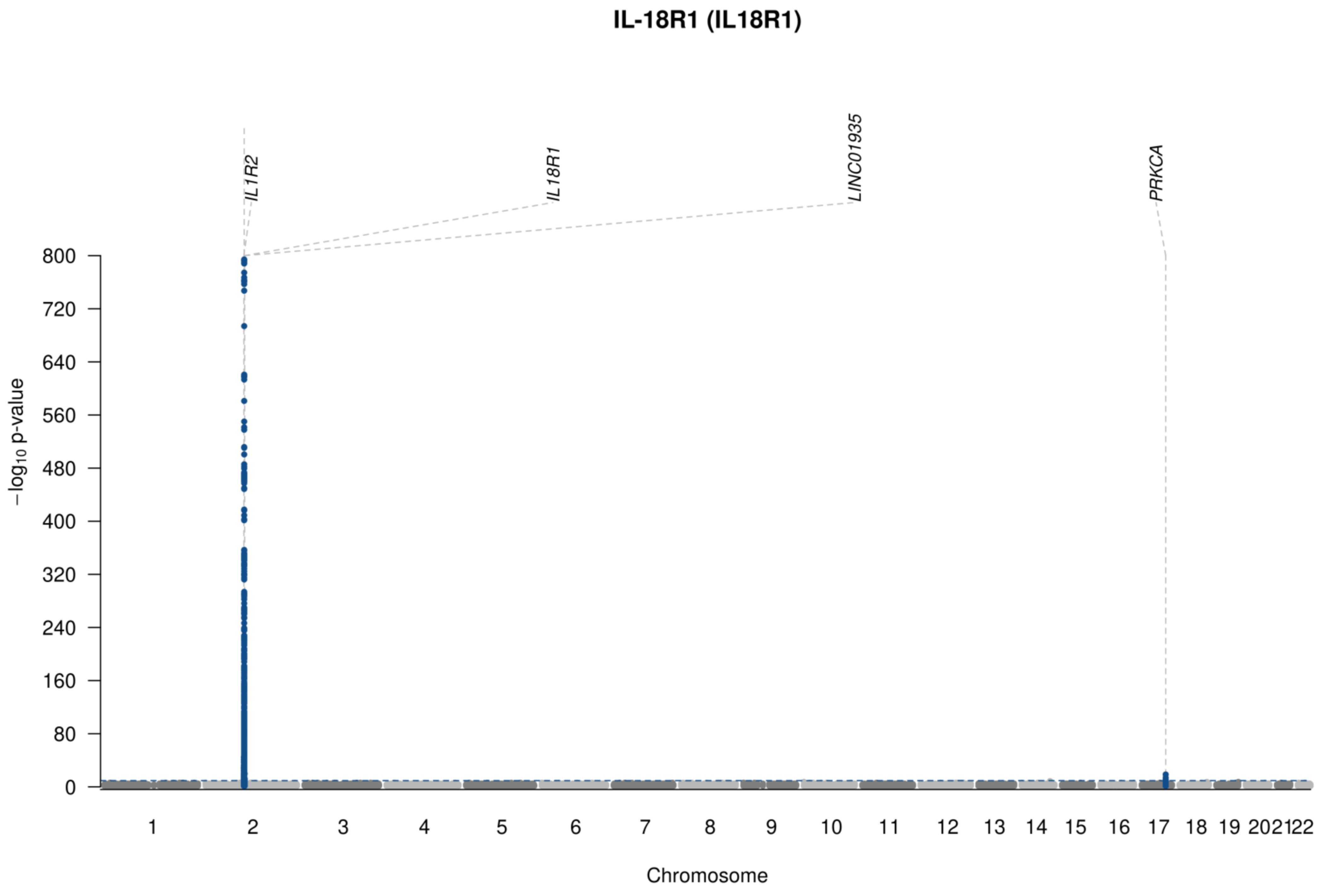
IL-18R1 (IL18R1)
IL1R2
IL18R1
LINC01935
PRKCA
−log10 p-value
800
720
640
560
480
400
320
240
160
80
0
1
2
3
4
5
6
7
8
9
10
11
12
13
14
15
16
17
18
19
202122
Chromosome

IL-20 (IL20)

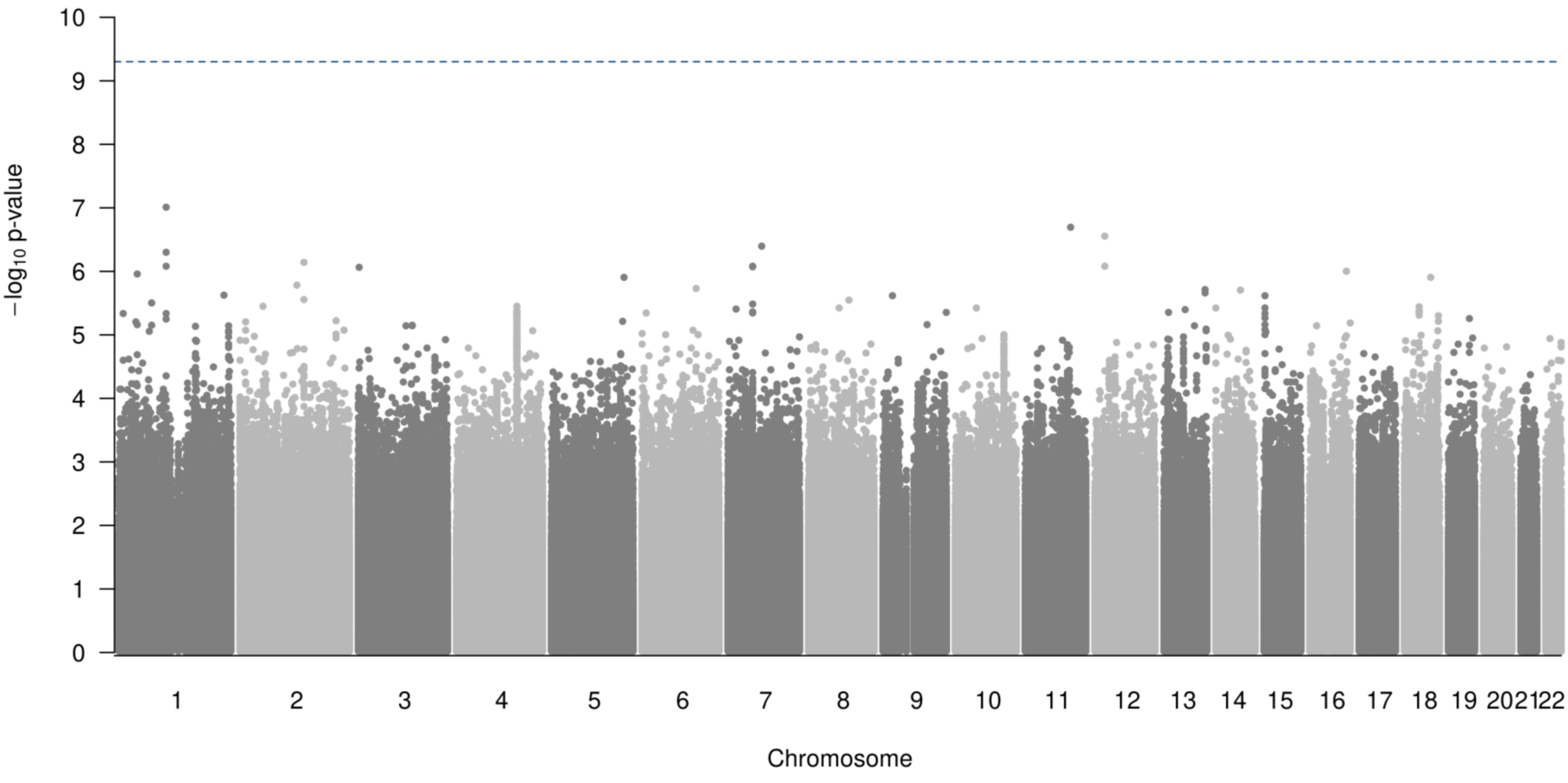

10
9
8
7
6
5
4
3
2
1
0
$-\log_{10}$ p-value
1
2
3
4
5
6
7
8
9
10
11
12
13
14
15
16
17
18
19
20
21
22
Chromosome

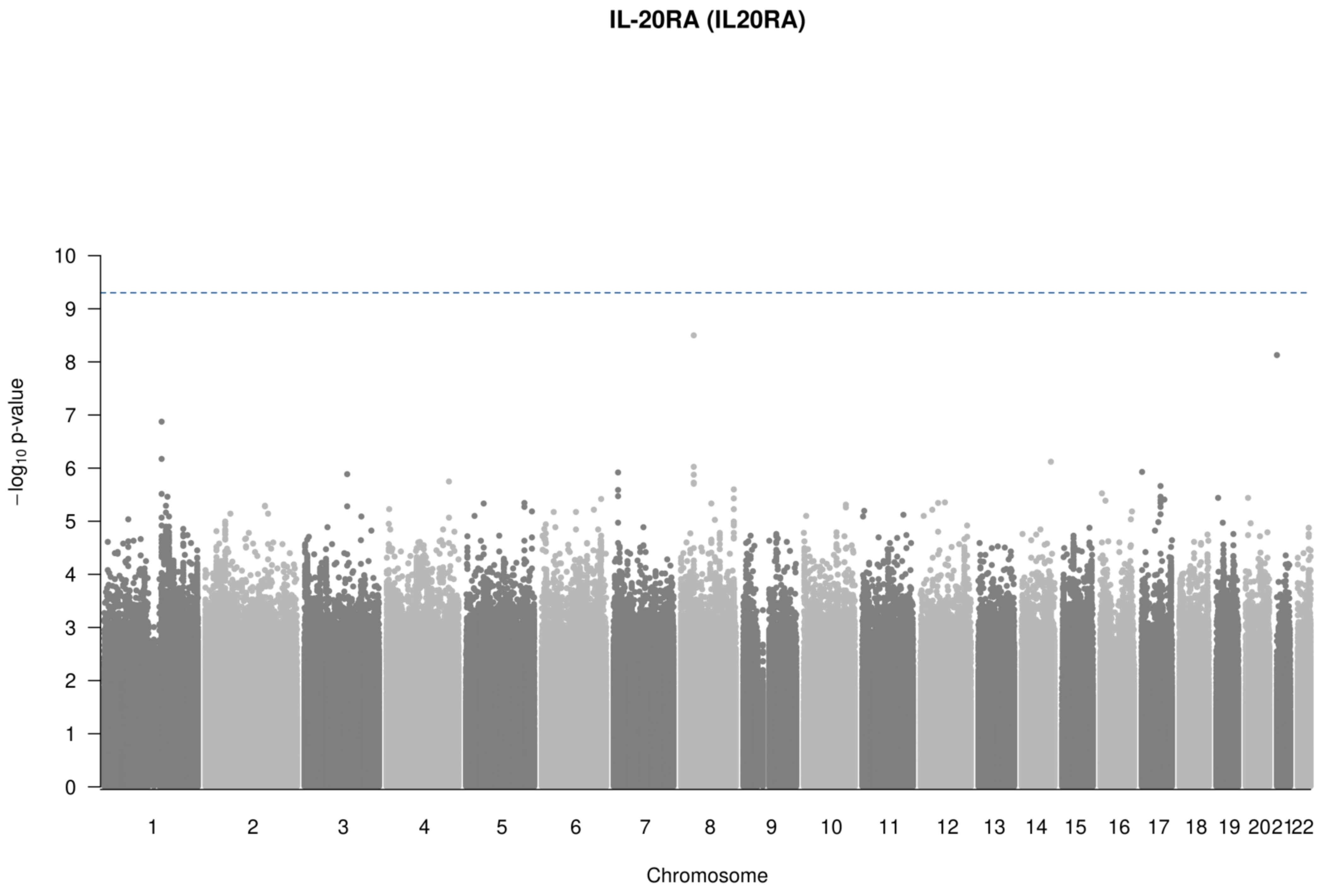

IL-20RA (IL20RA)
−log10 p-value
0
1
2
3
4
5
6
7
8
9
10
1
2
3
4
5
6
7
8
9
10
11
12
13
14
15
16
17
18
19
20
21
22
Chromosome

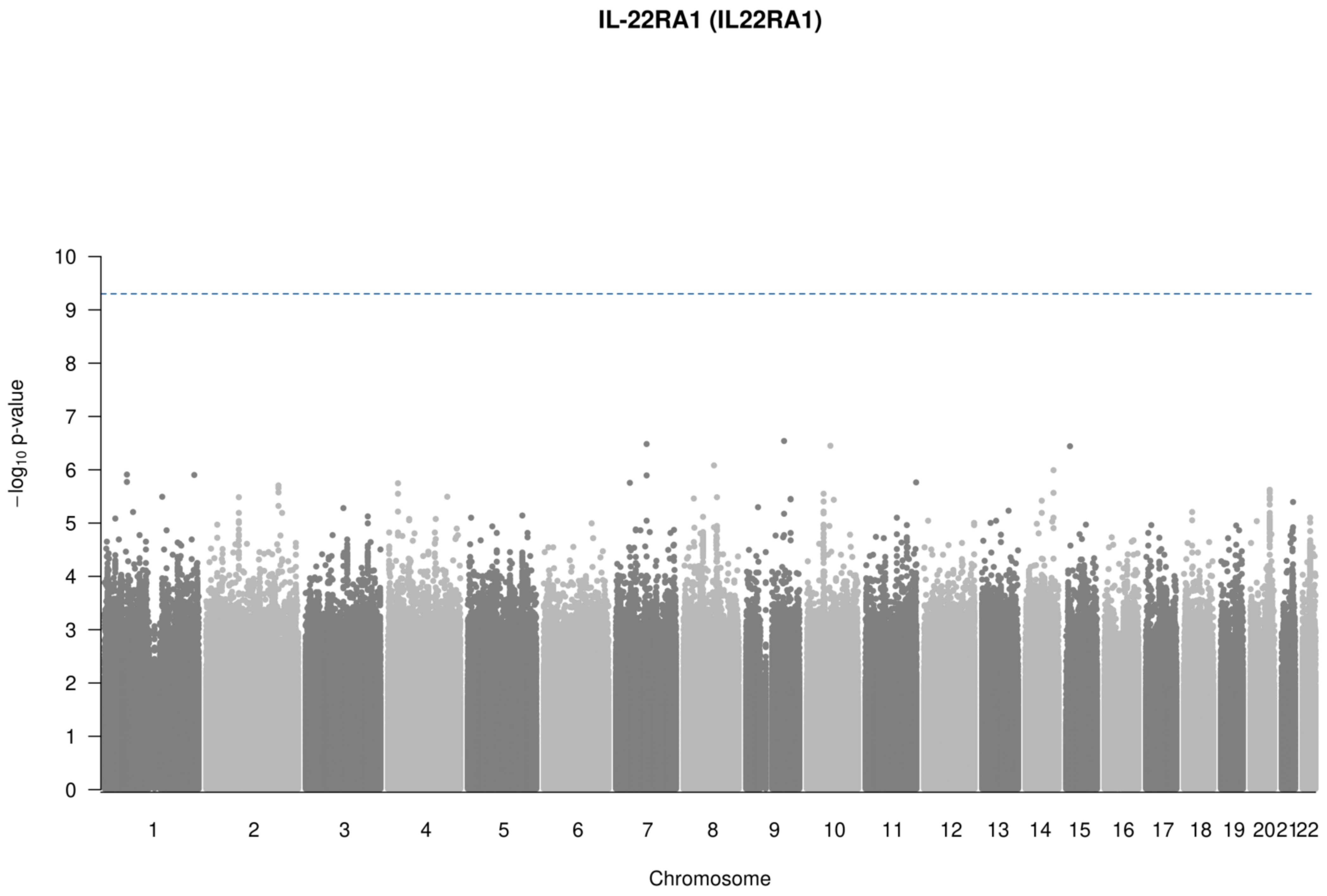
IL-22RA1 (IL22RA1)
−log10 p-value
10
9
8
7
6
5
4
3
2
1
0
1
2
3
4
5
6
7
8
9
10
11
12
13
14
15
16
17
18
19
20
21
22
Chromosome

IL-24 (IL24)

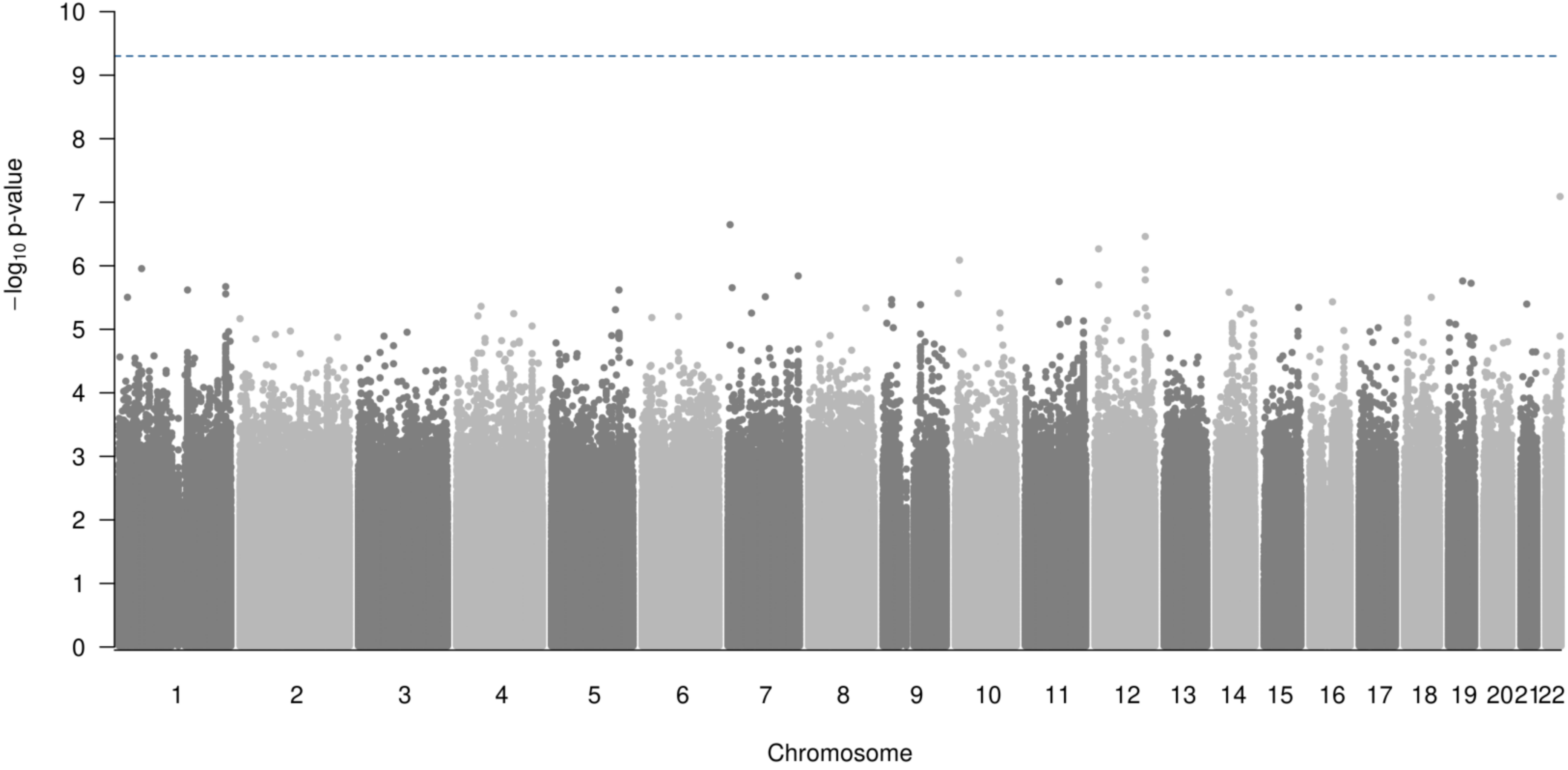

−log10 p-value
0
1
2
3
4
5
6
7
8
9
10
Chromosome
1
2
3
4
5
6
7
8
9
10
11
12
13
14
15
16
17
18
19
20
21
22

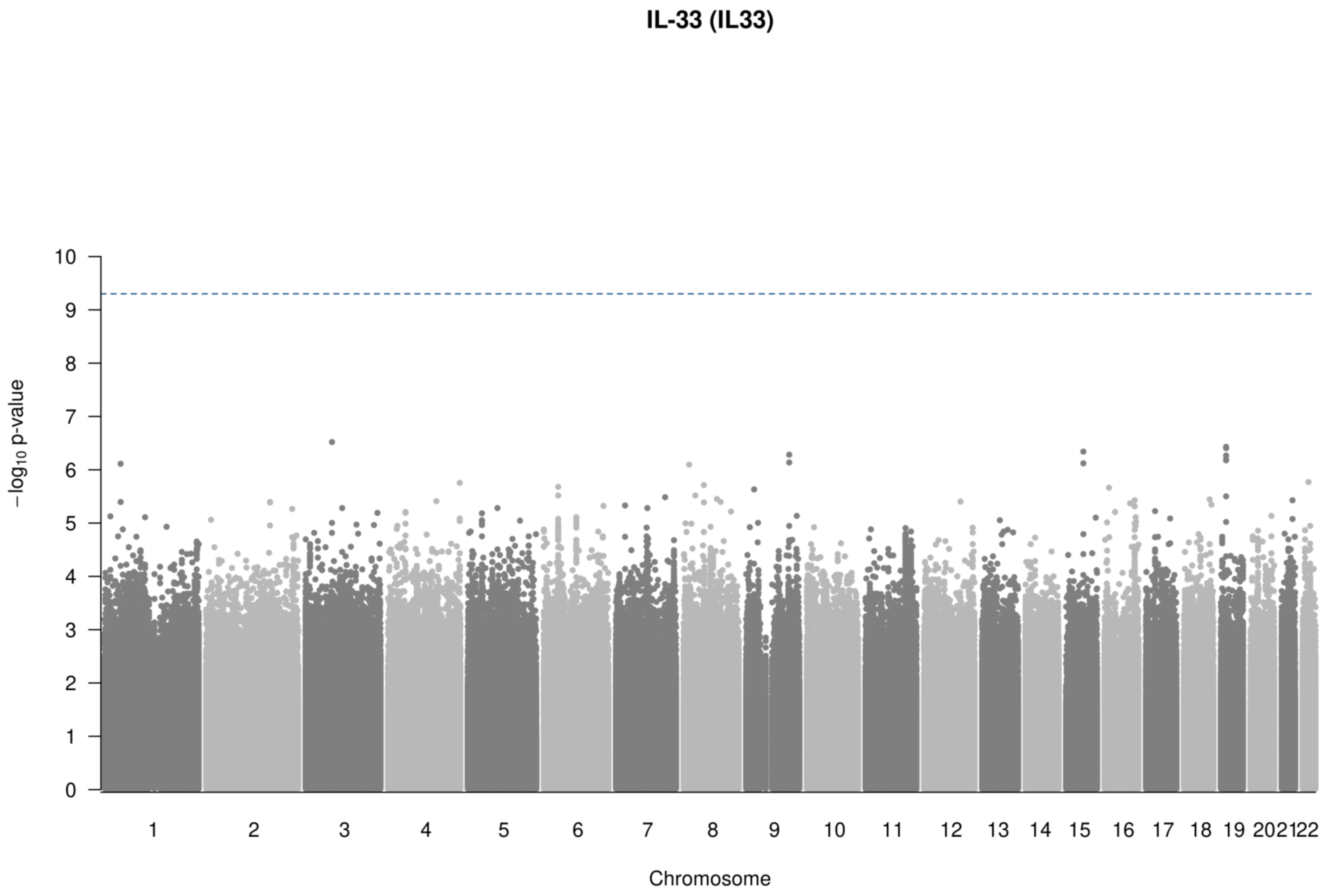
IL-33 (IL33)
−log10 p-value
0
1
2
3
4
5
6
7
8
9
10
1
2
3
4
5
6
7
8
9
10
11
12
13
14
15
16
17
18
19
202122
Chromosome

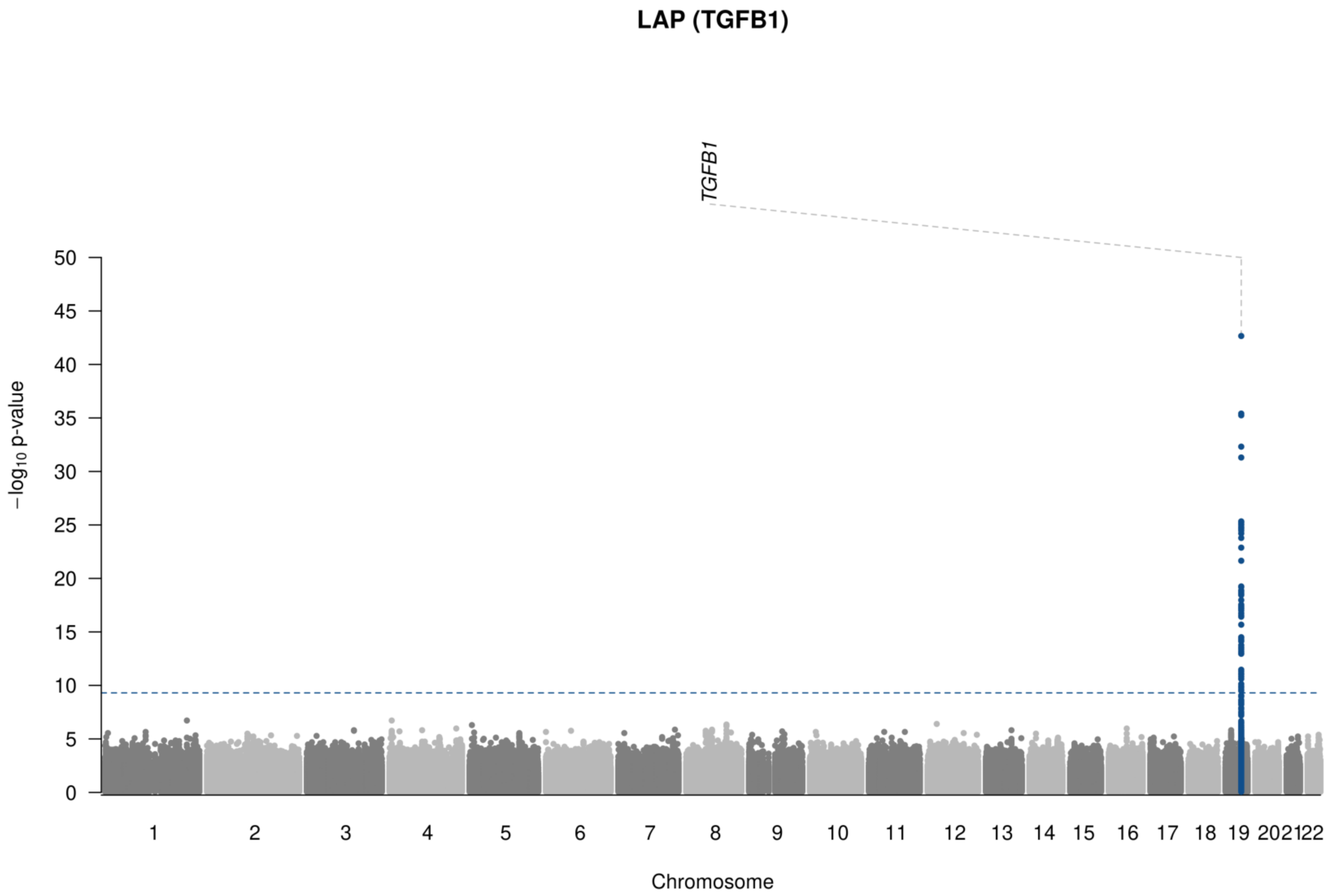
LAP (TGFB1)
TGFB1
−log10 p-value
0
5
10
15
20
25
30
35
40
45
50
1
2
3
4
5
6
7
8
9
10
11
12
13
14
15
16
17
18
19
20
21
22
Chromosome

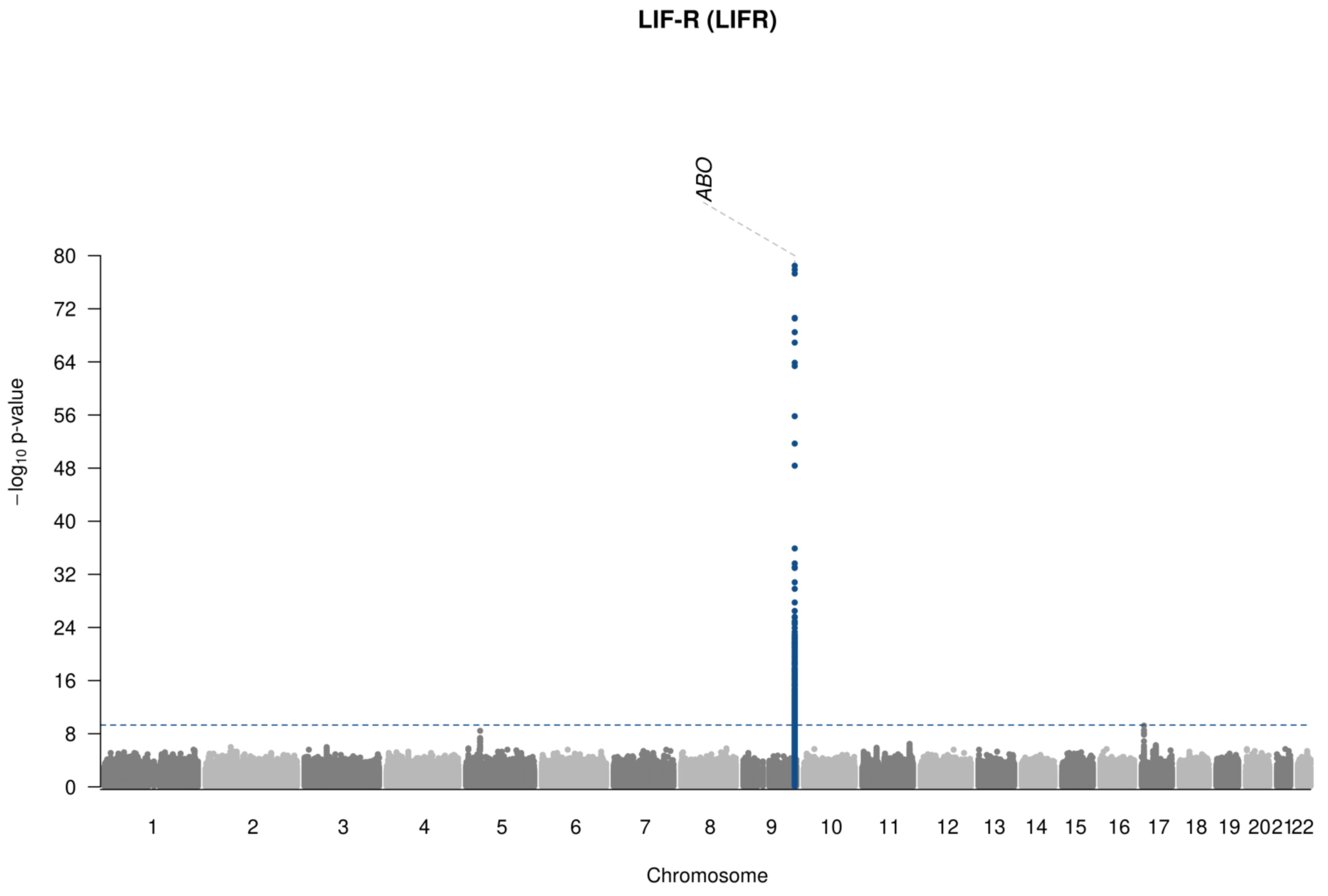
LIF-R (LIFR)
ABO
−log10 p-value
0
8
16
24
32
40
48
56
64
72
80
1
2
3
4
5
6
7
8
9
10
11
12
13
14
15
16
17
18
19
20
21
22
Chromosome

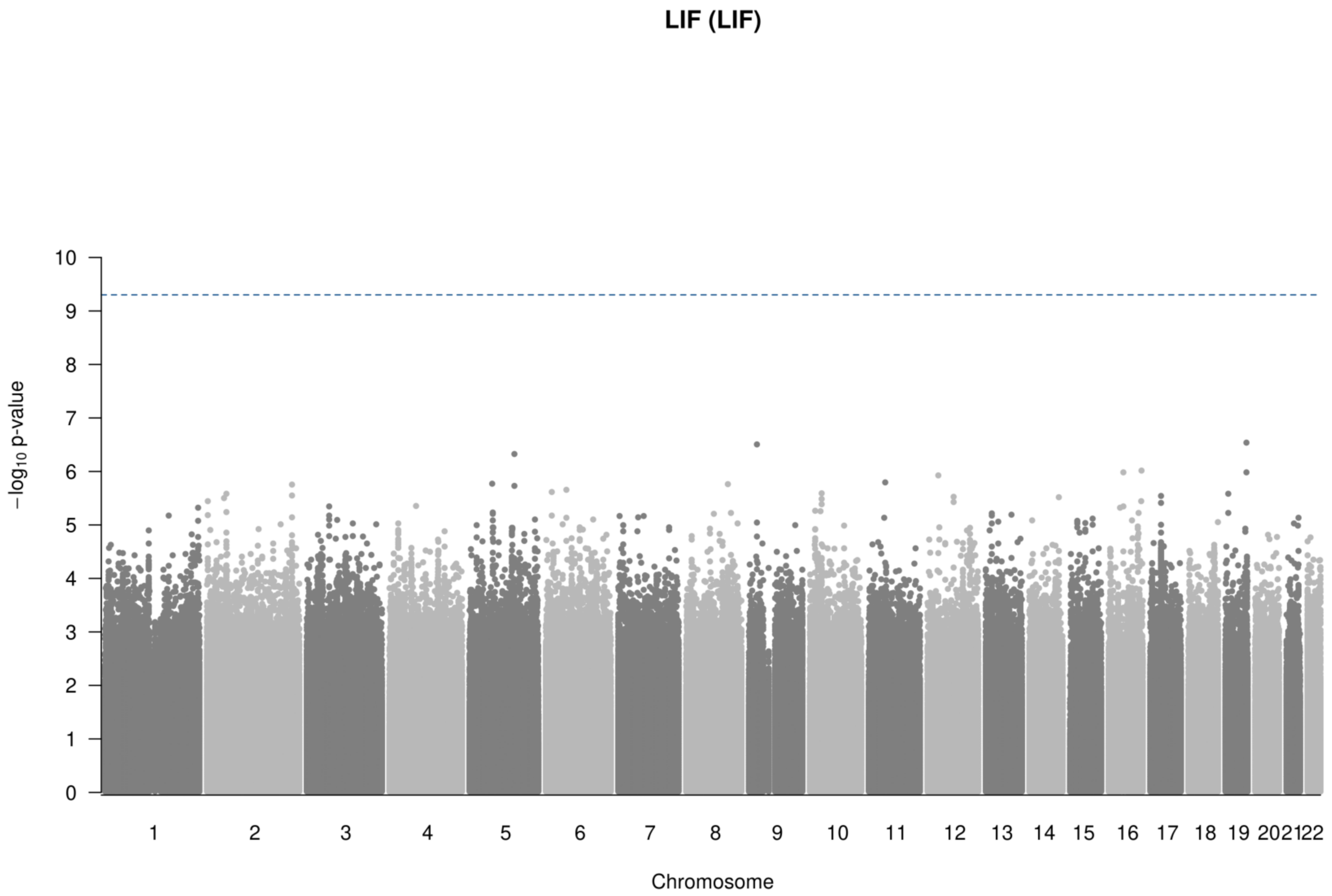
LIF (LIF)
−log10 p-value
0
1
2
3
4
5
6
7
8
9
10
1 2 3 4 5 6 7 8 9 10 11 12 13 14 15 16 17 18 19 20 21 22
Chromosome

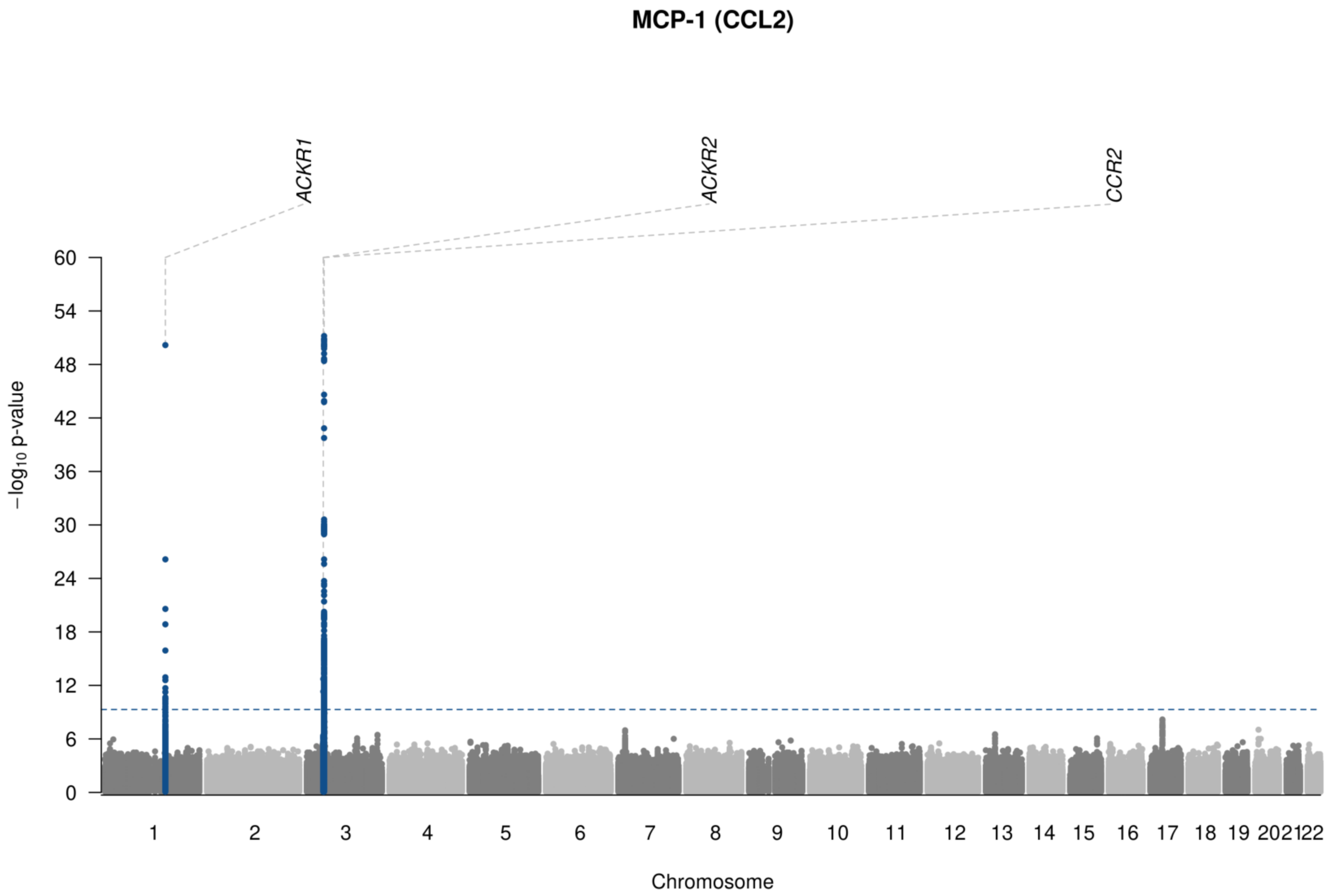
MCP-1 (CCL2)
ACKR1
ACKR2
CCR2
60
54
48
42
36
30
24
18
12
6
0
−log10 p-value
1
2
3
4
5
6
7
8
9
10
11
12
13
14
15
16
17
18
19
20
21
22
Chromosome

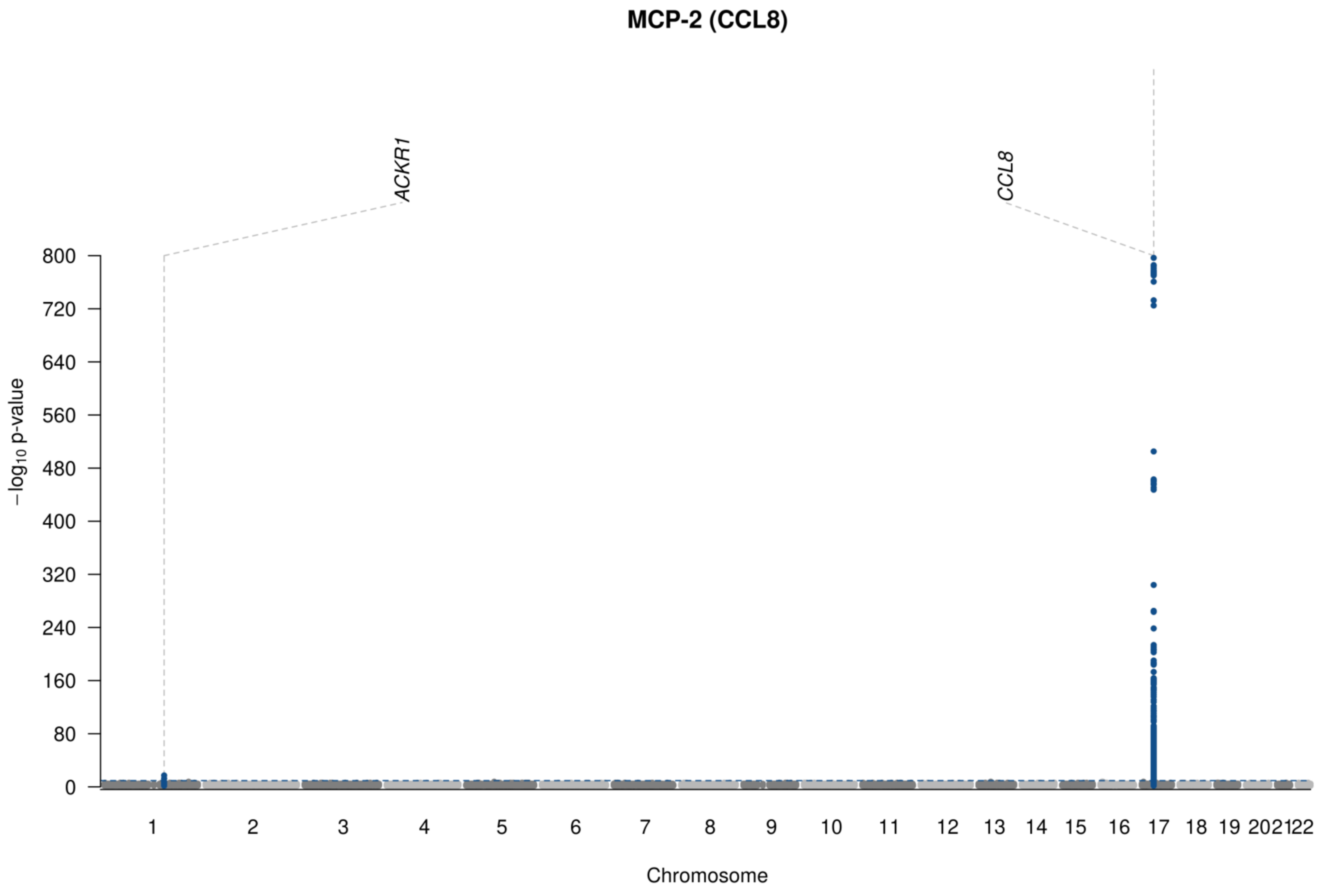

MCP-2 (CCL8)
ACKR1
CCL8
800
720
640
560
480
400
320
240
160
80
0
−log10 p-value
1
2
3
4
5
6
7
8
9
10
11
12
13
14
15
16
17
18
19
20
21
22
Chromosome

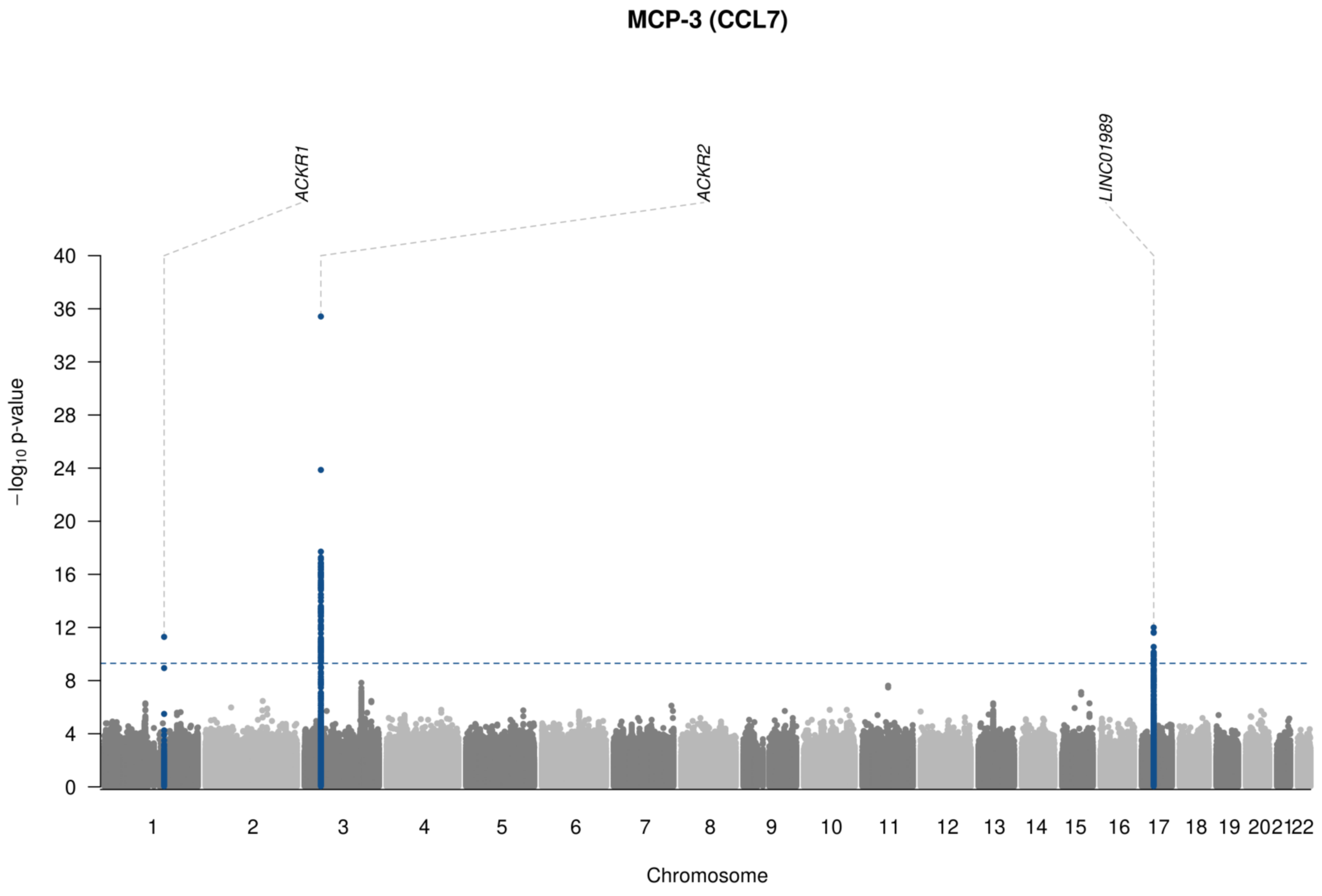
MCP-3 (CCL7)
ACKR1
ACKR2
LINC01989
−log10 p-value
40
36
32
28
24
20
16
12
8
4
0
1
2
3
4
5
6
7
8
9
10
11
12
13
14
15
16
17
18
19
20
21
22
Chromosome

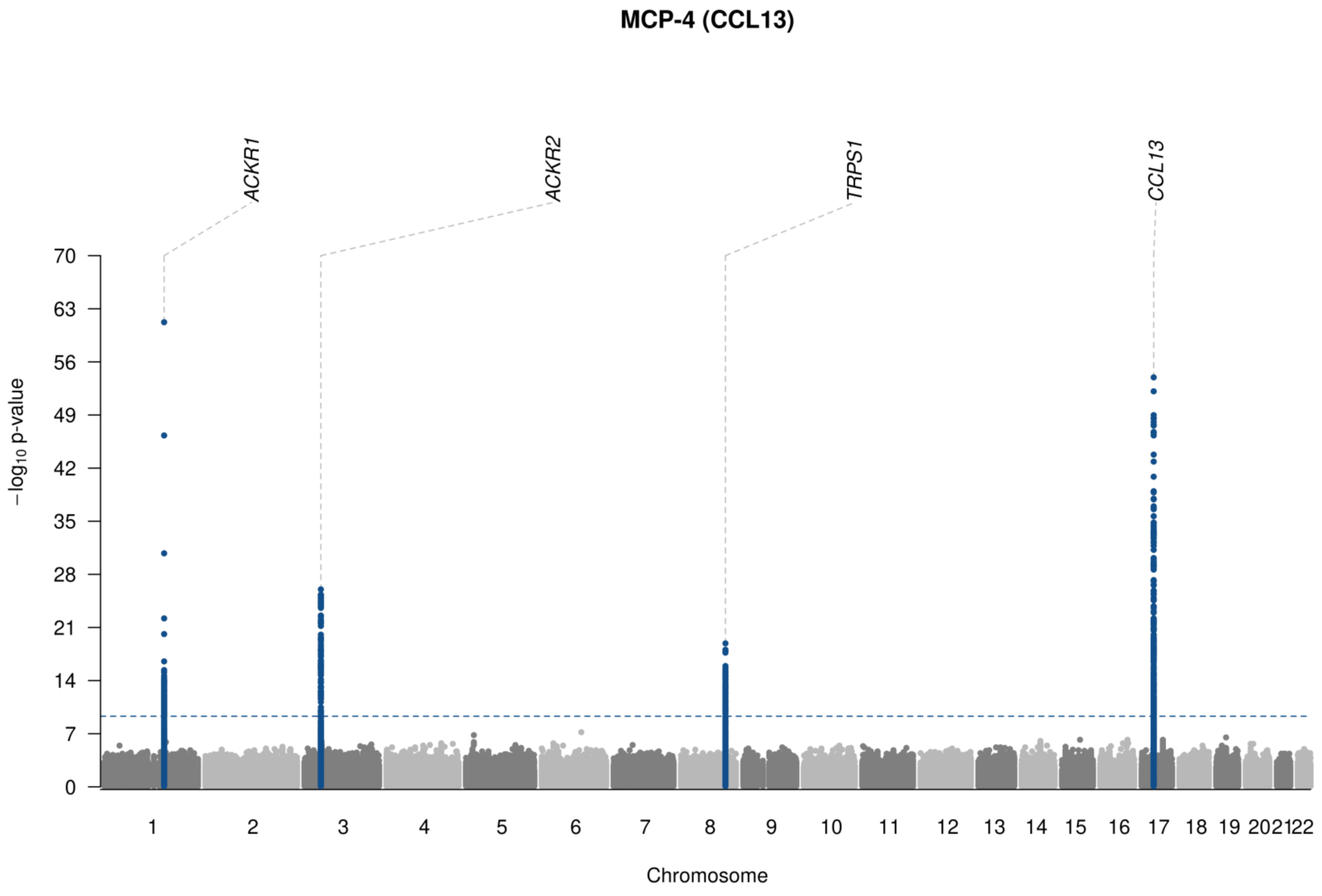

MCP-4 (CCL13)
ACKR1
ACKR2
TRPS1
CCL13
−log10 p-value
70
63
56
49
42
35
28
21
14
7
0
1
2
3
4
5
6
7
8
9
10
11
12
13
14
15
16
17
18
19
20
21
22
Chromosome

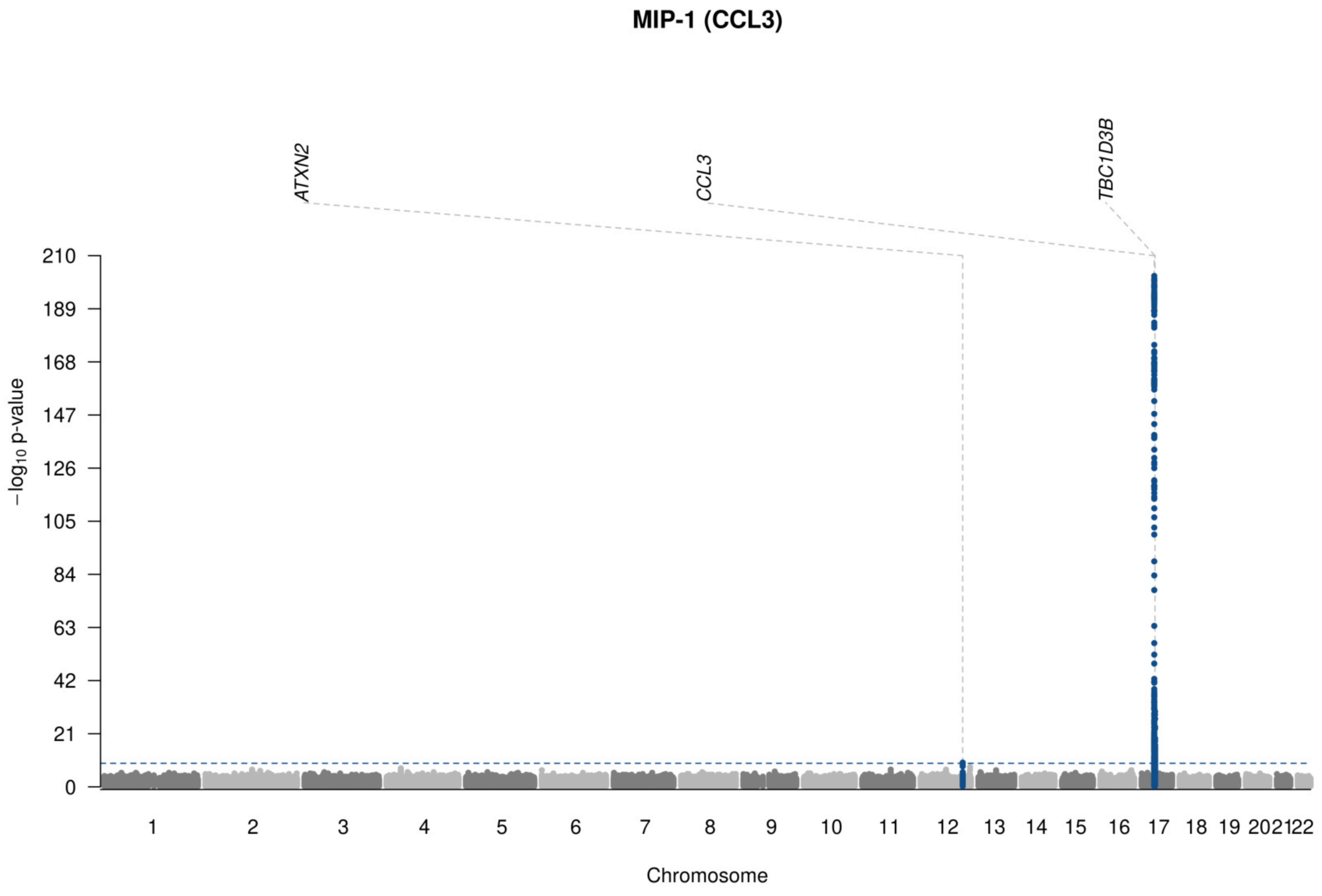

MIP-1 (CCL3)
ATXN2
CCL3
TBC1D3B
210
189
168
147
126
105
84
63
42
21
0
−log10 p-value
1
2
3
4
5
6
7
8
9
10
11
12
13
14
15
16
17
18
19
20
21
22
Chromosome

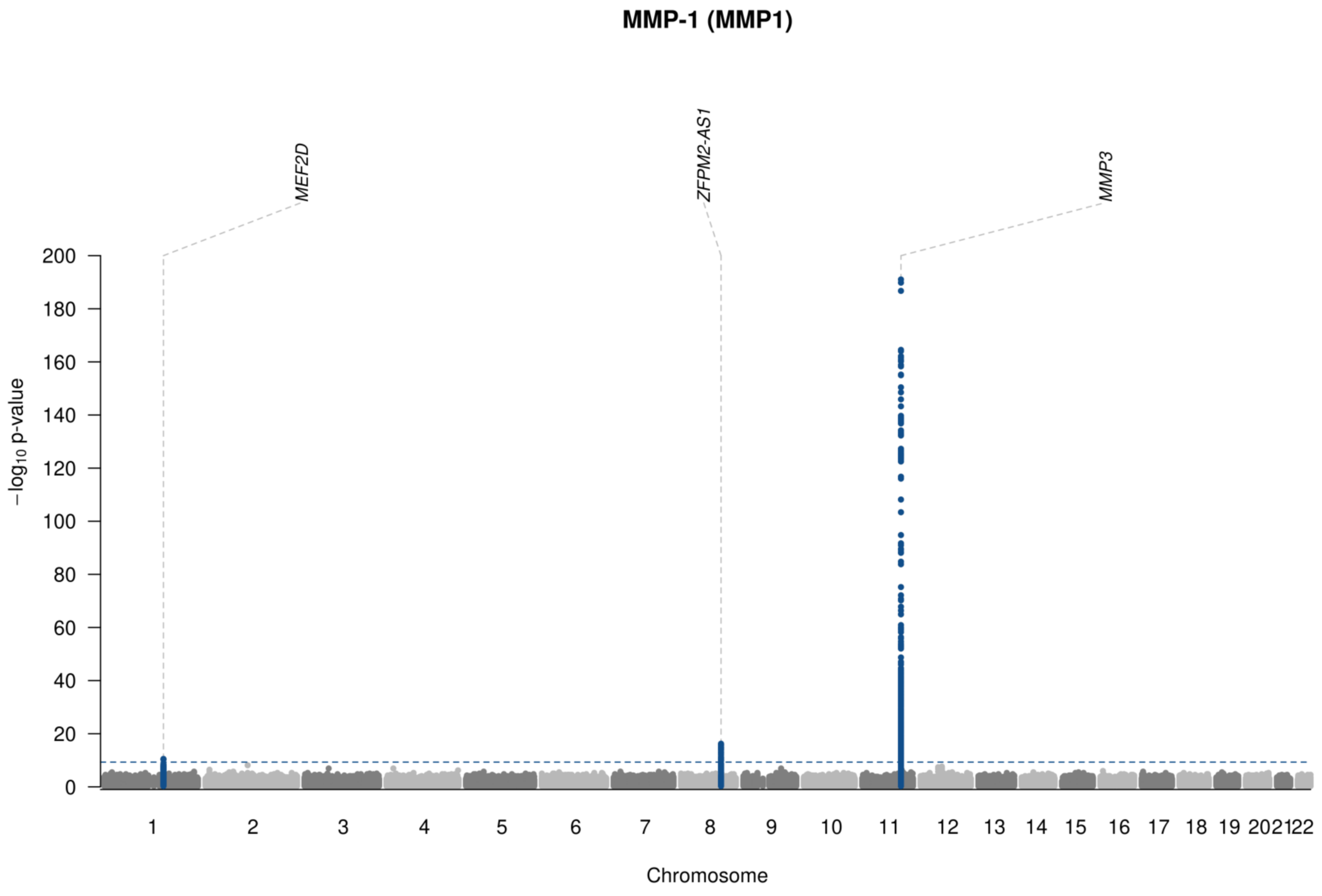

MMP-1 (MMP1)
MEF2D
ZFPM2-AS1
MMP3
−log10 p-value
0
20
40
60
80
100
120
140
160
180
200
1
2
3
4
5
6
7
8
9
10
11
12
13
14
15
16
17
18
19
20
21
22
Chromosome

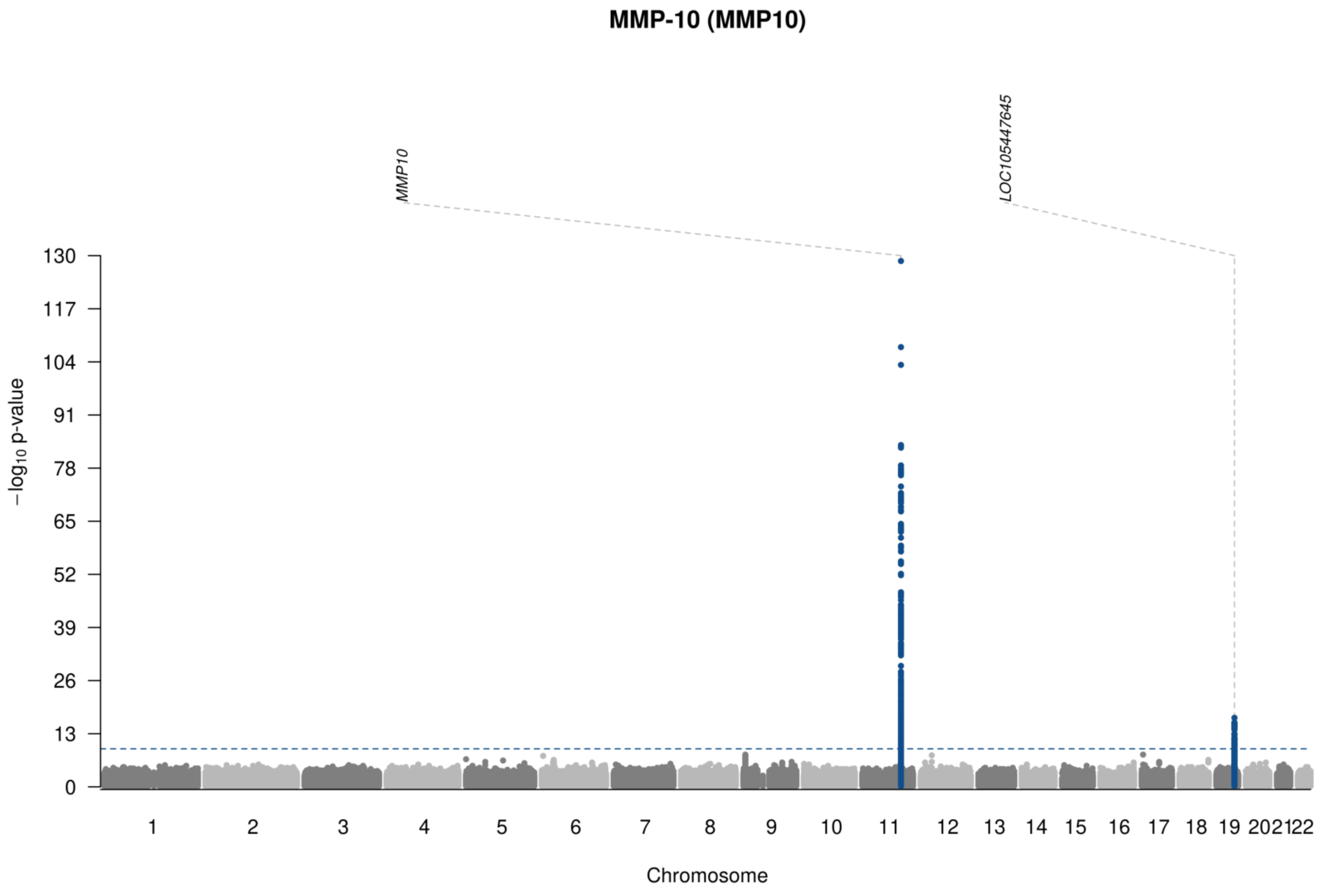

MMP-10 (MMP10)
MMP10
LOC105447645
−log10 p-value
130
117
104
91
78
65
52
39
26
13
0
1
2
3
4
5
6
7
8
9
10
11
12
13
14
15
16
17
18
19
20
21
22
Chromosome

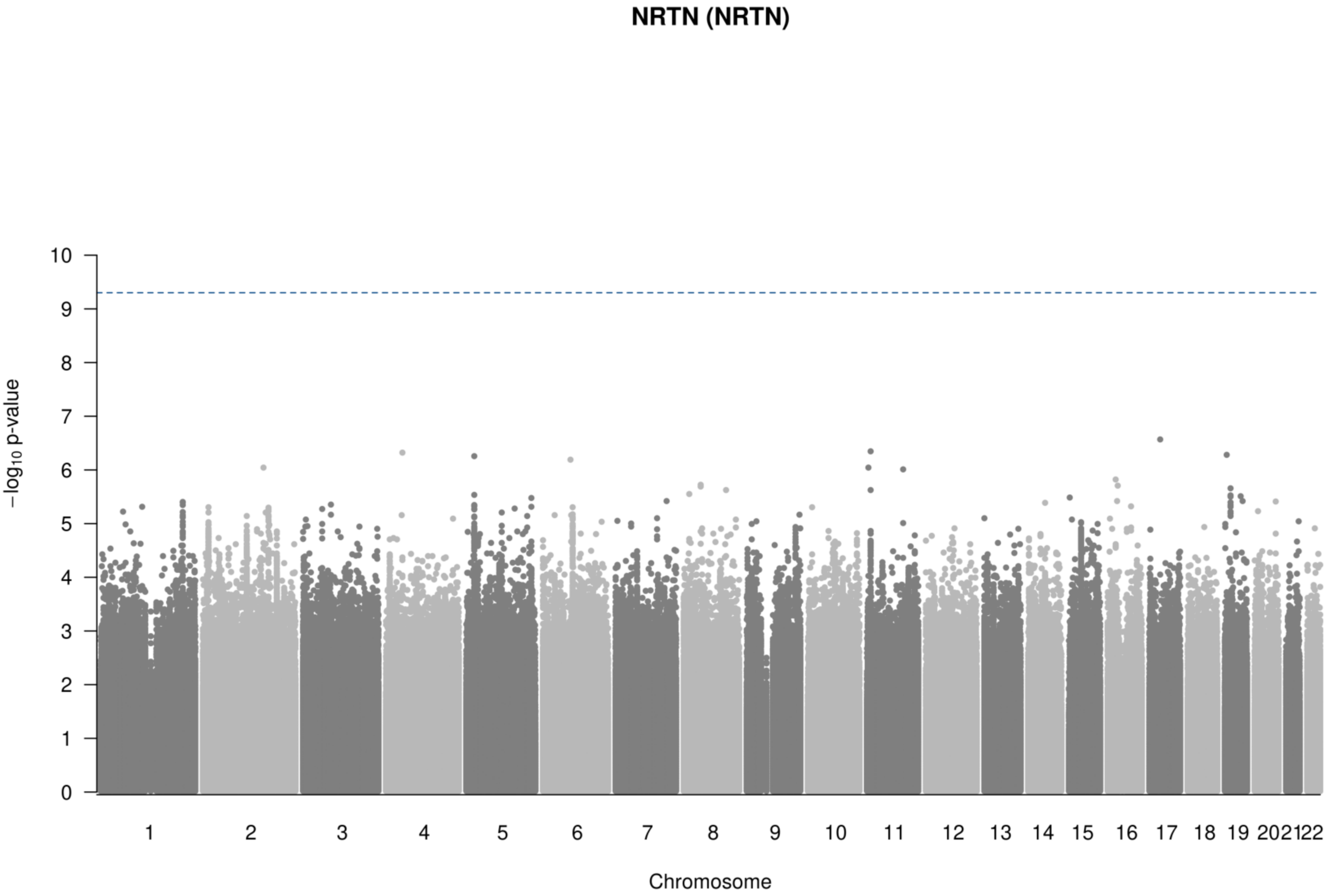

NRTN (NRTN)
−log10 p-value
0
1
2
3
4
5
6
7
8
9
10
1
2
3
4
5
6
7
8
9
10
11
12
13
14
15
16
17
18
19
20
21
22
Chromosome

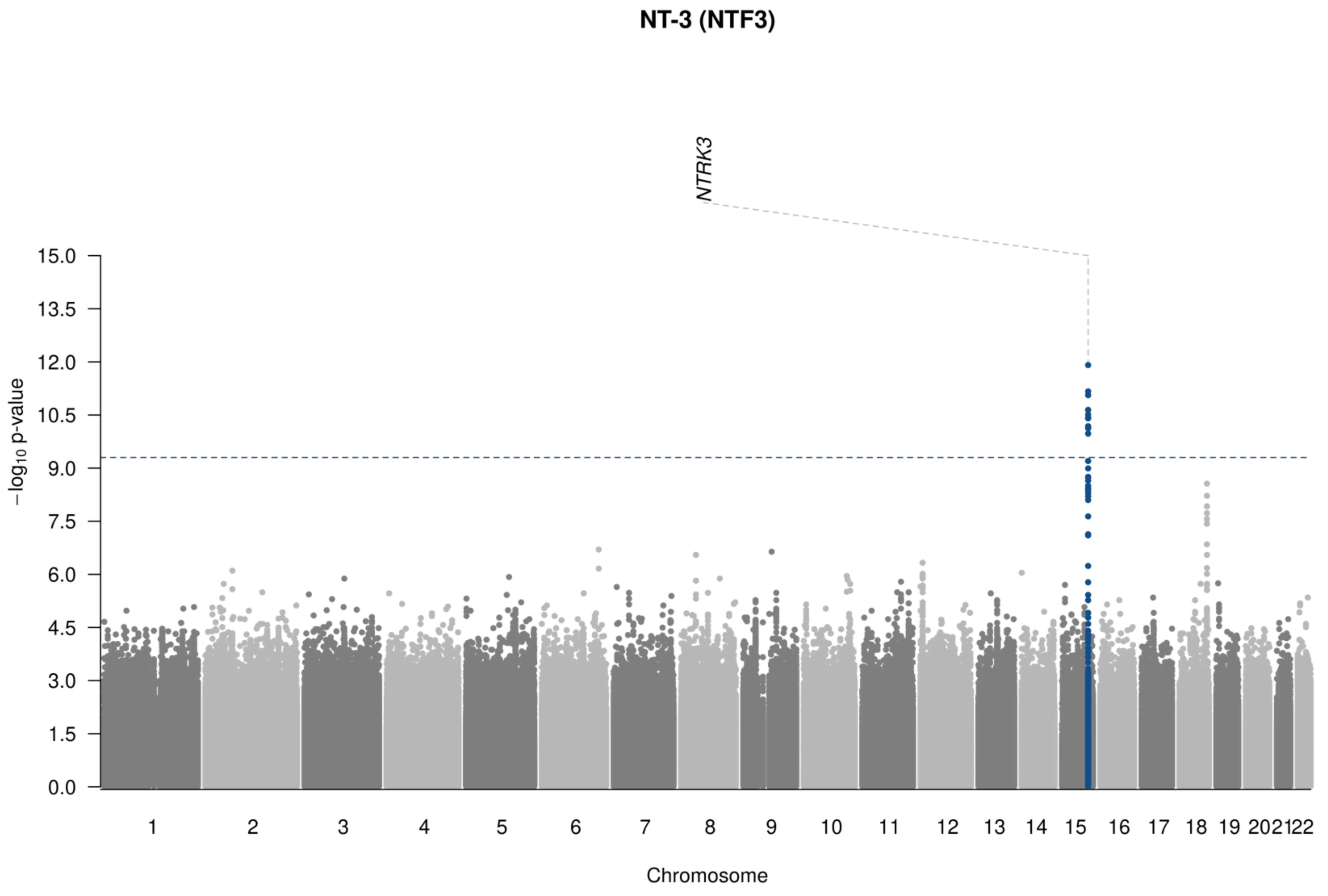
NT-3 (NTF3)
NTRK3
−log10 p-value
15.0
13.5
12.0
10.5
9.0
7.5
6.0
4.5
3.0
1.5
0.0
1
2
3
4
5
6
7
8
9
10
11
12
13
14
15
16
17
18
19
20
21
22
Chromosome

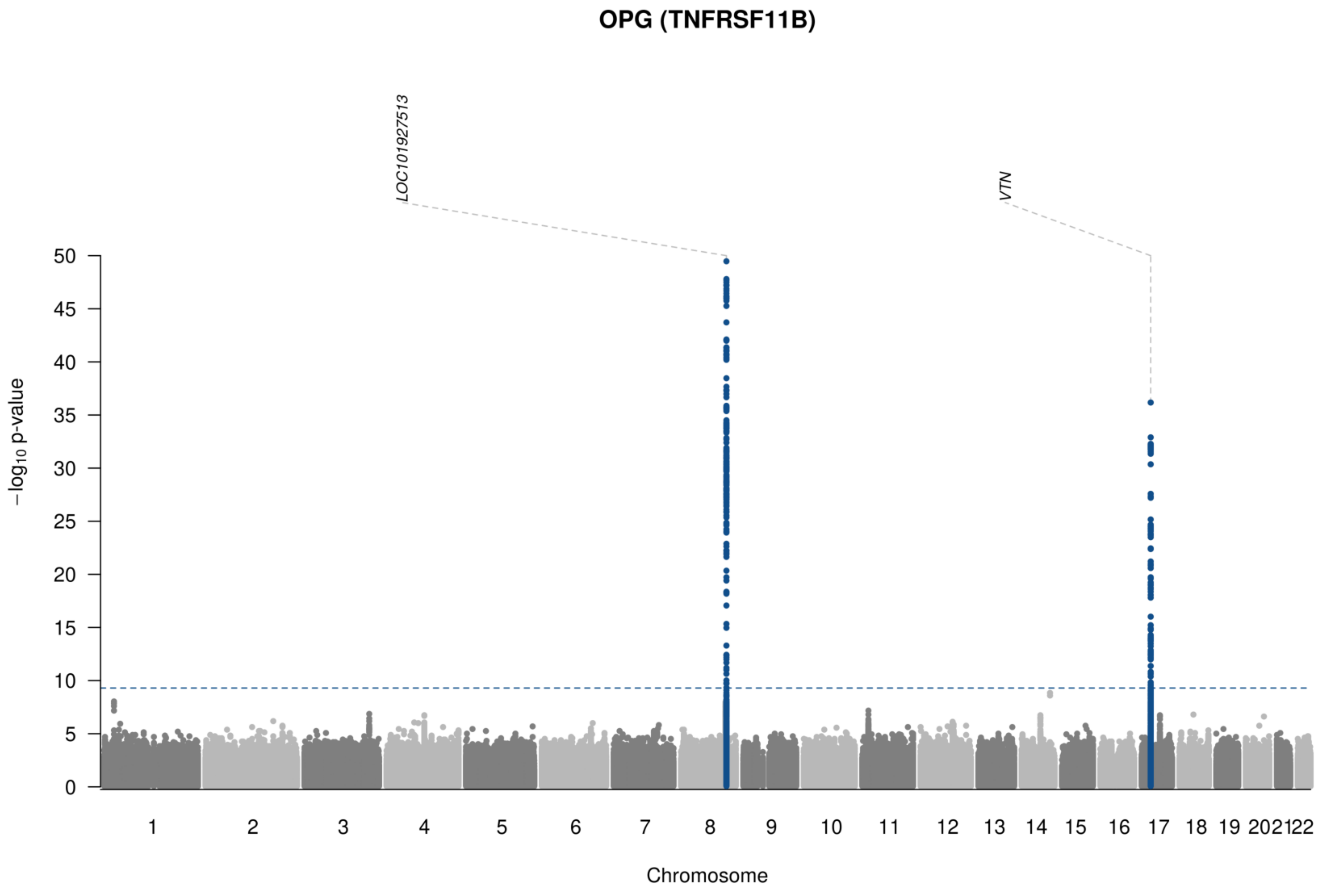

OPG (TNFRSF11B)
LOC101927513
VTN
−log10 p-value
50
45
40
35
30
25
20
15
10
5
0
1
2
3
4
5
6
7
8
9
10
11
12
13
14
15
16
17
18
19
20
21
22
Chromosome

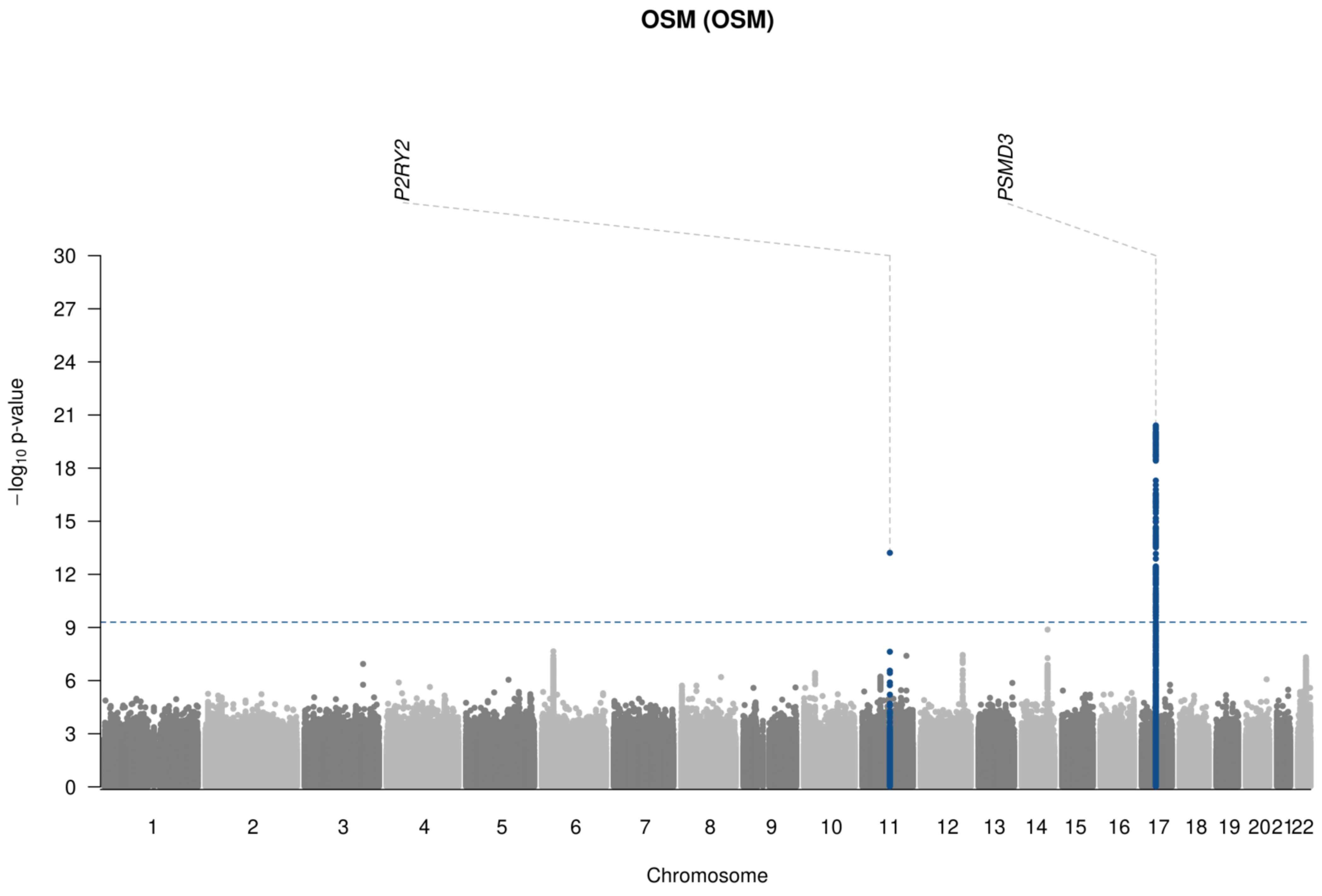

OSM (OSM)
P2RY2
PSMD3
30
27
24
21
18
15
12
9
6
3
0
−log10 p-value
1
2
3
4
5
6
7
8
9
10
11
12
13
14
15
16
17
18
19
20
21
22
Chromosome

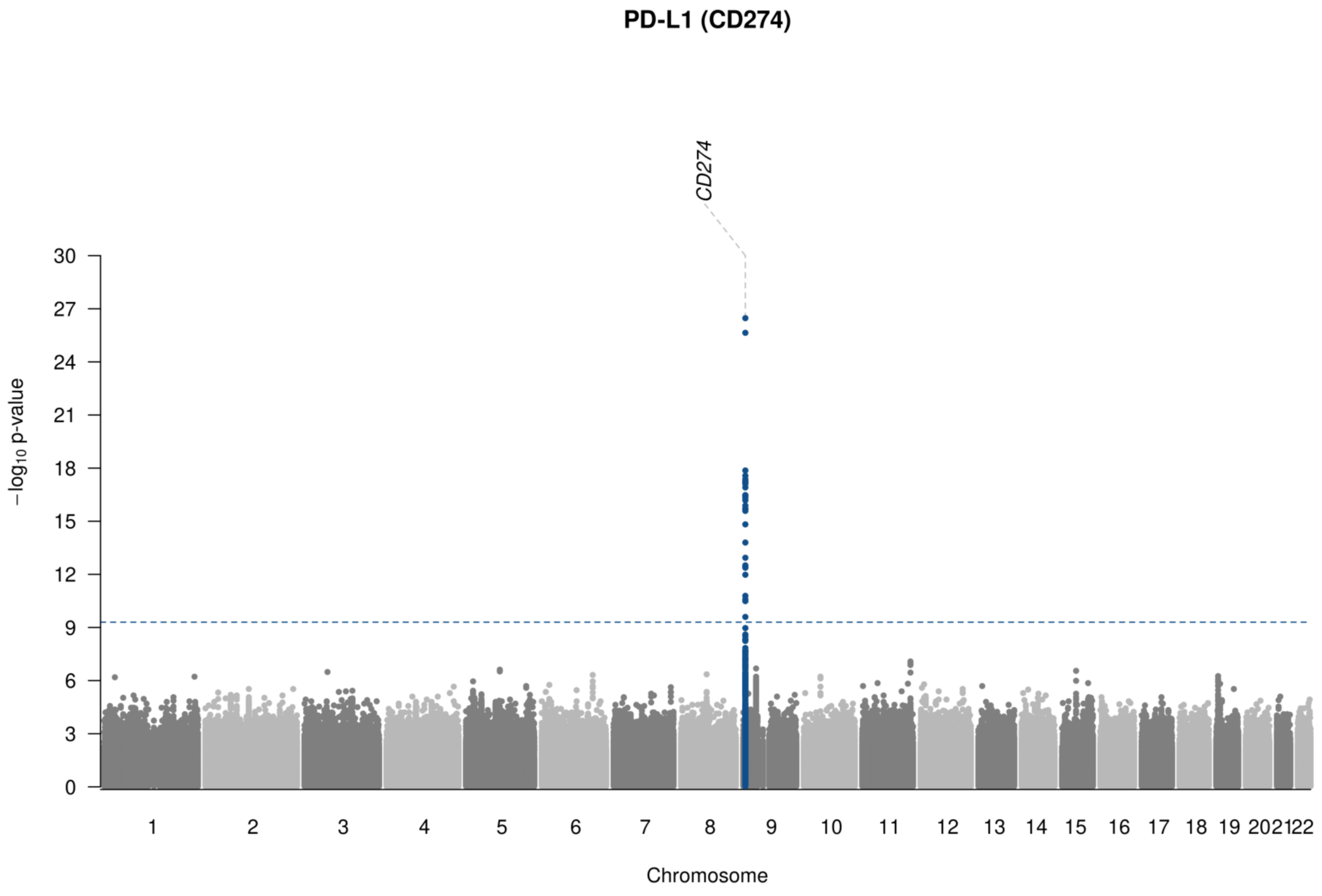
PD-L1 (CD274)
CD274
−log10 p-value
30
27
24
21
18
15
12
9
6
3
0
1
2
3
4
5
6
7
8
9
10
11
12
13
14
15
16
17
18
19
20
21
22
Chromosome

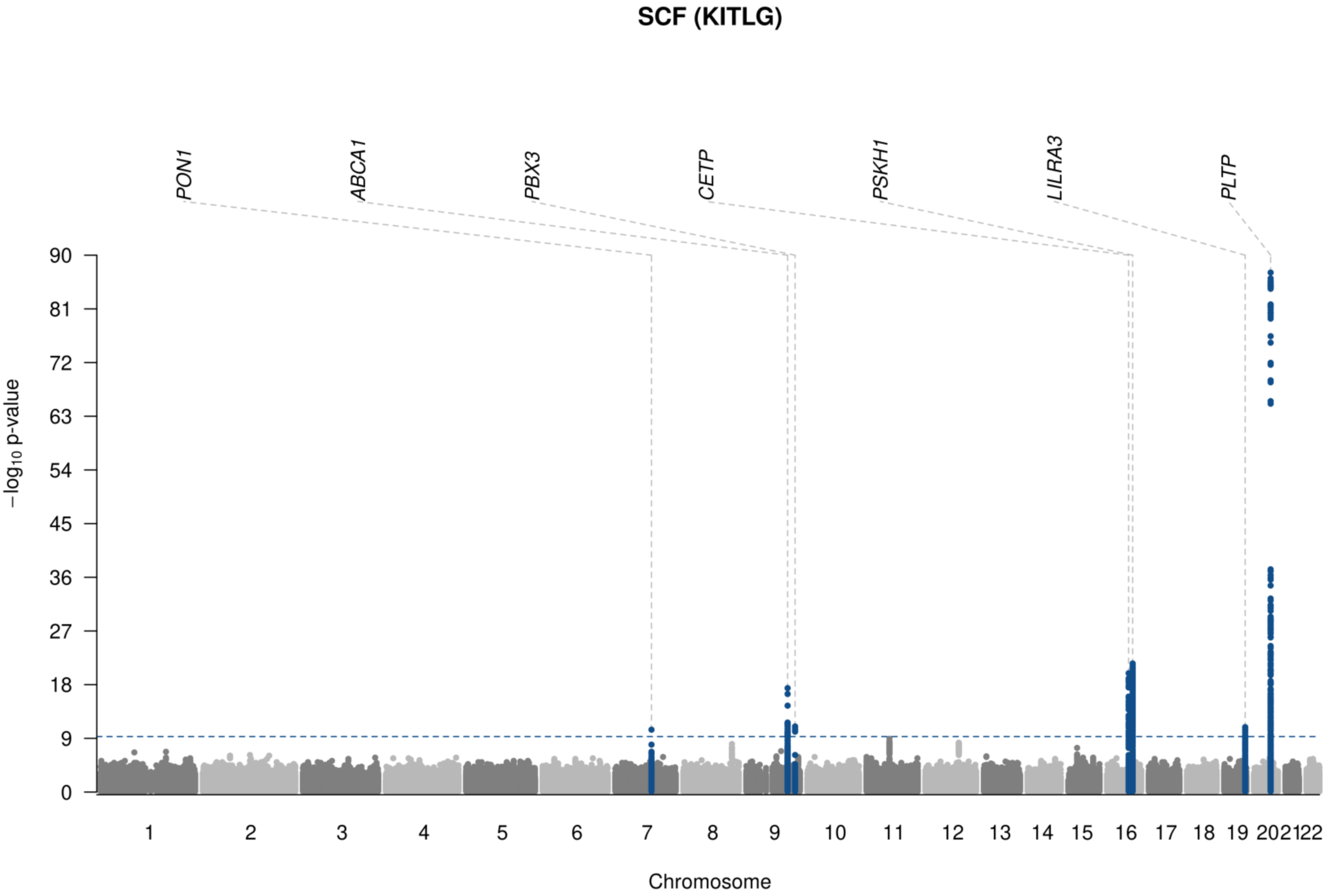
SCF (KITLG)
PON1
ABCA1
PBX3
CETP
PSKH1
LILRA3
PLTP
−log10 p-value
90
81
72
63
54
45
36
27
18
9
0
1
2
3
4
5
6
7
8
9
10
11
12
13
14
15
16
17
18
19
20
21
22
Chromosome

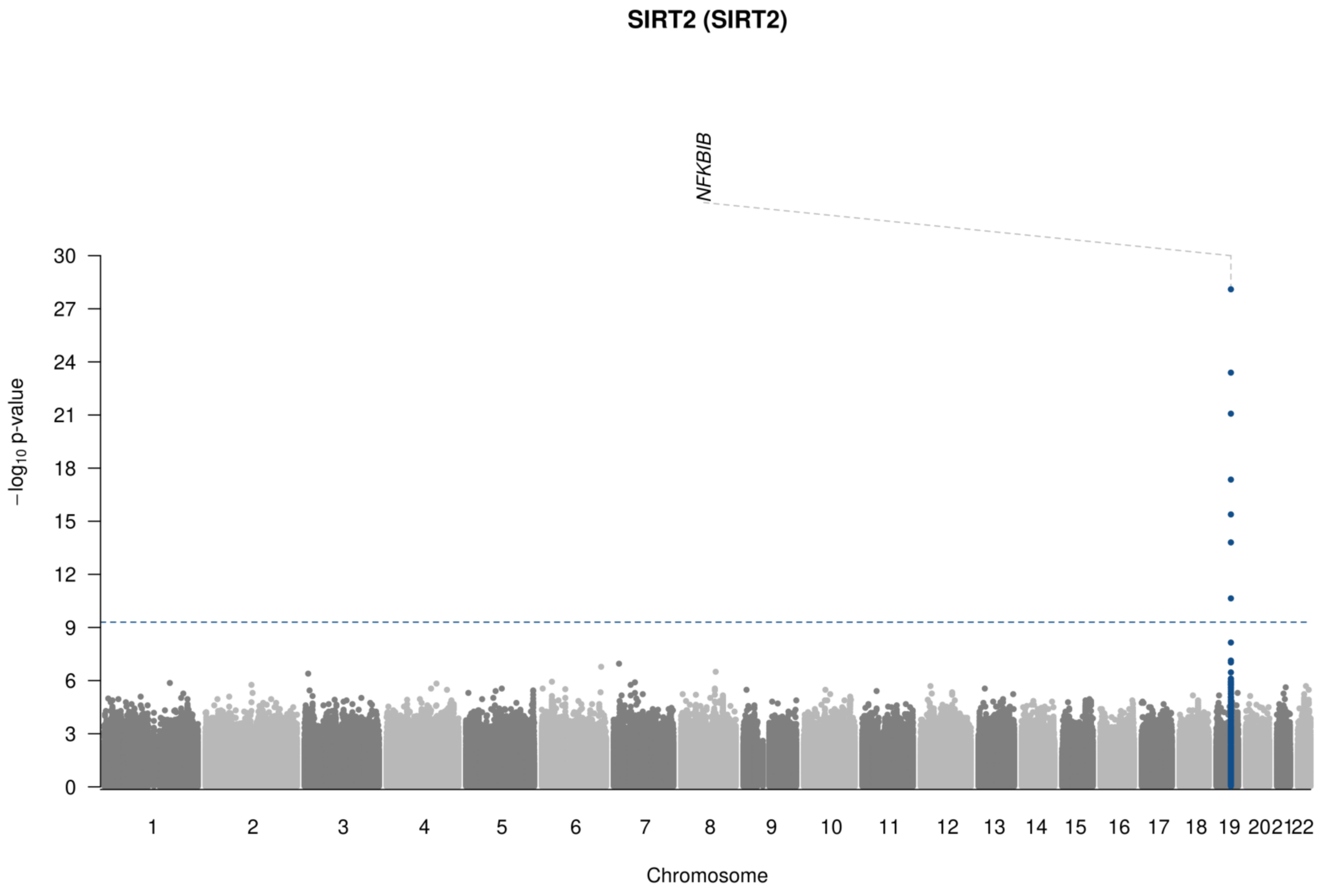

SIRT2 (SIRT2)
NFKBIB
30
27
24
21
18
15
12
9
6
3
0
−log10 p-value
1
2
3
4
5
6
7
8
9
10
11
12
13
14
15
16
17
18
19
20
21
22
Chromosome

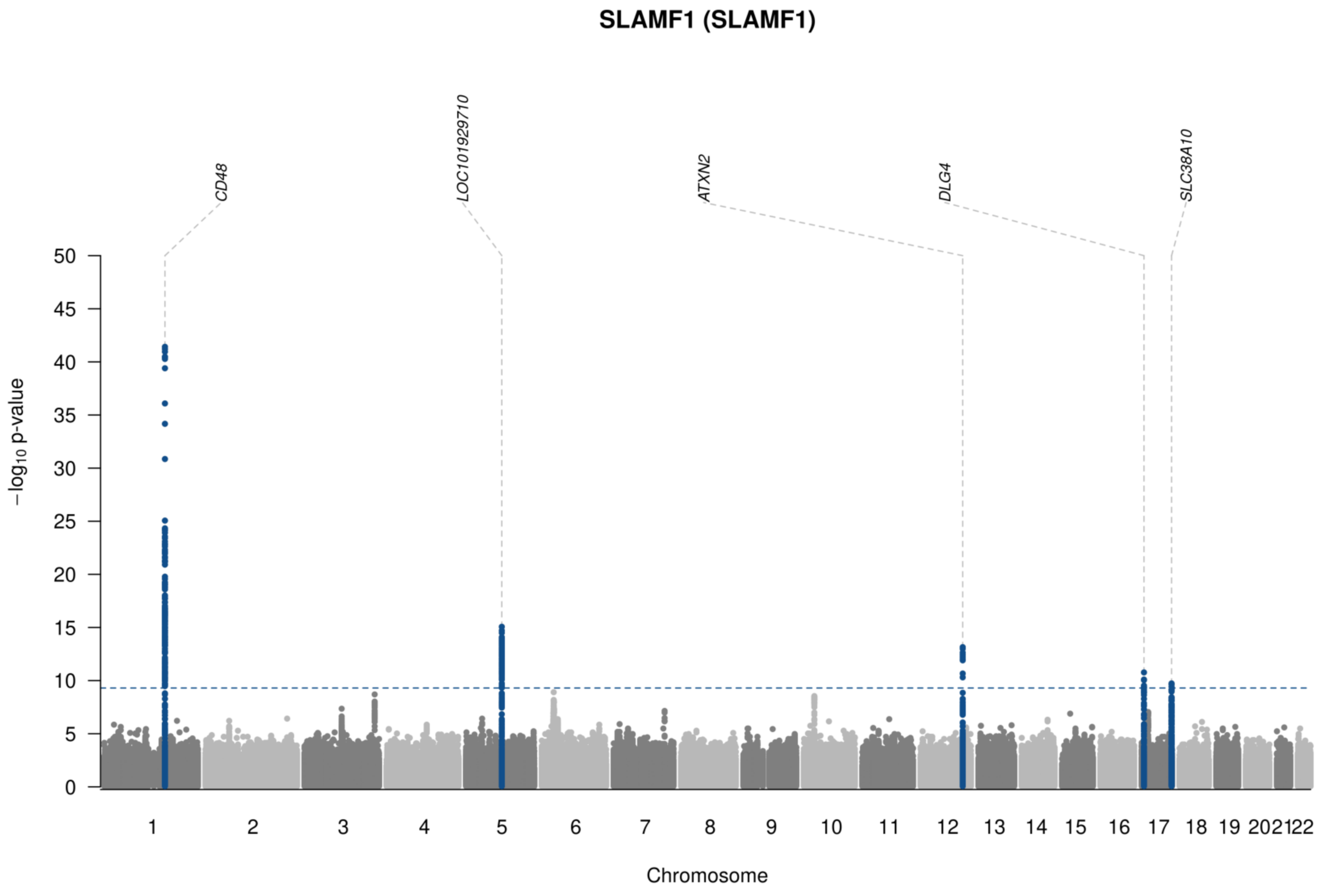

SLAMF1 (SLAMF1)
CD48
LOC101929710
ATXN2
DLG4
SLC38A10
−log10 p-value
50
45
40
35
30
25
20
15
10
5
0
1
2
3
4
5
6
7
8
9
10
11
12
13
14
15
16
17
18
19
20
21
22
Chromosome

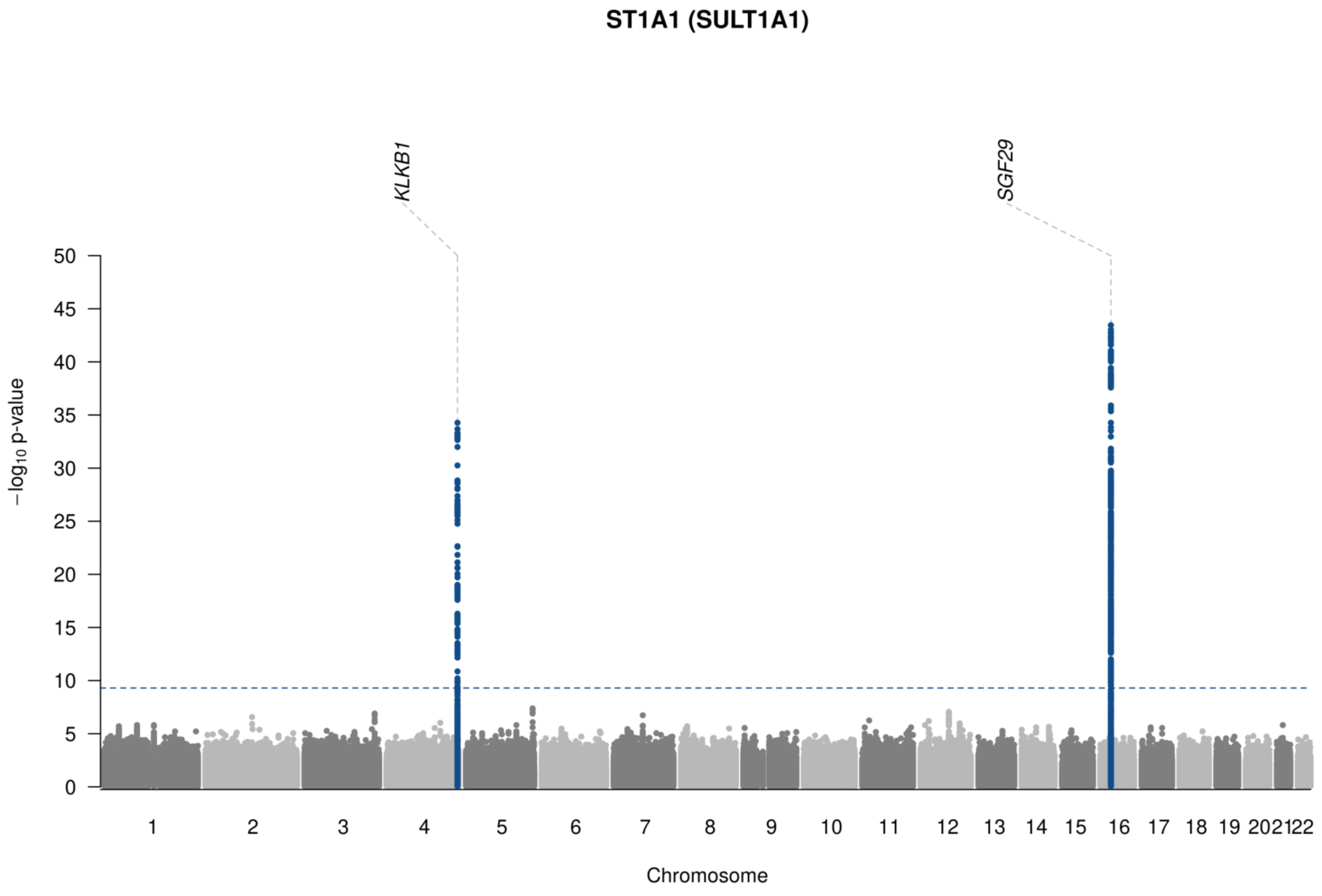

ST1A1 (SULT1A1)
KLKB1
SGF29
−log10 p-value
50
45
40
35
30
25
20
15
10
5
0
1
2
3
4
5
6
7
8
9
10
11
12
13
14
15
16
17
18
19
20
21
22
Chromosome

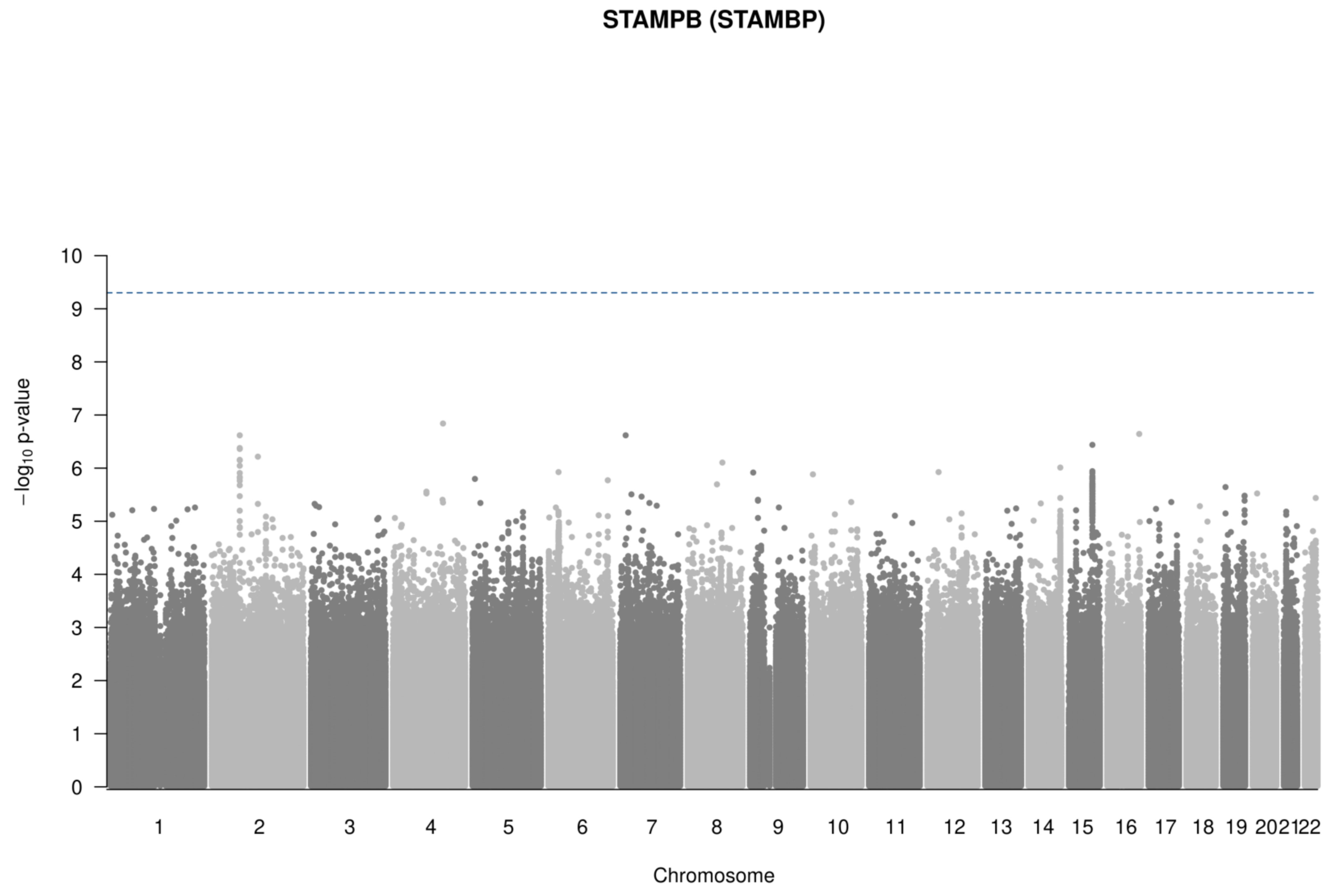
STAMPB (STAMBP)
$-\log_{10}$ p-value
0
1
2
3
4
5
6
7
8
9
10
1
2
3
4
5
6
7
8
9
10
11
12
13
14
15
16
17
18
19
20
21
22
Chromosome

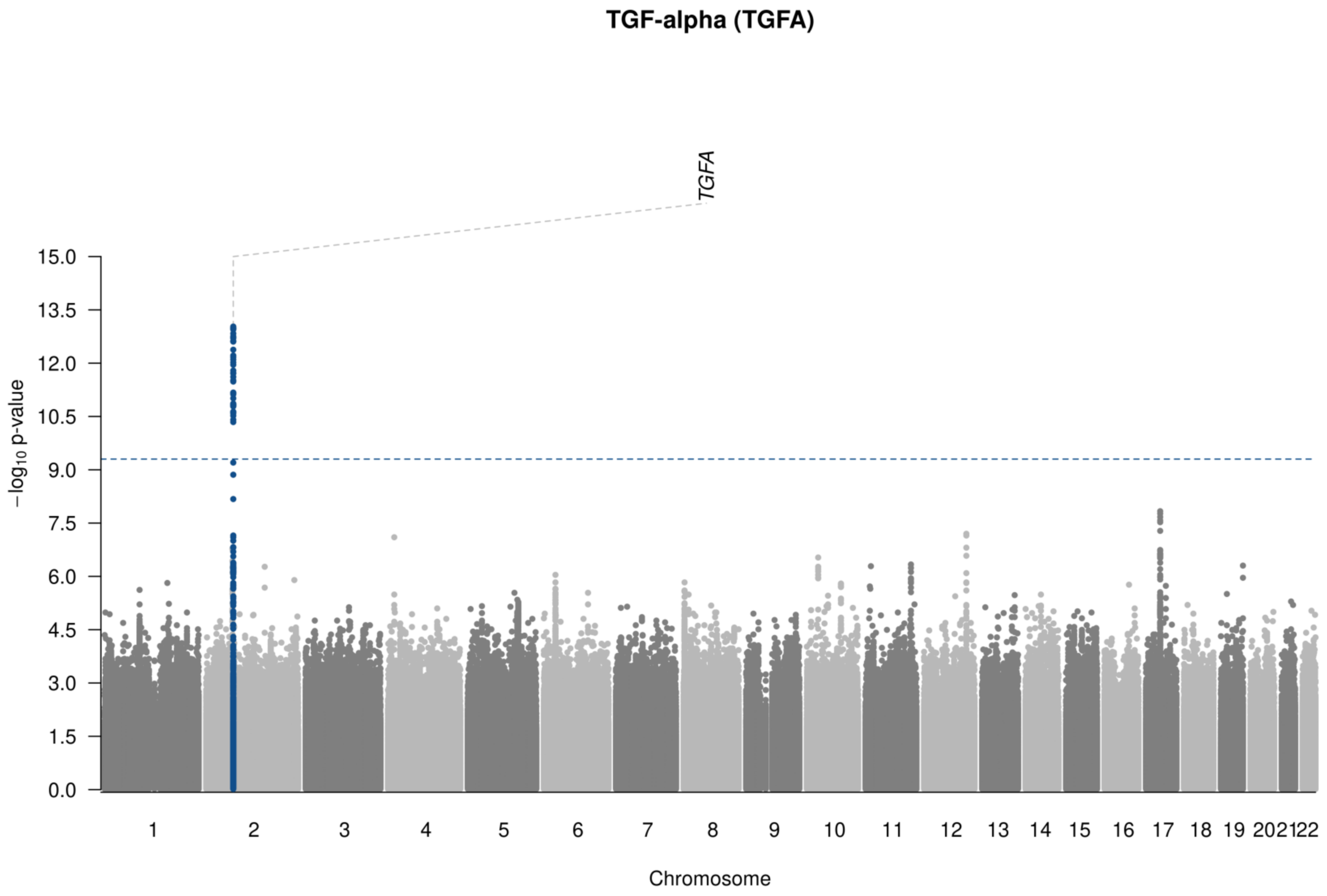

TGF-alpha (TGFA)
TGFA
−log10 p-value
15.0
13.5
12.0
10.5
9.0
7.5
6.0
4.5
3.0
1.5
0.0
1 2 3 4 5 6 7 8 9 10 11 12 13 14 15 16 17 18 19 20 21 22
Chromosome

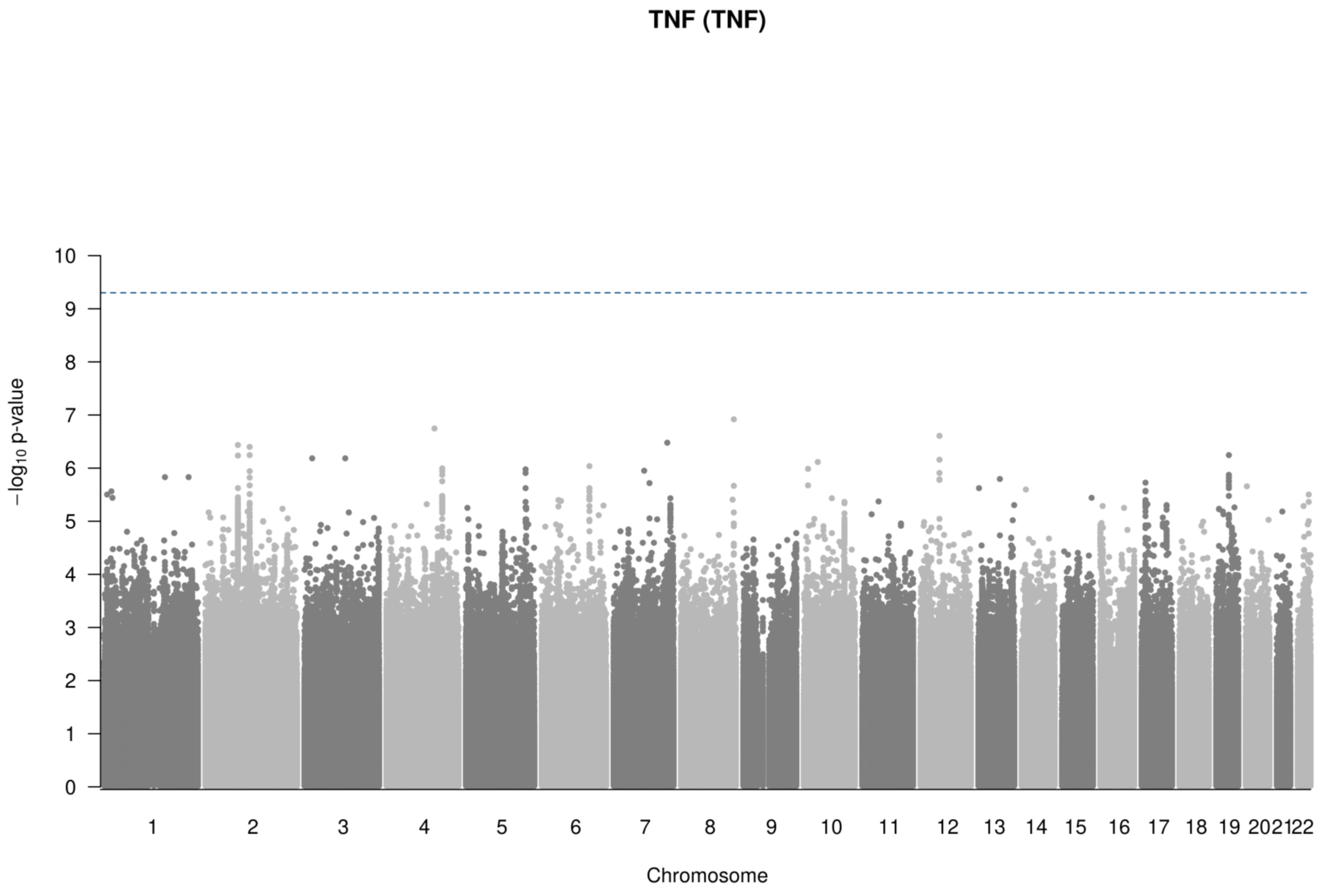

TNF (TNF)
−log10 p-value
10
9
8
7
6
5
4
3
2
1
0
1
2
3
4
5
6
7
8
9
10
11
12
13
14
15
16
17
18
19
202122
Chromosome

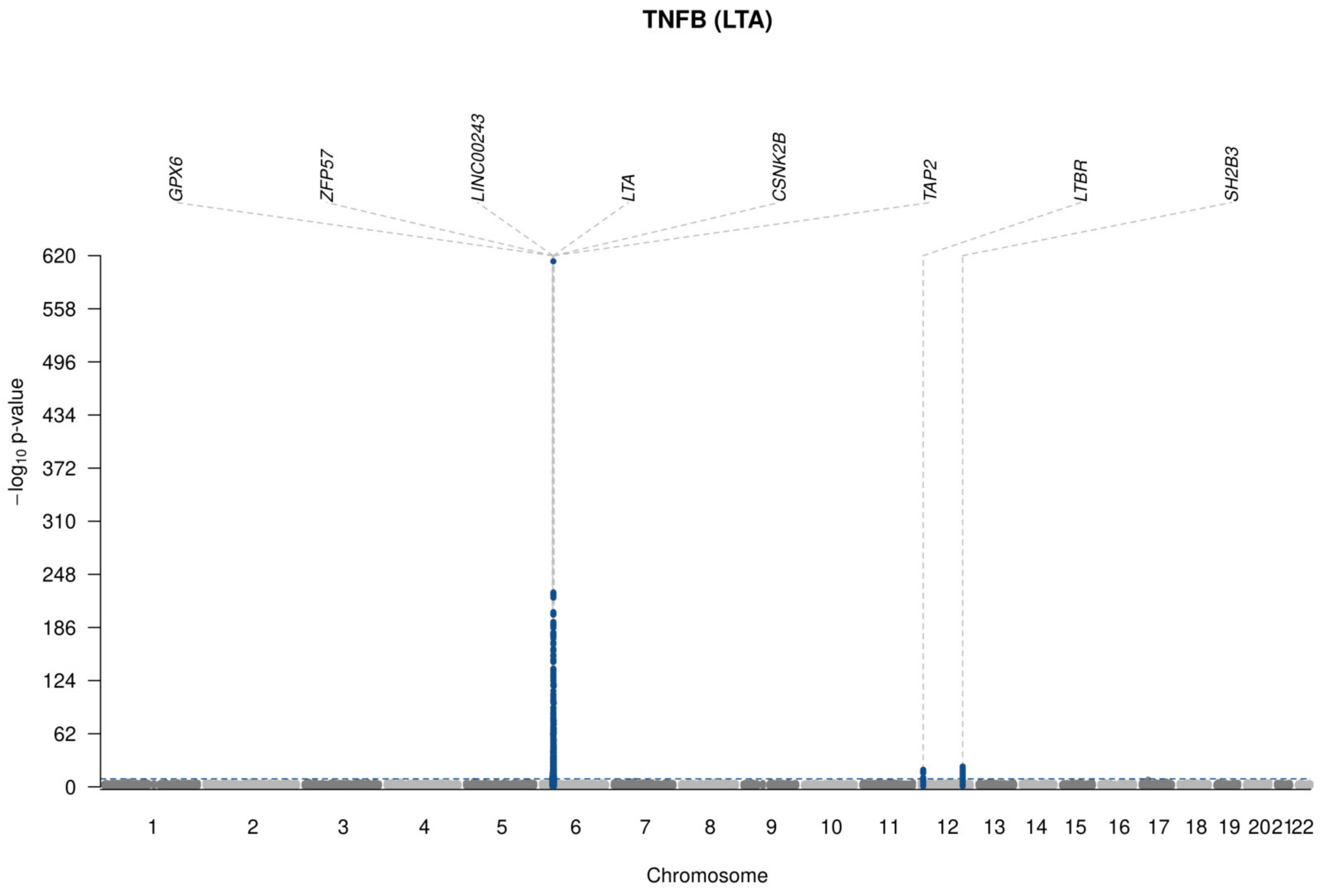

TNFB (LTA)
GPX6
ZFP57
LINC00243
LTA
CSNK2B
TAP2
LTBR
SH2B3
620
558
496
434
372
310
248
186
124
62
0
−log10 p-value
1
2
3
4
5
6
7
8
9
10
11
12
13
14
15
16
17
18
19
20
21
22
Chromosome

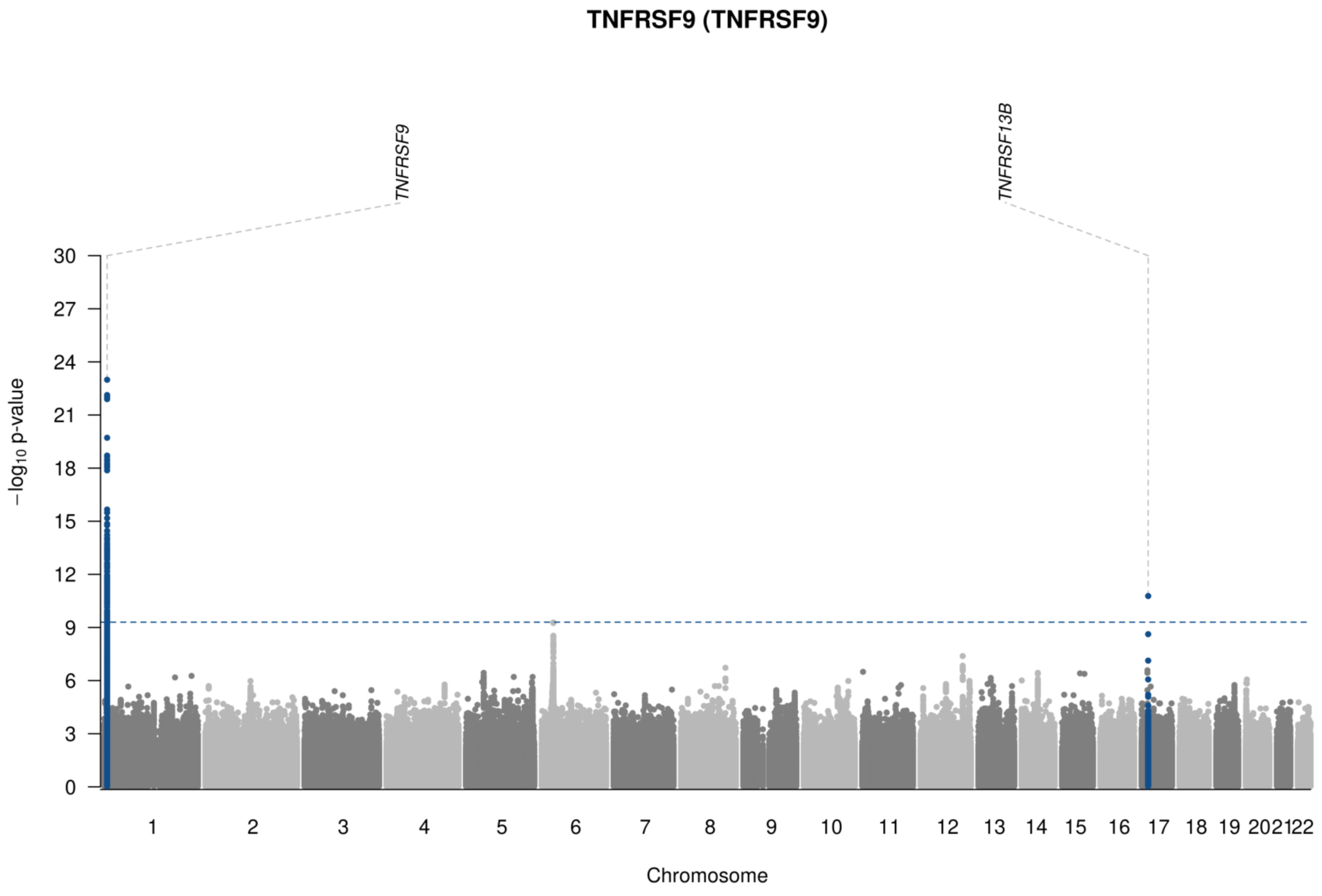

TNFRSF9 (TNFRSF9)
TNFRSF9
TNFRSF13B
−log10 p-value
30
27
24
21
18
15
12
9
6
3
0
1
2
3
4
5
6
7
8
9
10
11
12
13
14
15
16
17
18
19
20
21
22
Chromosome

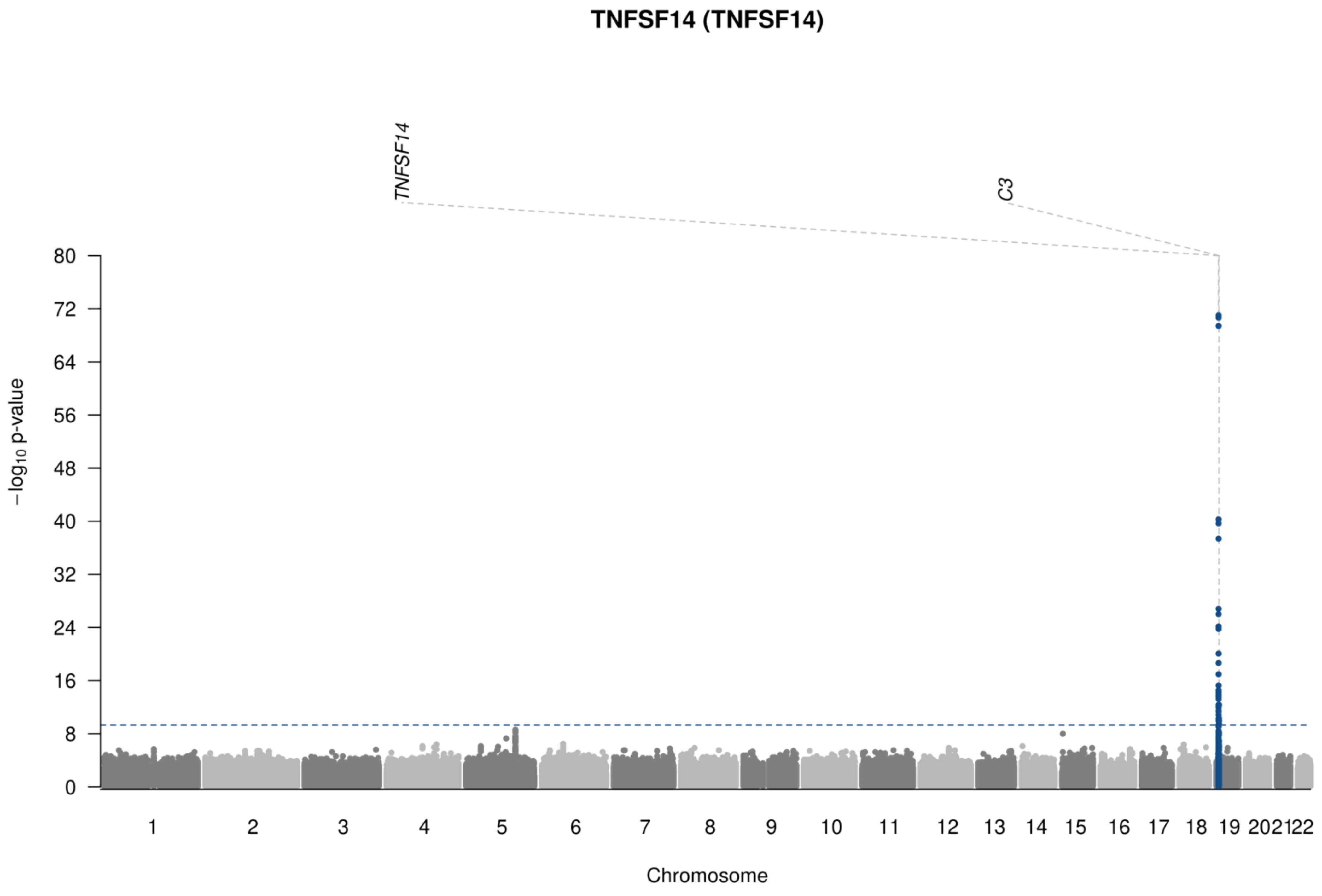

TNFSF14 (TNFSF14)
TNFSF14
C3
−log10 p-value
80
72
64
56
48
40
32
24
16
8
0
1
2
3
4
5
6
7
8
9
10
11
12
13
14
15
16
17
18
19
20
21
22
Chromosome

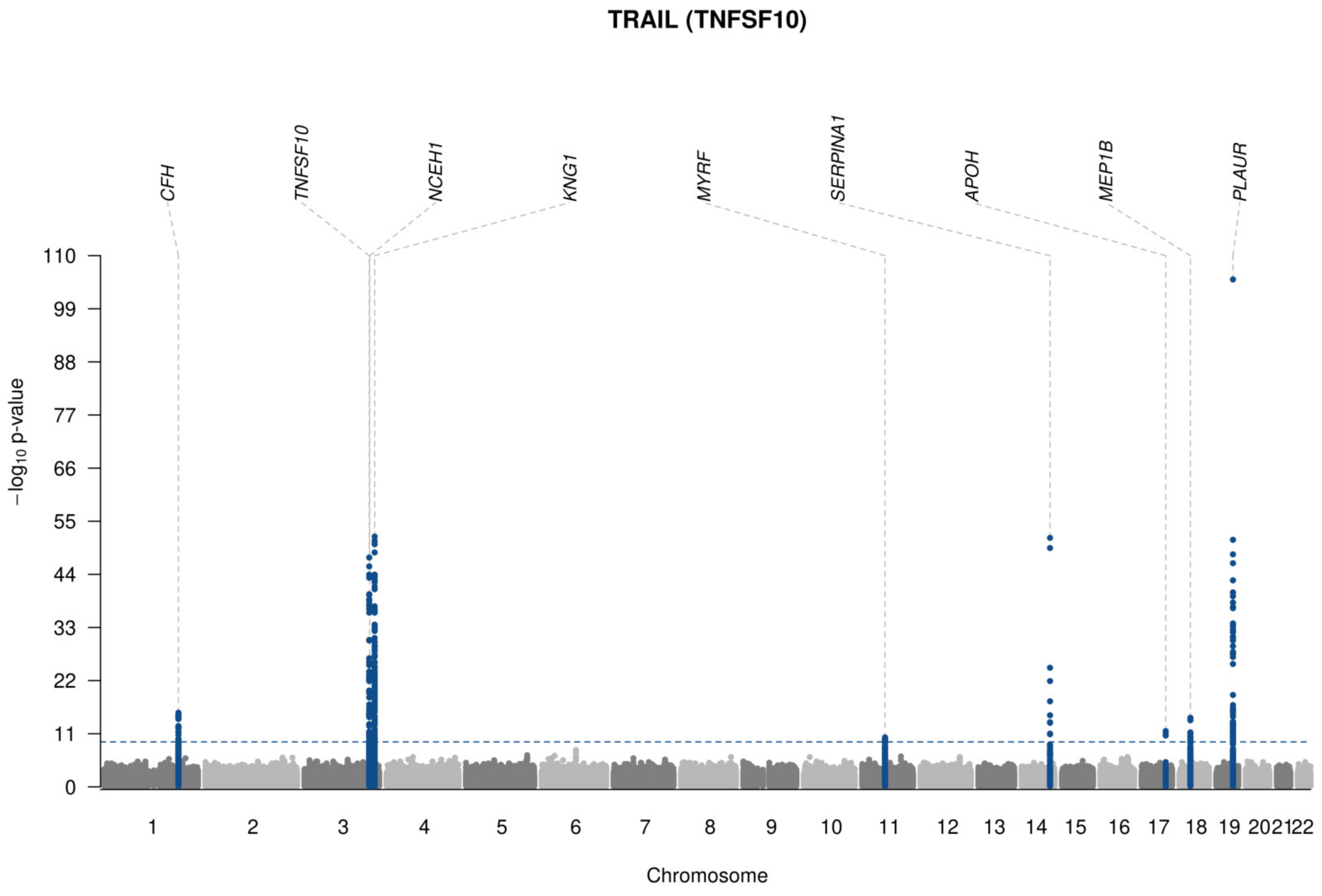

TRAIL (TNFSF10)
CFH
TNFSF10
NCEH1
KNG1
MYRF
SERPINA1
APOH
MEP1B
PLAUR
110
99
88
77
66
55
44
33
22
11
0
−log10 p-value
1
2
3
4
5
6
7
8
9
10
11
12
13
14
15
16
17
18
19
202122
Chromosome

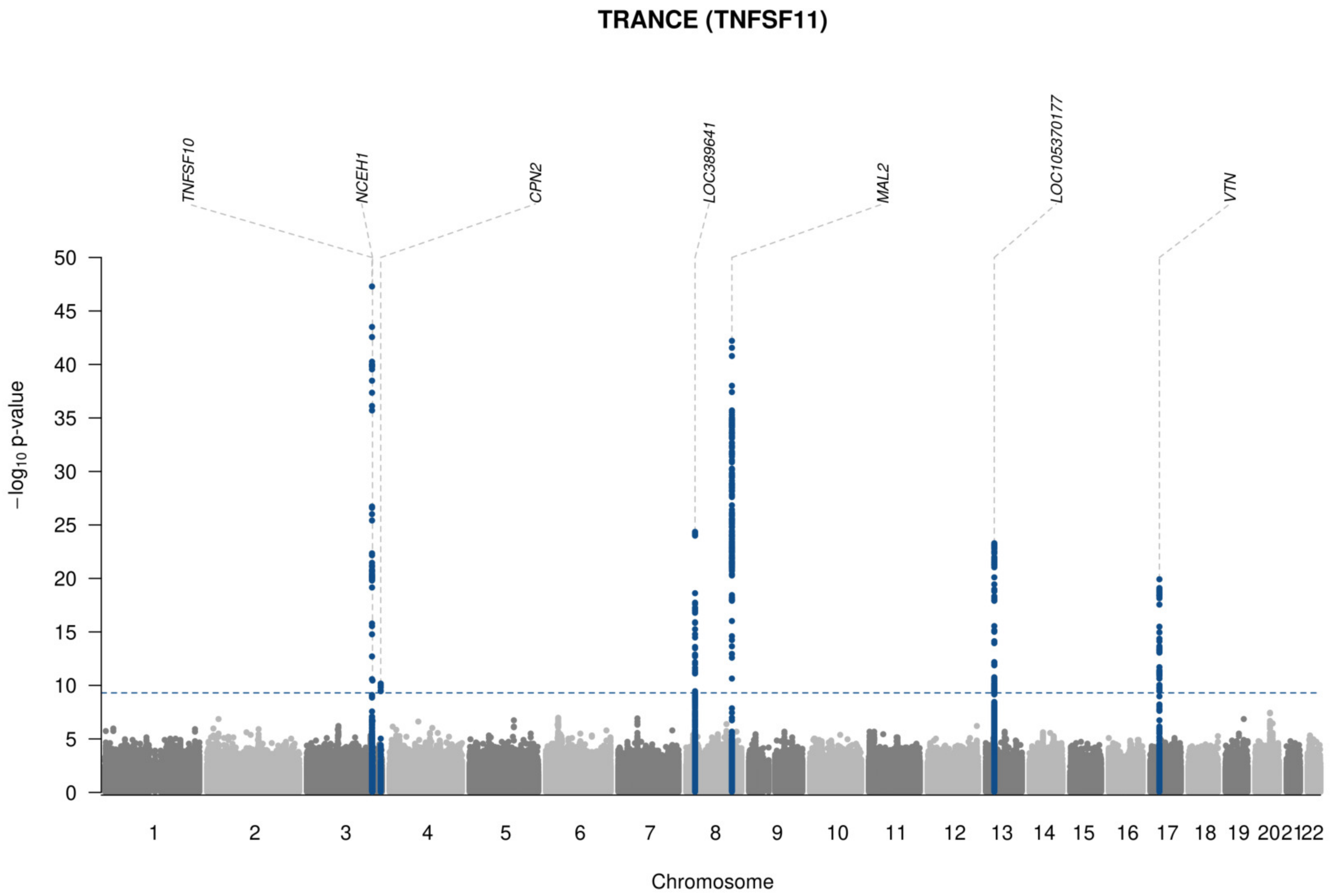

TRANCE (TNFSF11)
TNFSF10
NCEH1
CPN2
LOC389641
MAL2
LOC105370177
VTN
−log10 p-value
50
45
40
35
30
25
20
15
10
5
0
1
2
3
4
5
6
7
8
9
10
11
12
13
14
15
16
17
18
19
20
21
22
Chromosome

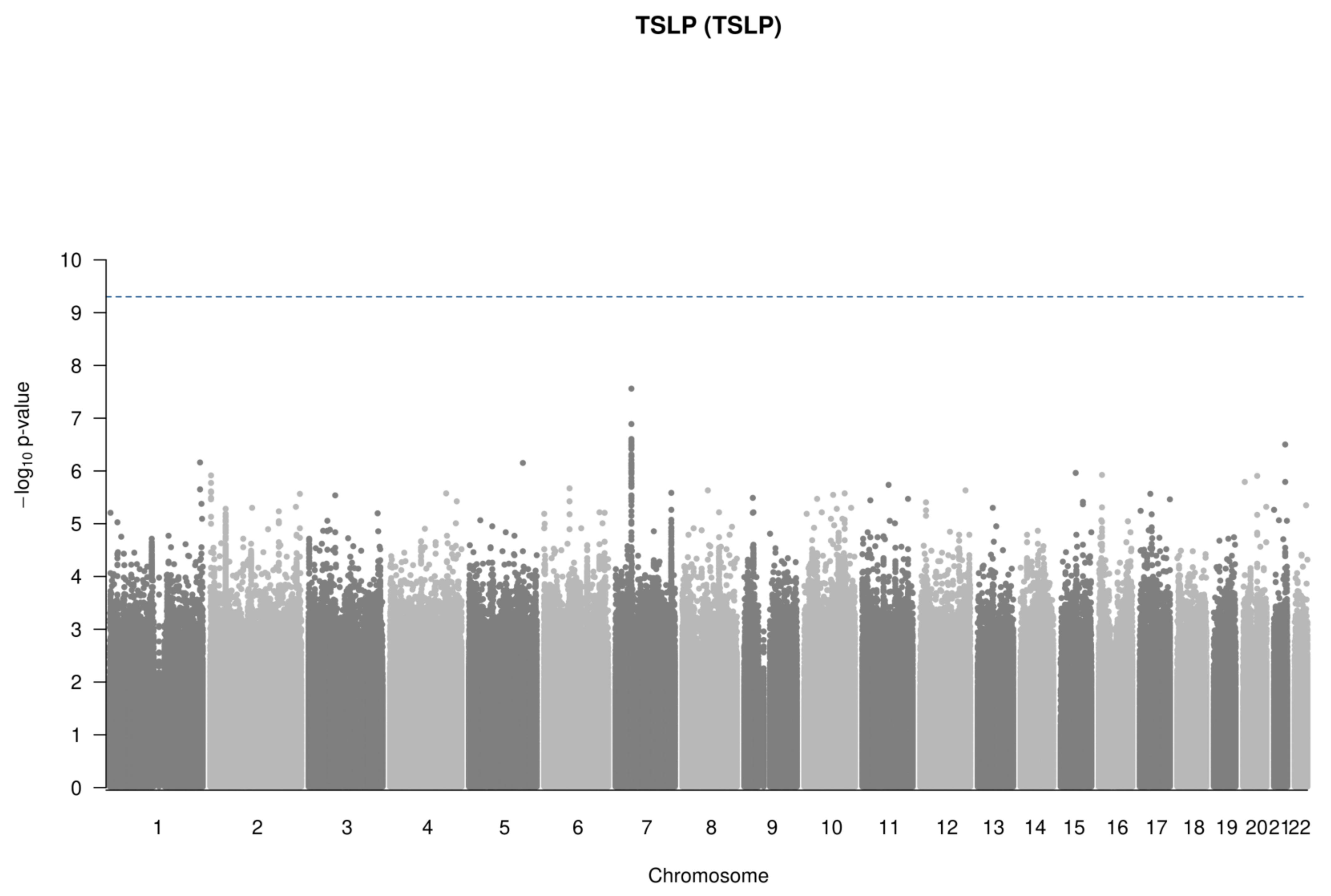

TSLP (TSLP)
−log10 p-value
0
1
2
3
4
5
6
7
8
9
10
1 2 3 4 5 6 7 8 9 10 11 12 13 14 15 16 17 18 19 20 21 22
Chromosome

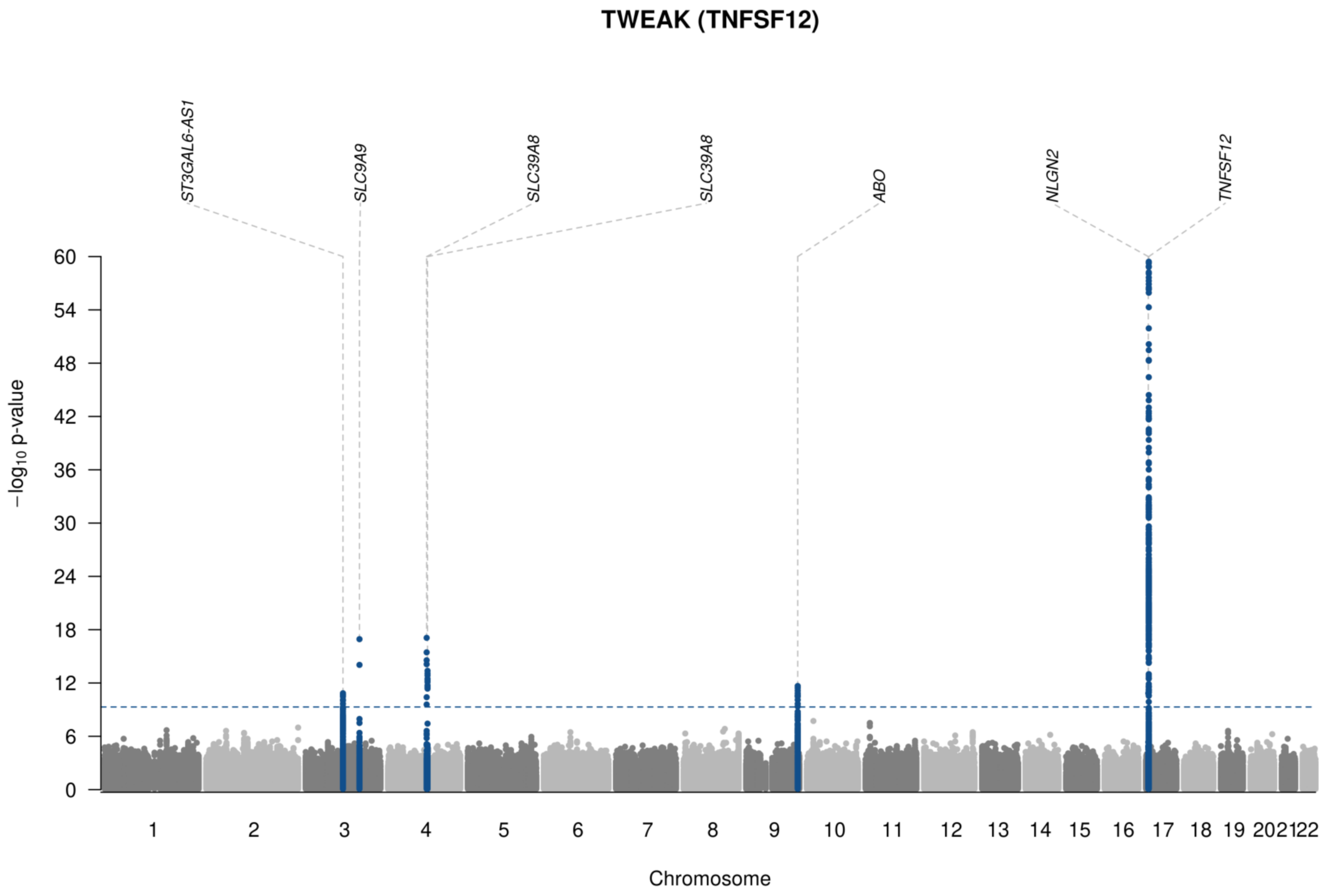

TWEAK (TNFSF12)
ST3GAL6-AS1
SLC9A9
SLC39A8
SLC39A8
ABO
NLGN2
TNFSF12
−log10 p-value
60
54
48
42
36
30
24
18
12
6
0
1
2
3
4
5
6
7
8
9
10
11
12
13
14
15
16
17
18
19
20
21
22
Chromosome

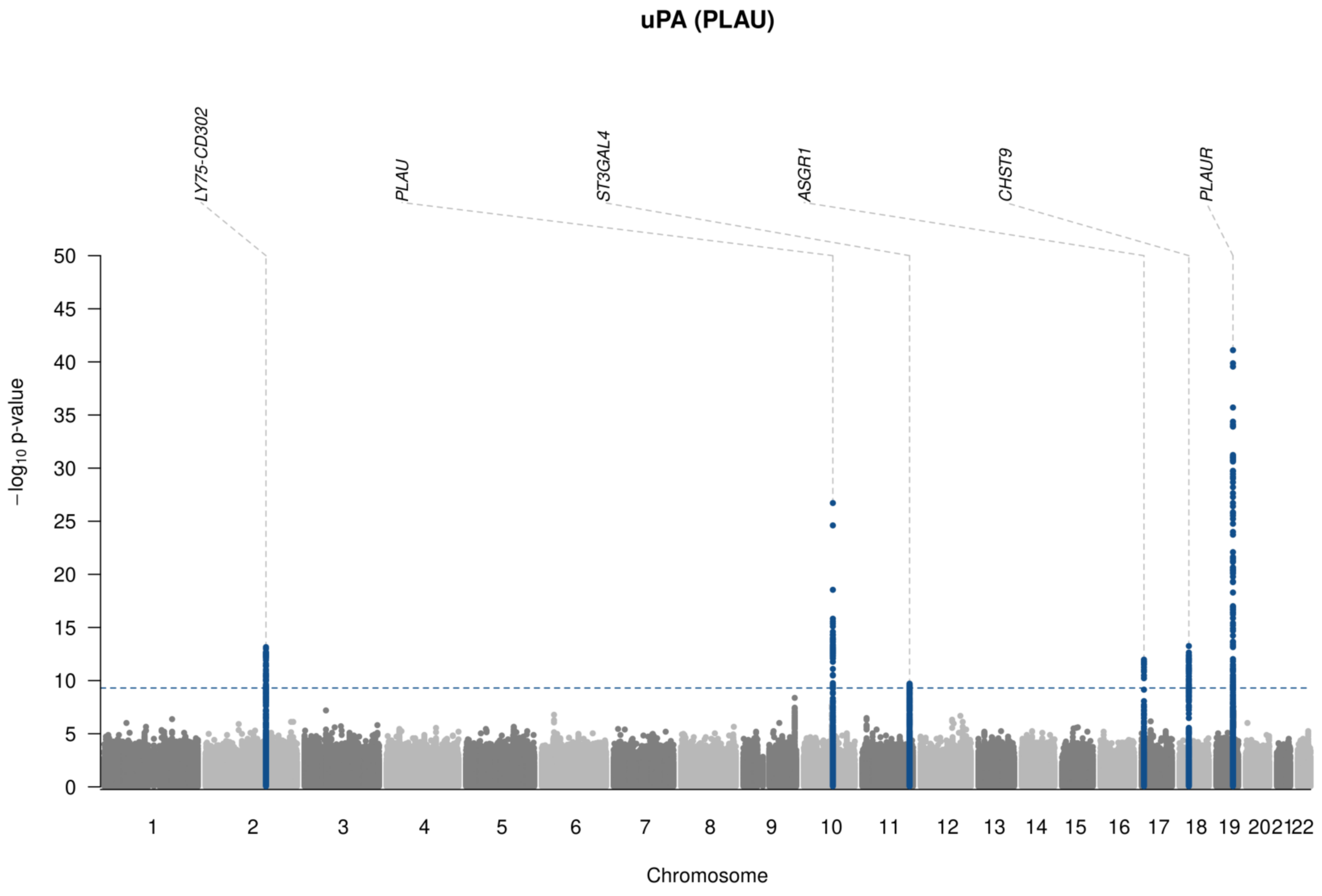

uPA (PLAU)
LY75-CD302
PLAU
ST3GAL4
ASGR1
CHST9
PLAUR
50
45
40
35
30
25
20
15
10
5
0
−log10 p-value
1
2
3
4
5
6
7
8
9
10
11
12
13
14
15
16
17
18
19
20
21
22
Chromosome

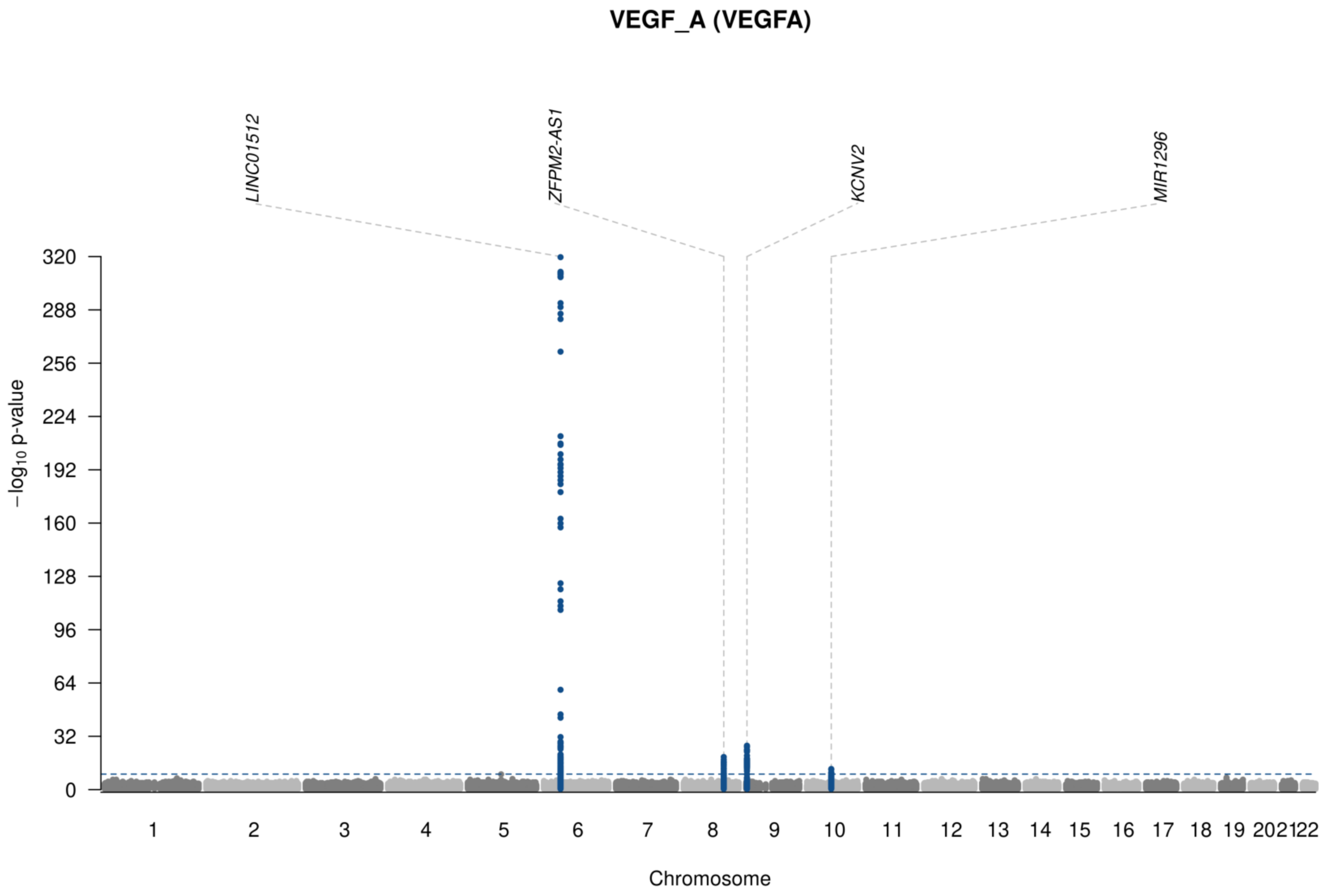

VEGF_A (VEGFA)
LINC01512
ZFPM2-AS1
KCNV2
MIR1296
320
288
256
224
192
160
128
96
64
32
0
−log10 p-value
1
2
3
4
5
6
7
8
9
10
11
12
13
14
15
16
17
18
19
202122
Chromosome

Supplementary Figure 2. Forest plots and regional association plots for the 180 pQTLs. Left panels: forest plots show the effect estimate and 95% confidence intervals in each cohort. Metrics of heterogeneity are provided. N= number of individuals. Right panels: locuszoom plots of the regional associations. Proteins are labelled with both the label provided by Olink, and with the encoding gene symbol in parentheses. Two-sided *P*-values are from meta-analysis of linear regression estimates.

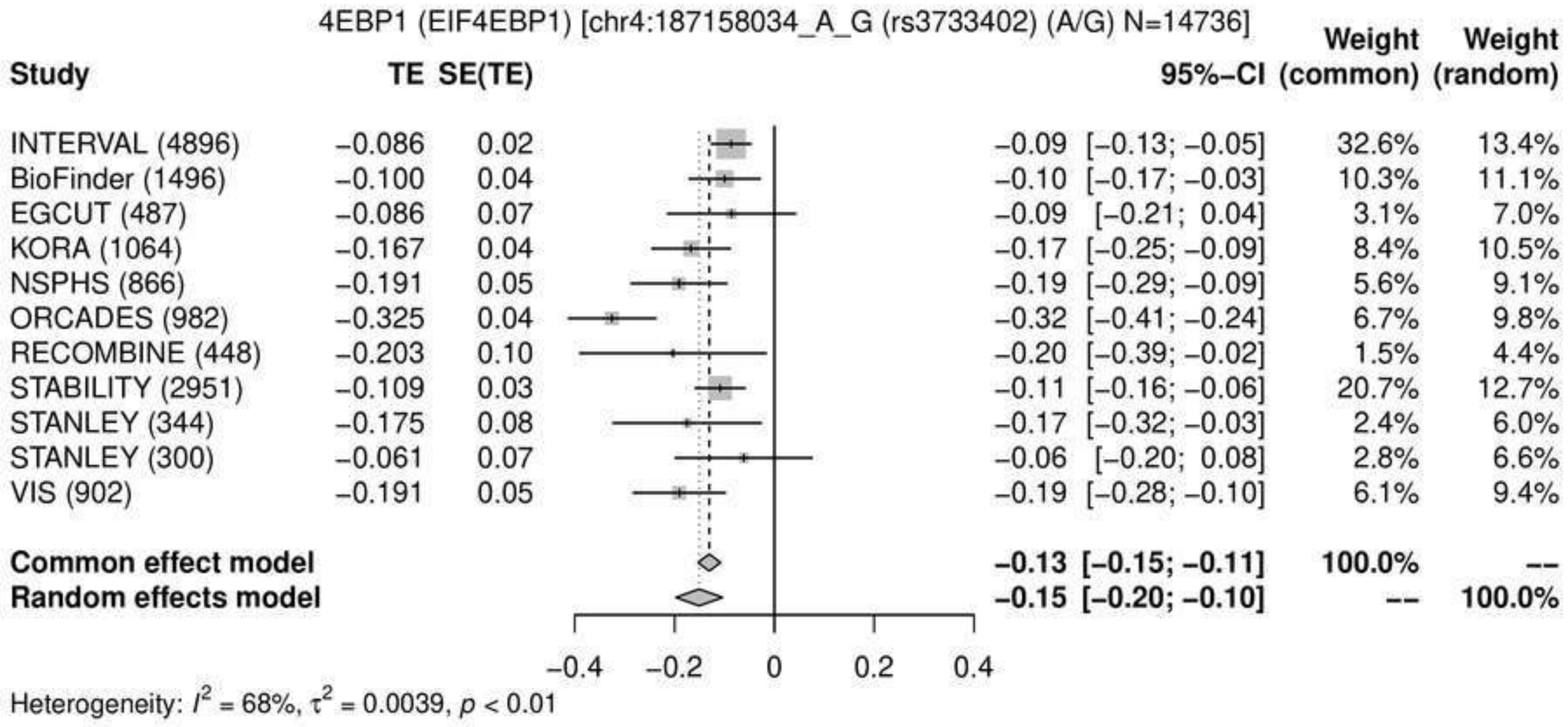
4EBP1 (EIF4EBP1) [chr4:187158034_A_G (rs3733402) (A/G) N=14736]
Study
TE
SE(TE)
95%-CI
Weight (common)
Weight (random)
INTERVAL (4896) −0.086 0.02 −0.09 [−0.13; −0.05] 32.6% 13.4%
BioFinder (1496) −0.100 0.04 −0.10 [−0.17; −0.03] 10.3% 11.1%
EGCUT (487) −0.086 0.07 −0.09 [−0.21; 0.04] 3.1% 7.0%
KORA (1064) −0.167 0.04 −0.17 [−0.25; −0.09] 8.4% 10.5%
NSPHS (866) −0.191 0.05 −0.19 [−0.29; −0.09] 5.6% 9.1%
ORCADES (982) −0.325 0.04 −0.32 [−0.41; −0.24] 6.7% 9.8%
RECOMBINE (448) −0.203 0.10 −0.20 [−0.39; −0.02] 1.5% 4.4%
STABILITY (2951) −0.109 0.03 −0.11 [−0.16; −0.06] 20.7% 12.7%
STANLEY (344) −0.175 0.08 −0.17 [−0.32; −0.03] 2.4% 6.0%
STANLEY (300) −0.061 0.07 −0.06 [−0.20; 0.08] 2.8% 6.6%
VIS (902) −0.191 0.05 −0.19 [−0.28; −0.10] 6.1% 9.4%
Common effect model −0.13 [−0.15; −0.11] 100.0% --
Random effects model −0.15 [−0.20; −0.10] -- 100.0%
−0.4 −0.2 0 0.2 0.4
Heterogeneity: I^2 = 68%, τ^2 = 0.0039, p < 0.01

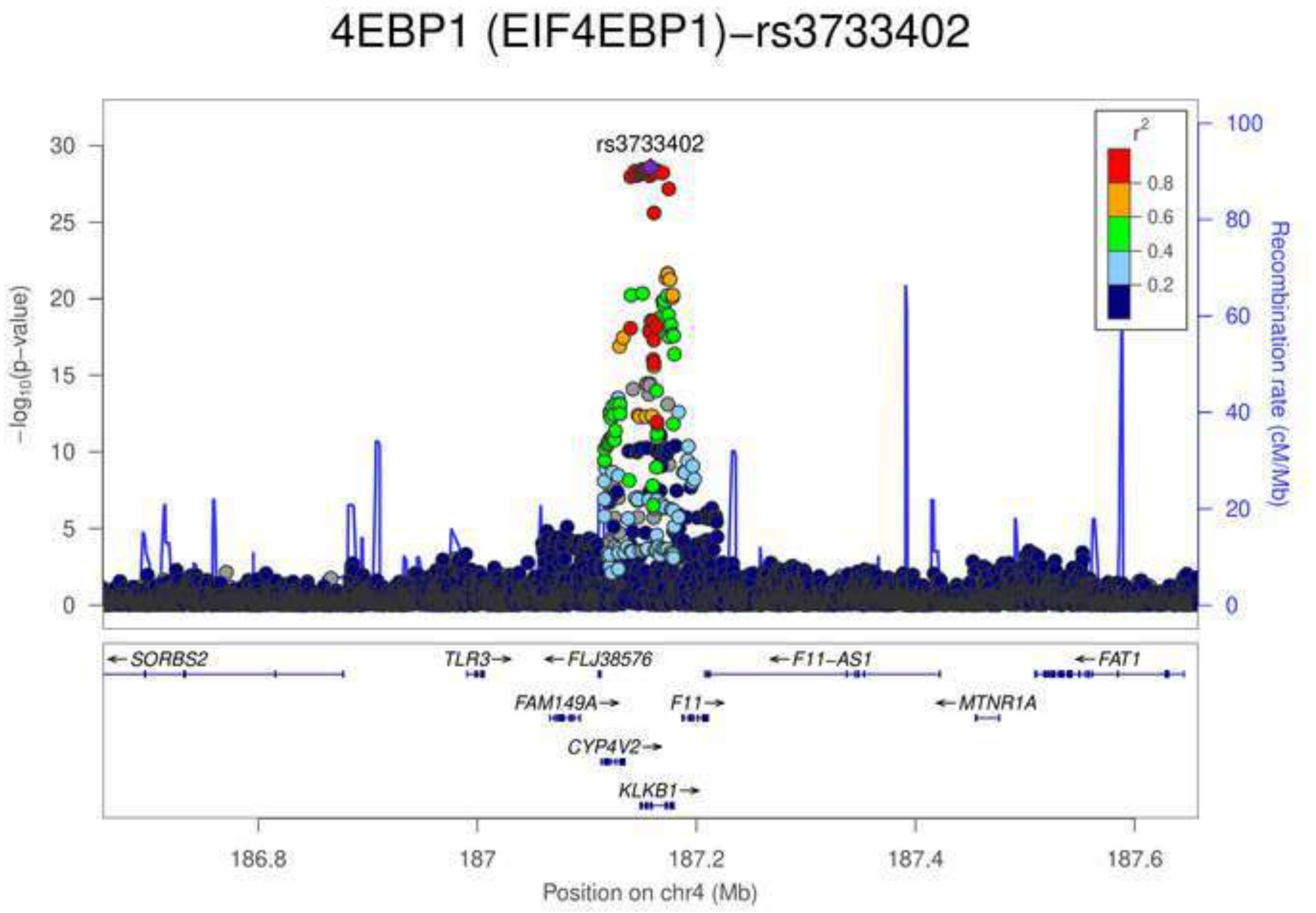
4EBP1 (EIF4EBP1)−rs3733402
rs3733402
$-\log_{10}$(p−value)
Recombination rate (cM/Mb)
r^2
0.8
0.6
0.4
0.2
SORBS2
TLR3
FLJ38576
F11−AS1
FAT1
FAM149A
F11
MTNR1A
CYP4V2
KLKB1
186.8 187 187.2 187.4 187.6
Position on chr4 (Mb)

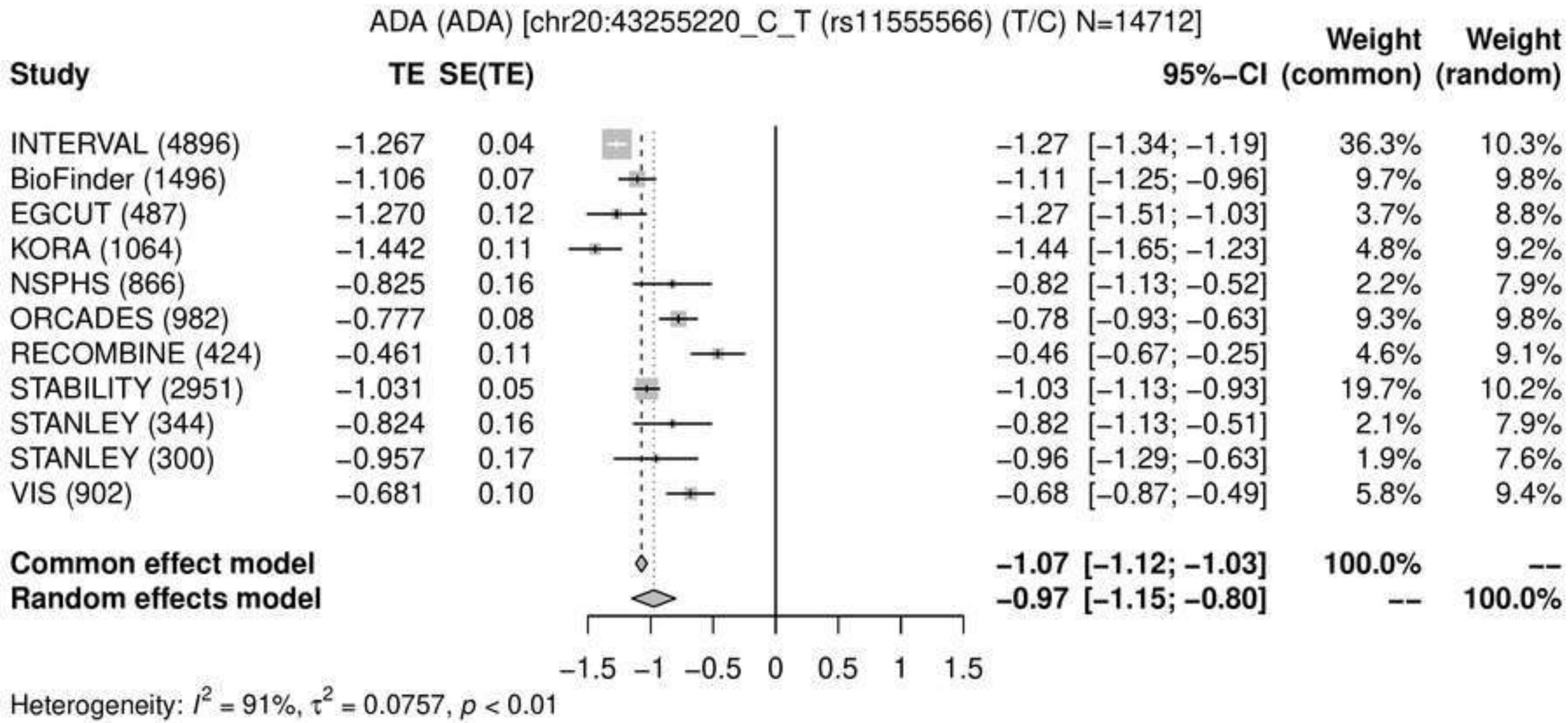
ADA (ADA) [chr20:43255220_C_T (rs11555566) (T/C) N=14712]
Study
TE
SE(TE)
95%-CI
Weight (common)
Weight (random)
INTERVAL (4896) −1.267 0.04 −1.27 [−1.34; −1.19] 36.3% 10.3%
BioFinder (1496) −1.106 0.07 −1.11 [−1.25; −0.96] 9.7% 9.8%
EGCUT (487) −1.270 0.12 −1.27 [−1.51; −1.03] 3.7% 8.8%
KORA (1064) −1.442 0.11 −1.44 [−1.65; −1.23] 4.8% 9.2%
NSPHS (866) −0.825 0.16 −0.82 [−1.13; −0.52] 2.2% 7.9%
ORCADES (982) −0.777 0.08 −0.78 [−0.93; −0.63] 9.3% 9.8%
RECOMBINE (424) −0.461 0.11 −0.46 [−0.67; −0.25] 4.6% 9.1%
STABILITY (2951) −1.031 0.05 −1.03 [−1.13; −0.93] 19.7% 10.2%
STANLEY (344) −0.824 0.16 −0.82 [−1.13; −0.51] 2.1% 7.9%
STANLEY (300) −0.957 0.17 −0.96 [−1.29; −0.63] 1.9% 7.6%
VIS (902) −0.681 0.10 −0.68 [−0.87; −0.49] 5.8% 9.4%
Common effect model −1.07 [−1.12; −1.03] 100.0% --
Random effects model −0.97 [−1.15; −0.80] -- 100.0%
−1.5 −1 −0.5 0 0.5 1 1.5
Heterogeneity: I^2 = 91%, τ^2 = 0.0757, p < 0.01

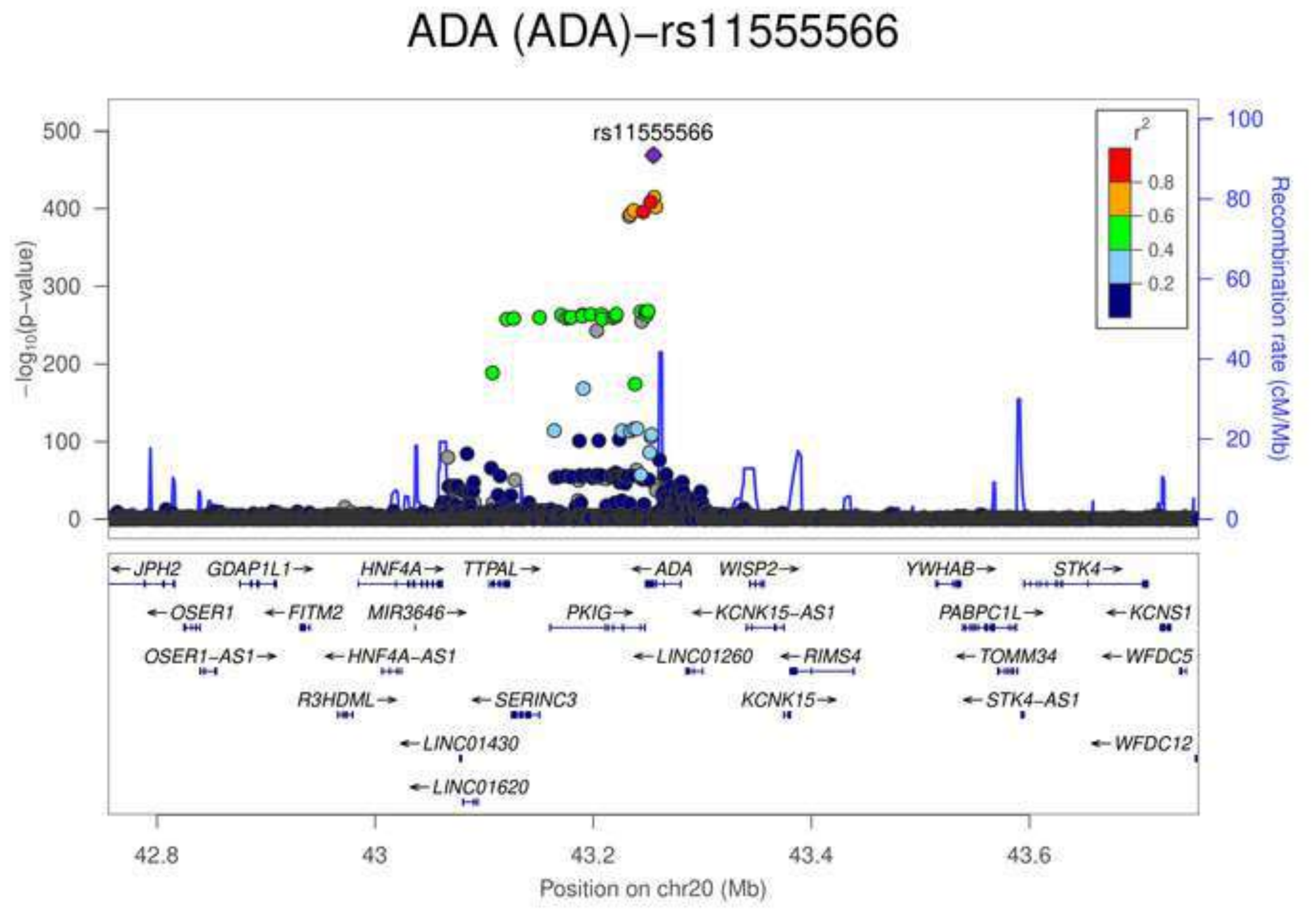
ADA (ADA)−rs11555566
rs11555566
$-\log_{10}$(p−value)
Recombination rate (cM/Mb)
r^2
0.8
0.6
0.4
0.2
0
100
200
300
400
500
20
40
60
80
JPH2
GDAP1L1
HNF4A
TTPAL
ADA
WISP2
YWHAB
STK4
OSER1
FITM2
MIR3646
PKIG
KCNK15−AS1
PABPC1L
KCNS1
OSER1−AS1
HNF4A−AS1
LINC01260
RIMS4
TOMM34
WFDC5
R3HDML
SERINC3
KCNK15
STK4−AS1
LINC01430
WFDC12
LINC01620
42.8
43
43.2
43.4
43.6
Position on chr20 (Mb)

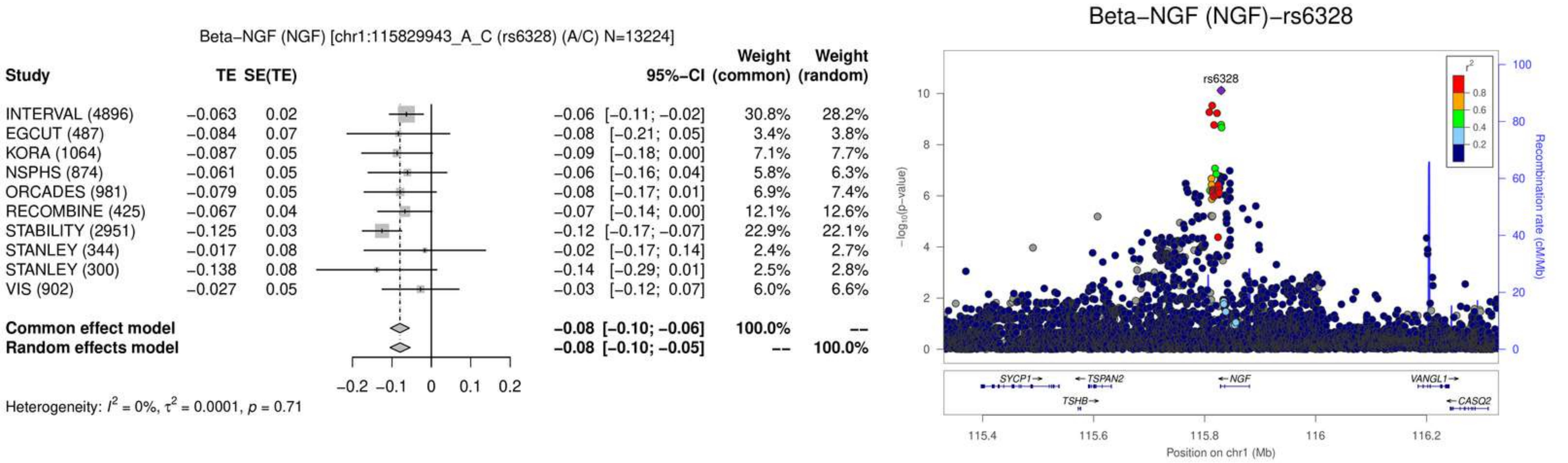

Beta−NGF (NGF) [chr1:115829943_A_C (rs6328) (A/C) N=13224]
Study
TE SE(TE)
95%−CI
Weight (common)
Weight (random)
INTERVAL (4896) −0.063 0.02 −0.06 [−0.11; −0.02] 30.8% 28.2%
EGCUT (487) −0.084 0.07 −0.08 [−0.21; 0.05] 3.4% 3.8%
KORA (1064) −0.087 0.05 −0.09 [−0.18; 0.00] 7.1% 7.7%
NSPHS (874) −0.061 0.05 −0.06 [−0.16; 0.04] 5.8% 6.3%
ORCADES (981) −0.079 0.05 −0.08 [−0.17; 0.01] 6.9% 7.4%
RECOMBINE (425) −0.067 0.04 −0.07 [−0.14; 0.00] 12.1% 12.6%
STABILITY (2951) −0.125 0.03 −0.12 [−0.17; −0.07] 22.9% 22.1%
STANLEY (344) −0.017 0.08 −0.02 [−0.17; 0.14] 2.4% 2.7%
STANLEY (300) −0.138 0.08 −0.14 [−0.29; 0.01] 2.5% 2.8%
VIS (902) −0.027 0.05 −0.03 [−0.12; 0.07] 6.0% 6.6%
Common effect model −0.08 [−0.10; −0.06] 100.0% --
Random effects model −0.08 [−0.10; −0.05] -- 100.0%
−0.2 −0.1 0 0.1 0.2
Heterogeneity: I^2 = 0%, τ^2 = 0.0001, p = 0.71
Beta−NGF (NGF)−rs6328
rs6328
r^2
0.8
0.6
0.4
0.2
$-\log_{10}$(p−value)
0
2
4
6
8
10
Recombination rate (cM/Mb)
0
20
40
60
80
100
SYCP1
TSPAN2
NGF
VANGL1
TSHB
CASQ2
115.4
115.6
115.8
116
116.2
Position on chr1 (Mb)

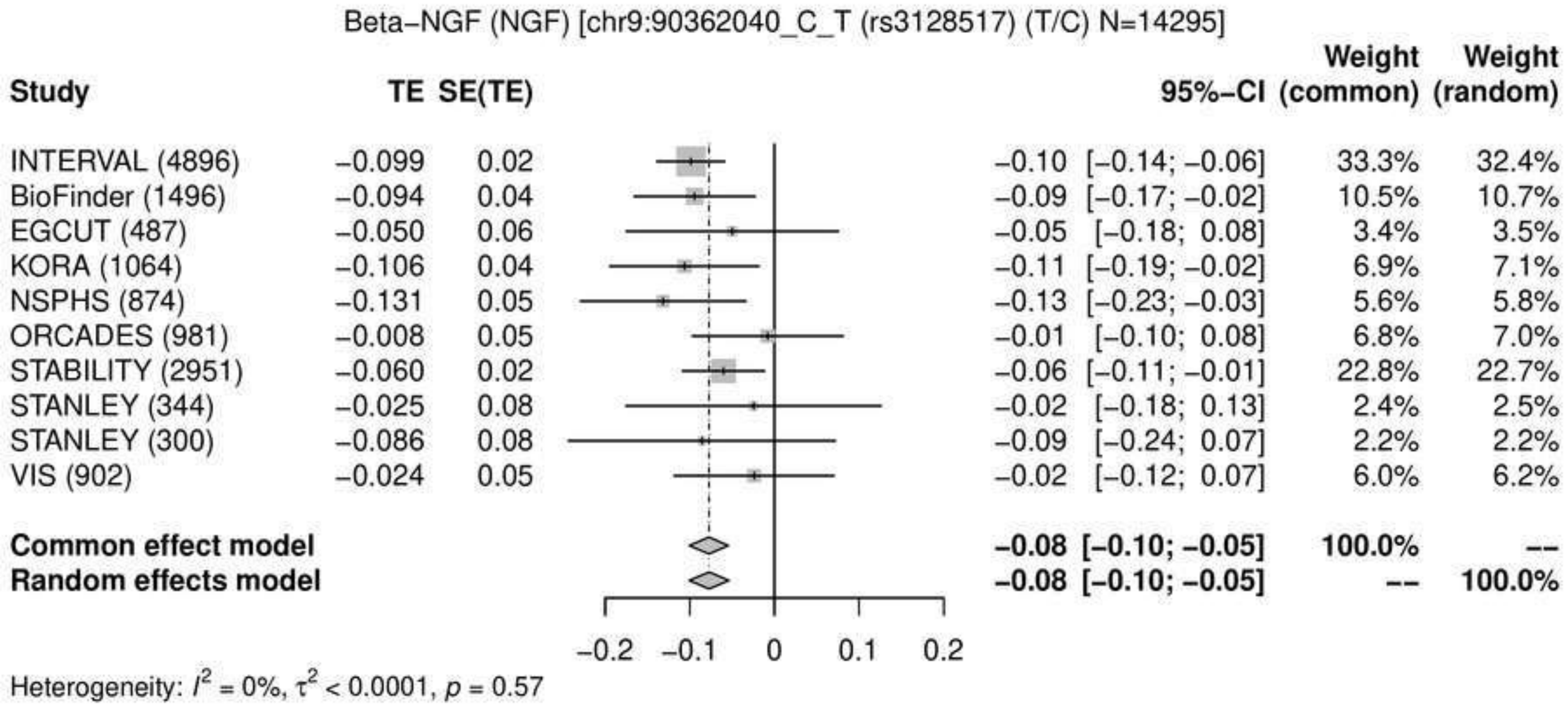
Beta−NGF (NGF) [chr9:90362040_C_T (rs3128517) (T/C) N=14295]
Study
TE
SE(TE)
95%−CI
Weight (common)
Weight (random)
INTERVAL (4896) −0.099 0.02 −0.10 [−0.14; −0.06] 33.3% 32.4%
BioFinder (1496) −0.094 0.04 −0.09 [−0.17; −0.02] 10.5% 10.7%
EGCUT (487) −0.050 0.06 −0.05 [−0.18; 0.08] 3.4% 3.5%
KORA (1064) −0.106 0.04 −0.11 [−0.19; −0.02] 6.9% 7.1%
NSPHS (874) −0.131 0.05 −0.13 [−0.23; −0.03] 5.6% 5.8%
ORCADES (981) −0.008 0.05 −0.01 [−0.10; 0.08] 6.8% 7.0%
STABILITY (2951) −0.060 0.02 −0.06 [−0.11; −0.01] 22.8% 22.7%
STANLEY (344) −0.025 0.08 −0.02 [−0.18; 0.13] 2.4% 2.5%
STANLEY (300) −0.086 0.08 −0.09 [−0.24; 0.07] 2.2% 2.2%
VIS (902) −0.024 0.05 −0.02 [−0.12; 0.07] 6.0% 6.2%
Common effect model −0.08 [−0.10; −0.05] 100.0% --
Random effects model −0.08 [−0.10; −0.05] -- 100.0%
−0.2 −0.1 0 0.1 0.2
Heterogeneity: I^2 = 0%, τ^2 < 0.0001, p = 0.57

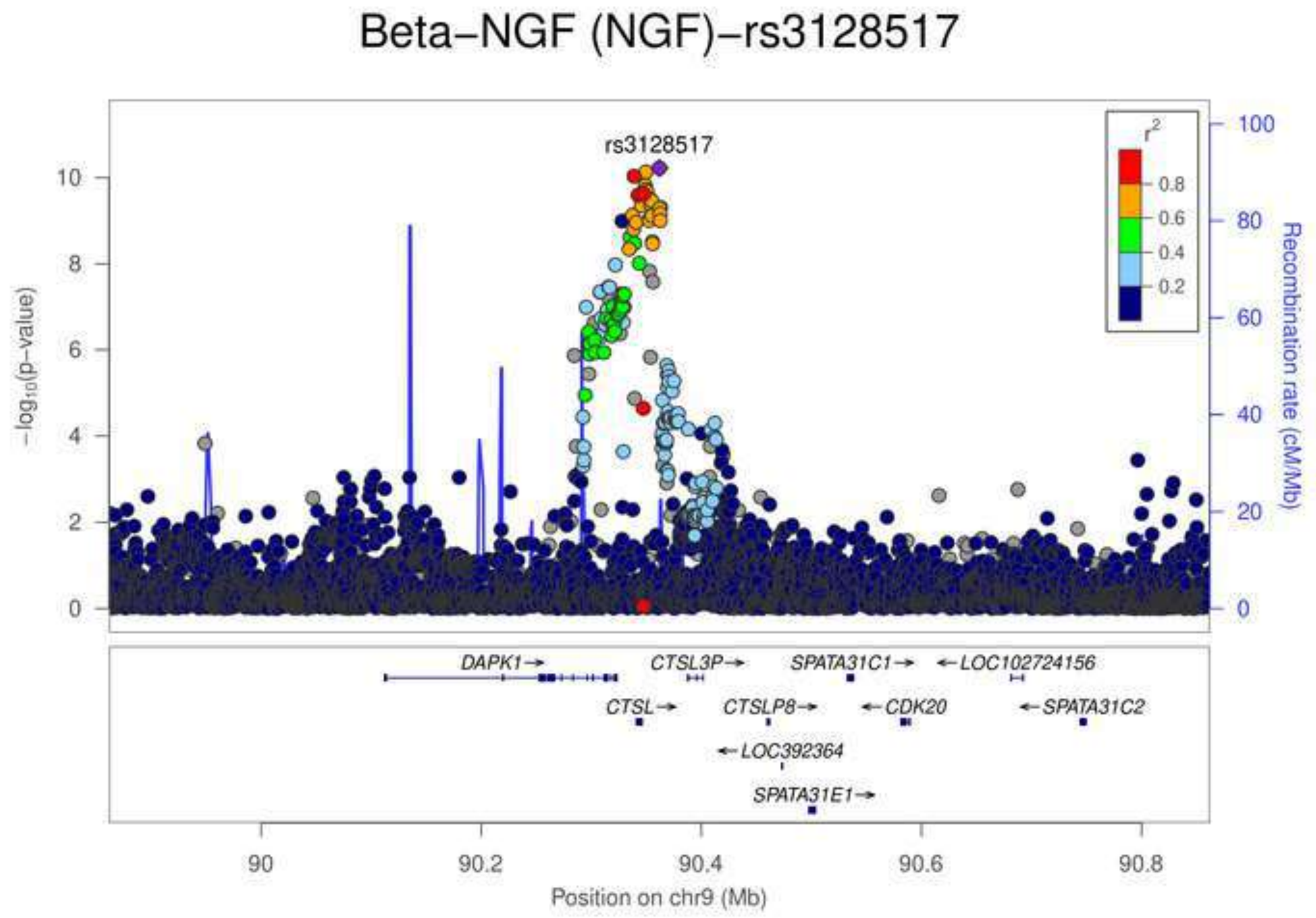
Beta−NGF (NGF)−rs3128517
rs3128517
$-\log_{10}$(p−value)
Recombination rate (cM/Mb)
r^2
0.8
0.6
0.4
0.2
DAPK1→
CTSL3P→
SPATA31C1→
←LOC102724156
CTSL→
CTSLP8→
←CDK20
←SPATA31C2
←LOC392364
SPATA31E1→
90
90.2
90.4
90.6
90.8
Position on chr9 (Mb)

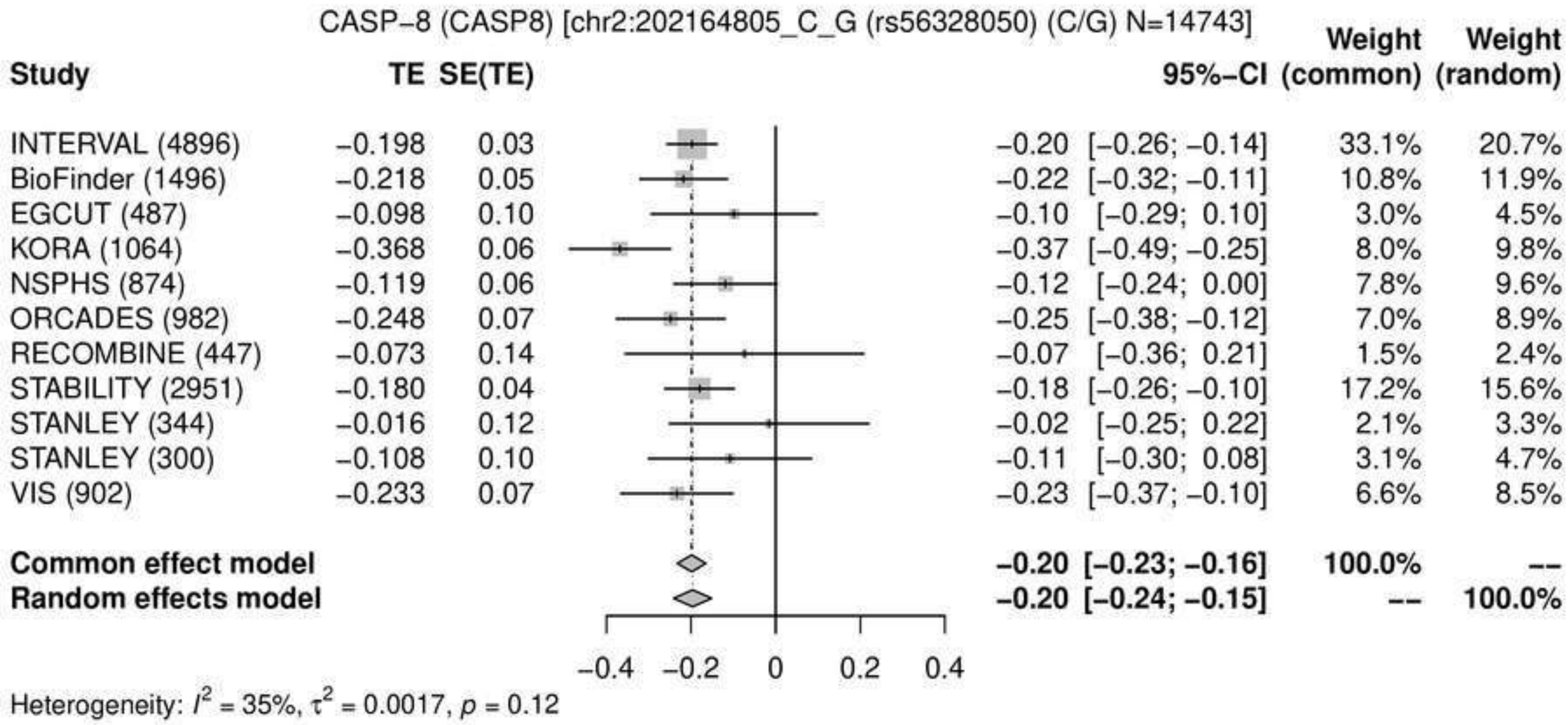
CASP-8 (CASP8) [chr2:202164805_C_G (rs56328050) (C/G) N=14743]
Study
TE
SE(TE)
95%-CI
Weight (common)
Weight (random)
INTERVAL (4896) −0.198 0.03 −0.20 [−0.26; −0.14] 33.1% 20.7%
BioFinder (1496) −0.218 0.05 −0.22 [−0.32; −0.11] 10.8% 11.9%
EGCUT (487) −0.098 0.10 −0.10 [−0.29; 0.10] 3.0% 4.5%
KORA (1064) −0.368 0.06 −0.37 [−0.49; −0.25] 8.0% 9.8%
NSPHS (874) −0.119 0.06 −0.12 [−0.24; 0.00] 7.8% 9.6%
ORCADES (982) −0.248 0.07 −0.25 [−0.38; −0.12] 7.0% 8.9%
RECOMBINE (447) −0.073 0.14 −0.07 [−0.36; 0.21] 1.5% 2.4%
STABILITY (2951) −0.180 0.04 −0.18 [−0.26; −0.10] 17.2% 15.6%
STANLEY (344) −0.016 0.12 −0.02 [−0.25; 0.22] 2.1% 3.3%
STANLEY (300) −0.108 0.10 −0.11 [−0.30; 0.08] 3.1% 4.7%
VIS (902) −0.233 0.07 −0.23 [−0.37; −0.10] 6.6% 8.5%
Common effect model −0.20 [−0.23; −0.16] 100.0% --
Random effects model −0.20 [−0.24; −0.15] -- 100.0%
−0.4 −0.2 0 0.2 0.4
Heterogeneity: I² = 35%, τ² = 0.0017, p = 0.12

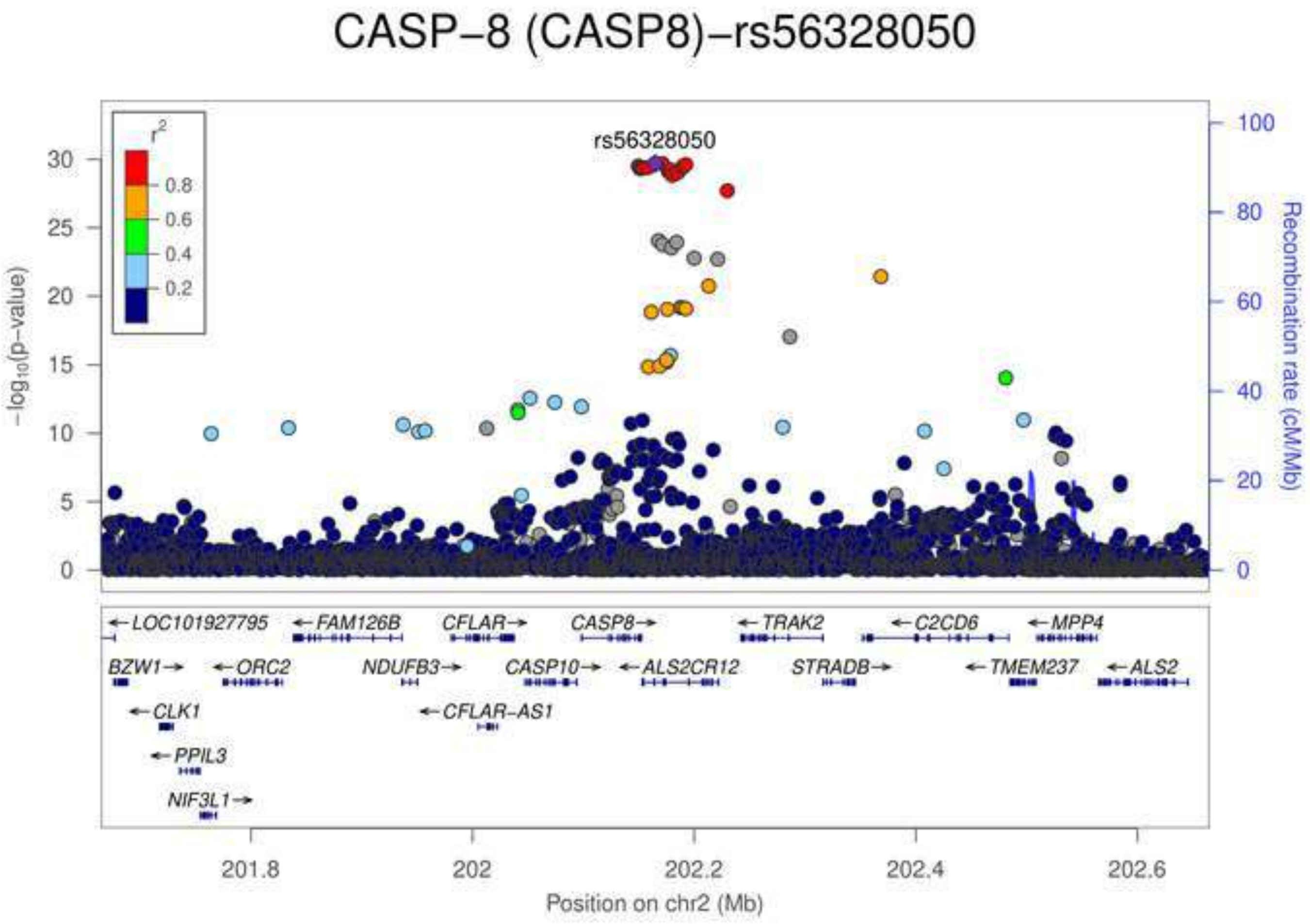
CASP−8 (CASP8)−rs56328050
rs56328050
r²
0.8
0.6
0.4
0.2
−log10(p−value)
Recombination rate (cM/Mb)
LOC101927795
FAM126B
CFLAR
CASP8
TRAK2
C2CD6
MPP4
BZW1
ORC2
NDUFB3
CASP10
ALS2CR12
STRADB
TMEM237
ALS2
CLK1
CFLAR−AS1
PPIL3
NIF3L1
201.8
202
202.2
202.4
202.6
Position on chr2 (Mb)

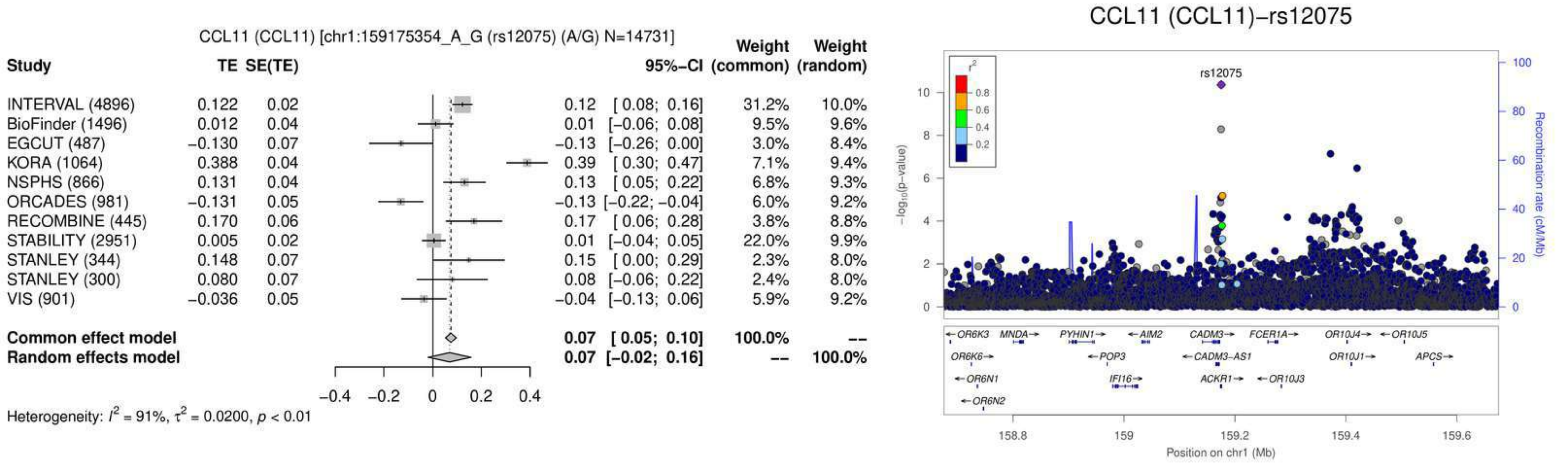
CCL11 (CCL11) [chr1:159175354_A_G (rs12075) (A/G) N=14731]
Study TE SE(TE) 95%−CI Weight (common) Weight (random)
INTERVAL (4896) 0.122 0.02 0.12 [0.08; 0.16] 31.2% 10.0%
BioFinder (1496) 0.012 0.04 0.01 [−0.06; 0.08] 9.5% 9.6%
EGCUT (487) −0.130 0.07 −0.13 [−0.26; 0.00] 3.0% 8.4%
KORA (1064) 0.388 0.04 0.39 [0.30; 0.47] 7.1% 9.4%
NSPHS (866) 0.131 0.04 0.13 [0.05; 0.22] 6.8% 9.3%
ORCADES (981) −0.131 0.05 −0.13 [−0.22; −0.04] 6.0% 9.2%
RECOMBINE (445) 0.170 0.06 0.17 [0.06; 0.28] 3.8% 8.8%
STABILITY (2951) 0.005 0.02 0.01 [−0.04; 0.05] 22.0% 9.9%
STANLEY (344) 0.148 0.07 0.15 [0.00; 0.29] 2.3% 8.0%
STANLEY (300) 0.080 0.07 0.08 [−0.06; 0.22] 2.4% 8.0%
VIS (901) −0.036 0.05 −0.04 [−0.13; 0.06] 5.9% 9.2%
Common effect model 0.07 [0.05; 0.10] 100.0% --
Random effects model 0.07 [−0.02; 0.16] -- 100.0%
−0.4 −0.2 0 0.2 0.4
Heterogeneity: I^2 = 91%, τ^2 = 0.0200, p < 0.01
CCL11 (CCL11)−rs12075
rs12075
r^2
0.8
0.6
0.4
0.2
$-\log_{10}$(p−value)
Recombination rate (cM/Mb)
OR6K3 MNDA PYHIN1 AIM2 CADM3 FCER1A OR10J4 OR10J5
OR6K6 POP3 CADM3-AS1 OR10J1 APCS
OR6N1 IFI16 ACKR1 OR10J3
OR6N2
158.8 159 159.2 159.4 159.6
Position on chr1 (Mb)

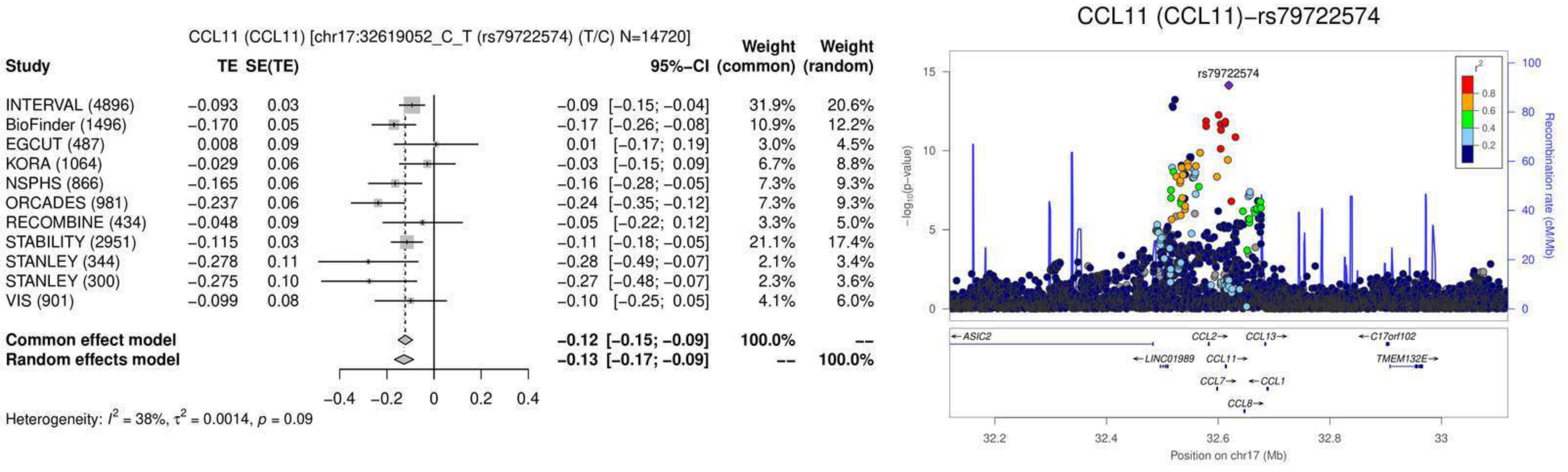

CCL11 (CCL11) [chr17:32619052_C_T (rs79722574) (T/C) N=14720]
Study
TE
SE(TE)
95%-CI
Weight (common)
Weight (random)
INTERVAL (4896) −0.093 0.03 −0.09 [−0.15; −0.04] 31.9% 20.6%
BioFinder (1496) −0.170 0.05 −0.17 [−0.26; −0.08] 10.9% 12.2%
EGCUT (487) 0.008 0.09 0.01 [−0.17; 0.19] 3.0% 4.5%
KORA (1064) −0.029 0.06 −0.03 [−0.15; 0.09] 6.7% 8.8%
NSPHS (866) −0.165 0.06 −0.16 [−0.28; −0.05] 7.3% 9.3%
ORCADES (981) −0.237 0.06 −0.24 [−0.35; −0.12] 7.3% 9.3%
RECOMBINE (434) −0.048 0.09 −0.05 [−0.22; 0.12] 3.3% 5.0%
STABILITY (2951) −0.115 0.03 −0.11 [−0.18; −0.05] 21.1% 17.4%
STANLEY (344) −0.278 0.11 −0.28 [−0.49; −0.07] 2.1% 3.4%
STANLEY (300) −0.275 0.10 −0.27 [−0.48; −0.07] 2.3% 3.6%
VIS (901) −0.099 0.08 −0.10 [−0.25; 0.05] 4.1% 6.0%
Common effect model −0.12 [−0.15; −0.09] 100.0% --
Random effects model −0.13 [−0.17; −0.09] -- 100.0%
−0.4 −0.2 0 0.2 0.4
Heterogeneity: I^2 = 38%, τ^2 = 0.0014, p = 0.09
CCL11 (CCL11)−rs79722574
rs79722574
$-\log_{10}$(p-value)
Recombination rate (cM/Mb)
r^2
0.8
0.6
0.4
0.2
ASIC2
CCL2
CCL13
C17orf102
LINC01989
CCL11
TMEM132E
CCL7
CCL1
CCL8
32.2 32.4 32.6 32.8 33
Position on chr17 (Mb)

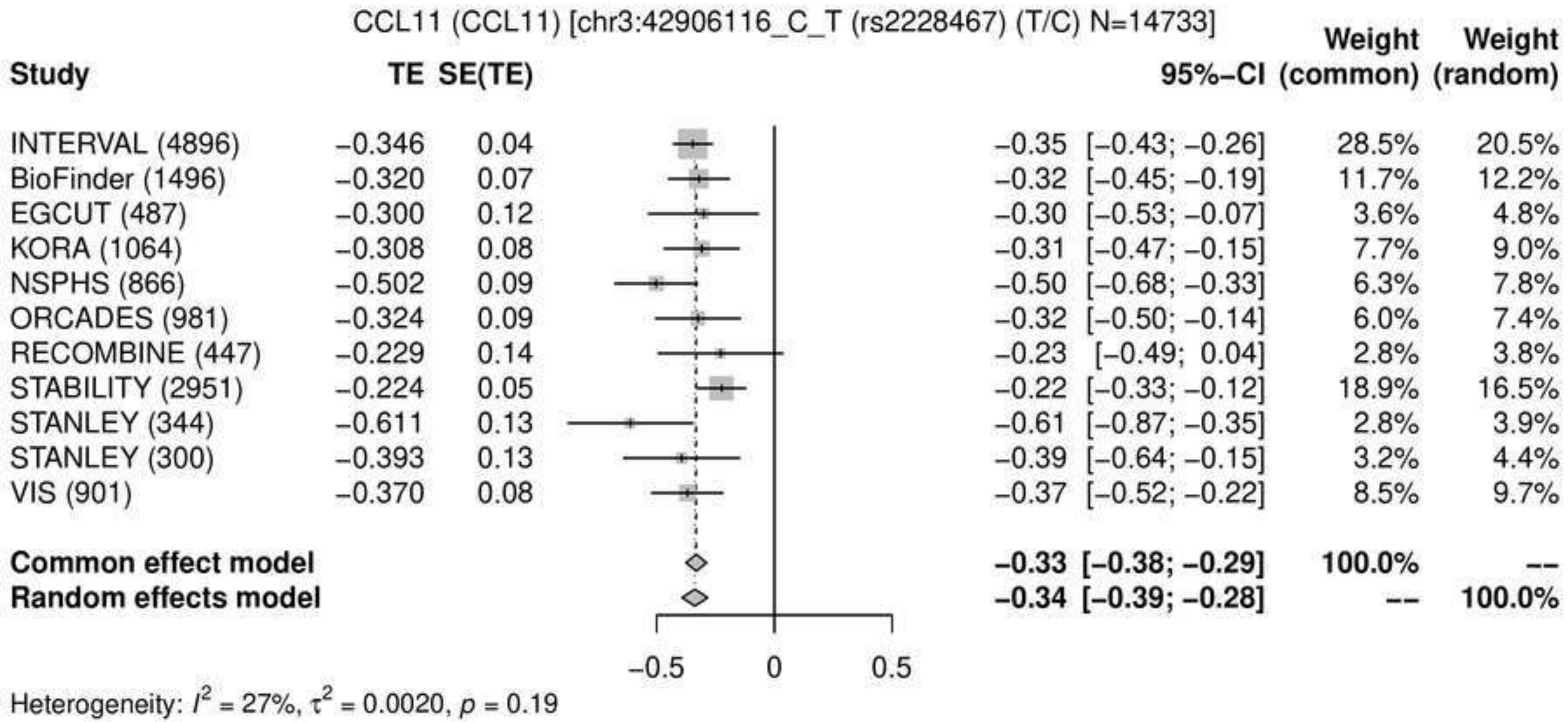

CCL11 (CCL11) [chr3:42906116_C_T (rs2228467) (T/C) N=14733]
Study
TE
SE(TE)
95%-CI
Weight (common)
Weight (random)
INTERVAL (4896) −0.346 0.04 −0.35 [−0.43; −0.26] 28.5% 20.5%
BioFinder (1496) −0.320 0.07 −0.32 [−0.45; −0.19] 11.7% 12.2%
EGCUT (487) −0.300 0.12 −0.30 [−0.53; −0.07] 3.6% 4.8%
KORA (1064) −0.308 0.08 −0.31 [−0.47; −0.15] 7.7% 9.0%
NSPHS (866) −0.502 0.09 −0.50 [−0.68; −0.33] 6.3% 7.8%
ORCADES (981) −0.324 0.09 −0.32 [−0.50; −0.14] 6.0% 7.4%
RECOMBINE (447) −0.229 0.14 −0.23 [−0.49; 0.04] 2.8% 3.8%
STABILITY (2951) −0.224 0.05 −0.22 [−0.33; −0.12] 18.9% 16.5%
STANLEY (344) −0.611 0.13 −0.61 [−0.87; −0.35] 2.8% 3.9%
STANLEY (300) −0.393 0.13 −0.39 [−0.64; −0.15] 3.2% 4.4%
VIS (901) −0.370 0.08 −0.37 [−0.52; −0.22] 8.5% 9.7%
Common effect model −0.33 [−0.38; −0.29] 100.0% --
Random effects model −0.34 [−0.39; −0.28] -- 100.0%
−0.5
0
0.5
Heterogeneity: I^2 = 27%, τ^2 = 0.0020, p = 0.19

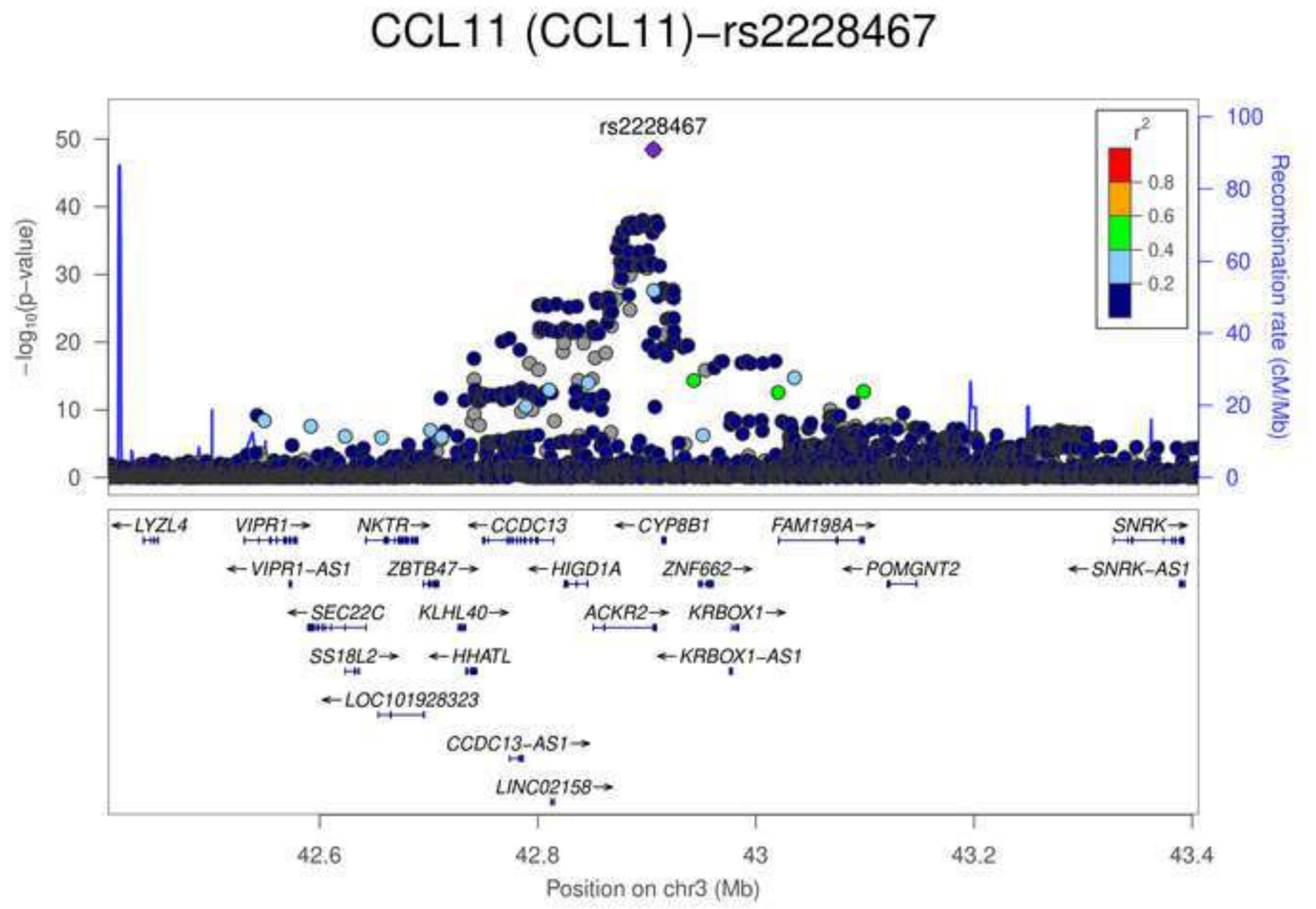

CCL11 (CCL11)−rs2228467
rs2228467
$-\log_{10}$(p-value)
Recombination rate (cM/Mb)
r^2
0.8
0.6
0.4
0.2
LYZL4
VIPR1
NKTR
CCDC13
CYP8B1
FAM198A
SNRK
VIPR1-AS1
ZBTB47
HIGD1A
ZNF662
POMGNT2
SNRK-AS1
SEC22C
KLHL40
ACKR2
KRBOX1
SS18L2
HHATL
KRBOX1-AS1
LOC101928323
CCDC13-AS1
LINC02158
42.6
42.8
43
43.2
43.4
Position on chr3 (Mb)

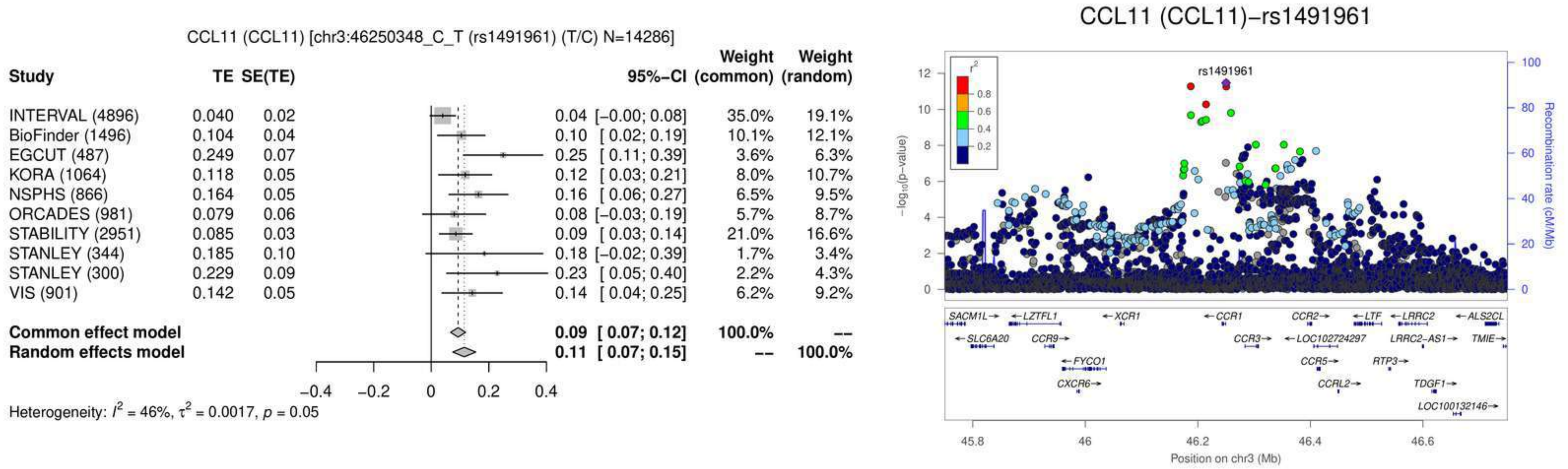
CCL11 (CCL11) [chr3:46250348_C_T (rs1491961) (T/C) N=14286]
Study TE SE(TE) 95%−CI Weight (common) Weight (random)
INTERVAL (4896) 0.040 0.02 0.04 [−0.00; 0.08] 35.0% 19.1%
BioFinder (1496) 0.104 0.04 0.10 [0.02; 0.19] 10.1% 12.1%
EGCUT (487) 0.249 0.07 0.25 [0.11; 0.39] 3.6% 6.3%
KORA (1064) 0.118 0.05 0.12 [0.03; 0.21] 8.0% 10.7%
NSPHS (866) 0.164 0.05 0.16 [0.06; 0.27] 6.5% 9.5%
ORCADES (981) 0.079 0.06 0.08 [−0.03; 0.19] 5.7% 8.7%
STABILITY (2951) 0.085 0.03 0.09 [0.03; 0.14] 21.0% 16.6%
STANLEY (344) 0.185 0.10 0.18 [−0.02; 0.39] 1.7% 3.4%
STANLEY (300) 0.229 0.09 0.23 [0.05; 0.40] 2.2% 4.3%
VIS (901) 0.142 0.05 0.14 [0.04; 0.25] 6.2% 9.2%
Common effect model 0.09 [0.07; 0.12] 100.0% --
Random effects model 0.11 [0.07; 0.15] -- 100.0%
−0.4 −0.2 0 0.2 0.4
Heterogeneity: I^2 = 46%, τ^2 = 0.0017, p = 0.05
CCL11 (CCL11)−rs1491961
rs1491961
r^2
0.8 0.6 0.4 0.2
$-\log_{10}$(p-value)
0 2 4 6 8 10 12
Recombination rate (cM/Mb)
0 20 40 60 80 100
SACM1L→ ←LZTFL1 ←XCR1 ←CCR1 CCR2→ ←LTF ←LRRC2 ←ALS2CL
←SLC6A20 CCR9→ CCR3→ ←LOC102724297 LRRC2−AS1→ TMIE→
←FYCO1 CCR5→ RTP3→
CXCR6→ CCRL2→ TDGF1→
LOC100132146→
45.8 46 46.2 46.4 46.6
Position on chr3 (Mb)

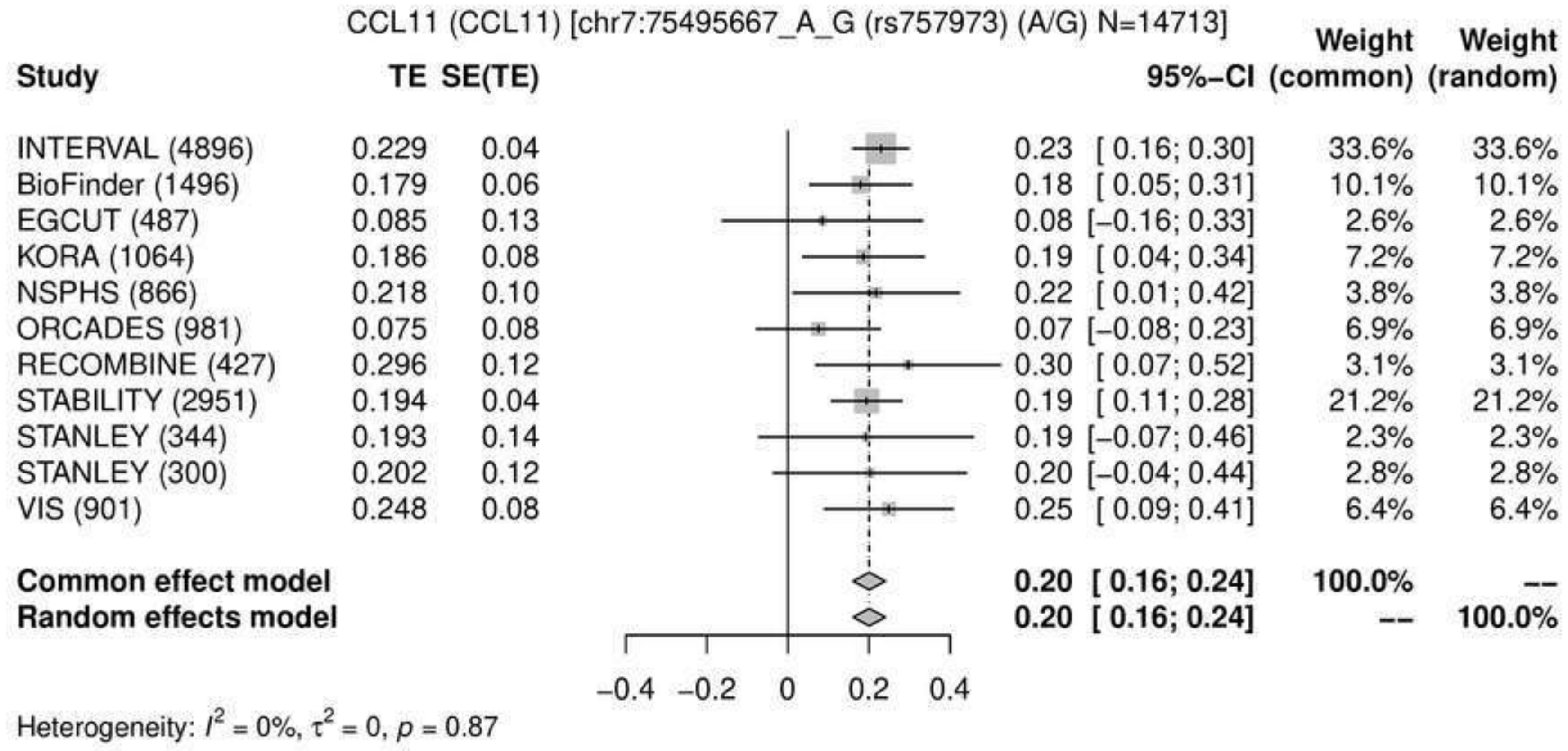
CCL11 (CCL11) [chr7:75495667_A_G (rs757973) (A/G) N=14713]
Study
TE
SE(TE)
95%−CI
Weight (common)
Weight (random)
INTERVAL (4896) 0.229 0.04 0.23 [0.16; 0.30] 33.6% 33.6%
BioFinder (1496) 0.179 0.06 0.18 [0.05; 0.31] 10.1% 10.1%
EGCUT (487) 0.085 0.13 0.08 [−0.16; 0.33] 2.6% 2.6%
KORA (1064) 0.186 0.08 0.19 [0.04; 0.34] 7.2% 7.2%
NSPHS (866) 0.218 0.10 0.22 [0.01; 0.42] 3.8% 3.8%
ORCADES (981) 0.075 0.08 0.07 [−0.08; 0.23] 6.9% 6.9%
RECOMBINE (427) 0.296 0.12 0.30 [0.07; 0.52] 3.1% 3.1%
STABILITY (2951) 0.194 0.04 0.19 [0.11; 0.28] 21.2% 21.2%
STANLEY (344) 0.193 0.14 0.19 [−0.07; 0.46] 2.3% 2.3%
STANLEY (300) 0.202 0.12 0.20 [−0.04; 0.44] 2.8% 2.8%
VIS (901) 0.248 0.08 0.25 [0.09; 0.41] 6.4% 6.4%
Common effect model 0.20 [0.16; 0.24] 100.0% --
Random effects model 0.20 [0.16; 0.24] -- 100.0%
−0.4 −0.2 0 0.2 0.4
Heterogeneity: I^2 = 0%, τ^2 = 0, p = 0.87

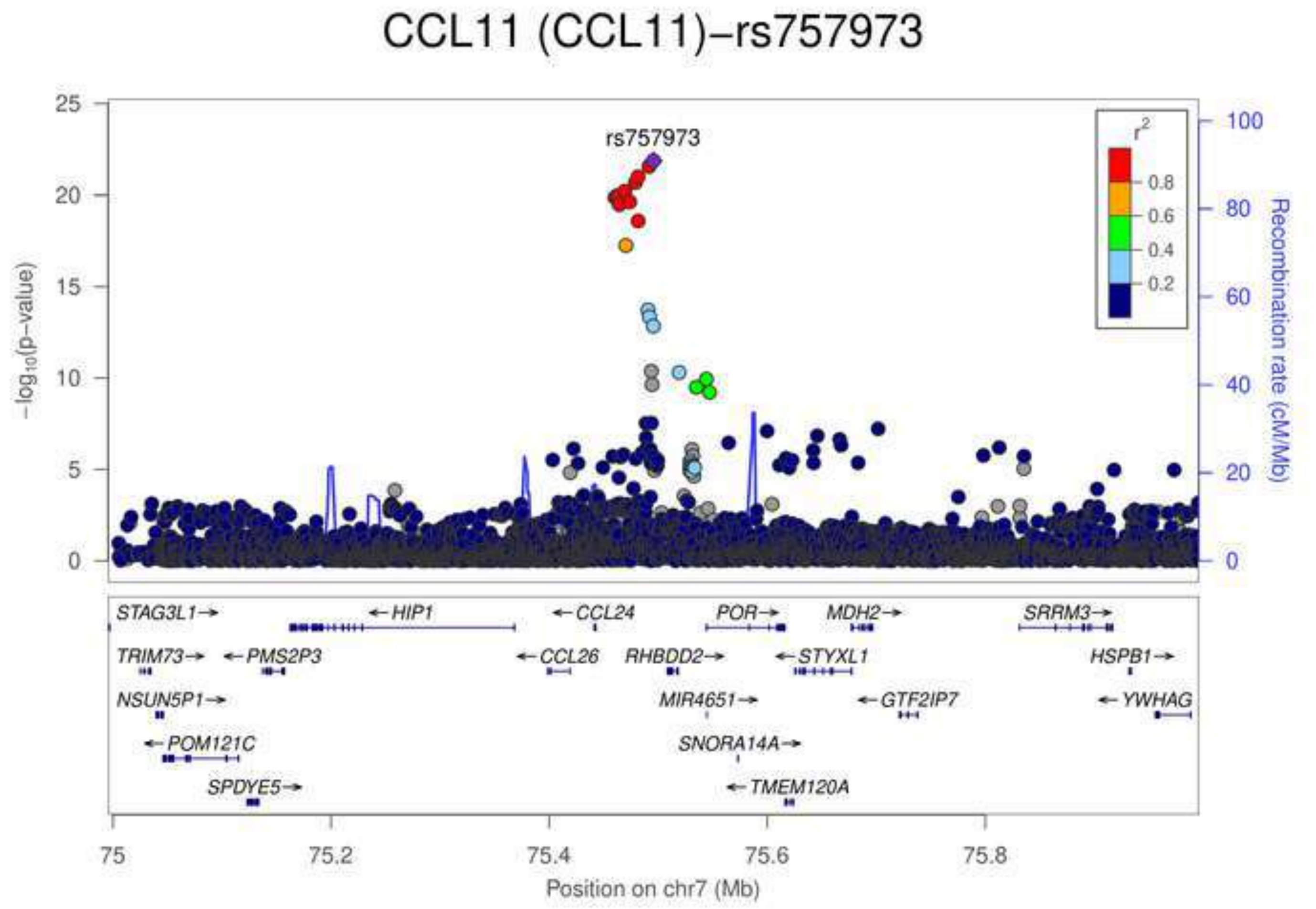
CCL11 (CCL11)−rs757973
rs757973
$-\log_{10}$(p−value)
Recombination rate (cM/Mb)
r^2
0.8
0.6
0.4
0.2
STAG3L1→
←HIP1
←CCL24
POR→
MDH2→
SRRM3→
TRIM73→
←PMS2P3
←CCL26
RHBDD2→
←STYXL1
HSPB1→
NSUN5P1→
MIR4651→
←GTF2IP7
←YWHAG
←POM121C
SNORA14A→
SPDYE5→
←TMEM120A
75
75.2
75.4
75.6
75.8
Position on chr7 (Mb)

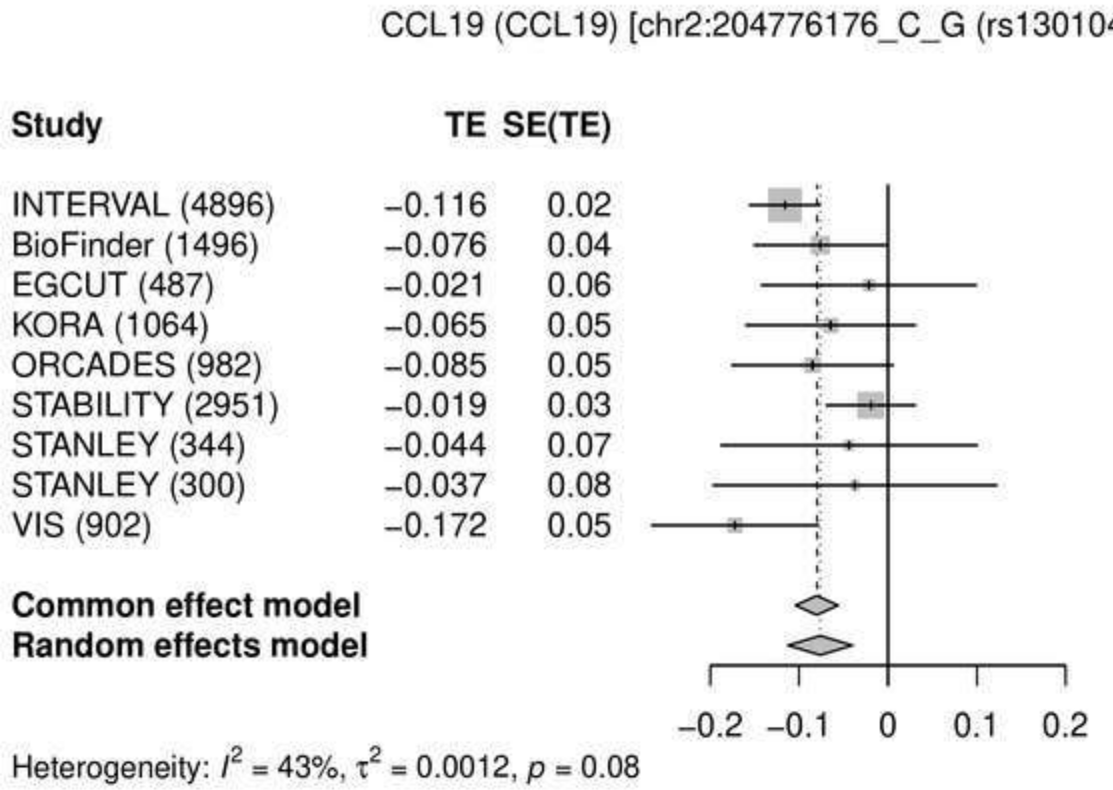
CCL19 (CCL19) [chr2:204776176_C_G (rs13010492) (C/G) N=13422]
Study
TE
SE(TE)
95%-CI
Weight (common)
Weight (random)
INTERVAL (4896) −0.116 0.02 −0.12 [−0.16; −0.08] 37.3% 21.5%
BioFinder (1496) −0.076 0.04 −0.08 [−0.15; −0.00] 10.6% 13.2%
EGCUT (487) −0.021 0.06 −0.02 [−0.14; 0.10] 4.0% 6.9%
KORA (1064) −0.065 0.05 −0.06 [−0.16; 0.03] 6.4% 9.7%
ORCADES (982) −0.085 0.05 −0.08 [−0.18; 0.01] 7.0% 10.4%
STABILITY (2951) −0.019 0.03 −0.02 [−0.07; 0.03] 23.1% 18.7%
STANLEY (344) −0.044 0.07 −0.04 [−0.19; 0.10] 2.8% 5.3%
STANLEY (300) −0.037 0.08 −0.04 [−0.20; 0.12] 2.3% 4.4%
VIS (902) −0.172 0.05 −0.17 [−0.27; −0.08] 6.6% 9.9%
Common effect model −0.08 [−0.10; −0.06] 100.0% --
Random effects model −0.08 [−0.11; −0.04] -- 100.0%
−0.2 −0.1 0 0.1 0.2
Heterogeneity: I^2 = 43%, τ^2 = 0.0012, p = 0.08

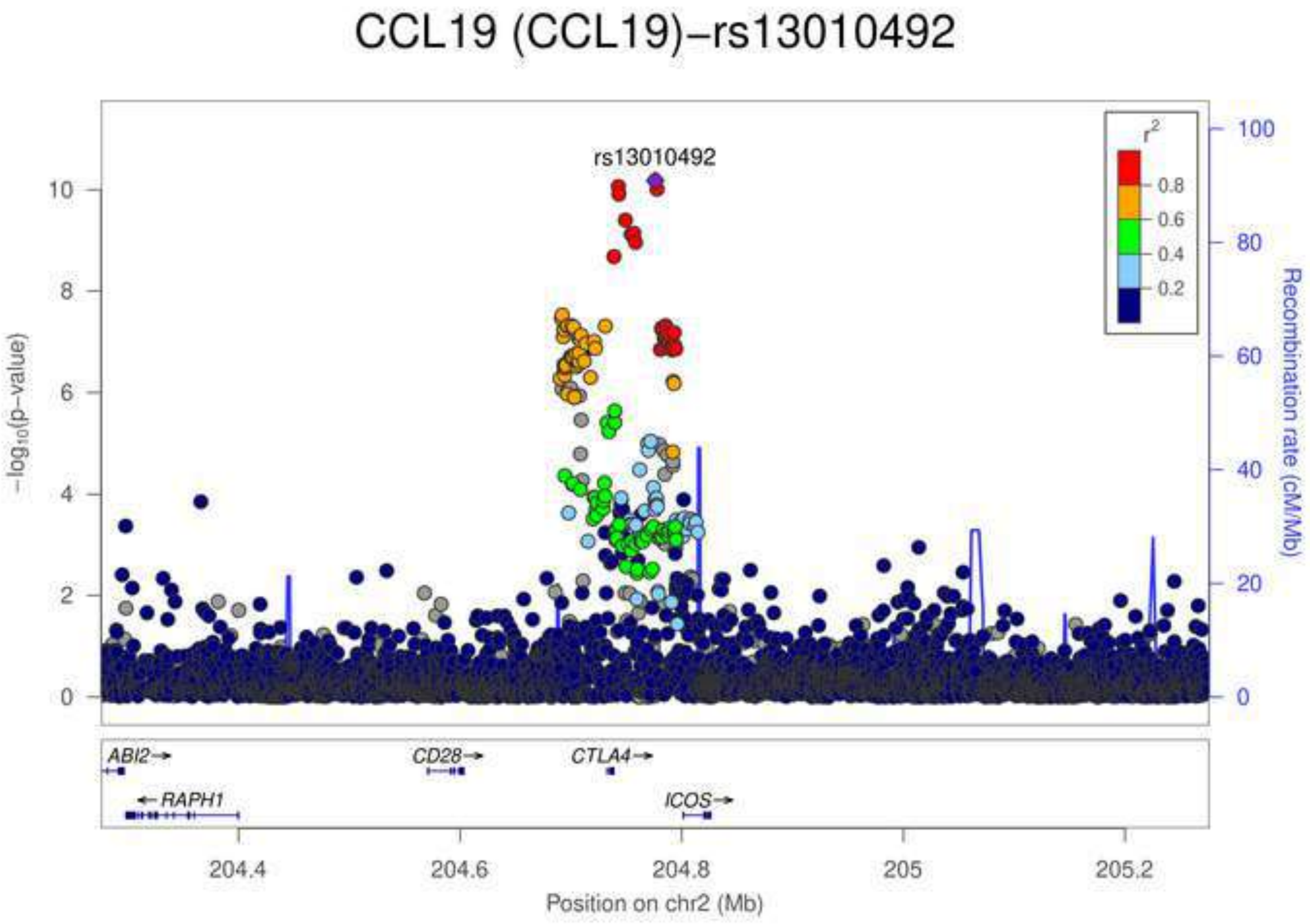
CCL19 (CCL19)−rs13010492
rs13010492
$-\log_{10}$(p−value)
Recombination rate (cM/Mb)
r^2
0.8
0.6
0.4
0.2
ABI2→
CD28→
CTLA4→
←RAPH1
ICOS→
204.4
204.6
204.8
205
205.2
Position on chr2 (Mb)

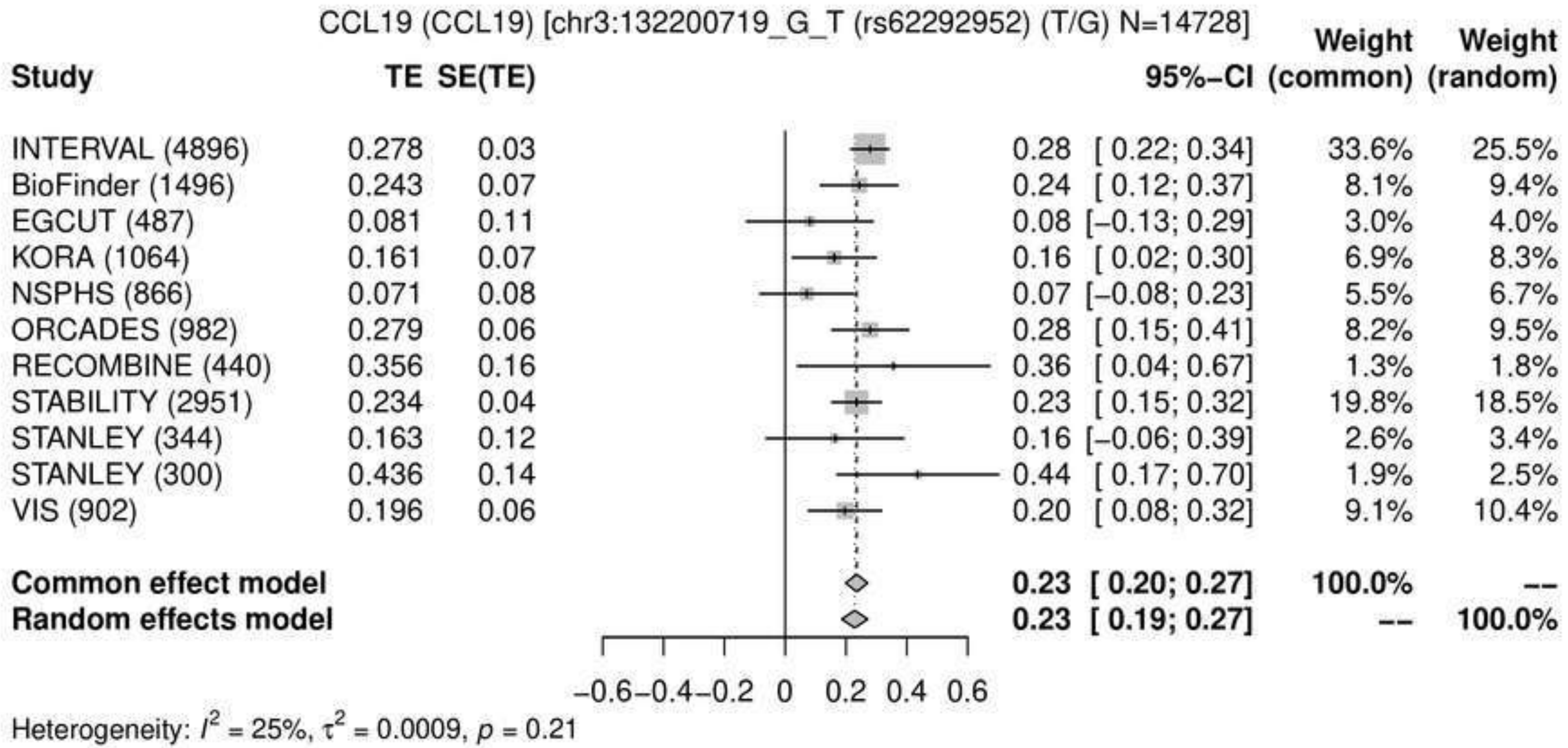
CCL19 (CCL19) [chr3:132200719_G_T (rs62292952) (T/G) N=14728]
Study
TE SE(TE)
95%-CI
Weight (common)
Weight (random)
INTERVAL (4896) 0.278 0.03 0.28 [0.22; 0.34] 33.6% 25.5%
BioFinder (1496) 0.243 0.07 0.24 [0.12; 0.37] 8.1% 9.4%
EGCUT (487) 0.081 0.11 0.08 [−0.13; 0.29] 3.0% 4.0%
KORA (1064) 0.161 0.07 0.16 [0.02; 0.30] 6.9% 8.3%
NSPHS (866) 0.071 0.08 0.07 [−0.08; 0.23] 5.5% 6.7%
ORCADES (982) 0.279 0.06 0.28 [0.15; 0.41] 8.2% 9.5%
RECOMBINE (440) 0.356 0.16 0.36 [0.04; 0.67] 1.3% 1.8%
STABILITY (2951) 0.234 0.04 0.23 [0.15; 0.32] 19.8% 18.5%
STANLEY (344) 0.163 0.12 0.16 [−0.06; 0.39] 2.6% 3.4%
STANLEY (300) 0.436 0.14 0.44 [0.17; 0.70] 1.9% 2.5%
VIS (902) 0.196 0.06 0.20 [0.08; 0.32] 9.1% 10.4%
Common effect model 0.23 [0.20; 0.27] 100.0% --
Random effects model 0.23 [0.19; 0.27] -- 100.0%
−0.6 −0.4 −0.2 0 0.2 0.4 0.6
Heterogeneity: I² = 25%, τ² = 0.0009, p = 0.21

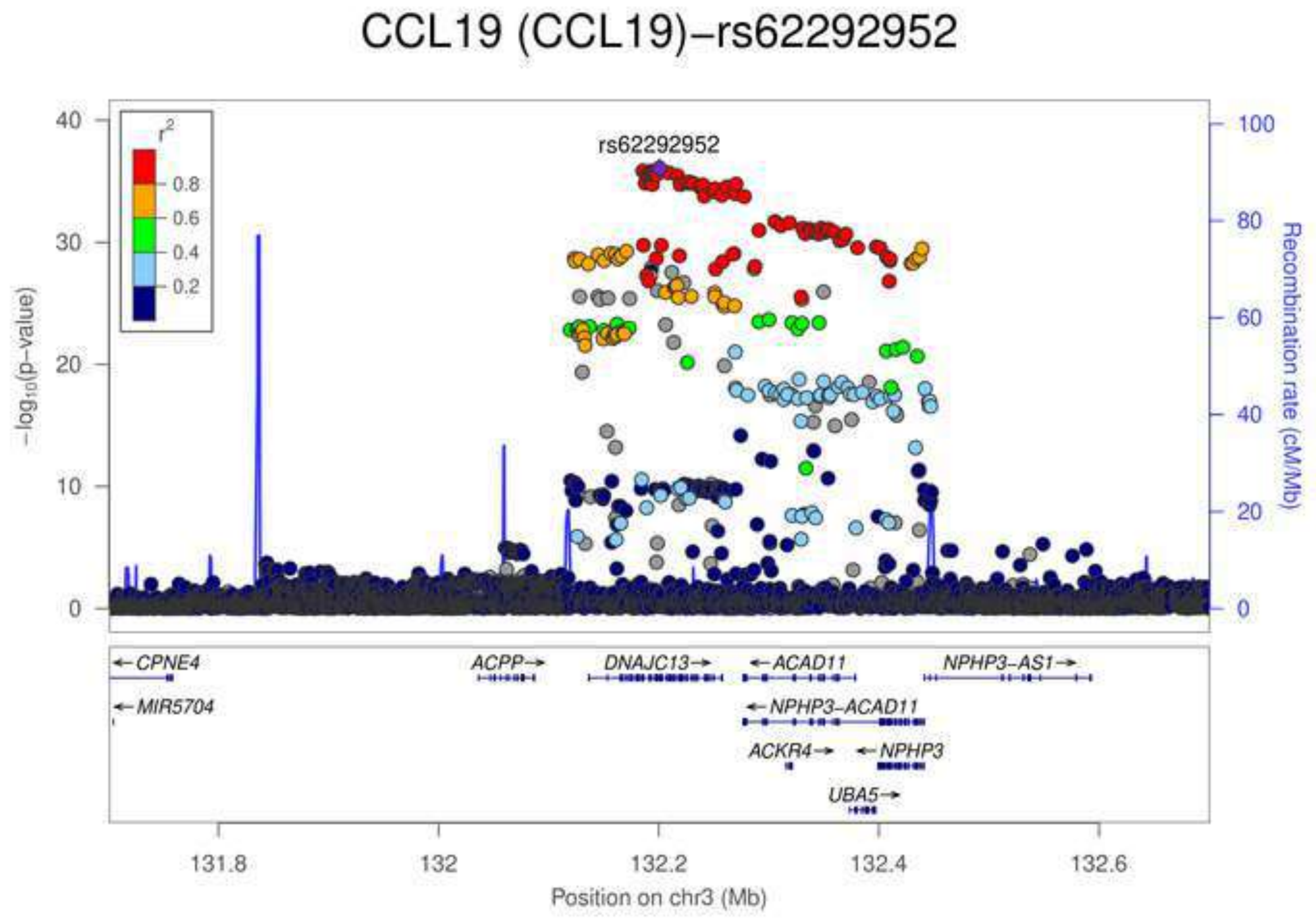
CCL19 (CCL19)−rs62292952
rs62292952
r²
0.8
0.6
0.4
0.2
−log10(p−value)
Recombination rate (cM/Mb)
CPNE4
MIR5704
ACPP
DNAJC13
ACAD11
NPHP3−ACAD11
ACKR4
NPHP3
UBA5
NPHP3−AS1
131.8
132
132.2
132.4
132.6
Position on chr3 (Mb)

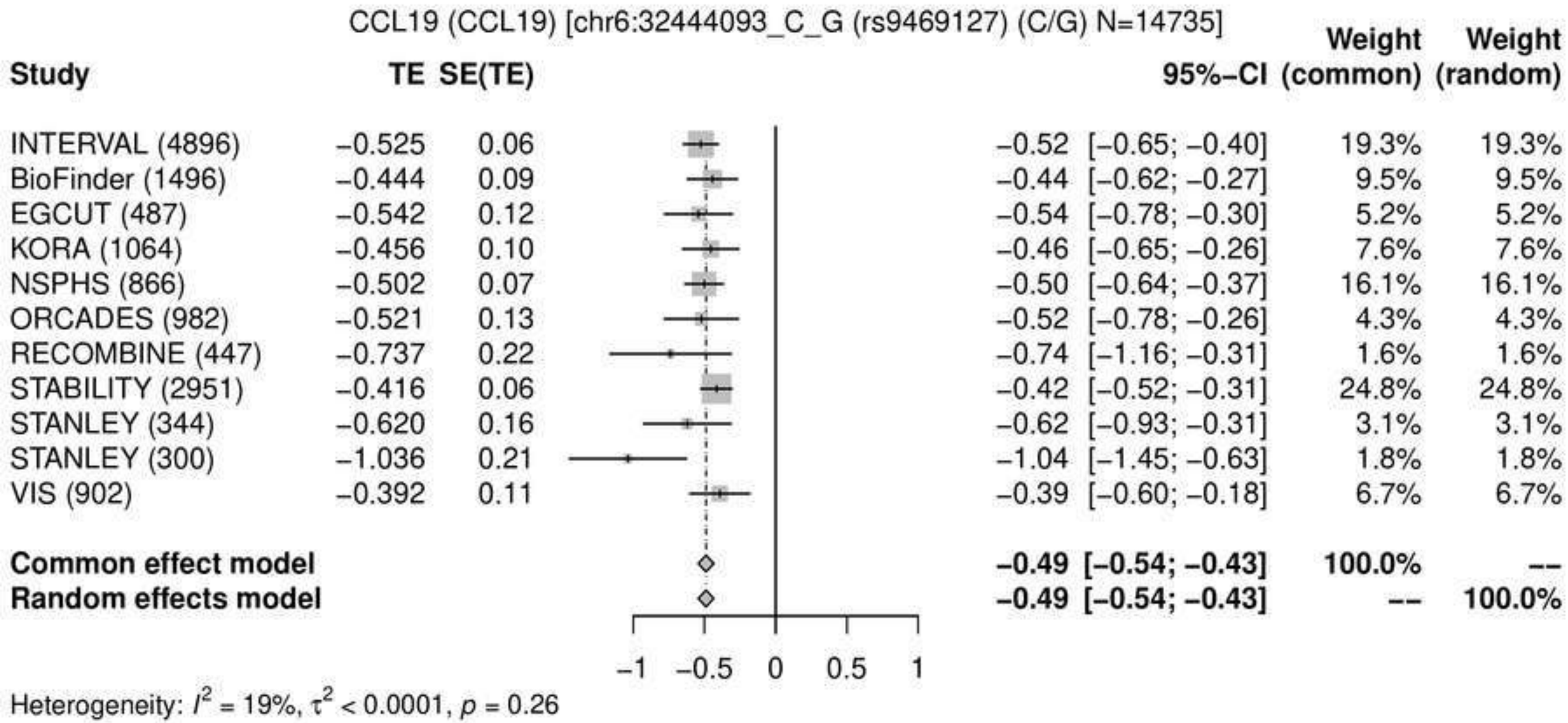
CCL19 (CCL19) [chr6:32444093_C_G (rs9469127) (C/G) N=14735]
Study
TE
SE(TE)
95%−CI
Weight (common)
Weight (random)
INTERVAL (4896) −0.525 0.06 −0.52 [−0.65; −0.40] 19.3% 19.3%
BioFinder (1496) −0.444 0.09 −0.44 [−0.62; −0.27] 9.5% 9.5%
EGCUT (487) −0.542 0.12 −0.54 [−0.78; −0.30] 5.2% 5.2%
KORA (1064) −0.456 0.10 −0.46 [−0.65; −0.26] 7.6% 7.6%
NSPHS (866) −0.502 0.07 −0.50 [−0.64; −0.37] 16.1% 16.1%
ORCADES (982) −0.521 0.13 −0.52 [−0.78; −0.26] 4.3% 4.3%
RECOMBINE (447) −0.737 0.22 −0.74 [−1.16; −0.31] 1.6% 1.6%
STABILITY (2951) −0.416 0.06 −0.42 [−0.52; −0.31] 24.8% 24.8%
STANLEY (344) −0.620 0.16 −0.62 [−0.93; −0.31] 3.1% 3.1%
STANLEY (300) −1.036 0.21 −1.04 [−1.45; −0.63] 1.8% 1.8%
VIS (902) −0.392 0.11 −0.39 [−0.60; −0.18] 6.7% 6.7%
Common effect model −0.49 [−0.54; −0.43] 100.0% --
Random effects model −0.49 [−0.54; −0.43] -- 100.0%
−1 −0.5 0 0.5 1
Heterogeneity: I^2 = 19%, τ^2 < 0.0001, p = 0.26

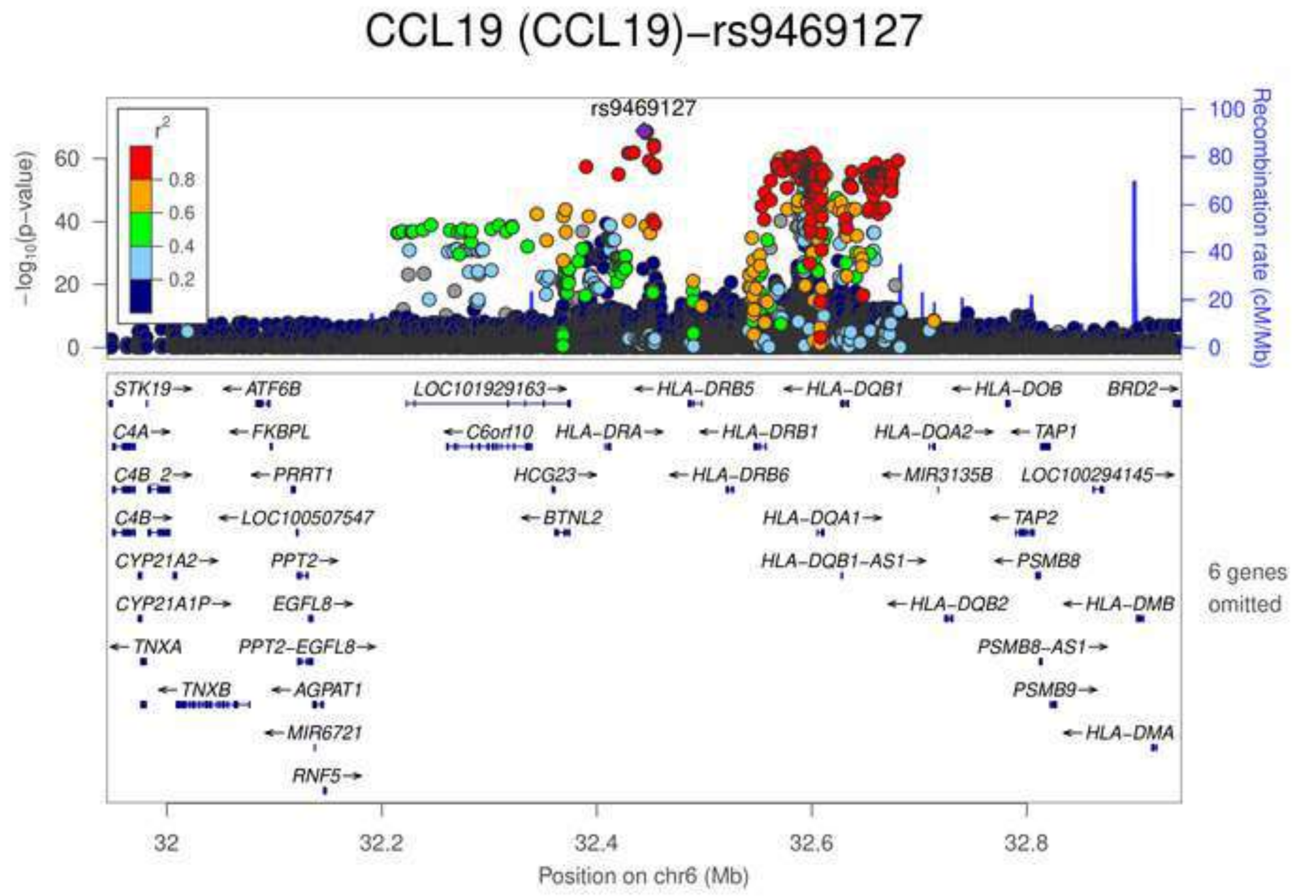
CCL19 (CCL19)−rs9469127
rs9469127
r^2
0.8
0.6
0.4
0.2
$-\log_{10}(p\text{-value})$
Recombination rate (cM/Mb)
STK19 ATF6B LOC101929163 HLA-DRB5 HLA-DQB1 HLA-DOB BRD2
C4A FKBPL C6orf10 HLA-DRA HLA-DRB1 HLA-DQA2 TAP1
C4B_2 PRRT1 HCG23 HLA-DRB6 MIR3135B LOC100294145
C4B LOC100507547 BTNL2 HLA-DQA1 TAP2
CYP21A2 PPT2 HLA-DQB1-AS1 PSMB8
CYP21A1P EGFL8 HLA-DQB2 HLA-DMB
TNXA PPT2-EGFL8 PSMB8-AS1
TNXB AGPAT1 PSMB9
MIR6721 HLA-DMA
RNF5
6 genes omitted
32 32.2 32.4 32.6 32.8
Position on chr6 (Mb)

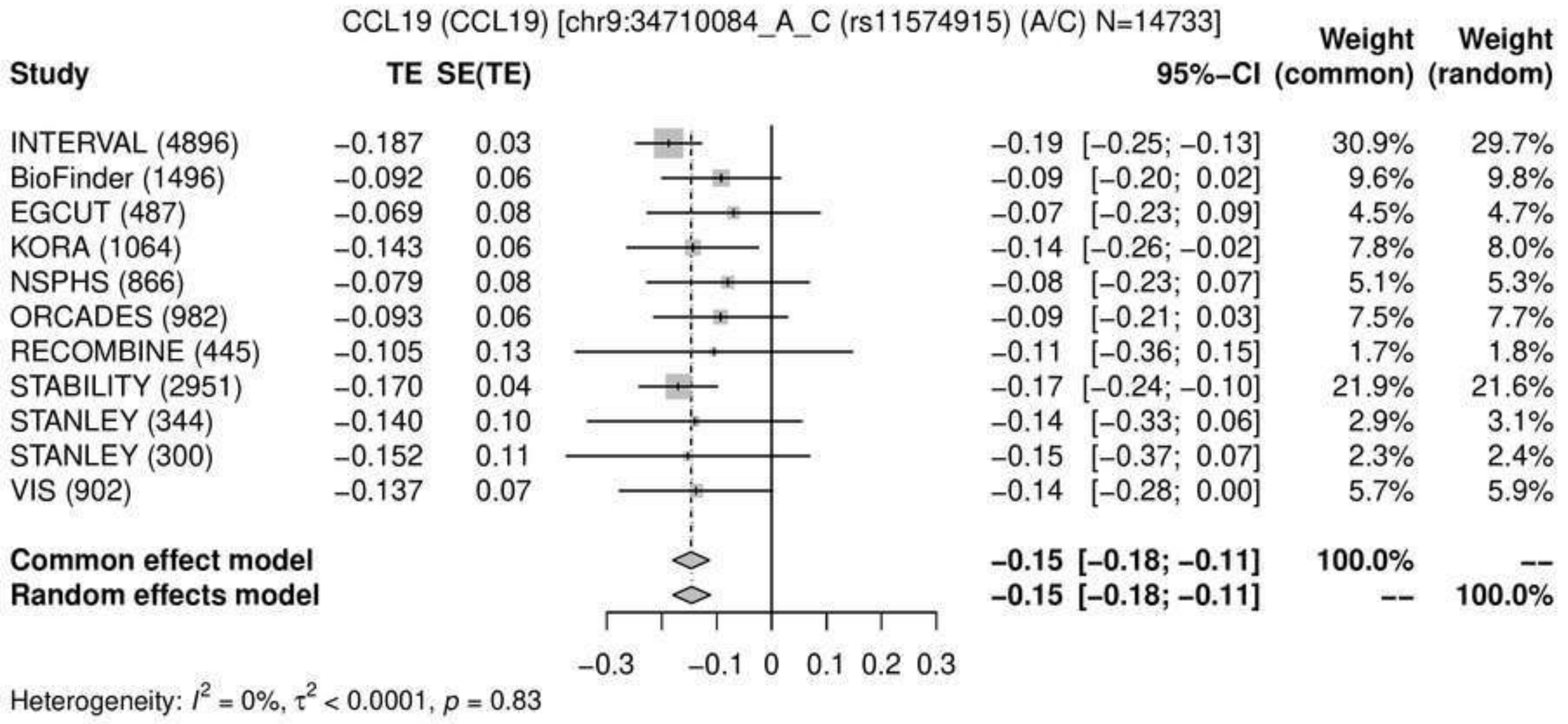

CCL19 (CCL19) [chr9:34710084_A_C (rs11574915) (A/C) N=14733]
Study TE SE(TE) 95%-CI Weight (common) Weight (random)
INTERVAL (4896) −0.187 0.03 −0.19 [−0.25; −0.13] 30.9% 29.7%
BioFinder (1496) −0.092 0.06 −0.09 [−0.20; 0.02] 9.6% 9.8%
EGCUT (487) −0.069 0.08 −0.07 [−0.23; 0.09] 4.5% 4.7%
KORA (1064) −0.143 0.06 −0.14 [−0.26; −0.02] 7.8% 8.0%
NSPHS (866) −0.079 0.08 −0.08 [−0.23; 0.07] 5.1% 5.3%
ORCADES (982) −0.093 0.06 −0.09 [−0.21; 0.03] 7.5% 7.7%
RECOMBINE (445) −0.105 0.13 −0.11 [−0.36; 0.15] 1.7% 1.8%
STABILITY (2951) −0.170 0.04 −0.17 [−0.24; −0.10] 21.9% 21.6%
STANLEY (344) −0.140 0.10 −0.14 [−0.33; 0.06] 2.9% 3.1%
STANLEY (300) −0.152 0.11 −0.15 [−0.37; 0.07] 2.3% 2.4%
VIS (902) −0.137 0.07 −0.14 [−0.28; 0.00] 5.7% 5.9%
Common effect model −0.15 [−0.18; −0.11] 100.0% --
Random effects model −0.15 [−0.18; −0.11] -- 100.0%
−0.3 −0.1 0 0.1 0.2 0.3
Heterogeneity: $I^2 = 0\%$, $\tau^2 < 0.0001$, $p = 0.83$

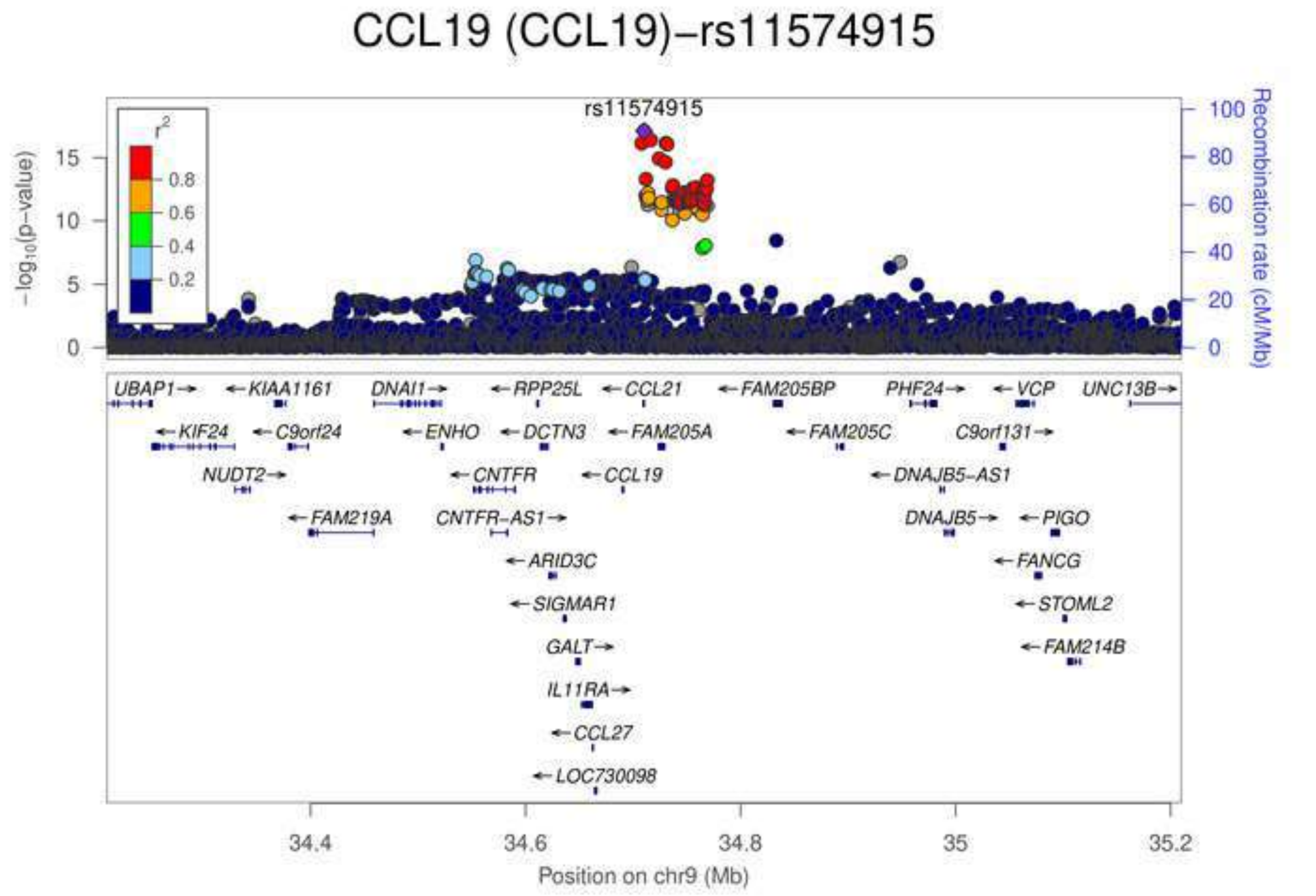

CCL19 (CCL19)−rs11574915
rs11574915
r^2
0.8
0.6
0.4
0.2
$-\log_{10}(p\text{-value})$
Recombination rate (cM/Mb)
UBAP1 KIAA1161 DNAI1 RPP25L CCL21 FAM205BP PHF24 VCP UNC13B
KIF24 C9orf24 ENHO DCTN3 FAM205A FAM205C C9orf131
NUDT2 CNTFR CCL19 DNAJB5-AS1
FAM219A CNTFR-AS1 DNAJB5 PIGO
ARID3C FANCG
SIGMAR1 STOML2
GALT FAM214B
IL11RA
CCL27
LOC730098
34.4 34.6 34.8 35 35.2
Position on chr9 (Mb)

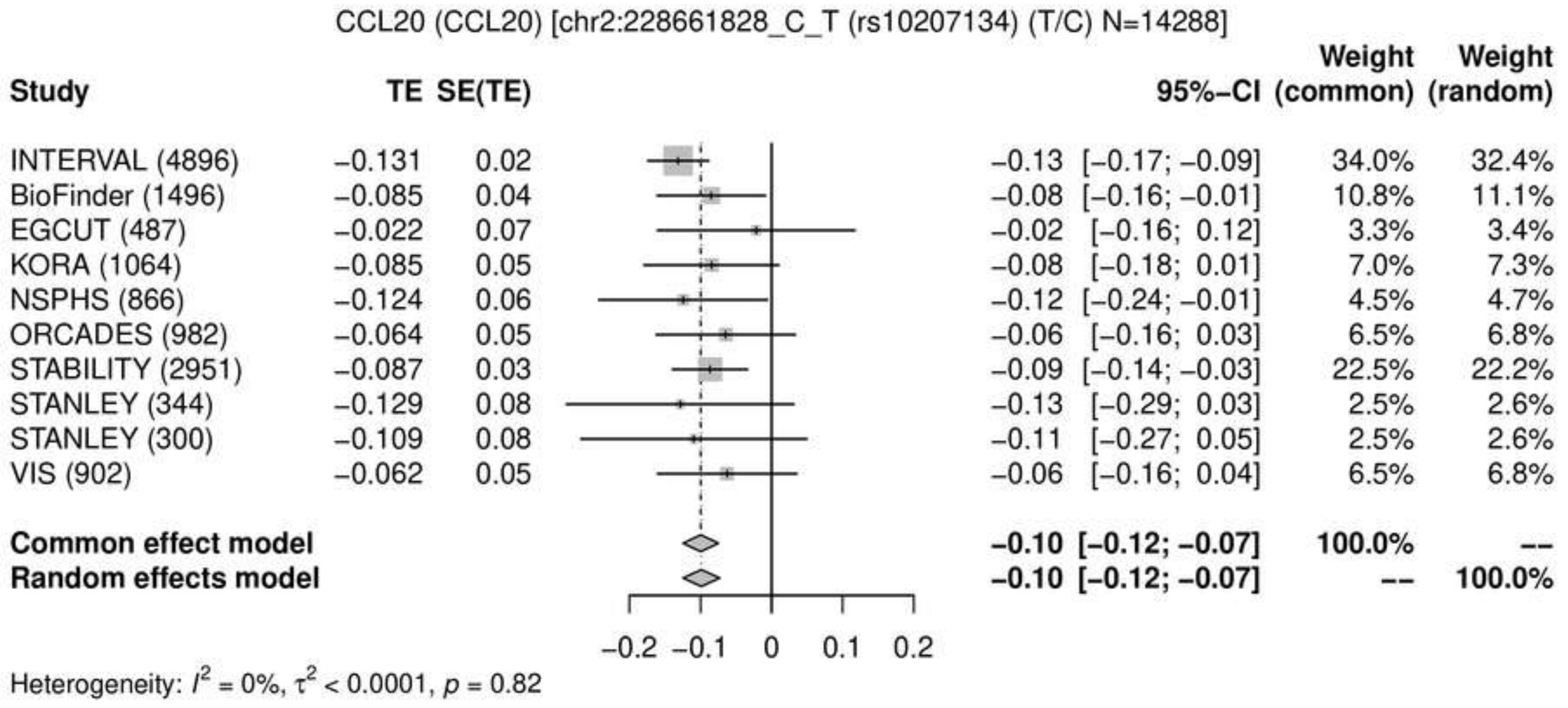
CCL20 (CCL20) [chr2:228661828_C_T (rs10207134) (T/C) N=14288]
Study TE SE(TE) 95%-CI Weight (common) Weight (random)
INTERVAL (4896) −0.131 0.02 −0.13 [−0.17; −0.09] 34.0% 32.4%
BioFinder (1496) −0.085 0.04 −0.08 [−0.16; −0.01] 10.8% 11.1%
EGCUT (487) −0.022 0.07 −0.02 [−0.16; 0.12] 3.3% 3.4%
KORA (1064) −0.085 0.05 −0.08 [−0.18; 0.01] 7.0% 7.3%
NSPHS (866) −0.124 0.06 −0.12 [−0.24; −0.01] 4.5% 4.7%
ORCADES (982) −0.064 0.05 −0.06 [−0.16; 0.03] 6.5% 6.8%
STABILITY (2951) −0.087 0.03 −0.09 [−0.14; −0.03] 22.5% 22.2%
STANLEY (344) −0.129 0.08 −0.13 [−0.29; 0.03] 2.5% 2.6%
STANLEY (300) −0.109 0.08 −0.11 [−0.27; 0.05] 2.5% 2.6%
VIS (902) −0.062 0.05 −0.06 [−0.16; 0.04] 6.5% 6.8%
Common effect model −0.10 [−0.12; −0.07] 100.0% --
Random effects model −0.10 [−0.12; −0.07] -- 100.0%
−0.2 −0.1 0 0.1 0.2
Heterogeneity: I^2 = 0%, τ^2 < 0.0001, p = 0.82

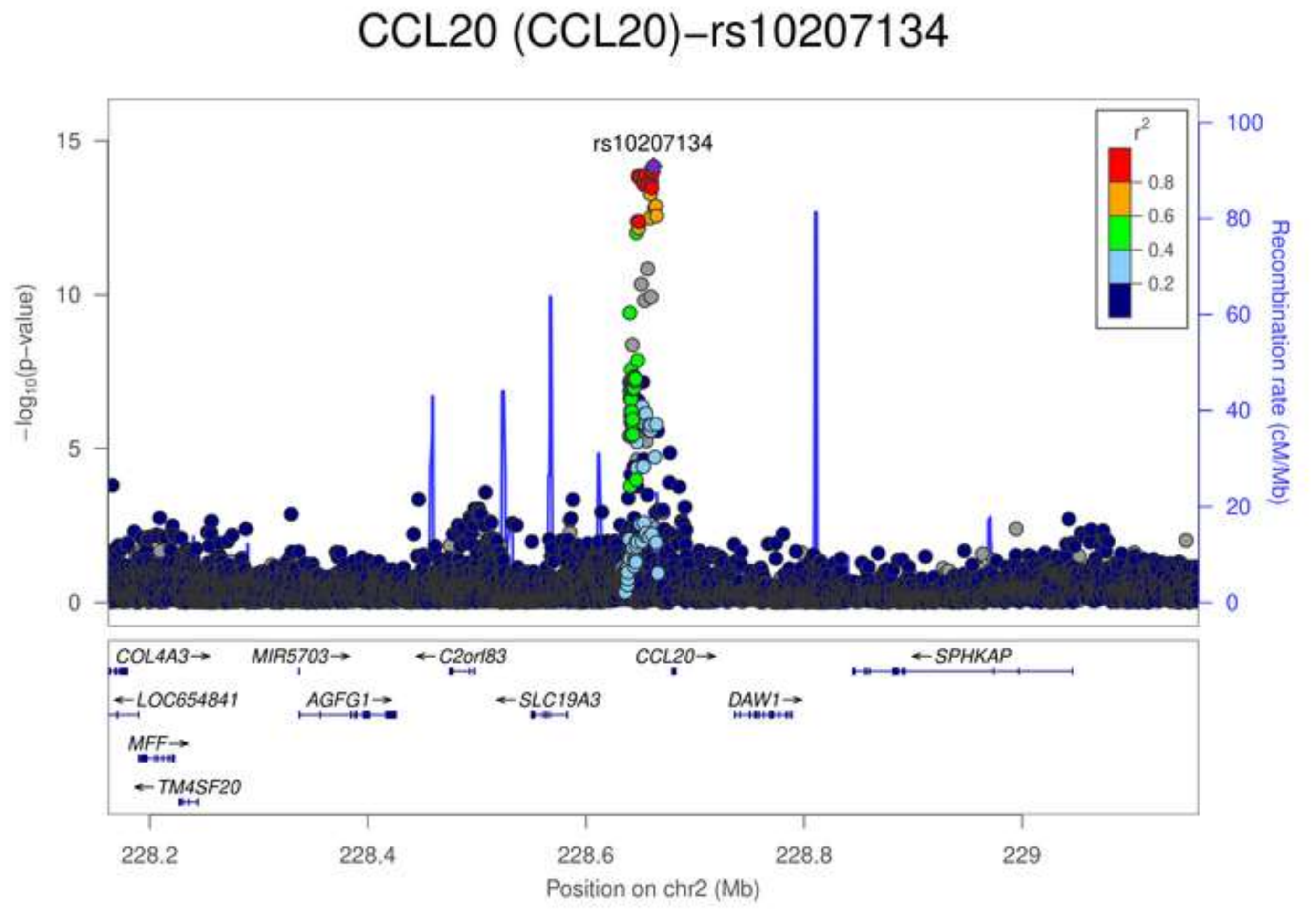
CCL20 (CCL20)−rs10207134
rs10207134
$-\log_{10}$(p-value)
Recombination rate (cM/Mb)
r^2
0.8
0.6
0.4
0.2
COL4A3→
MIR5703→
←C2orf83
CCL20→
←SPHKAP
←LOC654841
AGFG1→
←SLC19A3
DAW1→
MFF→
←TM4SF20
228.2 228.4 228.6 228.8 229
Position on chr2 (Mb)

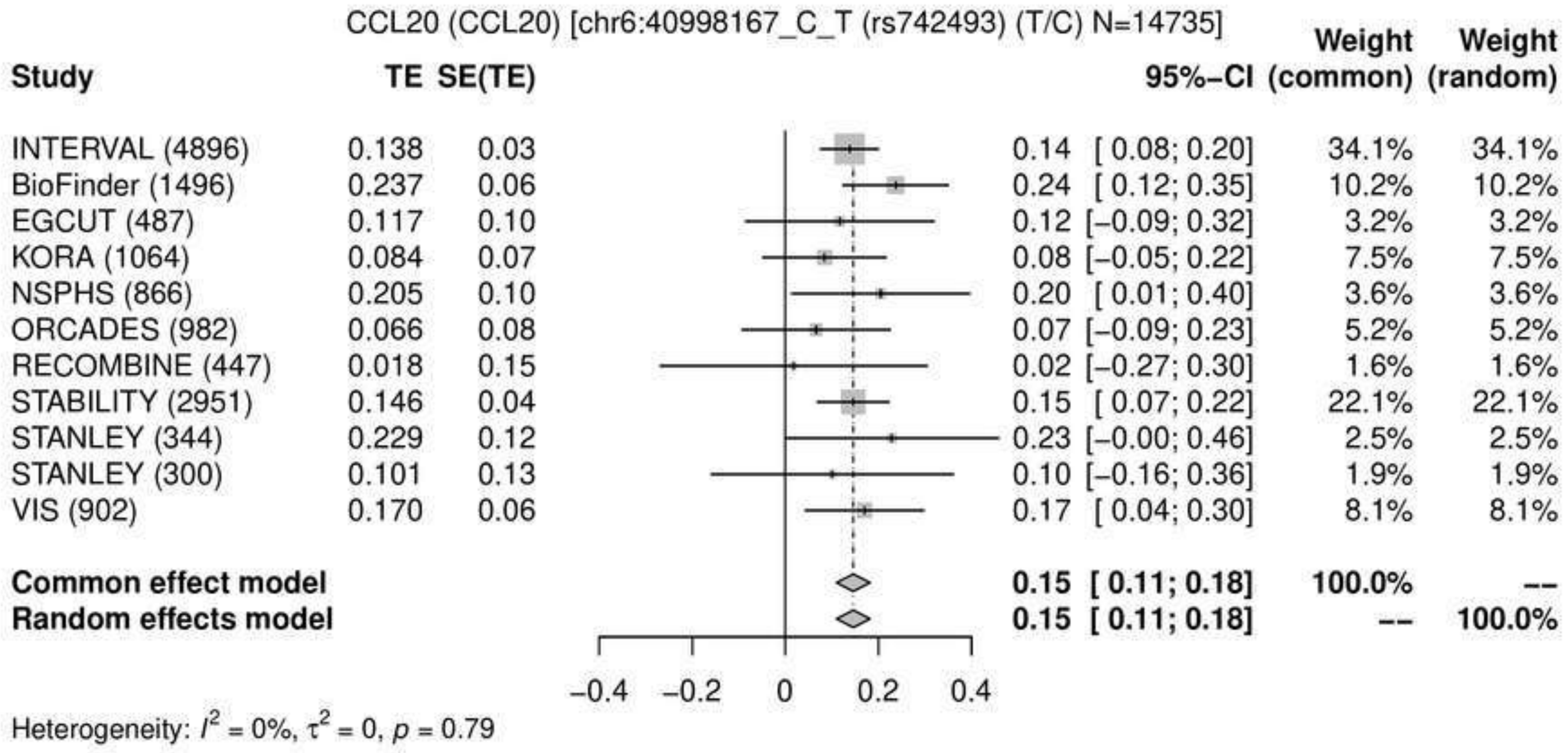
CCL20 (CCL20) [chr6:40998167_C_T (rs742493) (T/C) N=14735]
Study TE SE(TE) 95%−CI Weight (common) Weight (random)
INTERVAL (4896) 0.138 0.03 0.14 [0.08; 0.20] 34.1% 34.1%
BioFinder (1496) 0.237 0.06 0.24 [0.12; 0.35] 10.2% 10.2%
EGCUT (487) 0.117 0.10 0.12 [−0.09; 0.32] 3.2% 3.2%
KORA (1064) 0.084 0.07 0.08 [−0.05; 0.22] 7.5% 7.5%
NSPHS (866) 0.205 0.10 0.20 [0.01; 0.40] 3.6% 3.6%
ORCADES (982) 0.066 0.08 0.07 [−0.09; 0.23] 5.2% 5.2%
RECOMBINE (447) 0.018 0.15 0.02 [−0.27; 0.30] 1.6% 1.6%
STABILITY (2951) 0.146 0.04 0.15 [0.07; 0.22] 22.1% 22.1%
STANLEY (344) 0.229 0.12 0.23 [−0.00; 0.46] 2.5% 2.5%
STANLEY (300) 0.101 0.13 0.10 [−0.16; 0.36] 1.9% 1.9%
VIS (902) 0.170 0.06 0.17 [0.04; 0.30] 8.1% 8.1%
Common effect model 0.15 [0.11; 0.18] 100.0% --
Random effects model 0.15 [0.11; 0.18] -- 100.0%
−0.4 −0.2 0 0.2 0.4
Heterogeneity: I^2 = 0%, τ^2 = 0, p = 0.79

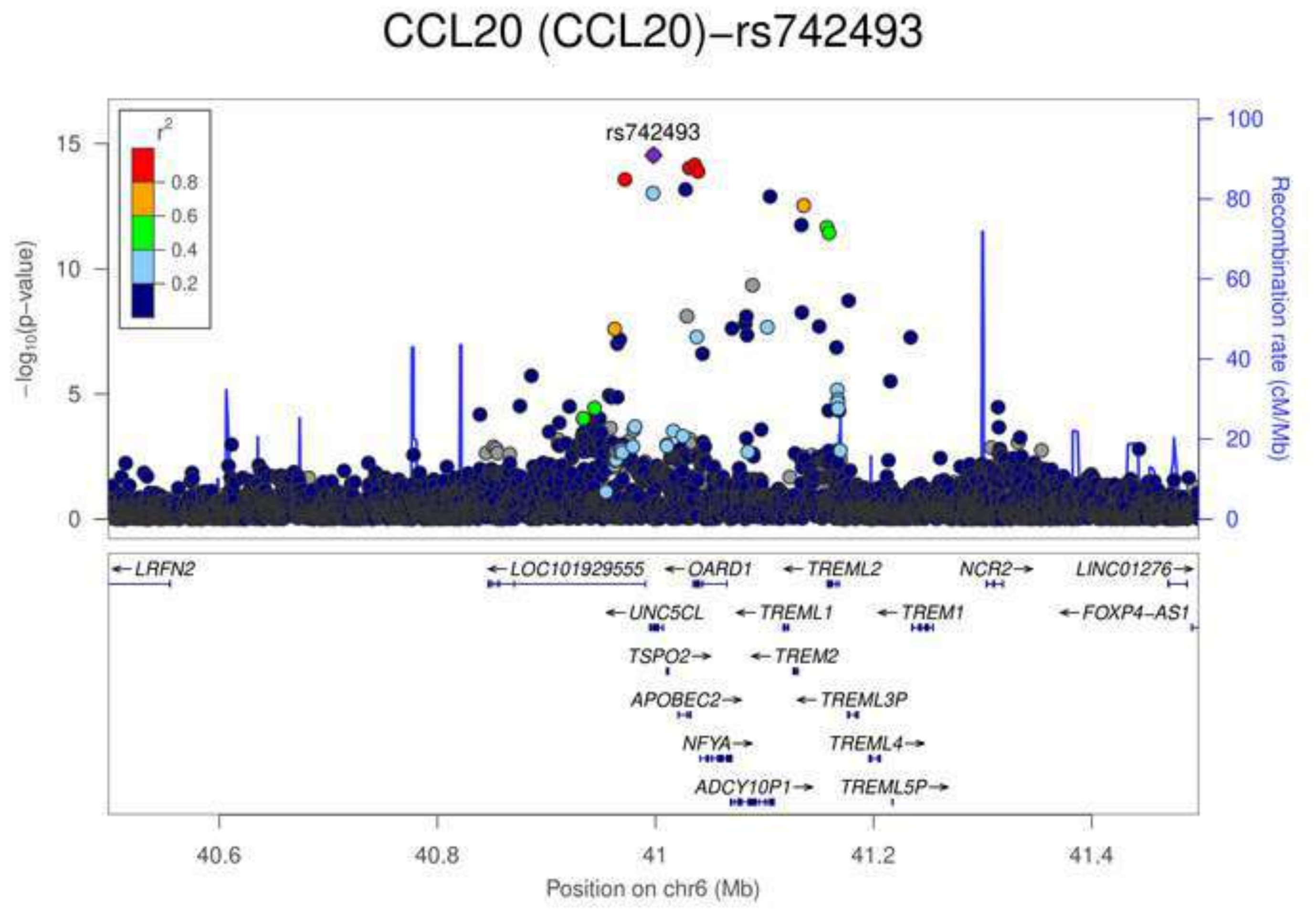
CCL20 (CCL20)−rs742493
rs742493
r^2
0.8
0.6
0.4
0.2
$-\log_{10}$(p-value)
Recombination rate (cM/Mb)
LRFN2
LOC101929555
OARD1
TREML2
NCR2
LINC01276
UNC5CL
TREML1
TREM1
FOXP4-AS1
TSPO2
TREM2
APOBEC2
TREML3P
NFYA
TREML4
ADCY10P1
TREML5P
40.6
40.8
41
41.2
41.4
Position on chr6 (Mb)

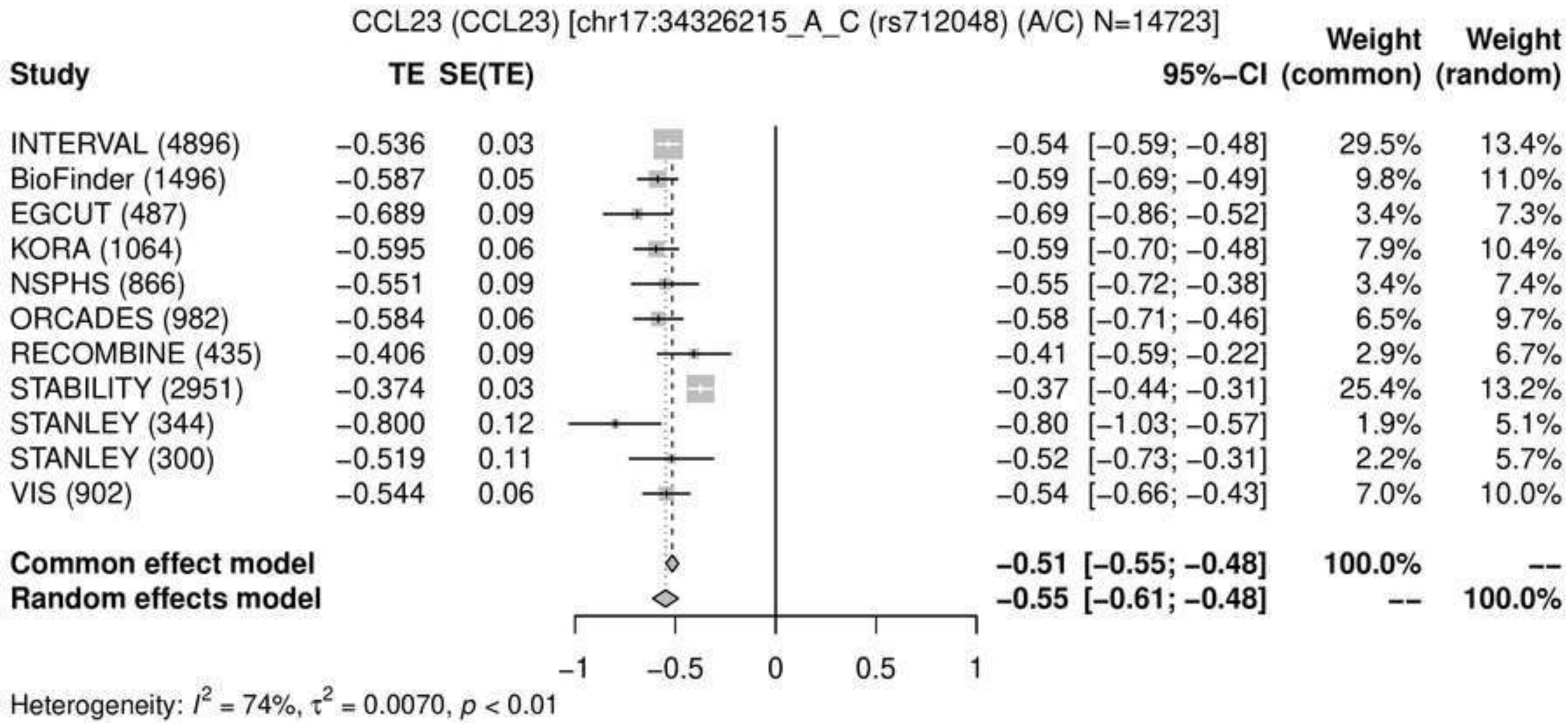
CCL23 (CCL23) [chr17:34326215_A_C (rs712048) (A/C) N=14723]
Study
TE
SE(TE)
95%−CI
Weight (common)
Weight (random)
INTERVAL (4896) −0.536 0.03 −0.54 [−0.59; −0.48] 29.5% 13.4%
BioFinder (1496) −0.587 0.05 −0.59 [−0.69; −0.49] 9.8% 11.0%
EGCUT (487) −0.689 0.09 −0.69 [−0.86; −0.52] 3.4% 7.3%
KORA (1064) −0.595 0.06 −0.59 [−0.70; −0.48] 7.9% 10.4%
NSPHS (866) −0.551 0.09 −0.55 [−0.72; −0.38] 3.4% 7.4%
ORCADES (982) −0.584 0.06 −0.58 [−0.71; −0.46] 6.5% 9.7%
RECOMBINE (435) −0.406 0.09 −0.41 [−0.59; −0.22] 2.9% 6.7%
STABILITY (2951) −0.374 0.03 −0.37 [−0.44; −0.31] 25.4% 13.2%
STANLEY (344) −0.800 0.12 −0.80 [−1.03; −0.57] 1.9% 5.1%
STANLEY (300) −0.519 0.11 −0.52 [−0.73; −0.31] 2.2% 5.7%
VIS (902) −0.544 0.06 −0.54 [−0.66; −0.43] 7.0% 10.0%
Common effect model −0.51 [−0.55; −0.48] 100.0% --
Random effects model −0.55 [−0.61; −0.48] -- 100.0%
−1 −0.5 0 0.5 1
Heterogeneity: I^2 = 74%, τ^2 = 0.0070, p < 0.01

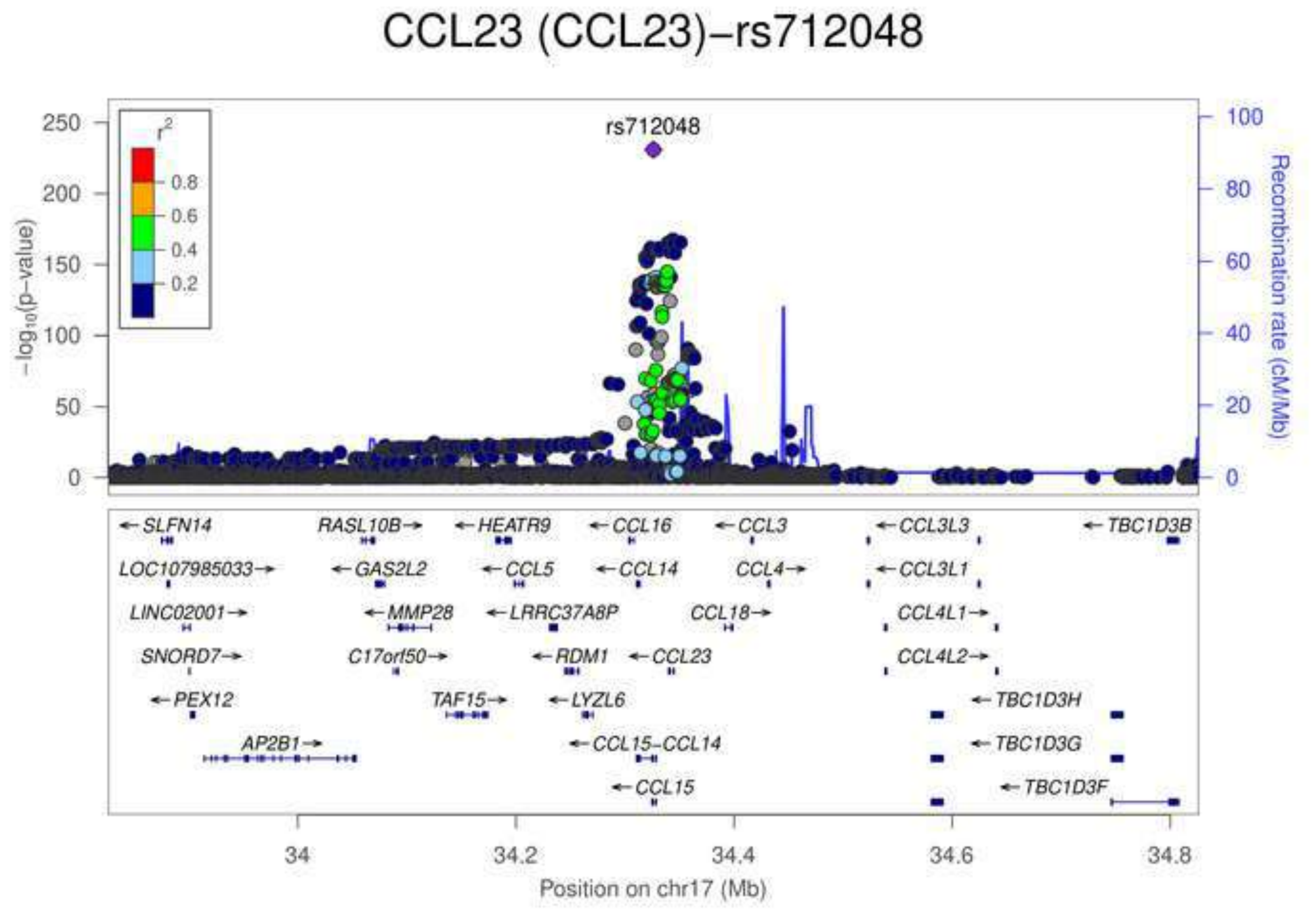
CCL23 (CCL23)−rs712048
rs712048
r^2
0.8
0.6
0.4
0.2
250
200
150
100
50
0
$-\log_{10}$(p−value)
100
80
60
40
20
0
Recombination rate (cM/Mb)
SLFN14
RASL10B
HEATR9
CCL16
CCL3
CCL3L3
TBC1D3B
LOC107985033
GAS2L2
CCL5
CCL14
CCL4
CCL3L1
LINC02001
MMP28
LRRC37A8P
CCL18
CCL4L1
SNORD7
C17orf50
RDM1
CCL23
CCL4L2
PEX12
TAF15
LYZL6
TBC1D3H
AP2B1
CCL15−CCL14
TBC1D3G
CCL15
TBC1D3F
34
34.2
34.4
34.6
34.8
Position on chr17 (Mb)

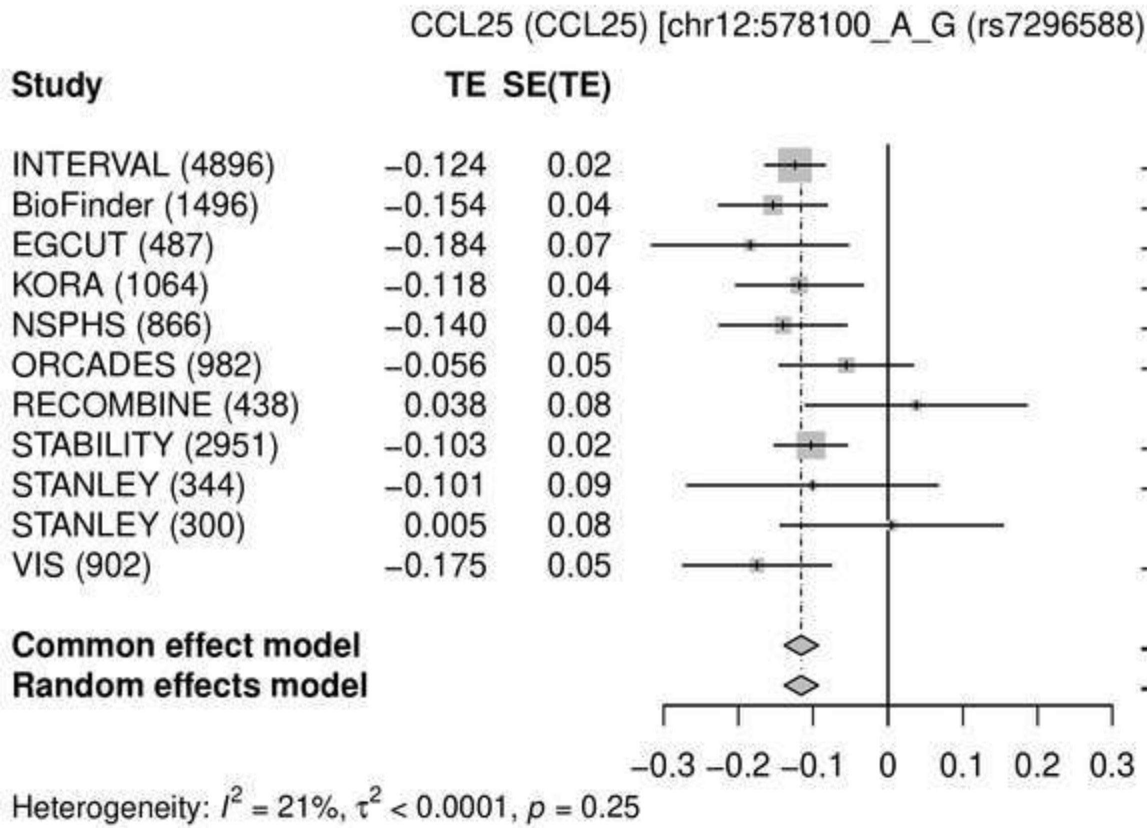
CCL25 (CCL25) [chr12:578100_A_G (rs7296588) (A/G) N=14726]
Study
TE SE(TE)
95%−CI
Weight (common)
Weight (random)
INTERVAL (4896) −0.124 0.02 −0.12 [−0.16; −0.08] 32.5% 32.4%
BioFinder (1496) −0.154 0.04 −0.15 [−0.23; −0.08] 9.9% 9.9%
EGCUT (487) −0.184 0.07 −0.18 [−0.32; −0.05] 3.0% 3.0%
KORA (1064) −0.118 0.04 −0.12 [−0.20; −0.03] 7.2% 7.2%
NSPHS (866) −0.140 0.04 −0.14 [−0.23; −0.06] 7.2% 7.2%
ORCADES (982) −0.056 0.05 −0.06 [−0.15; 0.03] 6.5% 6.5%
RECOMBINE (438) 0.038 0.08 0.04 [−0.11; 0.19] 2.4% 2.4%
STABILITY (2951) −0.103 0.02 −0.10 [−0.15; −0.05] 21.9% 21.9%
STANLEY (344) −0.101 0.09 −0.10 [−0.27; 0.07] 1.8% 1.9%
STANLEY (300) 0.005 0.08 0.01 [−0.14; 0.15] 2.3% 2.4%
VIS (902) −0.175 0.05 −0.17 [−0.27; −0.08] 5.3% 5.3%
Common effect model −0.12 [−0.14; −0.09] 100.0% --
Random effects model −0.12 [−0.14; −0.09] -- 100.0%
−0.3 −0.2 −0.1 0 0.1 0.2 0.3
Heterogeneity: I^2 = 21%, τ^2 < 0.0001, p = 0.25

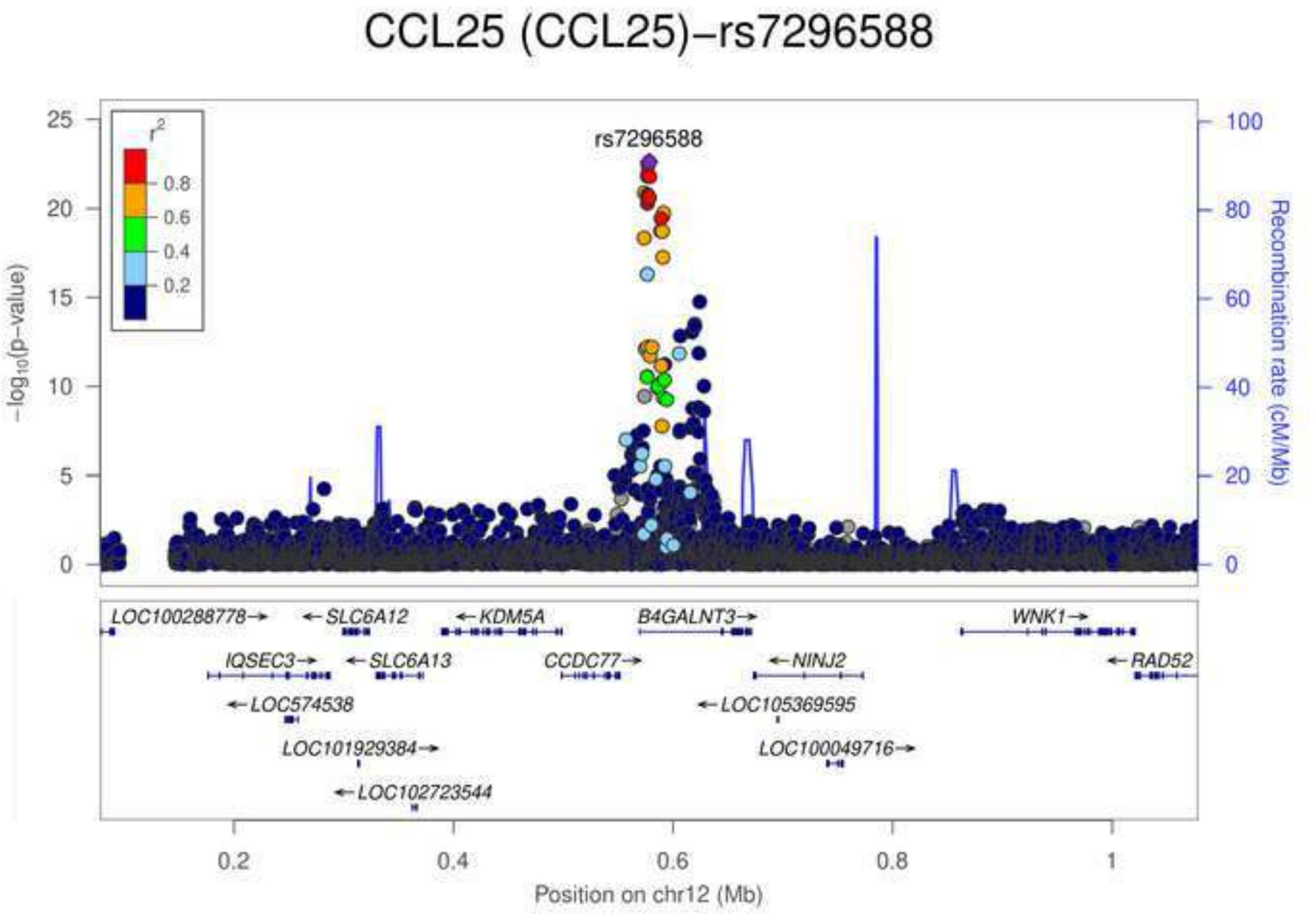
CCL25 (CCL25)−rs7296588
rs7296588
r^2
0.8
0.6
0.4
0.2
−log10(p−value)
Recombination rate (cM/Mb)
LOC100288778→
←SLC6A12
←KDM5A
B4GALNT3→
WNK1→
IQSEC3→
←SLC6A13
CCDC77→
←NINJ2
←RAD52
←LOC574538
←LOC105369595
LOC101929384→
LOC100049716→
←LOC102723544
Position on chr12 (Mb)

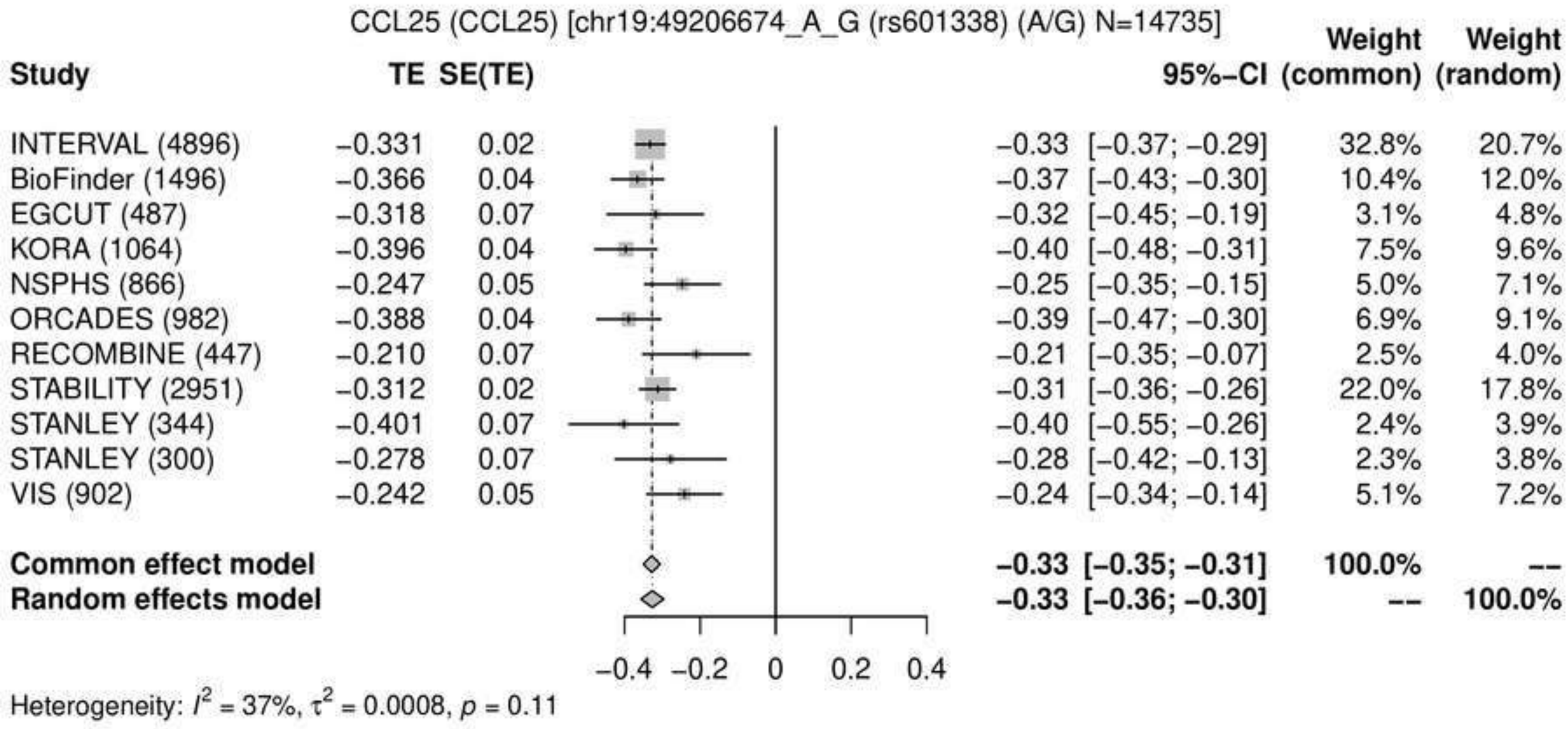
CCL25 (CCL25) [chr19:49206674_A_G (rs601338) (A/G) N=14735]
Study
TE
SE(TE)
95%-CI
Weight (common)
Weight (random)
INTERVAL (4896) −0.331 0.02 −0.33 [−0.37; −0.29] 32.8% 20.7%
BioFinder (1496) −0.366 0.04 −0.37 [−0.43; −0.30] 10.4% 12.0%
EGCUT (487) −0.318 0.07 −0.32 [−0.45; −0.19] 3.1% 4.8%
KORA (1064) −0.396 0.04 −0.40 [−0.48; −0.31] 7.5% 9.6%
NSPHS (866) −0.247 0.05 −0.25 [−0.35; −0.15] 5.0% 7.1%
ORCADES (982) −0.388 0.04 −0.39 [−0.47; −0.30] 6.9% 9.1%
RECOMBINE (447) −0.210 0.07 −0.21 [−0.35; −0.07] 2.5% 4.0%
STABILITY (2951) −0.312 0.02 −0.31 [−0.36; −0.26] 22.0% 17.8%
STANLEY (344) −0.401 0.07 −0.40 [−0.55; −0.26] 2.4% 3.9%
STANLEY (300) −0.278 0.07 −0.28 [−0.42; −0.13] 2.3% 3.8%
VIS (902) −0.242 0.05 −0.24 [−0.34; −0.14] 5.1% 7.2%
Common effect model −0.33 [−0.35; −0.31] 100.0% --
Random effects model −0.33 [−0.36; −0.30] -- 100.0%
−0.4 −0.2 0 0.2 0.4
Heterogeneity: I^2 = 37%, τ^2 = 0.0008, p = 0.11

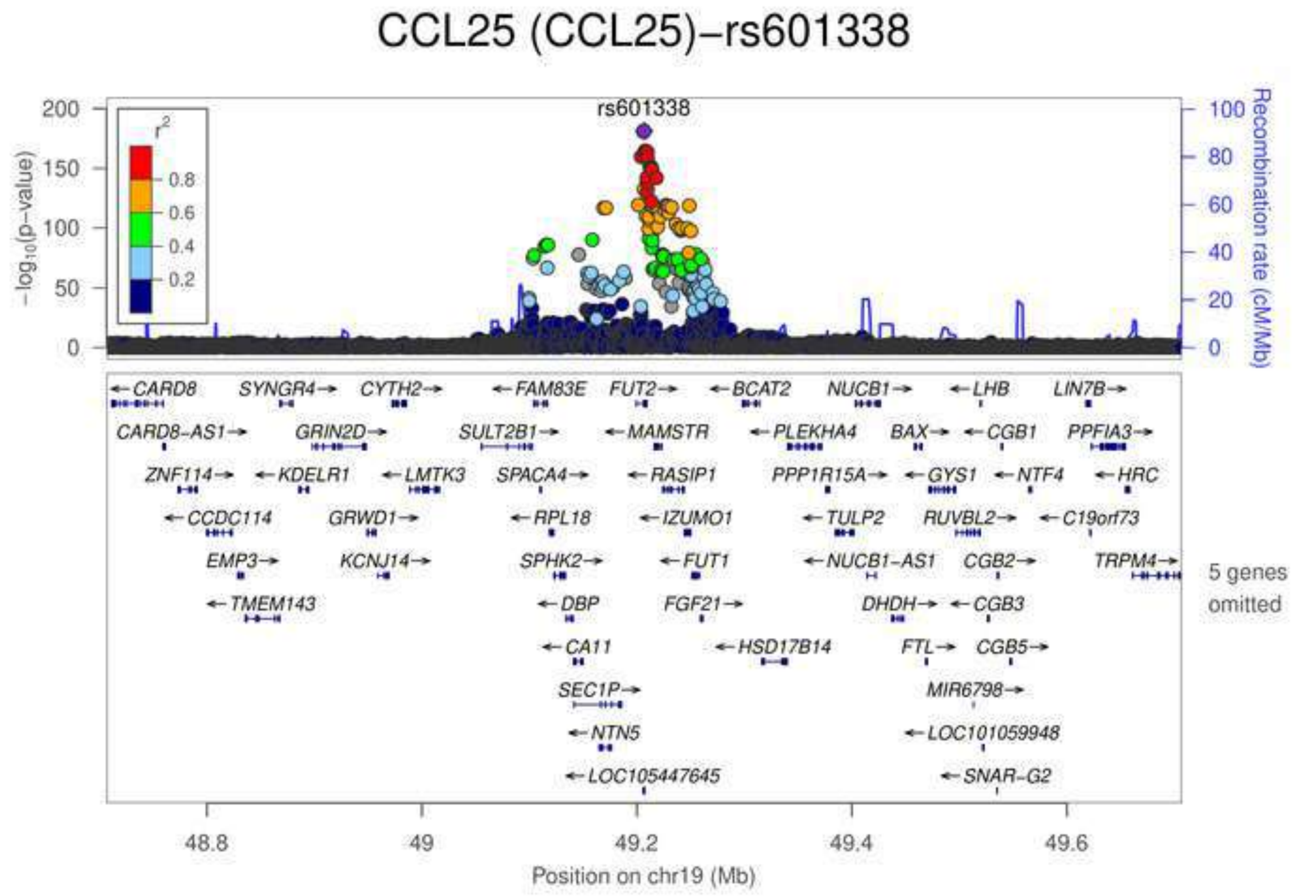
CCL25 (CCL25)−rs601338
rs601338
$-\log_{10}$(p-value)
Recombination rate (cM/Mb)
r^2
0.8
0.6
0.4
0.2
200
150
100
50
0
CARD8
SYNGR4
CYTH2
FAM83E
FUT2
BCAT2
NUCB1
LHB
LIN7B
CARD8-AS1
GRIN2D
SULT2B1
MAMSTR
PLEKHA4
BAX
CGB1
PPFIA3
ZNF114
KDELR1
LMTK3
SPACA4
RASIP1
PPP1R15A
GYS1
NTF4
HRC
CCDC114
GRWD1
RPL18
IZUMO1
TULP2
RUVBL2
C19orf73
EMP3
KCNJ14
SPHK2
FUT1
NUCB1-AS1
CGB2
TRPM4
TMEM143
DBP
FGF21
DHDH
CGB3
CA11
HSD17B14
FTL
CGB5
SEC1P
MIR6798
NTN5
LOC101059948
LOC105447645
SNAR-G2
5 genes omitted
48.8
49
49.2
49.4
49.6
Position on chr19 (Mb)

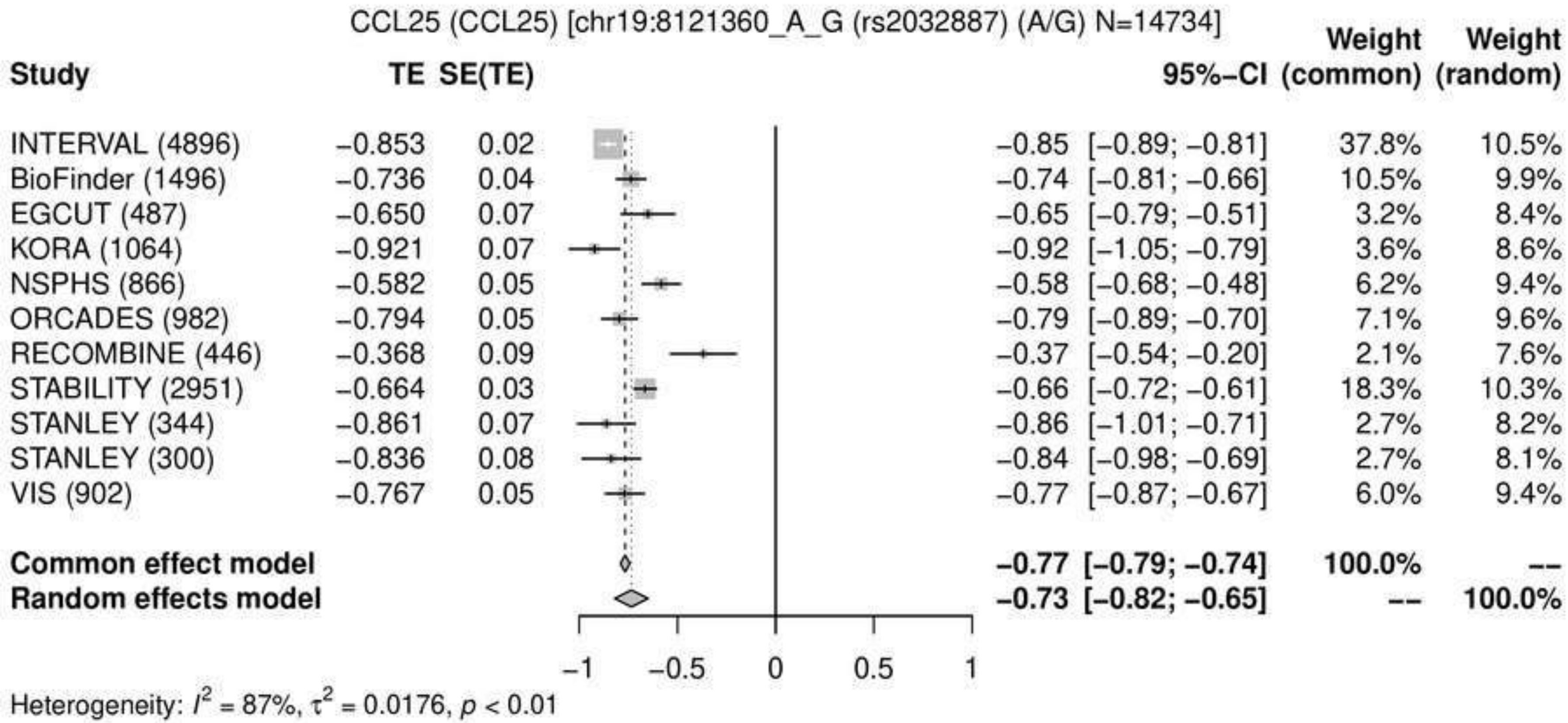
CCL25 (CCL25) [chr19:8121360_A_G (rs2032887) (A/G) N=14734]
Study
TE
SE(TE)
95%-CI
Weight (common)
Weight (random)
INTERVAL (4896) −0.853 0.02 −0.85 [−0.89; −0.81] 37.8% 10.5%
BioFinder (1496) −0.736 0.04 −0.74 [−0.81; −0.66] 10.5% 9.9%
EGCUT (487) −0.650 0.07 −0.65 [−0.79; −0.51] 3.2% 8.4%
KORA (1064) −0.921 0.07 −0.92 [−1.05; −0.79] 3.6% 8.6%
NSPHS (866) −0.582 0.05 −0.58 [−0.68; −0.48] 6.2% 9.4%
ORCADES (982) −0.794 0.05 −0.79 [−0.89; −0.70] 7.1% 9.6%
RECOMBINE (446) −0.368 0.09 −0.37 [−0.54; −0.20] 2.1% 7.6%
STABILITY (2951) −0.664 0.03 −0.66 [−0.72; −0.61] 18.3% 10.3%
STANLEY (344) −0.861 0.07 −0.86 [−1.01; −0.71] 2.7% 8.2%
STANLEY (300) −0.836 0.08 −0.84 [−0.98; −0.69] 2.7% 8.1%
VIS (902) −0.767 0.05 −0.77 [−0.87; −0.67] 6.0% 9.4%
Common effect model −0.77 [−0.79; −0.74] 100.0% --
Random effects model −0.73 [−0.82; −0.65] -- 100.0%
−1
−0.5
0
0.5
1
Heterogeneity: I^2 = 87%, τ^2 = 0.0176, p < 0.01

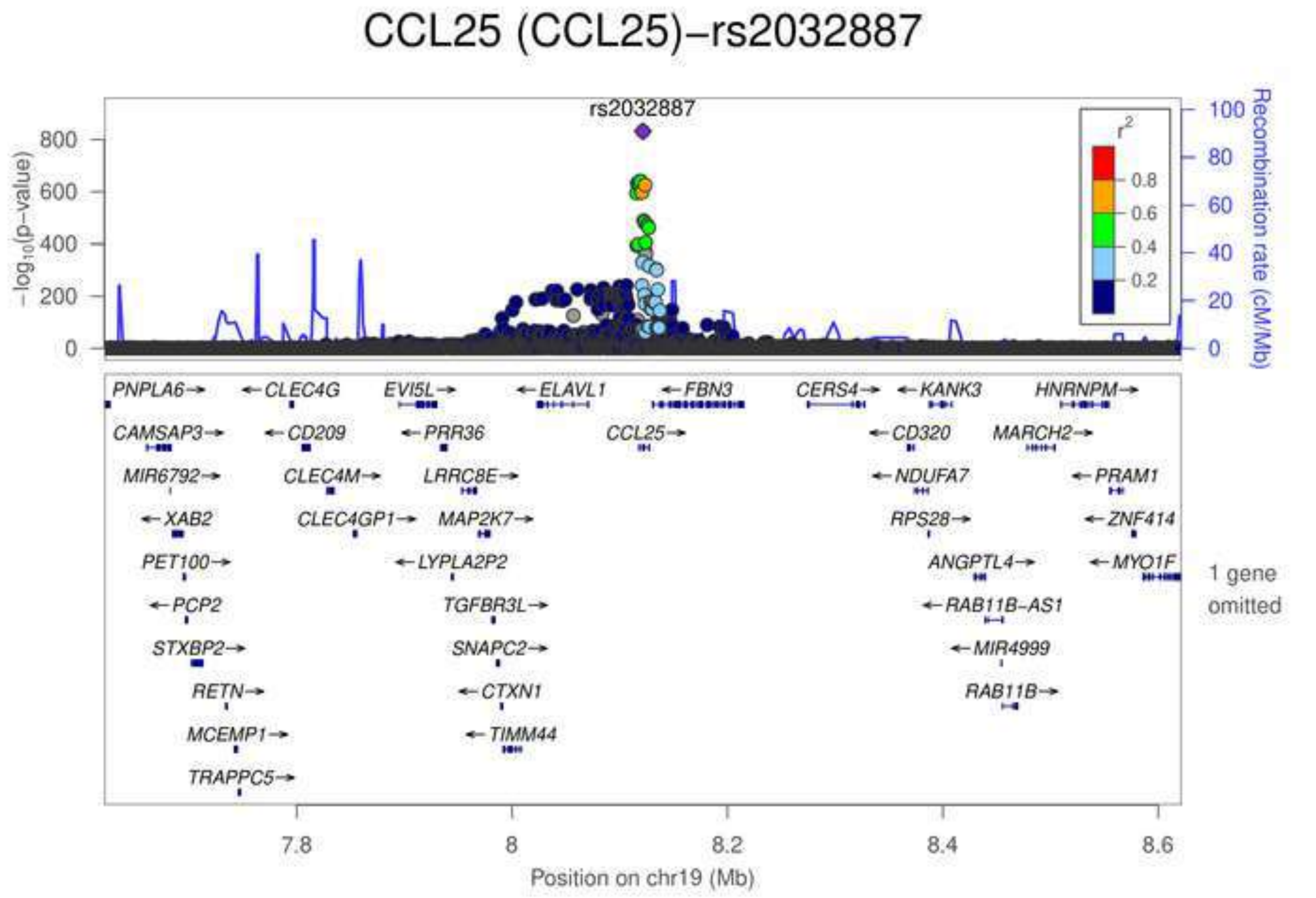
CCL25 (CCL25)−rs2032887
rs2032887
$-\log_{10}$(p−value)
Recombination rate (cM/Mb)
r^2
0.8
0.6
0.4
0.2
PNPLA6 CLEC4G EVI5L ELAVL1 FBN3 CERS4 KANK3 HNRNPM
CAMSAP3 CD209 PRR36 CCL25 CD320 MARCH2
MIR6792 CLEC4M LRRC8E NDUFA7 PRAM1
XAB2 CLEC4GP1 MAP2K7 RPS28 ZNF414
PET100 LYPLA2P2 ANGPTL4 MYO1F
PCP2 TGFBR3L RAB11B-AS1
STXBP2 SNAPC2 MIR4999
RETN CTXN1 RAB11B
MCEMP1 TIMM44
TRAPPC5
1 gene omitted
7.8
8
8.2
8.4
8.6
Position on chr19 (Mb)

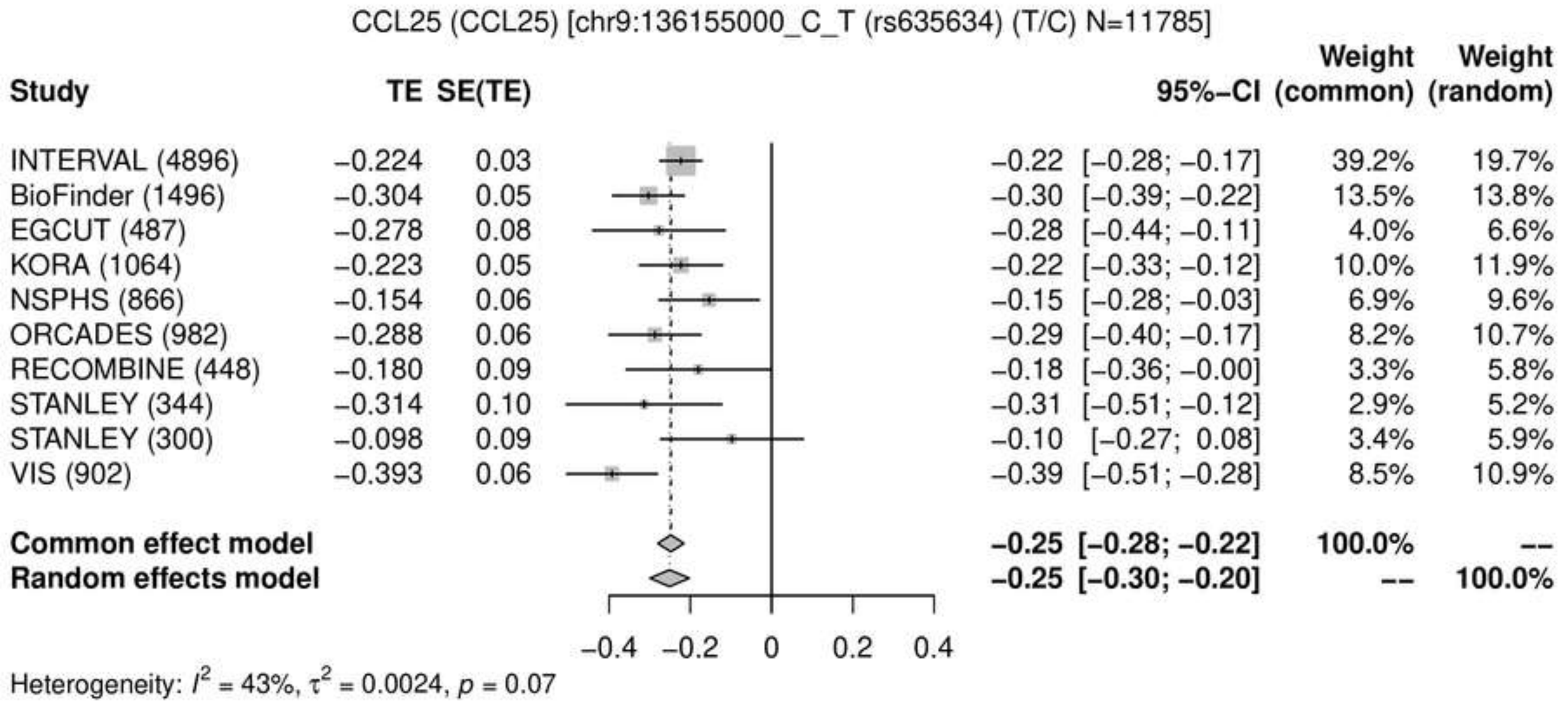

CCL25 (CCL25) [chr9:136155000_C_T (rs635634) (T/C) N=11785]
Study
TE
SE(TE)
95%-CI
Weight (common)
Weight (random)
INTERVAL (4896) −0.224 0.03 −0.22 [−0.28; −0.17] 39.2% 19.7%
BioFinder (1496) −0.304 0.05 −0.30 [−0.39; −0.22] 13.5% 13.8%
EGCUT (487) −0.278 0.08 −0.28 [−0.44; −0.11] 4.0% 6.6%
KORA (1064) −0.223 0.05 −0.22 [−0.33; −0.12] 10.0% 11.9%
NSPHS (866) −0.154 0.06 −0.15 [−0.28; −0.03] 6.9% 9.6%
ORCADES (982) −0.288 0.06 −0.29 [−0.40; −0.17] 8.2% 10.7%
RECOMBINE (448) −0.180 0.09 −0.18 [−0.36; −0.00] 3.3% 5.8%
STANLEY (344) −0.314 0.10 −0.31 [−0.51; −0.12] 2.9% 5.2%
STANLEY (300) −0.098 0.09 −0.10 [−0.27; 0.08] 3.4% 5.9%
VIS (902) −0.393 0.06 −0.39 [−0.51; −0.28] 8.5% 10.9%
Common effect model −0.25 [−0.28; −0.22] 100.0% --
Random effects model −0.25 [−0.30; −0.20] -- 100.0%
−0.4 −0.2 0 0.2 0.4
Heterogeneity: I^2 = 43%, τ^2 = 0.0024, p = 0.07

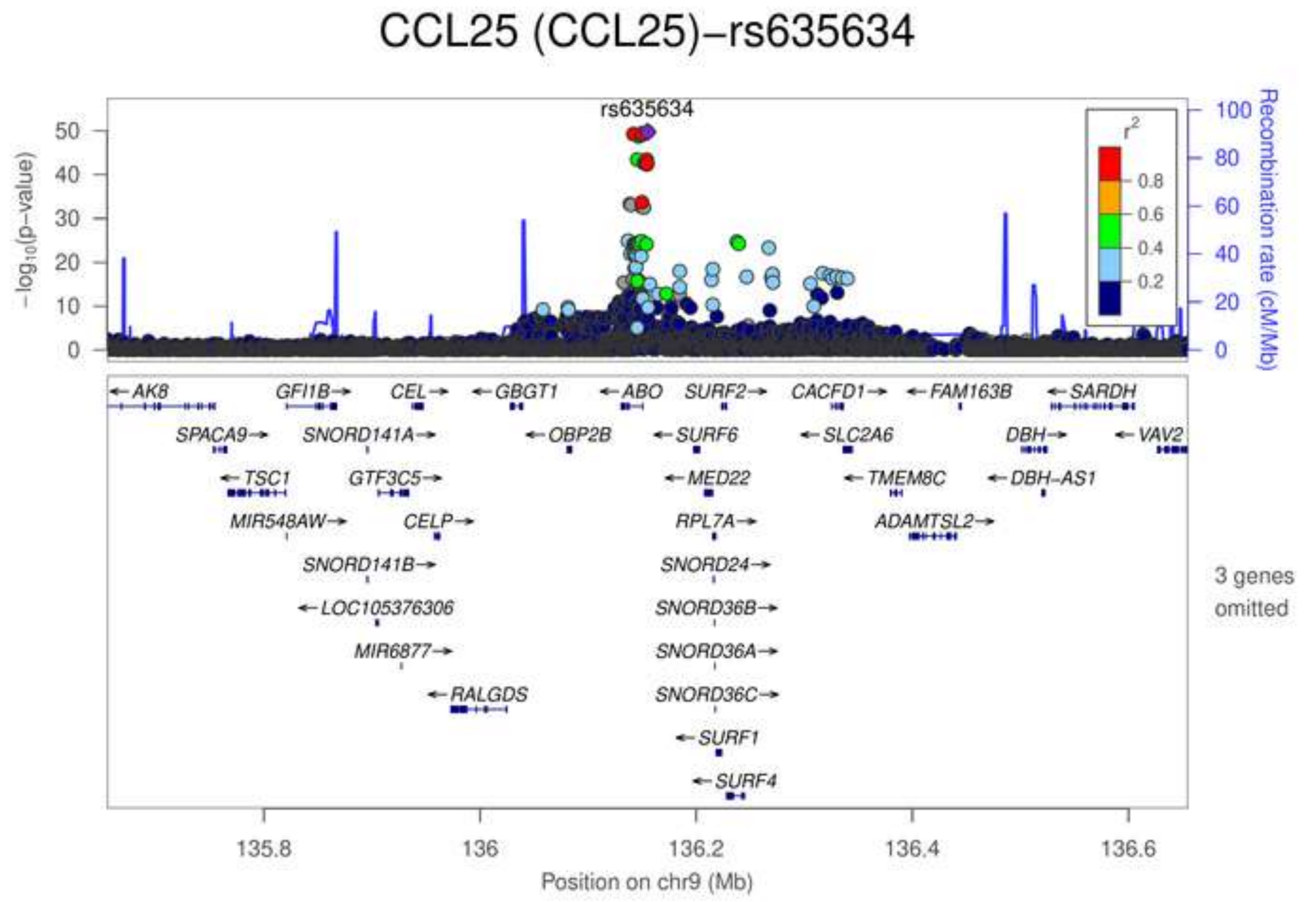

CCL25 (CCL25)−rs635634
rs635634
$-\log_{10}(p\text{-value})$
Recombination rate (cM/Mb)
r^2
0.8
0.6
0.4
0.2
AK8
GFI1B
CEL
GBGT1
ABO
SURF2
CACFD1
FAM163B
SARDH
SPACA9
SNORD141A
OBP2B
SURF6
SLC2A6
DBH
VAV2
TSC1
GTF3C5
MED22
TMEM8C
DBH−AS1
MIR548AW
CELP
RPL7A
ADAMTSL2
SNORD141B
SNORD24
LOC105376306
SNORD36B
MIR6877
SNORD36A
RALGDS
SNORD36C
SURF1
SURF4
3 genes omitted
135.8
136
136.2
136.4
136.6
Position on chr9 (Mb)

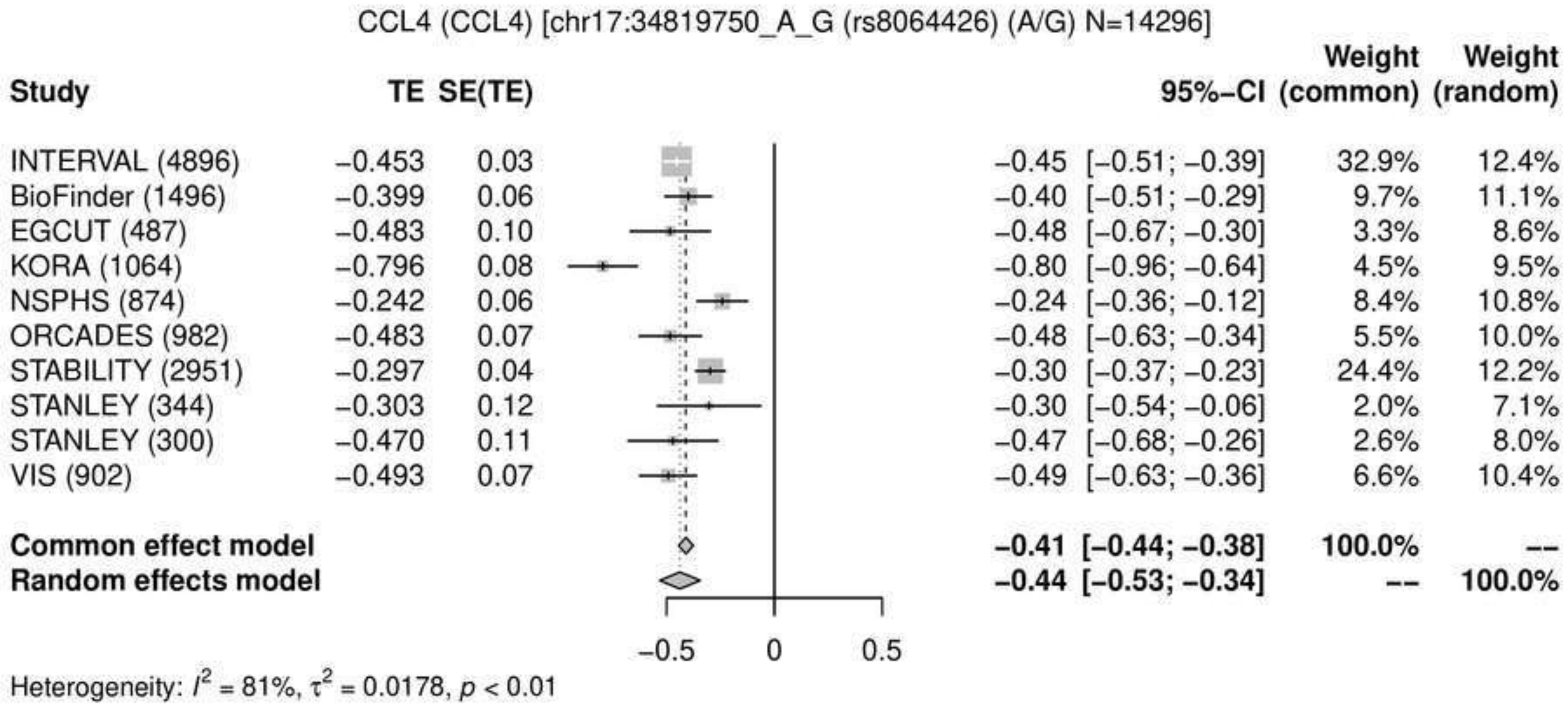
CCL4 (CCL4) [chr17:34819750_A_G (rs8064426) (A/G) N=14296]
Study TE SE(TE) 95%−CI Weight (common) Weight (random)
INTERVAL (4896) −0.453 0.03 −0.45 [−0.51; −0.39] 32.9% 12.4%
BioFinder (1496) −0.399 0.06 −0.40 [−0.51; −0.29] 9.7% 11.1%
EGCUT (487) −0.483 0.10 −0.48 [−0.67; −0.30] 3.3% 8.6%
KORA (1064) −0.796 0.08 −0.80 [−0.96; −0.64] 4.5% 9.5%
NSPHS (874) −0.242 0.06 −0.24 [−0.36; −0.12] 8.4% 10.8%
ORCADES (982) −0.483 0.07 −0.48 [−0.63; −0.34] 5.5% 10.0%
STABILITY (2951) −0.297 0.04 −0.30 [−0.37; −0.23] 24.4% 12.2%
STANLEY (344) −0.303 0.12 −0.30 [−0.54; −0.06] 2.0% 7.1%
STANLEY (300) −0.470 0.11 −0.47 [−0.68; −0.26] 2.6% 8.0%
VIS (902) −0.493 0.07 −0.49 [−0.63; −0.36] 6.6% 10.4%
Common effect model −0.41 [−0.44; −0.38] 100.0% --
Random effects model −0.44 [−0.53; −0.34] -- 100.0%
−0.5 0 0.5
Heterogeneity: I^2 = 81%, τ^2 = 0.0178, p < 0.01

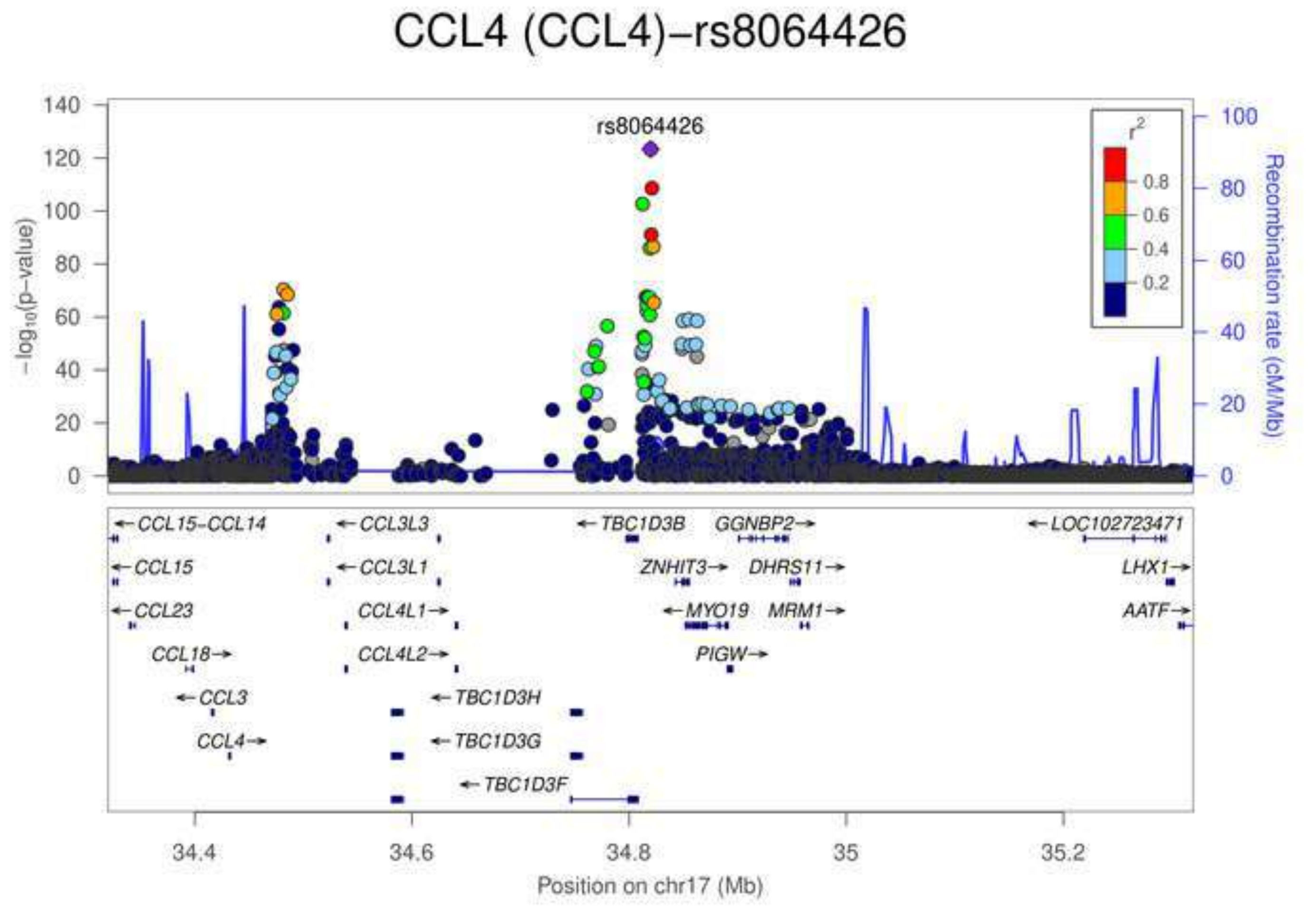
CCL4 (CCL4)−rs8064426
rs8064426
$-\log_{10}$(p−value)
Recombination rate (cM/Mb)
r^2
0.8
0.6
0.4
0.2
CCL15−CCL14
CCL15
CCL23
CCL18
CCL3
CCL4
CCL3L3
CCL3L1
CCL4L1
CCL4L2
TBC1D3H
TBC1D3G
TBC1D3F
TBC1D3B
ZNHIT3
MYO19
PIGW
GGNBP2
DHRS11
MRM1
LOC102723471
LHX1
AATF
34.4
34.6
34.8
35
35.2
Position on chr17 (Mb)

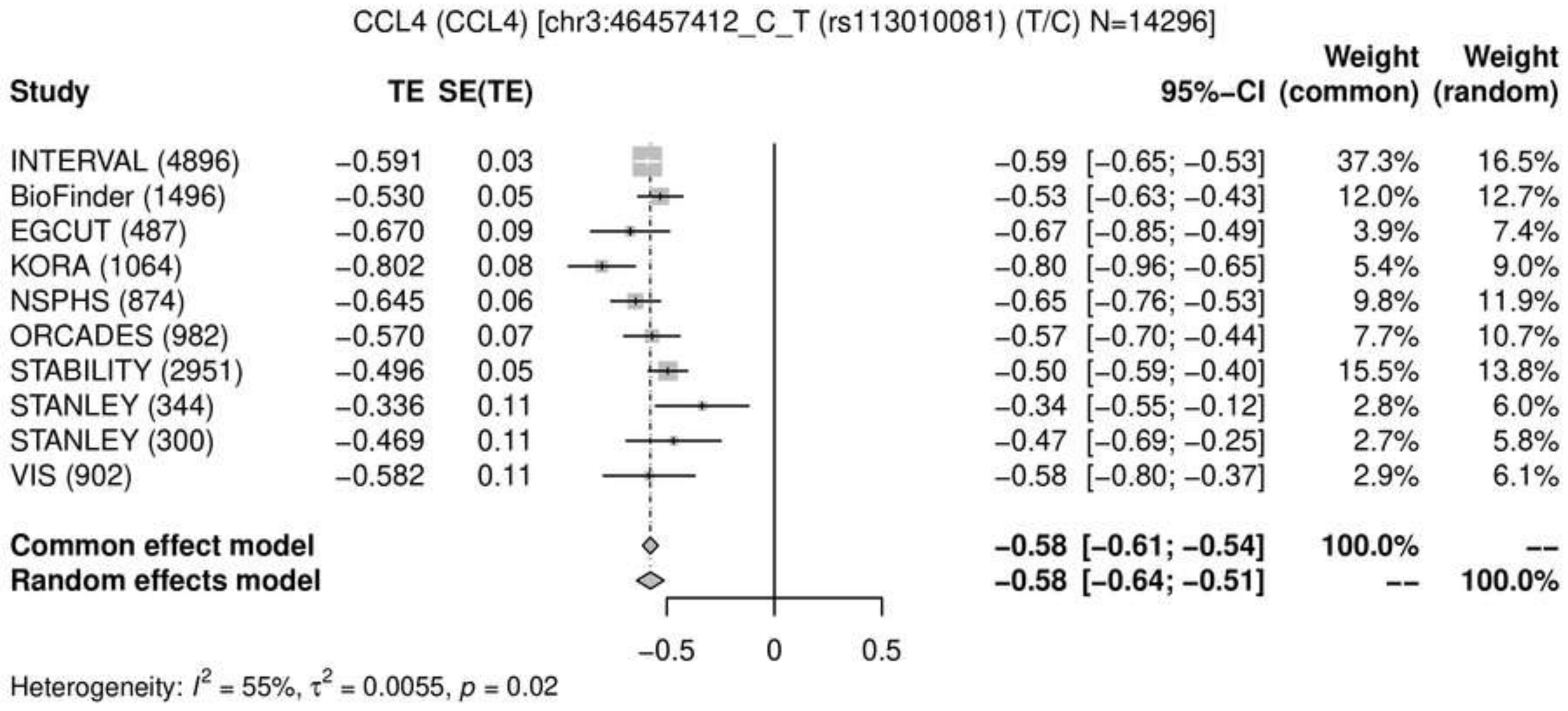
CCL4 (CCL4) [chr3:46457412_C_T (rs113010081) (T/C) N=14296]
Study
TE
SE(TE)
95%-CI
Weight (common)
Weight (random)
INTERVAL (4896) −0.591 0.03 −0.59 [−0.65; −0.53] 37.3% 16.5%
BioFinder (1496) −0.530 0.05 −0.53 [−0.63; −0.43] 12.0% 12.7%
EGCUT (487) −0.670 0.09 −0.67 [−0.85; −0.49] 3.9% 7.4%
KORA (1064) −0.802 0.08 −0.80 [−0.96; −0.65] 5.4% 9.0%
NSPHS (874) −0.645 0.06 −0.65 [−0.76; −0.53] 9.8% 11.9%
ORCADES (982) −0.570 0.07 −0.57 [−0.70; −0.44] 7.7% 10.7%
STABILITY (2951) −0.496 0.05 −0.50 [−0.59; −0.40] 15.5% 13.8%
STANLEY (344) −0.336 0.11 −0.34 [−0.55; −0.12] 2.8% 6.0%
STANLEY (300) −0.469 0.11 −0.47 [−0.69; −0.25] 2.7% 5.8%
VIS (902) −0.582 0.11 −0.58 [−0.80; −0.37] 2.9% 6.1%
Common effect model −0.58 [−0.61; −0.54] 100.0% --
Random effects model −0.58 [−0.64; −0.51] -- 100.0%
−0.5
0
0.5
Heterogeneity: I^2 = 55%, τ^2 = 0.0055, p = 0.02

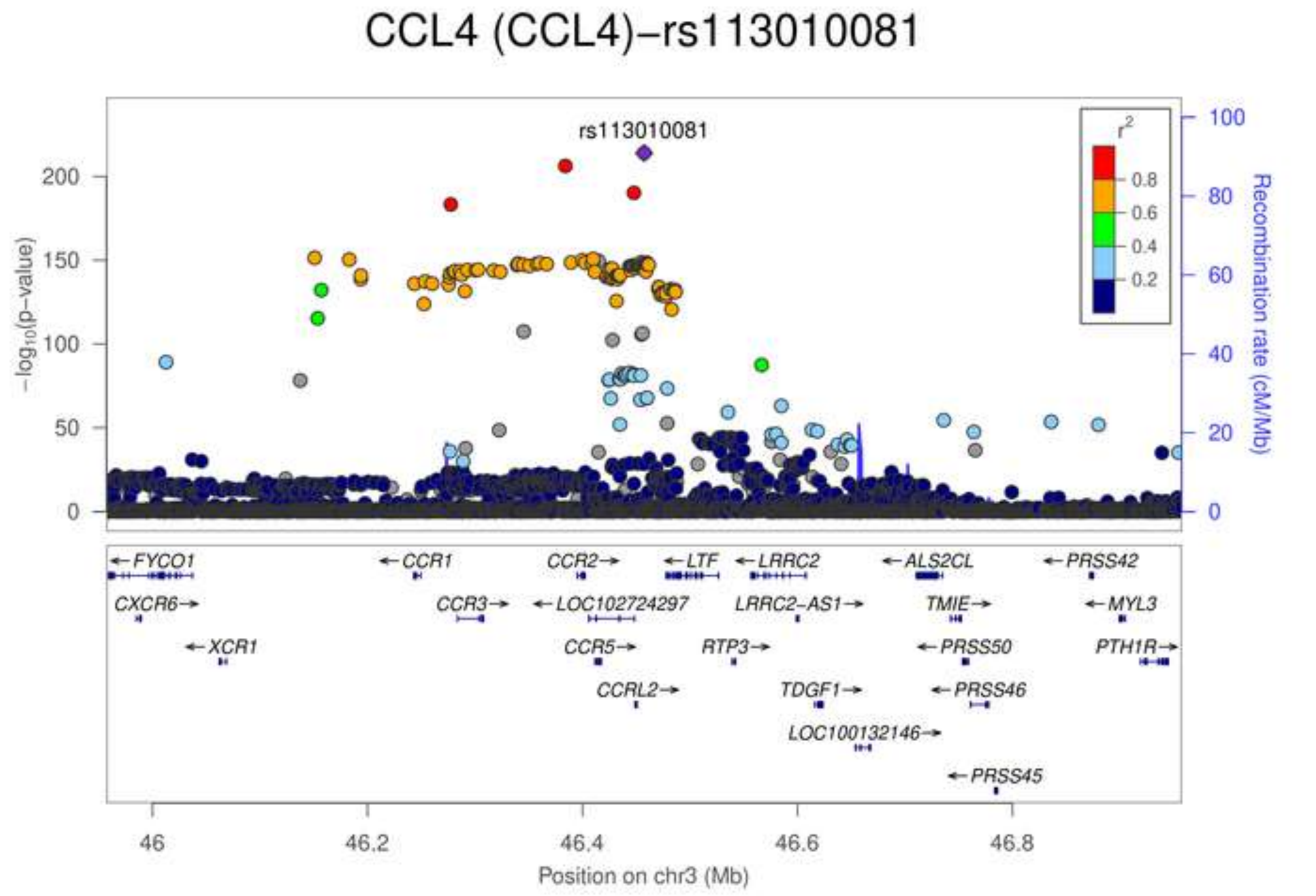
CCL4 (CCL4)−rs113010081
rs113010081
$-\log_{10}$(p−value)
Recombination rate (cM/Mb)
r^2
0.8
0.6
0.4
0.2
200
150
100
50
0
100
80
60
40
20
0
FYCO1
CXCR6
XCR1
CCR1
CCR3
CCR2
CCR5
CCRL2
LOC102724297
LTF
RTP3
LRRC2
LRRC2−AS1
TDGF1
LOC100132146
ALS2CL
TMIE
PRSS50
PRSS46
PRSS45
PRSS42
MYL3
PTH1R
46
46.2
46.4
46.6
46.8
Position on chr3 (Mb)

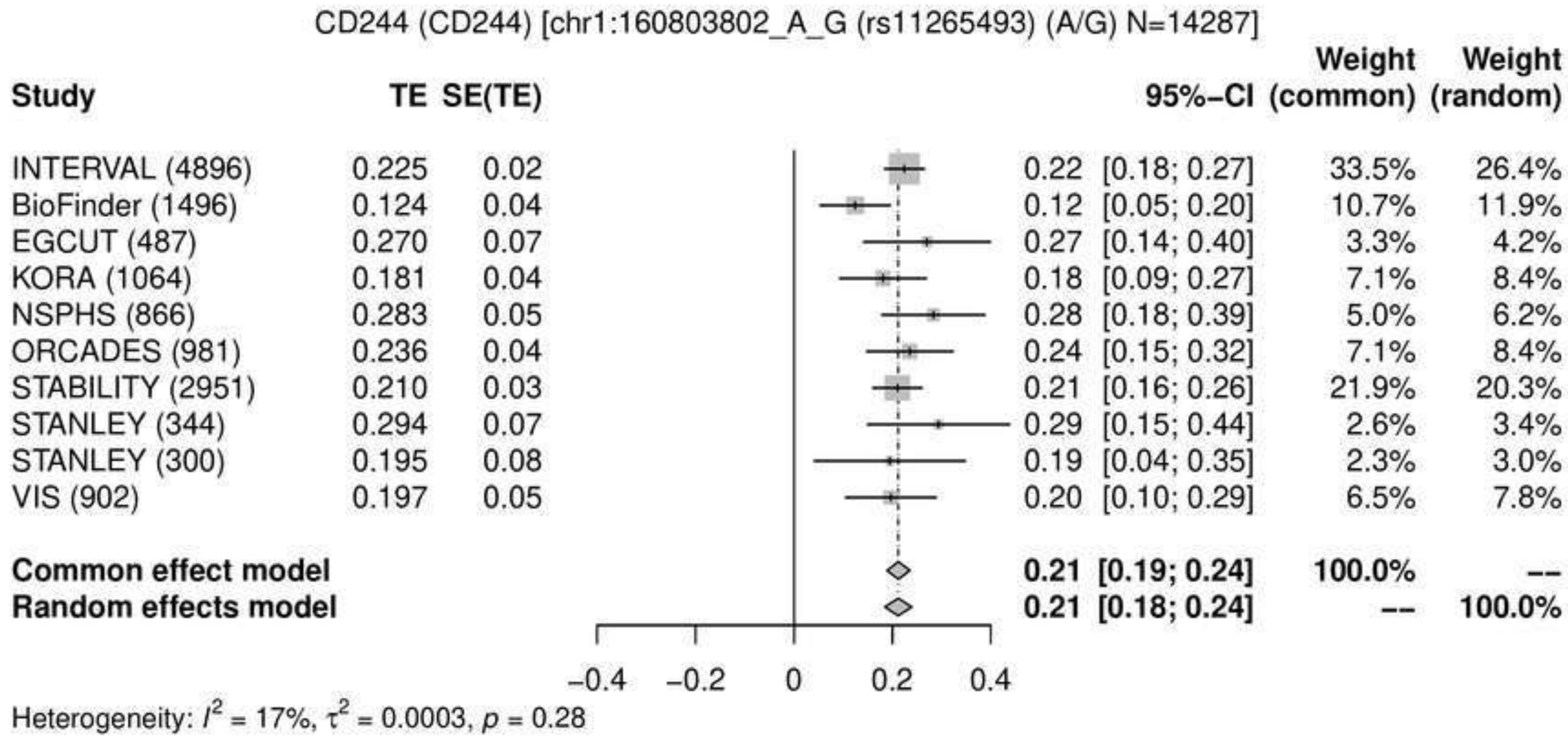
CD244 (CD244) [chr1:160803802_A_G (rs11265493) (A/G) N=14287]
Study TE SE(TE) 95%-CI Weight (common) Weight (random)
INTERVAL (4896) 0.225 0.02 0.22 [0.18; 0.27] 33.5% 26.4%
BioFinder (1496) 0.124 0.04 0.12 [0.05; 0.20] 10.7% 11.9%
EGCUT (487) 0.270 0.07 0.27 [0.14; 0.40] 3.3% 4.2%
KORA (1064) 0.181 0.04 0.18 [0.09; 0.27] 7.1% 8.4%
NSPHS (866) 0.283 0.05 0.28 [0.18; 0.39] 5.0% 6.2%
ORCADES (981) 0.236 0.04 0.24 [0.15; 0.32] 7.1% 8.4%
STABILITY (2951) 0.210 0.03 0.21 [0.16; 0.26] 21.9% 20.3%
STANLEY (344) 0.294 0.07 0.29 [0.15; 0.44] 2.6% 3.4%
STANLEY (300) 0.195 0.08 0.19 [0.04; 0.35] 2.3% 3.0%
VIS (902) 0.197 0.05 0.20 [0.10; 0.29] 6.5% 7.8%
Common effect model 0.21 [0.19; 0.24] 100.0% --
Random effects model 0.21 [0.18; 0.24] -- 100.0%
−0.4 −0.2 0 0.2 0.4
Heterogeneity: I^2 = 17%, τ^2 = 0.0003, p = 0.28

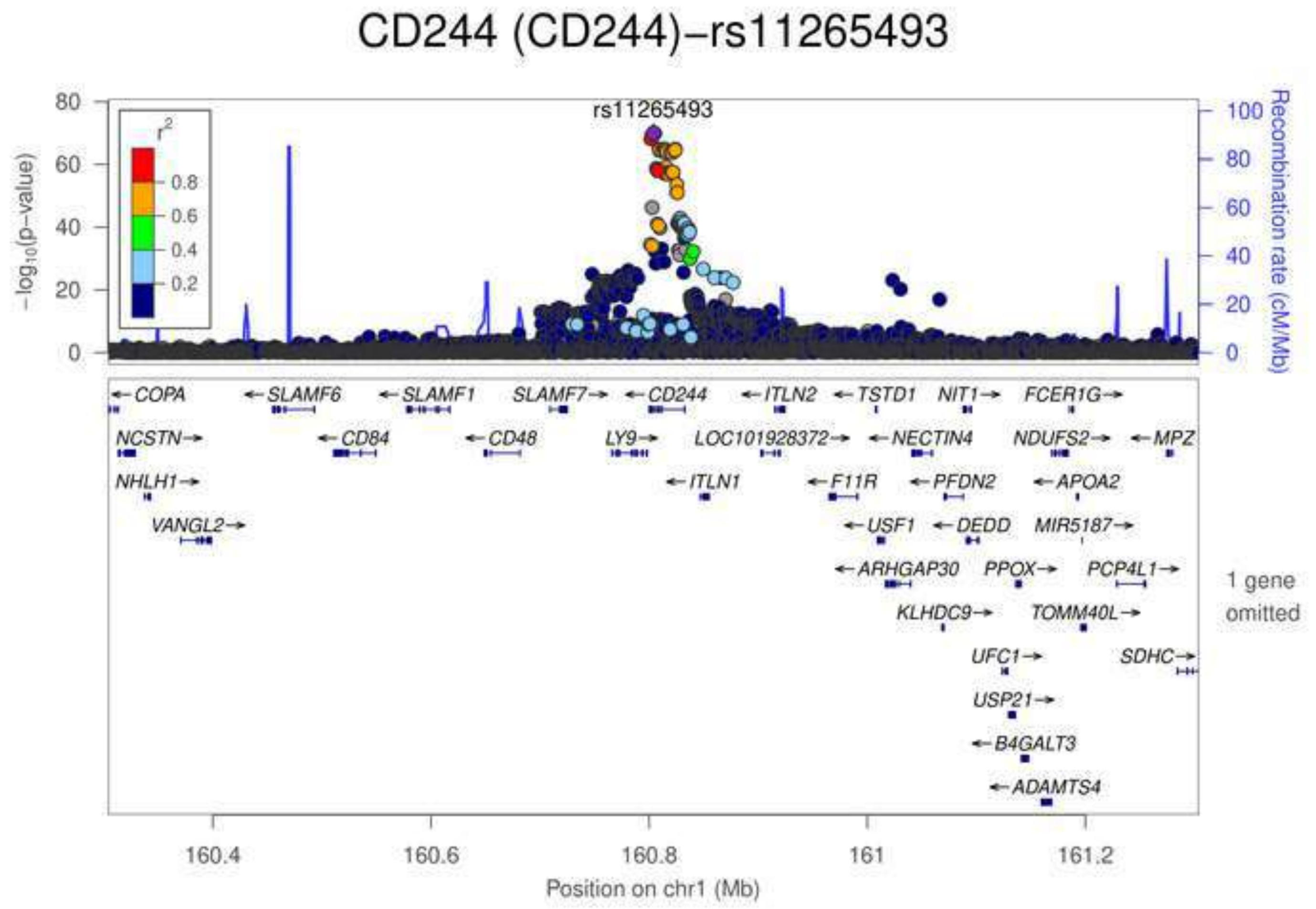
CD244 (CD244)−rs11265493
rs11265493
$-\log_{10}$(p−value)
Recombination rate (cM/Mb)
r^2
0.8
0.6
0.4
0.2
COPA
SLAMF6
SLAMF1
SLAMF7
CD244
ITLN2
TSTD1
NIT1
FCER1G
NCSTN
CD84
CD48
LY9
LOC101928372
NECTIN4
NDUFS2
MPZ
NHLH1
ITLN1
F11R
PFDN2
APOA2
VANGL2
USF1
DEDD
MIR5187
ARHGAP30
PPOX
PCP4L1
KLHDC9
TOMM40L
UFC1
SDHC
USP21
B4GALT3
ADAMTS4
1 gene omitted
160.4
160.6
160.8
161
161.2
Position on chr1 (Mb)

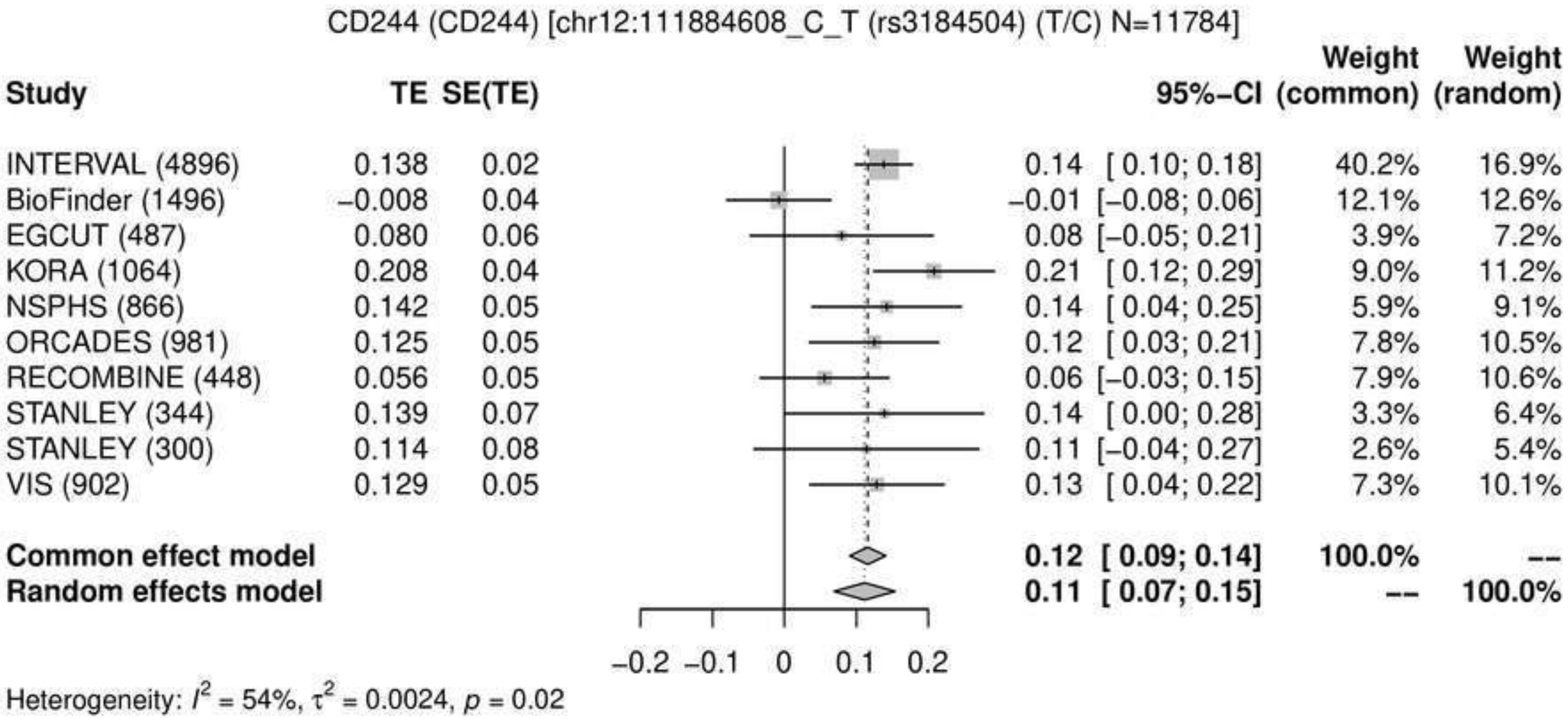
CD244 (CD244) [chr12:111884608_C_T (rs3184504) (T/C) N=11784]
Study | TE | SE(TE) | 95%−CI | Weight (common) | Weight (random)
INTERVAL (4896) | 0.138 | 0.02 | 0.14 [0.10; 0.18] | 40.2% | 16.9%
BioFinder (1496) | −0.008 | 0.04 | −0.01 [−0.08; 0.06] | 12.1% | 12.6%
EGCUT (487) | 0.080 | 0.06 | 0.08 [−0.05; 0.21] | 3.9% | 7.2%
KORA (1064) | 0.208 | 0.04 | 0.21 [0.12; 0.29] | 9.0% | 11.2%
NSPHS (866) | 0.142 | 0.05 | 0.14 [0.04; 0.25] | 5.9% | 9.1%
ORCADES (981) | 0.125 | 0.05 | 0.12 [0.03; 0.21] | 7.8% | 10.5%
RECOMBINE (448) | 0.056 | 0.05 | 0.06 [−0.03; 0.15] | 7.9% | 10.6%
STANLEY (344) | 0.139 | 0.07 | 0.14 [0.00; 0.28] | 3.3% | 6.4%
STANLEY (300) | 0.114 | 0.08 | 0.11 [−0.04; 0.27] | 2.6% | 5.4%
VIS (902) | 0.129 | 0.05 | 0.13 [0.04; 0.22] | 7.3% | 10.1%
Common effect model | 0.12 [0.09; 0.14] | 100.0% | --
Random effects model | 0.11 [0.07; 0.15] | -- | 100.0%
−0.2 −0.1 0 0.1 0.2
Heterogeneity: I^2 = 54%, τ^2 = 0.0024, p = 0.02

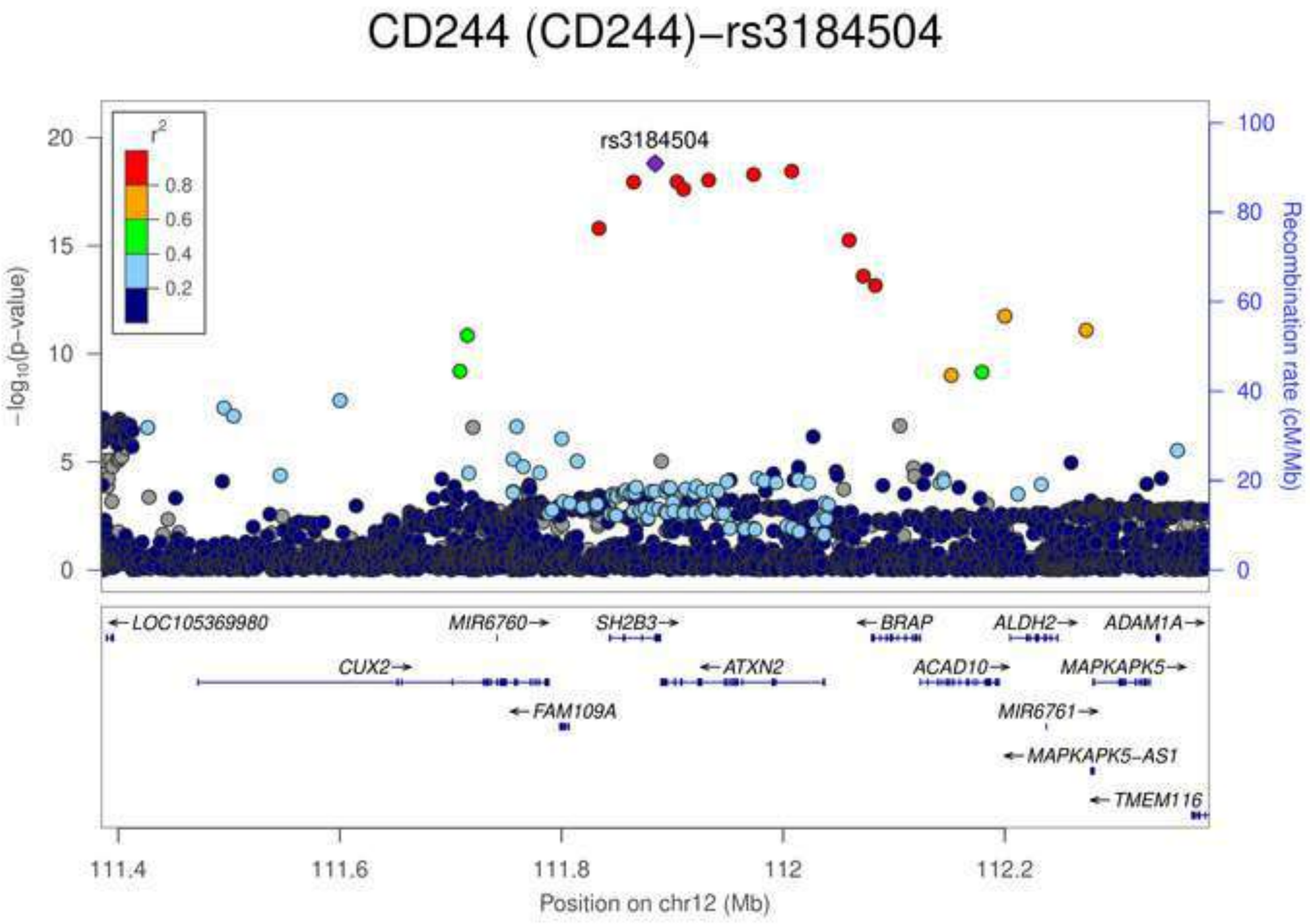
CD244 (CD244)−rs3184504
rs3184504
r^2
0.8
0.6
0.4
0.2
$-\log_{10}$(p-value)
Recombination rate (cM/Mb)
LOC105369980
MIR6760
SH2B3
BRAP
ALDH2
ADAM1A
CUX2
ATXN2
ACAD10
MAPKAPK5
FAM109A
MIR6761
MAPKAPK5-AS1
TMEM116
111.4
111.6
111.8
112
112.2
Position on chr12 (Mb)

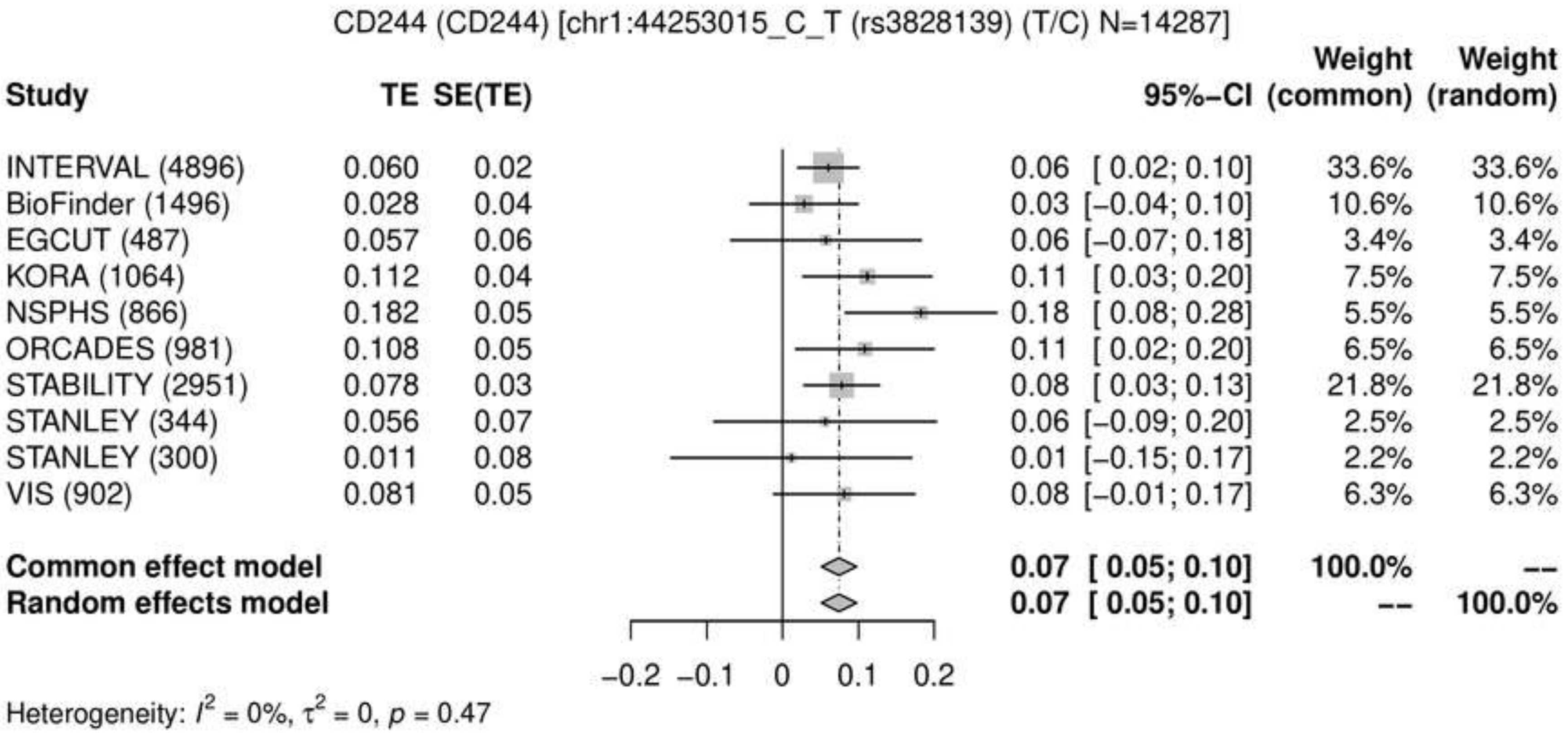
CD244 (CD244) [chr1:44253015_C_T (rs3828139) (T/C) N=14287]
Study
TE
SE(TE)
95%−CI
Weight (common)
Weight (random)
INTERVAL (4896) 0.060 0.02 0.06 [0.02; 0.10] 33.6% 33.6%
BioFinder (1496) 0.028 0.04 0.03 [−0.04; 0.10] 10.6% 10.6%
EGCUT (487) 0.057 0.06 0.06 [−0.07; 0.18] 3.4% 3.4%
KORA (1064) 0.112 0.04 0.11 [0.03; 0.20] 7.5% 7.5%
NSPHS (866) 0.182 0.05 0.18 [0.08; 0.28] 5.5% 5.5%
ORCADES (981) 0.108 0.05 0.11 [0.02; 0.20] 6.5% 6.5%
STABILITY (2951) 0.078 0.03 0.08 [0.03; 0.13] 21.8% 21.8%
STANLEY (344) 0.056 0.07 0.06 [−0.09; 0.20] 2.5% 2.5%
STANLEY (300) 0.011 0.08 0.01 [−0.15; 0.17] 2.2% 2.2%
VIS (902) 0.081 0.05 0.08 [−0.01; 0.17] 6.3% 6.3%
Common effect model 0.07 [0.05; 0.10] 100.0% --
Random effects model 0.07 [0.05; 0.10] -- 100.0%
−0.2 −0.1 0 0.1 0.2
Heterogeneity: I^2 = 0%, τ^2 = 0, p = 0.47

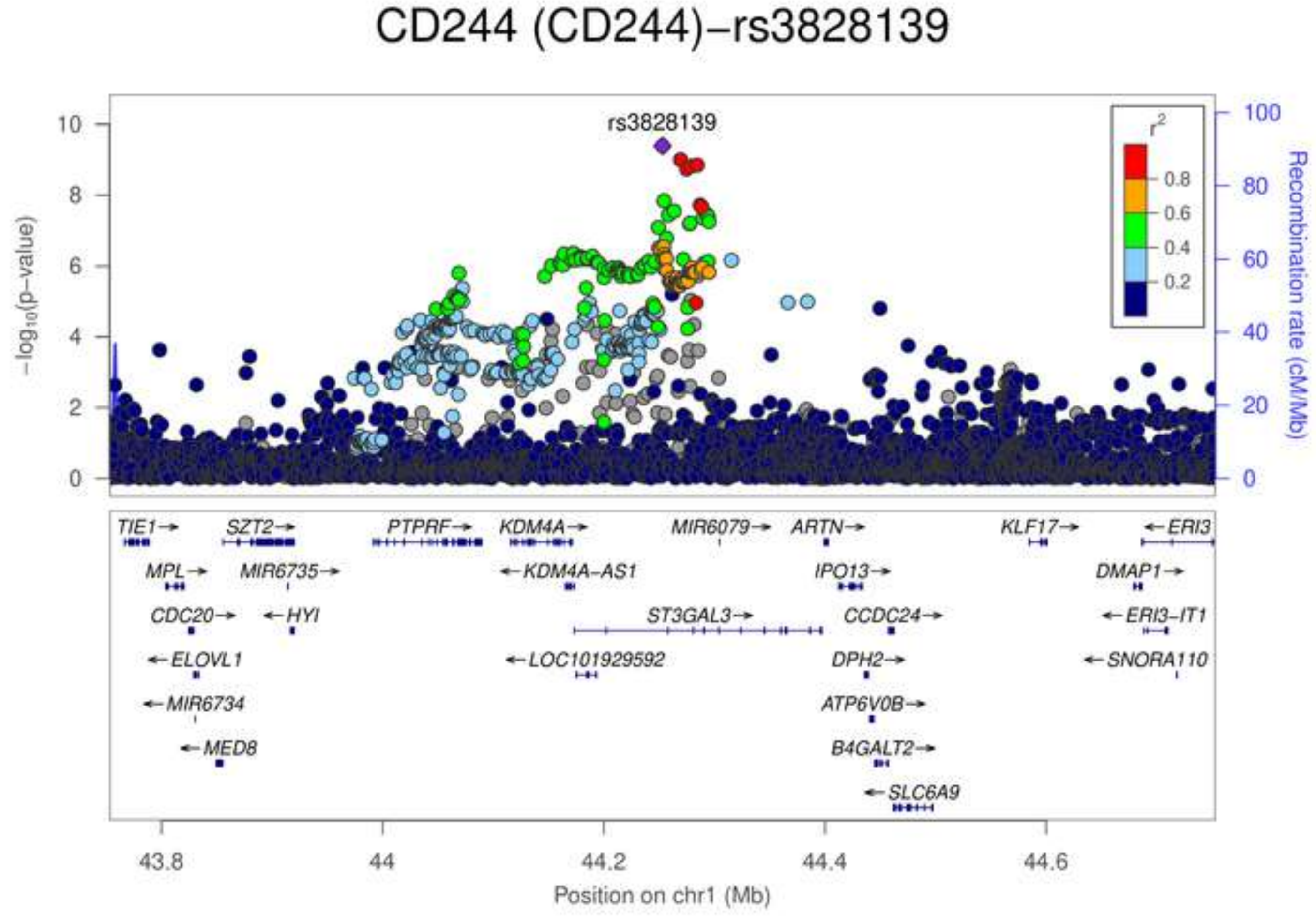
CD244 (CD244)−rs3828139
rs3828139
$-\log_{10}$(p−value)
Recombination rate (cM/Mb)
r^2
0.8
0.6
0.4
0.2
TIE1→
SZT2→
PTPRF→
KDM4A→
MIR6079→
ARTN→
KLF17→
←ERI3
MPL→
MIR6735→
←KDM4A-AS1
IPO13→
DMAP1→
CDC20→
←HYI
ST3GAL3→
CCDC24→
←ERI3-IT1
←ELOVL1
←LOC101929592
DPH2→
←SNORA110
←MIR6734
ATP6V0B→
←MED8
B4GALT2→
←SLC6A9
43.8
44
44.2
44.4
44.6
Position on chr1 (Mb)

CD40 (CD40) [chr20:44746982_C_T (rs1883832) (T/C) N=14736]

Study	TE	SE(TE)	95%-CI	Weight (common)	Weight (random)
INTERVAL (4896)	−0.453	0.02	−0.45 [−0.50; −0.41]	29.9%	10.4%
BioFinder (1496)	−0.311	0.04	−0.31 [−0.39; −0.23]	8.8%	9.7%
EGCUT (487)	−0.590	0.07	−0.59 [−0.73; −0.45]	3.0%	8.1%
KORA (1064)	−0.639	0.05	−0.64 [−0.73; −0.55]	7.3%	9.5%
NSPHS (866)	−0.557	0.06	−0.56 [−0.66; −0.45]	5.2%	9.1%
ORCADES (982)	−0.584	0.05	−0.58 [−0.68; −0.49]	7.2%	9.5%
RECOMBINE (448)	−0.234	0.05	−0.23 [−0.33; −0.14]	7.1%	9.4%
STABILITY (2951)	−0.486	0.03	−0.49 [−0.54; −0.43]	20.9%	10.2%
STANLEY (344)	−0.299	0.09	−0.30 [−0.47; −0.13]	2.0%	7.3%
STANLEY (300)	−0.301	0.08	−0.30 [−0.46; −0.14]	2.3%	7.6%
VIS (902)	−0.360	0.05	−0.36 [−0.46; −0.26]	6.3%	9.3%
**Common effect model**			**−0.45 [−0.48; −0.43]**	**100.0%**	**--**
**Random effects model**			**−0.44 [−0.52; −0.36]**	**--**	**100.0%**

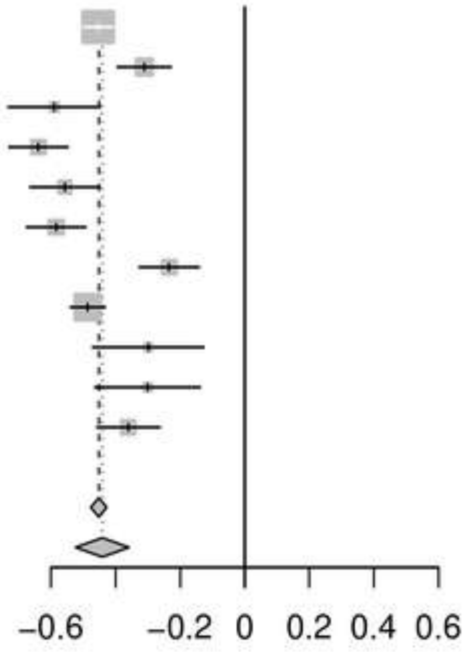

Heterogeneity: $I^2 = 87\%$, $\tau^2 = 0.0167$, $p < 0.01$

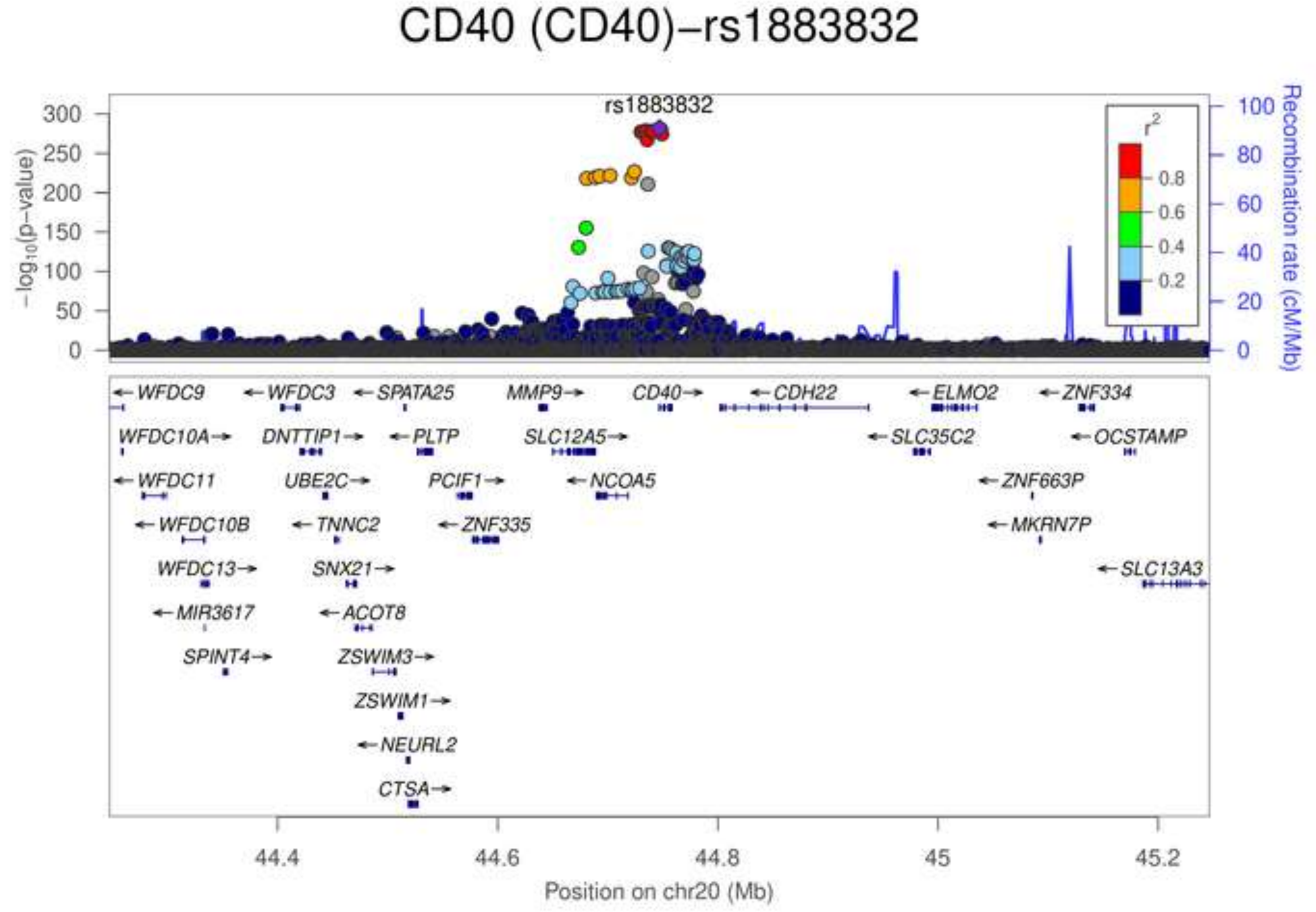

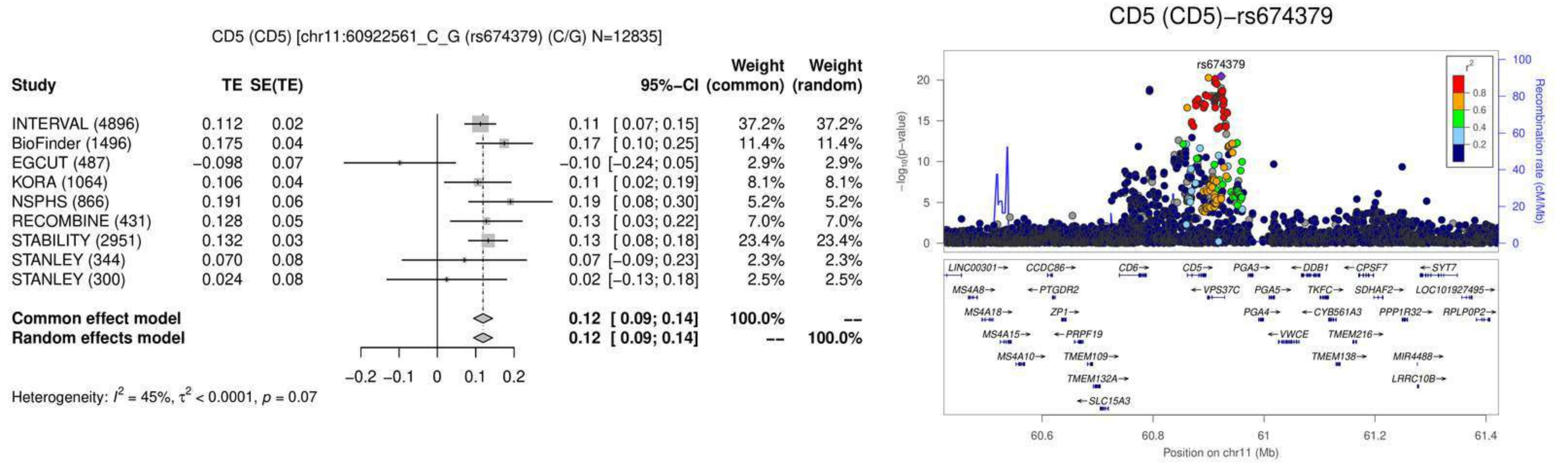
CD5 (CD5) [chr11:60922561_C_G (rs674379) (C/G) N=12835]
Study TE SE(TE) 95%-CI Weight (common) Weight (random)
INTERVAL (4896) 0.112 0.02 0.11 [0.07; 0.15] 37.2% 37.2%
BioFinder (1496) 0.175 0.04 0.17 [0.10; 0.25] 11.4% 11.4%
EGCUT (487) −0.098 0.07 −0.10 [−0.24; 0.05] 2.9% 2.9%
KORA (1064) 0.106 0.04 0.11 [0.02; 0.19] 8.1% 8.1%
NSPHS (866) 0.191 0.06 0.19 [0.08; 0.30] 5.2% 5.2%
RECOMBINE (431) 0.128 0.05 0.13 [0.03; 0.22] 7.0% 7.0%
STABILITY (2951) 0.132 0.03 0.13 [0.08; 0.18] 23.4% 23.4%
STANLEY (344) 0.070 0.08 0.07 [−0.09; 0.23] 2.3% 2.3%
STANLEY (300) 0.024 0.08 0.02 [−0.13; 0.18] 2.5% 2.5%
Common effect model 0.12 [0.09; 0.14] 100.0% --
Random effects model 0.12 [0.09; 0.14] -- 100.0%
−0.2 −0.1 0 0.1 0.2
Heterogeneity: I^2 = 45%, τ^2 < 0.0001, p = 0.07
CD5 (CD5)−rs674379
rs674379
$-\log_{10}$(p−value)
Recombination rate (cM/Mb)
r^2
0.8
0.6
0.4
0.2
LINC00301→ CCDC86→ CD6→ CD5→ PGA3→ ←DDB1 ←CPSF7 ←SYT7
MS4A8→ ←PTGDR2 ←VPS37C PGA5→ TKFC→ SDHAF2→ LOC101927495→
MS4A18→ ZP1→ PGA4→ ←CYB561A3 PPP1R32→ RPLP0P2→
MS4A15→ ←PRPF19 ←VWCE TMEM216→
MS4A10→ TMEM109→ TMEM138→ MIR4488→
TMEM132A→ LRRC10B→
←SLC15A3
60.6 60.8 61 61.2 61.4
Position on chr11 (Mb)

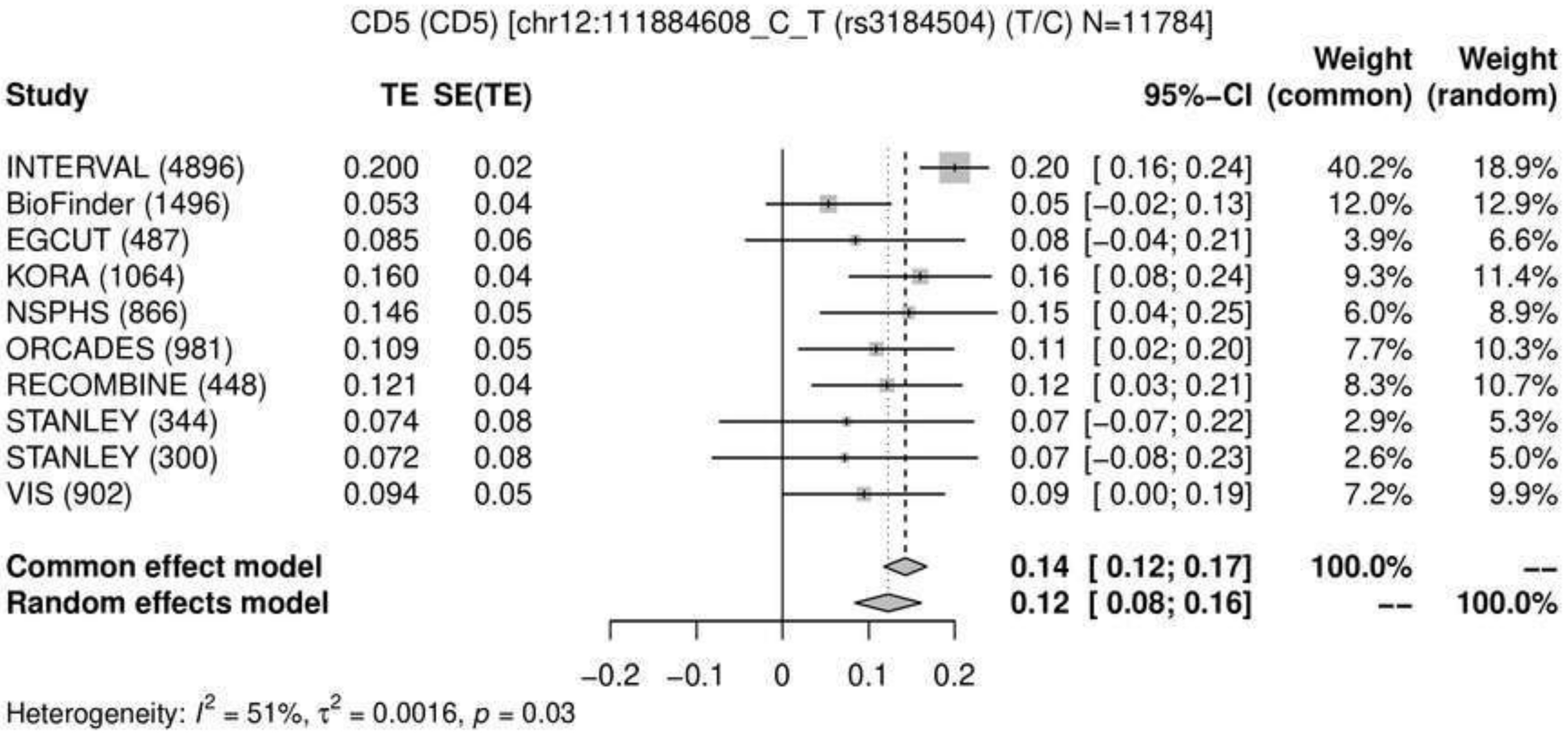
CD5 (CD5) [chr12:111884608_C_T (rs3184504) (T/C) N=11784]
Study TE SE(TE) 95%−CI Weight (common) Weight (random)
INTERVAL (4896) 0.200 0.02 0.20 [0.16; 0.24] 40.2% 18.9%
BioFinder (1496) 0.053 0.04 0.05 [−0.02; 0.13] 12.0% 12.9%
EGCUT (487) 0.085 0.06 0.08 [−0.04; 0.21] 3.9% 6.6%
KORA (1064) 0.160 0.04 0.16 [0.08; 0.24] 9.3% 11.4%
NSPHS (866) 0.146 0.05 0.15 [0.04; 0.25] 6.0% 8.9%
ORCADES (981) 0.109 0.05 0.11 [0.02; 0.20] 7.7% 10.3%
RECOMBINE (448) 0.121 0.04 0.12 [0.03; 0.21] 8.3% 10.7%
STANLEY (344) 0.074 0.08 0.07 [−0.07; 0.22] 2.9% 5.3%
STANLEY (300) 0.072 0.08 0.07 [−0.08; 0.23] 2.6% 5.0%
VIS (902) 0.094 0.05 0.09 [0.00; 0.19] 7.2% 9.9%
Common effect model 0.14 [0.12; 0.17] 100.0% --
Random effects model 0.12 [0.08; 0.16] -- 100.0%
−0.2 −0.1 0 0.1 0.2
Heterogeneity: I^2 = 51%, τ^2 = 0.0016, p = 0.03

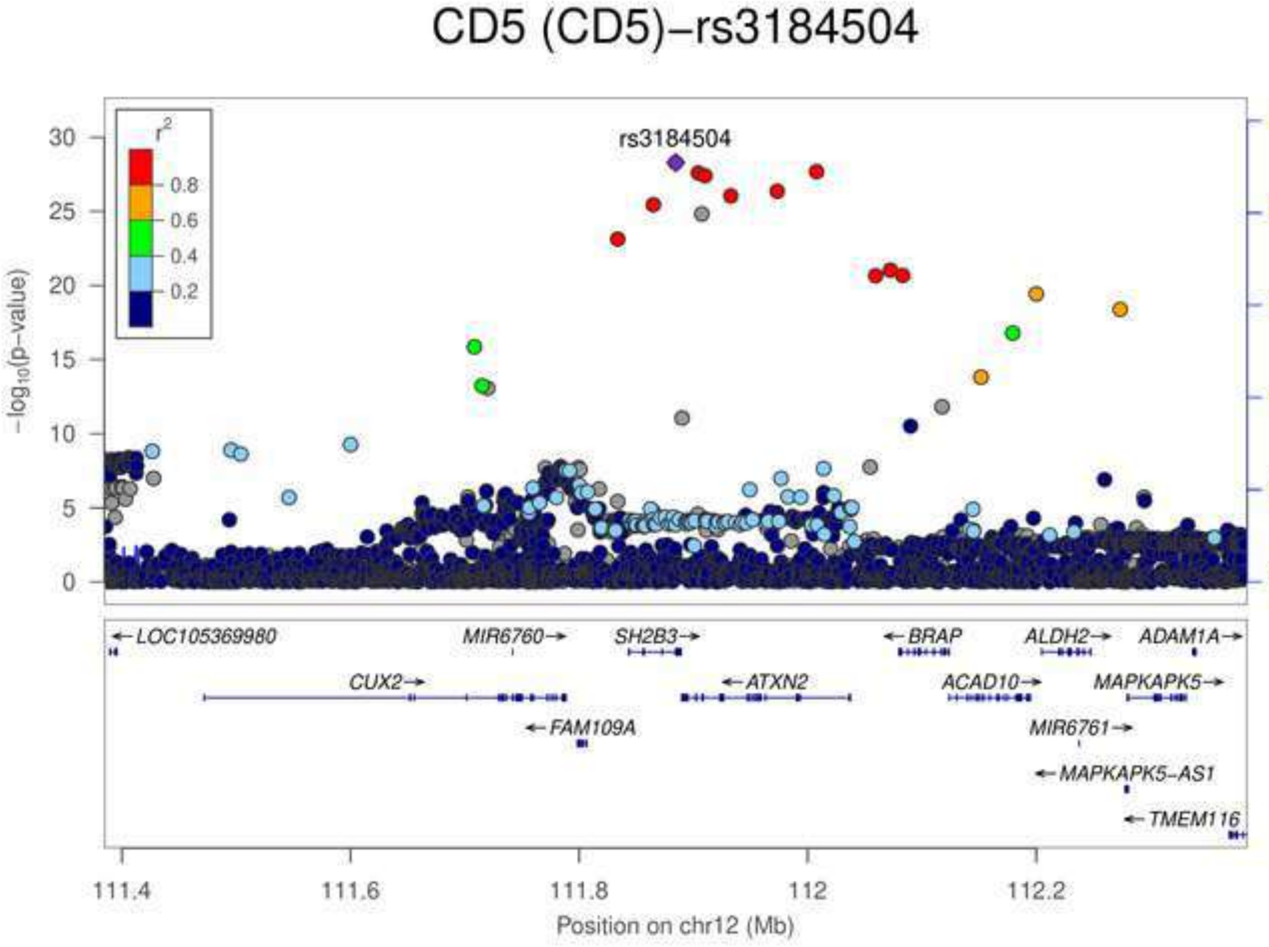
CD5 (CD5)−rs3184504
rs3184504
r^2
0.8
0.6
0.4
0.2
$-\log_{10}$(p-value)
Recombination rate (cM/Mb)
LOC105369980
MIR6760
SH2B3
BRAP
ALDH2
ADAM1A
CUX2
ATXN2
ACAD10
MAPKAPK5
FAM109A
MIR6761
MAPKAPK5-AS1
TMEM116
111.4 111.6 111.8 112 112.2
Position on chr12 (Mb)

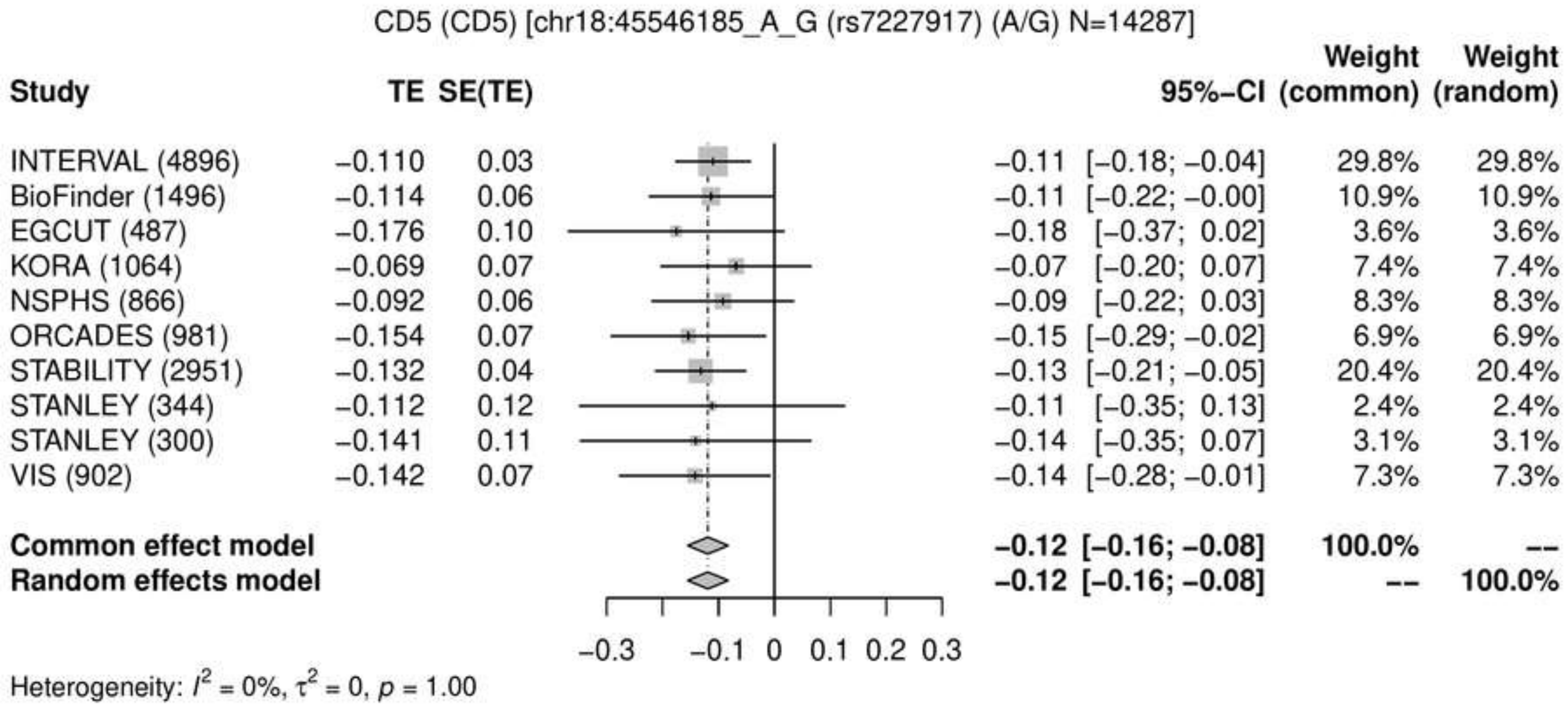
CD5 (CD5) [chr18:45546185_A_G (rs7227917) (A/G) N=14287]
Study | TE | SE(TE) | 95%-CI | Weight (common) | Weight (random)
INTERVAL (4896) | −0.110 | 0.03 | −0.11 [−0.18; −0.04] | 29.8% | 29.8%
BioFinder (1496) | −0.114 | 0.06 | −0.11 [−0.22; −0.00] | 10.9% | 10.9%
EGCUT (487) | −0.176 | 0.10 | −0.18 [−0.37; 0.02] | 3.6% | 3.6%
KORA (1064) | −0.069 | 0.07 | −0.07 [−0.20; 0.07] | 7.4% | 7.4%
NSPHS (866) | −0.092 | 0.06 | −0.09 [−0.22; 0.03] | 8.3% | 8.3%
ORCADES (981) | −0.154 | 0.07 | −0.15 [−0.29; −0.02] | 6.9% | 6.9%
STABILITY (2951) | −0.132 | 0.04 | −0.13 [−0.21; −0.05] | 20.4% | 20.4%
STANLEY (344) | −0.112 | 0.12 | −0.11 [−0.35; 0.13] | 2.4% | 2.4%
STANLEY (300) | −0.141 | 0.11 | −0.14 [−0.35; 0.07] | 3.1% | 3.1%
VIS (902) | −0.142 | 0.07 | −0.14 [−0.28; −0.01] | 7.3% | 7.3%
Common effect model | −0.12 [−0.16; −0.08] | 100.0% | --
Random effects model | −0.12 [−0.16; −0.08] | -- | 100.0%
−0.3 −0.1 0 0.1 0.2 0.3
Heterogeneity: I^2 = 0%, τ^2 = 0, p = 1.00

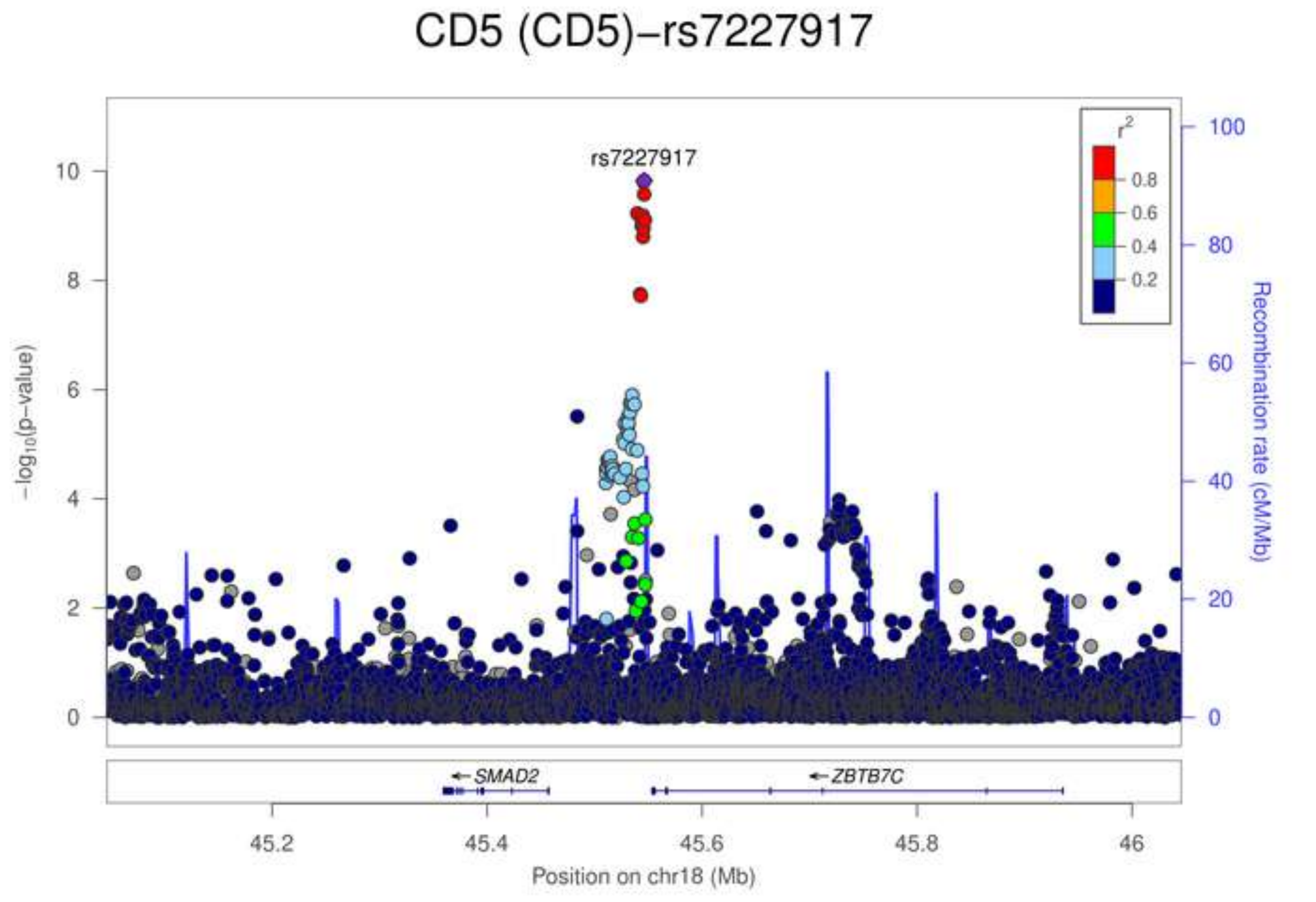
CD5 (CD5)−rs7227917
rs7227917
$-\log_{10}$(p−value)
Recombination rate (cM/Mb)
r^2
0.8
0.6
0.4
0.2
SMAD2
ZBTB7C
45.2
45.4
45.6
45.8
46
Position on chr18 (Mb)

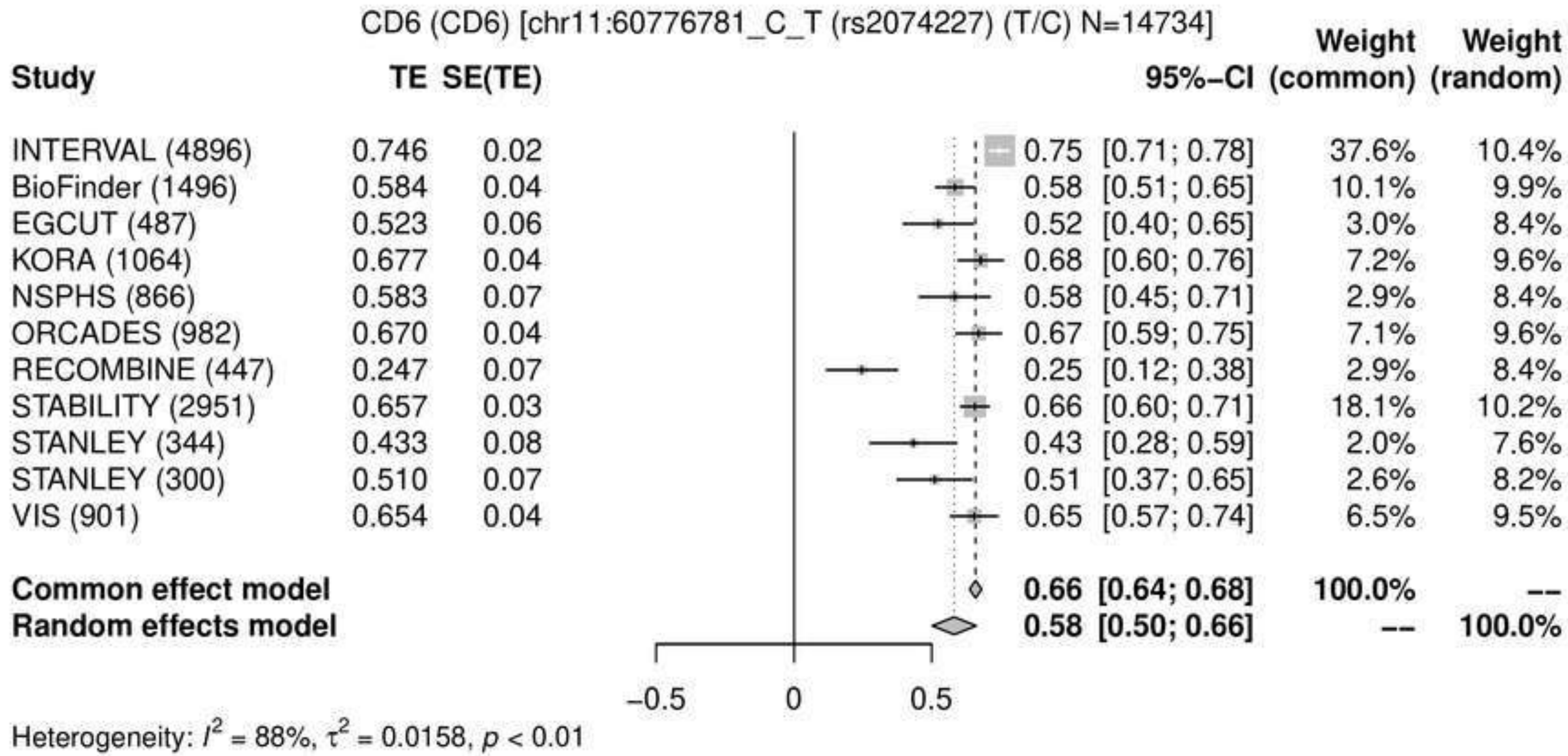
CD6 (CD6) [chr11:60776781_C_T (rs2074227) (T/C) N=14734]
Study
TE
SE(TE)
95%-CI
Weight (common)
Weight (random)
INTERVAL (4896) 0.746 0.02 0.75 [0.71; 0.78] 37.6% 10.4%
BioFinder (1496) 0.584 0.04 0.58 [0.51; 0.65] 10.1% 9.9%
EGCUT (487) 0.523 0.06 0.52 [0.40; 0.65] 3.0% 8.4%
KORA (1064) 0.677 0.04 0.68 [0.60; 0.76] 7.2% 9.6%
NSPHS (866) 0.583 0.07 0.58 [0.45; 0.71] 2.9% 8.4%
ORCADES (982) 0.670 0.04 0.67 [0.59; 0.75] 7.1% 9.6%
RECOMBINE (447) 0.247 0.07 0.25 [0.12; 0.38] 2.9% 8.4%
STABILITY (2951) 0.657 0.03 0.66 [0.60; 0.71] 18.1% 10.2%
STANLEY (344) 0.433 0.08 0.43 [0.28; 0.59] 2.0% 7.6%
STANLEY (300) 0.510 0.07 0.51 [0.37; 0.65] 2.6% 8.2%
VIS (901) 0.654 0.04 0.65 [0.57; 0.74] 6.5% 9.5%
Common effect model 0.66 [0.64; 0.68] 100.0% --
Random effects model 0.58 [0.50; 0.66] -- 100.0%
−0.5
0
0.5
Heterogeneity: I^2 = 88%, τ^2 = 0.0158, p < 0.01
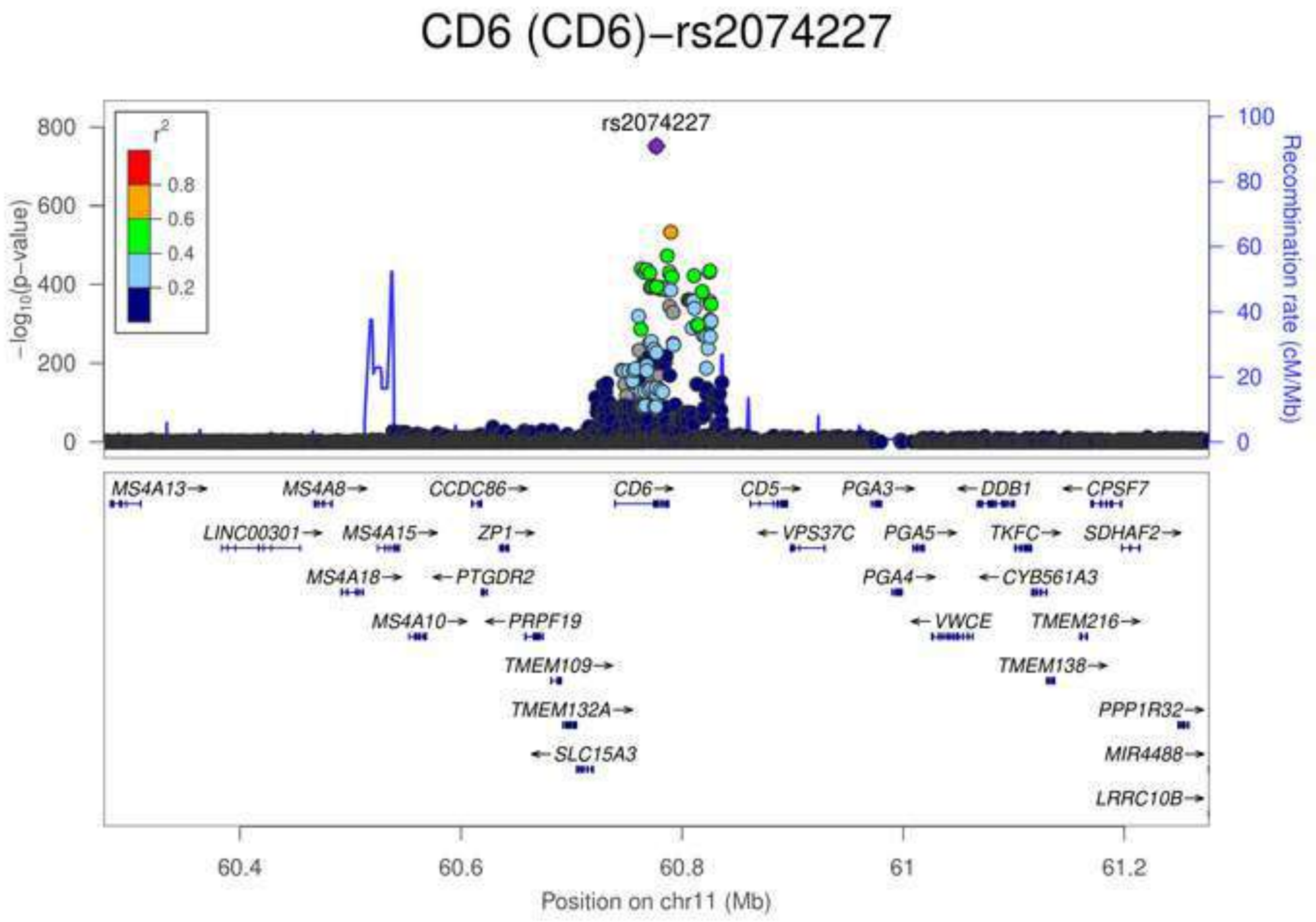
CD6 (CD6)−rs2074227
rs2074227
$-\log_{10}$(p−value)
800
600
400
200
0
r^2
0.8
0.6
0.4
0.2
Recombination rate (cM/Mb)
100
80
60
40
20
0
MS4A13→
MS4A8→
CCDC86→
CD6→
CD5→
PGA3→
←DDB1
←CPSF7
LINC00301→
MS4A15→
ZP1→
←VPS37C
PGA5→
TKFC→
SDHAF2→
MS4A18→
←PTGDR2
PGA4→
←CYB561A3
MS4A10→
←PRPF19
←VWCE
TMEM216→
TMEM109→
TMEM138→
TMEM132A→
PPP1R32→
←SLC15A3
MIR4488→
LRRC10B→
60.4
60.6
60.8
61
61.2
Position on chr11 (Mb)

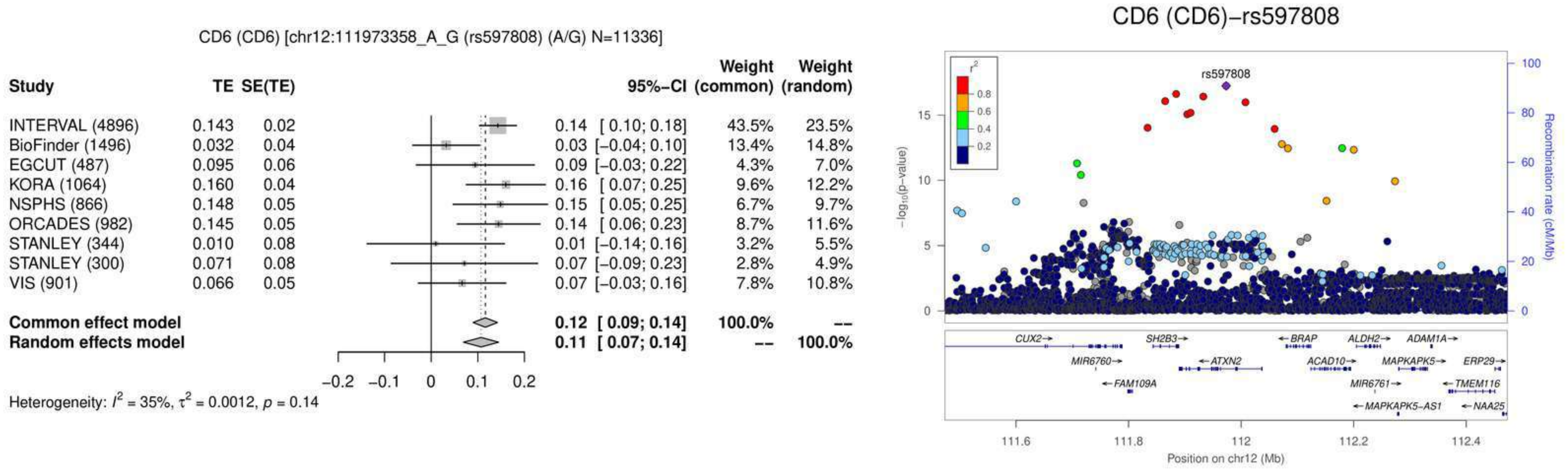

CD6 (CD6) [chr12:111973358_A_G (rs597808) (A/G) N=11336]
Study
TE
SE(TE)
95%-CI
Weight (common)
Weight (random)
INTERVAL (4896) 0.143 0.02 0.14 [0.10; 0.18] 43.5% 23.5%
BioFinder (1496) 0.032 0.04 0.03 [−0.04; 0.10] 13.4% 14.8%
EGCUT (487) 0.095 0.06 0.09 [−0.03; 0.22] 4.3% 7.0%
KORA (1064) 0.160 0.04 0.16 [0.07; 0.25] 9.6% 12.2%
NSPHS (866) 0.148 0.05 0.15 [0.05; 0.25] 6.7% 9.7%
ORCADES (982) 0.145 0.05 0.14 [0.06; 0.23] 8.7% 11.6%
STANLEY (344) 0.010 0.08 0.01 [−0.14; 0.16] 3.2% 5.5%
STANLEY (300) 0.071 0.08 0.07 [−0.09; 0.23] 2.8% 4.9%
VIS (901) 0.066 0.05 0.07 [−0.03; 0.16] 7.8% 10.8%
Common effect model 0.12 [0.09; 0.14] 100.0% --
Random effects model 0.11 [0.07; 0.14] -- 100.0%
−0.2 −0.1 0 0.1 0.2
Heterogeneity: I^2 = 35%, τ^2 = 0.0012, p = 0.14
CD6 (CD6)−rs597808
rs597808
r^2
0.8
0.6
0.4
0.2
$-\log_{10}$(p−value)
Recombination rate (cM/Mb)
0 5 10 15
0 20 40 60 80 100
CUX2→
SH2B3→
←BRAP
ALDH2→
ADAM1A→
MIR6760→
←ATXN2
ACAD10→
MAPKAPK5→
ERP29→
←FAM109A
MIR6761→
←TMEM116
←MAPKAPK5−AS1
←NAA25
111.6 111.8 112 112.2 112.4
Position on chr12 (Mb)

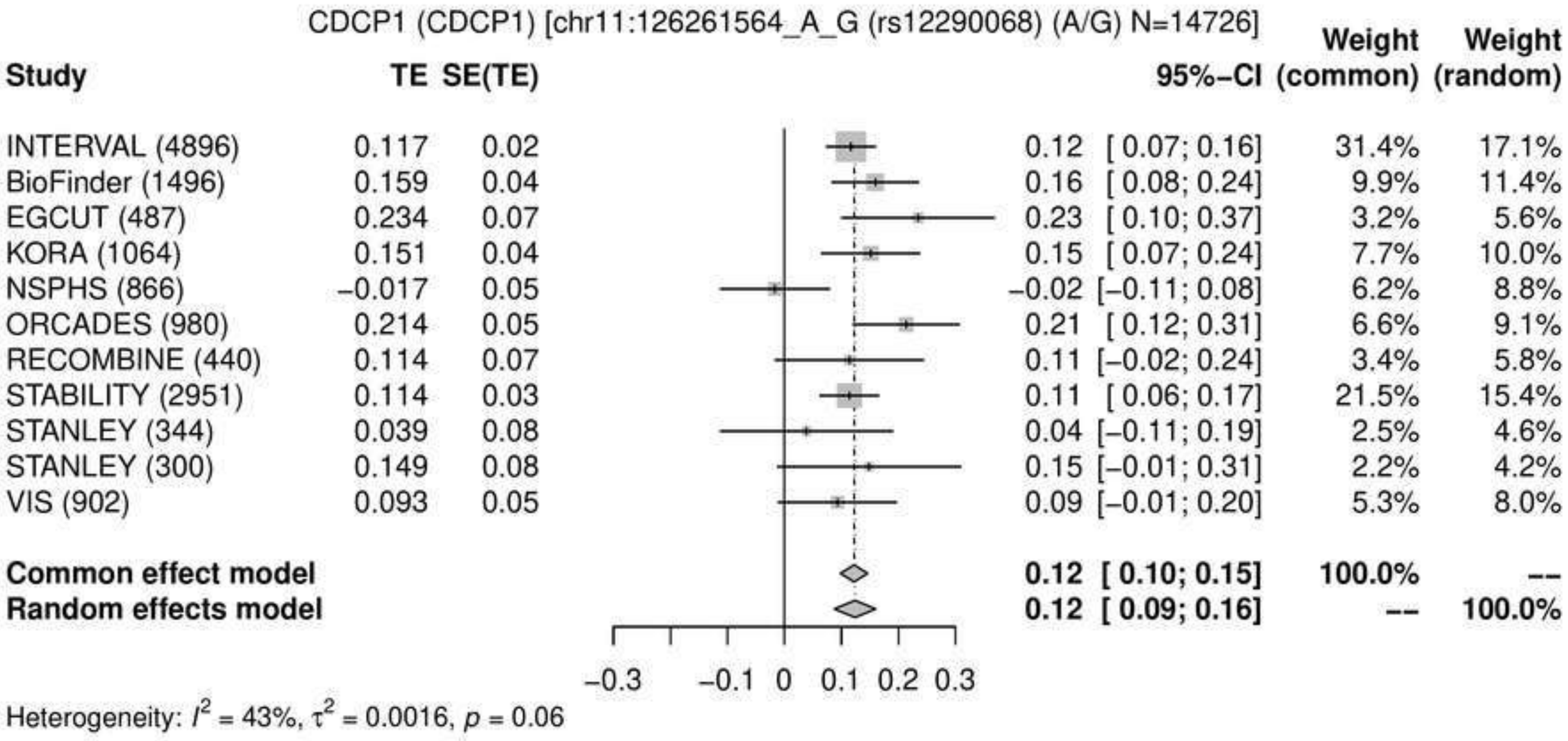
CDCP1 (CDCP1) [chr11:126261564_A_G (rs12290068) (A/G) N=14726]
Study TE SE(TE) 95%−CI Weight (common) Weight (random)
INTERVAL (4896) 0.117 0.02 0.12 [0.07; 0.16] 31.4% 17.1%
BioFinder (1496) 0.159 0.04 0.16 [0.08; 0.24] 9.9% 11.4%
EGCUT (487) 0.234 0.07 0.23 [0.10; 0.37] 3.2% 5.6%
KORA (1064) 0.151 0.04 0.15 [0.07; 0.24] 7.7% 10.0%
NSPHS (866) −0.017 0.05 −0.02 [−0.11; 0.08] 6.2% 8.8%
ORCADES (980) 0.214 0.05 0.21 [0.12; 0.31] 6.6% 9.1%
RECOMBINE (440) 0.114 0.07 0.11 [−0.02; 0.24] 3.4% 5.8%
STABILITY (2951) 0.114 0.03 0.11 [0.06; 0.17] 21.5% 15.4%
STANLEY (344) 0.039 0.08 0.04 [−0.11; 0.19] 2.5% 4.6%
STANLEY (300) 0.149 0.08 0.15 [−0.01; 0.31] 2.2% 4.2%
VIS (902) 0.093 0.05 0.09 [−0.01; 0.20] 5.3% 8.0%
Common effect model 0.12 [0.10; 0.15] 100.0% --
Random effects model 0.12 [0.09; 0.16] -- 100.0%
−0.3 −0.1 0 0.1 0.2 0.3
Heterogeneity: I^2 = 43%, τ^2 = 0.0016, p = 0.06

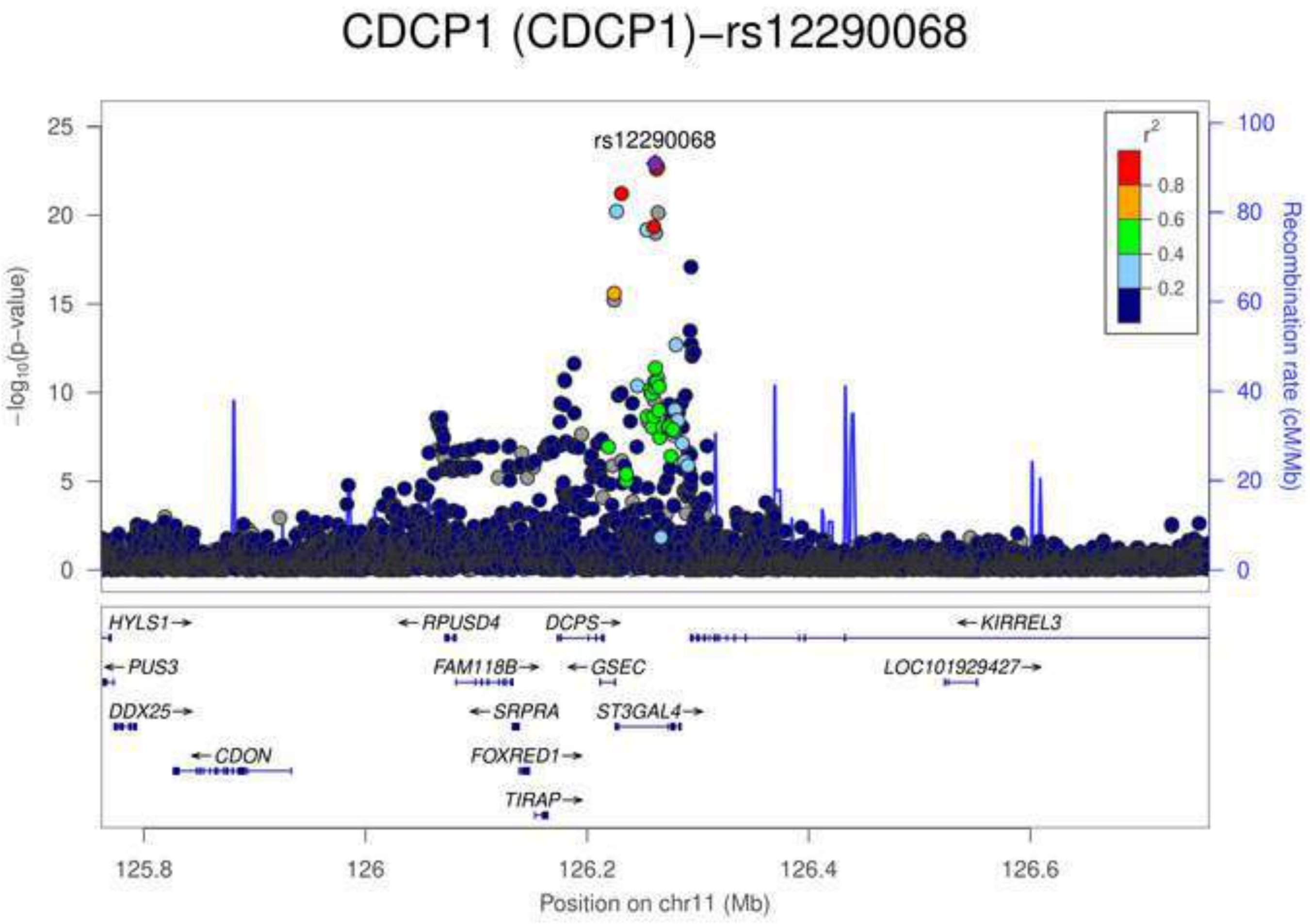
CDCP1 (CDCP1)−rs12290068
rs12290068
$-\log_{10}$(p-value)
Recombination rate (cM/Mb)
r^2
0.8
0.6
0.4
0.2
HYLS1
PUS3
DDX25
CDON
RPUSD4
FAM118B
SRPRA
FOXRED1
TIRAP
DCPS
GSEC
ST3GAL4
KIRREL3
LOC101929427
125.8 126 126.2 126.4 126.6
Position on chr11 (Mb)

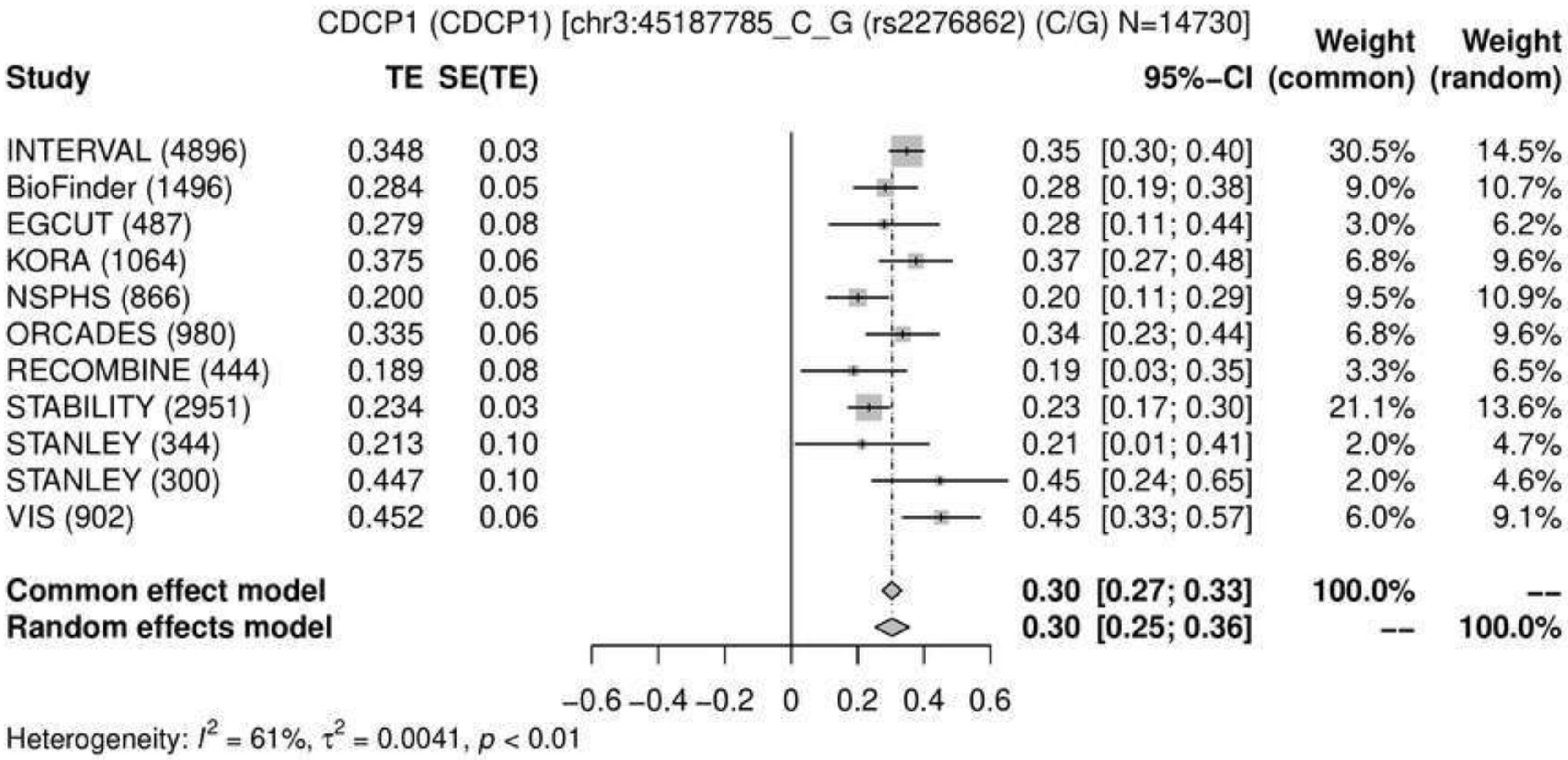
CDCP1 (CDCP1) [chr3:45187785_C_G (rs2276862) (C/G) N=14730]
Study | TE | SE(TE) | 95%-CI | Weight (common) | Weight (random)
INTERVAL (4896) | 0.348 | 0.03 | 0.35 [0.30; 0.40] | 30.5% | 14.5%
BioFinder (1496) | 0.284 | 0.05 | 0.28 [0.19; 0.38] | 9.0% | 10.7%
EGCUT (487) | 0.279 | 0.08 | 0.28 [0.11; 0.44] | 3.0% | 6.2%
KORA (1064) | 0.375 | 0.06 | 0.37 [0.27; 0.48] | 6.8% | 9.6%
NSPHS (866) | 0.200 | 0.05 | 0.20 [0.11; 0.29] | 9.5% | 10.9%
ORCADES (980) | 0.335 | 0.06 | 0.34 [0.23; 0.44] | 6.8% | 9.6%
RECOMBINE (444) | 0.189 | 0.08 | 0.19 [0.03; 0.35] | 3.3% | 6.5%
STABILITY (2951) | 0.234 | 0.03 | 0.23 [0.17; 0.30] | 21.1% | 13.6%
STANLEY (344) | 0.213 | 0.10 | 0.21 [0.01; 0.41] | 2.0% | 4.7%
STANLEY (300) | 0.447 | 0.10 | 0.45 [0.24; 0.65] | 2.0% | 4.6%
VIS (902) | 0.452 | 0.06 | 0.45 [0.33; 0.57] | 6.0% | 9.1%
Common effect model | 0.30 [0.27; 0.33] | 100.0% | --
Random effects model | 0.30 [0.25; 0.36] | -- | 100.0%
−0.6 −0.4 −0.2 0 0.2 0.4 0.6
Heterogeneity: I^2 = 61%, τ^2 = 0.0041, p < 0.01

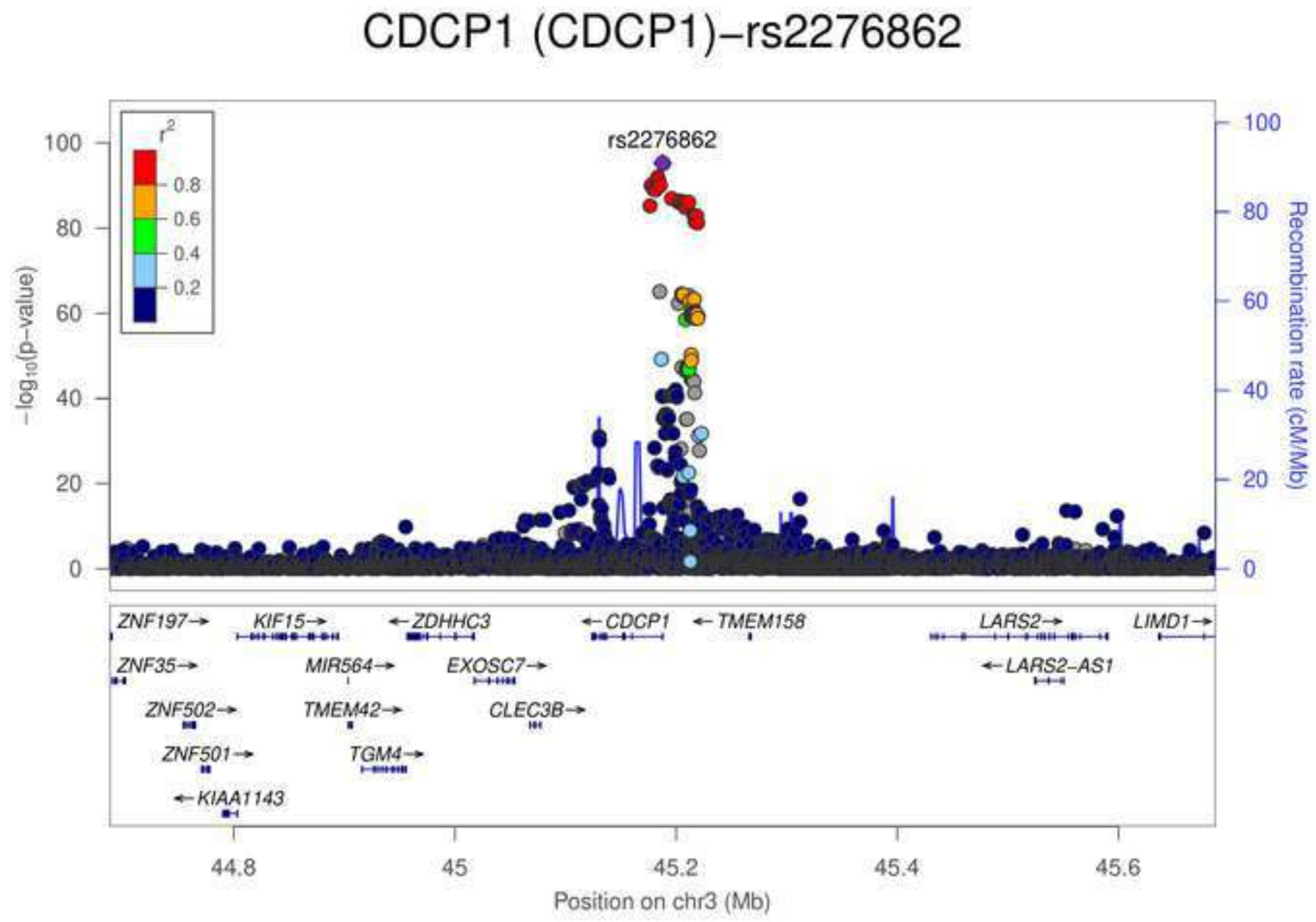
CDCP1 (CDCP1)−rs2276862
rs2276862
r^2
0.8
0.6
0.4
0.2
$-\log_{10}$(p−value)
Recombination rate (cM/Mb)
ZNF197→
KIF15→
←ZDHHC3
←CDCP1
←TMEM158
LARS2→
LIMD1→
ZNF35→
MIR564→
EXOSC7→
←LARS2-AS1
ZNF502→
TMEM42→
CLEC3B→
ZNF501→
TGM4→
←KIAA1143
44.8
45
45.2
45.4
45.6
Position on chr3 (Mb)

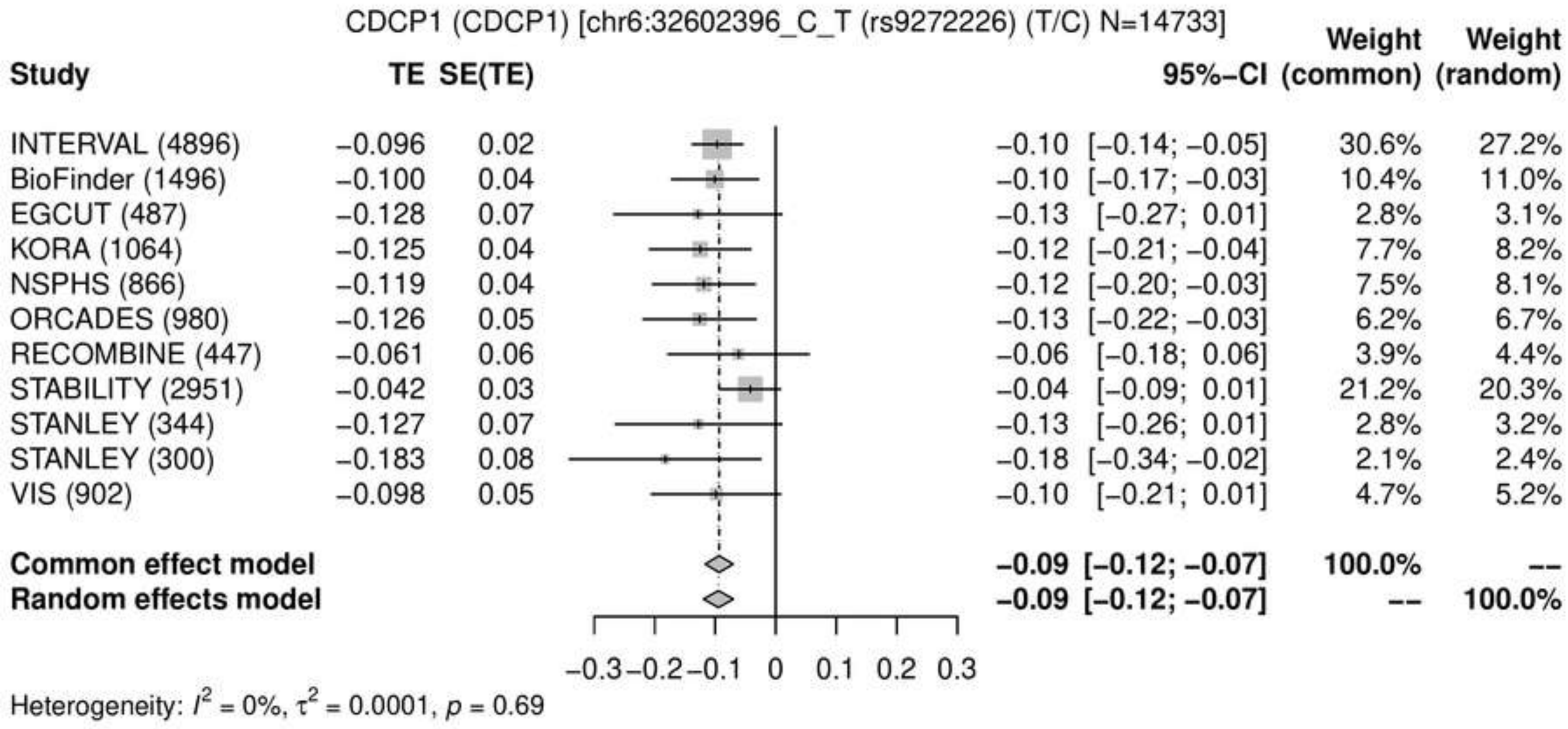
CDCP1 (CDCP1) [chr6:32602396_C_T (rs9272226) (T/C) N=14733]
Study
TE
SE(TE)
95%-CI
Weight (common)
Weight (random)
INTERVAL (4896) −0.096 0.02 −0.10 [−0.14; −0.05] 30.6% 27.2%
BioFinder (1496) −0.100 0.04 −0.10 [−0.17; −0.03] 10.4% 11.0%
EGCUT (487) −0.128 0.07 −0.13 [−0.27; 0.01] 2.8% 3.1%
KORA (1064) −0.125 0.04 −0.12 [−0.21; −0.04] 7.7% 8.2%
NSPHS (866) −0.119 0.04 −0.12 [−0.20; −0.03] 7.5% 8.1%
ORCADES (980) −0.126 0.05 −0.13 [−0.22; −0.03] 6.2% 6.7%
RECOMBINE (447) −0.061 0.06 −0.06 [−0.18; 0.06] 3.9% 4.4%
STABILITY (2951) −0.042 0.03 −0.04 [−0.09; 0.01] 21.2% 20.3%
STANLEY (344) −0.127 0.07 −0.13 [−0.26; 0.01] 2.8% 3.2%
STANLEY (300) −0.183 0.08 −0.18 [−0.34; −0.02] 2.1% 2.4%
VIS (902) −0.098 0.05 −0.10 [−0.21; 0.01] 4.7% 5.2%
Common effect model −0.09 [−0.12; −0.07] 100.0% --
Random effects model −0.09 [−0.12; −0.07] -- 100.0%
−0.3 −0.2 −0.1 0 0.1 0.2 0.3
Heterogeneity: I^2 = 0%, τ^2 = 0.0001, p = 0.69

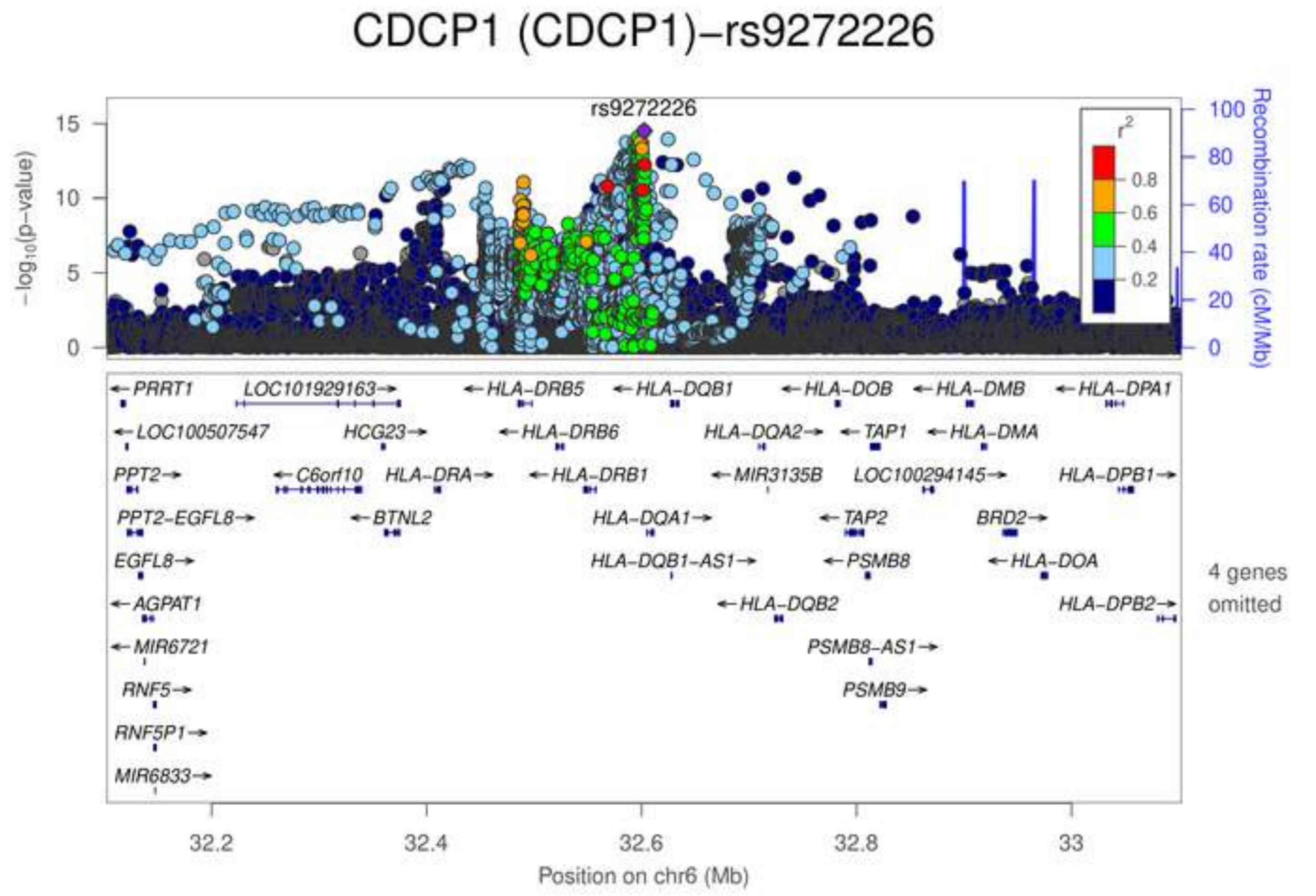
CDCP1 (CDCP1)−rs9272226
rs9272226
$-\log_{10}(p\text{-value})$
Recombination rate (cM/Mb)
r^2
0.8
0.6
0.4
0.2
PRRT1
LOC101929163
HLA−DRB5
HLA−DQB1
HLA−DOB
HLA−DMB
HLA−DPA1
LOC100507547
HCG23
HLA−DRB6
HLA−DQA2
TAP1
HLA−DMA
PPT2
C6orf10
HLA−DRA
HLA−DRB1
MIR3135B
LOC100294145
HLA−DPB1
PPT2−EGFL8
BTNL2
HLA−DQA1
TAP2
BRD2
EGFL8
HLA−DQB1−AS1
PSMB8
HLA−DOA
AGPAT1
HLA−DQB2
HLA−DPB2
MIR6721
PSMB8−AS1
RNF5
PSMB9
RNF5P1
MIR6833
4 genes omitted
32.2
32.4
32.6
32.8
33
Position on chr6 (Mb)

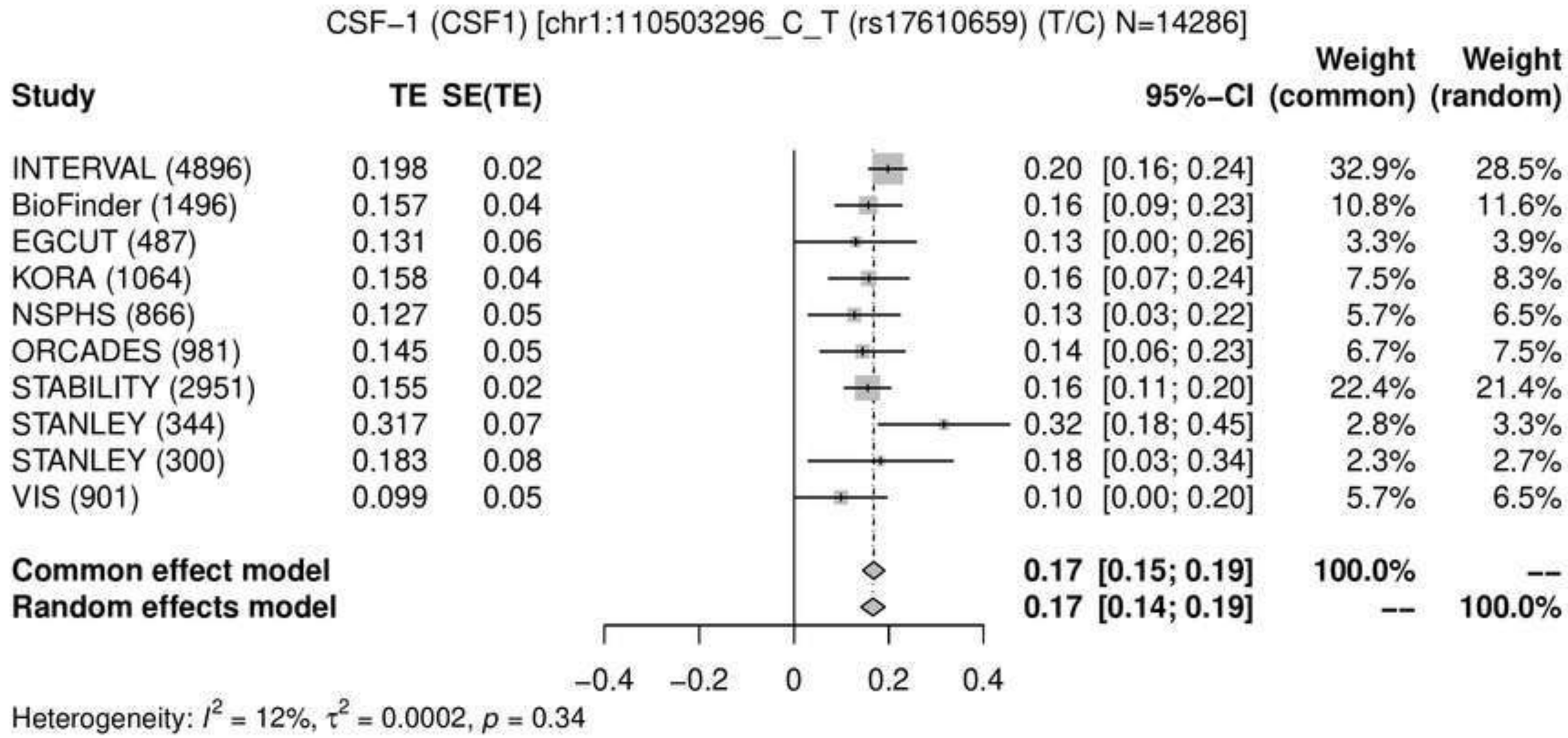
CSF−1 (CSF1) [chr1:110503296_C_T (rs17610659) (T/C) N=14286]
Study TE SE(TE) 95%−CI Weight (common) Weight (random)
INTERVAL (4896) 0.198 0.02 0.20 [0.16; 0.24] 32.9% 28.5%
BioFinder (1496) 0.157 0.04 0.16 [0.09; 0.23] 10.8% 11.6%
EGCUT (487) 0.131 0.06 0.13 [0.00; 0.26] 3.3% 3.9%
KORA (1064) 0.158 0.04 0.16 [0.07; 0.24] 7.5% 8.3%
NSPHS (866) 0.127 0.05 0.13 [0.03; 0.22] 5.7% 6.5%
ORCADES (981) 0.145 0.05 0.14 [0.06; 0.23] 6.7% 7.5%
STABILITY (2951) 0.155 0.02 0.16 [0.11; 0.20] 22.4% 21.4%
STANLEY (344) 0.317 0.07 0.32 [0.18; 0.45] 2.8% 3.3%
STANLEY (300) 0.183 0.08 0.18 [0.03; 0.34] 2.3% 2.7%
VIS (901) 0.099 0.05 0.10 [0.00; 0.20] 5.7% 6.5%
Common effect model 0.17 [0.15; 0.19] 100.0% --
Random effects model 0.17 [0.14; 0.19] -- 100.0%
−0.4 −0.2 0 0.2 0.4
Heterogeneity: I^2 = 12%, τ^2 = 0.0002, p = 0.34

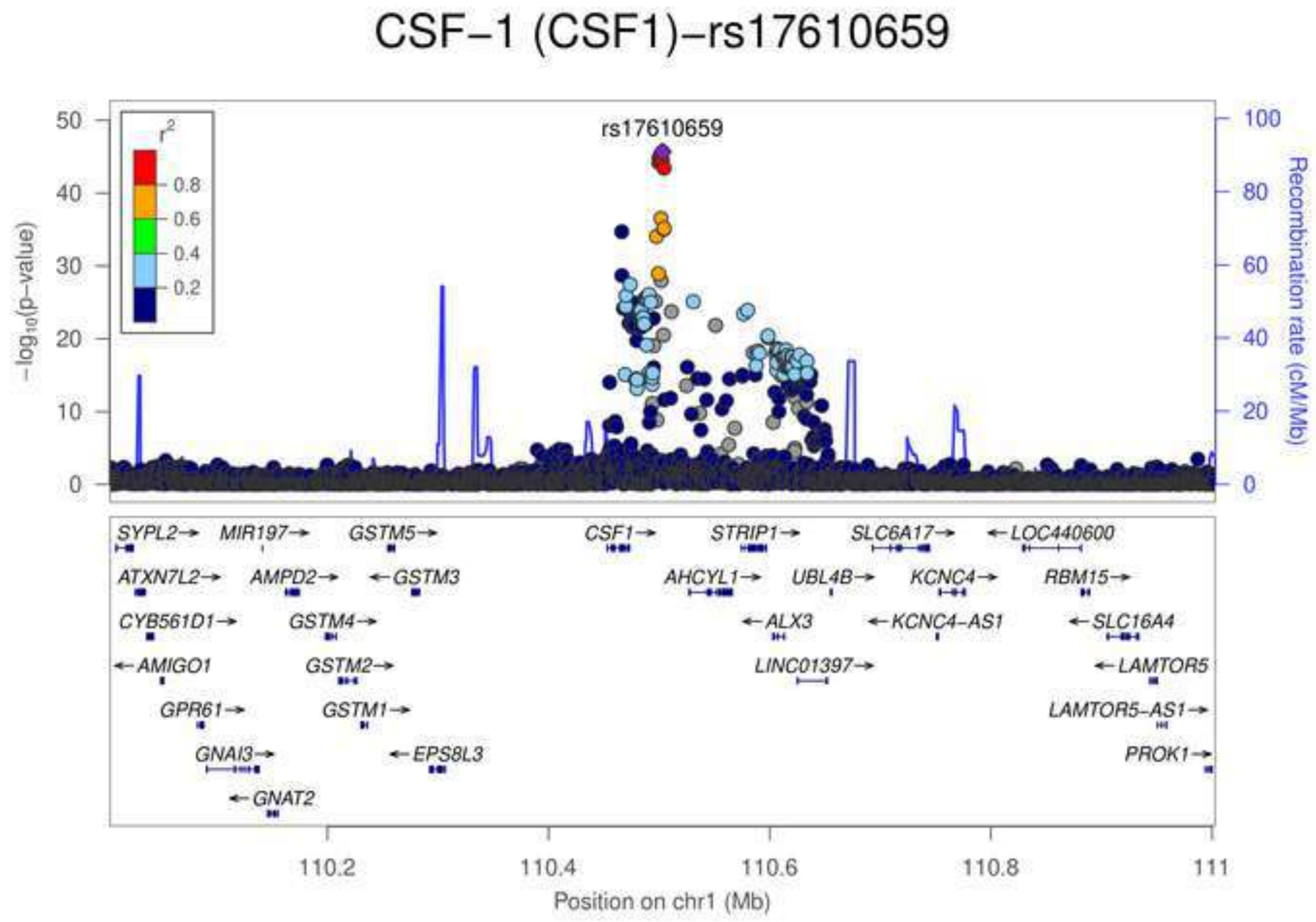
CSF−1 (CSF1)−rs17610659
rs17610659
$-\log_{10}$(p−value)
Recombination rate (cM/Mb)
r^2
0.8
0.6
0.4
0.2
SYPL2→ MIR197→ GSTM5→ CSF1→ STRIP1→ SLC6A17→ ←LOC440600
ATXN7L2→ AMPD2→ ←GSTM3 AHCYL1→ UBL4B→ KCNC4→ RBM15→
CYB561D1→ GSTM4→ ←ALX3 ←KCNC4−AS1 ←SLC16A4
←AMIGO1 GSTM2→ LINC01397→ ←LAMTOR5
GPR61→ GSTM1→ LAMTOR5−AS1→
GNAI3→ ←EPS8L3 PROK1→
←GNAT2
110.2 110.4 110.6 110.8 111
Position on chr1 (Mb)

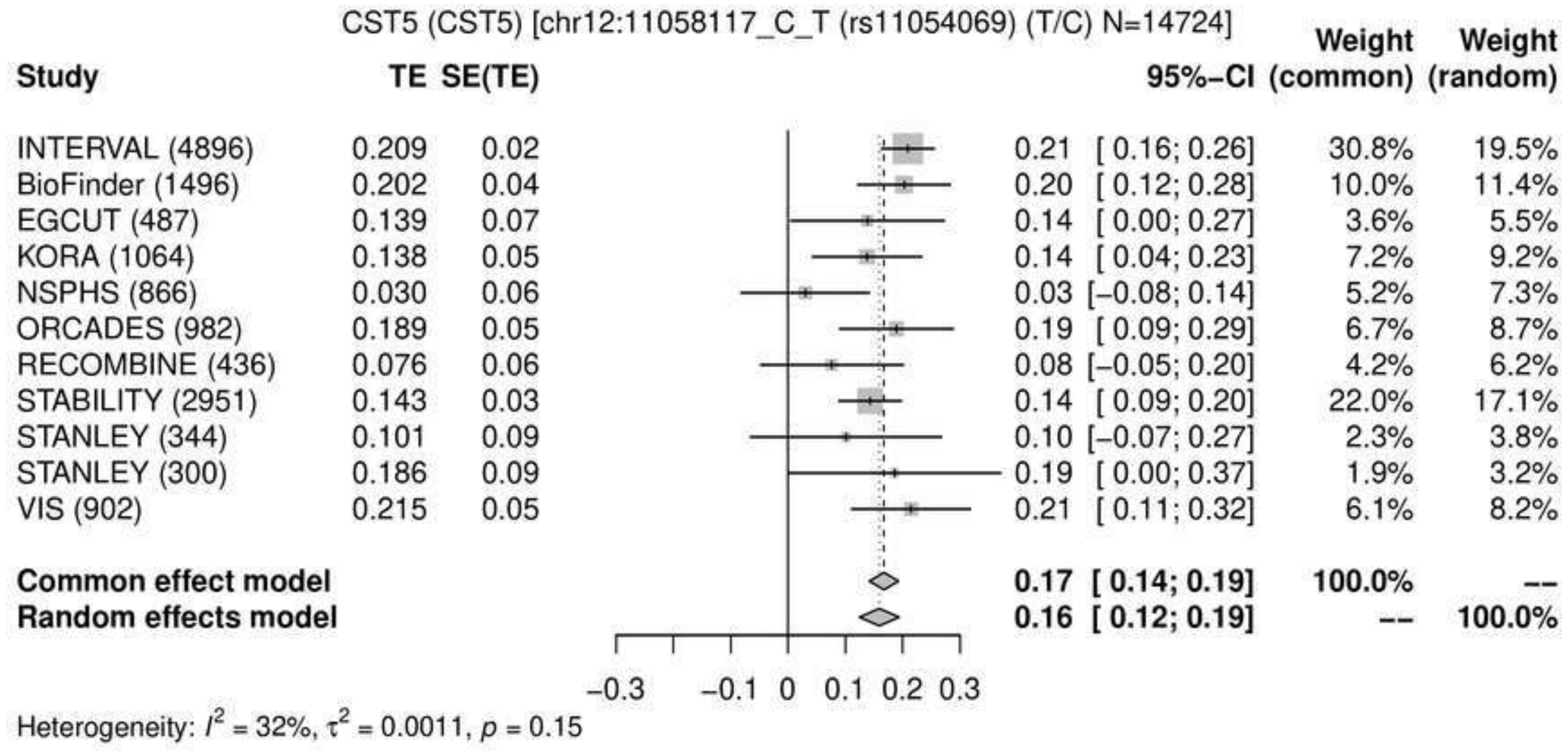

CST5 (CST5) [chr12:11058117_C_T (rs11054069) (T/C) N=14724]
Study
TE
SE(TE)
95%-CI
Weight (common)
Weight (random)
INTERVAL (4896) 0.209 0.02 0.21 [0.16; 0.26] 30.8% 19.5%
BioFinder (1496) 0.202 0.04 0.20 [0.12; 0.28] 10.0% 11.4%
EGCUT (487) 0.139 0.07 0.14 [0.00; 0.27] 3.6% 5.5%
KORA (1064) 0.138 0.05 0.14 [0.04; 0.23] 7.2% 9.2%
NSPHS (866) 0.030 0.06 0.03 [−0.08; 0.14] 5.2% 7.3%
ORCADES (982) 0.189 0.05 0.19 [0.09; 0.29] 6.7% 8.7%
RECOMBINE (436) 0.076 0.06 0.08 [−0.05; 0.20] 4.2% 6.2%
STABILITY (2951) 0.143 0.03 0.14 [0.09; 0.20] 22.0% 17.1%
STANLEY (344) 0.101 0.09 0.10 [−0.07; 0.27] 2.3% 3.8%
STANLEY (300) 0.186 0.09 0.19 [0.00; 0.37] 1.9% 3.2%
VIS (902) 0.215 0.05 0.21 [0.11; 0.32] 6.1% 8.2%
Common effect model 0.17 [0.14; 0.19] 100.0% --
Random effects model 0.16 [0.12; 0.19] -- 100.0%
−0.3 −0.1 0 0.1 0.2 0.3
Heterogeneity: I^2 = 32%, τ^2 = 0.0011, p = 0.15

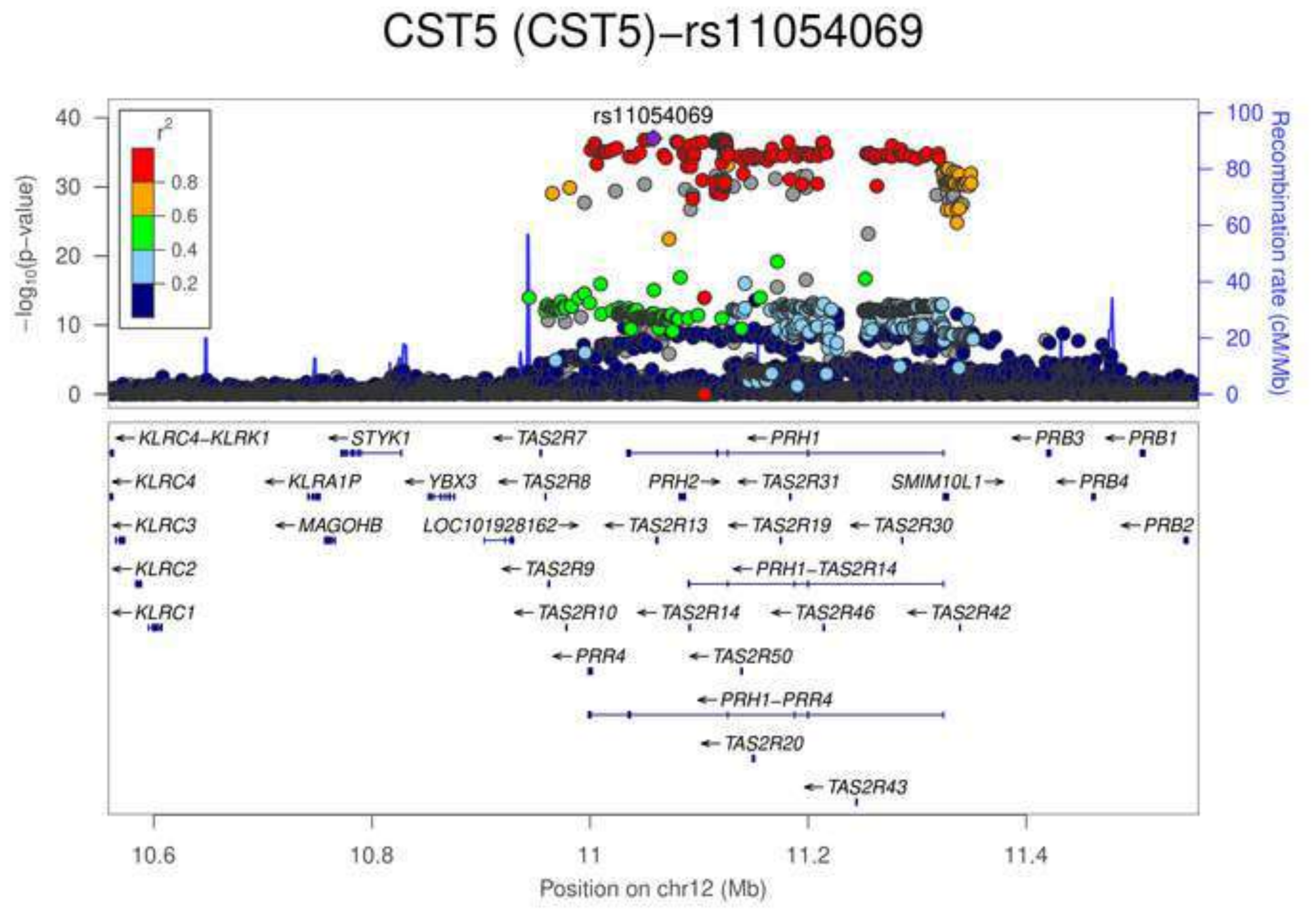

CST5 (CST5)−rs11054069
rs11054069
$-\log_{10}$(p-value)
Recombination rate (cM/Mb)
r^2
0.8
0.6
0.4
0.2
KLRC4−KLRK1
STYK1
TAS2R7
PRH1
PRB3
PRB1
KLRC4
KLRA1P
YBX3
TAS2R8
PRH2
TAS2R31
SMIM10L1
PRB4
KLRC3
MAGOHB
LOC101928162
TAS2R13
TAS2R19
TAS2R30
PRB2
KLRC2
TAS2R9
PRH1−TAS2R14
KLRC1
TAS2R10
TAS2R14
TAS2R46
TAS2R42
PRR4
TAS2R50
PRH1−PRR4
TAS2R20
TAS2R43
10.6
10.8
11
11.2
11.4
Position on chr12 (Mb)

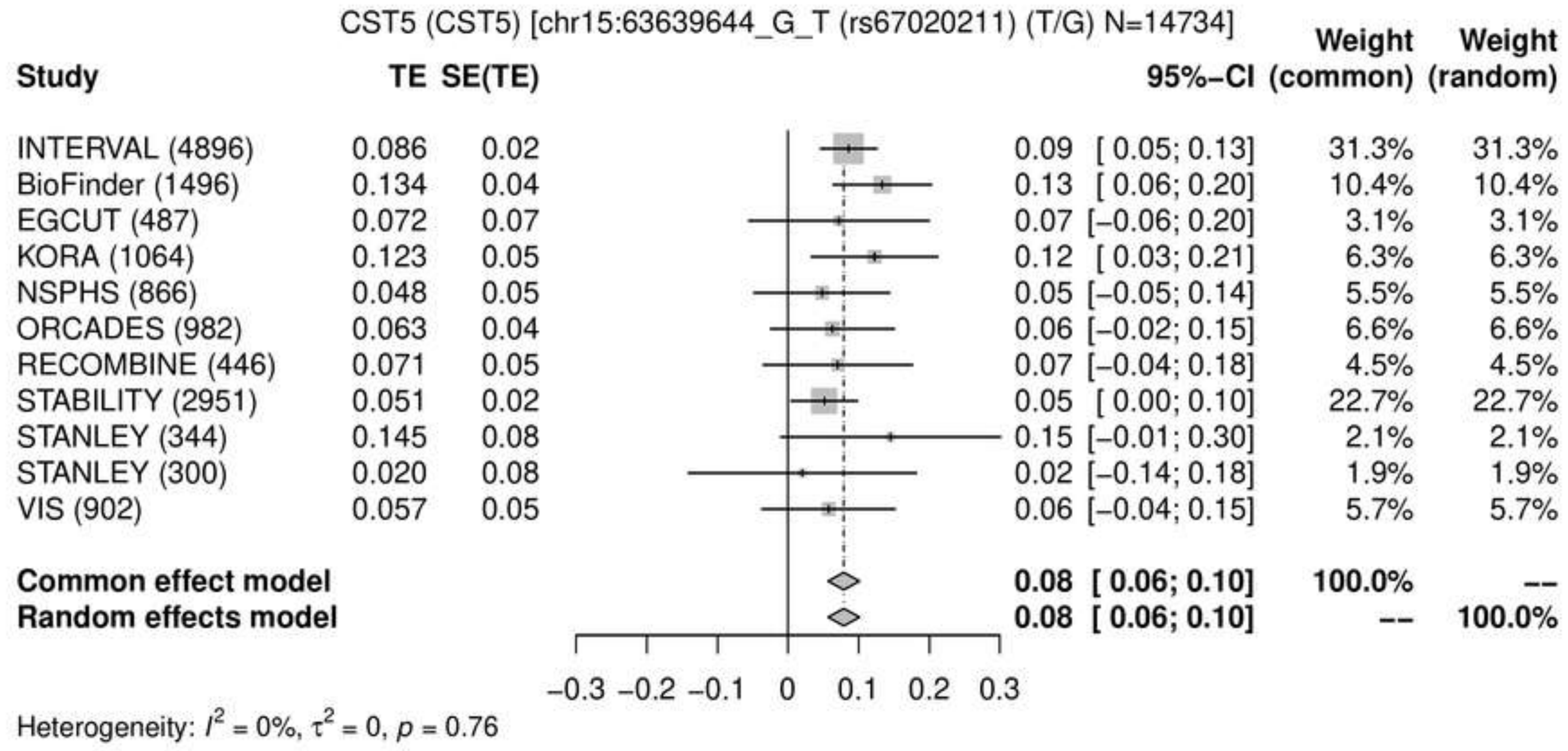
CST5 (CST5) [chr15:63639644_G_T (rs67020211) (T/G) N=14734]
Study
TE
SE(TE)
95%-CI
Weight (common)
Weight (random)
INTERVAL (4896) 0.086 0.02 0.09 [0.05; 0.13] 31.3% 31.3%
BioFinder (1496) 0.134 0.04 0.13 [0.06; 0.20] 10.4% 10.4%
EGCUT (487) 0.072 0.07 0.07 [−0.06; 0.20] 3.1% 3.1%
KORA (1064) 0.123 0.05 0.12 [0.03; 0.21] 6.3% 6.3%
NSPHS (866) 0.048 0.05 0.05 [−0.05; 0.14] 5.5% 5.5%
ORCADES (982) 0.063 0.04 0.06 [−0.02; 0.15] 6.6% 6.6%
RECOMBINE (446) 0.071 0.05 0.07 [−0.04; 0.18] 4.5% 4.5%
STABILITY (2951) 0.051 0.02 0.05 [0.00; 0.10] 22.7% 22.7%
STANLEY (344) 0.145 0.08 0.15 [−0.01; 0.30] 2.1% 2.1%
STANLEY (300) 0.020 0.08 0.02 [−0.14; 0.18] 1.9% 1.9%
VIS (902) 0.057 0.05 0.06 [−0.04; 0.15] 5.7% 5.7%
Common effect model 0.08 [0.06; 0.10] 100.0% --
Random effects model 0.08 [0.06; 0.10] -- 100.0%
−0.3 −0.2 −0.1 0 0.1 0.2 0.3
Heterogeneity: I^2 = 0%, τ^2 = 0, p = 0.76

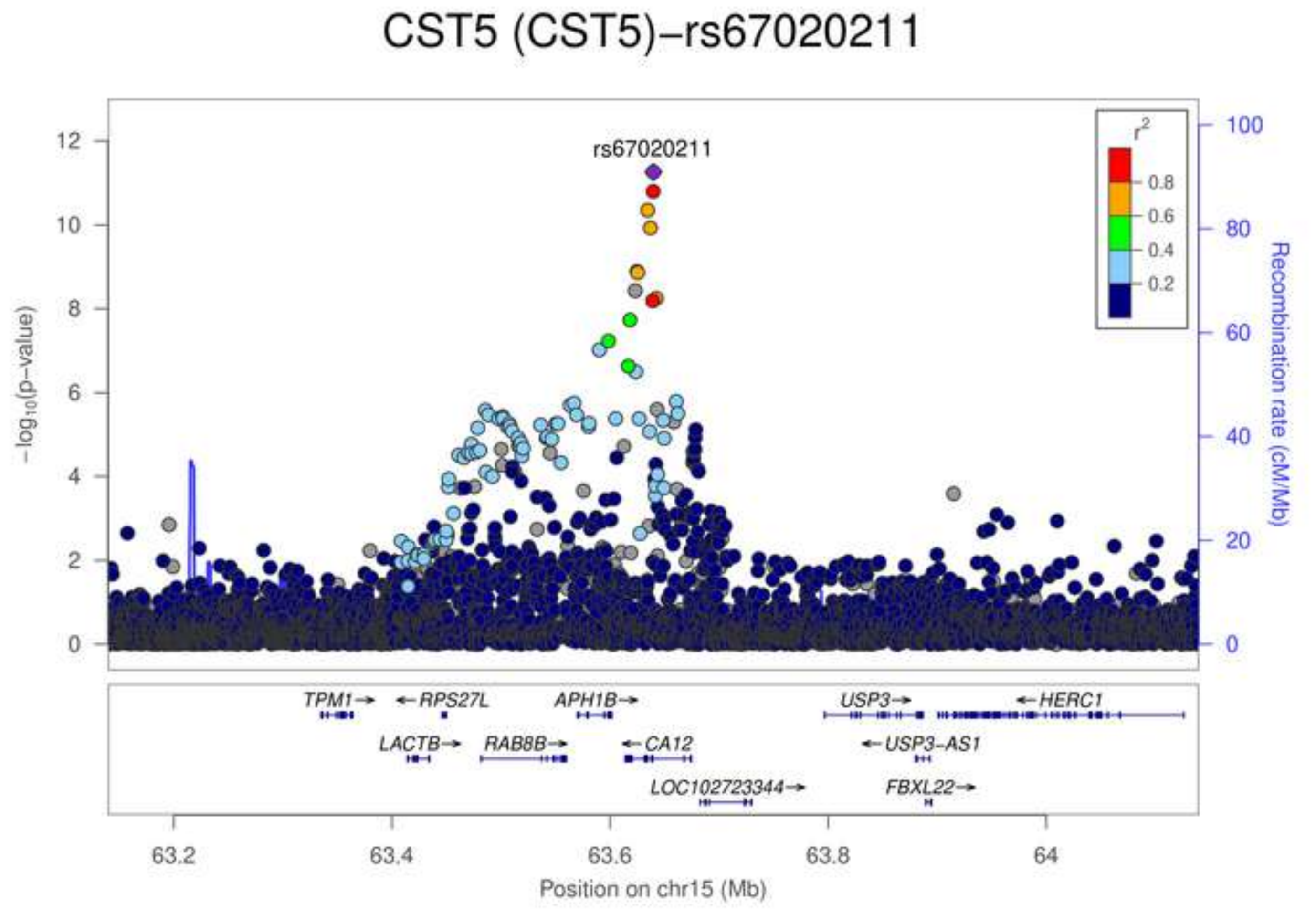
CST5 (CST5)−rs67020211
rs67020211
$-\log_{10}$(p-value)
Recombination rate (cM/Mb)
r^2
0.8
0.6
0.4
0.2
TPM1→
←RPS27L
APH1B→
USP3→
←HERC1
LACTB→
RAB8B→
←CA12
←USP3-AS1
LOC102723344→
FBXL22→
63.2
63.4
63.6
63.8
64
Position on chr15 (Mb)

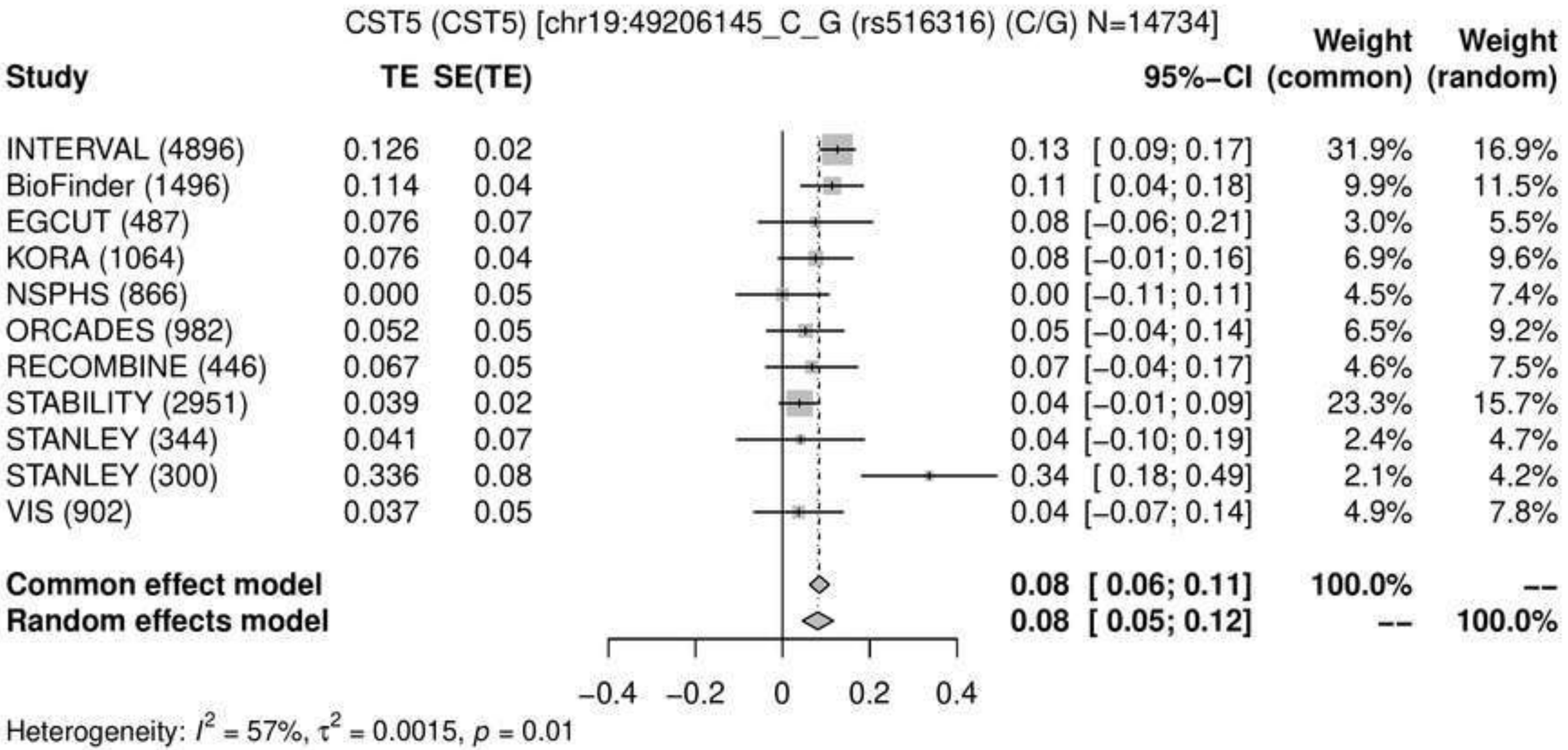
CST5 (CST5) [chr19:49206145_C_G (rs516316) (C/G) N=14734]
Study
TE
SE(TE)
95%-CI
Weight (common)
Weight (random)
INTERVAL (4896) 0.126 0.02 0.13 [0.09; 0.17] 31.9% 16.9%
BioFinder (1496) 0.114 0.04 0.11 [0.04; 0.18] 9.9% 11.5%
EGCUT (487) 0.076 0.07 0.08 [−0.06; 0.21] 3.0% 5.5%
KORA (1064) 0.076 0.04 0.08 [−0.01; 0.16] 6.9% 9.6%
NSPHS (866) 0.000 0.05 0.00 [−0.11; 0.11] 4.5% 7.4%
ORCADES (982) 0.052 0.05 0.05 [−0.04; 0.14] 6.5% 9.2%
RECOMBINE (446) 0.067 0.05 0.07 [−0.04; 0.17] 4.6% 7.5%
STABILITY (2951) 0.039 0.02 0.04 [−0.01; 0.09] 23.3% 15.7%
STANLEY (344) 0.041 0.07 0.04 [−0.10; 0.19] 2.4% 4.7%
STANLEY (300) 0.336 0.08 0.34 [0.18; 0.49] 2.1% 4.2%
VIS (902) 0.037 0.05 0.04 [−0.07; 0.14] 4.9% 7.8%
Common effect model 0.08 [0.06; 0.11] 100.0% --
Random effects model 0.08 [0.05; 0.12] -- 100.0%
−0.4 −0.2 0 0.2 0.4
Heterogeneity: I^2 = 57%, τ^2 = 0.0015, p = 0.01

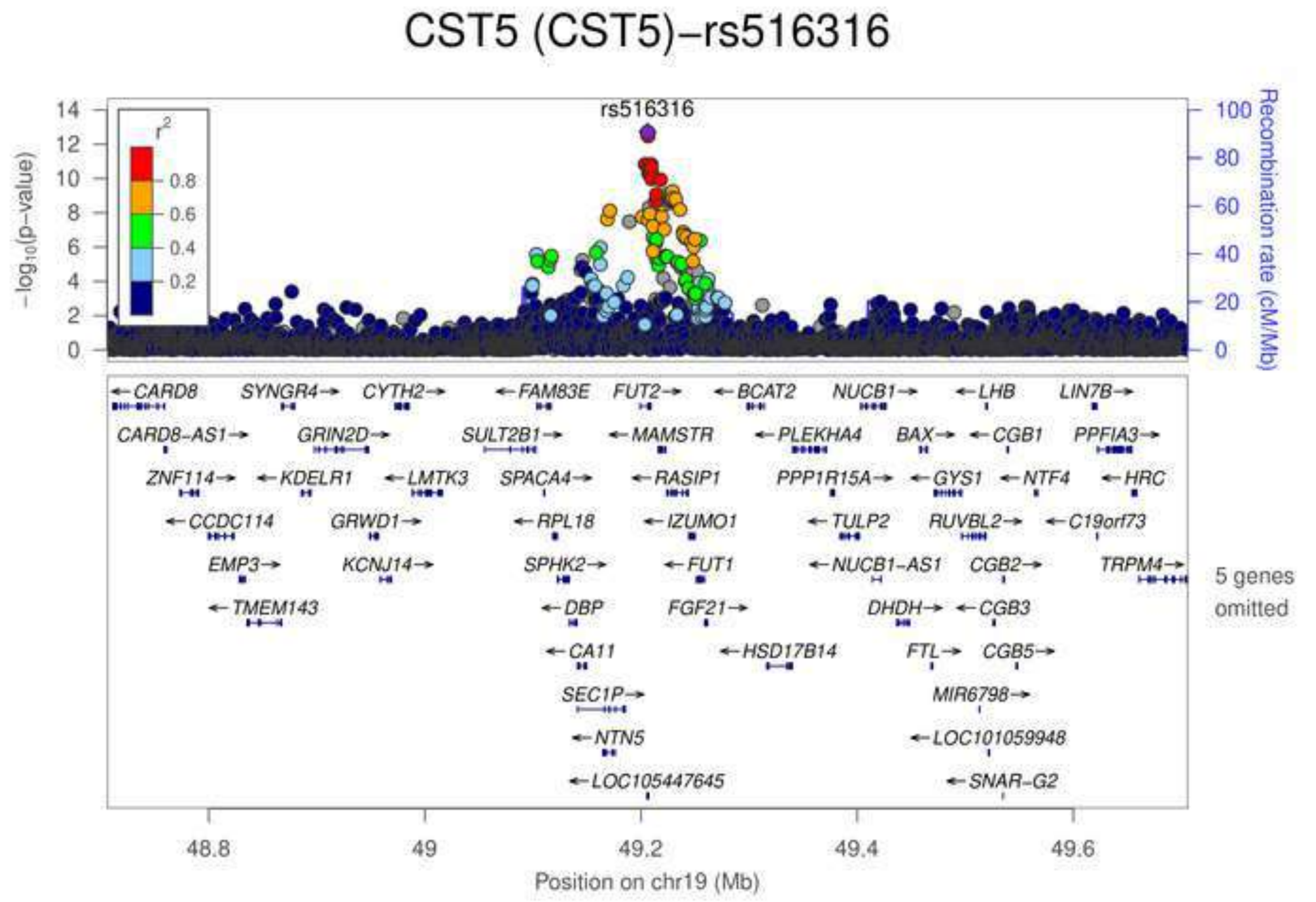
CST5 (CST5)−rs516316
rs516316
r^2
0.8
0.6
0.4
0.2
$-\log_{10}$(p−value)
Recombination rate (cM/Mb)
CARD8
SYNGR4
CYTH2
FAM83E
FUT2
BCAT2
NUCB1
LHB
LIN7B
CARD8−AS1
GRIN2D
SULT2B1
MAMSTR
PLEKHA4
BAX
CGB1
PPFIA3
ZNF114
KDELR1
LMTK3
SPACA4
RASIP1
PPP1R15A
GYS1
NTF4
HRC
CCDC114
GRWD1
RPL18
IZUMO1
TULP2
RUVBL2
C19orf73
EMP3
KCNJ14
SPHK2
FUT1
NUCB1−AS1
CGB2
TRPM4
5 genes omitted
TMEM143
DBP
FGF21
DHDH
CGB3
CA11
HSD17B14
FTL
CGB5
SEC1P
MIR6798
NTN5
LOC101059948
LOC105447645
SNAR−G2
48.8
49
49.2
49.4
49.6
Position on chr19 (Mb)

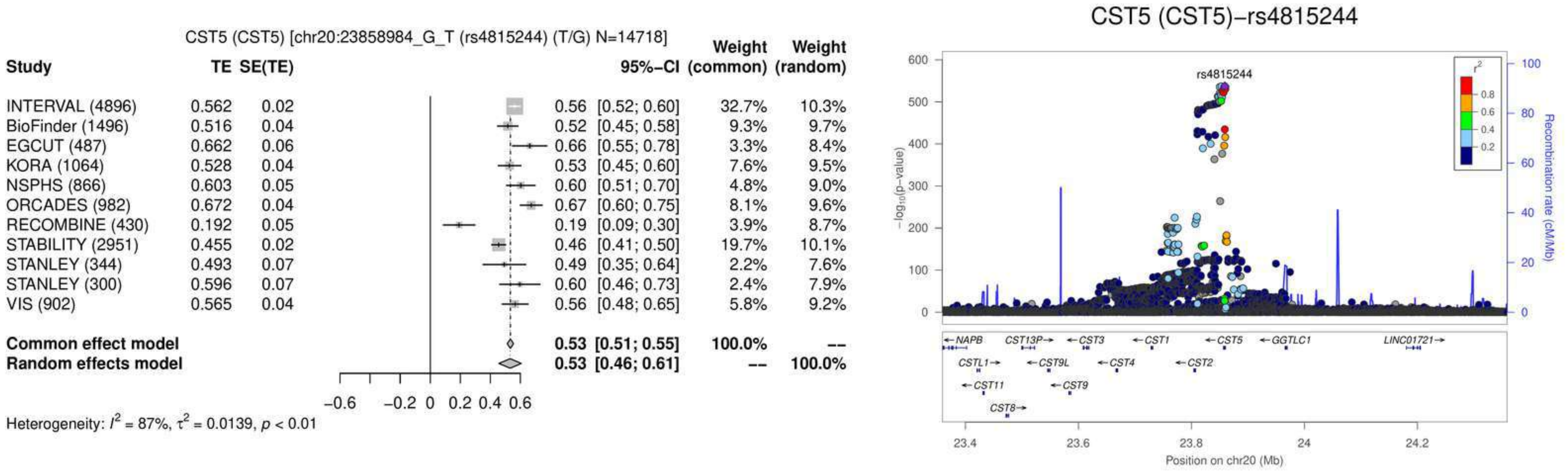
CST5 (CST5) [chr20:23858984_G_T (rs4815244) (T/G) N=14718]
Study
TE
SE(TE)
95%−CI
Weight (common)
Weight (random)
INTERVAL (4896) 0.562 0.02 0.56 [0.52; 0.60] 32.7% 10.3%
BioFinder (1496) 0.516 0.04 0.52 [0.45; 0.58] 9.3% 9.7%
EGCUT (487) 0.662 0.06 0.66 [0.55; 0.78] 3.3% 8.4%
KORA (1064) 0.528 0.04 0.53 [0.45; 0.60] 7.6% 9.5%
NSPHS (866) 0.603 0.05 0.60 [0.51; 0.70] 4.8% 9.0%
ORCADES (982) 0.672 0.04 0.67 [0.60; 0.75] 8.1% 9.6%
RECOMBINE (430) 0.192 0.05 0.19 [0.09; 0.30] 3.9% 8.7%
STABILITY (2951) 0.455 0.02 0.46 [0.41; 0.50] 19.7% 10.1%
STANLEY (344) 0.493 0.07 0.49 [0.35; 0.64] 2.2% 7.6%
STANLEY (300) 0.596 0.07 0.60 [0.46; 0.73] 2.4% 7.9%
VIS (902) 0.565 0.04 0.56 [0.48; 0.65] 5.8% 9.2%
Common effect model 0.53 [0.51; 0.55] 100.0% --
Random effects model 0.53 [0.46; 0.61] -- 100.0%
−0.6 −0.2 0 0.2 0.4 0.6
Heterogeneity: I^2 = 87%, τ^2 = 0.0139, p < 0.01
CST5 (CST5)−rs4815244
rs4815244
$-\log_{10}$(p−value)
Recombination rate (cM/Mb)
r^2
0.8
0.6
0.4
0.2
NAPB
CST13P
CST3
CST1
CST5
GGTLC1
LINC01721
CSTL1
CST9L
CST4
CST2
CST11
CST9
CST8
23.4 23.6 23.8 24 24.2
Position on chr20 (Mb)

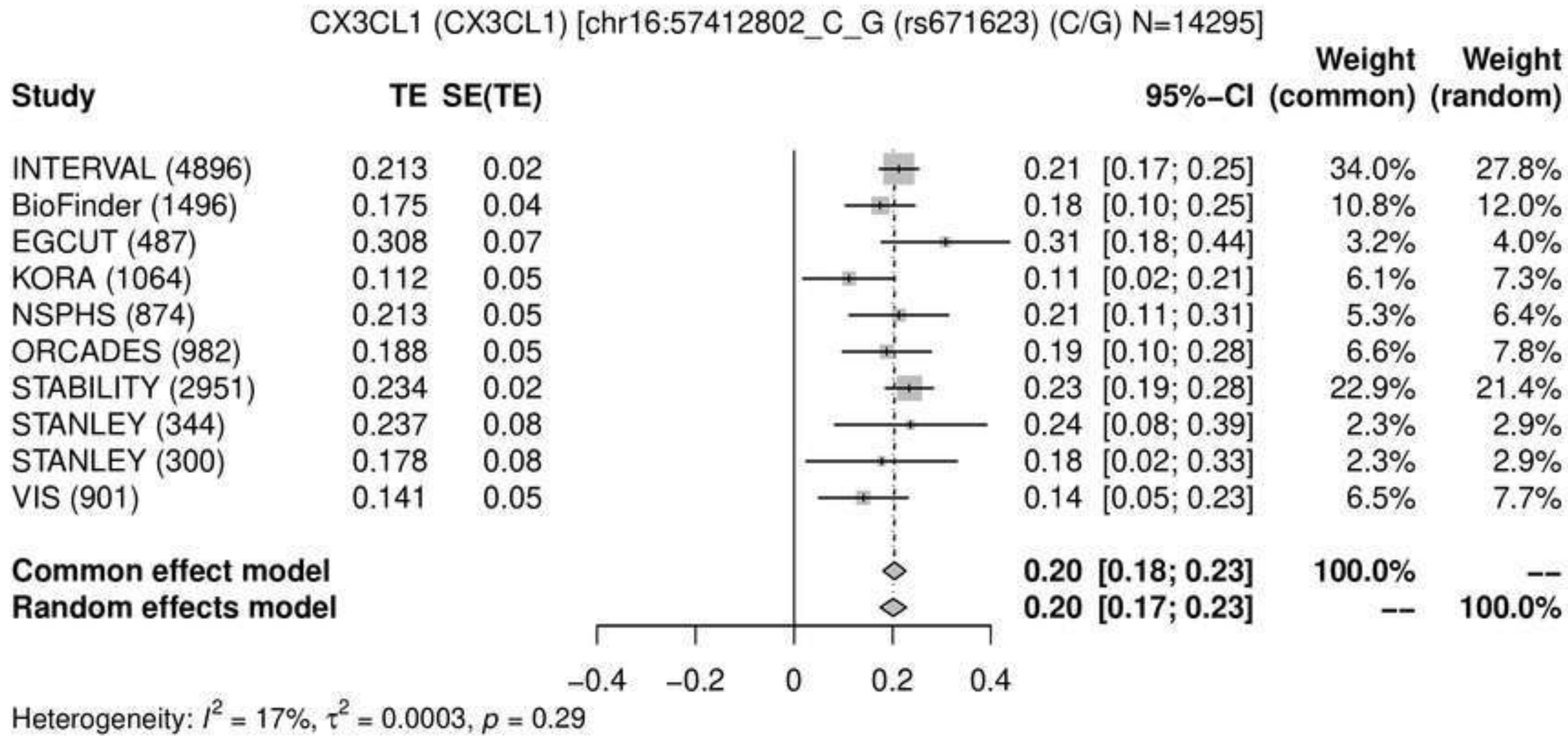
CX3CL1 (CX3CL1) [chr16:57412802_C_G (rs671623) (C/G) N=14295]
Study
TE
SE(TE)
95%−CI
Weight (common)
Weight (random)
INTERVAL (4896) 0.213 0.02 0.21 [0.17; 0.25] 34.0% 27.8%
BioFinder (1496) 0.175 0.04 0.18 [0.10; 0.25] 10.8% 12.0%
EGCUT (487) 0.308 0.07 0.31 [0.18; 0.44] 3.2% 4.0%
KORA (1064) 0.112 0.05 0.11 [0.02; 0.21] 6.1% 7.3%
NSPHS (874) 0.213 0.05 0.21 [0.11; 0.31] 5.3% 6.4%
ORCADES (982) 0.188 0.05 0.19 [0.10; 0.28] 6.6% 7.8%
STABILITY (2951) 0.234 0.02 0.23 [0.19; 0.28] 22.9% 21.4%
STANLEY (344) 0.237 0.08 0.24 [0.08; 0.39] 2.3% 2.9%
STANLEY (300) 0.178 0.08 0.18 [0.02; 0.33] 2.3% 2.9%
VIS (901) 0.141 0.05 0.14 [0.05; 0.23] 6.5% 7.7%
Common effect model 0.20 [0.18; 0.23] 100.0% --
Random effects model 0.20 [0.17; 0.23] -- 100.0%
−0.4 −0.2 0 0.2 0.4
Heterogeneity: I^2 = 17%, τ^2 = 0.0003, p = 0.29

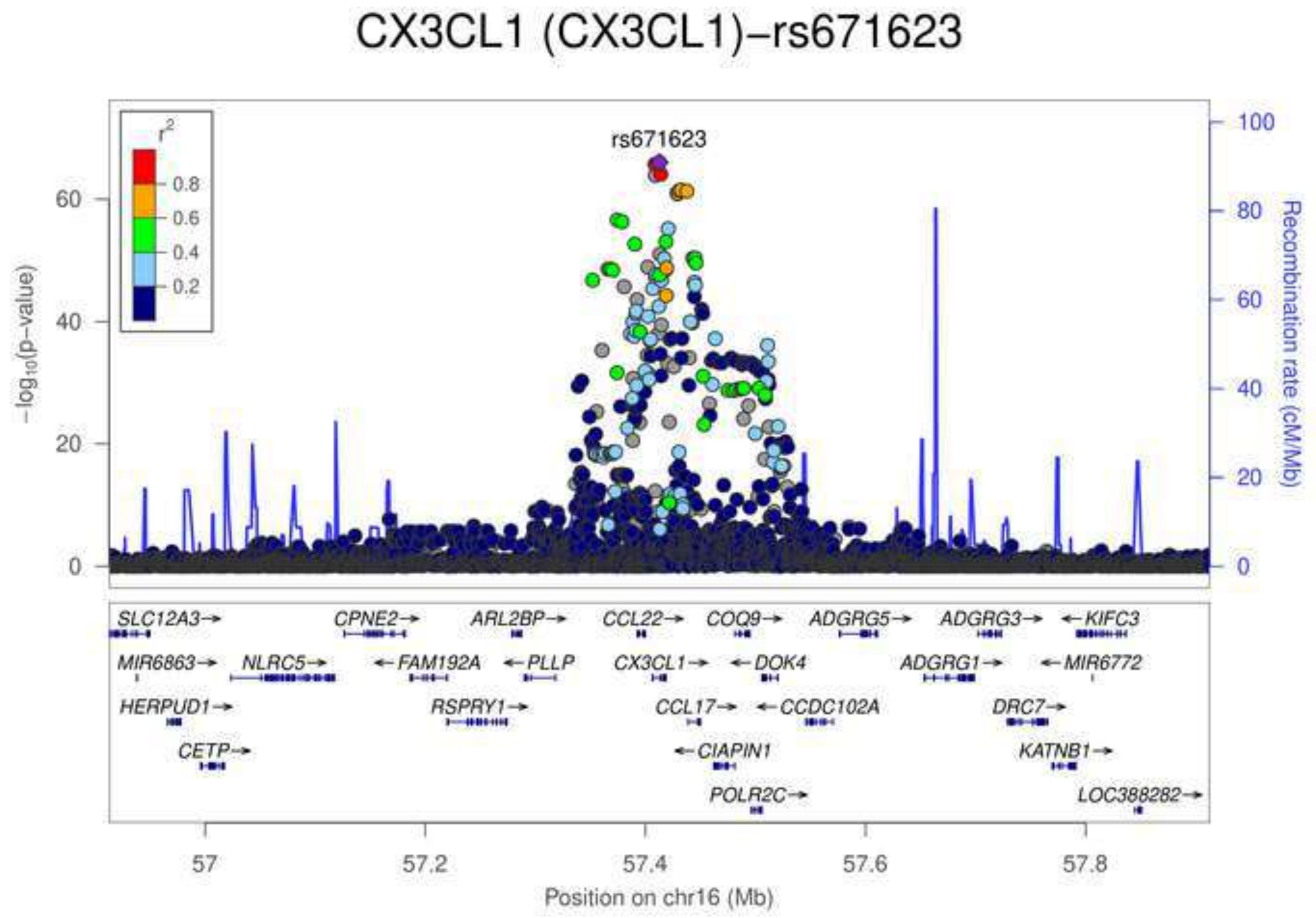
CX3CL1 (CX3CL1)−rs671623
rs671623
r^2
0.8
0.6
0.4
0.2
−log10(p−value)
Recombination rate (cM/Mb)
0
20
40
60
80
100
SLC12A3→
CPNE2→
ARL2BP→
CCL22→
COQ9→
ADGRG5→
ADGRG3→
←KIFC3
MIR6863→
NLRC5→
←FAM192A
←PLLP
CX3CL1→
←DOK4
ADGRG1→
←MIR6772
HERPUD1→
RSPRY1→
CCL17→
←CCDC102A
DRC7→
CETP→
←CIAPIN1
KATNB1→
POLR2C→
LOC388282→
57
57.2
57.4
57.6
57.8
Position on chr16 (Mb)

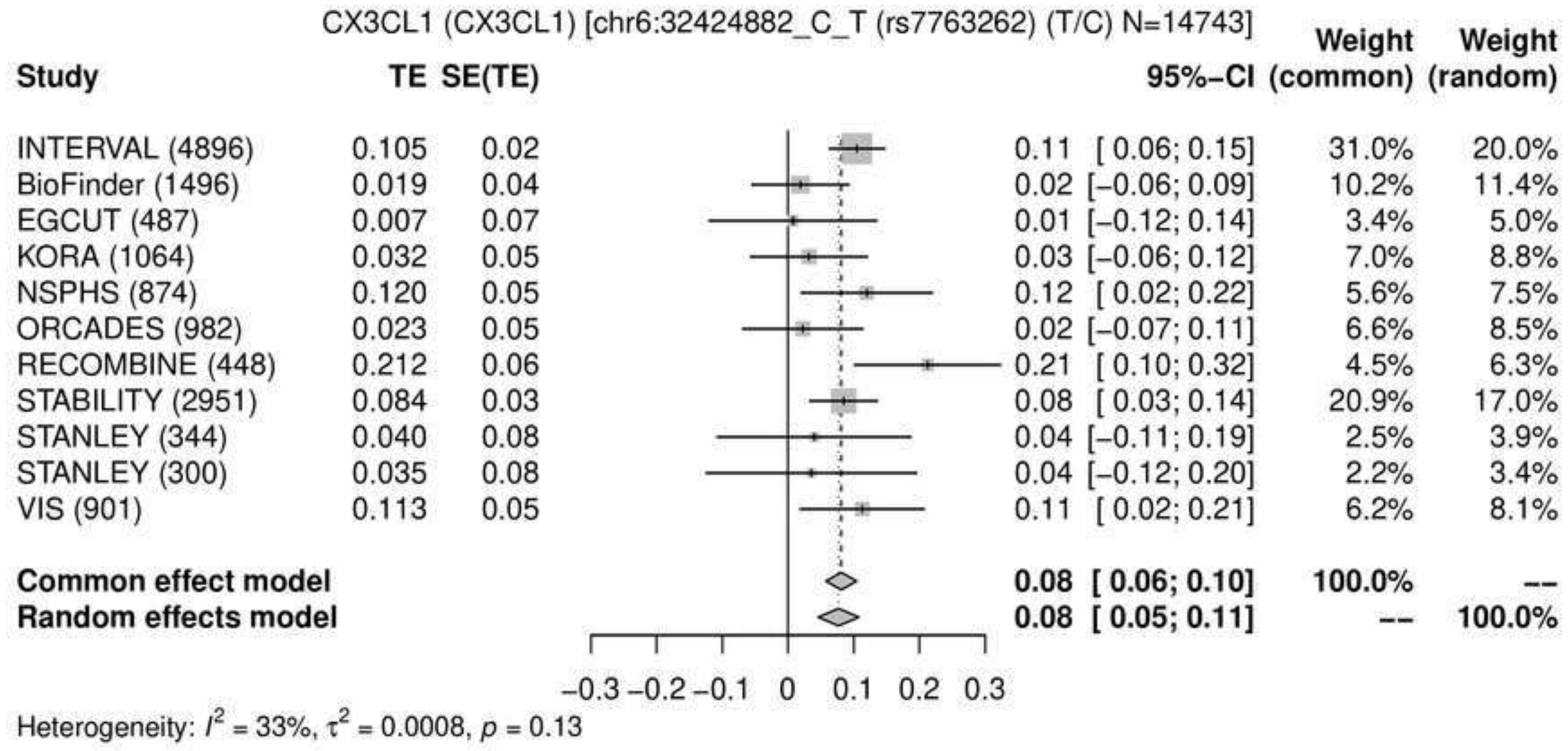
CX3CL1 (CX3CL1) [chr6:32424882_C_T (rs7763262) (T/C) N=14743]
Study
TE
SE(TE)
95%-CI
Weight (common)
Weight (random)
INTERVAL (4896) 0.105 0.02 0.11 [0.06; 0.15] 31.0% 20.0%
BioFinder (1496) 0.019 0.04 0.02 [−0.06; 0.09] 10.2% 11.4%
EGCUT (487) 0.007 0.07 0.01 [−0.12; 0.14] 3.4% 5.0%
KORA (1064) 0.032 0.05 0.03 [−0.06; 0.12] 7.0% 8.8%
NSPHS (874) 0.120 0.05 0.12 [0.02; 0.22] 5.6% 7.5%
ORCADES (982) 0.023 0.05 0.02 [−0.07; 0.11] 6.6% 8.5%
RECOMBINE (448) 0.212 0.06 0.21 [0.10; 0.32] 4.5% 6.3%
STABILITY (2951) 0.084 0.03 0.08 [0.03; 0.14] 20.9% 17.0%
STANLEY (344) 0.040 0.08 0.04 [−0.11; 0.19] 2.5% 3.9%
STANLEY (300) 0.035 0.08 0.04 [−0.12; 0.20] 2.2% 3.4%
VIS (901) 0.113 0.05 0.11 [0.02; 0.21] 6.2% 8.1%
Common effect model 0.08 [0.06; 0.10] 100.0% --
Random effects model 0.08 [0.05; 0.11] -- 100.0%
−0.3 −0.2 −0.1 0 0.1 0.2 0.3
Heterogeneity: I^2 = 33%, τ^2 = 0.0008, p = 0.13

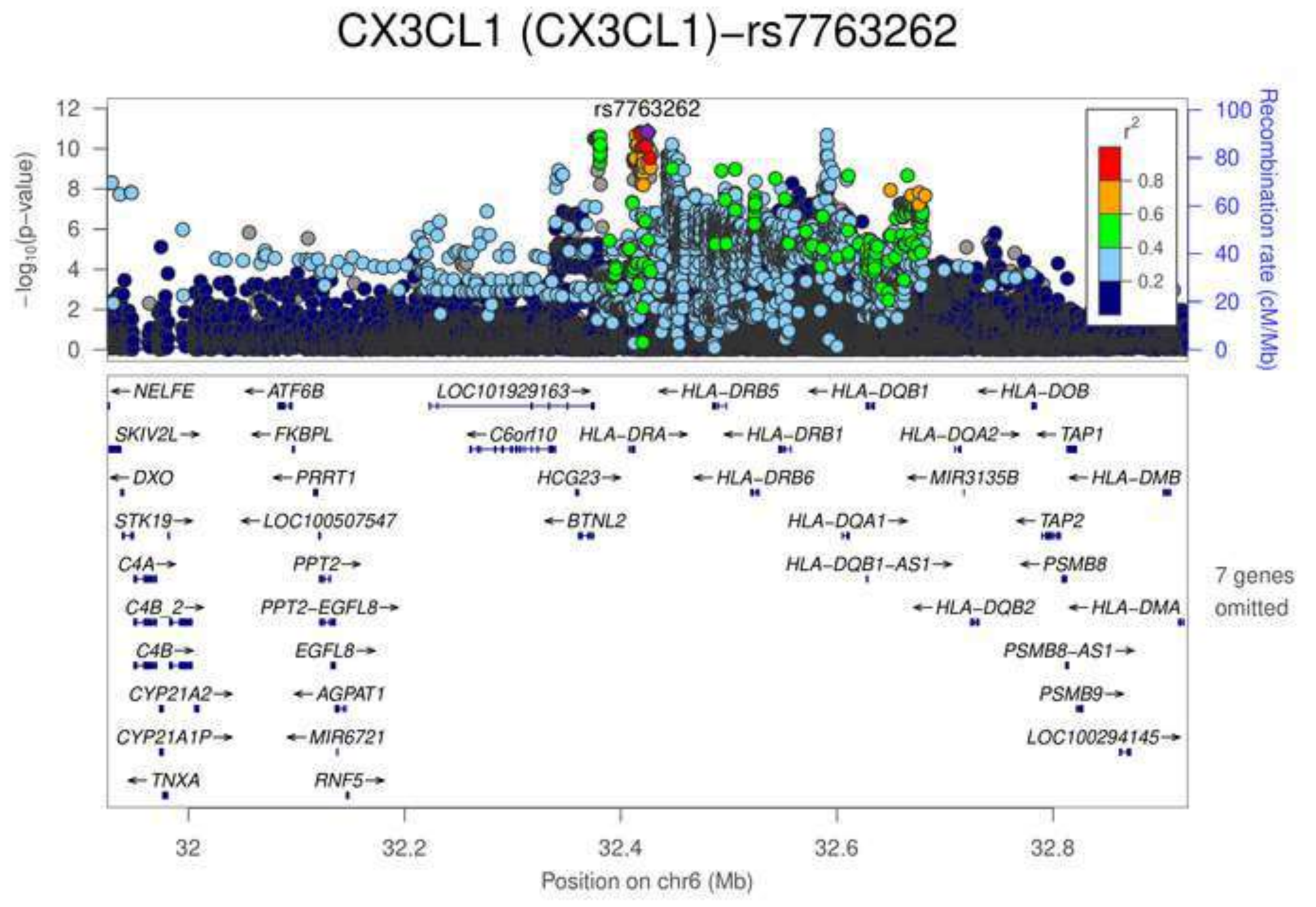
CX3CL1 (CX3CL1)−rs7763262
rs7763262
−log10(p−value)
Recombination rate (cM/Mb)
r^2
0.8
0.6
0.4
0.2
NELFE
ATF6B
LOC101929163
HLA-DRB5
HLA-DQB1
HLA-DOB
SKIV2L
FKBPL
C6orf10
HLA-DRA
HLA-DRB1
HLA-DQA2
TAP1
DXO
PRRT1
HCG23
HLA-DRB6
MIR3135B
HLA-DMB
STK19
LOC100507547
BTNL2
HLA-DQA1
TAP2
C4A
PPT2
HLA-DQB1-AS1
PSMB8
C4B_2
PPT2-EGFL8
HLA-DQB2
HLA-DMA
C4B
EGFL8
PSMB8-AS1
CYP21A2
AGPAT1
PSMB9
CYP21A1P
MIR6721
LOC100294145
TNXA
RNF5
7 genes omitted
32
32.2
32.4
32.6
32.8
Position on chr6 (Mb)

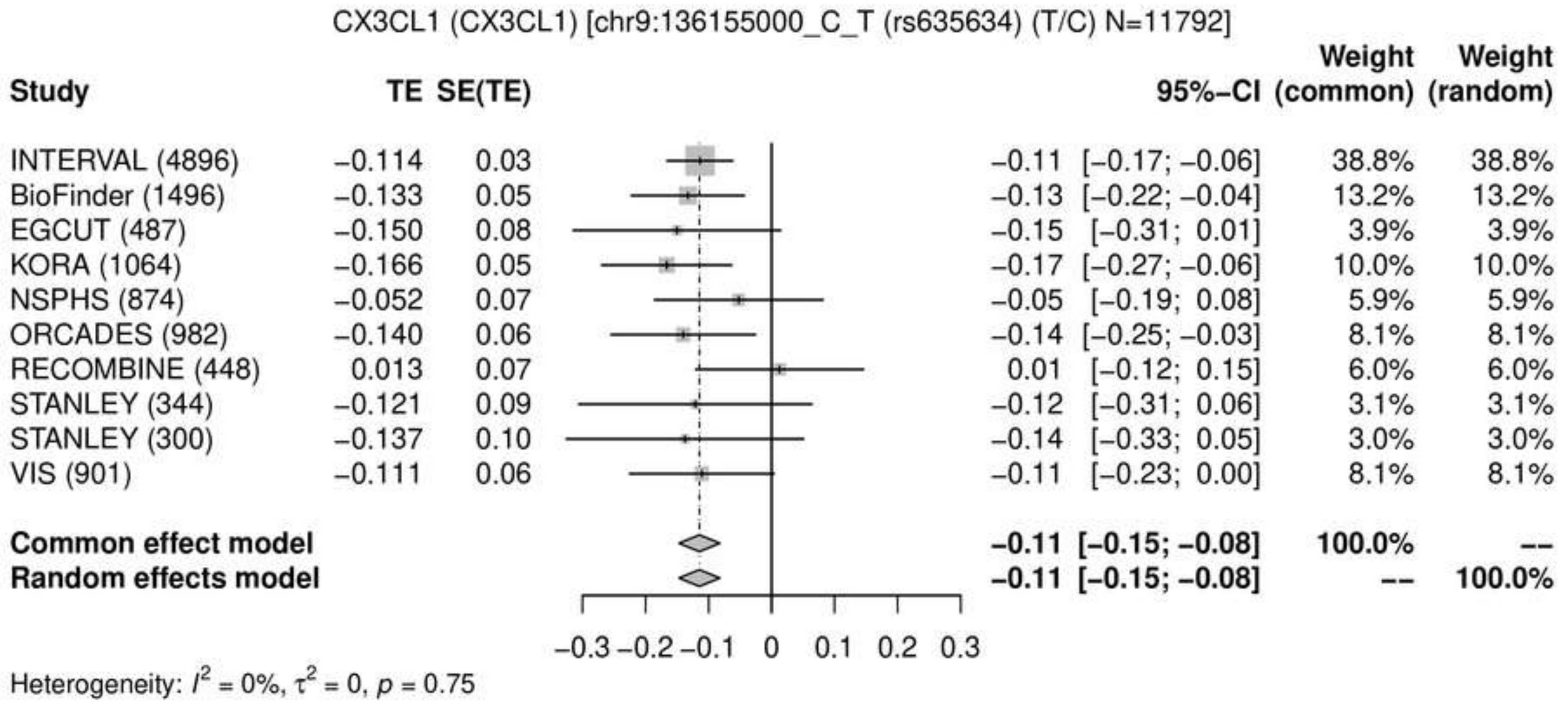
CX3CL1 (CX3CL1) [chr9:136155000_C_T (rs635634) (T/C) N=11792]
Study TE SE(TE) 95%−CI Weight (common) Weight (random)
INTERVAL (4896) −0.114 0.03 −0.11 [−0.17; −0.06] 38.8% 38.8%
BioFinder (1496) −0.133 0.05 −0.13 [−0.22; −0.04] 13.2% 13.2%
EGCUT (487) −0.150 0.08 −0.15 [−0.31; 0.01] 3.9% 3.9%
KORA (1064) −0.166 0.05 −0.17 [−0.27; −0.06] 10.0% 10.0%
NSPHS (874) −0.052 0.07 −0.05 [−0.19; 0.08] 5.9% 5.9%
ORCADES (982) −0.140 0.06 −0.14 [−0.25; −0.03] 8.1% 8.1%
RECOMBINE (448) 0.013 0.07 0.01 [−0.12; 0.15] 6.0% 6.0%
STANLEY (344) −0.121 0.09 −0.12 [−0.31; 0.06] 3.1% 3.1%
STANLEY (300) −0.137 0.10 −0.14 [−0.33; 0.05] 3.0% 3.0%
VIS (901) −0.111 0.06 −0.11 [−0.23; 0.00] 8.1% 8.1%
Common effect model −0.11 [−0.15; −0.08] 100.0% --
Random effects model −0.11 [−0.15; −0.08] -- 100.0%
−0.3 −0.2 −0.1 0 0.1 0.2 0.3
Heterogeneity: I^2 = 0%, τ^2 = 0, p = 0.75

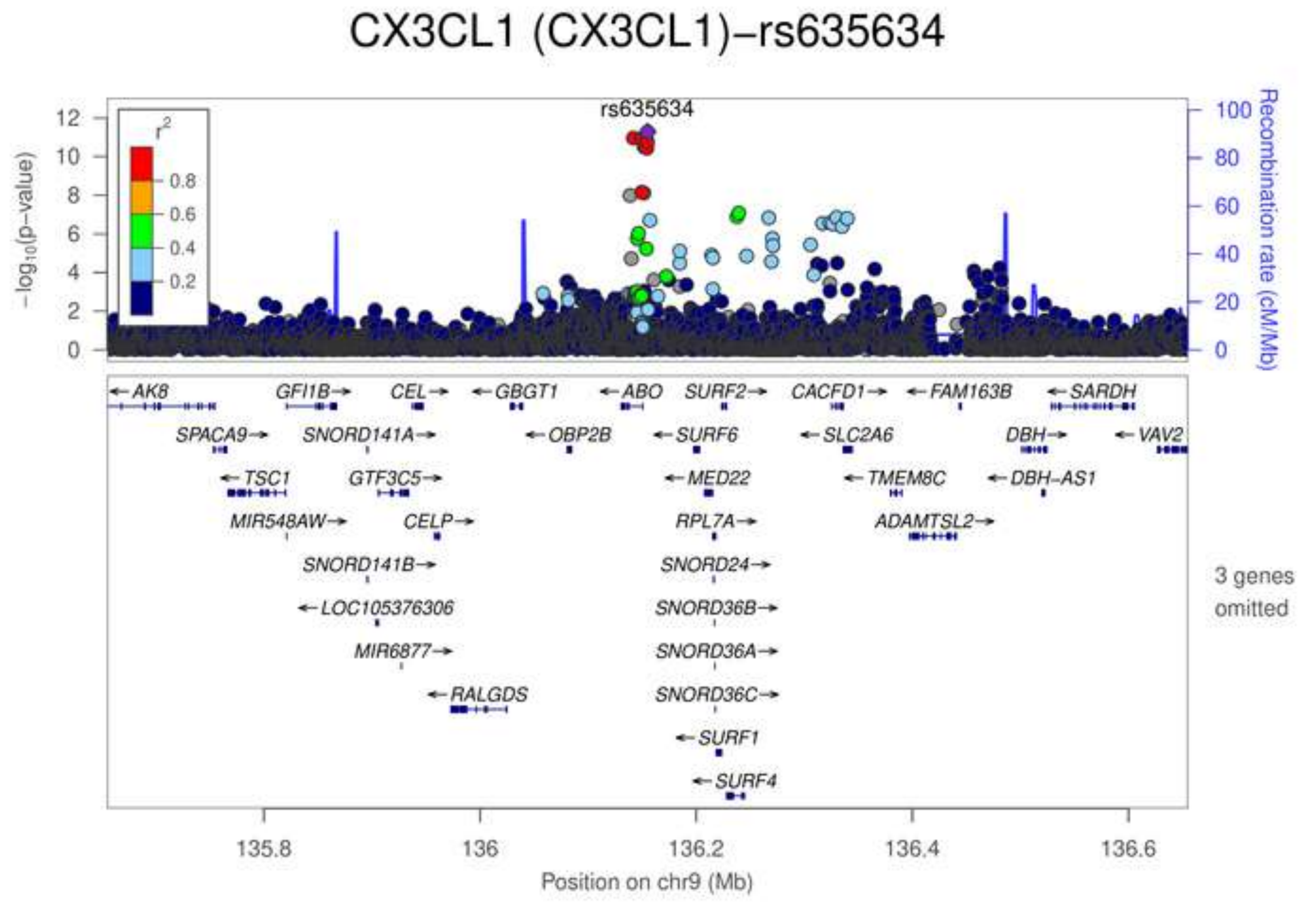
CX3CL1 (CX3CL1)−rs635634
rs635634
$-\log_{10}(p\text{-value})$
Recombination rate (cM/Mb)
r^2
0.8
0.6
0.4
0.2
AK8
GFI1B
CEL
GBGT1
ABO
SURF2
CACFD1
FAM163B
SARDH
SPACA9
SNORD141A
OBP2B
SURF6
SLC2A6
DBH
VAV2
TSC1
GTF3C5
MED22
TMEM8C
DBH-AS1
MIR548AW
CELP
RPL7A
ADAMTSL2
SNORD141B
SNORD24
LOC105376306
SNORD36B
MIR6877
SNORD36A
RALGDS
SNORD36C
SURF1
SURF4
3 genes omitted
135.8 136 136.2 136.4 136.6
Position on chr9 (Mb)

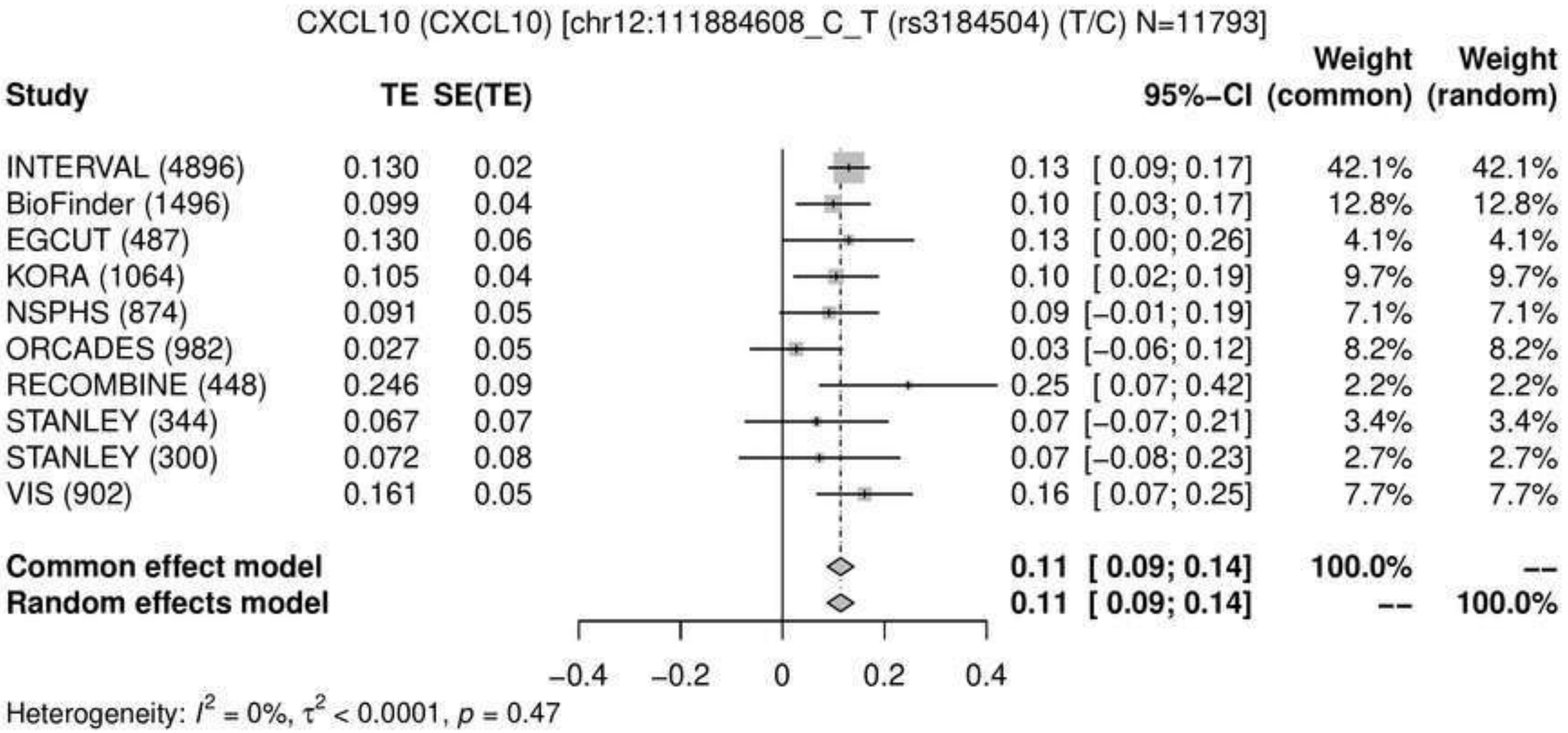
CXCL10 (CXCL10) [chr12:111884608_C_T (rs3184504) (T/C) N=11793]
Study
TE
SE(TE)
95%−CI
Weight (common)
Weight (random)
INTERVAL (4896) 0.130 0.02 0.13 [0.09; 0.17] 42.1% 42.1%
BioFinder (1496) 0.099 0.04 0.10 [0.03; 0.17] 12.8% 12.8%
EGCUT (487) 0.130 0.06 0.13 [0.00; 0.26] 4.1% 4.1%
KORA (1064) 0.105 0.04 0.10 [0.02; 0.19] 9.7% 9.7%
NSPHS (874) 0.091 0.05 0.09 [−0.01; 0.19] 7.1% 7.1%
ORCADES (982) 0.027 0.05 0.03 [−0.06; 0.12] 8.2% 8.2%
RECOMBINE (448) 0.246 0.09 0.25 [0.07; 0.42] 2.2% 2.2%
STANLEY (344) 0.067 0.07 0.07 [−0.07; 0.21] 3.4% 3.4%
STANLEY (300) 0.072 0.08 0.07 [−0.08; 0.23] 2.7% 2.7%
VIS (902) 0.161 0.05 0.16 [0.07; 0.25] 7.7% 7.7%
Common effect model 0.11 [0.09; 0.14] 100.0% --
Random effects model 0.11 [0.09; 0.14] -- 100.0%
−0.4 −0.2 0 0.2 0.4
Heterogeneity: $I^2 = 0\%$, $\tau^2 < 0.0001$, $p = 0.47$
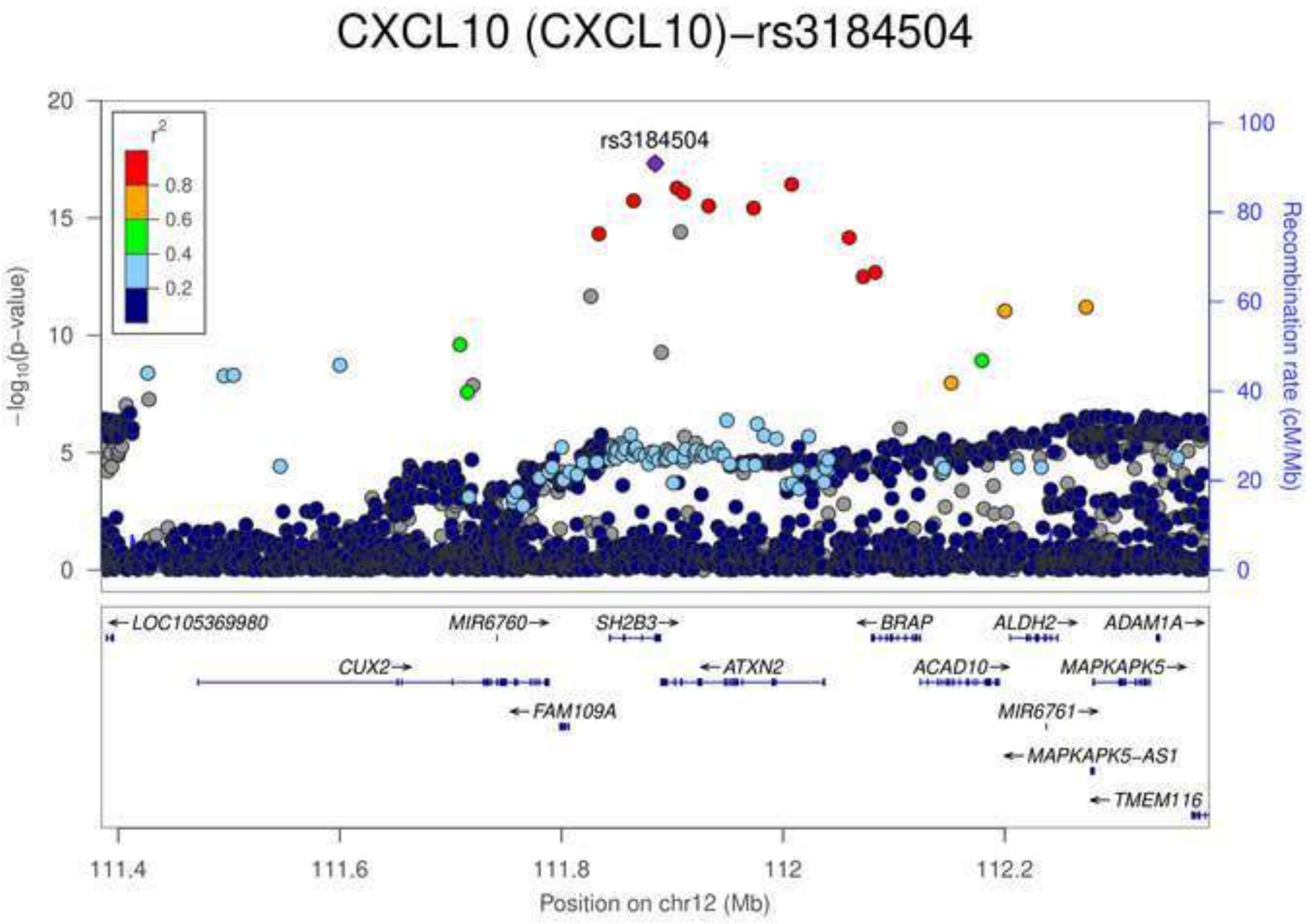
CXCL10 (CXCL10)−rs3184504
rs3184504
r^2
0.8
0.6
0.4
0.2
$-\log_{10}$(p-value)
Recombination rate (cM/Mb)
LOC105369980
MIR6760
SH2B3
BRAP
ALDH2
ADAM1A
CUX2
ATXN2
ACAD10
MAPKAPK5
FAM109A
MIR6761
MAPKAPK5-AS1
TMEM116
111.4
111.6
111.8
112
112.2
Position on chr12 (Mb)

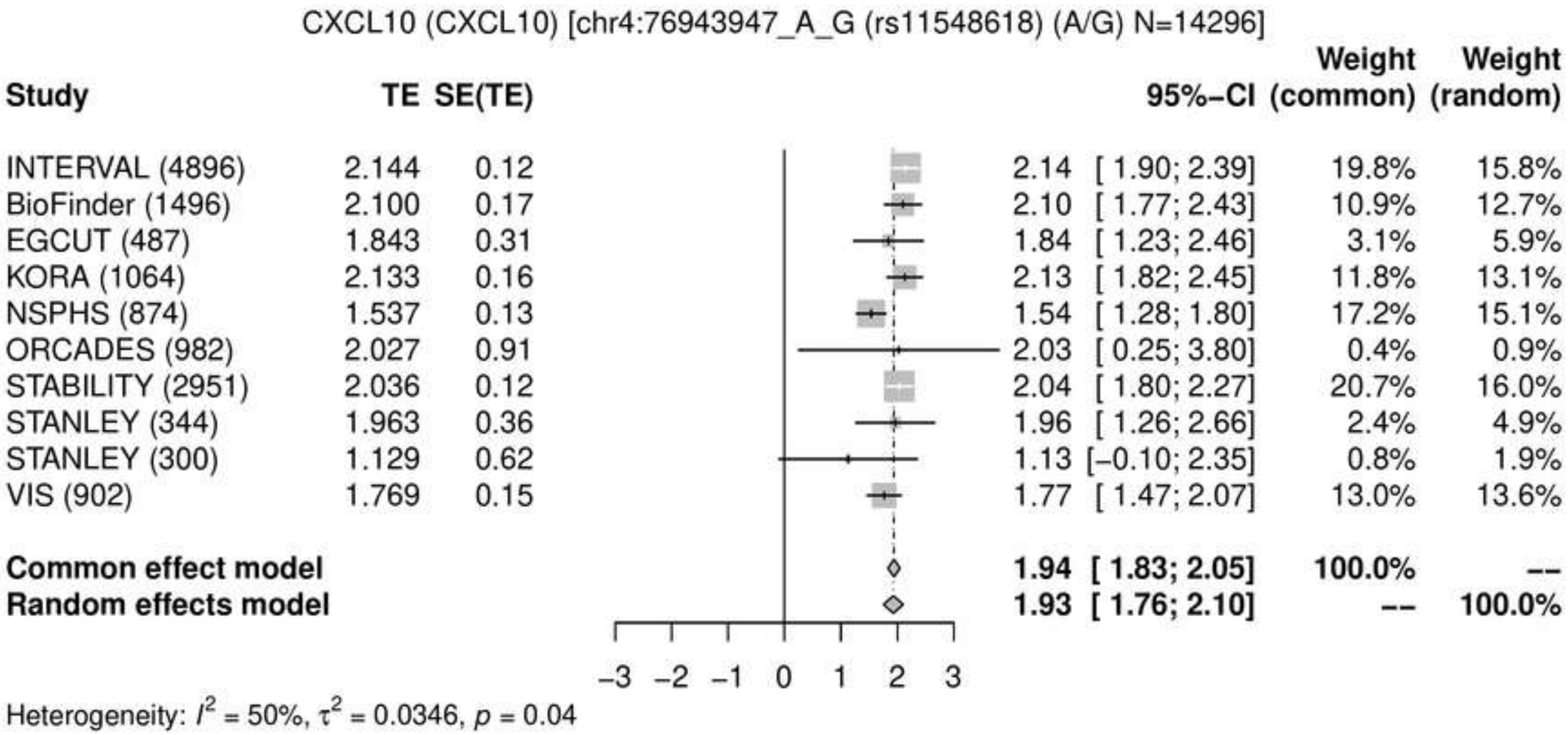
CXCL10 (CXCL10) [chr4:76943947_A_G (rs11548618) (A/G) N=14296]
Study | TE | SE(TE) | 95%−CI | Weight (common) | Weight (random)
INTERVAL (4896) | 2.144 | 0.12 | 2.14 [1.90; 2.39] | 19.8% | 15.8%
BioFinder (1496) | 2.100 | 0.17 | 2.10 [1.77; 2.43] | 10.9% | 12.7%
EGCUT (487) | 1.843 | 0.31 | 1.84 [1.23; 2.46] | 3.1% | 5.9%
KORA (1064) | 2.133 | 0.16 | 2.13 [1.82; 2.45] | 11.8% | 13.1%
NSPHS (874) | 1.537 | 0.13 | 1.54 [1.28; 1.80] | 17.2% | 15.1%
ORCADES (982) | 2.027 | 0.91 | 2.03 [0.25; 3.80] | 0.4% | 0.9%
STABILITY (2951) | 2.036 | 0.12 | 2.04 [1.80; 2.27] | 20.7% | 16.0%
STANLEY (344) | 1.963 | 0.36 | 1.96 [1.26; 2.66] | 2.4% | 4.9%
STANLEY (300) | 1.129 | 0.62 | 1.13 [−0.10; 2.35] | 0.8% | 1.9%
VIS (902) | 1.769 | 0.15 | 1.77 [1.47; 2.07] | 13.0% | 13.6%
Common effect model | 1.94 [1.83; 2.05] | 100.0% | --
Random effects model | 1.93 [1.76; 2.10] | -- | 100.0%
−3 −2 −1 0 1 2 3
Heterogeneity: I^2 = 50%, τ^2 = 0.0346, p = 0.04

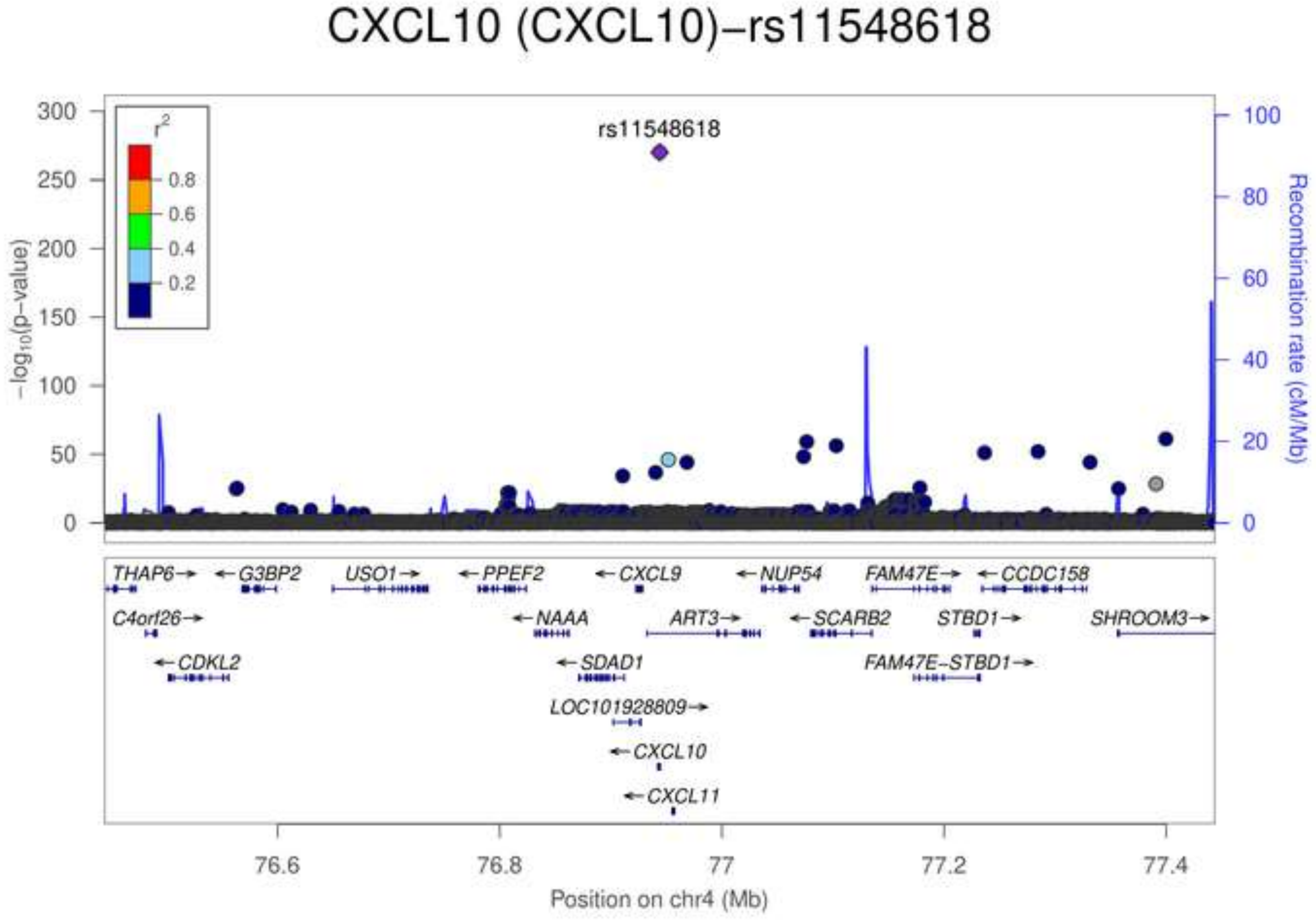
CXCL10 (CXCL10)−rs11548618
rs11548618
r^2
0.8
0.6
0.4
0.2
$-\log_{10}$(p−value)
Recombination rate (cM/Mb)
THAP6→ ←G3BP2 USO1→ ←PPEF2 ←CXCL9 ←NUP54 FAM47E→ ←CCDC158
C4orf26→ ←NAAA ART3→ ←SCARB2 STBD1→ SHROOM3→
←CDKL2 ←SDAD1 FAM47E−STBD1→
LOC101928809→
←CXCL10
←CXCL11
76.6 76.8 77 77.2 77.4
Position on chr4 (Mb)

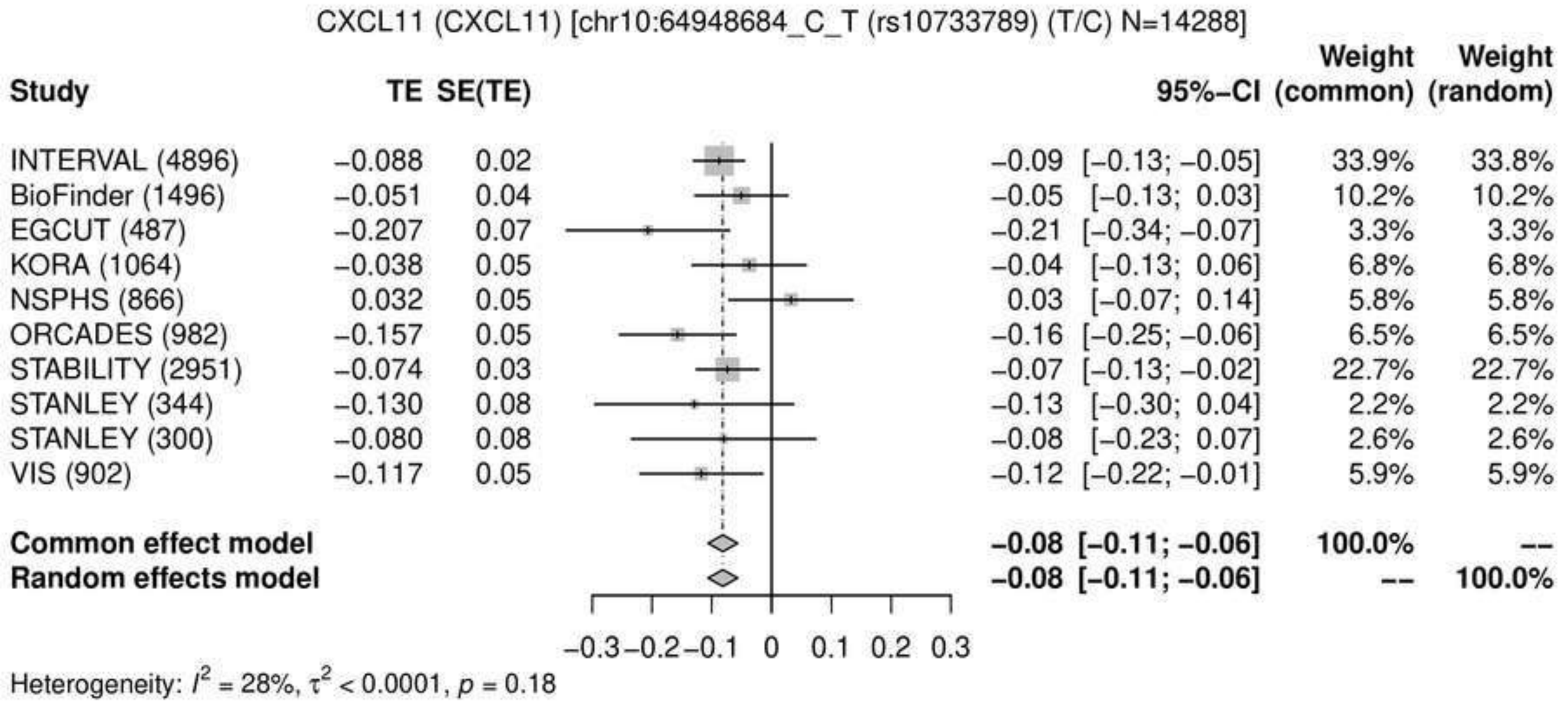
CXCL11 (CXCL11) [chr10:64948684_C_T (rs10733789) (T/C) N=14288]
Study
TE
SE(TE)
95%-CI
Weight (common)
Weight (random)
INTERVAL (4896) −0.088 0.02 −0.09 [−0.13; −0.05] 33.9% 33.8%
BioFinder (1496) −0.051 0.04 −0.05 [−0.13; 0.03] 10.2% 10.2%
EGCUT (487) −0.207 0.07 −0.21 [−0.34; −0.07] 3.3% 3.3%
KORA (1064) −0.038 0.05 −0.04 [−0.13; 0.06] 6.8% 6.8%
NSPHS (866) 0.032 0.05 0.03 [−0.07; 0.14] 5.8% 5.8%
ORCADES (982) −0.157 0.05 −0.16 [−0.25; −0.06] 6.5% 6.5%
STABILITY (2951) −0.074 0.03 −0.07 [−0.13; −0.02] 22.7% 22.7%
STANLEY (344) −0.130 0.08 −0.13 [−0.30; 0.04] 2.2% 2.2%
STANLEY (300) −0.080 0.08 −0.08 [−0.23; 0.07] 2.6% 2.6%
VIS (902) −0.117 0.05 −0.12 [−0.22; −0.01] 5.9% 5.9%
Common effect model −0.08 [−0.11; −0.06] 100.0% --
Random effects model −0.08 [−0.11; −0.06] -- 100.0%
−0.3 −0.2 −0.1 0 0.1 0.2 0.3
Heterogeneity: I^2 = 28%, τ^2 < 0.0001, p = 0.18

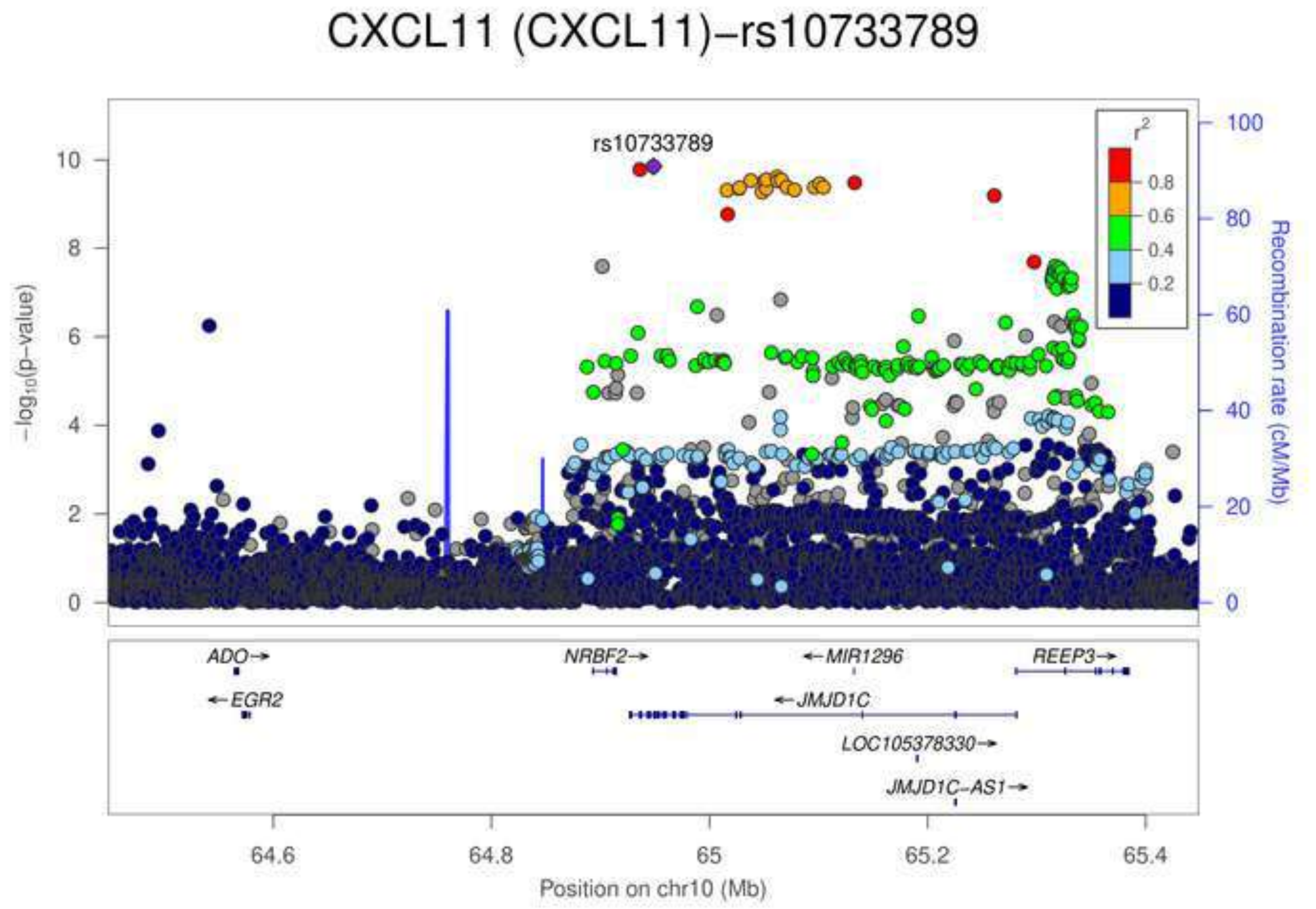
CXCL11 (CXCL11)−rs10733789
rs10733789
$-\log_{10}$(p-value)
Recombination rate (cM/Mb)
r^2
0.8
0.6
0.4
0.2
ADO→
←EGR2
NRBF2→
←MIR1296
←JMJD1C
REEP3→
LOC105378330→
JMJD1C−AS1→
64.6
64.8
65
65.2
65.4
Position on chr10 (Mb)

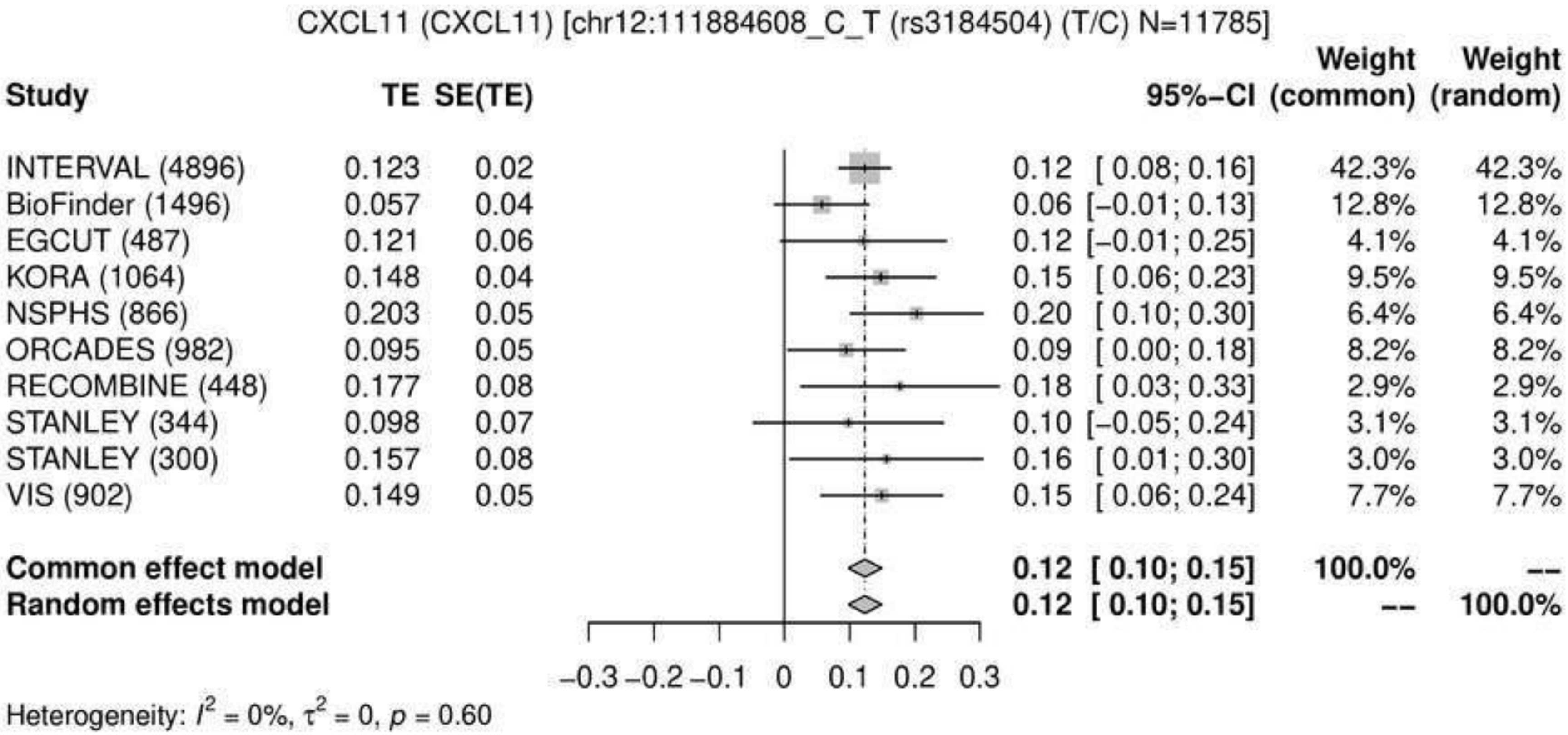
CXCL11 (CXCL11) [chr12:111884608_C_T (rs3184504) (T/C) N=11785]
Study
TE
SE(TE)
95%−CI
Weight (common)
Weight (random)
INTERVAL (4896) 0.123 0.02 0.12 [0.08; 0.16] 42.3% 42.3%
BioFinder (1496) 0.057 0.04 0.06 [−0.01; 0.13] 12.8% 12.8%
EGCUT (487) 0.121 0.06 0.12 [−0.01; 0.25] 4.1% 4.1%
KORA (1064) 0.148 0.04 0.15 [0.06; 0.23] 9.5% 9.5%
NSPHS (866) 0.203 0.05 0.20 [0.10; 0.30] 6.4% 6.4%
ORCADES (982) 0.095 0.05 0.09 [0.00; 0.18] 8.2% 8.2%
RECOMBINE (448) 0.177 0.08 0.18 [0.03; 0.33] 2.9% 2.9%
STANLEY (344) 0.098 0.07 0.10 [−0.05; 0.24] 3.1% 3.1%
STANLEY (300) 0.157 0.08 0.16 [0.01; 0.30] 3.0% 3.0%
VIS (902) 0.149 0.05 0.15 [0.06; 0.24] 7.7% 7.7%
Common effect model 0.12 [0.10; 0.15] 100.0% --
Random effects model 0.12 [0.10; 0.15] -- 100.0%
−0.3 −0.2 −0.1 0 0.1 0.2 0.3
Heterogeneity: I^2 = 0%, τ^2 = 0, p = 0.60
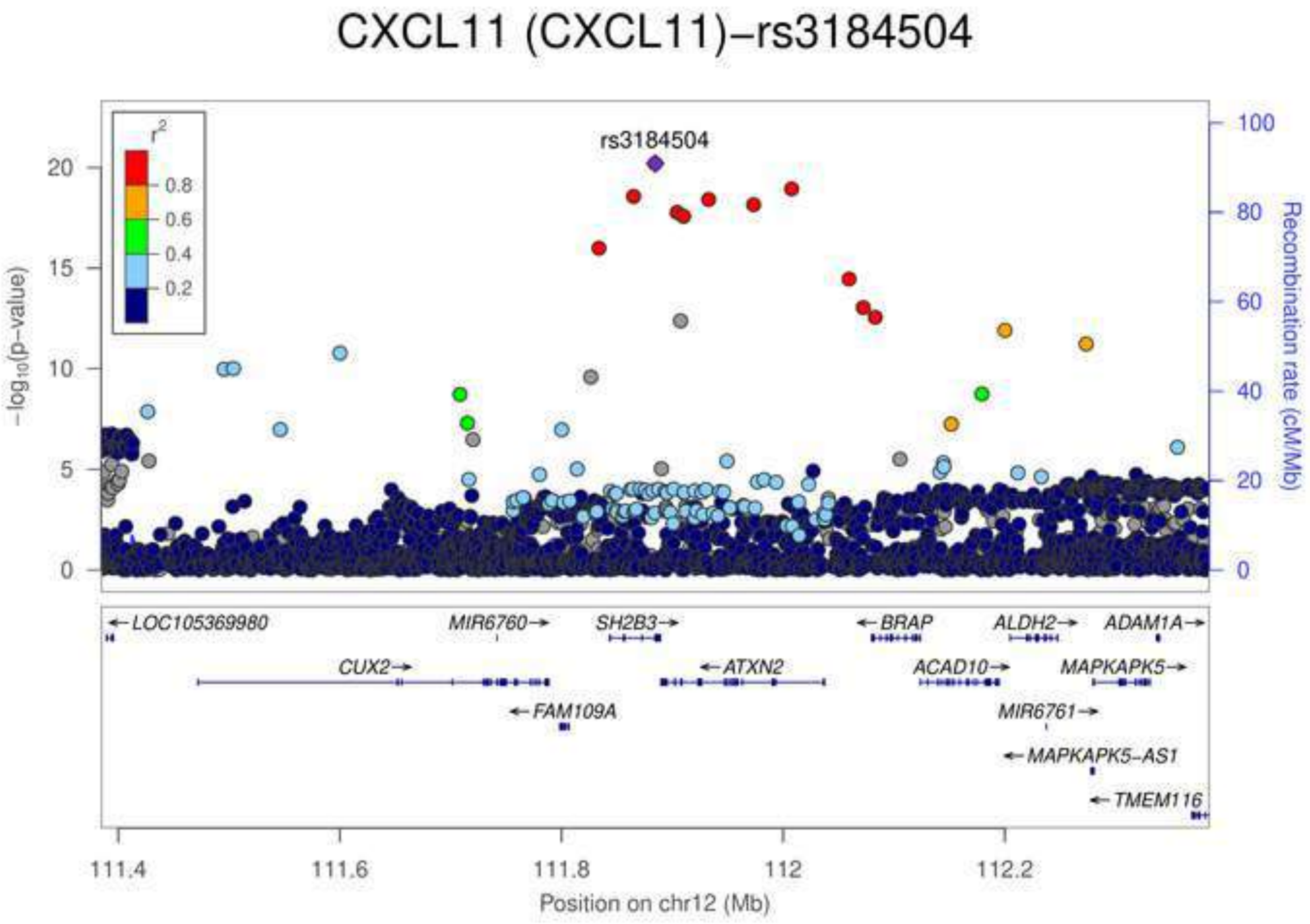
CXCL11 (CXCL11)−rs3184504
rs3184504
r^2
0.8
0.6
0.4
0.2
−log10(p−value)
Recombination rate (cM/Mb)
LOC105369980
MIR6760
SH2B3
BRAP
ALDH2
ADAM1A
CUX2
ATXN2
ACAD10
MAPKAPK5
FAM109A
MIR6761
MAPKAPK5-AS1
TMEM116
111.4
111.6
111.8
112
112.2
Position on chr12 (Mb)

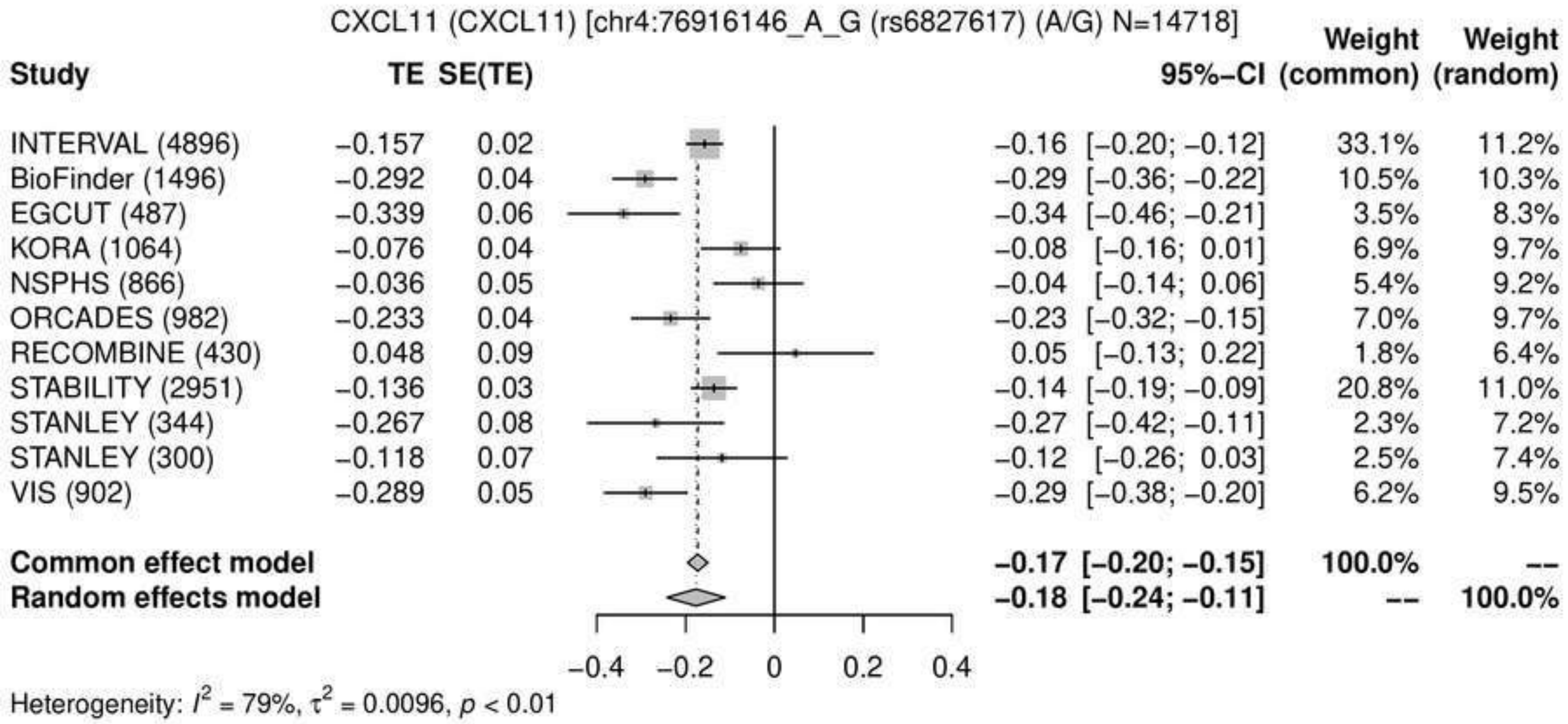
CXCL11 (CXCL11) [chr4:76916146_A_G (rs6827617) (A/G) N=14718]
Study
TE
SE(TE)
95%-CI
Weight (common)
Weight (random)
INTERVAL (4896) −0.157 0.02 −0.16 [−0.20; −0.12] 33.1% 11.2%
BioFinder (1496) −0.292 0.04 −0.29 [−0.36; −0.22] 10.5% 10.3%
EGCUT (487) −0.339 0.06 −0.34 [−0.46; −0.21] 3.5% 8.3%
KORA (1064) −0.076 0.04 −0.08 [−0.16; 0.01] 6.9% 9.7%
NSPHS (866) −0.036 0.05 −0.04 [−0.14; 0.06] 5.4% 9.2%
ORCADES (982) −0.233 0.04 −0.23 [−0.32; −0.15] 7.0% 9.7%
RECOMBINE (430) 0.048 0.09 0.05 [−0.13; 0.22] 1.8% 6.4%
STABILITY (2951) −0.136 0.03 −0.14 [−0.19; −0.09] 20.8% 11.0%
STANLEY (344) −0.267 0.08 −0.27 [−0.42; −0.11] 2.3% 7.2%
STANLEY (300) −0.118 0.07 −0.12 [−0.26; 0.03] 2.5% 7.4%
VIS (902) −0.289 0.05 −0.29 [−0.38; −0.20] 6.2% 9.5%
Common effect model −0.17 [−0.20; −0.15] 100.0% --
Random effects model −0.18 [−0.24; −0.11] -- 100.0%
−0.4 −0.2 0 0.2 0.4
Heterogeneity: I² = 79%, τ² = 0.0096, p < 0.01

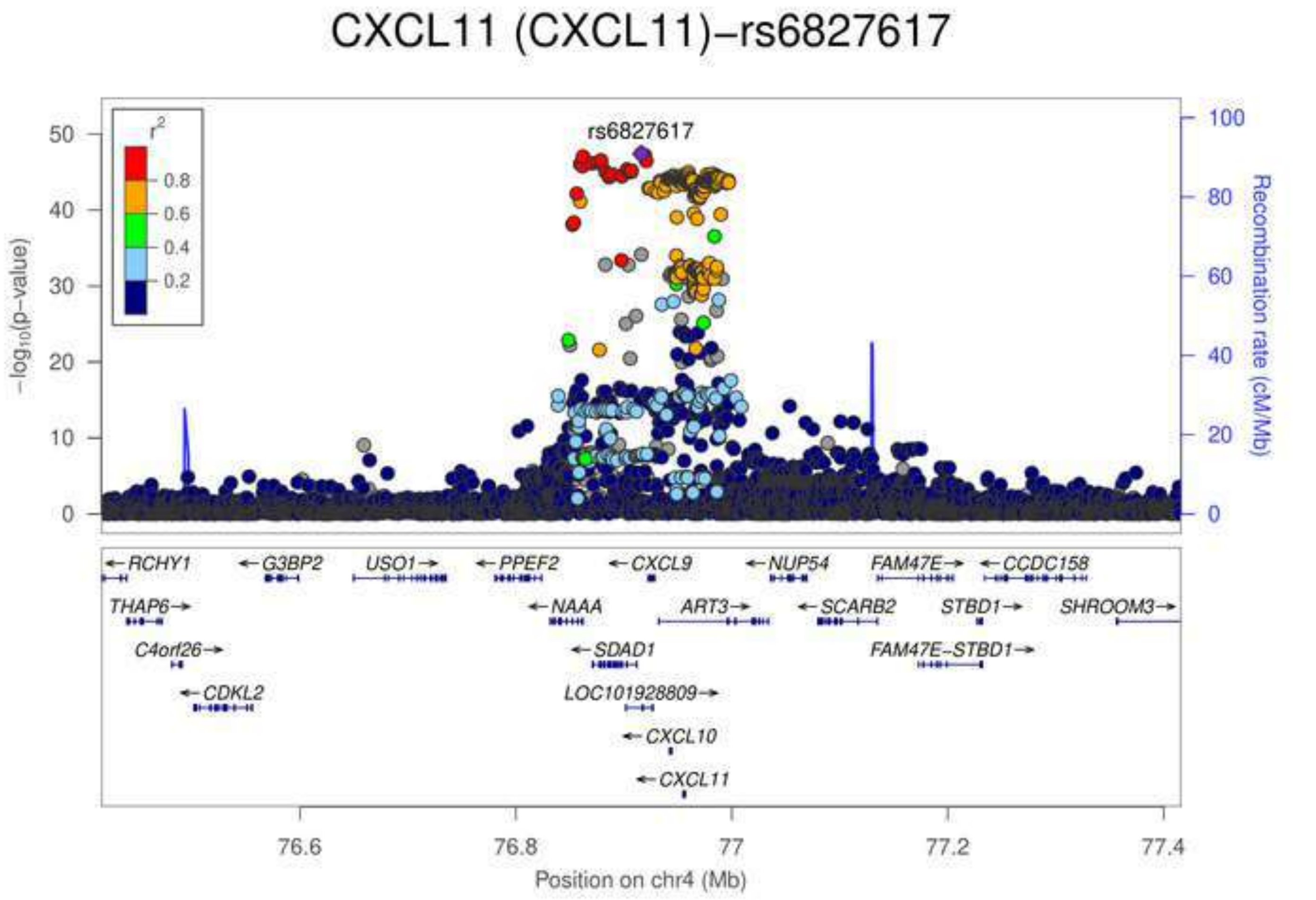
CXCL11 (CXCL11)−rs6827617
rs6827617
r²
0.8
0.6
0.4
0.2
−log10(p−value)
Recombination rate (cM/Mb)
RCHY1
G3BP2
USO1
PPEF2
CXCL9
NUP54
FAM47E
CCDC158
THAP6
NAAA
ART3
SCARB2
STBD1
SHROOM3
C4orf26
SDAD1
FAM47E−STBD1
CDKL2
LOC101928809
CXCL10
CXCL11
76.6
76.8
77
77.2
77.4
Position on chr4 (Mb)

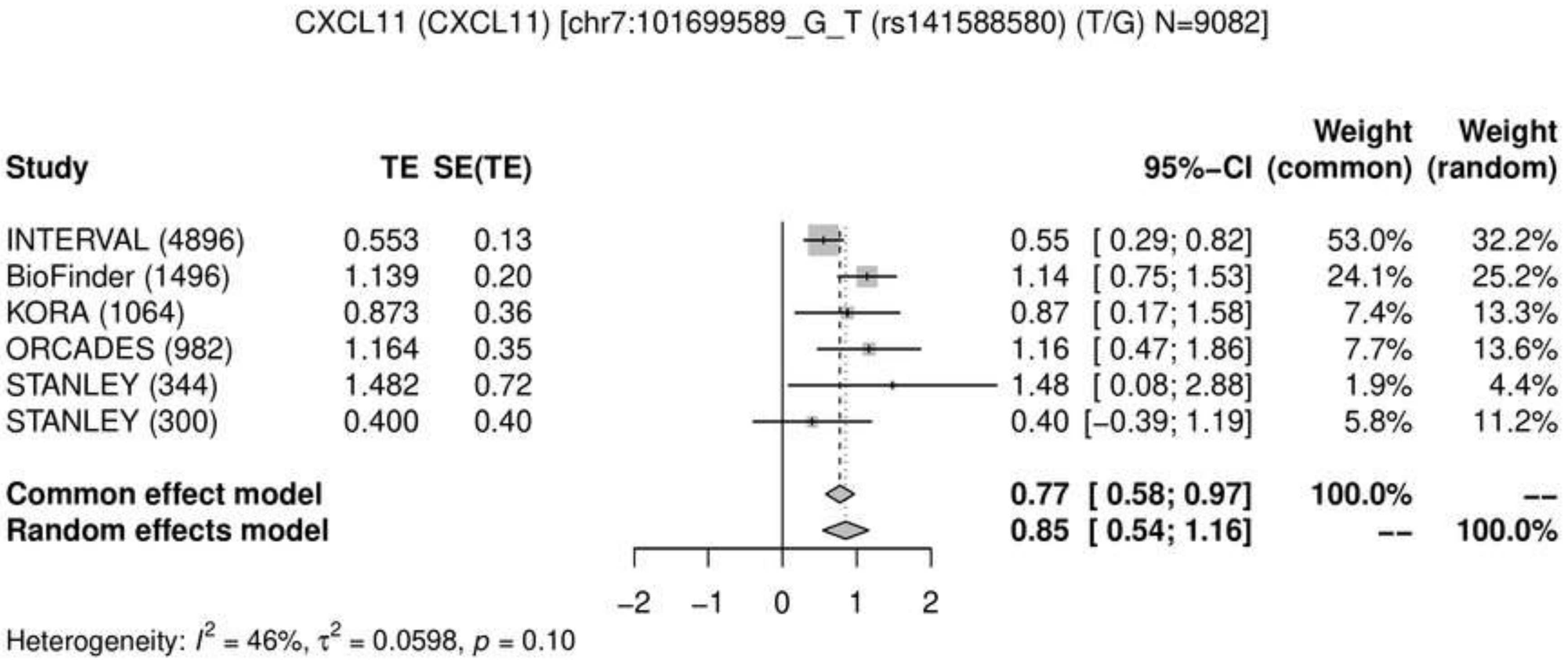
CXCL11 (CXCL11) [chr7:101699589_G_T (rs141588580) (T/G) N=9082]
Study
TE
SE(TE)
95%-CI
Weight (common)
Weight (random)
INTERVAL (4896) 0.553 0.13 0.55 [0.29; 0.82] 53.0% 32.2%
BioFinder (1496) 1.139 0.20 1.14 [0.75; 1.53] 24.1% 25.2%
KORA (1064) 0.873 0.36 0.87 [0.17; 1.58] 7.4% 13.3%
ORCADES (982) 1.164 0.35 1.16 [0.47; 1.86] 7.7% 13.6%
STANLEY (344) 1.482 0.72 1.48 [0.08; 2.88] 1.9% 4.4%
STANLEY (300) 0.400 0.40 0.40 [−0.39; 1.19] 5.8% 11.2%
Common effect model 0.77 [0.58; 0.97] 100.0% --
Random effects model 0.85 [0.54; 1.16] -- 100.0%
−2
−1
0
1
2
Heterogeneity: I^2 = 46%, τ^2 = 0.0598, p = 0.10

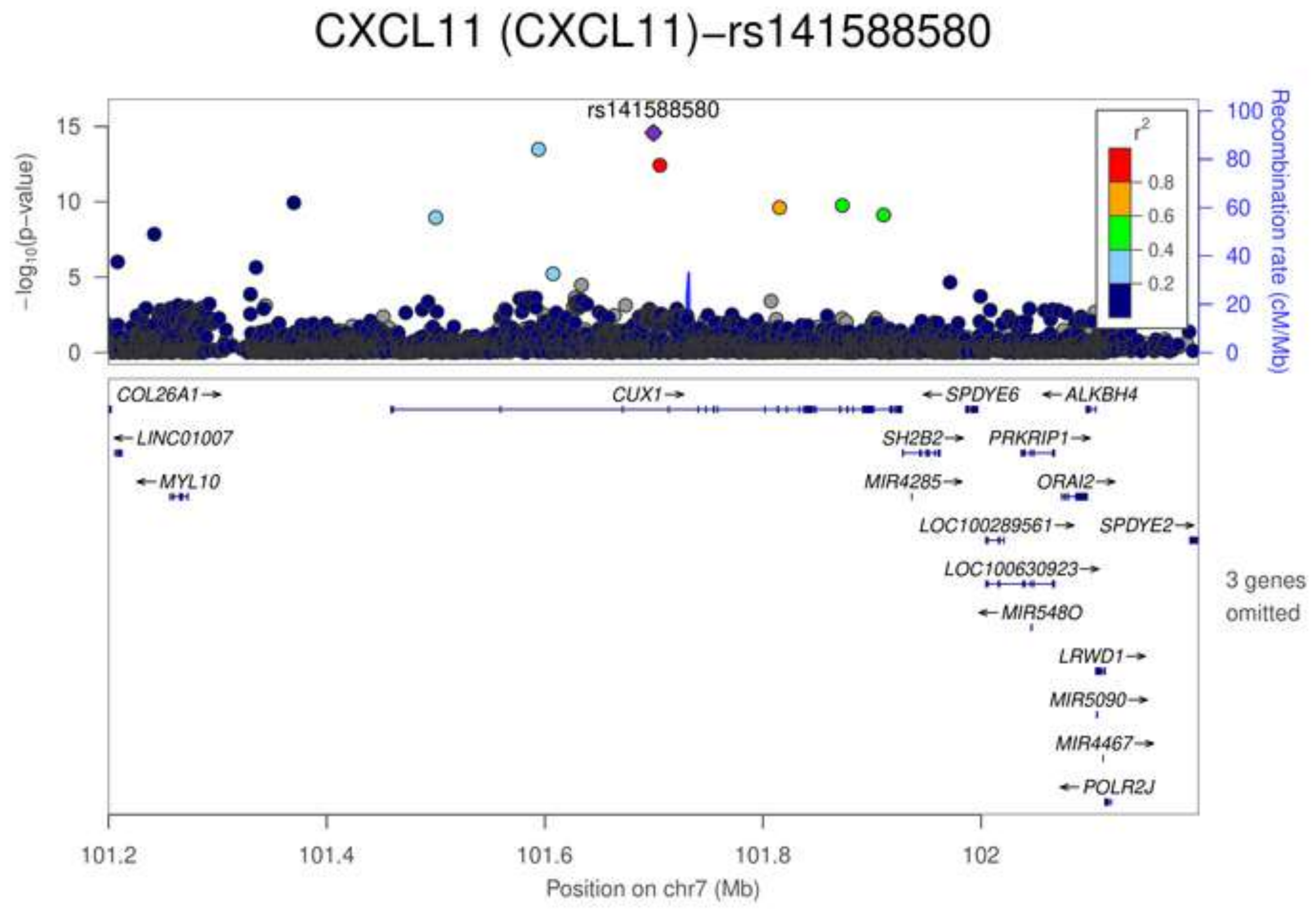
CXCL11 (CXCL11)−rs141588580
rs141588580
$-\log_{10}$(p−value)
Recombination rate (cM/Mb)
r^2
0.8
0.6
0.4
0.2
15
10
5
0
100
80
60
40
20
0
COL26A1→
←LINC01007
←MYL10
CUX1→
←SPDYE6
←ALKBH4
SH2B2→
PRKRIP1→
MIR4285→
ORAI2→
LOC100289561→
SPDYE2→
LOC100630923→
←MIR548O
LRWD1→
MIR5090→
MIR4467→
←POLR2J
3 genes omitted
101.2
101.4
101.6
101.8
102
Position on chr7 (Mb)

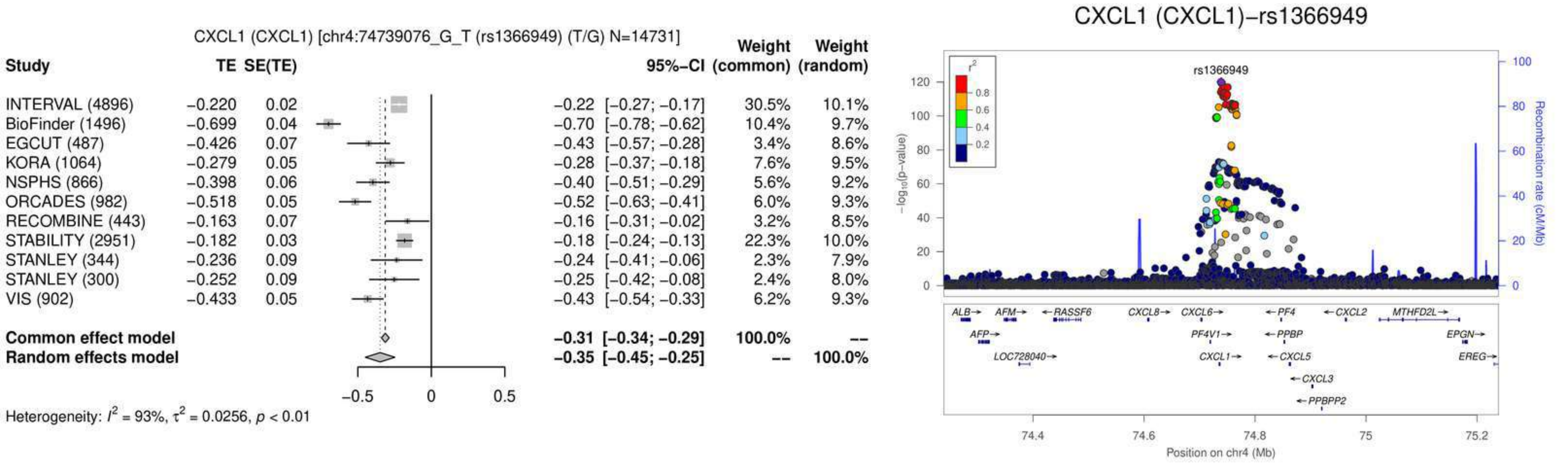
CXCL1 (CXCL1) [chr4:74739076_G_T (rs1366949) (T/G) N=14731]
Study
TE
SE(TE)
95%-CI
Weight (common)
Weight (random)
INTERVAL (4896) −0.220 0.02 −0.22 [−0.27; −0.17] 30.5% 10.1%
BioFinder (1496) −0.699 0.04 −0.70 [−0.78; −0.62] 10.4% 9.7%
EGCUT (487) −0.426 0.07 −0.43 [−0.57; −0.28] 3.4% 8.6%
KORA (1064) −0.279 0.05 −0.28 [−0.37; −0.18] 7.6% 9.5%
NSPHS (866) −0.398 0.06 −0.40 [−0.51; −0.29] 5.6% 9.2%
ORCADES (982) −0.518 0.05 −0.52 [−0.63; −0.41] 6.0% 9.3%
RECOMBINE (443) −0.163 0.07 −0.16 [−0.31; −0.02] 3.2% 8.5%
STABILITY (2951) −0.182 0.03 −0.18 [−0.24; −0.13] 22.3% 10.0%
STANLEY (344) −0.236 0.09 −0.24 [−0.41; −0.06] 2.3% 7.9%
STANLEY (300) −0.252 0.09 −0.25 [−0.42; −0.08] 2.4% 8.0%
VIS (902) −0.433 0.05 −0.43 [−0.54; −0.33] 6.2% 9.3%
Common effect model −0.31 [−0.34; −0.29] 100.0% --
Random effects model −0.35 [−0.45; −0.25] -- 100.0%
−0.5
0
0.5
Heterogeneity: $I^2 = 93\%$, $\tau^2 = 0.0256$, $p < 0.01$
CXCL1 (CXCL1)−rs1366949
rs1366949
r^2
0.8
0.6
0.4
0.2
$-\log_{10}$(p-value)
Recombination rate (cM/Mb)
ALB→
AFM→
←RASSF6
CXCL8→
CXCL6→
←PF4
←CXCL2
MTHFD2L→
AFP→
PF4V1→
←PPBP
EPGN→
LOC728040→
CXCL1→
←CXCL5
EREG→
←CXCL3
←PPBPP2
74.4
74.6
74.8
75
75.2
Position on chr4 (Mb)

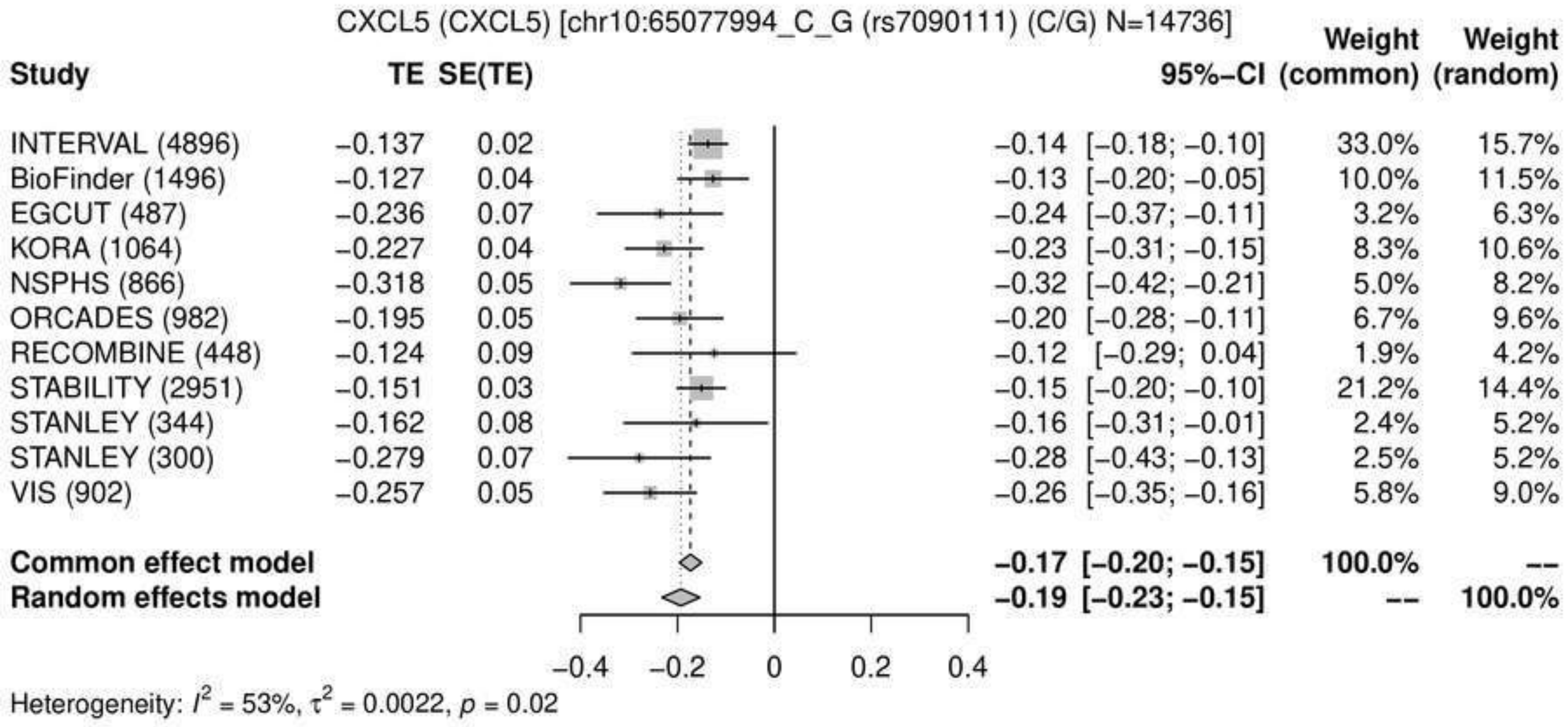
CXCL5 (CXCL5) [chr10:65077994_C_G (rs7090111) (C/G) N=14736]
Study
TE
SE(TE)
95%−CI
Weight (common)
Weight (random)
INTERVAL (4896) −0.137 0.02 −0.14 [−0.18; −0.10] 33.0% 15.7%
BioFinder (1496) −0.127 0.04 −0.13 [−0.20; −0.05] 10.0% 11.5%
EGCUT (487) −0.236 0.07 −0.24 [−0.37; −0.11] 3.2% 6.3%
KORA (1064) −0.227 0.04 −0.23 [−0.31; −0.15] 8.3% 10.6%
NSPHS (866) −0.318 0.05 −0.32 [−0.42; −0.21] 5.0% 8.2%
ORCADES (982) −0.195 0.05 −0.20 [−0.28; −0.11] 6.7% 9.6%
RECOMBINE (448) −0.124 0.09 −0.12 [−0.29; 0.04] 1.9% 4.2%
STABILITY (2951) −0.151 0.03 −0.15 [−0.20; −0.10] 21.2% 14.4%
STANLEY (344) −0.162 0.08 −0.16 [−0.31; −0.01] 2.4% 5.2%
STANLEY (300) −0.279 0.07 −0.28 [−0.43; −0.13] 2.5% 5.2%
VIS (902) −0.257 0.05 −0.26 [−0.35; −0.16] 5.8% 9.0%
Common effect model −0.17 [−0.20; −0.15] 100.0% --
Random effects model −0.19 [−0.23; −0.15] -- 100.0%
−0.4
−0.2
0
0.2
0.4
Heterogeneity: I^2 = 53%, τ^2 = 0.0022, p = 0.02
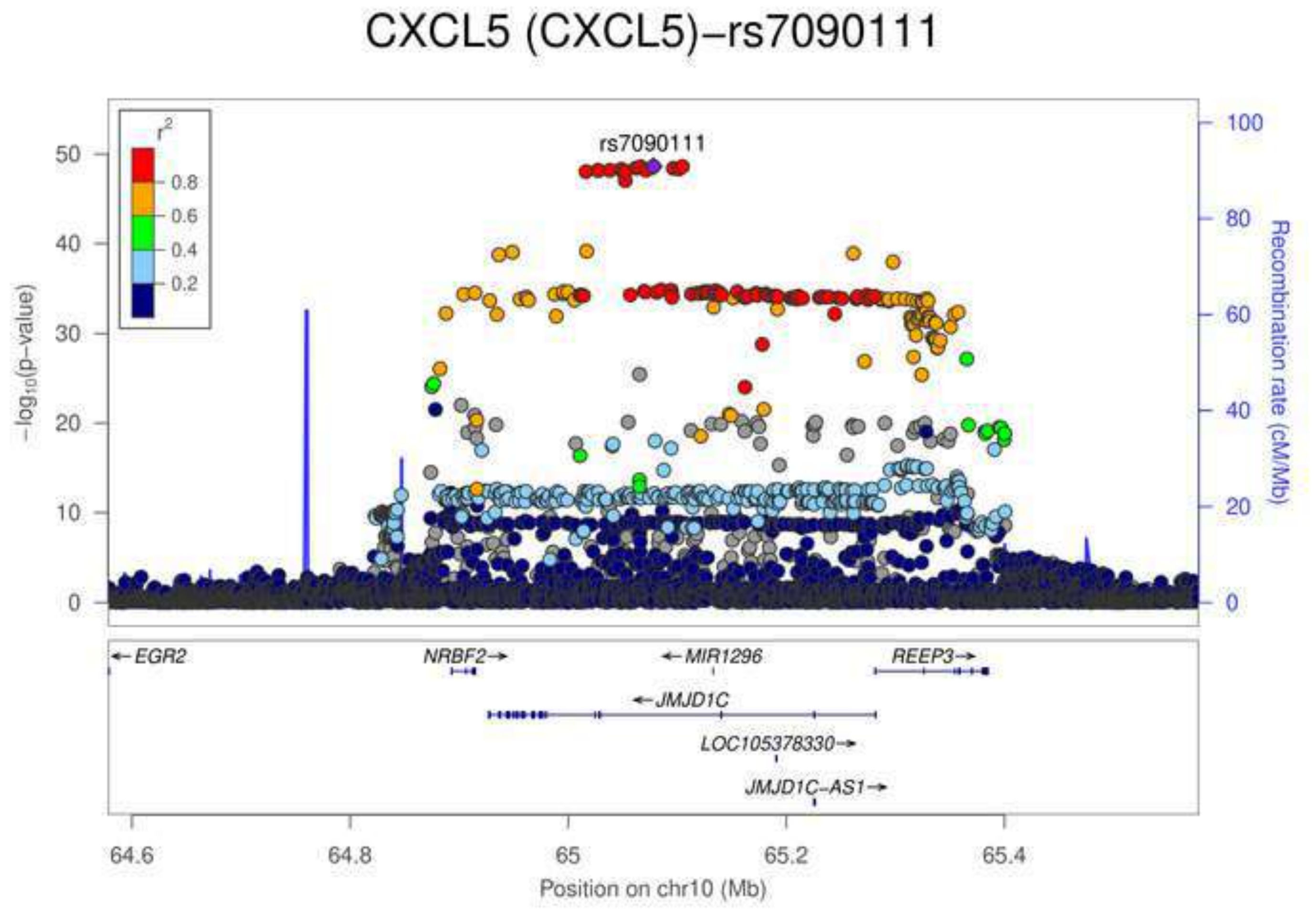
CXCL5 (CXCL5)−rs7090111
rs7090111
r^2
0.8
0.6
0.4
0.2
$-\log_{10}$(p−value)
Recombination rate (cM/Mb)
EGR2
NRBF2
MIR1296
REEP3
JMJD1C
LOC105378330
JMJD1C−AS1
64.6
64.8
65
65.2
65.4
Position on chr10 (Mb)

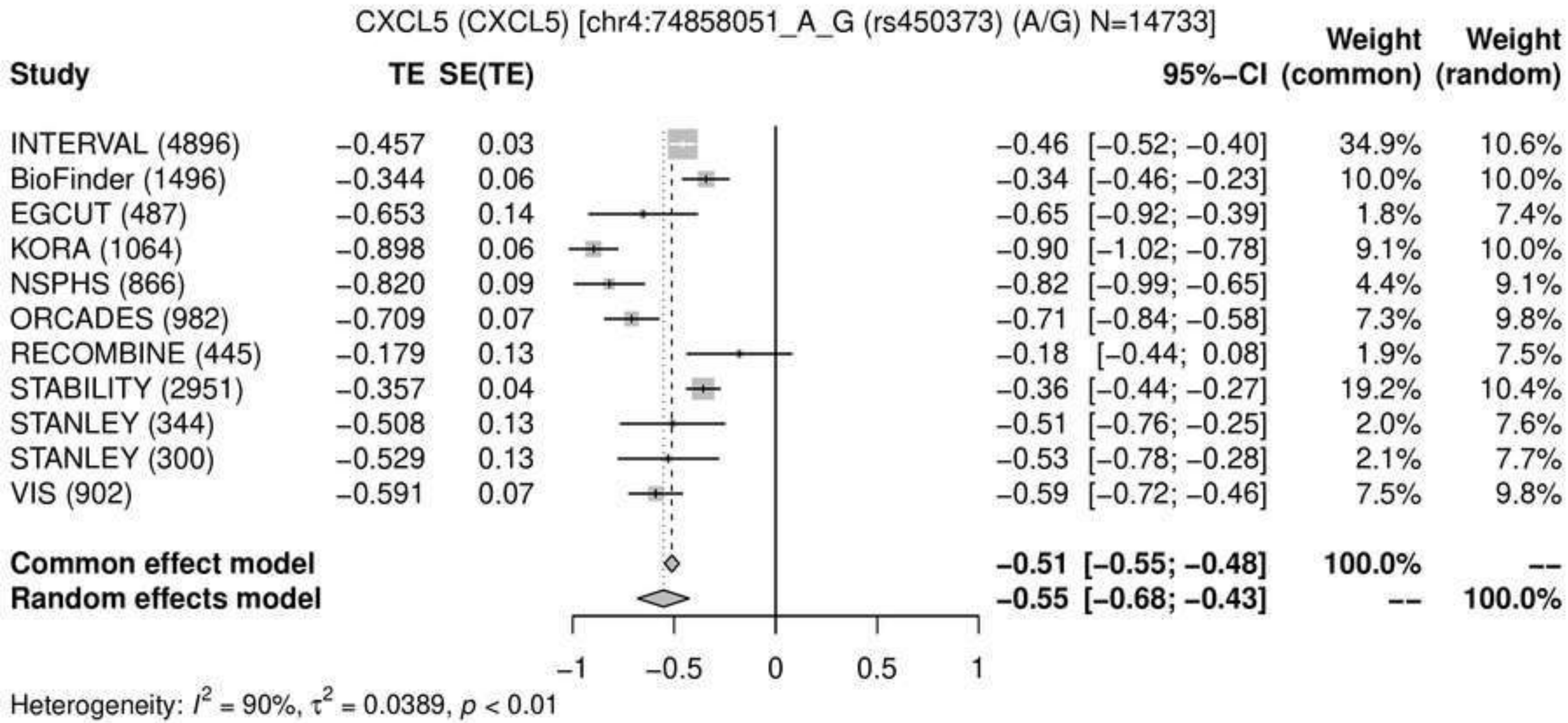
CXCL5 (CXCL5) [chr4:74858051_A_G (rs450373) (A/G) N=14733]
Study TE SE(TE) 95%-CI Weight (common) Weight (random)
INTERVAL (4896) −0.457 0.03 −0.46 [−0.52; −0.40] 34.9% 10.6%
BioFinder (1496) −0.344 0.06 −0.34 [−0.46; −0.23] 10.0% 10.0%
EGCUT (487) −0.653 0.14 −0.65 [−0.92; −0.39] 1.8% 7.4%
KORA (1064) −0.898 0.06 −0.90 [−1.02; −0.78] 9.1% 10.0%
NSPHS (866) −0.820 0.09 −0.82 [−0.99; −0.65] 4.4% 9.1%
ORCADES (982) −0.709 0.07 −0.71 [−0.84; −0.58] 7.3% 9.8%
RECOMBINE (445) −0.179 0.13 −0.18 [−0.44; 0.08] 1.9% 7.5%
STABILITY (2951) −0.357 0.04 −0.36 [−0.44; −0.27] 19.2% 10.4%
STANLEY (344) −0.508 0.13 −0.51 [−0.76; −0.25] 2.0% 7.6%
STANLEY (300) −0.529 0.13 −0.53 [−0.78; −0.28] 2.1% 7.7%
VIS (902) −0.591 0.07 −0.59 [−0.72; −0.46] 7.5% 9.8%
Common effect model −0.51 [−0.55; −0.48] 100.0% --
Random effects model −0.55 [−0.68; −0.43] -- 100.0%
−1 −0.5 0 0.5 1
Heterogeneity: I^2 = 90%, τ^2 = 0.0389, p < 0.01

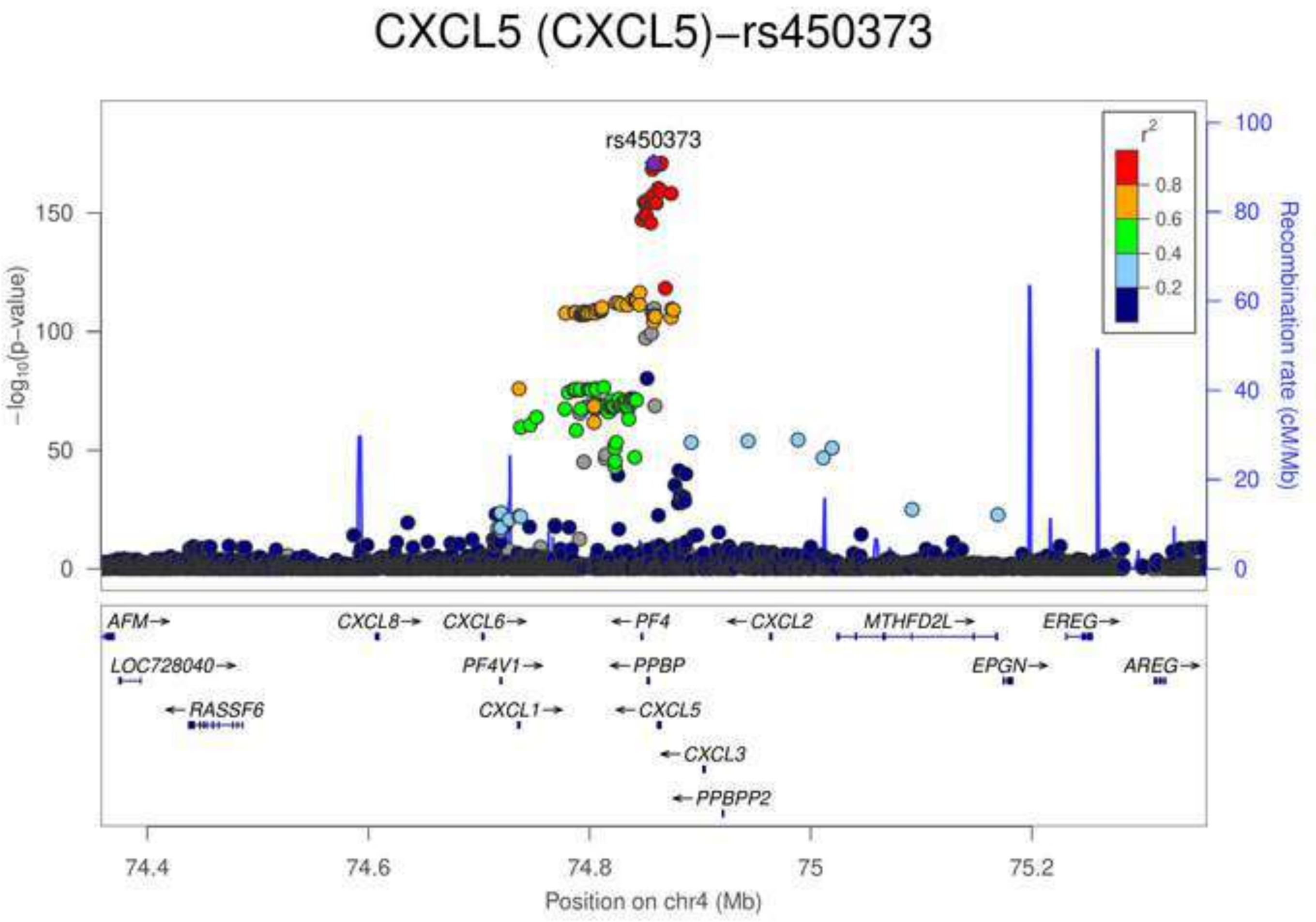
CXCL5 (CXCL5)−rs450373
rs450373
$-\log_{10}$(p−value)
Recombination rate (cM/Mb)
r^2
0.8
0.6
0.4
0.2
0
50
100
150
20
40
60
80
AFM→
LOC728040→
←RASSF6
CXCL8→
CXCL6→
PF4V1→
CXCL1→
←PF4
←PPBP
←CXCL5
←CXCL3
←PPBPP2
←CXCL2
MTHFD2L→
EPGN→
EREG→
AREG→
74.4
74.6
74.8
75
75.2
Position on chr4 (Mb)

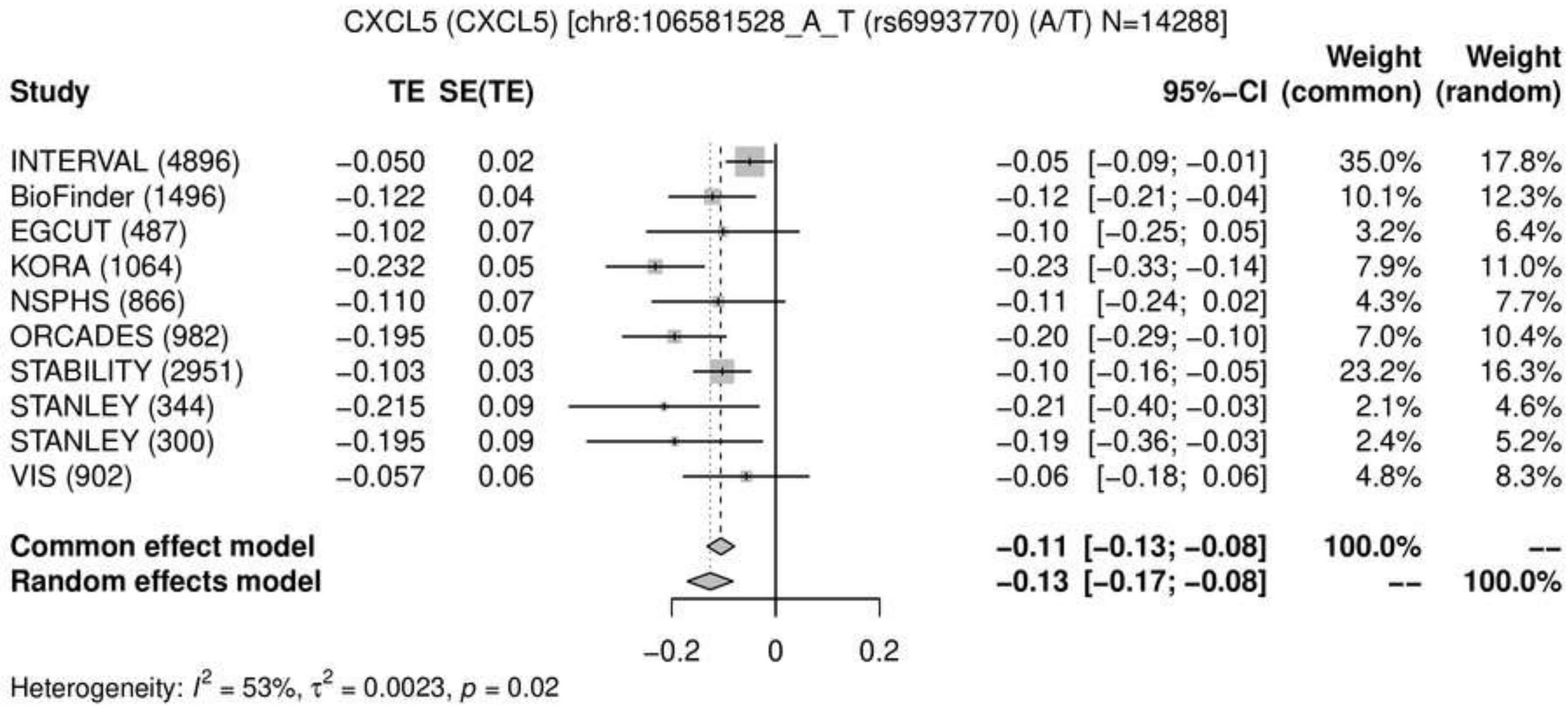
CXCL5 (CXCL5) [chr8:106581528_A_T (rs6993770) (A/T) N=14288]
Study
TE
SE(TE)
95%–CI
Weight (common)
Weight (random)
INTERVAL (4896) –0.050 0.02 –0.05 [–0.09; –0.01] 35.0% 17.8%
BioFinder (1496) –0.122 0.04 –0.12 [–0.21; –0.04] 10.1% 12.3%
EGCUT (487) –0.102 0.07 –0.10 [–0.25; 0.05] 3.2% 6.4%
KORA (1064) –0.232 0.05 –0.23 [–0.33; –0.14] 7.9% 11.0%
NSPHS (866) –0.110 0.07 –0.11 [–0.24; 0.02] 4.3% 7.7%
ORCADES (982) –0.195 0.05 –0.20 [–0.29; –0.10] 7.0% 10.4%
STABILITY (2951) –0.103 0.03 –0.10 [–0.16; –0.05] 23.2% 16.3%
STANLEY (344) –0.215 0.09 –0.21 [–0.40; –0.03] 2.1% 4.6%
STANLEY (300) –0.195 0.09 –0.19 [–0.36; –0.03] 2.4% 5.2%
VIS (902) –0.057 0.06 –0.06 [–0.18; 0.06] 4.8% 8.3%
Common effect model –0.11 [–0.13; –0.08] 100.0% --
Random effects model –0.13 [–0.17; –0.08] -- 100.0%
–0.2
0
0.2
Heterogeneity: I^2 = 53%, τ^2 = 0.0023, p = 0.02

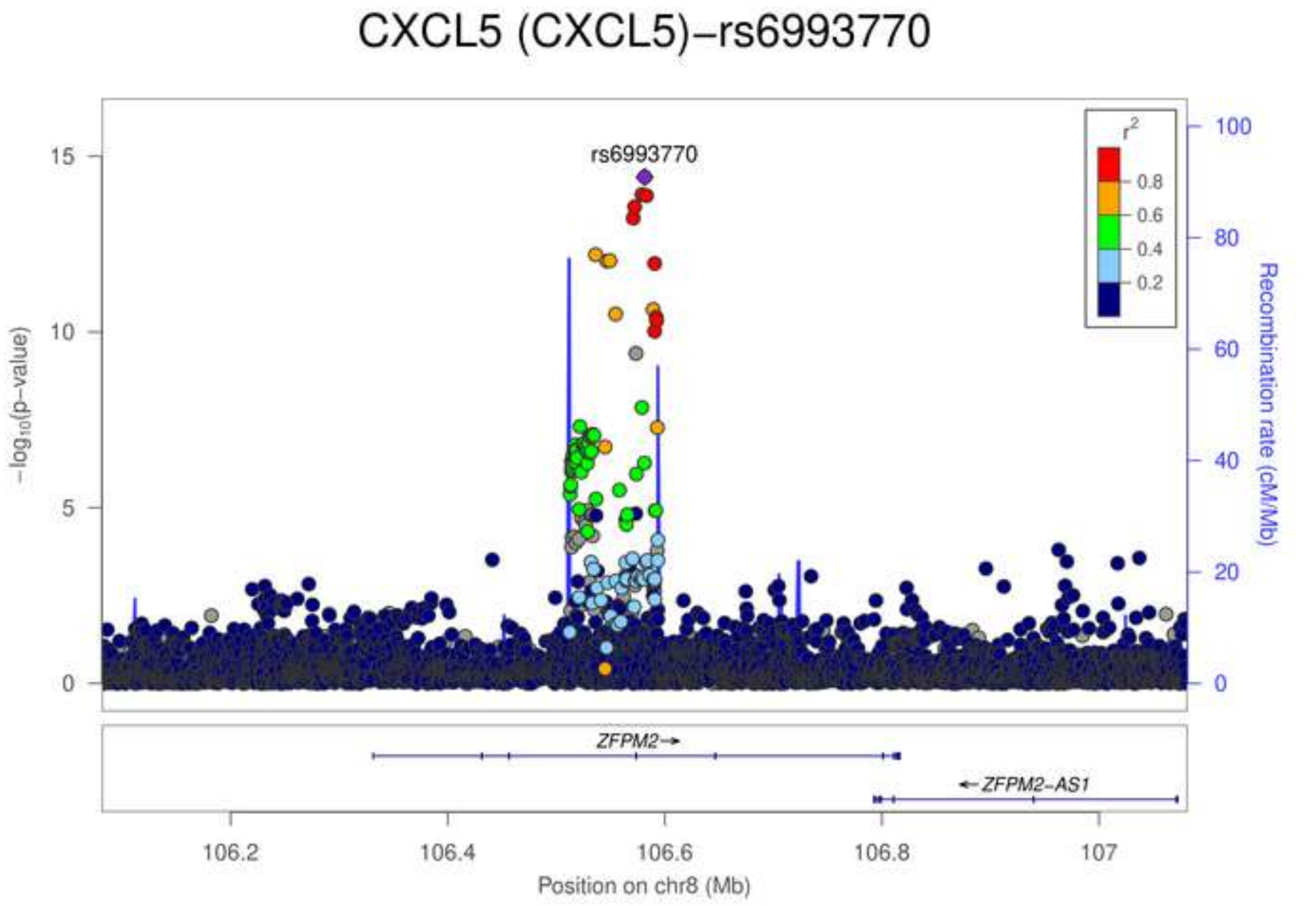
CXCL5 (CXCL5)–rs6993770
rs6993770
r^2
0.8
0.6
0.4
0.2
15
10
5
0
100
80
60
40
20
0
$-\log_{10}$(p–value)
Recombination rate (cM/Mb)
ZFPM2→
←ZFPM2-AS1
106.2
106.4
106.6
106.8
107
Position on chr8 (Mb)

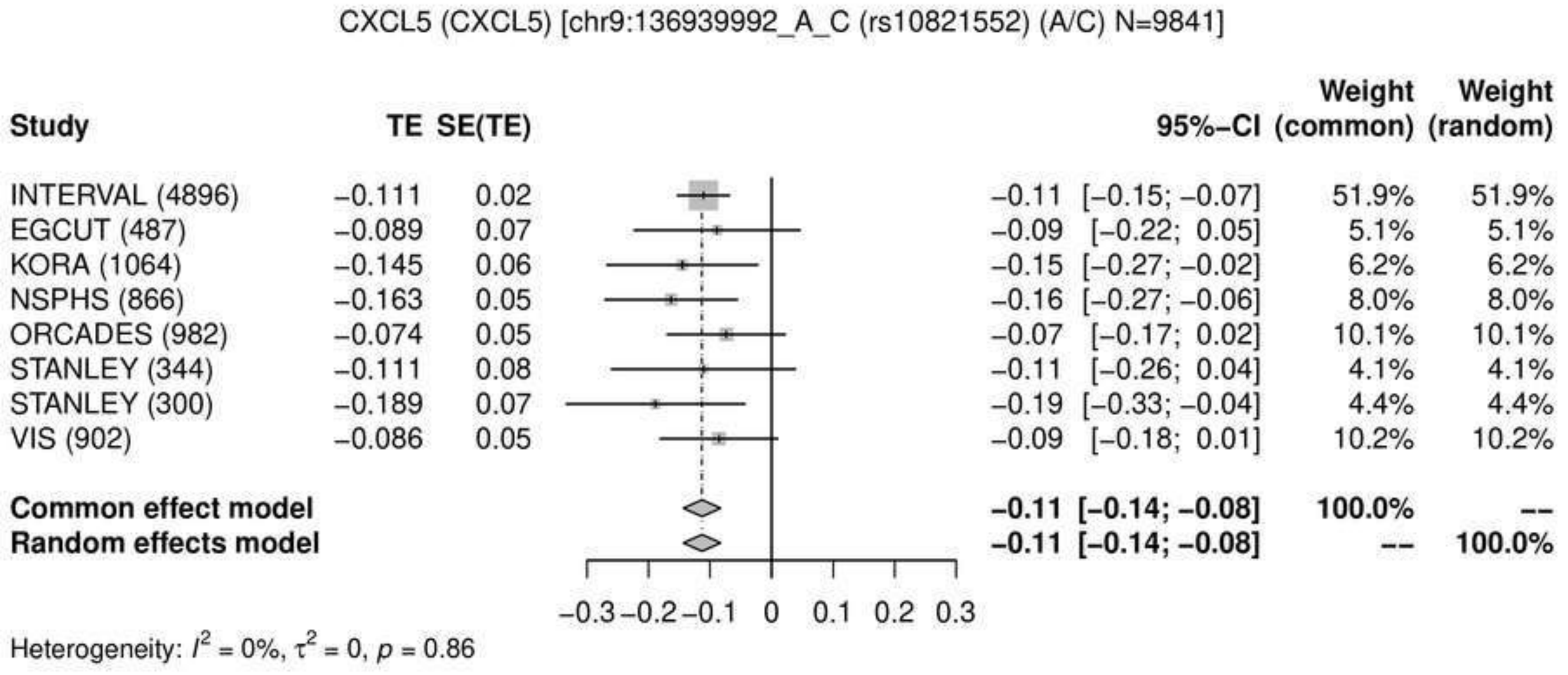

CXCL5 (CXCL5) [chr9:136939992_A_C (rs10821552) (A/C) N=9841]
Study
TE SE(TE)
95%-CI
Weight (common)
Weight (random)
INTERVAL (4896) −0.111 0.02 −0.11 [−0.15; −0.07] 51.9% 51.9%
EGCUT (487) −0.089 0.07 −0.09 [−0.22; 0.05] 5.1% 5.1%
KORA (1064) −0.145 0.06 −0.15 [−0.27; −0.02] 6.2% 6.2%
NSPHS (866) −0.163 0.05 −0.16 [−0.27; −0.06] 8.0% 8.0%
ORCADES (982) −0.074 0.05 −0.07 [−0.17; 0.02] 10.1% 10.1%
STANLEY (344) −0.111 0.08 −0.11 [−0.26; 0.04] 4.1% 4.1%
STANLEY (300) −0.189 0.07 −0.19 [−0.33; −0.04] 4.4% 4.4%
VIS (902) −0.086 0.05 −0.09 [−0.18; 0.01] 10.2% 10.2%
Common effect model −0.11 [−0.14; −0.08] 100.0% --
Random effects model −0.11 [−0.14; −0.08] -- 100.0%
−0.3 −0.2 −0.1 0 0.1 0.2 0.3
Heterogeneity: I^2 = 0%, τ^2 = 0, p = 0.86

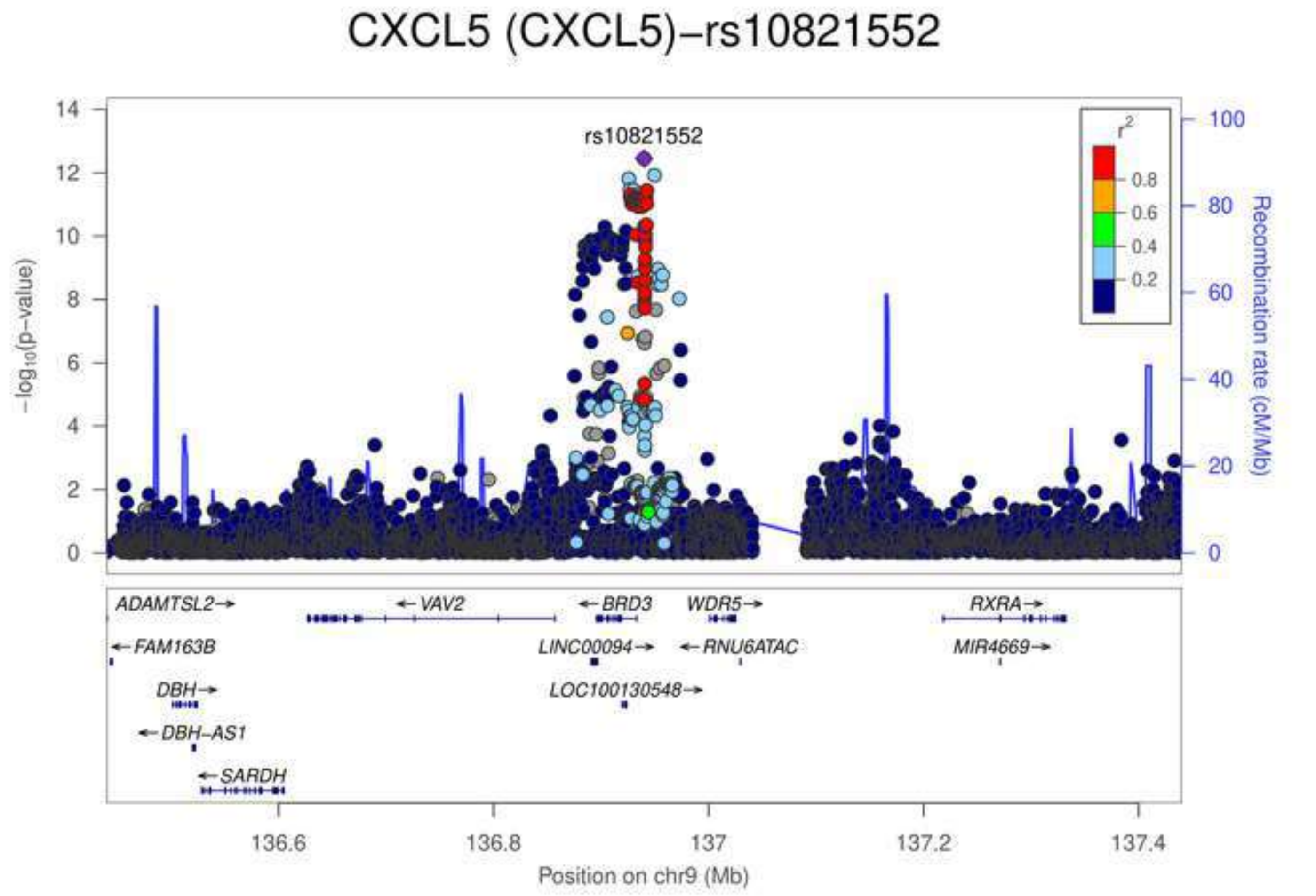

CXCL5 (CXCL5)−rs10821552
rs10821552
$-\log_{10}$(p-value)
Recombination rate (cM/Mb)
r^2
0.8
0.6
0.4
0.2
ADAMTSL2→
← VAV2
← BRD3
WDR5→
RXRA→
← FAM163B
LINC00094→
← RNU6ATAC
MIR4669→
DBH→
LOC100130548→
← DBH-AS1
← SARDH
136.6 136.8 137 137.2 137.4
Position on chr9 (Mb)

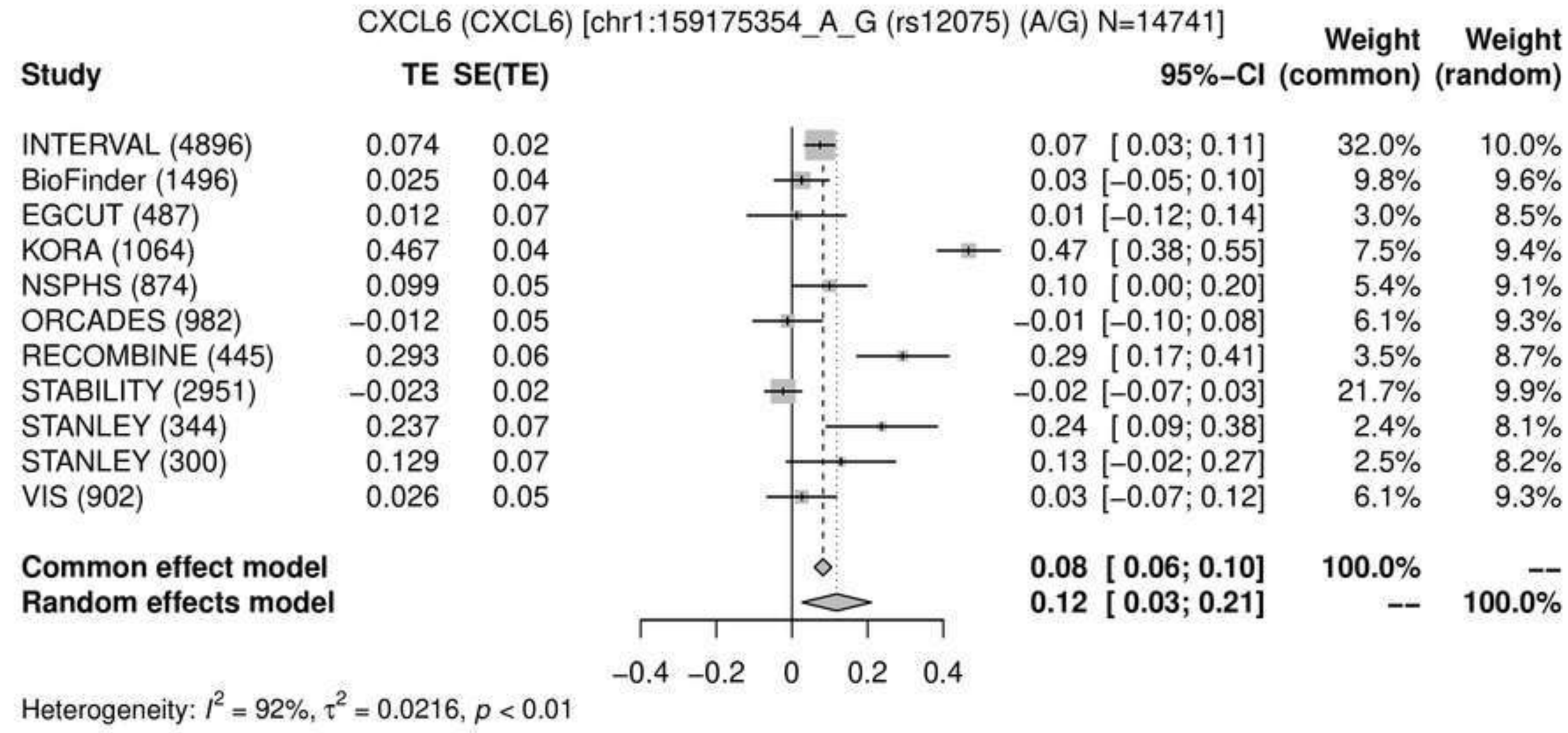
CXCL6 (CXCL6) [chr1:159175354_A_G (rs12075) (A/G) N=14741]
Study TE SE(TE) 95%-CI Weight (common) Weight (random)
INTERVAL (4896) 0.074 0.02 0.07 [0.03; 0.11] 32.0% 10.0%
BioFinder (1496) 0.025 0.04 0.03 [−0.05; 0.10] 9.8% 9.6%
EGCUT (487) 0.012 0.07 0.01 [−0.12; 0.14] 3.0% 8.5%
KORA (1064) 0.467 0.04 0.47 [0.38; 0.55] 7.5% 9.4%
NSPHS (874) 0.099 0.05 0.10 [0.00; 0.20] 5.4% 9.1%
ORCADES (982) −0.012 0.05 −0.01 [−0.10; 0.08] 6.1% 9.3%
RECOMBINE (445) 0.293 0.06 0.29 [0.17; 0.41] 3.5% 8.7%
STABILITY (2951) −0.023 0.02 −0.02 [−0.07; 0.03] 21.7% 9.9%
STANLEY (344) 0.237 0.07 0.24 [0.09; 0.38] 2.4% 8.1%
STANLEY (300) 0.129 0.07 0.13 [−0.02; 0.27] 2.5% 8.2%
VIS (902) 0.026 0.05 0.03 [−0.07; 0.12] 6.1% 9.3%
Common effect model 0.08 [0.06; 0.10] 100.0% --
Random effects model 0.12 [0.03; 0.21] -- 100.0%
−0.4 −0.2 0 0.2 0.4
Heterogeneity: I^2 = 92%, τ^2 = 0.0216, p < 0.01

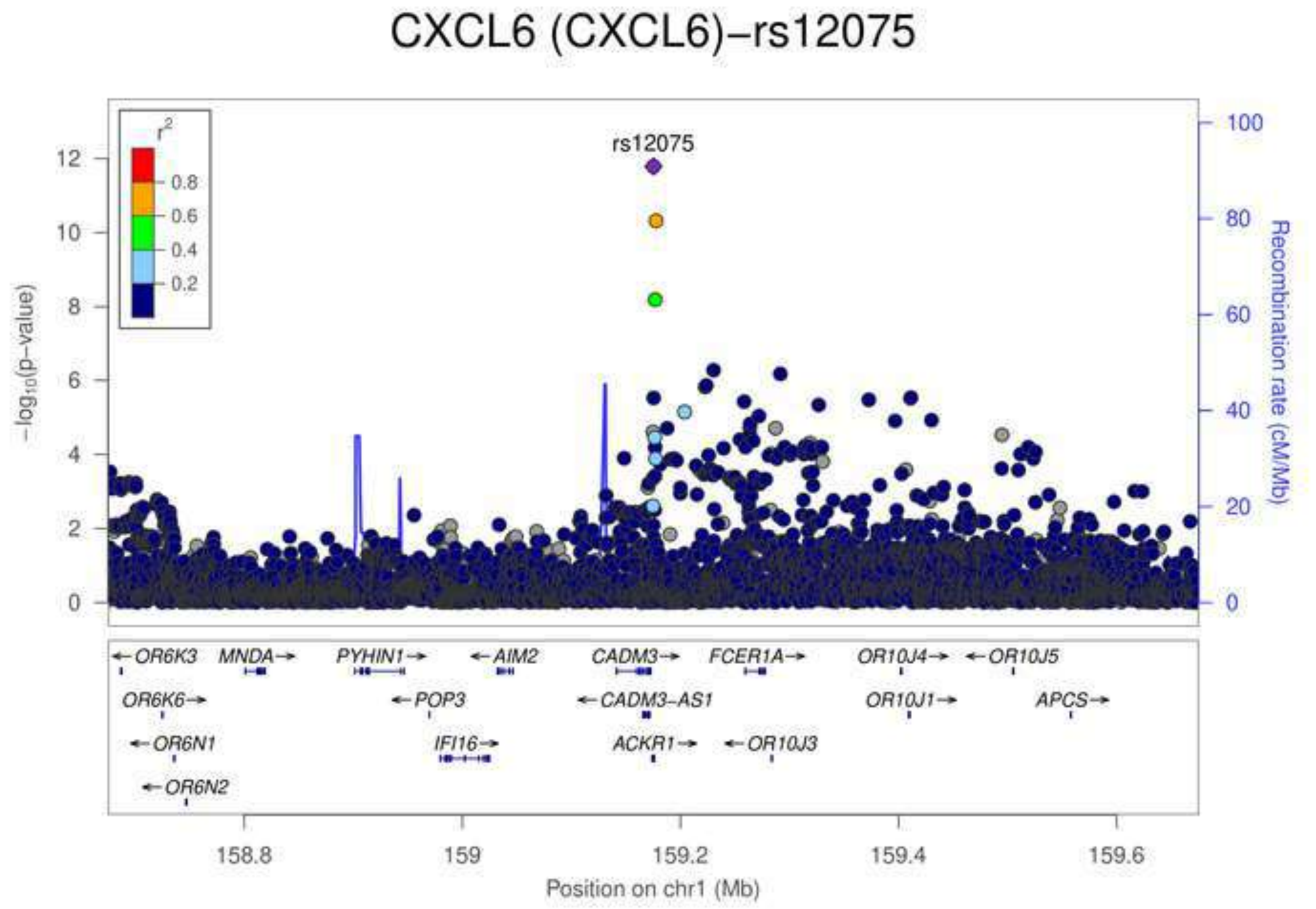
CXCL6 (CXCL6)−rs12075
rs12075
r^2
0.8
0.6
0.4
0.2
$-\log_{10}$(p-value)
Recombination rate (cM/Mb)
OR6K3 MNDA PYHIN1 AIM2 CADM3 FCER1A OR10J4 OR10J5
OR6K6 POP3 CADM3-AS1 OR10J1 APCS
OR6N1 IFI16 ACKR1 OR10J3
OR6N2
158.8 159 159.2 159.4 159.6
Position on chr1 (Mb)

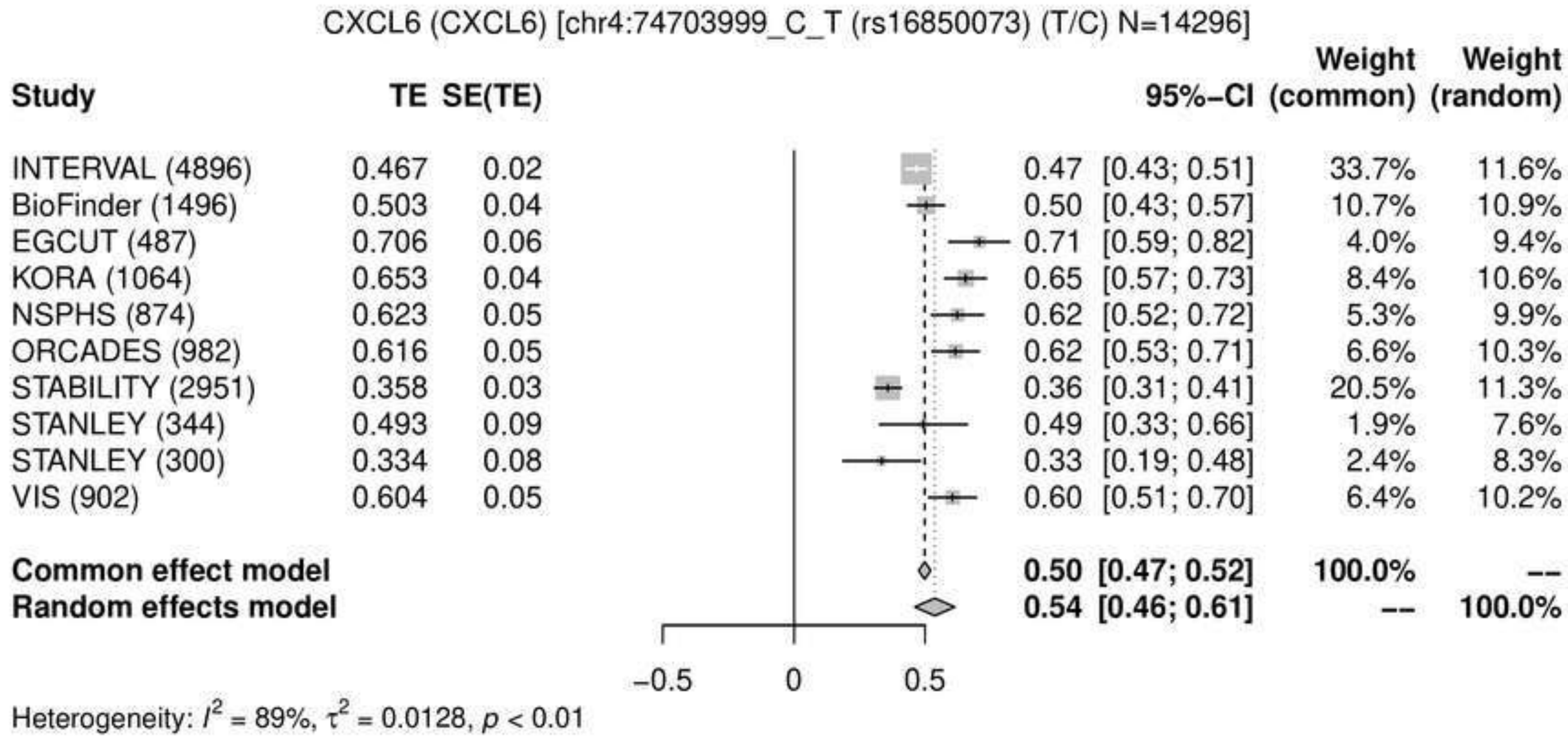
CXCL6 (CXCL6) [chr4:74703999_C_T (rs16850073) (T/C) N=14296]
Study TE SE(TE) 95%−CI Weight (common) Weight (random)
INTERVAL (4896) 0.467 0.02 0.47 [0.43; 0.51] 33.7% 11.6%
BioFinder (1496) 0.503 0.04 0.50 [0.43; 0.57] 10.7% 10.9%
EGCUT (487) 0.706 0.06 0.71 [0.59; 0.82] 4.0% 9.4%
KORA (1064) 0.653 0.04 0.65 [0.57; 0.73] 8.4% 10.6%
NSPHS (874) 0.623 0.05 0.62 [0.52; 0.72] 5.3% 9.9%
ORCADES (982) 0.616 0.05 0.62 [0.53; 0.71] 6.6% 10.3%
STABILITY (2951) 0.358 0.03 0.36 [0.31; 0.41] 20.5% 11.3%
STANLEY (344) 0.493 0.09 0.49 [0.33; 0.66] 1.9% 7.6%
STANLEY (300) 0.334 0.08 0.33 [0.19; 0.48] 2.4% 8.3%
VIS (902) 0.604 0.05 0.60 [0.51; 0.70] 6.4% 10.2%
Common effect model 0.50 [0.47; 0.52] 100.0% --
Random effects model 0.54 [0.46; 0.61] -- 100.0%
−0.5
0
0.5
Heterogeneity: I^2 = 89%, τ^2 = 0.0128, p < 0.01

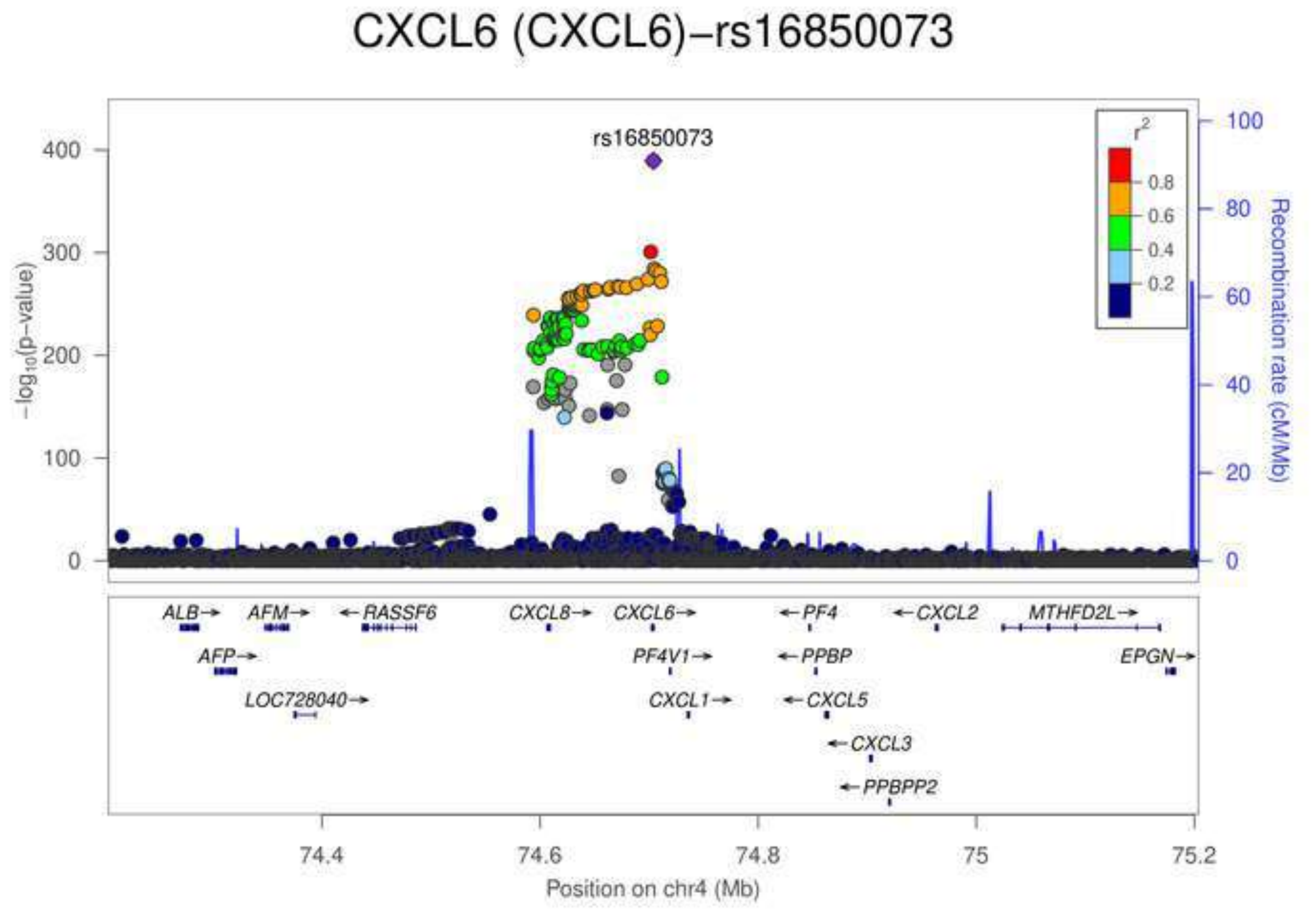
CXCL6 (CXCL6)−rs16850073
rs16850073
$-\log_{10}$(p-value)
0
100
200
300
400
Recombination rate (cM/Mb)
0
20
40
60
80
100
r^2
0.8
0.6
0.4
0.2
ALB→
AFM→
←RASSF6
CXCL8→
CXCL6→
←PF4
←CXCL2
MTHFD2L→
AFP→
PF4V1→
←PPBP
EPGN→
LOC728040→
CXCL1→
←CXCL5
←CXCL3
←PPBPP2
74.4
74.6
74.8
75
75.2
Position on chr4 (Mb)

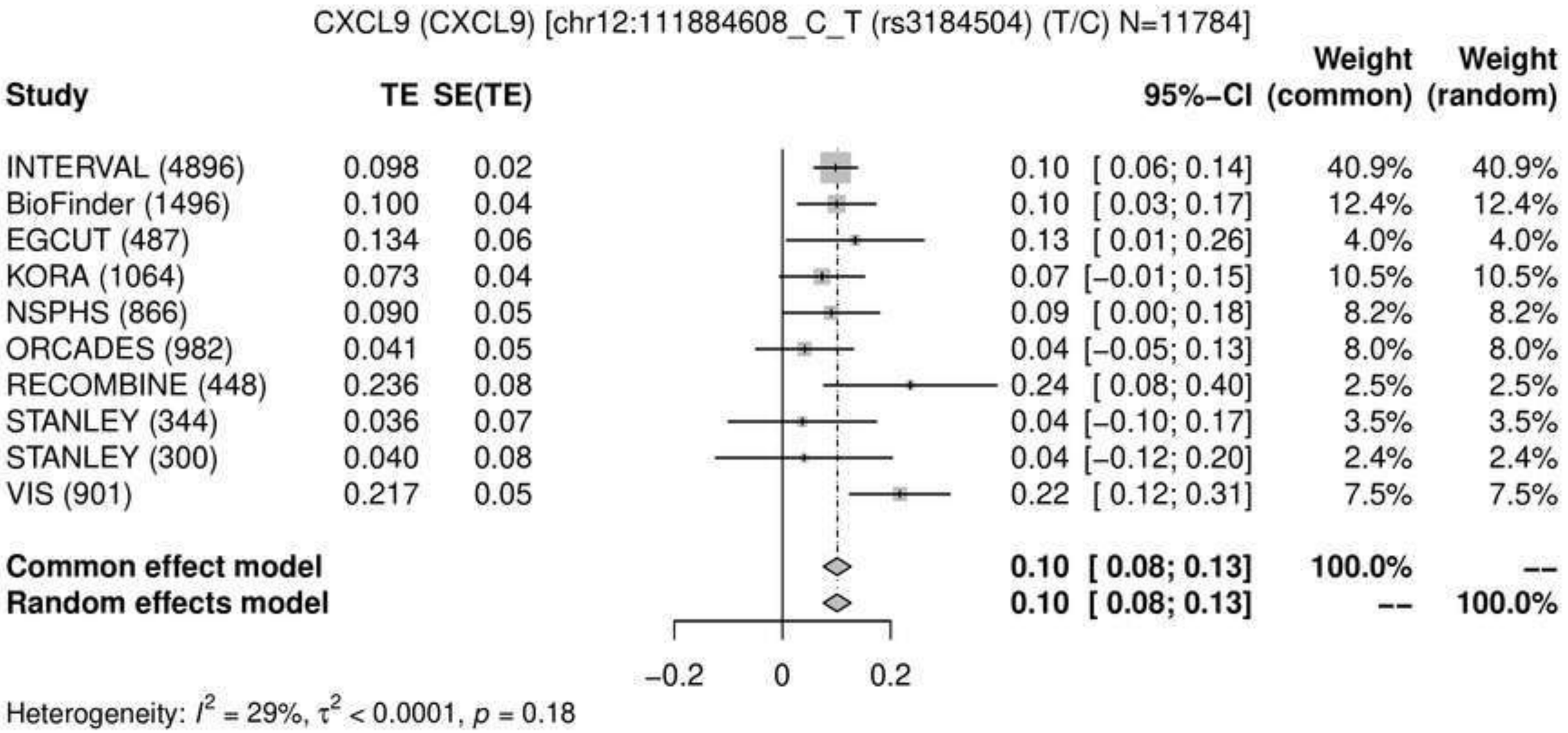
CXCL9 (CXCL9) [chr12:111884608_C_T (rs3184504) (T/C) N=11784]
Study TE SE(TE) 95%−CI Weight (common) Weight (random)
INTERVAL (4896) 0.098 0.02 0.10 [0.06; 0.14] 40.9% 40.9%
BioFinder (1496) 0.100 0.04 0.10 [0.03; 0.17] 12.4% 12.4%
EGCUT (487) 0.134 0.06 0.13 [0.01; 0.26] 4.0% 4.0%
KORA (1064) 0.073 0.04 0.07 [−0.01; 0.15] 10.5% 10.5%
NSPHS (866) 0.090 0.05 0.09 [0.00; 0.18] 8.2% 8.2%
ORCADES (982) 0.041 0.05 0.04 [−0.05; 0.13] 8.0% 8.0%
RECOMBINE (448) 0.236 0.08 0.24 [0.08; 0.40] 2.5% 2.5%
STANLEY (344) 0.036 0.07 0.04 [−0.10; 0.17] 3.5% 3.5%
STANLEY (300) 0.040 0.08 0.04 [−0.12; 0.20] 2.4% 2.4%
VIS (901) 0.217 0.05 0.22 [0.12; 0.31] 7.5% 7.5%
Common effect model 0.10 [0.08; 0.13] 100.0% --
Random effects model 0.10 [0.08; 0.13] -- 100.0%
−0.2 0 0.2
Heterogeneity: I^2 = 29%, τ^2 < 0.0001, p = 0.18

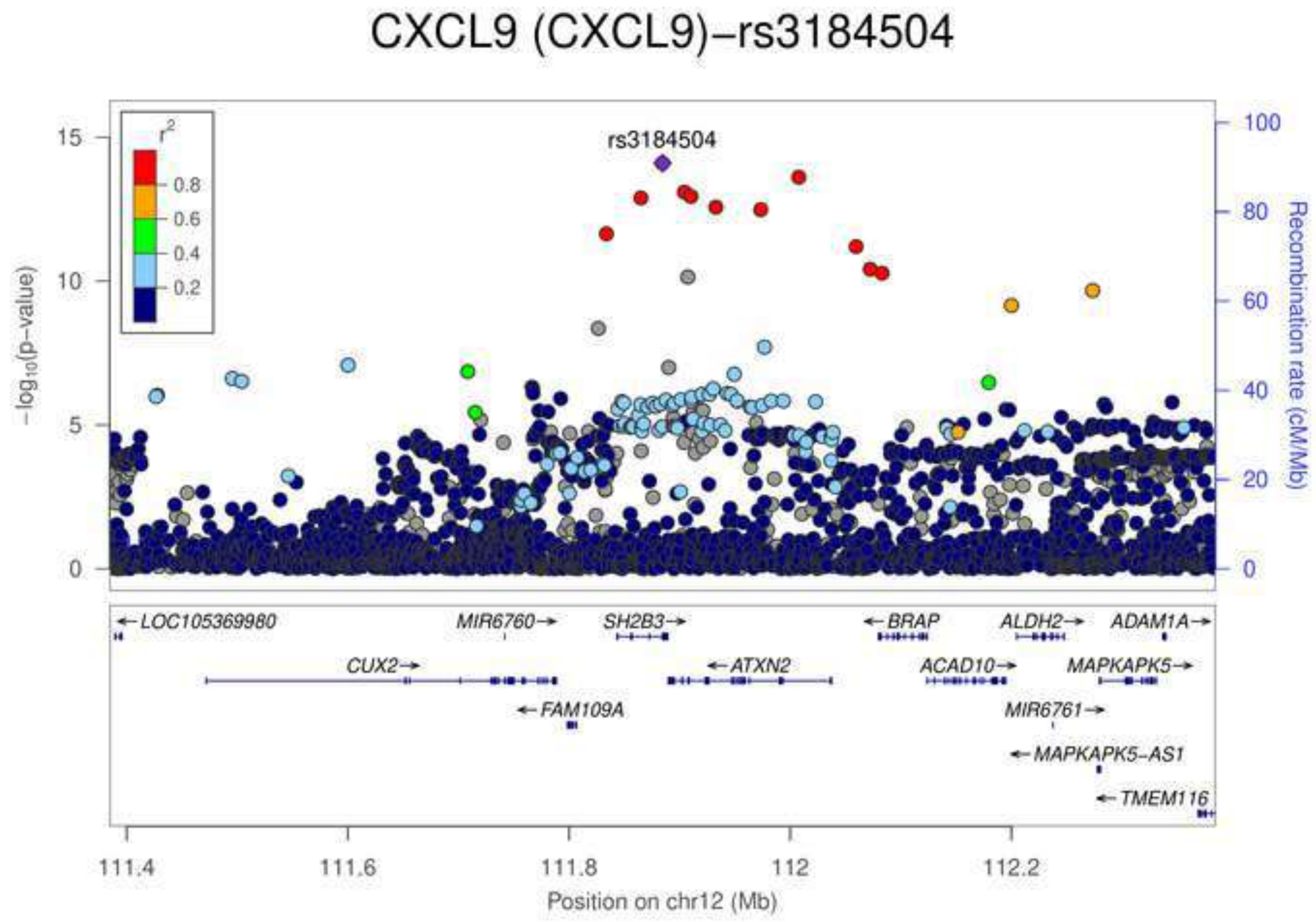
CXCL9 (CXCL9)−rs3184504
rs3184504
r^2
0.8
0.6
0.4
0.2
−log10(p−value)
Recombination rate (cM/Mb)
LOC105369980
MIR6760
SH2B3
BRAP
ALDH2
ADAM1A
CUX2
ATXN2
ACAD10
MAPKAPK5
FAM109A
MIR6761
MAPKAPK5-AS1
TMEM116
111.4
111.6
111.8
112
112.2
Position on chr12 (Mb)

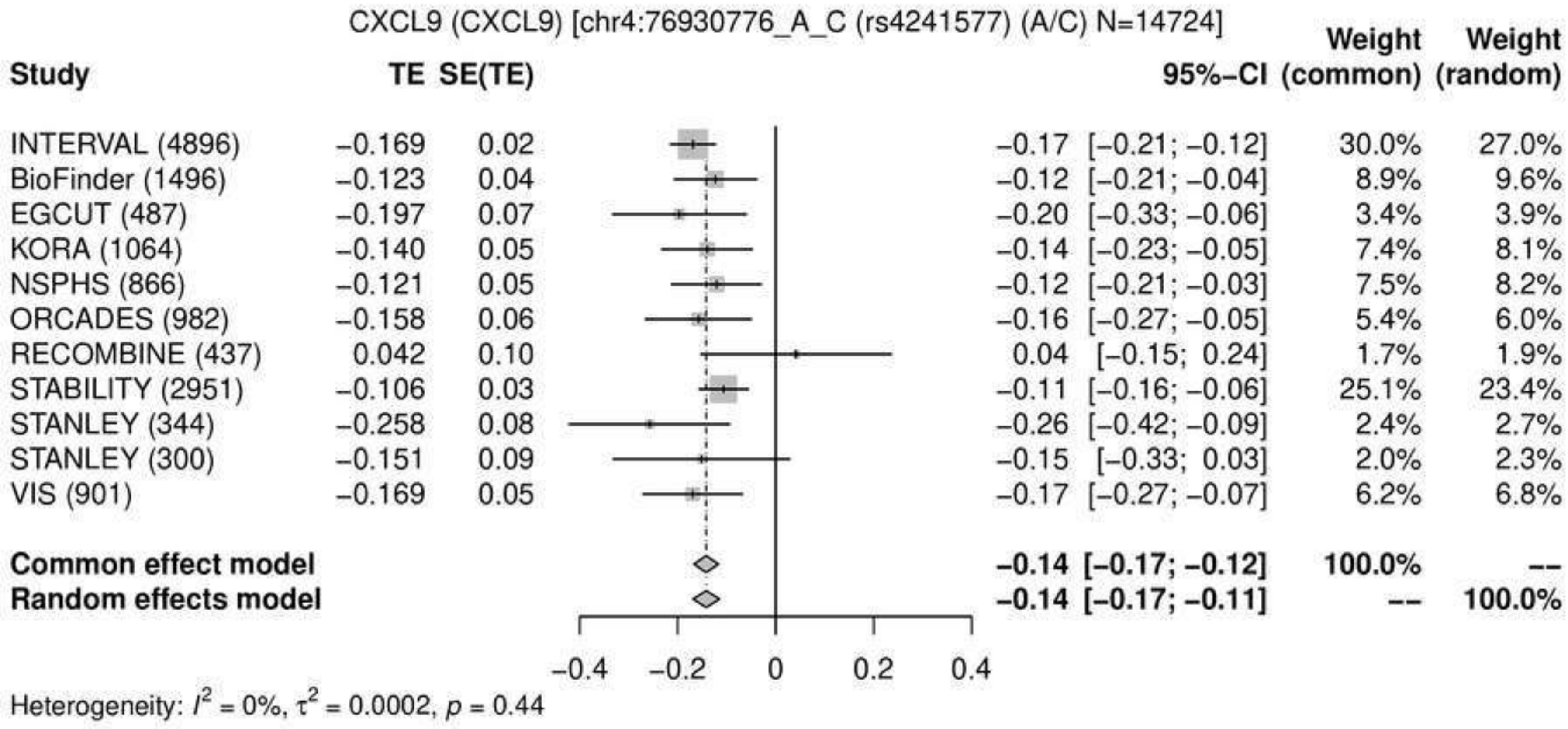
CXCL9 (CXCL9) [chr4:76930776_A_C (rs4241577) (A/C) N=14724]
Study | TE | SE(TE) | 95%-CI | Weight (common) | Weight (random)
INTERVAL (4896) | −0.169 | 0.02 | −0.17 [−0.21; −0.12] | 30.0% | 27.0%
BioFinder (1496) | −0.123 | 0.04 | −0.12 [−0.21; −0.04] | 8.9% | 9.6%
EGCUT (487) | −0.197 | 0.07 | −0.20 [−0.33; −0.06] | 3.4% | 3.9%
KORA (1064) | −0.140 | 0.05 | −0.14 [−0.23; −0.05] | 7.4% | 8.1%
NSPHS (866) | −0.121 | 0.05 | −0.12 [−0.21; −0.03] | 7.5% | 8.2%
ORCADES (982) | −0.158 | 0.06 | −0.16 [−0.27; −0.05] | 5.4% | 6.0%
RECOMBINE (437) | 0.042 | 0.10 | 0.04 [−0.15; 0.24] | 1.7% | 1.9%
STABILITY (2951) | −0.106 | 0.03 | −0.11 [−0.16; −0.06] | 25.1% | 23.4%
STANLEY (344) | −0.258 | 0.08 | −0.26 [−0.42; −0.09] | 2.4% | 2.7%
STANLEY (300) | −0.151 | 0.09 | −0.15 [−0.33; 0.03] | 2.0% | 2.3%
VIS (901) | −0.169 | 0.05 | −0.17 [−0.27; −0.07] | 6.2% | 6.8%
Common effect model | −0.14 [−0.17; −0.12] | 100.0% | --
Random effects model | −0.14 [−0.17; −0.11] | -- | 100.0%
−0.4 −0.2 0 0.2 0.4
Heterogeneity: I^2 = 0%, τ^2 = 0.0002, p = 0.44

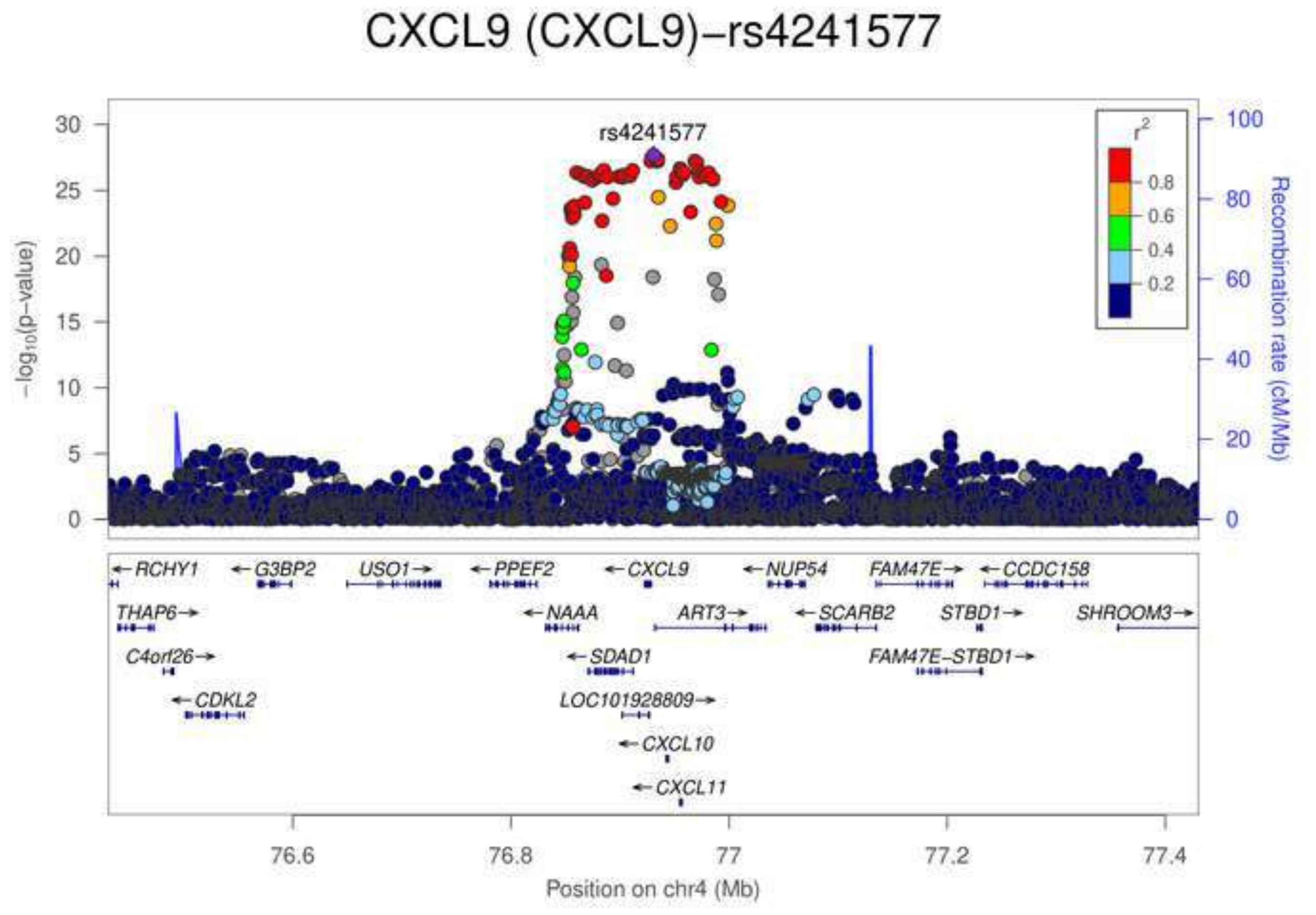
CXCL9 (CXCL9)−rs4241577
rs4241577
$-\log_{10}$(p-value)
Recombination rate (cM/Mb)
r^2
0.8
0.6
0.4
0.2
RCHY1
G3BP2
USO1
PPEF2
CXCL9
NUP54
FAM47E
CCDC158
THAP6
NAAA
ART3
SCARB2
STBD1
SHROOM3
C4orf26
SDAD1
FAM47E−STBD1
CDKL2
LOC101928809
CXCL10
CXCL11
Position on chr4 (Mb)

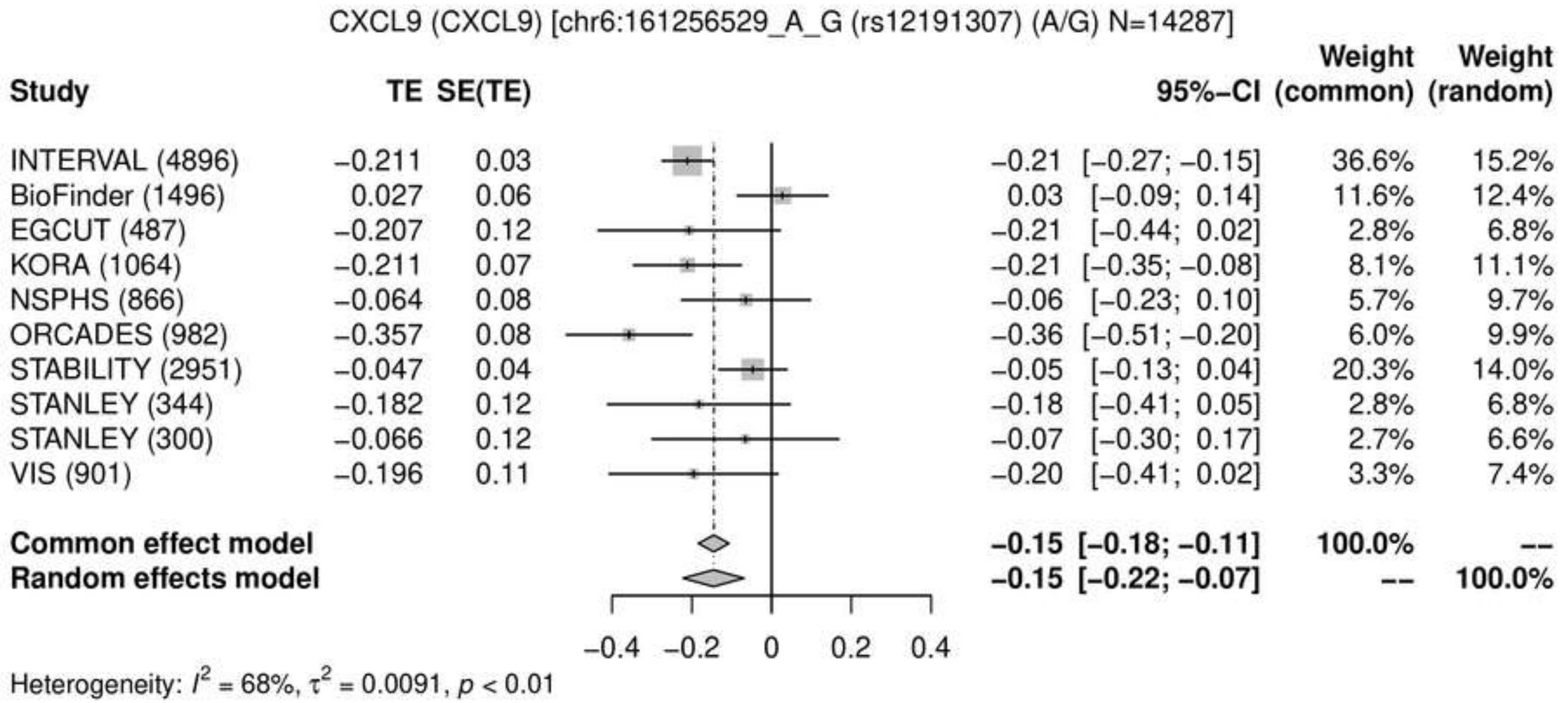
CXCL9 (CXCL9) [chr6:161256529_A_G (rs12191307) (A/G) N=14287]
Study | TE | SE(TE) | 95%-CI | Weight (common) | Weight (random)
INTERVAL (4896) | −0.211 | 0.03 | −0.21 [−0.27; −0.15] | 36.6% | 15.2%
BioFinder (1496) | 0.027 | 0.06 | 0.03 [−0.09; 0.14] | 11.6% | 12.4%
EGCUT (487) | −0.207 | 0.12 | −0.21 [−0.44; 0.02] | 2.8% | 6.8%
KORA (1064) | −0.211 | 0.07 | −0.21 [−0.35; −0.08] | 8.1% | 11.1%
NSPHS (866) | −0.064 | 0.08 | −0.06 [−0.23; 0.10] | 5.7% | 9.7%
ORCADES (982) | −0.357 | 0.08 | −0.36 [−0.51; −0.20] | 6.0% | 9.9%
STABILITY (2951) | −0.047 | 0.04 | −0.05 [−0.13; 0.04] | 20.3% | 14.0%
STANLEY (344) | −0.182 | 0.12 | −0.18 [−0.41; 0.05] | 2.8% | 6.8%
STANLEY (300) | −0.066 | 0.12 | −0.07 [−0.30; 0.17] | 2.7% | 6.6%
VIS (901) | −0.196 | 0.11 | −0.20 [−0.41; 0.02] | 3.3% | 7.4%
Common effect model | −0.15 [−0.18; −0.11] | 100.0% | --
Random effects model | −0.15 [−0.22; −0.07] | -- | 100.0%
−0.4 −0.2 0 0.2 0.4
Heterogeneity: $I^2 = 68\%$, $\tau^2 = 0.0091$, $p < 0.01$

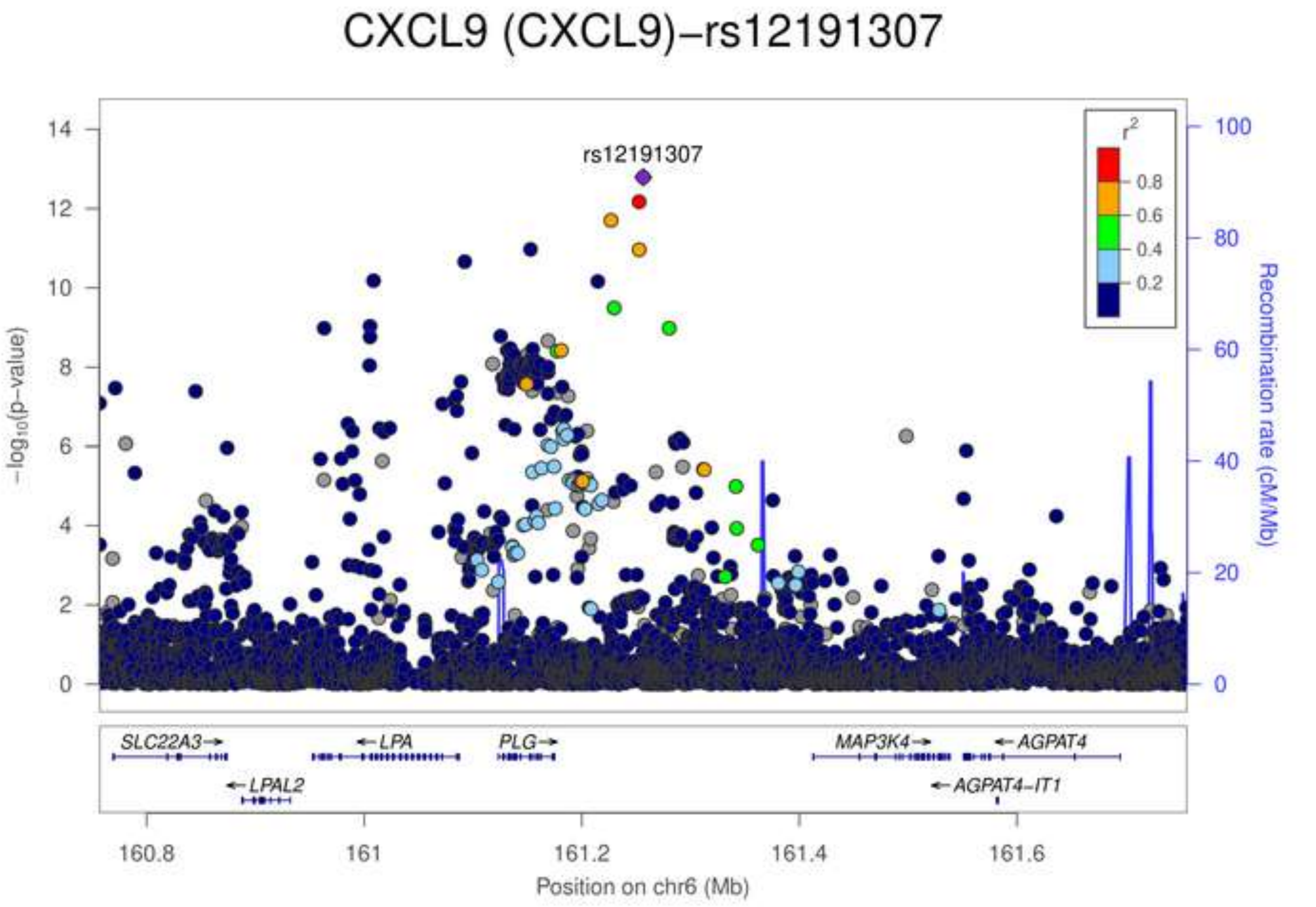
CXCL9 (CXCL9)−rs12191307
rs12191307
$-\log_{10}$(p-value)
Recombination rate (cM/Mb)
r^2
0.8
0.6
0.4
0.2
SLC22A3
LPAL2
LPA
PLG
MAP3K4
AGPAT4
AGPAT4-IT1
160.8
161
161.2
161.4
161.6
Position on chr6 (Mb)

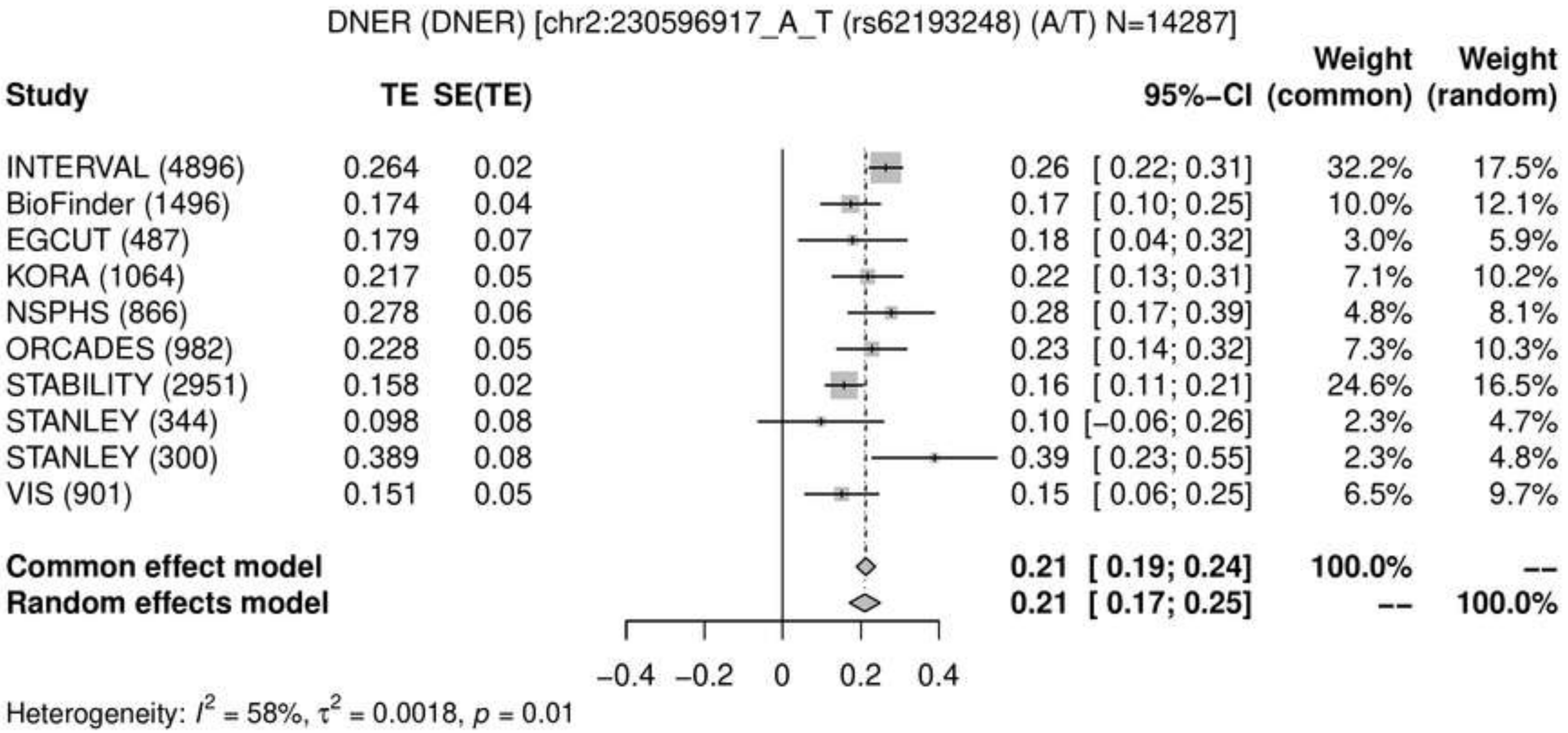
DNER (DNER) [chr2:230596917_A_T (rs62193248) (A/T) N=14287]
Study TE SE(TE) 95%−CI Weight (common) Weight (random)
INTERVAL (4896) 0.264 0.02 0.26 [0.22; 0.31] 32.2% 17.5%
BioFinder (1496) 0.174 0.04 0.17 [0.10; 0.25] 10.0% 12.1%
EGCUT (487) 0.179 0.07 0.18 [0.04; 0.32] 3.0% 5.9%
KORA (1064) 0.217 0.05 0.22 [0.13; 0.31] 7.1% 10.2%
NSPHS (866) 0.278 0.06 0.28 [0.17; 0.39] 4.8% 8.1%
ORCADES (982) 0.228 0.05 0.23 [0.14; 0.32] 7.3% 10.3%
STABILITY (2951) 0.158 0.02 0.16 [0.11; 0.21] 24.6% 16.5%
STANLEY (344) 0.098 0.08 0.10 [−0.06; 0.26] 2.3% 4.7%
STANLEY (300) 0.389 0.08 0.39 [0.23; 0.55] 2.3% 4.8%
VIS (901) 0.151 0.05 0.15 [0.06; 0.25] 6.5% 9.7%
Common effect model 0.21 [0.19; 0.24] 100.0% --
Random effects model 0.21 [0.17; 0.25] -- 100.0%
−0.4 −0.2 0 0.2 0.4
Heterogeneity: I^2 = 58%, τ^2 = 0.0018, p = 0.01

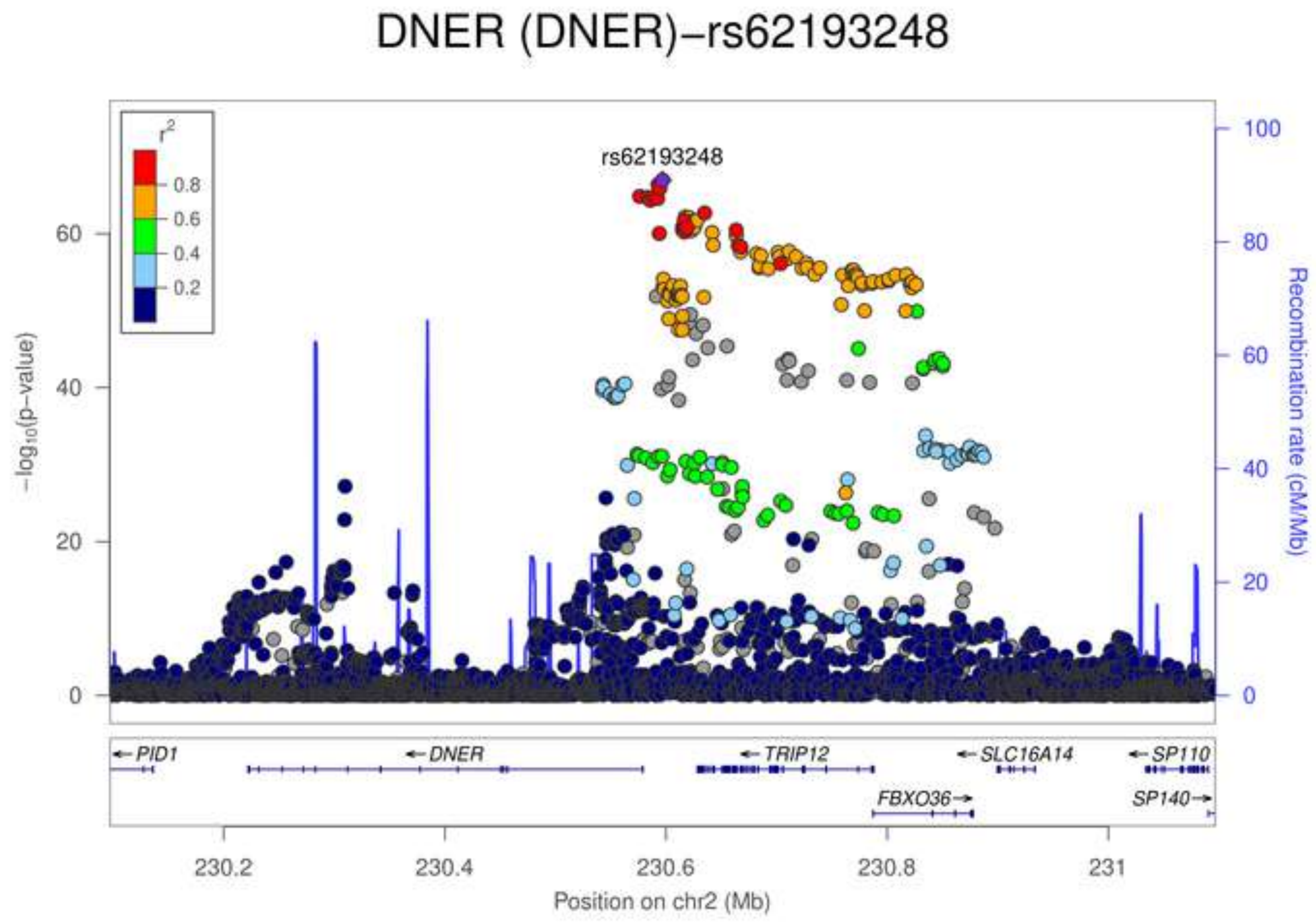
DNER (DNER)−rs62193248
rs62193248
r^2
0.8
0.6
0.4
0.2
$-\log_{10}$(p−value)
Recombination rate (cM/Mb)
0 20 40 60
0 20 40 60 80 100
PID1
DNER
TRIP12
SLC16A14
SP110
FBXO36
SP140
230.2 230.4 230.6 230.8 231
Position on chr2 (Mb)

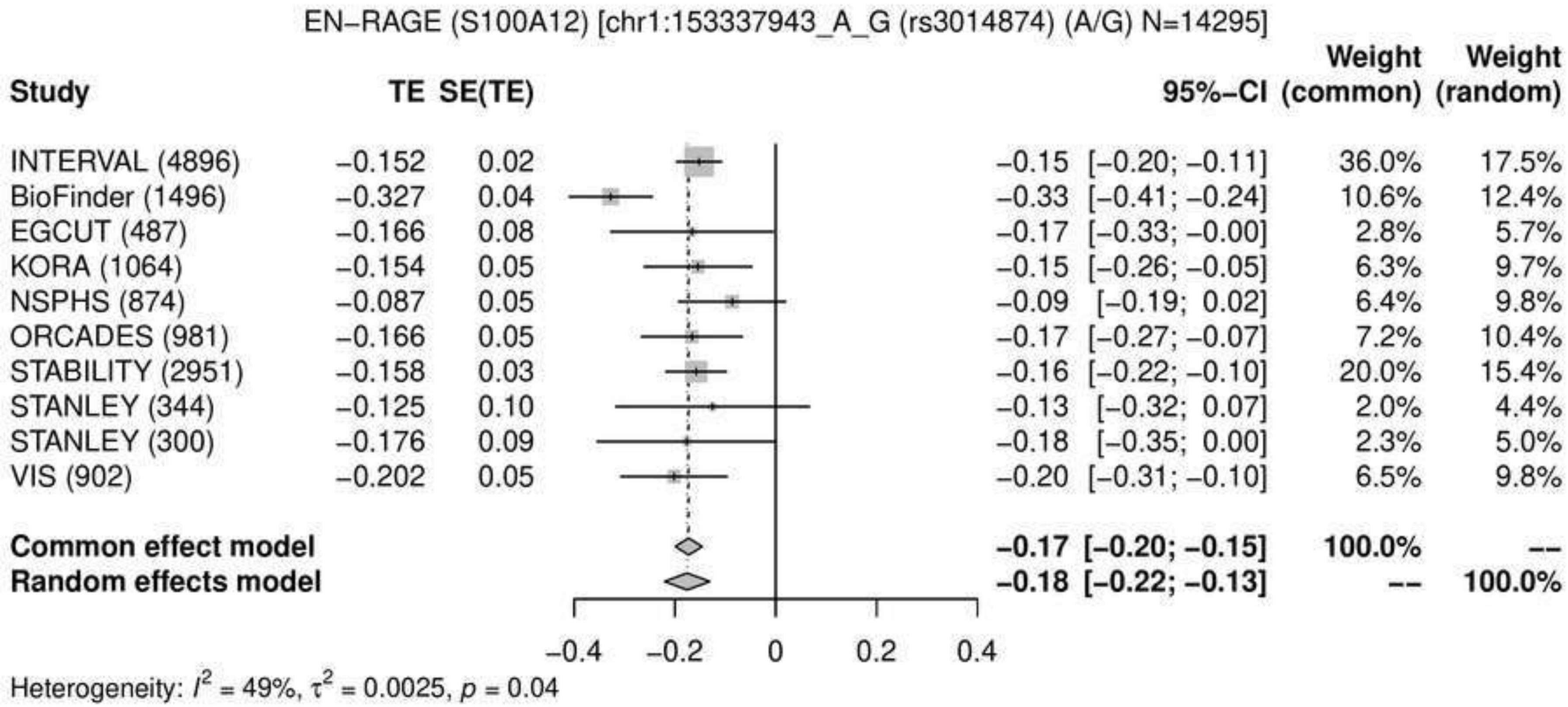
EN−RAGE (S100A12) [chr1:153337943_A_G (rs3014874) (A/G) N=14295]
Study
TE
SE(TE)
95%−CI
Weight (common)
Weight (random)
INTERVAL (4896) −0.152 0.02 −0.15 [−0.20; −0.11] 36.0% 17.5%
BioFinder (1496) −0.327 0.04 −0.33 [−0.41; −0.24] 10.6% 12.4%
EGCUT (487) −0.166 0.08 −0.17 [−0.33; −0.00] 2.8% 5.7%
KORA (1064) −0.154 0.05 −0.15 [−0.26; −0.05] 6.3% 9.7%
NSPHS (874) −0.087 0.05 −0.09 [−0.19; 0.02] 6.4% 9.8%
ORCADES (981) −0.166 0.05 −0.17 [−0.27; −0.07] 7.2% 10.4%
STABILITY (2951) −0.158 0.03 −0.16 [−0.22; −0.10] 20.0% 15.4%
STANLEY (344) −0.125 0.10 −0.13 [−0.32; 0.07] 2.0% 4.4%
STANLEY (300) −0.176 0.09 −0.18 [−0.35; 0.00] 2.3% 5.0%
VIS (902) −0.202 0.05 −0.20 [−0.31; −0.10] 6.5% 9.8%
Common effect model −0.17 [−0.20; −0.15] 100.0% --
Random effects model −0.18 [−0.22; −0.13] -- 100.0%
−0.4 −0.2 0 0.2 0.4
Heterogeneity: I^2 = 49%, τ^2 = 0.0025, p = 0.04

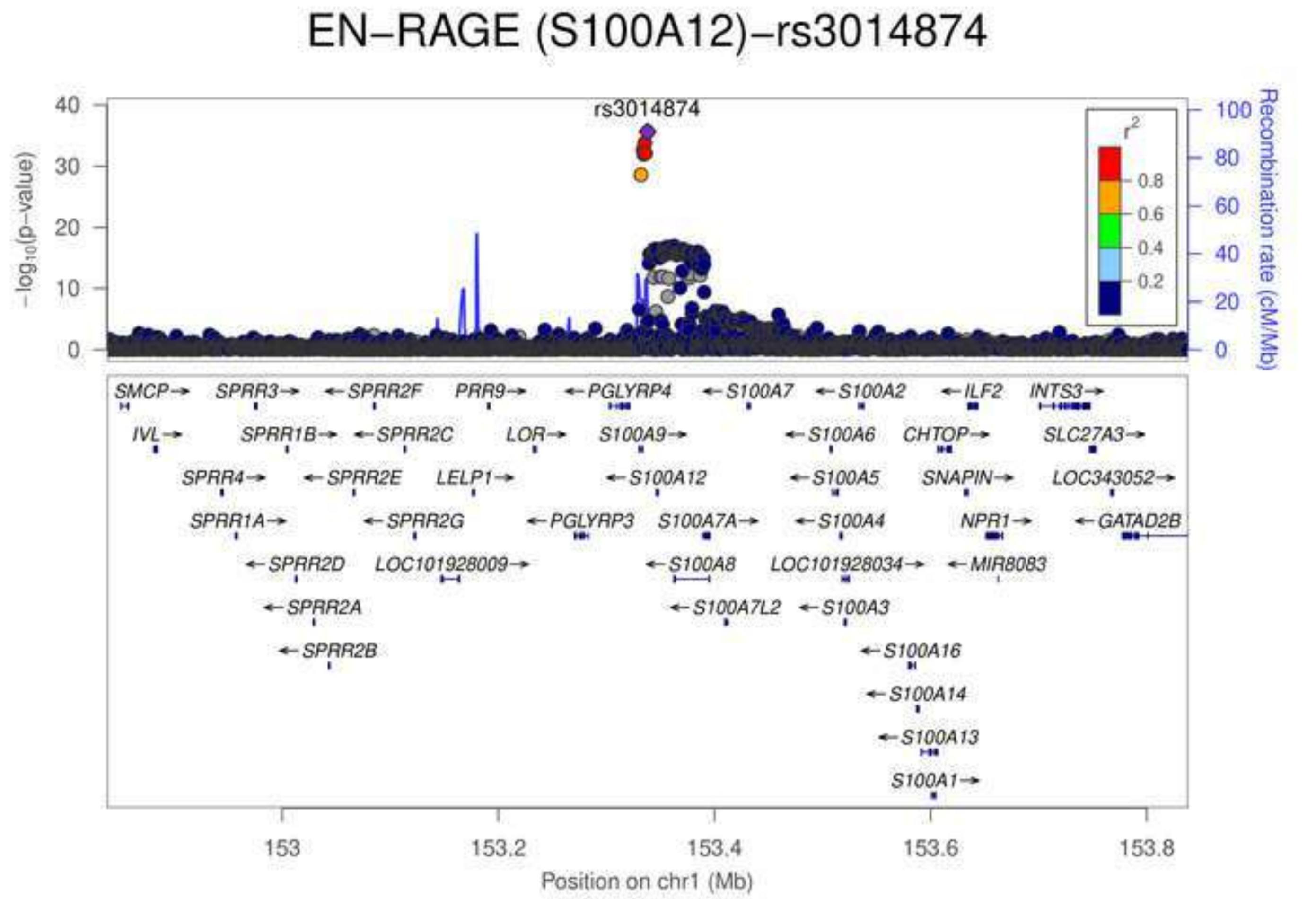
EN−RAGE (S100A12)−rs3014874
rs3014874
$-\log_{10}$(p−value)
Recombination rate (cM/Mb)
r^2
0.8
0.6
0.4
0.2
SMCP SPRR3 SPRR2F PRR9 PGLYRP4 S100A7 S100A2 ILF2 INTS3
IVL SPRR1B SPRR2C LOR S100A9 S100A6 CHTOP SLC27A3
SPRR4 SPRR2E LELP1 S100A12 S100A5 SNAPIN LOC343052
SPRR1A SPRR2G PGLYRP3 S100A7A S100A4 NPR1 GATAD2B
SPRR2D LOC101928009 S100A8 LOC101928034 MIR8083
SPRR2A S100A7L2 S100A3
SPRR2B S100A16
S100A14
S100A13
S100A1
153 153.2 153.4 153.6 153.8
Position on chr1 (Mb)

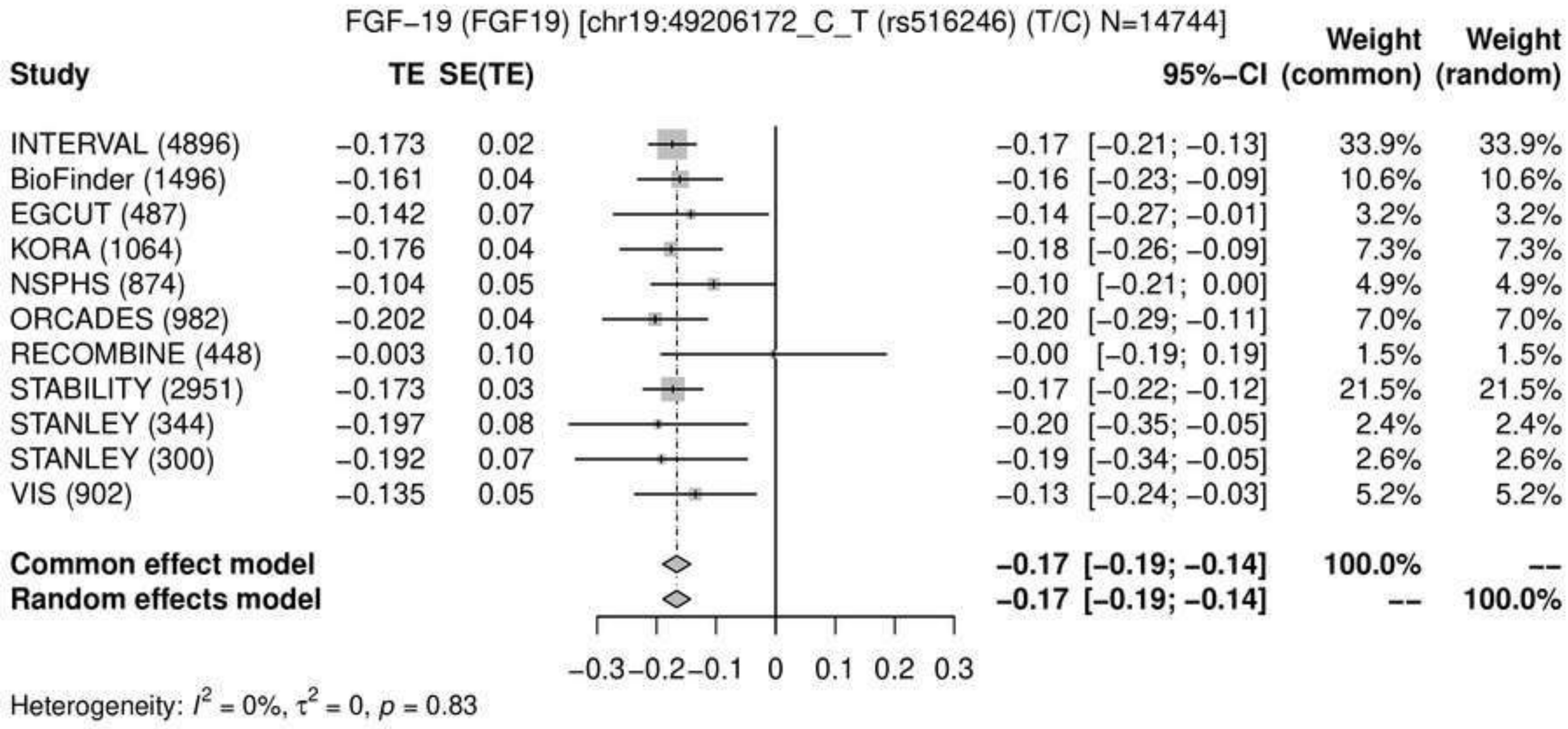
FGF−19 (FGF19) [chr19:49206172_C_T (rs516246) (T/C) N=14744]
Study
TE SE(TE)
95%−CI
Weight (common)
Weight (random)
INTERVAL (4896) −0.173 0.02 −0.17 [−0.21; −0.13] 33.9% 33.9%
BioFinder (1496) −0.161 0.04 −0.16 [−0.23; −0.09] 10.6% 10.6%
EGCUT (487) −0.142 0.07 −0.14 [−0.27; −0.01] 3.2% 3.2%
KORA (1064) −0.176 0.04 −0.18 [−0.26; −0.09] 7.3% 7.3%
NSPHS (874) −0.104 0.05 −0.10 [−0.21; 0.00] 4.9% 4.9%
ORCADES (982) −0.202 0.04 −0.20 [−0.29; −0.11] 7.0% 7.0%
RECOMBINE (448) −0.003 0.10 −0.00 [−0.19; 0.19] 1.5% 1.5%
STABILITY (2951) −0.173 0.03 −0.17 [−0.22; −0.12] 21.5% 21.5%
STANLEY (344) −0.197 0.08 −0.20 [−0.35; −0.05] 2.4% 2.4%
STANLEY (300) −0.192 0.07 −0.19 [−0.34; −0.05] 2.6% 2.6%
VIS (902) −0.135 0.05 −0.13 [−0.24; −0.03] 5.2% 5.2%
Common effect model −0.17 [−0.19; −0.14] 100.0% --
Random effects model −0.17 [−0.19; −0.14] -- 100.0%
−0.3 −0.2 −0.1 0 0.1 0.2 0.3
Heterogeneity: $I^2 = 0\%$, $\tau^2 = 0$, $p = 0.83$

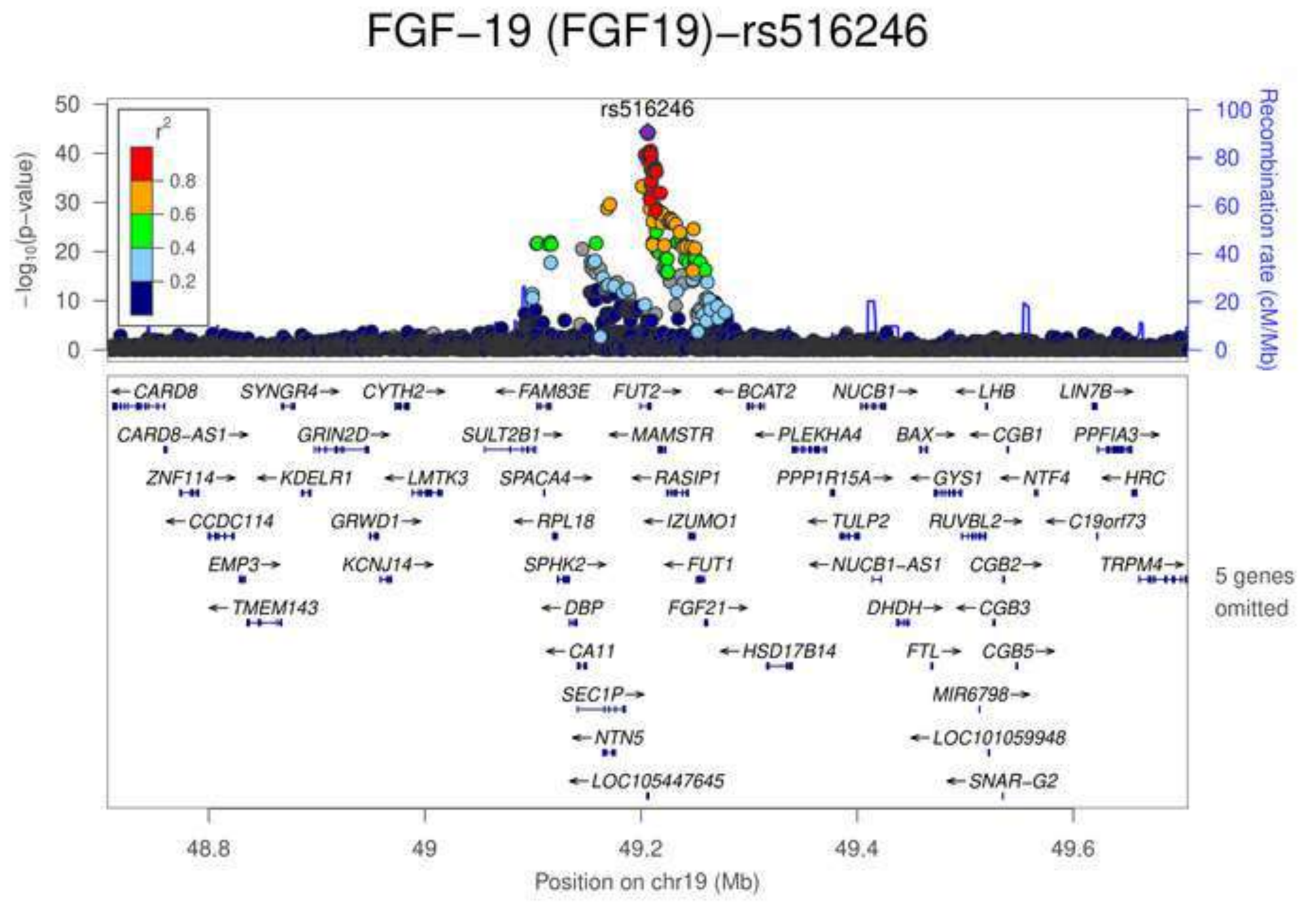
FGF−19 (FGF19)−rs516246
rs516246
$-\log_{10}(p\text{-value})$
Recombination rate (cM/Mb)
r^2
0.8
0.6
0.4
0.2
CARD8 SYNGR4 CYTH2 FAM83E FUT2 BCAT2 NUCB1 LHB LIN7B
CARD8-AS1 GRIN2D SULT2B1 MAMSTR PLEKHA4 BAX CGB1 PPFIA3
ZNF114 KDELR1 LMTK3 SPACA4 RASIP1 PPP1R15A GYS1 NTF4 HRC
CCDC114 GRWD1 RPL18 IZUMO1 TULP2 RUVBL2 C19orf73
EMP3 KCNJ14 SPHK2 FUT1 NUCB1-AS1 CGB2 TRPM4
TMEM143 DBP FGF21 DHDH CGB3
CA11 HSD17B14 FTL CGB5
SEC1P MIR6798
NTN5 LOC101059948
LOC105447645 SNAR-G2
5 genes omitted
48.8 49 49.2 49.4 49.6
Position on chr19 (Mb)

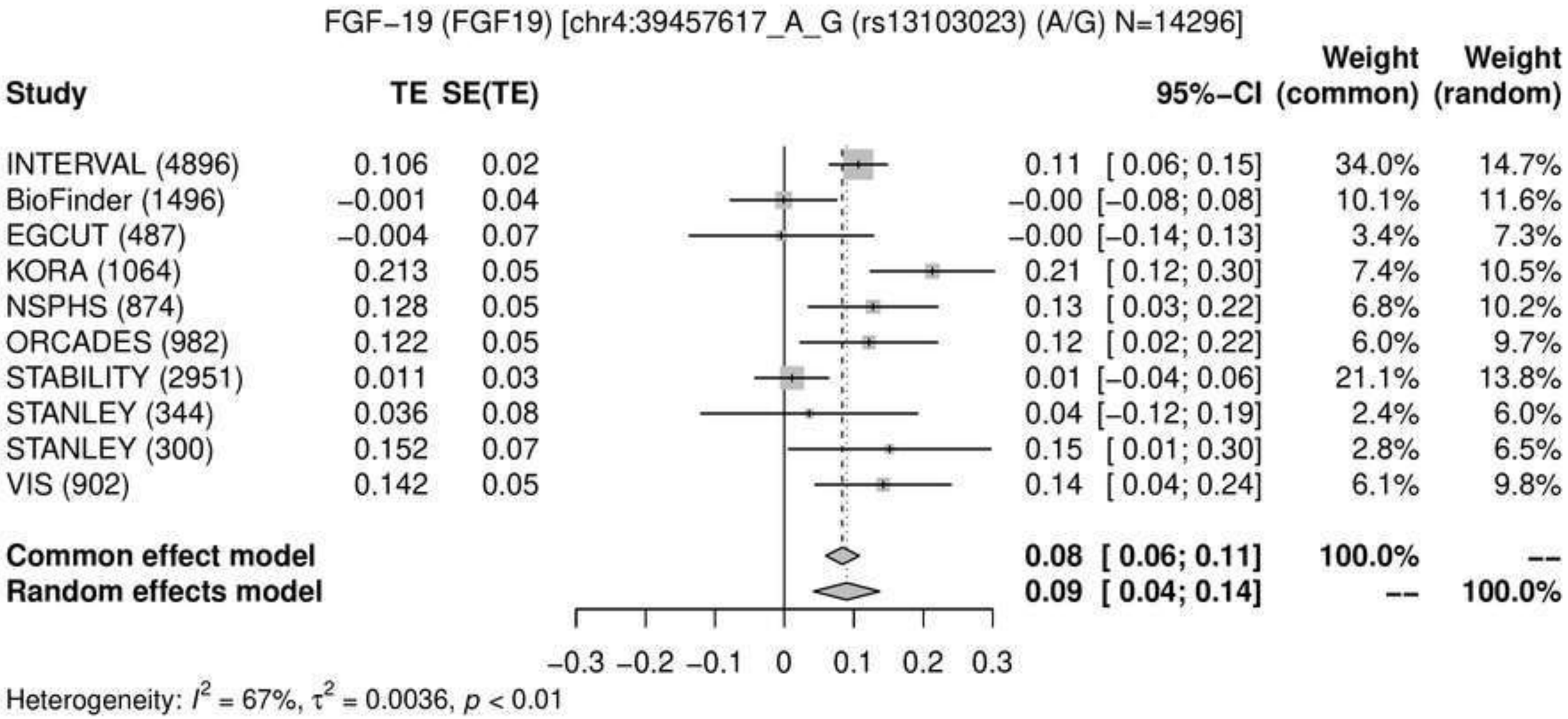
FGF−19 (FGF19) [chr4:39457617_A_G (rs13103023) (A/G) N=14296]
Study
TE SE(TE)
95%−CI
Weight (common)
Weight (random)
INTERVAL (4896) 0.106 0.02 0.11 [0.06; 0.15] 34.0% 14.7%
BioFinder (1496) −0.001 0.04 −0.00 [−0.08; 0.08] 10.1% 11.6%
EGCUT (487) −0.004 0.07 −0.00 [−0.14; 0.13] 3.4% 7.3%
KORA (1064) 0.213 0.05 0.21 [0.12; 0.30] 7.4% 10.5%
NSPHS (874) 0.128 0.05 0.13 [0.03; 0.22] 6.8% 10.2%
ORCADES (982) 0.122 0.05 0.12 [0.02; 0.22] 6.0% 9.7%
STABILITY (2951) 0.011 0.03 0.01 [−0.04; 0.06] 21.1% 13.8%
STANLEY (344) 0.036 0.08 0.04 [−0.12; 0.19] 2.4% 6.0%
STANLEY (300) 0.152 0.07 0.15 [0.01; 0.30] 2.8% 6.5%
VIS (902) 0.142 0.05 0.14 [0.04; 0.24] 6.1% 9.8%
Common effect model 0.08 [0.06; 0.11] 100.0% --
Random effects model 0.09 [0.04; 0.14] -- 100.0%
−0.3 −0.2 −0.1 0 0.1 0.2 0.3
Heterogeneity: I^2 = 67%, τ^2 = 0.0036, p < 0.01

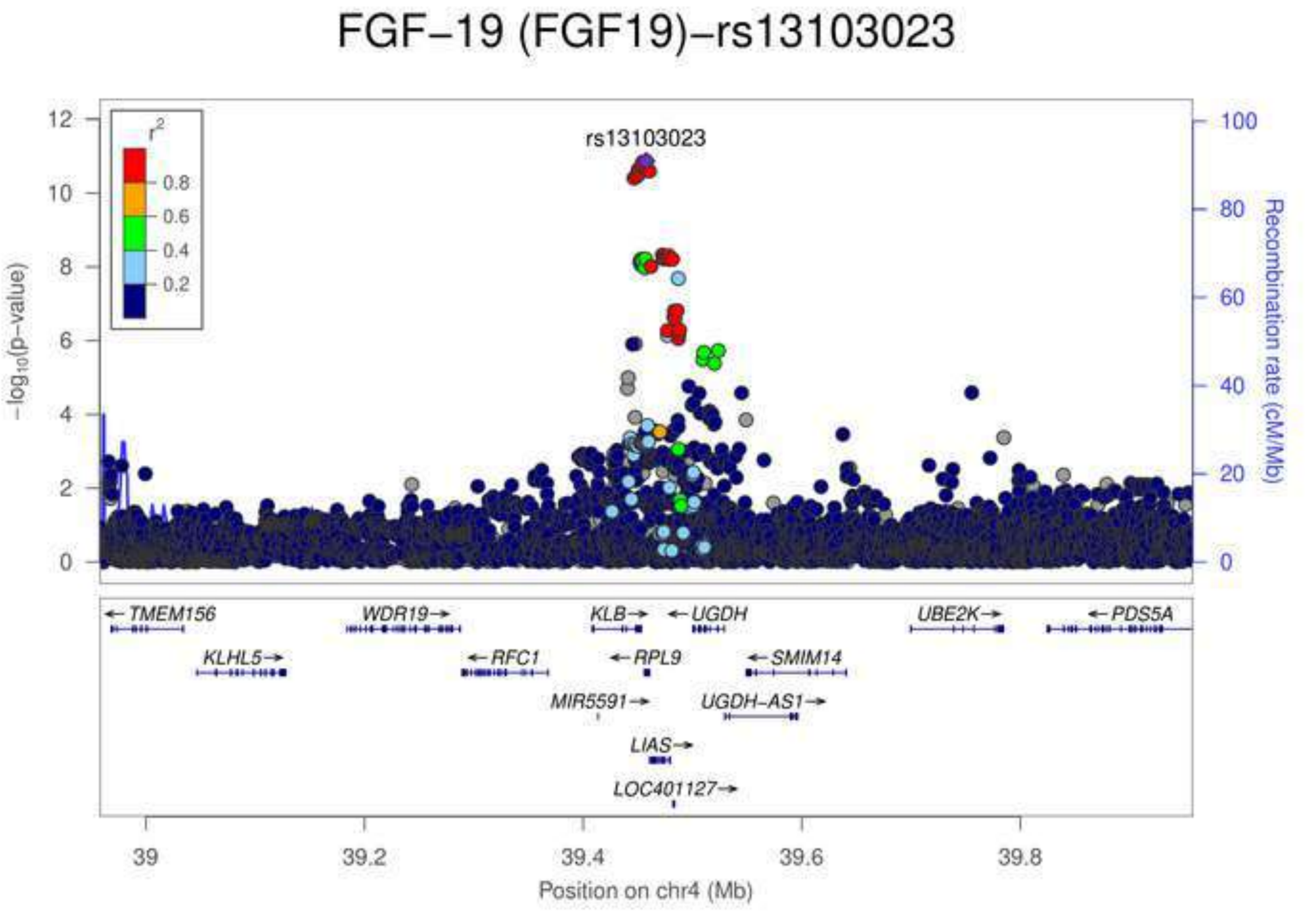
FGF−19 (FGF19)−rs13103023
rs13103023
r^2
0.8
0.6
0.4
0.2
12
10
8
6
4
2
0
$-\log_{10}$(p−value)
100
80
60
40
20
0
Recombination rate (cM/Mb)
← TMEM156
WDR19→
KLB→
← UGDH
UBE2K→
← PDS5A
KLHL5→
← RFC1
← RPL9
← SMIM14
MIR5591→
UGDH−AS1→
LIAS→
LOC401127→
39
39.2
39.4
39.6
39.8
Position on chr4 (Mb)

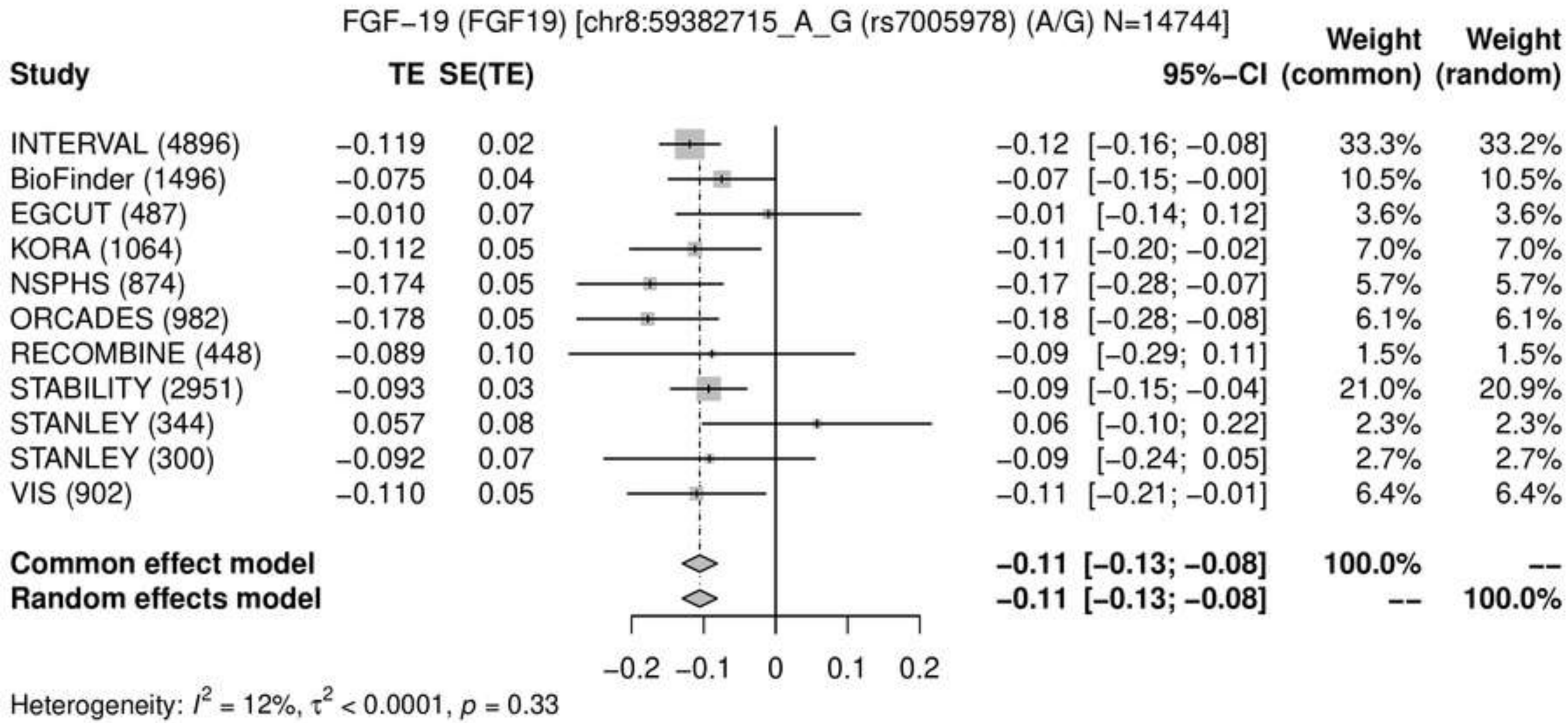
FGF−19 (FGF19) [chr8:59382715_A_G (rs7005978) (A/G) N=14744]
Study
TE
SE(TE)
95%−CI
Weight (common)
Weight (random)
INTERVAL (4896) −0.119 0.02 −0.12 [−0.16; −0.08] 33.3% 33.2%
BioFinder (1496) −0.075 0.04 −0.07 [−0.15; −0.00] 10.5% 10.5%
EGCUT (487) −0.010 0.07 −0.01 [−0.14; 0.12] 3.6% 3.6%
KORA (1064) −0.112 0.05 −0.11 [−0.20; −0.02] 7.0% 7.0%
NSPHS (874) −0.174 0.05 −0.17 [−0.28; −0.07] 5.7% 5.7%
ORCADES (982) −0.178 0.05 −0.18 [−0.28; −0.08] 6.1% 6.1%
RECOMBINE (448) −0.089 0.10 −0.09 [−0.29; 0.11] 1.5% 1.5%
STABILITY (2951) −0.093 0.03 −0.09 [−0.15; −0.04] 21.0% 20.9%
STANLEY (344) 0.057 0.08 0.06 [−0.10; 0.22] 2.3% 2.3%
STANLEY (300) −0.092 0.07 −0.09 [−0.24; 0.05] 2.7% 2.7%
VIS (902) −0.110 0.05 −0.11 [−0.21; −0.01] 6.4% 6.4%
Common effect model −0.11 [−0.13; −0.08] 100.0% --
Random effects model −0.11 [−0.13; −0.08] -- 100.0%
−0.2 −0.1 0 0.1 0.2
Heterogeneity: I^2 = 12%, τ^2 < 0.0001, p = 0.33

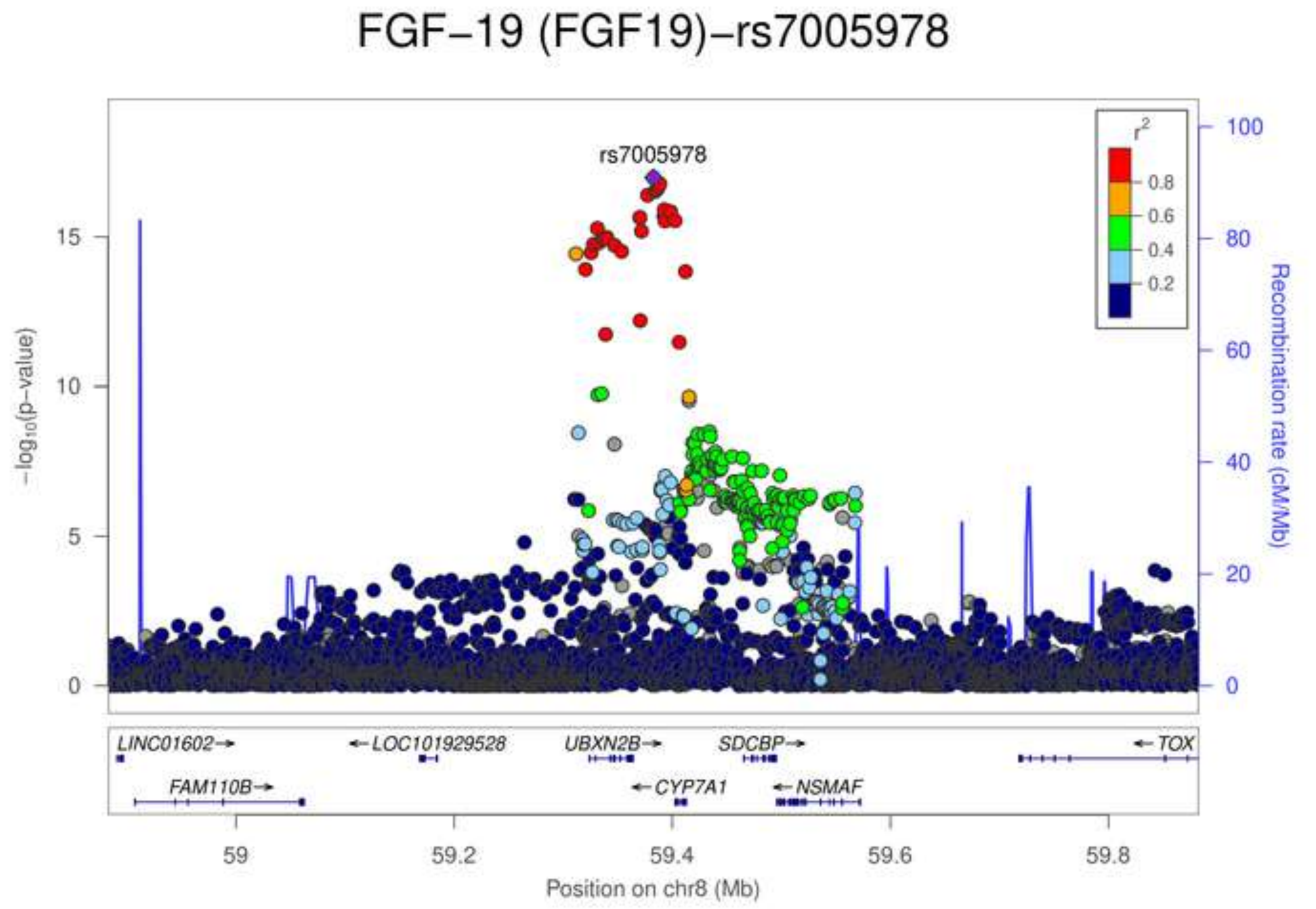
FGF−19 (FGF19)−rs7005978
rs7005978
r^2
0.8
0.6
0.4
0.2
$-\log_{10}$(p−value)
Recombination rate (cM/Mb)
LINC01602
LOC101929528
UBXN2B
SDCBP
TOX
FAM110B
CYP7A1
NSMAF
59
59.2
59.4
59.6
59.8
Position on chr8 (Mb)

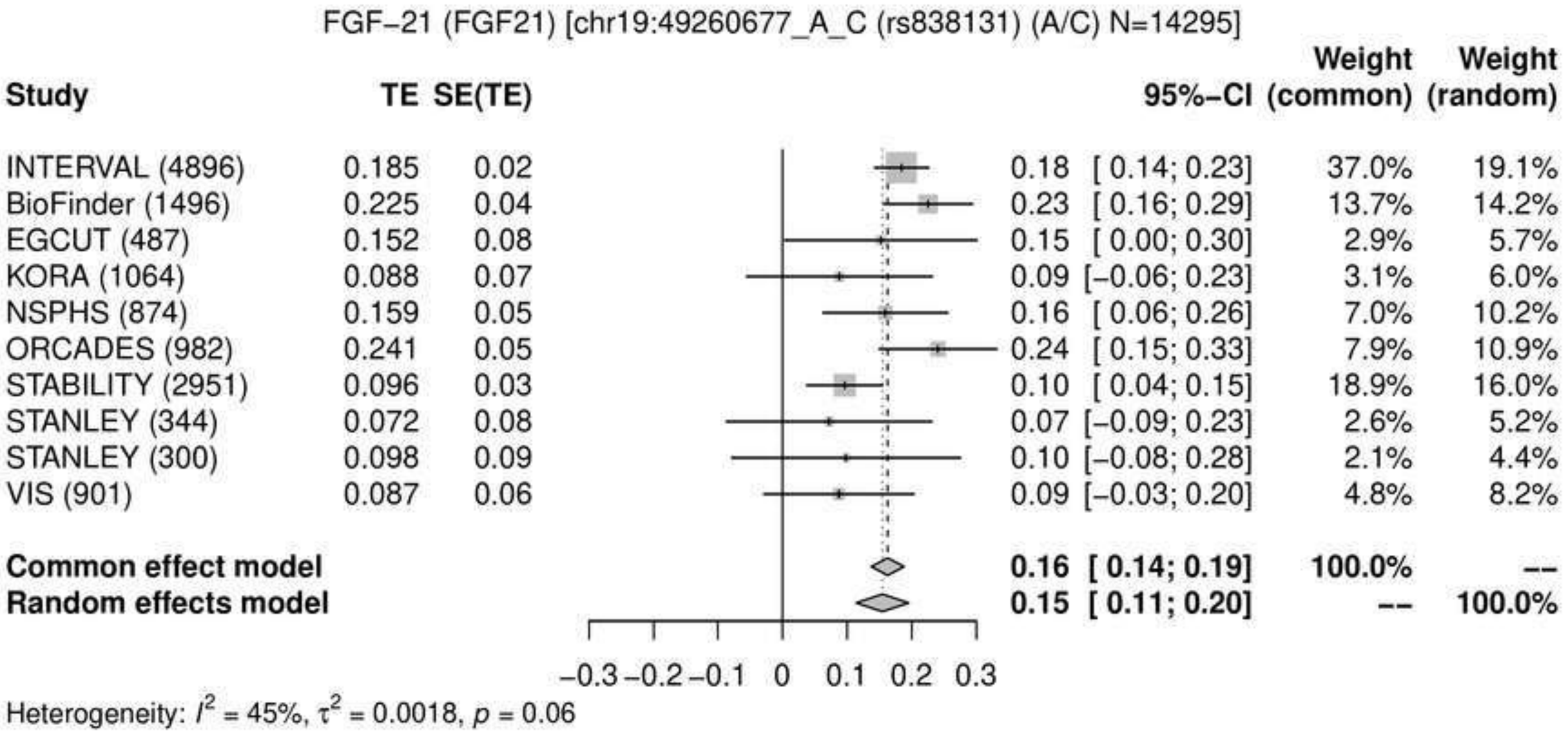
FGF−21 (FGF21) [chr19:49260677_A_C (rs838131) (A/C) N=14295]
Study
TE
SE(TE)
95%−CI
Weight (common)
Weight (random)
INTERVAL (4896) 0.185 0.02 0.18 [0.14; 0.23] 37.0% 19.1%
BioFinder (1496) 0.225 0.04 0.23 [0.16; 0.29] 13.7% 14.2%
EGCUT (487) 0.152 0.08 0.15 [0.00; 0.30] 2.9% 5.7%
KORA (1064) 0.088 0.07 0.09 [−0.06; 0.23] 3.1% 6.0%
NSPHS (874) 0.159 0.05 0.16 [0.06; 0.26] 7.0% 10.2%
ORCADES (982) 0.241 0.05 0.24 [0.15; 0.33] 7.9% 10.9%
STABILITY (2951) 0.096 0.03 0.10 [0.04; 0.15] 18.9% 16.0%
STANLEY (344) 0.072 0.08 0.07 [−0.09; 0.23] 2.6% 5.2%
STANLEY (300) 0.098 0.09 0.10 [−0.08; 0.28] 2.1% 4.4%
VIS (901) 0.087 0.06 0.09 [−0.03; 0.20] 4.8% 8.2%
Common effect model 0.16 [0.14; 0.19] 100.0% --
Random effects model 0.15 [0.11; 0.20] -- 100.0%
−0.3 −0.2 −0.1 0 0.1 0.2 0.3
Heterogeneity: I^2 = 45%, τ^2 = 0.0018, p = 0.06

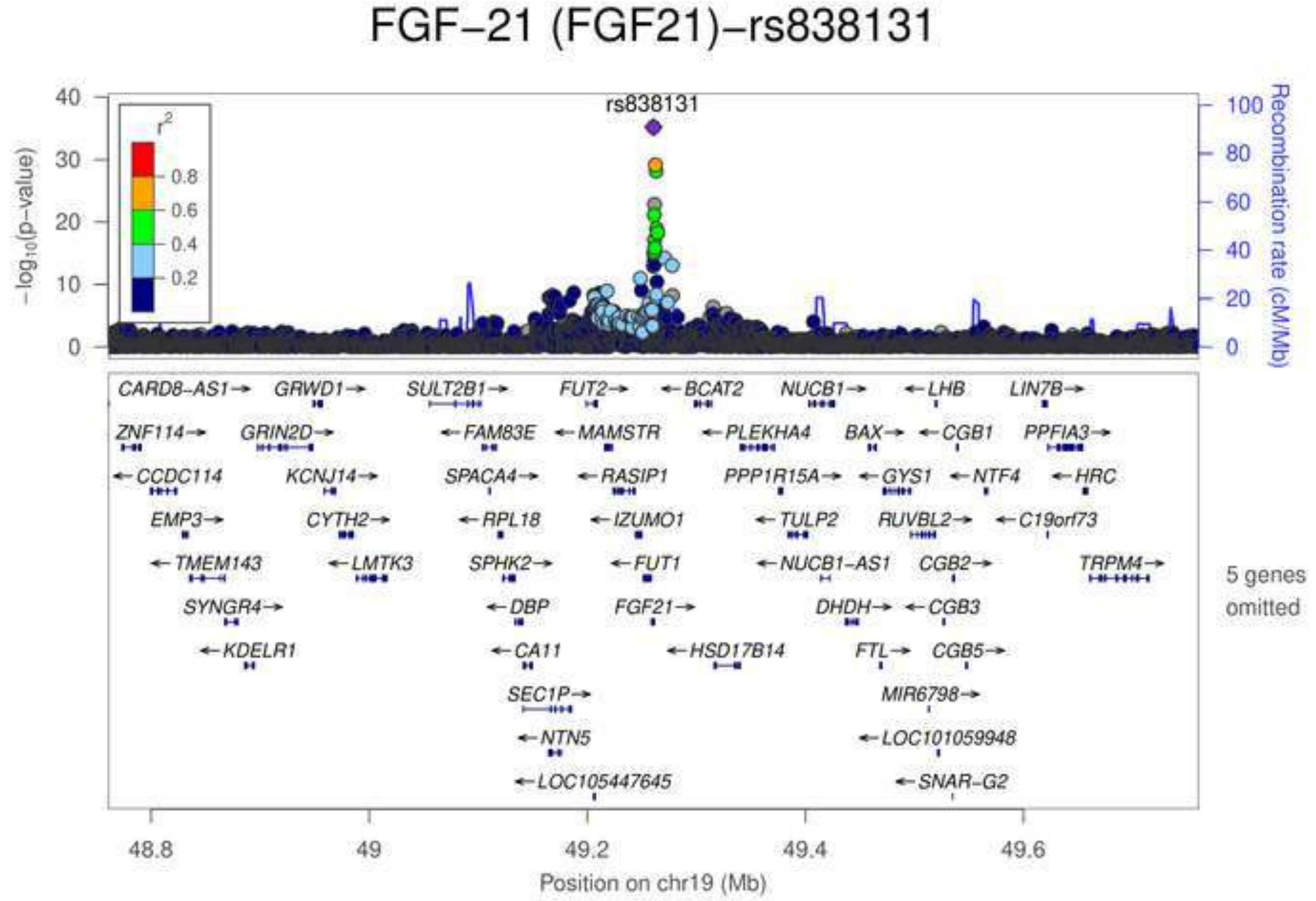
FGF−21 (FGF21)−rs838131
rs838131
$-\log_{10}$(p−value)
Recombination rate (cM/Mb)
r^2
0.8
0.6
0.4
0.2
0 10 20 30 40
0 20 40 60 80 100
CARD8−AS1→ GRWD1→ SULT2B1→ FUT2→ ←BCAT2 NUCB1→ ←LHB LIN7B→
ZNF114→ GRIN2D→ ←FAM83E ←MAMSTR ←PLEKHA4 BAX→ ←CGB1 PPFIA3→
←CCDC114 KCNJ14→ SPACA4→ ←RASIP1 PPP1R15A→ ←GYS1 ←NTF4 ←HRC
EMP3→ CYTH2→ ←RPL18 ←IZUMO1 ←TULP2 RUVBL2→ ←C19orf73
←TMEM143 ←LMTK3 SPHK2→ ←FUT1 ←NUCB1−AS1 CGB2→ TRPM4→
SYNGR4→ ←DBP FGF21→ DHDH→ ←CGB3
←KDELR1 ←CA11 ←HSD17B14 FTL→ CGB5→
SEC1P→ MIR6798→
←NTN5 ←LOC101059948
←LOC105447645 ←SNAR−G2
5 genes omitted
48.8 49 49.2 49.4 49.6
Position on chr19 (Mb)

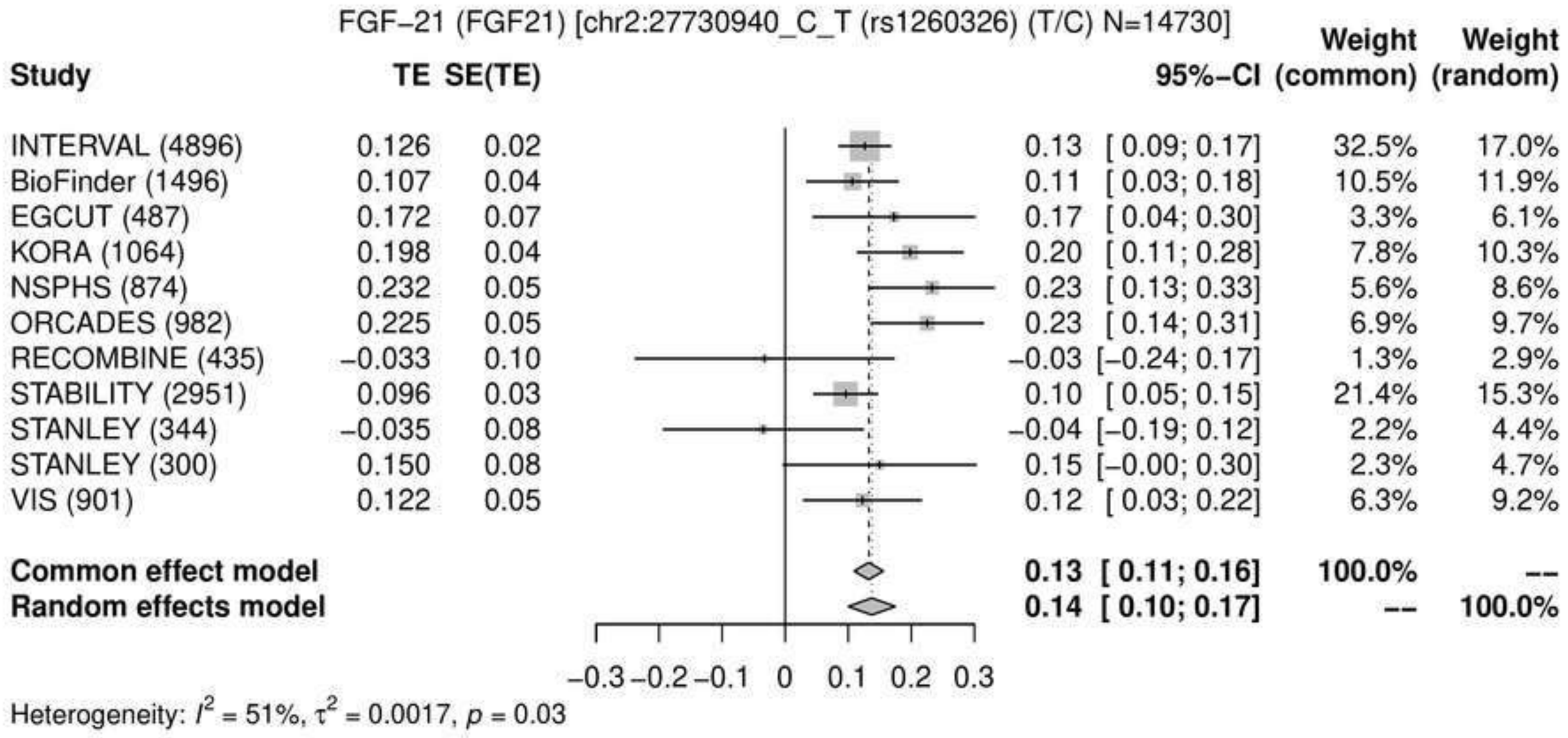
FGF−21 (FGF21) [chr2:27730940_C_T (rs1260326) (T/C) N=14730]
Study
TE
SE(TE)
95%−CI
Weight (common)
Weight (random)
INTERVAL (4896) 0.126 0.02 0.13 [0.09; 0.17] 32.5% 17.0%
BioFinder (1496) 0.107 0.04 0.11 [0.03; 0.18] 10.5% 11.9%
EGCUT (487) 0.172 0.07 0.17 [0.04; 0.30] 3.3% 6.1%
KORA (1064) 0.198 0.04 0.20 [0.11; 0.28] 7.8% 10.3%
NSPHS (874) 0.232 0.05 0.23 [0.13; 0.33] 5.6% 8.6%
ORCADES (982) 0.225 0.05 0.23 [0.14; 0.31] 6.9% 9.7%
RECOMBINE (435) −0.033 0.10 −0.03 [−0.24; 0.17] 1.3% 2.9%
STABILITY (2951) 0.096 0.03 0.10 [0.05; 0.15] 21.4% 15.3%
STANLEY (344) −0.035 0.08 −0.04 [−0.19; 0.12] 2.2% 4.4%
STANLEY (300) 0.150 0.08 0.15 [−0.00; 0.30] 2.3% 4.7%
VIS (901) 0.122 0.05 0.12 [0.03; 0.22] 6.3% 9.2%
Common effect model 0.13 [0.11; 0.16] 100.0% --
Random effects model 0.14 [0.10; 0.17] -- 100.0%
−0.3 −0.2 −0.1 0 0.1 0.2 0.3
Heterogeneity: I^2 = 51%, τ^2 = 0.0017, p = 0.03

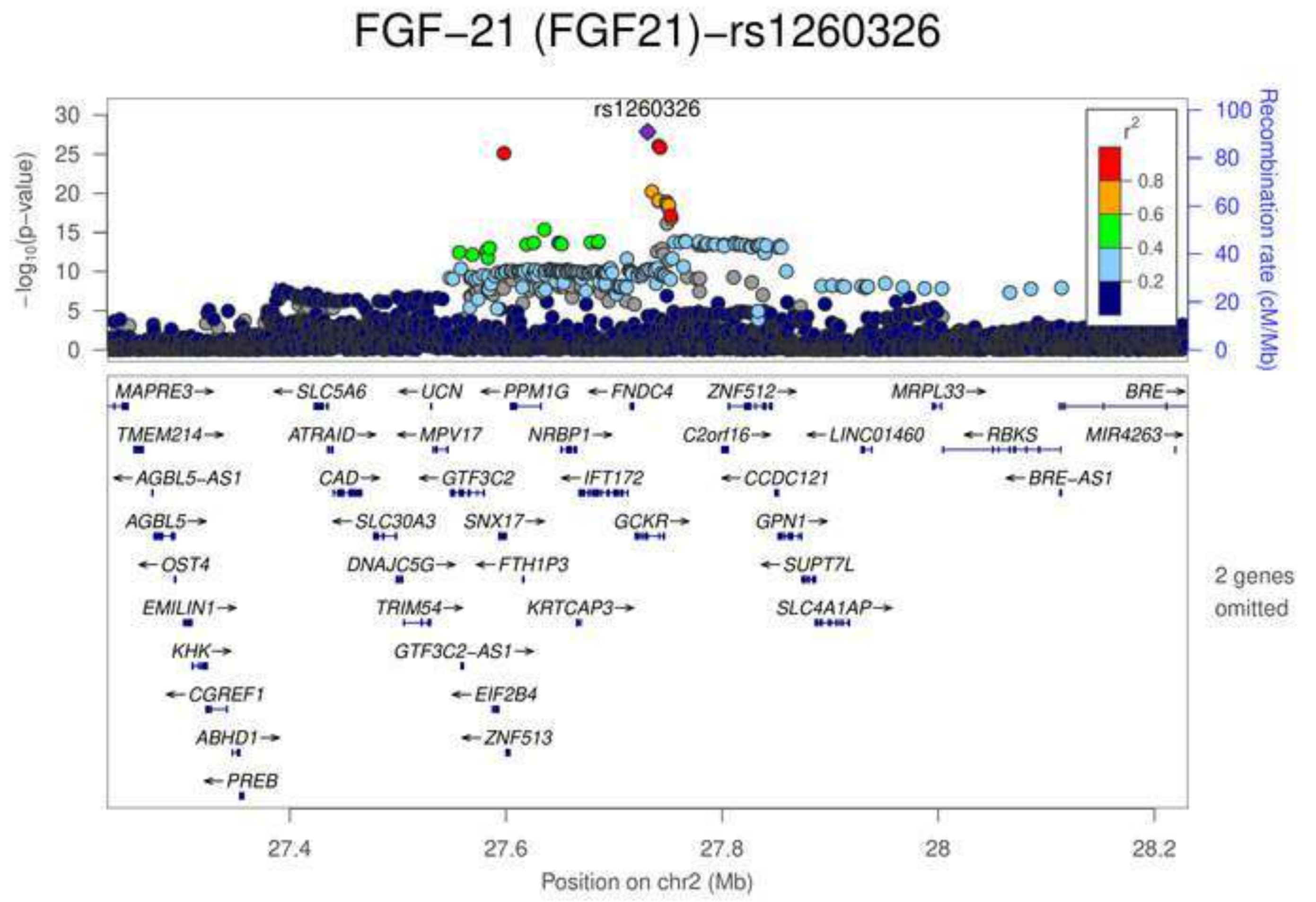
FGF−21 (FGF21)−rs1260326
rs1260326
$-\log_{10}(p\text{-value})$
Recombination rate (cM/Mb)
r^2
0.8
0.6
0.4
0.2
MAPRE3 SLC5A6 UCN PPM1G FNDC4 ZNF512 MRPL33 BRE
TMEM214 ATRAID MPV17 NRBP1 C2orf16 LINC01460 RBKS MIR4263
AGBL5−AS1 CAD GTF3C2 IFT172 CCDC121 BRE−AS1
AGBL5 SLC30A3 SNX17 GCKR GPN1
OST4 DNAJC5G FTH1P3 SUPT7L
EMILIN1 TRIM54 KRTCAP3 SLC4A1AP
KHK GTF3C2−AS1
CGREF1 EIF2B4
ABHD1 ZNF513
PREB
2 genes omitted
27.4 27.6 27.8 28 28.2
Position on chr2 (Mb)

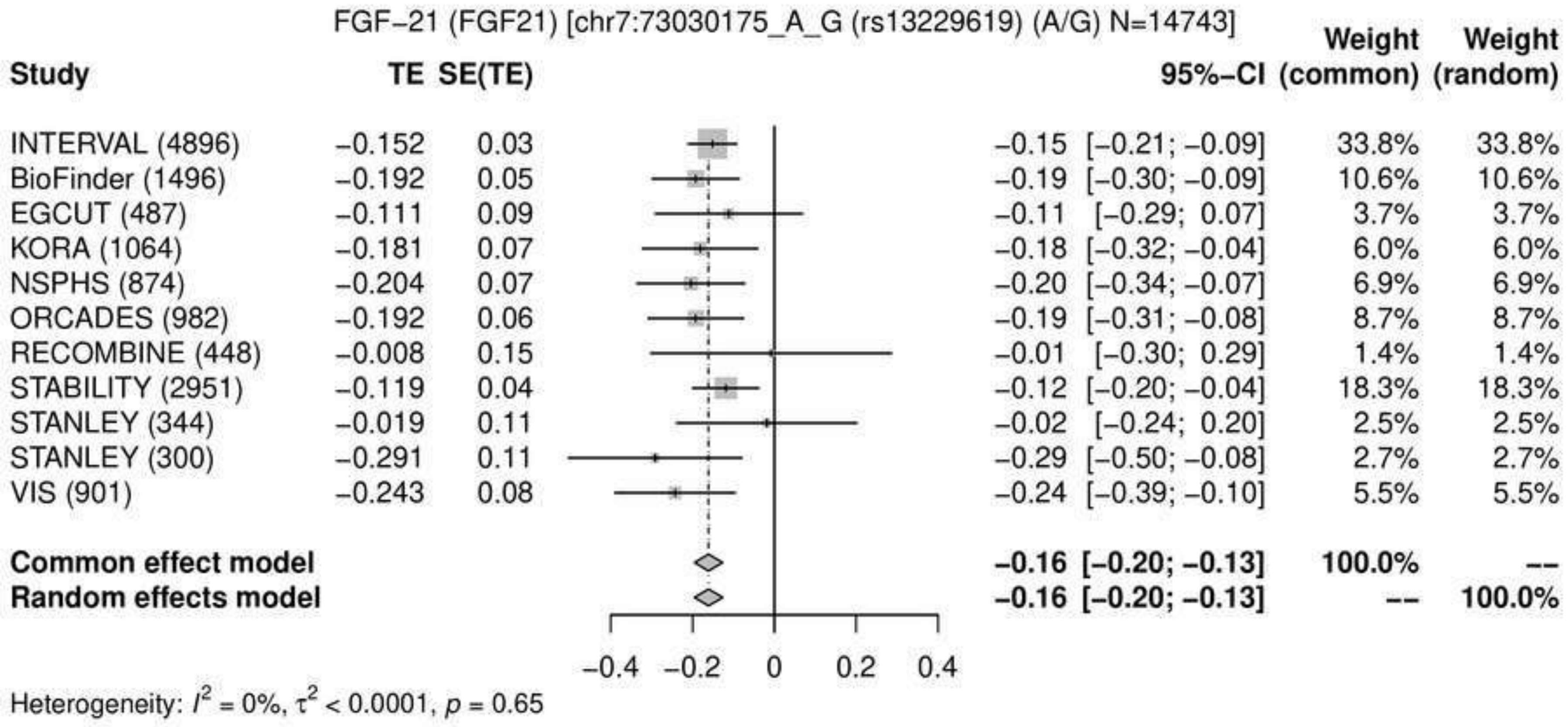
FGF−21 (FGF21) [chr7:73030175_A_G (rs13229619) (A/G) N=14743]
Study
TE
SE(TE)
95%−CI
Weight (common)
Weight (random)
INTERVAL (4896) −0.152 0.03 −0.15 [−0.21; −0.09] 33.8% 33.8%
BioFinder (1496) −0.192 0.05 −0.19 [−0.30; −0.09] 10.6% 10.6%
EGCUT (487) −0.111 0.09 −0.11 [−0.29; 0.07] 3.7% 3.7%
KORA (1064) −0.181 0.07 −0.18 [−0.32; −0.04] 6.0% 6.0%
NSPHS (874) −0.204 0.07 −0.20 [−0.34; −0.07] 6.9% 6.9%
ORCADES (982) −0.192 0.06 −0.19 [−0.31; −0.08] 8.7% 8.7%
RECOMBINE (448) −0.008 0.15 −0.01 [−0.30; 0.29] 1.4% 1.4%
STABILITY (2951) −0.119 0.04 −0.12 [−0.20; −0.04] 18.3% 18.3%
STANLEY (344) −0.019 0.11 −0.02 [−0.24; 0.20] 2.5% 2.5%
STANLEY (300) −0.291 0.11 −0.29 [−0.50; −0.08] 2.7% 2.7%
VIS (901) −0.243 0.08 −0.24 [−0.39; −0.10] 5.5% 5.5%
Common effect model −0.16 [−0.20; −0.13] 100.0% --
Random effects model −0.16 [−0.20; −0.13] -- 100.0%
−0.4 −0.2 0 0.2 0.4
Heterogeneity: I² = 0%, τ² < 0.0001, p = 0.65

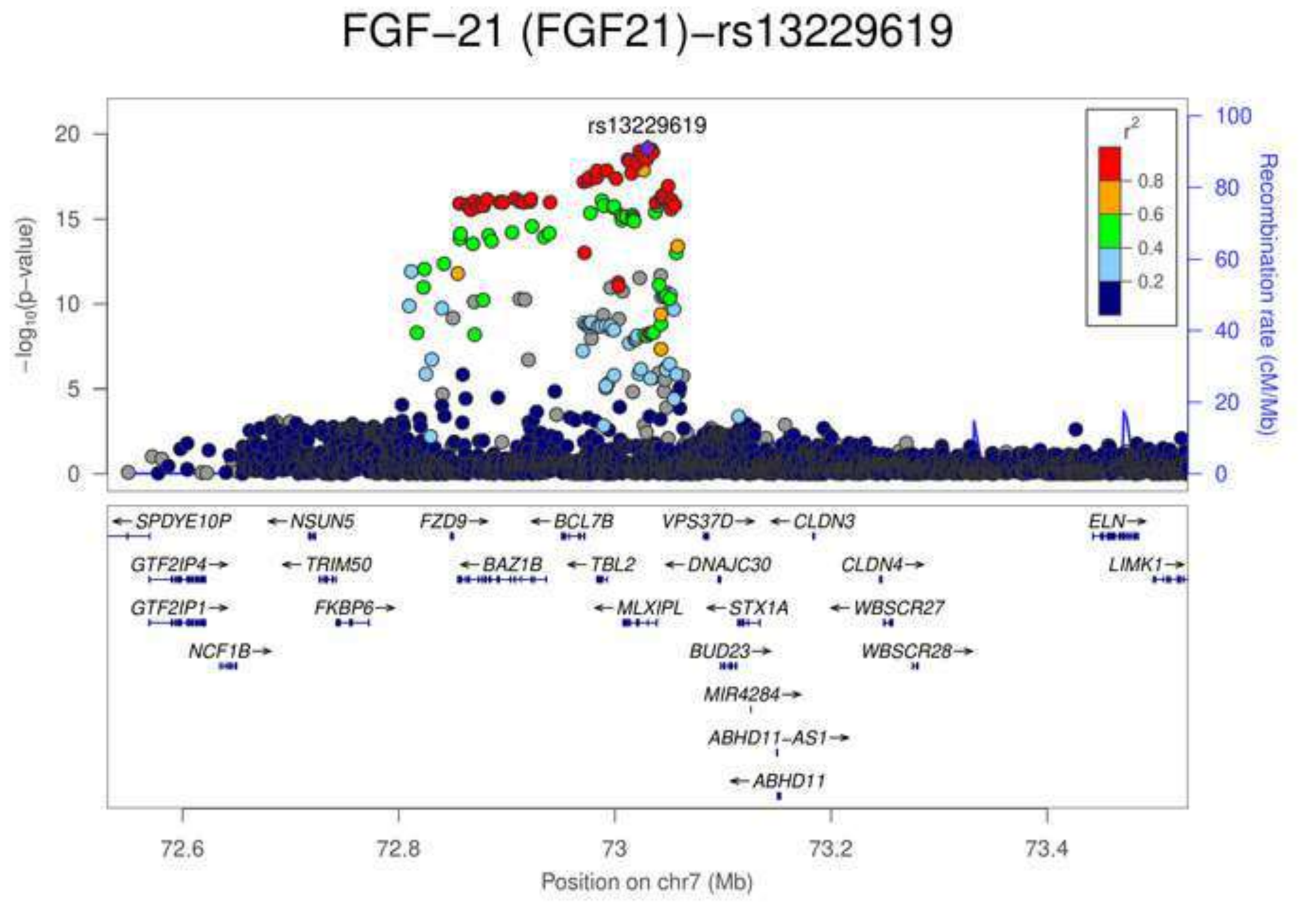
FGF−21 (FGF21)−rs13229619
rs13229619
−log10(p−value)
Recombination rate (cM/Mb)
r²
0.8
0.6
0.4
0.2
0
5
10
15
20
40
60
80
100
SPDYE10P
NSUN5
FZD9
BCL7B
VPS37D
CLDN3
ELN
GTF2IP4
TRIM50
BAZ1B
TBL2
DNAJC30
CLDN4
LIMK1
GTF2IP1
FKBP6
MLXIPL
STX1A
WBSCR27
NCF1B
BUD23
WBSCR28
MIR4284
ABHD11−AS1
ABHD11
72.6
72.8
73
73.2
73.4
Position on chr7 (Mb)

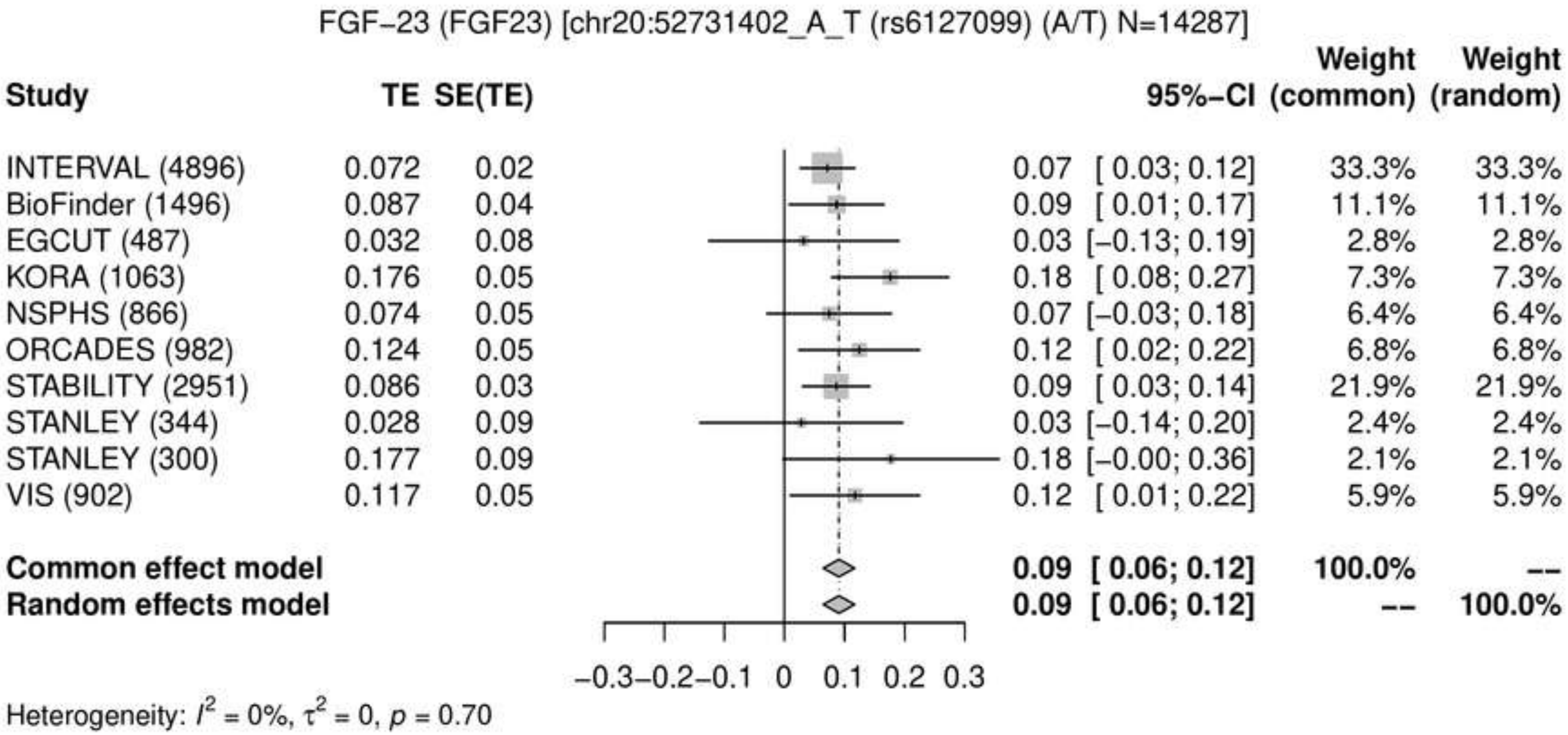
FGF−23 (FGF23) [chr20:52731402_A_T (rs6127099) (A/T) N=14287]
Study | TE | SE(TE) | 95%−CI | Weight (common) | Weight (random)
INTERVAL (4896) 0.072 0.02 0.07 [0.03; 0.12] 33.3% 33.3%
BioFinder (1496) 0.087 0.04 0.09 [0.01; 0.17] 11.1% 11.1%
EGCUT (487) 0.032 0.08 0.03 [−0.13; 0.19] 2.8% 2.8%
KORA (1063) 0.176 0.05 0.18 [0.08; 0.27] 7.3% 7.3%
NSPHS (866) 0.074 0.05 0.07 [−0.03; 0.18] 6.4% 6.4%
ORCADES (982) 0.124 0.05 0.12 [0.02; 0.22] 6.8% 6.8%
STABILITY (2951) 0.086 0.03 0.09 [0.03; 0.14] 21.9% 21.9%
STANLEY (344) 0.028 0.09 0.03 [−0.14; 0.20] 2.4% 2.4%
STANLEY (300) 0.177 0.09 0.18 [−0.00; 0.36] 2.1% 2.1%
VIS (902) 0.117 0.05 0.12 [0.01; 0.22] 5.9% 5.9%
Common effect model 0.09 [0.06; 0.12] 100.0% --
Random effects model 0.09 [0.06; 0.12] -- 100.0%
−0.3 −0.2 −0.1 0 0.1 0.2 0.3
Heterogeneity: I^2 = 0%, τ^2 = 0, p = 0.70

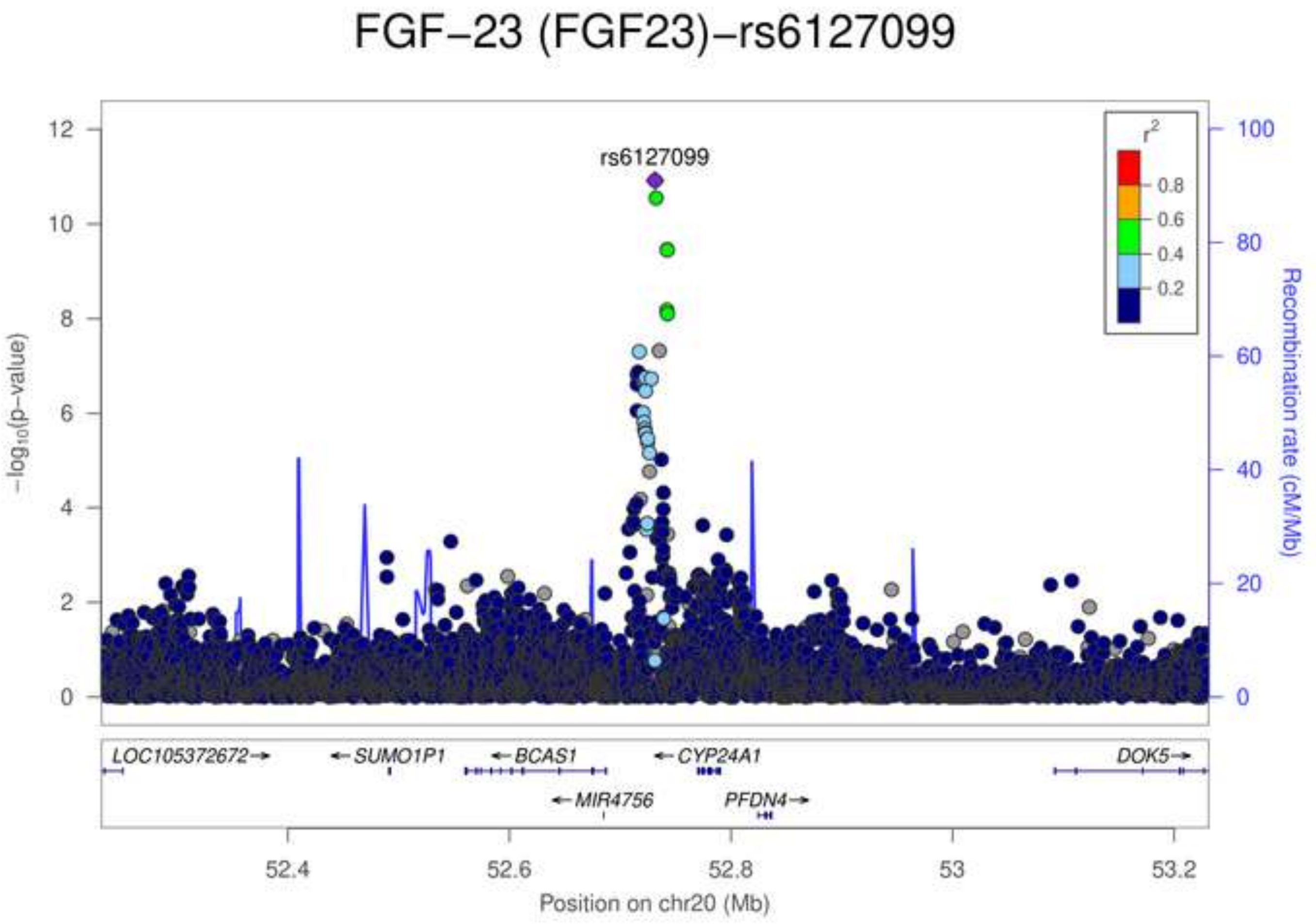
FGF−23 (FGF23)−rs6127099
rs6127099
$-\log_{10}$(p−value)
Recombination rate (cM/Mb)
r^2
0.8
0.6
0.4
0.2
LOC105372672→
←SUMO1P1
←BCAS1
←CYP24A1
DOK5→
←MIR4756
PFDN4→
52.4
52.6
52.8
53
53.2
Position on chr20 (Mb)

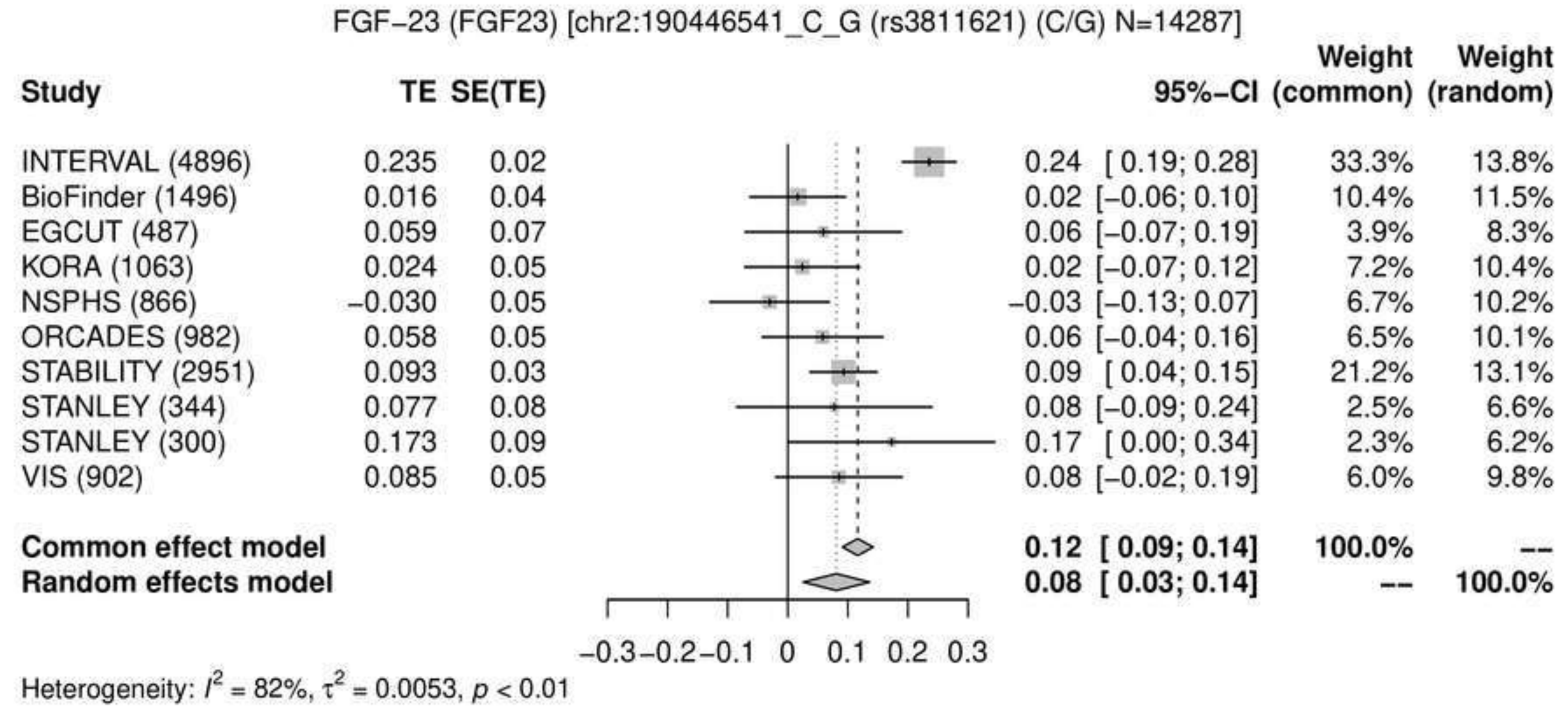
FGF−23 (FGF23) [chr2:190446541_C_G (rs3811621) (C/G) N=14287]
Study
TE
SE(TE)
95%−CI
Weight (common)
Weight (random)
INTERVAL (4896) 0.235 0.02 0.24 [0.19; 0.28] 33.3% 13.8%
BioFinder (1496) 0.016 0.04 0.02 [−0.06; 0.10] 10.4% 11.5%
EGCUT (487) 0.059 0.07 0.06 [−0.07; 0.19] 3.9% 8.3%
KORA (1063) 0.024 0.05 0.02 [−0.07; 0.12] 7.2% 10.4%
NSPHS (866) −0.030 0.05 −0.03 [−0.13; 0.07] 6.7% 10.2%
ORCADES (982) 0.058 0.05 0.06 [−0.04; 0.16] 6.5% 10.1%
STABILITY (2951) 0.093 0.03 0.09 [0.04; 0.15] 21.2% 13.1%
STANLEY (344) 0.077 0.08 0.08 [−0.09; 0.24] 2.5% 6.6%
STANLEY (300) 0.173 0.09 0.17 [0.00; 0.34] 2.3% 6.2%
VIS (902) 0.085 0.05 0.08 [−0.02; 0.19] 6.0% 9.8%
Common effect model 0.12 [0.09; 0.14] 100.0% --
Random effects model 0.08 [0.03; 0.14] -- 100.0%
−0.3 −0.2 −0.1 0 0.1 0.2 0.3
Heterogeneity: I^2 = 82%, τ^2 = 0.0053, p < 0.01

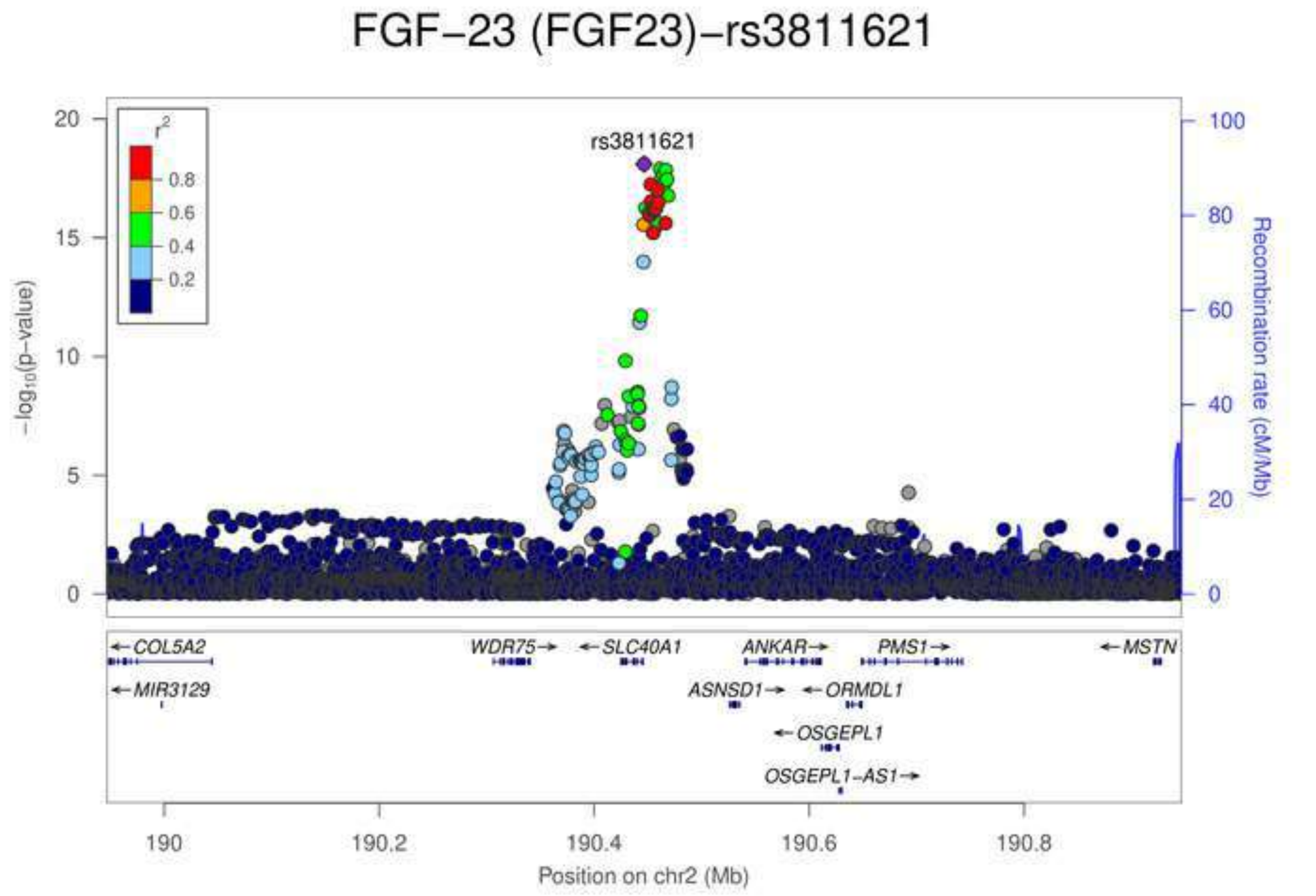
FGF−23 (FGF23)−rs3811621
rs3811621
r^2
0.8
0.6
0.4
0.2
$-\log_{10}$(p−value)
Recombination rate (cM/Mb)
COL5A2
MIR3129
WDR75
SLC40A1
ANKAR
PMS1
MSTN
ASNSD1
ORMDL1
OSGEPL1
OSGEPL1−AS1
190
190.2
190.4
190.6
190.8
Position on chr2 (Mb)

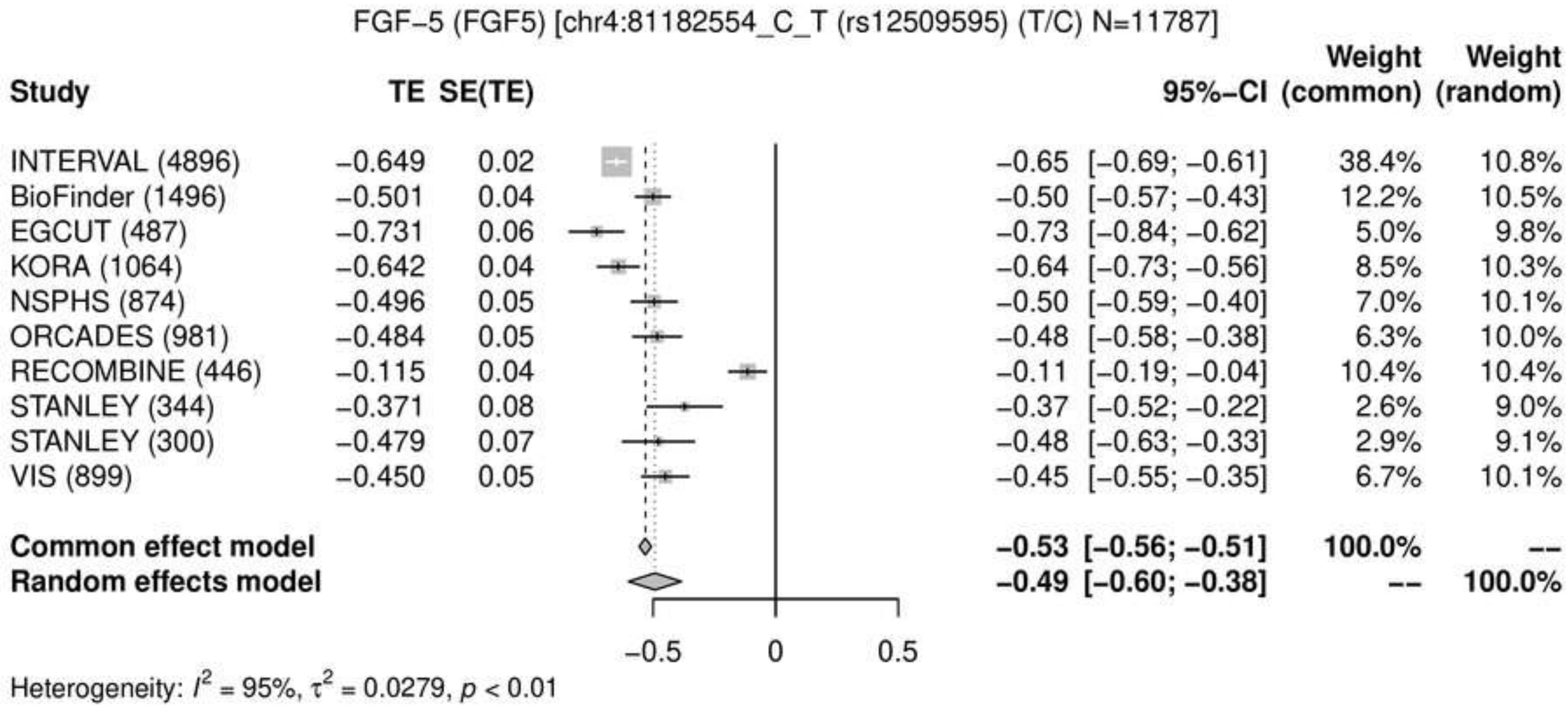
FGF−5 (FGF5) [chr4:81182554_C_T (rs12509595) (T/C) N=11787]
Study
TE
SE(TE)
95%−CI
Weight (common)
Weight (random)
INTERVAL (4896) −0.649 0.02 −0.65 [−0.69; −0.61] 38.4% 10.8%
BioFinder (1496) −0.501 0.04 −0.50 [−0.57; −0.43] 12.2% 10.5%
EGCUT (487) −0.731 0.06 −0.73 [−0.84; −0.62] 5.0% 9.8%
KORA (1064) −0.642 0.04 −0.64 [−0.73; −0.56] 8.5% 10.3%
NSPHS (874) −0.496 0.05 −0.50 [−0.59; −0.40] 7.0% 10.1%
ORCADES (981) −0.484 0.05 −0.48 [−0.58; −0.38] 6.3% 10.0%
RECOMBINE (446) −0.115 0.04 −0.11 [−0.19; −0.04] 10.4% 10.4%
STANLEY (344) −0.371 0.08 −0.37 [−0.52; −0.22] 2.6% 9.0%
STANLEY (300) −0.479 0.07 −0.48 [−0.63; −0.33] 2.9% 9.1%
VIS (899) −0.450 0.05 −0.45 [−0.55; −0.35] 6.7% 10.1%
Common effect model −0.53 [−0.56; −0.51] 100.0% --
Random effects model −0.49 [−0.60; −0.38] -- 100.0%
−0.5
0
0.5
Heterogeneity: I^2 = 95%, τ^2 = 0.0279, p < 0.01
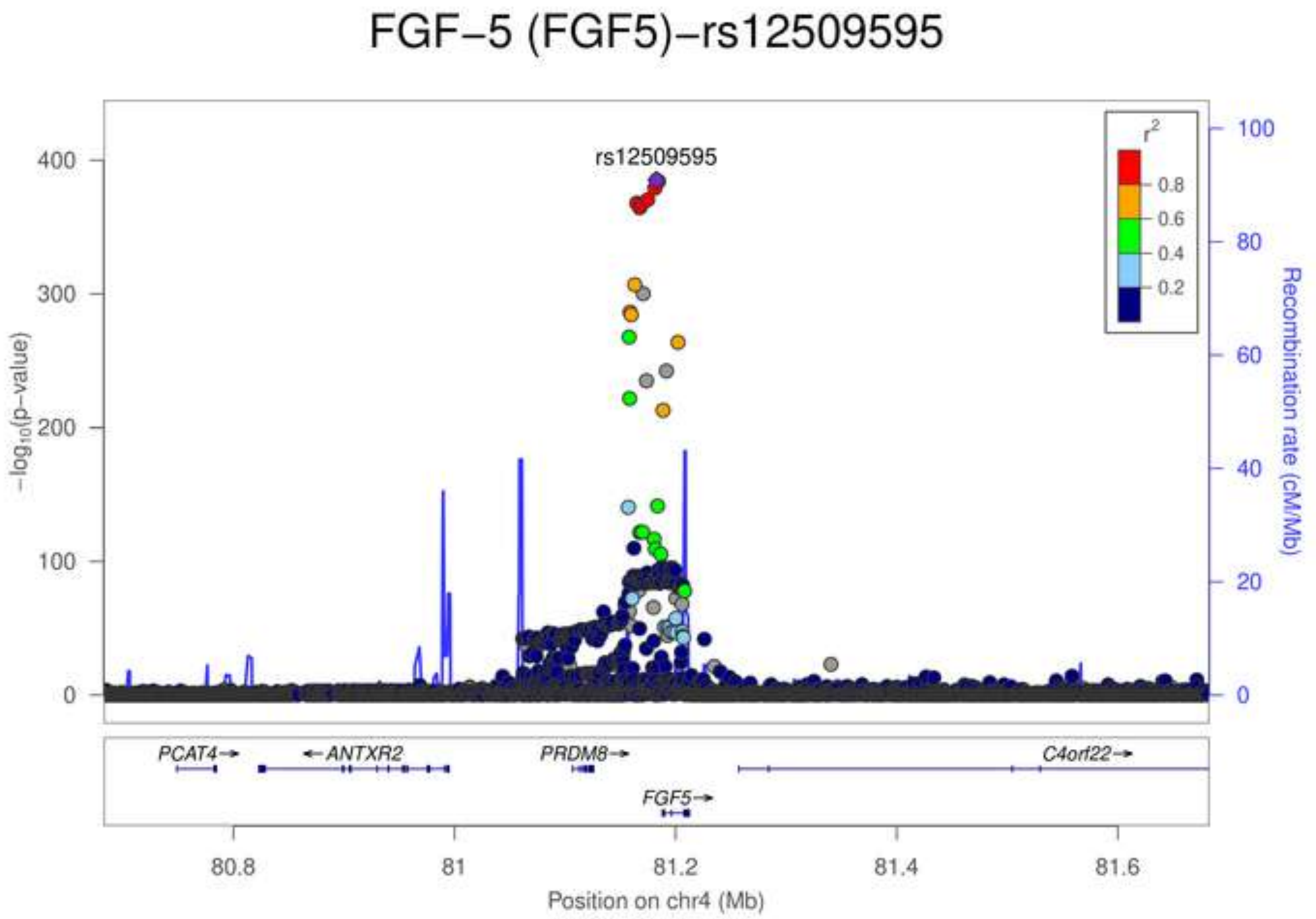
FGF−5 (FGF5)−rs12509595
rs12509595
r^2
0.8
0.6
0.4
0.2
$-\log_{10}$(p−value)
Recombination rate (cM/Mb)
400
300
200
100
0
100
80
60
40
20
0
PCAT4→
←ANTXR2
PRDM8→
C4orf22→
FGF5→
80.8
81
81.2
81.4
81.6
Position on chr4 (Mb)

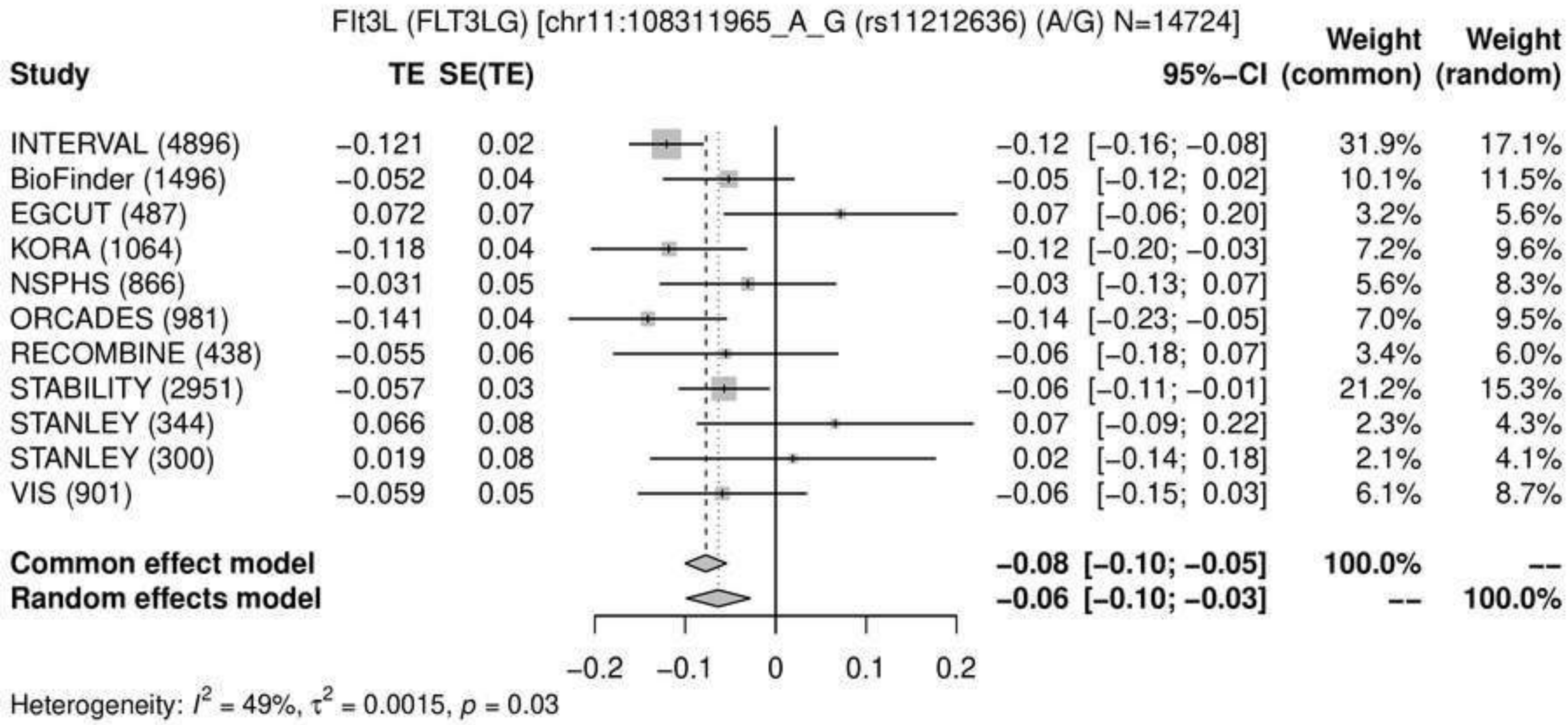

Flt3L (FLT3LG) [chr11:108311965_A_G (rs11212636) (A/G) N=14724]
Study
TE
SE(TE)
95%-CI
Weight (common)
Weight (random)
INTERVAL (4896) −0.121 0.02 −0.12 [−0.16; −0.08] 31.9% 17.1%
BioFinder (1496) −0.052 0.04 −0.05 [−0.12; 0.02] 10.1% 11.5%
EGCUT (487) 0.072 0.07 0.07 [−0.06; 0.20] 3.2% 5.6%
KORA (1064) −0.118 0.04 −0.12 [−0.20; −0.03] 7.2% 9.6%
NSPHS (866) −0.031 0.05 −0.03 [−0.13; 0.07] 5.6% 8.3%
ORCADES (981) −0.141 0.04 −0.14 [−0.23; −0.05] 7.0% 9.5%
RECOMBINE (438) −0.055 0.06 −0.06 [−0.18; 0.07] 3.4% 6.0%
STABILITY (2951) −0.057 0.03 −0.06 [−0.11; −0.01] 21.2% 15.3%
STANLEY (344) 0.066 0.08 0.07 [−0.09; 0.22] 2.3% 4.3%
STANLEY (300) 0.019 0.08 0.02 [−0.14; 0.18] 2.1% 4.1%
VIS (901) −0.059 0.05 −0.06 [−0.15; 0.03] 6.1% 8.7%
Common effect model −0.08 [−0.10; −0.05] 100.0% --
Random effects model −0.06 [−0.10; −0.03] -- 100.0%
−0.2
−0.1
0
0.1
0.2
Heterogeneity: I^2 = 49%, τ^2 = 0.0015, p = 0.03

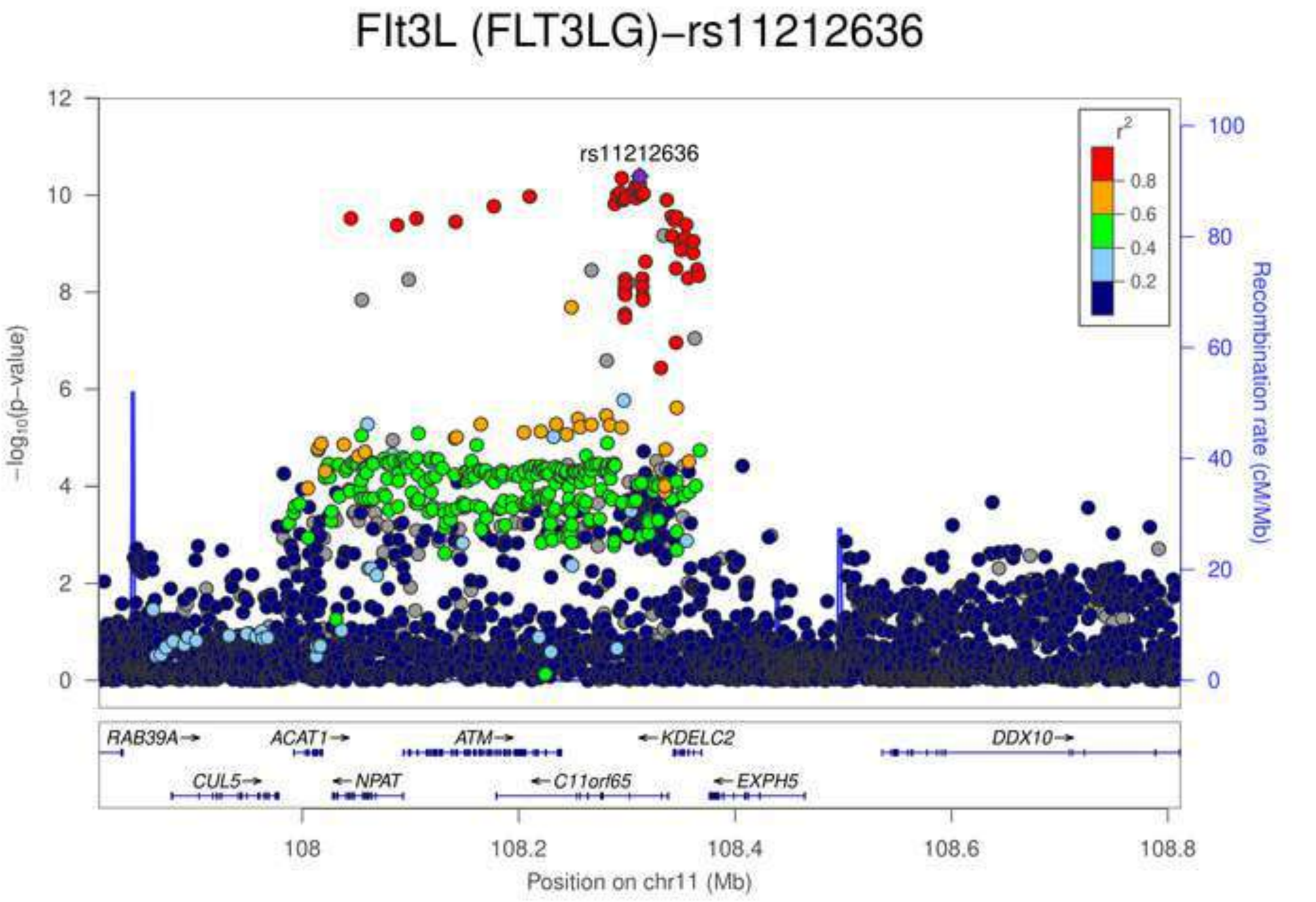

Flt3L (FLT3LG)−rs11212636
rs11212636
$-\log_{10}$(p−value)
Recombination rate (cM/Mb)
r^2
0.8
0.6
0.4
0.2
0
2
4
6
8
10
12
0
20
40
60
80
100
RAB39A→
ACAT1→
ATM→
←KDELC2
DDX10→
CUL5→
←NPAT
←C11orf65
←EXPH5
108
108.2
108.4
108.6
108.8
Position on chr11 (Mb)

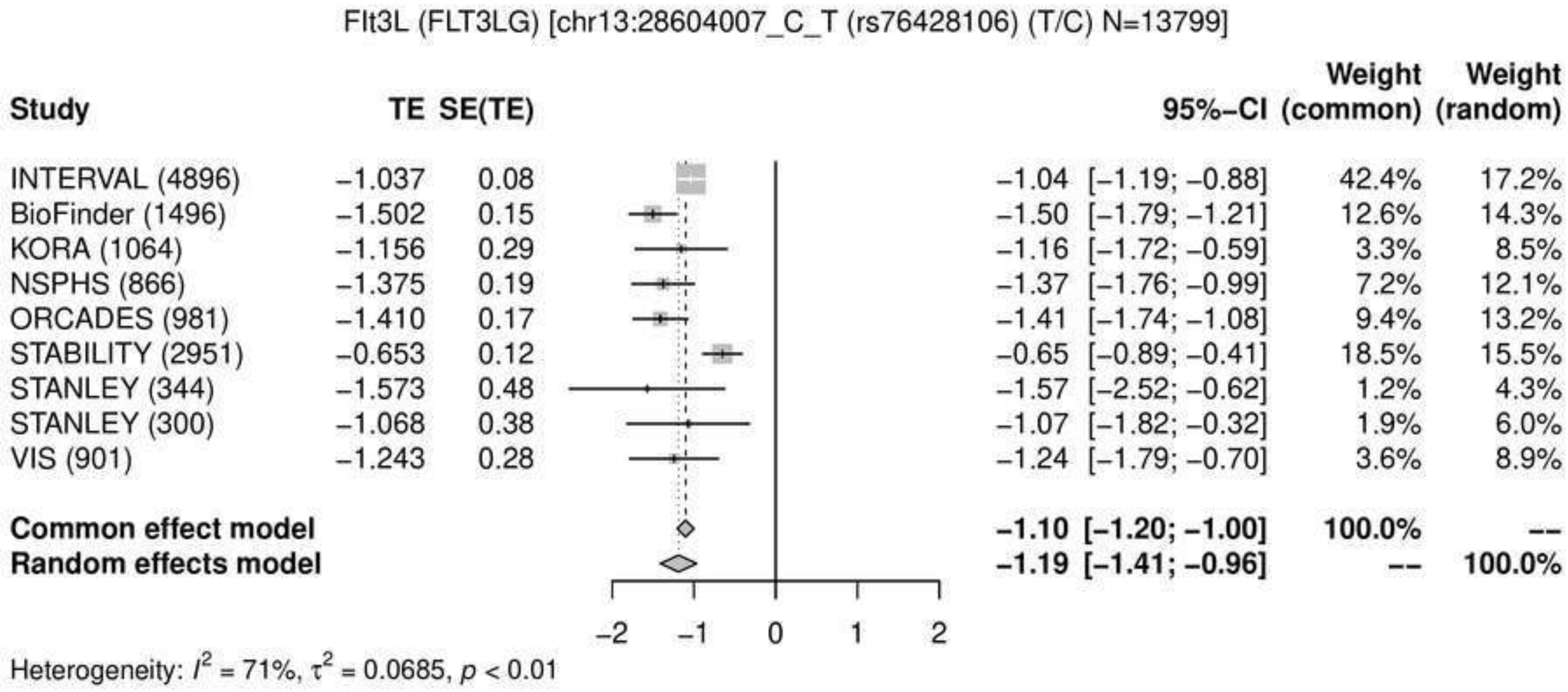
Flt3L (FLT3LG) [chr13:28604007_C_T (rs76428106) (T/C) N=13799]
Study TE SE(TE) 95%-CI Weight (common) Weight (random)
INTERVAL (4896) −1.037 0.08 −1.04 [−1.19; −0.88] 42.4% 17.2%
BioFinder (1496) −1.502 0.15 −1.50 [−1.79; −1.21] 12.6% 14.3%
KORA (1064) −1.156 0.29 −1.16 [−1.72; −0.59] 3.3% 8.5%
NSPHS (866) −1.375 0.19 −1.37 [−1.76; −0.99] 7.2% 12.1%
ORCADES (981) −1.410 0.17 −1.41 [−1.74; −1.08] 9.4% 13.2%
STABILITY (2951) −0.653 0.12 −0.65 [−0.89; −0.41] 18.5% 15.5%
STANLEY (344) −1.573 0.48 −1.57 [−2.52; −0.62] 1.2% 4.3%
STANLEY (300) −1.068 0.38 −1.07 [−1.82; −0.32] 1.9% 6.0%
VIS (901) −1.243 0.28 −1.24 [−1.79; −0.70] 3.6% 8.9%
Common effect model −1.10 [−1.20; −1.00] 100.0% --
Random effects model −1.19 [−1.41; −0.96] -- 100.0%
−2 −1 0 1 2
Heterogeneity: I^2 = 71%, τ^2 = 0.0685, p < 0.01

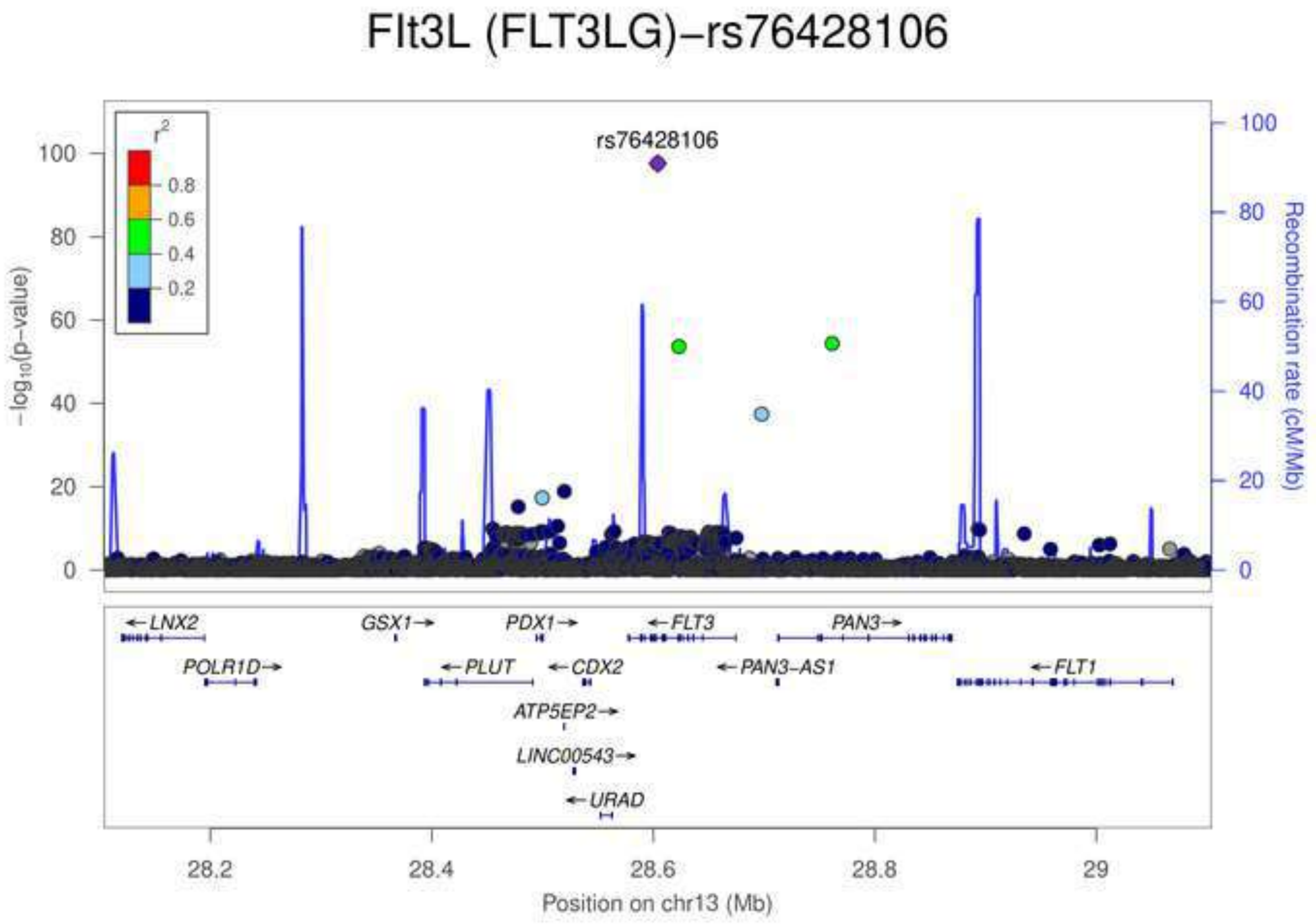
Flt3L (FLT3LG)−rs76428106
rs76428106
r^2
0.8
0.6
0.4
0.2
$-\log_{10}$(p-value)
Recombination rate (cM/Mb)
LNX2
GSX1
PDX1
FLT3
PAN3
POLR1D
PLUT
CDX2
PAN3-AS1
FLT1
ATP5EP2
LINC00543
URAD
28.2 28.4 28.6 28.8 29
Position on chr13 (Mb)

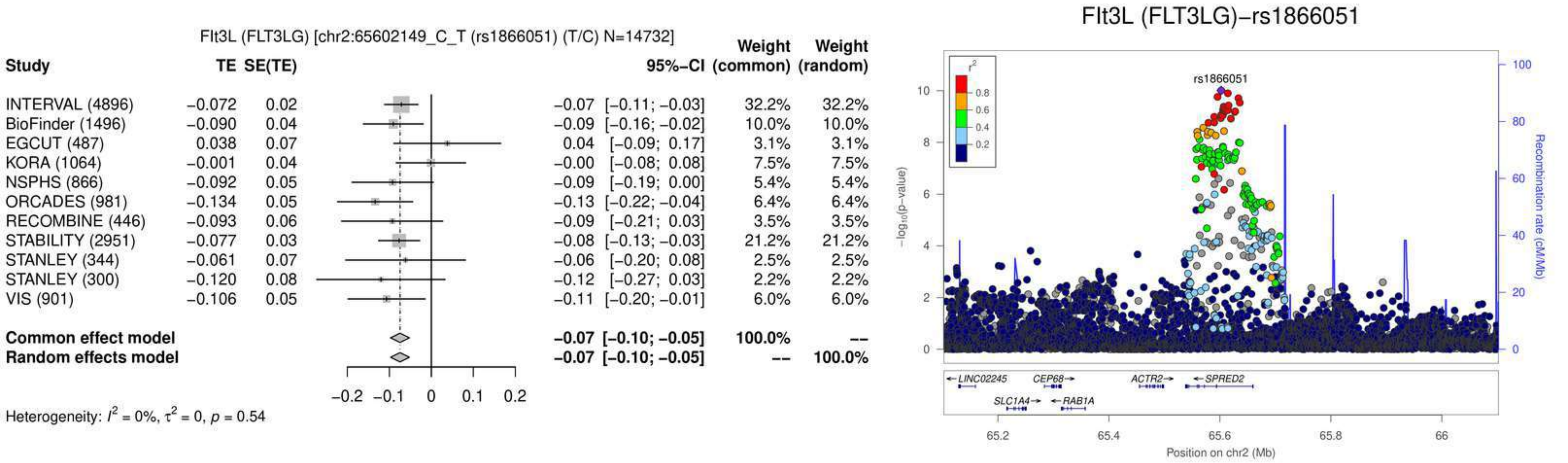

Flt3L (FLT3LG) [chr2:65602149_C_T (rs1866051) (T/C) N=14732]
Study
TE
SE(TE)
95%−CI
Weight (common)
Weight (random)
INTERVAL (4896) −0.072 0.02 −0.07 [−0.11; −0.03] 32.2% 32.2%
BioFinder (1496) −0.090 0.04 −0.09 [−0.16; −0.02] 10.0% 10.0%
EGCUT (487) 0.038 0.07 0.04 [−0.09; 0.17] 3.1% 3.1%
KORA (1064) −0.001 0.04 −0.00 [−0.08; 0.08] 7.5% 7.5%
NSPHS (866) −0.092 0.05 −0.09 [−0.19; 0.00] 5.4% 5.4%
ORCADES (981) −0.134 0.05 −0.13 [−0.22; −0.04] 6.4% 6.4%
RECOMBINE (446) −0.093 0.06 −0.09 [−0.21; 0.03] 3.5% 3.5%
STABILITY (2951) −0.077 0.03 −0.08 [−0.13; −0.03] 21.2% 21.2%
STANLEY (344) −0.061 0.07 −0.06 [−0.20; 0.08] 2.5% 2.5%
STANLEY (300) −0.120 0.08 −0.12 [−0.27; 0.03] 2.2% 2.2%
VIS (901) −0.106 0.05 −0.11 [−0.20; −0.01] 6.0% 6.0%
Common effect model −0.07 [−0.10; −0.05] 100.0% --
Random effects model −0.07 [−0.10; −0.05] -- 100.0%
−0.2 −0.1 0 0.1 0.2
Heterogeneity: $I^2 = 0\%$, $\tau^2 = 0$, $p = 0.54$
Flt3L (FLT3LG)−rs1866051
rs1866051
r^2
0.8
0.6
0.4
0.2
$-\log_{10}(p\text{-value})$
Recombination rate (cM/Mb)
LINC02245
CEP68
ACTR2
SPRED2
SLC1A4
RAB1A
65.2
65.4
65.6
65.8
66
Position on chr2 (Mb)

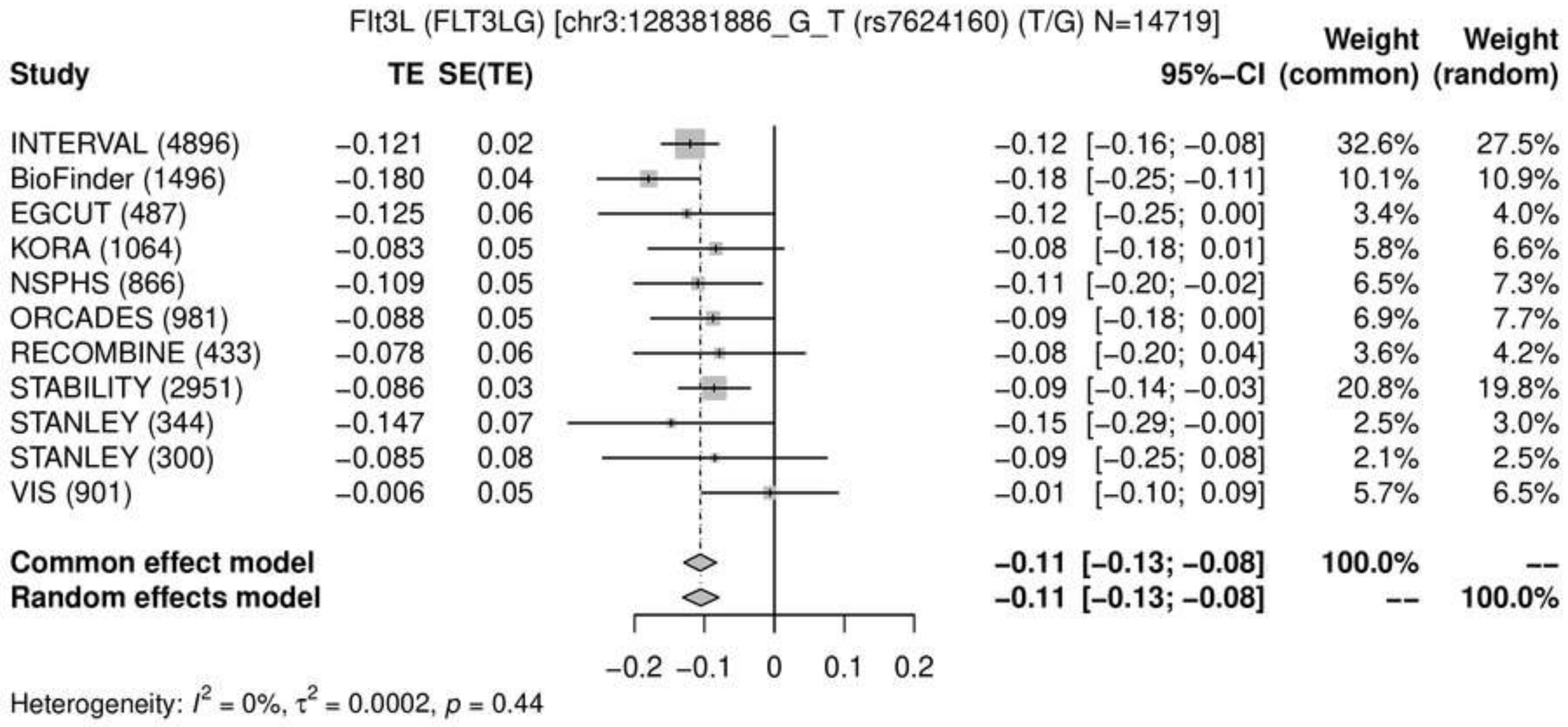
Flt3L (FLT3LG) [chr3:128381886_G_T (rs7624160) (T/G) N=14719]
Study
TE
SE(TE)
95%–CI
Weight (common)
Weight (random)
INTERVAL (4896) –0.121 0.02 –0.12 [–0.16; –0.08] 32.6% 27.5%
BioFinder (1496) –0.180 0.04 –0.18 [–0.25; –0.11] 10.1% 10.9%
EGCUT (487) –0.125 0.06 –0.12 [–0.25; 0.00] 3.4% 4.0%
KORA (1064) –0.083 0.05 –0.08 [–0.18; 0.01] 5.8% 6.6%
NSPHS (866) –0.109 0.05 –0.11 [–0.20; –0.02] 6.5% 7.3%
ORCADES (981) –0.088 0.05 –0.09 [–0.18; 0.00] 6.9% 7.7%
RECOMBINE (433) –0.078 0.06 –0.08 [–0.20; 0.04] 3.6% 4.2%
STABILITY (2951) –0.086 0.03 –0.09 [–0.14; –0.03] 20.8% 19.8%
STANLEY (344) –0.147 0.07 –0.15 [–0.29; –0.00] 2.5% 3.0%
STANLEY (300) –0.085 0.08 –0.09 [–0.25; 0.08] 2.1% 2.5%
VIS (901) –0.006 0.05 –0.01 [–0.10; 0.09] 5.7% 6.5%
Common effect model –0.11 [–0.13; –0.08] 100.0% --
Random effects model –0.11 [–0.13; –0.08] -- 100.0%
–0.2 –0.1 0 0.1 0.2
Heterogeneity: I^2 = 0%, τ^2 = 0.0002, p = 0.44

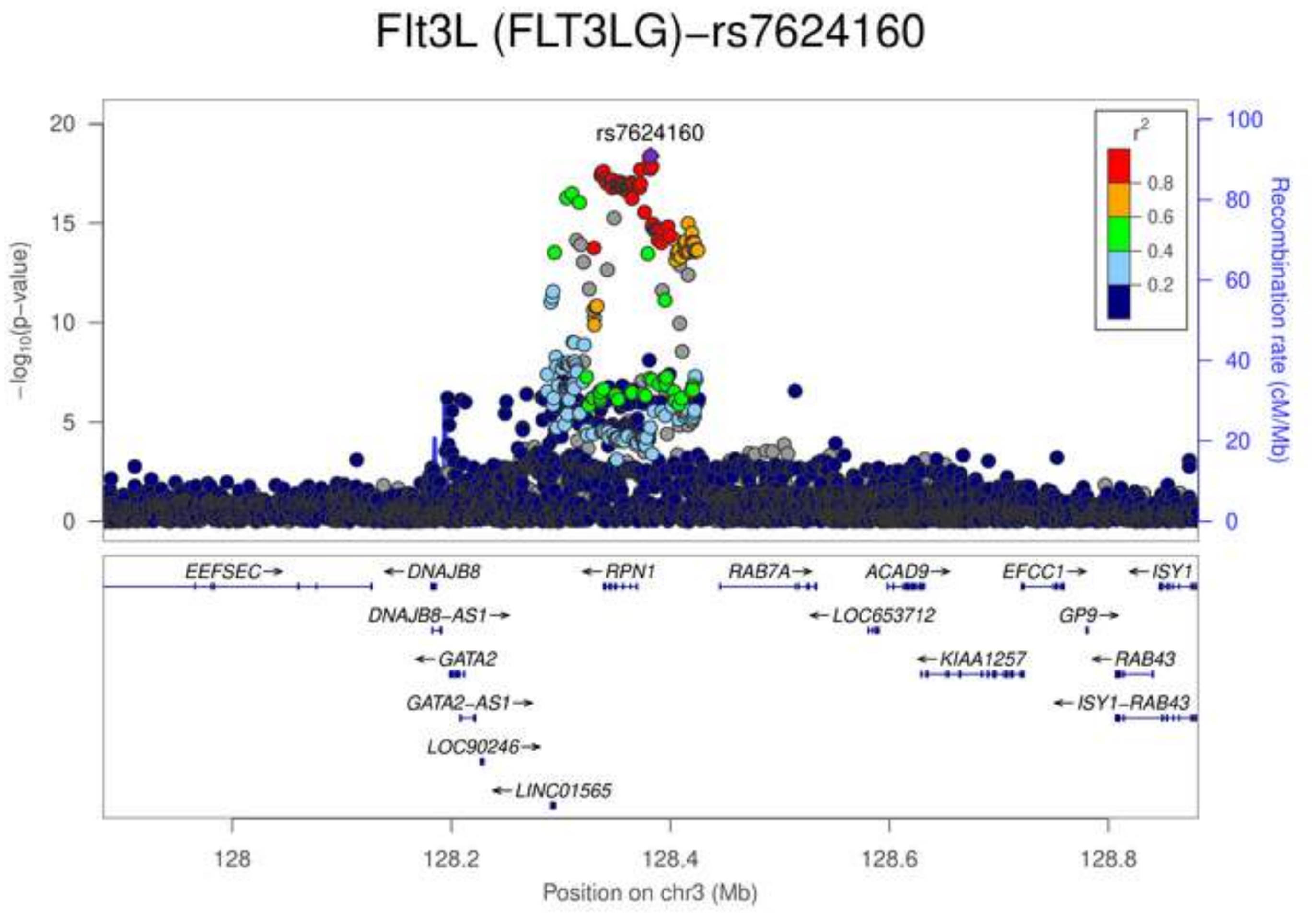
Flt3L (FLT3LG)–rs7624160
rs7624160
$-\log_{10}$(p-value)
Recombination rate (cM/Mb)
r^2
0.8
0.6
0.4
0.2
EEFSEC→
←DNAJB8
←RPN1
RAB7A→
ACAD9→
EFCC1→
←ISY1
DNAJB8–AS1→
←LOC653712
GP9→
←GATA2
←KIAA1257
←RAB43
GATA2–AS1→
←ISY1–RAB43
LOC90246→
←LINC01565
128
128.2
128.4
128.6
128.8
Position on chr3 (Mb)

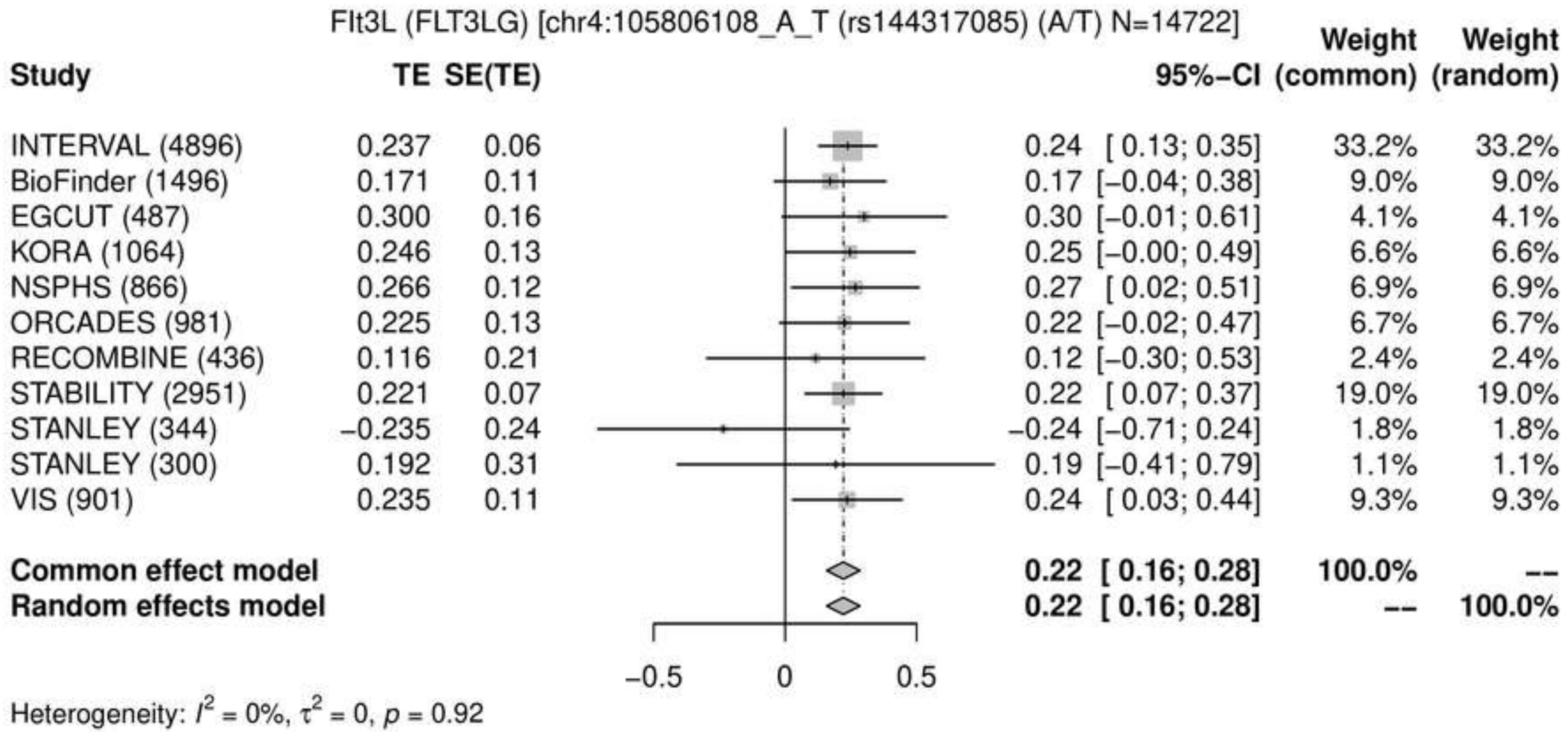
Flt3L (FLT3LG) [chr4:105806108_A_T (rs144317085) (A/T) N=14722]
Study TE SE(TE) 95%-CI Weight (common) Weight (random)
INTERVAL (4896) 0.237 0.06 0.24 [0.13; 0.35] 33.2% 33.2%
BioFinder (1496) 0.171 0.11 0.17 [−0.04; 0.38] 9.0% 9.0%
EGCUT (487) 0.300 0.16 0.30 [−0.01; 0.61] 4.1% 4.1%
KORA (1064) 0.246 0.13 0.25 [−0.00; 0.49] 6.6% 6.6%
NSPHS (866) 0.266 0.12 0.27 [0.02; 0.51] 6.9% 6.9%
ORCADES (981) 0.225 0.13 0.22 [−0.02; 0.47] 6.7% 6.7%
RECOMBINE (436) 0.116 0.21 0.12 [−0.30; 0.53] 2.4% 2.4%
STABILITY (2951) 0.221 0.07 0.22 [0.07; 0.37] 19.0% 19.0%
STANLEY (344) −0.235 0.24 −0.24 [−0.71; 0.24] 1.8% 1.8%
STANLEY (300) 0.192 0.31 0.19 [−0.41; 0.79] 1.1% 1.1%
VIS (901) 0.235 0.11 0.24 [0.03; 0.44] 9.3% 9.3%
Common effect model 0.22 [0.16; 0.28] 100.0% --
Random effects model 0.22 [0.16; 0.28] -- 100.0%
−0.5 0 0.5
Heterogeneity: I^2 = 0%, τ^2 = 0, p = 0.92

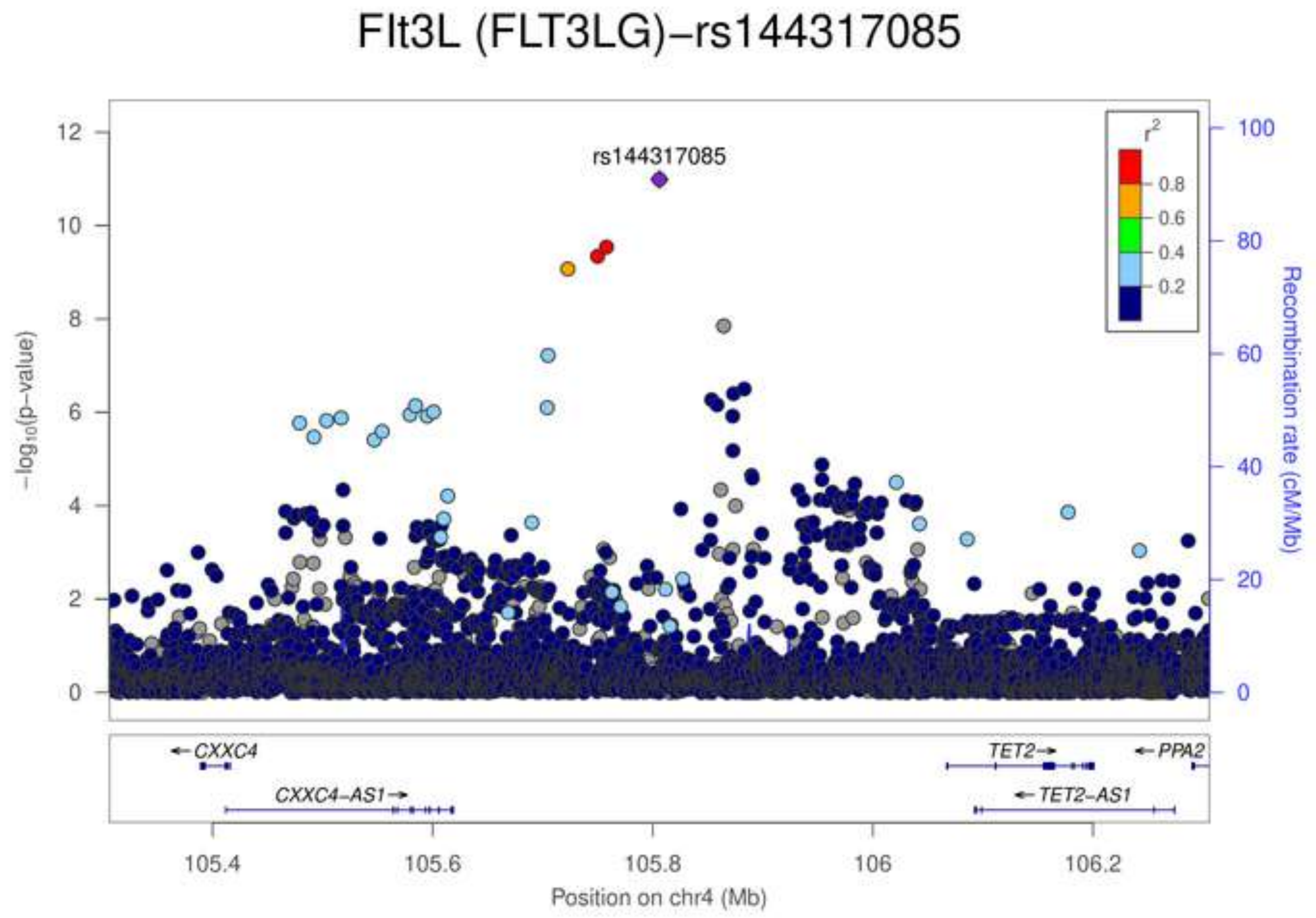
Flt3L (FLT3LG)−rs144317085
rs144317085
$-\log_{10}$(p-value)
Recombination rate (cM/Mb)
r^2
0.8
0.6
0.4
0.2
0 2 4 6 8 10 12
0 20 40 60 80 100
CXXC4
CXXC4-AS1
TET2
TET2-AS1
PPA2
105.4 105.6 105.8 106 106.2
Position on chr4 (Mb)

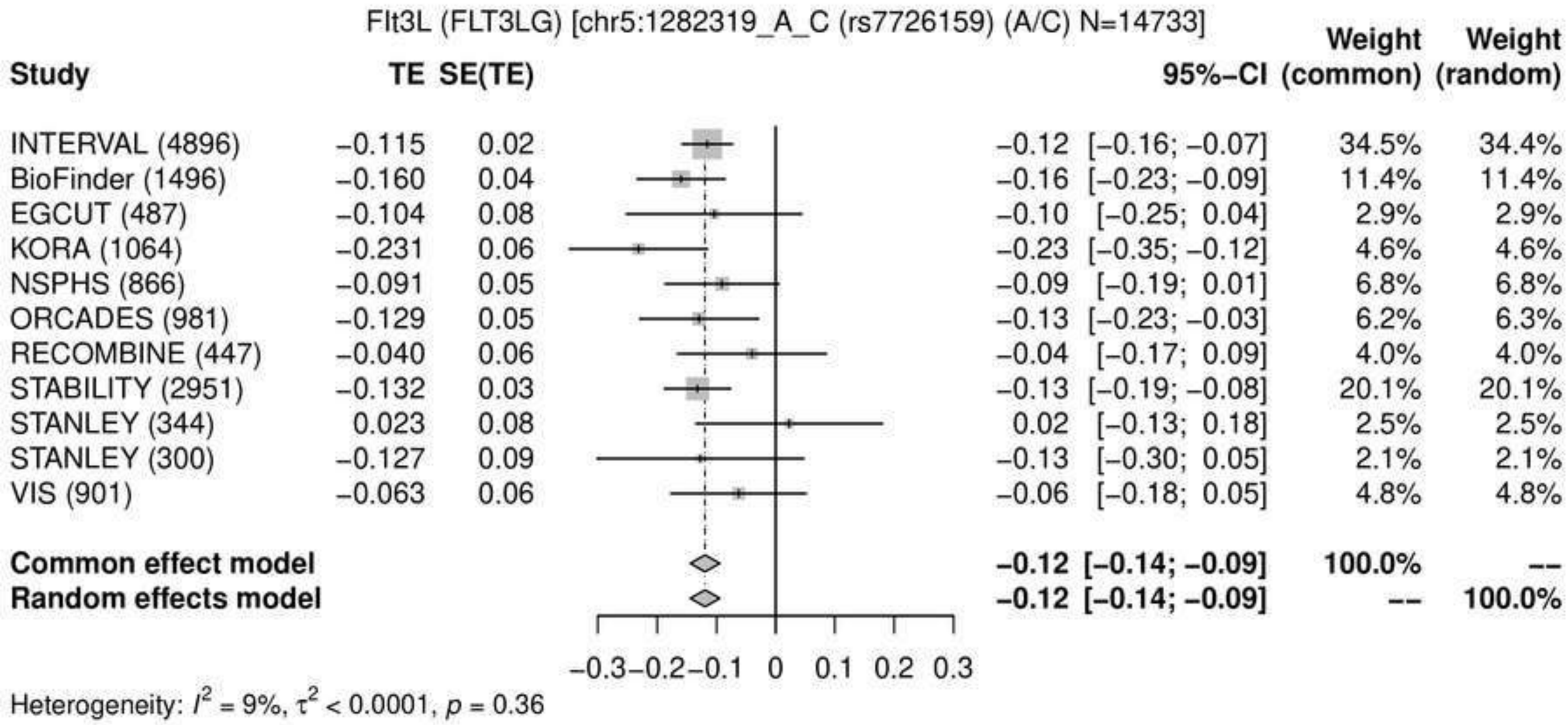
Flt3L (FLT3LG) [chr5:1282319_A_C (rs7726159) (A/C) N=14733]
Study
TE
SE(TE)
95%−CI
Weight (common)
Weight (random)
INTERVAL (4896) −0.115 0.02 −0.12 [−0.16; −0.07] 34.5% 34.4%
BioFinder (1496) −0.160 0.04 −0.16 [−0.23; −0.09] 11.4% 11.4%
EGCUT (487) −0.104 0.08 −0.10 [−0.25; 0.04] 2.9% 2.9%
KORA (1064) −0.231 0.06 −0.23 [−0.35; −0.12] 4.6% 4.6%
NSPHS (866) −0.091 0.05 −0.09 [−0.19; 0.01] 6.8% 6.8%
ORCADES (981) −0.129 0.05 −0.13 [−0.23; −0.03] 6.2% 6.3%
RECOMBINE (447) −0.040 0.06 −0.04 [−0.17; 0.09] 4.0% 4.0%
STABILITY (2951) −0.132 0.03 −0.13 [−0.19; −0.08] 20.1% 20.1%
STANLEY (344) 0.023 0.08 0.02 [−0.13; 0.18] 2.5% 2.5%
STANLEY (300) −0.127 0.09 −0.13 [−0.30; 0.05] 2.1% 2.1%
VIS (901) −0.063 0.06 −0.06 [−0.18; 0.05] 4.8% 4.8%
Common effect model −0.12 [−0.14; −0.09] 100.0% --
Random effects model −0.12 [−0.14; −0.09] -- 100.0%
−0.3 −0.2 −0.1 0 0.1 0.2 0.3
Heterogeneity: I^2 = 9%, τ^2 < 0.0001, p = 0.36

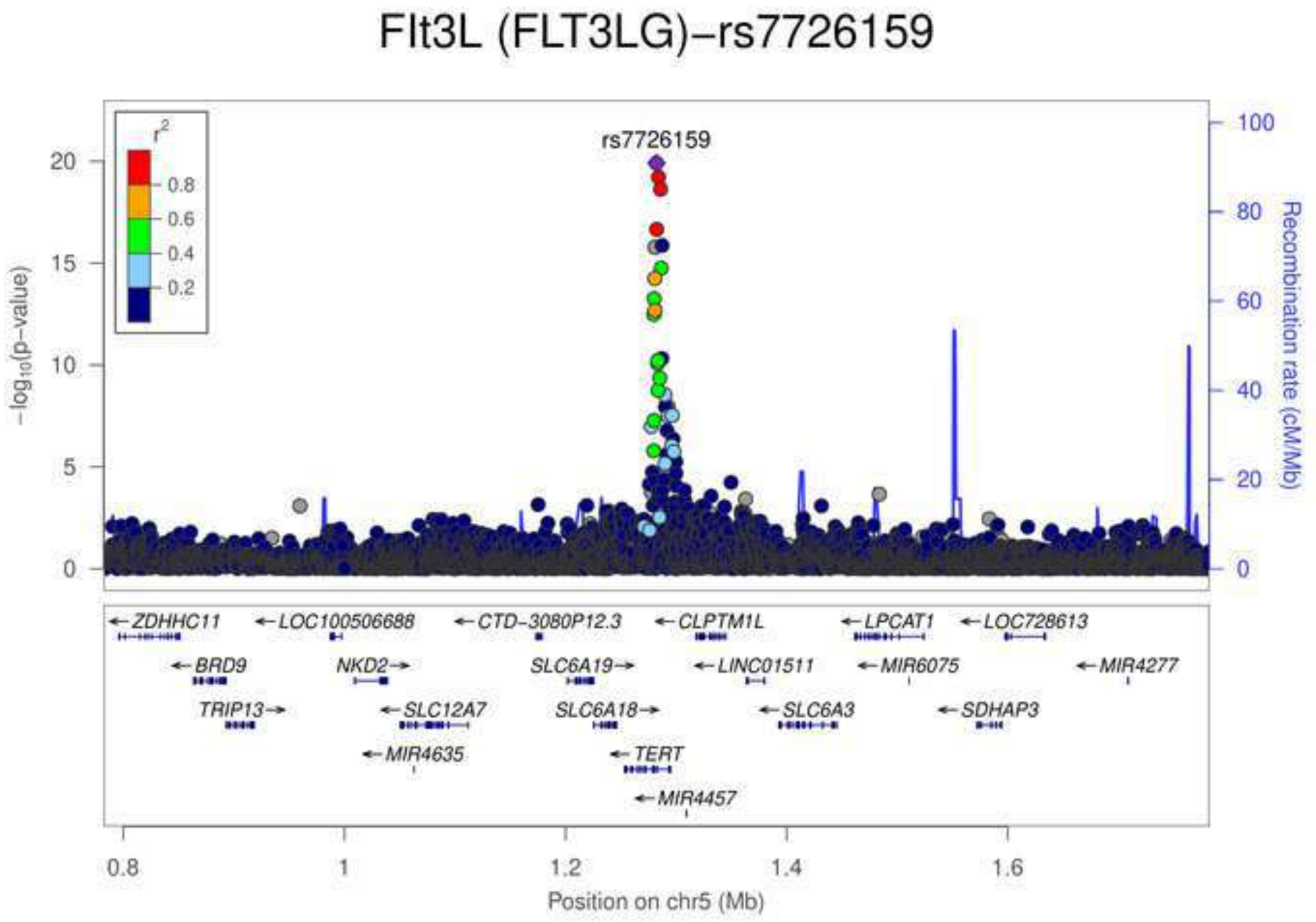
Flt3L (FLT3LG)−rs7726159
rs7726159
r^2
0.8
0.6
0.4
0.2
$-\log_{10}$(p-value)
Recombination rate (cM/Mb)
ZDHHC11
LOC100506688
CTD-3080P12.3
CLPTM1L
LPCAT1
LOC728613
BRD9
NKD2
SLC6A19
LINC01511
MIR6075
MIR4277
TRIP13
SLC12A7
SLC6A18
SLC6A3
SDHAP3
MIR4635
TERT
MIR4457
0.8
1
1.2
1.4
1.6
Position on chr5 (Mb)

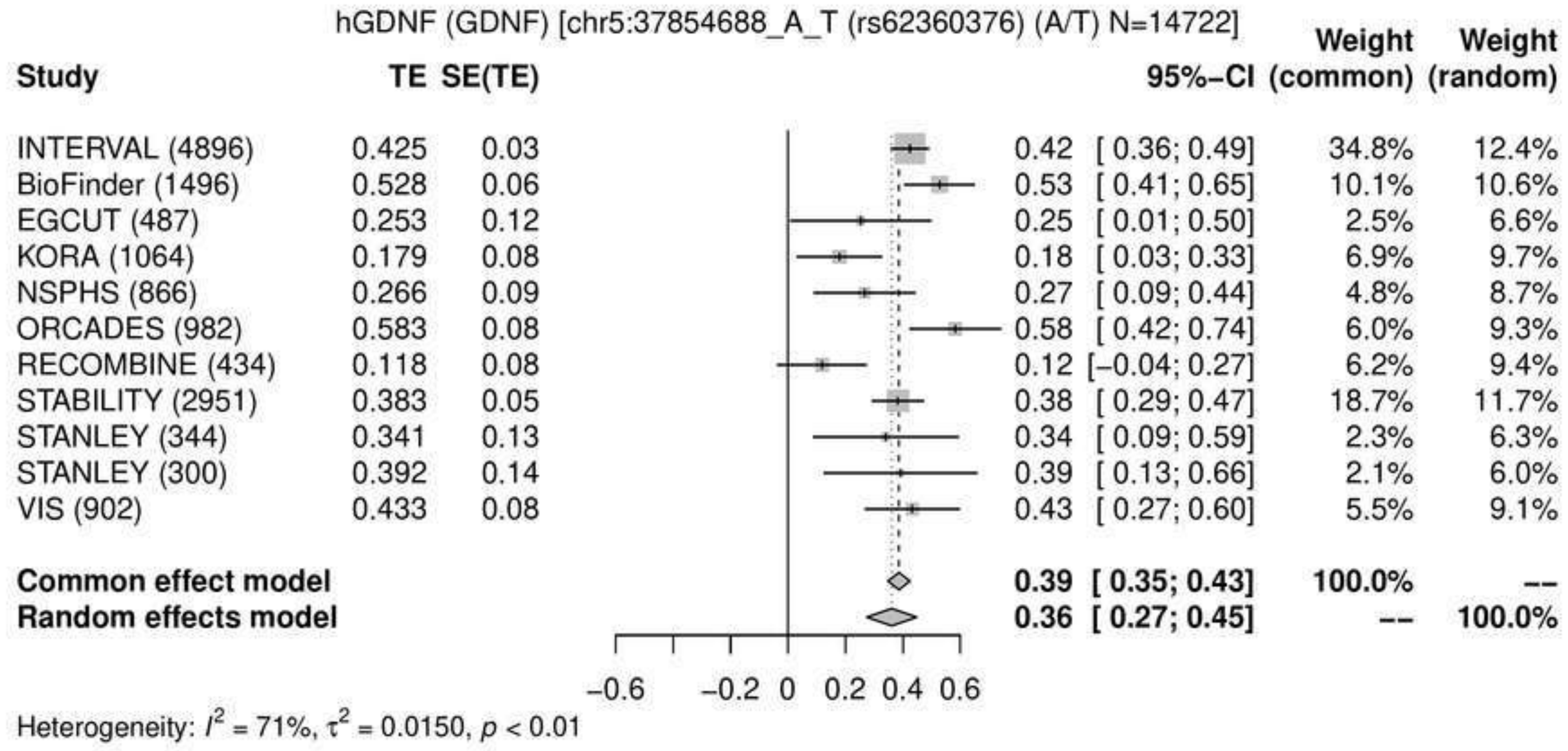
hGDNF (GDNF) [chr5:37854688_A_T (rs62360376) (A/T) N=14722]
Study TE SE(TE) 95%−CI Weight (common) Weight (random)
INTERVAL (4896) 0.425 0.03 0.42 [0.36; 0.49] 34.8% 12.4%
BioFinder (1496) 0.528 0.06 0.53 [0.41; 0.65] 10.1% 10.6%
EGCUT (487) 0.253 0.12 0.25 [0.01; 0.50] 2.5% 6.6%
KORA (1064) 0.179 0.08 0.18 [0.03; 0.33] 6.9% 9.7%
NSPHS (866) 0.266 0.09 0.27 [0.09; 0.44] 4.8% 8.7%
ORCADES (982) 0.583 0.08 0.58 [0.42; 0.74] 6.0% 9.3%
RECOMBINE (434) 0.118 0.08 0.12 [−0.04; 0.27] 6.2% 9.4%
STABILITY (2951) 0.383 0.05 0.38 [0.29; 0.47] 18.7% 11.7%
STANLEY (344) 0.341 0.13 0.34 [0.09; 0.59] 2.3% 6.3%
STANLEY (300) 0.392 0.14 0.39 [0.13; 0.66] 2.1% 6.0%
VIS (902) 0.433 0.08 0.43 [0.27; 0.60] 5.5% 9.1%
Common effect model 0.39 [0.35; 0.43] 100.0% --
Random effects model 0.36 [0.27; 0.45] -- 100.0%
−0.6 −0.2 0 0.2 0.4 0.6
Heterogeneity: I^2 = 71%, τ^2 = 0.0150, p < 0.01

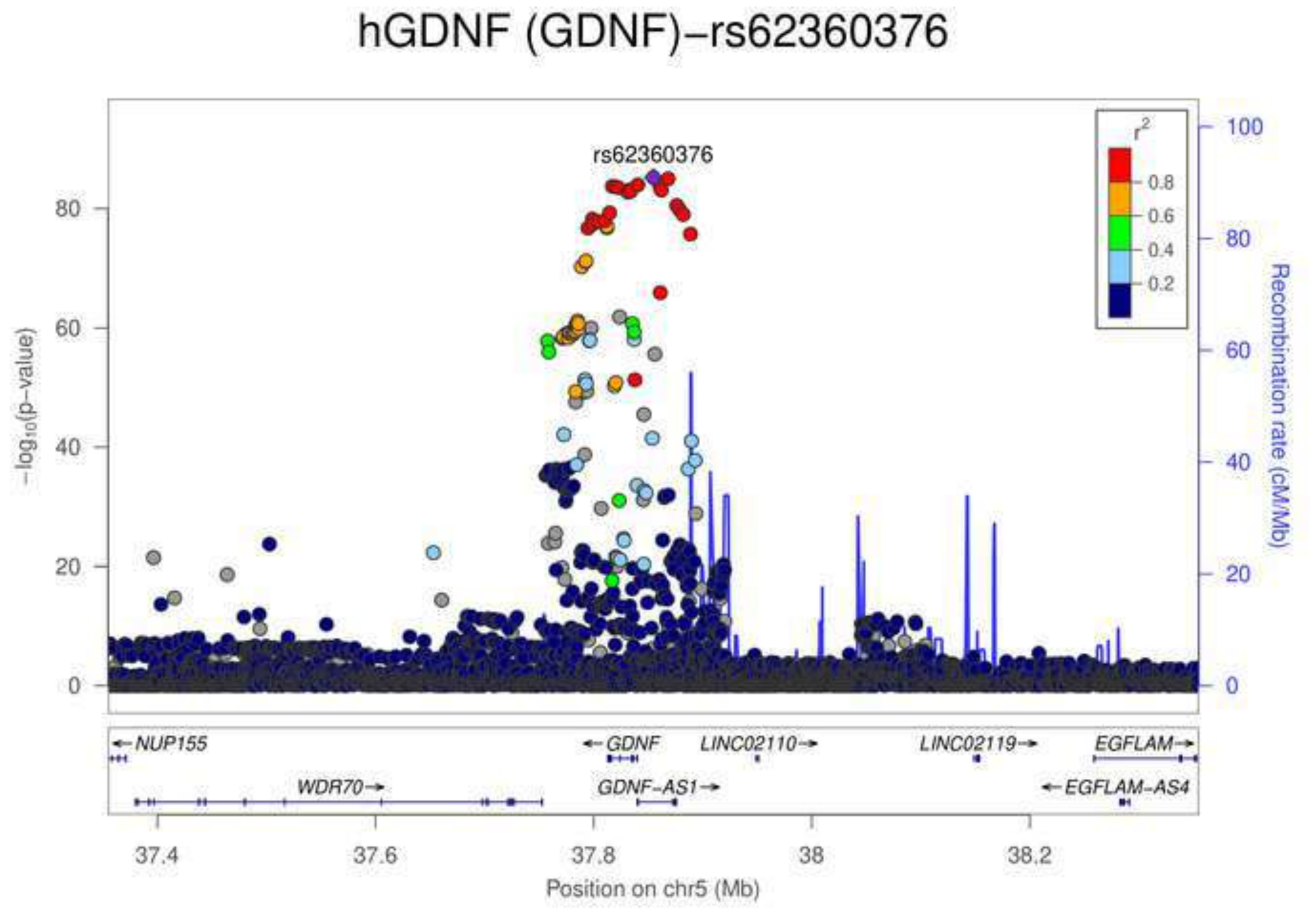
hGDNF (GDNF)−rs62360376
rs62360376
$-\log_{10}$(p−value)
Recombination rate (cM/Mb)
r^2
0.8
0.6
0.4
0.2
0
20
40
60
80
100
NUP155
WDR70
GDNF
GDNF-AS1
LINC02110
LINC02119
EGFLAM
EGFLAM-AS4
37.4
37.6
37.8
38
38.2
Position on chr5 (Mb)

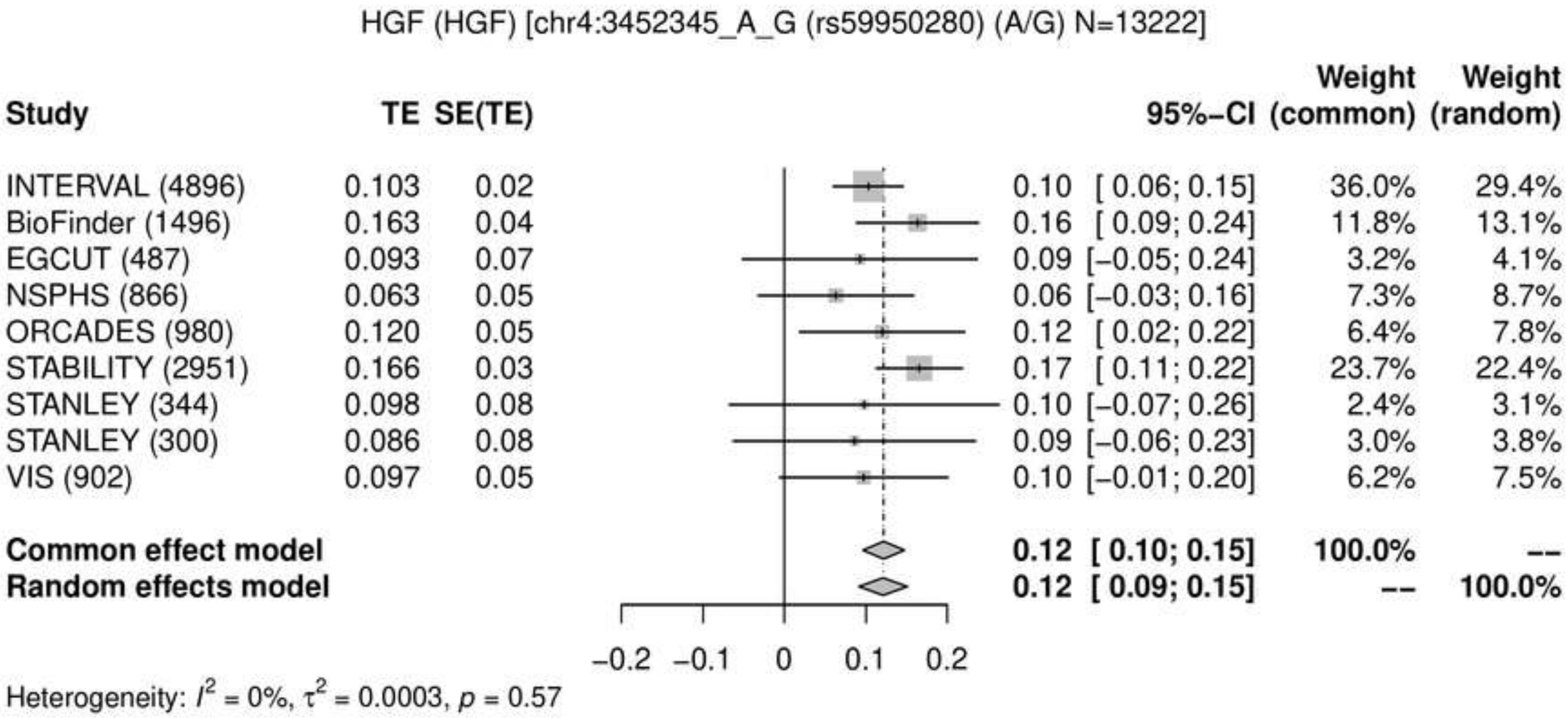
HGF (HGF) [chr4:3452345_A_G (rs59950280) (A/G) N=13222]
Study
TE
SE(TE)
95%-CI
Weight (common)
Weight (random)
INTERVAL (4896) 0.103 0.02 0.10 [0.06; 0.15] 36.0% 29.4%
BioFinder (1496) 0.163 0.04 0.16 [0.09; 0.24] 11.8% 13.1%
EGCUT (487) 0.093 0.07 0.09 [−0.05; 0.24] 3.2% 4.1%
NSPHS (866) 0.063 0.05 0.06 [−0.03; 0.16] 7.3% 8.7%
ORCADES (980) 0.120 0.05 0.12 [0.02; 0.22] 6.4% 7.8%
STABILITY (2951) 0.166 0.03 0.17 [0.11; 0.22] 23.7% 22.4%
STANLEY (344) 0.098 0.08 0.10 [−0.07; 0.26] 2.4% 3.1%
STANLEY (300) 0.086 0.08 0.09 [−0.06; 0.23] 3.0% 3.8%
VIS (902) 0.097 0.05 0.10 [−0.01; 0.20] 6.2% 7.5%
Common effect model 0.12 [0.10; 0.15] 100.0% --
Random effects model 0.12 [0.09; 0.15] -- 100.0%
−0.2 −0.1 0 0.1 0.2
Heterogeneity: I^2 = 0%, τ^2 = 0.0003, p = 0.57
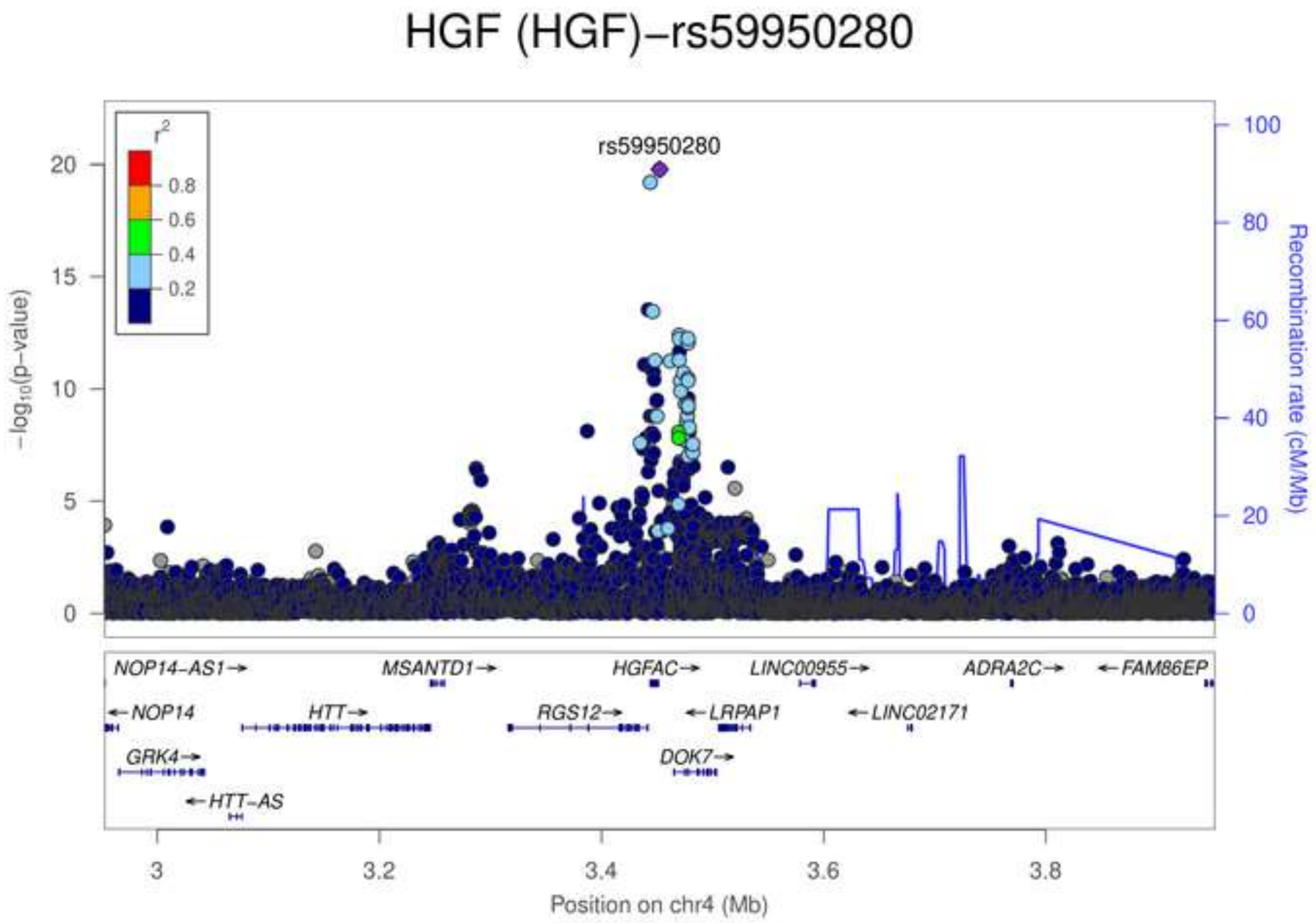
HGF (HGF)−rs59950280
rs59950280
r^2
0.8
0.6
0.4
0.2
$-\log_{10}$(p-value)
Recombination rate (cM/Mb)
NOP14-AS1→
MSANTD1→
HGFAC→
LINC00955→
ADRA2C→
←FAM86EP
←NOP14
HTT→
RGS12→
←LRPAP1
←LINC02171
GRK4→
DOK7→
←HTT-AS
3 3.2 3.4 3.6 3.8
Position on chr4 (Mb)

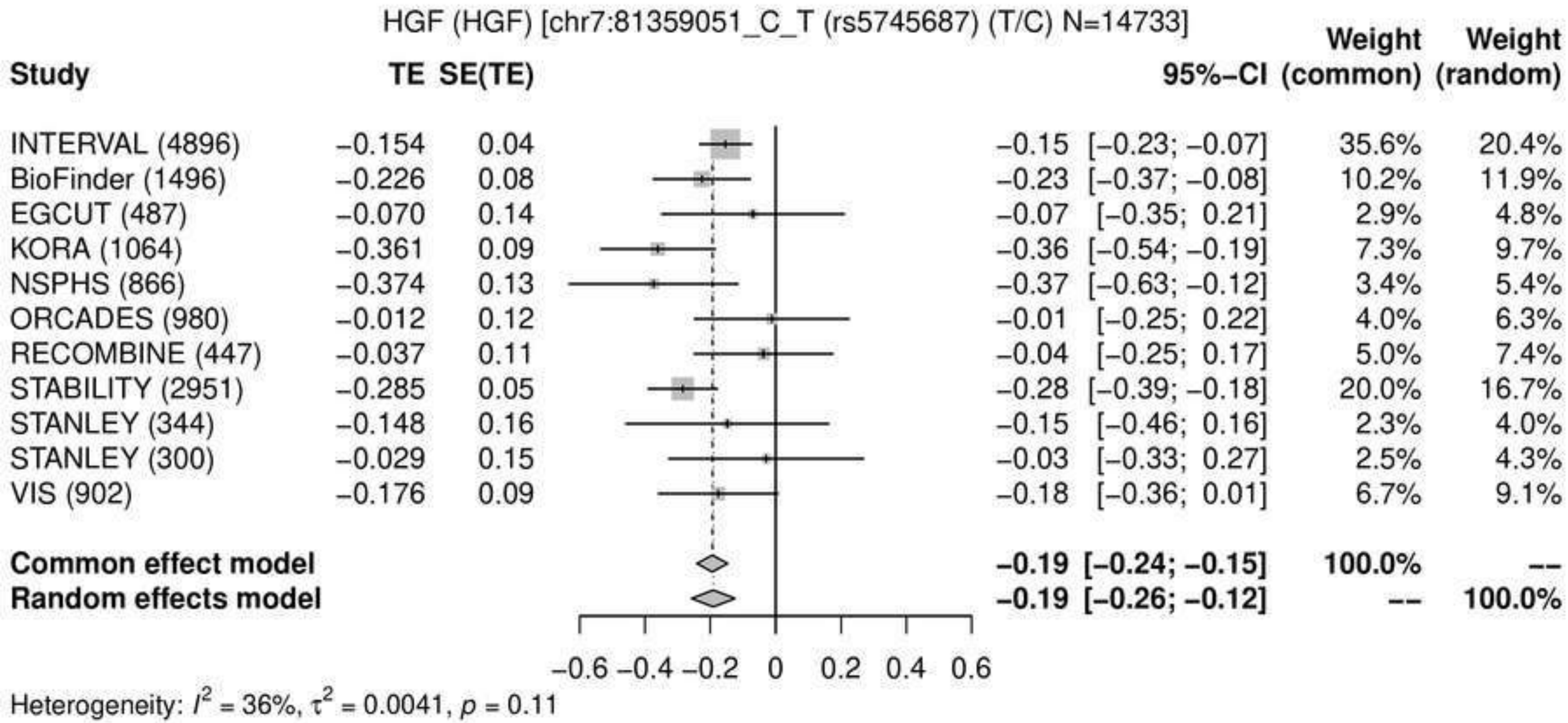
HGF (HGF) [chr7:81359051_C_T (rs5745687) (T/C) N=14733]
Study
TE
SE(TE)
95%−CI
Weight (common)
Weight (random)
INTERVAL (4896) −0.154 0.04 −0.15 [−0.23; −0.07] 35.6% 20.4%
BioFinder (1496) −0.226 0.08 −0.23 [−0.37; −0.08] 10.2% 11.9%
EGCUT (487) −0.070 0.14 −0.07 [−0.35; 0.21] 2.9% 4.8%
KORA (1064) −0.361 0.09 −0.36 [−0.54; −0.19] 7.3% 9.7%
NSPHS (866) −0.374 0.13 −0.37 [−0.63; −0.12] 3.4% 5.4%
ORCADES (980) −0.012 0.12 −0.01 [−0.25; 0.22] 4.0% 6.3%
RECOMBINE (447) −0.037 0.11 −0.04 [−0.25; 0.17] 5.0% 7.4%
STABILITY (2951) −0.285 0.05 −0.28 [−0.39; −0.18] 20.0% 16.7%
STANLEY (344) −0.148 0.16 −0.15 [−0.46; 0.16] 2.3% 4.0%
STANLEY (300) −0.029 0.15 −0.03 [−0.33; 0.27] 2.5% 4.3%
VIS (902) −0.176 0.09 −0.18 [−0.36; 0.01] 6.7% 9.1%
Common effect model −0.19 [−0.24; −0.15] 100.0% --
Random effects model −0.19 [−0.26; −0.12] -- 100.0%
−0.6 −0.4 −0.2 0 0.2 0.4 0.6
Heterogeneity: I^2 = 36%, τ^2 = 0.0041, p = 0.11

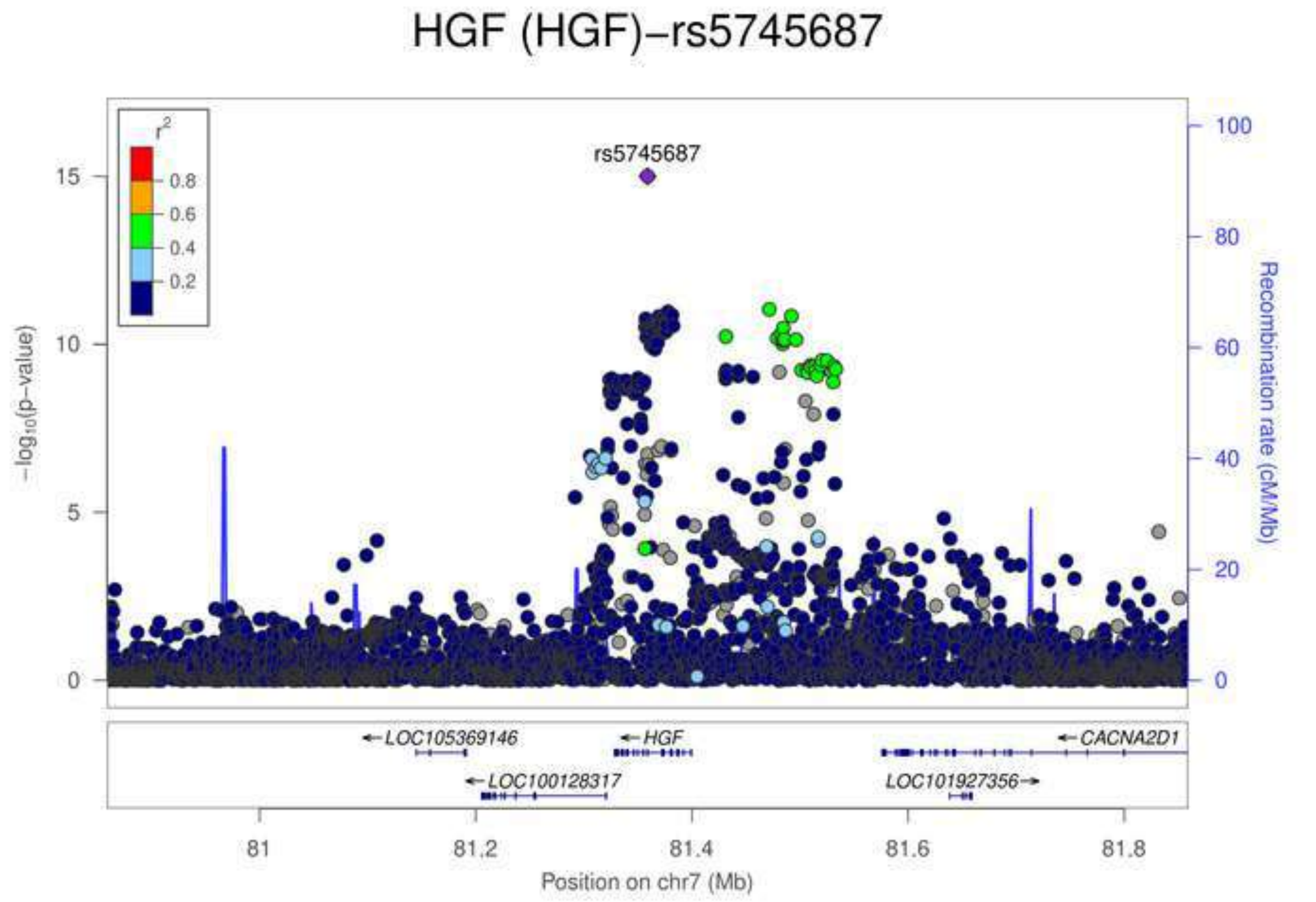
HGF (HGF)−rs5745687
rs5745687
r^2
0.8
0.6
0.4
0.2
$-\log_{10}$(p-value)
Recombination rate (cM/Mb)
LOC105369146
HGF
CACNA2D1
LOC100128317
LOC101927356
81
81.2
81.4
81.6
81.8
Position on chr7 (Mb)

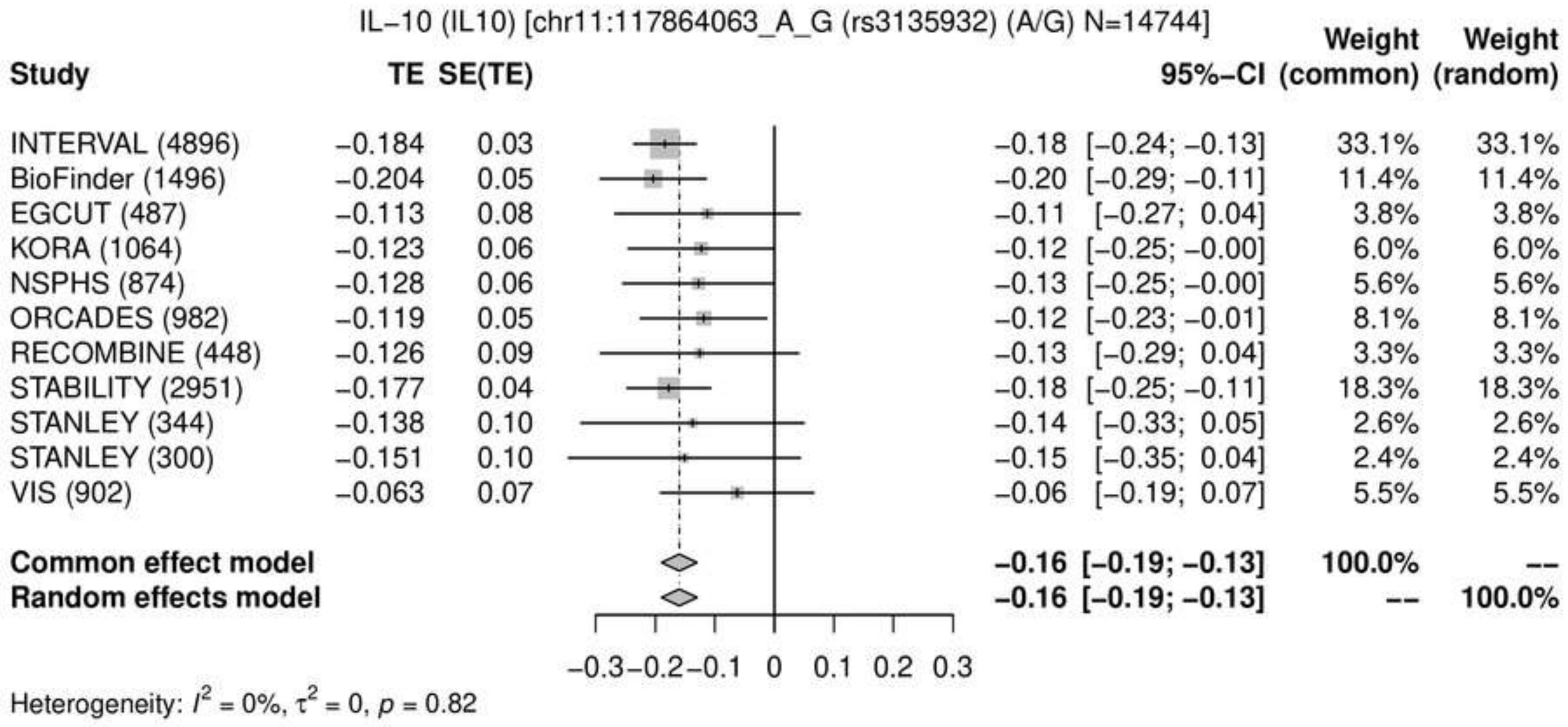
IL−10 (IL10) [chr11:117864063_A_G (rs3135932) (A/G) N=14744]
Study
TE
SE(TE)
95%−CI
Weight (common)
Weight (random)
INTERVAL (4896) −0.184 0.03 −0.18 [−0.24; −0.13] 33.1% 33.1%
BioFinder (1496) −0.204 0.05 −0.20 [−0.29; −0.11] 11.4% 11.4%
EGCUT (487) −0.113 0.08 −0.11 [−0.27; 0.04] 3.8% 3.8%
KORA (1064) −0.123 0.06 −0.12 [−0.25; −0.00] 6.0% 6.0%
NSPHS (874) −0.128 0.06 −0.13 [−0.25; −0.00] 5.6% 5.6%
ORCADES (982) −0.119 0.05 −0.12 [−0.23; −0.01] 8.1% 8.1%
RECOMBINE (448) −0.126 0.09 −0.13 [−0.29; 0.04] 3.3% 3.3%
STABILITY (2951) −0.177 0.04 −0.18 [−0.25; −0.11] 18.3% 18.3%
STANLEY (344) −0.138 0.10 −0.14 [−0.33; 0.05] 2.6% 2.6%
STANLEY (300) −0.151 0.10 −0.15 [−0.35; 0.04] 2.4% 2.4%
VIS (902) −0.063 0.07 −0.06 [−0.19; 0.07] 5.5% 5.5%
Common effect model −0.16 [−0.19; −0.13] 100.0% --
Random effects model −0.16 [−0.19; −0.13] -- 100.0%
−0.3 −0.2 −0.1 0 0.1 0.2 0.3
Heterogeneity: I^2 = 0%, τ^2 = 0, p = 0.82

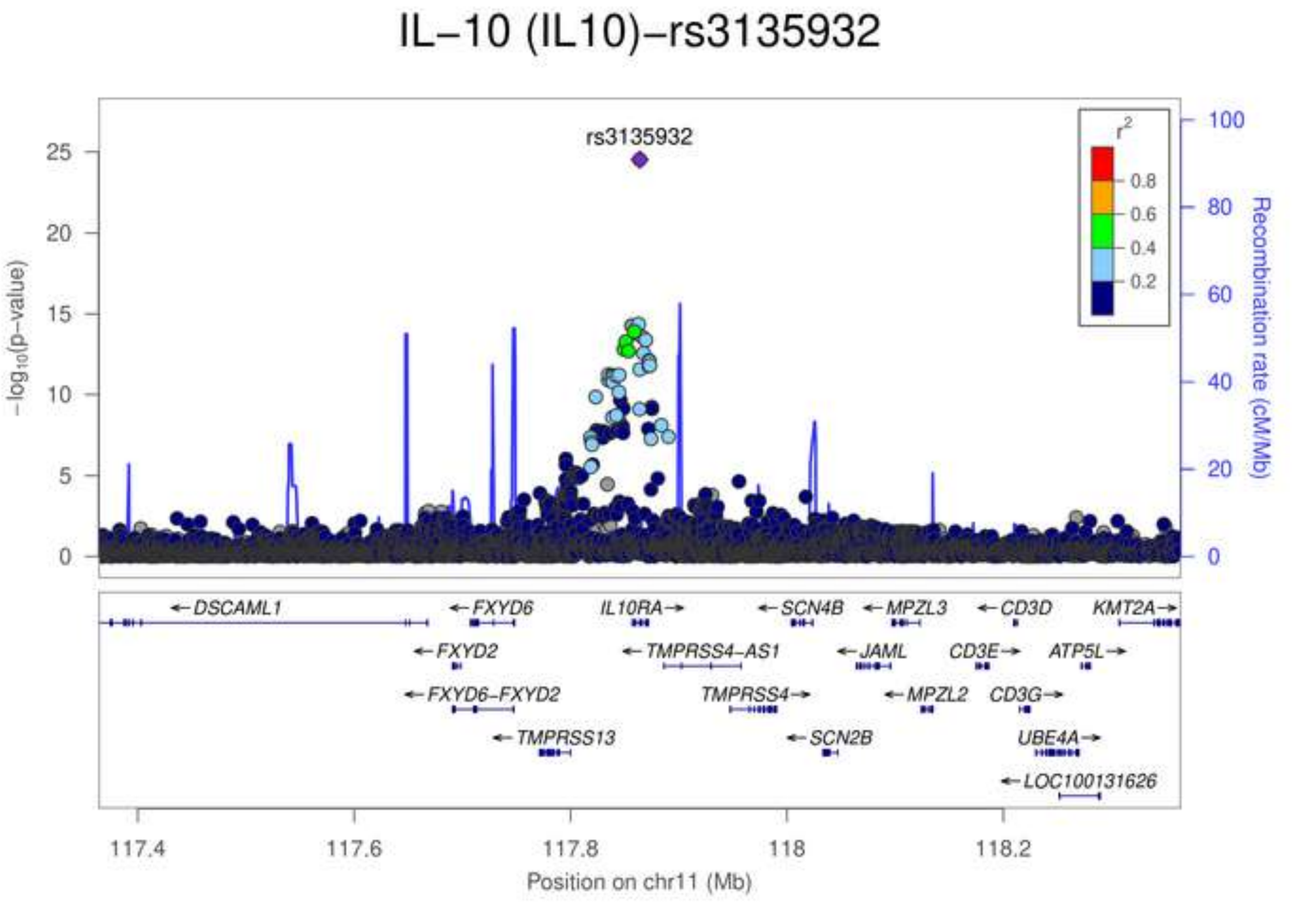
IL−10 (IL10)−rs3135932
rs3135932
$-\log_{10}$(p-value)
Recombination rate (cM/Mb)
r^2
0.8
0.6
0.4
0.2
DSCAML1
FXYD6
FXYD2
FXYD6−FXYD2
TMPRSS13
IL10RA
TMPRSS4−AS1
TMPRSS4
SCN4B
SCN2B
MPZL3
JAML
MPZL2
CD3D
CD3E
CD3G
UBE4A
LOC100131626
KMT2A
ATP5L
117.4
117.6
117.8
118
118.2
Position on chr11 (Mb)

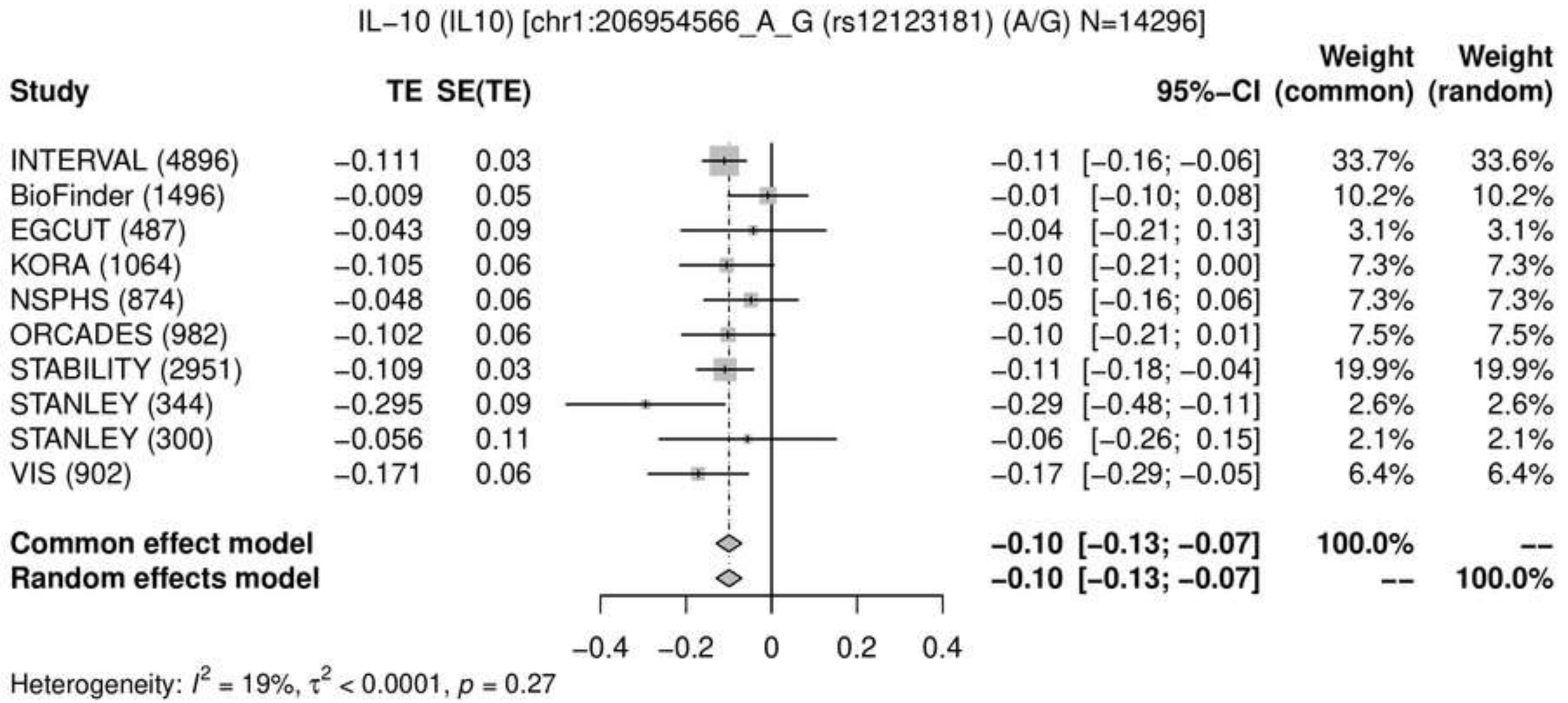
IL−10 (IL10) [chr1:206954566_A_G (rs12123181) (A/G) N=14296]
Study
TE
SE(TE)
95%−CI
Weight (common)
Weight (random)
INTERVAL (4896) −0.111 0.03 −0.11 [−0.16; −0.06] 33.7% 33.6%
BioFinder (1496) −0.009 0.05 −0.01 [−0.10; 0.08] 10.2% 10.2%
EGCUT (487) −0.043 0.09 −0.04 [−0.21; 0.13] 3.1% 3.1%
KORA (1064) −0.105 0.06 −0.10 [−0.21; 0.00] 7.3% 7.3%
NSPHS (874) −0.048 0.06 −0.05 [−0.16; 0.06] 7.3% 7.3%
ORCADES (982) −0.102 0.06 −0.10 [−0.21; 0.01] 7.5% 7.5%
STABILITY (2951) −0.109 0.03 −0.11 [−0.18; −0.04] 19.9% 19.9%
STANLEY (344) −0.295 0.09 −0.29 [−0.48; −0.11] 2.6% 2.6%
STANLEY (300) −0.056 0.11 −0.06 [−0.26; 0.15] 2.1% 2.1%
VIS (902) −0.171 0.06 −0.17 [−0.29; −0.05] 6.4% 6.4%
Common effect model −0.10 [−0.13; −0.07] 100.0% --
Random effects model −0.10 [−0.13; −0.07] -- 100.0%
−0.4 −0.2 0 0.2 0.4
Heterogeneity: I^2 = 19%, τ^2 < 0.0001, p = 0.27

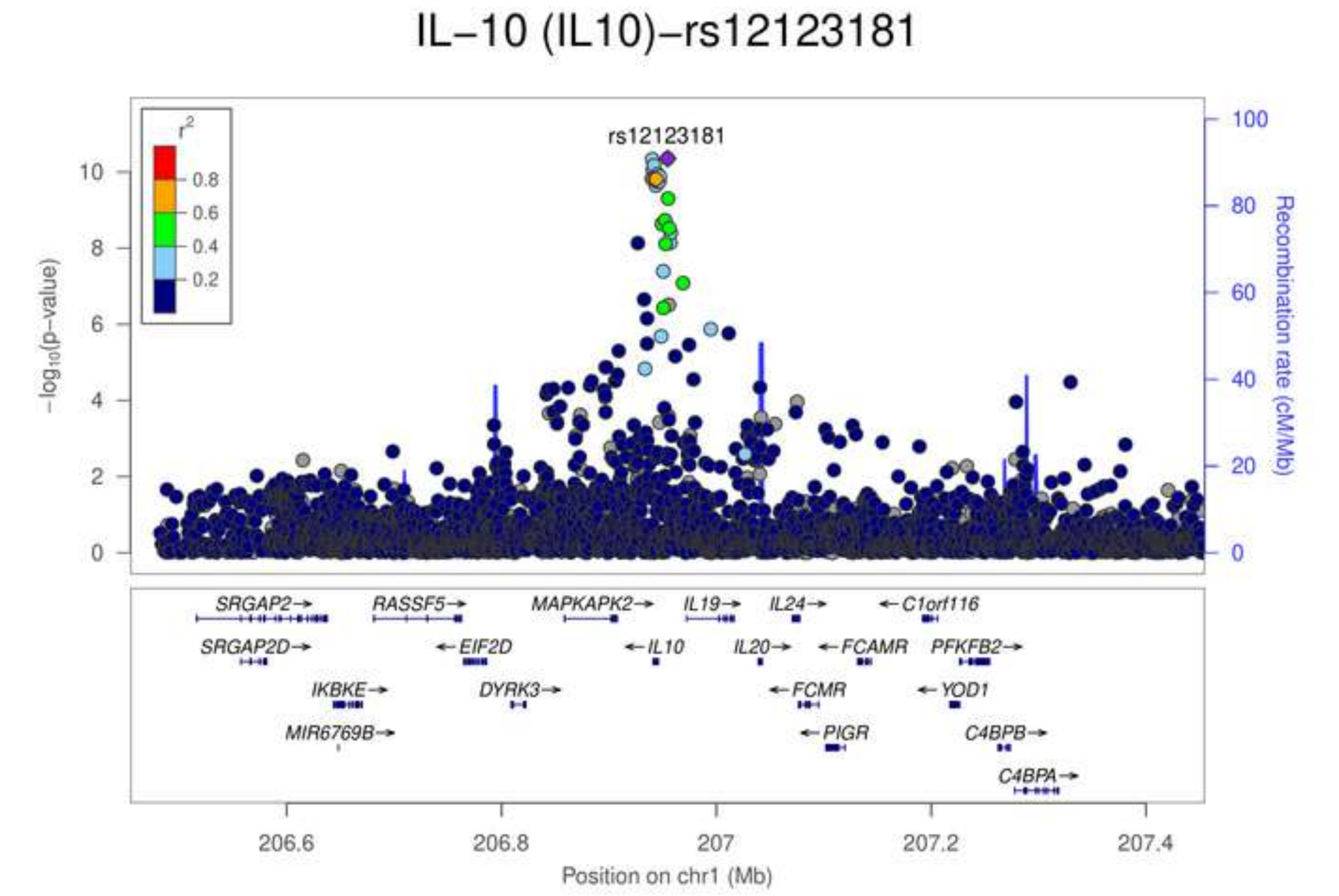
IL−10 (IL10)−rs12123181
rs12123181
r^2
0.8
0.6
0.4
0.2
$-\log_{10}$(p-value)
Recombination rate (cM/Mb)
SRGAP2→
RASSF5→
MAPKAPK2→
IL19→
IL24→
←C1orf116
SRGAP2D→
←EIF2D
←IL10
IL20→
←FCAMR
PFKFB2→
IKBKE→
DYRK3→
←FCMR
←YOD1
MIR6769B→
←PIGR
C4BPB→
C4BPA→
206.6
206.8
207
207.2
207.4
Position on chr1 (Mb)

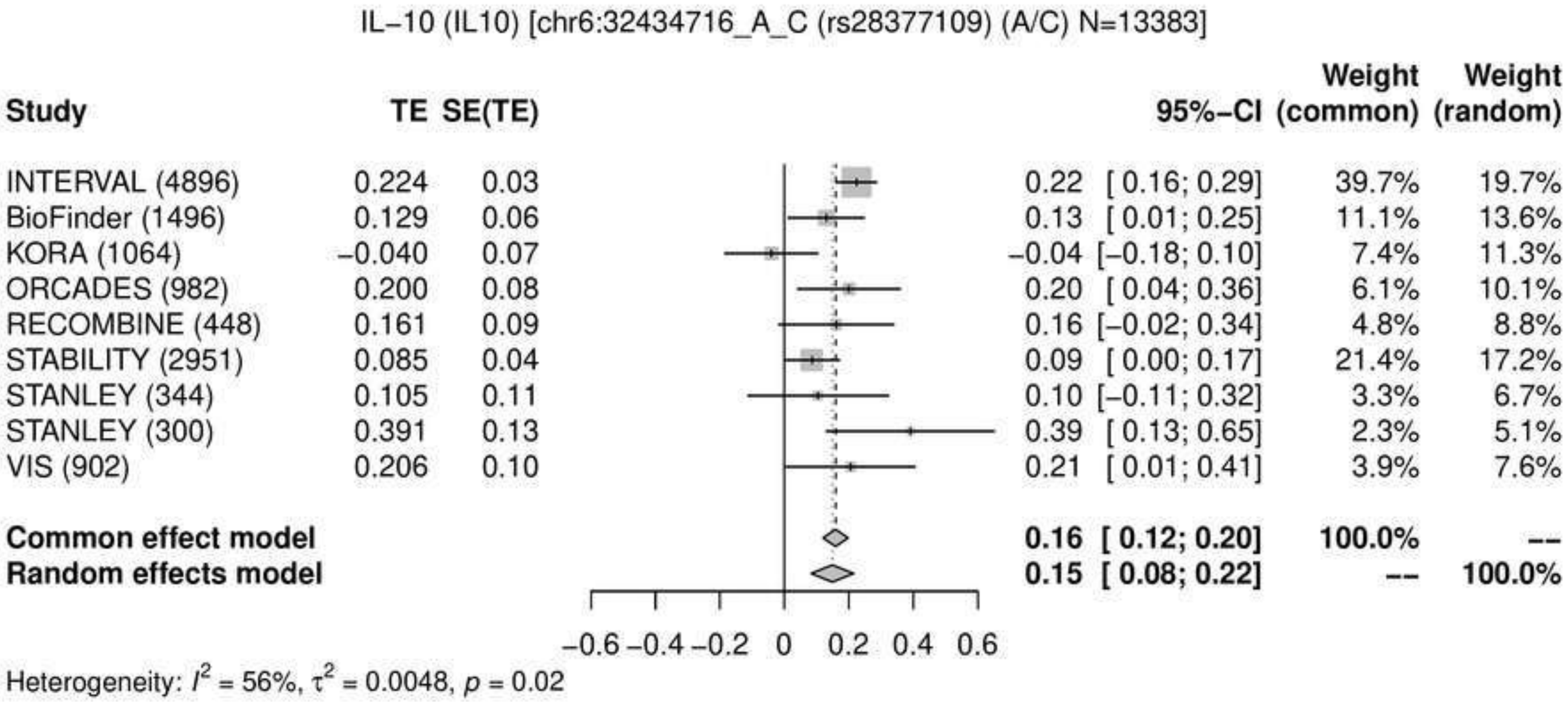
IL−10 (IL10) [chr6:32434716_A_C (rs28377109) (A/C) N=13383]
Study
TE
SE(TE)
95%−CI
Weight (common)
Weight (random)
INTERVAL (4896) 0.224 0.03 0.22 [0.16; 0.29] 39.7% 19.7%
BioFinder (1496) 0.129 0.06 0.13 [0.01; 0.25] 11.1% 13.6%
KORA (1064) −0.040 0.07 −0.04 [−0.18; 0.10] 7.4% 11.3%
ORCADES (982) 0.200 0.08 0.20 [0.04; 0.36] 6.1% 10.1%
RECOMBINE (448) 0.161 0.09 0.16 [−0.02; 0.34] 4.8% 8.8%
STABILITY (2951) 0.085 0.04 0.09 [0.00; 0.17] 21.4% 17.2%
STANLEY (344) 0.105 0.11 0.10 [−0.11; 0.32] 3.3% 6.7%
STANLEY (300) 0.391 0.13 0.39 [0.13; 0.65] 2.3% 5.1%
VIS (902) 0.206 0.10 0.21 [0.01; 0.41] 3.9% 7.6%
Common effect model 0.16 [0.12; 0.20] 100.0% --
Random effects model 0.15 [0.08; 0.22] -- 100.0%
−0.6 −0.4 −0.2 0 0.2 0.4 0.6
Heterogeneity: I^2 = 56%, τ^2 = 0.0048, p = 0.02

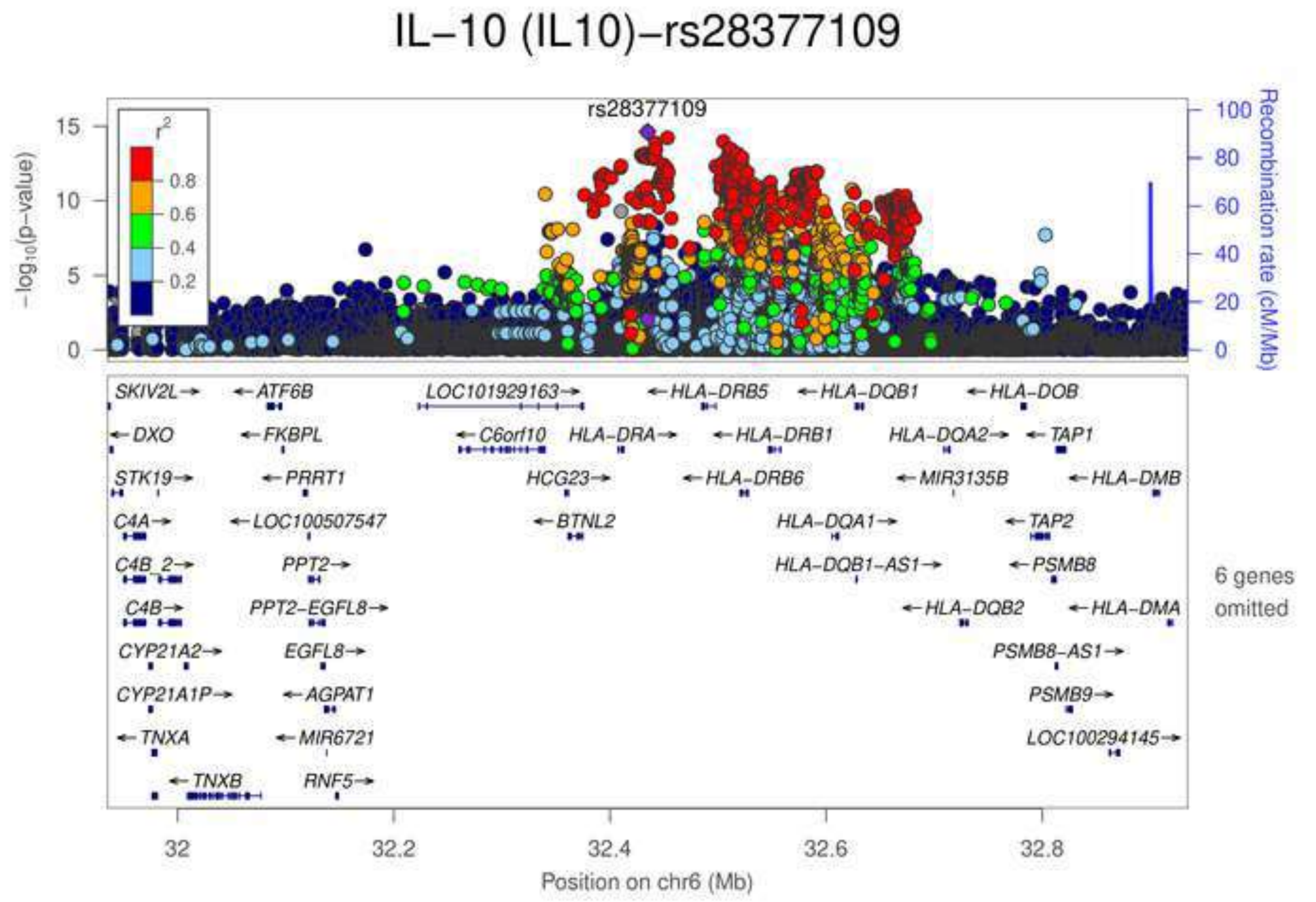
IL−10 (IL10)−rs28377109
rs28377109
$-\log_{10}$(p−value)
Recombination rate (cM/Mb)
r^2
0.8
0.6
0.4
0.2
SKIV2L ATF6B LOC101929163 HLA−DRB5 HLA−DQB1 HLA−DOB
DXO FKBPL C6orf10 HLA−DRA HLA−DRB1 HLA−DQA2 TAP1
STK19 PRRT1 HCG23 HLA−DRB6 MIR3135B HLA−DMB
C4A LOC100507547 BTNL2 HLA−DQA1 TAP2
C4B_2 PPT2 HLA−DQB1−AS1 PSMB8
C4B PPT2−EGFL8 HLA−DQB2 HLA−DMA
CYP21A2 EGFL8 PSMB8−AS1
CYP21A1P AGPAT1 PSMB9
TNXA MIR6721 LOC100294145
TNXB RNF5
6 genes omitted
32 32.2 32.4 32.6 32.8
Position on chr6 (Mb)

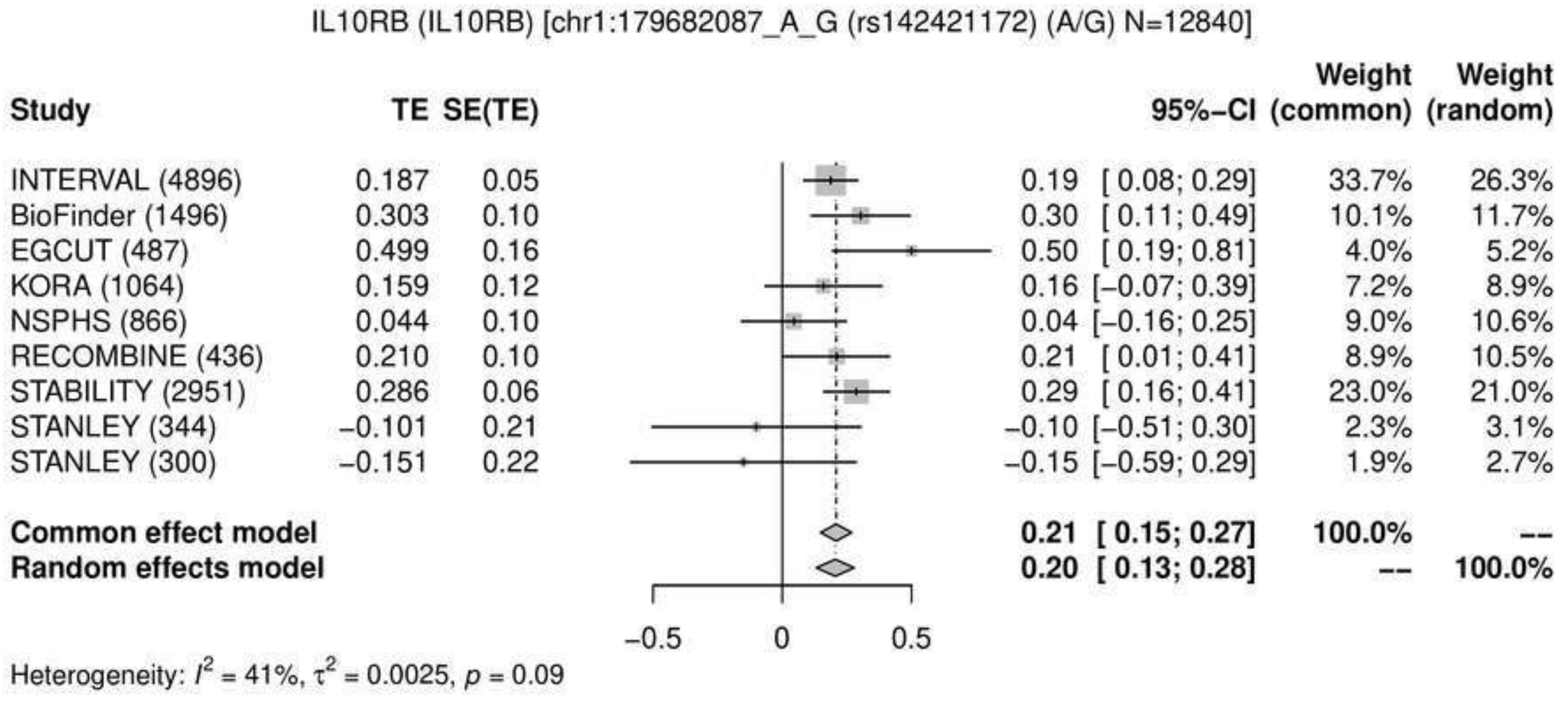
IL10RB (IL10RB) [chr1:179682087_A_G (rs142421172) (A/G) N=12840]
Study
TE
SE(TE)
95%-CI
Weight (common)
Weight (random)
INTERVAL (4896) 0.187 0.05 0.19 [0.08; 0.29] 33.7% 26.3%
BioFinder (1496) 0.303 0.10 0.30 [0.11; 0.49] 10.1% 11.7%
EGCUT (487) 0.499 0.16 0.50 [0.19; 0.81] 4.0% 5.2%
KORA (1064) 0.159 0.12 0.16 [−0.07; 0.39] 7.2% 8.9%
NSPHS (866) 0.044 0.10 0.04 [−0.16; 0.25] 9.0% 10.6%
RECOMBINE (436) 0.210 0.10 0.21 [0.01; 0.41] 8.9% 10.5%
STABILITY (2951) 0.286 0.06 0.29 [0.16; 0.41] 23.0% 21.0%
STANLEY (344) −0.101 0.21 −0.10 [−0.51; 0.30] 2.3% 3.1%
STANLEY (300) −0.151 0.22 −0.15 [−0.59; 0.29] 1.9% 2.7%
Common effect model 0.21 [0.15; 0.27] 100.0% --
Random effects model 0.20 [0.13; 0.28] -- 100.0%
−0.5
0
0.5
Heterogeneity: I^2 = 41%, τ^2 = 0.0025, p = 0.09

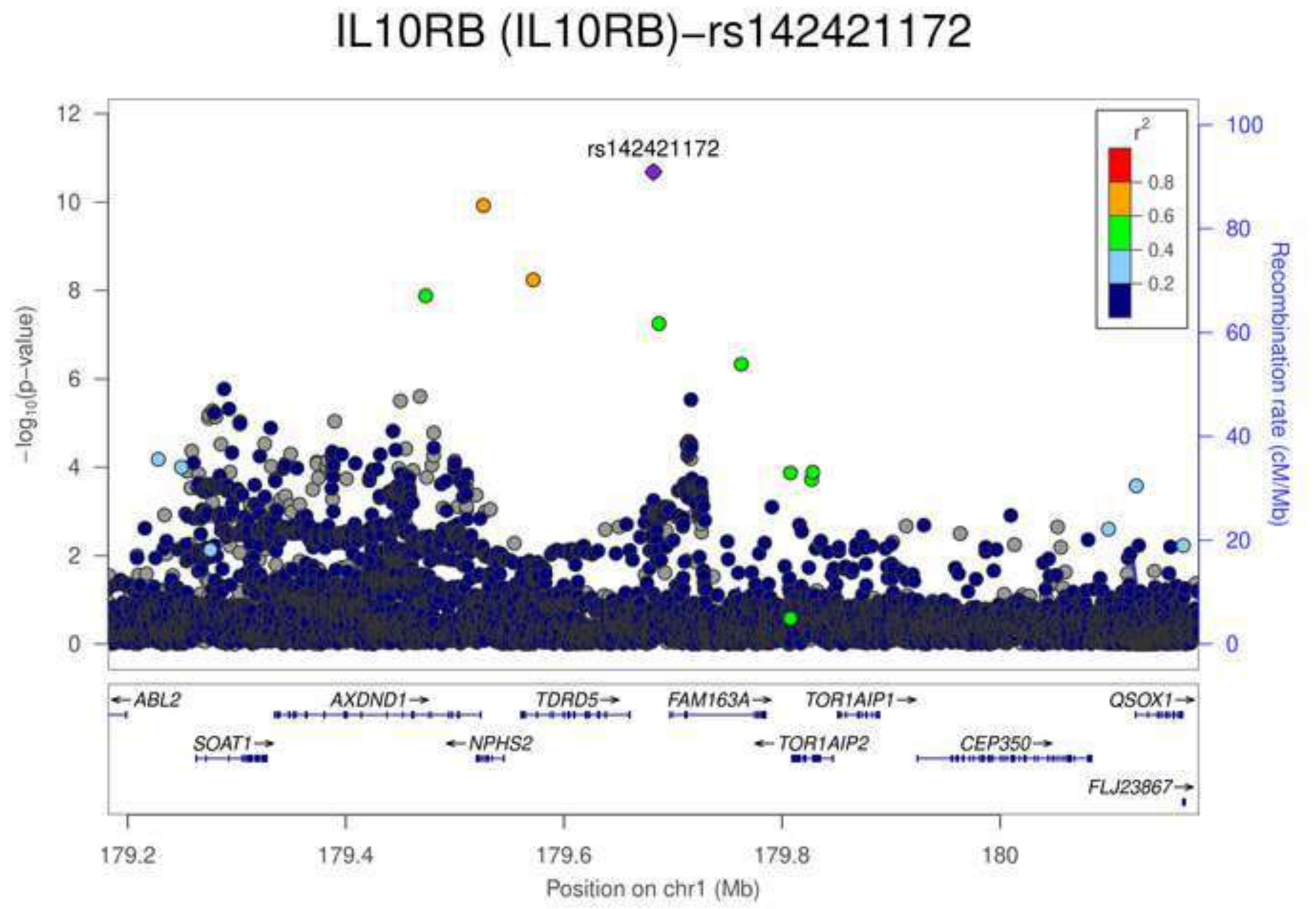
IL10RB (IL10RB)−rs142421172
rs142421172
$-\log_{10}$(p-value)
Recombination rate (cM/Mb)
r^2
0.8
0.6
0.4
0.2
ABL2
AXDND1
TDRD5
FAM163A
TOR1AIP1
QSOX1
SOAT1
NPHS2
TOR1AIP2
CEP350
FLJ23867
179.2
179.4
179.6
179.8
180
Position on chr1 (Mb)

IL10RB (IL10RB) [chr21:34659396_A_G (rs2266590) (A/G) N=14714]

Study	TE	SE(TE)	95%−CI	Weight (common)	Weight (random)
INTERVAL (4896)	−0.493	0.02	−0.49 [−0.53; −0.46]	31.7%	10.8%
BioFinder (1496)	−0.390	0.03	−0.39 [−0.46; −0.32]	9.7%	9.9%
EGCUT (487)	−0.533	0.06	−0.53 [−0.64; −0.42]	3.6%	8.2%
KORA (1064)	−0.374	0.04	−0.37 [−0.46; −0.29]	6.7%	9.4%
NSPHS (866)	−0.423	0.05	−0.42 [−0.52; −0.33]	4.7%	8.8%
ORCADES (981)	−0.451	0.04	−0.45 [−0.53; −0.37]	6.7%	9.4%
RECOMBINE (428)	−0.143	0.04	−0.14 [−0.22; −0.07]	7.8%	9.6%
STABILITY (2951)	−0.375	0.03	−0.38 [−0.42; −0.33]	18.6%	10.5%
STANLEY (344)	−0.377	0.06	−0.38 [−0.50; −0.25]	3.0%	7.8%
STANLEY (300)	−0.373	0.08	−0.37 [−0.53; −0.22]	1.9%	6.5%
VIS (901)	−0.405	0.05	−0.41 [−0.50; −0.32]	5.5%	9.1%
**Common effect model**			**−0.41 [−0.43; −0.39]**	**100.0%**	**--**
**Random effects model**			**−0.39 [−0.45; −0.33]**	**--**	**100.0%**

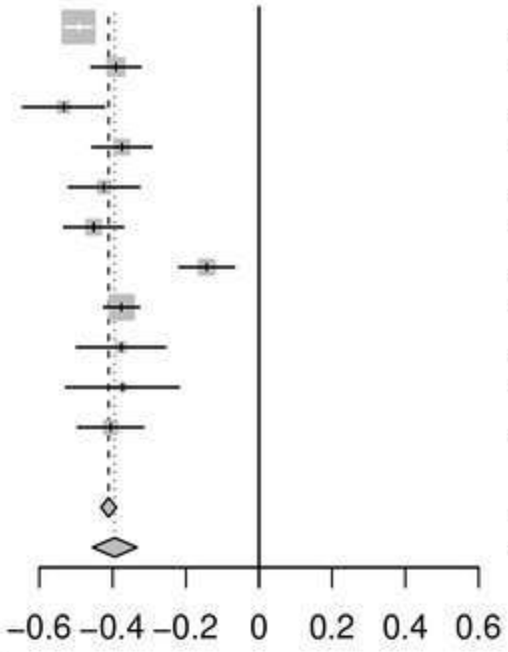

Heterogeneity: $I^2 = 87\%$, $\tau^2 = 0.0086$, $p < 0.01$

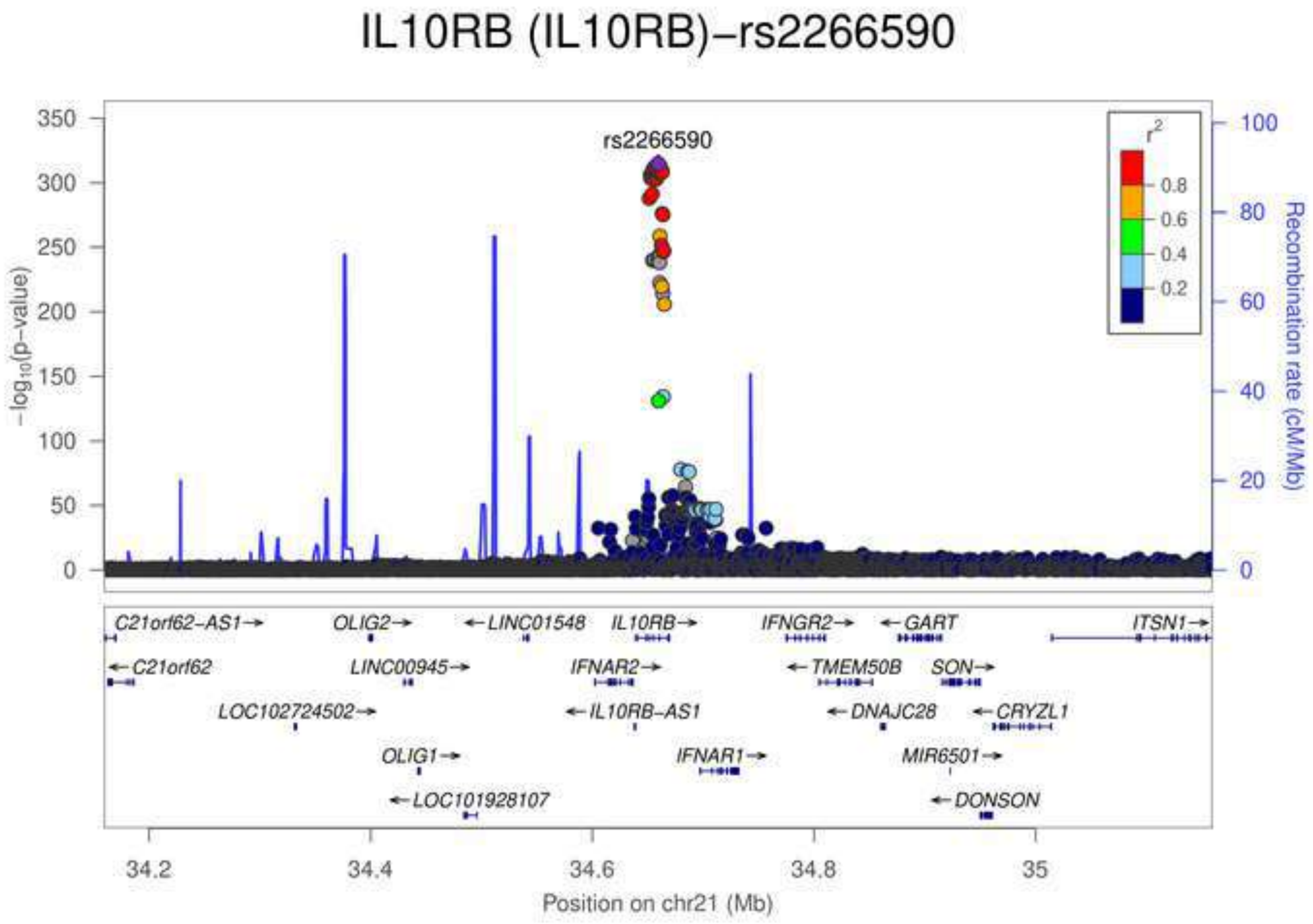

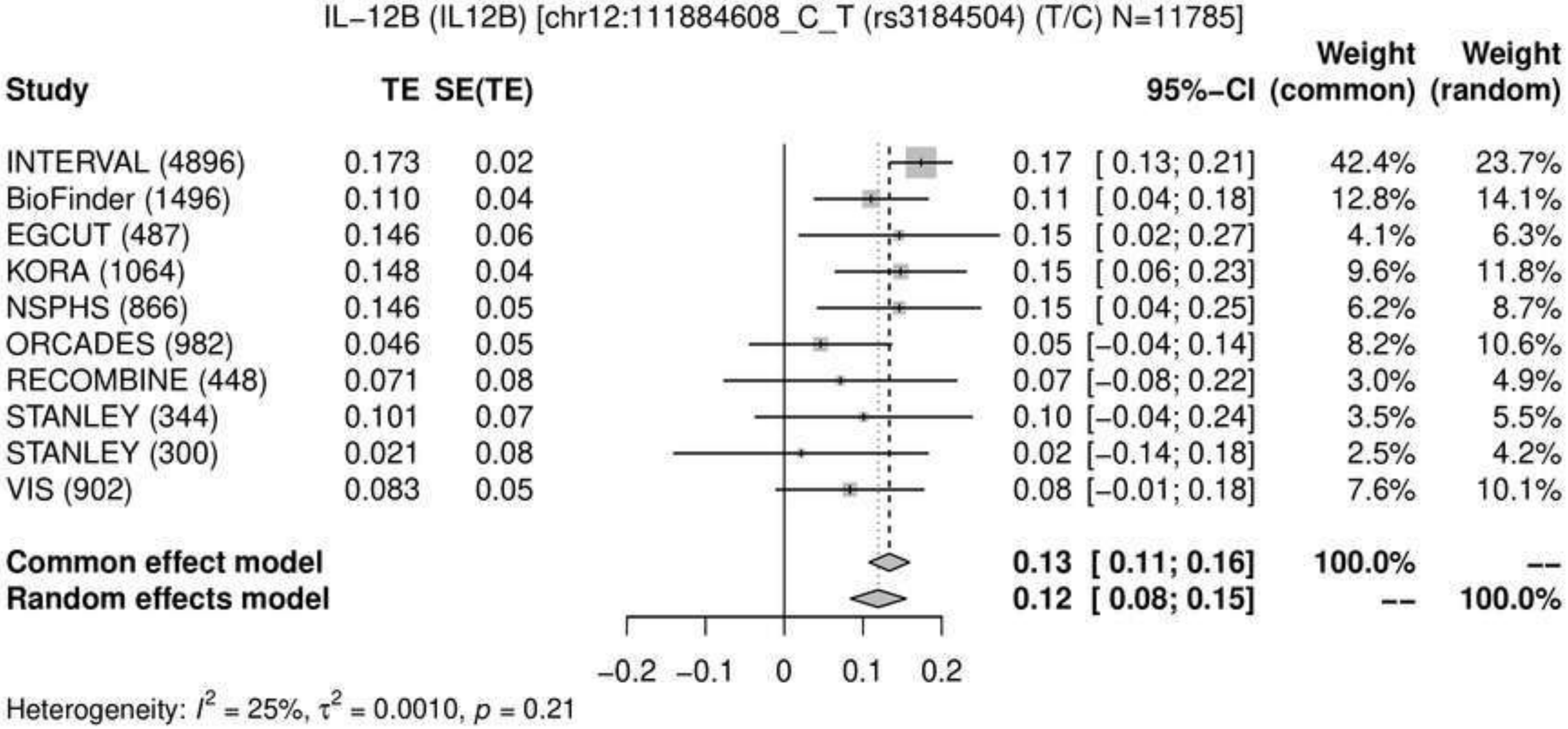
IL−12B (IL12B) [chr12:111884608_C_T (rs3184504) (T/C) N=11785]
Study TE SE(TE) 95%−CI Weight (common) Weight (random)
INTERVAL (4896) 0.173 0.02 0.17 [0.13; 0.21] 42.4% 23.7%
BioFinder (1496) 0.110 0.04 0.11 [0.04; 0.18] 12.8% 14.1%
EGCUT (487) 0.146 0.06 0.15 [0.02; 0.27] 4.1% 6.3%
KORA (1064) 0.148 0.04 0.15 [0.06; 0.23] 9.6% 11.8%
NSPHS (866) 0.146 0.05 0.15 [0.04; 0.25] 6.2% 8.7%
ORCADES (982) 0.046 0.05 0.05 [−0.04; 0.14] 8.2% 10.6%
RECOMBINE (448) 0.071 0.08 0.07 [−0.08; 0.22] 3.0% 4.9%
STANLEY (344) 0.101 0.07 0.10 [−0.04; 0.24] 3.5% 5.5%
STANLEY (300) 0.021 0.08 0.02 [−0.14; 0.18] 2.5% 4.2%
VIS (902) 0.083 0.05 0.08 [−0.01; 0.18] 7.6% 10.1%
Common effect model 0.13 [0.11; 0.16] 100.0% --
Random effects model 0.12 [0.08; 0.15] -- 100.0%
−0.2 −0.1 0 0.1 0.2
Heterogeneity: I^2 = 25%, τ^2 = 0.0010, p = 0.21

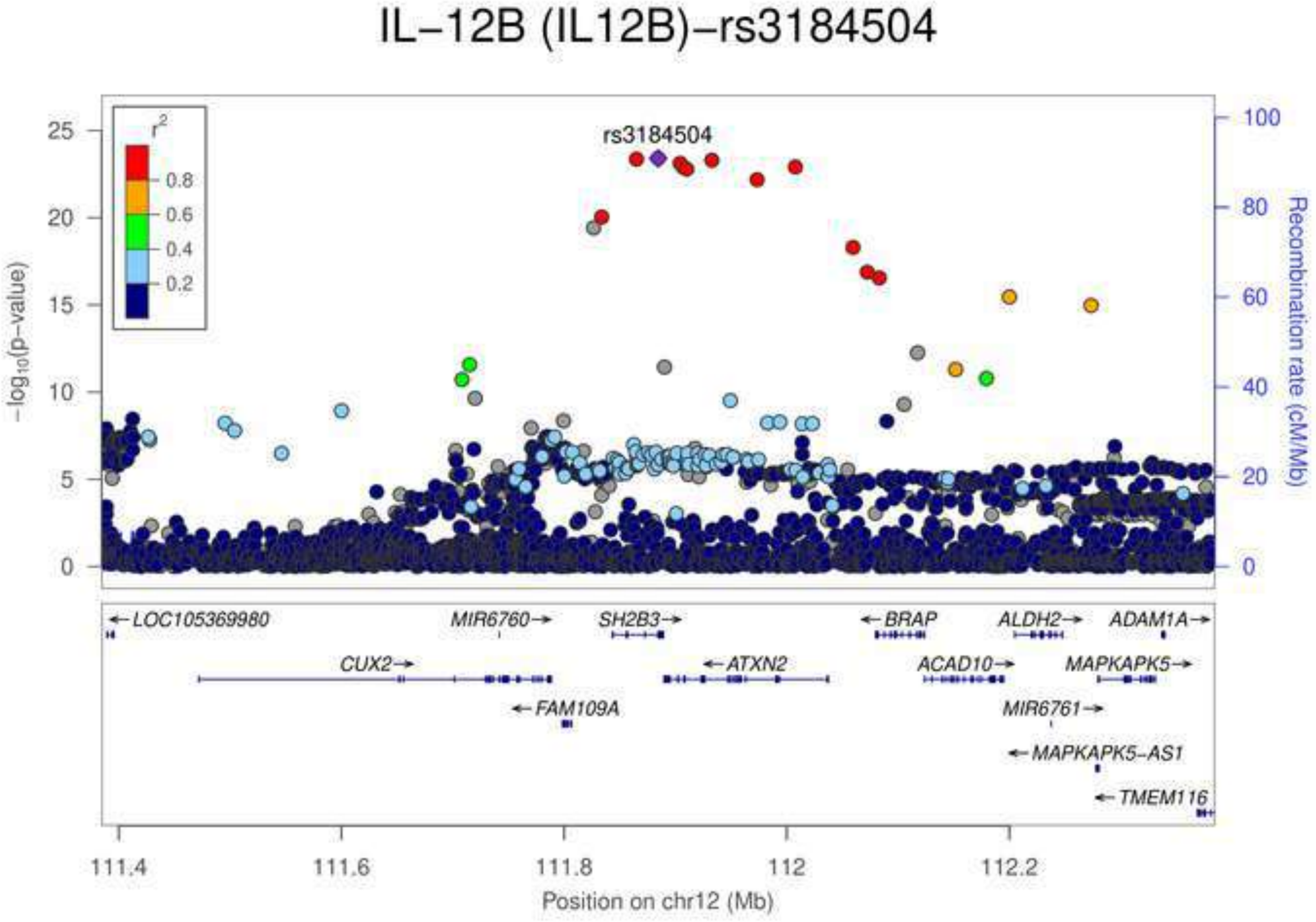
IL−12B (IL12B)−rs3184504
rs3184504
r^2
0.8
0.6
0.4
0.2
$-\log_{10}$(p−value)
Recombination rate (cM/Mb)
LOC105369980
MIR6760
SH2B3
BRAP
ALDH2
ADAM1A
CUX2
ATXN2
ACAD10
MAPKAPK5
FAM109A
MIR6761
MAPKAPK5−AS1
TMEM116
111.4
111.6
111.8
112
112.2
Position on chr12 (Mb)

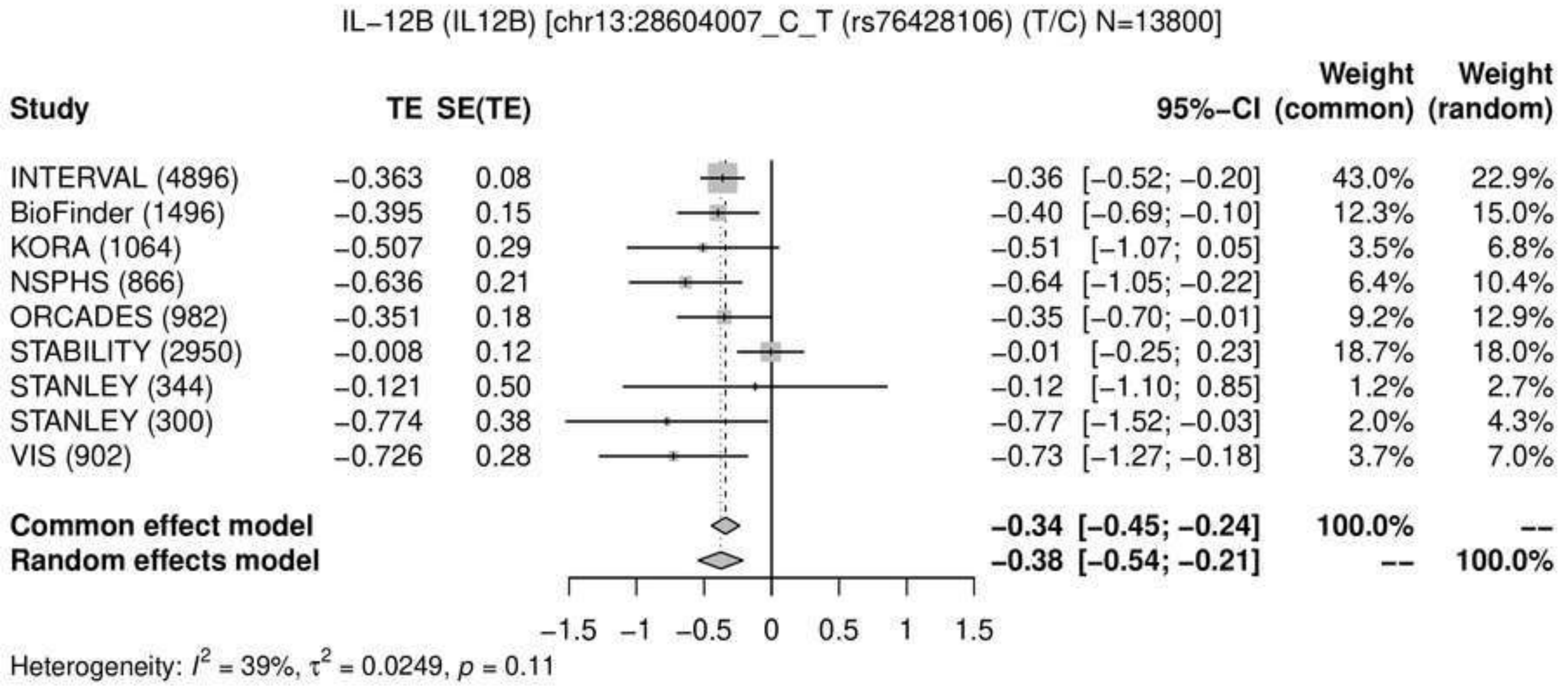
IL−12B (IL12B) [chr13:28604007_C_T (rs76428106) (T/C) N=13800]
Study
TE
SE(TE)
95%−CI
Weight (common)
Weight (random)
INTERVAL (4896) −0.363 0.08 −0.36 [−0.52; −0.20] 43.0% 22.9%
BioFinder (1496) −0.395 0.15 −0.40 [−0.69; −0.10] 12.3% 15.0%
KORA (1064) −0.507 0.29 −0.51 [−1.07; 0.05] 3.5% 6.8%
NSPHS (866) −0.636 0.21 −0.64 [−1.05; −0.22] 6.4% 10.4%
ORCADES (982) −0.351 0.18 −0.35 [−0.70; −0.01] 9.2% 12.9%
STABILITY (2950) −0.008 0.12 −0.01 [−0.25; 0.23] 18.7% 18.0%
STANLEY (344) −0.121 0.50 −0.12 [−1.10; 0.85] 1.2% 2.7%
STANLEY (300) −0.774 0.38 −0.77 [−1.52; −0.03] 2.0% 4.3%
VIS (902) −0.726 0.28 −0.73 [−1.27; −0.18] 3.7% 7.0%
Common effect model −0.34 [−0.45; −0.24] 100.0% --
Random effects model −0.38 [−0.54; −0.21] -- 100.0%
−1.5 −1 −0.5 0 0.5 1 1.5
Heterogeneity: I^2 = 39%, τ^2 = 0.0249, p = 0.11

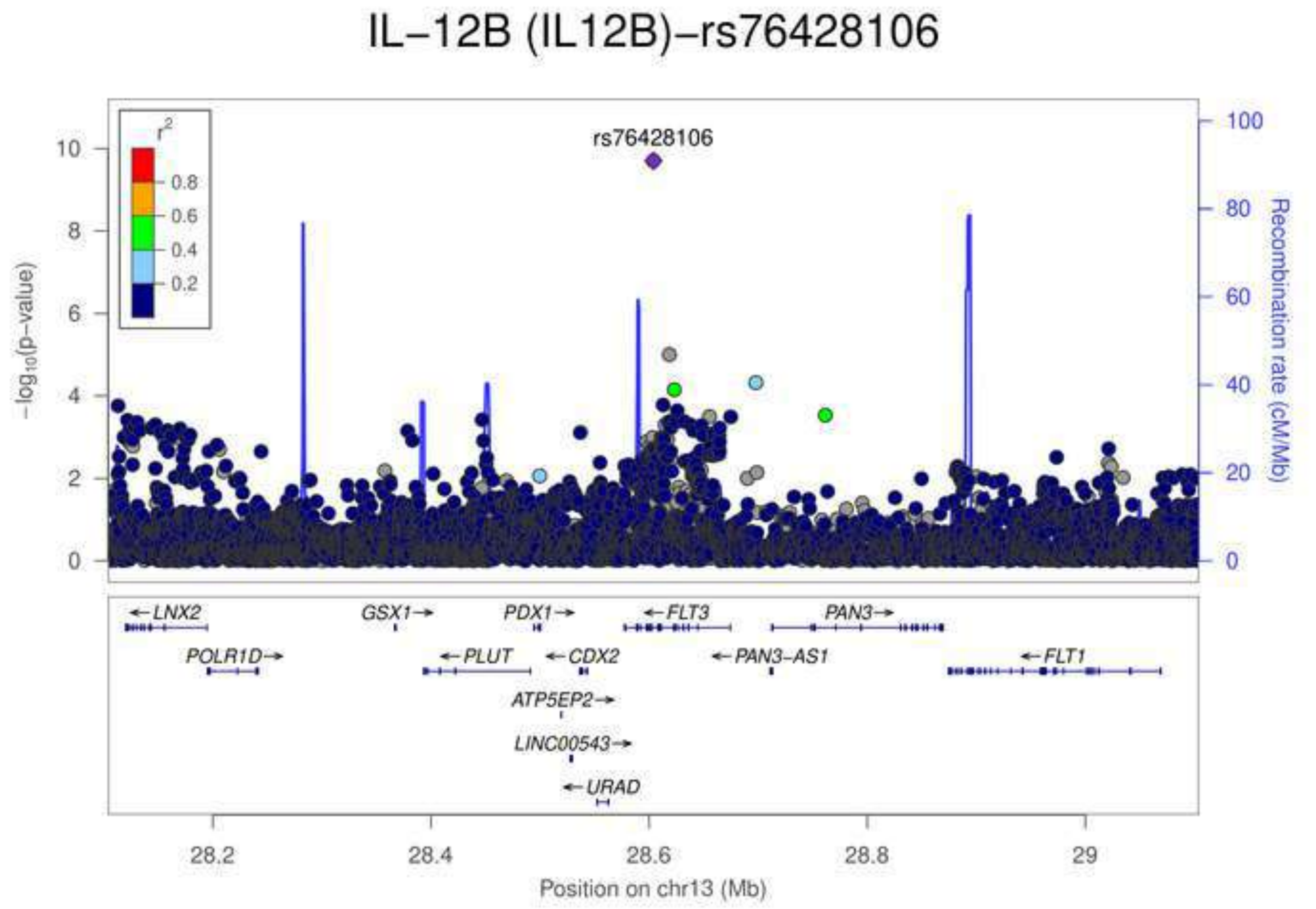
IL−12B (IL12B)−rs76428106
rs76428106
r^2
0.8
0.6
0.4
0.2
$-\log_{10}$(p-value)
Recombination rate (cM/Mb)
0
2
4
6
8
10
20
40
60
80
100
LNX2
GSX1
PDX1
FLT3
PAN3
POLR1D
PLUT
CDX2
PAN3-AS1
FLT1
ATP5EP2
LINC00543
URAD
28.2
28.4
28.6
28.8
29
Position on chr13 (Mb)

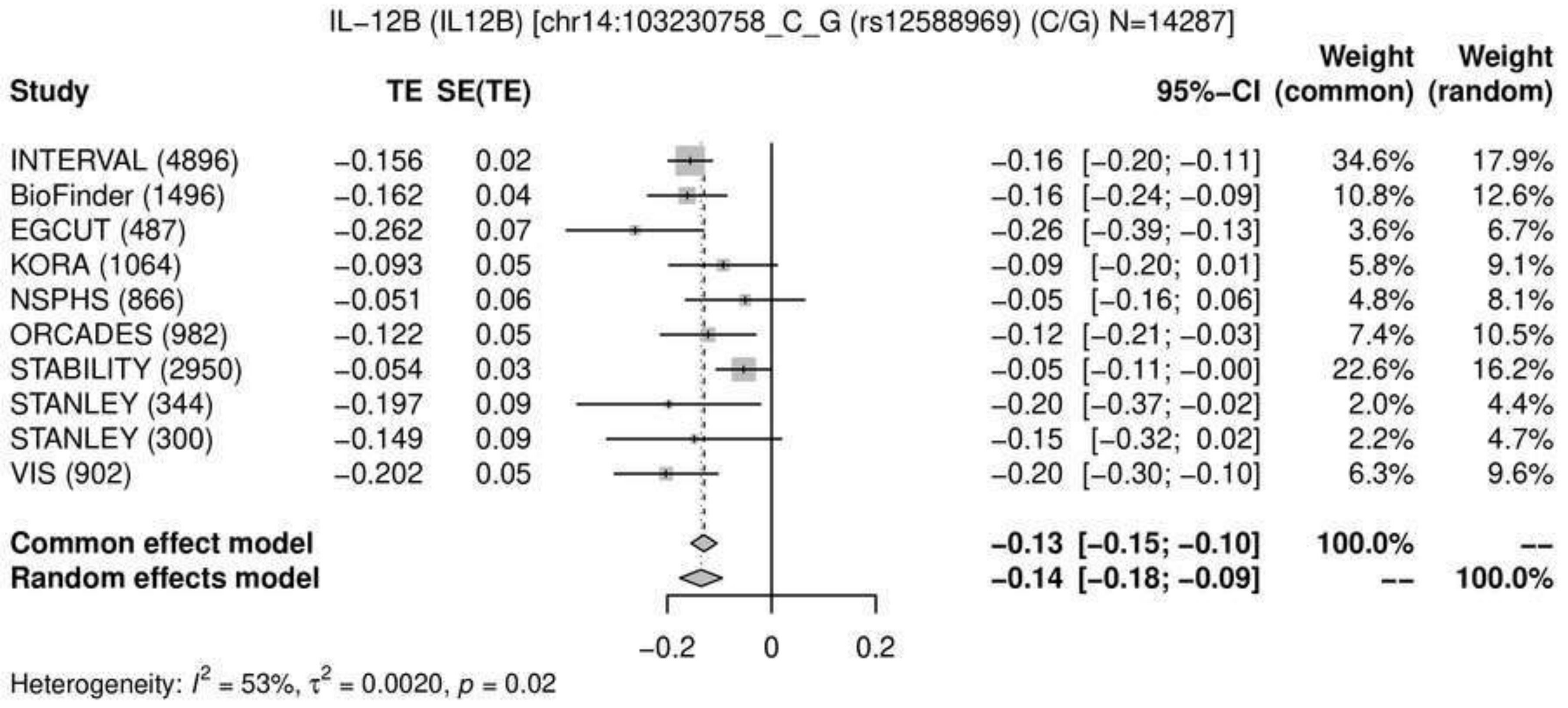
IL−12B (IL12B) [chr14:103230758_C_G (rs12588969) (C/G) N=14287]
Study
TE
SE(TE)
95%−CI
Weight (common)
Weight (random)
INTERVAL (4896) −0.156 0.02 −0.16 [−0.20; −0.11] 34.6% 17.9%
BioFinder (1496) −0.162 0.04 −0.16 [−0.24; −0.09] 10.8% 12.6%
EGCUT (487) −0.262 0.07 −0.26 [−0.39; −0.13] 3.6% 6.7%
KORA (1064) −0.093 0.05 −0.09 [−0.20; 0.01] 5.8% 9.1%
NSPHS (866) −0.051 0.06 −0.05 [−0.16; 0.06] 4.8% 8.1%
ORCADES (982) −0.122 0.05 −0.12 [−0.21; −0.03] 7.4% 10.5%
STABILITY (2950) −0.054 0.03 −0.05 [−0.11; −0.00] 22.6% 16.2%
STANLEY (344) −0.197 0.09 −0.20 [−0.37; −0.02] 2.0% 4.4%
STANLEY (300) −0.149 0.09 −0.15 [−0.32; 0.02] 2.2% 4.7%
VIS (902) −0.202 0.05 −0.20 [−0.30; −0.10] 6.3% 9.6%
Common effect model −0.13 [−0.15; −0.10] 100.0% --
Random effects model −0.14 [−0.18; −0.09] -- 100.0%
−0.2
0
0.2
Heterogeneity: I^2 = 53%, τ^2 = 0.0020, p = 0.02

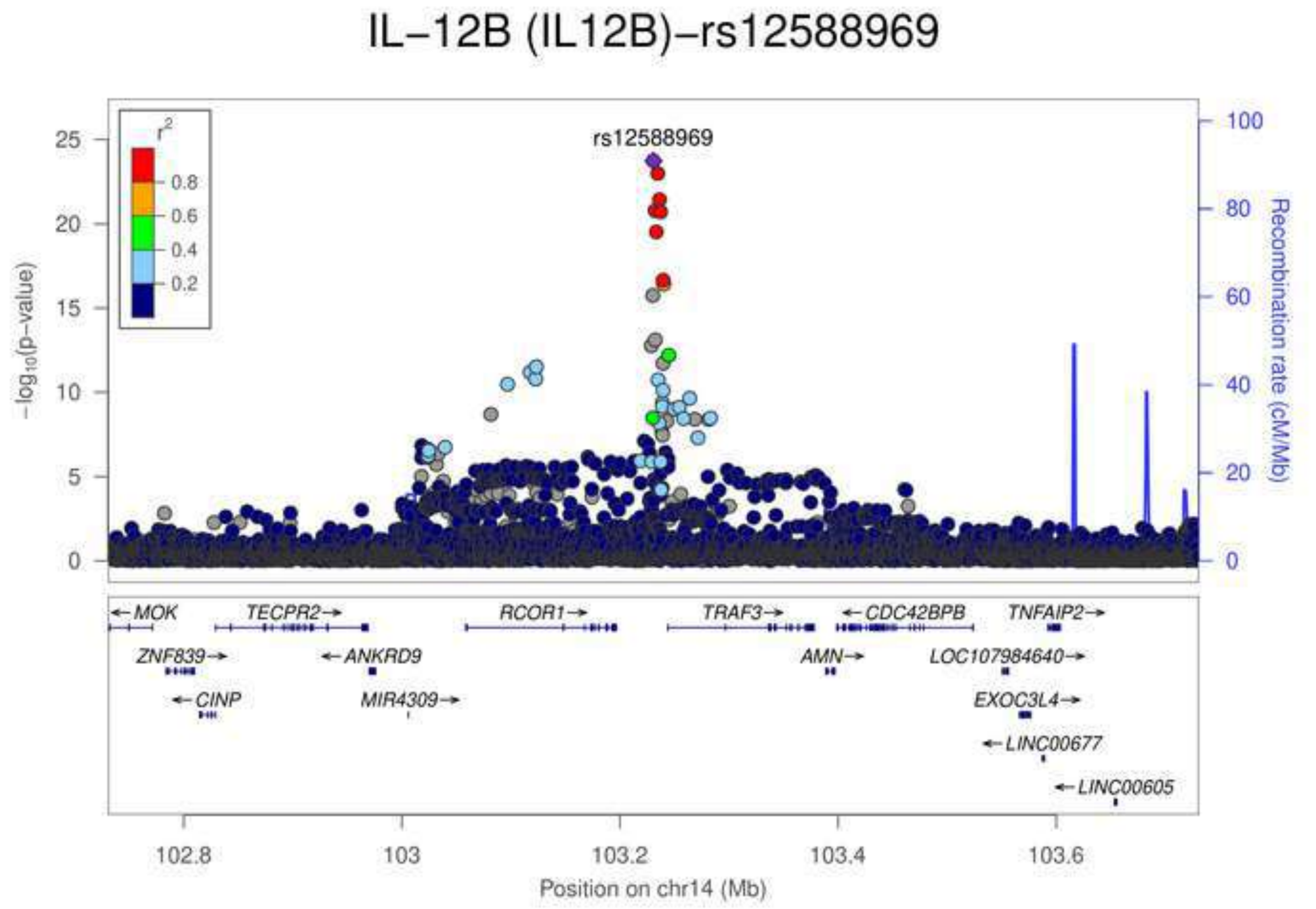
IL−12B (IL12B)−rs12588969
rs12588969
r^2
0.8
0.6
0.4
0.2
$-\log_{10}$(p-value)
Recombination rate (cM/Mb)
MOK
TECPR2
RCOR1
TRAF3
CDC42BPB
TNFAIP2
ZNF839
ANKRD9
AMN
LOC107984640
CINP
MIR4309
EXOC3L4
LINC00677
LINC00605
102.8
103
103.2
103.4
103.6
Position on chr14 (Mb)

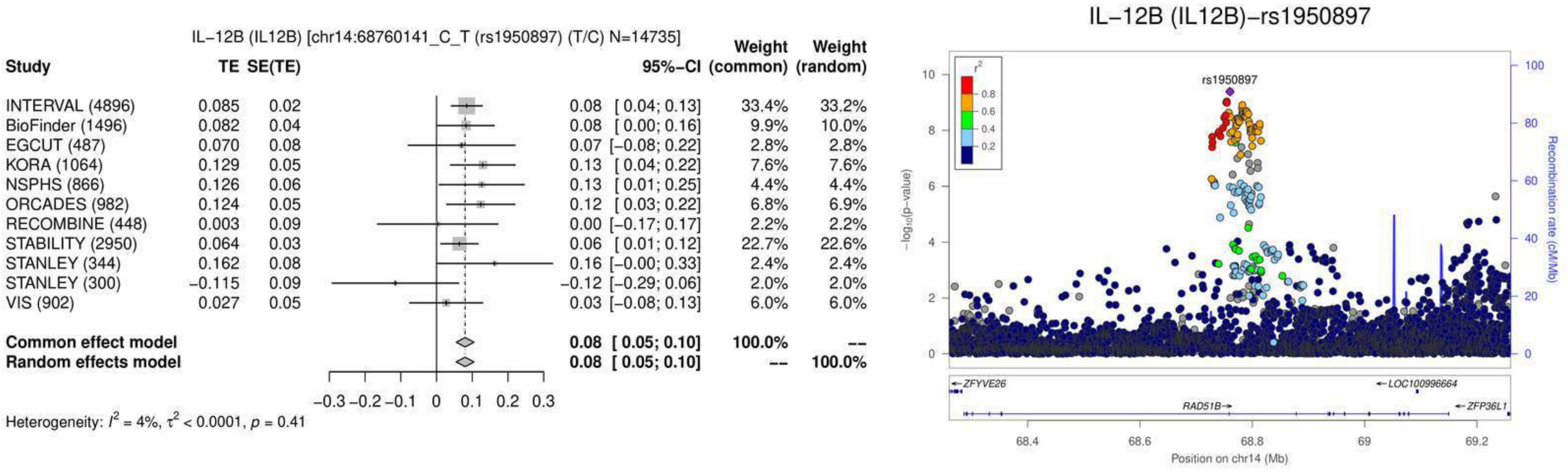
IL−12B (IL12B) [chr14:68760141_C_T (rs1950897) (T/C) N=14735]
Study
TE
SE(TE)
95%−CI
Weight (common)
Weight (random)
INTERVAL (4896) 0.085 0.02 0.08 [0.04; 0.13] 33.4% 33.2%
BioFinder (1496) 0.082 0.04 0.08 [0.00; 0.16] 9.9% 10.0%
EGCUT (487) 0.070 0.08 0.07 [−0.08; 0.22] 2.8% 2.8%
KORA (1064) 0.129 0.05 0.13 [0.04; 0.22] 7.6% 7.6%
NSPHS (866) 0.126 0.06 0.13 [0.01; 0.25] 4.4% 4.4%
ORCADES (982) 0.124 0.05 0.12 [0.03; 0.22] 6.8% 6.9%
RECOMBINE (448) 0.003 0.09 0.00 [−0.17; 0.17] 2.2% 2.2%
STABILITY (2950) 0.064 0.03 0.06 [0.01; 0.12] 22.7% 22.6%
STANLEY (344) 0.162 0.08 0.16 [−0.00; 0.33] 2.4% 2.4%
STANLEY (300) −0.115 0.09 −0.12 [−0.29; 0.06] 2.0% 2.0%
VIS (902) 0.027 0.05 0.03 [−0.08; 0.13] 6.0% 6.0%
Common effect model 0.08 [0.05; 0.10] 100.0% --
Random effects model 0.08 [0.05; 0.10] -- 100.0%
−0.3 −0.2 −0.1 0 0.1 0.2 0.3
Heterogeneity: I^2 = 4%, τ^2 < 0.0001, p = 0.41
IL−12B (IL12B)−rs1950897
rs1950897
r^2
0.8
0.6
0.4
0.2
$-\log_{10}$(p−value)
Recombination rate (cM/Mb)
ZFYVE26
LOC100996664
RAD51B
ZFP36L1
68.4
68.6
68.8
69
69.2
Position on chr14 (Mb)

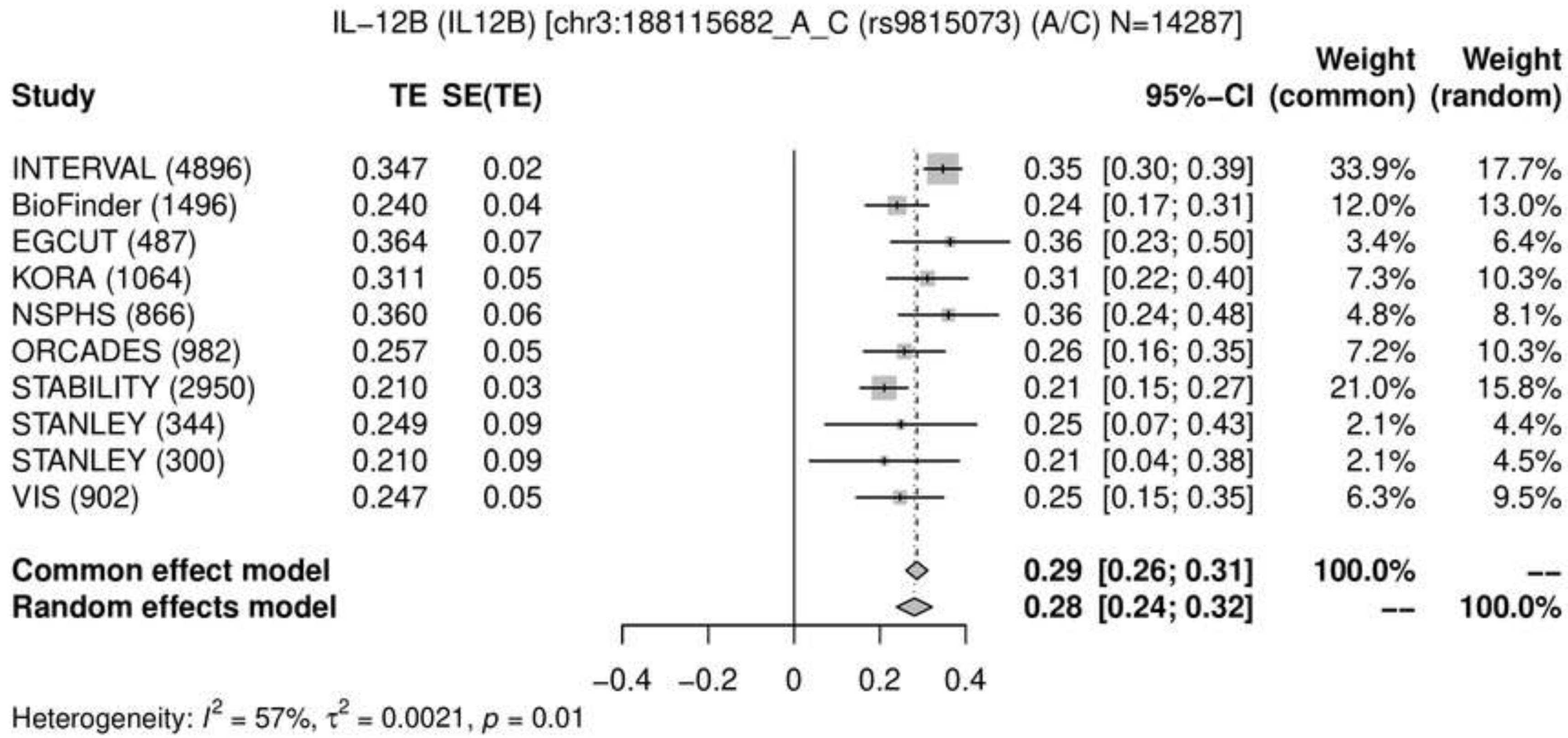
IL−12B (IL12B) [chr3:188115682_A_C (rs9815073) (A/C) N=14287]
Study
TE
SE(TE)
95%−CI
Weight (common)
Weight (random)
INTERVAL (4896) 0.347 0.02 0.35 [0.30; 0.39] 33.9% 17.7%
BioFinder (1496) 0.240 0.04 0.24 [0.17; 0.31] 12.0% 13.0%
EGCUT (487) 0.364 0.07 0.36 [0.23; 0.50] 3.4% 6.4%
KORA (1064) 0.311 0.05 0.31 [0.22; 0.40] 7.3% 10.3%
NSPHS (866) 0.360 0.06 0.36 [0.24; 0.48] 4.8% 8.1%
ORCADES (982) 0.257 0.05 0.26 [0.16; 0.35] 7.2% 10.3%
STABILITY (2950) 0.210 0.03 0.21 [0.15; 0.27] 21.0% 15.8%
STANLEY (344) 0.249 0.09 0.25 [0.07; 0.43] 2.1% 4.4%
STANLEY (300) 0.210 0.09 0.21 [0.04; 0.38] 2.1% 4.5%
VIS (902) 0.247 0.05 0.25 [0.15; 0.35] 6.3% 9.5%
Common effect model 0.29 [0.26; 0.31] 100.0% --
Random effects model 0.28 [0.24; 0.32] -- 100.0%
−0.4 −0.2 0 0.2 0.4
Heterogeneity: I^2 = 57%, τ^2 = 0.0021, p = 0.01

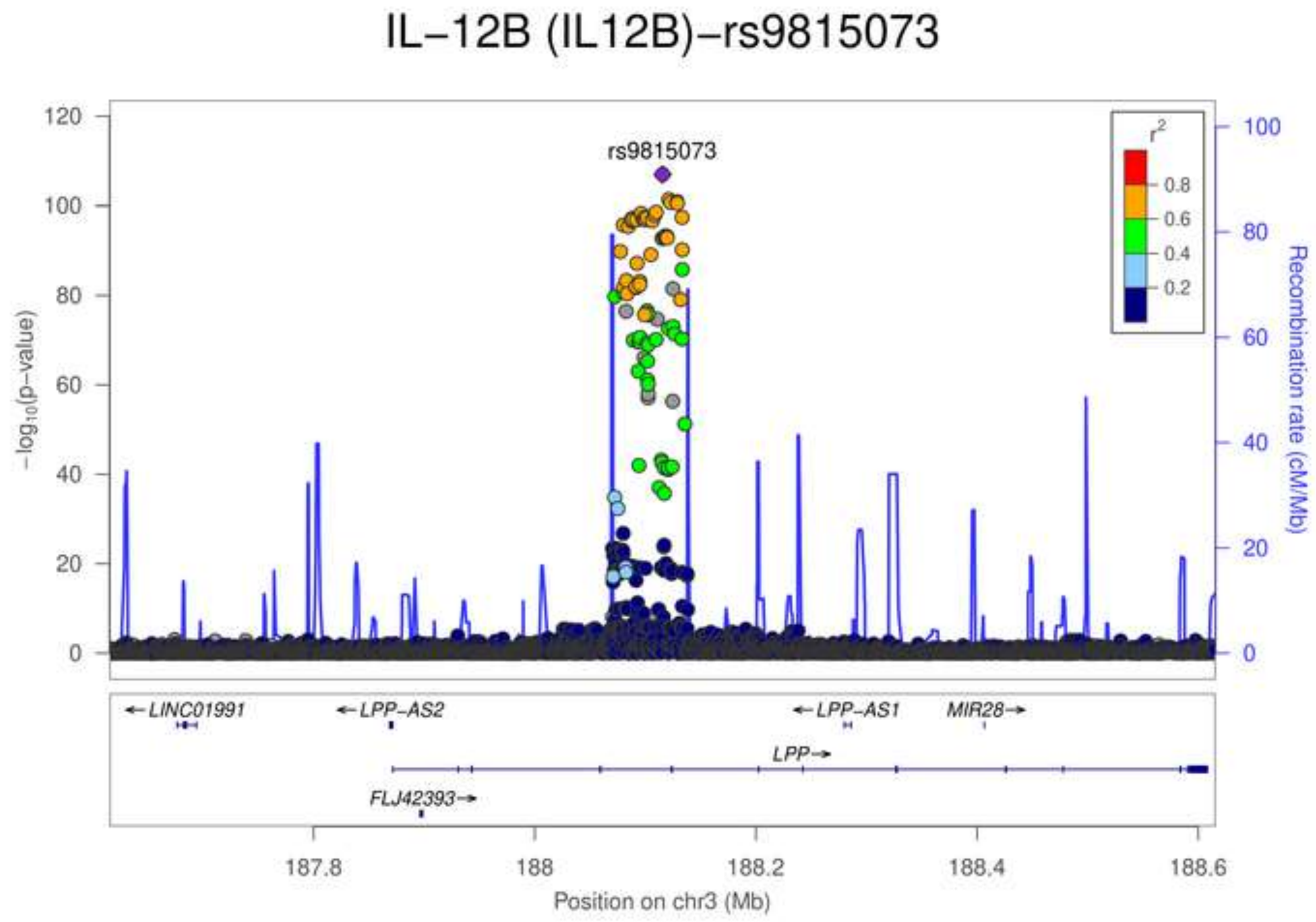
IL−12B (IL12B)−rs9815073
rs9815073
$-\log_{10}$(p−value)
Recombination rate (cM/Mb)
r^2
0.8
0.6
0.4
0.2
0
20
40
60
80
100
120
LINC01991
LPP−AS2
LPP−AS1
MIR28
LPP
FLJ42393
187.8
188
188.2
188.4
188.6
Position on chr3 (Mb)

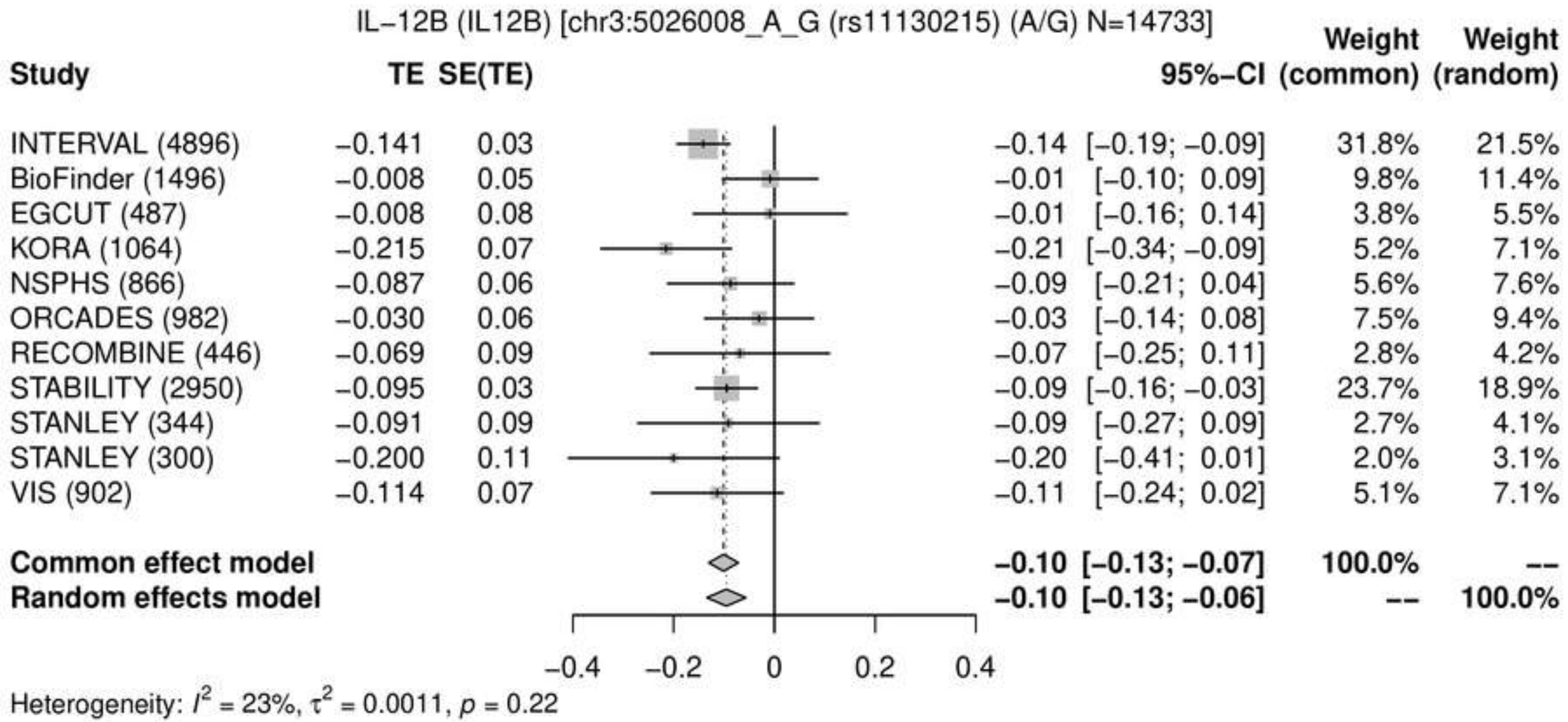
IL−12B (IL12B) [chr3:5026008_A_G (rs11130215) (A/G) N=14733]
Study
TE
SE(TE)
95%−CI
Weight (common)
Weight (random)
INTERVAL (4896) −0.141 0.03 −0.14 [−0.19; −0.09] 31.8% 21.5%
BioFinder (1496) −0.008 0.05 −0.01 [−0.10; 0.09] 9.8% 11.4%
EGCUT (487) −0.008 0.08 −0.01 [−0.16; 0.14] 3.8% 5.5%
KORA (1064) −0.215 0.07 −0.21 [−0.34; −0.09] 5.2% 7.1%
NSPHS (866) −0.087 0.06 −0.09 [−0.21; 0.04] 5.6% 7.6%
ORCADES (982) −0.030 0.06 −0.03 [−0.14; 0.08] 7.5% 9.4%
RECOMBINE (446) −0.069 0.09 −0.07 [−0.25; 0.11] 2.8% 4.2%
STABILITY (2950) −0.095 0.03 −0.09 [−0.16; −0.03] 23.7% 18.9%
STANLEY (344) −0.091 0.09 −0.09 [−0.27; 0.09] 2.7% 4.1%
STANLEY (300) −0.200 0.11 −0.20 [−0.41; 0.01] 2.0% 3.1%
VIS (902) −0.114 0.07 −0.11 [−0.24; 0.02] 5.1% 7.1%
Common effect model −0.10 [−0.13; −0.07] 100.0% --
Random effects model −0.10 [−0.13; −0.06] -- 100.0%
−0.4 −0.2 0 0.2 0.4
Heterogeneity: I² = 23%, τ² = 0.0011, p = 0.22

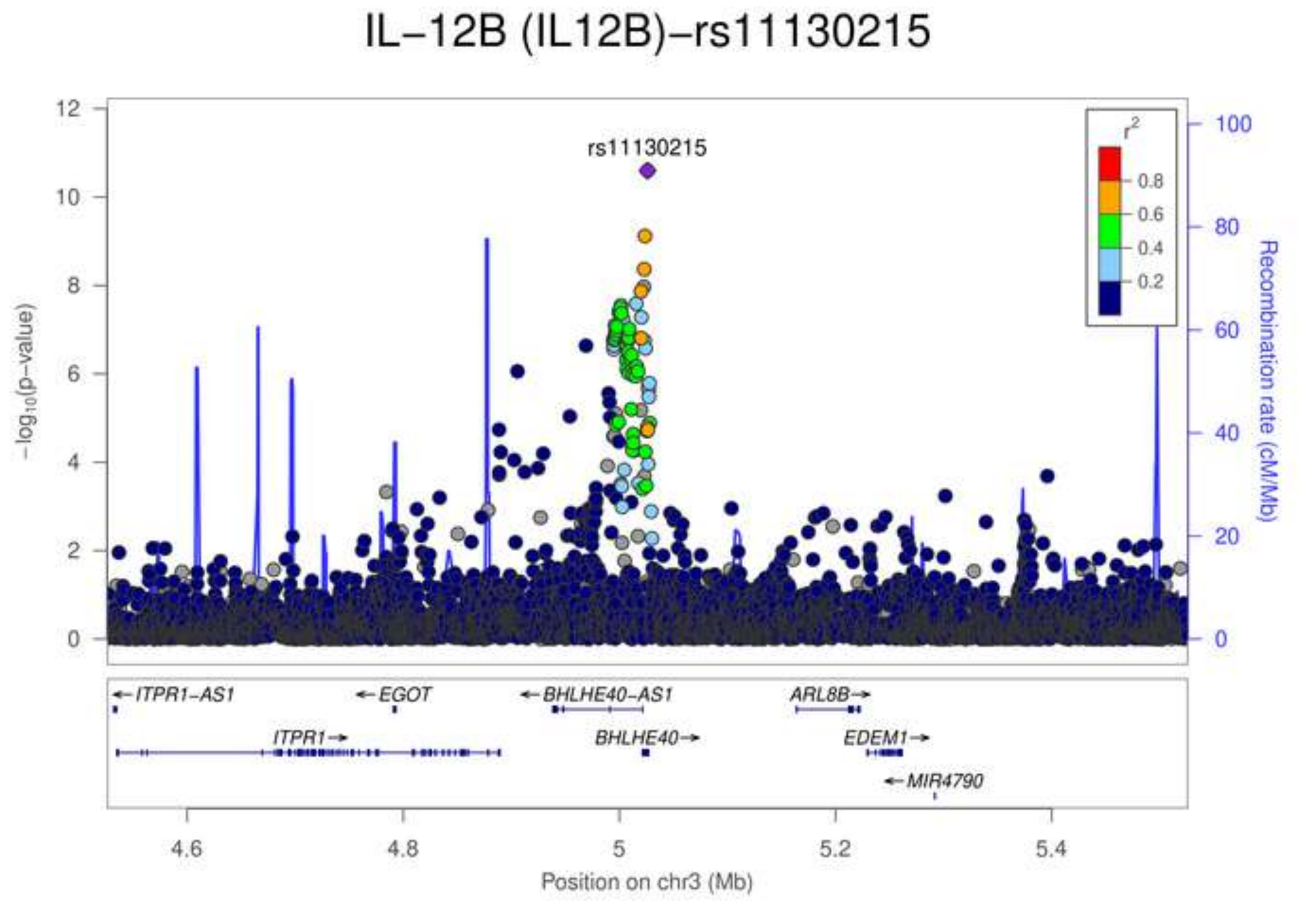
IL−12B (IL12B)−rs11130215
rs11130215
r²
0.8
0.6
0.4
0.2
−log10(p−value)
Recombination rate (cM/Mb)
ITPR1−AS1
EGOT
BHLHE40−AS1
ARL8B
ITPR1
BHLHE40
EDEM1
MIR4790
4.6 4.8 5 5.2 5.4
Position on chr3 (Mb)

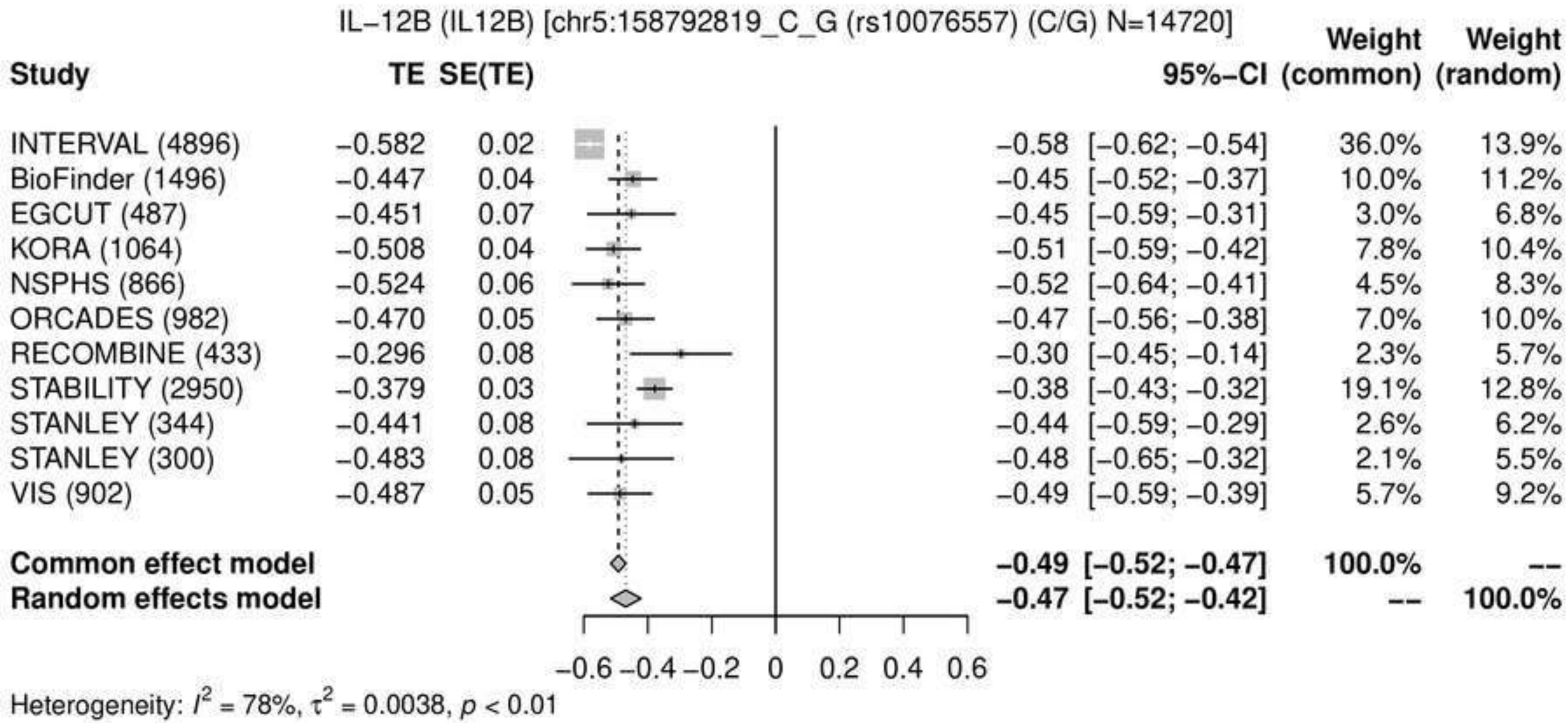
IL−12B (IL12B) [chr5:158792819_C_G (rs10076557) (C/G) N=14720]
Study TE SE(TE) 95%−CI Weight (common) Weight (random)
INTERVAL (4896) −0.582 0.02 −0.58 [−0.62; −0.54] 36.0% 13.9%
BioFinder (1496) −0.447 0.04 −0.45 [−0.52; −0.37] 10.0% 11.2%
EGCUT (487) −0.451 0.07 −0.45 [−0.59; −0.31] 3.0% 6.8%
KORA (1064) −0.508 0.04 −0.51 [−0.59; −0.42] 7.8% 10.4%
NSPHS (866) −0.524 0.06 −0.52 [−0.64; −0.41] 4.5% 8.3%
ORCADES (982) −0.470 0.05 −0.47 [−0.56; −0.38] 7.0% 10.0%
RECOMBINE (433) −0.296 0.08 −0.30 [−0.45; −0.14] 2.3% 5.7%
STABILITY (2950) −0.379 0.03 −0.38 [−0.43; −0.32] 19.1% 12.8%
STANLEY (344) −0.441 0.08 −0.44 [−0.59; −0.29] 2.6% 6.2%
STANLEY (300) −0.483 0.08 −0.48 [−0.65; −0.32] 2.1% 5.5%
VIS (902) −0.487 0.05 −0.49 [−0.59; −0.39] 5.7% 9.2%
Common effect model −0.49 [−0.52; −0.47] 100.0% --
Random effects model −0.47 [−0.52; −0.42] -- 100.0%
−0.6 −0.4 −0.2 0 0.2 0.4 0.6
Heterogeneity: I² = 78%, τ² = 0.0038, p < 0.01

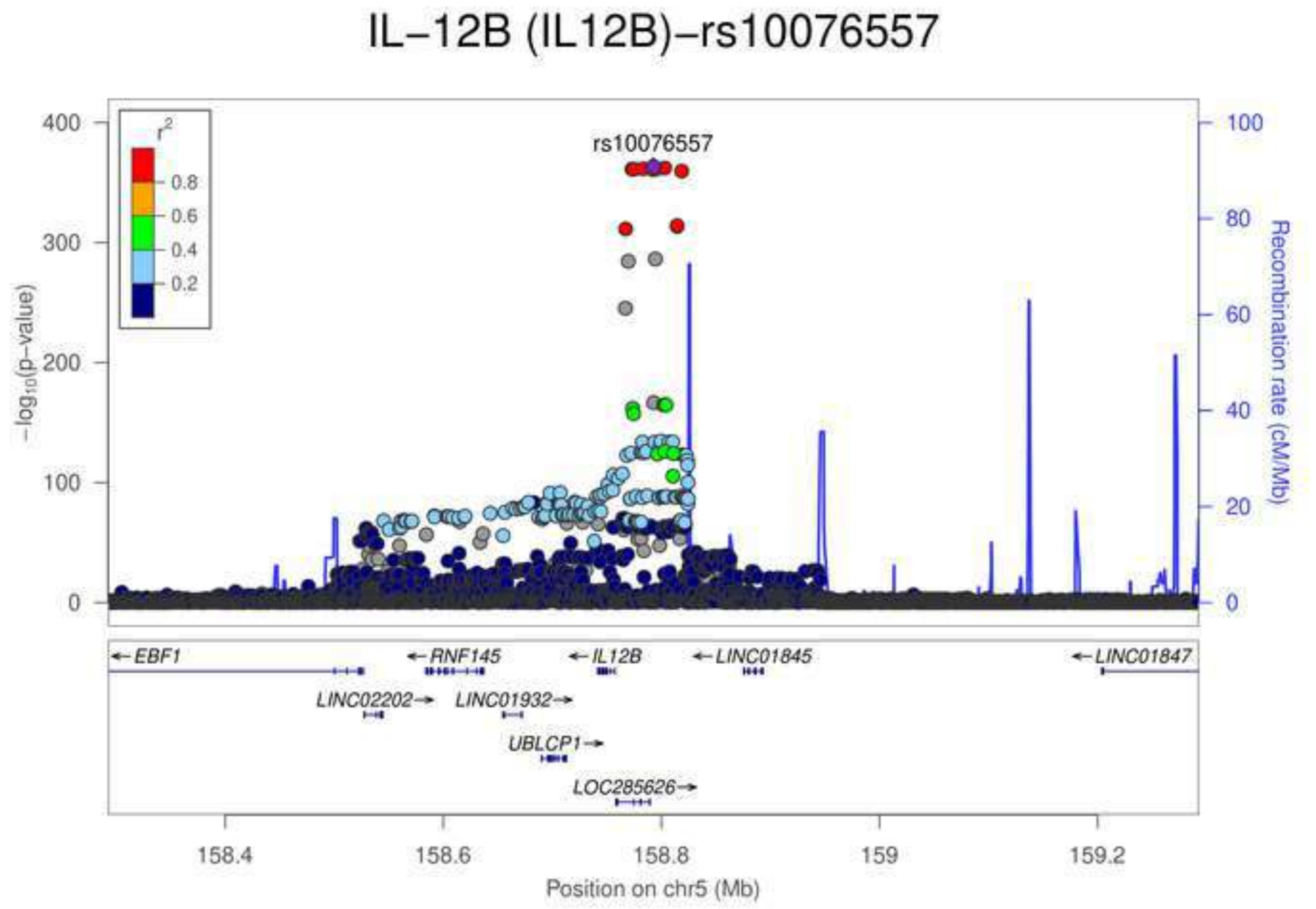
IL−12B (IL12B)−rs10076557
rs10076557
r²
0.8
0.6
0.4
0.2
−log10(p−value)
Recombination rate (cM/Mb)
EBF1
RNF145
IL12B
LINC01845
LINC01847
LINC02202
LINC01932
UBLCP1
LOC285626
158.4
158.6
158.8
159
159.2
Position on chr5 (Mb)

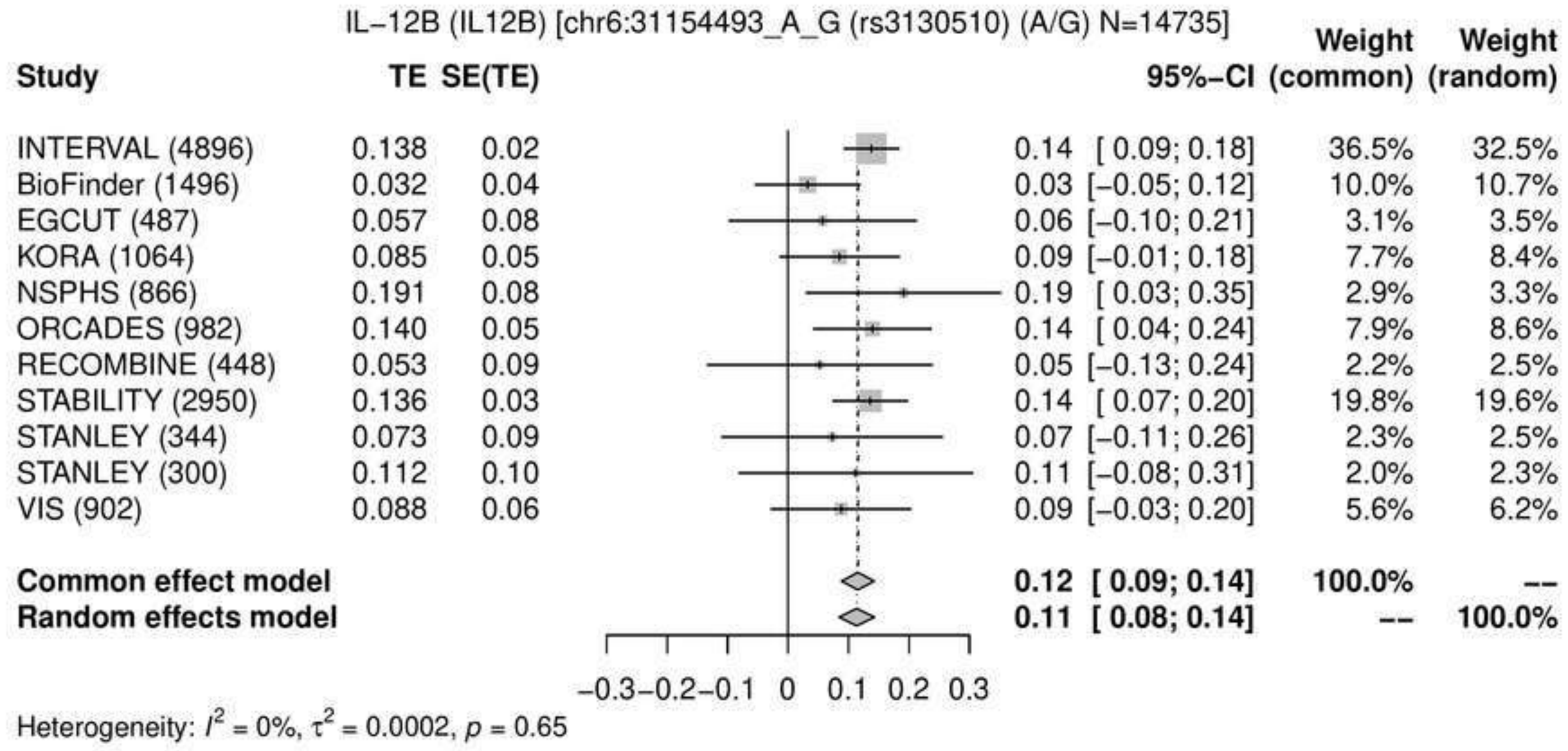
IL−12B (IL12B) [chr6:31154493_A_G (rs3130510) (A/G) N=14735]
Study
TE
SE(TE)
95%−CI
Weight (common)
Weight (random)
INTERVAL (4896) 0.138 0.02 0.14 [0.09; 0.18] 36.5% 32.5%
BioFinder (1496) 0.032 0.04 0.03 [−0.05; 0.12] 10.0% 10.7%
EGCUT (487) 0.057 0.08 0.06 [−0.10; 0.21] 3.1% 3.5%
KORA (1064) 0.085 0.05 0.09 [−0.01; 0.18] 7.7% 8.4%
NSPHS (866) 0.191 0.08 0.19 [0.03; 0.35] 2.9% 3.3%
ORCADES (982) 0.140 0.05 0.14 [0.04; 0.24] 7.9% 8.6%
RECOMBINE (448) 0.053 0.09 0.05 [−0.13; 0.24] 2.2% 2.5%
STABILITY (2950) 0.136 0.03 0.14 [0.07; 0.20] 19.8% 19.6%
STANLEY (344) 0.073 0.09 0.07 [−0.11; 0.26] 2.3% 2.5%
STANLEY (300) 0.112 0.10 0.11 [−0.08; 0.31] 2.0% 2.3%
VIS (902) 0.088 0.06 0.09 [−0.03; 0.20] 5.6% 6.2%
Common effect model 0.12 [0.09; 0.14] 100.0% --
Random effects model 0.11 [0.08; 0.14] -- 100.0%
−0.3 −0.2 −0.1 0 0.1 0.2 0.3
Heterogeneity: I^2 = 0%, τ^2 = 0.0002, p = 0.65

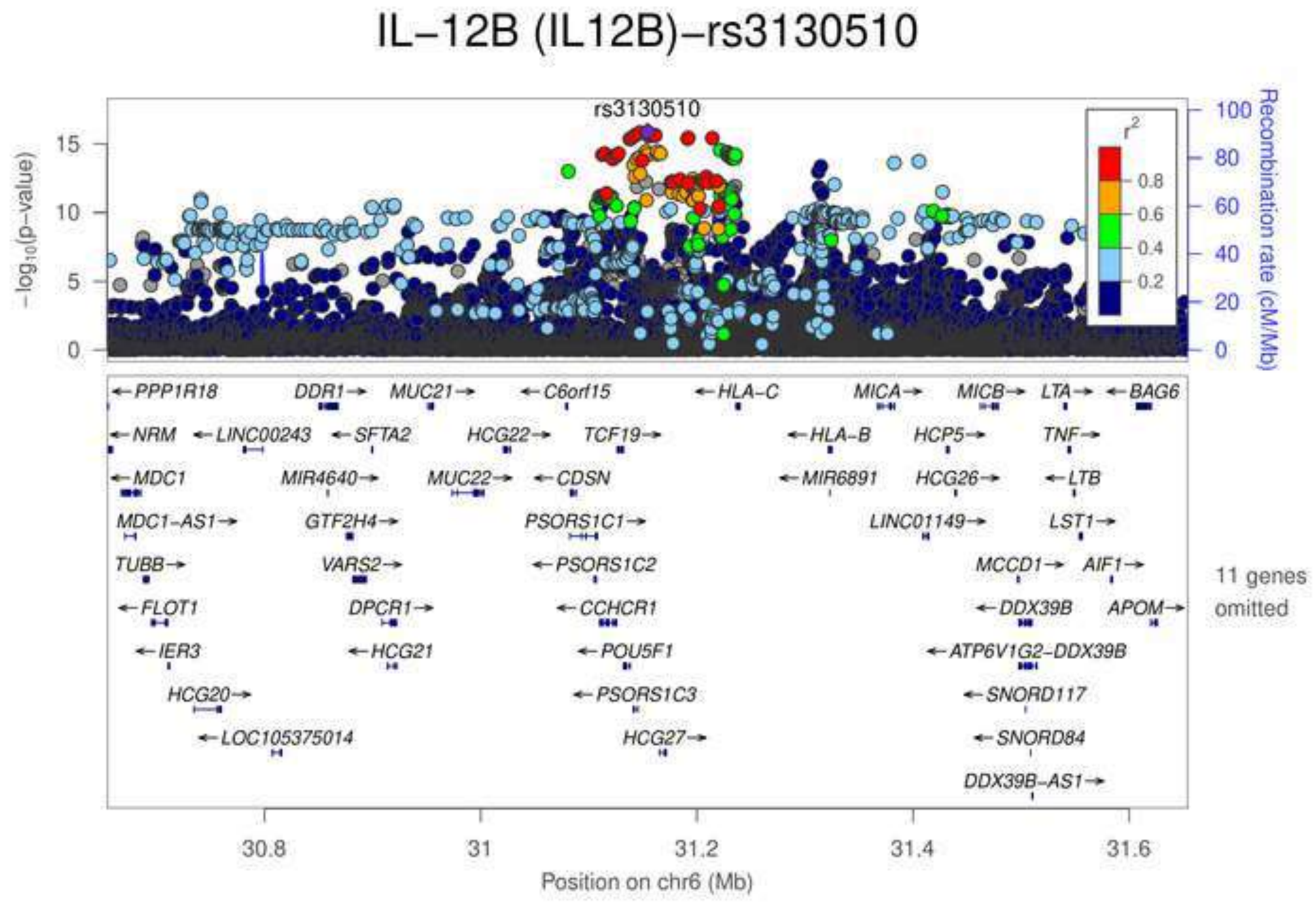
IL−12B (IL12B)−rs3130510
rs3130510
$-\log_{10}$(p−value)
Recombination rate (cM/Mb)
r^2
0.8
0.6
0.4
0.2
PPP1R18
DDR1
MUC21
C6orf15
HLA−C
MICA
MICB
LTA
BAG6
NRM
LINC00243
SFTA2
HCG22
TCF19
HLA−B
HCP5
TNF
MDC1
MIR4640
MUC22
CDSN
MIR6891
HCG26
LTB
MDC1−AS1
GTF2H4
PSORS1C1
LINC01149
LST1
TUBB
VARS2
PSORS1C2
MCCD1
AIF1
FLOT1
DPCR1
CCHCR1
DDX39B
APOM
IER3
HCG21
POU5F1
ATP6V1G2−DDX39B
HCG20
PSORS1C3
SNORD117
LOC105375014
HCG27
SNORD84
DDX39B−AS1
11 genes omitted
30.8
31
31.2
31.4
31.6
Position on chr6 (Mb)

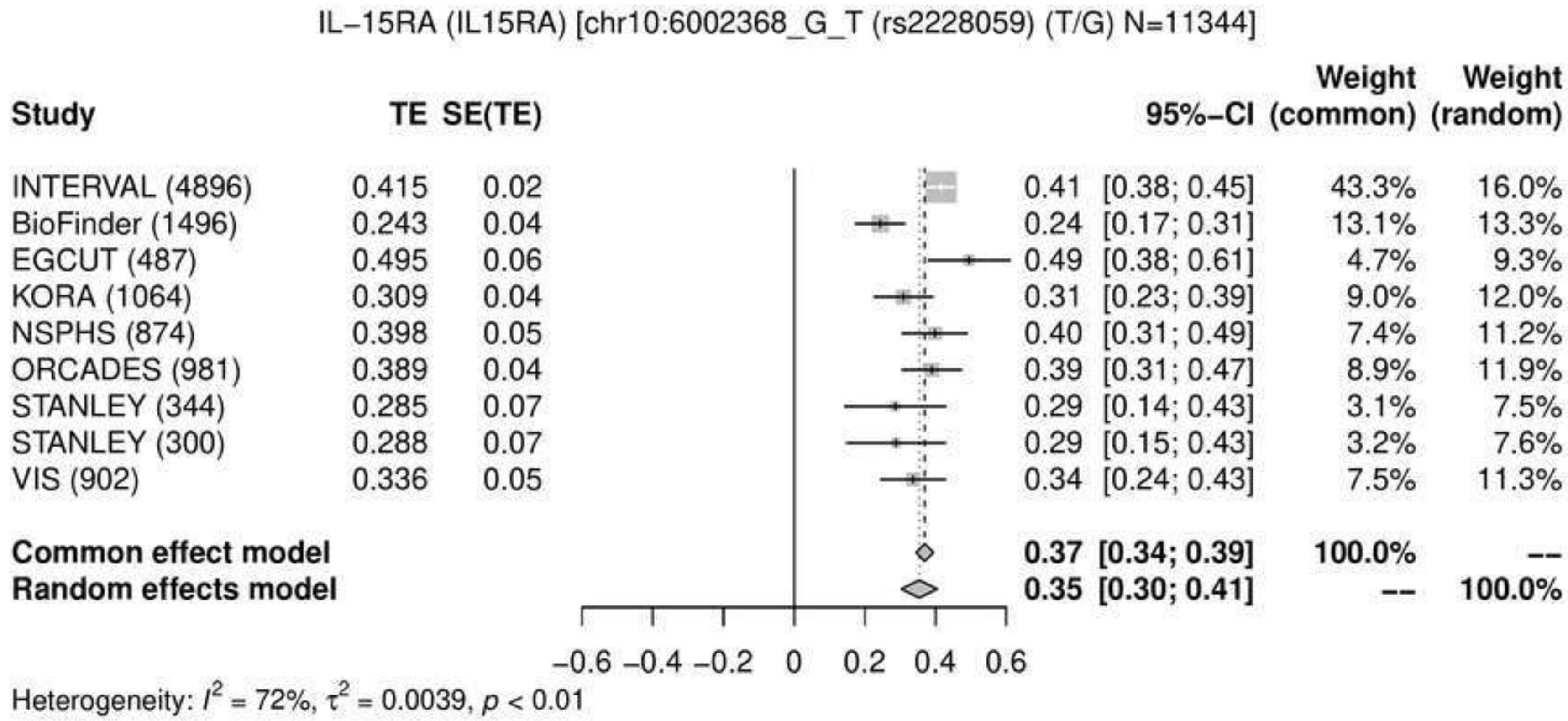
IL−15RA (IL15RA) [chr10:6002368_G_T (rs2228059) (T/G) N=11344]
Study TE SE(TE) 95%−CI Weight (common) Weight (random)
INTERVAL (4896) 0.415 0.02 0.41 [0.38; 0.45] 43.3% 16.0%
BioFinder (1496) 0.243 0.04 0.24 [0.17; 0.31] 13.1% 13.3%
EGCUT (487) 0.495 0.06 0.49 [0.38; 0.61] 4.7% 9.3%
KORA (1064) 0.309 0.04 0.31 [0.23; 0.39] 9.0% 12.0%
NSPHS (874) 0.398 0.05 0.40 [0.31; 0.49] 7.4% 11.2%
ORCADES (981) 0.389 0.04 0.39 [0.31; 0.47] 8.9% 11.9%
STANLEY (344) 0.285 0.07 0.29 [0.14; 0.43] 3.1% 7.5%
STANLEY (300) 0.288 0.07 0.29 [0.15; 0.43] 3.2% 7.6%
VIS (902) 0.336 0.05 0.34 [0.24; 0.43] 7.5% 11.3%
Common effect model 0.37 [0.34; 0.39] 100.0% --
Random effects model 0.35 [0.30; 0.41] -- 100.0%
−0.6 −0.4 −0.2 0 0.2 0.4 0.6
Heterogeneity: I^2 = 72%, τ^2 = 0.0039, p < 0.01

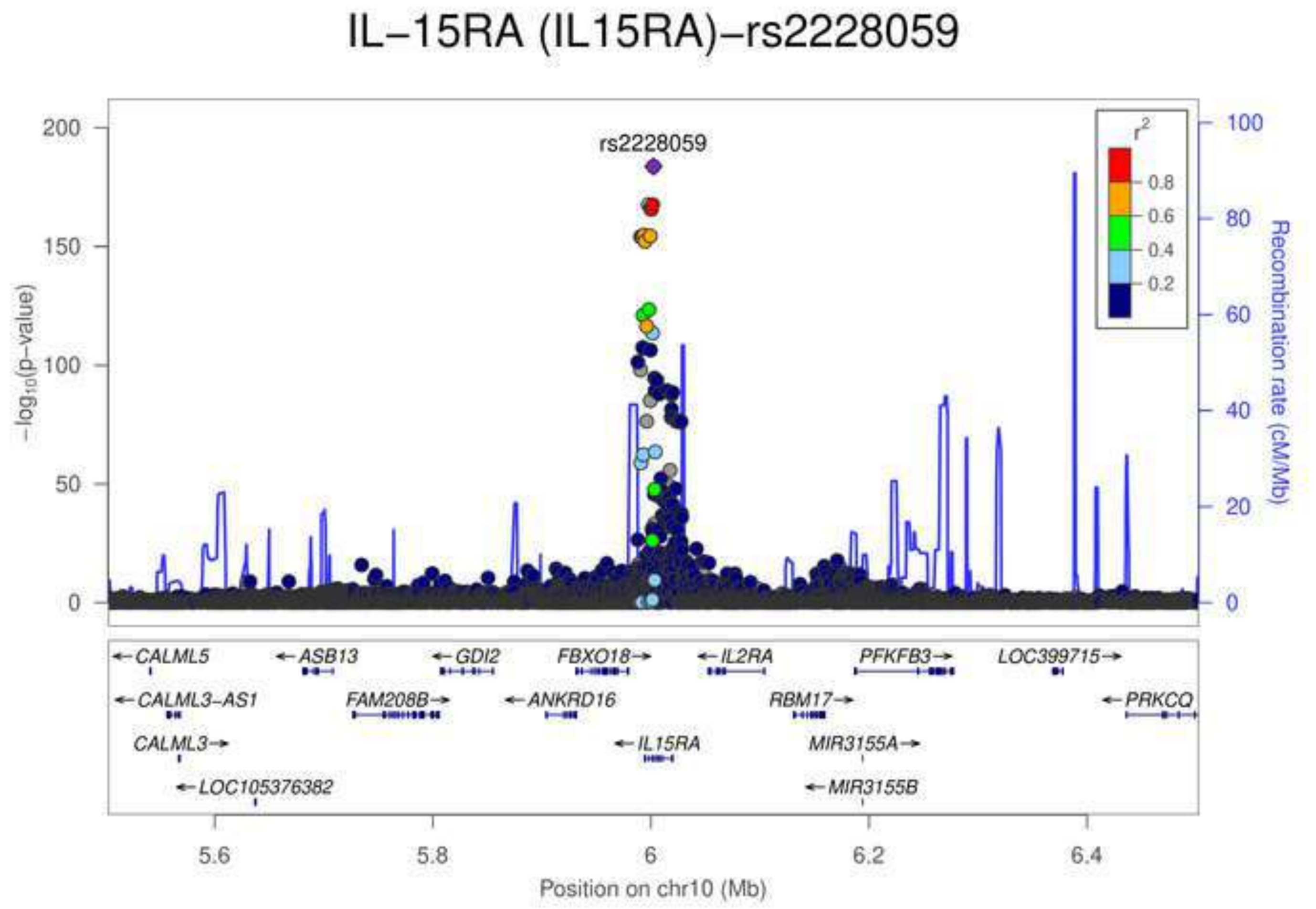
IL−15RA (IL15RA)−rs2228059
rs2228059
−log10(p−value)
Recombination rate (cM/Mb)
r2
0.8
0.6
0.4
0.2
CALML5
ASB13
GDI2
FBXO18
IL2RA
PFKFB3
LOC399715
CALML3−AS1
FAM208B
ANKRD16
RBM17
PRKCQ
CALML3
IL15RA
MIR3155A
LOC105376382
MIR3155B
5.6
5.8
6
6.2
6.4
Position on chr10 (Mb)

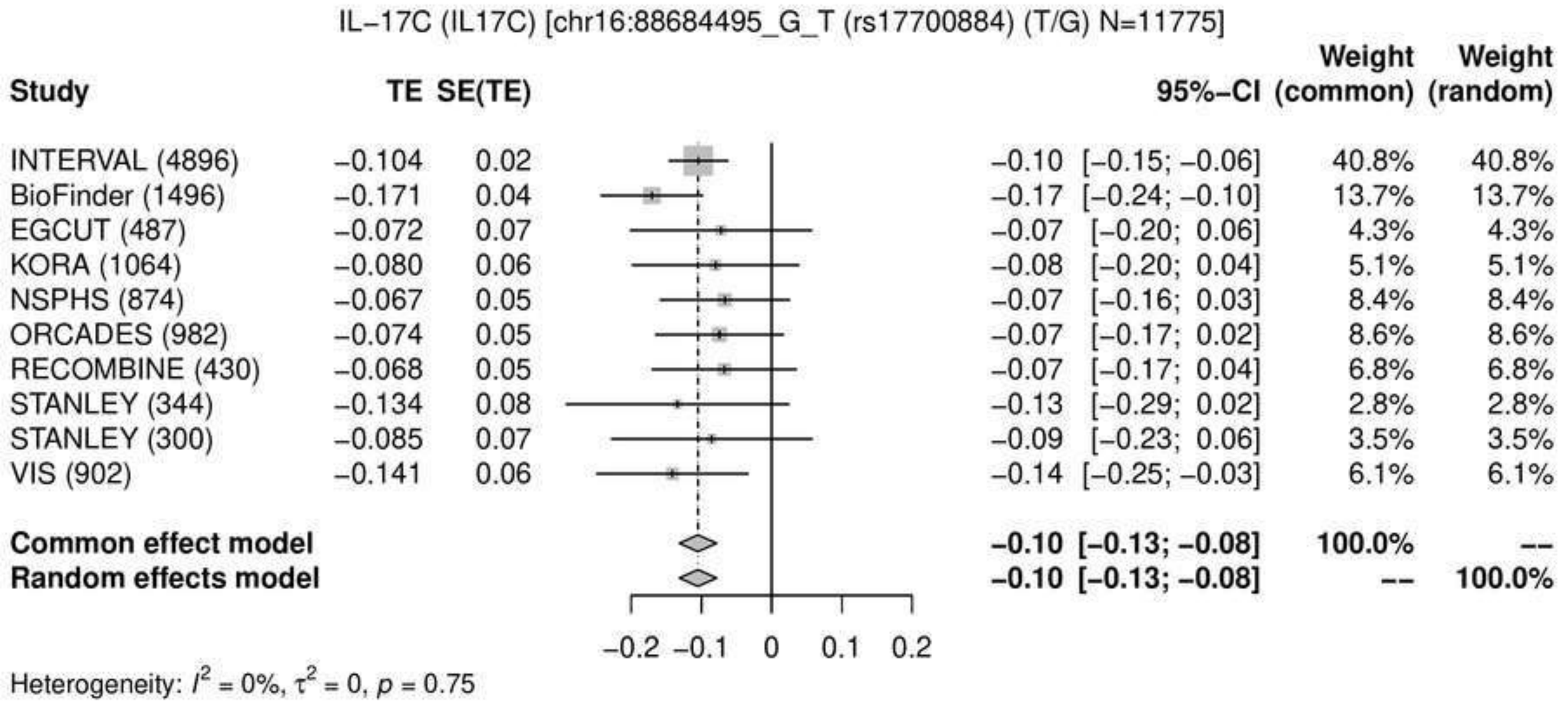
IL−17C (IL17C) [chr16:88684495_G_T (rs17700884) (T/G) N=11775]
Study
TE
SE(TE)
95%−CI
Weight (common)
Weight (random)
INTERVAL (4896) −0.104 0.02 −0.10 [−0.15; −0.06] 40.8% 40.8%
BioFinder (1496) −0.171 0.04 −0.17 [−0.24; −0.10] 13.7% 13.7%
EGCUT (487) −0.072 0.07 −0.07 [−0.20; 0.06] 4.3% 4.3%
KORA (1064) −0.080 0.06 −0.08 [−0.20; 0.04] 5.1% 5.1%
NSPHS (874) −0.067 0.05 −0.07 [−0.16; 0.03] 8.4% 8.4%
ORCADES (982) −0.074 0.05 −0.07 [−0.17; 0.02] 8.6% 8.6%
RECOMBINE (430) −0.068 0.05 −0.07 [−0.17; 0.04] 6.8% 6.8%
STANLEY (344) −0.134 0.08 −0.13 [−0.29; 0.02] 2.8% 2.8%
STANLEY (300) −0.085 0.07 −0.09 [−0.23; 0.06] 3.5% 3.5%
VIS (902) −0.141 0.06 −0.14 [−0.25; −0.03] 6.1% 6.1%
Common effect model −0.10 [−0.13; −0.08] 100.0% --
Random effects model −0.10 [−0.13; −0.08] -- 100.0%
−0.2 −0.1 0 0.1 0.2
Heterogeneity: I² = 0%, τ² = 0, p = 0.75

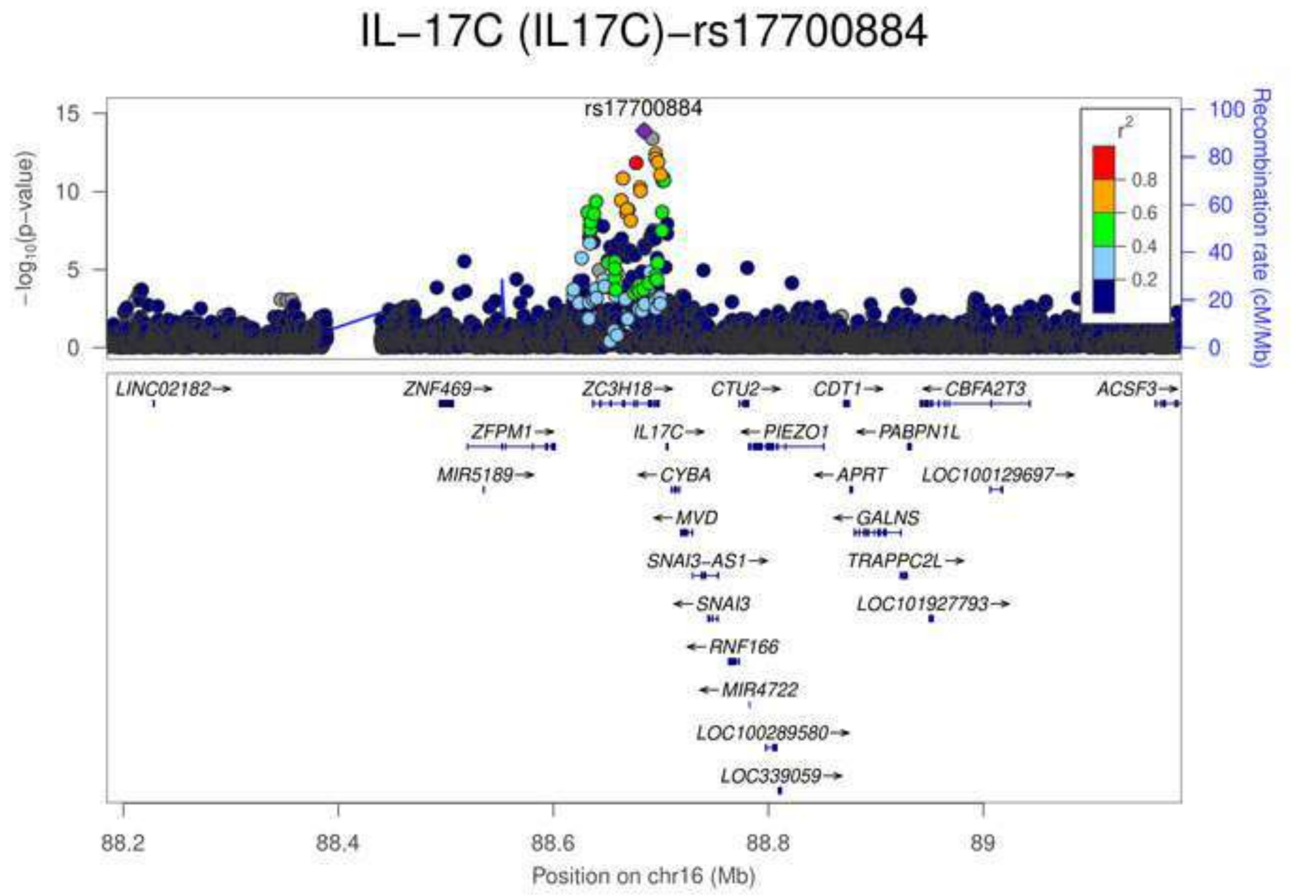
IL−17C (IL17C)−rs17700884
rs17700884
−log10(p−value)
Recombination rate (cM/Mb)
r²
0.8
0.6
0.4
0.2
LINC02182
ZNF469
ZC3H18
CTU2
CDT1
CBFA2T3
ACSF3
ZFPM1
IL17C
PIEZO1
PABPN1L
MIR5189
CYBA
APRT
LOC100129697
MVD
GALNS
SNAI3−AS1
TRAPPC2L
SNAI3
LOC101927793
RNF166
MIR4722
LOC100289580
LOC339059
88.2
88.4
88.6
88.8
89
Position on chr16 (Mb)

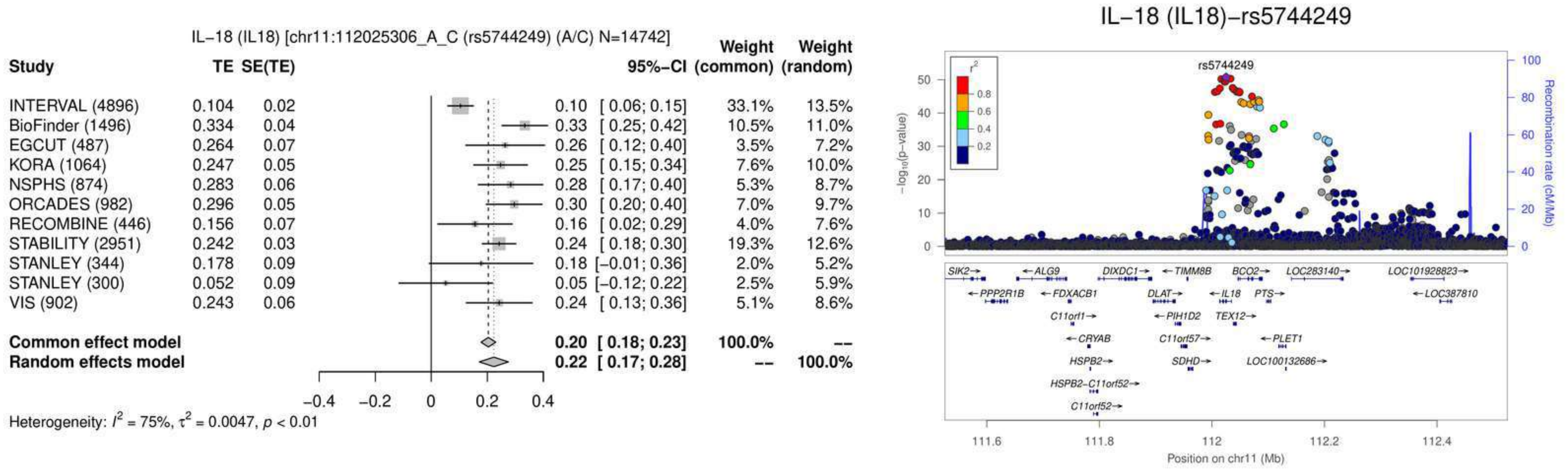
IL−18 (IL18) [chr11:112025306_A_C (rs5744249) (A/C) N=14742]
Study
TE
SE(TE)
95%−CI
Weight (common)
Weight (random)
INTERVAL (4896) 0.104 0.02 0.10 [0.06; 0.15] 33.1% 13.5%
BioFinder (1496) 0.334 0.04 0.33 [0.25; 0.42] 10.5% 11.0%
EGCUT (487) 0.264 0.07 0.26 [0.12; 0.40] 3.5% 7.2%
KORA (1064) 0.247 0.05 0.25 [0.15; 0.34] 7.6% 10.0%
NSPHS (874) 0.283 0.06 0.28 [0.17; 0.40] 5.3% 8.7%
ORCADES (982) 0.296 0.05 0.30 [0.20; 0.40] 7.0% 9.7%
RECOMBINE (446) 0.156 0.07 0.16 [0.02; 0.29] 4.0% 7.6%
STABILITY (2951) 0.242 0.03 0.24 [0.18; 0.30] 19.3% 12.6%
STANLEY (344) 0.178 0.09 0.18 [−0.01; 0.36] 2.0% 5.2%
STANLEY (300) 0.052 0.09 0.05 [−0.12; 0.22] 2.5% 5.9%
VIS (902) 0.243 0.06 0.24 [0.13; 0.36] 5.1% 8.6%
Common effect model 0.20 [0.18; 0.23] 100.0% --
Random effects model 0.22 [0.17; 0.28] -- 100.0%
−0.4 −0.2 0 0.2 0.4
Heterogeneity: I^2 = 75%, τ^2 = 0.0047, p < 0.01
IL−18 (IL18)−rs5744249
rs5744249
r^2
0.8
0.6
0.4
0.2
$-\log_{10}$(p−value)
Recombination rate (cM/Mb)
SIK2→
←ALG9
DIXDC1→
←TIMM8B
BCO2→
LOC283140→
LOC101928823→
←PPP2R1B
←FDXACB1
DLAT→
←IL18
PTS→
←LOC387810
C11orf1→
←PIH1D2
TEX12→
←CRYAB
C11orf57→
←PLET1
HSPB2→
SDHD→
LOC100132686→
HSPB2−C11orf52→
C11orf52→
111.6 111.8 112 112.2 112.4
Position on chr11 (Mb)

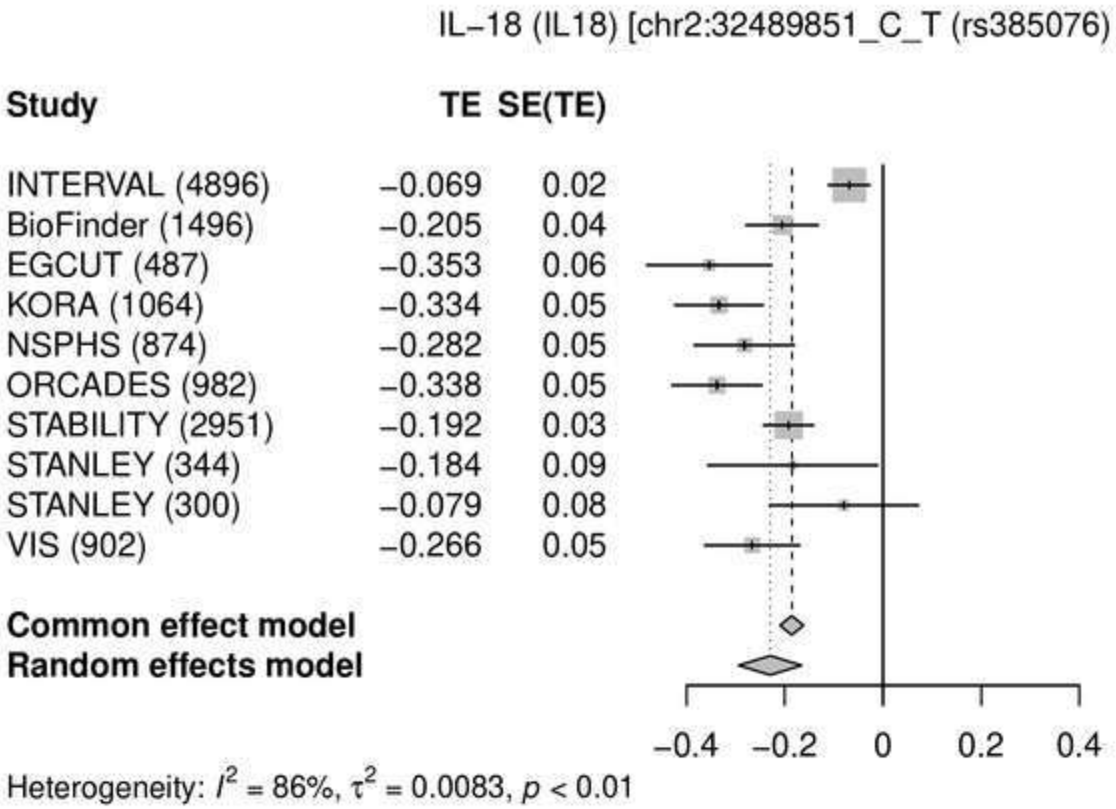
IL−18 (IL18) [chr2:32489851_C_T (rs385076) (T/C) N=14296]
Study | TE | SE(TE) | 95%−CI | Weight (common) | Weight (random)
INTERVAL (4896) | −0.069 | 0.02 | −0.07 [−0.11; −0.03] | 33.0% | 12.5%
BioFinder (1496) | −0.205 | 0.04 | −0.21 [−0.28; −0.13] | 10.7% | 11.2%
EGCUT (487) | −0.353 | 0.06 | −0.35 [−0.48; −0.23] | 3.6% | 8.7%
KORA (1064) | −0.334 | 0.05 | −0.33 [−0.42; −0.24] | 7.2% | 10.5%
NSPHS (874) | −0.282 | 0.05 | −0.28 [−0.38; −0.18] | 5.6% | 9.9%
ORCADES (982) | −0.338 | 0.05 | −0.34 [−0.43; −0.25] | 6.9% | 10.4%
STABILITY (2951) | −0.192 | 0.03 | −0.19 [−0.24; −0.14] | 22.3% | 12.2%
STANLEY (344) | −0.184 | 0.09 | −0.18 [−0.36; −0.01] | 1.9% | 6.8%
STANLEY (300) | −0.079 | 0.08 | −0.08 [−0.23; 0.07] | 2.5% | 7.6%
VIS (902) | −0.266 | 0.05 | −0.27 [−0.36; −0.17] | 6.2% | 10.2%
Common effect model | −0.19 [−0.21; −0.16] | 100.0% | --
Random effects model | −0.23 [−0.29; −0.17] | -- | 100.0%
−0.4 −0.2 0 0.2 0.4
Heterogeneity: I^2 = 86%, τ^2 = 0.0083, p < 0.01

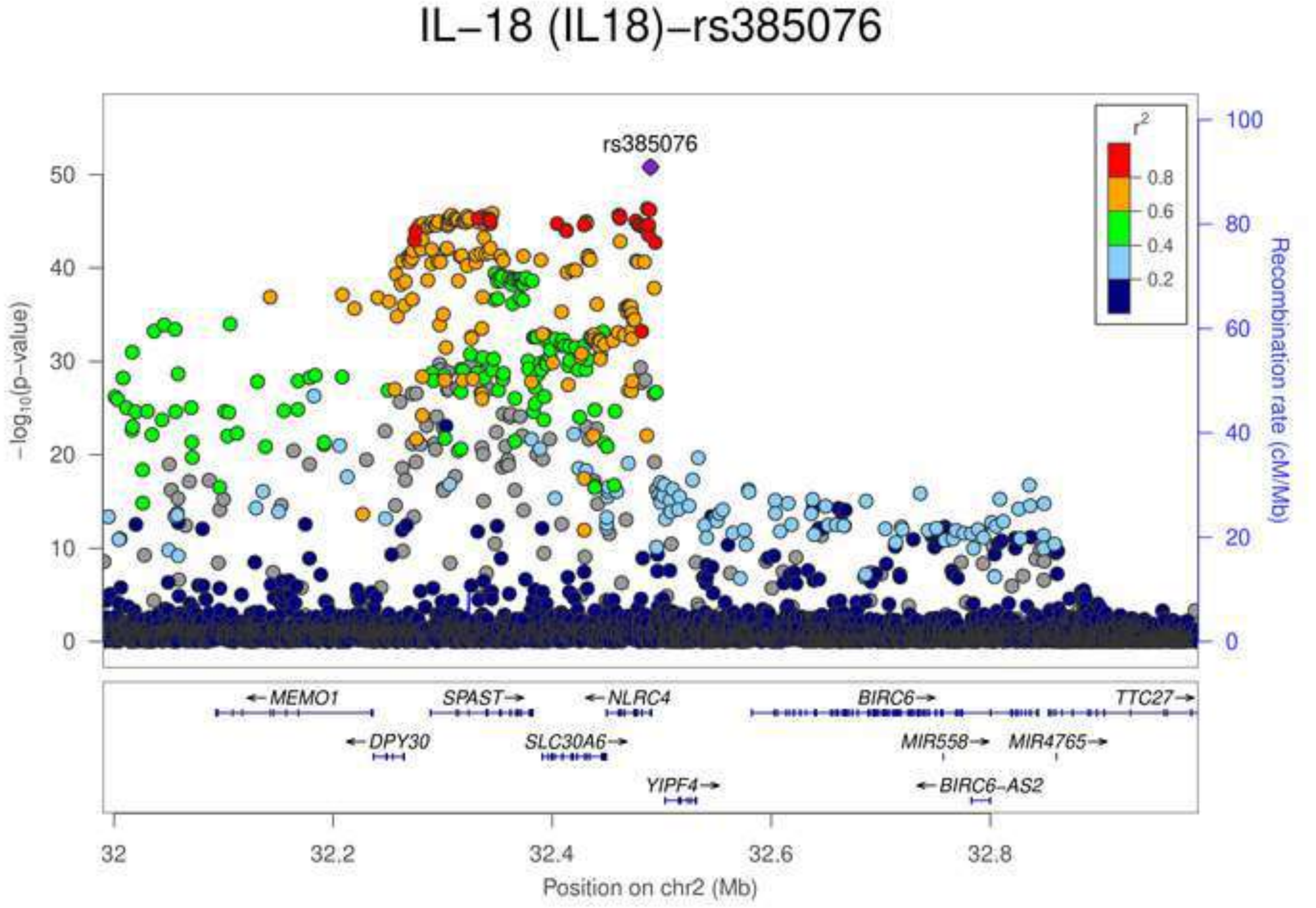
IL−18 (IL18)−rs385076
rs385076
r^2
0.8
0.6
0.4
0.2
$-\log_{10}$(p−value)
0 10 20 30 40 50
Recombination rate (cM/Mb)
0 20 40 60 80 100
MEMO1
SPAST
NLRC4
BIRC6
TTC27
DPY30
SLC30A6
MIR558
MIR4765
YIPF4
BIRC6−AS2
32 32.2 32.4 32.6 32.8
Position on chr2 (Mb)

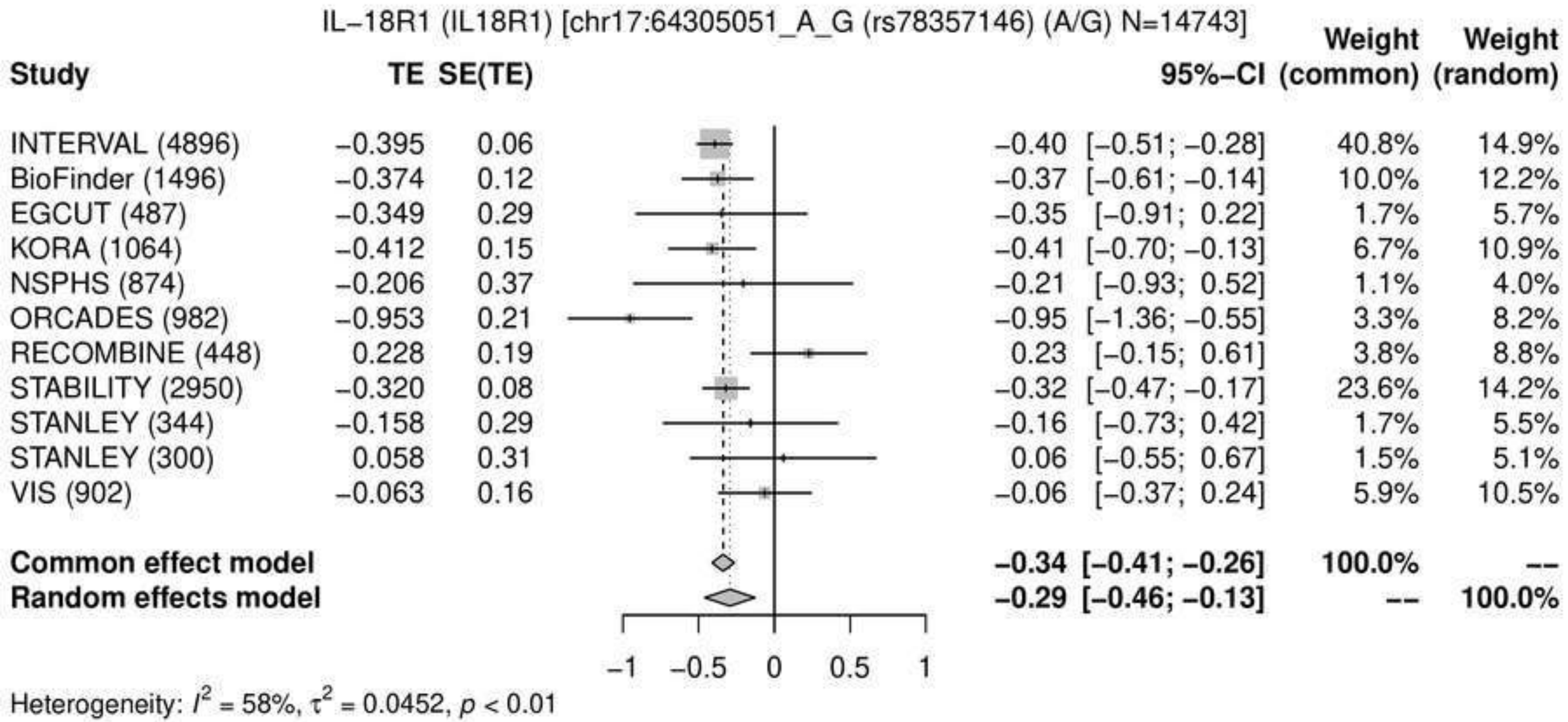
IL−18R1 (IL18R1) [chr17:64305051_A_G (rs78357146) (A/G) N=14743]
Study
TE
SE(TE)
95%−CI
Weight (common)
Weight (random)
INTERVAL (4896) −0.395 0.06 −0.40 [−0.51; −0.28] 40.8% 14.9%
BioFinder (1496) −0.374 0.12 −0.37 [−0.61; −0.14] 10.0% 12.2%
EGCUT (487) −0.349 0.29 −0.35 [−0.91; 0.22] 1.7% 5.7%
KORA (1064) −0.412 0.15 −0.41 [−0.70; −0.13] 6.7% 10.9%
NSPHS (874) −0.206 0.37 −0.21 [−0.93; 0.52] 1.1% 4.0%
ORCADES (982) −0.953 0.21 −0.95 [−1.36; −0.55] 3.3% 8.2%
RECOMBINE (448) 0.228 0.19 0.23 [−0.15; 0.61] 3.8% 8.8%
STABILITY (2950) −0.320 0.08 −0.32 [−0.47; −0.17] 23.6% 14.2%
STANLEY (344) −0.158 0.29 −0.16 [−0.73; 0.42] 1.7% 5.5%
STANLEY (300) 0.058 0.31 0.06 [−0.55; 0.67] 1.5% 5.1%
VIS (902) −0.063 0.16 −0.06 [−0.37; 0.24] 5.9% 10.5%
Common effect model −0.34 [−0.41; −0.26] 100.0% --
Random effects model −0.29 [−0.46; −0.13] -- 100.0%
−1 −0.5 0 0.5 1
Heterogeneity: I^2 = 58%, τ^2 = 0.0452, p < 0.01

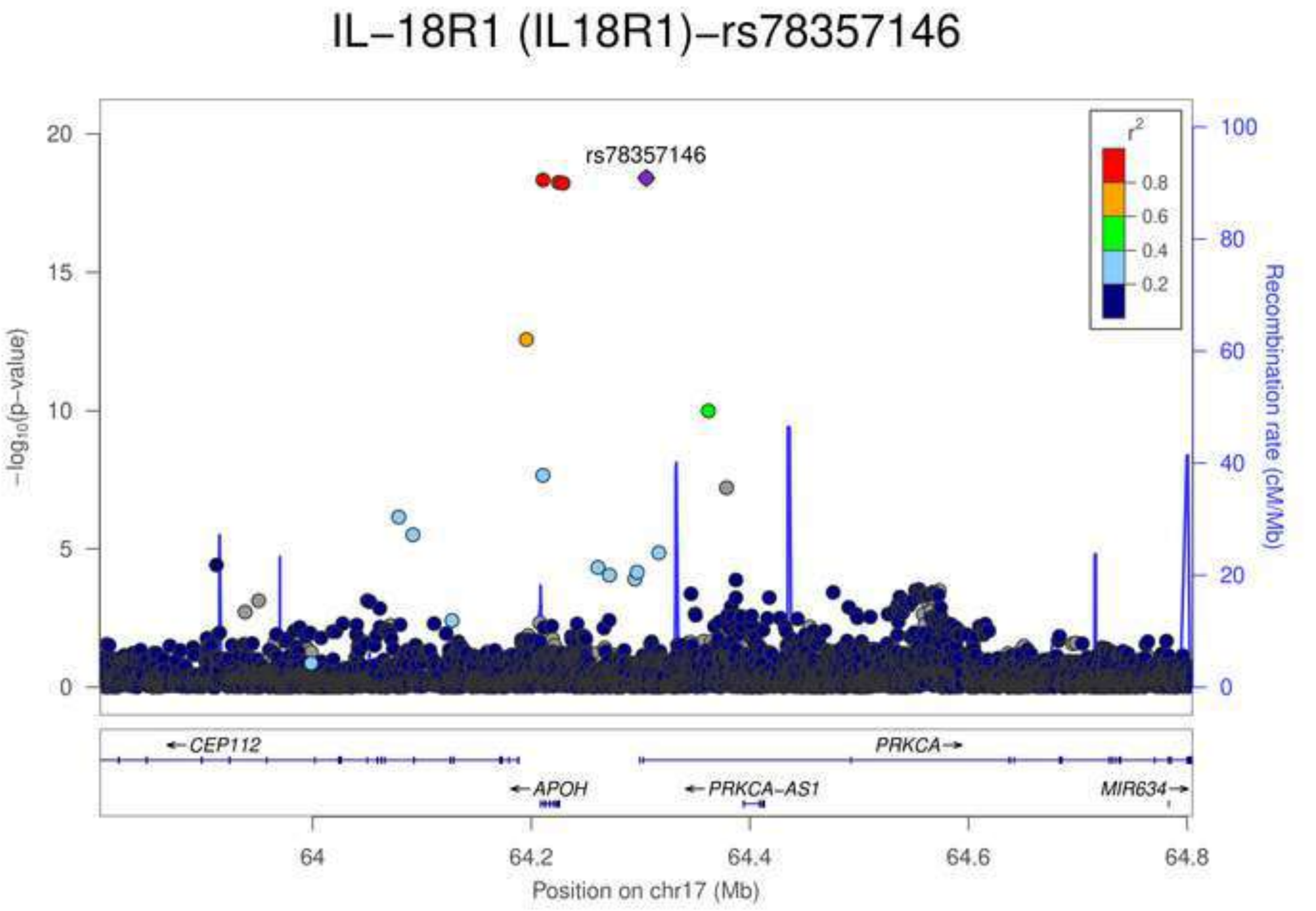
IL−18R1 (IL18R1)−rs78357146
rs78357146
r^2
0.8
0.6
0.4
0.2
$-\log_{10}$(p−value)
0
5
10
15
20
Recombination rate (cM/Mb)
0
20
40
60
80
100
CEP112
PRKCA
APOH
PRKCA−AS1
MIR634
64
64.2
64.4
64.6
64.8
Position on chr17 (Mb)

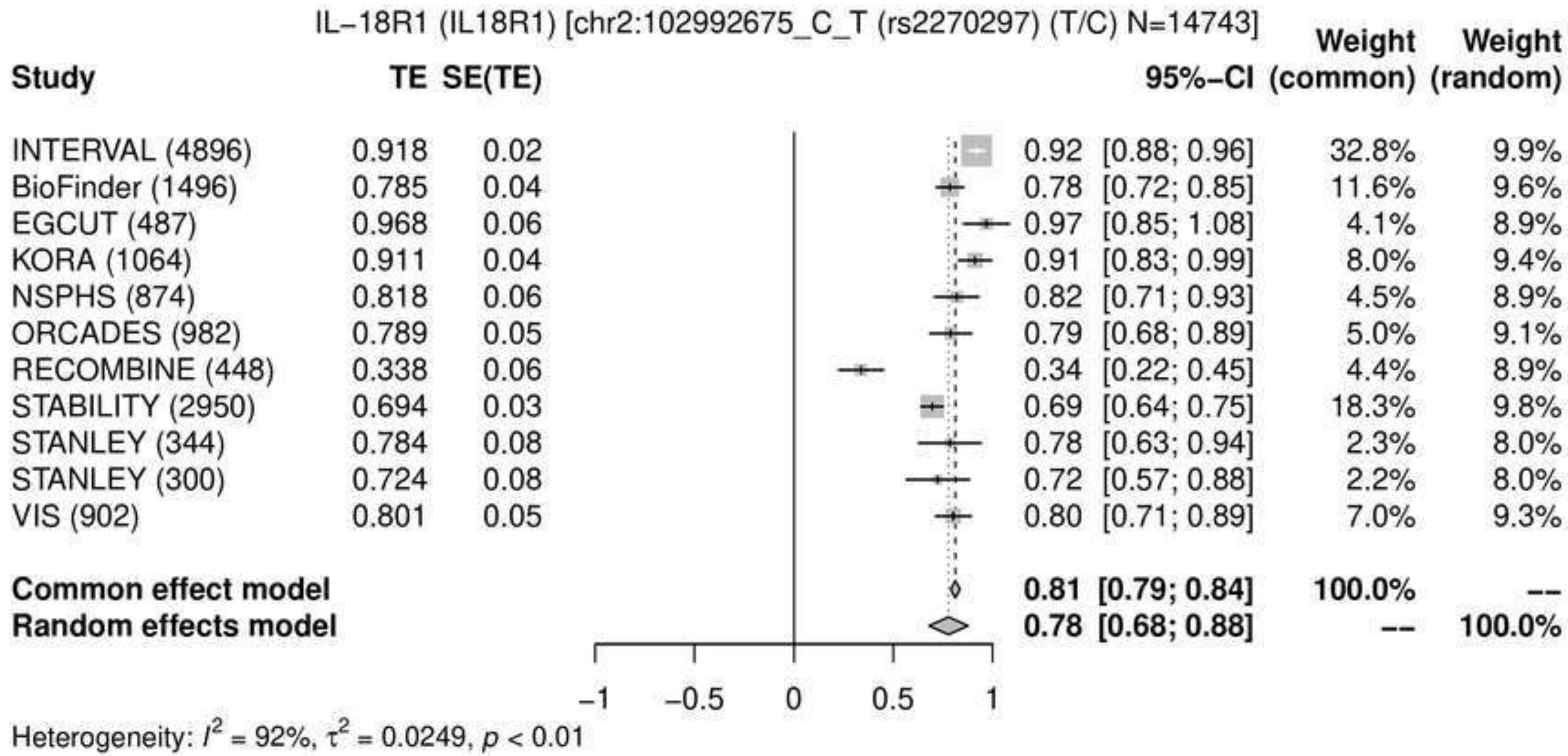

IL−18R1 (IL18R1) [chr2:102992675_C_T (rs2270297) (T/C) N=14743]
Study
TE
SE(TE)
95%−CI
Weight (common)
Weight (random)
INTERVAL (4896) 0.918 0.02 0.92 [0.88; 0.96] 32.8% 9.9%
BioFinder (1496) 0.785 0.04 0.78 [0.72; 0.85] 11.6% 9.6%
EGCUT (487) 0.968 0.06 0.97 [0.85; 1.08] 4.1% 8.9%
KORA (1064) 0.911 0.04 0.91 [0.83; 0.99] 8.0% 9.4%
NSPHS (874) 0.818 0.06 0.82 [0.71; 0.93] 4.5% 8.9%
ORCADES (982) 0.789 0.05 0.79 [0.68; 0.89] 5.0% 9.1%
RECOMBINE (448) 0.338 0.06 0.34 [0.22; 0.45] 4.4% 8.9%
STABILITY (2950) 0.694 0.03 0.69 [0.64; 0.75] 18.3% 9.8%
STANLEY (344) 0.784 0.08 0.78 [0.63; 0.94] 2.3% 8.0%
STANLEY (300) 0.724 0.08 0.72 [0.57; 0.88] 2.2% 8.0%
VIS (902) 0.801 0.05 0.80 [0.71; 0.89] 7.0% 9.3%
Common effect model 0.81 [0.79; 0.84] 100.0% --
Random effects model 0.78 [0.68; 0.88] -- 100.0%
−1
−0.5
0
0.5
1
Heterogeneity: I^2 = 92%, τ^2 = 0.0249, p < 0.01

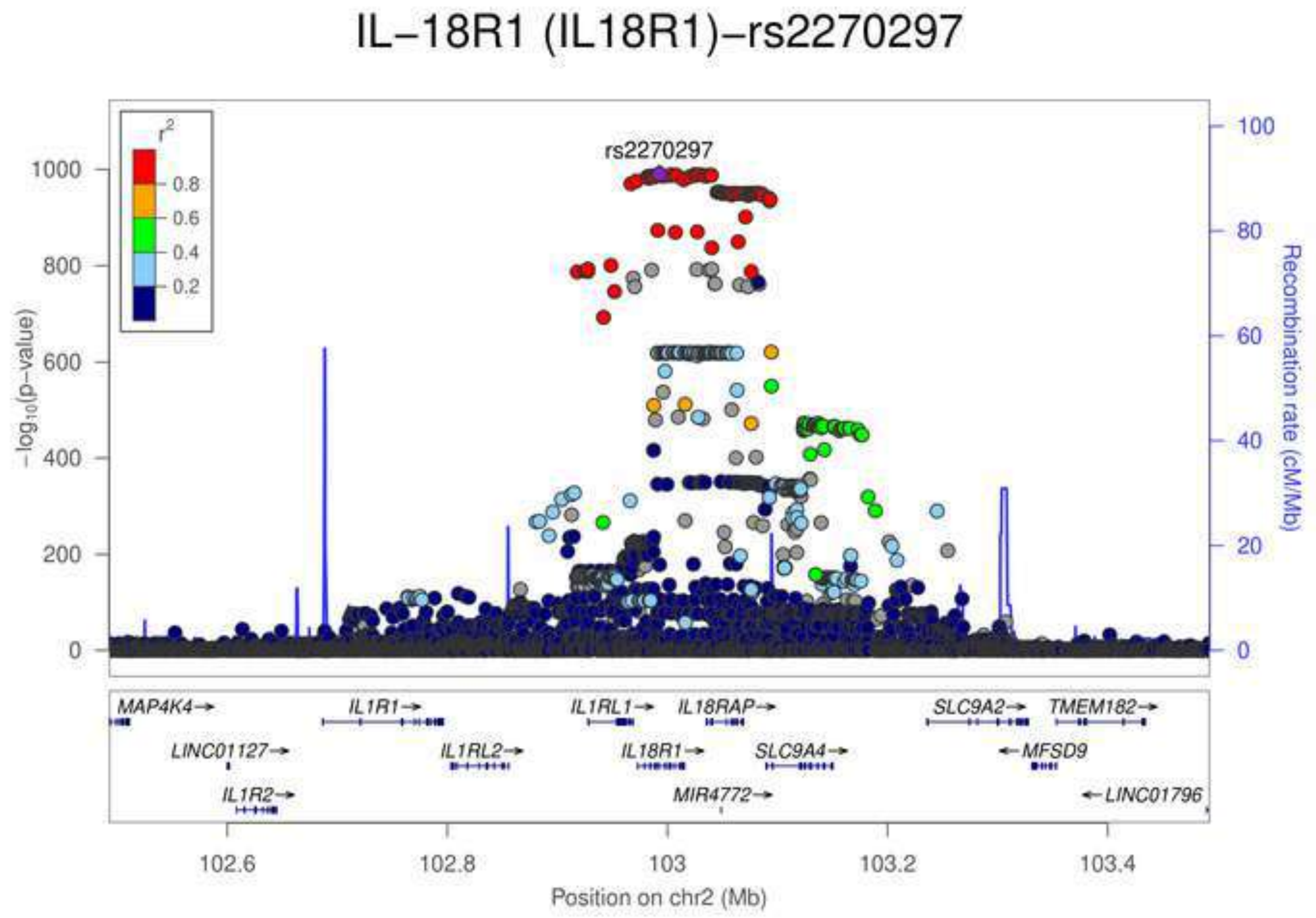

IL−18R1 (IL18R1)−rs2270297
rs2270297
r^2
0.8
0.6
0.4
0.2
$-\log_{10}$(p−value)
0
200
400
600
800
1000
Recombination rate (cM/Mb)
0
20
40
60
80
100
MAP4K4→
IL1R1→
IL1RL1→
IL18RAP→
SLC9A2→
TMEM182→
LINC01127→
IL1RL2→
IL18R1→
SLC9A4→
←MFSD9
IL1R2→
MIR4772→
←LINC01796
102.6
102.8
103
103.2
103.4
Position on chr2 (Mb)

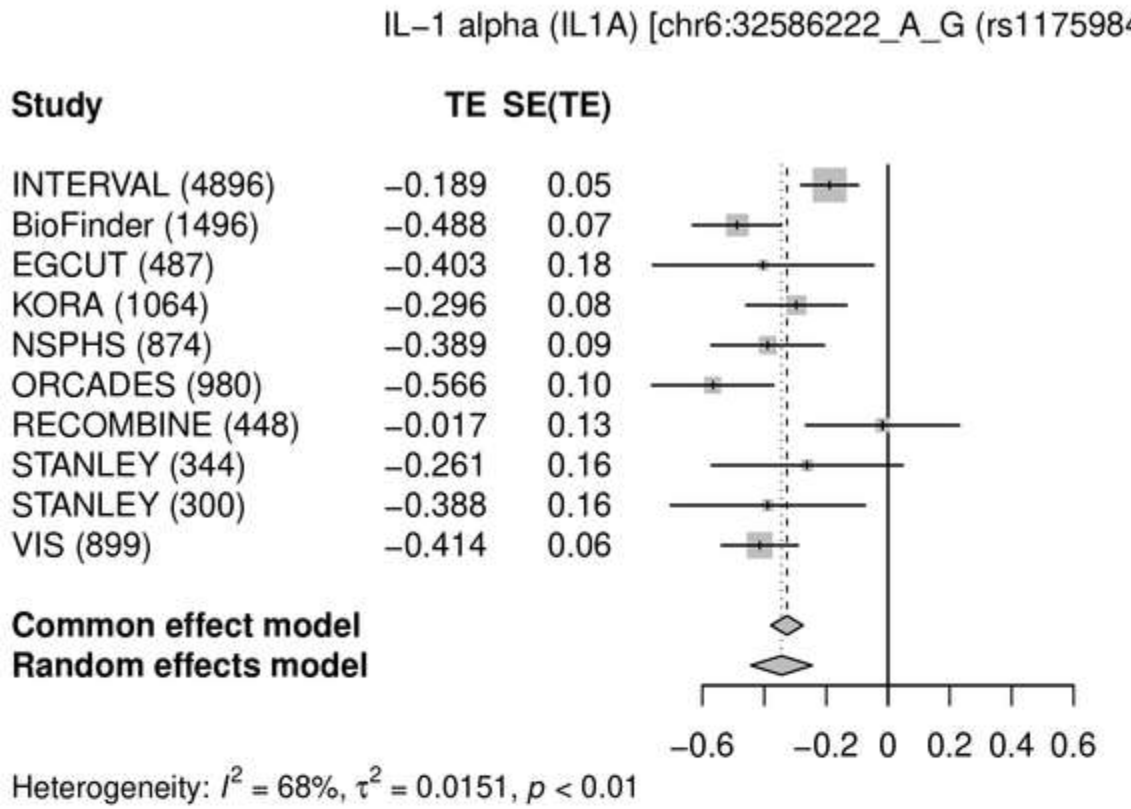
IL–1 alpha (IL1A) [chr6:32586222_A_G (rs11759846) (A/G) N=11788]
Study TE SE(TE) 95%–CI Weight (common) Weight (random)
INTERVAL (4896) –0.189 0.05 –0.19 [–0.28; –0.10] 31.5% 14.8%
BioFinder (1496) –0.488 0.07 –0.49 [–0.63; –0.34] 13.1% 12.6%
EGCUT (487) –0.403 0.18 –0.40 [–0.76; –0.05] 2.1% 5.3%
KORA (1064) –0.296 0.08 –0.30 [–0.46; –0.13] 10.3% 11.7%
NSPHS (874) –0.389 0.09 –0.39 [–0.57; –0.21] 8.1% 10.8%
ORCADES (980) –0.566 0.10 –0.57 [–0.76; –0.37] 7.0% 10.2%
RECOMBINE (448) –0.017 0.13 –0.02 [–0.27; 0.23] 4.4% 8.3%
STANLEY (344) –0.261 0.16 –0.26 [–0.57; 0.05] 2.9% 6.4%
STANLEY (300) –0.388 0.16 –0.39 [–0.70; –0.07] 2.8% 6.3%
VIS (899) –0.414 0.06 –0.41 [–0.54; –0.29] 17.7% 13.5%
Common effect model –0.33 [–0.38; –0.27] 100.0% --
Random effects model –0.34 [–0.44; –0.24] -- 100.0%
–0.6 –0.2 0 0.2 0.4 0.6
Heterogeneity: I^2 = 68%, τ^2 = 0.0151, p < 0.01

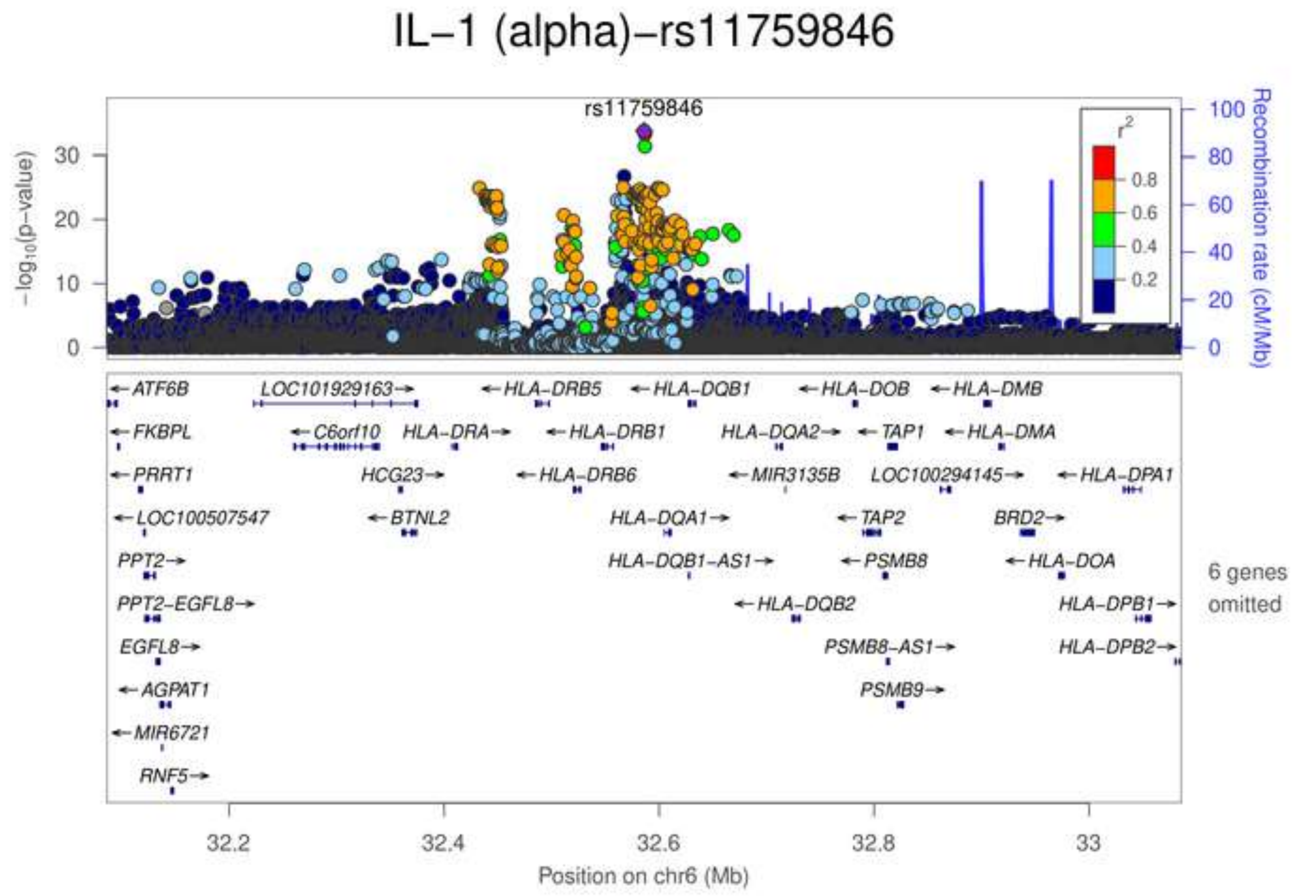
IL–1 (alpha)–rs11759846
rs11759846
$-\log_{10}$(p–value)
Recombination rate (cM/Mb)
r^2
0.8
0.6
0.4
0.2
ATF6B
LOC101929163
HLA–DRB5
HLA–DQB1
HLA–DOB
HLA–DMB
FKBPL
C6orf10
HLA–DRA
HLA–DRB1
HLA–DQA2
TAP1
HLA–DMA
PRRT1
HCG23
HLA–DRB6
MIR3135B
LOC100294145
HLA–DPA1
LOC100507547
BTNL2
HLA–DQA1
TAP2
BRD2
PPT2
HLA–DQB1–AS1
PSMB8
HLA–DOA
PPT2–EGFL8
HLA–DQB2
HLA–DPB1
EGFL8
PSMB8–AS1
HLA–DPB2
AGPAT1
PSMB9
MIR6721
RNF5
6 genes omitted
32.2
32.4
32.6
32.8
33
Position on chr6 (Mb)

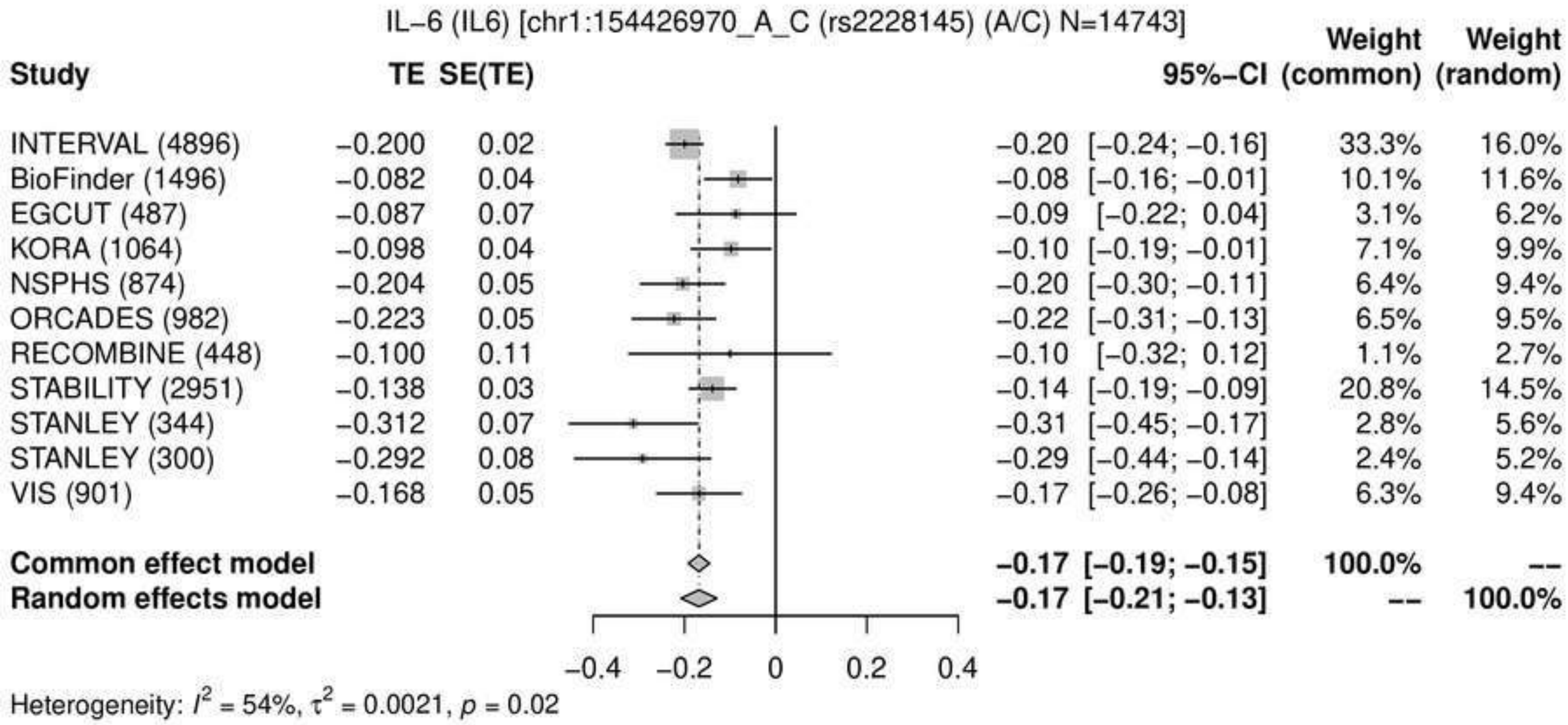
IL−6 (IL6) [chr1:154426970_A_C (rs2228145) (A/C) N=14743]
Study
TE
SE(TE)
95%−CI
Weight (common)
Weight (random)
INTERVAL (4896) −0.200 0.02 −0.20 [−0.24; −0.16] 33.3% 16.0%
BioFinder (1496) −0.082 0.04 −0.08 [−0.16; −0.01] 10.1% 11.6%
EGCUT (487) −0.087 0.07 −0.09 [−0.22; 0.04] 3.1% 6.2%
KORA (1064) −0.098 0.04 −0.10 [−0.19; −0.01] 7.1% 9.9%
NSPHS (874) −0.204 0.05 −0.20 [−0.30; −0.11] 6.4% 9.4%
ORCADES (982) −0.223 0.05 −0.22 [−0.31; −0.13] 6.5% 9.5%
RECOMBINE (448) −0.100 0.11 −0.10 [−0.32; 0.12] 1.1% 2.7%
STABILITY (2951) −0.138 0.03 −0.14 [−0.19; −0.09] 20.8% 14.5%
STANLEY (344) −0.312 0.07 −0.31 [−0.45; −0.17] 2.8% 5.6%
STANLEY (300) −0.292 0.08 −0.29 [−0.44; −0.14] 2.4% 5.2%
VIS (901) −0.168 0.05 −0.17 [−0.26; −0.08] 6.3% 9.4%
Common effect model −0.17 [−0.19; −0.15] 100.0% --
Random effects model −0.17 [−0.21; −0.13] -- 100.0%
−0.4 −0.2 0 0.2 0.4
Heterogeneity: I^2 = 54%, τ^2 = 0.0021, p = 0.02

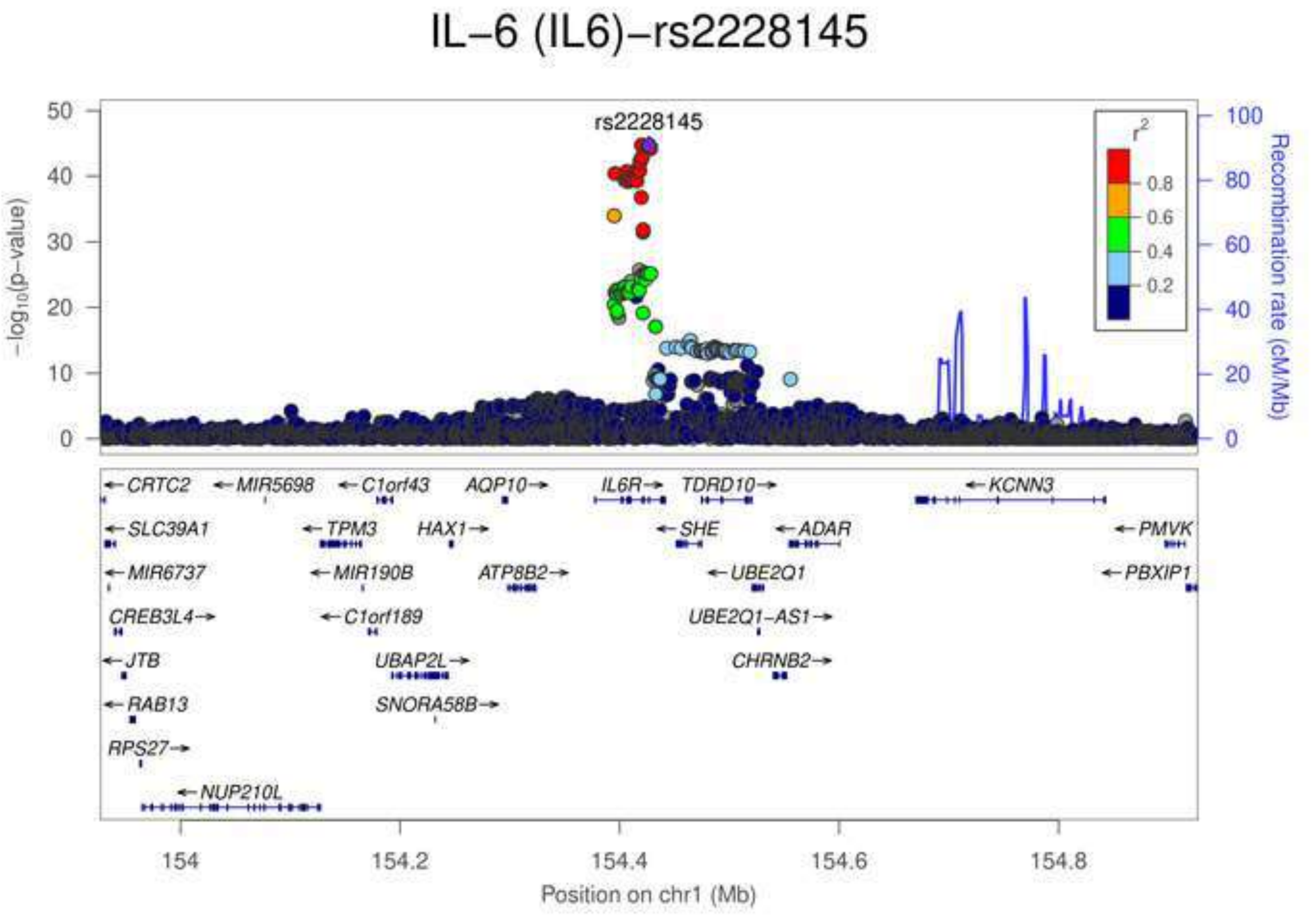
IL−6 (IL6)−rs2228145
rs2228145
$-\log_{10}$(p−value)
Recombination rate (cM/Mb)
r^2
0.8
0.6
0.4
0.2
CRTC2
MIR5698
C1orf43
AQP10
IL6R
TDRD10
KCNN3
SLC39A1
TPM3
HAX1
SHE
ADAR
PMVK
MIR6737
MIR190B
ATP8B2
UBE2Q1
PBXIP1
CREB3L4
C1orf189
UBE2Q1−AS1
JTB
UBAP2L
CHRNB2
RAB13
SNORA58B
RPS27
NUP210L
154 154.2 154.4 154.6 154.8
Position on chr1 (Mb)

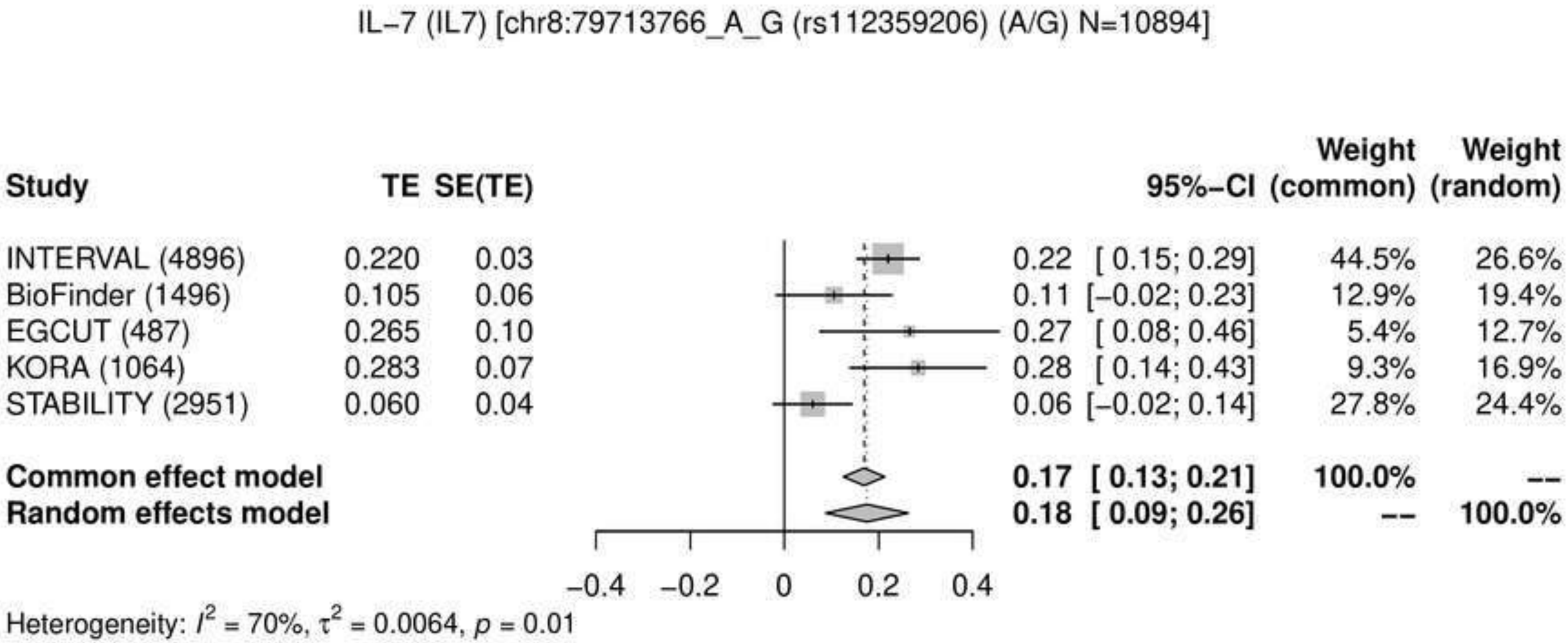
IL−7 (IL7) [chr8:79713766_A_G (rs112359206) (A/G) N=10894]
Study
TE SE(TE)
95%−CI
Weight (common)
Weight (random)
INTERVAL (4896) 0.220 0.03 0.22 [0.15; 0.29] 44.5% 26.6%
BioFinder (1496) 0.105 0.06 0.11 [−0.02; 0.23] 12.9% 19.4%
EGCUT (487) 0.265 0.10 0.27 [0.08; 0.46] 5.4% 12.7%
KORA (1064) 0.283 0.07 0.28 [0.14; 0.43] 9.3% 16.9%
STABILITY (2951) 0.060 0.04 0.06 [−0.02; 0.14] 27.8% 24.4%
Common effect model 0.17 [0.13; 0.21] 100.0% --
Random effects model 0.18 [0.09; 0.26] -- 100.0%
−0.4 −0.2 0 0.2 0.4
Heterogeneity: I² = 70%, τ² = 0.0064, p = 0.01

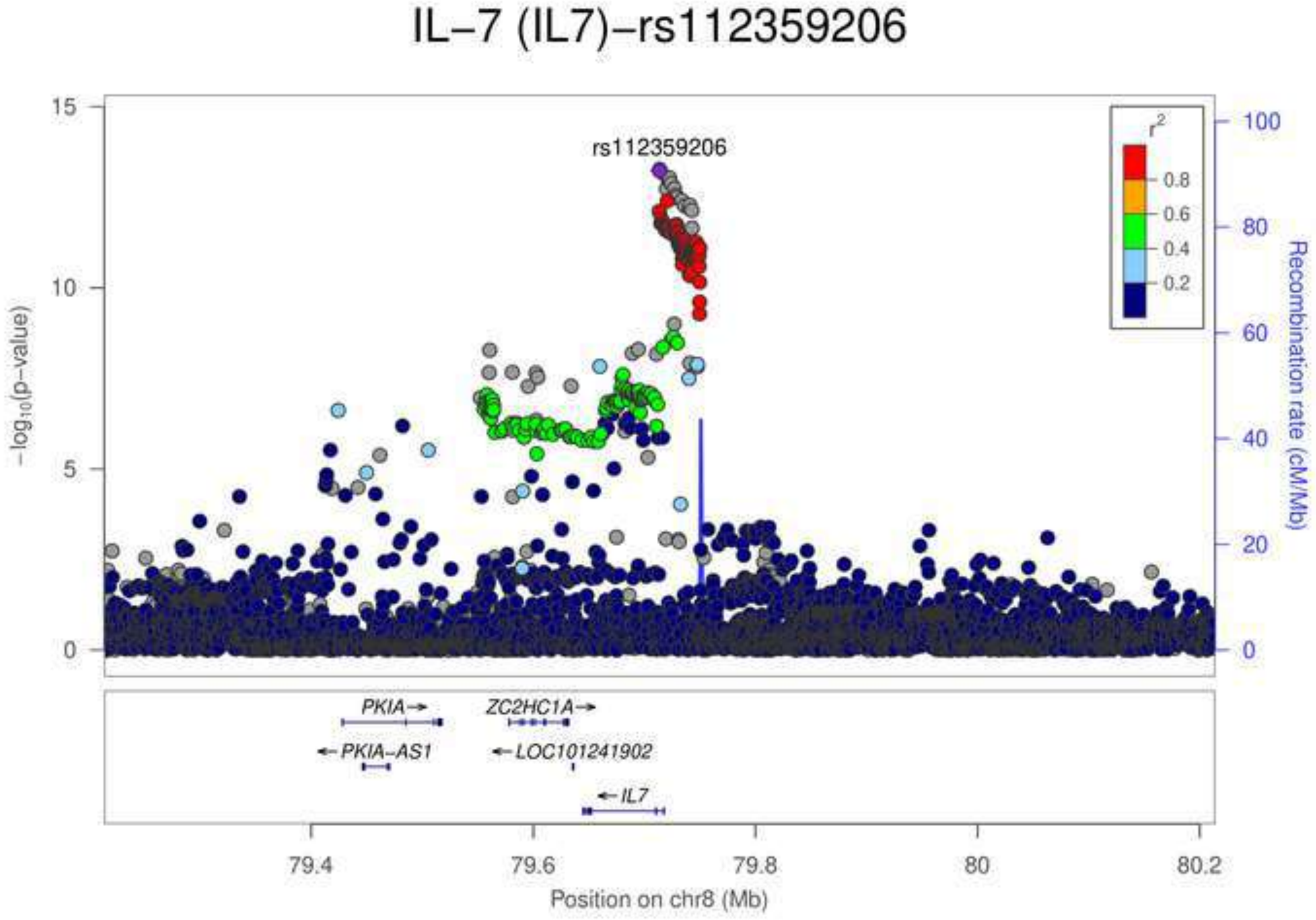
IL−7 (IL7)−rs112359206
rs112359206
−log10(p−value)
Recombination rate (cM/Mb)
r²
0.8
0.6
0.4
0.2
PKIA→
ZC2HC1A→
←PKIA−AS1
←LOC101241902
←IL7
79.4
79.6
79.8
80
80.2
Position on chr8 (Mb)

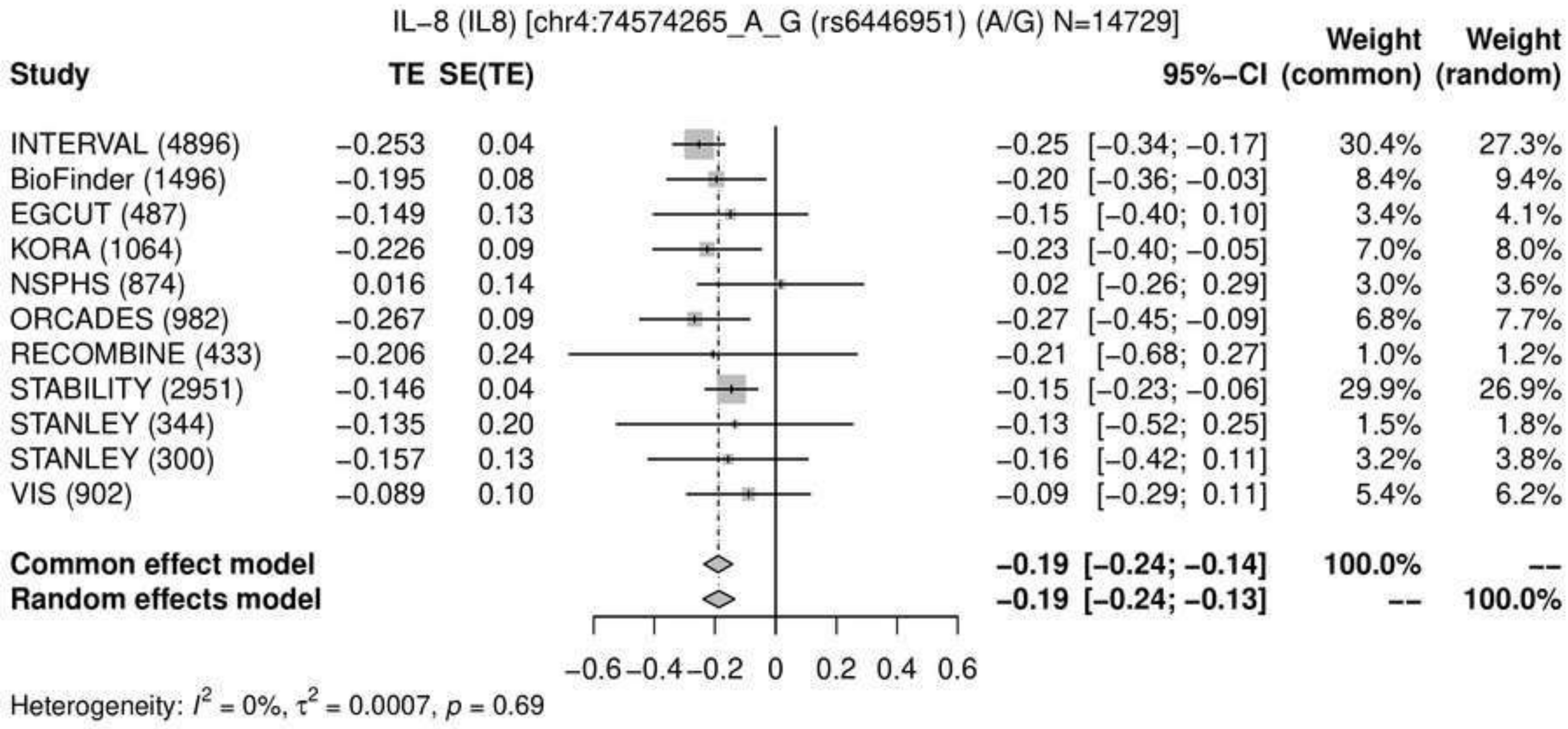
IL−8 (IL8) [chr4:74574265_A_G (rs6446951) (A/G) N=14729]
Study
TE
SE(TE)
95%−CI
Weight (common)
Weight (random)
INTERVAL (4896) −0.253 0.04 −0.25 [−0.34; −0.17] 30.4% 27.3%
BioFinder (1496) −0.195 0.08 −0.20 [−0.36; −0.03] 8.4% 9.4%
EGCUT (487) −0.149 0.13 −0.15 [−0.40; 0.10] 3.4% 4.1%
KORA (1064) −0.226 0.09 −0.23 [−0.40; −0.05] 7.0% 8.0%
NSPHS (874) 0.016 0.14 0.02 [−0.26; 0.29] 3.0% 3.6%
ORCADES (982) −0.267 0.09 −0.27 [−0.45; −0.09] 6.8% 7.7%
RECOMBINE (433) −0.206 0.24 −0.21 [−0.68; 0.27] 1.0% 1.2%
STABILITY (2951) −0.146 0.04 −0.15 [−0.23; −0.06] 29.9% 26.9%
STANLEY (344) −0.135 0.20 −0.13 [−0.52; 0.25] 1.5% 1.8%
STANLEY (300) −0.157 0.13 −0.16 [−0.42; 0.11] 3.2% 3.8%
VIS (902) −0.089 0.10 −0.09 [−0.29; 0.11] 5.4% 6.2%
Common effect model −0.19 [−0.24; −0.14] 100.0% --
Random effects model −0.19 [−0.24; −0.13] -- 100.0%
−0.6 −0.4 −0.2 0 0.2 0.4 0.6
Heterogeneity: I^2 = 0%, τ^2 = 0.0007, p = 0.69

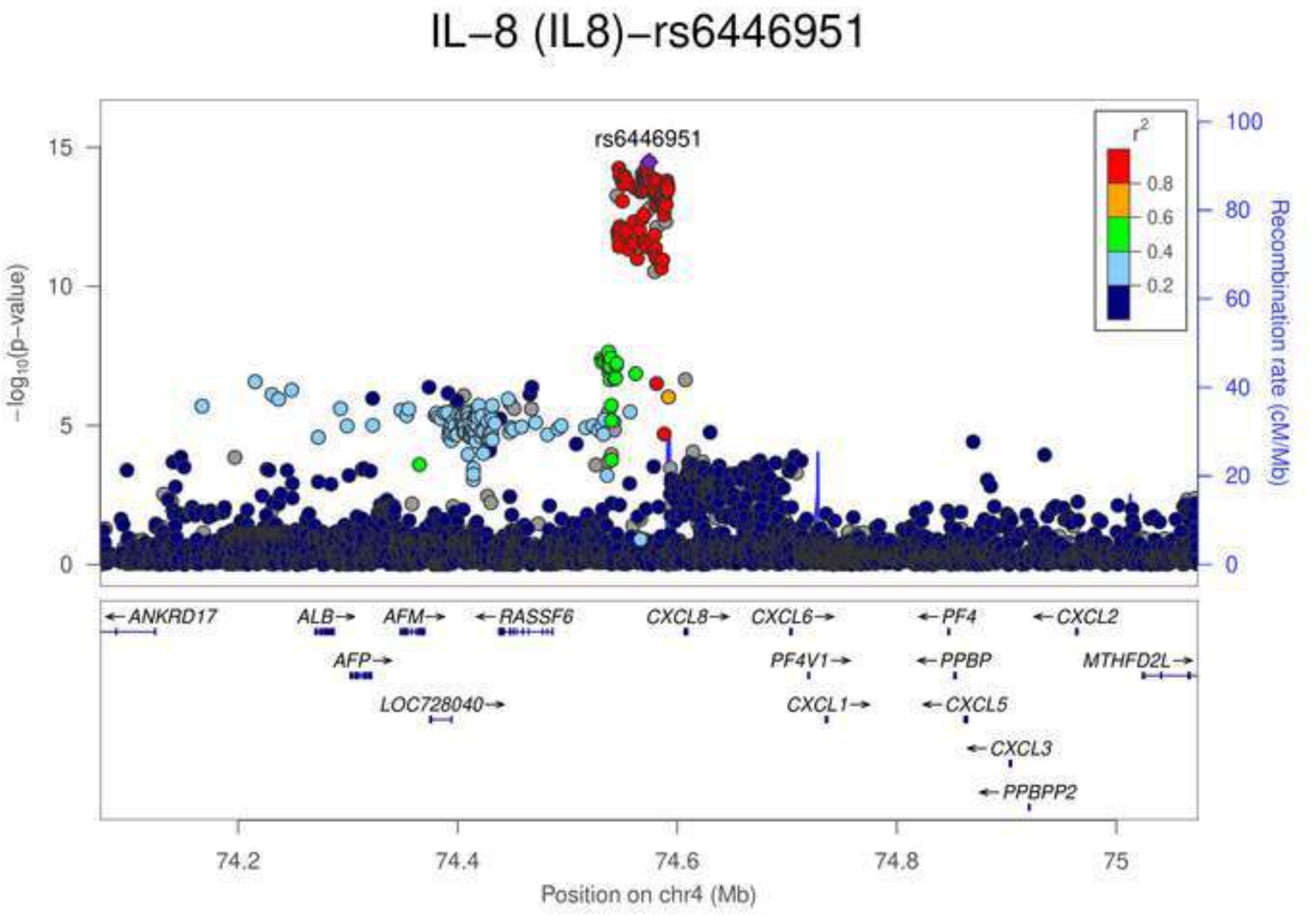
IL−8 (IL8)−rs6446951
rs6446951
r^2
0.8
0.6
0.4
0.2
−log10(p−value)
Recombination rate (cM/Mb)
ANKRD17
ALB
AFM
RASSF6
CXCL8
CXCL6
PF4
CXCL2
AFP
PF4V1
PPBP
MTHFD2L
LOC728040
CXCL1
CXCL5
CXCL3
PPBPP2
74.2
74.4
74.6
74.8
75
Position on chr4 (Mb)

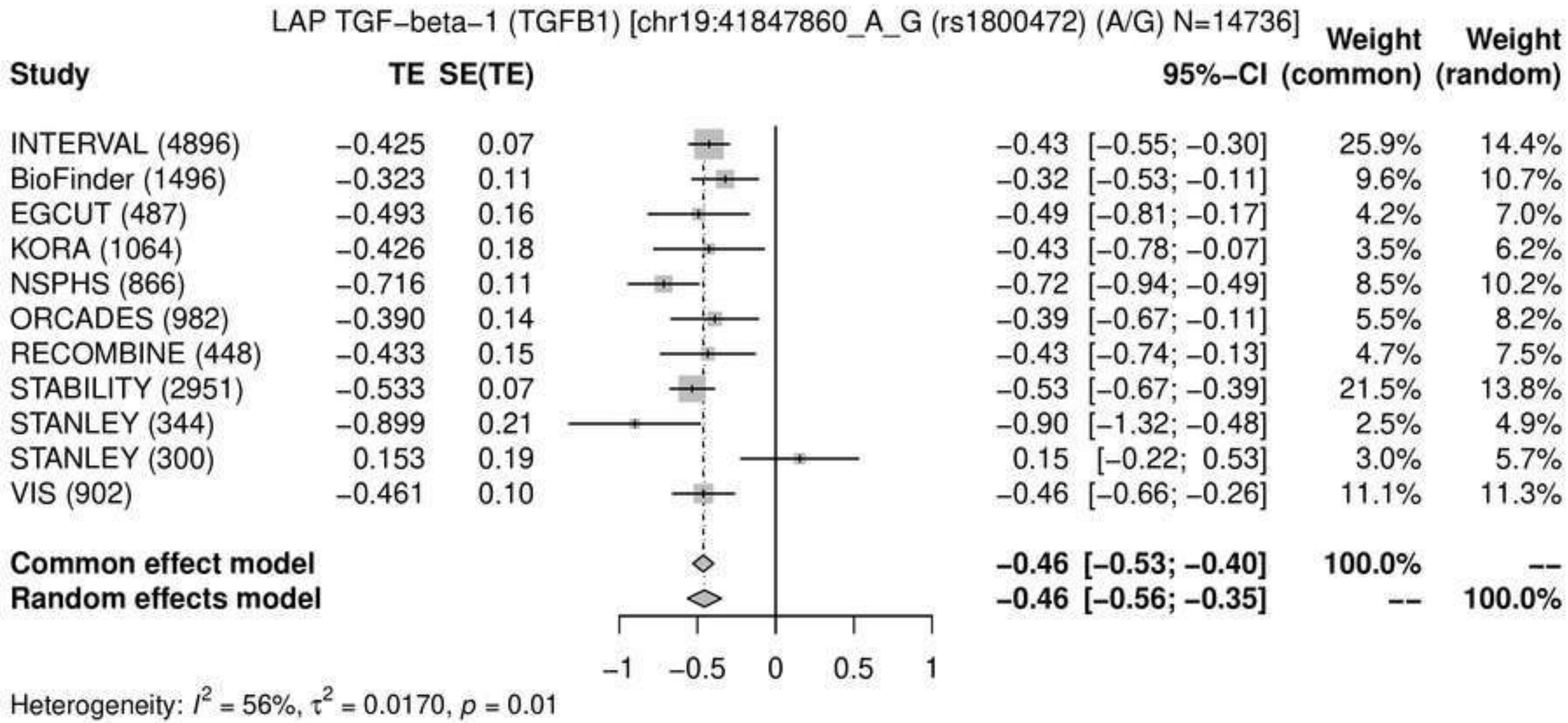
LAP TGF−beta−1 (TGFB1) [chr19:41847860_A_G (rs1800472) (A/G) N=14736]
Study
TE
SE(TE)
95%−CI
Weight (common)
Weight (random)
INTERVAL (4896) −0.425 0.07 −0.43 [−0.55; −0.30] 25.9% 14.4%
BioFinder (1496) −0.323 0.11 −0.32 [−0.53; −0.11] 9.6% 10.7%
EGCUT (487) −0.493 0.16 −0.49 [−0.81; −0.17] 4.2% 7.0%
KORA (1064) −0.426 0.18 −0.43 [−0.78; −0.07] 3.5% 6.2%
NSPHS (866) −0.716 0.11 −0.72 [−0.94; −0.49] 8.5% 10.2%
ORCADES (982) −0.390 0.14 −0.39 [−0.67; −0.11] 5.5% 8.2%
RECOMBINE (448) −0.433 0.15 −0.43 [−0.74; −0.13] 4.7% 7.5%
STABILITY (2951) −0.533 0.07 −0.53 [−0.67; −0.39] 21.5% 13.8%
STANLEY (344) −0.899 0.21 −0.90 [−1.32; −0.48] 2.5% 4.9%
STANLEY (300) 0.153 0.19 0.15 [−0.22; 0.53] 3.0% 5.7%
VIS (902) −0.461 0.10 −0.46 [−0.66; −0.26] 11.1% 11.3%
Common effect model −0.46 [−0.53; −0.40] 100.0% --
Random effects model −0.46 [−0.56; −0.35] -- 100.0%
−1 −0.5 0 0.5 1
Heterogeneity: I^2 = 56%, τ^2 = 0.0170, p = 0.01

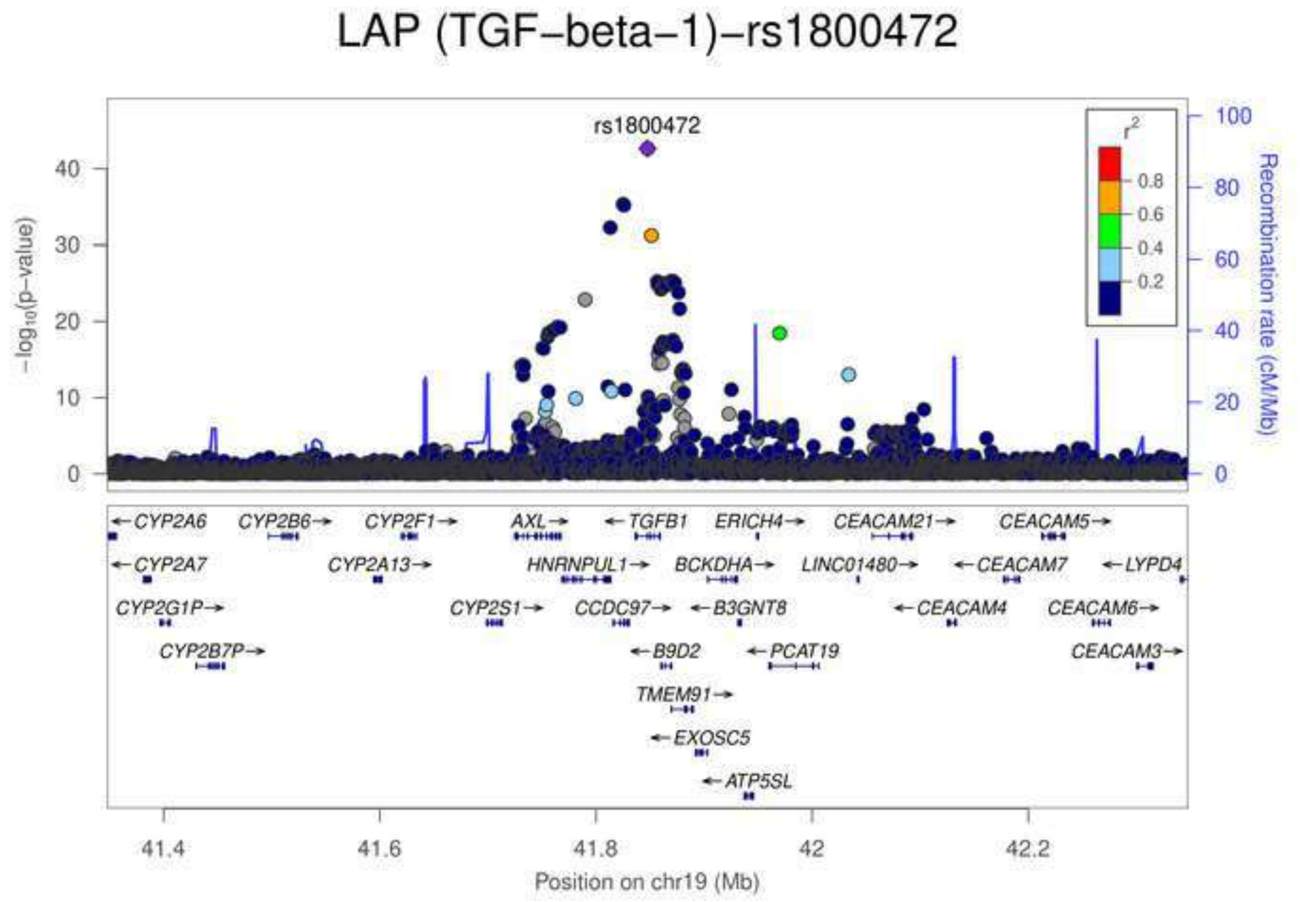
LAP (TGF−beta−1)−rs1800472
rs1800472
$-\log_{10}$(p−value)
Recombination rate (cM/Mb)
r^2
0.8
0.6
0.4
0.2
CYP2A6
CYP2B6
CYP2F1
AXL
TGFB1
ERICH4
CEACAM21
CEACAM5
CYP2A7
CYP2A13
HNRNPUL1
BCKDHA
LINC01480
CEACAM7
LYPD4
CYP2G1P
CYP2S1
CCDC97
B3GNT8
CEACAM4
CEACAM6
CYP2B7P
B9D2
PCAT19
CEACAM3
TMEM91
EXOSC5
ATP5SL
41.4
41.6
41.8
42
42.2
Position on chr19 (Mb)

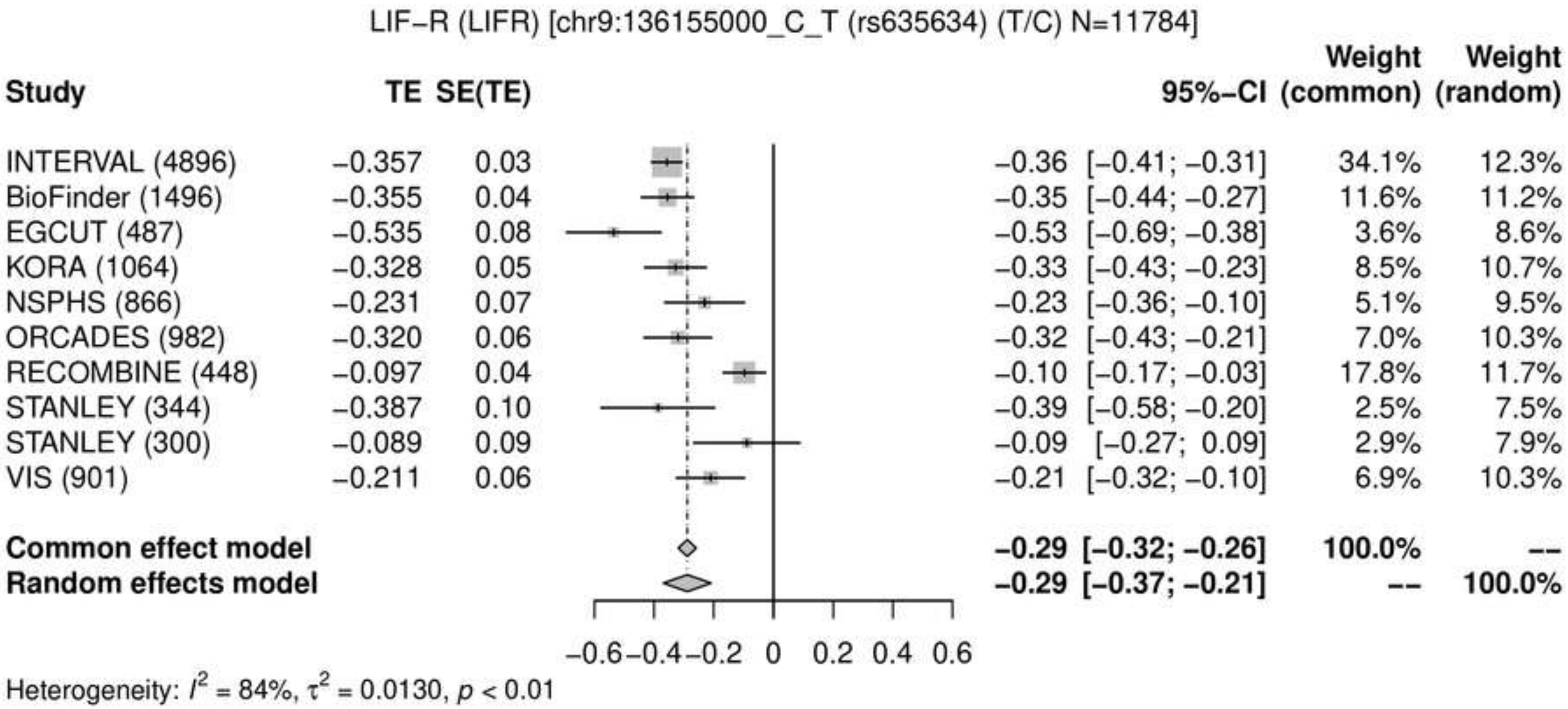
LIF−R (LIFR) [chr9:136155000_C_T (rs635634) (T/C) N=11784]
Study
TE
SE(TE)
95%−CI
Weight (common)
Weight (random)
INTERVAL (4896) −0.357 0.03 −0.36 [−0.41; −0.31] 34.1% 12.3%
BioFinder (1496) −0.355 0.04 −0.35 [−0.44; −0.27] 11.6% 11.2%
EGCUT (487) −0.535 0.08 −0.53 [−0.69; −0.38] 3.6% 8.6%
KORA (1064) −0.328 0.05 −0.33 [−0.43; −0.23] 8.5% 10.7%
NSPHS (866) −0.231 0.07 −0.23 [−0.36; −0.10] 5.1% 9.5%
ORCADES (982) −0.320 0.06 −0.32 [−0.43; −0.21] 7.0% 10.3%
RECOMBINE (448) −0.097 0.04 −0.10 [−0.17; −0.03] 17.8% 11.7%
STANLEY (344) −0.387 0.10 −0.39 [−0.58; −0.20] 2.5% 7.5%
STANLEY (300) −0.089 0.09 −0.09 [−0.27; 0.09] 2.9% 7.9%
VIS (901) −0.211 0.06 −0.21 [−0.32; −0.10] 6.9% 10.3%
Common effect model −0.29 [−0.32; −0.26] 100.0% --
Random effects model −0.29 [−0.37; −0.21] -- 100.0%
−0.6 −0.4 −0.2 0 0.2 0.4 0.6
Heterogeneity: I^2 = 84%, τ^2 = 0.0130, $p < 0.01$

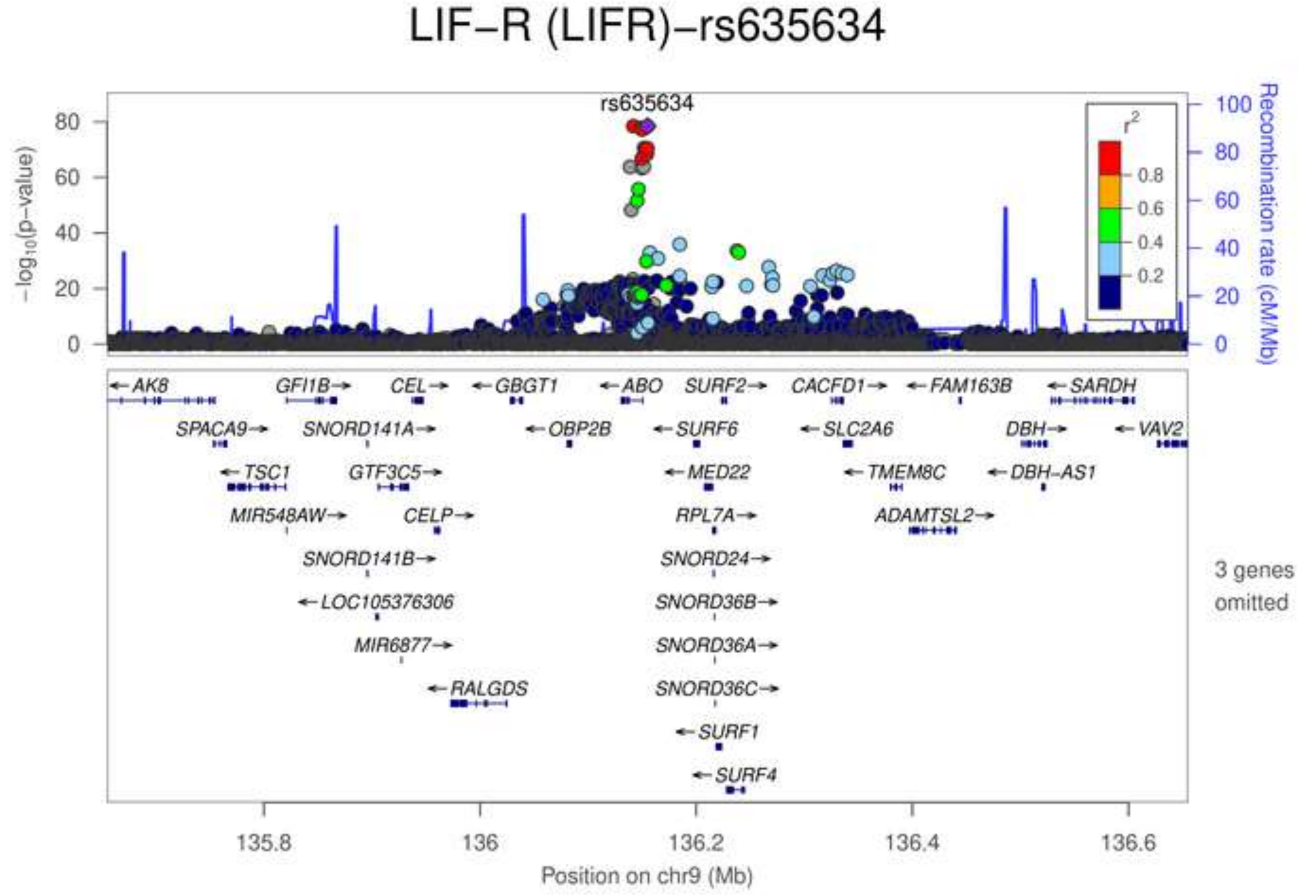
LIF−R (LIFR)−rs635634
rs635634
$-\log_{10}$(p-value)
80
60
40
20
0
Recombination rate (cM/Mb)
100
80
60
40
20
0
r^2
0.8
0.6
0.4
0.2
AK8
GFI1B
CEL
GBGT1
ABO
SURF2
CACFD1
FAM163B
SARDH
SPACA9
SNORD141A
OBP2B
SURF6
SLC2A6
DBH
VAV2
TSC1
GTF3C5
MED22
TMEM8C
DBH−AS1
MIR548AW
CELP
RPL7A
ADAMTSL2
SNORD141B
SNORD24
LOC105376306
SNORD36B
MIR6877
SNORD36A
RALGDS
SNORD36C
SURF1
SURF4
3 genes omitted
135.8
136
136.2
136.4
136.6
Position on chr9 (Mb)

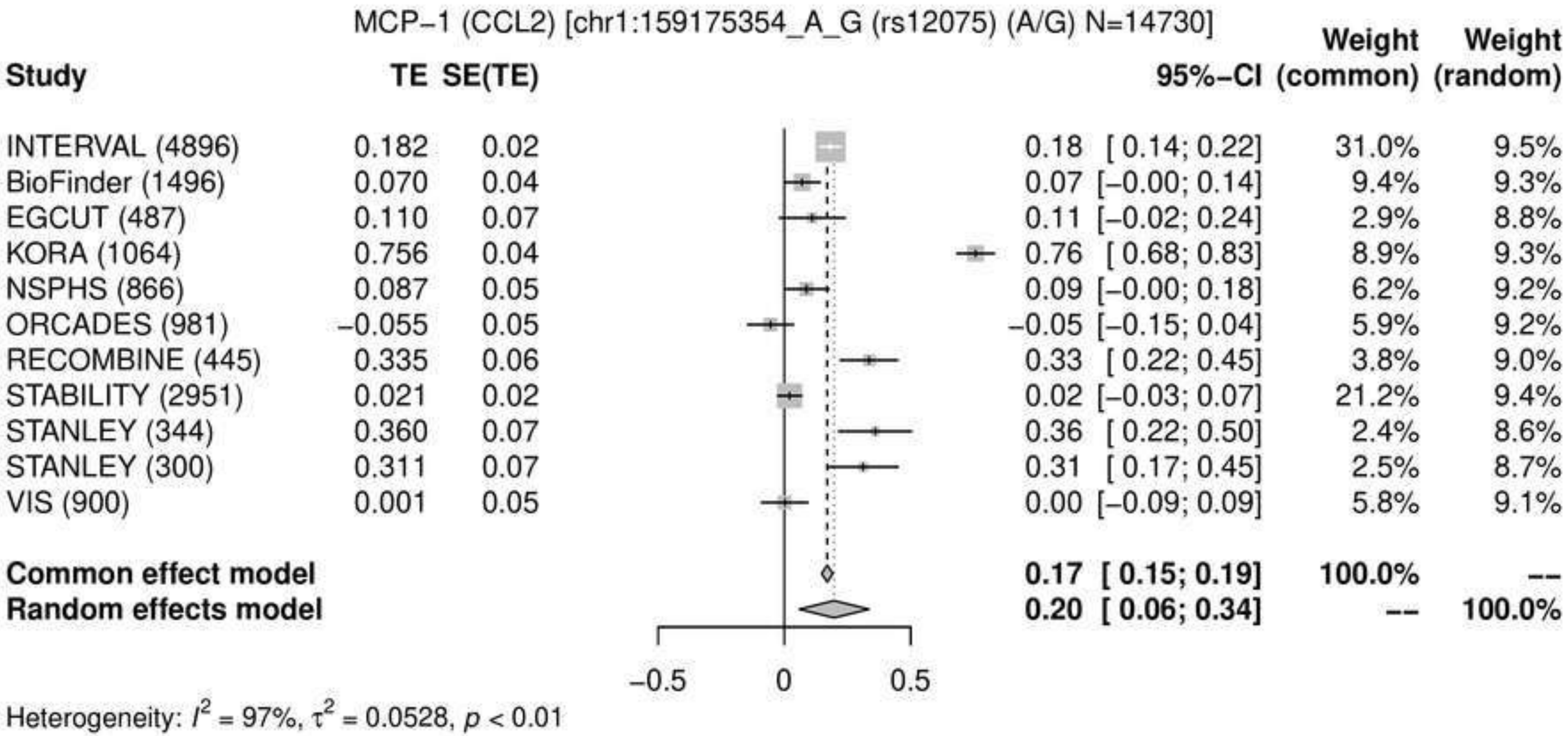
MCP−1 (CCL2) [chr1:159175354_A_G (rs12075) (A/G) N=14730]
Study TE SE(TE) 95%−CI Weight (common) Weight (random)
INTERVAL (4896) 0.182 0.02 0.18 [0.14; 0.22] 31.0% 9.5%
BioFinder (1496) 0.070 0.04 0.07 [−0.00; 0.14] 9.4% 9.3%
EGCUT (487) 0.110 0.07 0.11 [−0.02; 0.24] 2.9% 8.8%
KORA (1064) 0.756 0.04 0.76 [0.68; 0.83] 8.9% 9.3%
NSPHS (866) 0.087 0.05 0.09 [−0.00; 0.18] 6.2% 9.2%
ORCADES (981) −0.055 0.05 −0.05 [−0.15; 0.04] 5.9% 9.2%
RECOMBINE (445) 0.335 0.06 0.33 [0.22; 0.45] 3.8% 9.0%
STABILITY (2951) 0.021 0.02 0.02 [−0.03; 0.07] 21.2% 9.4%
STANLEY (344) 0.360 0.07 0.36 [0.22; 0.50] 2.4% 8.6%
STANLEY (300) 0.311 0.07 0.31 [0.17; 0.45] 2.5% 8.7%
VIS (900) 0.001 0.05 0.00 [−0.09; 0.09] 5.8% 9.1%
Common effect model 0.17 [0.15; 0.19] 100.0% --
Random effects model 0.20 [0.06; 0.34] -- 100.0%
−0.5
0
0.5
Heterogeneity: I^2 = 97%, τ^2 = 0.0528, p < 0.01

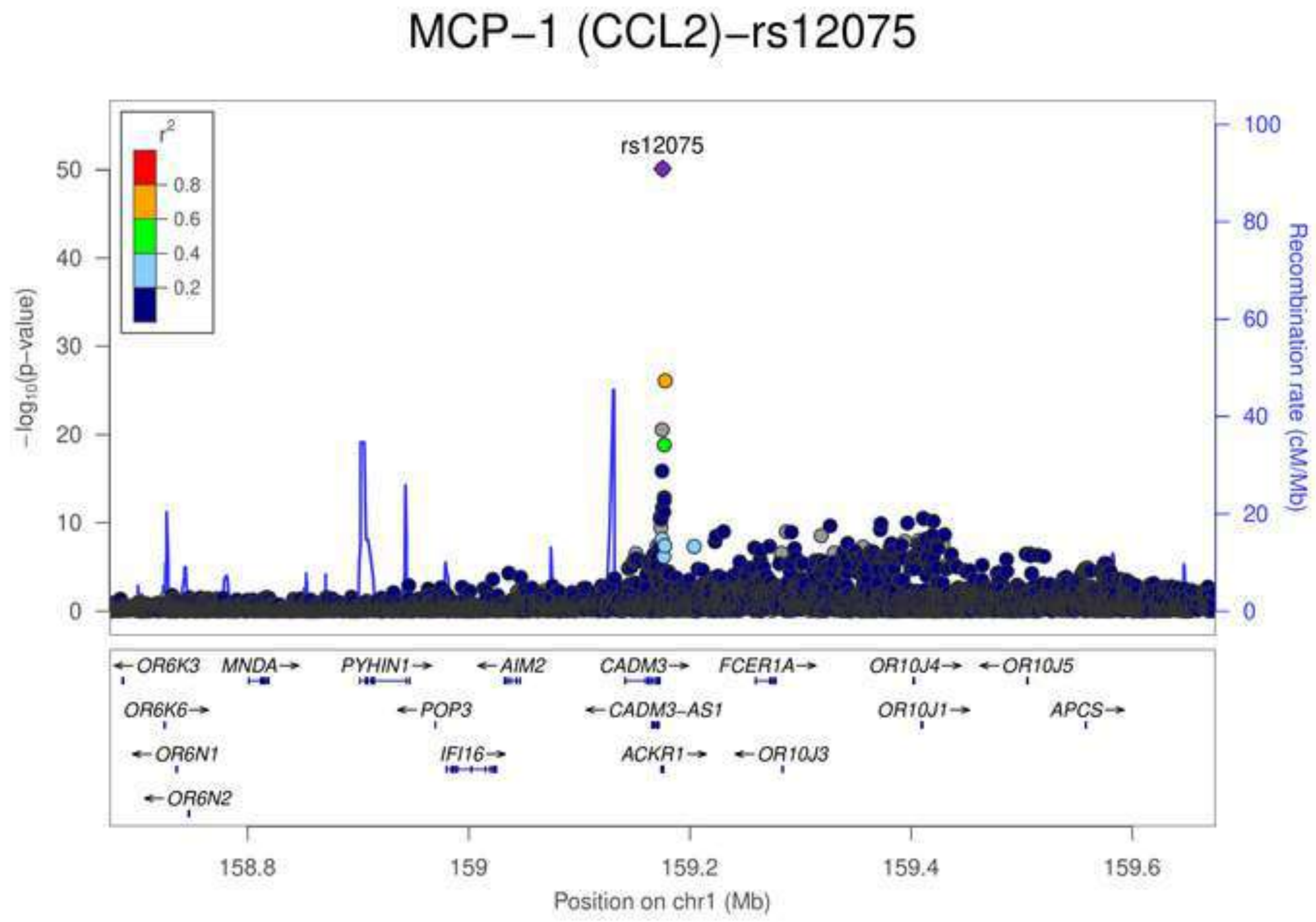
MCP−1 (CCL2)−rs12075
rs12075
r^2
0.8
0.6
0.4
0.2
−log10(p−value)
Recombination rate (cM/Mb)
0
10
20
30
40
50
0
20
40
60
80
100
←OR6K3 MNDA→ PYHIN1→ ←AIM2 CADM3→ FCER1A→ OR10J4→ ←OR10J5
OR6K6→ ←POP3 ←CADM3−AS1 OR10J1→ APCS→
←OR6N1 IFI16→ ACKR1→ ←OR10J3
←OR6N2
158.8
159
159.2
159.4
159.6
Position on chr1 (Mb)

MCP−1 (CCL2) [chr3:42906116_C_T (rs2228467) (T/C) N=14732]

Study	TE	SE(TE)	95%−CI	Weight (common)	Weight (random)
INTERVAL (4896)	−0.067	0.04	−0.07 [−0.15; 0.02]	28.7%	19.4%
BioFinder (1496)	−0.156	0.07	−0.16 [−0.29; −0.03]	11.8%	12.3%
EGCUT (487)	−0.189	0.12	−0.19 [−0.42; 0.05]	3.6%	5.1%
KORA (1064)	−0.271	0.08	−0.27 [−0.43; −0.11]	7.7%	9.3%
NSPHS (866)	−0.278	0.09	−0.28 [−0.46; −0.10]	6.0%	7.7%
ORCADES (981)	−0.243	0.09	−0.24 [−0.42; −0.06]	6.1%	7.8%
RECOMBINE (447)	−0.030	0.14	−0.03 [−0.30; 0.24]	2.7%	3.9%
STABILITY (2951)	−0.171	0.05	−0.17 [−0.27; −0.07]	18.8%	16.0%
STANLEY (344)	−0.266	0.14	−0.27 [−0.54; 0.01]	2.6%	3.9%
STANLEY (300)	−0.233	0.13	−0.23 [−0.48; 0.01]	3.3%	4.7%
VIS (900)	−0.257	0.08	−0.26 [−0.41; −0.10]	8.6%	9.9%
**Common effect model**			**−0.17 [−0.21; −0.12]**	**100.0%**	**--**
**Random effects model**			**−0.18 [−0.24; −0.13]**	**--**	**100.0%**

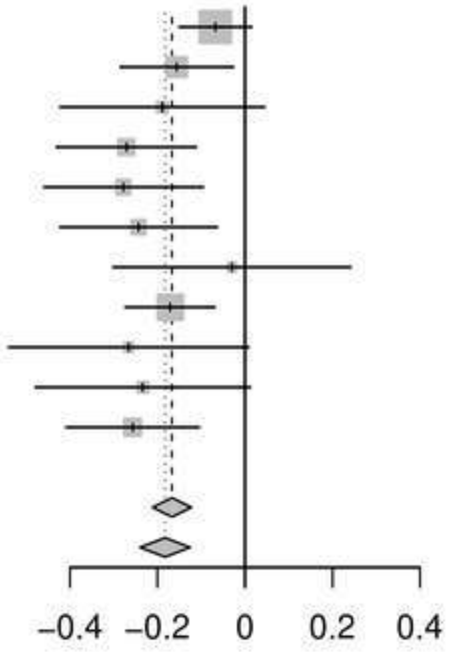

Heterogeneity: $I^2 = 19\%$, $\tau^2 = 0.0026$, $p = 0.26$

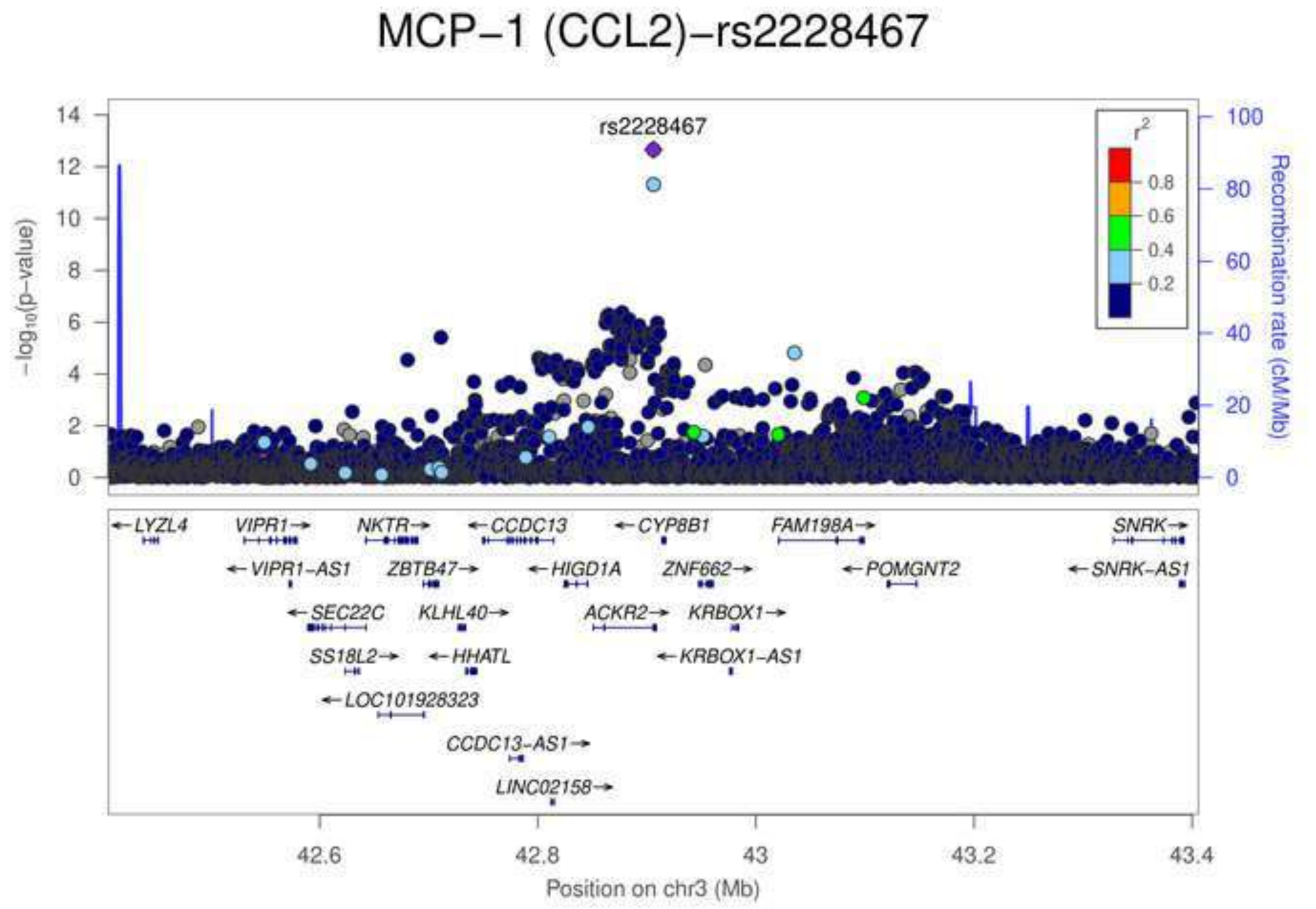

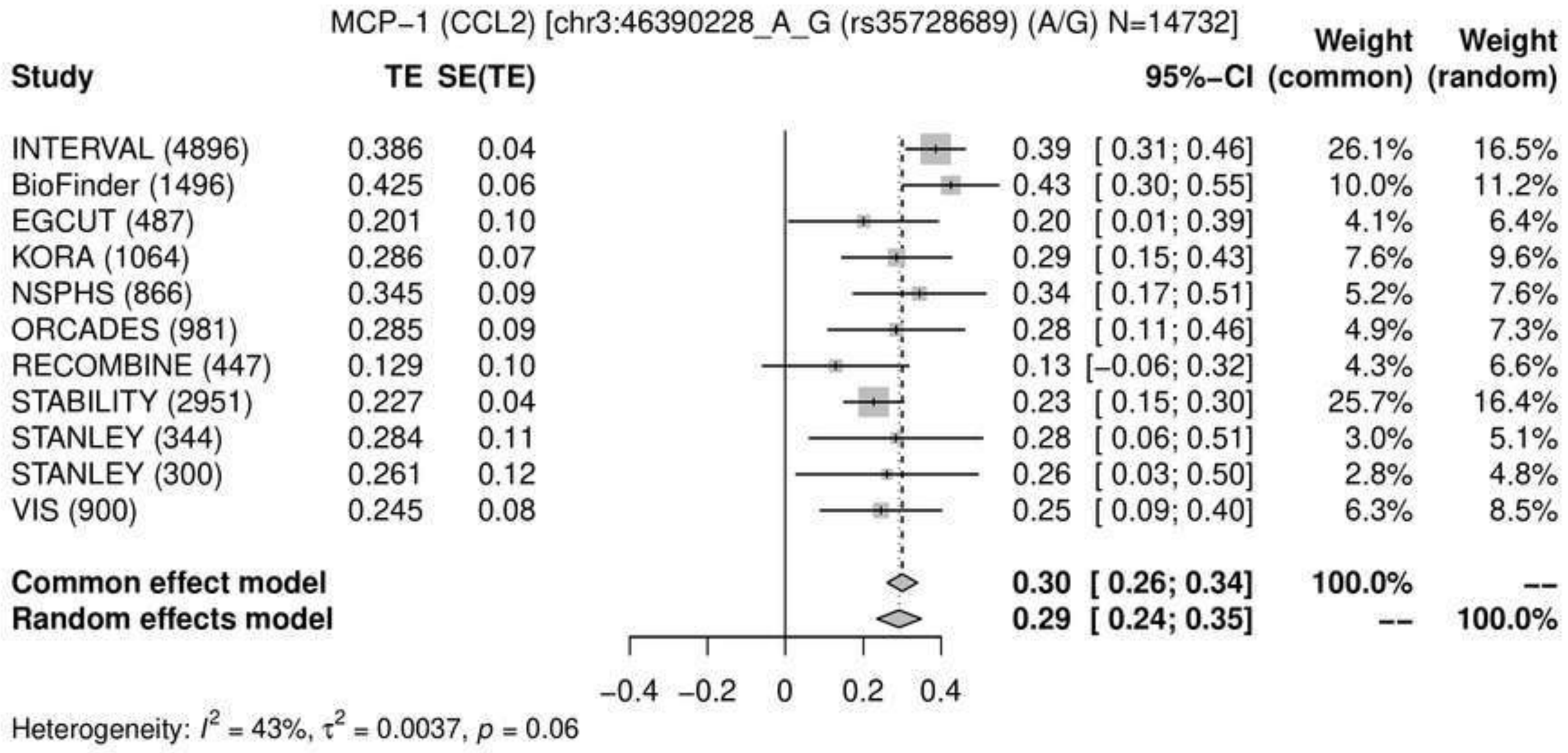
MCP−1 (CCL2) [chr3:46390228_A_G (rs35728689) (A/G) N=14732]
Study
TE
SE(TE)
95%−CI
Weight (common)
Weight (random)
INTERVAL (4896) 0.386 0.04 0.39 [0.31; 0.46] 26.1% 16.5%
BioFinder (1496) 0.425 0.06 0.43 [0.30; 0.55] 10.0% 11.2%
EGCUT (487) 0.201 0.10 0.20 [0.01; 0.39] 4.1% 6.4%
KORA (1064) 0.286 0.07 0.29 [0.15; 0.43] 7.6% 9.6%
NSPHS (866) 0.345 0.09 0.34 [0.17; 0.51] 5.2% 7.6%
ORCADES (981) 0.285 0.09 0.28 [0.11; 0.46] 4.9% 7.3%
RECOMBINE (447) 0.129 0.10 0.13 [−0.06; 0.32] 4.3% 6.6%
STABILITY (2951) 0.227 0.04 0.23 [0.15; 0.30] 25.7% 16.4%
STANLEY (344) 0.284 0.11 0.28 [0.06; 0.51] 3.0% 5.1%
STANLEY (300) 0.261 0.12 0.26 [0.03; 0.50] 2.8% 4.8%
VIS (900) 0.245 0.08 0.25 [0.09; 0.40] 6.3% 8.5%
Common effect model 0.30 [0.26; 0.34] 100.0% --
Random effects model 0.29 [0.24; 0.35] -- 100.0%
−0.4 −0.2 0 0.2 0.4
Heterogeneity: I^2 = 43%, τ^2 = 0.0037, p = 0.06

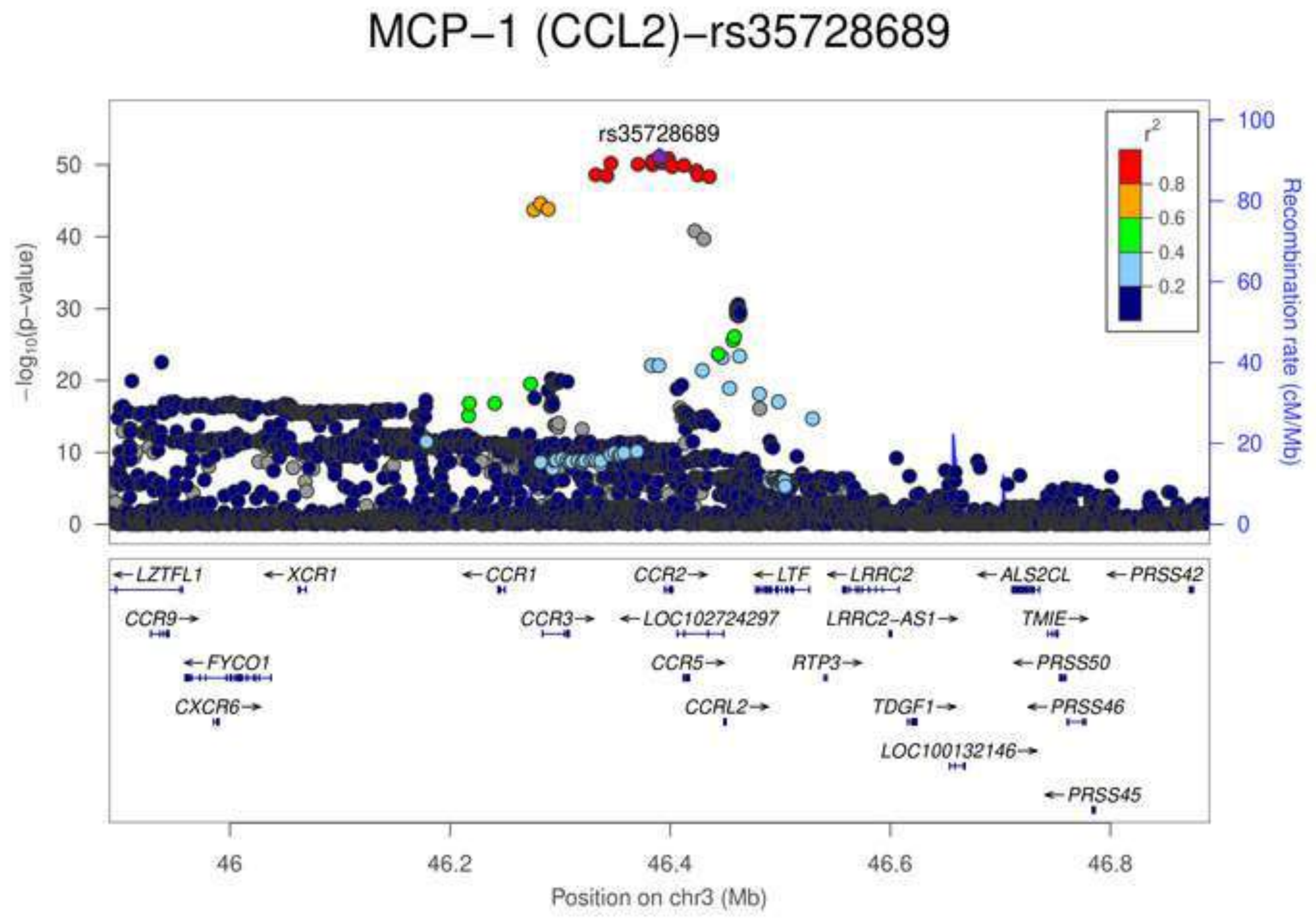
MCP−1 (CCL2)−rs35728689
rs35728689
$-\log_{10}$(p-value)
Recombination rate (cM/Mb)
r^2
0.8
0.6
0.4
0.2
0
10
20
30
40
50
60
80
100
LZTFL1
XCR1
CCR1
CCR2
LTF
LRRC2
ALS2CL
PRSS42
CCR9
CCR3
LOC102724297
LRRC2-AS1
TMIE
FYCO1
CCR5
RTP3
PRSS50
CXCR6
CCRL2
TDGF1
PRSS46
LOC100132146
PRSS45
46
46.2
46.4
46.6
46.8
Position on chr3 (Mb)

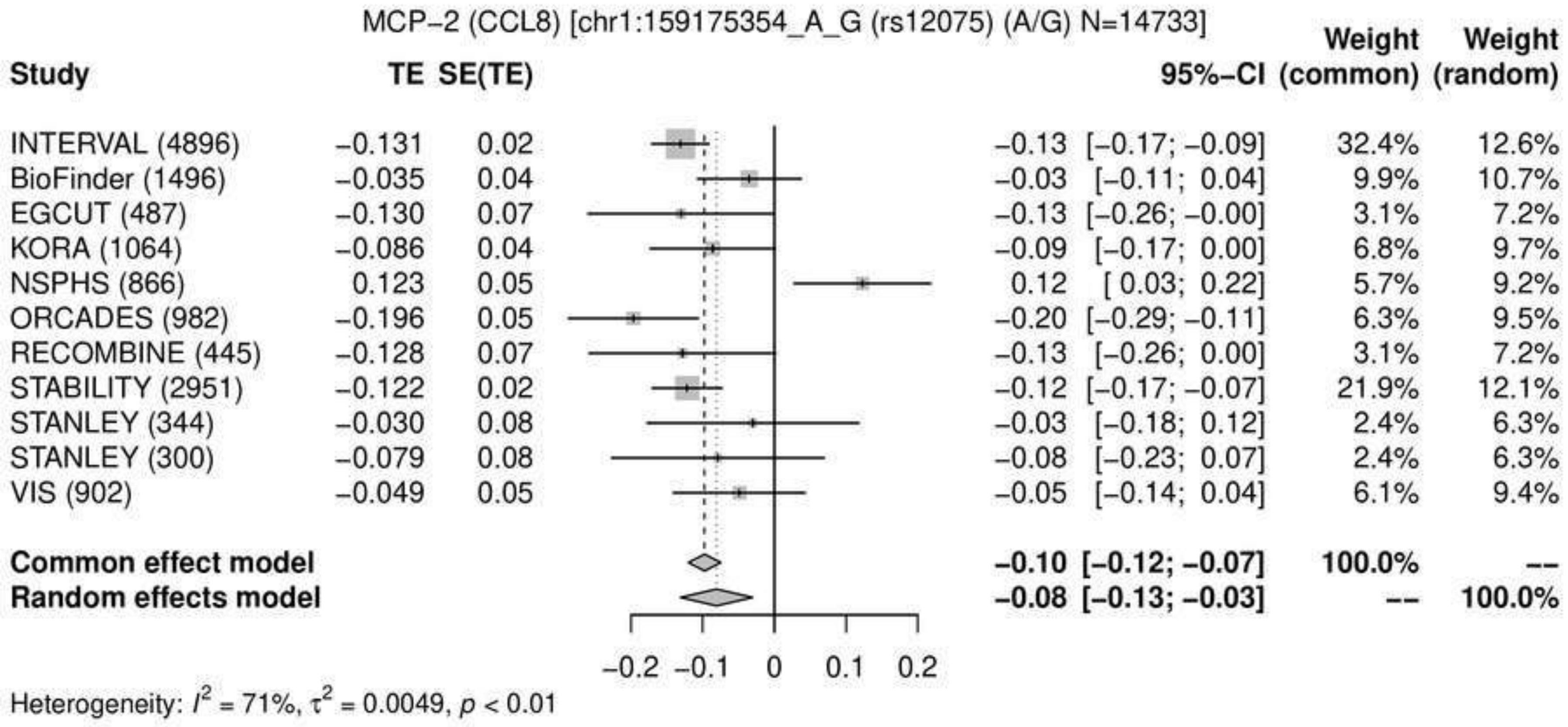
MCP−2 (CCL8) [chr1:159175354_A_G (rs12075) (A/G) N=14733]
Study
TE
SE(TE)
95%−CI
Weight (common)
Weight (random)
INTERVAL (4896) −0.131 0.02 −0.13 [−0.17; −0.09] 32.4% 12.6%
BioFinder (1496) −0.035 0.04 −0.03 [−0.11; 0.04] 9.9% 10.7%
EGCUT (487) −0.130 0.07 −0.13 [−0.26; −0.00] 3.1% 7.2%
KORA (1064) −0.086 0.04 −0.09 [−0.17; 0.00] 6.8% 9.7%
NSPHS (866) 0.123 0.05 0.12 [0.03; 0.22] 5.7% 9.2%
ORCADES (982) −0.196 0.05 −0.20 [−0.29; −0.11] 6.3% 9.5%
RECOMBINE (445) −0.128 0.07 −0.13 [−0.26; 0.00] 3.1% 7.2%
STABILITY (2951) −0.122 0.02 −0.12 [−0.17; −0.07] 21.9% 12.1%
STANLEY (344) −0.030 0.08 −0.03 [−0.18; 0.12] 2.4% 6.3%
STANLEY (300) −0.079 0.08 −0.08 [−0.23; 0.07] 2.4% 6.3%
VIS (902) −0.049 0.05 −0.05 [−0.14; 0.04] 6.1% 9.4%
Common effect model −0.10 [−0.12; −0.07] 100.0% --
Random effects model −0.08 [−0.13; −0.03] -- 100.0%
−0.2 −0.1 0 0.1 0.2
Heterogeneity: I^2 = 71%, τ^2 = 0.0049, p < 0.01

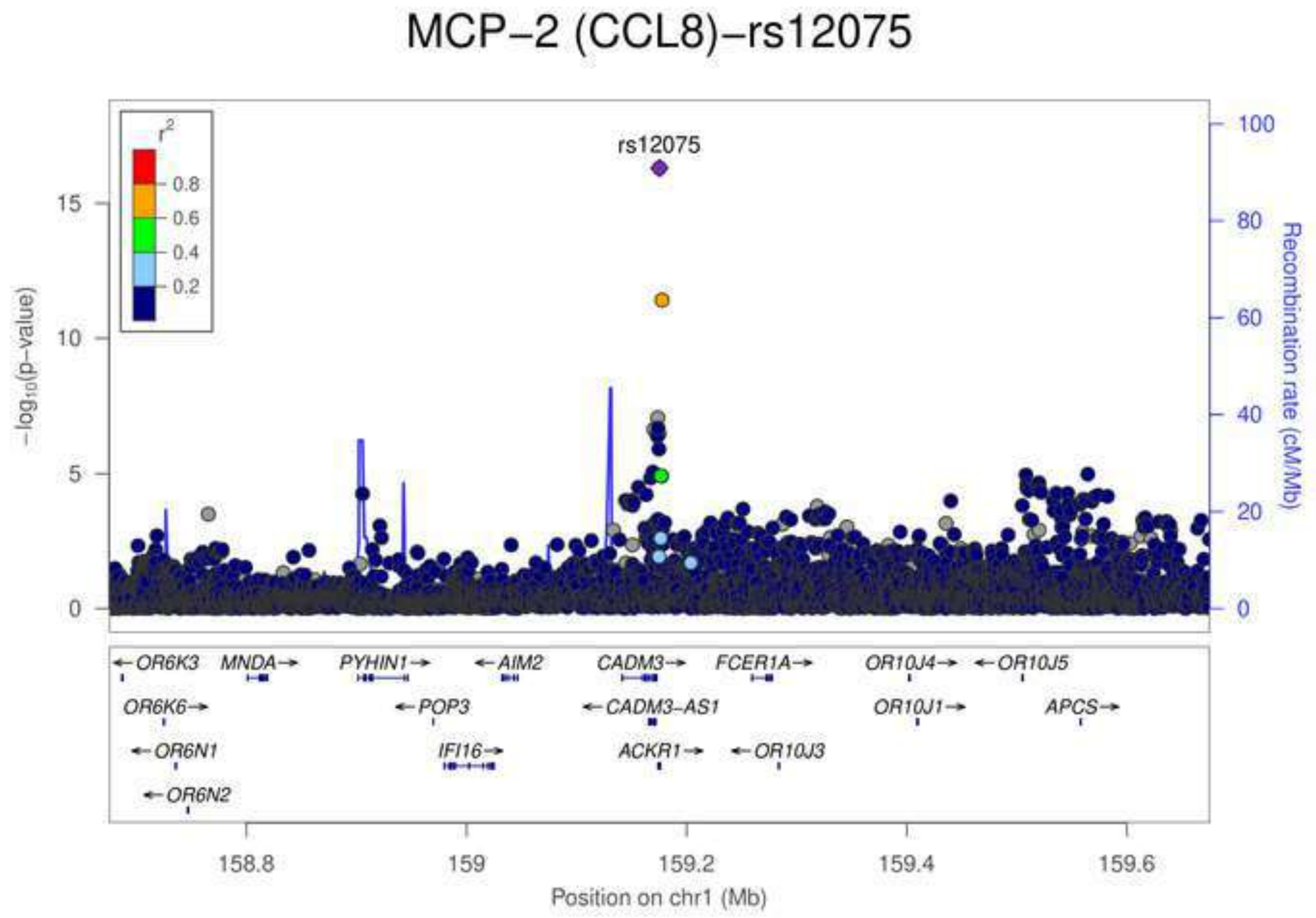
MCP−2 (CCL8)−rs12075
rs12075
r^2
0.8
0.6
0.4
0.2
$-\log_{10}$(p−value)
Recombination rate (cM/Mb)
OR6K3
MNDA
PYHIN1
AIM2
CADM3
FCER1A
OR10J4
OR10J5
OR6K6
POP3
CADM3−AS1
OR10J1
APCS
OR6N1
IFI16
ACKR1
OR10J3
OR6N2
158.8
159
159.2
159.4
159.6
Position on chr1 (Mb)

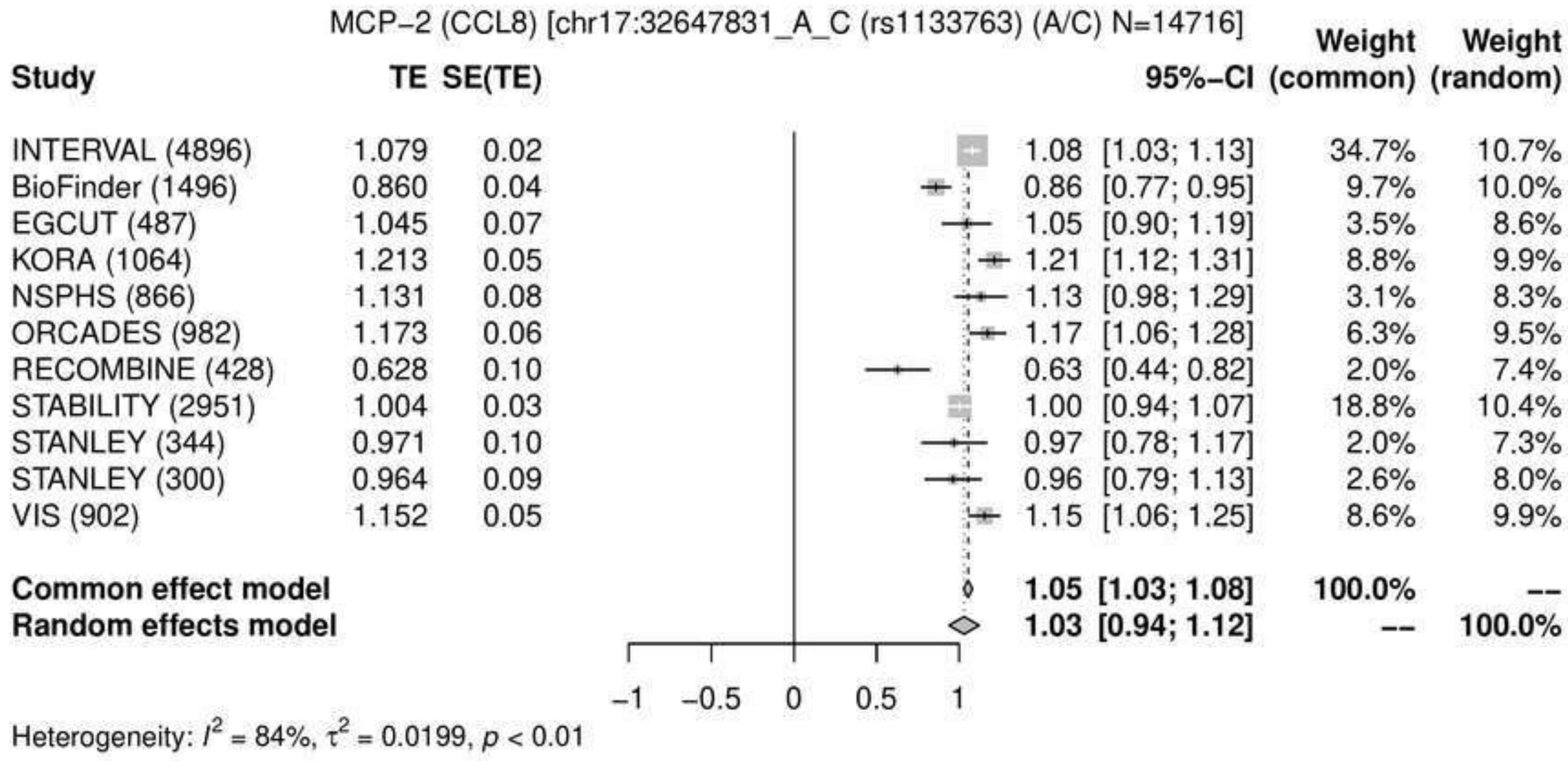
MCP−2 (CCL8) [chr17:32647831_A_C (rs1133763) (A/C) N=14716]
Study | TE | SE(TE) | 95%−CI | Weight (common) | Weight (random)
INTERVAL (4896) | 1.079 | 0.02 | 1.08 [1.03; 1.13] | 34.7% | 10.7%
BioFinder (1496) | 0.860 | 0.04 | 0.86 [0.77; 0.95] | 9.7% | 10.0%
EGCUT (487) | 1.045 | 0.07 | 1.05 [0.90; 1.19] | 3.5% | 8.6%
KORA (1064) | 1.213 | 0.05 | 1.21 [1.12; 1.31] | 8.8% | 9.9%
NSPHS (866) | 1.131 | 0.08 | 1.13 [0.98; 1.29] | 3.1% | 8.3%
ORCADES (982) | 1.173 | 0.06 | 1.17 [1.06; 1.28] | 6.3% | 9.5%
RECOMBINE (428) | 0.628 | 0.10 | 0.63 [0.44; 0.82] | 2.0% | 7.4%
STABILITY (2951) | 1.004 | 0.03 | 1.00 [0.94; 1.07] | 18.8% | 10.4%
STANLEY (344) | 0.971 | 0.10 | 0.97 [0.78; 1.17] | 2.0% | 7.3%
STANLEY (300) | 0.964 | 0.09 | 0.96 [0.79; 1.13] | 2.6% | 8.0%
VIS (902) | 1.152 | 0.05 | 1.15 [1.06; 1.25] | 8.6% | 9.9%
Common effect model | 1.05 [1.03; 1.08] | 100.0% | --
Random effects model | 1.03 [0.94; 1.12] | -- | 100.0%
−1 −0.5 0 0.5 1
Heterogeneity: I^2 = 84%, τ^2 = 0.0199, p < 0.01

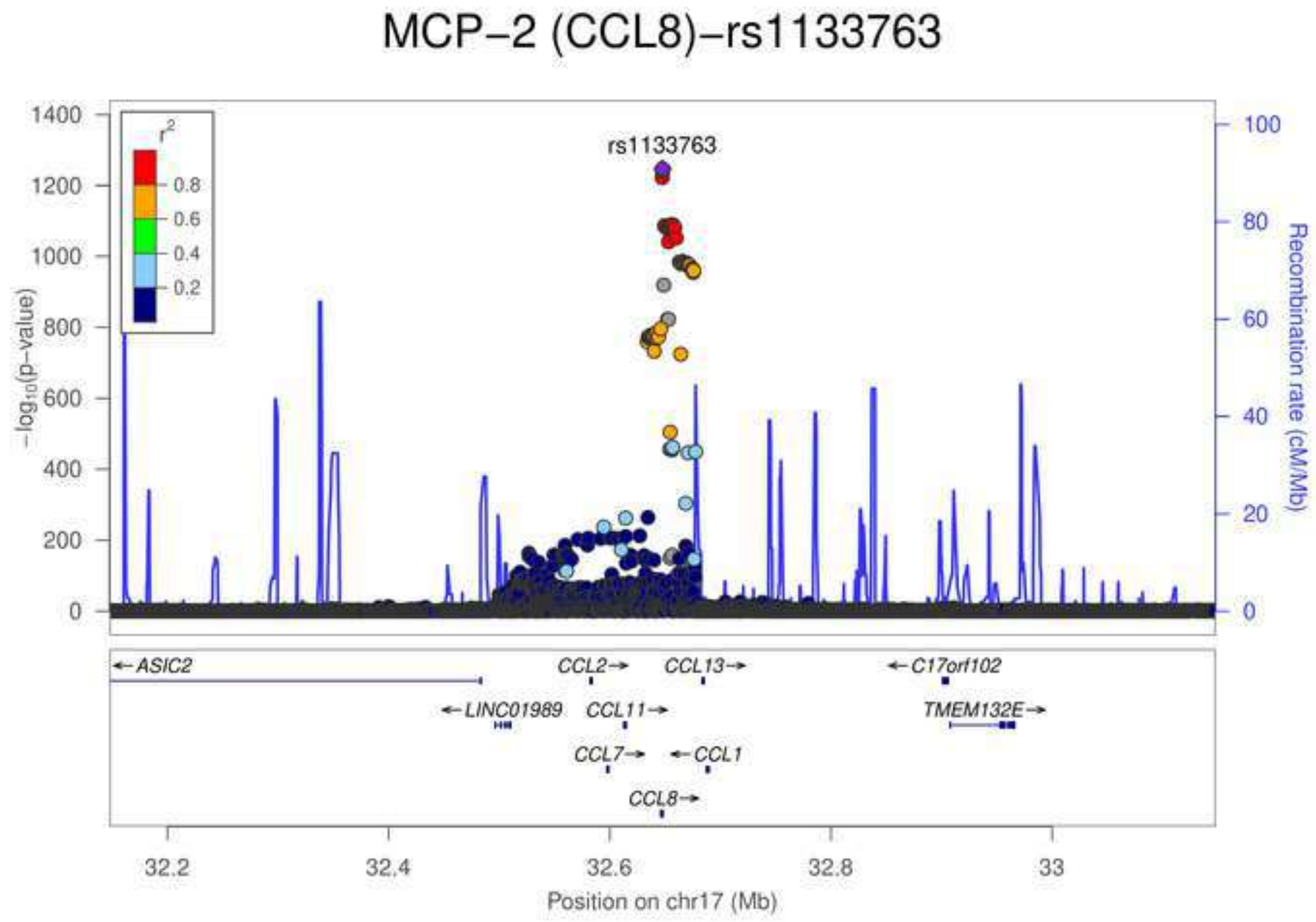
MCP−2 (CCL8)−rs1133763
rs1133763
r^2
0.8
0.6
0.4
0.2
$-\log_{10}$(p−value)
Recombination rate (cM/Mb)
ASIC2
CCL2
CCL13
C17orf102
LINC01989
CCL11
TMEM132E
CCL7
CCL1
CCL8
32.2 32.4 32.6 32.8 33
Position on chr17 (Mb)

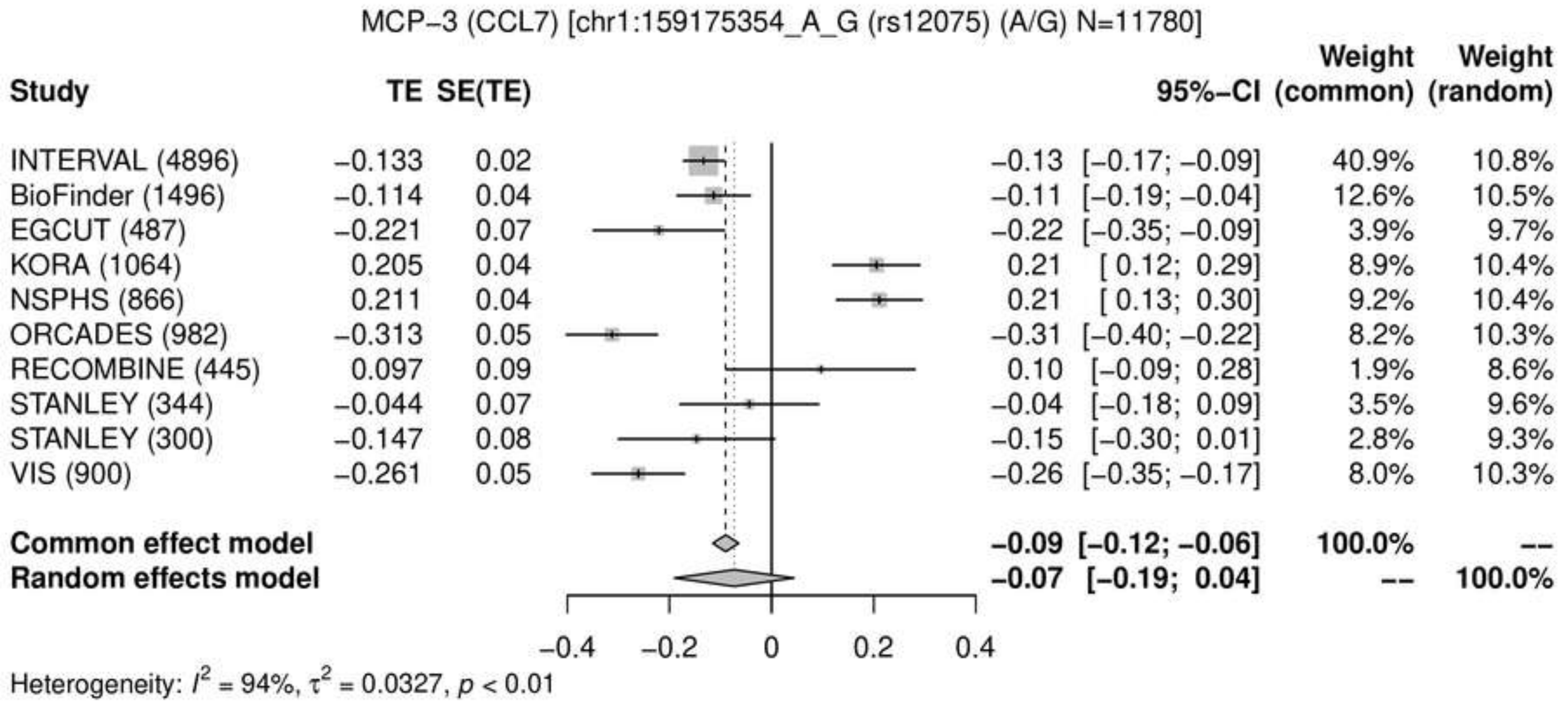
MCP-3 (CCL7) [chr1:159175354_A_G (rs12075) (A/G) N=11780]
Study
TE
SE(TE)
95%-CI
Weight (common)
Weight (random)
INTERVAL (4896) −0.133 0.02 −0.13 [−0.17; −0.09] 40.9% 10.8%
BioFinder (1496) −0.114 0.04 −0.11 [−0.19; −0.04] 12.6% 10.5%
EGCUT (487) −0.221 0.07 −0.22 [−0.35; −0.09] 3.9% 9.7%
KORA (1064) 0.205 0.04 0.21 [0.12; 0.29] 8.9% 10.4%
NSPHS (866) 0.211 0.04 0.21 [0.13; 0.30] 9.2% 10.4%
ORCADES (982) −0.313 0.05 −0.31 [−0.40; −0.22] 8.2% 10.3%
RECOMBINE (445) 0.097 0.09 0.10 [−0.09; 0.28] 1.9% 8.6%
STANLEY (344) −0.044 0.07 −0.04 [−0.18; 0.09] 3.5% 9.6%
STANLEY (300) −0.147 0.08 −0.15 [−0.30; 0.01] 2.8% 9.3%
VIS (900) −0.261 0.05 −0.26 [−0.35; −0.17] 8.0% 10.3%
Common effect model −0.09 [−0.12; −0.06] 100.0% --
Random effects model −0.07 [−0.19; 0.04] -- 100.0%
−0.4 −0.2 0 0.2 0.4
Heterogeneity: I^2 = 94%, τ^2 = 0.0327, p < 0.01

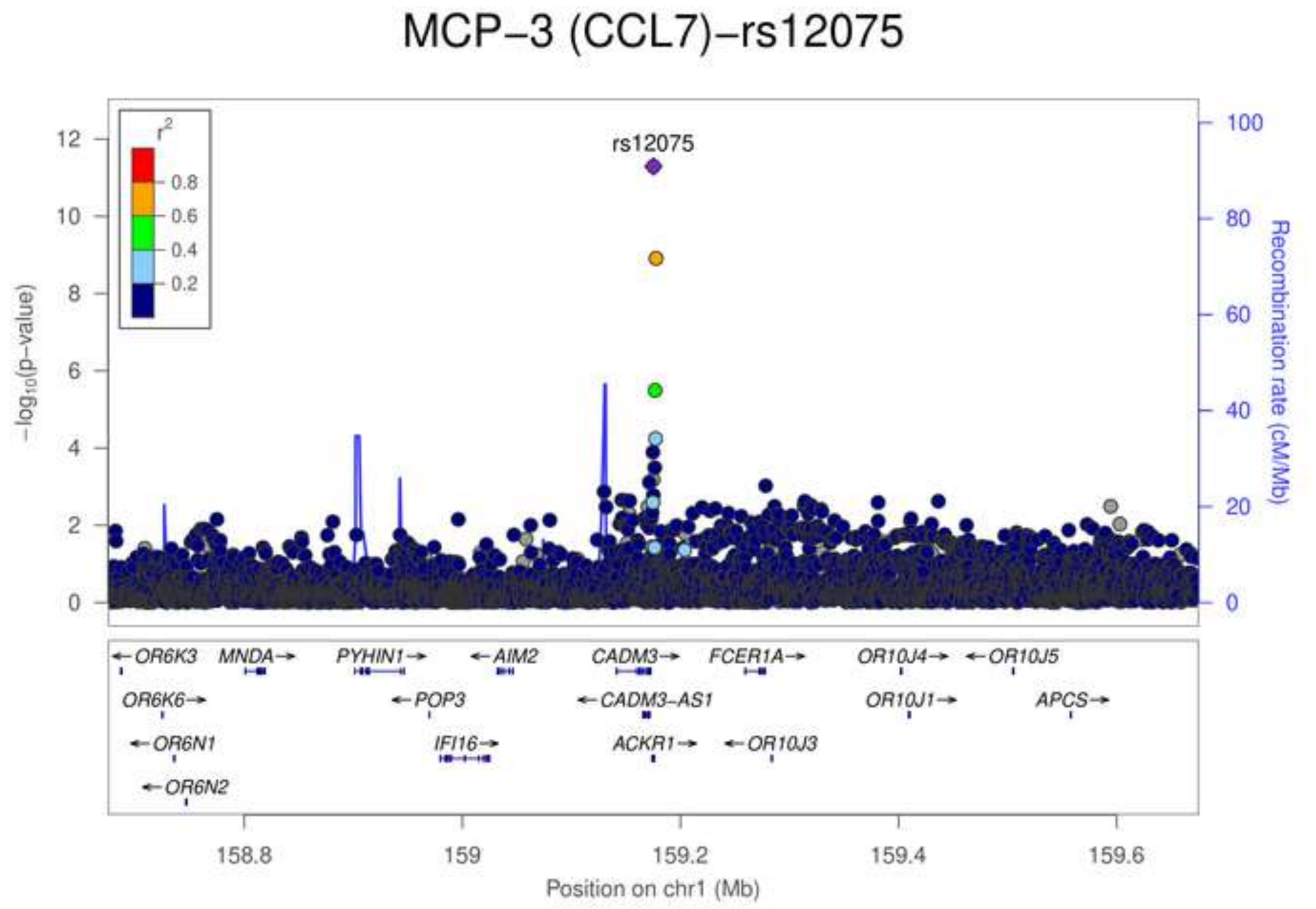
MCP−3 (CCL7)−rs12075
rs12075
r^2
0.8
0.6
0.4
0.2
−log10(p−value)
Recombination rate (cM/Mb)
OR6K3
MNDA
PYHIN1
AIM2
CADM3
FCER1A
OR10J4
OR10J5
OR6K6
POP3
CADM3−AS1
OR10J1
APCS
OR6N1
IFI16
ACKR1
OR10J3
OR6N2
158.8
159
159.2
159.4
159.6
Position on chr1 (Mb)

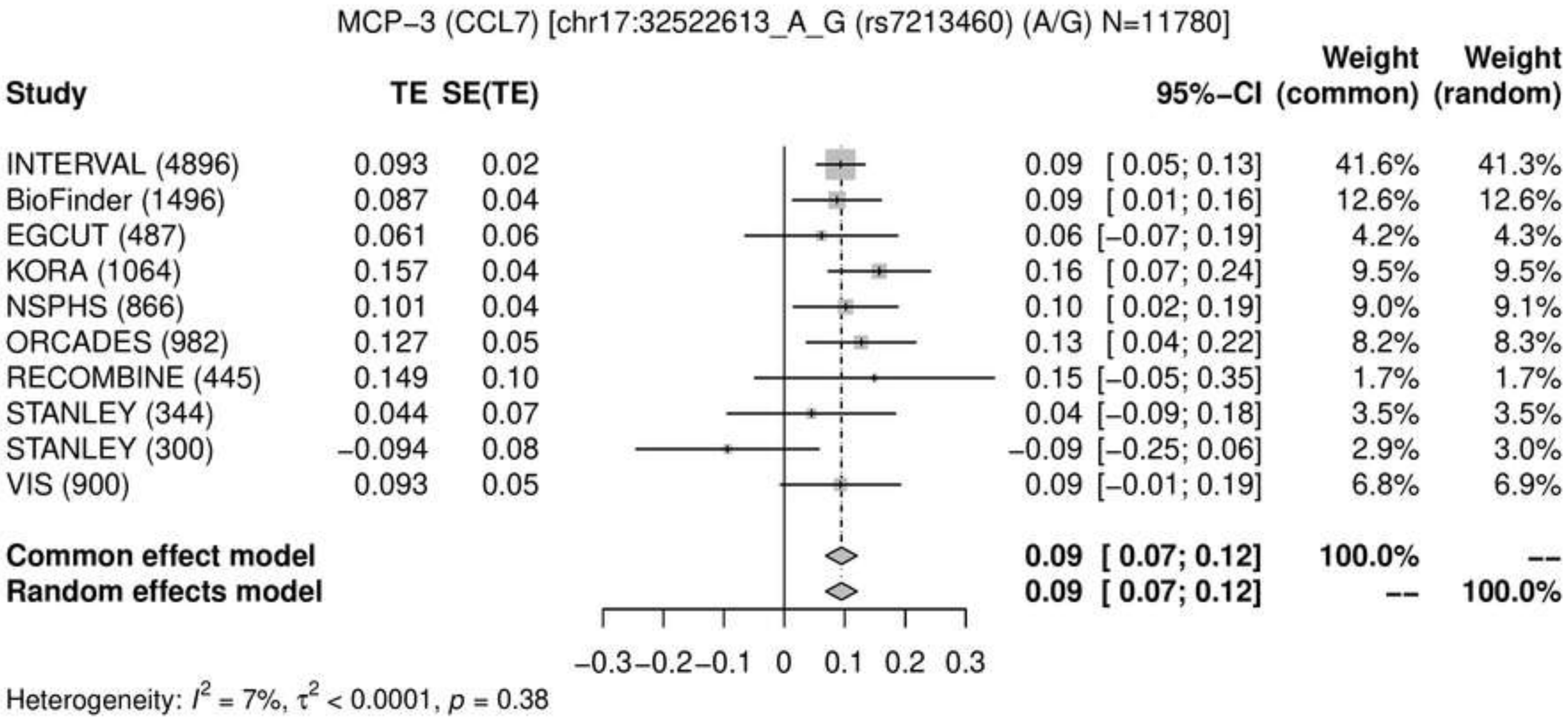
MCP−3 (CCL7) [chr17:32522613_A_G (rs7213460) (A/G) N=11780]
Study
TE
SE(TE)
95%−CI
Weight (common)
Weight (random)
INTERVAL (4896) 0.093 0.02 0.09 [0.05; 0.13] 41.6% 41.3%
BioFinder (1496) 0.087 0.04 0.09 [0.01; 0.16] 12.6% 12.6%
EGCUT (487) 0.061 0.06 0.06 [−0.07; 0.19] 4.2% 4.3%
KORA (1064) 0.157 0.04 0.16 [0.07; 0.24] 9.5% 9.5%
NSPHS (866) 0.101 0.04 0.10 [0.02; 0.19] 9.0% 9.1%
ORCADES (982) 0.127 0.05 0.13 [0.04; 0.22] 8.2% 8.3%
RECOMBINE (445) 0.149 0.10 0.15 [−0.05; 0.35] 1.7% 1.7%
STANLEY (344) 0.044 0.07 0.04 [−0.09; 0.18] 3.5% 3.5%
STANLEY (300) −0.094 0.08 −0.09 [−0.25; 0.06] 2.9% 3.0%
VIS (900) 0.093 0.05 0.09 [−0.01; 0.19] 6.8% 6.9%
Common effect model 0.09 [0.07; 0.12] 100.0% --
Random effects model 0.09 [0.07; 0.12] -- 100.0%
−0.3 −0.2 −0.1 0 0.1 0.2 0.3
Heterogeneity: I² = 7%, τ² < 0.0001, p = 0.38
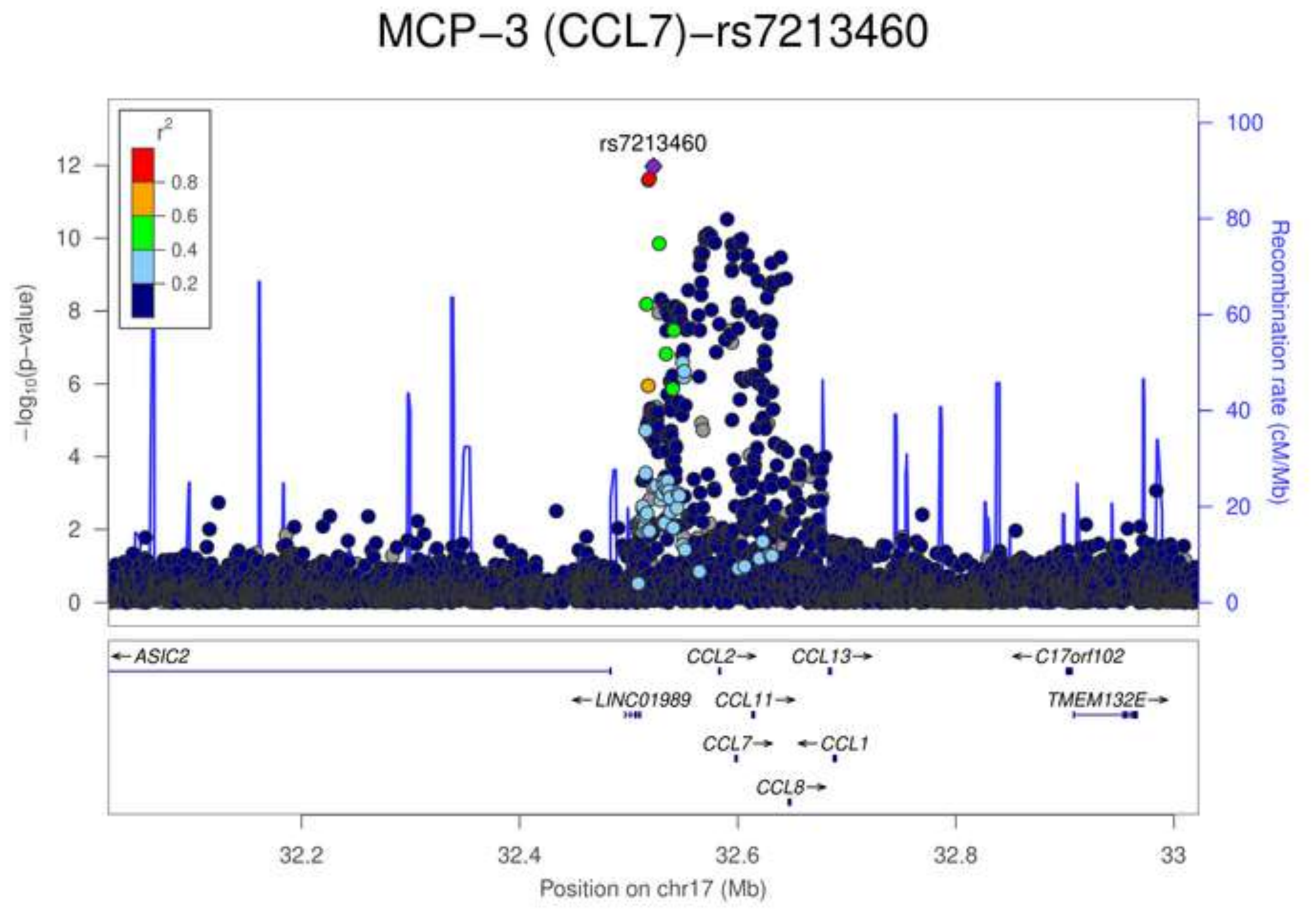
MCP−3 (CCL7)−rs7213460
rs7213460
r²
0.8
0.6
0.4
0.2
−log10(p−value)
Recombination rate (cM/Mb)
ASIC2
CCL2
CCL13
C17orf102
LINC01989
CCL11
TMEM132E
CCL7
CCL1
CCL8
32.2
32.4
32.6
32.8
33
Position on chr17 (Mb)

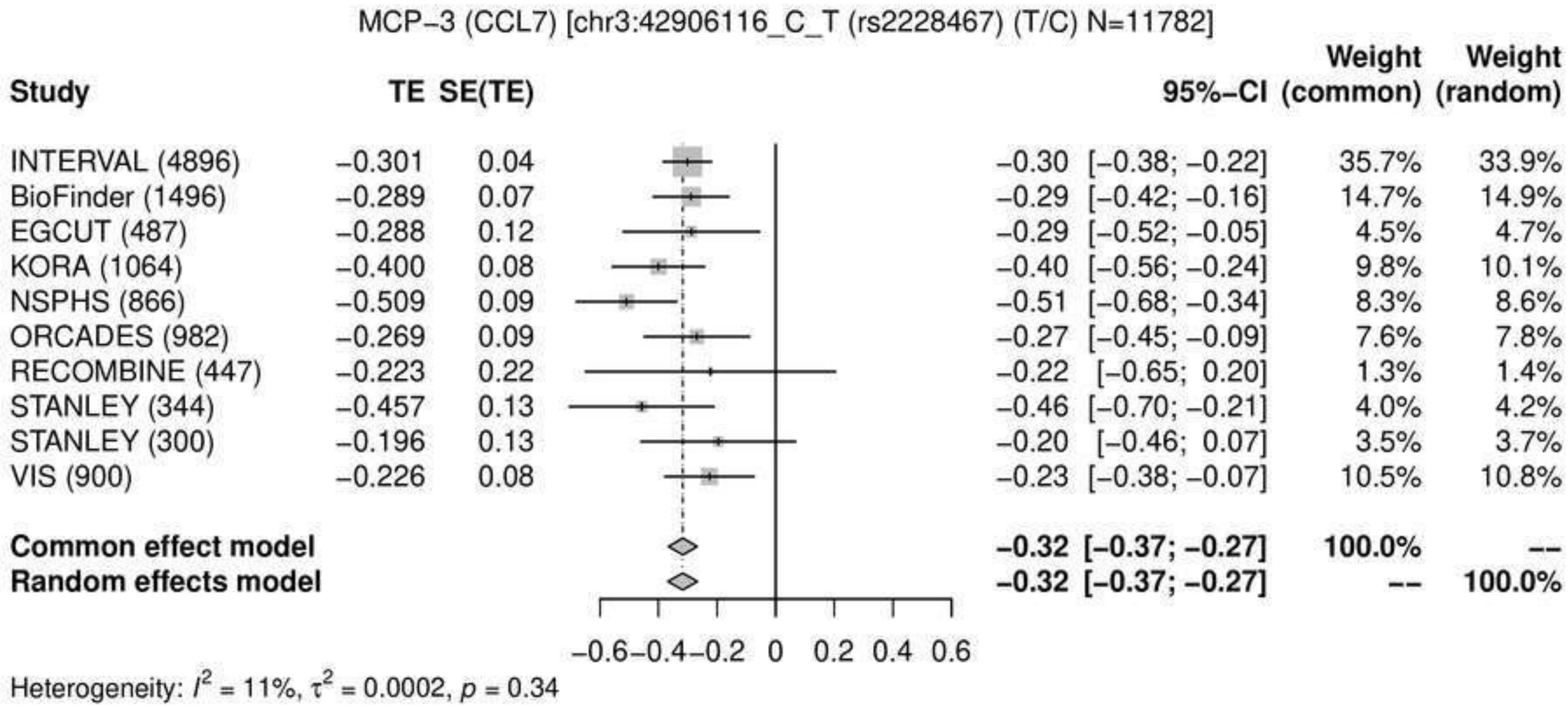
MCP−3 (CCL7) [chr3:42906116_C_T (rs2228467) (T/C) N=11782]
Study
TE SE(TE)
95%−CI
Weight (common)
Weight (random)
INTERVAL (4896) −0.301 0.04 −0.30 [−0.38; −0.22] 35.7% 33.9%
BioFinder (1496) −0.289 0.07 −0.29 [−0.42; −0.16] 14.7% 14.9%
EGCUT (487) −0.288 0.12 −0.29 [−0.52; −0.05] 4.5% 4.7%
KORA (1064) −0.400 0.08 −0.40 [−0.56; −0.24] 9.8% 10.1%
NSPHS (866) −0.509 0.09 −0.51 [−0.68; −0.34] 8.3% 8.6%
ORCADES (982) −0.269 0.09 −0.27 [−0.45; −0.09] 7.6% 7.8%
RECOMBINE (447) −0.223 0.22 −0.22 [−0.65; 0.20] 1.3% 1.4%
STANLEY (344) −0.457 0.13 −0.46 [−0.70; −0.21] 4.0% 4.2%
STANLEY (300) −0.196 0.13 −0.20 [−0.46; 0.07] 3.5% 3.7%
VIS (900) −0.226 0.08 −0.23 [−0.38; −0.07] 10.5% 10.8%
Common effect model −0.32 [−0.37; −0.27] 100.0% --
Random effects model −0.32 [−0.37; −0.27] -- 100.0%
−0.6 −0.4 −0.2 0 0.2 0.4 0.6
Heterogeneity: I^2 = 11%, τ^2 = 0.0002, p = 0.34

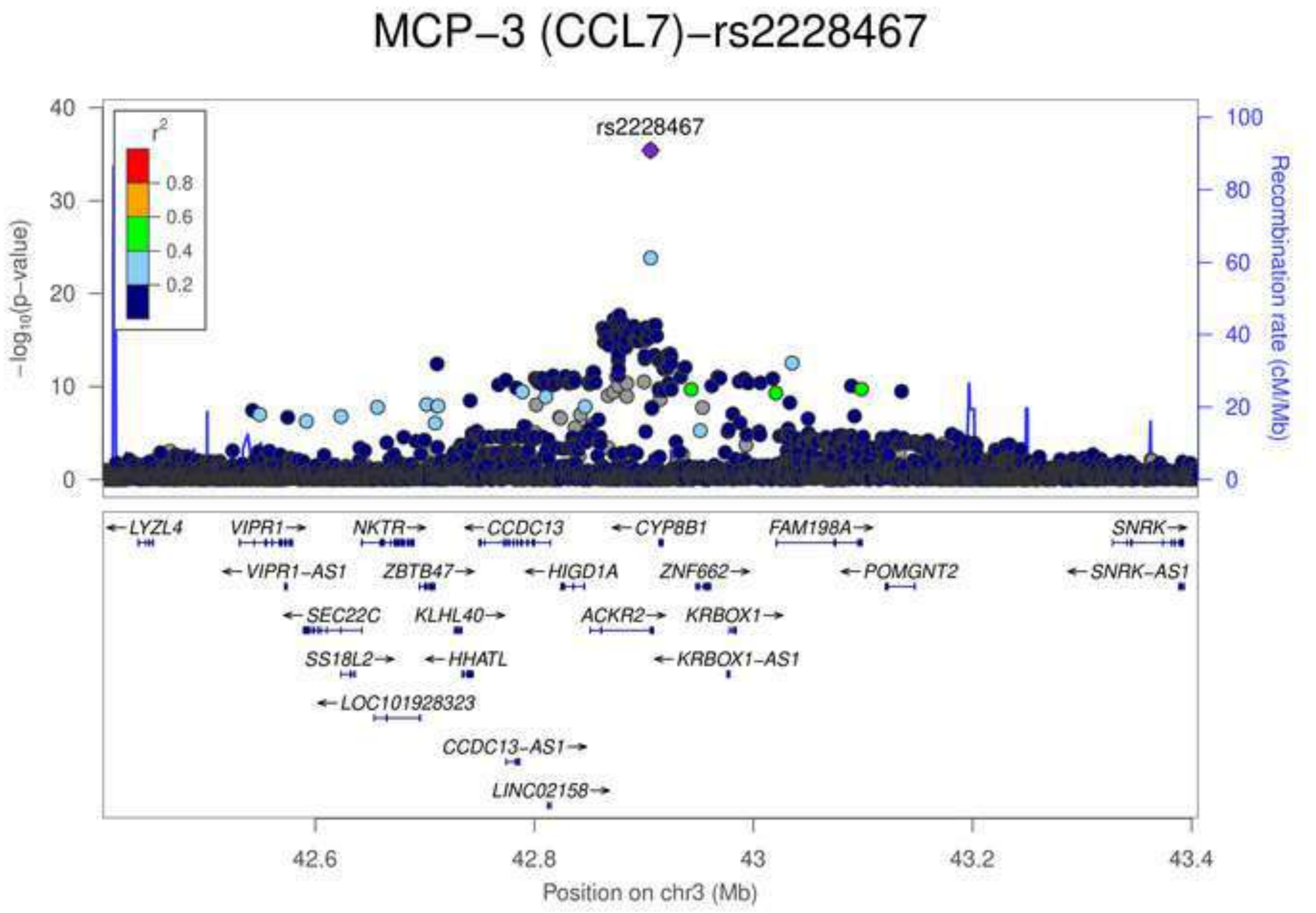
MCP−3 (CCL7)−rs2228467
rs2228467
r^2
0.8
0.6
0.4
0.2
$-\log_{10}(p\text{-value})$
Recombination rate (cM/Mb)
LYZL4
VIPR1
NKTR
CCDC13
CYP8B1
FAM198A
SNRK
VIPR1-AS1
ZBTB47
HIGD1A
ZNF662
POMGNT2
SNRK-AS1
SEC22C
KLHL40
ACKR2
KRBOX1
SS18L2
HHATL
KRBOX1-AS1
LOC101928323
CCDC13-AS1
LINC02158
42.6
42.8
43
43.2
43.4
Position on chr3 (Mb)

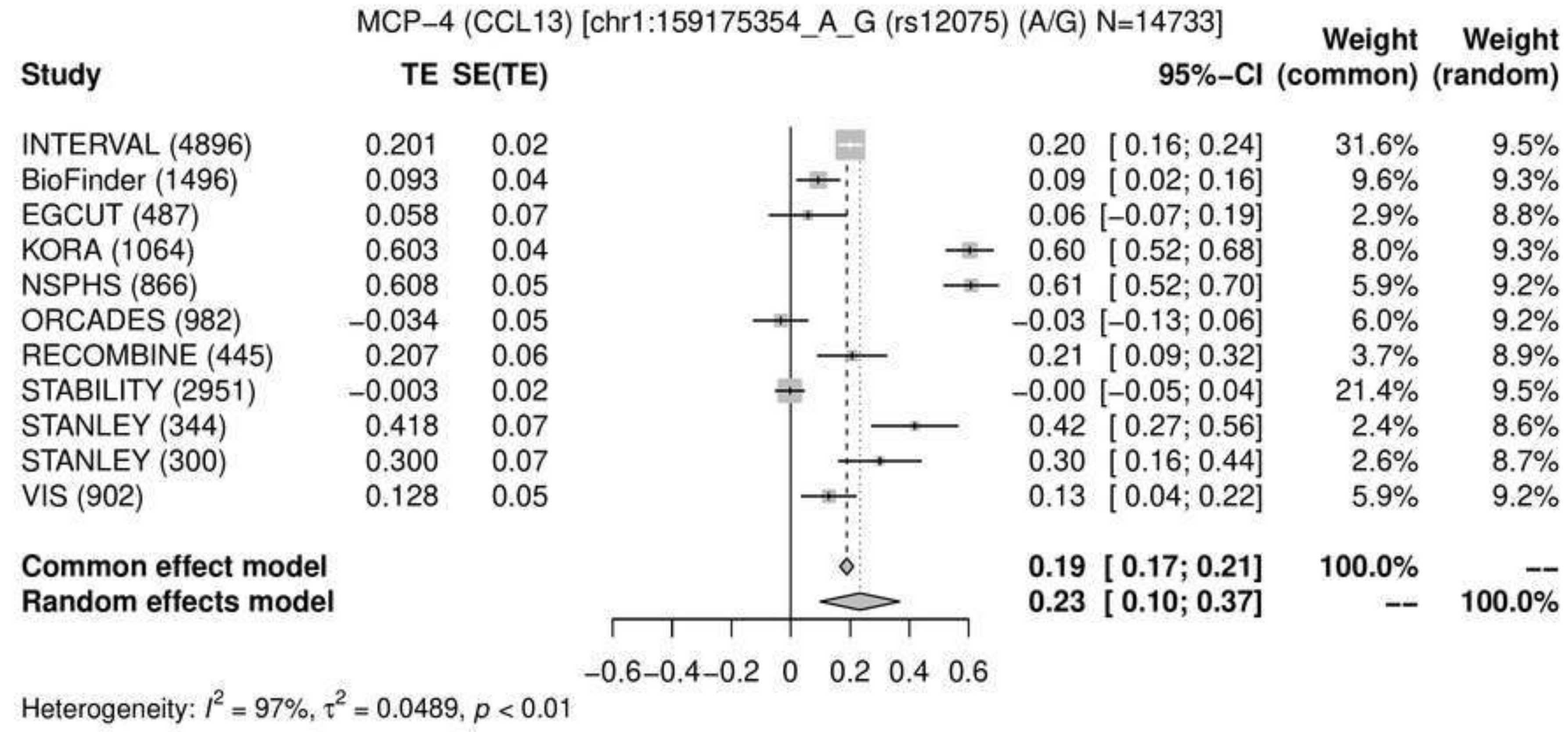
MCP−4 (CCL13) [chr1:159175354_A_G (rs12075) (A/G) N=14733]
Study
TE
SE(TE)
95%−CI
Weight (common)
Weight (random)
INTERVAL (4896) 0.201 0.02 0.20 [0.16; 0.24] 31.6% 9.5%
BioFinder (1496) 0.093 0.04 0.09 [0.02; 0.16] 9.6% 9.3%
EGCUT (487) 0.058 0.07 0.06 [−0.07; 0.19] 2.9% 8.8%
KORA (1064) 0.603 0.04 0.60 [0.52; 0.68] 8.0% 9.3%
NSPHS (866) 0.608 0.05 0.61 [0.52; 0.70] 5.9% 9.2%
ORCADES (982) −0.034 0.05 −0.03 [−0.13; 0.06] 6.0% 9.2%
RECOMBINE (445) 0.207 0.06 0.21 [0.09; 0.32] 3.7% 8.9%
STABILITY (2951) −0.003 0.02 −0.00 [−0.05; 0.04] 21.4% 9.5%
STANLEY (344) 0.418 0.07 0.42 [0.27; 0.56] 2.4% 8.6%
STANLEY (300) 0.300 0.07 0.30 [0.16; 0.44] 2.6% 8.7%
VIS (902) 0.128 0.05 0.13 [0.04; 0.22] 5.9% 9.2%
Common effect model 0.19 [0.17; 0.21] 100.0% --
Random effects model 0.23 [0.10; 0.37] -- 100.0%
−0.6 −0.4 −0.2 0 0.2 0.4 0.6
Heterogeneity: I^2 = 97%, τ^2 = 0.0489, p < 0.01

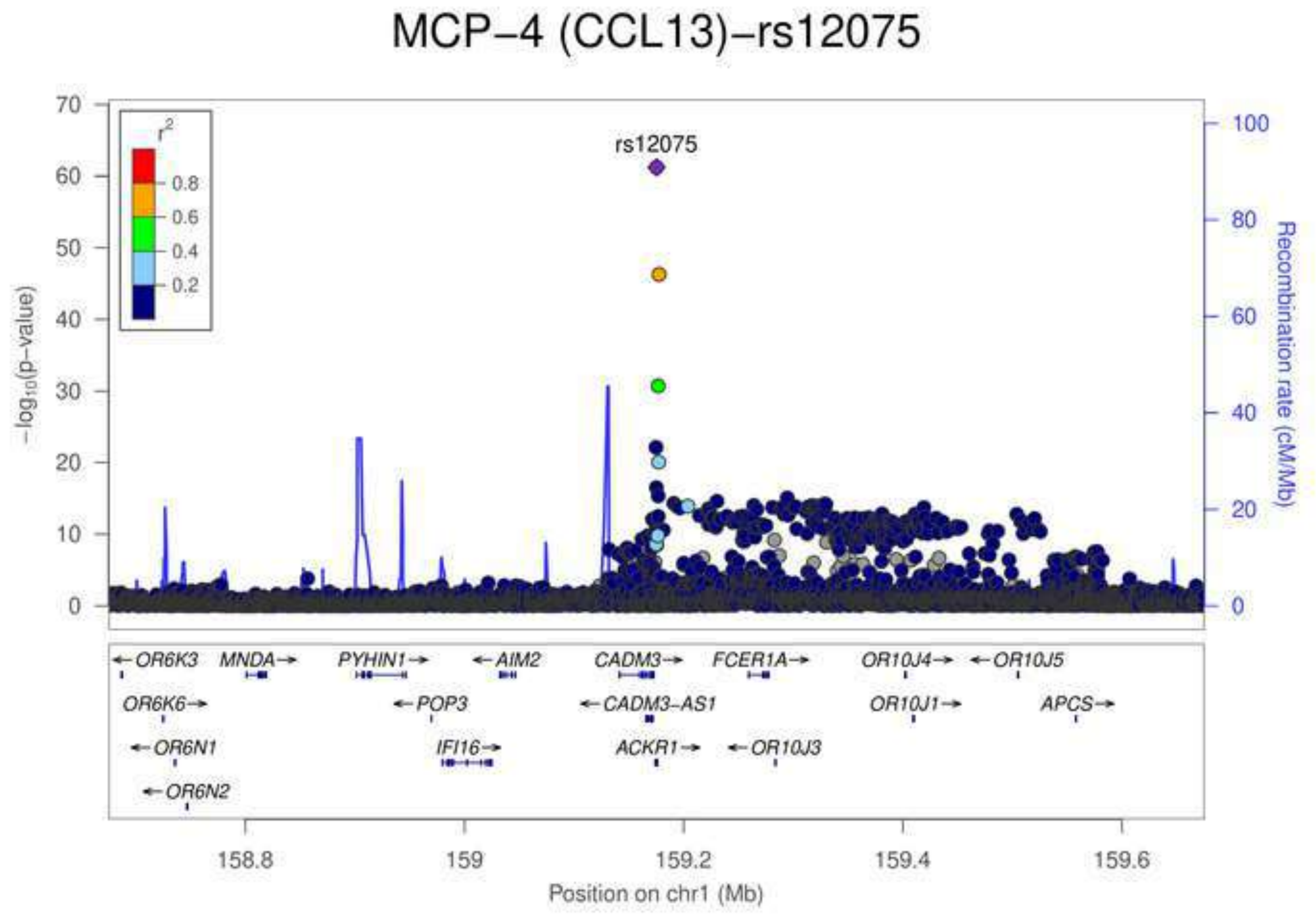
MCP−4 (CCL13)−rs12075
rs12075
r^2
0.8
0.6
0.4
0.2
$-\log_{10}$(p−value)
Recombination rate (cM/Mb)
0 10 20 30 40 50 60 70
0 20 40 60 80 100
OR6K3
MNDA
PYHIN1
AIM2
CADM3
FCER1A
OR10J4
OR10J5
OR6K6
POP3
CADM3−AS1
OR10J1
APCS
OR6N1
IFI16
ACKR1
OR10J3
OR6N2
158.8 159 159.2 159.4 159.6
Position on chr1 (Mb)

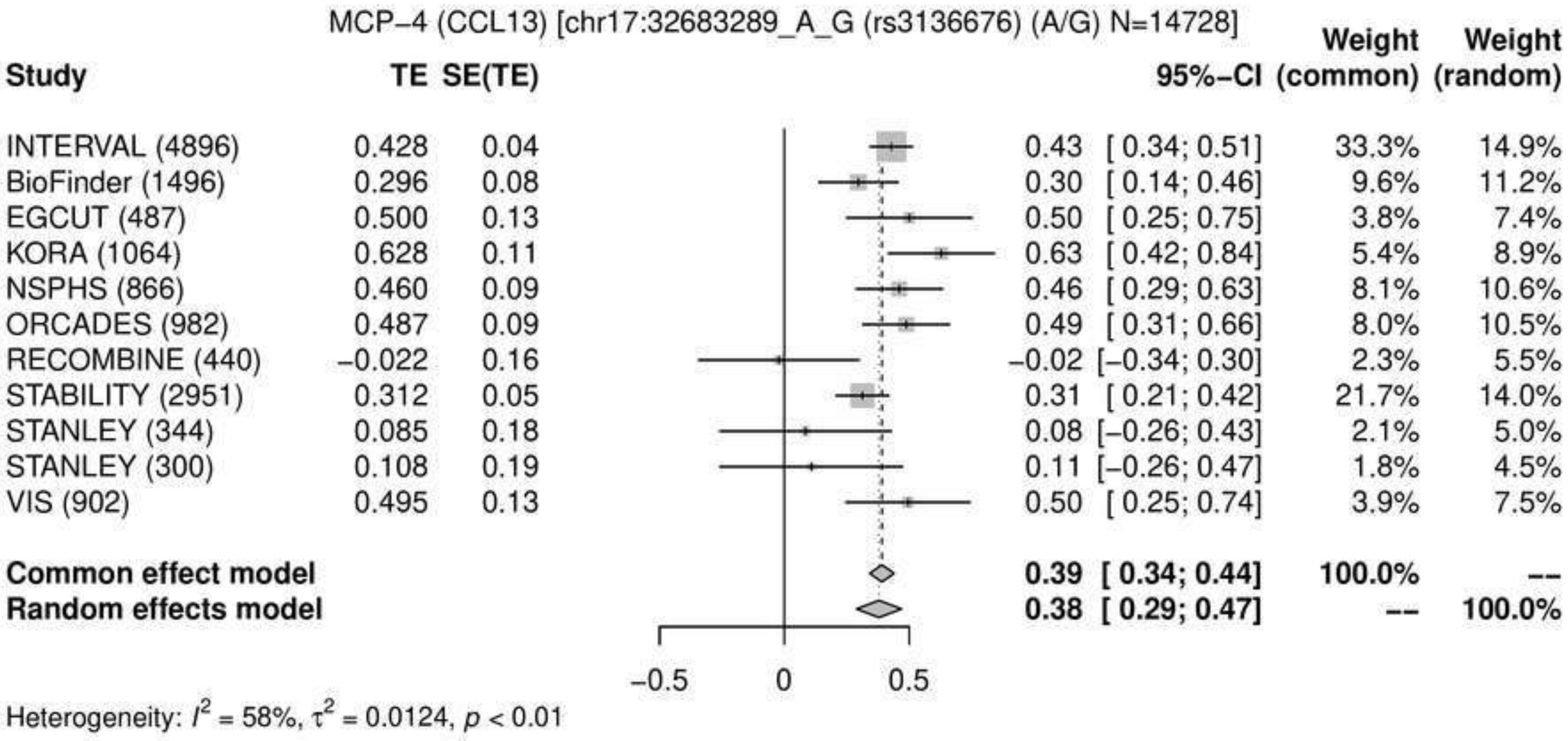
MCP−4 (CCL13) [chr17:32683289_A_G (rs3136676) (A/G) N=14728]
Study
TE
SE(TE)
95%−CI
Weight (common)
Weight (random)
INTERVAL (4896) 0.428 0.04 0.43 [0.34; 0.51] 33.3% 14.9%
BioFinder (1496) 0.296 0.08 0.30 [0.14; 0.46] 9.6% 11.2%
EGCUT (487) 0.500 0.13 0.50 [0.25; 0.75] 3.8% 7.4%
KORA (1064) 0.628 0.11 0.63 [0.42; 0.84] 5.4% 8.9%
NSPHS (866) 0.460 0.09 0.46 [0.29; 0.63] 8.1% 10.6%
ORCADES (982) 0.487 0.09 0.49 [0.31; 0.66] 8.0% 10.5%
RECOMBINE (440) −0.022 0.16 −0.02 [−0.34; 0.30] 2.3% 5.5%
STABILITY (2951) 0.312 0.05 0.31 [0.21; 0.42] 21.7% 14.0%
STANLEY (344) 0.085 0.18 0.08 [−0.26; 0.43] 2.1% 5.0%
STANLEY (300) 0.108 0.19 0.11 [−0.26; 0.47] 1.8% 4.5%
VIS (902) 0.495 0.13 0.50 [0.25; 0.74] 3.9% 7.5%
Common effect model 0.39 [0.34; 0.44] 100.0% --
Random effects model 0.38 [0.29; 0.47] -- 100.0%
−0.5
0
0.5
Heterogeneity: I^2 = 58%, τ^2 = 0.0124, p < 0.01

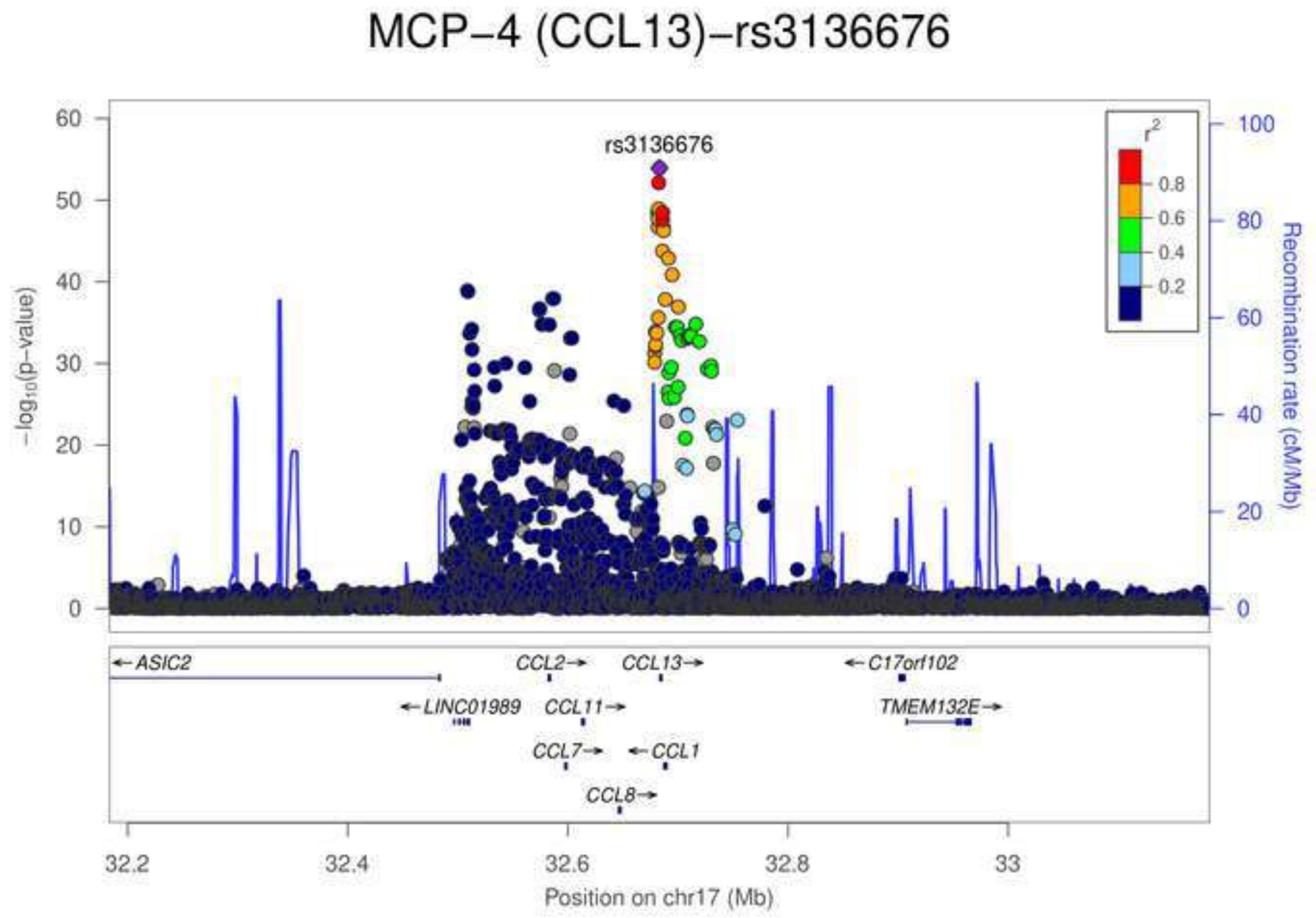
MCP−4 (CCL13)−rs3136676
rs3136676
r^2
0.8
0.6
0.4
0.2
$-\log_{10}$(p−value)
Recombination rate (cM/Mb)
0
10
20
30
40
50
60
0
20
40
60
80
100
ASIC2
CCL2
CCL13
C17orf102
LINC01989
CCL11
TMEM132E
CCL7
CCL1
CCL8
32.2
32.4
32.6
32.8
33
Position on chr17 (Mb)

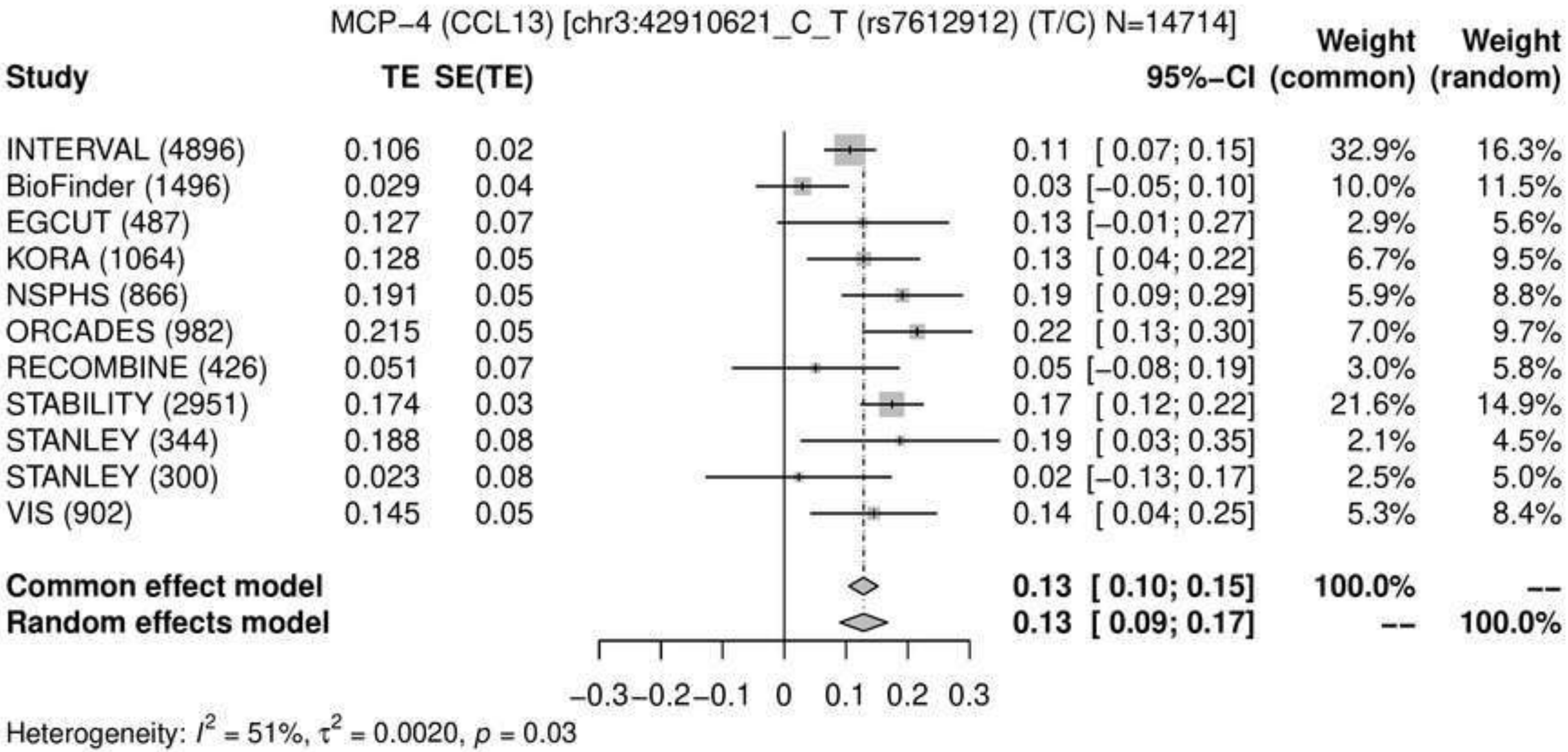
MCP−4 (CCL13) [chr3:42910621_C_T (rs7612912) (T/C) N=14714]
Study
TE
SE(TE)
95%−CI
Weight (common)
Weight (random)
INTERVAL (4896) 0.106 0.02 0.11 [0.07; 0.15] 32.9% 16.3%
BioFinder (1496) 0.029 0.04 0.03 [−0.05; 0.10] 10.0% 11.5%
EGCUT (487) 0.127 0.07 0.13 [−0.01; 0.27] 2.9% 5.6%
KORA (1064) 0.128 0.05 0.13 [0.04; 0.22] 6.7% 9.5%
NSPHS (866) 0.191 0.05 0.19 [0.09; 0.29] 5.9% 8.8%
ORCADES (982) 0.215 0.05 0.22 [0.13; 0.30] 7.0% 9.7%
RECOMBINE (426) 0.051 0.07 0.05 [−0.08; 0.19] 3.0% 5.8%
STABILITY (2951) 0.174 0.03 0.17 [0.12; 0.22] 21.6% 14.9%
STANLEY (344) 0.188 0.08 0.19 [0.03; 0.35] 2.1% 4.5%
STANLEY (300) 0.023 0.08 0.02 [−0.13; 0.17] 2.5% 5.0%
VIS (902) 0.145 0.05 0.14 [0.04; 0.25] 5.3% 8.4%
Common effect model 0.13 [0.10; 0.15] 100.0% --
Random effects model 0.13 [0.09; 0.17] -- 100.0%
−0.3 −0.2 −0.1 0 0.1 0.2 0.3
Heterogeneity: I^2 = 51%, τ^2 = 0.0020, p = 0.03

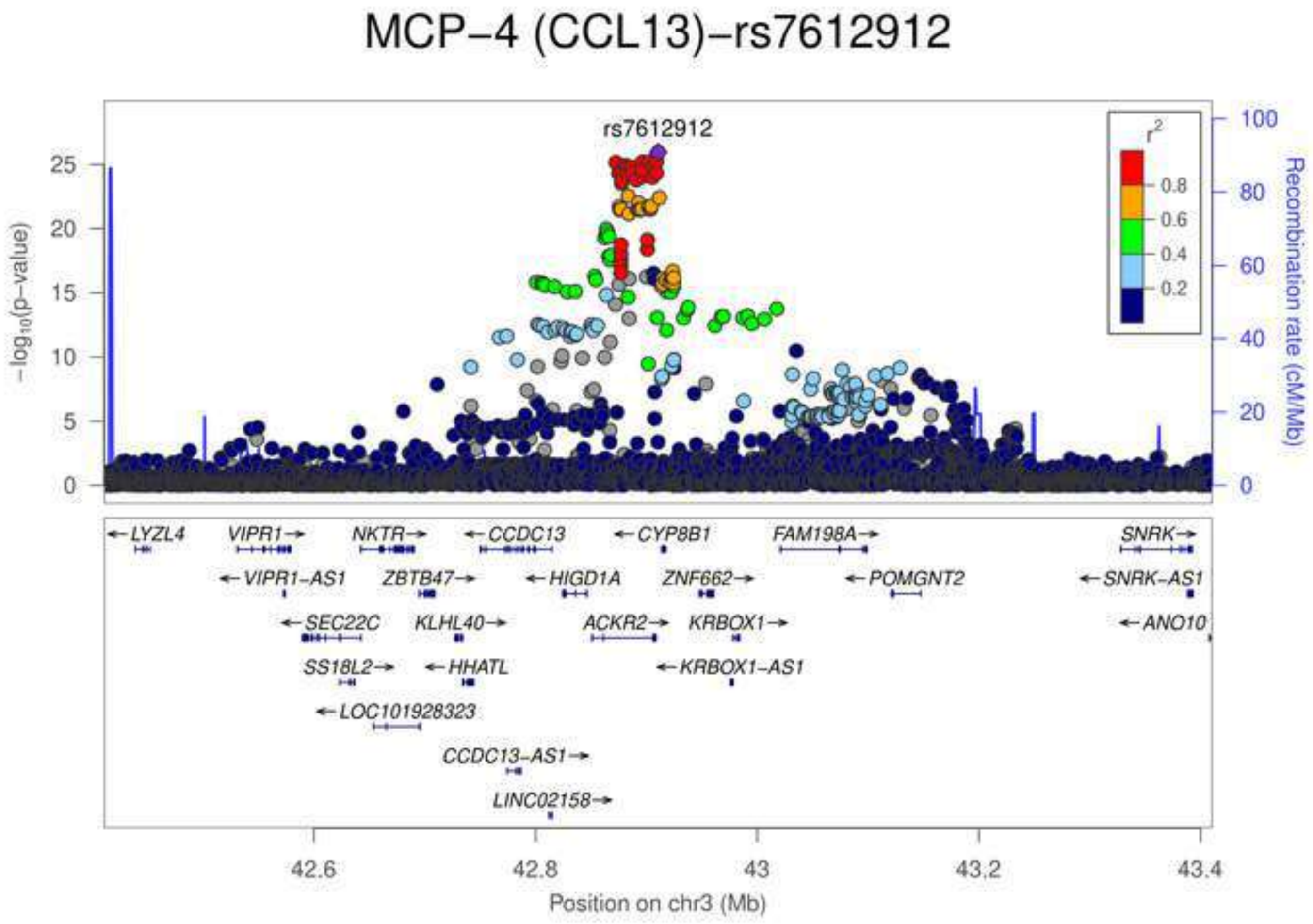
MCP−4 (CCL13)−rs7612912
rs7612912
$-\log_{10}$(p−value)
0
5
10
15
20
25
Recombination rate (cM/Mb)
0
20
40
60
80
100
r^2
0.8
0.6
0.4
0.2
LYZL4
VIPR1
NKTR
CCDC13
CYP8B1
FAM198A
SNRK
VIPR1−AS1
ZBTB47
HIGD1A
ZNF662
POMGNT2
SNRK−AS1
SEC22C
KLHL40
ACKR2
KRBOX1
ANO10
SS18L2
HHATL
KRBOX1−AS1
LOC101928323
CCDC13−AS1
LINC02158
42.6
42.8
43
43.2
43.4
Position on chr3 (Mb)

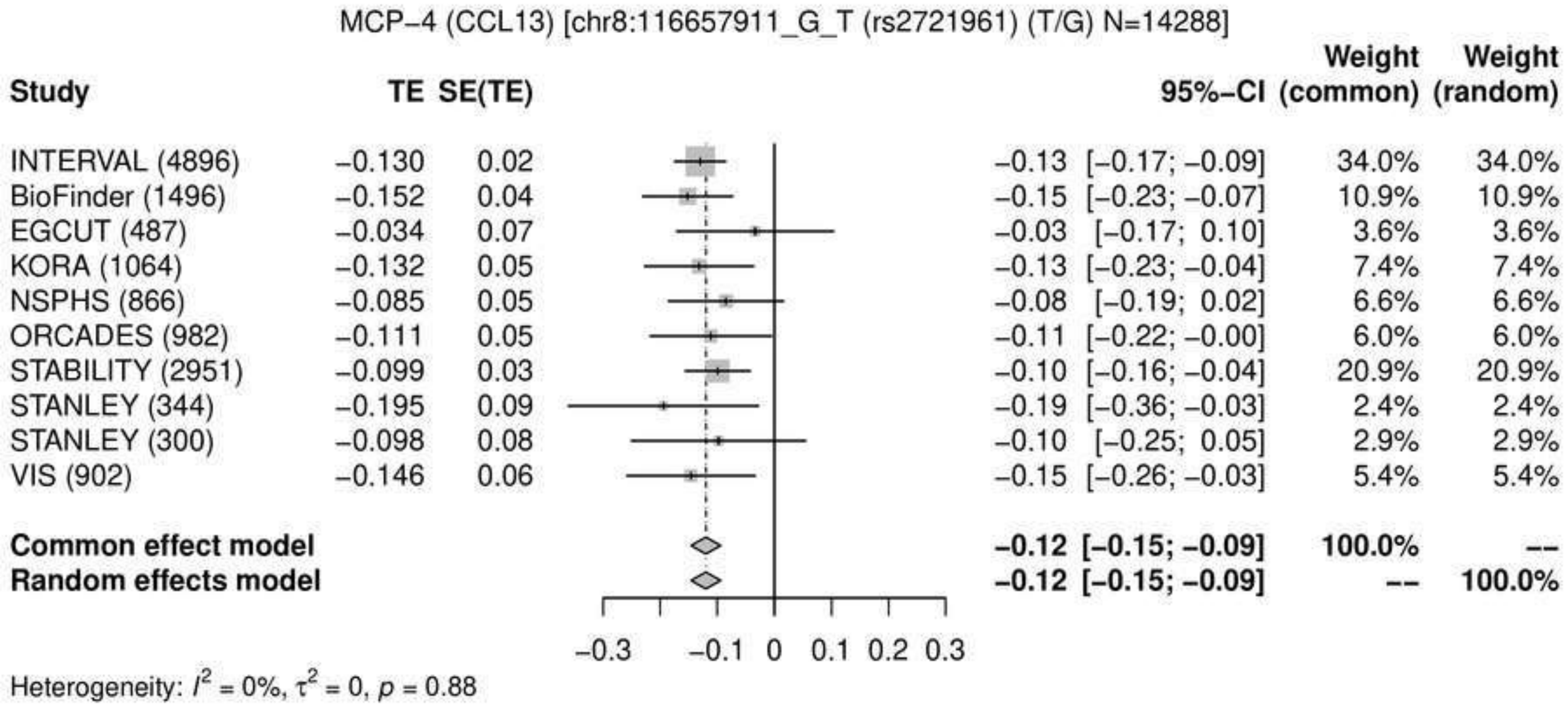

MCP−4 (CCL13) [chr8:116657911_G_T (rs2721961) (T/G) N=14288]
Study | TE | SE(TE) | 95%−CI | Weight (common) | Weight (random)
INTERVAL (4896) | −0.130 | 0.02 | −0.13 [−0.17; −0.09] | 34.0% | 34.0%
BioFinder (1496) | −0.152 | 0.04 | −0.15 [−0.23; −0.07] | 10.9% | 10.9%
EGCUT (487) | −0.034 | 0.07 | −0.03 [−0.17; 0.10] | 3.6% | 3.6%
KORA (1064) | −0.132 | 0.05 | −0.13 [−0.23; −0.04] | 7.4% | 7.4%
NSPHS (866) | −0.085 | 0.05 | −0.08 [−0.19; 0.02] | 6.6% | 6.6%
ORCADES (982) | −0.111 | 0.05 | −0.11 [−0.22; −0.00] | 6.0% | 6.0%
STABILITY (2951) | −0.099 | 0.03 | −0.10 [−0.16; −0.04] | 20.9% | 20.9%
STANLEY (344) | −0.195 | 0.09 | −0.19 [−0.36; −0.03] | 2.4% | 2.4%
STANLEY (300) | −0.098 | 0.08 | −0.10 [−0.25; 0.05] | 2.9% | 2.9%
VIS (902) | −0.146 | 0.06 | −0.15 [−0.26; −0.03] | 5.4% | 5.4%
Common effect model | −0.12 [−0.15; −0.09] | 100.0% | --
Random effects model | −0.12 [−0.15; −0.09] | -- | 100.0%
−0.3 −0.1 0 0.1 0.2 0.3
Heterogeneity: I^2 = 0%, τ^2 = 0, p = 0.88

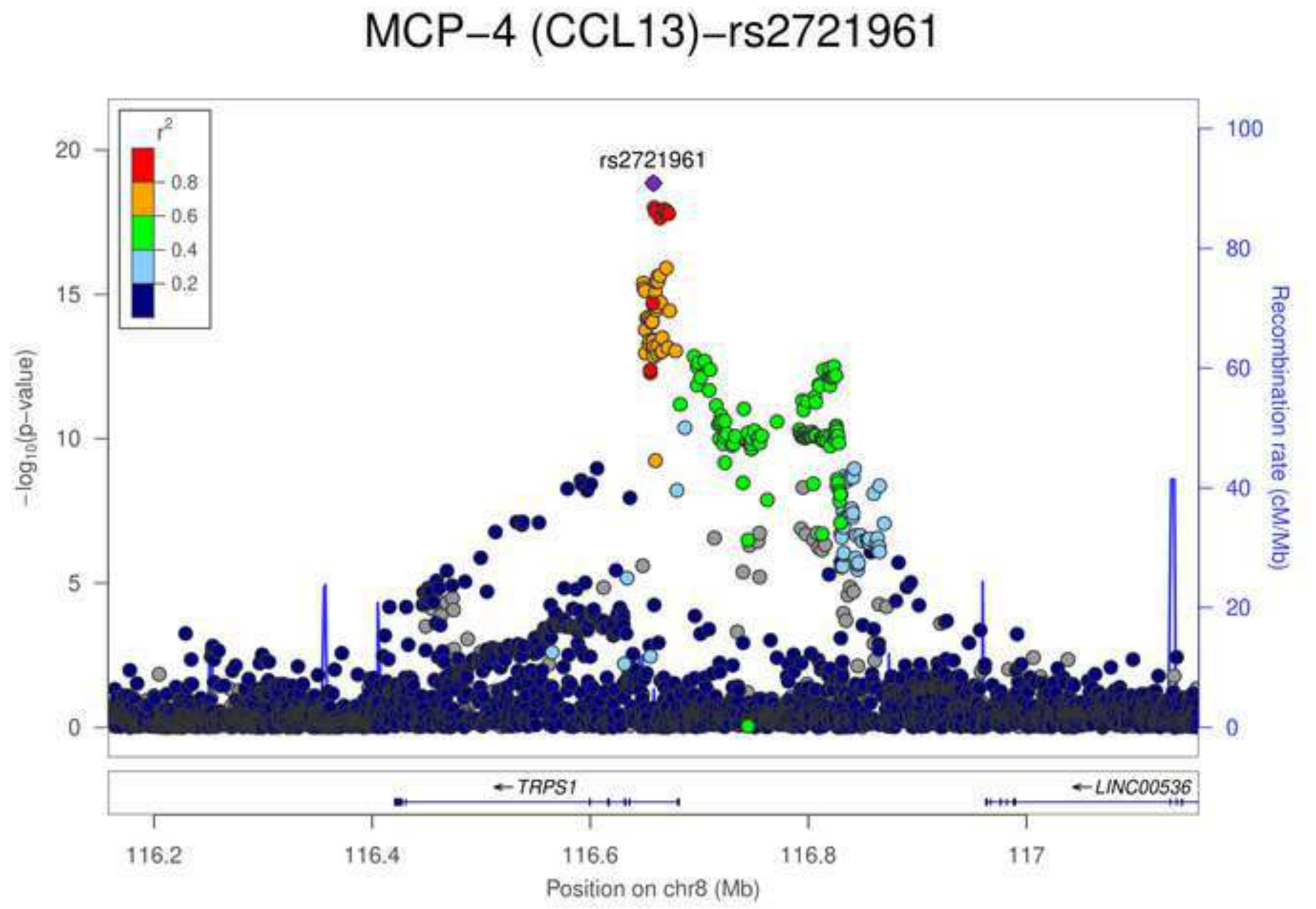

MCP−4 (CCL13)−rs2721961
rs2721961
r^2
0.8
0.6
0.4
0.2
−log10(p−value)
Recombination rate (cM/Mb)
0 5 10 15 20
0 20 40 60 80 100
TRPS1
LINC00536
116.2 116.4 116.6 116.8 117
Position on chr8 (Mb)

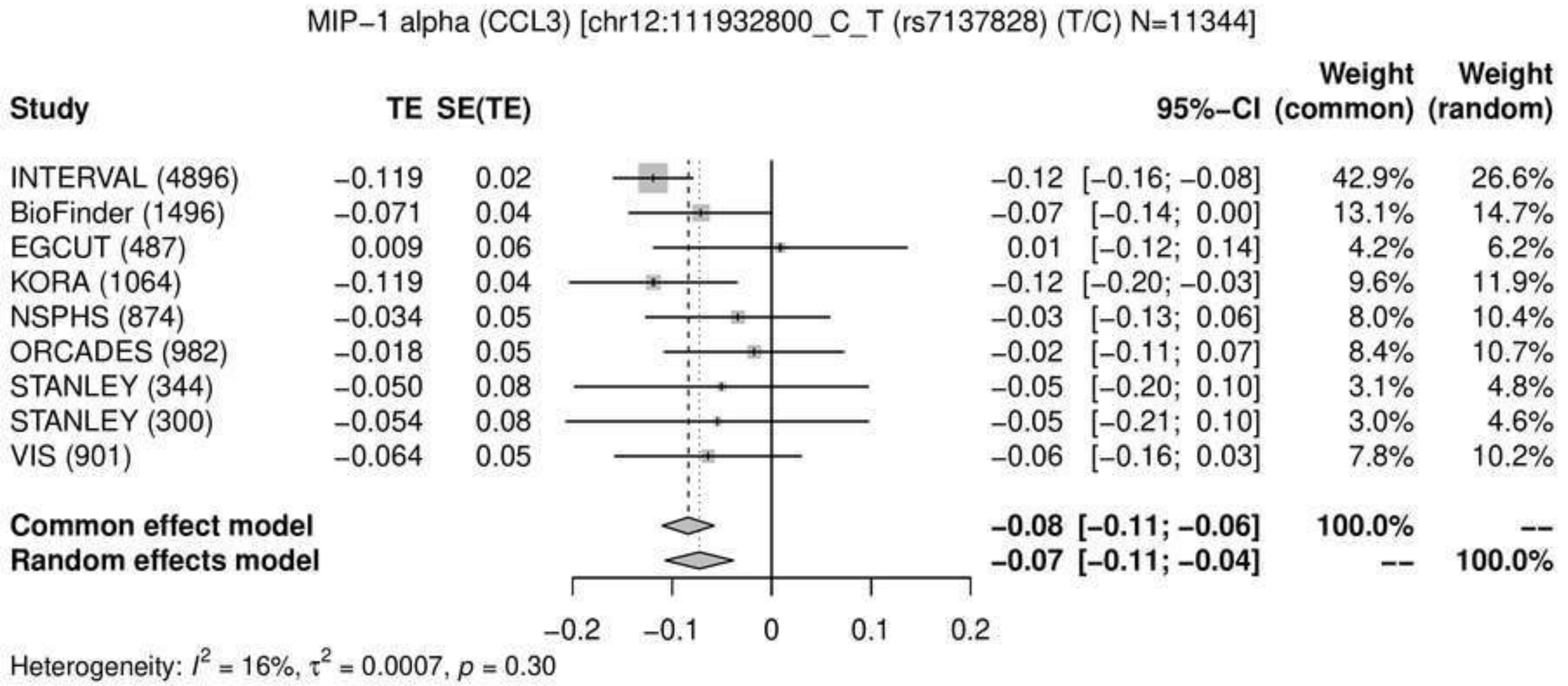
MIP−1 alpha (CCL3) [chr12:111932800_C_T (rs7137828) (T/C) N=11344]
Study
TE
SE(TE)
95%−CI
Weight (common)
Weight (random)
INTERVAL (4896) −0.119 0.02 −0.12 [−0.16; −0.08] 42.9% 26.6%
BioFinder (1496) −0.071 0.04 −0.07 [−0.14; 0.00] 13.1% 14.7%
EGCUT (487) 0.009 0.06 0.01 [−0.12; 0.14] 4.2% 6.2%
KORA (1064) −0.119 0.04 −0.12 [−0.20; −0.03] 9.6% 11.9%
NSPHS (874) −0.034 0.05 −0.03 [−0.13; 0.06] 8.0% 10.4%
ORCADES (982) −0.018 0.05 −0.02 [−0.11; 0.07] 8.4% 10.7%
STANLEY (344) −0.050 0.08 −0.05 [−0.20; 0.10] 3.1% 4.8%
STANLEY (300) −0.054 0.08 −0.05 [−0.21; 0.10] 3.0% 4.6%
VIS (901) −0.064 0.05 −0.06 [−0.16; 0.03] 7.8% 10.2%
Common effect model −0.08 [−0.11; −0.06] 100.0% --
Random effects model −0.07 [−0.11; −0.04] -- 100.0%
−0.2 −0.1 0 0.1 0.2
Heterogeneity: I^2 = 16%, τ^2 = 0.0007, p = 0.30

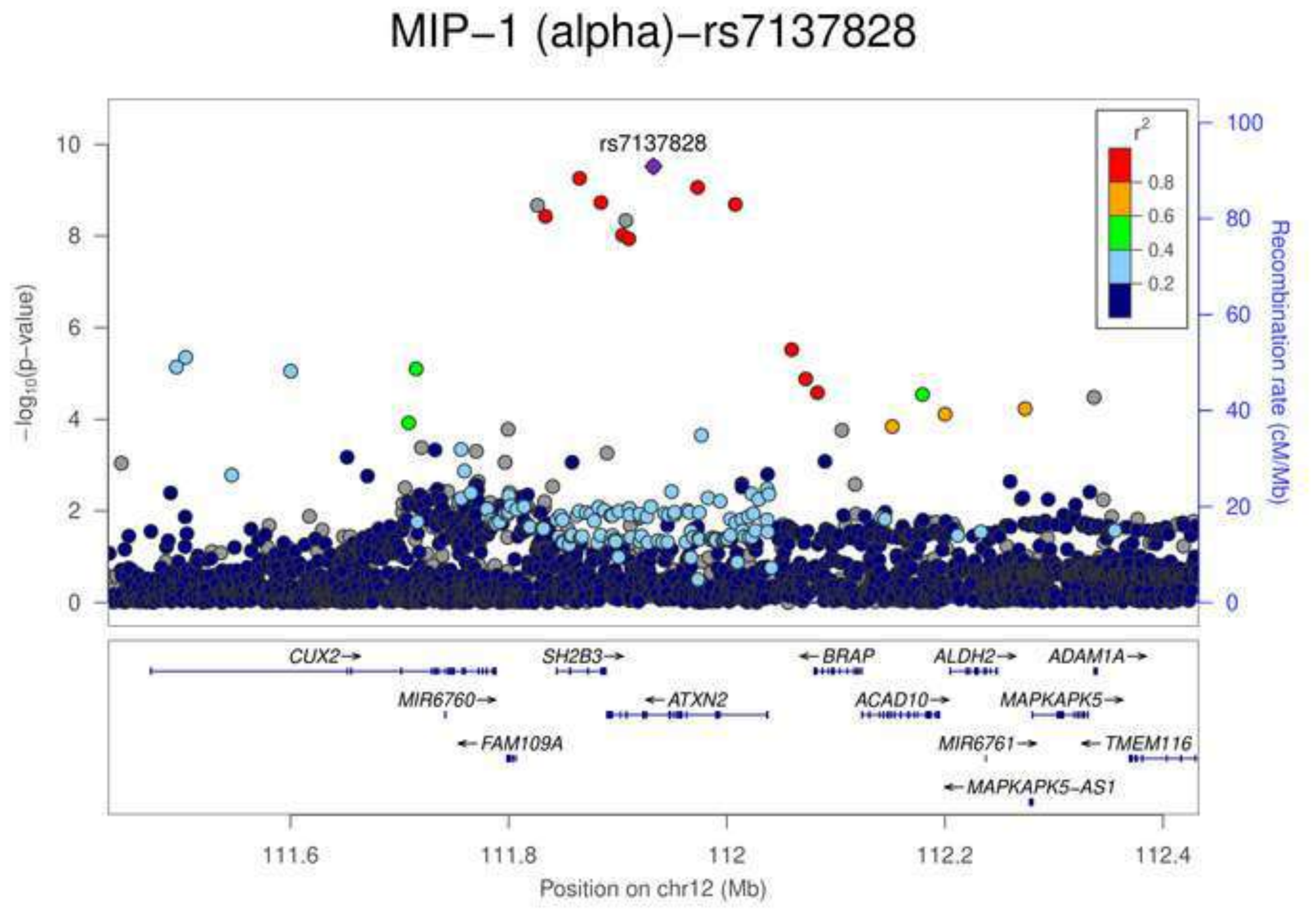
MIP−1 (alpha)−rs7137828
rs7137828
$-\log_{10}$(p−value)
Recombination rate (cM/Mb)
r^2
0.8
0.6
0.4
0.2
CUX2→
SH2B3→
←BRAP
ALDH2→
ADAM1A→
MIR6760→
←ATXN2
ACAD10→
MAPKAPK5→
←FAM109A
MIR6761→
←TMEM116
←MAPKAPK5-AS1
111.6
111.8
112
112.2
112.4
Position on chr12 (Mb)

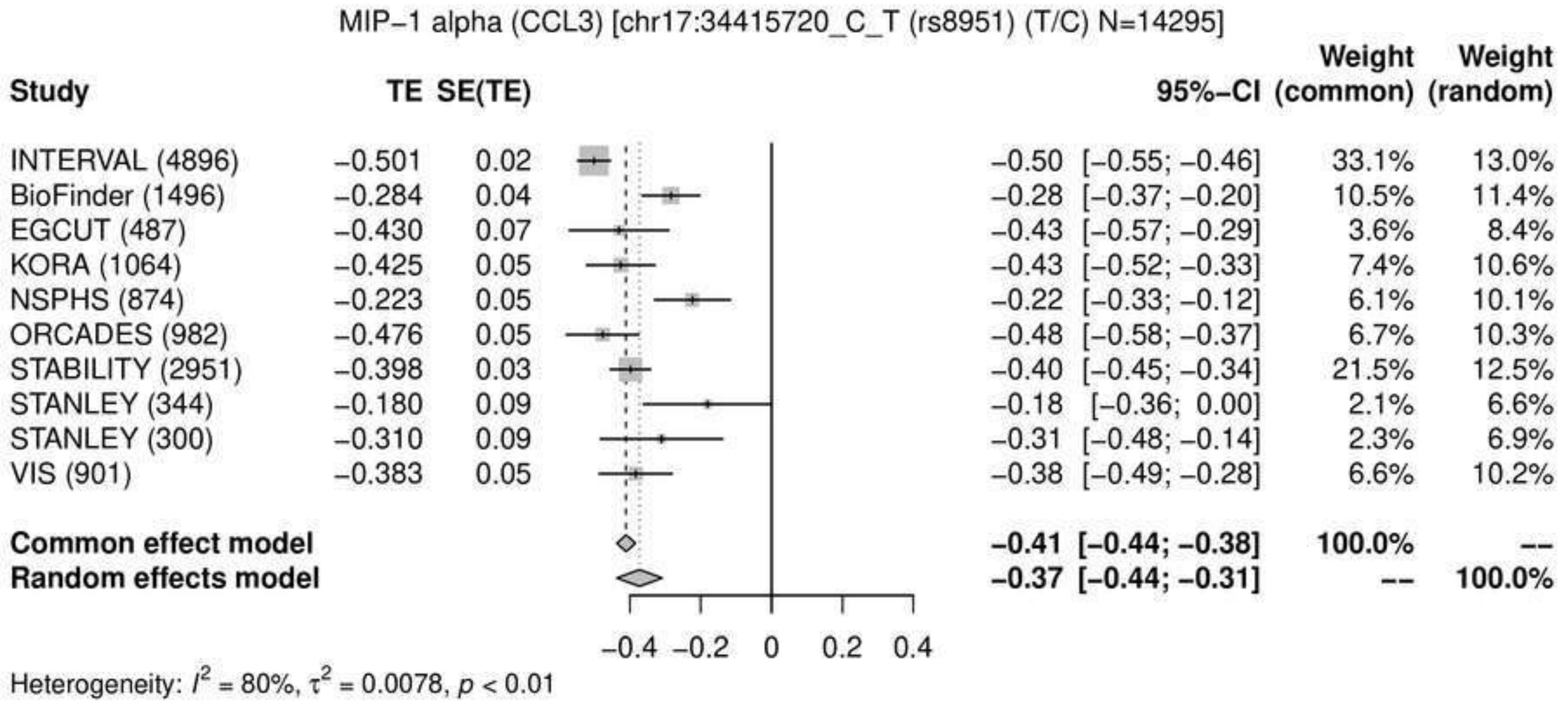
MIP−1 alpha (CCL3) [chr17:34415720_C_T (rs8951) (T/C) N=14295]
Study TE SE(TE) 95%−CI Weight (common) Weight (random)
INTERVAL (4896) −0.501 0.02 −0.50 [−0.55; −0.46] 33.1% 13.0%
BioFinder (1496) −0.284 0.04 −0.28 [−0.37; −0.20] 10.5% 11.4%
EGCUT (487) −0.430 0.07 −0.43 [−0.57; −0.29] 3.6% 8.4%
KORA (1064) −0.425 0.05 −0.43 [−0.52; −0.33] 7.4% 10.6%
NSPHS (874) −0.223 0.05 −0.22 [−0.33; −0.12] 6.1% 10.1%
ORCADES (982) −0.476 0.05 −0.48 [−0.58; −0.37] 6.7% 10.3%
STABILITY (2951) −0.398 0.03 −0.40 [−0.45; −0.34] 21.5% 12.5%
STANLEY (344) −0.180 0.09 −0.18 [−0.36; 0.00] 2.1% 6.6%
STANLEY (300) −0.310 0.09 −0.31 [−0.48; −0.14] 2.3% 6.9%
VIS (901) −0.383 0.05 −0.38 [−0.49; −0.28] 6.6% 10.2%
Common effect model −0.41 [−0.44; −0.38] 100.0% --
Random effects model −0.37 [−0.44; −0.31] -- 100.0%
−0.4 −0.2 0 0.2 0.4
Heterogeneity: I^2 = 80%, τ^2 = 0.0078, p < 0.01

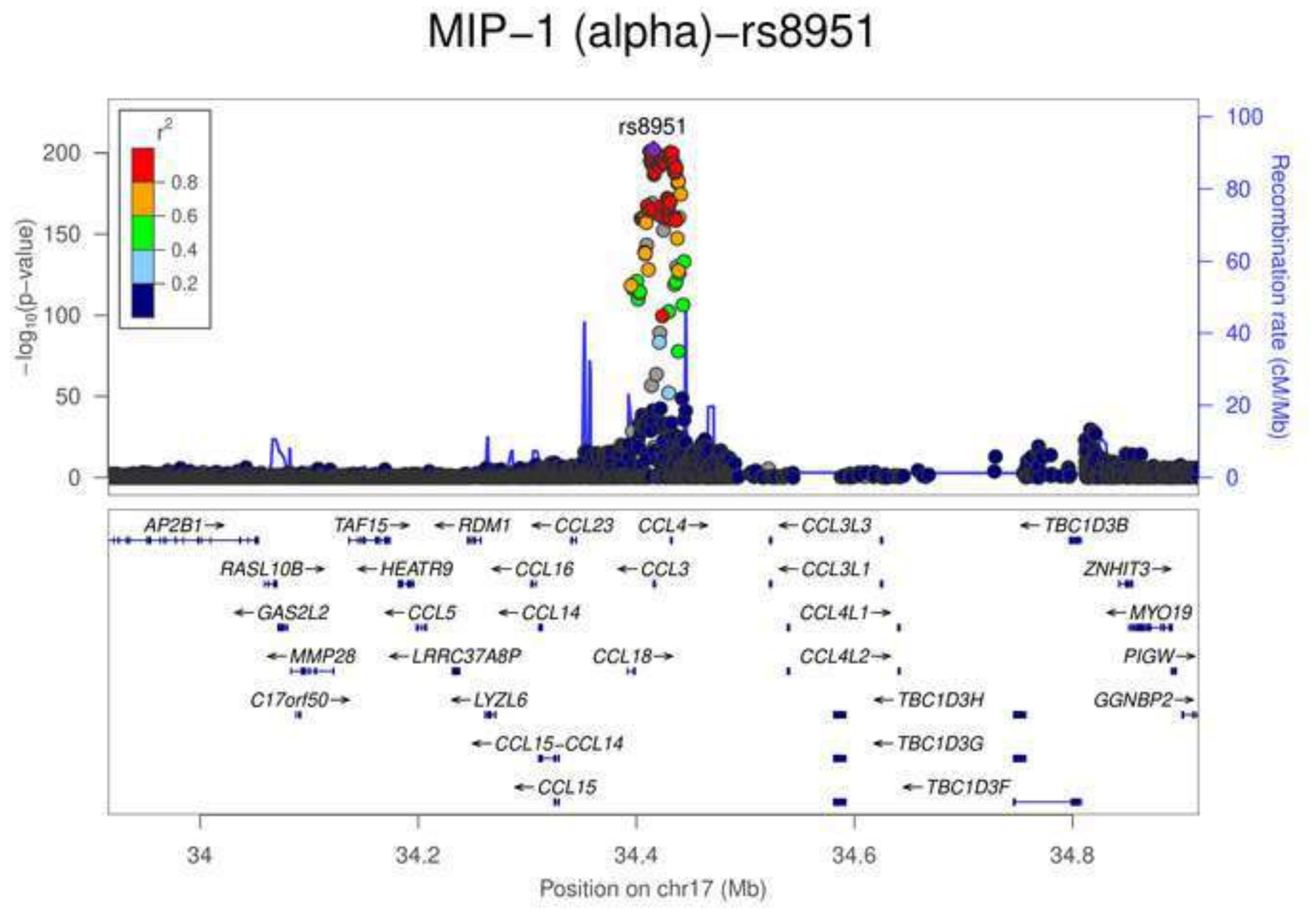
MIP−1 (alpha)−rs8951
rs8951
r^2
0.8
0.6
0.4
0.2
$-\log_{10}$(p−value)
Recombination rate (cM/Mb)
AP2B1
TAF15
RDM1
CCL23
CCL4
CCL3L3
TBC1D3B
RASL10B
HEATR9
CCL16
CCL3
CCL3L1
ZNHIT3
GAS2L2
CCL5
CCL14
CCL4L1
MYO19
MMP28
LRRC37A8P
CCL18
CCL4L2
PIGW
C17orf50
LYZL6
TBC1D3H
GGNBP2
CCL15−CCL14
TBC1D3G
CCL15
TBC1D3F
34 34.2 34.4 34.6 34.8
Position on chr17 (Mb)

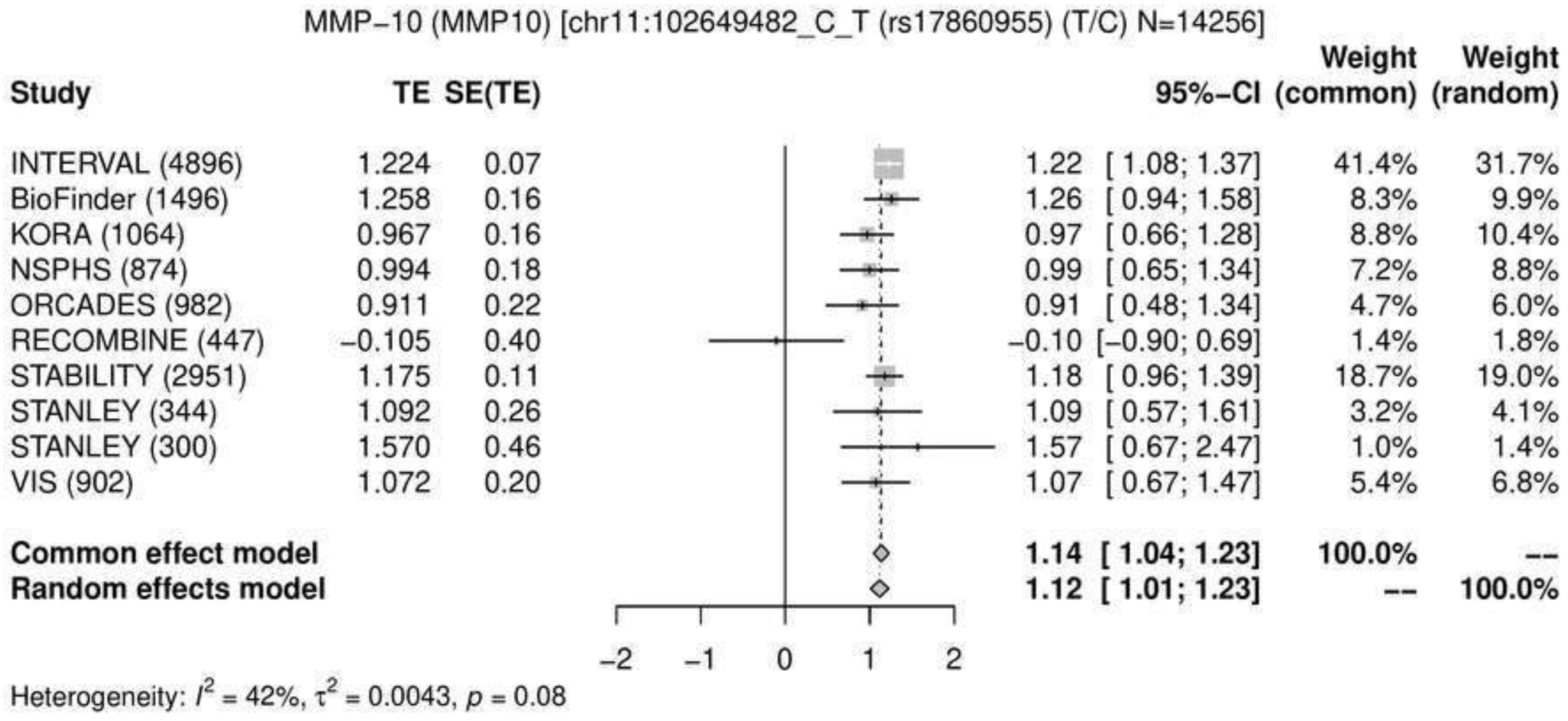
MMP−10 (MMP10) [chr11:102649482_C_T (rs17860955) (T/C) N=14256]
Study TE SE(TE) 95%−CI Weight (common) Weight (random)
INTERVAL (4896) 1.224 0.07 1.22 [1.08; 1.37] 41.4% 31.7%
BioFinder (1496) 1.258 0.16 1.26 [0.94; 1.58] 8.3% 9.9%
KORA (1064) 0.967 0.16 0.97 [0.66; 1.28] 8.8% 10.4%
NSPHS (874) 0.994 0.18 0.99 [0.65; 1.34] 7.2% 8.8%
ORCADES (982) 0.911 0.22 0.91 [0.48; 1.34] 4.7% 6.0%
RECOMBINE (447) −0.105 0.40 −0.10 [−0.90; 0.69] 1.4% 1.8%
STABILITY (2951) 1.175 0.11 1.18 [0.96; 1.39] 18.7% 19.0%
STANLEY (344) 1.092 0.26 1.09 [0.57; 1.61] 3.2% 4.1%
STANLEY (300) 1.570 0.46 1.57 [0.67; 2.47] 1.0% 1.4%
VIS (902) 1.072 0.20 1.07 [0.67; 1.47] 5.4% 6.8%
Common effect model 1.14 [1.04; 1.23] 100.0% --
Random effects model 1.12 [1.01; 1.23] -- 100.0%
−2 −1 0 1 2
Heterogeneity: I^2 = 42%, τ^2 = 0.0043, p = 0.08

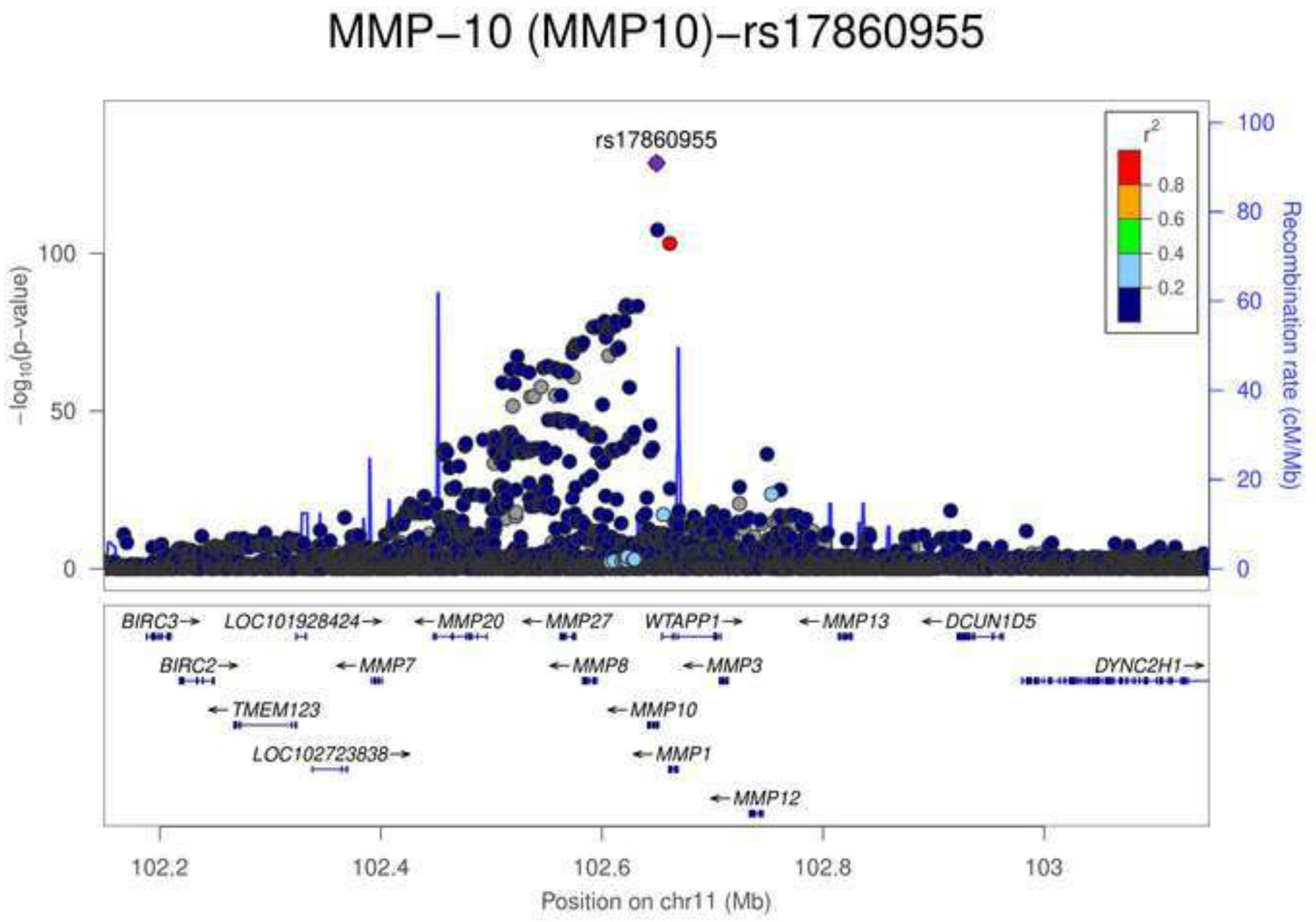
MMP−10 (MMP10)−rs17860955
rs17860955
r^2
0.8
0.6
0.4
0.2
$-\log_{10}$(p-value)
Recombination rate (cM/Mb)
0
50
100
0
20
40
60
80
100
BIRC3→
LOC101928424→
←MMP20
←MMP27
WTAPP1→
←MMP13
←DCUN1D5
BIRC2→
←MMP7
←MMP8
←MMP3
DYNC2H1→
←TMEM123
←MMP10
LOC102723838→
←MMP1
←MMP12
102.2
102.4
102.6
102.8
103
Position on chr11 (Mb)

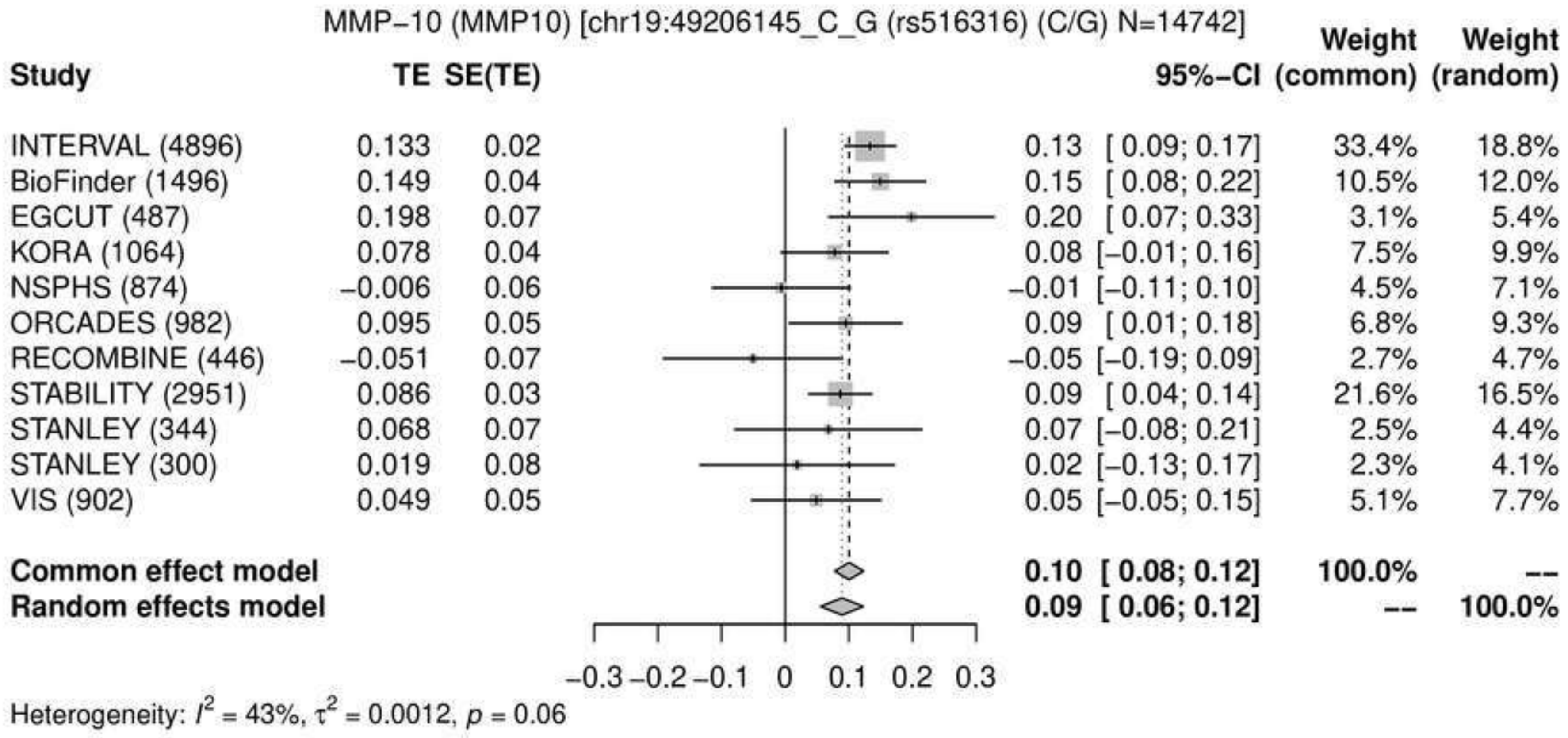
MMP−10 (MMP10) [chr19:49206145_C_G (rs516316) (C/G) N=14742]
Study TE SE(TE) 95%−CI Weight (common) Weight (random)
INTERVAL (4896) 0.133 0.02 0.13 [0.09; 0.17] 33.4% 18.8%
BioFinder (1496) 0.149 0.04 0.15 [0.08; 0.22] 10.5% 12.0%
EGCUT (487) 0.198 0.07 0.20 [0.07; 0.33] 3.1% 5.4%
KORA (1064) 0.078 0.04 0.08 [−0.01; 0.16] 7.5% 9.9%
NSPHS (874) −0.006 0.06 −0.01 [−0.11; 0.10] 4.5% 7.1%
ORCADES (982) 0.095 0.05 0.09 [0.01; 0.18] 6.8% 9.3%
RECOMBINE (446) −0.051 0.07 −0.05 [−0.19; 0.09] 2.7% 4.7%
STABILITY (2951) 0.086 0.03 0.09 [0.04; 0.14] 21.6% 16.5%
STANLEY (344) 0.068 0.07 0.07 [−0.08; 0.21] 2.5% 4.4%
STANLEY (300) 0.019 0.08 0.02 [−0.13; 0.17] 2.3% 4.1%
VIS (902) 0.049 0.05 0.05 [−0.05; 0.15] 5.1% 7.7%
Common effect model 0.10 [0.08; 0.12] 100.0% --
Random effects model 0.09 [0.06; 0.12] -- 100.0%
−0.3 −0.2 −0.1 0 0.1 0.2 0.3
Heterogeneity: I² = 43%, τ² = 0.0012, p = 0.06

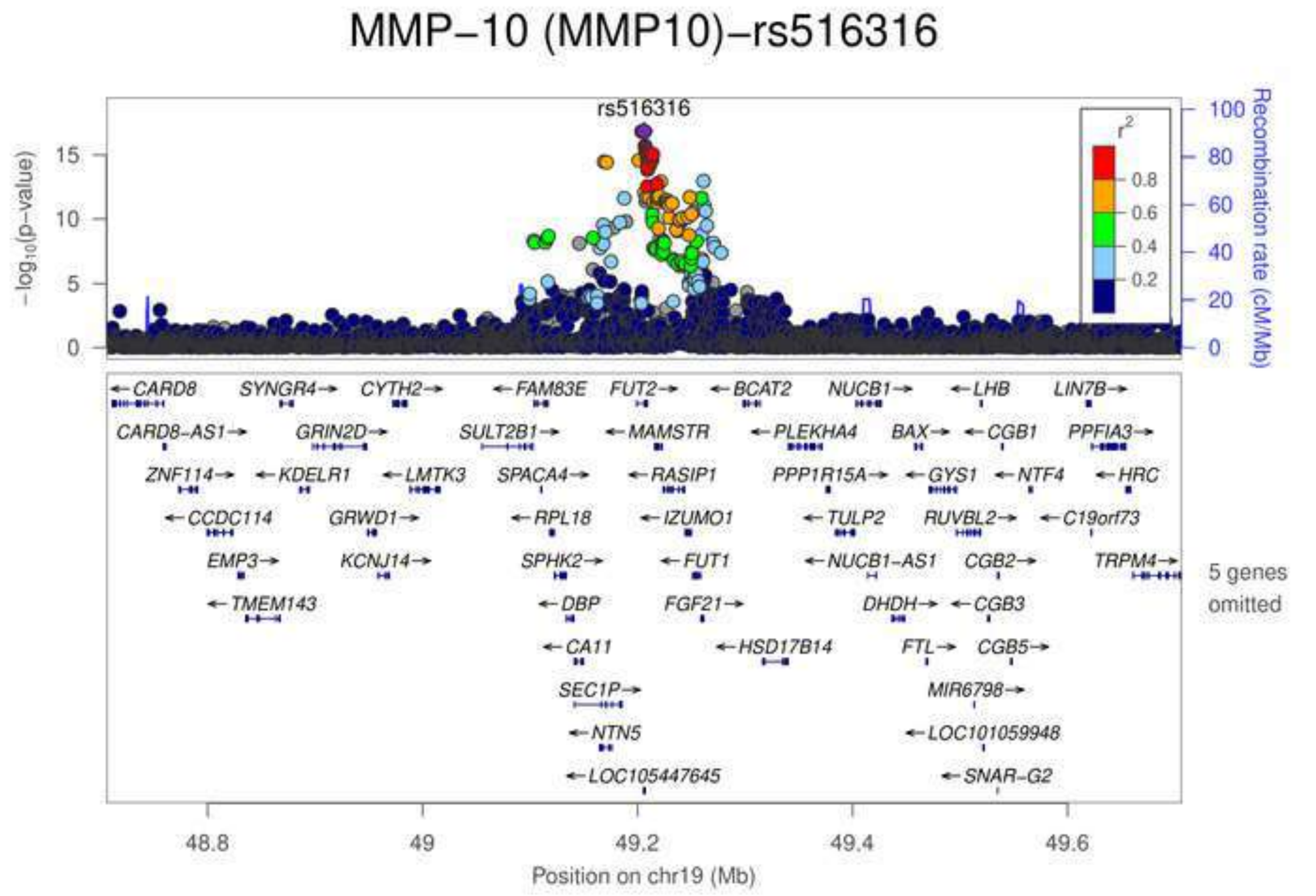
MMP−10 (MMP10)−rs516316
rs516316
−log10(p−value)
Recombination rate (cM/Mb)
r²
0.8
0.6
0.4
0.2
CARD8 SYNGR4 CYTH2 FAM83E FUT2 BCAT2 NUCB1 LHB LIN7B
CARD8−AS1 GRIN2D SULT2B1 MAMSTR PLEKHA4 BAX CGB1 PPFIA3
ZNF114 KDELR1 LMTK3 SPACA4 RASIP1 PPP1R15A GYS1 NTF4 HRC
CCDC114 GRWD1 RPL18 IZUMO1 TULP2 RUVBL2 C19orf73
EMP3 KCNJ14 SPHK2 FUT1 NUCB1−AS1 CGB2 TRPM4
TMEM143 DBP FGF21 DHDH CGB3
CA11 HSD17B14 FTL CGB5
SEC1P MIR6798
NTN5 LOC101059948
LOC105447645 SNAR−G2
5 genes omitted
48.8 49 49.2 49.4 49.6
Position on chr19 (Mb)

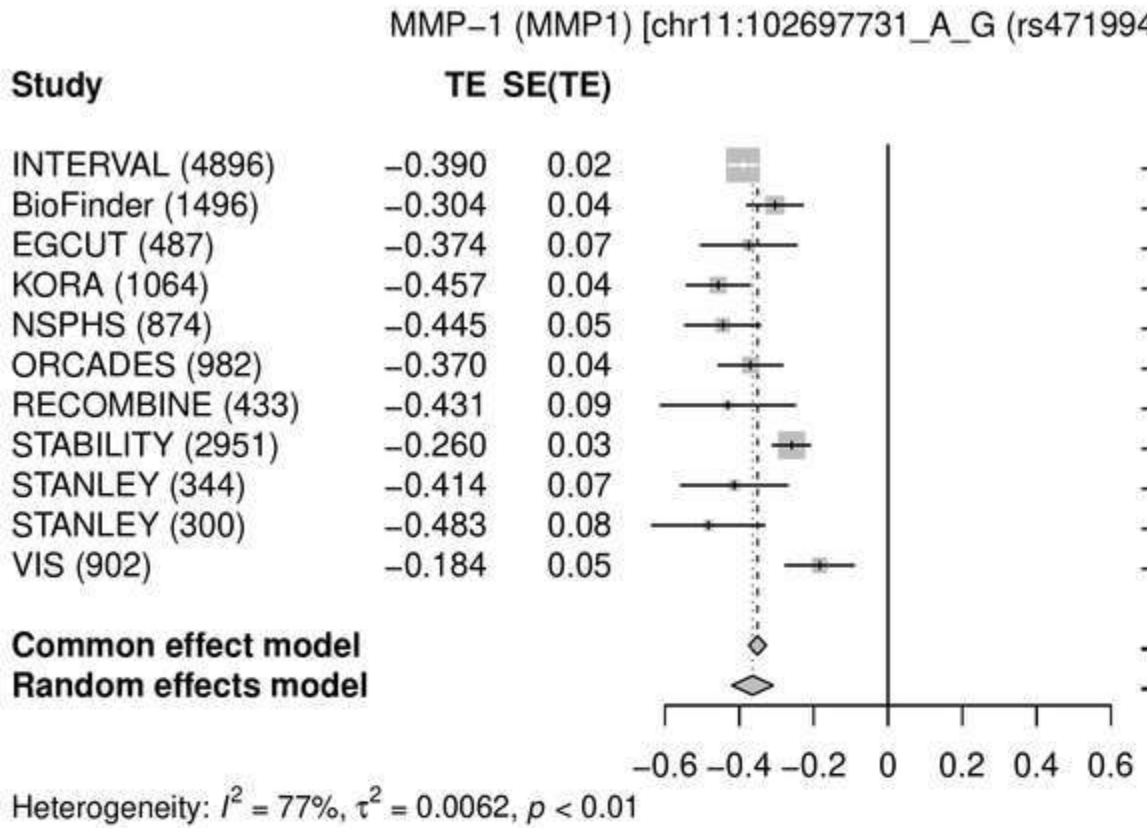
MMP−1 (MMP1) [chr11:102697731_A_G (rs471994) (A/G) N=14729]
Study
TE
SE(TE)
95%−CI
Weight (common)
Weight (random)
INTERVAL (4896) −0.390 0.02 −0.39 [−0.43; −0.35] 33.4% 12.2%
BioFinder (1496) −0.304 0.04 −0.30 [−0.38; −0.23] 9.5% 10.5%
EGCUT (487) −0.374 0.07 −0.37 [−0.50; −0.24] 3.2% 7.6%
KORA (1064) −0.457 0.04 −0.46 [−0.54; −0.37] 7.5% 10.0%
NSPHS (874) −0.445 0.05 −0.44 [−0.55; −0.34] 5.3% 9.1%
ORCADES (982) −0.370 0.04 −0.37 [−0.46; −0.28] 7.2% 9.9%
RECOMBINE (433) −0.431 0.09 −0.43 [−0.61; −0.25] 1.6% 5.5%
STABILITY (2951) −0.260 0.03 −0.26 [−0.31; −0.21] 20.9% 11.8%
STANLEY (344) −0.414 0.07 −0.41 [−0.56; −0.27] 2.6% 7.0%
STANLEY (300) −0.483 0.08 −0.48 [−0.64; −0.33] 2.3% 6.6%
VIS (902) −0.184 0.05 −0.18 [−0.28; −0.09] 6.3% 9.6%
Common effect model −0.35 [−0.37; −0.33] 100.0% --
Random effects model −0.36 [−0.42; −0.31] -- 100.0%
−0.6 −0.4 −0.2 0 0.2 0.4 0.6
Heterogeneity: I^2 = 77%, τ^2 = 0.0062, p < 0.01

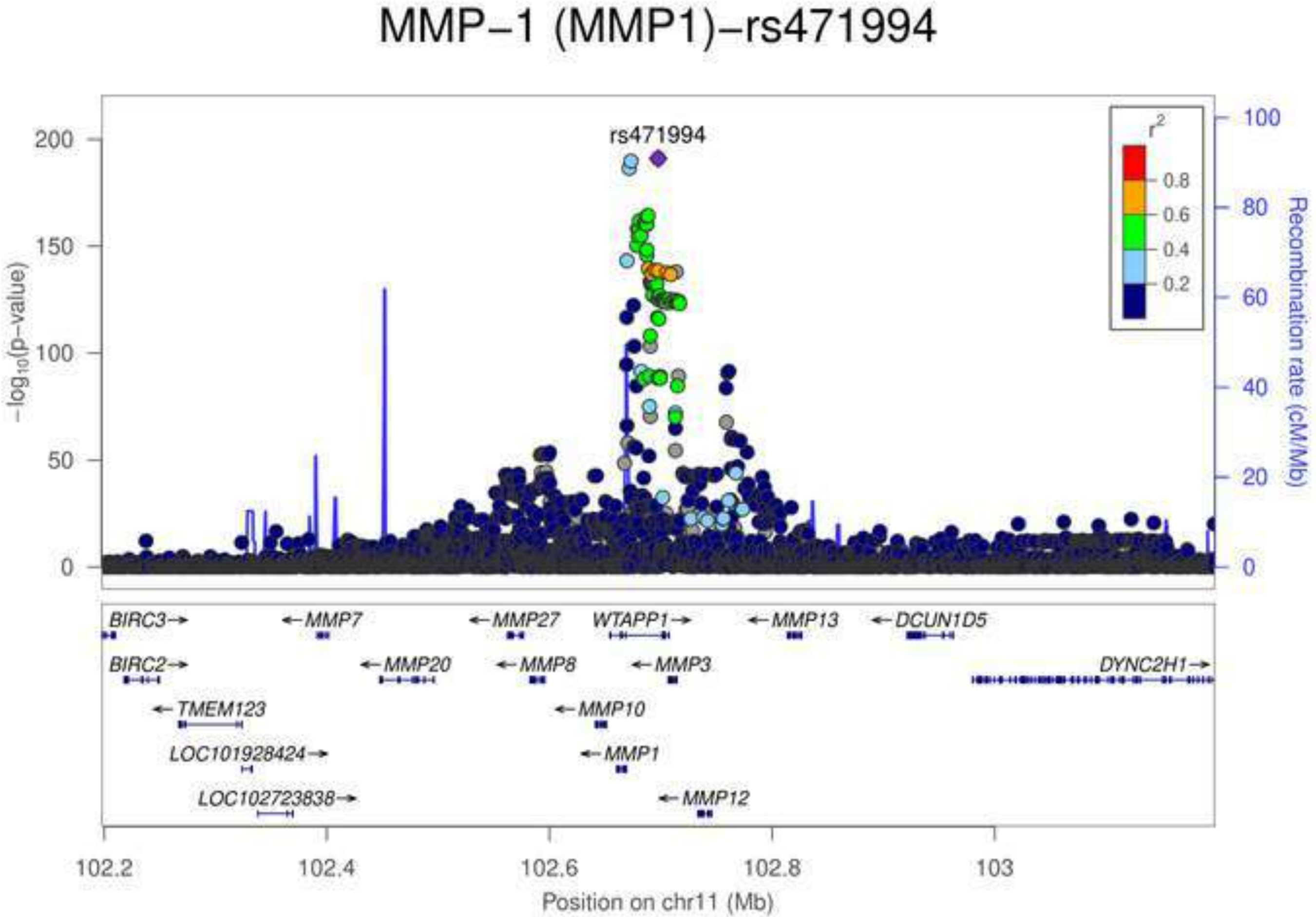
MMP−1 (MMP1)−rs471994
rs471994
$-\log_{10}$(p-value)
Recombination rate (cM/Mb)
r^2
0.8
0.6
0.4
0.2
BIRC3→
←MMP7
←MMP27
WTAPP1→
←MMP13
←DCUN1D5
BIRC2→
←MMP20
←MMP8
←MMP3
DYNC2H1→
←TMEM123
←MMP10
LOC101928424→
←MMP1
LOC102723838→
←MMP12
102.2
102.4
102.6
102.8
103
Position on chr11 (Mb)

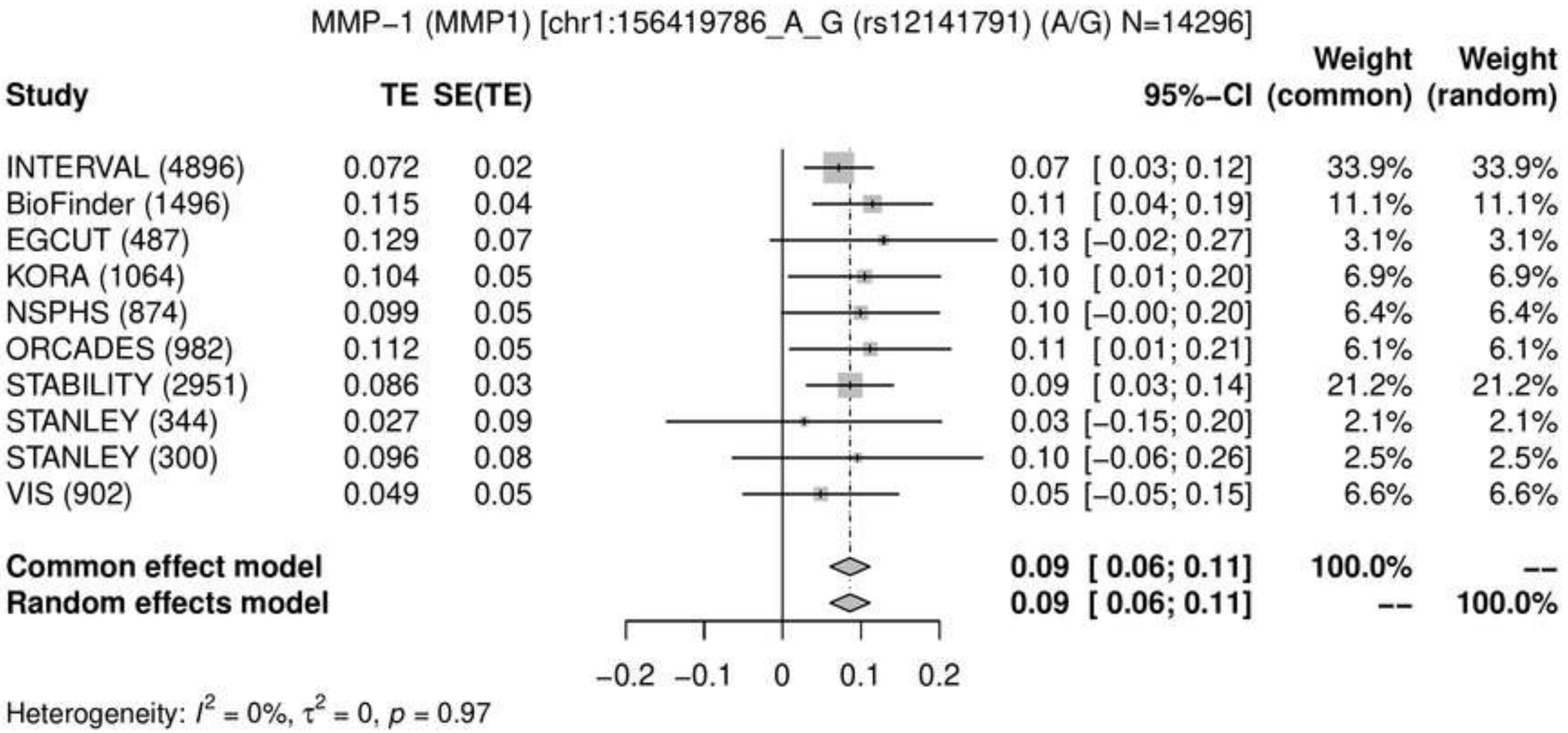
MMP−1 (MMP1) [chr1:156419786_A_G (rs12141791) (A/G) N=14296]
Study
TE
SE(TE)
95%−CI
Weight (common)
Weight (random)
INTERVAL (4896) 0.072 0.02 0.07 [0.03; 0.12] 33.9% 33.9%
BioFinder (1496) 0.115 0.04 0.11 [0.04; 0.19] 11.1% 11.1%
EGCUT (487) 0.129 0.07 0.13 [−0.02; 0.27] 3.1% 3.1%
KORA (1064) 0.104 0.05 0.10 [0.01; 0.20] 6.9% 6.9%
NSPHS (874) 0.099 0.05 0.10 [−0.00; 0.20] 6.4% 6.4%
ORCADES (982) 0.112 0.05 0.11 [0.01; 0.21] 6.1% 6.1%
STABILITY (2951) 0.086 0.03 0.09 [0.03; 0.14] 21.2% 21.2%
STANLEY (344) 0.027 0.09 0.03 [−0.15; 0.20] 2.1% 2.1%
STANLEY (300) 0.096 0.08 0.10 [−0.06; 0.26] 2.5% 2.5%
VIS (902) 0.049 0.05 0.05 [−0.05; 0.15] 6.6% 6.6%
Common effect model 0.09 [0.06; 0.11] 100.0% --
Random effects model 0.09 [0.06; 0.11] -- 100.0%
−0.2 −0.1 0 0.1 0.2
Heterogeneity: I^2 = 0%, τ^2 = 0, p = 0.97

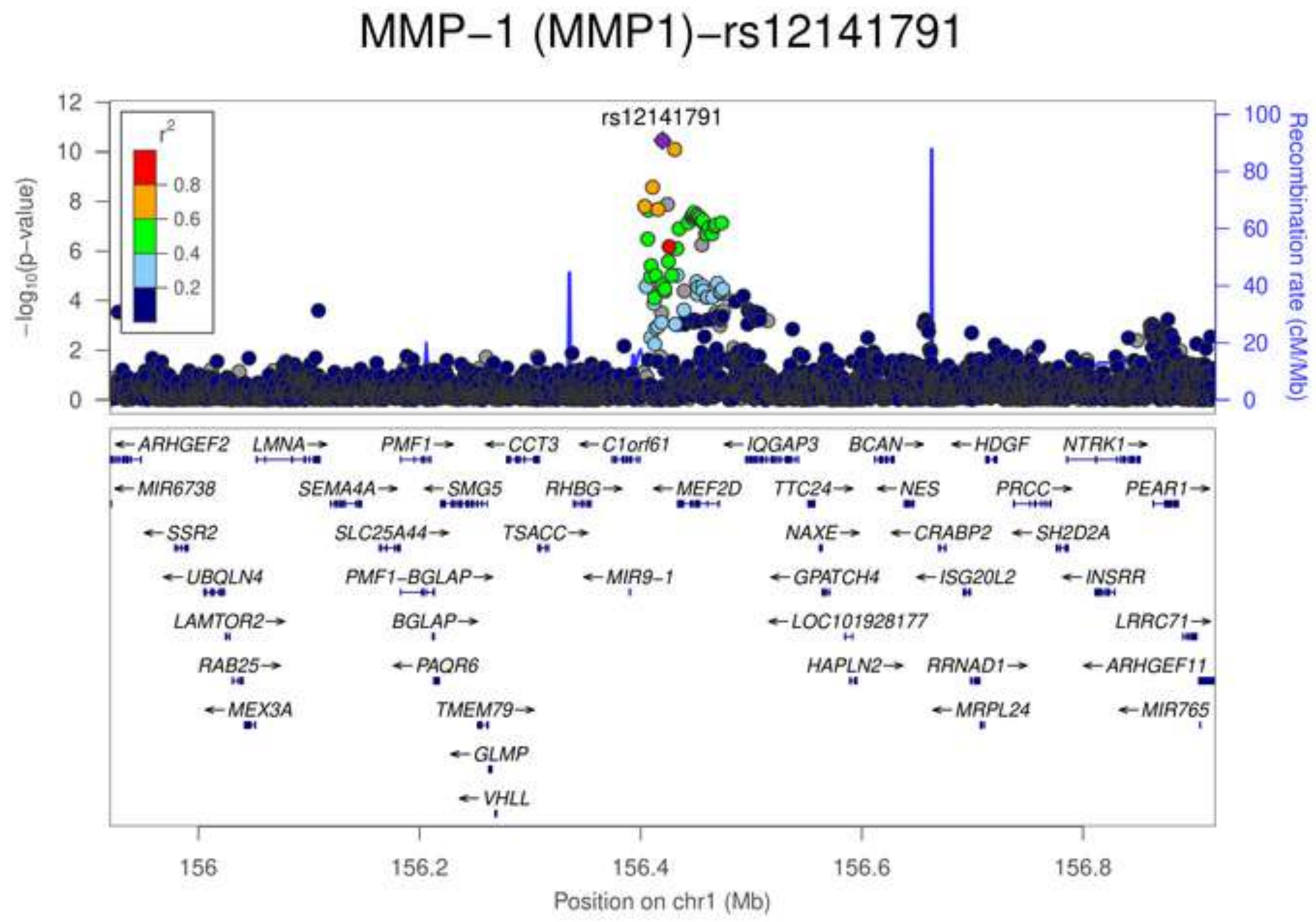
MMP−1 (MMP1)−rs12141791
rs12141791
$-\log_{10}$(p−value)
Recombination rate (cM/Mb)
r^2
0.8
0.6
0.4
0.2
ARHGEF2
LMNA
PMF1
CCT3
C1orf61
IQGAP3
BCAN
HDGF
NTRK1
MIR6738
SEMA4A
SMG5
RHBG
MEF2D
TTC24
NES
PRCC
PEAR1
SSR2
SLC25A44
TSACC
NAXE
CRABP2
SH2D2A
UBQLN4
PMF1−BGLAP
MIR9−1
GPATCH4
ISG20L2
INSRR
LAMTOR2
BGLAP
LOC101928177
LRRC71
RAB25
PAQR6
HAPLN2
RRNAD1
ARHGEF11
MEX3A
TMEM79
MRPL24
MIR765
GLMP
VHLL
156
156.2
156.4
156.6
156.8
Position on chr1 (Mb)

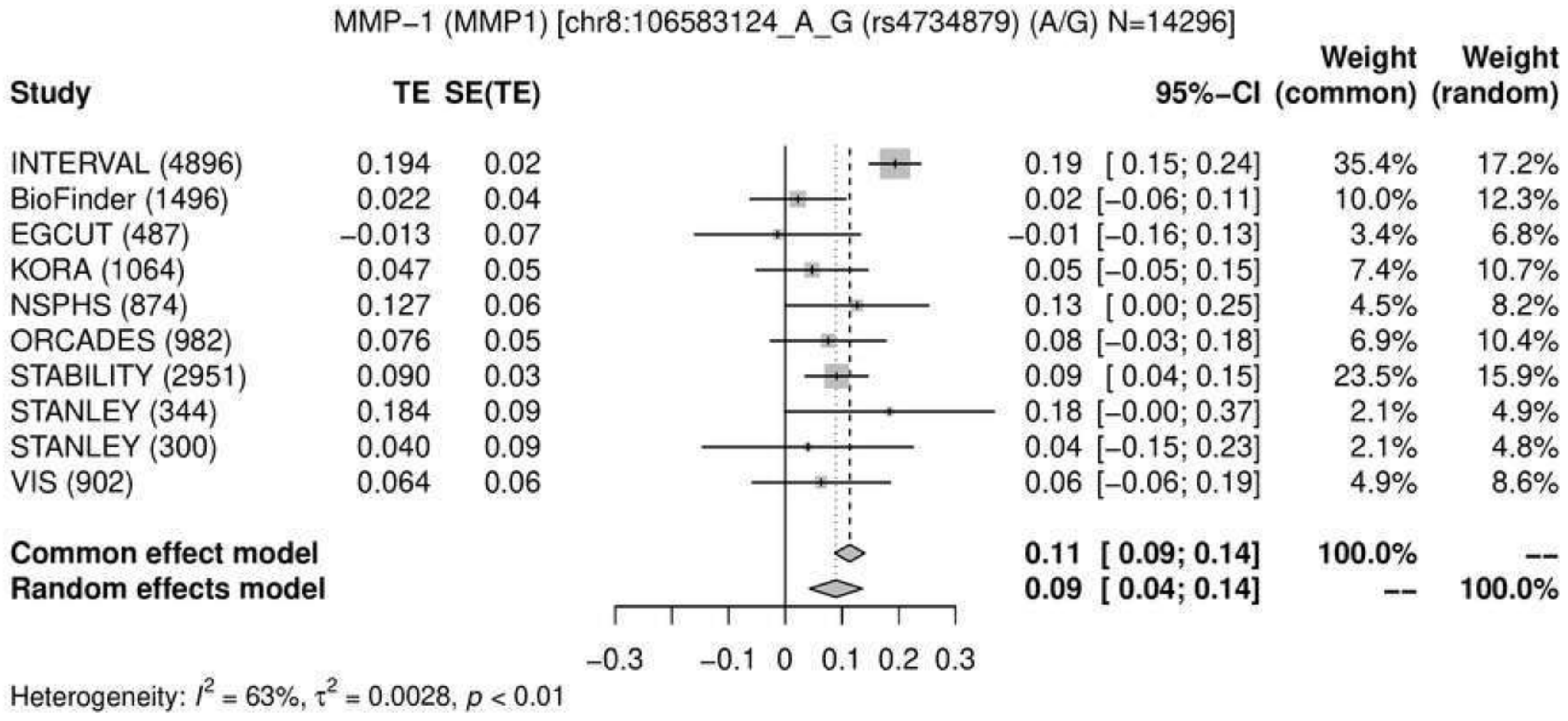
MMP−1 (MMP1) [chr8:106583124_A_G (rs4734879) (A/G) N=14296]
Study
TE
SE(TE)
95%−CI
Weight (common)
Weight (random)
INTERVAL (4896) 0.194 0.02 0.19 [0.15; 0.24] 35.4% 17.2%
BioFinder (1496) 0.022 0.04 0.02 [−0.06; 0.11] 10.0% 12.3%
EGCUT (487) −0.013 0.07 −0.01 [−0.16; 0.13] 3.4% 6.8%
KORA (1064) 0.047 0.05 0.05 [−0.05; 0.15] 7.4% 10.7%
NSPHS (874) 0.127 0.06 0.13 [0.00; 0.25] 4.5% 8.2%
ORCADES (982) 0.076 0.05 0.08 [−0.03; 0.18] 6.9% 10.4%
STABILITY (2951) 0.090 0.03 0.09 [0.04; 0.15] 23.5% 15.9%
STANLEY (344) 0.184 0.09 0.18 [−0.00; 0.37] 2.1% 4.9%
STANLEY (300) 0.040 0.09 0.04 [−0.15; 0.23] 2.1% 4.8%
VIS (902) 0.064 0.06 0.06 [−0.06; 0.19] 4.9% 8.6%
Common effect model 0.11 [0.09; 0.14] 100.0% --
Random effects model 0.09 [0.04; 0.14] -- 100.0%
−0.3 −0.1 0 0.1 0.2 0.3
Heterogeneity: I^2 = 63%, τ^2 = 0.0028, p < 0.01

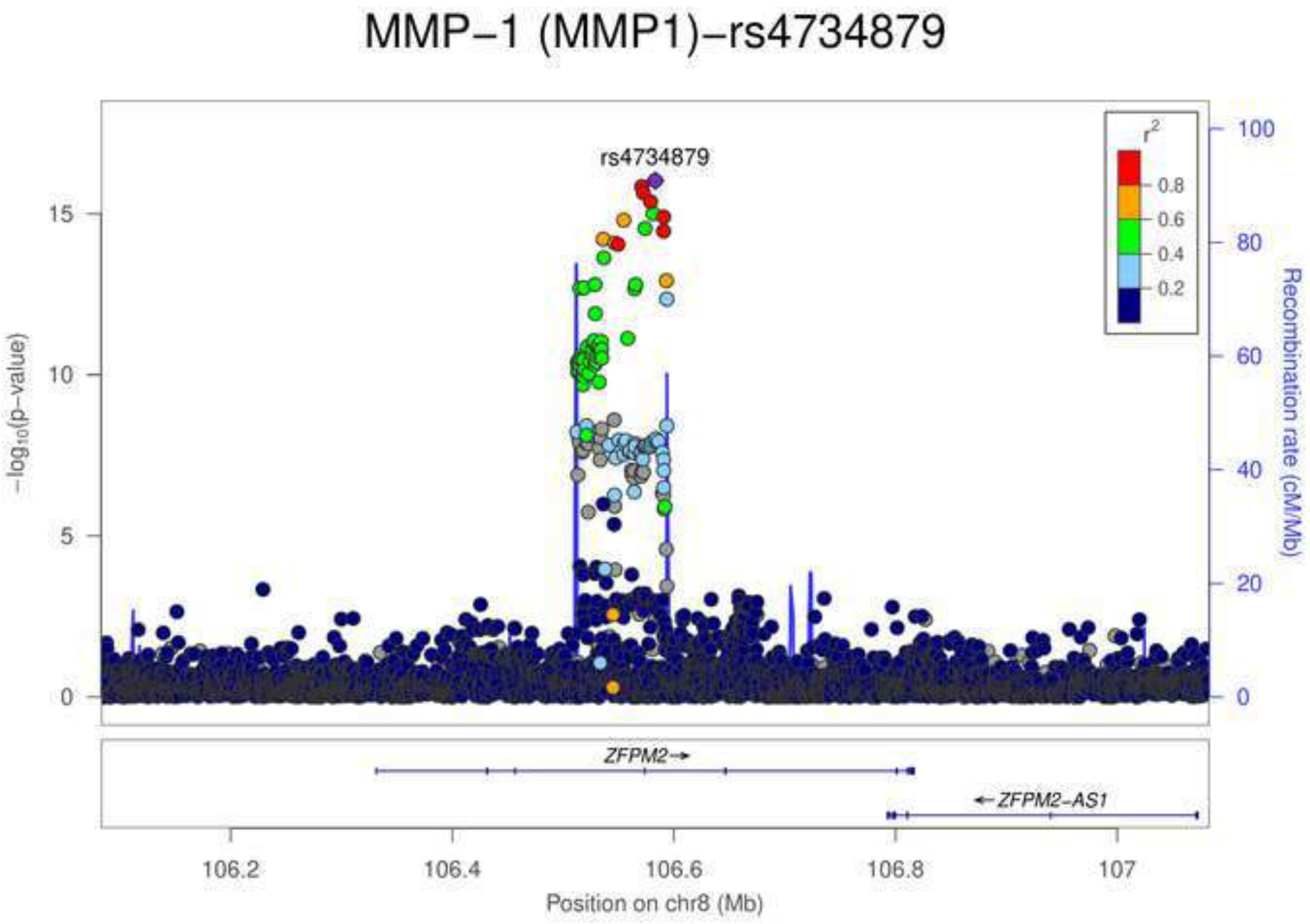
MMP−1 (MMP1)−rs4734879
rs4734879
r^2
0.8
0.6
0.4
0.2
$-\log_{10}$(p−value)
Recombination rate (cM/Mb)
ZFPM2→
←ZFPM2-AS1
106.2
106.4
106.6
106.8
107
Position on chr8 (Mb)

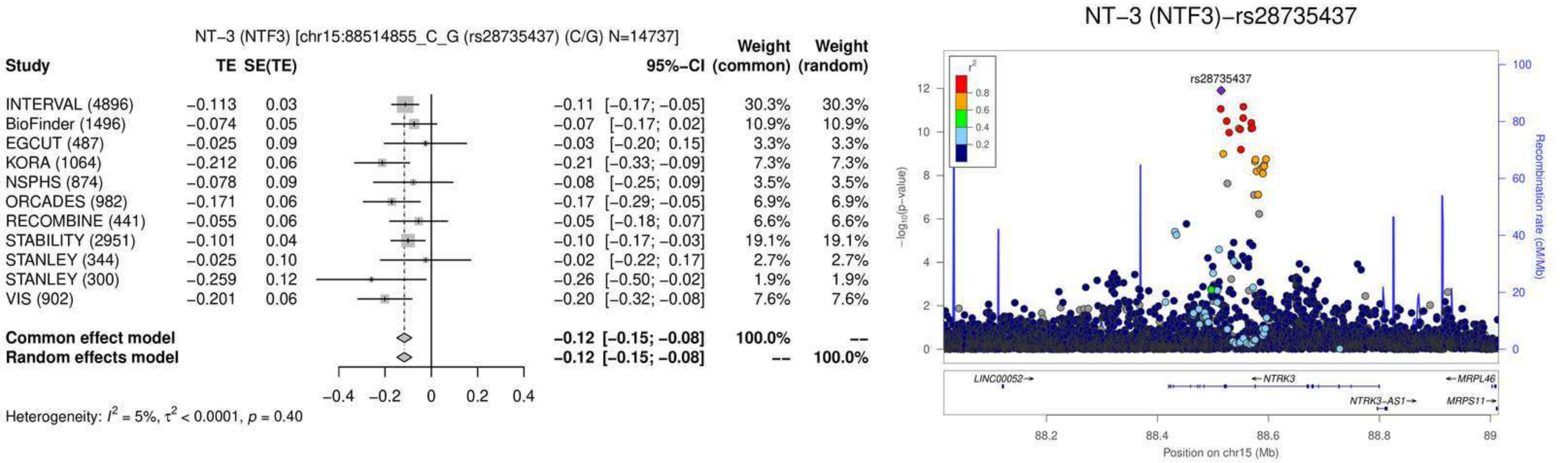

NT−3 (NTF3) [chr15:88514855_C_G (rs28735437) (C/G) N=14737]
Study
TE SE(TE)
95%−CI
Weight (common)
Weight (random)
INTERVAL (4896) −0.113 0.03 −0.11 [−0.17; −0.05] 30.3% 30.3%
BioFinder (1496) −0.074 0.05 −0.07 [−0.17; 0.02] 10.9% 10.9%
EGCUT (487) −0.025 0.09 −0.03 [−0.20; 0.15] 3.3% 3.3%
KORA (1064) −0.212 0.06 −0.21 [−0.33; −0.09] 7.3% 7.3%
NSPHS (874) −0.078 0.09 −0.08 [−0.25; 0.09] 3.5% 3.5%
ORCADES (982) −0.171 0.06 −0.17 [−0.29; −0.05] 6.9% 6.9%
RECOMBINE (441) −0.055 0.06 −0.05 [−0.18; 0.07] 6.6% 6.6%
STABILITY (2951) −0.101 0.04 −0.10 [−0.17; −0.03] 19.1% 19.1%
STANLEY (344) −0.025 0.10 −0.02 [−0.22; 0.17] 2.7% 2.7%
STANLEY (300) −0.259 0.12 −0.26 [−0.50; −0.02] 1.9% 1.9%
VIS (902) −0.201 0.06 −0.20 [−0.32; −0.08] 7.6% 7.6%
Common effect model −0.12 [−0.15; −0.08] 100.0% --
Random effects model −0.12 [−0.15; −0.08] -- 100.0%
−0.4 −0.2 0 0.2 0.4
Heterogeneity: I^2 = 5%, τ^2 < 0.0001, p = 0.40
NT−3 (NTF3)−rs28735437
rs28735437
r^2
0.8
0.6
0.4
0.2
$-\log_{10}$(p−value)
Recombination rate (cM/Mb)
LINC00052→
←NTRK3
←MRPL46
NTRK3−AS1→
MRPS11→
88.2 88.4 88.6 88.8 89
Position on chr15 (Mb)

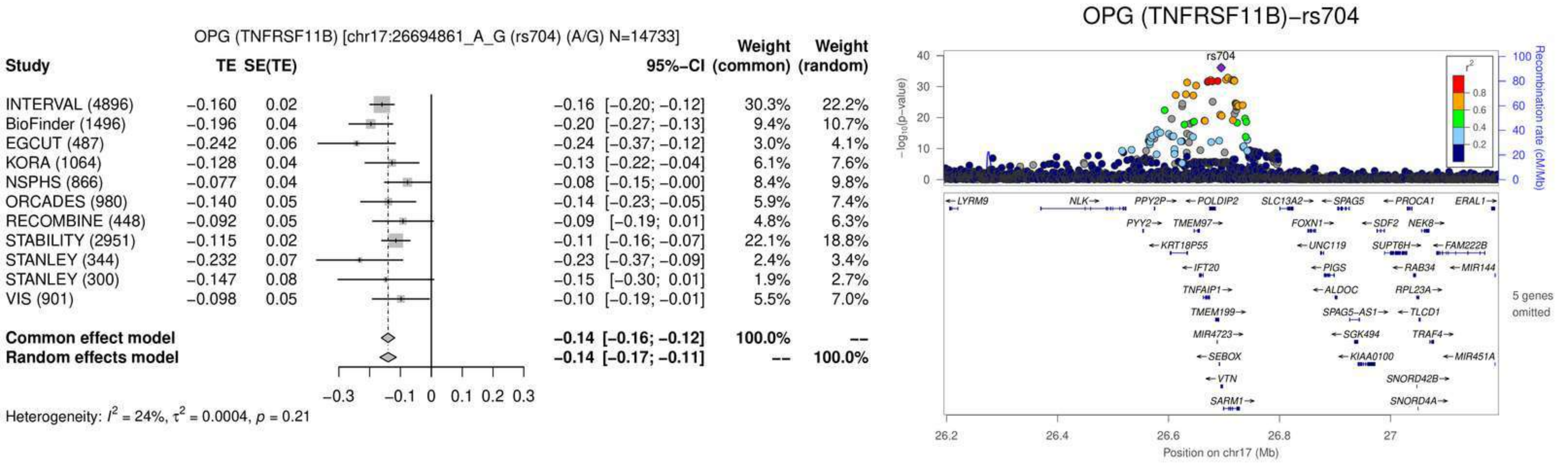
OPG (TNFRSF11B) [chr17:26694861_A_G (rs704) (A/G) N=14733]
Study
TE
SE(TE)
95%-CI
Weight (common)
Weight (random)
INTERVAL (4896) −0.160 0.02 −0.16 [−0.20; −0.12] 30.3% 22.2%
BioFinder (1496) −0.196 0.04 −0.20 [−0.27; −0.13] 9.4% 10.7%
EGCUT (487) −0.242 0.06 −0.24 [−0.37; −0.12] 3.0% 4.1%
KORA (1064) −0.128 0.04 −0.13 [−0.22; −0.04] 6.1% 7.6%
NSPHS (866) −0.077 0.04 −0.08 [−0.15; −0.00] 8.4% 9.8%
ORCADES (980) −0.140 0.05 −0.14 [−0.23; −0.05] 5.9% 7.4%
RECOMBINE (448) −0.092 0.05 −0.09 [−0.19; 0.01] 4.8% 6.3%
STABILITY (2951) −0.115 0.02 −0.11 [−0.16; −0.07] 22.1% 18.8%
STANLEY (344) −0.232 0.07 −0.23 [−0.37; −0.09] 2.4% 3.4%
STANLEY (300) −0.147 0.08 −0.15 [−0.30; 0.01] 1.9% 2.7%
VIS (901) −0.098 0.05 −0.10 [−0.19; −0.01] 5.5% 7.0%
Common effect model −0.14 [−0.16; −0.12] 100.0% --
Random effects model −0.14 [−0.17; −0.11] -- 100.0%
−0.3 −0.1 0 0.1 0.2 0.3
Heterogeneity: I^2 = 24%, τ^2 = 0.0004, p = 0.21
OPG (TNFRSF11B)−rs704
rs704
$-\log_{10}(p\text{-value})$
Recombination rate (cM/Mb)
r^2
0.8
0.6
0.4
0.2
LYRM9
NLK
PPY2P
POLDIP2
SLC13A2
SPAG5
PROCA1
ERAL1
PYY2
TMEM97
FOXN1
SDF2
NEK8
KRT18P55
UNC119
SUPT6H
FAM222B
IFT20
PIGS
RAB34
MIR144
TNFAIP1
ALDOC
RPL23A
TMEM199
SPAG5−AS1
TLCD1
MIR4723
SGK494
TRAF4
SEBOX
KIAA0100
MIR451A
VTN
SNORD42B
SARM1
SNORD4A
5 genes omitted
26.2 26.4 26.6 26.8 27
Position on chr17 (Mb)

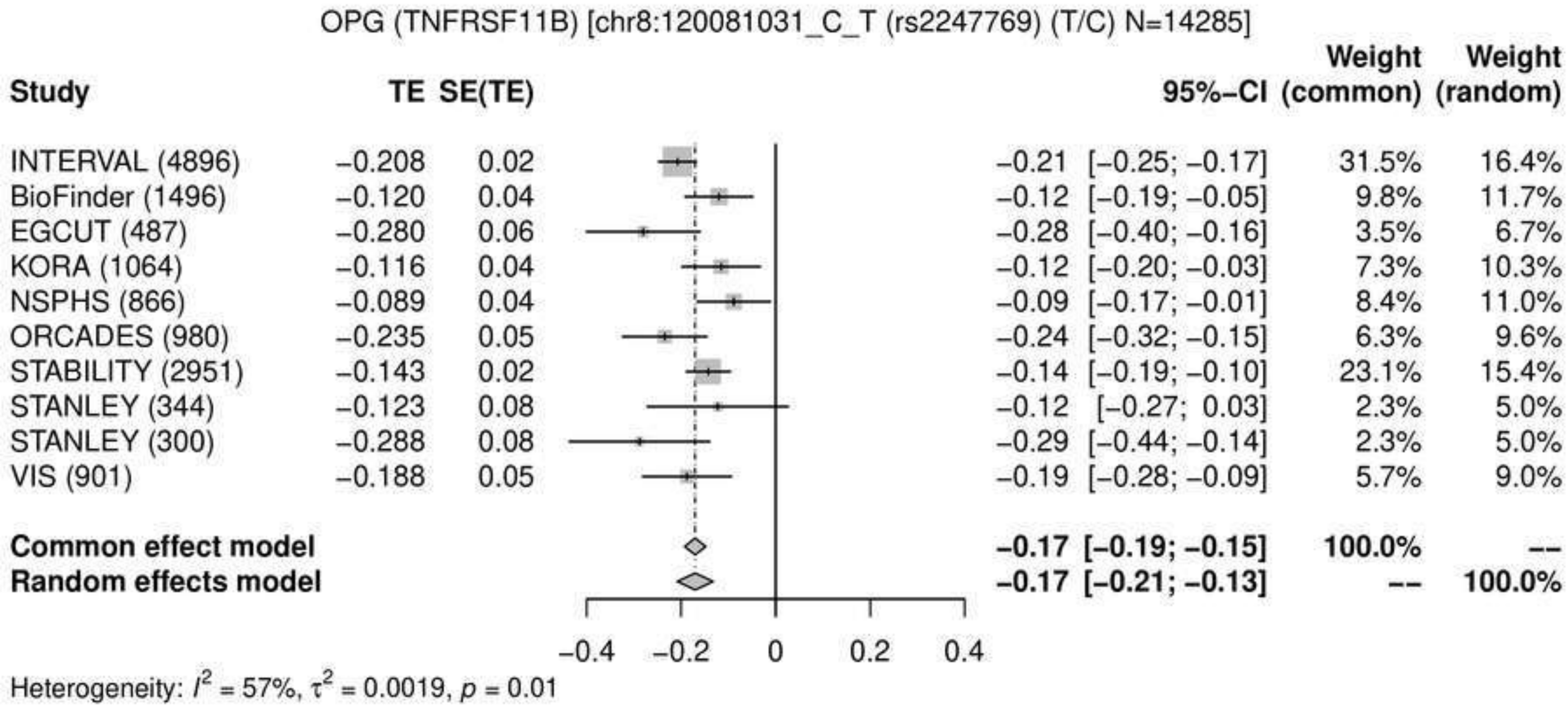
OPG (TNFRSF11B) [chr8:120081031_C_T (rs2247769) (T/C) N=14285]
Study
TE
SE(TE)
95%−CI
Weight (common)
Weight (random)
INTERVAL (4896) −0.208 0.02 −0.21 [−0.25; −0.17] 31.5% 16.4%
BioFinder (1496) −0.120 0.04 −0.12 [−0.19; −0.05] 9.8% 11.7%
EGCUT (487) −0.280 0.06 −0.28 [−0.40; −0.16] 3.5% 6.7%
KORA (1064) −0.116 0.04 −0.12 [−0.20; −0.03] 7.3% 10.3%
NSPHS (866) −0.089 0.04 −0.09 [−0.17; −0.01] 8.4% 11.0%
ORCADES (980) −0.235 0.05 −0.24 [−0.32; −0.15] 6.3% 9.6%
STABILITY (2951) −0.143 0.02 −0.14 [−0.19; −0.10] 23.1% 15.4%
STANLEY (344) −0.123 0.08 −0.12 [−0.27; 0.03] 2.3% 5.0%
STANLEY (300) −0.288 0.08 −0.29 [−0.44; −0.14] 2.3% 5.0%
VIS (901) −0.188 0.05 −0.19 [−0.28; −0.09] 5.7% 9.0%
Common effect model −0.17 [−0.19; −0.15] 100.0% --
Random effects model −0.17 [−0.21; −0.13] -- 100.0%
−0.4
−0.2
0
0.2
0.4
Heterogeneity: I^2 = 57%, τ^2 = 0.0019, p = 0.01

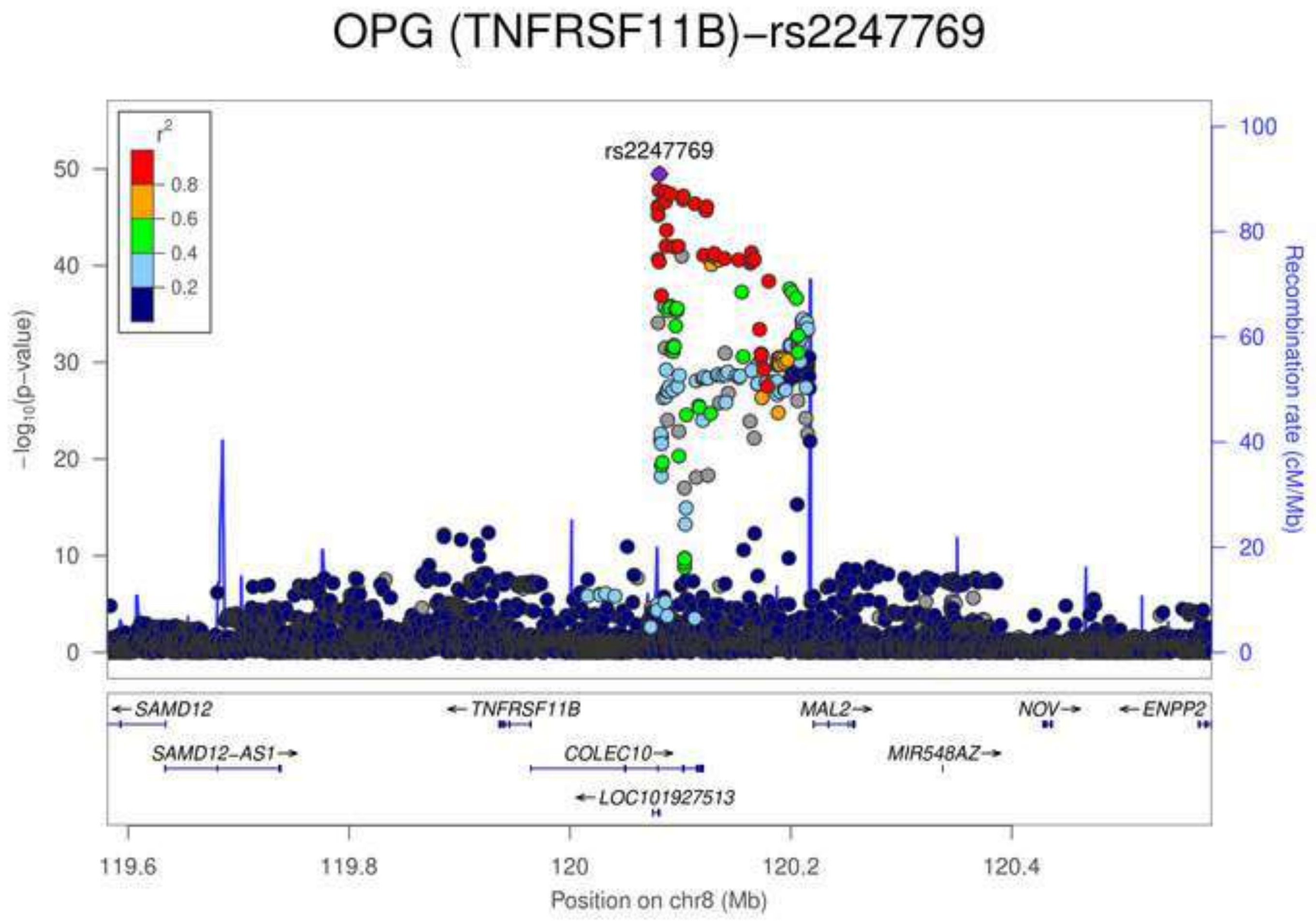
OPG (TNFRSF11B)−rs2247769
rs2247769
r^2
0.8
0.6
0.4
0.2
$-\log_{10}$(p−value)
Recombination rate (cM/Mb)
SAMD12
SAMD12−AS1
TNFRSF11B
COLEC10
LOC101927513
MAL2
MIR548AZ
NOV
ENPP2
119.6
119.8
120
120.2
120.4
Position on chr8 (Mb)

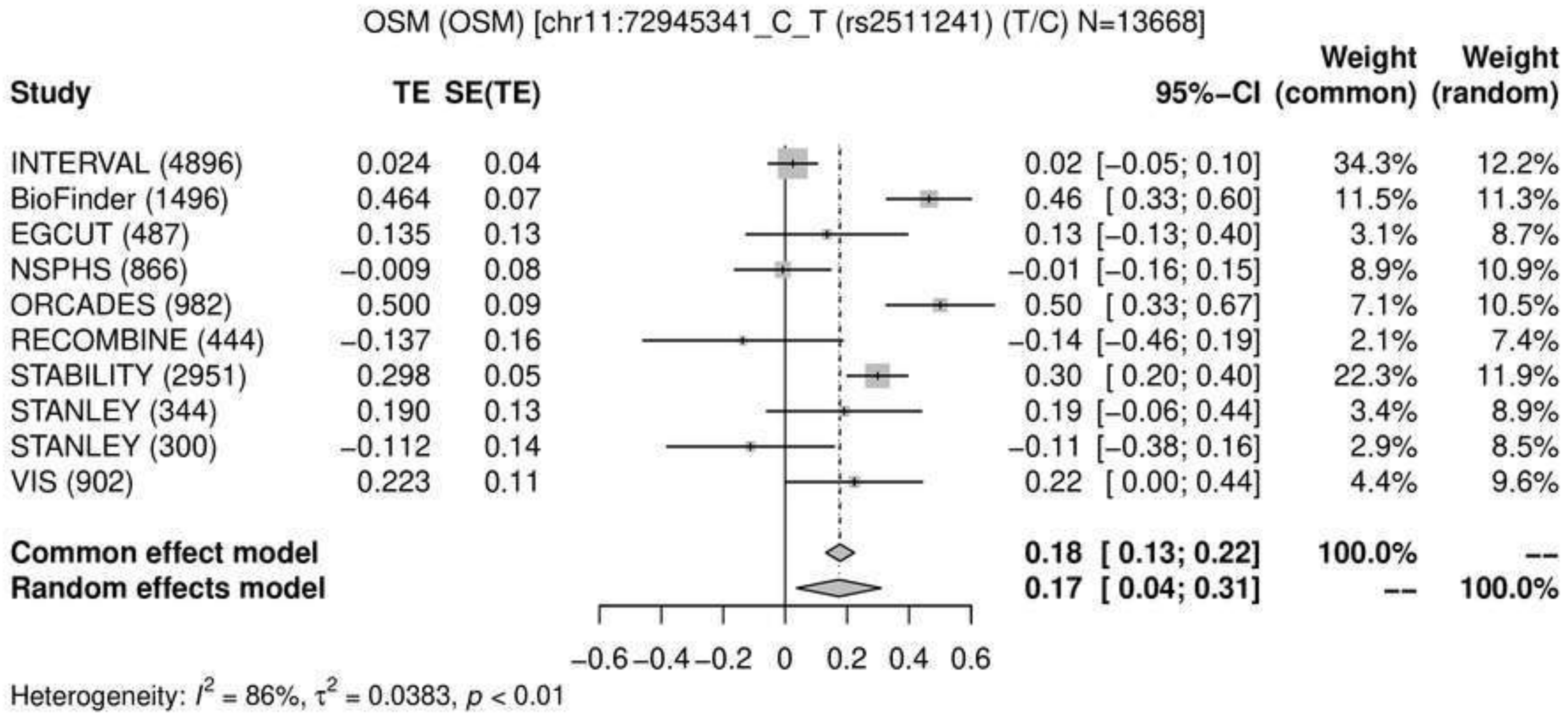
OSM (OSM) [chr11:72945341_C_T (rs2511241) (T/C) N=13668]
Study
TE
SE(TE)
95%-CI
Weight (common)
Weight (random)
INTERVAL (4896) 0.024 0.04 0.02 [−0.05; 0.10] 34.3% 12.2%
BioFinder (1496) 0.464 0.07 0.46 [0.33; 0.60] 11.5% 11.3%
EGCUT (487) 0.135 0.13 0.13 [−0.13; 0.40] 3.1% 8.7%
NSPHS (866) −0.009 0.08 −0.01 [−0.16; 0.15] 8.9% 10.9%
ORCADES (982) 0.500 0.09 0.50 [0.33; 0.67] 7.1% 10.5%
RECOMBINE (444) −0.137 0.16 −0.14 [−0.46; 0.19] 2.1% 7.4%
STABILITY (2951) 0.298 0.05 0.30 [0.20; 0.40] 22.3% 11.9%
STANLEY (344) 0.190 0.13 0.19 [−0.06; 0.44] 3.4% 8.9%
STANLEY (300) −0.112 0.14 −0.11 [−0.38; 0.16] 2.9% 8.5%
VIS (902) 0.223 0.11 0.22 [0.00; 0.44] 4.4% 9.6%
Common effect model 0.18 [0.13; 0.22] 100.0% --
Random effects model 0.17 [0.04; 0.31] -- 100.0%
−0.6 −0.4 −0.2 0 0.2 0.4 0.6
Heterogeneity: I^2 = 86%, τ^2 = 0.0383, p < 0.01

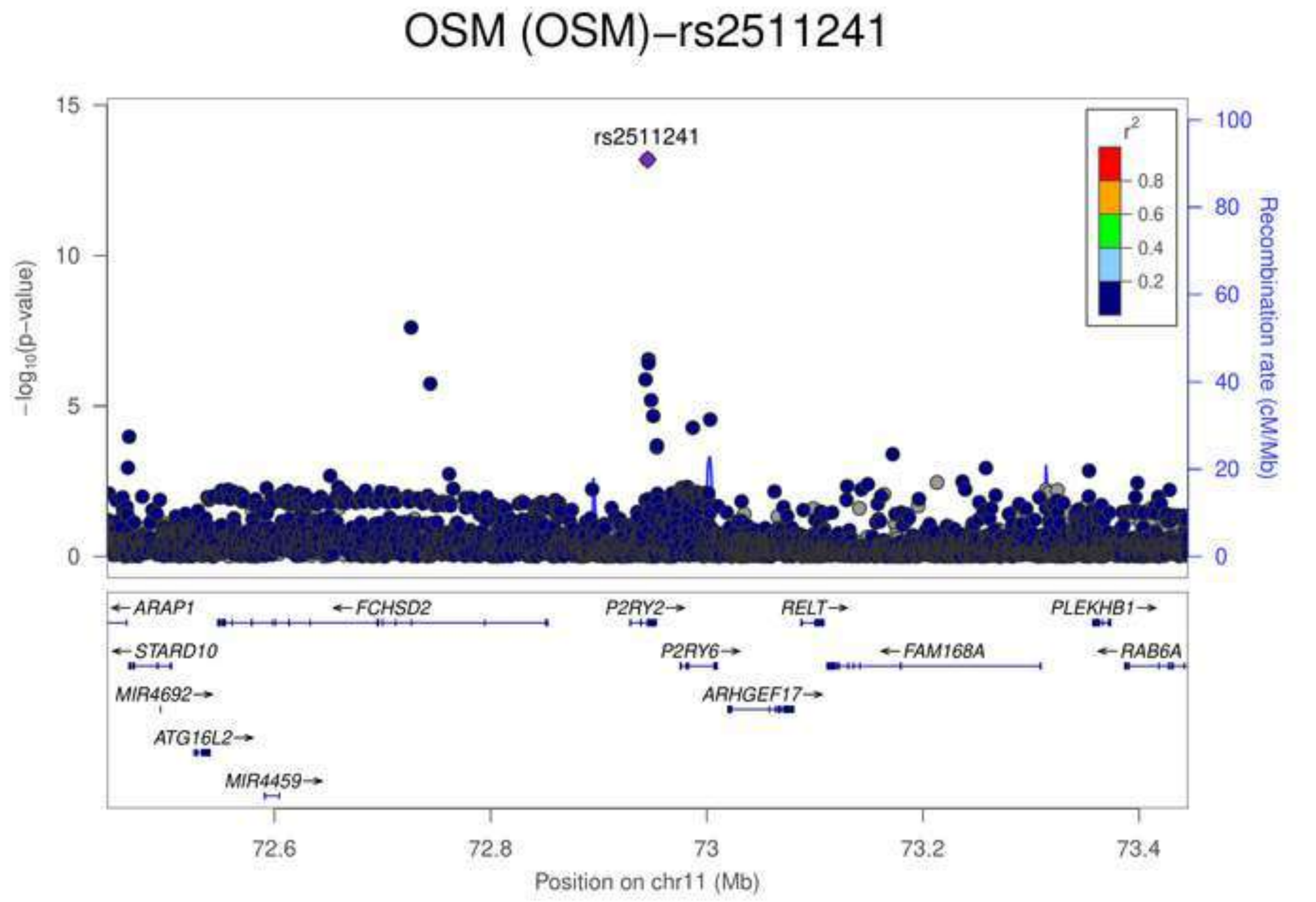
OSM (OSM)−rs2511241
rs2511241
$-\log_{10}$(p-value)
Recombination rate (cM/Mb)
r^2
0.8
0.6
0.4
0.2
15
10
5
0
100
80
60
40
20
0
←ARAP1
←FCHSD2
P2RY2→
RELT→
PLEKHB1→
←STARD10
P2RY6→
←FAM168A
←RAB6A
MIR4692→
ARHGEF17→
ATG16L2→
MIR4459→
72.6
72.8
73
73.2
73.4
Position on chr11 (Mb)

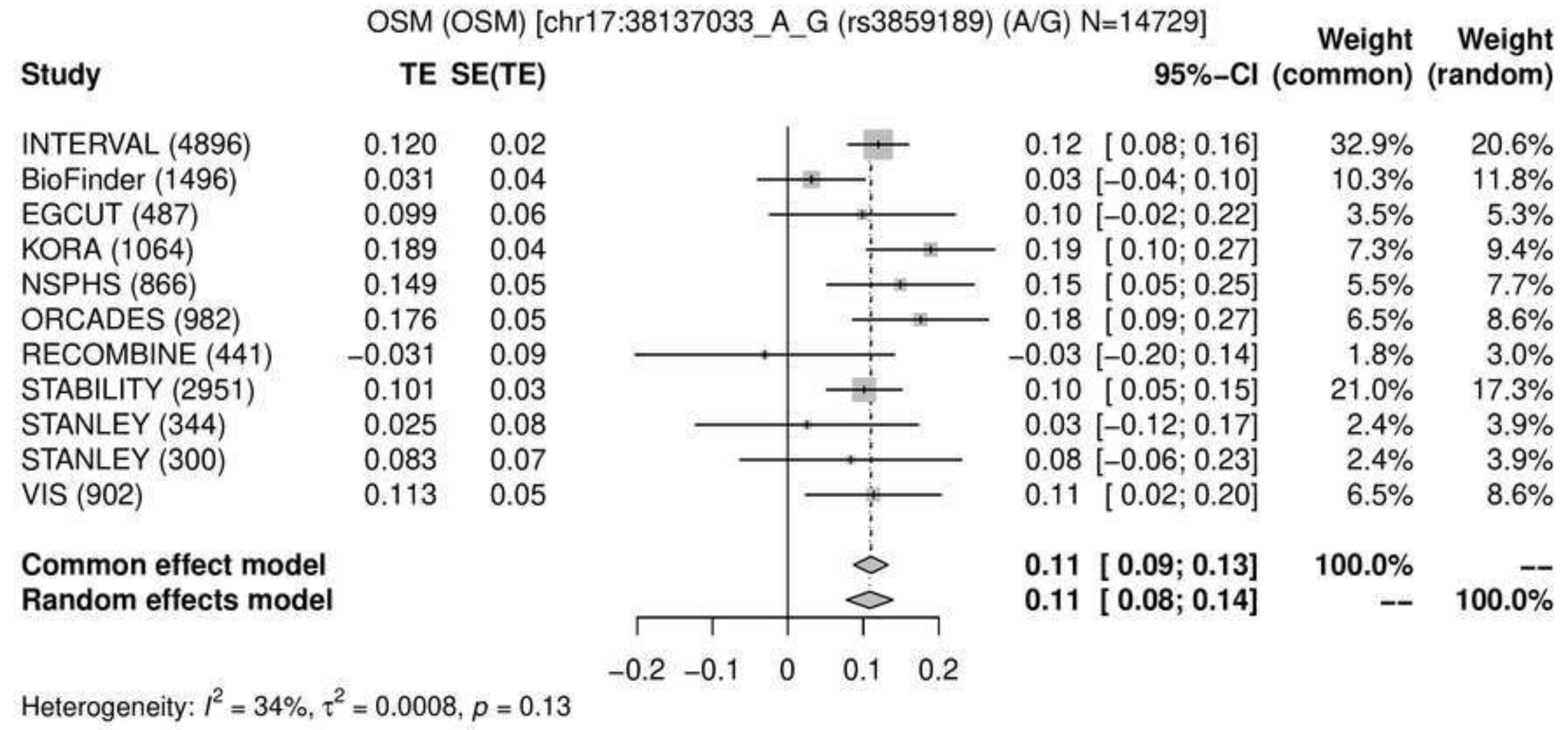
OSM (OSM) [chr17:38137033_A_G (rs3859189) (A/G) N=14729]
Study
TE
SE(TE)
95%-CI
Weight (common)
Weight (random)
INTERVAL (4896) 0.120 0.02 0.12 [0.08; 0.16] 32.9% 20.6%
BioFinder (1496) 0.031 0.04 0.03 [−0.04; 0.10] 10.3% 11.8%
EGCUT (487) 0.099 0.06 0.10 [−0.02; 0.22] 3.5% 5.3%
KORA (1064) 0.189 0.04 0.19 [0.10; 0.27] 7.3% 9.4%
NSPHS (866) 0.149 0.05 0.15 [0.05; 0.25] 5.5% 7.7%
ORCADES (982) 0.176 0.05 0.18 [0.09; 0.27] 6.5% 8.6%
RECOMBINE (441) −0.031 0.09 −0.03 [−0.20; 0.14] 1.8% 3.0%
STABILITY (2951) 0.101 0.03 0.10 [0.05; 0.15] 21.0% 17.3%
STANLEY (344) 0.025 0.08 0.03 [−0.12; 0.17] 2.4% 3.9%
STANLEY (300) 0.083 0.07 0.08 [−0.06; 0.23] 2.4% 3.9%
VIS (902) 0.113 0.05 0.11 [0.02; 0.20] 6.5% 8.6%
Common effect model 0.11 [0.09; 0.13] 100.0% --
Random effects model 0.11 [0.08; 0.14] -- 100.0%
−0.2 −0.1 0 0.1 0.2
Heterogeneity: I^2 = 34%, τ^2 = 0.0008, p = 0.13

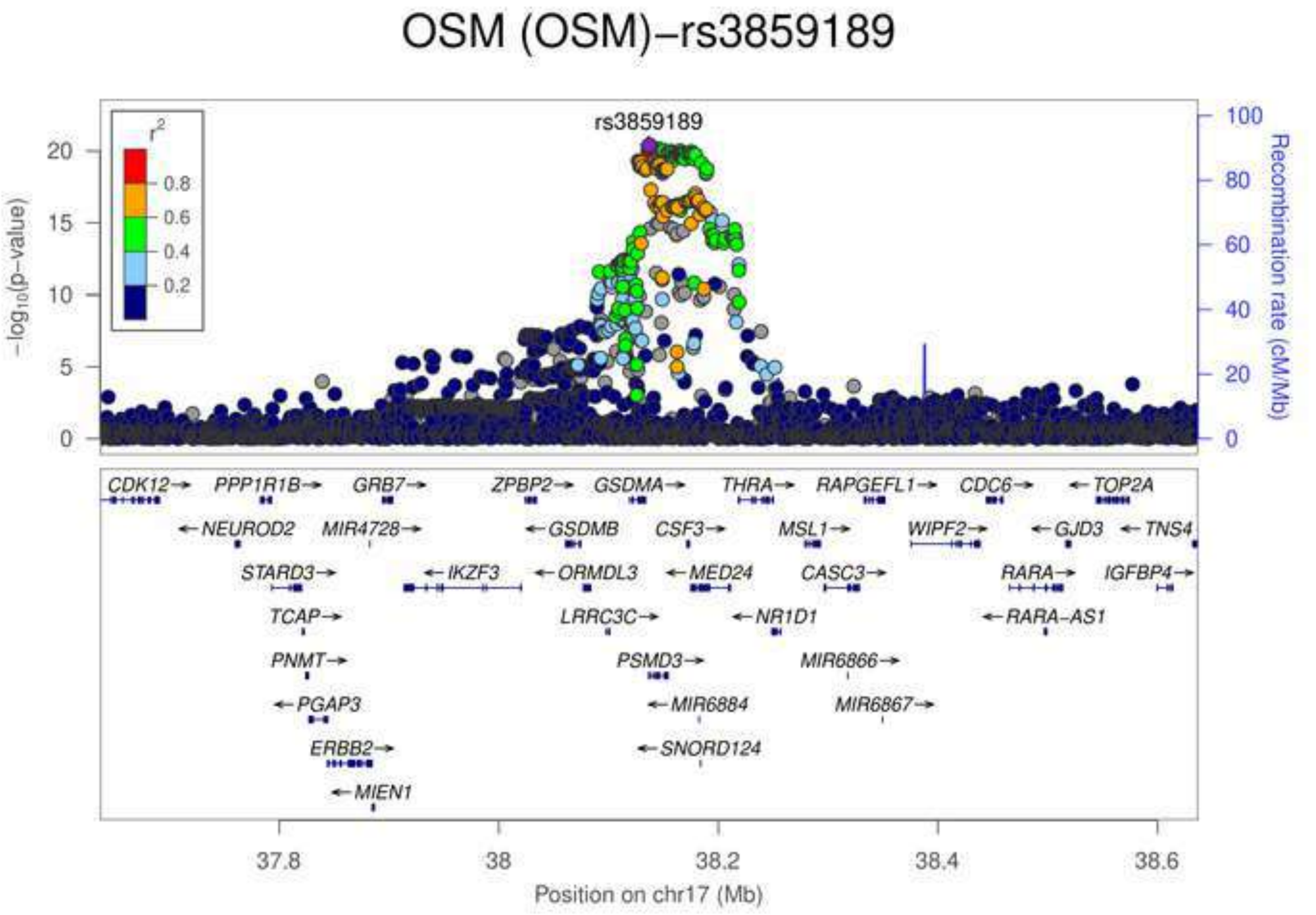
OSM (OSM)−rs3859189
rs3859189
r^2
0.8
0.6
0.4
0.2
$-\log_{10}$(p-value)
Recombination rate (cM/Mb)
CDK12 PPP1R1B GRB7 ZPBP2 GSDMA THRA RAPGEFL1 CDC6 TOP2A
NEUROD2 MIR4728 GSDMB CSF3 MSL1 WIPF2 GJD3 TNS4
STARD3 IKZF3 ORMDL3 MED24 CASC3 RARA IGFBP4
TCAP LRRC3C NR1D1 RARA-AS1
PNMT PSMD3 MIR6866
PGAP3 MIR6884 MIR6867
ERBB2 SNORD124
MIEN1
37.8 38 38.2 38.4 38.6
Position on chr17 (Mb)

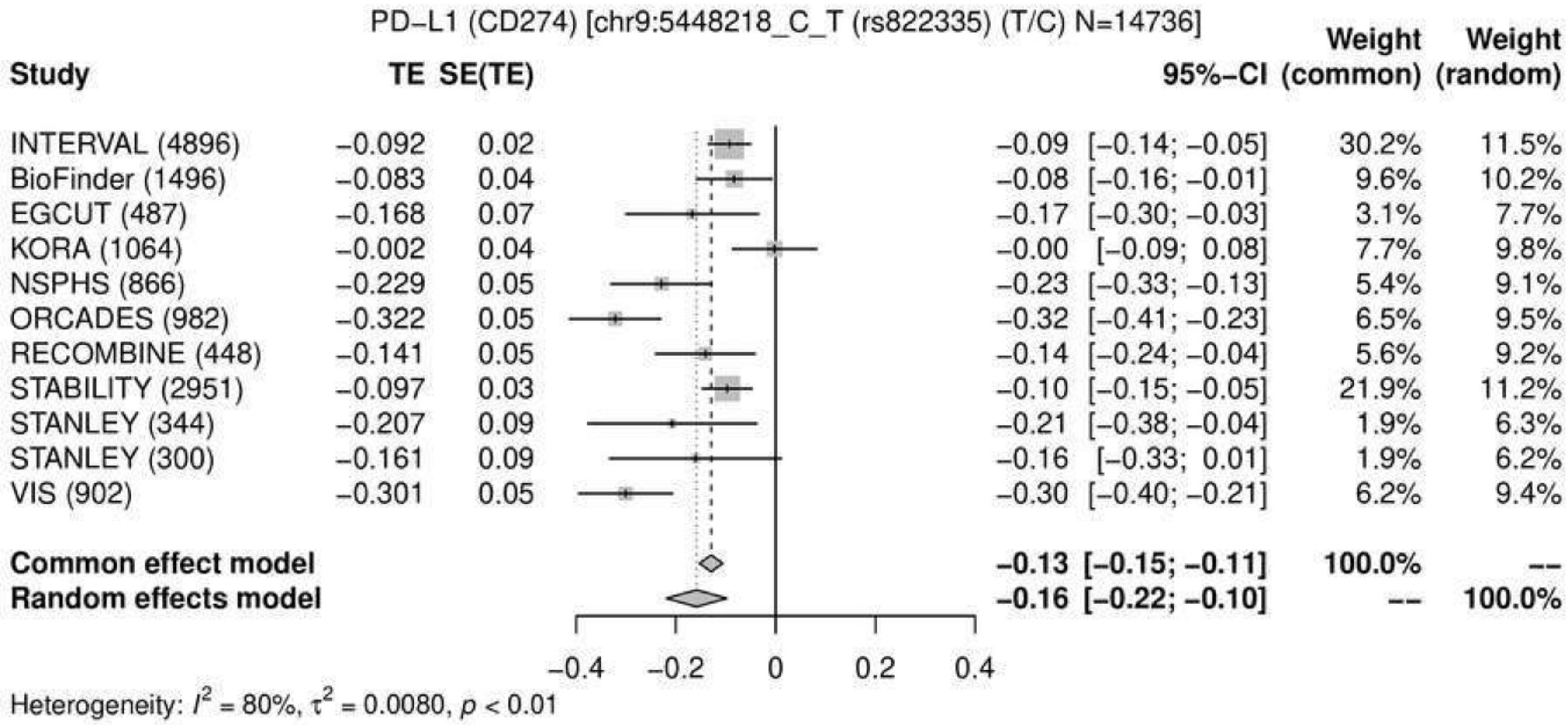
PD−L1 (CD274) [chr9:5448218_C_T (rs822335) (T/C) N=14736]
Study
TE
SE(TE)
95%−CI
Weight (common)
Weight (random)
INTERVAL (4896) −0.092 0.02 −0.09 [−0.14; −0.05] 30.2% 11.5%
BioFinder (1496) −0.083 0.04 −0.08 [−0.16; −0.01] 9.6% 10.2%
EGCUT (487) −0.168 0.07 −0.17 [−0.30; −0.03] 3.1% 7.7%
KORA (1064) −0.002 0.04 −0.00 [−0.09; 0.08] 7.7% 9.8%
NSPHS (866) −0.229 0.05 −0.23 [−0.33; −0.13] 5.4% 9.1%
ORCADES (982) −0.322 0.05 −0.32 [−0.41; −0.23] 6.5% 9.5%
RECOMBINE (448) −0.141 0.05 −0.14 [−0.24; −0.04] 5.6% 9.2%
STABILITY (2951) −0.097 0.03 −0.10 [−0.15; −0.05] 21.9% 11.2%
STANLEY (344) −0.207 0.09 −0.21 [−0.38; −0.04] 1.9% 6.3%
STANLEY (300) −0.161 0.09 −0.16 [−0.33; 0.01] 1.9% 6.2%
VIS (902) −0.301 0.05 −0.30 [−0.40; −0.21] 6.2% 9.4%
Common effect model −0.13 [−0.15; −0.11] 100.0% --
Random effects model −0.16 [−0.22; −0.10] -- 100.0%
−0.4 −0.2 0 0.2 0.4
Heterogeneity: I^2 = 80%, τ^2 = 0.0080, p < 0.01

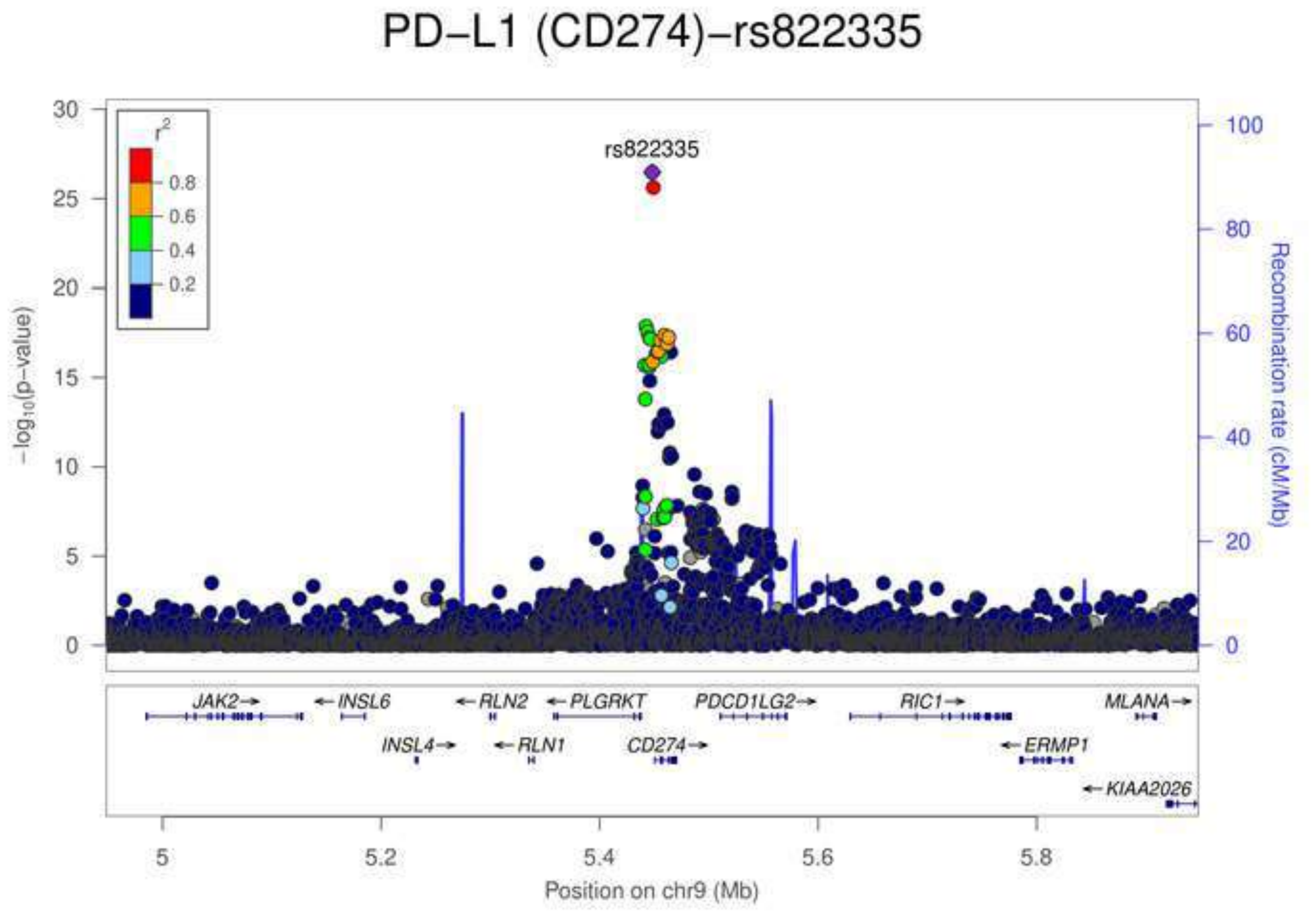
PD−L1 (CD274)−rs822335
rs822335
r^2
0.8
0.6
0.4
0.2
−log10(p−value)
Recombination rate (cM/Mb)
0 5 10 15 20 25 30
0 20 40 60 80 100
JAK2
INSL6
RLN2
PLGRKT
PDCD1LG2
RIC1
MLANA
INSL4
RLN1
CD274
ERMP1
KIAA2026
5 5.2 5.4 5.6 5.8
Position on chr9 (Mb)

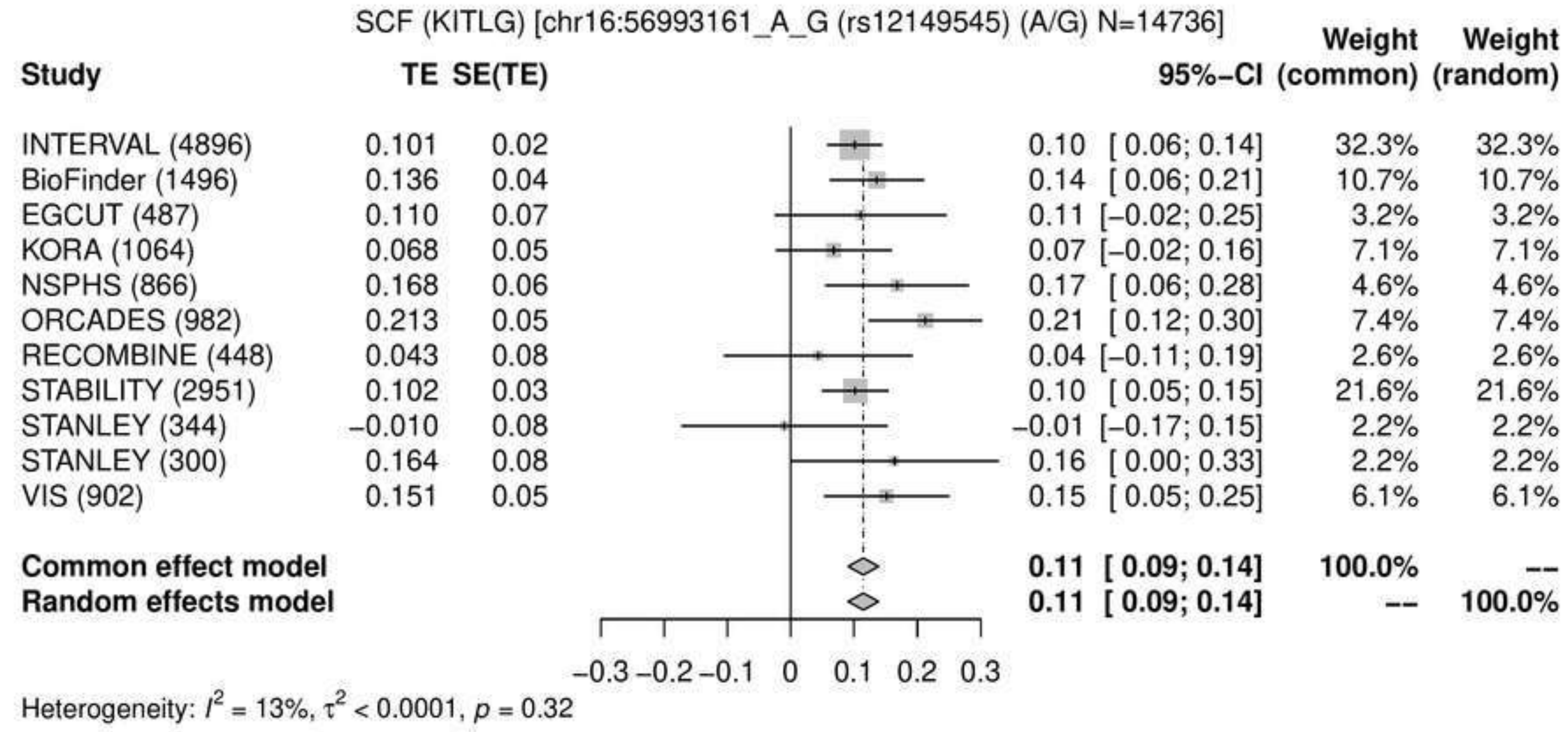
SCF (KITLG) [chr16:56993161_A_G (rs12149545) (A/G) N=14736]
Study
TE
SE(TE)
95%-CI
Weight (common)
Weight (random)
INTERVAL (4896) 0.101 0.02 0.10 [0.06; 0.14] 32.3% 32.3%
BioFinder (1496) 0.136 0.04 0.14 [0.06; 0.21] 10.7% 10.7%
EGCUT (487) 0.110 0.07 0.11 [−0.02; 0.25] 3.2% 3.2%
KORA (1064) 0.068 0.05 0.07 [−0.02; 0.16] 7.1% 7.1%
NSPHS (866) 0.168 0.06 0.17 [0.06; 0.28] 4.6% 4.6%
ORCADES (982) 0.213 0.05 0.21 [0.12; 0.30] 7.4% 7.4%
RECOMBINE (448) 0.043 0.08 0.04 [−0.11; 0.19] 2.6% 2.6%
STABILITY (2951) 0.102 0.03 0.10 [0.05; 0.15] 21.6% 21.6%
STANLEY (344) −0.010 0.08 −0.01 [−0.17; 0.15] 2.2% 2.2%
STANLEY (300) 0.164 0.08 0.16 [0.00; 0.33] 2.2% 2.2%
VIS (902) 0.151 0.05 0.15 [0.05; 0.25] 6.1% 6.1%
Common effect model 0.11 [0.09; 0.14] 100.0% --
Random effects model 0.11 [0.09; 0.14] -- 100.0%
−0.3 −0.2 −0.1 0 0.1 0.2 0.3
Heterogeneity: I^2 = 13%, τ^2 < 0.0001, p = 0.32
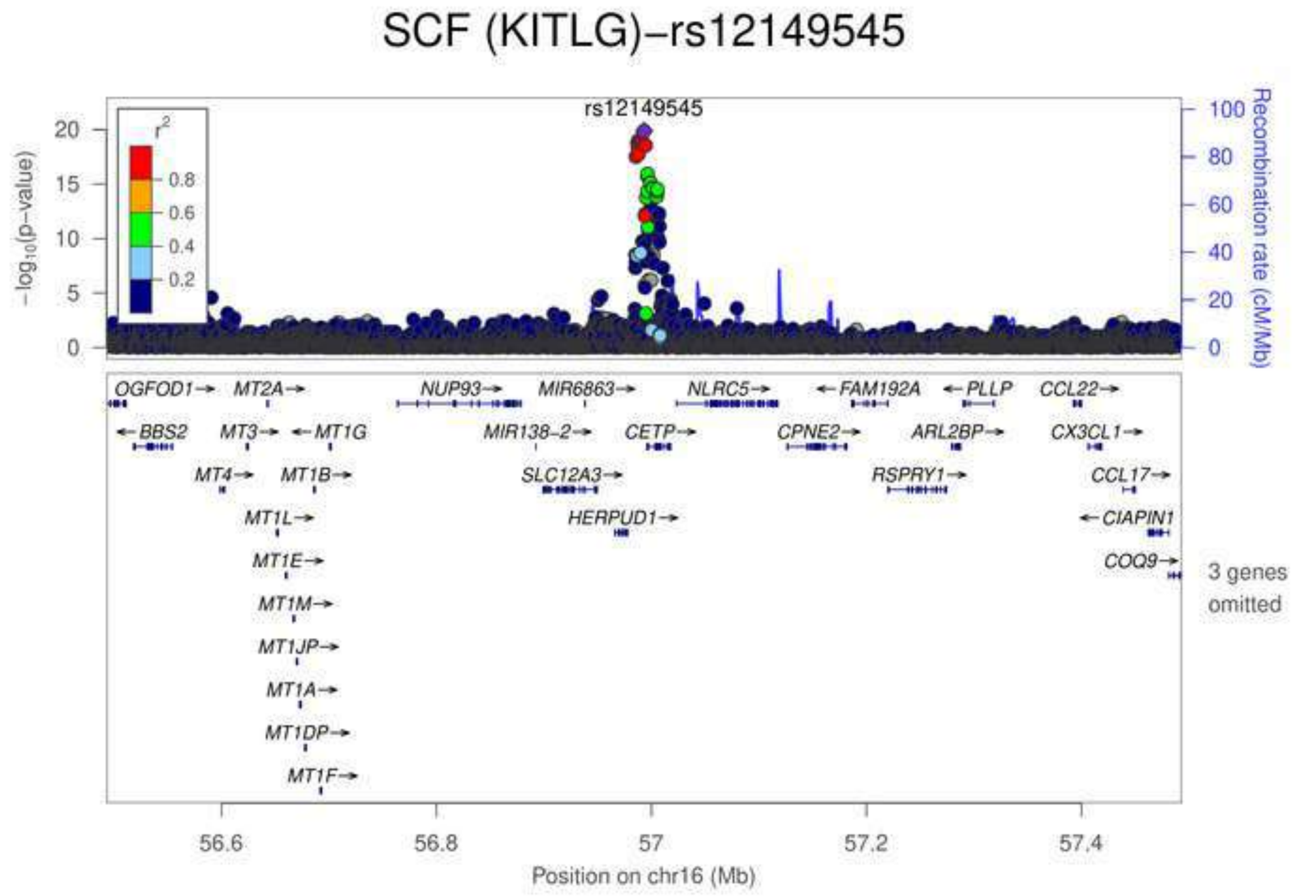
SCF (KITLG)−rs12149545
rs12149545
r^2
0.8
0.6
0.4
0.2
$-\log_{10}(p\text{-value})$
Recombination rate (cM/Mb)
OGFOD1
MT2A
NUP93
MIR6863
NLRC5
FAM192A
PLLP
CCL22
BBS2
MT3
MT1G
MIR138−2
CETP
CPNE2
ARL2BP
CX3CL1
MT4
MT1B
SLC12A3
RSPRY1
CCL17
MT1L
HERPUD1
CIAPIN1
MT1E
COQ9
3 genes omitted
MT1M
MT1JP
MT1A
MT1DP
MT1F
56.6
56.8
57
57.2
57.4
Position on chr16 (Mb)

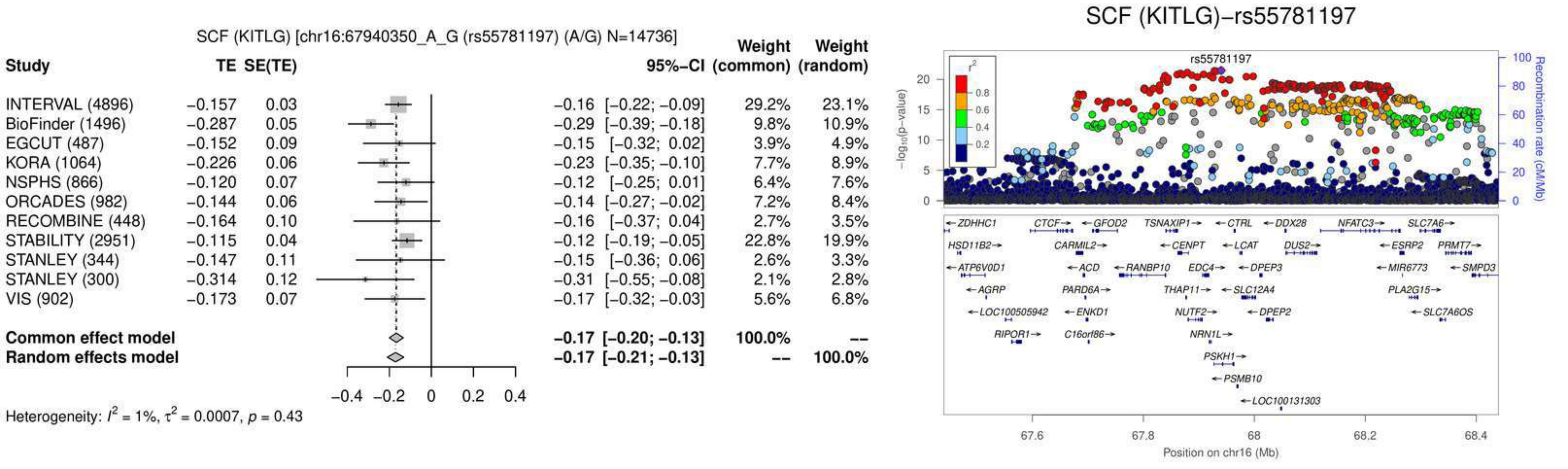
SCF (KITLG) [chr16:67940350_A_G (rs55781197) (A/G) N=14736]
Study
TE
SE(TE)
95%-CI
Weight (common)
Weight (random)
INTERVAL (4896) −0.157 0.03 −0.16 [−0.22; −0.09] 29.2% 23.1%
BioFinder (1496) −0.287 0.05 −0.29 [−0.39; −0.18] 9.8% 10.9%
EGCUT (487) −0.152 0.09 −0.15 [−0.32; 0.02] 3.9% 4.9%
KORA (1064) −0.226 0.06 −0.23 [−0.35; −0.10] 7.7% 8.9%
NSPHS (866) −0.120 0.07 −0.12 [−0.25; 0.01] 6.4% 7.6%
ORCADES (982) −0.144 0.06 −0.14 [−0.27; −0.02] 7.2% 8.4%
RECOMBINE (448) −0.164 0.10 −0.16 [−0.37; 0.04] 2.7% 3.5%
STABILITY (2951) −0.115 0.04 −0.12 [−0.19; −0.05] 22.8% 19.9%
STANLEY (344) −0.147 0.11 −0.15 [−0.36; 0.06] 2.6% 3.3%
STANLEY (300) −0.314 0.12 −0.31 [−0.55; −0.08] 2.1% 2.8%
VIS (902) −0.173 0.07 −0.17 [−0.32; −0.03] 5.6% 6.8%
Common effect model −0.17 [−0.20; −0.13] 100.0% --
Random effects model −0.17 [−0.21; −0.13] -- 100.0%
−0.4 −0.2 0 0.2 0.4
Heterogeneity: I^2 = 1%, τ^2 = 0.0007, p = 0.43
SCF (KITLG)−rs55781197
rs55781197
r^2
0.8
0.6
0.4
0.2
$-\log_{10}$(p-value)
Recombination rate (cM/Mb)
ZDHHC1
CTCF
GFOD2
TSNAXIP1
CTRL
DDX28
NFATC3
SLC7A6
HSD11B2
CARMIL2
CENPT
LCAT
DUS2
ESRP2
PRMT7
ATP6V0D1
ACD
RANBP10
EDC4
DPEP3
MIR6773
SMPD3
AGRP
PARD6A
THAP11
SLC12A4
PLA2G15
LOC100505942
ENKD1
NUTF2
DPEP2
SLC7A6OS
RIPOR1
C16orf86
NRN1L
PSKH1
PSMB10
LOC100131303
67.6
67.8
68
68.2
68.4
Position on chr16 (Mb)

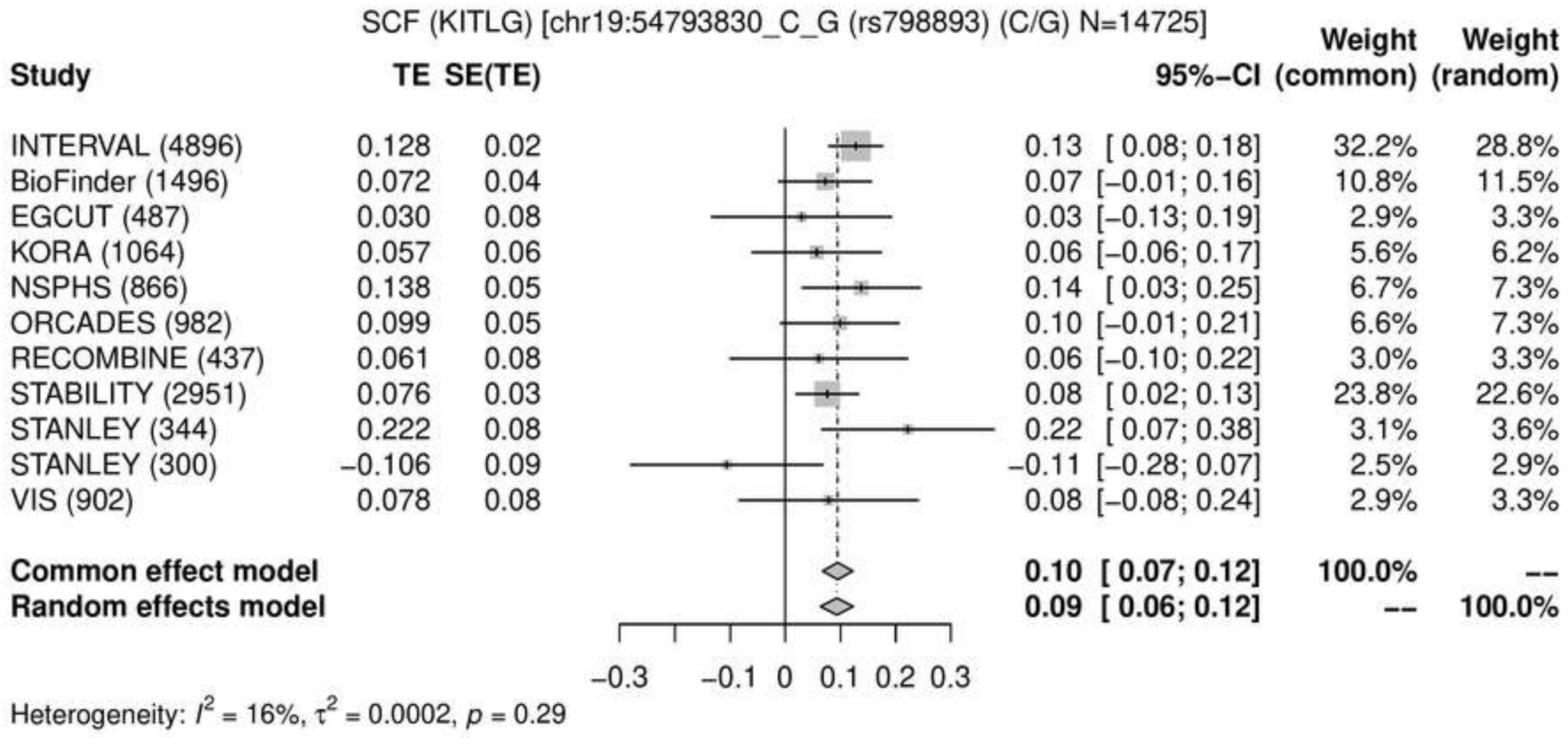
SCF (KITLG) [chr19:54793830_C_G (rs798893) (C/G) N=14725]
Study
TE
SE(TE)
95%-CI
Weight (common)
Weight (random)
INTERVAL (4896) 0.128 0.02 0.13 [0.08; 0.18] 32.2% 28.8%
BioFinder (1496) 0.072 0.04 0.07 [−0.01; 0.16] 10.8% 11.5%
EGCUT (487) 0.030 0.08 0.03 [−0.13; 0.19] 2.9% 3.3%
KORA (1064) 0.057 0.06 0.06 [−0.06; 0.17] 5.6% 6.2%
NSPHS (866) 0.138 0.05 0.14 [0.03; 0.25] 6.7% 7.3%
ORCADES (982) 0.099 0.05 0.10 [−0.01; 0.21] 6.6% 7.3%
RECOMBINE (437) 0.061 0.08 0.06 [−0.10; 0.22] 3.0% 3.3%
STABILITY (2951) 0.076 0.03 0.08 [0.02; 0.13] 23.8% 22.6%
STANLEY (344) 0.222 0.08 0.22 [0.07; 0.38] 3.1% 3.6%
STANLEY (300) −0.106 0.09 −0.11 [−0.28; 0.07] 2.5% 2.9%
VIS (902) 0.078 0.08 0.08 [−0.08; 0.24] 2.9% 3.3%
Common effect model 0.10 [0.07; 0.12] 100.0% --
Random effects model 0.09 [0.06; 0.12] -- 100.0%
−0.3 −0.1 0 0.1 0.2 0.3
Heterogeneity: I^2 = 16%, τ^2 = 0.0002, p = 0.29

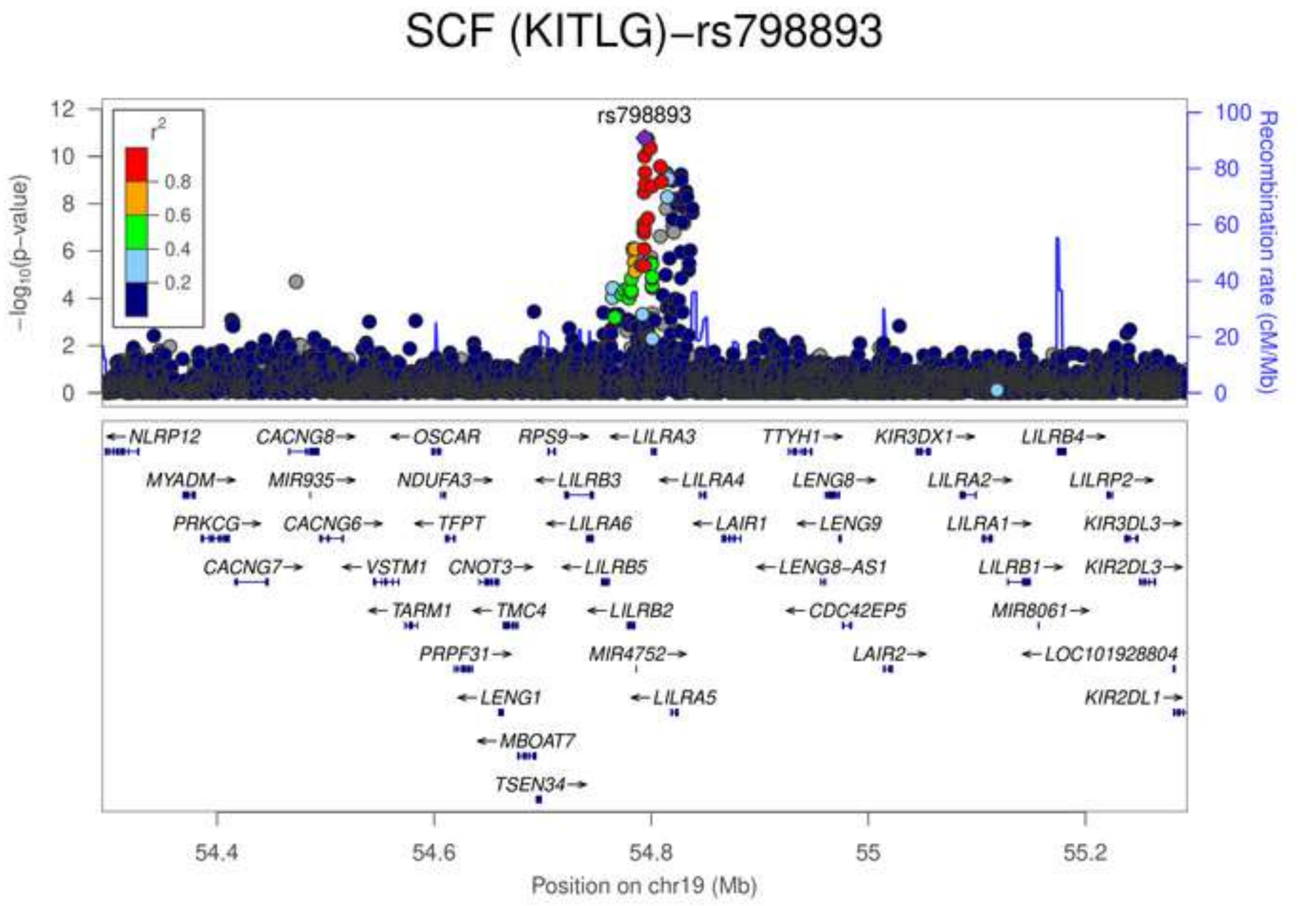
SCF (KITLG)−rs798893
rs798893
r^2
0.8
0.6
0.4
0.2
$-\log_{10}$(p−value)
Recombination rate (cM/Mb)
NLRP12
CACNG8
OSCAR
RPS9
LILRA3
TTYH1
KIR3DX1
LILRB4
MYADM
MIR935
NDUFA3
LILRB3
LILRA4
LENG8
LILRA2
LILRP2
PRKCG
CACNG6
TFPT
LILRA6
LAIR1
LENG9
LILRA1
KIR3DL3
CACNG7
VSTM1
CNOT3
LILRB5
LENG8−AS1
LILRB1
KIR2DL3
TARM1
TMC4
LILRB2
CDC42EP5
MIR8061
PRPF31
MIR4752
LAIR2
LOC101928804
LENG1
LILRA5
KIR2DL1
MBOAT7
TSEN34
54.4
54.6
54.8
55
55.2
Position on chr19 (Mb)

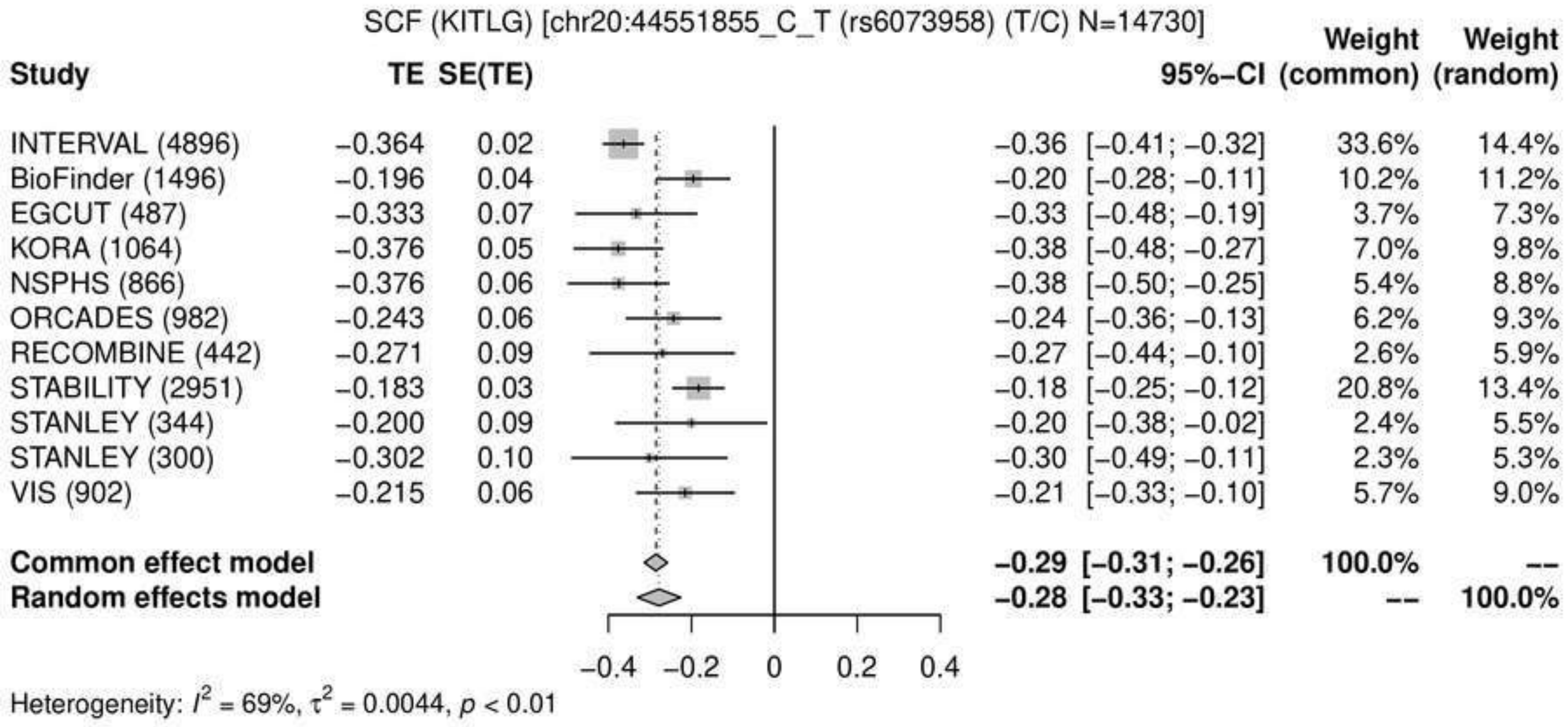
SCF (KITLG) [chr20:44551855_C_T (rs6073958) (T/C) N=14730]
Study | TE | SE(TE) | 95%-CI | Weight (common) | Weight (random)
INTERVAL (4896) | −0.364 | 0.02 | −0.36 [−0.41; −0.32] | 33.6% | 14.4%
BioFinder (1496) | −0.196 | 0.04 | −0.20 [−0.28; −0.11] | 10.2% | 11.2%
EGCUT (487) | −0.333 | 0.07 | −0.33 [−0.48; −0.19] | 3.7% | 7.3%
KORA (1064) | −0.376 | 0.05 | −0.38 [−0.48; −0.27] | 7.0% | 9.8%
NSPHS (866) | −0.376 | 0.06 | −0.38 [−0.50; −0.25] | 5.4% | 8.8%
ORCADES (982) | −0.243 | 0.06 | −0.24 [−0.36; −0.13] | 6.2% | 9.3%
RECOMBINE (442) | −0.271 | 0.09 | −0.27 [−0.44; −0.10] | 2.6% | 5.9%
STABILITY (2951) | −0.183 | 0.03 | −0.18 [−0.25; −0.12] | 20.8% | 13.4%
STANLEY (344) | −0.200 | 0.09 | −0.20 [−0.38; −0.02] | 2.4% | 5.5%
STANLEY (300) | −0.302 | 0.10 | −0.30 [−0.49; −0.11] | 2.3% | 5.3%
VIS (902) | −0.215 | 0.06 | −0.21 [−0.33; −0.10] | 5.7% | 9.0%
Common effect model | −0.29 [−0.31; −0.26] | 100.0% | --
Random effects model | −0.28 [−0.33; −0.23] | -- | 100.0%
−0.4 −0.2 0 0.2 0.4
Heterogeneity: I^2 = 69%, τ^2 = 0.0044, p < 0.01

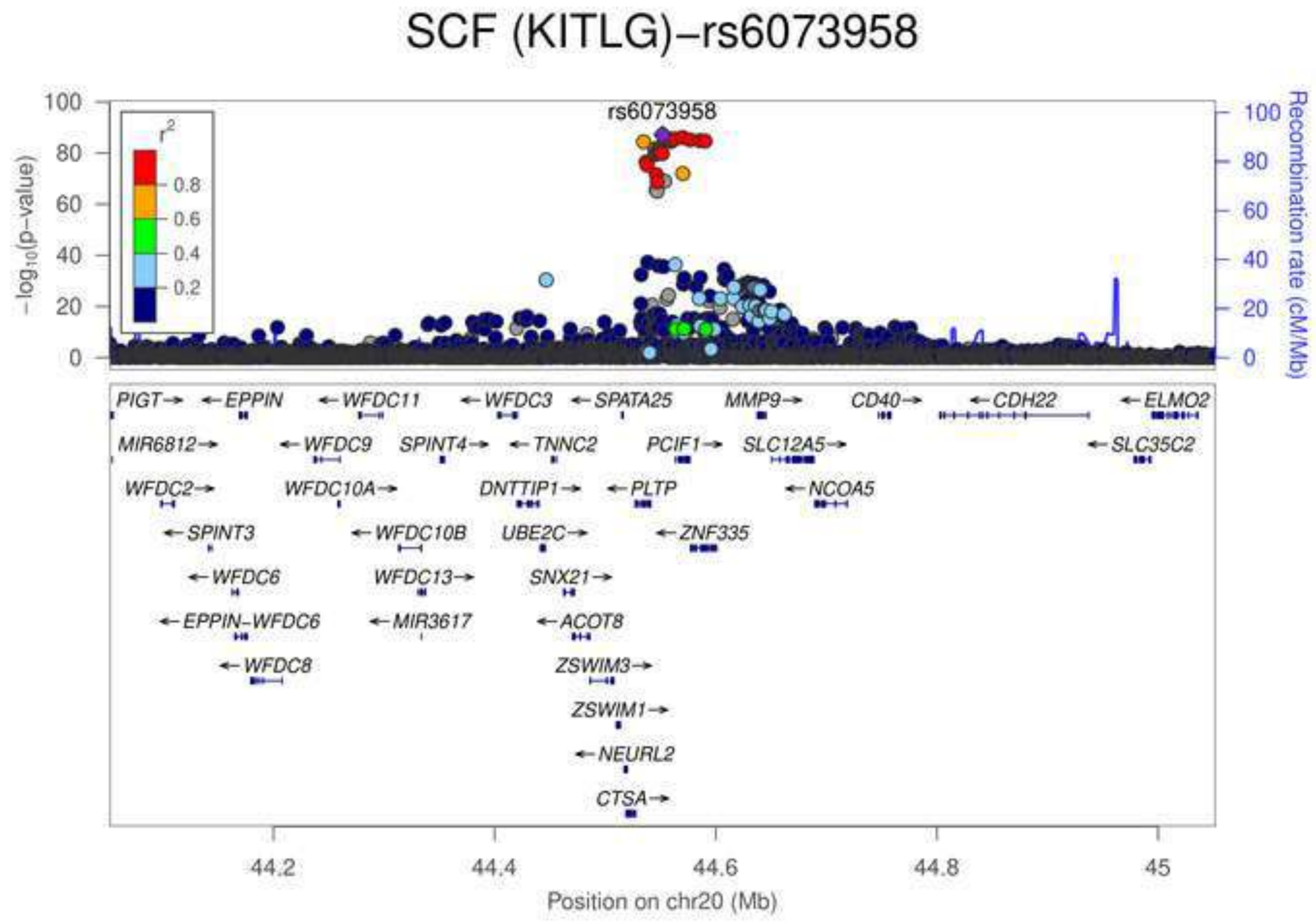
SCF (KITLG)−rs6073958
rs6073958
r^2
0.8
0.6
0.4
0.2
$-\log_{10}$(p−value)
Recombination rate (cM/Mb)
PIGT→ ←EPPIN ←WFDC11 ←WFDC3 ←SPATA25 MMP9→ CD40→ ←CDH22 ←ELMO2
MIR6812→ ←WFDC9 SPINT4→ ←TNNC2 PCIF1→ SLC12A5→ ←SLC35C2
WFDC2→ WFDC10A→ DNTTIP1→ ←PLTP ←NCOA5
←SPINT3 ←WFDC10B UBE2C→ ←ZNF335
←WFDC6 WFDC13→ SNX21→
←EPPIN−WFDC6 ←MIR3617 ←ACOT8
←WFDC8 ZSWIM3→
ZSWIM1→
←NEURL2
CTSA→
44.2 44.4 44.6 44.8 45
Position on chr20 (Mb)

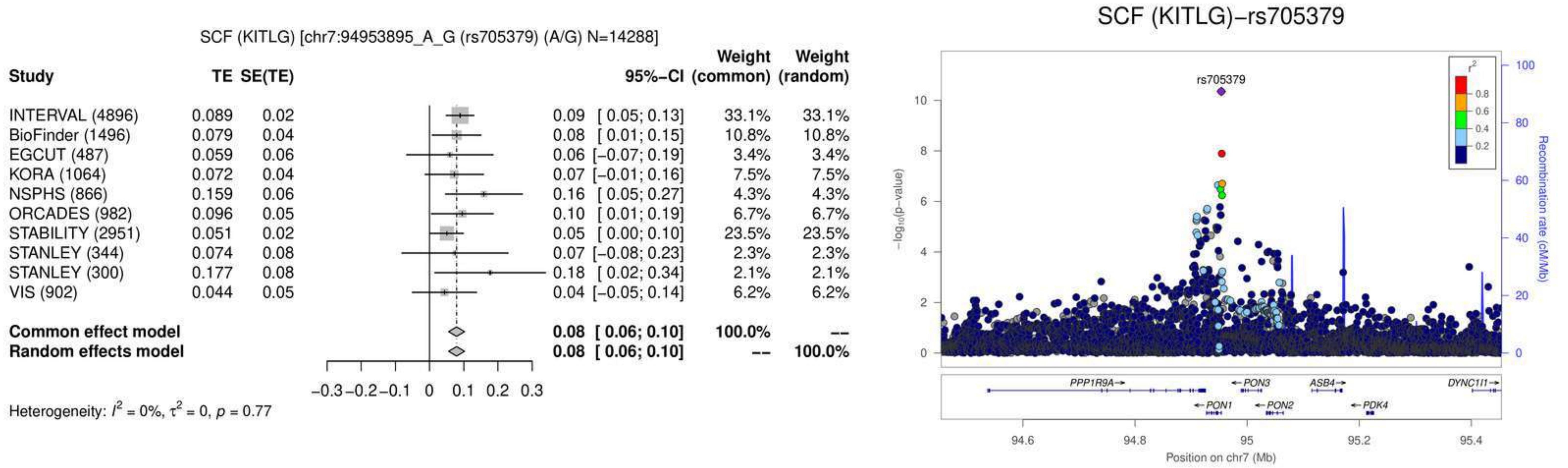

SCF (KITLG) [chr7:94953895_A_G (rs705379) (A/G) N=14288]
Study
TE
SE(TE)
95%−CI
Weight (common)
Weight (random)
INTERVAL (4896) 0.089 0.02 0.09 [0.05; 0.13] 33.1% 33.1%
BioFinder (1496) 0.079 0.04 0.08 [0.01; 0.15] 10.8% 10.8%
EGCUT (487) 0.059 0.06 0.06 [−0.07; 0.19] 3.4% 3.4%
KORA (1064) 0.072 0.04 0.07 [−0.01; 0.16] 7.5% 7.5%
NSPHS (866) 0.159 0.06 0.16 [0.05; 0.27] 4.3% 4.3%
ORCADES (982) 0.096 0.05 0.10 [0.01; 0.19] 6.7% 6.7%
STABILITY (2951) 0.051 0.02 0.05 [0.00; 0.10] 23.5% 23.5%
STANLEY (344) 0.074 0.08 0.07 [−0.08; 0.23] 2.3% 2.3%
STANLEY (300) 0.177 0.08 0.18 [0.02; 0.34] 2.1% 2.1%
VIS (902) 0.044 0.05 0.04 [−0.05; 0.14] 6.2% 6.2%
Common effect model 0.08 [0.06; 0.10] 100.0% --
Random effects model 0.08 [0.06; 0.10] -- 100.0%
−0.3 −0.2 −0.1 0 0.1 0.2 0.3
Heterogeneity: $I^2 = 0\%$, $\tau^2 = 0$, $p = 0.77$
SCF (KITLG)−rs705379
rs705379
r^2
0.8
0.6
0.4
0.2
$-\log_{10}(p\text{-value})$
Recombination rate (cM/Mb)
PPP1R9A
PON3
ASB4
DYNC1I1
PON1
PON2
PDK4
94.6
94.8
95
95.2
95.4
Position on chr7 (Mb)

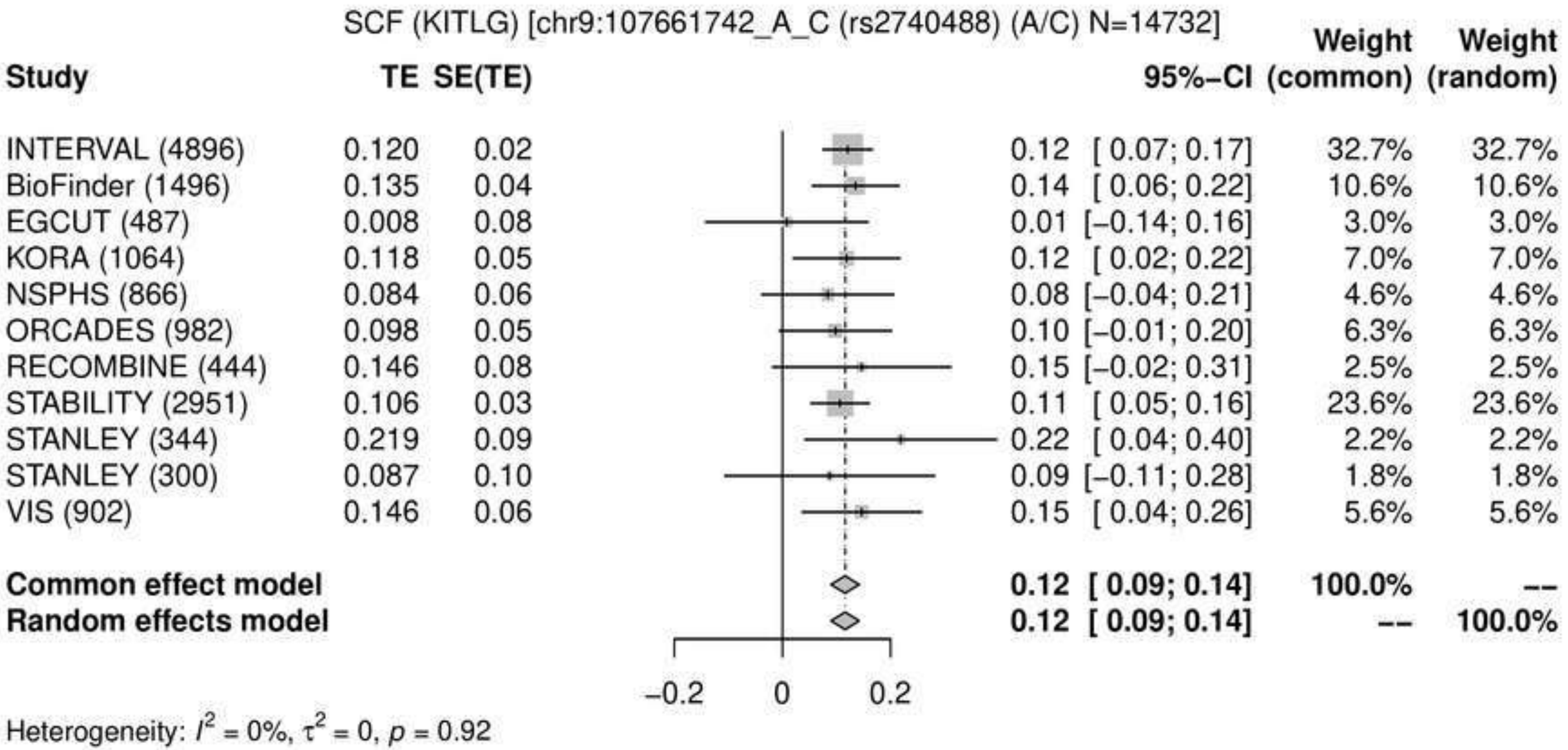
SCF (KITLG) [chr9:107661742_A_C (rs2740488) (A/C) N=14732]
Study TE SE(TE) 95%−CI Weight (common) Weight (random)
INTERVAL (4896) 0.120 0.02 0.12 [0.07; 0.17] 32.7% 32.7%
BioFinder (1496) 0.135 0.04 0.14 [0.06; 0.22] 10.6% 10.6%
EGCUT (487) 0.008 0.08 0.01 [−0.14; 0.16] 3.0% 3.0%
KORA (1064) 0.118 0.05 0.12 [0.02; 0.22] 7.0% 7.0%
NSPHS (866) 0.084 0.06 0.08 [−0.04; 0.21] 4.6% 4.6%
ORCADES (982) 0.098 0.05 0.10 [−0.01; 0.20] 6.3% 6.3%
RECOMBINE (444) 0.146 0.08 0.15 [−0.02; 0.31] 2.5% 2.5%
STABILITY (2951) 0.106 0.03 0.11 [0.05; 0.16] 23.6% 23.6%
STANLEY (344) 0.219 0.09 0.22 [0.04; 0.40] 2.2% 2.2%
STANLEY (300) 0.087 0.10 0.09 [−0.11; 0.28] 1.8% 1.8%
VIS (902) 0.146 0.06 0.15 [0.04; 0.26] 5.6% 5.6%
Common effect model 0.12 [0.09; 0.14] 100.0% --
Random effects model 0.12 [0.09; 0.14] -- 100.0%
−0.2 0 0.2
Heterogeneity: I^2 = 0%, τ^2 = 0, p = 0.92

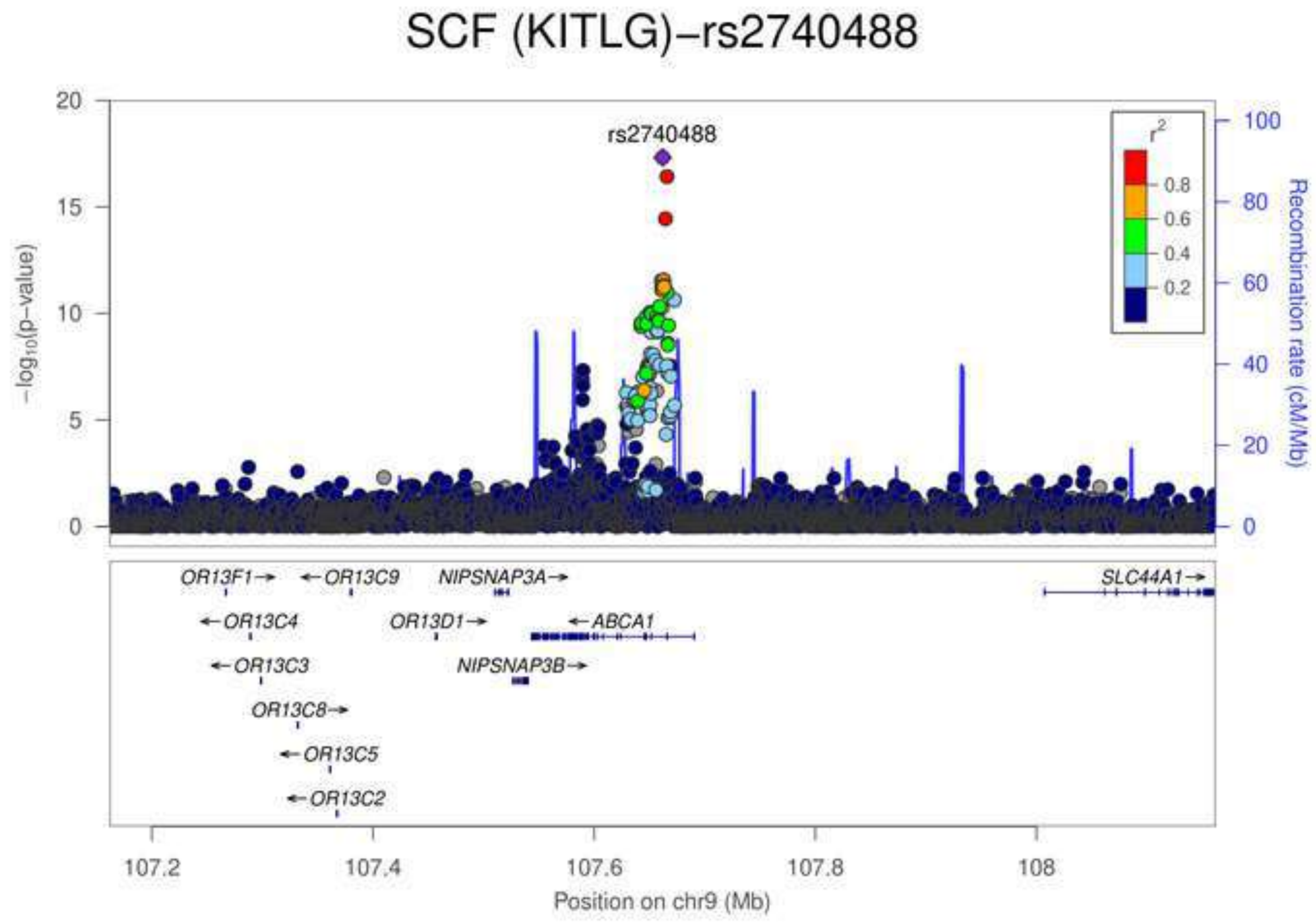
SCF (KITLG)−rs2740488
rs2740488
$-\log_{10}$(p-value)
Recombination rate (cM/Mb)
r^2
0.8
0.6
0.4
0.2
OR13F1→
←OR13C9
NIPSNAP3A→
SLC44A1→
←OR13C4
OR13D1→
←ABCA1
←OR13C3
NIPSNAP3B→
OR13C8→
←OR13C5
←OR13C2
107.2 107.4 107.6 107.8 108
Position on chr9 (Mb)

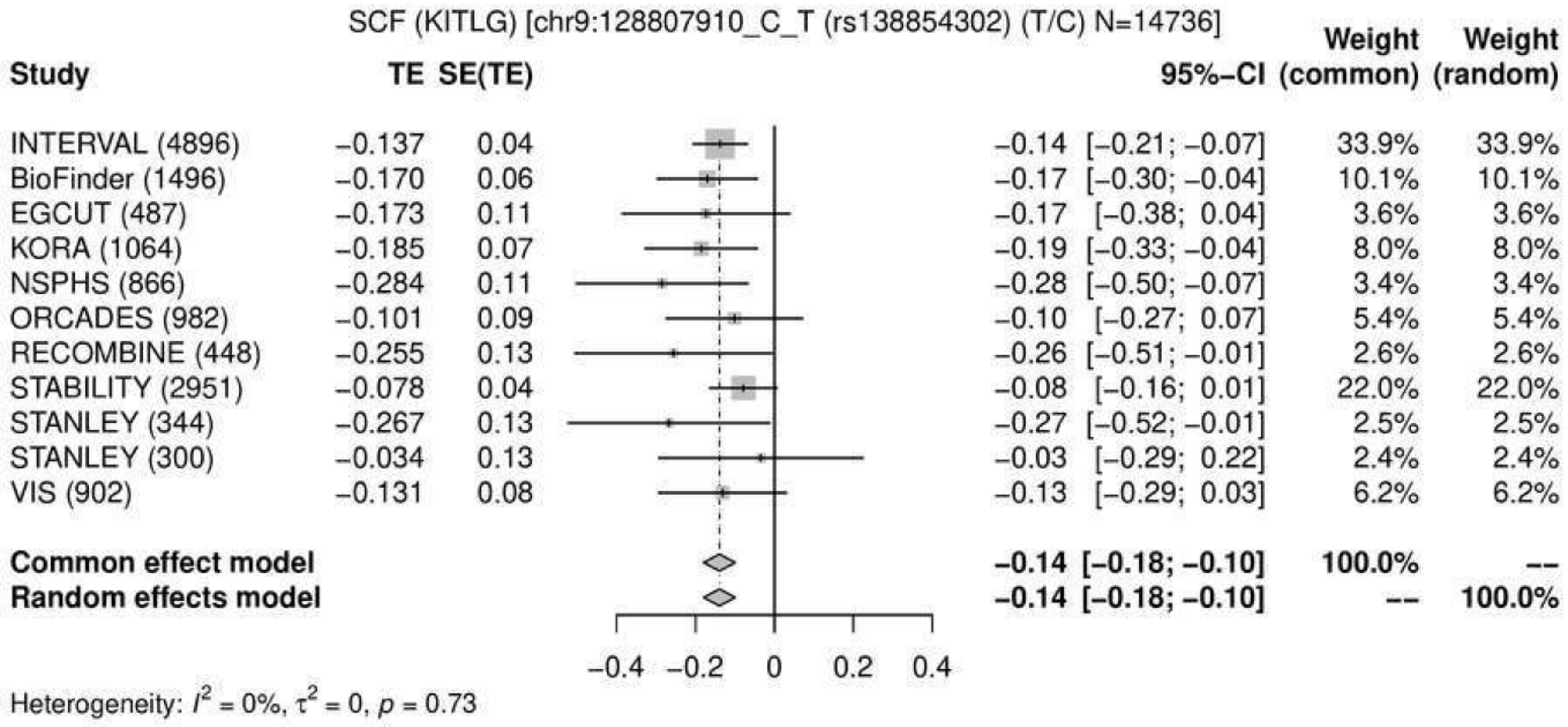

SCF (KITLG) [chr9:128807910_C_T (rs138854302) (T/C) N=14736]
Study
TE SE(TE)
95%–CI
Weight (common)
Weight (random)
INTERVAL (4896) −0.137 0.04 −0.14 [−0.21; −0.07] 33.9% 33.9%
BioFinder (1496) −0.170 0.06 −0.17 [−0.30; −0.04] 10.1% 10.1%
EGCUT (487) −0.173 0.11 −0.17 [−0.38; 0.04] 3.6% 3.6%
KORA (1064) −0.185 0.07 −0.19 [−0.33; −0.04] 8.0% 8.0%
NSPHS (866) −0.284 0.11 −0.28 [−0.50; −0.07] 3.4% 3.4%
ORCADES (982) −0.101 0.09 −0.10 [−0.27; 0.07] 5.4% 5.4%
RECOMBINE (448) −0.255 0.13 −0.26 [−0.51; −0.01] 2.6% 2.6%
STABILITY (2951) −0.078 0.04 −0.08 [−0.16; 0.01] 22.0% 22.0%
STANLEY (344) −0.267 0.13 −0.27 [−0.52; −0.01] 2.5% 2.5%
STANLEY (300) −0.034 0.13 −0.03 [−0.29; 0.22] 2.4% 2.4%
VIS (902) −0.131 0.08 −0.13 [−0.29; 0.03] 6.2% 6.2%
Common effect model −0.14 [−0.18; −0.10] 100.0% --
Random effects model −0.14 [−0.18; −0.10] -- 100.0%
−0.4 −0.2 0 0.2 0.4
Heterogeneity: $I^2 = 0\%$, $\tau^2 = 0$, $p = 0.73$

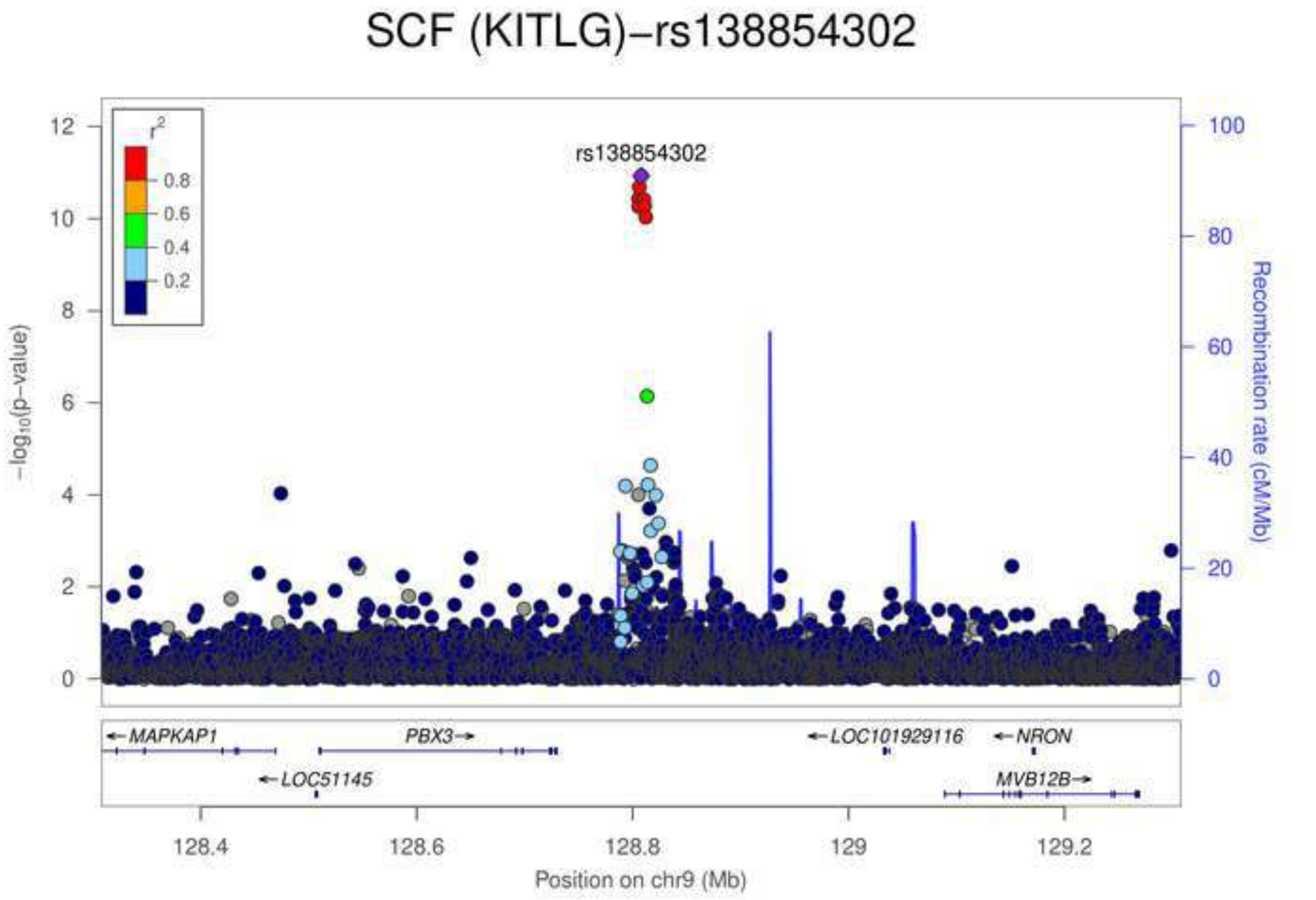

SCF (KITLG)−rs138854302
rs138854302
r^2
0.8
0.6
0.4
0.2
12
10
8
6
4
2
0
$-\log_{10}$(p−value)
100
80
60
40
20
0
Recombination rate (cM/Mb)
MAPKAP1
PBX3
LOC101929116
NRON
LOC51145
MVB12B
128.4
128.6
128.8
129
129.2
Position on chr9 (Mb)

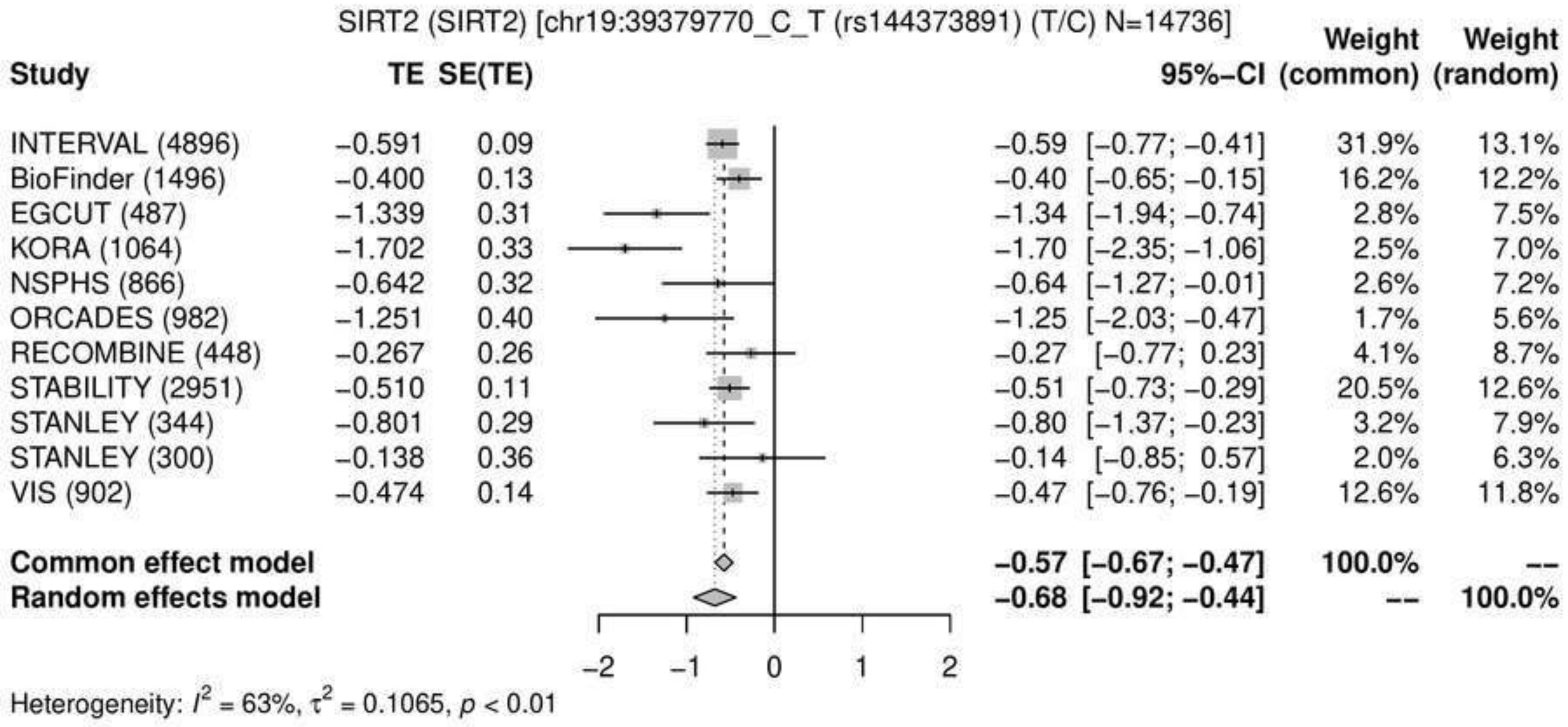
SIRT2 (SIRT2) [chr19:39379770_C_T (rs144373891) (T/C) N=14736]
Study
TE SE(TE)
95%-CI
Weight (common)
Weight (random)
INTERVAL (4896) −0.591 0.09 −0.59 [−0.77; −0.41] 31.9% 13.1%
BioFinder (1496) −0.400 0.13 −0.40 [−0.65; −0.15] 16.2% 12.2%
EGCUT (487) −1.339 0.31 −1.34 [−1.94; −0.74] 2.8% 7.5%
KORA (1064) −1.702 0.33 −1.70 [−2.35; −1.06] 2.5% 7.0%
NSPHS (866) −0.642 0.32 −0.64 [−1.27; −0.01] 2.6% 7.2%
ORCADES (982) −1.251 0.40 −1.25 [−2.03; −0.47] 1.7% 5.6%
RECOMBINE (448) −0.267 0.26 −0.27 [−0.77; 0.23] 4.1% 8.7%
STABILITY (2951) −0.510 0.11 −0.51 [−0.73; −0.29] 20.5% 12.6%
STANLEY (344) −0.801 0.29 −0.80 [−1.37; −0.23] 3.2% 7.9%
STANLEY (300) −0.138 0.36 −0.14 [−0.85; 0.57] 2.0% 6.3%
VIS (902) −0.474 0.14 −0.47 [−0.76; −0.19] 12.6% 11.8%
Common effect model −0.57 [−0.67; −0.47] 100.0% --
Random effects model −0.68 [−0.92; −0.44] -- 100.0%
−2 −1 0 1 2
Heterogeneity: I^2 = 63%, τ^2 = 0.1065, p < 0.01
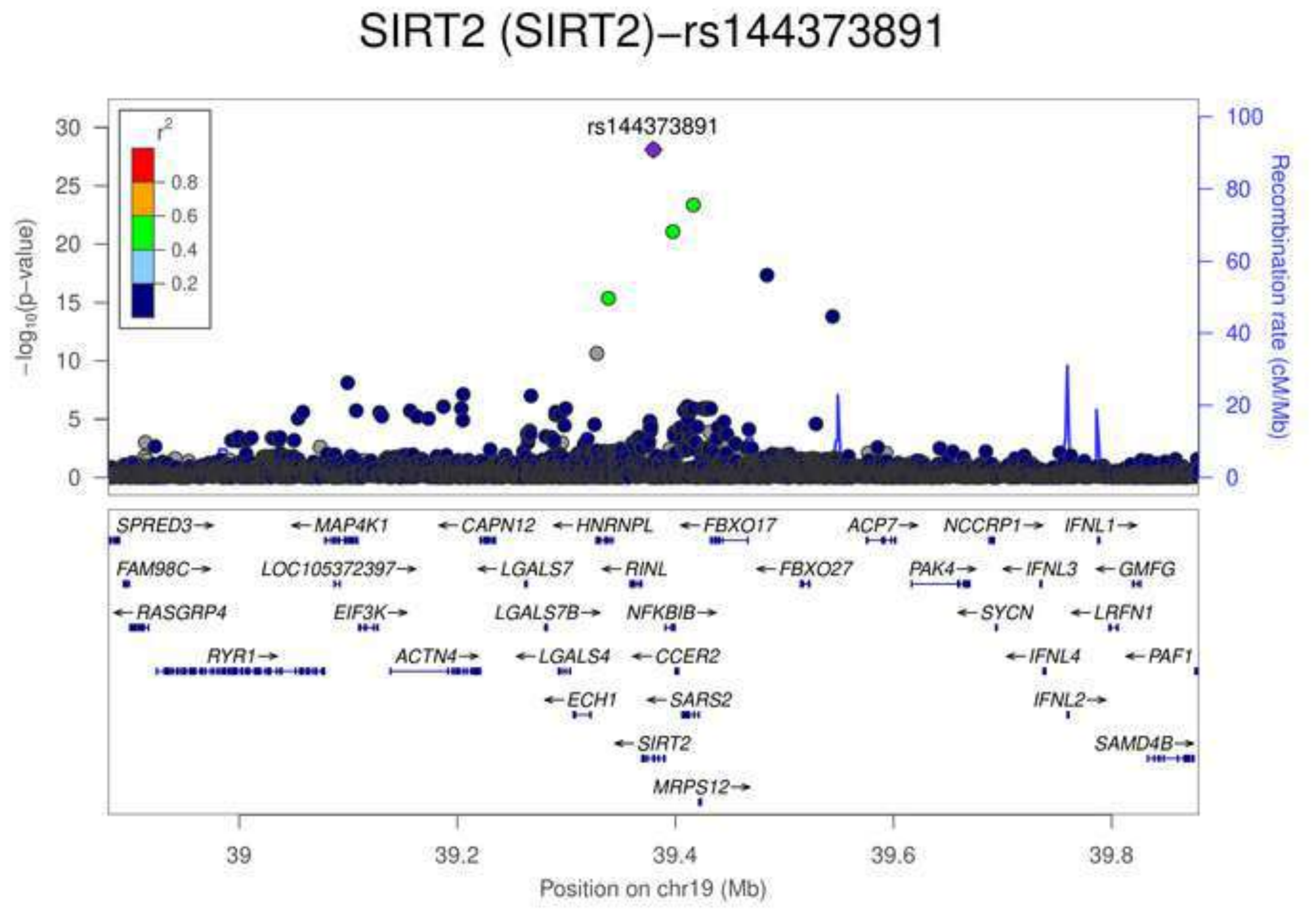
SIRT2 (SIRT2)−rs144373891
rs144373891
r^2
0.8
0.6
0.4
0.2
$-\log_{10}$(p-value)
Recombination rate (cM/Mb)
SPRED3 MAP4K1 CAPN12 HNRNPL FBXO17 ACP7 NCCRP1 IFNL1
FAM98C LOC105372397 LGALS7 RINL FBXO27 PAK4 IFNL3 GMFG
RASGRP4 EIF3K LGALS7B NFKBIB SYCN LRFN1
RYR1 ACTN4 LGALS4 CCER2 IFNL4 PAF1
ECH1 SARS2 IFNL2
SIRT2 SAMD4B
MRPS12
39 39.2 39.4 39.6 39.8
Position on chr19 (Mb)

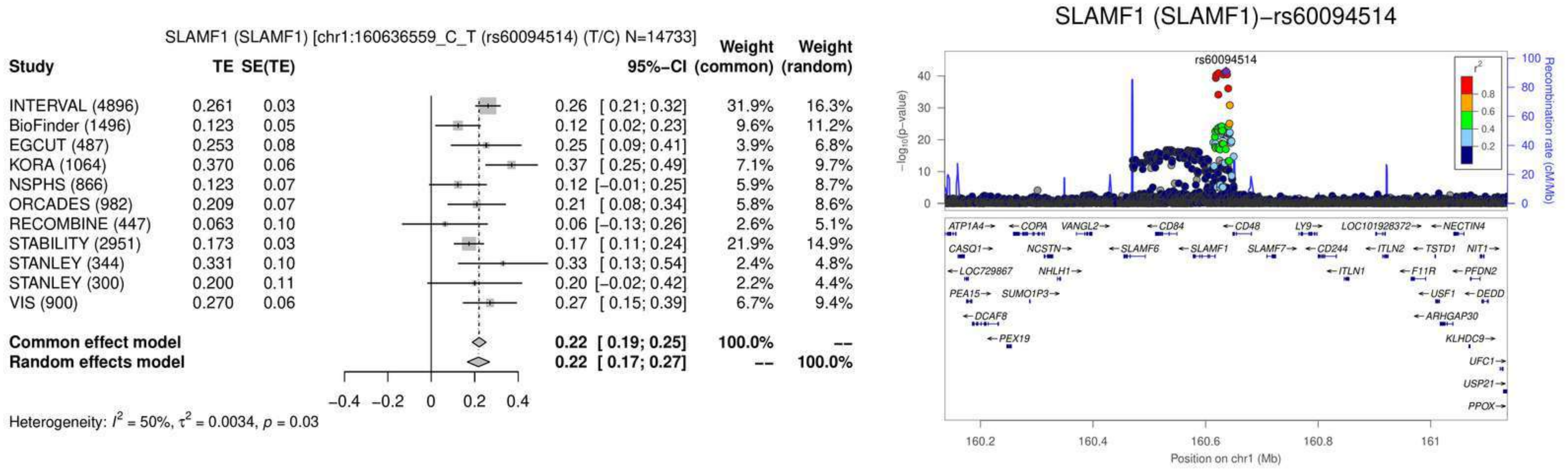

SLAMF1 (SLAMF1) [chr1:160636559_C_T (rs60094514) (T/C) N=14733]
Study TE SE(TE) 95%-CI Weight (common) Weight (random)
INTERVAL (4896) 0.261 0.03 0.26 [0.21; 0.32] 31.9% 16.3%
BioFinder (1496) 0.123 0.05 0.12 [0.02; 0.23] 9.6% 11.2%
EGCUT (487) 0.253 0.08 0.25 [0.09; 0.41] 3.9% 6.8%
KORA (1064) 0.370 0.06 0.37 [0.25; 0.49] 7.1% 9.7%
NSPHS (866) 0.123 0.07 0.12 [−0.01; 0.25] 5.9% 8.7%
ORCADES (982) 0.209 0.07 0.21 [0.08; 0.34] 5.8% 8.6%
RECOMBINE (447) 0.063 0.10 0.06 [−0.13; 0.26] 2.6% 5.1%
STABILITY (2951) 0.173 0.03 0.17 [0.11; 0.24] 21.9% 14.9%
STANLEY (344) 0.331 0.10 0.33 [0.13; 0.54] 2.4% 4.8%
STANLEY (300) 0.200 0.11 0.20 [−0.02; 0.42] 2.2% 4.4%
VIS (900) 0.270 0.06 0.27 [0.15; 0.39] 6.7% 9.4%
Common effect model 0.22 [0.19; 0.25] 100.0% --
Random effects model 0.22 [0.17; 0.27] -- 100.0%
−0.4 −0.2 0 0.2 0.4
Heterogeneity: I^2 = 50%, τ^2 = 0.0034, p = 0.03
SLAMF1 (SLAMF1)−rs60094514
rs60094514
$-\log_{10}$(p−value)
Recombination rate (cM/Mb)
r^2
0.8
0.6
0.4
0.2
ATP1A4 COPA VANGL2 CD84 CD48 LY9 LOC101928372 NECTIN4
CASQ1 NCSTN SLAMF6 SLAMF1 SLAMF7 CD244 ITLN2 TSTD1 NIT1
LOC729867 NHLH1 ITLN1 F11R PFDN2
PEA15 SUMO1P3 USF1 DEDD
DCAF8 ARHGAP30
PEX19 KLHDC9
UFC1
USP21
PPOX
160.2 160.4 160.6 160.8 161
Position on chr1 (Mb)

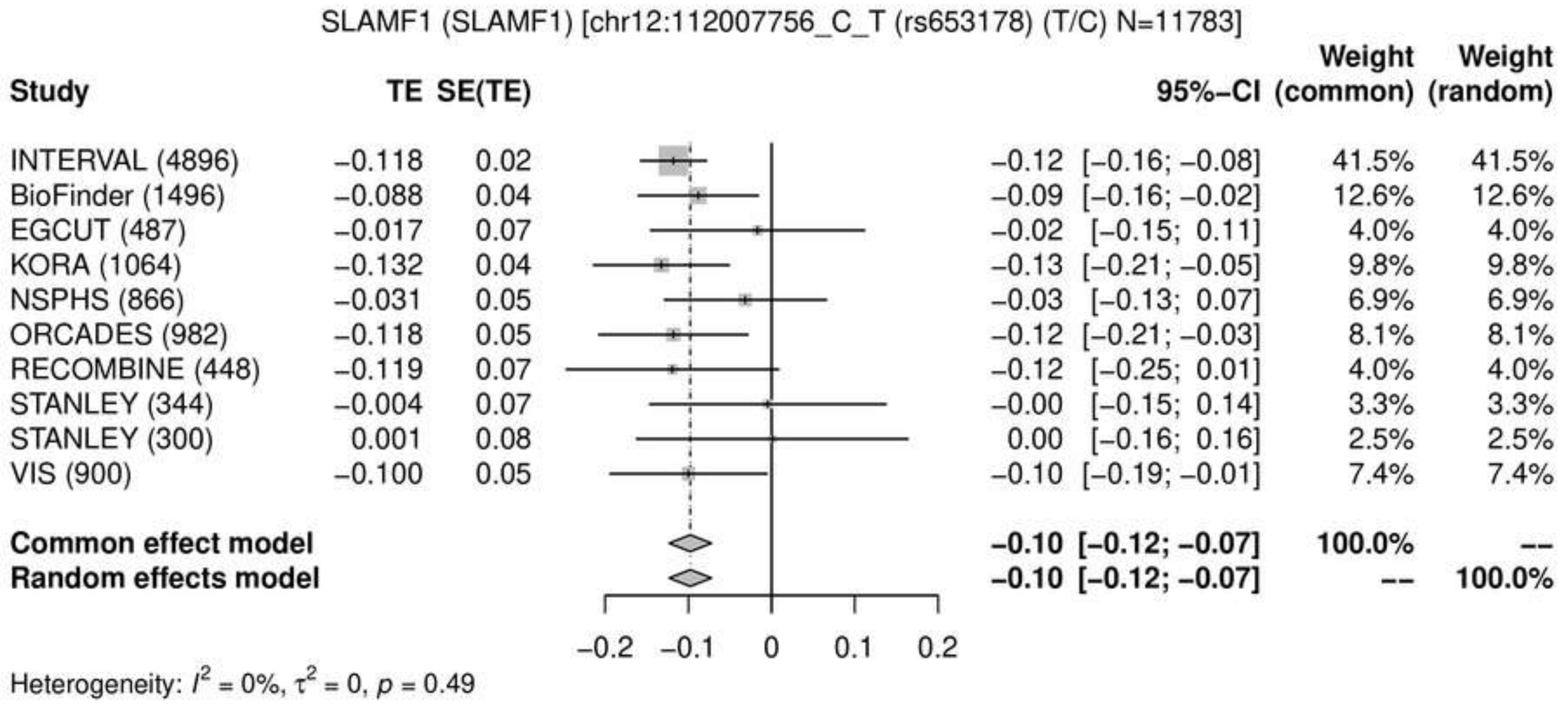
SLAMF1 (SLAMF1) [chr12:112007756_C_T (rs653178) (T/C) N=11783]
Study
TE
SE(TE)
95%-CI
Weight (common)
Weight (random)
INTERVAL (4896) −0.118 0.02 −0.12 [−0.16; −0.08] 41.5% 41.5%
BioFinder (1496) −0.088 0.04 −0.09 [−0.16; −0.02] 12.6% 12.6%
EGCUT (487) −0.017 0.07 −0.02 [−0.15; 0.11] 4.0% 4.0%
KORA (1064) −0.132 0.04 −0.13 [−0.21; −0.05] 9.8% 9.8%
NSPHS (866) −0.031 0.05 −0.03 [−0.13; 0.07] 6.9% 6.9%
ORCADES (982) −0.118 0.05 −0.12 [−0.21; −0.03] 8.1% 8.1%
RECOMBINE (448) −0.119 0.07 −0.12 [−0.25; 0.01] 4.0% 4.0%
STANLEY (344) −0.004 0.07 −0.00 [−0.15; 0.14] 3.3% 3.3%
STANLEY (300) 0.001 0.08 0.00 [−0.16; 0.16] 2.5% 2.5%
VIS (900) −0.100 0.05 −0.10 [−0.19; −0.01] 7.4% 7.4%
Common effect model −0.10 [−0.12; −0.07] 100.0% --
Random effects model −0.10 [−0.12; −0.07] -- 100.0%
−0.2 −0.1 0 0.1 0.2
Heterogeneity: I^2 = 0%, τ^2 = 0, p = 0.49

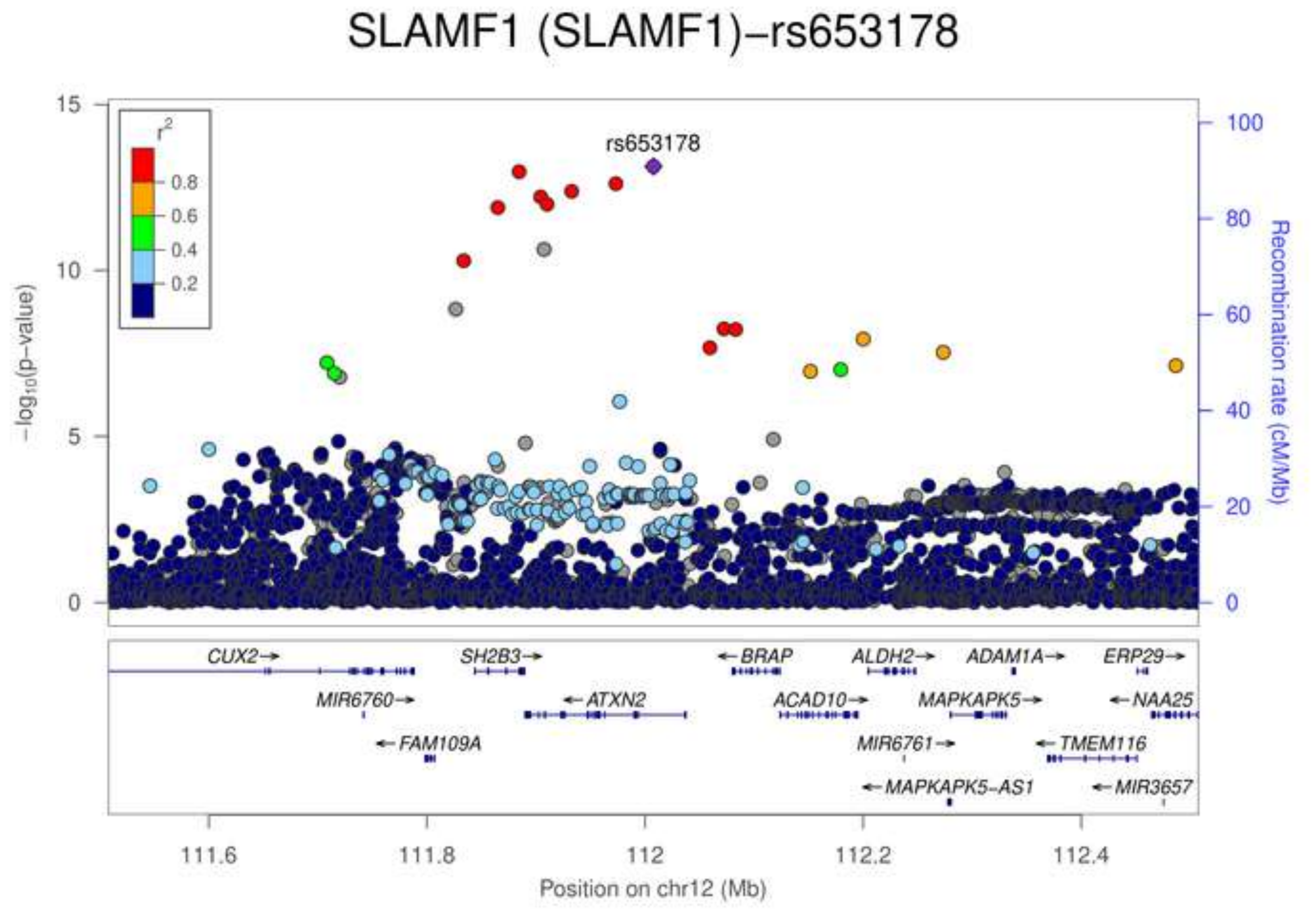
SLAMF1 (SLAMF1)−rs653178
rs653178
r^2
0.8
0.6
0.4
0.2
$-\log_{10}$(p−value)
Recombination rate (cM/Mb)
CUX2→
SH2B3→
←BRAP
ALDH2→
ADAM1A→
ERP29→
MIR6760→
←ATXN2
ACAD10→
MAPKAPK5→
←NAA25
←FAM109A
MIR6761→
←TMEM116
←MAPKAPK5-AS1
←MIR3657
111.6
111.8
112
112.2
112.4
Position on chr12 (Mb)

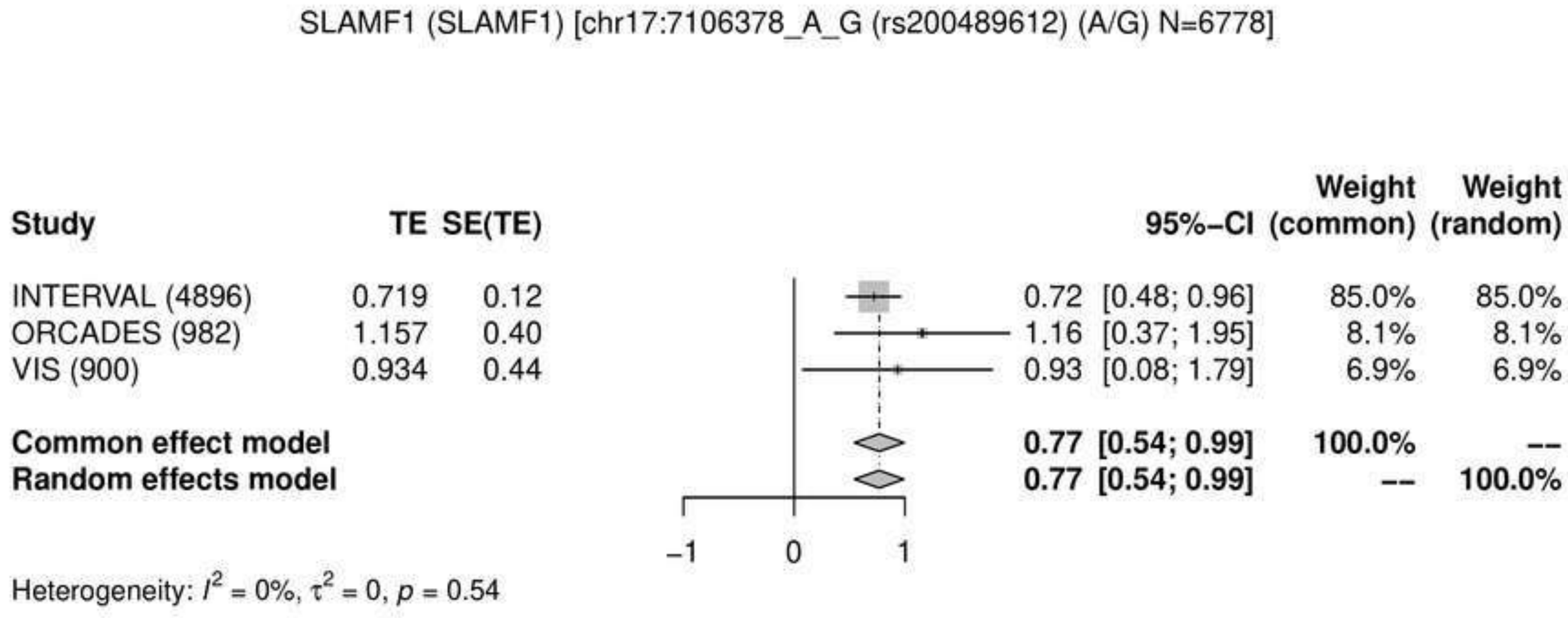
SLAMF1 (SLAMF1) [chr17:7106378_A_G (rs200489612) (A/G) N=6778]
Study
TE
SE(TE)
95%-CI
Weight (common)
Weight (random)
INTERVAL (4896) 0.719 0.12 0.72 [0.48; 0.96] 85.0% 85.0%
ORCADES (982) 1.157 0.40 1.16 [0.37; 1.95] 8.1% 8.1%
VIS (900) 0.934 0.44 0.93 [0.08; 1.79] 6.9% 6.9%
Common effect model 0.77 [0.54; 0.99] 100.0% --
Random effects model 0.77 [0.54; 0.99] -- 100.0%
−1
0
1
Heterogeneity: I^2 = 0%, τ^2 = 0, p = 0.54

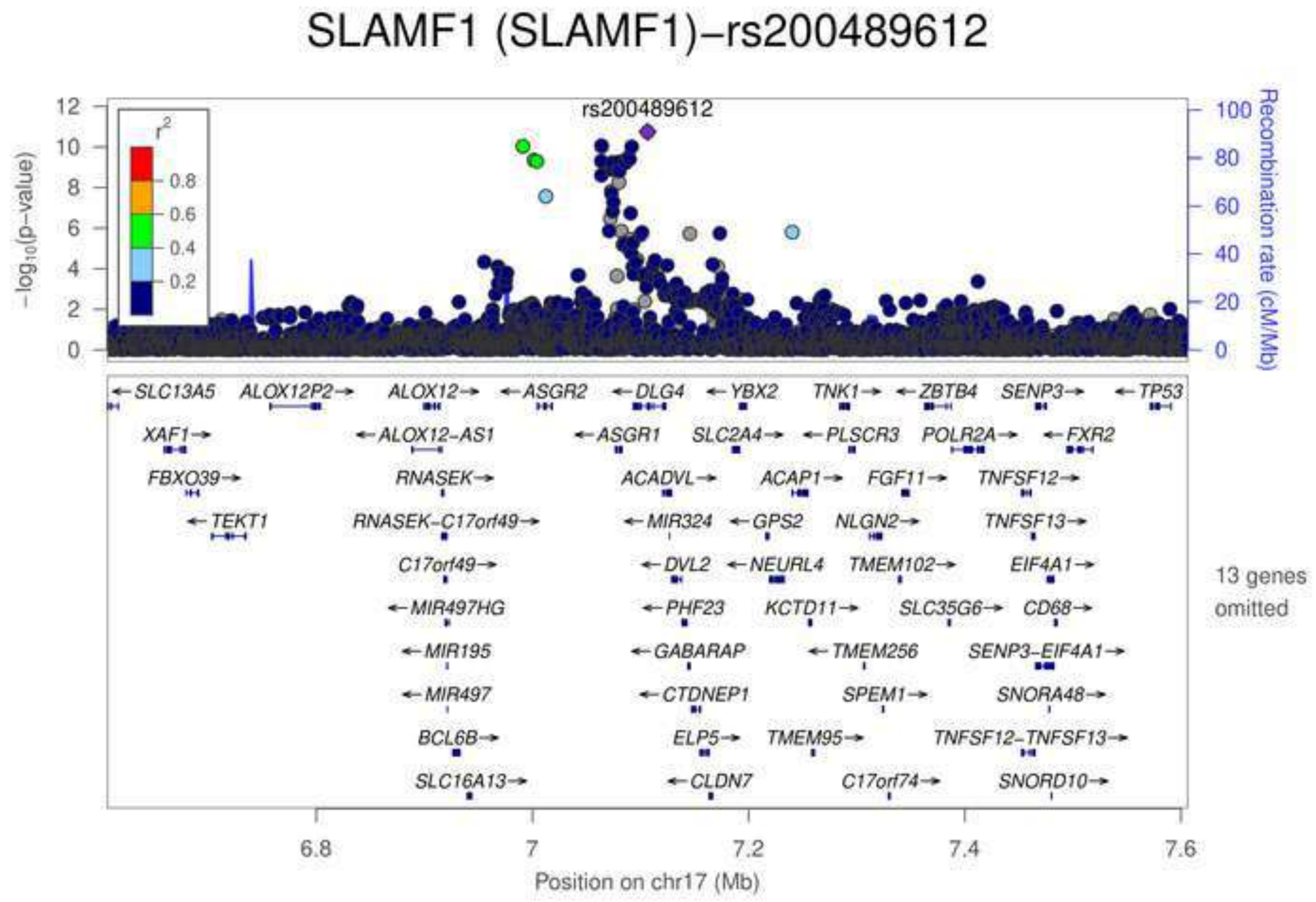
SLAMF1 (SLAMF1)−rs200489612
rs200489612
$-\log_{10}$(p−value)
Recombination rate (cM/Mb)
r^2
0.8
0.6
0.4
0.2
13 genes omitted
Position on chr17 (Mb)
6.8
7
7.2
7.4
7.6

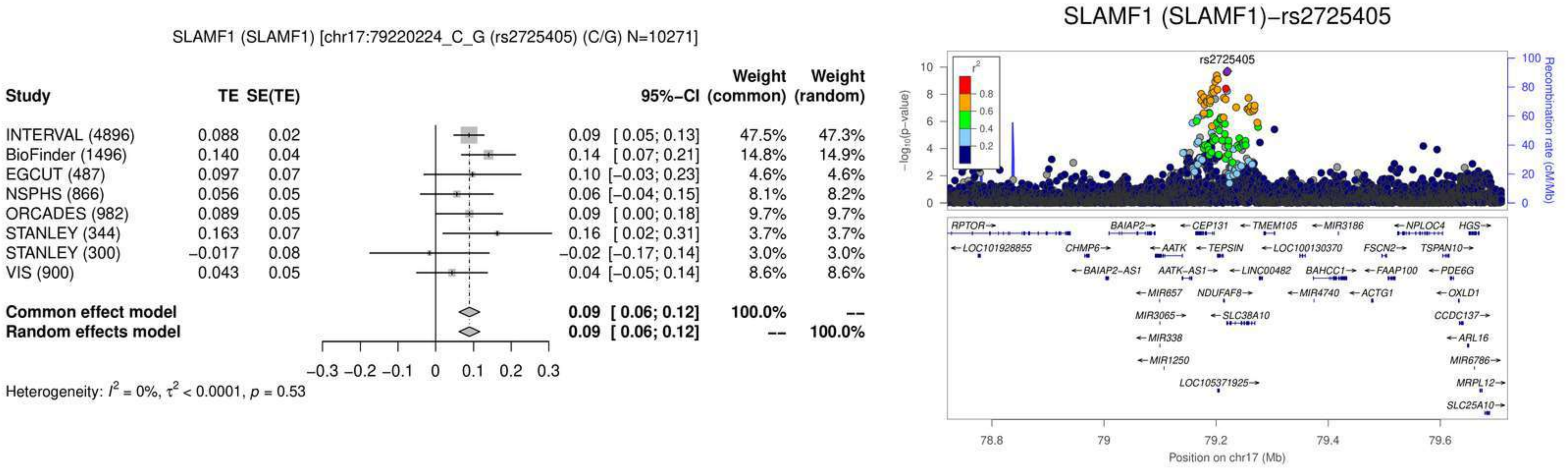
SLAMF1 (SLAMF1) [chr17:79220224_C_G (rs2725405) (C/G) N=10271]
Study | TE | SE(TE) | 95%-CI | Weight (common) | Weight (random)
INTERVAL (4896) | 0.088 | 0.02 | 0.09 [0.05; 0.13] | 47.5% | 47.3%
BioFinder (1496) | 0.140 | 0.04 | 0.14 [0.07; 0.21] | 14.8% | 14.9%
EGCUT (487) | 0.097 | 0.07 | 0.10 [−0.03; 0.23] | 4.6% | 4.6%
NSPHS (866) | 0.056 | 0.05 | 0.06 [−0.04; 0.15] | 8.1% | 8.2%
ORCADES (982) | 0.089 | 0.05 | 0.09 [0.00; 0.18] | 9.7% | 9.7%
STANLEY (344) | 0.163 | 0.07 | 0.16 [0.02; 0.31] | 3.7% | 3.7%
STANLEY (300) | −0.017 | 0.08 | −0.02 [−0.17; 0.14] | 3.0% | 3.0%
VIS (900) | 0.043 | 0.05 | 0.04 [−0.05; 0.14] | 8.6% | 8.6%
Common effect model | 0.09 [0.06; 0.12] | 100.0% | --
Random effects model | 0.09 [0.06; 0.12] | -- | 100.0%
−0.3 −0.2 −0.1 0 0.1 0.2 0.3
Heterogeneity: I^2 = 0%, τ^2 < 0.0001, p = 0.53
SLAMF1 (SLAMF1)−rs2725405
rs2725405
$-\log_{10}$(p-value)
Recombination rate (cM/Mb)
r^2
0.8
0.6
0.4
0.2
RPTOR→
←LOC101928855
BAIAP2→
CHMP6→
←BAIAP2-AS1
←CEP131
←AATK
←TEPSIN
AATK-AS1→
←LINC00482
←MIR657
NDUFAF8→
MIR3065→
←SLC38A10
←MIR338
←MIR1250
LOC105371925→
←TMEM105
←LOC100130370
←MIR3186
BAHCC1→
←MIR4740
←ACTG1
FSCN2→
←FAAP100
←NPLOC4
TSPAN10→
←PDE6G
←OXLD1
CCDC137→
←ARL16
MIR6786→
MRPL12→
SLC25A10→
HGS→
78.8
79
79.2
79.4
79.6
Position on chr17 (Mb)

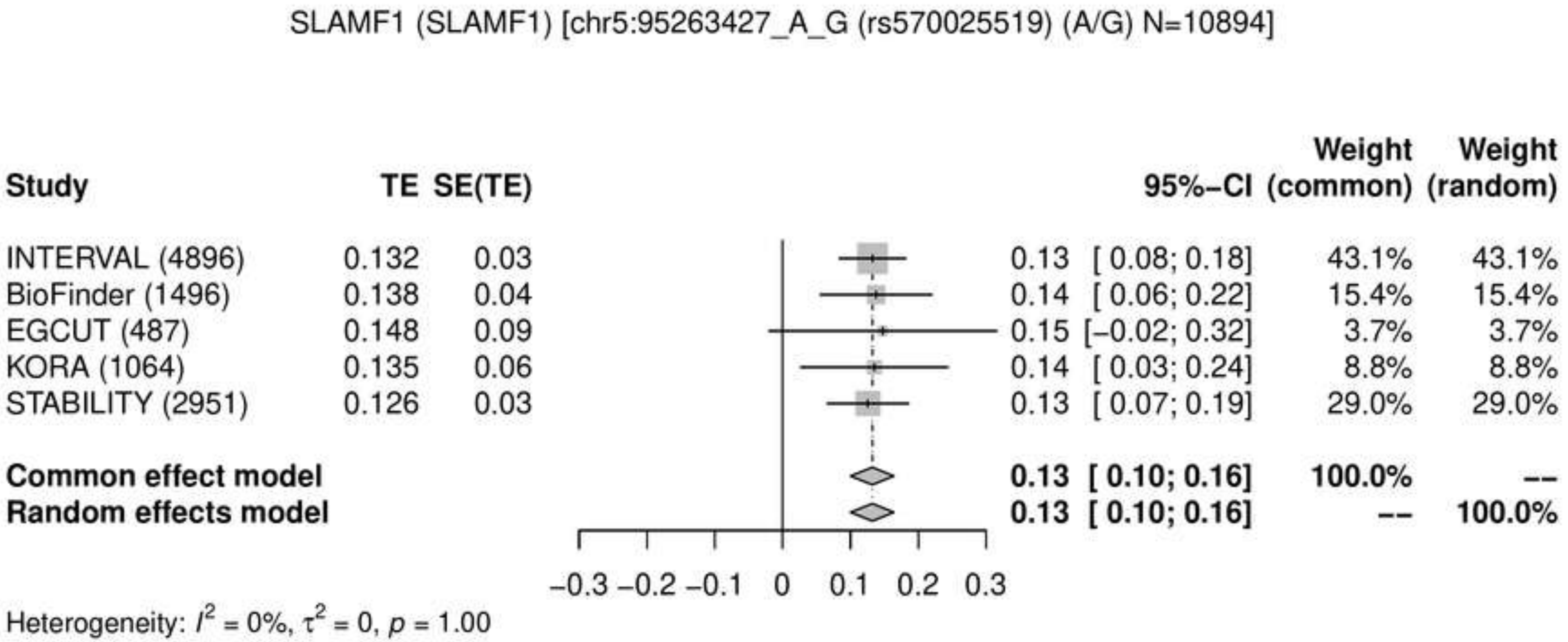
SLAMF1 (SLAMF1) [chr5:95263427_A_G (rs570025519) (A/G) N=10894]
Study
TE
SE(TE)
95%-CI
Weight (common)
Weight (random)
INTERVAL (4896) 0.132 0.03 0.13 [0.08; 0.18] 43.1% 43.1%
BioFinder (1496) 0.138 0.04 0.14 [0.06; 0.22] 15.4% 15.4%
EGCUT (487) 0.148 0.09 0.15 [−0.02; 0.32] 3.7% 3.7%
KORA (1064) 0.135 0.06 0.14 [0.03; 0.24] 8.8% 8.8%
STABILITY (2951) 0.126 0.03 0.13 [0.07; 0.19] 29.0% 29.0%
Common effect model 0.13 [0.10; 0.16] 100.0% --
Random effects model 0.13 [0.10; 0.16] -- 100.0%
−0.3 −0.2 −0.1 0 0.1 0.2 0.3
Heterogeneity: I^2 = 0%, τ^2 = 0, p = 1.00

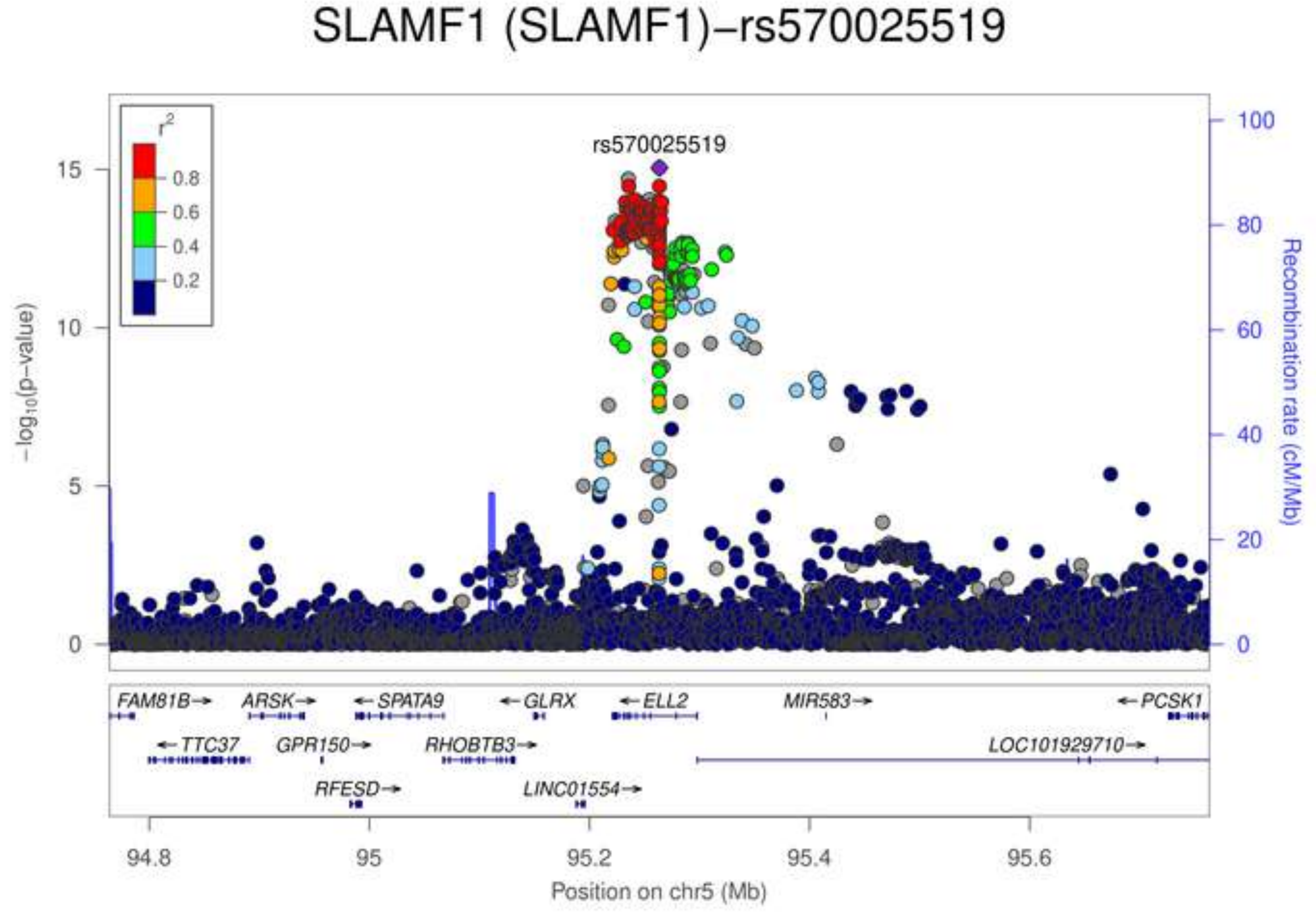
SLAMF1 (SLAMF1)−rs570025519
rs570025519
r^2
0.8
0.6
0.4
0.2
15
10
5
0
$-\log_{10}(p\text{-value})$
100
80
60
40
20
0
Recombination rate (cM/Mb)
FAM81B→
ARSK→
←SPATA9
←GLRX
←ELL2
MIR583→
←PCSK1
←TTC37
GPR150→
RHOBTB3→
LOC101929710→
RFESD→
LINC01554→
94.8
95
95.2
95.4
95.6
Position on chr5 (Mb)

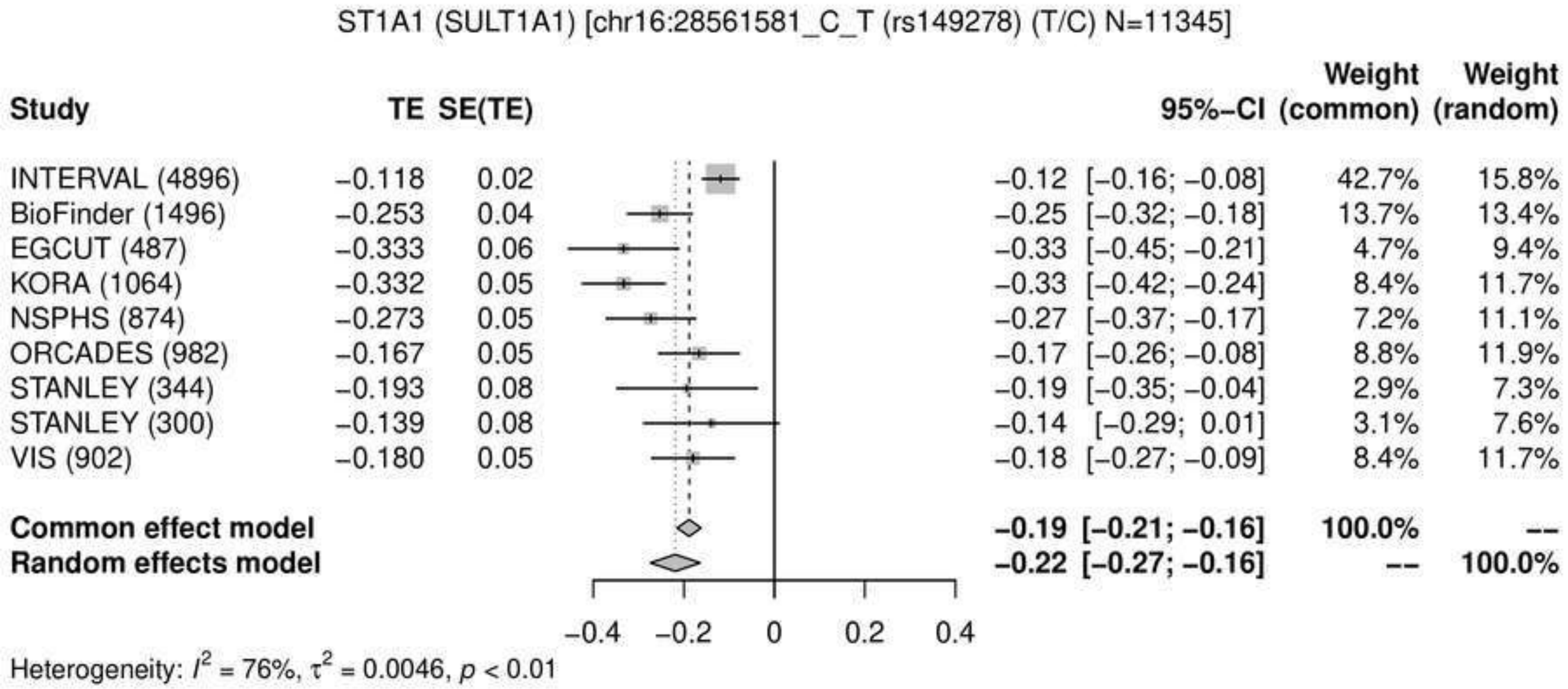
ST1A1 (SULT1A1) [chr16:28561581_C_T (rs149278) (T/C) N=11345]
Study
TE
SE(TE)
95%-CI
Weight (common)
Weight (random)
INTERVAL (4896) −0.118 0.02 −0.12 [−0.16; −0.08] 42.7% 15.8%
BioFinder (1496) −0.253 0.04 −0.25 [−0.32; −0.18] 13.7% 13.4%
EGCUT (487) −0.333 0.06 −0.33 [−0.45; −0.21] 4.7% 9.4%
KORA (1064) −0.332 0.05 −0.33 [−0.42; −0.24] 8.4% 11.7%
NSPHS (874) −0.273 0.05 −0.27 [−0.37; −0.17] 7.2% 11.1%
ORCADES (982) −0.167 0.05 −0.17 [−0.26; −0.08] 8.8% 11.9%
STANLEY (344) −0.193 0.08 −0.19 [−0.35; −0.04] 2.9% 7.3%
STANLEY (300) −0.139 0.08 −0.14 [−0.29; 0.01] 3.1% 7.6%
VIS (902) −0.180 0.05 −0.18 [−0.27; −0.09] 8.4% 11.7%
Common effect model −0.19 [−0.21; −0.16] 100.0% --
Random effects model −0.22 [−0.27; −0.16] -- 100.0%
−0.4 −0.2 0 0.2 0.4
Heterogeneity: I² = 76%, τ² = 0.0046, p < 0.01

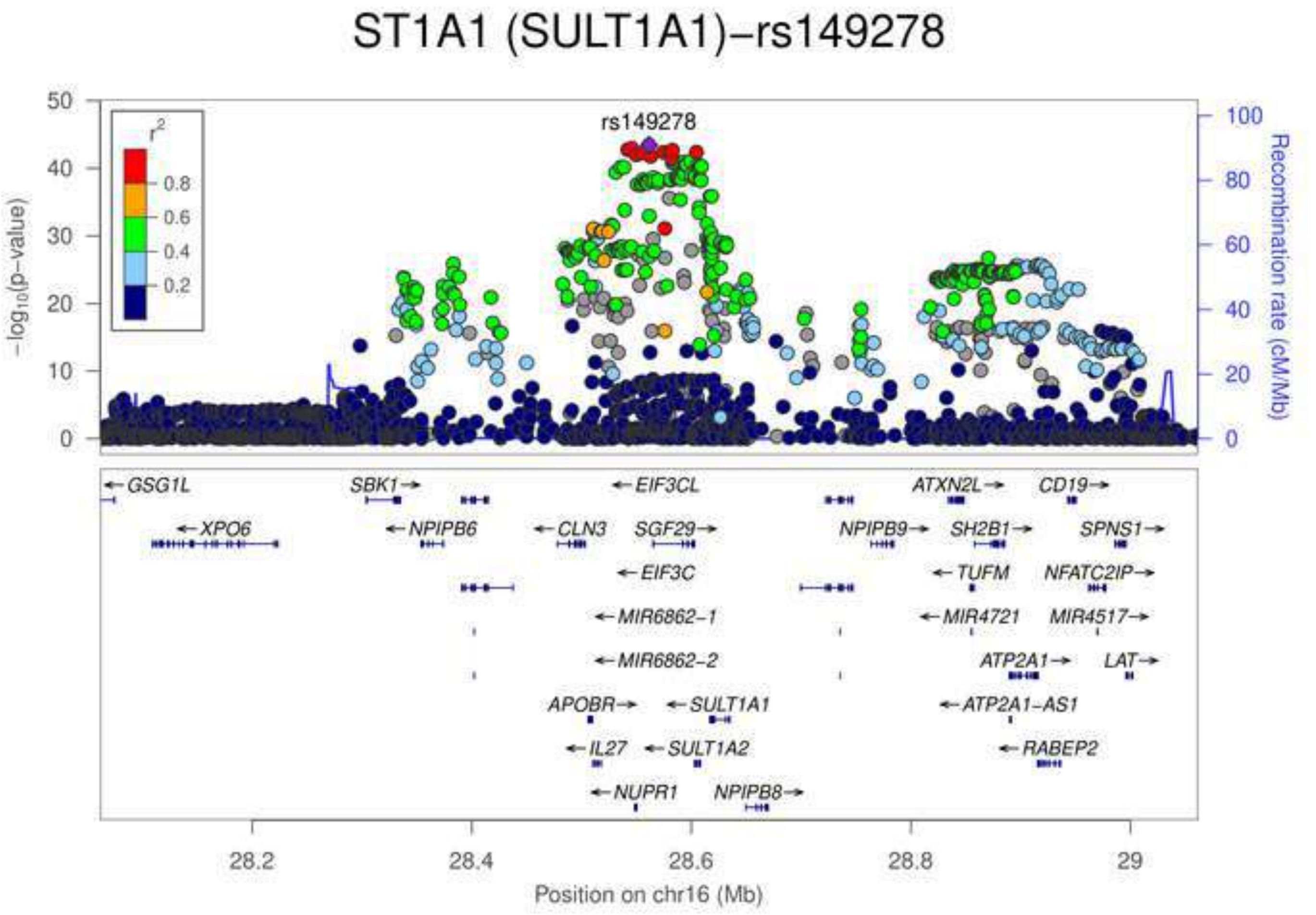
ST1A1 (SULT1A1)−rs149278
rs149278
r²
0.8
0.6
0.4
0.2
−log10(p−value)
Recombination rate (cM/Mb)
0 10 20 30 40 50
0 20 40 60 80 100
GSG1L
XPO6
SBK1
NPIPB6
EIF3CL
CLN3
SGF29
EIF3C
MIR6862−1
MIR6862−2
APOBR
SULT1A1
IL27
SULT1A2
NUPR1
NPIPB8
NPIPB9
ATXN2L
CD19
SH2B1
SPNS1
TUFM
NFATC2IP
MIR4721
MIR4517
ATP2A1
LAT
ATP2A1−AS1
RABEP2
28.2 28.4 28.6 28.8 29
Position on chr16 (Mb)

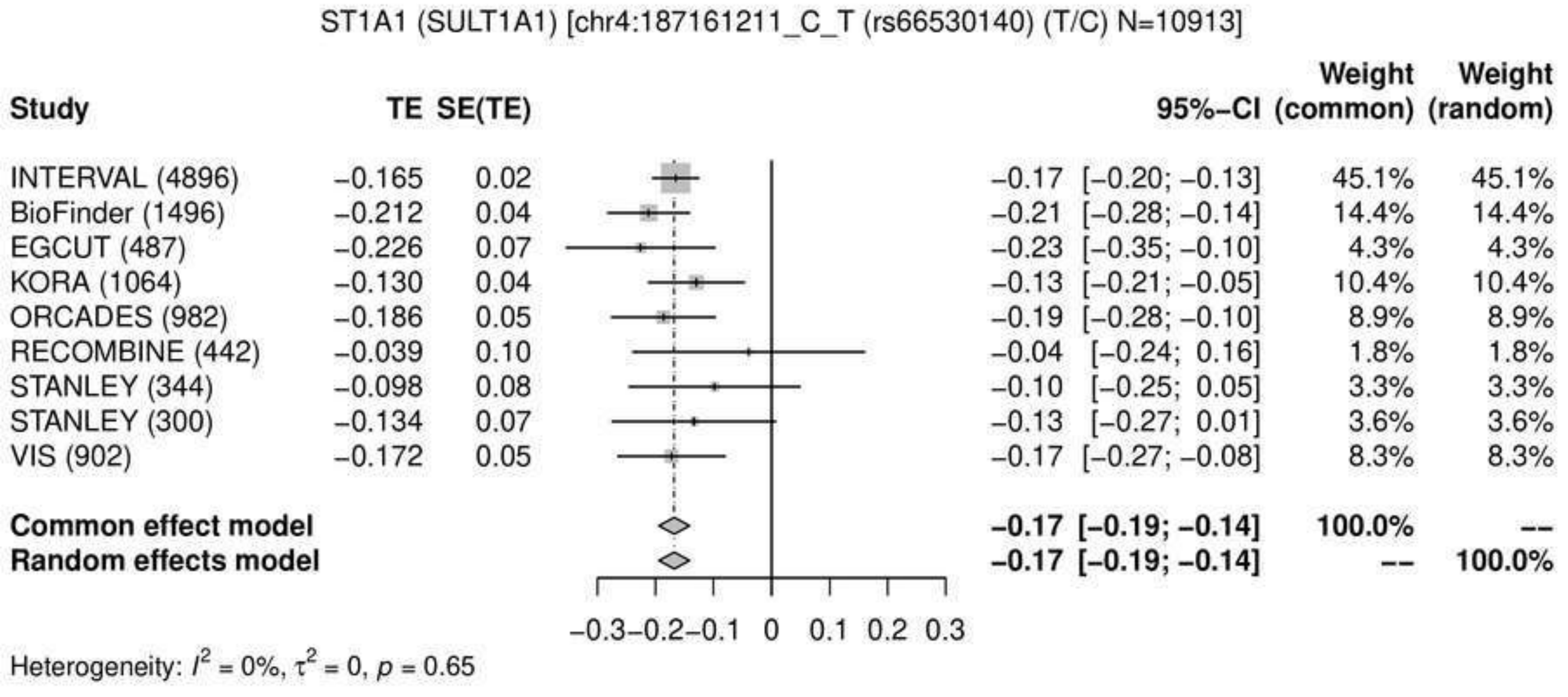
ST1A1 (SULT1A1) [chr4:187161211_C_T (rs66530140) (T/C) N=10913]
Study TE SE(TE) 95%−CI Weight (common) Weight (random)
INTERVAL (4896) −0.165 0.02 −0.17 [−0.20; −0.13] 45.1% 45.1%
BioFinder (1496) −0.212 0.04 −0.21 [−0.28; −0.14] 14.4% 14.4%
EGCUT (487) −0.226 0.07 −0.23 [−0.35; −0.10] 4.3% 4.3%
KORA (1064) −0.130 0.04 −0.13 [−0.21; −0.05] 10.4% 10.4%
ORCADES (982) −0.186 0.05 −0.19 [−0.28; −0.10] 8.9% 8.9%
RECOMBINE (442) −0.039 0.10 −0.04 [−0.24; 0.16] 1.8% 1.8%
STANLEY (344) −0.098 0.08 −0.10 [−0.25; 0.05] 3.3% 3.3%
STANLEY (300) −0.134 0.07 −0.13 [−0.27; 0.01] 3.6% 3.6%
VIS (902) −0.172 0.05 −0.17 [−0.27; −0.08] 8.3% 8.3%
Common effect model −0.17 [−0.19; −0.14] 100.0% --
Random effects model −0.17 [−0.19; −0.14] -- 100.0%
−0.3 −0.2 −0.1 0 0.1 0.2 0.3
Heterogeneity: I^2 = 0%, τ^2 = 0, p = 0.65

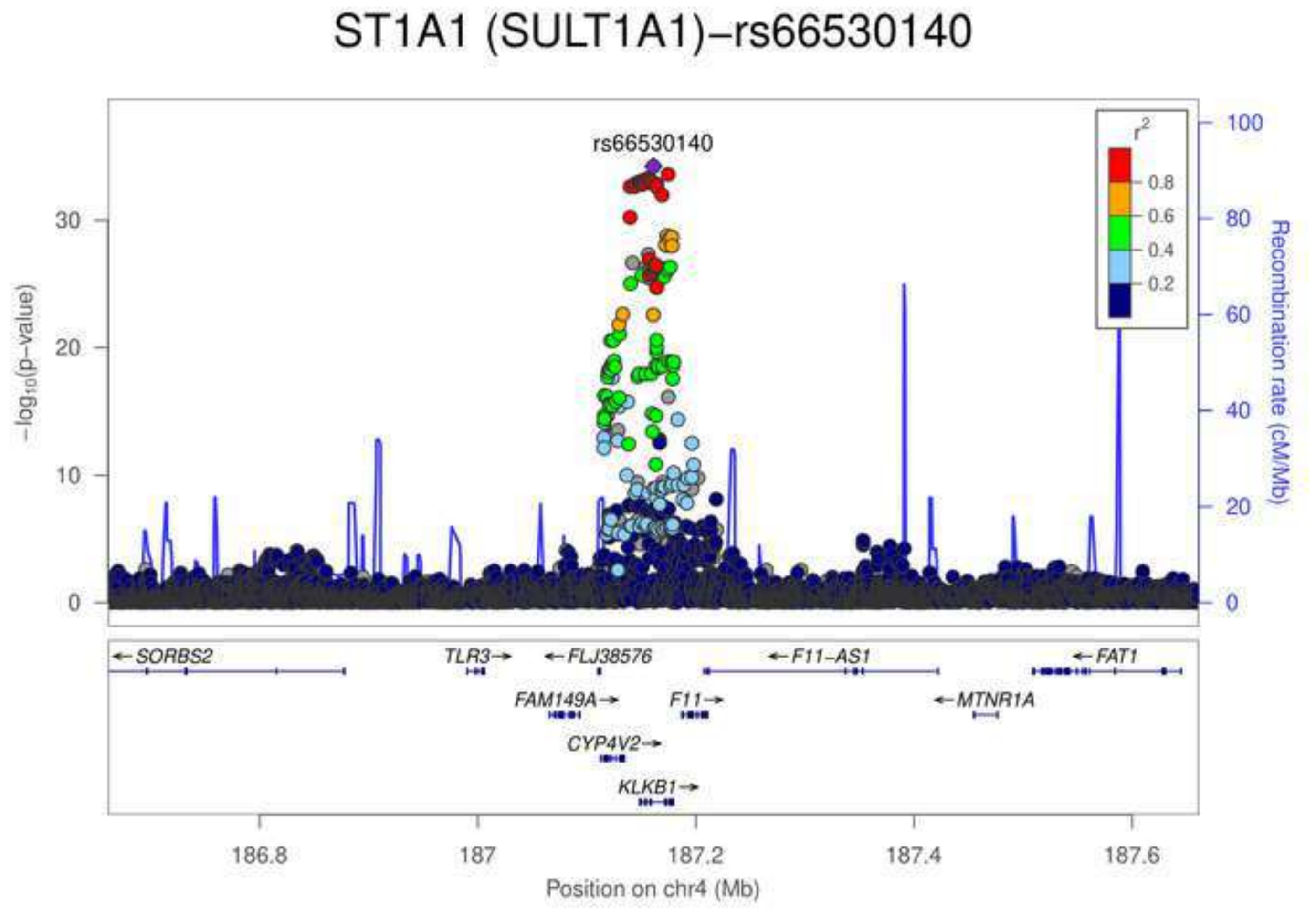
ST1A1 (SULT1A1)−rs66530140
rs66530140
$-\log_{10}$(p-value)
Recombination rate (cM/Mb)
r^2
0.8
0.6
0.4
0.2
SORBS2
TLR3
FLJ38576
F11-AS1
FAT1
FAM149A
F11
MTNR1A
CYP4V2
KLKB1
186.8
187
187.2
187.4
187.6
Position on chr4 (Mb)

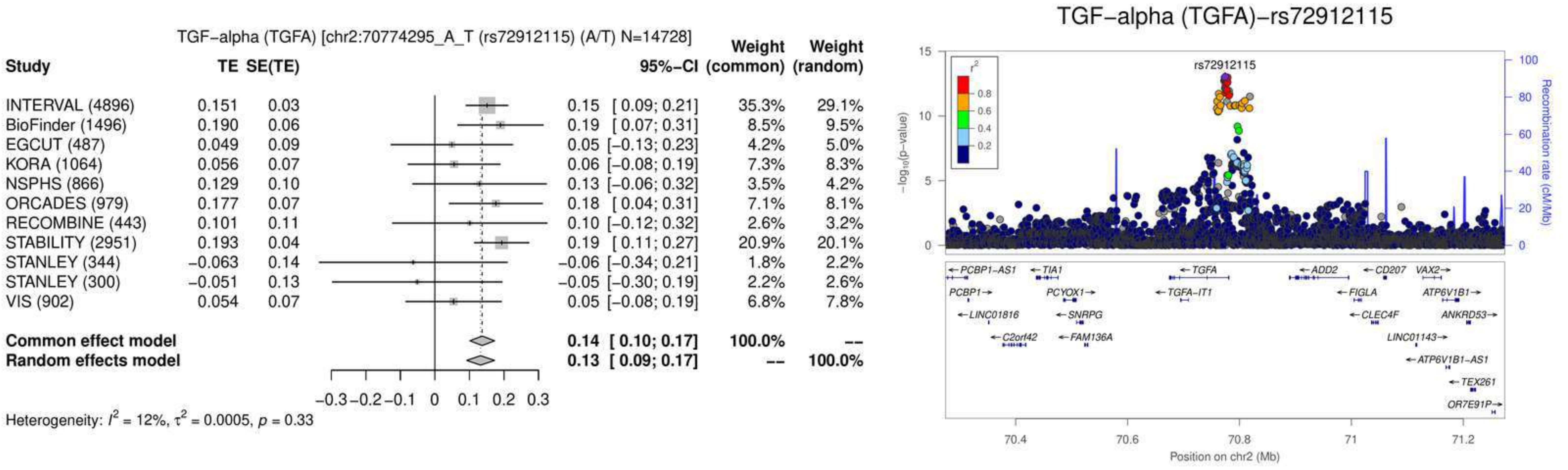
TGF−alpha (TGFA) [chr2:70774295_A_T (rs72912115) (A/T) N=14728]
Study | TE | SE(TE) | 95%−CI | Weight (common) | Weight (random)
INTERVAL (4896) | 0.151 | 0.03 | 0.15 [0.09; 0.21] | 35.3% | 29.1%
BioFinder (1496) | 0.190 | 0.06 | 0.19 [0.07; 0.31] | 8.5% | 9.5%
EGCUT (487) | 0.049 | 0.09 | 0.05 [−0.13; 0.23] | 4.2% | 5.0%
KORA (1064) | 0.056 | 0.07 | 0.06 [−0.08; 0.19] | 7.3% | 8.3%
NSPHS (866) | 0.129 | 0.10 | 0.13 [−0.06; 0.32] | 3.5% | 4.2%
ORCADES (979) | 0.177 | 0.07 | 0.18 [0.04; 0.31] | 7.1% | 8.1%
RECOMBINE (443) | 0.101 | 0.11 | 0.10 [−0.12; 0.32] | 2.6% | 3.2%
STABILITY (2951) | 0.193 | 0.04 | 0.19 [0.11; 0.27] | 20.9% | 20.1%
STANLEY (344) | −0.063 | 0.14 | −0.06 [−0.34; 0.21] | 1.8% | 2.2%
STANLEY (300) | −0.051 | 0.13 | −0.05 [−0.30; 0.19] | 2.2% | 2.6%
VIS (902) | 0.054 | 0.07 | 0.05 [−0.08; 0.19] | 6.8% | 7.8%
Common effect model | 0.14 [0.10; 0.17] | 100.0% | --
Random effects model | 0.13 [0.09; 0.17] | -- | 100.0%
−0.3 −0.2 −0.1 0 0.1 0.2 0.3
Heterogeneity: I² = 12%, τ² = 0.0005, p = 0.33
TGF−alpha (TGFA)−rs72912115
rs72912115
r²
0.8
0.6
0.4
0.2
−log10(p−value)
Recombination rate (cM/Mb)
PCBP1−AS1
TIA1
TGFA
ADD2
CD207
VAX2
PCBP1
PCYOX1
TGFA−IT1
FIGLA
ATP6V1B1
LINC01816
SNRPG
CLEC4F
ANKRD53
C2orf42
FAM136A
LINC01143
ATP6V1B1−AS1
TEX261
OR7E91P
70.4
70.6
70.8
71
71.2
Position on chr2 (Mb)

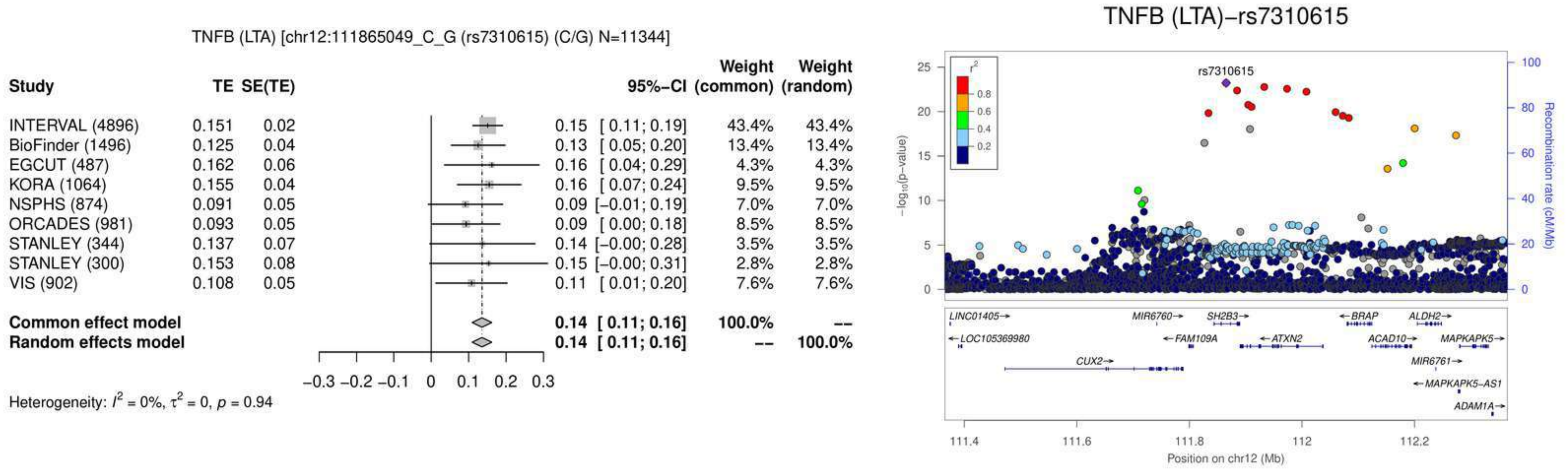
TNFB (LTA) [chr12:111865049_C_G (rs7310615) (C/G) N=11344]
Study
TE
SE(TE)
95%-CI
Weight (common)
Weight (random)
INTERVAL (4896) 0.151 0.02 0.15 [0.11; 0.19] 43.4% 43.4%
BioFinder (1496) 0.125 0.04 0.13 [0.05; 0.20] 13.4% 13.4%
EGCUT (487) 0.162 0.06 0.16 [0.04; 0.29] 4.3% 4.3%
KORA (1064) 0.155 0.04 0.16 [0.07; 0.24] 9.5% 9.5%
NSPHS (874) 0.091 0.05 0.09 [-0.01; 0.19] 7.0% 7.0%
ORCADES (981) 0.093 0.05 0.09 [0.00; 0.18] 8.5% 8.5%
STANLEY (344) 0.137 0.07 0.14 [-0.00; 0.28] 3.5% 3.5%
STANLEY (300) 0.153 0.08 0.15 [-0.00; 0.31] 2.8% 2.8%
VIS (902) 0.108 0.05 0.11 [0.01; 0.20] 7.6% 7.6%
Common effect model 0.14 [0.11; 0.16] 100.0% --
Random effects model 0.14 [0.11; 0.16] -- 100.0%
-0.3 -0.2 -0.1 0 0.1 0.2 0.3
Heterogeneity: I^2 = 0%, τ^2 = 0, p = 0.94
TNFB (LTA)-rs7310615
rs7310615
r^2
0.8
0.6
0.4
0.2
$-\log_{10}$(p-value)
Recombination rate (cM/Mb)
LINC01405
LOC105369980
CUX2
MIR6760
FAM109A
SH2B3
ATXN2
BRAP
ACAD10
ALDH2
MAPKAPK5
MIR6761
MAPKAPK5-AS1
ADAM1A
111.4
111.6
111.8
112
112.2
Position on chr12 (Mb)

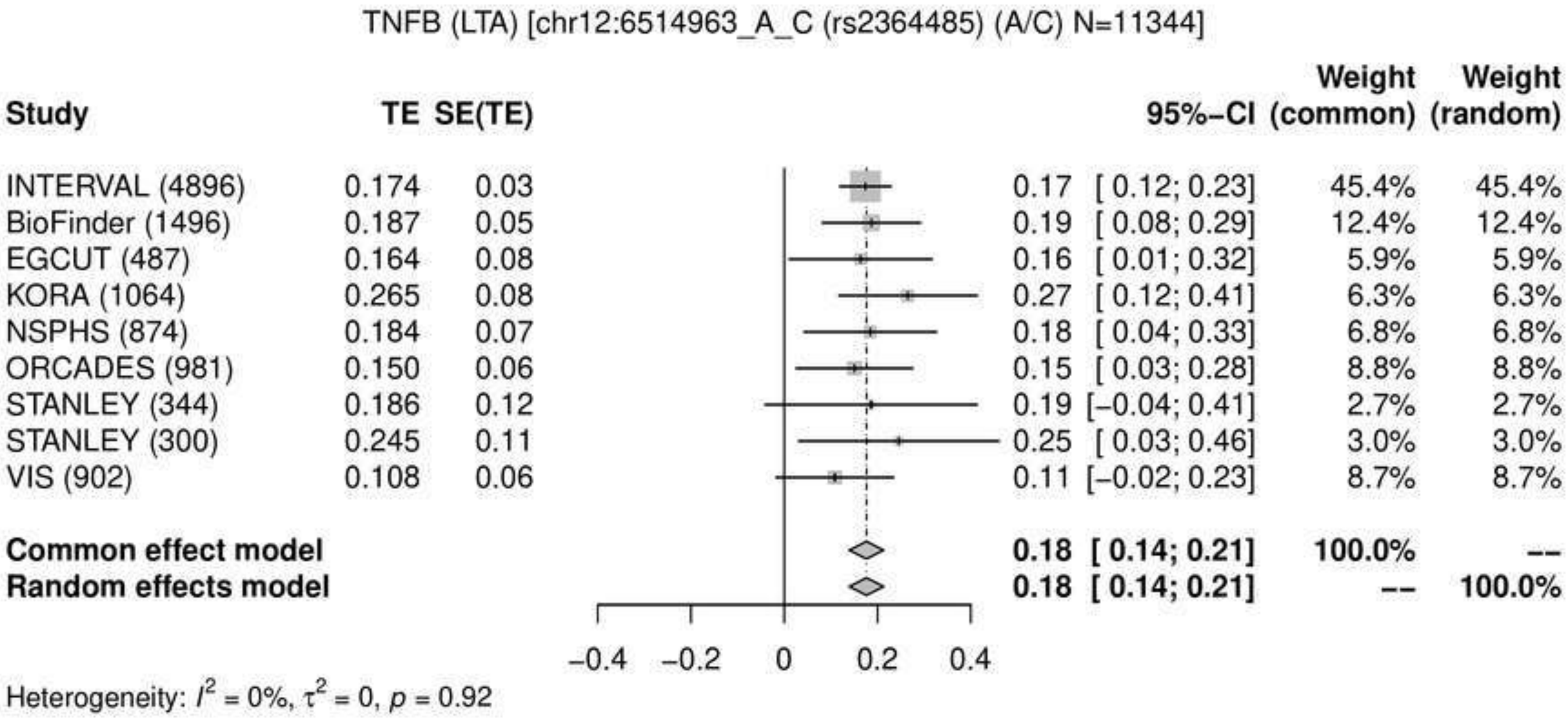
TNFB (LTA) [chr12:6514963_A_C (rs2364485) (A/C) N=11344]
Study
TE
SE(TE)
95%-CI
Weight (common)
Weight (random)
INTERVAL (4896) 0.174 0.03 0.17 [0.12; 0.23] 45.4% 45.4%
BioFinder (1496) 0.187 0.05 0.19 [0.08; 0.29] 12.4% 12.4%
EGCUT (487) 0.164 0.08 0.16 [0.01; 0.32] 5.9% 5.9%
KORA (1064) 0.265 0.08 0.27 [0.12; 0.41] 6.3% 6.3%
NSPHS (874) 0.184 0.07 0.18 [0.04; 0.33] 6.8% 6.8%
ORCADES (981) 0.150 0.06 0.15 [0.03; 0.28] 8.8% 8.8%
STANLEY (344) 0.186 0.12 0.19 [−0.04; 0.41] 2.7% 2.7%
STANLEY (300) 0.245 0.11 0.25 [0.03; 0.46] 3.0% 3.0%
VIS (902) 0.108 0.06 0.11 [−0.02; 0.23] 8.7% 8.7%
Common effect model 0.18 [0.14; 0.21] 100.0% --
Random effects model 0.18 [0.14; 0.21] -- 100.0%
−0.4 −0.2 0 0.2 0.4
Heterogeneity: $I^2 = 0\%$, $\tau^2 = 0$, $p = 0.92$

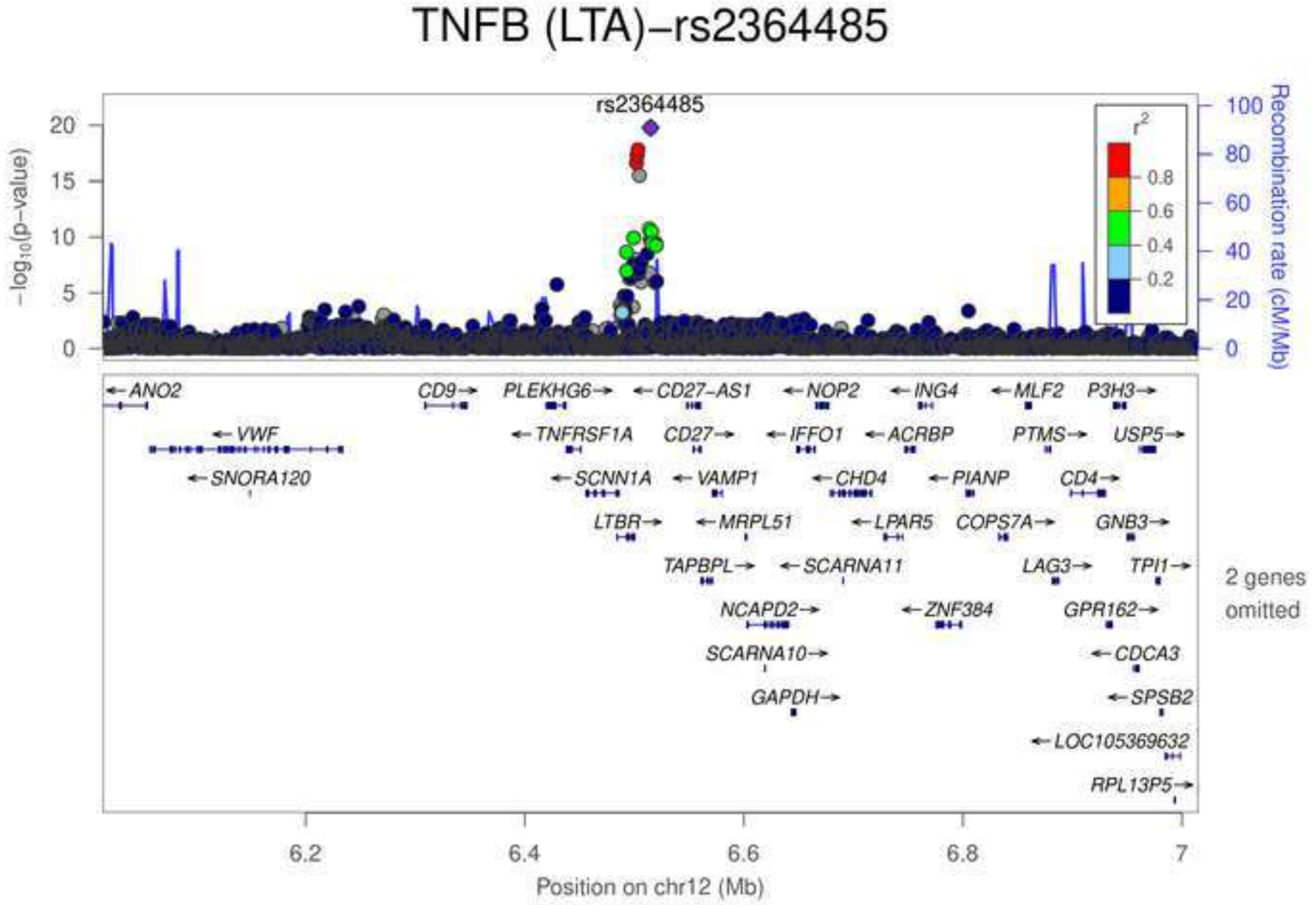
TNFB (LTA)−rs2364485
rs2364485
−log10(p−value)
Recombination rate (cM/Mb)
r2
0.8
0.6
0.4
0.2
ANO2
VWF
SNORA120
CD9
PLEKHG6
TNFRSF1A
SCNN1A
LTBR
CD27−AS1
CD27
VAMP1
MRPL51
TAPBPL
NCAPD2
SCARNA10
GAPDH
NOP2
IFFO1
CHD4
LPAR5
SCARNA11
ZNF384
ING4
ACRBP
PIANP
COPS7A
MLF2
PTMS
LAG3
P3H3
USP5
CD4
GNB3
TPI1
GPR162
CDCA3
SPSB2
LOC105369632
RPL13P5
2 genes omitted
6.2
6.4
6.6
6.8
7
Position on chr12 (Mb)

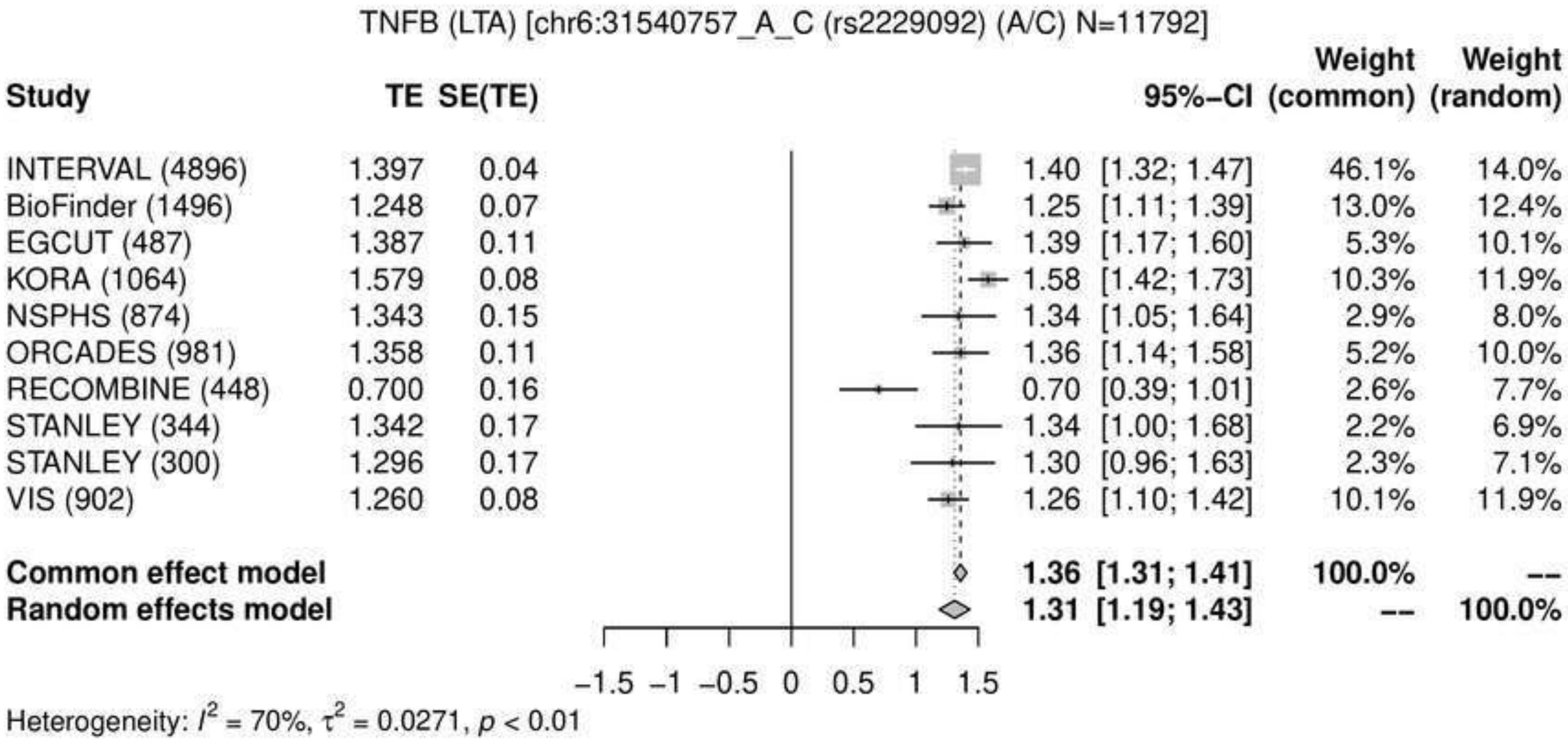
TNFB (LTA) [chr6:31540757_A_C (rs2229092) (A/C) N=11792]
Study TE SE(TE) 95%-CI Weight (common) Weight (random)
INTERVAL (4896) 1.397 0.04 1.40 [1.32; 1.47] 46.1% 14.0%
BioFinder (1496) 1.248 0.07 1.25 [1.11; 1.39] 13.0% 12.4%
EGCUT (487) 1.387 0.11 1.39 [1.17; 1.60] 5.3% 10.1%
KORA (1064) 1.579 0.08 1.58 [1.42; 1.73] 10.3% 11.9%
NSPHS (874) 1.343 0.15 1.34 [1.05; 1.64] 2.9% 8.0%
ORCADES (981) 1.358 0.11 1.36 [1.14; 1.58] 5.2% 10.0%
RECOMBINE (448) 0.700 0.16 0.70 [0.39; 1.01] 2.6% 7.7%
STANLEY (344) 1.342 0.17 1.34 [1.00; 1.68] 2.2% 6.9%
STANLEY (300) 1.296 0.17 1.30 [0.96; 1.63] 2.3% 7.1%
VIS (902) 1.260 0.08 1.26 [1.10; 1.42] 10.1% 11.9%
Common effect model 1.36 [1.31; 1.41] 100.0% --
Random effects model 1.31 [1.19; 1.43] -- 100.0%
−1.5 −1 −0.5 0 0.5 1 1.5
Heterogeneity: I^2 = 70%, τ^2 = 0.0271, p < 0.01

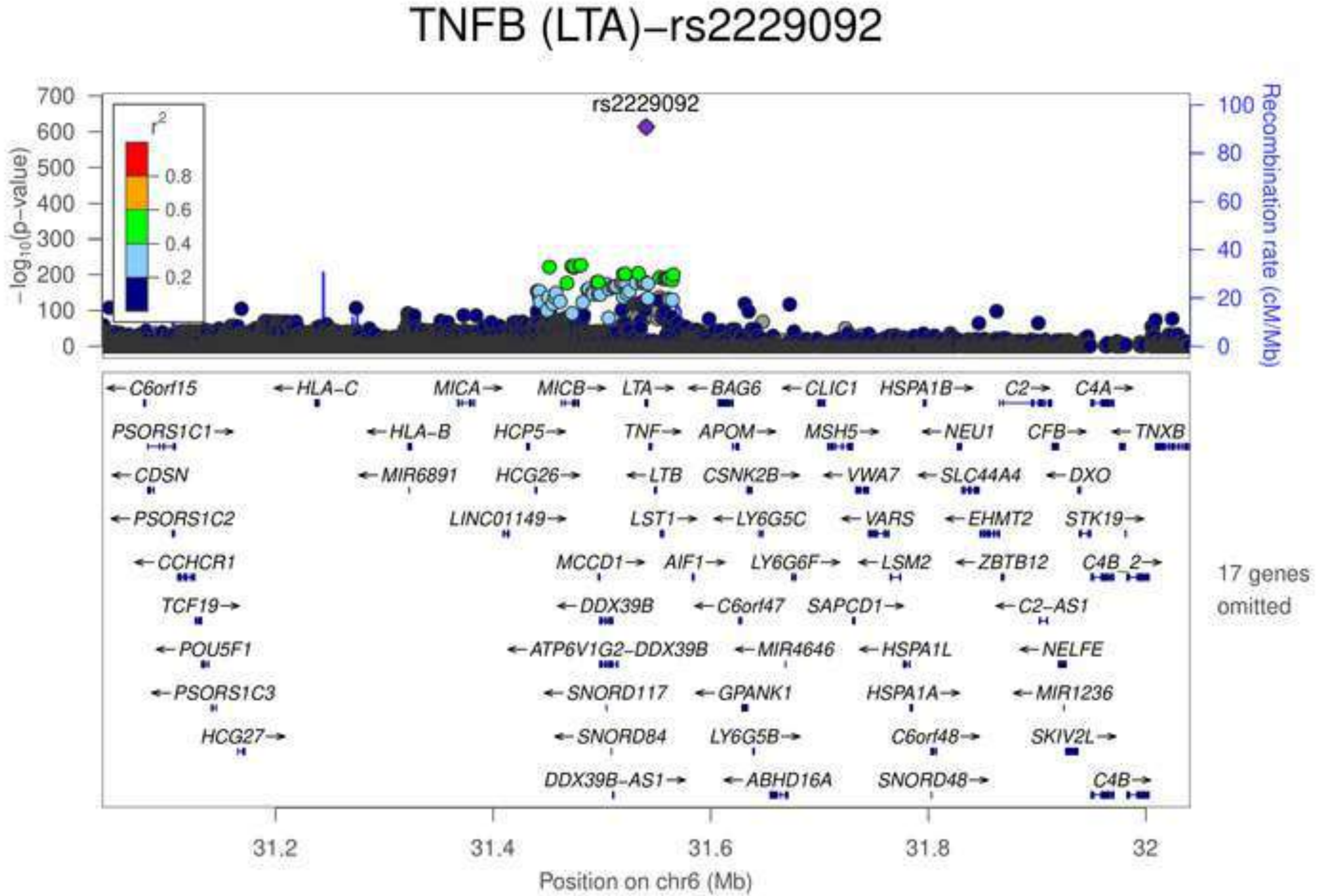
TNFB (LTA)−rs2229092
rs2229092
$-\log_{10}$(p−value)
Recombination rate (cM/Mb)
r^2
0.8
0.6
0.4
0.2
17 genes omitted
Position on chr6 (Mb)
31.2
31.4
31.6
31.8
32

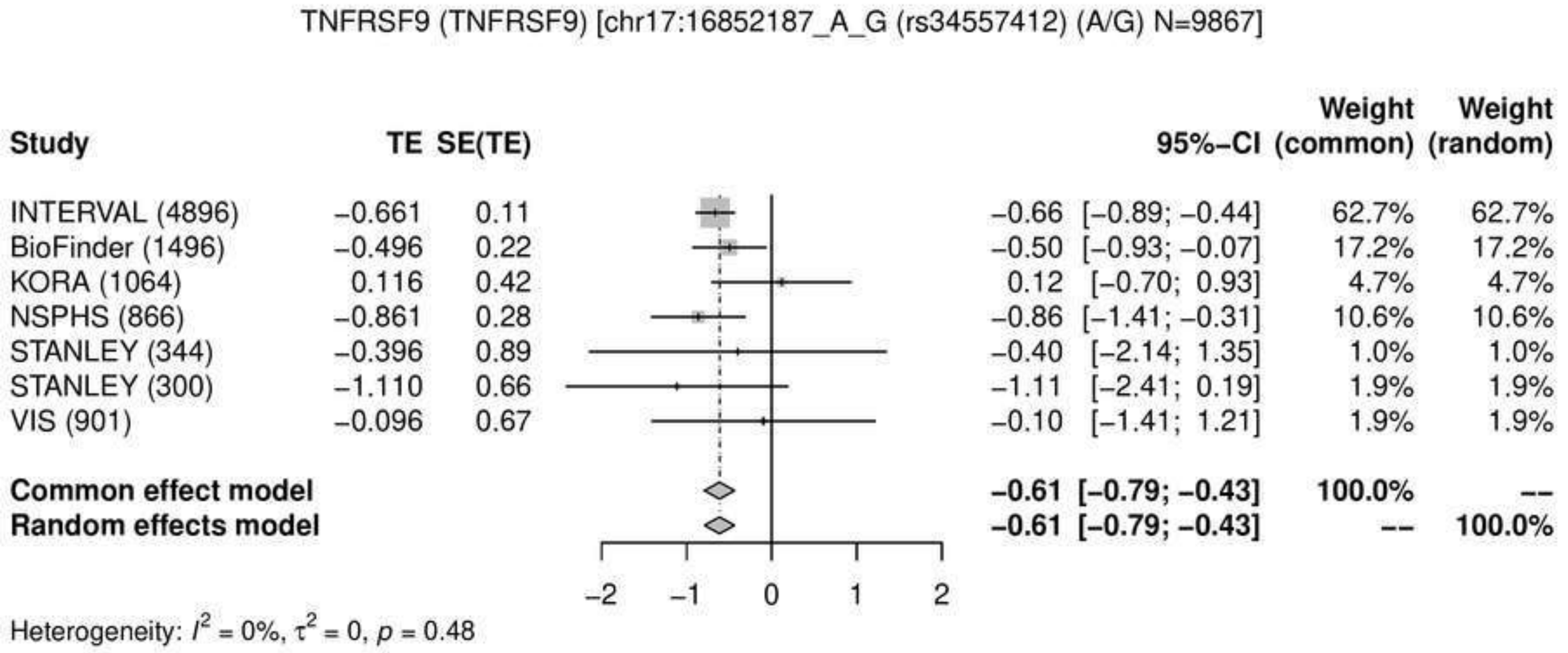
TNFRSF9 (TNFRSF9) [chr17:16852187_A_G (rs34557412) (A/G) N=9867]
Study
TE
SE(TE)
95%-CI
Weight (common)
Weight (random)
INTERVAL (4896) −0.661 0.11 −0.66 [−0.89; −0.44] 62.7% 62.7%
BioFinder (1496) −0.496 0.22 −0.50 [−0.93; −0.07] 17.2% 17.2%
KORA (1064) 0.116 0.42 0.12 [−0.70; 0.93] 4.7% 4.7%
NSPHS (866) −0.861 0.28 −0.86 [−1.41; −0.31] 10.6% 10.6%
STANLEY (344) −0.396 0.89 −0.40 [−2.14; 1.35] 1.0% 1.0%
STANLEY (300) −1.110 0.66 −1.11 [−2.41; 0.19] 1.9% 1.9%
VIS (901) −0.096 0.67 −0.10 [−1.41; 1.21] 1.9% 1.9%
Common effect model −0.61 [−0.79; −0.43] 100.0% --
Random effects model −0.61 [−0.79; −0.43] -- 100.0%
−2 −1 0 1 2
Heterogeneity: I^2 = 0%, τ^2 = 0, p = 0.48
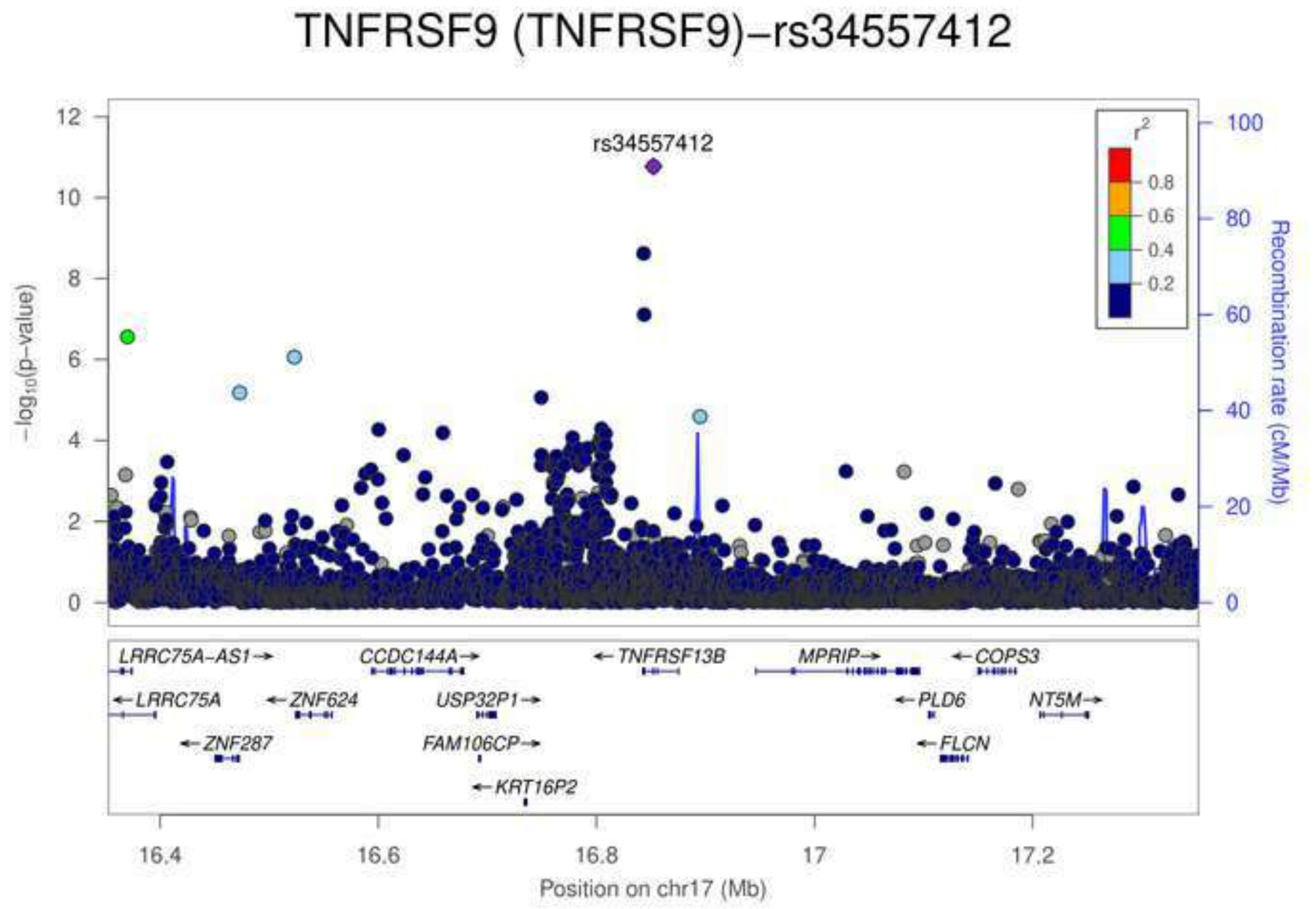
TNFRSF9 (TNFRSF9)−rs34557412
rs34557412
$-\log_{10}$(p-value)
Recombination rate (cM/Mb)
r^2
0.8
0.6
0.4
0.2
0 2 4 6 8 10 12
0 20 40 60 80 100
LRRC75A-AS1→
CCDC144A→
←TNFRSF13B
MPRIP→
←COPS3
←LRRC75A
←ZNF624
USP32P1→
←PLD6
NT5M→
←ZNF287
FAM106CP→
←FLCN
←KRT16P2
16.4 16.6 16.8 17 17.2
Position on chr17 (Mb)

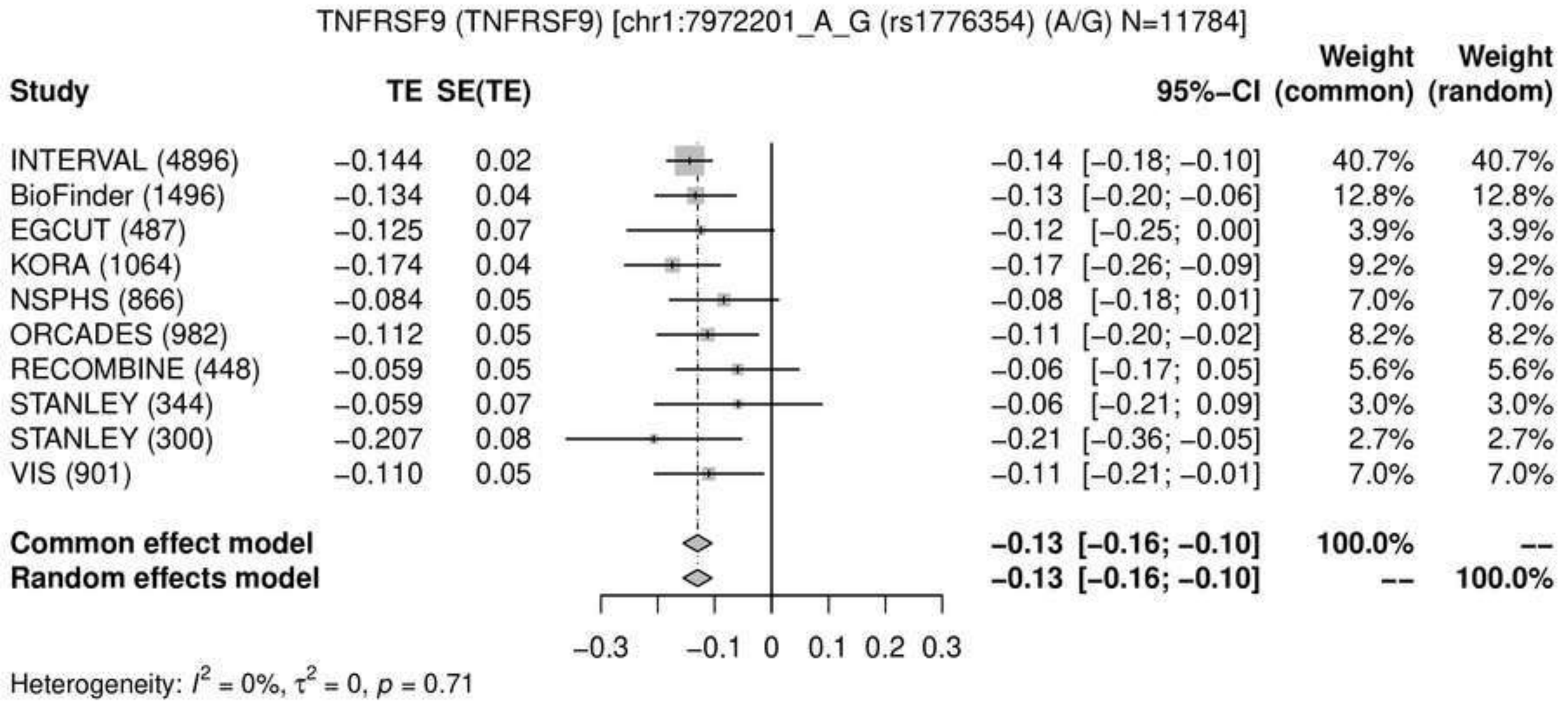

TNFRSF9 (TNFRSF9) [chr1:7972201_A_G (rs1776354) (A/G) N=11784]
Study
TE
SE(TE)
95%-CI
Weight (common)
Weight (random)
INTERVAL (4896) −0.144 0.02 −0.14 [−0.18; −0.10] 40.7% 40.7%
BioFinder (1496) −0.134 0.04 −0.13 [−0.20; −0.06] 12.8% 12.8%
EGCUT (487) −0.125 0.07 −0.12 [−0.25; 0.00] 3.9% 3.9%
KORA (1064) −0.174 0.04 −0.17 [−0.26; −0.09] 9.2% 9.2%
NSPHS (866) −0.084 0.05 −0.08 [−0.18; 0.01] 7.0% 7.0%
ORCADES (982) −0.112 0.05 −0.11 [−0.20; −0.02] 8.2% 8.2%
RECOMBINE (448) −0.059 0.05 −0.06 [−0.17; 0.05] 5.6% 5.6%
STANLEY (344) −0.059 0.07 −0.06 [−0.21; 0.09] 3.0% 3.0%
STANLEY (300) −0.207 0.08 −0.21 [−0.36; −0.05] 2.7% 2.7%
VIS (901) −0.110 0.05 −0.11 [−0.21; −0.01] 7.0% 7.0%
Common effect model −0.13 [−0.16; −0.10] 100.0% --
Random effects model −0.13 [−0.16; −0.10] -- 100.0%
−0.3 −0.1 0 0.1 0.2 0.3
Heterogeneity: I^2 = 0%, τ^2 = 0, p = 0.71

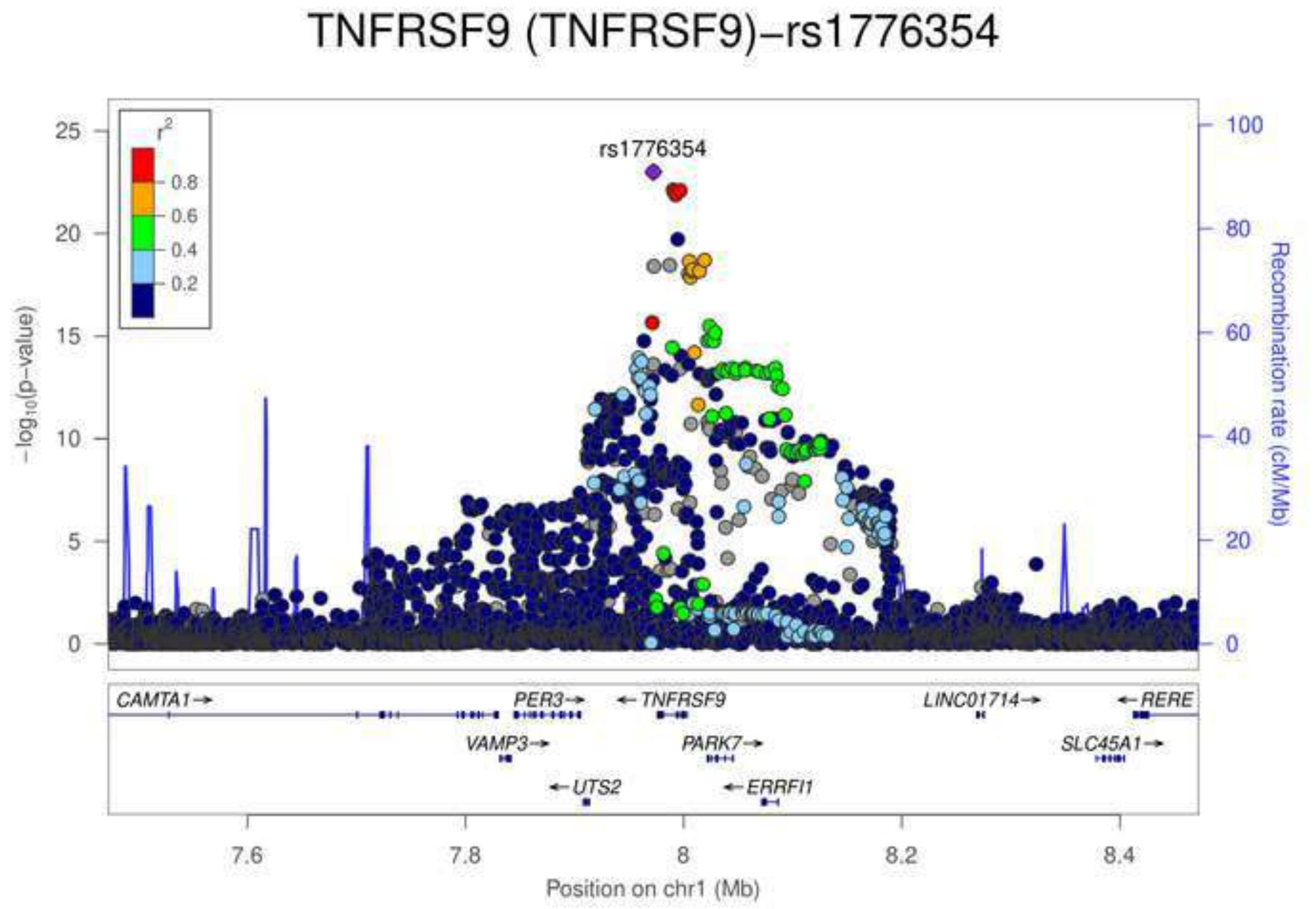

TNFRSF9 (TNFRSF9)−rs1776354
rs1776354
r^2
0.8
0.6
0.4
0.2
$-\log_{10}$(p-value)
Recombination rate (cM/Mb)
CAMTA1→
PER3→
←TNFRSF9
LINC01714→
←RERE
VAMP3→
PARK7→
SLC45A1→
←UTS2
←ERRFI1
7.6
7.8
8
8.2
8.4
Position on chr1 (Mb)

TNFSF14 (TNFSF14) [chr19:6661549_C_T (rs344562) (T/C) N=11789]

Study	TE	SE(TE)	95%-CI	Weight (common)	Weight (random)
INTERVAL (4896)	−0.516	0.04	−0.52 [−0.60; −0.43]	41.0%	13.6%
BioFinder (1496)	−0.277	0.08	−0.28 [−0.43; −0.12]	11.1%	11.7%
EGCUT (487)	−0.606	0.13	−0.61 [−0.85; −0.36]	4.5%	9.2%
KORA (1064)	−0.352	0.09	−0.35 [−0.53; −0.17]	8.2%	11.0%
NSPHS (874)	−0.417	0.10	−0.42 [−0.61; −0.23]	7.6%	10.8%
ORCADES (982)	−0.809	0.11	−0.81 [−1.03; −0.59]	5.5%	9.9%
RECOMBINE (444)	−0.115	0.12	−0.11 [−0.36; 0.13]	4.7%	9.4%
STANLEY (344)	−0.555	0.24	−0.56 [−1.02; −0.09]	1.3%	4.8%
STANLEY (300)	−0.311	0.16	−0.31 [−0.63; 0.01]	2.7%	7.5%
VIS (902)	−0.619	0.07	−0.62 [−0.76; −0.48]	13.3%	12.1%
**Common effect model**			**−0.48 [−0.53; −0.43]**	**100.0%**	**--**
**Random effects model**			**−0.46 [−0.58; −0.33]**	**--**	**100.0%**

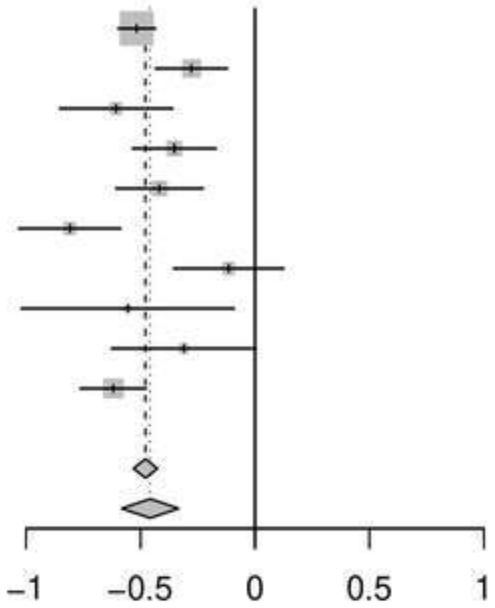

Heterogeneity: $I^2 = 72\%$, $\tau^2 = 0.0280$, $p < 0.01$

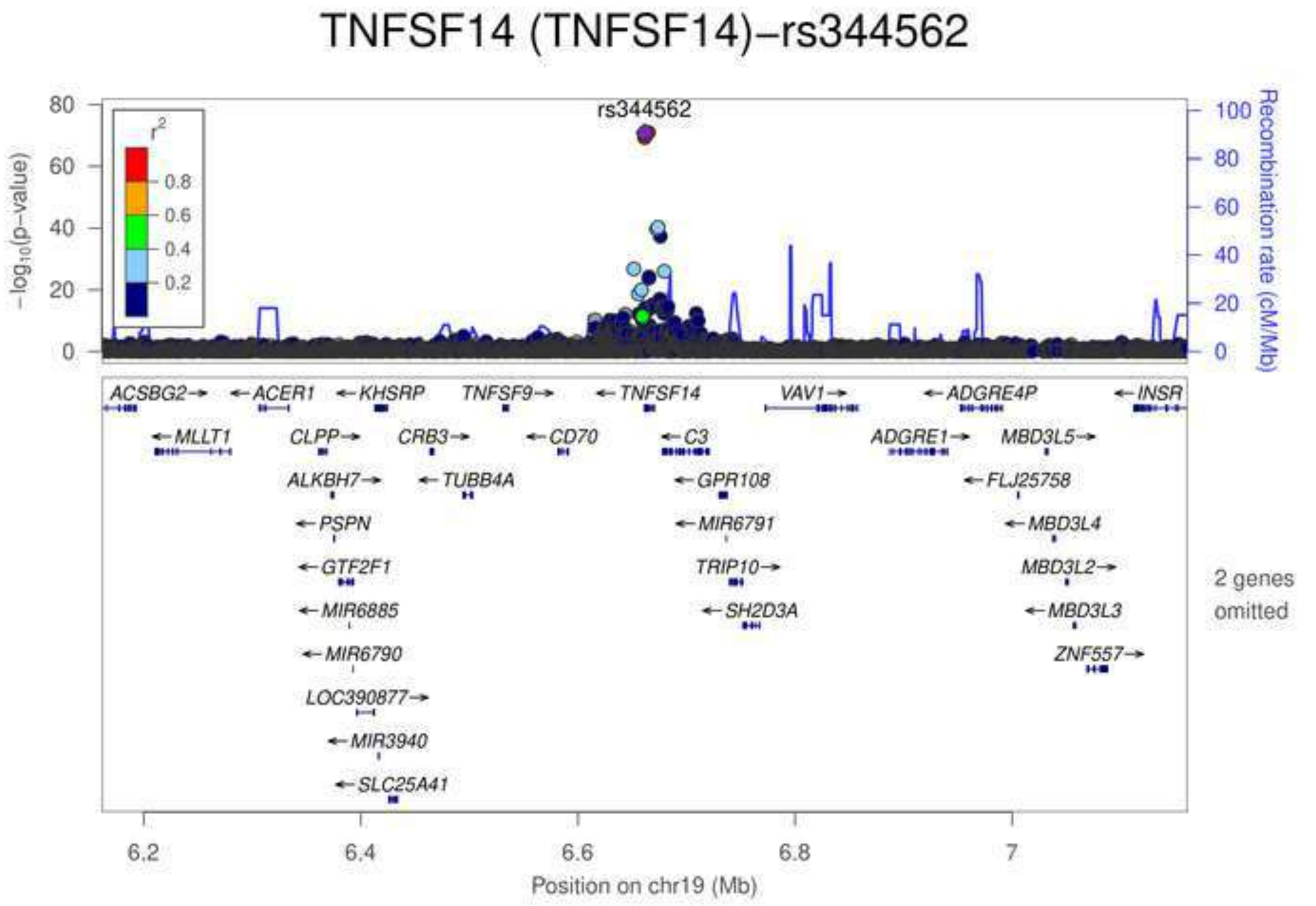

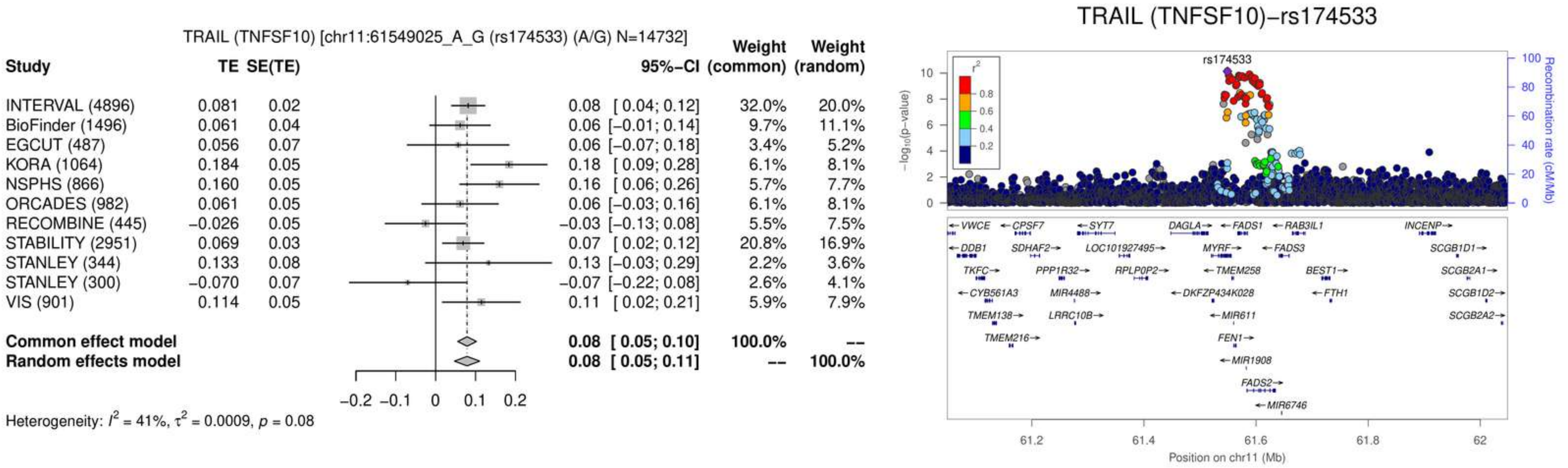
TRAIL (TNFSF10) [chr11:61549025_A_G (rs174533) (A/G) N=14732]
Study | TE | SE(TE) | 95%-CI | Weight (common) | Weight (random)
INTERVAL (4896) | 0.081 | 0.02 | 0.08 [0.04; 0.12] | 32.0% | 20.0%
BioFinder (1496) | 0.061 | 0.04 | 0.06 [−0.01; 0.14] | 9.7% | 11.1%
EGCUT (487) | 0.056 | 0.07 | 0.06 [−0.07; 0.18] | 3.4% | 5.2%
KORA (1064) | 0.184 | 0.05 | 0.18 [0.09; 0.28] | 6.1% | 8.1%
NSPHS (866) | 0.160 | 0.05 | 0.16 [0.06; 0.26] | 5.7% | 7.7%
ORCADES (982) | 0.061 | 0.05 | 0.06 [−0.03; 0.16] | 6.1% | 8.1%
RECOMBINE (445) | −0.026 | 0.05 | −0.03 [−0.13; 0.08] | 5.5% | 7.5%
STABILITY (2951) | 0.069 | 0.03 | 0.07 [0.02; 0.12] | 20.8% | 16.9%
STANLEY (344) | 0.133 | 0.08 | 0.13 [−0.03; 0.29] | 2.2% | 3.6%
STANLEY (300) | −0.070 | 0.07 | −0.07 [−0.22; 0.08] | 2.6% | 4.1%
VIS (901) | 0.114 | 0.05 | 0.11 [0.02; 0.21] | 5.9% | 7.9%
Common effect model | 0.08 [0.05; 0.10] | 100.0% | --
Random effects model | 0.08 [0.05; 0.11] | -- | 100.0%
−0.2 −0.1 0 0.1 0.2
Heterogeneity: I^2 = 41%, τ^2 = 0.0009, p = 0.08
TRAIL (TNFSF10)−rs174533
rs174533
r^2
0.8
0.6
0.4
0.2
$-\log_{10}$(p−value)
Recombination rate (cM/Mb)
VWCE, CPSF7, SYT7, DAGLA, FADS1, RAB3IL1, INCENP
DDB1, SDHAF2, LOC101927495, MYRF, FADS3, SCGB1D1
TKFC, PPP1R32, RPLP0P2, TMEM258, BEST1, SCGB2A1
CYB561A3, MIR4488, DKFZP434K028, FTH1, SCGB1D2
TMEM138, LRRC10B, MIR611, SCGB2A2
TMEM216, FEN1
MIR1908
FADS2
MIR6746
61.2 61.4 61.6 61.8 62
Position on chr11 (Mb)

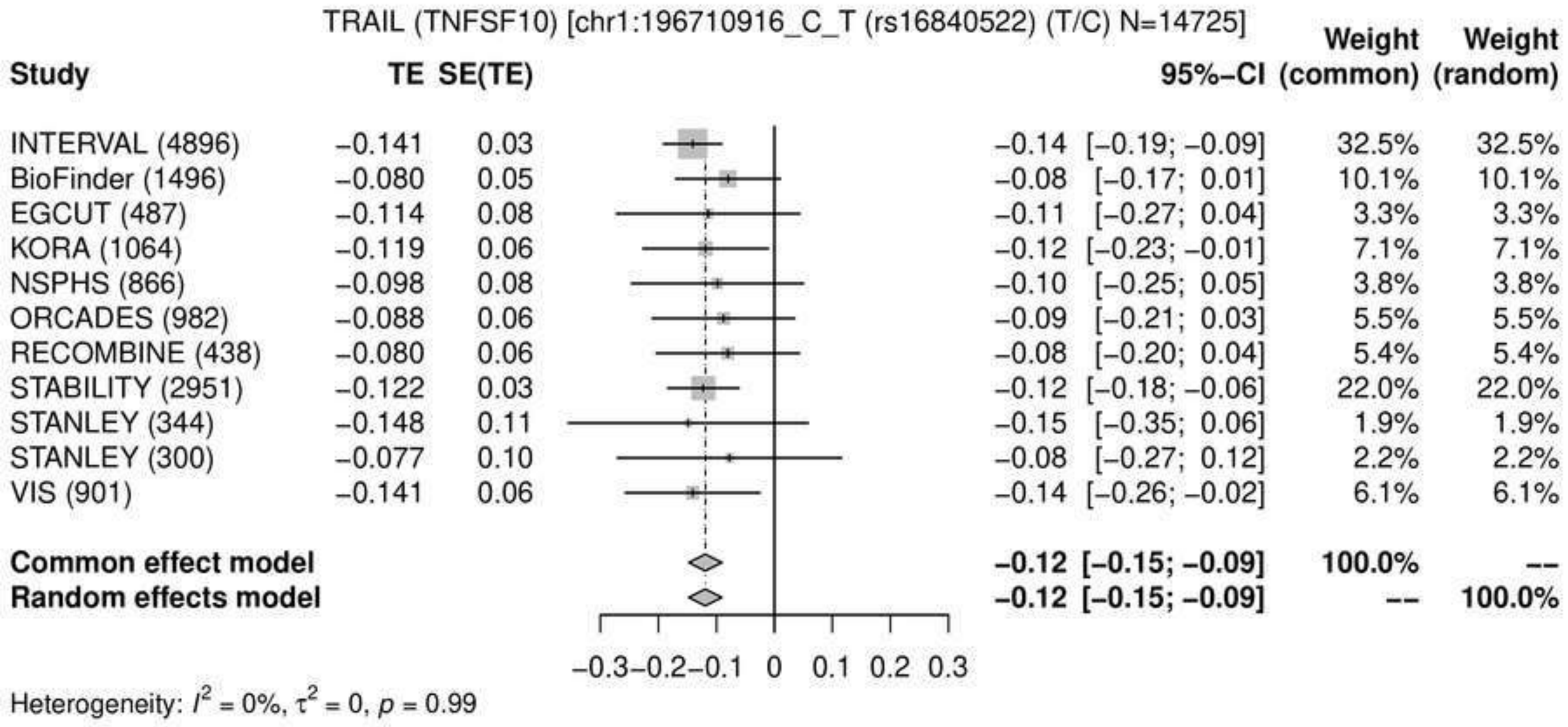
TRAIL (TNFSF10) [chr1:196710916_C_T (rs16840522) (T/C) N=14725]
Study TE SE(TE) 95%−CI Weight (common) Weight (random)
INTERVAL (4896) −0.141 0.03 −0.14 [−0.19; −0.09] 32.5% 32.5%
BioFinder (1496) −0.080 0.05 −0.08 [−0.17; 0.01] 10.1% 10.1%
EGCUT (487) −0.114 0.08 −0.11 [−0.27; 0.04] 3.3% 3.3%
KORA (1064) −0.119 0.06 −0.12 [−0.23; −0.01] 7.1% 7.1%
NSPHS (866) −0.098 0.08 −0.10 [−0.25; 0.05] 3.8% 3.8%
ORCADES (982) −0.088 0.06 −0.09 [−0.21; 0.03] 5.5% 5.5%
RECOMBINE (438) −0.080 0.06 −0.08 [−0.20; 0.04] 5.4% 5.4%
STABILITY (2951) −0.122 0.03 −0.12 [−0.18; −0.06] 22.0% 22.0%
STANLEY (344) −0.148 0.11 −0.15 [−0.35; 0.06] 1.9% 1.9%
STANLEY (300) −0.077 0.10 −0.08 [−0.27; 0.12] 2.2% 2.2%
VIS (901) −0.141 0.06 −0.14 [−0.26; −0.02] 6.1% 6.1%
Common effect model −0.12 [−0.15; −0.09] 100.0% --
Random effects model −0.12 [−0.15; −0.09] -- 100.0%
−0.3 −0.2 −0.1 0 0.1 0.2 0.3
Heterogeneity: $I^2 = 0\%$, $\tau^2 = 0$, $p = 0.99$

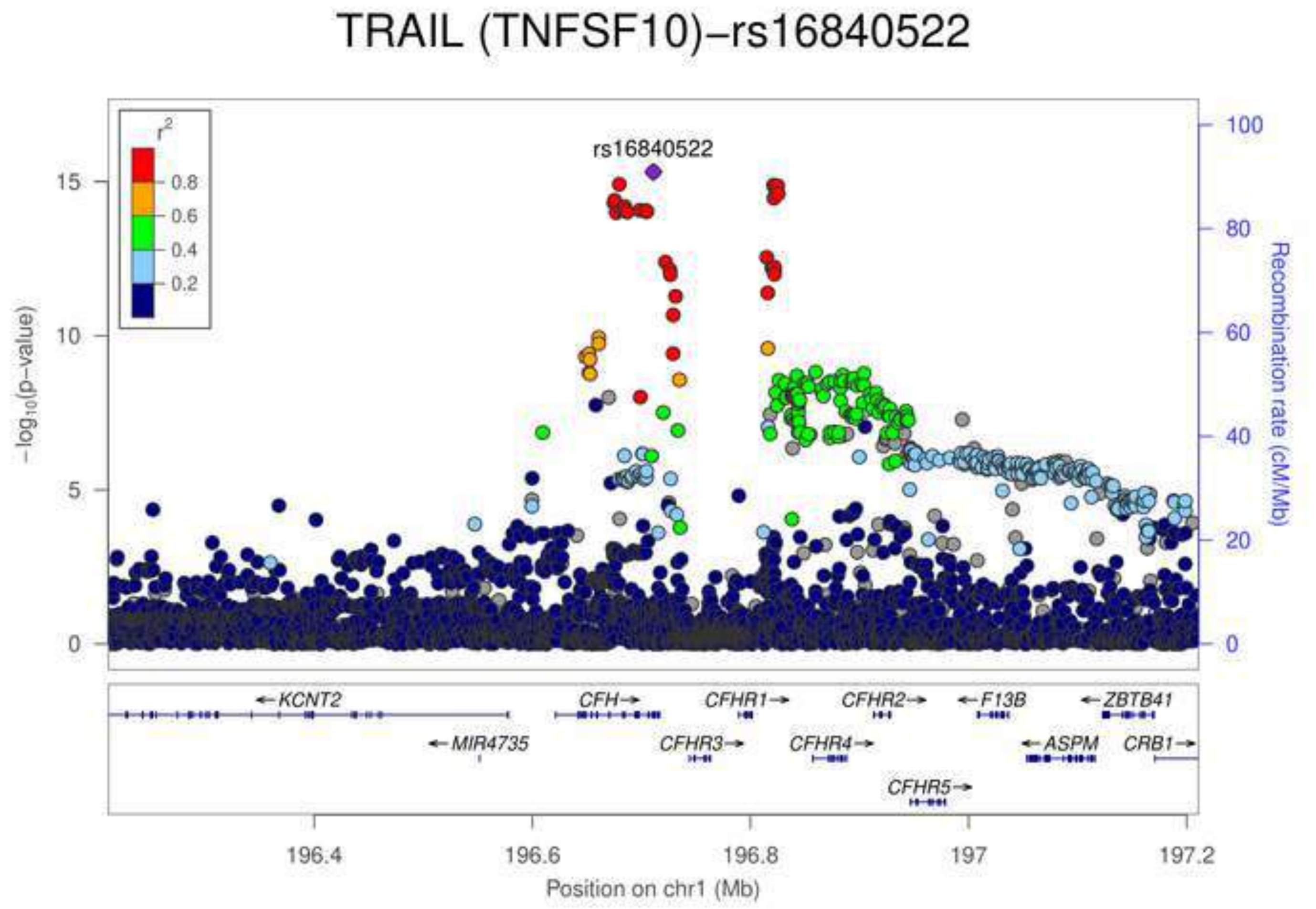
TRAIL (TNFSF10)−rs16840522
rs16840522
r^2
0.8
0.6
0.4
0.2
$-\log_{10}$(p−value)
Recombination rate (cM/Mb)
KCNT2
CFH
CFHR1
CFHR2
F13B
ZBTB41
MIR4735
CFHR3
CFHR4
ASPM
CRB1
CFHR5
196.4 196.6 196.8 197 197.2
Position on chr1 (Mb)

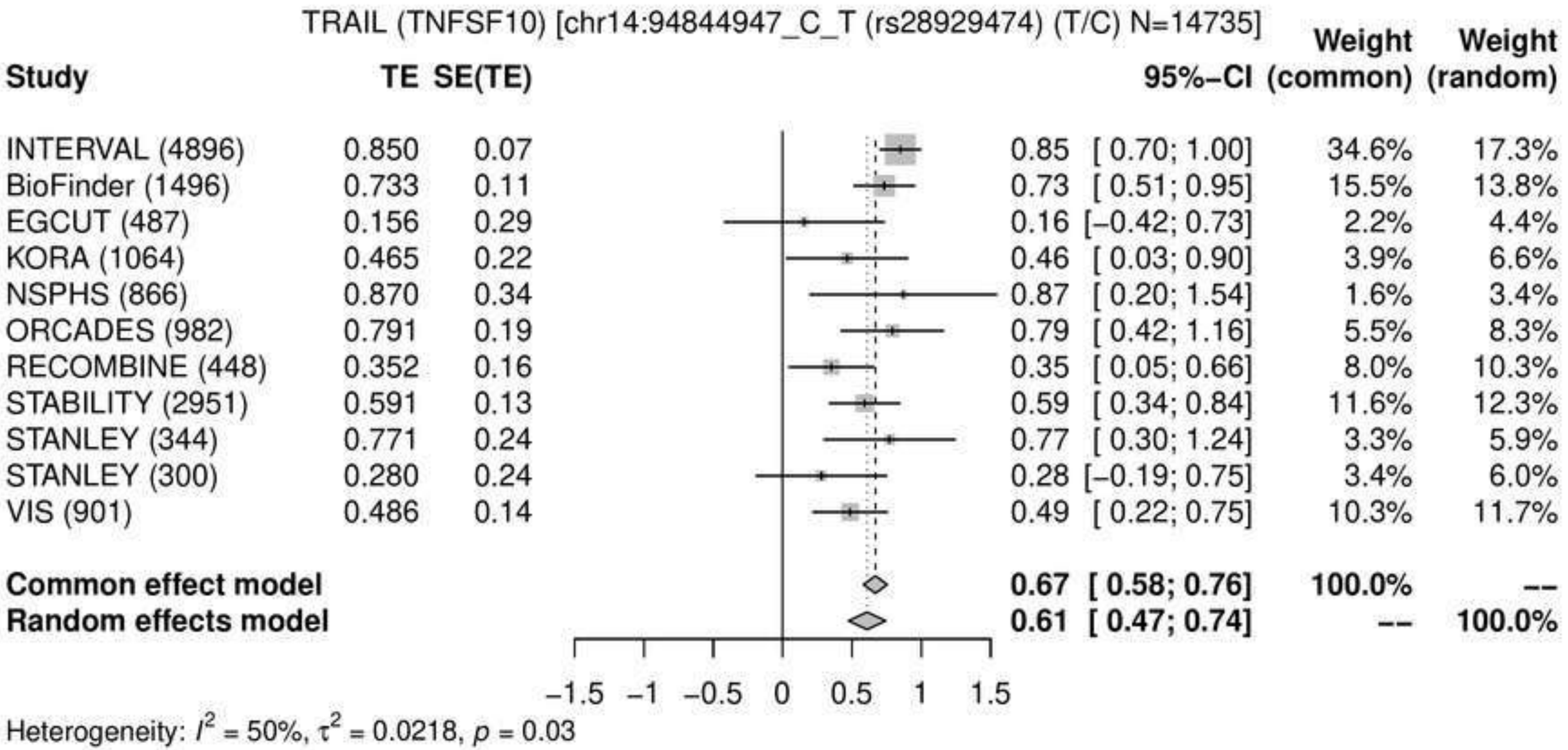

TRAIL (TNFSF10) [chr14:94844947_C_T (rs28929474) (T/C) N=14735]
Study
TE
SE(TE)
95%-CI
Weight (common)
Weight (random)
INTERVAL (4896) 0.850 0.07 0.85 [0.70; 1.00] 34.6% 17.3%
BioFinder (1496) 0.733 0.11 0.73 [0.51; 0.95] 15.5% 13.8%
EGCUT (487) 0.156 0.29 0.16 [−0.42; 0.73] 2.2% 4.4%
KORA (1064) 0.465 0.22 0.46 [0.03; 0.90] 3.9% 6.6%
NSPHS (866) 0.870 0.34 0.87 [0.20; 1.54] 1.6% 3.4%
ORCADES (982) 0.791 0.19 0.79 [0.42; 1.16] 5.5% 8.3%
RECOMBINE (448) 0.352 0.16 0.35 [0.05; 0.66] 8.0% 10.3%
STABILITY (2951) 0.591 0.13 0.59 [0.34; 0.84] 11.6% 12.3%
STANLEY (344) 0.771 0.24 0.77 [0.30; 1.24] 3.3% 5.9%
STANLEY (300) 0.280 0.24 0.28 [−0.19; 0.75] 3.4% 6.0%
VIS (901) 0.486 0.14 0.49 [0.22; 0.75] 10.3% 11.7%
Common effect model 0.67 [0.58; 0.76] 100.0% --
Random effects model 0.61 [0.47; 0.74] -- 100.0%
−1.5 −1 −0.5 0 0.5 1 1.5
Heterogeneity: I^2 = 50%, τ^2 = 0.0218, p = 0.03

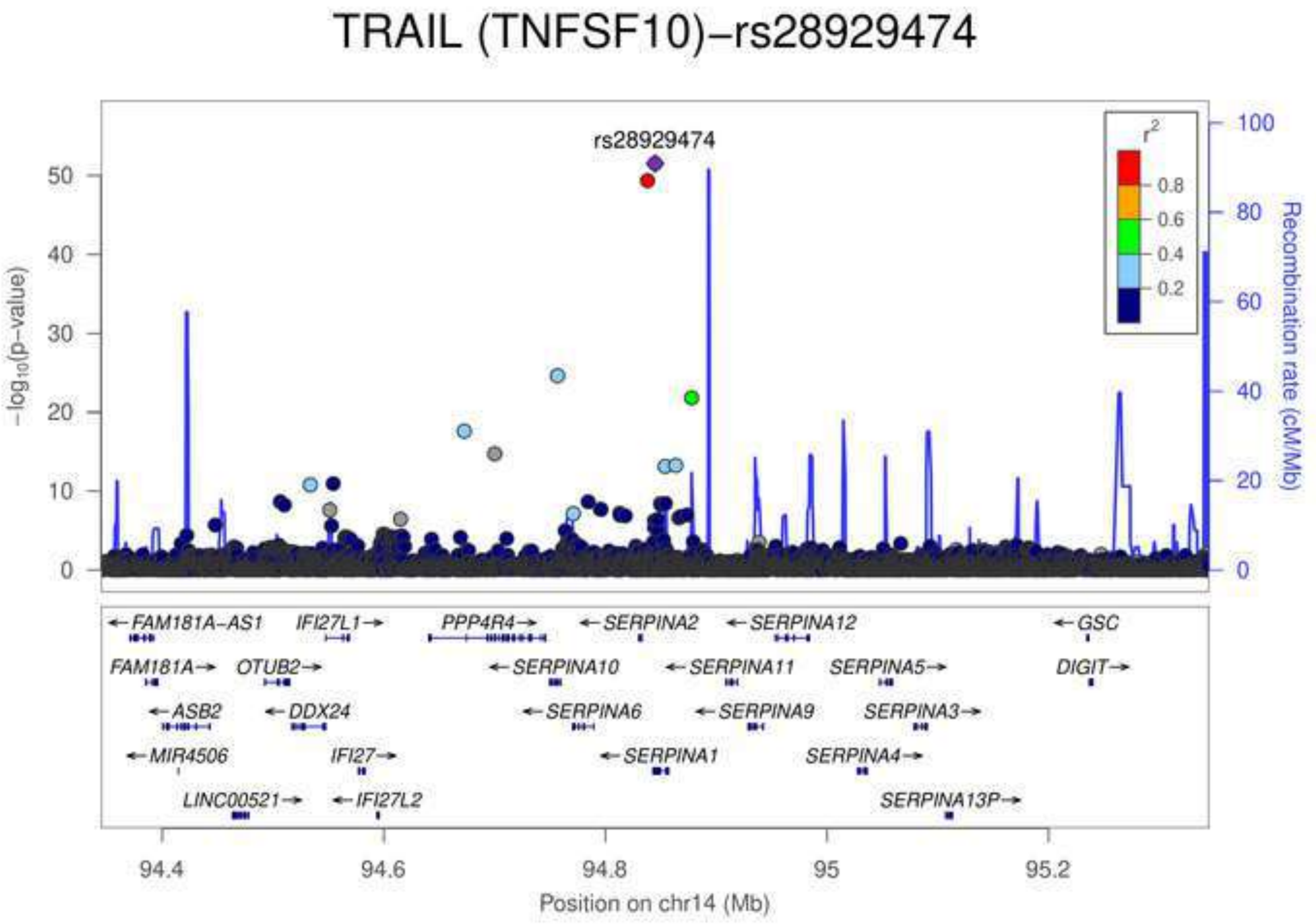

TRAIL (TNFSF10)−rs28929474
rs28929474
$-\log_{10}$(p−value)
Recombination rate (cM/Mb)
r^2
0.8
0.6
0.4
0.2
FAM181A-AS1
IFI27L1
PPP4R4
SERPINA2
SERPINA12
GSC
FAM181A
OTUB2
SERPINA10
SERPINA11
SERPINA5
DIGIT
ASB2
DDX24
SERPINA6
SERPINA9
SERPINA3
MIR4506
IFI27
SERPINA1
SERPINA4
LINC00521
IFI27L2
SERPINA13P
94.4 94.6 94.8 95 95.2
Position on chr14 (Mb)

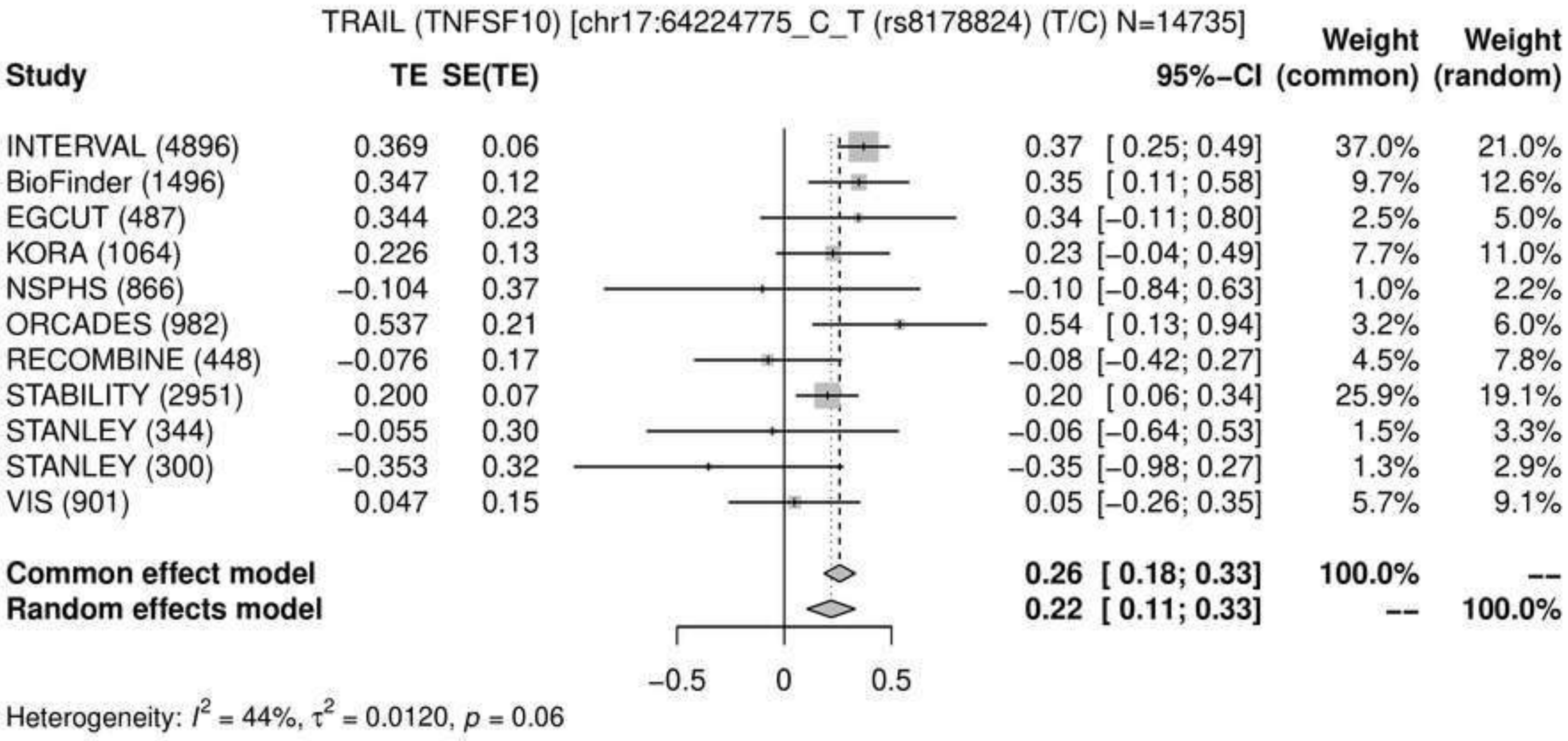
TRAIL (TNFSF10) [chr17:64224775_C_T (rs8178824) (T/C) N=14735]
Study TE SE(TE) 95%-CI Weight (common) Weight (random)
INTERVAL (4896) 0.369 0.06 0.37 [0.25; 0.49] 37.0% 21.0%
BioFinder (1496) 0.347 0.12 0.35 [0.11; 0.58] 9.7% 12.6%
EGCUT (487) 0.344 0.23 0.34 [−0.11; 0.80] 2.5% 5.0%
KORA (1064) 0.226 0.13 0.23 [−0.04; 0.49] 7.7% 11.0%
NSPHS (866) −0.104 0.37 −0.10 [−0.84; 0.63] 1.0% 2.2%
ORCADES (982) 0.537 0.21 0.54 [0.13; 0.94] 3.2% 6.0%
RECOMBINE (448) −0.076 0.17 −0.08 [−0.42; 0.27] 4.5% 7.8%
STABILITY (2951) 0.200 0.07 0.20 [0.06; 0.34] 25.9% 19.1%
STANLEY (344) −0.055 0.30 −0.06 [−0.64; 0.53] 1.5% 3.3%
STANLEY (300) −0.353 0.32 −0.35 [−0.98; 0.27] 1.3% 2.9%
VIS (901) 0.047 0.15 0.05 [−0.26; 0.35] 5.7% 9.1%
Common effect model 0.26 [0.18; 0.33] 100.0% --
Random effects model 0.22 [0.11; 0.33] -- 100.0%
−0.5 0 0.5
Heterogeneity: I^2 = 44%, τ^2 = 0.0120, p = 0.06

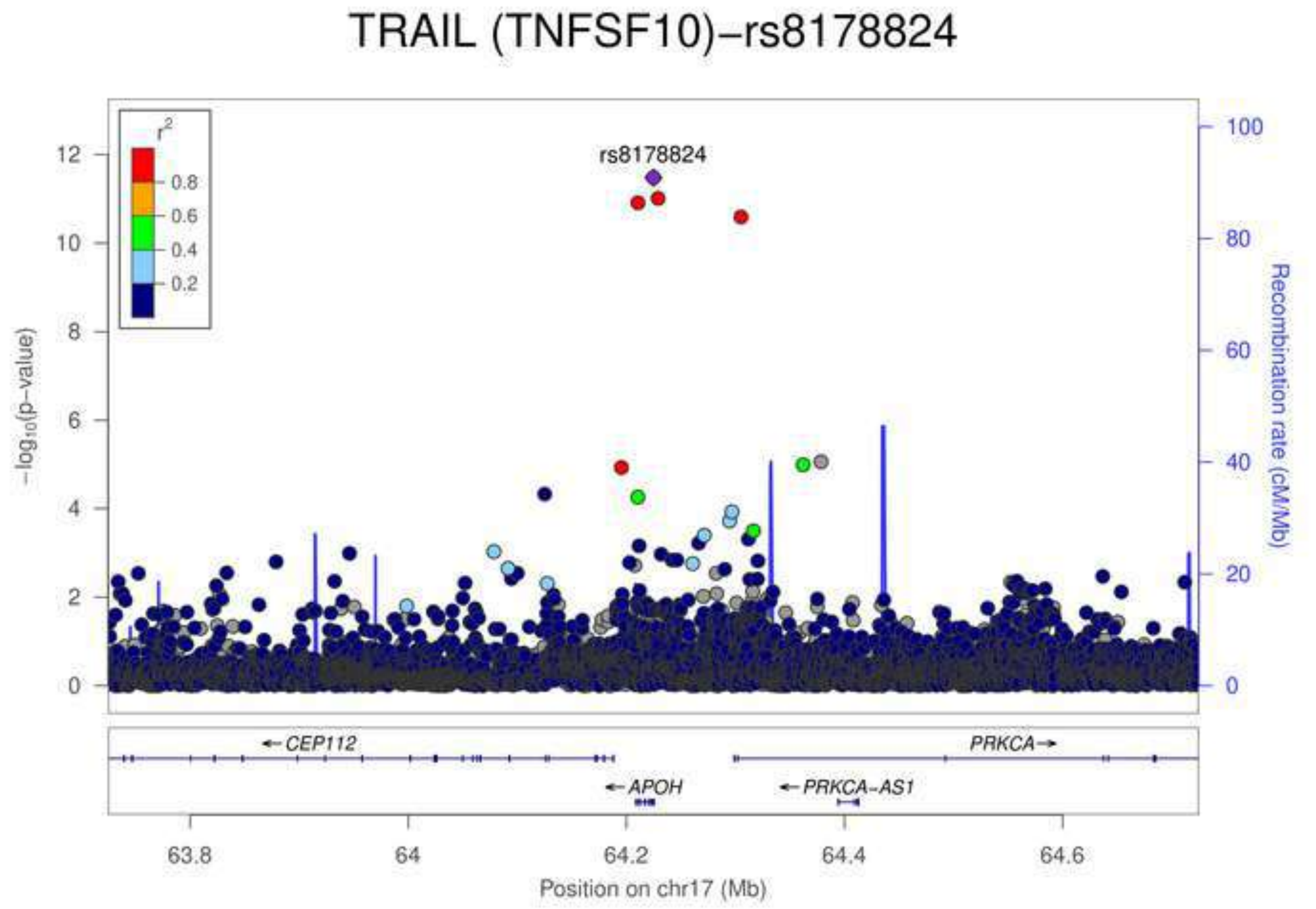
TRAIL (TNFSF10)−rs8178824
rs8178824
r^2
0.8
0.6
0.4
0.2
12
10
8
6
4
2
0
−log10(p−value)
100
80
60
40
20
0
Recombination rate (cM/Mb)
←CEP112
PRKCA→
←APOH
←PRKCA-AS1
63.8
64
64.2
64.4
64.6
Position on chr17 (Mb)

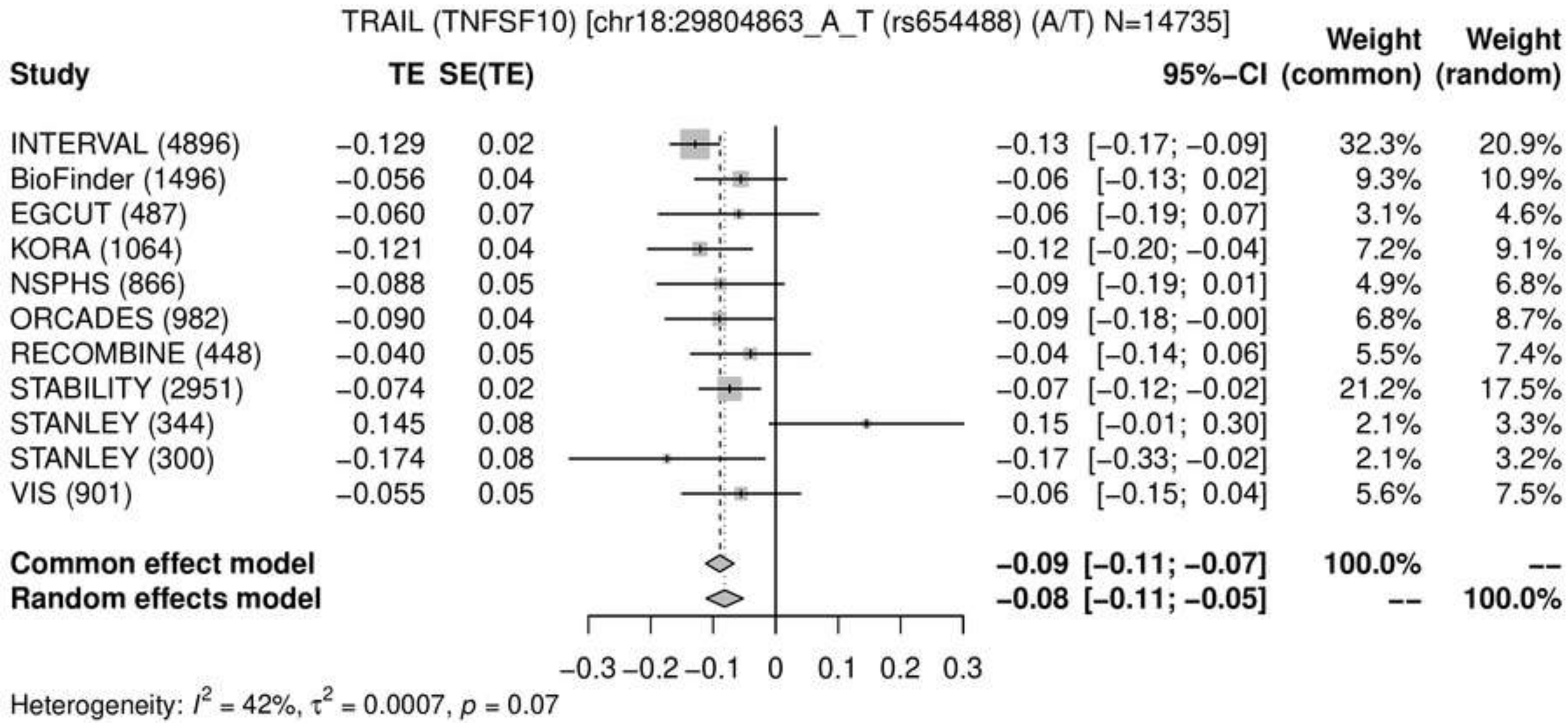
TRAIL (TNFSF10) [chr18:29804863_A_T (rs654488) (A/T) N=14735]
Study TE SE(TE) 95%-CI Weight (common) Weight (random)
INTERVAL (4896) −0.129 0.02 −0.13 [−0.17; −0.09] 32.3% 20.9%
BioFinder (1496) −0.056 0.04 −0.06 [−0.13; 0.02] 9.3% 10.9%
EGCUT (487) −0.060 0.07 −0.06 [−0.19; 0.07] 3.1% 4.6%
KORA (1064) −0.121 0.04 −0.12 [−0.20; −0.04] 7.2% 9.1%
NSPHS (866) −0.088 0.05 −0.09 [−0.19; 0.01] 4.9% 6.8%
ORCADES (982) −0.090 0.04 −0.09 [−0.18; −0.00] 6.8% 8.7%
RECOMBINE (448) −0.040 0.05 −0.04 [−0.14; 0.06] 5.5% 7.4%
STABILITY (2951) −0.074 0.02 −0.07 [−0.12; −0.02] 21.2% 17.5%
STANLEY (344) 0.145 0.08 0.15 [−0.01; 0.30] 2.1% 3.3%
STANLEY (300) −0.174 0.08 −0.17 [−0.33; −0.02] 2.1% 3.2%
VIS (901) −0.055 0.05 −0.06 [−0.15; 0.04] 5.6% 7.5%
Common effect model −0.09 [−0.11; −0.07] 100.0% --
Random effects model −0.08 [−0.11; −0.05] -- 100.0%
−0.3 −0.2 −0.1 0 0.1 0.2 0.3
Heterogeneity: I² = 42%, τ² = 0.0007, p = 0.07

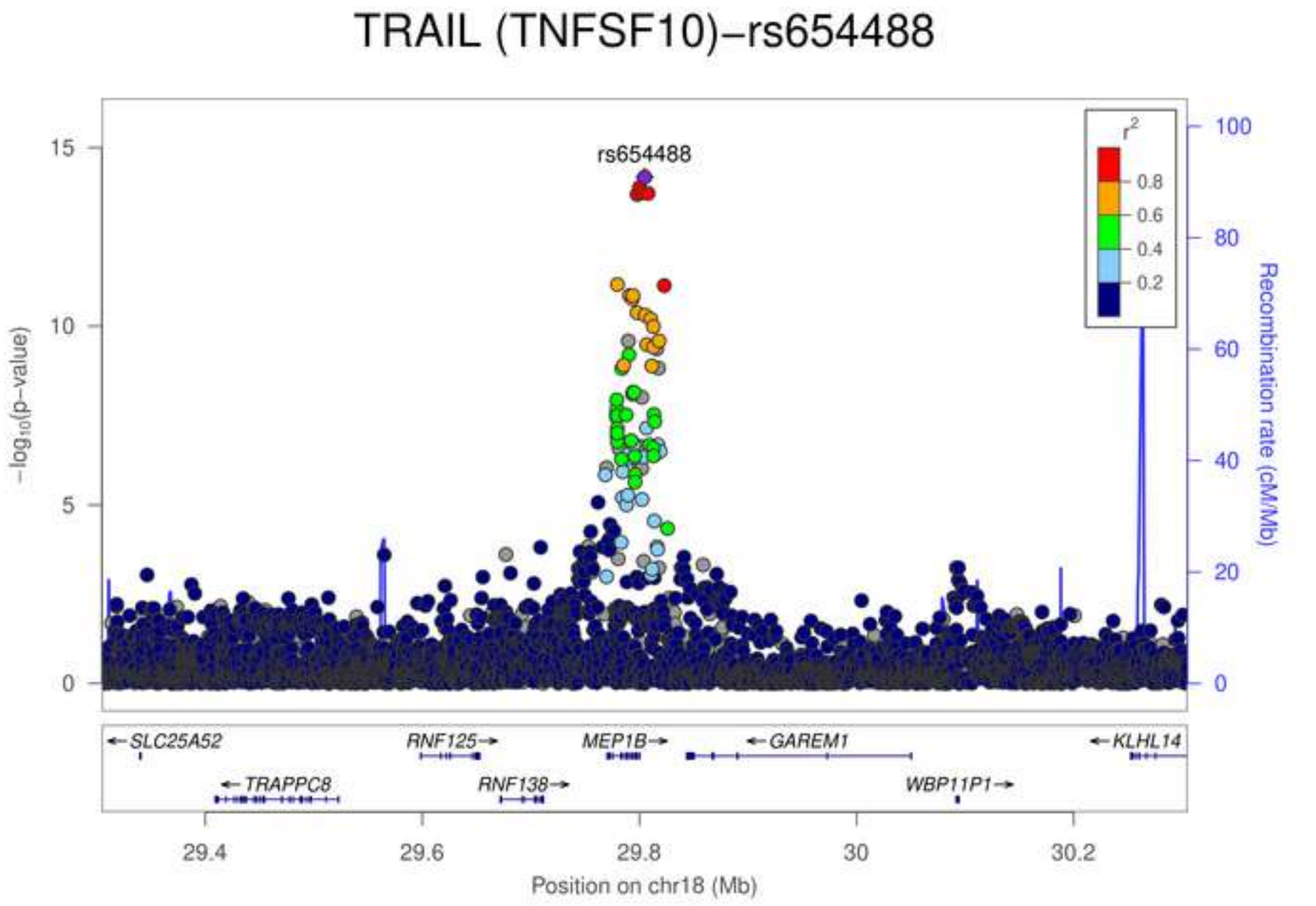
TRAIL (TNFSF10)−rs654488
rs654488
r²
0.8
0.6
0.4
0.2
−log10(p−value)
Recombination rate (cM/Mb)
SLC25A52
TRAPPC8
RNF125
RNF138
MEP1B
GAREM1
WBP11P1
KLHL14
29.4
29.6
29.8
30
30.2
Position on chr18 (Mb)

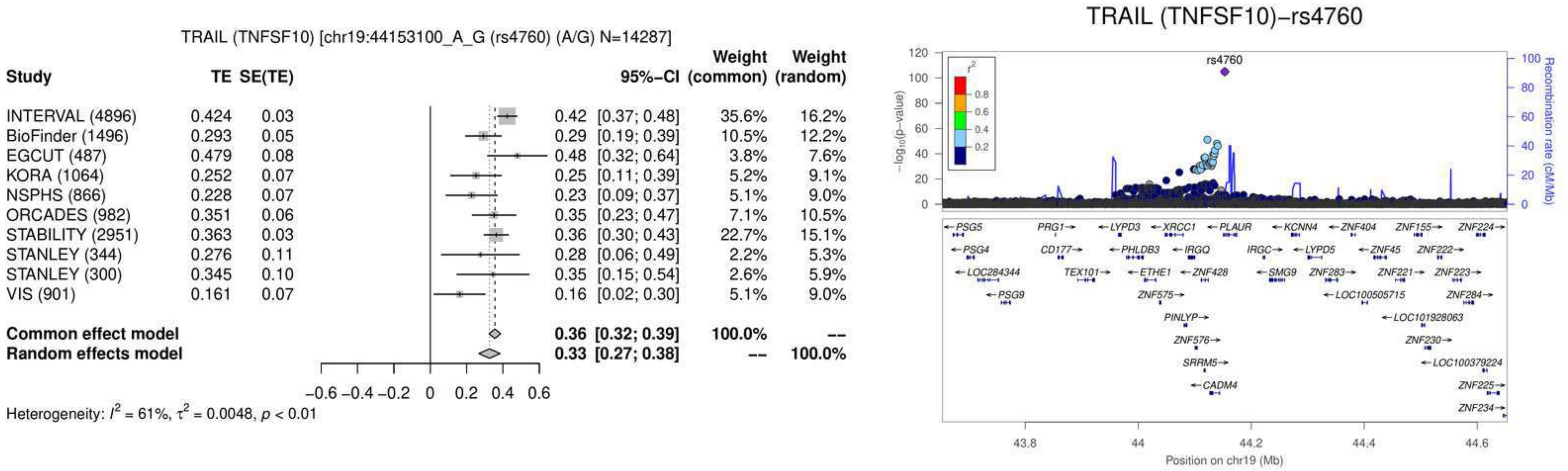
TRAIL (TNFSF10) [chr19:44153100_A_G (rs4760) (A/G) N=14287]
Study
TE
SE(TE)
95%-CI
Weight (common)
Weight (random)
INTERVAL (4896) 0.424 0.03 0.42 [0.37; 0.48] 35.6% 16.2%
BioFinder (1496) 0.293 0.05 0.29 [0.19; 0.39] 10.5% 12.2%
EGCUT (487) 0.479 0.08 0.48 [0.32; 0.64] 3.8% 7.6%
KORA (1064) 0.252 0.07 0.25 [0.11; 0.39] 5.2% 9.1%
NSPHS (866) 0.228 0.07 0.23 [0.09; 0.37] 5.1% 9.0%
ORCADES (982) 0.351 0.06 0.35 [0.23; 0.47] 7.1% 10.5%
STABILITY (2951) 0.363 0.03 0.36 [0.30; 0.43] 22.7% 15.1%
STANLEY (344) 0.276 0.11 0.28 [0.06; 0.49] 2.2% 5.3%
STANLEY (300) 0.345 0.10 0.35 [0.15; 0.54] 2.6% 5.9%
VIS (901) 0.161 0.07 0.16 [0.02; 0.30] 5.1% 9.0%
Common effect model 0.36 [0.32; 0.39] 100.0% --
Random effects model 0.33 [0.27; 0.38] -- 100.0%
−0.6 −0.4 −0.2 0 0.2 0.4 0.6
Heterogeneity: I^2 = 61%, τ^2 = 0.0048, p < 0.01
TRAIL (TNFSF10)−rs4760
rs4760
r^2
0.8
0.6
0.4
0.2
$-\log_{10}$(p-value)
Recombination rate (cM/Mb)
0 20 40 60 80 100 120
PSG5 PSG4 LOC284344 PSG9 PRG1 CD177 TEX101 LYPD3 PHLDB3 ETHE1 ZNF575 PINLYP ZNF576 SRRM5 CADM4 XRCC1 IRGQ ZNF428 PLAUR IRGC SMG9 KCNN4 LYPD5 ZNF283 LOC100505715 ZNF404 ZNF45 ZNF221 LOC101928063 ZNF230 LOC100379224 ZNF155 ZNF222 ZNF223 ZNF224 ZNF284 ZNF225 ZNF234
43.8 44 44.2 44.4 44.6
Position on chr19 (Mb)

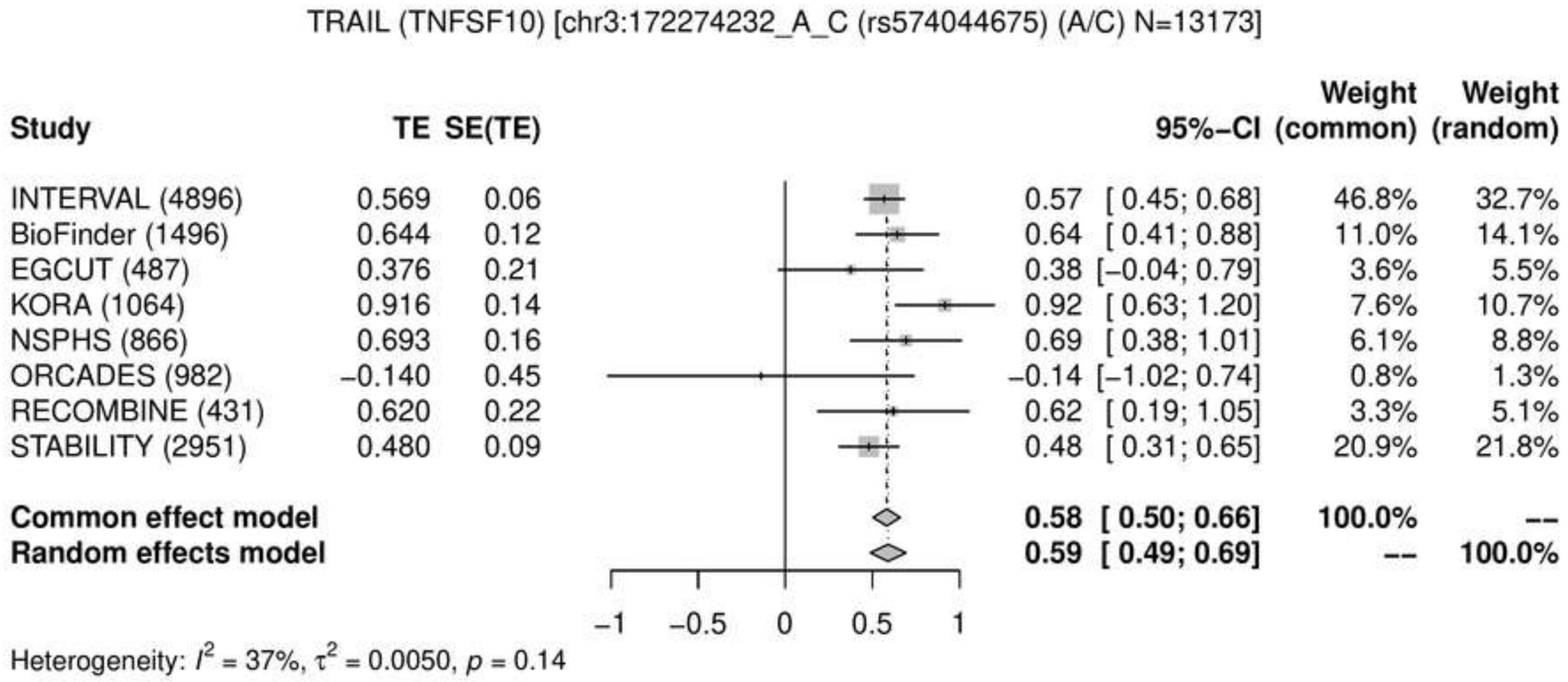
TRAIL (TNFSF10) [chr3:172274232_A_C (rs574044675) (A/C) N=13173]
Study
TE
SE(TE)
95%-CI
Weight (common)
Weight (random)
INTERVAL (4896) 0.569 0.06 0.57 [0.45; 0.68] 46.8% 32.7%
BioFinder (1496) 0.644 0.12 0.64 [0.41; 0.88] 11.0% 14.1%
EGCUT (487) 0.376 0.21 0.38 [−0.04; 0.79] 3.6% 5.5%
KORA (1064) 0.916 0.14 0.92 [0.63; 1.20] 7.6% 10.7%
NSPHS (866) 0.693 0.16 0.69 [0.38; 1.01] 6.1% 8.8%
ORCADES (982) −0.140 0.45 −0.14 [−1.02; 0.74] 0.8% 1.3%
RECOMBINE (431) 0.620 0.22 0.62 [0.19; 1.05] 3.3% 5.1%
STABILITY (2951) 0.480 0.09 0.48 [0.31; 0.65] 20.9% 21.8%
Common effect model 0.58 [0.50; 0.66] 100.0% --
Random effects model 0.59 [0.49; 0.69] -- 100.0%
−1
−0.5
0
0.5
1
Heterogeneity: I^2 = 37%, τ^2 = 0.0050, p = 0.14
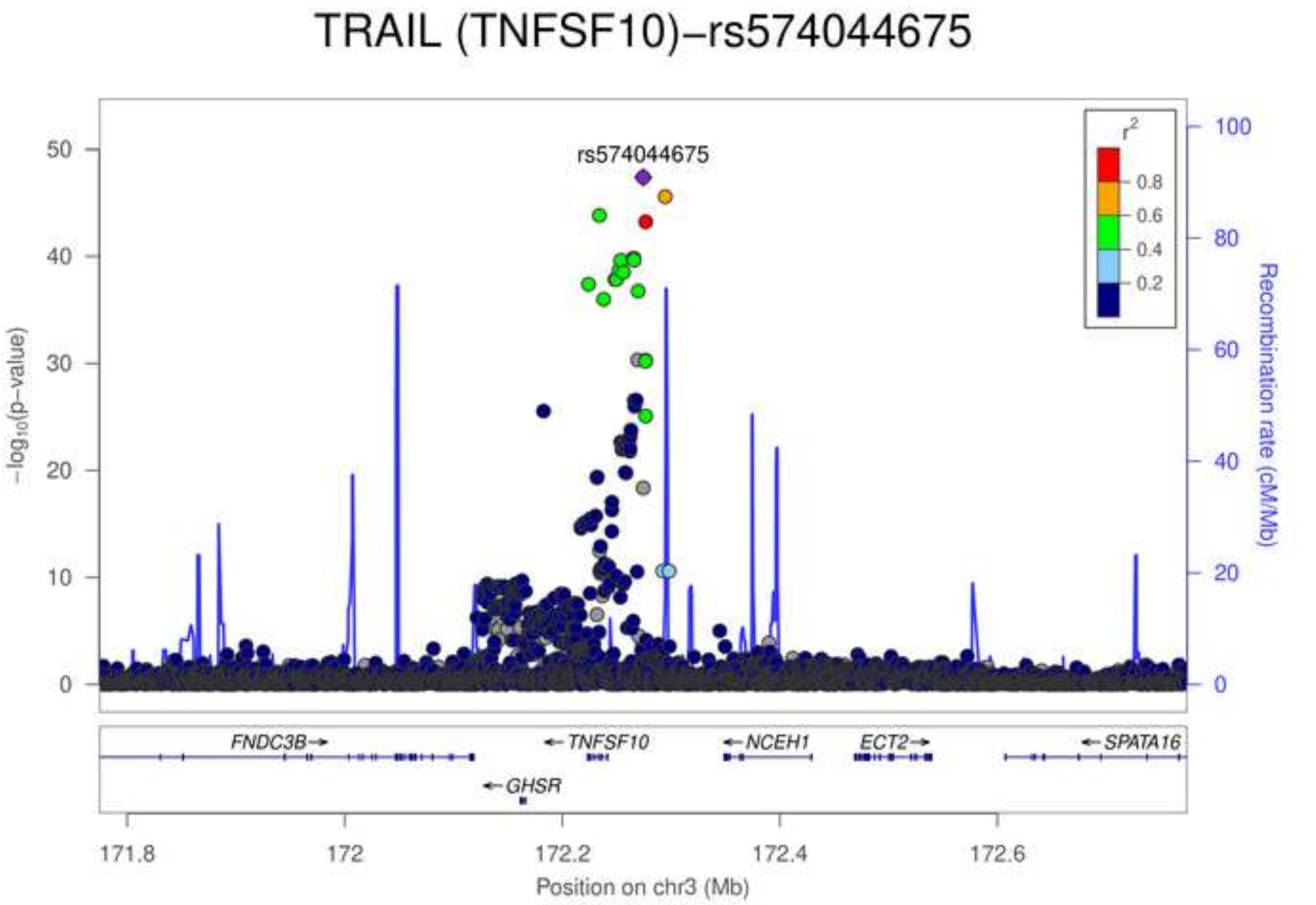
TRAIL (TNFSF10)−rs574044675
rs574044675
$-\log_{10}$(p−value)
Recombination rate (cM/Mb)
r^2
0.8
0.6
0.4
0.2
0
10
20
30
40
50
0
20
40
60
80
100
FNDC3B
TNFSF10
NCEH1
ECT2
SPATA16
GHSR
171.8
172
172.2
172.4
172.6
Position on chr3 (Mb)

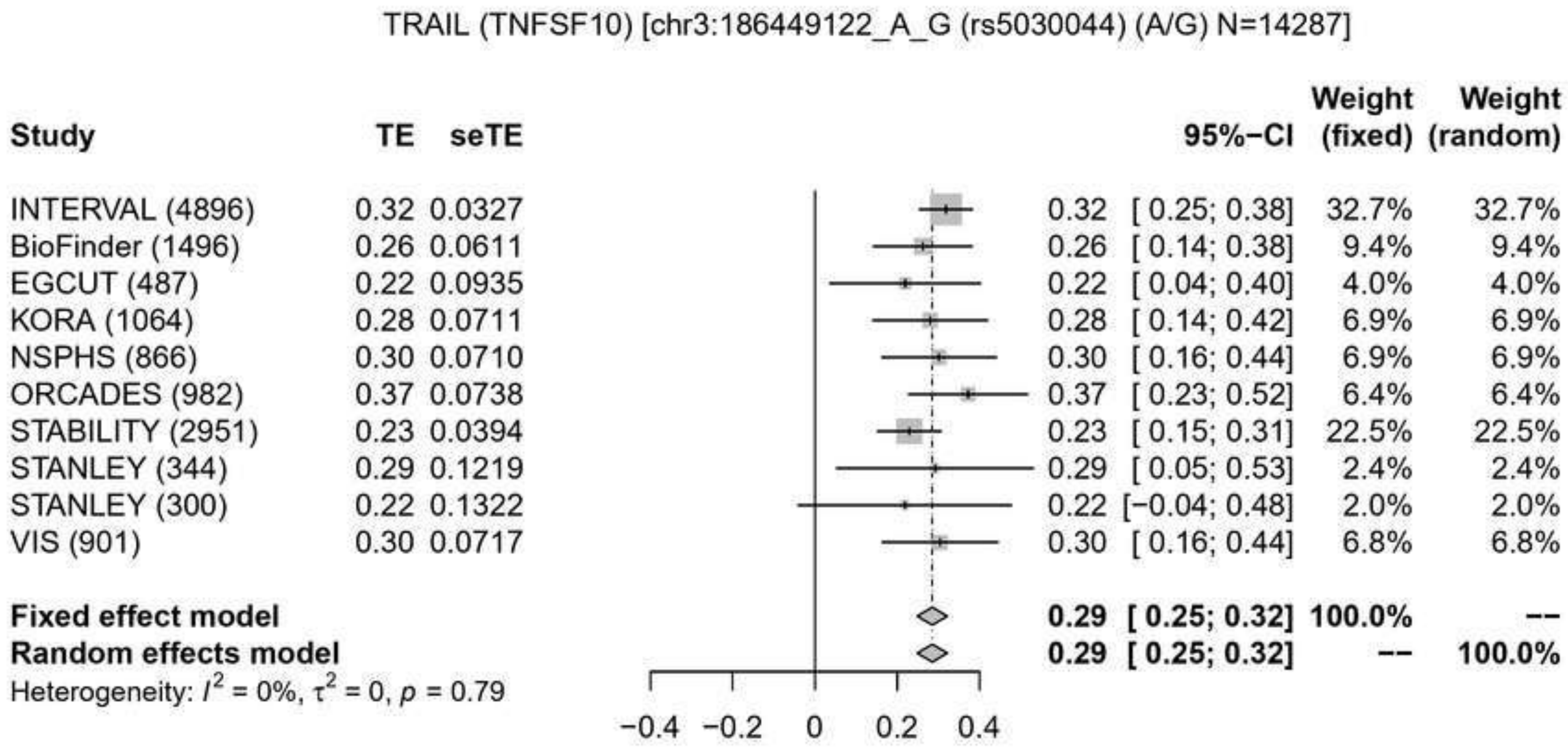
TRAIL (TNFSF10) [chr3:186449122_A_G (rs5030044) (A/G) N=14287]
Study TE seTE 95%−CI Weight (fixed) Weight (random)
INTERVAL (4896) 0.32 0.0327 0.32 [0.25; 0.38] 32.7% 32.7%
BioFinder (1496) 0.26 0.0611 0.26 [0.14; 0.38] 9.4% 9.4%
EGCUT (487) 0.22 0.0935 0.22 [0.04; 0.40] 4.0% 4.0%
KORA (1064) 0.28 0.0711 0.28 [0.14; 0.42] 6.9% 6.9%
NSPHS (866) 0.30 0.0710 0.30 [0.16; 0.44] 6.9% 6.9%
ORCADES (982) 0.37 0.0738 0.37 [0.23; 0.52] 6.4% 6.4%
STABILITY (2951) 0.23 0.0394 0.23 [0.15; 0.31] 22.5% 22.5%
STANLEY (344) 0.29 0.1219 0.29 [0.05; 0.53] 2.4% 2.4%
STANLEY (300) 0.22 0.1322 0.22 [−0.04; 0.48] 2.0% 2.0%
VIS (901) 0.30 0.0717 0.30 [0.16; 0.44] 6.8% 6.8%
Fixed effect model 0.29 [0.25; 0.32] 100.0% --
Random effects model 0.29 [0.25; 0.32] -- 100.0%
Heterogeneity: I^2 = 0%, τ^2 = 0, p = 0.79
−0.4 −0.2 0 0.2 0.4

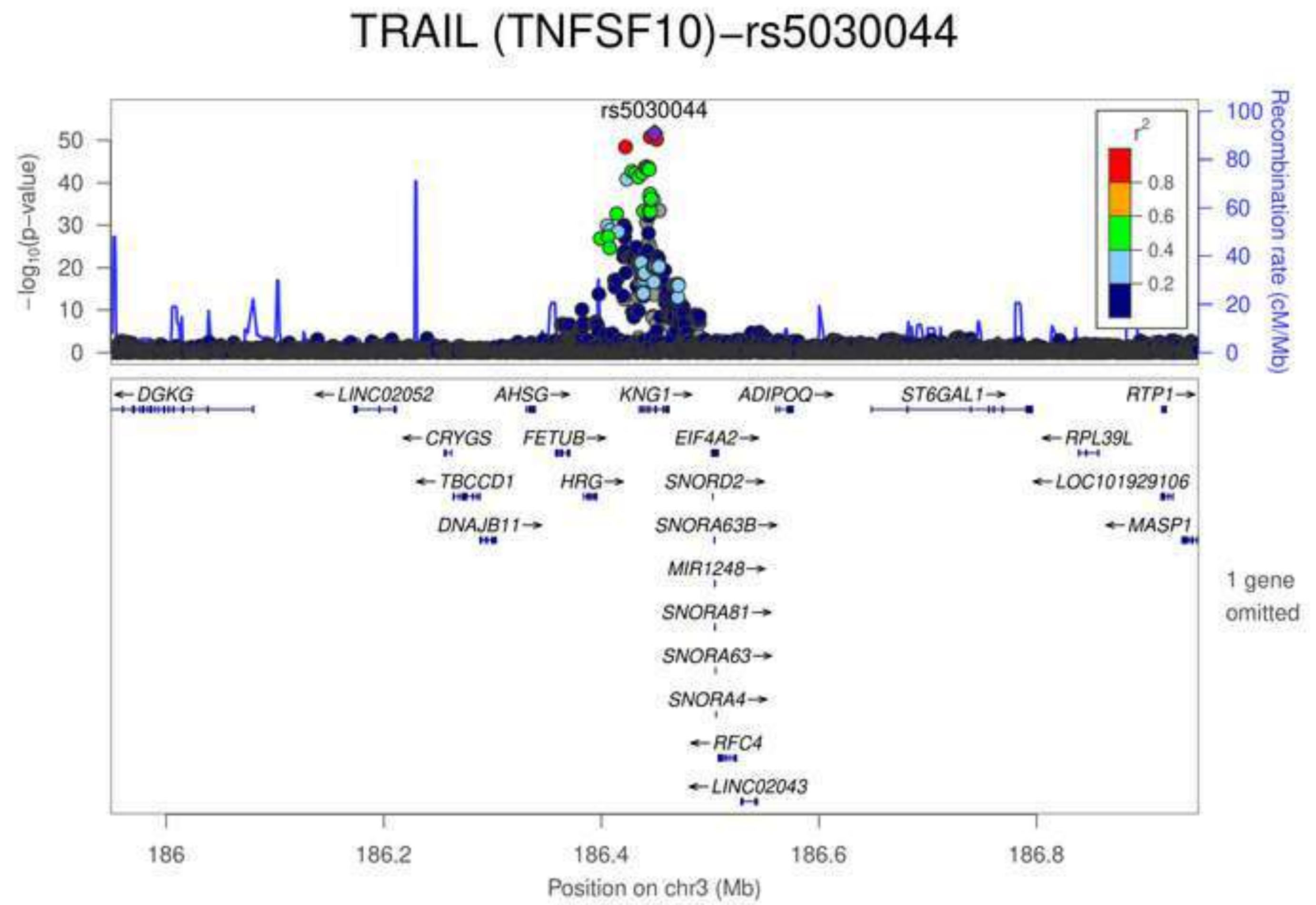
TRAIL (TNFSF10)−rs5030044
rs5030044
$-\log_{10}$(p−value)
Recombination rate (cM/Mb)
r^2
0.8
0.6
0.4
0.2
DGKG
LINC02052
AHSG
KNG1
ADIPOQ
ST6GAL1
RTP1
CRYGS
FETUB
EIF4A2
RPL39L
TBCCD1
HRG
SNORD2
LOC101929106
DNAJB11
SNORA63B
MASP1
MIR1248
SNORA81
SNORA63
SNORA4
RFC4
LINC02043
1 gene omitted
186 186.2 186.4 186.6 186.8
Position on chr3 (Mb)

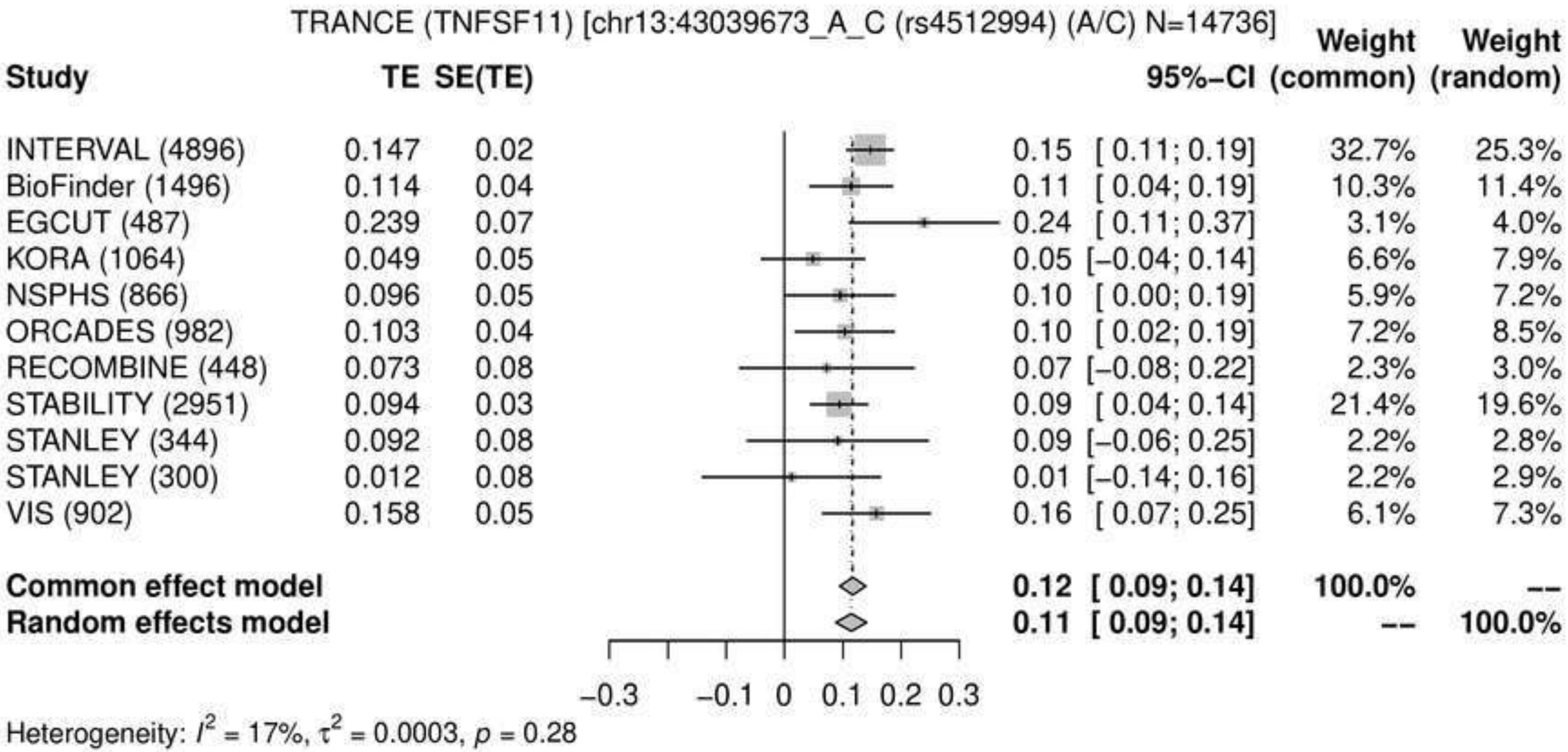
TRANCE (TNFSF11) [chr13:43039673_A_C (rs4512994) (A/C) N=14736]
Study
TE
SE(TE)
95%−CI
Weight (common)
Weight (random)
INTERVAL (4896) 0.147 0.02 0.15 [0.11; 0.19] 32.7% 25.3%
BioFinder (1496) 0.114 0.04 0.11 [0.04; 0.19] 10.3% 11.4%
EGCUT (487) 0.239 0.07 0.24 [0.11; 0.37] 3.1% 4.0%
KORA (1064) 0.049 0.05 0.05 [−0.04; 0.14] 6.6% 7.9%
NSPHS (866) 0.096 0.05 0.10 [0.00; 0.19] 5.9% 7.2%
ORCADES (982) 0.103 0.04 0.10 [0.02; 0.19] 7.2% 8.5%
RECOMBINE (448) 0.073 0.08 0.07 [−0.08; 0.22] 2.3% 3.0%
STABILITY (2951) 0.094 0.03 0.09 [0.04; 0.14] 21.4% 19.6%
STANLEY (344) 0.092 0.08 0.09 [−0.06; 0.25] 2.2% 2.8%
STANLEY (300) 0.012 0.08 0.01 [−0.14; 0.16] 2.2% 2.9%
VIS (902) 0.158 0.05 0.16 [0.07; 0.25] 6.1% 7.3%
Common effect model 0.12 [0.09; 0.14] 100.0% --
Random effects model 0.11 [0.09; 0.14] -- 100.0%
−0.3 −0.1 0 0.1 0.2 0.3
Heterogeneity: I^2 = 17%, τ^2 = 0.0003, p = 0.28

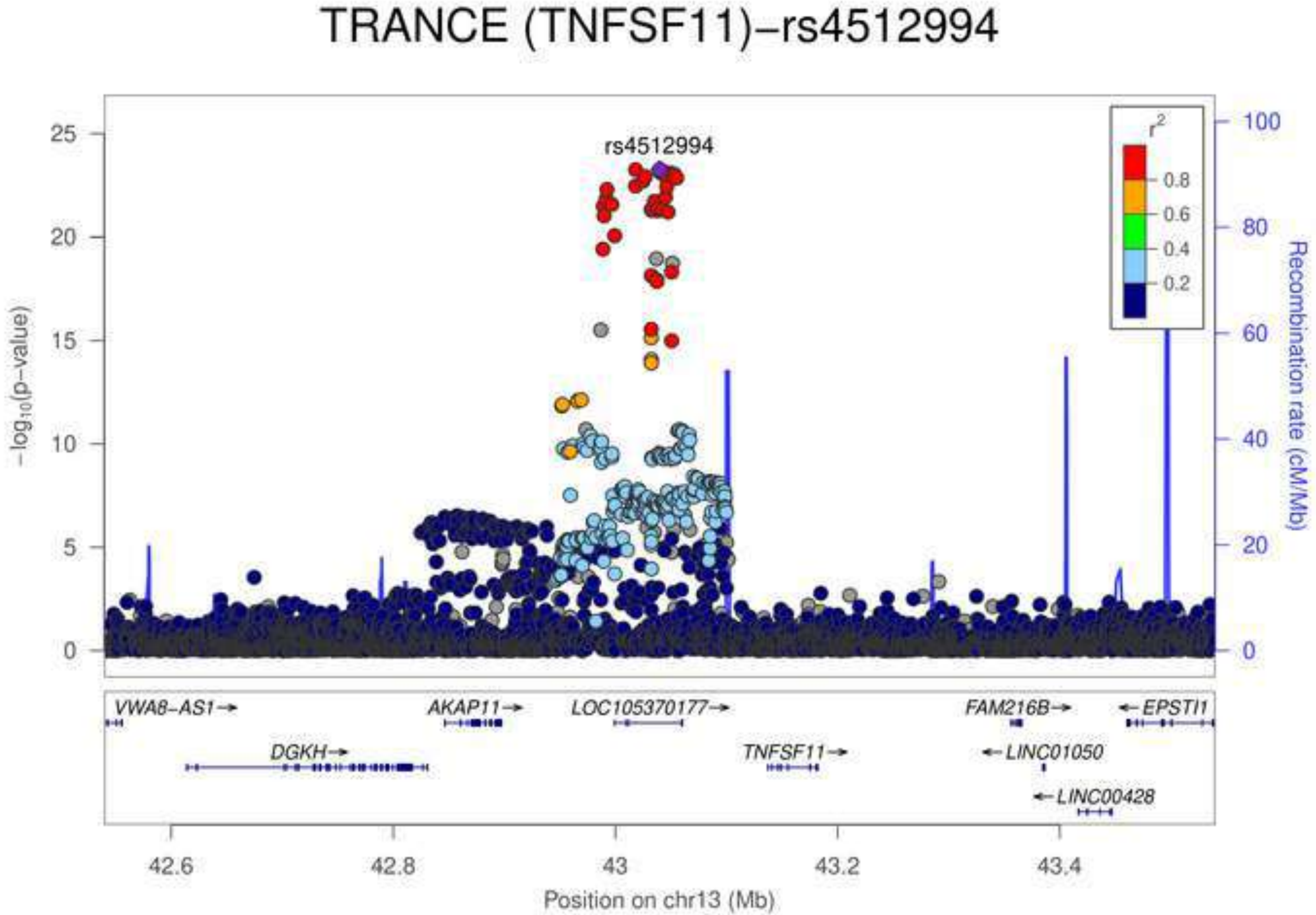
TRANCE (TNFSF11)−rs4512994
rs4512994
$-\log_{10}$(p−value)
Recombination rate (cM/Mb)
r^2
0.8
0.6
0.4
0.2
VWA8−AS1→
AKAP11→
LOC105370177→
FAM216B→
←EPSTI1
DGKH→
TNFSF11→
←LINC01050
←LINC00428
42.6
42.8
43
43.2
43.4
Position on chr13 (Mb)

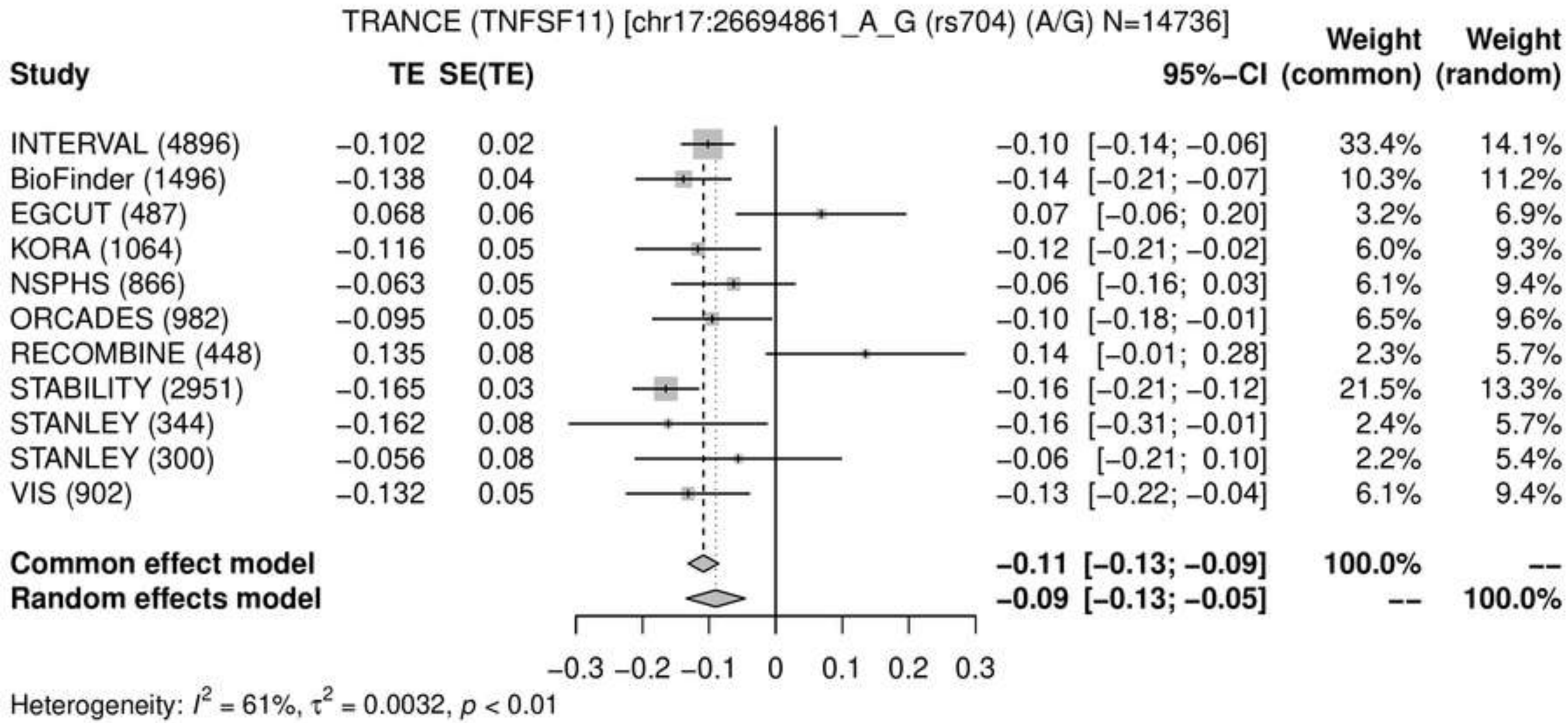
TRANCE (TNFSF11) [chr17:26694861_A_G (rs704) (A/G) N=14736]
Study TE SE(TE) 95%-CI Weight (common) Weight (random)
INTERVAL (4896) −0.102 0.02 −0.10 [−0.14; −0.06] 33.4% 14.1%
BioFinder (1496) −0.138 0.04 −0.14 [−0.21; −0.07] 10.3% 11.2%
EGCUT (487) 0.068 0.06 0.07 [−0.06; 0.20] 3.2% 6.9%
KORA (1064) −0.116 0.05 −0.12 [−0.21; −0.02] 6.0% 9.3%
NSPHS (866) −0.063 0.05 −0.06 [−0.16; 0.03] 6.1% 9.4%
ORCADES (982) −0.095 0.05 −0.10 [−0.18; −0.01] 6.5% 9.6%
RECOMBINE (448) 0.135 0.08 0.14 [−0.01; 0.28] 2.3% 5.7%
STABILITY (2951) −0.165 0.03 −0.16 [−0.21; −0.12] 21.5% 13.3%
STANLEY (344) −0.162 0.08 −0.16 [−0.31; −0.01] 2.4% 5.7%
STANLEY (300) −0.056 0.08 −0.06 [−0.21; 0.10] 2.2% 5.4%
VIS (902) −0.132 0.05 −0.13 [−0.22; −0.04] 6.1% 9.4%
Common effect model −0.11 [−0.13; −0.09] 100.0% --
Random effects model −0.09 [−0.13; −0.05] -- 100.0%
−0.3 −0.2 −0.1 0 0.1 0.2 0.3
Heterogeneity: I^2 = 61%, τ^2 = 0.0032, p < 0.01

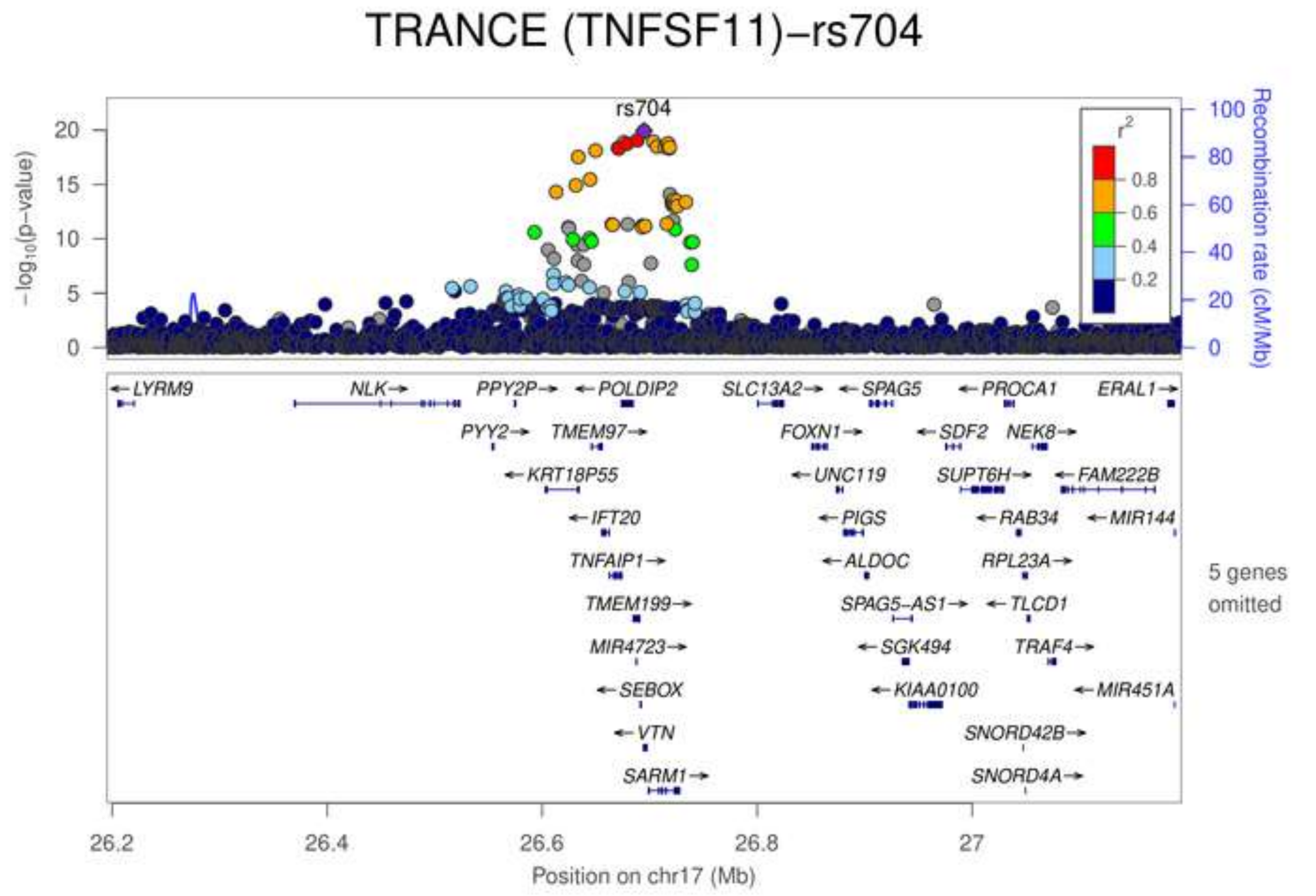
TRANCE (TNFSF11)−rs704
rs704
$-\log_{10}$(p-value)
Recombination rate (cM/Mb)
r^2
0.8
0.6
0.4
0.2
LYRM9
NLK
PPY2P
POLDIP2
SLC13A2
SPAG5
PROCA1
ERAL1
PYY2
TMEM97
FOXN1
SDF2
NEK8
KRT18P55
UNC119
SUPT6H
FAM222B
IFT20
PIGS
RAB34
MIR144
TNFAIP1
ALDOC
RPL23A
TMEM199
SPAG5-AS1
TLCD1
MIR4723
SGK494
TRAF4
SEBOX
KIAA0100
MIR451A
VTN
SNORD42B
SARM1
SNORD4A
5 genes omitted
26.2
26.4
26.6
26.8
27
Position on chr17 (Mb)

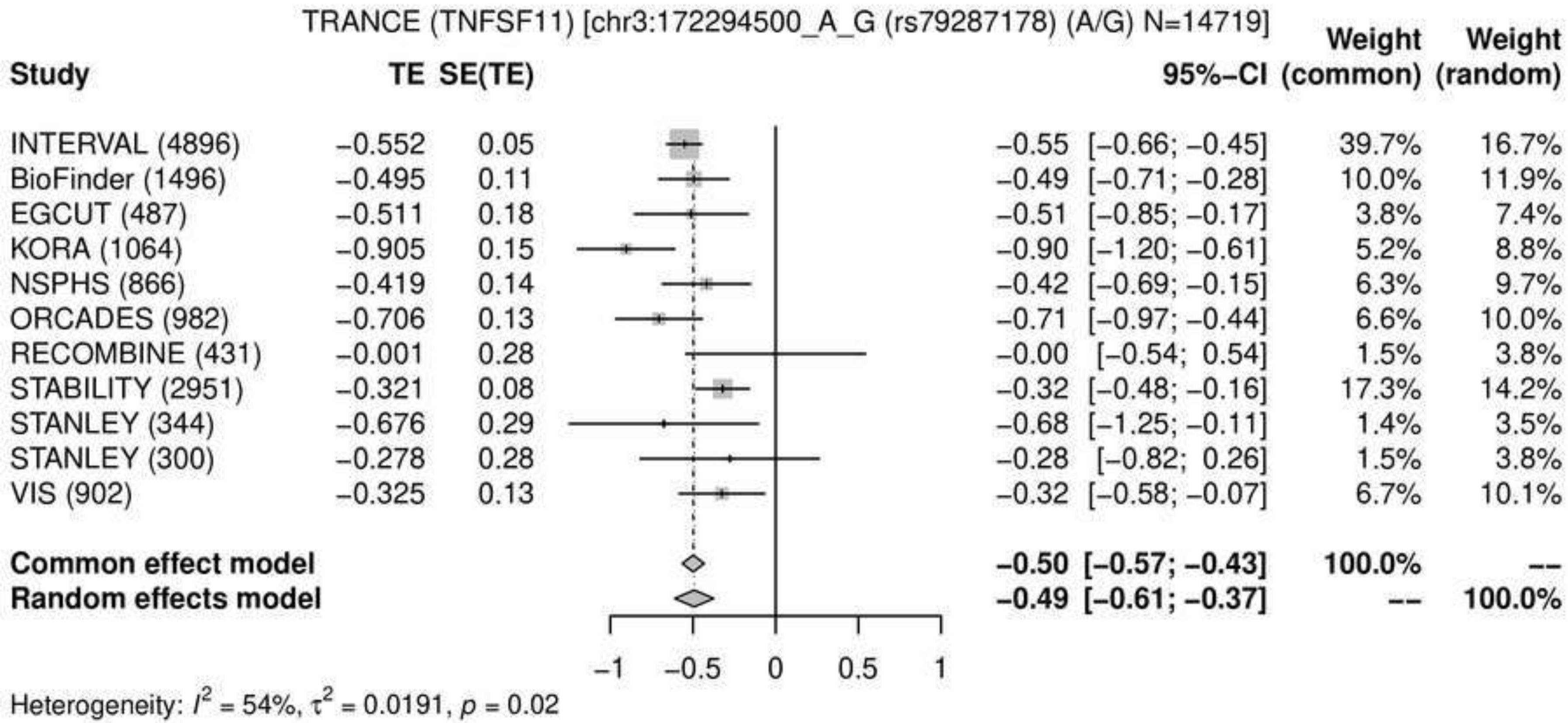

TRANCE (TNFSF11) [chr3:172294500_A_G (rs79287178) (A/G) N=14719]
Study
TE
SE(TE)
95%−CI
Weight (common)
Weight (random)
INTERVAL (4896) −0.552 0.05 −0.55 [−0.66; −0.45] 39.7% 16.7%
BioFinder (1496) −0.495 0.11 −0.49 [−0.71; −0.28] 10.0% 11.9%
EGCUT (487) −0.511 0.18 −0.51 [−0.85; −0.17] 3.8% 7.4%
KORA (1064) −0.905 0.15 −0.90 [−1.20; −0.61] 5.2% 8.8%
NSPHS (866) −0.419 0.14 −0.42 [−0.69; −0.15] 6.3% 9.7%
ORCADES (982) −0.706 0.13 −0.71 [−0.97; −0.44] 6.6% 10.0%
RECOMBINE (431) −0.001 0.28 −0.00 [−0.54; 0.54] 1.5% 3.8%
STABILITY (2951) −0.321 0.08 −0.32 [−0.48; −0.16] 17.3% 14.2%
STANLEY (344) −0.676 0.29 −0.68 [−1.25; −0.11] 1.4% 3.5%
STANLEY (300) −0.278 0.28 −0.28 [−0.82; 0.26] 1.5% 3.8%
VIS (902) −0.325 0.13 −0.32 [−0.58; −0.07] 6.7% 10.1%
Common effect model −0.50 [−0.57; −0.43] 100.0% --
Random effects model −0.49 [−0.61; −0.37] -- 100.0%
−1 −0.5 0 0.5 1
Heterogeneity: I^2 = 54%, τ^2 = 0.0191, p = 0.02

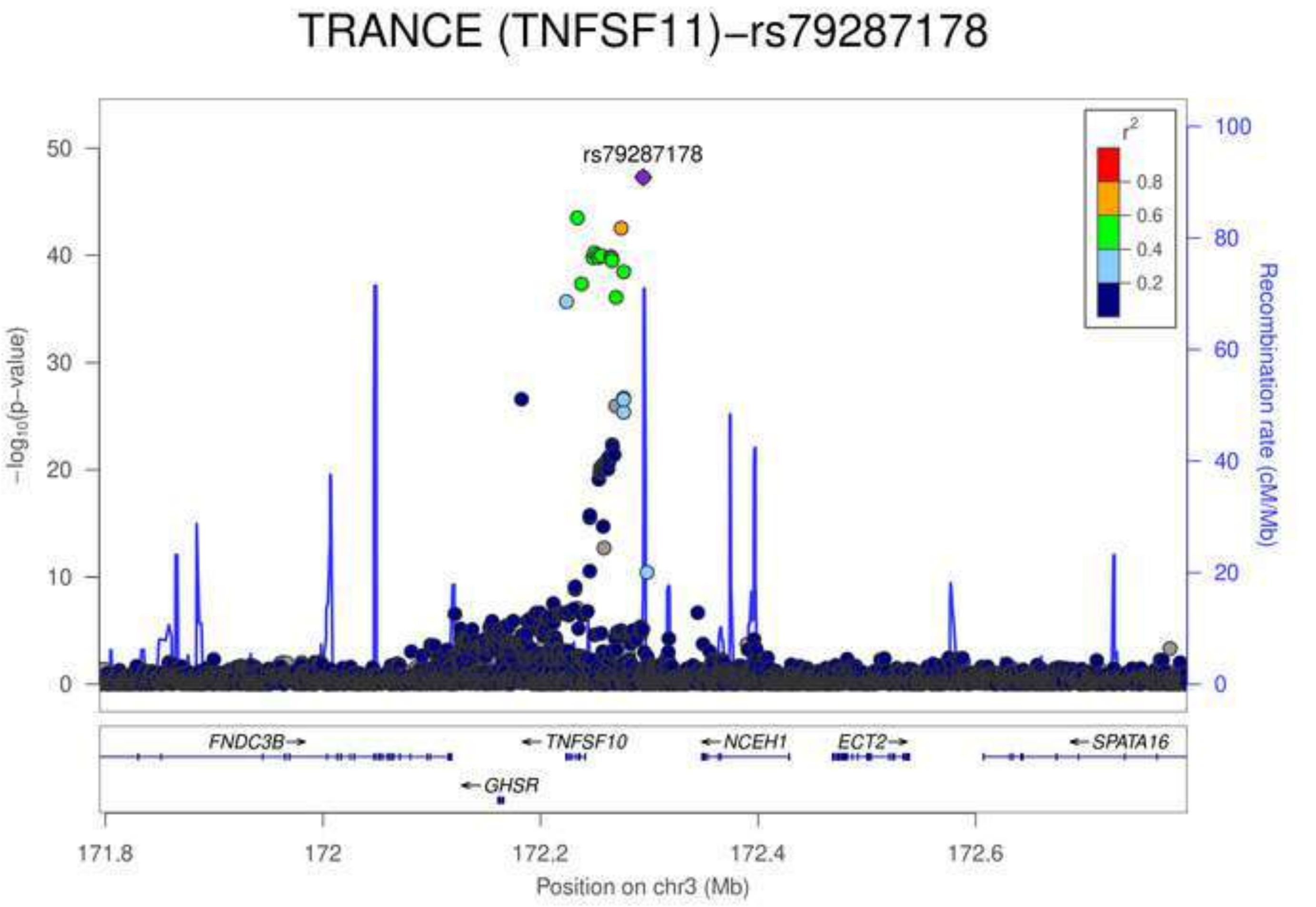

TRANCE (TNFSF11)−rs79287178
rs79287178
$-\log_{10}$(p-value)
Recombination rate (cM/Mb)
r^2
0.8
0.6
0.4
0.2
FNDC3B
TNFSF10
NCEH1
ECT2
SPATA16
GHSR
171.8
172
172.2
172.4
172.6
Position on chr3 (Mb)

TRANCE (TNFSF11) [chr3:194061578_A_G (rs11713634) (A/G) N=11337]

Study	TE	SE(TE)		95%-CI	Weight (common)	Weight (random)
INTERVAL (4896)	−0.119	0.02		−0.12 [−0.16; −0.08]	44.8%	27.5%
BioFinder (1496)	−0.115	0.04		−0.12 [−0.20; −0.03]	12.7%	14.6%
EGCUT (487)	−0.009	0.07		−0.01 [−0.15; 0.13]	4.6%	6.8%
KORA (1064)	−0.062	0.07		−0.06 [−0.20; 0.08]	4.5%	6.7%
NSPHS (866)	−0.068	0.05		−0.07 [−0.16; 0.03]	10.0%	12.4%
ORCADES (982)	−0.005	0.05		−0.01 [−0.11; 0.10]	8.5%	11.1%
STANLEY (344)	−0.045	0.09		−0.04 [−0.22; 0.13]	3.0%	4.7%
STANLEY (300)	−0.053	0.08		−0.05 [−0.22; 0.11]	3.1%	4.9%
VIS (902)	−0.187	0.05		−0.19 [−0.29; −0.09]	8.7%	11.2%
**Common effect model**				**−0.10 [−0.13; −0.07]**	**100.0%**	**--**
**Random effects model**				**−0.09 [−0.13; −0.05]**	**--**	**100.0%**

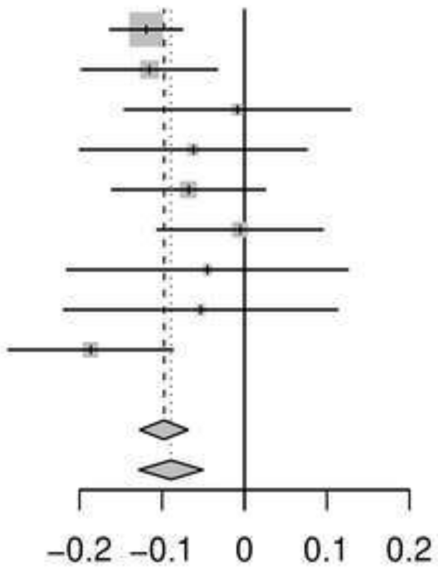

Heterogeneity: $I^2 = 22\%$, $\tau^2 = 0.0009$, $p = 0.24$

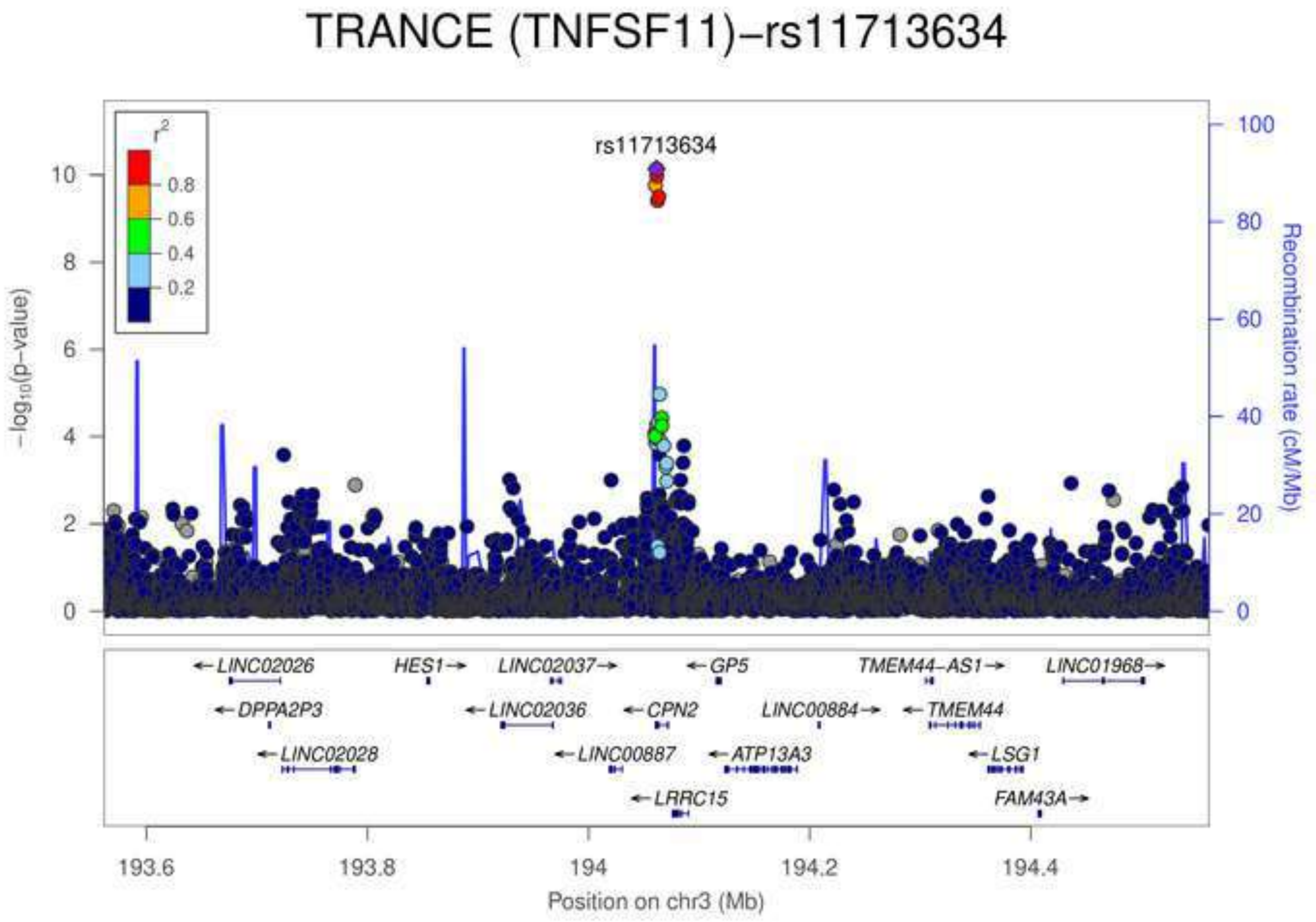

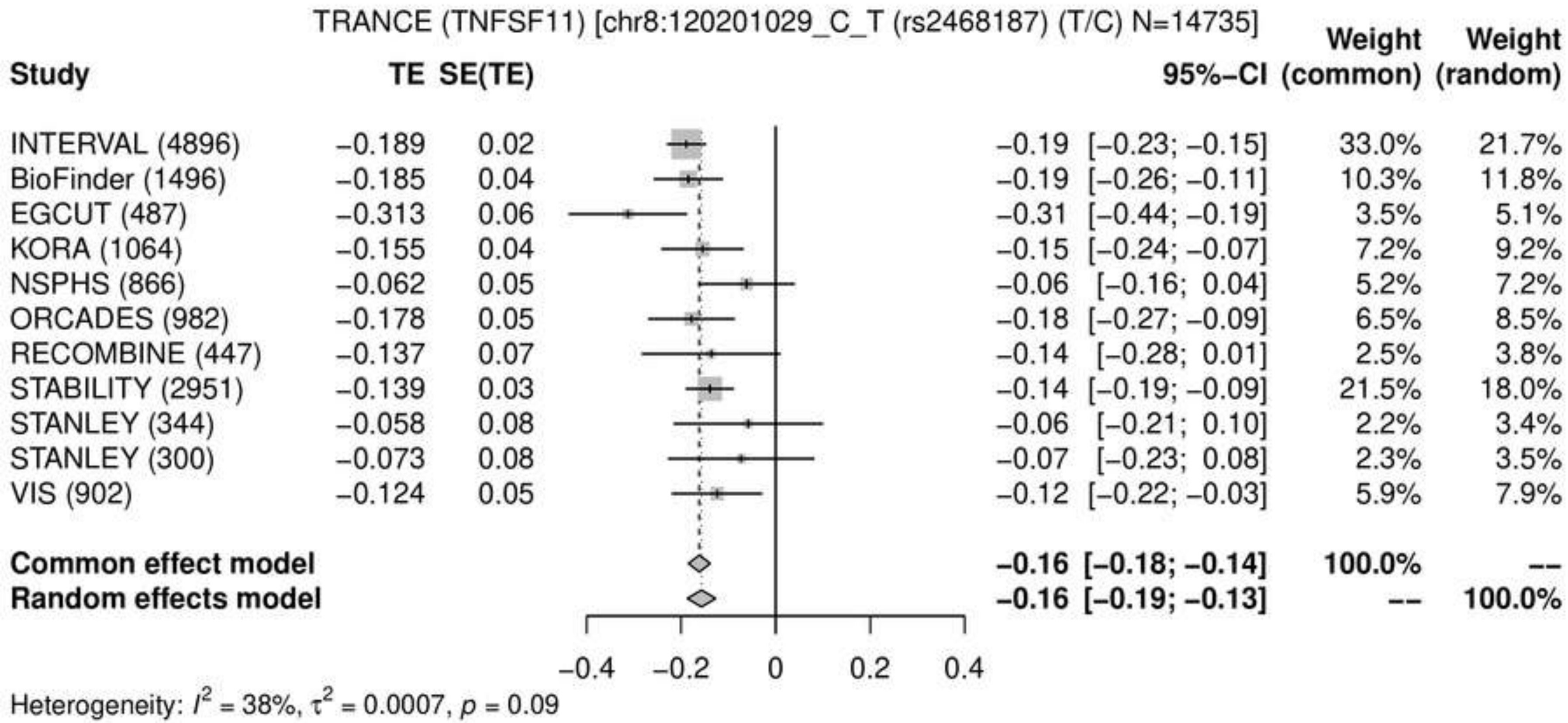
TRANCE (TNFSF11) [chr8:120201029_C_T (rs2468187) (T/C) N=14735]
Study
TE
SE(TE)
95%−CI
Weight (common)
Weight (random)
INTERVAL (4896) −0.189 0.02 −0.19 [−0.23; −0.15] 33.0% 21.7%
BioFinder (1496) −0.185 0.04 −0.19 [−0.26; −0.11] 10.3% 11.8%
EGCUT (487) −0.313 0.06 −0.31 [−0.44; −0.19] 3.5% 5.1%
KORA (1064) −0.155 0.04 −0.15 [−0.24; −0.07] 7.2% 9.2%
NSPHS (866) −0.062 0.05 −0.06 [−0.16; 0.04] 5.2% 7.2%
ORCADES (982) −0.178 0.05 −0.18 [−0.27; −0.09] 6.5% 8.5%
RECOMBINE (447) −0.137 0.07 −0.14 [−0.28; 0.01] 2.5% 3.8%
STABILITY (2951) −0.139 0.03 −0.14 [−0.19; −0.09] 21.5% 18.0%
STANLEY (344) −0.058 0.08 −0.06 [−0.21; 0.10] 2.2% 3.4%
STANLEY (300) −0.073 0.08 −0.07 [−0.23; 0.08] 2.3% 3.5%
VIS (902) −0.124 0.05 −0.12 [−0.22; −0.03] 5.9% 7.9%
Common effect model −0.16 [−0.18; −0.14] 100.0% --
Random effects model −0.16 [−0.19; −0.13] -- 100.0%
−0.4 −0.2 0 0.2 0.4
Heterogeneity: I^2 = 38%, τ^2 = 0.0007, p = 0.09

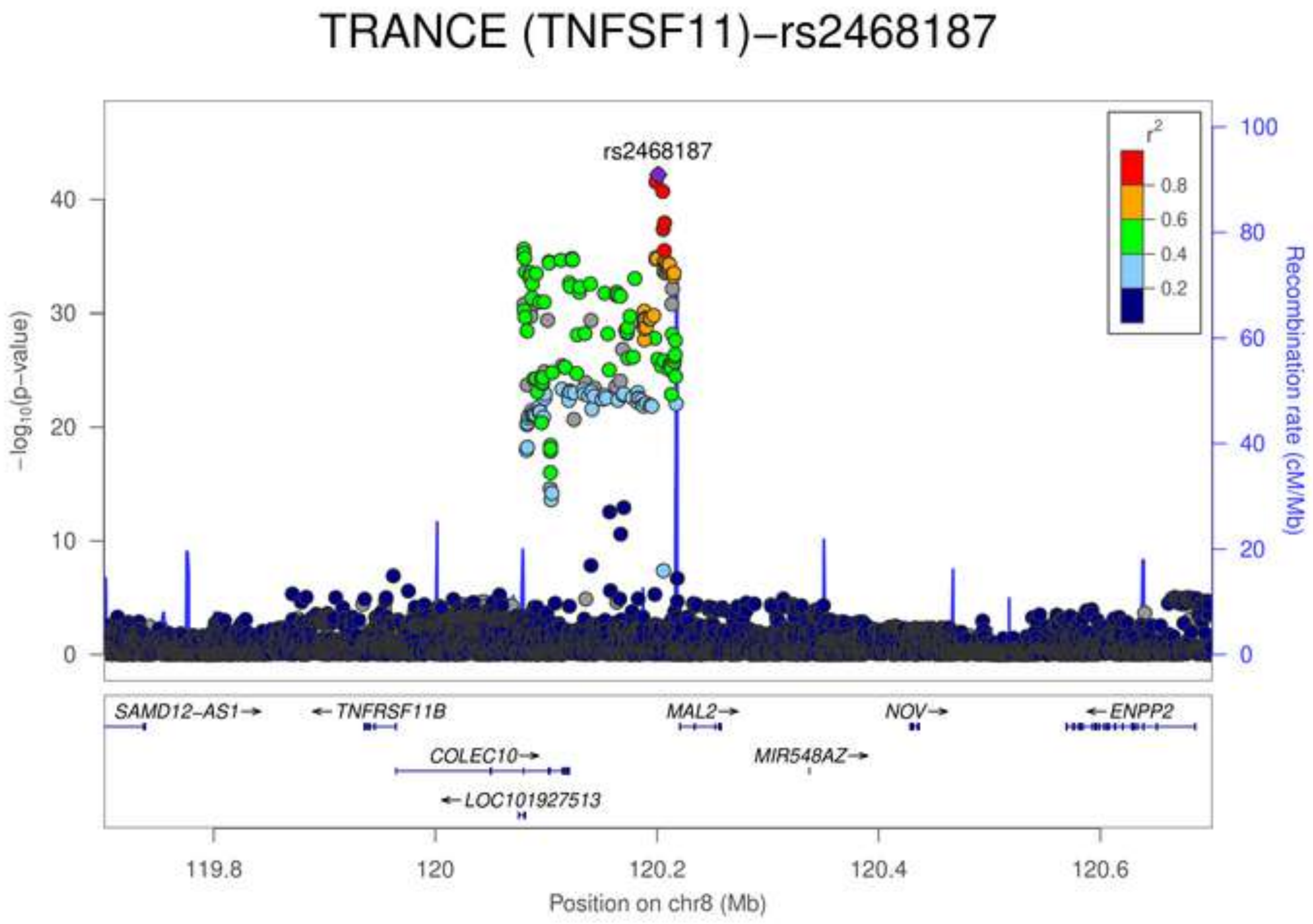
TRANCE (TNFSF11)−rs2468187
rs2468187
r^2
0.8
0.6
0.4
0.2
$-\log_{10}$(p-value)
Recombination rate (cM/Mb)
0
10
20
30
40
0
20
40
60
80
100
SAMD12−AS1→
←TNFRSF11B
MAL2→
NOV→
←ENPP2
COLEC10→
MIR548AZ→
←LOC101927513
119.8
120
120.2
120.4
120.6
Position on chr8 (Mb)

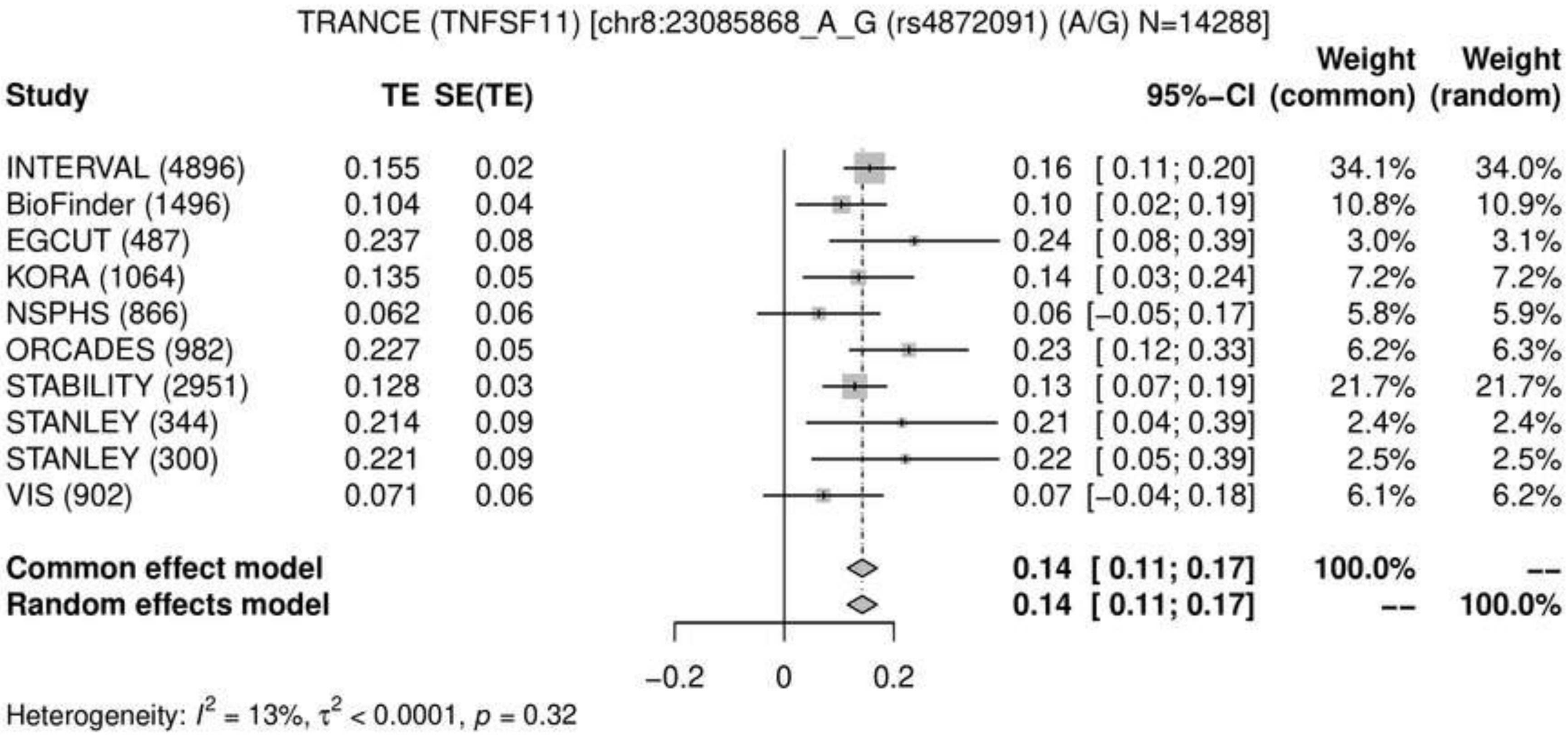
TRANCE (TNFSF11) [chr8:23085868_A_G (rs4872091) (A/G) N=14288]
Study TE SE(TE) 95%−CI Weight (common) Weight (random)
INTERVAL (4896) 0.155 0.02 0.16 [0.11; 0.20] 34.1% 34.0%
BioFinder (1496) 0.104 0.04 0.10 [0.02; 0.19] 10.8% 10.9%
EGCUT (487) 0.237 0.08 0.24 [0.08; 0.39] 3.0% 3.1%
KORA (1064) 0.135 0.05 0.14 [0.03; 0.24] 7.2% 7.2%
NSPHS (866) 0.062 0.06 0.06 [−0.05; 0.17] 5.8% 5.9%
ORCADES (982) 0.227 0.05 0.23 [0.12; 0.33] 6.2% 6.3%
STABILITY (2951) 0.128 0.03 0.13 [0.07; 0.19] 21.7% 21.7%
STANLEY (344) 0.214 0.09 0.21 [0.04; 0.39] 2.4% 2.4%
STANLEY (300) 0.221 0.09 0.22 [0.05; 0.39] 2.5% 2.5%
VIS (902) 0.071 0.06 0.07 [−0.04; 0.18] 6.1% 6.2%
Common effect model 0.14 [0.11; 0.17] 100.0% --
Random effects model 0.14 [0.11; 0.17] -- 100.0%
−0.2 0 0.2
Heterogeneity: I^2 = 13%, τ^2 < 0.0001, p = 0.32

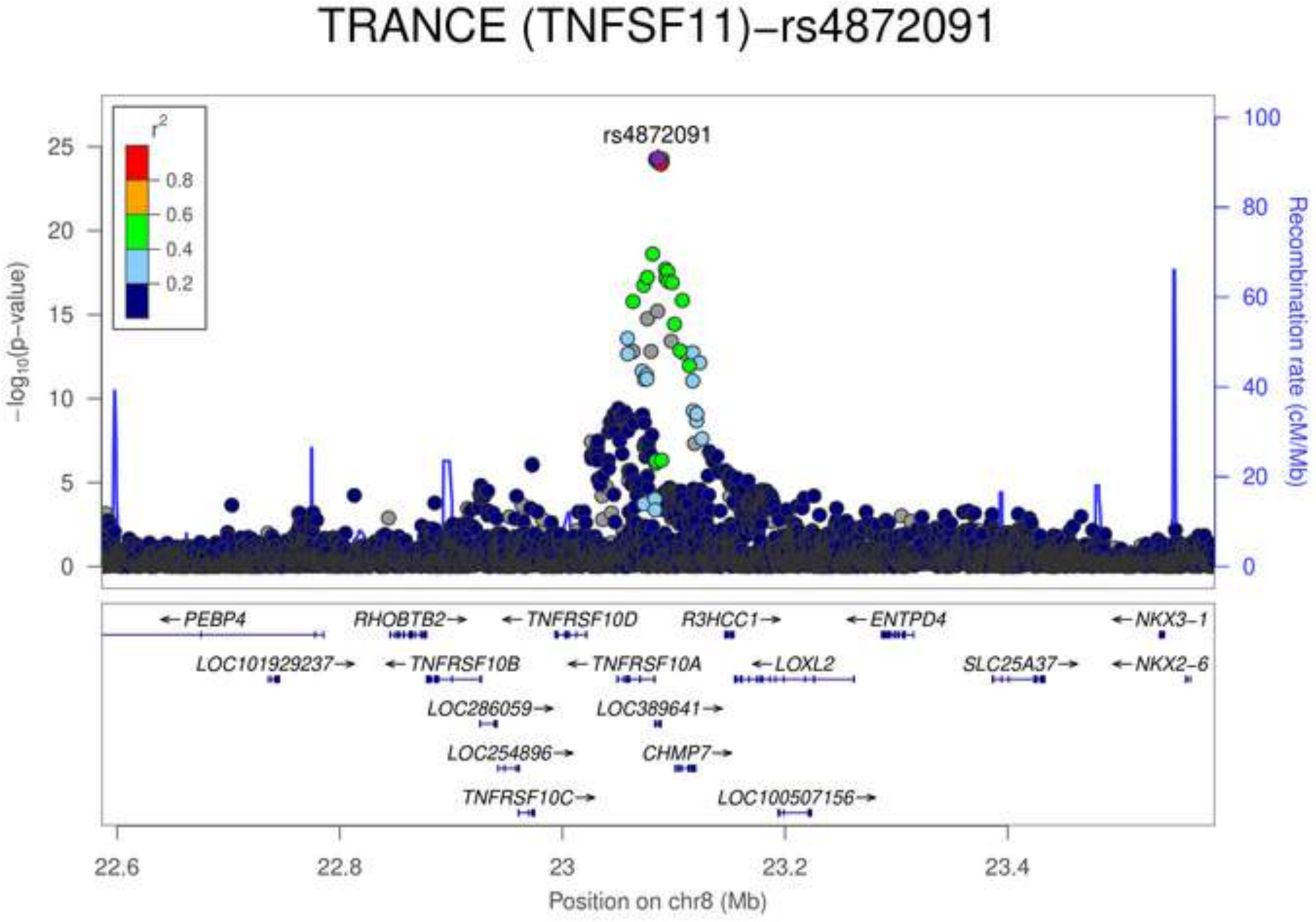
TRANCE (TNFSF11)−rs4872091
rs4872091
r^2
0.8
0.6
0.4
0.2
−log10(p−value)
Recombination rate (cM/Mb)
PEBP4
RHOBTB2
TNFRSF10D
R3HCC1
ENTPD4
NKX3−1
LOC101929237
TNFRSF10B
TNFRSF10A
LOXL2
SLC25A37
NKX2−6
LOC286059
LOC389641
LOC254896
CHMP7
TNFRSF10C
LOC100507156
22.6 22.8 23 23.2 23.4
Position on chr8 (Mb)

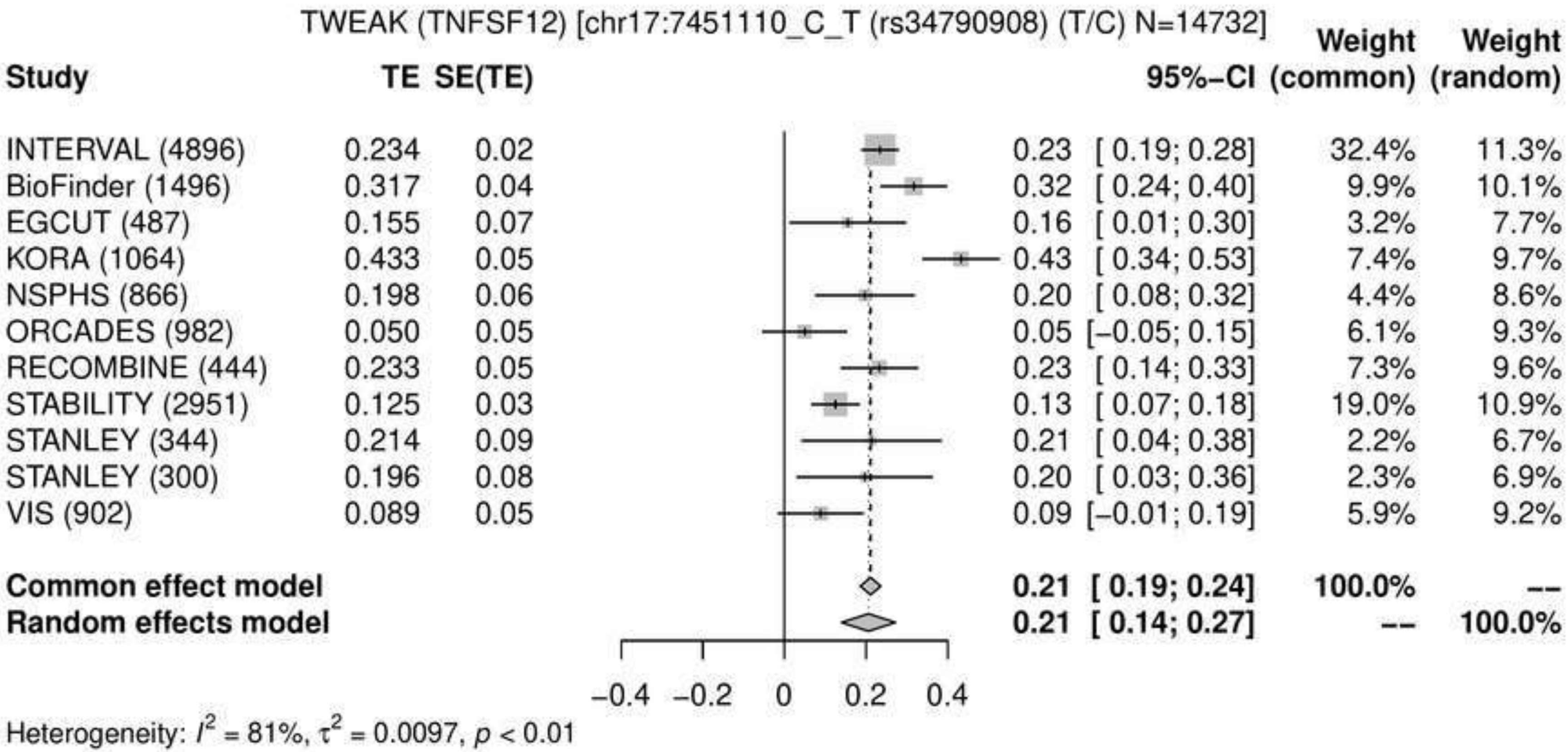
TWEAK (TNFSF12) [chr17:7451110_C_T (rs34790908) (T/C) N=14732]
Study TE SE(TE) 95%−CI Weight (common) Weight (random)
INTERVAL (4896) 0.234 0.02 0.23 [0.19; 0.28] 32.4% 11.3%
BioFinder (1496) 0.317 0.04 0.32 [0.24; 0.40] 9.9% 10.1%
EGCUT (487) 0.155 0.07 0.16 [0.01; 0.30] 3.2% 7.7%
KORA (1064) 0.433 0.05 0.43 [0.34; 0.53] 7.4% 9.7%
NSPHS (866) 0.198 0.06 0.20 [0.08; 0.32] 4.4% 8.6%
ORCADES (982) 0.050 0.05 0.05 [−0.05; 0.15] 6.1% 9.3%
RECOMBINE (444) 0.233 0.05 0.23 [0.14; 0.33] 7.3% 9.6%
STABILITY (2951) 0.125 0.03 0.13 [0.07; 0.18] 19.0% 10.9%
STANLEY (344) 0.214 0.09 0.21 [0.04; 0.38] 2.2% 6.7%
STANLEY (300) 0.196 0.08 0.20 [0.03; 0.36] 2.3% 6.9%
VIS (902) 0.089 0.05 0.09 [−0.01; 0.19] 5.9% 9.2%
Common effect model 0.21 [0.19; 0.24] 100.0% --
Random effects model 0.21 [0.14; 0.27] -- 100.0%
−0.4 −0.2 0 0.2 0.4
Heterogeneity: I^2 = 81%, τ^2 = 0.0097, p < 0.01

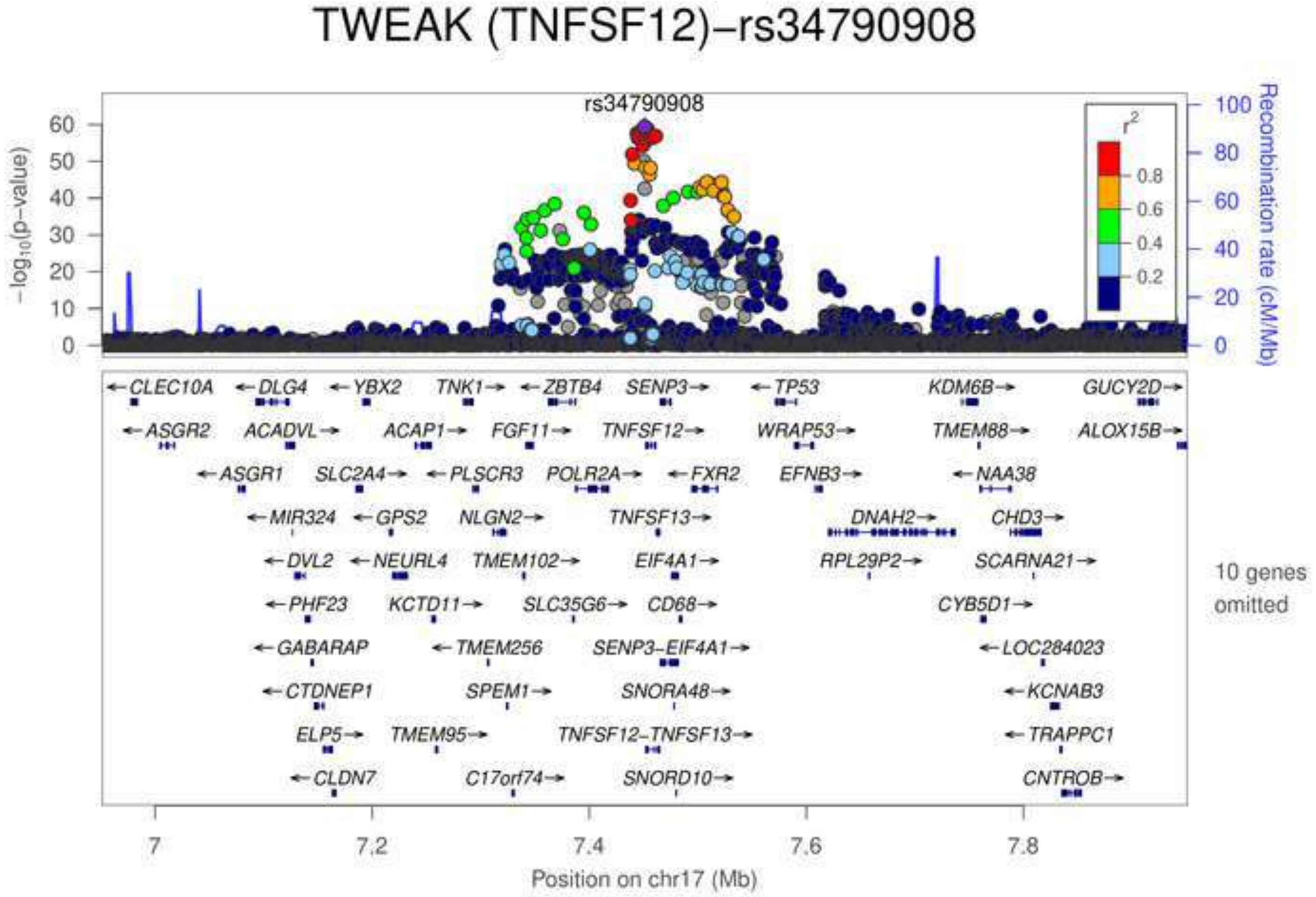
TWEAK (TNFSF12)−rs34790908
rs34790908
$-\log_{10}$(p−value)
Recombination rate (cM/Mb)
r^2
0.8
0.6
0.4
0.2
CLEC10A DLG4 YBX2 TNK1 ZBTB4 SENP3 TP53 KDM6B GUCY2D
ASGR2 ACADVL ACAP1 FGF11 TNFSF12 WRAP53 TMEM88 ALOX15B
ASGR1 SLC2A4 PLSCR3 POLR2A FXR2 EFNB3 NAA38
MIR324 GPS2 NLGN2 TNFSF13 DNAH2 CHD3
DVL2 NEURL4 TMEM102 EIF4A1 RPL29P2 SCARNA21
PHF23 KCTD11 SLC35G6 CD68 CYB5D1
GABARAP TMEM256 SENP3−EIF4A1 LOC284023
CTDNEP1 SPEM1 SNORA48 KCNAB3
ELP5 TMEM95 TNFSF12−TNFSF13 TRAPPC1
CLDN7 C17orf74 SNORD10 CNTROB
10 genes omitted
Position on chr17 (Mb)

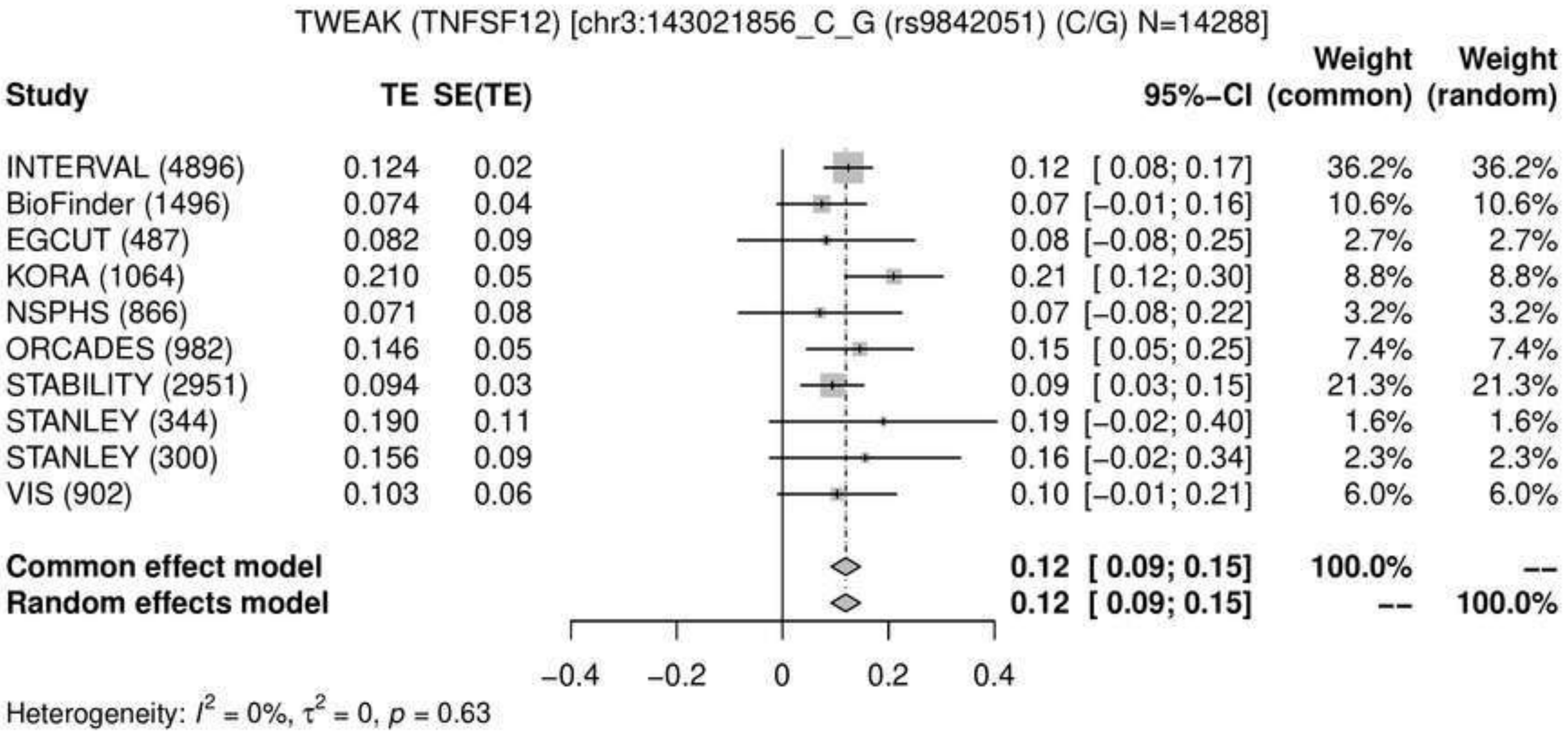
TWEAK (TNFSF12) [chr3:143021856_C_G (rs9842051) (C/G) N=14288]
Study TE SE(TE) 95%−CI Weight (common) Weight (random)
INTERVAL (4896) 0.124 0.02 0.12 [0.08; 0.17] 36.2% 36.2%
BioFinder (1496) 0.074 0.04 0.07 [−0.01; 0.16] 10.6% 10.6%
EGCUT (487) 0.082 0.09 0.08 [−0.08; 0.25] 2.7% 2.7%
KORA (1064) 0.210 0.05 0.21 [0.12; 0.30] 8.8% 8.8%
NSPHS (866) 0.071 0.08 0.07 [−0.08; 0.22] 3.2% 3.2%
ORCADES (982) 0.146 0.05 0.15 [0.05; 0.25] 7.4% 7.4%
STABILITY (2951) 0.094 0.03 0.09 [0.03; 0.15] 21.3% 21.3%
STANLEY (344) 0.190 0.11 0.19 [−0.02; 0.40] 1.6% 1.6%
STANLEY (300) 0.156 0.09 0.16 [−0.02; 0.34] 2.3% 2.3%
VIS (902) 0.103 0.06 0.10 [−0.01; 0.21] 6.0% 6.0%
Common effect model 0.12 [0.09; 0.15] 100.0% --
Random effects model 0.12 [0.09; 0.15] -- 100.0%
−0.4 −0.2 0 0.2 0.4
Heterogeneity: I^2 = 0%, τ^2 = 0, p = 0.63

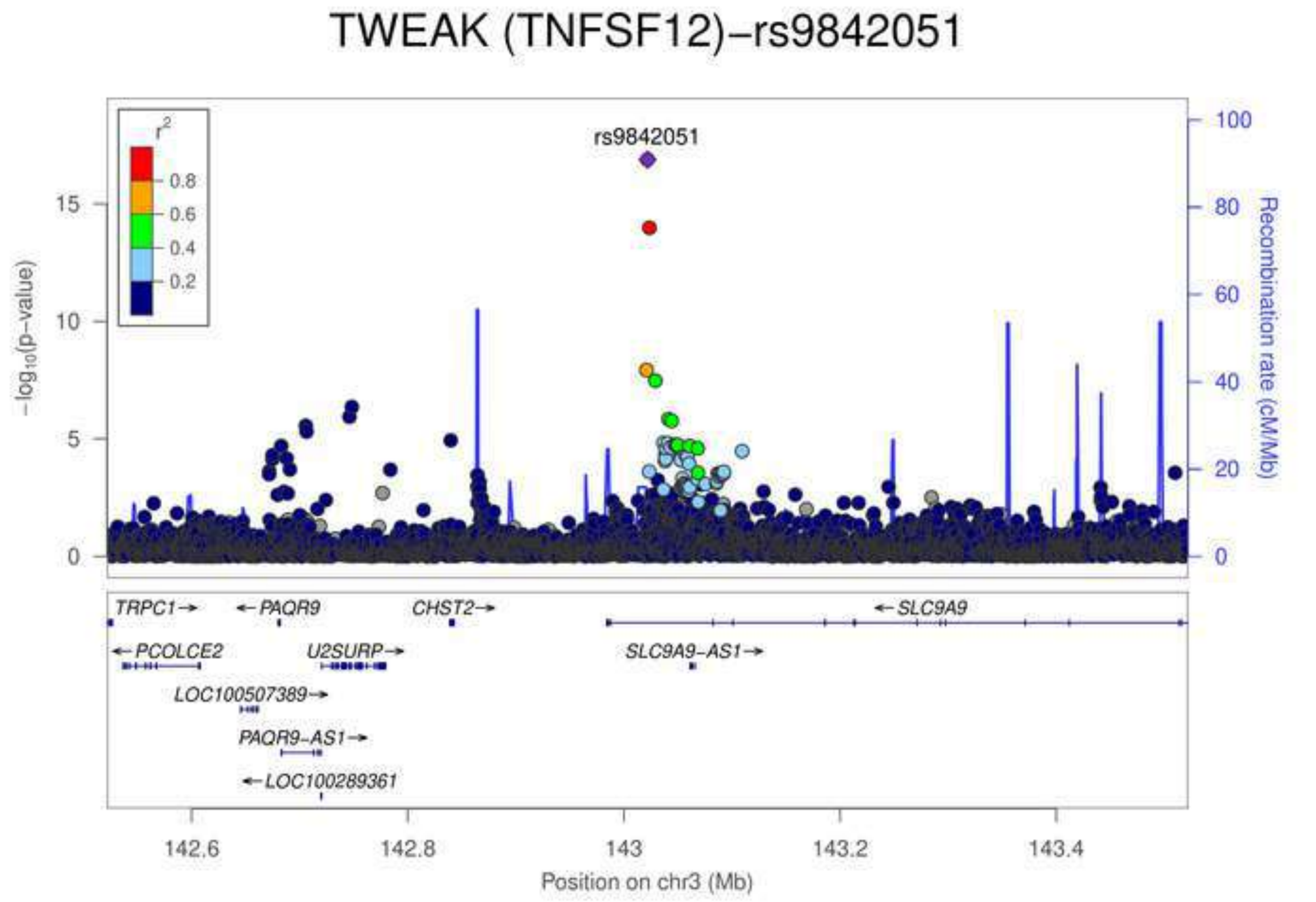
TWEAK (TNFSF12)−rs9842051
rs9842051
r^2
0.8
0.6
0.4
0.2
$-\log_{10}$(p-value)
Recombination rate (cM/Mb)
TRPC1→
←PAQR9
CHST2→
←SLC9A9
←PCOLCE2
U2SURP→
SLC9A9−AS1→
LOC100507389→
PAQR9−AS1→
←LOC100289361
142.6 142.8 143 143.2 143.4
Position on chr3 (Mb)

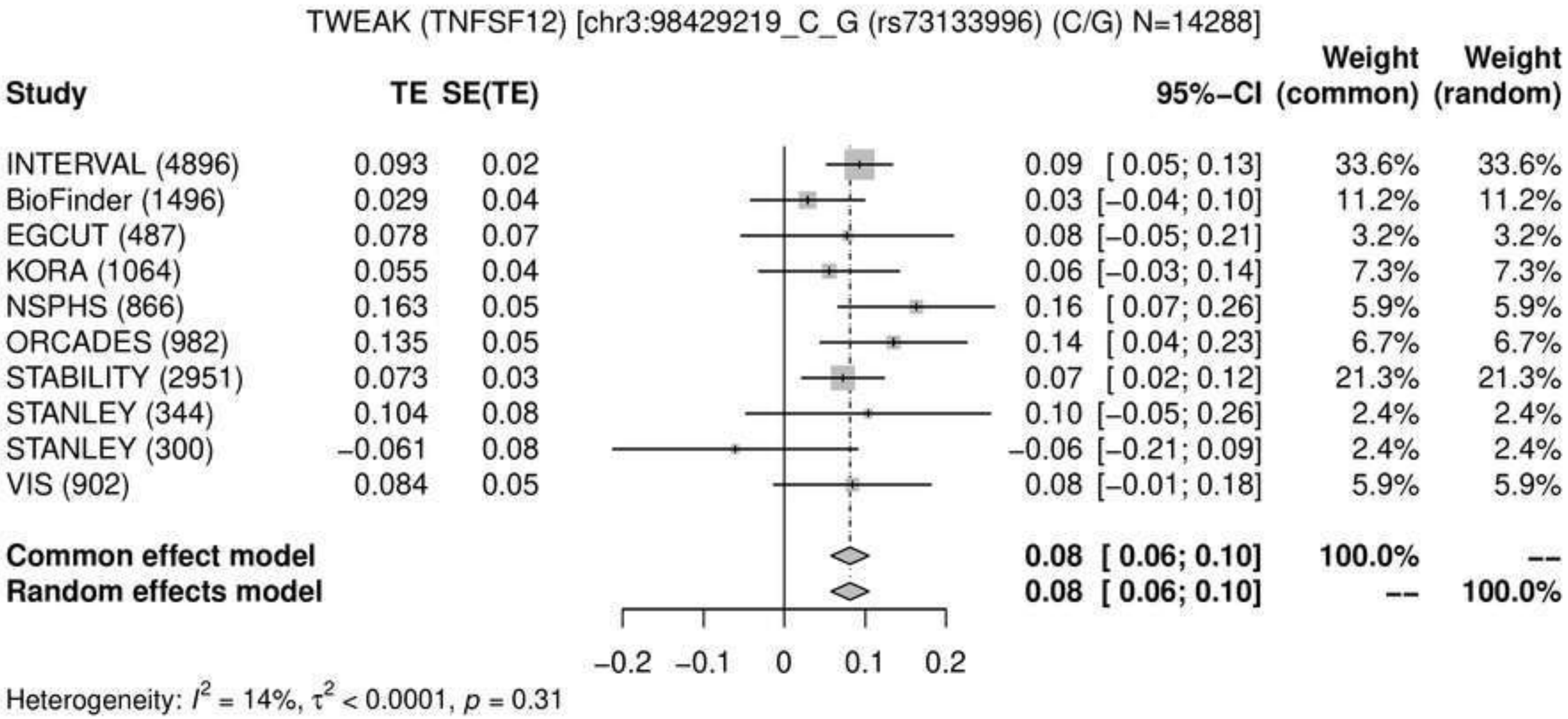
TWEAK (TNFSF12) [chr3:98429219_C_G (rs73133996) (C/G) N=14288]
Study
TE
SE(TE)
95%−CI
Weight (common)
Weight (random)
INTERVAL (4896) 0.093 0.02 0.09 [0.05; 0.13] 33.6% 33.6%
BioFinder (1496) 0.029 0.04 0.03 [−0.04; 0.10] 11.2% 11.2%
EGCUT (487) 0.078 0.07 0.08 [−0.05; 0.21] 3.2% 3.2%
KORA (1064) 0.055 0.04 0.06 [−0.03; 0.14] 7.3% 7.3%
NSPHS (866) 0.163 0.05 0.16 [0.07; 0.26] 5.9% 5.9%
ORCADES (982) 0.135 0.05 0.14 [0.04; 0.23] 6.7% 6.7%
STABILITY (2951) 0.073 0.03 0.07 [0.02; 0.12] 21.3% 21.3%
STANLEY (344) 0.104 0.08 0.10 [−0.05; 0.26] 2.4% 2.4%
STANLEY (300) −0.061 0.08 −0.06 [−0.21; 0.09] 2.4% 2.4%
VIS (902) 0.084 0.05 0.08 [−0.01; 0.18] 5.9% 5.9%
Common effect model 0.08 [0.06; 0.10] 100.0% --
Random effects model 0.08 [0.06; 0.10] -- 100.0%
−0.2 −0.1 0 0.1 0.2
Heterogeneity: I^2 = 14%, τ^2 < 0.0001, p = 0.31
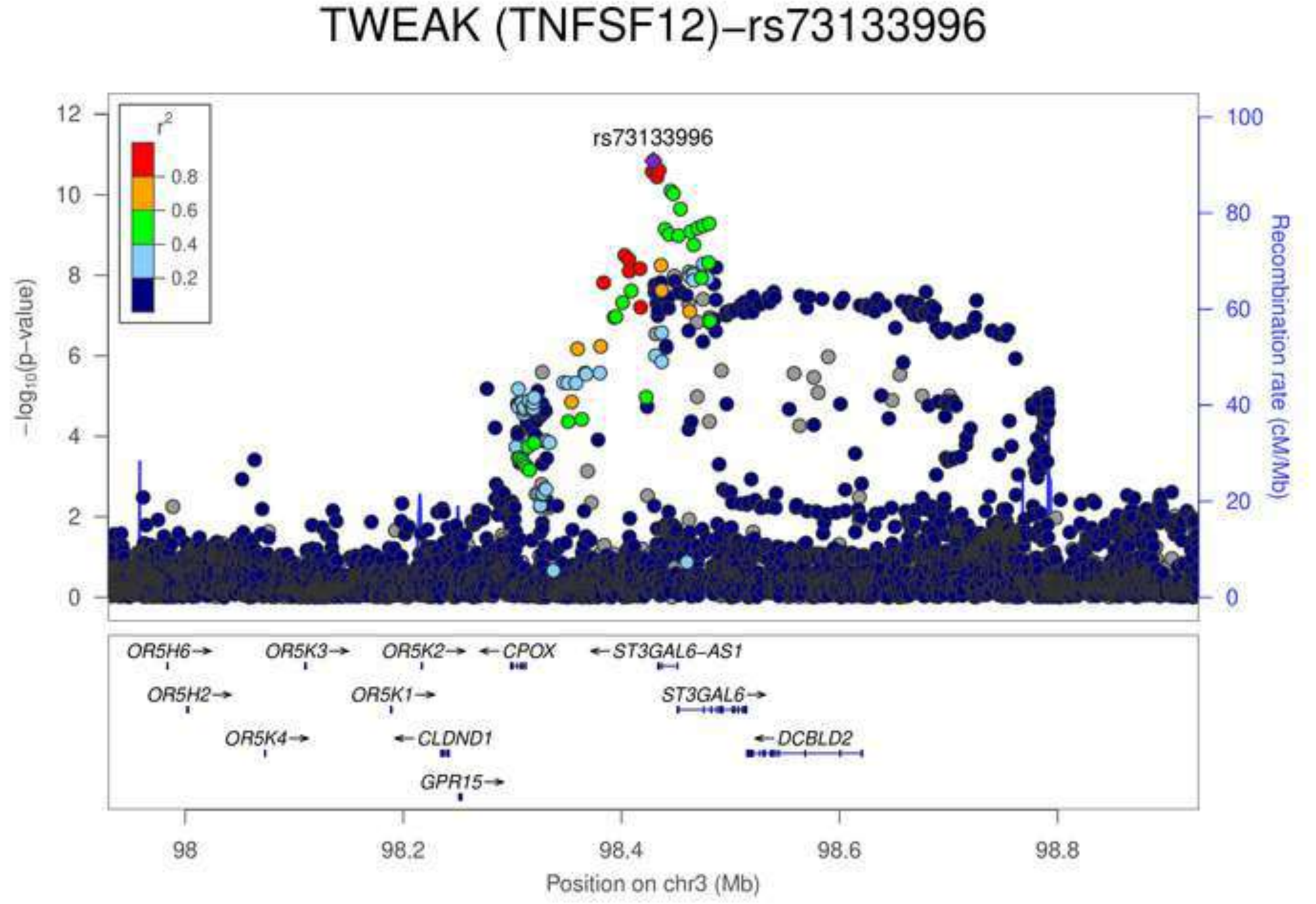
TWEAK (TNFSF12)−rs73133996
rs73133996
r^2
0.8
0.6
0.4
0.2
−log10(p−value)
Recombination rate (cM/Mb)
OR5H6→
OR5K3→
OR5K2→
←CPOX
←ST3GAL6−AS1
OR5H2→
OR5K1→
ST3GAL6→
OR5K4→
←CLDND1
←DCBLD2
GPR15→
98
98.2
98.4
98.6
98.8
Position on chr3 (Mb)

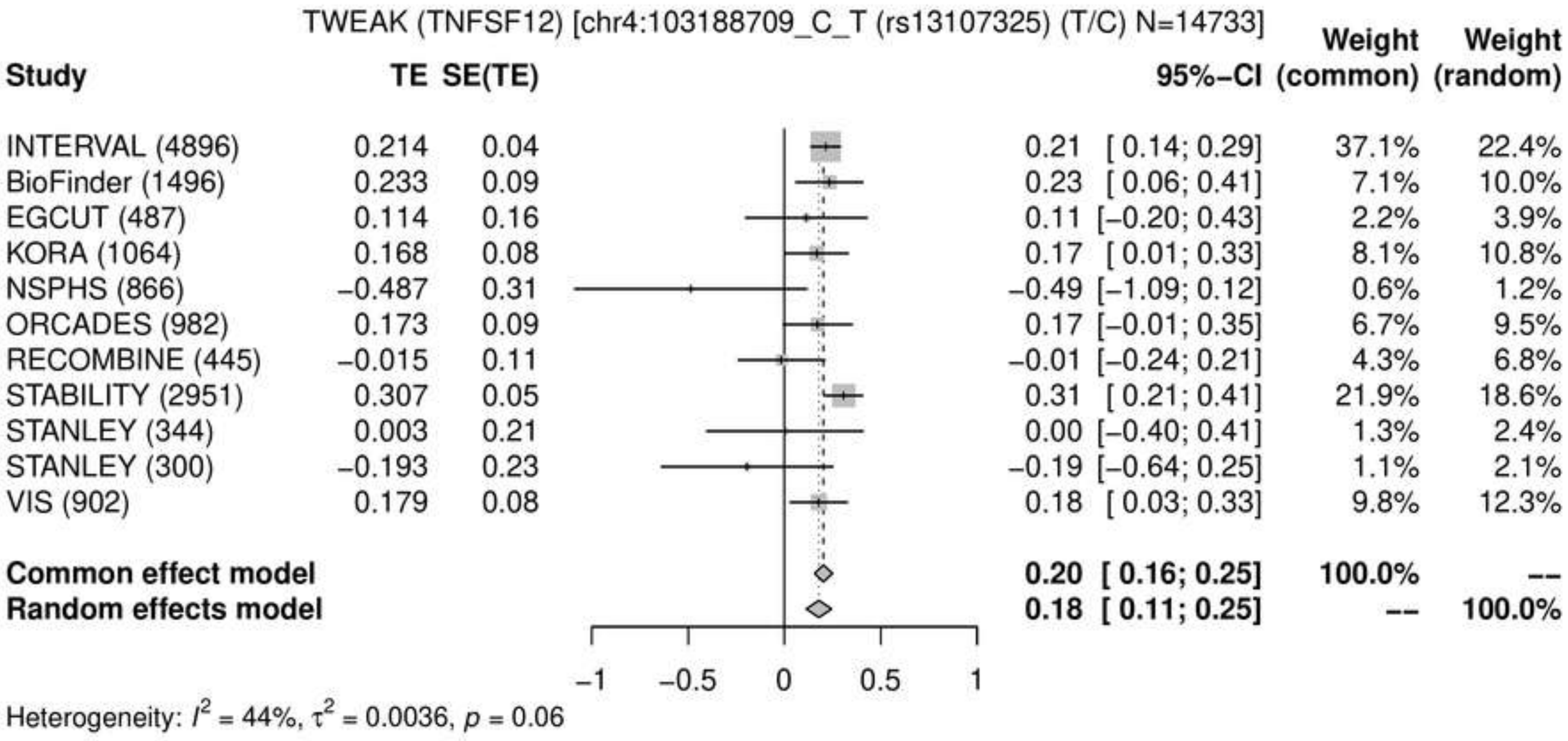
TWEAK (TNFSF12) [chr4:103188709_C_T (rs13107325) (T/C) N=14733]
Study | TE | SE(TE) | 95%-CI | Weight (common) | Weight (random)
INTERVAL (4896) | 0.214 | 0.04 | 0.21 [0.14; 0.29] | 37.1% | 22.4%
BioFinder (1496) | 0.233 | 0.09 | 0.23 [0.06; 0.41] | 7.1% | 10.0%
EGCUT (487) | 0.114 | 0.16 | 0.11 [−0.20; 0.43] | 2.2% | 3.9%
KORA (1064) | 0.168 | 0.08 | 0.17 [0.01; 0.33] | 8.1% | 10.8%
NSPHS (866) | −0.487 | 0.31 | −0.49 [−1.09; 0.12] | 0.6% | 1.2%
ORCADES (982) | 0.173 | 0.09 | 0.17 [−0.01; 0.35] | 6.7% | 9.5%
RECOMBINE (445) | −0.015 | 0.11 | −0.01 [−0.24; 0.21] | 4.3% | 6.8%
STABILITY (2951) | 0.307 | 0.05 | 0.31 [0.21; 0.41] | 21.9% | 18.6%
STANLEY (344) | 0.003 | 0.21 | 0.00 [−0.40; 0.41] | 1.3% | 2.4%
STANLEY (300) | −0.193 | 0.23 | −0.19 [−0.64; 0.25] | 1.1% | 2.1%
VIS (902) | 0.179 | 0.08 | 0.18 [0.03; 0.33] | 9.8% | 12.3%
Common effect model | 0.20 [0.16; 0.25] | 100.0% | --
Random effects model | 0.18 [0.11; 0.25] | -- | 100.0%
−1 −0.5 0 0.5 1
Heterogeneity: I^2 = 44%, τ^2 = 0.0036, p = 0.06

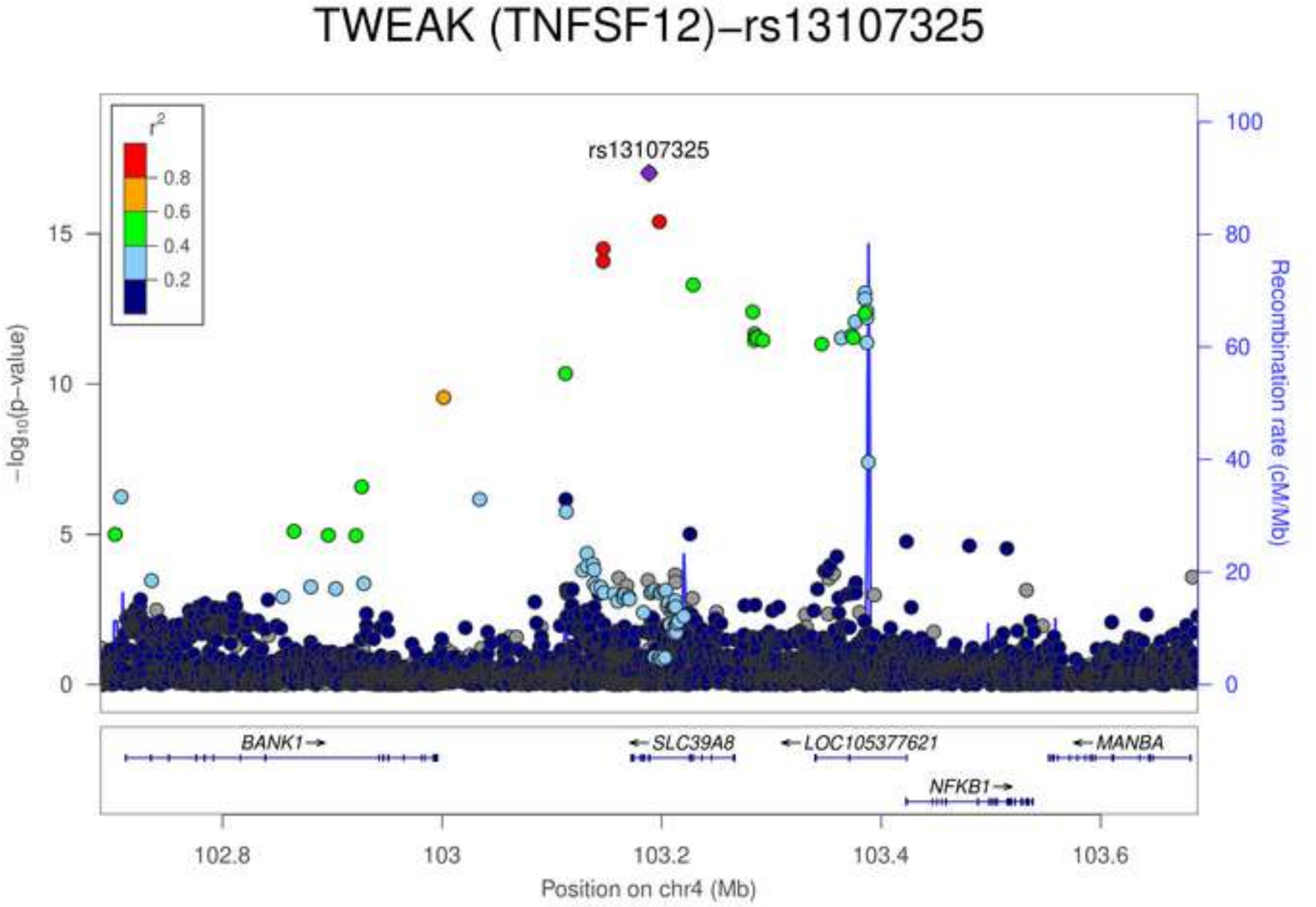
TWEAK (TNFSF12)−rs13107325
rs13107325
r^2
0.8
0.6
0.4
0.2
$-\log_{10}$(p−value)
Recombination rate (cM/Mb)
BANK1
SLC39A8
LOC105377621
MANBA
NFKB1
102.8 103 103.2 103.4 103.6
Position on chr4 (Mb)

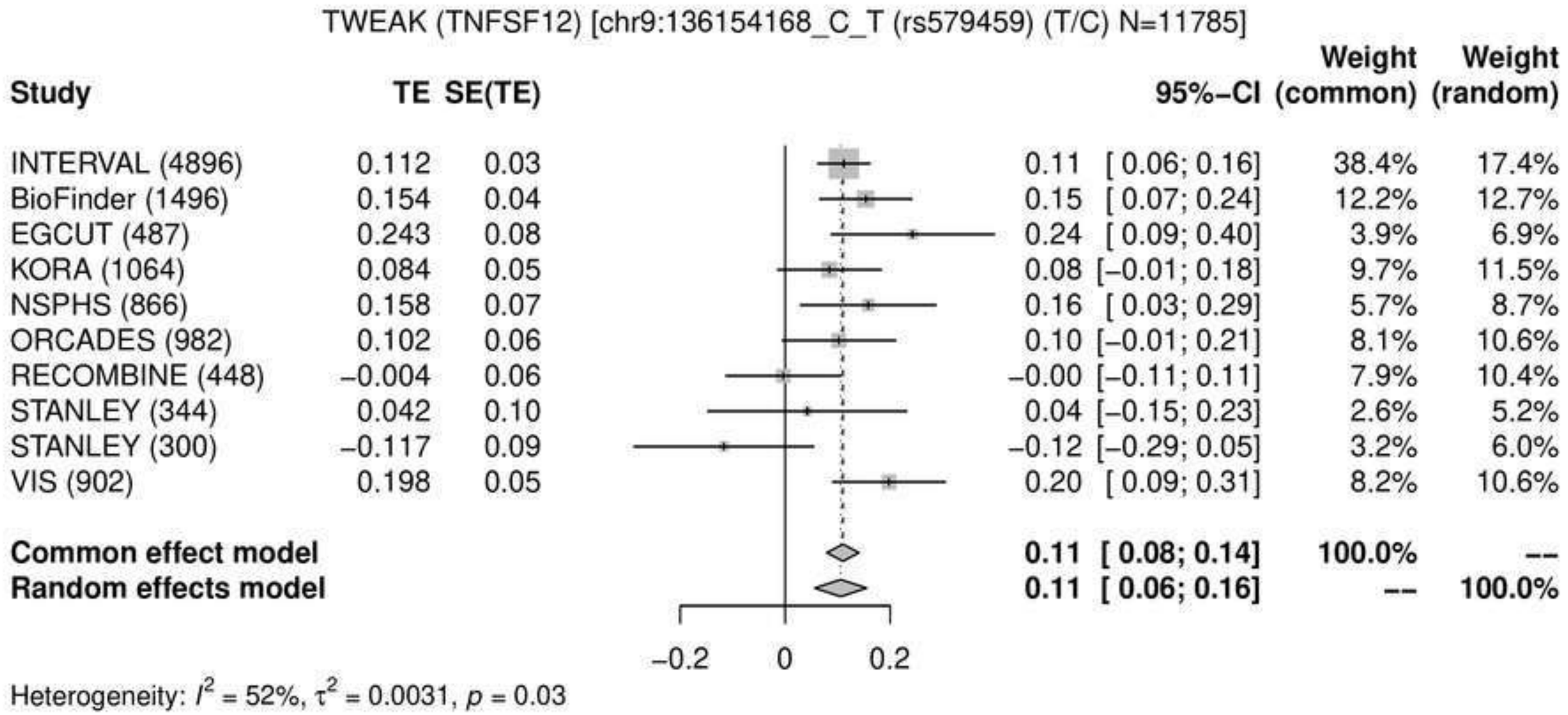
TWEAK (TNFSF12) [chr9:136154168_C_T (rs579459) (T/C) N=11785]
Study TE SE(TE) 95%−CI Weight (common) Weight (random)
INTERVAL (4896) 0.112 0.03 0.11 [0.06; 0.16] 38.4% 17.4%
BioFinder (1496) 0.154 0.04 0.15 [0.07; 0.24] 12.2% 12.7%
EGCUT (487) 0.243 0.08 0.24 [0.09; 0.40] 3.9% 6.9%
KORA (1064) 0.084 0.05 0.08 [−0.01; 0.18] 9.7% 11.5%
NSPHS (866) 0.158 0.07 0.16 [0.03; 0.29] 5.7% 8.7%
ORCADES (982) 0.102 0.06 0.10 [−0.01; 0.21] 8.1% 10.6%
RECOMBINE (448) −0.004 0.06 −0.00 [−0.11; 0.11] 7.9% 10.4%
STANLEY (344) 0.042 0.10 0.04 [−0.15; 0.23] 2.6% 5.2%
STANLEY (300) −0.117 0.09 −0.12 [−0.29; 0.05] 3.2% 6.0%
VIS (902) 0.198 0.05 0.20 [0.09; 0.31] 8.2% 10.6%
Common effect model 0.11 [0.08; 0.14] 100.0% --
Random effects model 0.11 [0.06; 0.16] -- 100.0%
−0.2 0 0.2
Heterogeneity: I^2 = 52%, τ^2 = 0.0031, p = 0.03

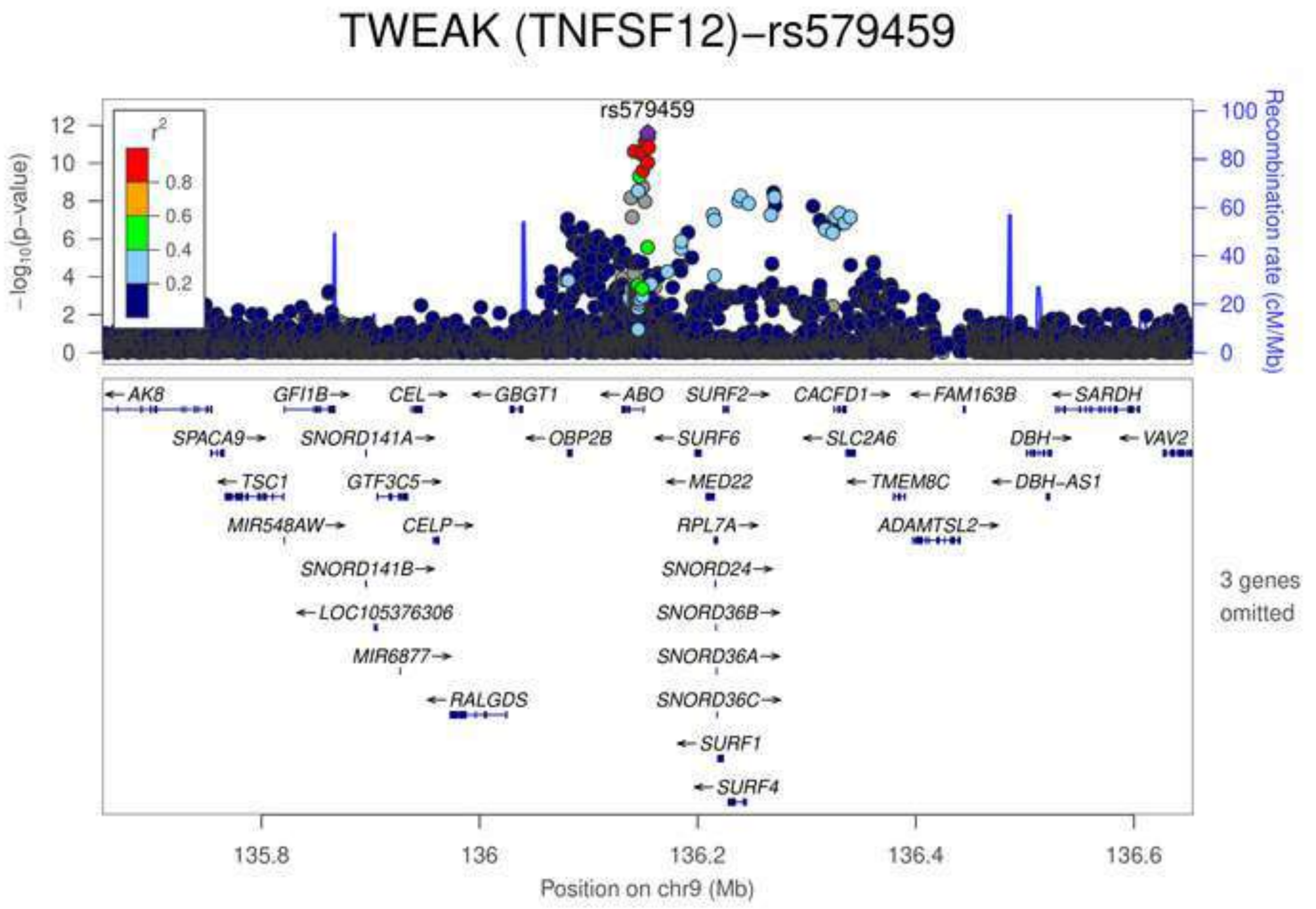
TWEAK (TNFSF12)−rs579459
rs579459
$-\log_{10}$(p−value)
Recombination rate (cM/Mb)
r^2
0.8
0.6
0.4
0.2
AK8
GFI1B
CEL
GBGT1
ABO
SURF2
CACFD1
FAM163B
SARDH
SPACA9
SNORD141A
OBP2B
SURF6
SLC2A6
DBH
VAV2
TSC1
GTF3C5
MED22
TMEM8C
DBH−AS1
MIR548AW
CELP
RPL7A
ADAMTSL2
SNORD141B
SNORD24
LOC105376306
SNORD36B
MIR6877
SNORD36A
RALGDS
SNORD36C
SURF1
SURF4
3 genes omitted
135.8
136
136.2
136.4
136.6
Position on chr9 (Mb)

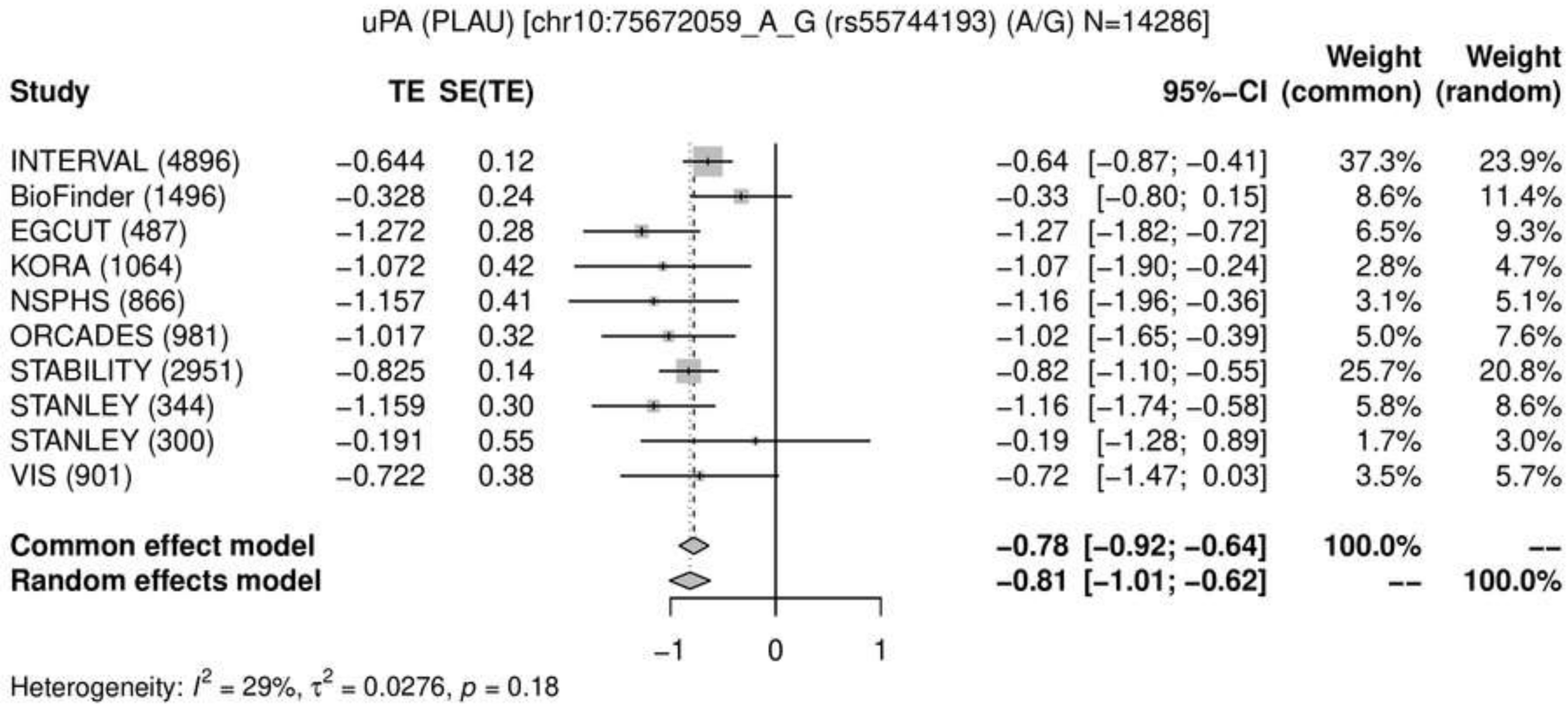
uPA (PLAU) [chr10:75672059_A_G (rs55744193) (A/G) N=14286]
Study
TE SE(TE)
95%−CI
Weight (common)
Weight (random)
INTERVAL (4896) −0.644 0.12 −0.64 [−0.87; −0.41] 37.3% 23.9%
BioFinder (1496) −0.328 0.24 −0.33 [−0.80; 0.15] 8.6% 11.4%
EGCUT (487) −1.272 0.28 −1.27 [−1.82; −0.72] 6.5% 9.3%
KORA (1064) −1.072 0.42 −1.07 [−1.90; −0.24] 2.8% 4.7%
NSPHS (866) −1.157 0.41 −1.16 [−1.96; −0.36] 3.1% 5.1%
ORCADES (981) −1.017 0.32 −1.02 [−1.65; −0.39] 5.0% 7.6%
STABILITY (2951) −0.825 0.14 −0.82 [−1.10; −0.55] 25.7% 20.8%
STANLEY (344) −1.159 0.30 −1.16 [−1.74; −0.58] 5.8% 8.6%
STANLEY (300) −0.191 0.55 −0.19 [−1.28; 0.89] 1.7% 3.0%
VIS (901) −0.722 0.38 −0.72 [−1.47; 0.03] 3.5% 5.7%
Common effect model −0.78 [−0.92; −0.64] 100.0% --
Random effects model −0.81 [−1.01; −0.62] -- 100.0%
−1
0
1
Heterogeneity: I^2 = 29%, τ^2 = 0.0276, p = 0.18
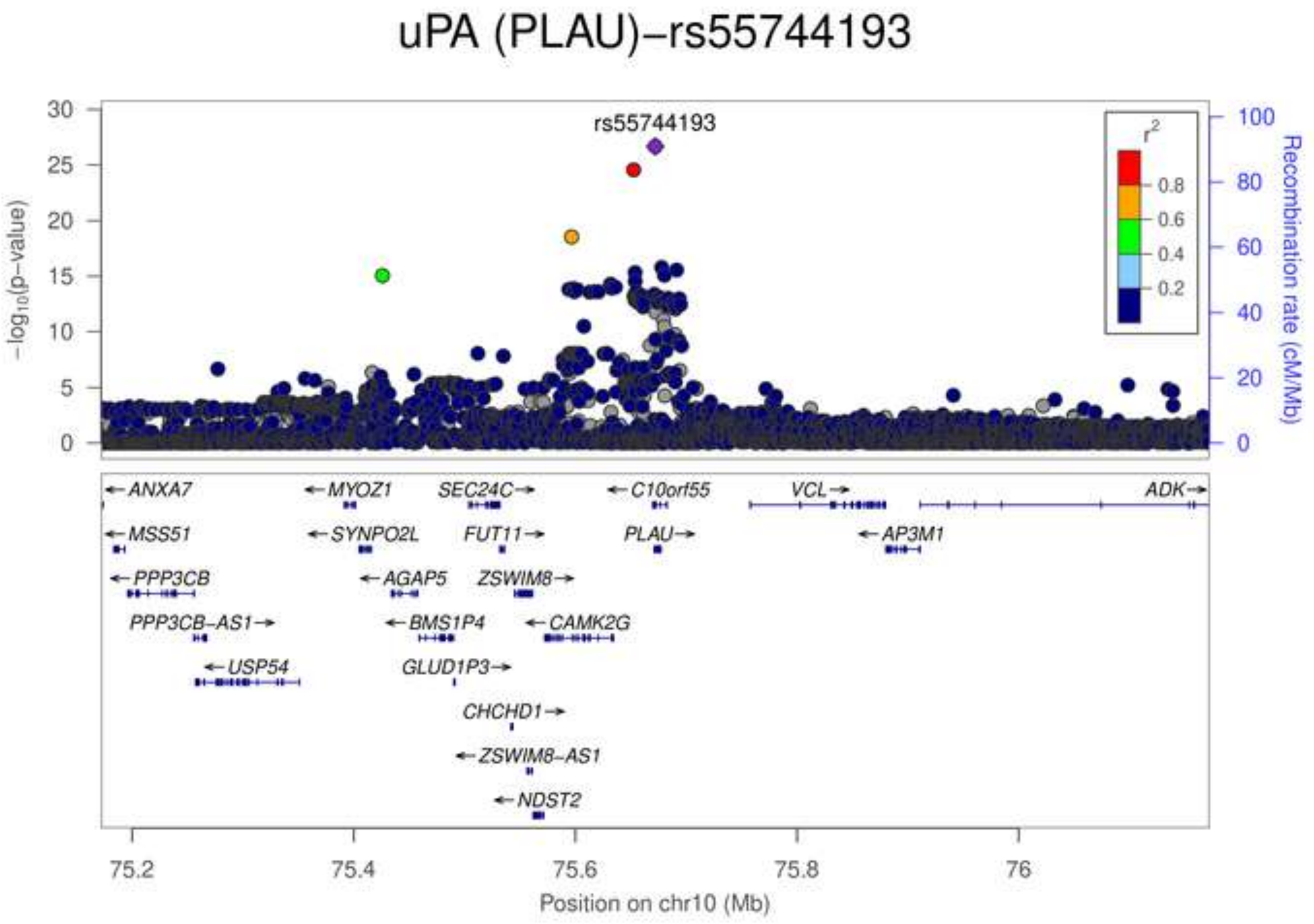
uPA (PLAU)−rs55744193
rs55744193
$-\log_{10}$(p−value)
Recombination rate (cM/Mb)
r^2
0.8
0.6
0.4
0.2
ANXA7
MYOZ1
SEC24C
C10orf55
VCL
ADK
MSS51
SYNPO2L
FUT11
PLAU
AP3M1
PPP3CB
AGAP5
ZSWIM8
PPP3CB-AS1
BMS1P4
CAMK2G
USP54
GLUD1P3
CHCHD1
ZSWIM8-AS1
NDST2
75.2
75.4
75.6
75.8
76
Position on chr10 (Mb)

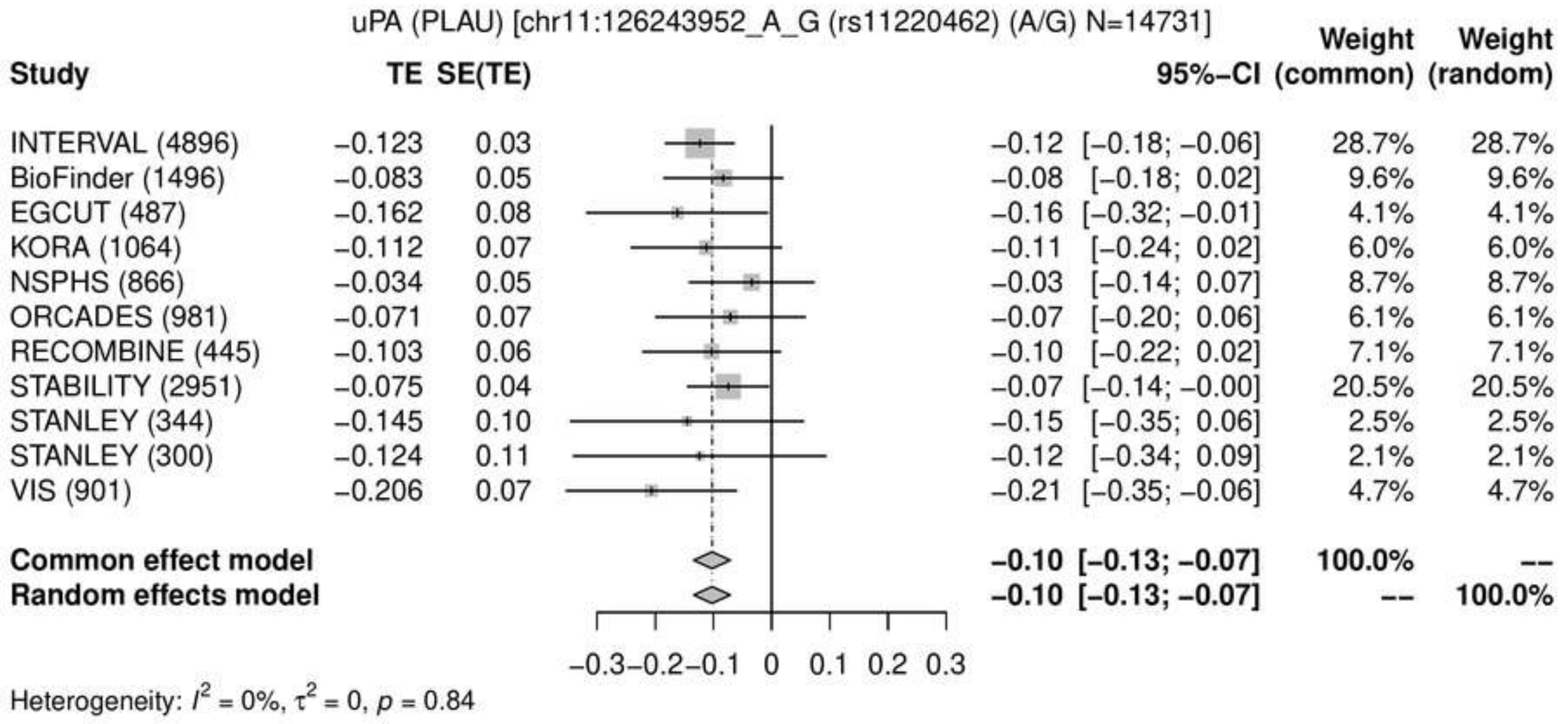
uPA (PLAU) [chr11:126243952_A_G (rs11220462) (A/G) N=14731]
Study
TE
SE(TE)
95%-CI
Weight (common)
Weight (random)
INTERVAL (4896) −0.123 0.03 −0.12 [−0.18; −0.06] 28.7% 28.7%
BioFinder (1496) −0.083 0.05 −0.08 [−0.18; 0.02] 9.6% 9.6%
EGCUT (487) −0.162 0.08 −0.16 [−0.32; −0.01] 4.1% 4.1%
KORA (1064) −0.112 0.07 −0.11 [−0.24; 0.02] 6.0% 6.0%
NSPHS (866) −0.034 0.05 −0.03 [−0.14; 0.07] 8.7% 8.7%
ORCADES (981) −0.071 0.07 −0.07 [−0.20; 0.06] 6.1% 6.1%
RECOMBINE (445) −0.103 0.06 −0.10 [−0.22; 0.02] 7.1% 7.1%
STABILITY (2951) −0.075 0.04 −0.07 [−0.14; −0.00] 20.5% 20.5%
STANLEY (344) −0.145 0.10 −0.15 [−0.35; 0.06] 2.5% 2.5%
STANLEY (300) −0.124 0.11 −0.12 [−0.34; 0.09] 2.1% 2.1%
VIS (901) −0.206 0.07 −0.21 [−0.35; −0.06] 4.7% 4.7%
Common effect model −0.10 [−0.13; −0.07] 100.0% --
Random effects model −0.10 [−0.13; −0.07] -- 100.0%
−0.3 −0.2 −0.1 0 0.1 0.2 0.3
Heterogeneity: $I^2 = 0\%$, $\tau^2 = 0$, $p = 0.84$

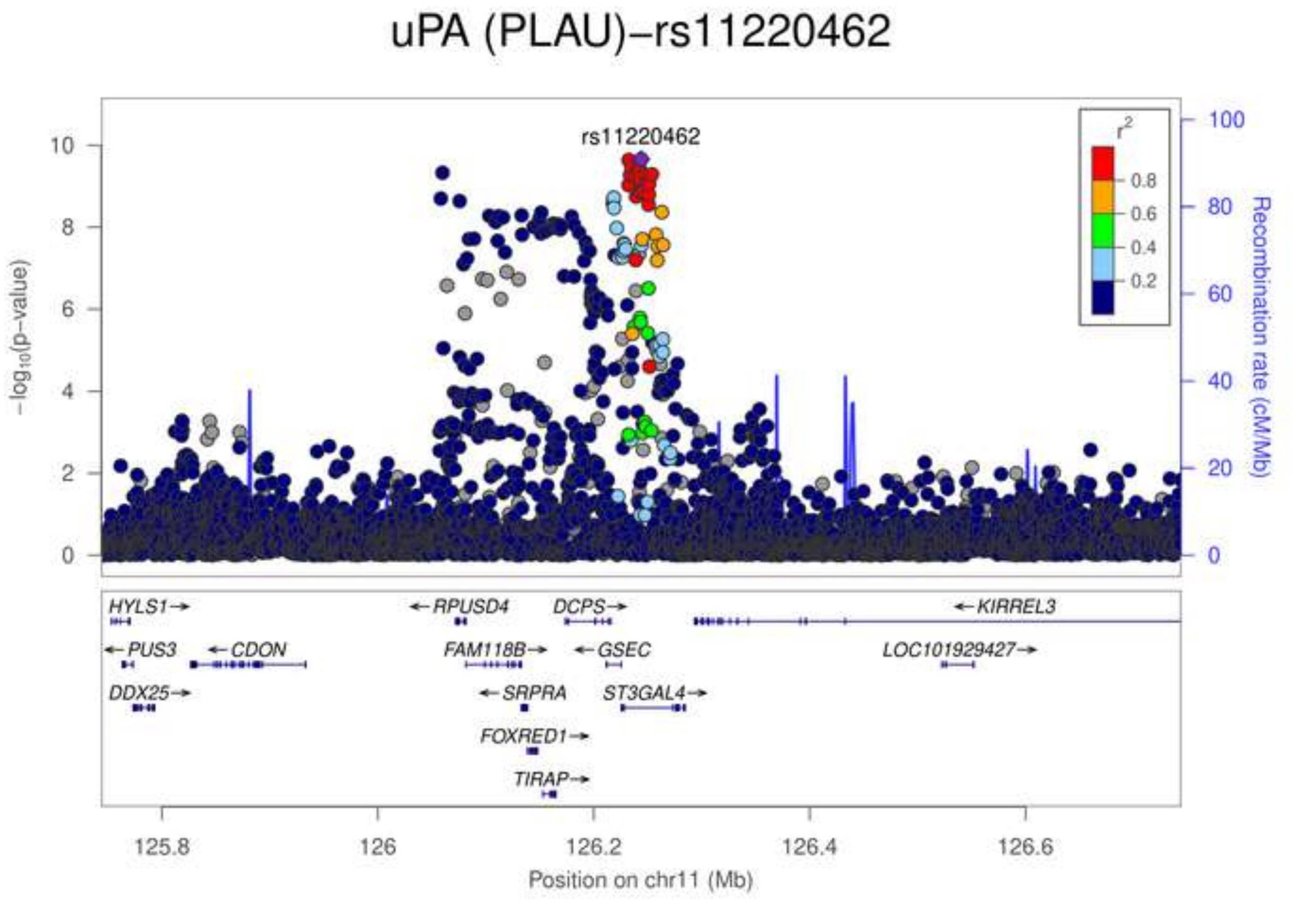
uPA (PLAU)−rs11220462
rs11220462
$-\log_{10}$(p-value)
Recombination rate (cM/Mb)
r^2
0.8
0.6
0.4
0.2
0
2
4
6
8
10
20
40
60
80
100
HYLS1→
←PUS3
←CDON
DDX25→
←RPUSD4
DCPS→
FAM118B→
←GSEC
←SRPRA
ST3GAL4→
FOXRED1→
TIRAP→
←KIRREL3
LOC101929427→
125.8
126
126.2
126.4
126.6
Position on chr11 (Mb)

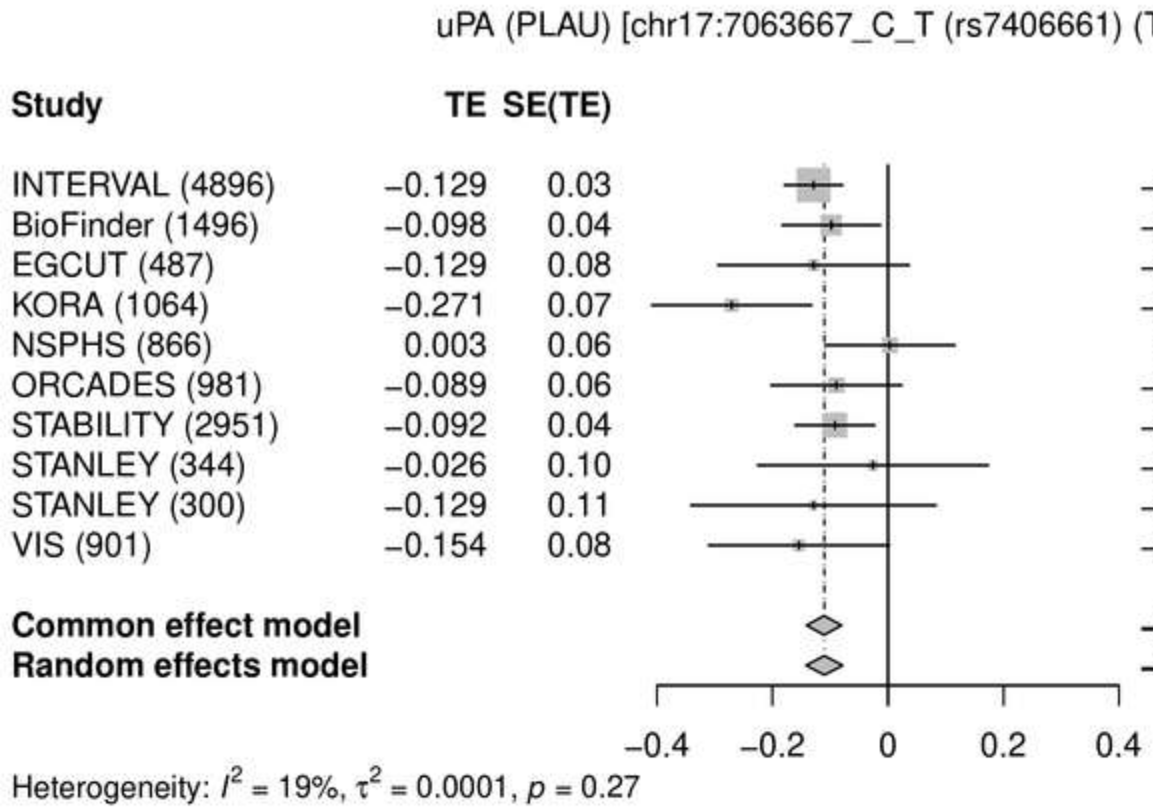
uPA (PLAU) [chr17:7063667_C_T (rs7406661) (T/C) N=14286]
Study
TE
SE(TE)
95%–CI
Weight (common)
Weight (random)
INTERVAL (4896) –0.129 0.03 –0.13 [–0.18; –0.08] 36.6% 34.0%
BioFinder (1496) –0.098 0.04 –0.10 [–0.18; –0.01] 12.8% 13.2%
EGCUT (487) –0.129 0.08 –0.13 [–0.29; 0.04] 3.4% 3.7%
KORA (1064) –0.271 0.07 –0.27 [–0.41; –0.13] 4.9% 5.2%
NSPHS (866) 0.003 0.06 0.00 [–0.11; 0.12] 7.4% 7.8%
ORCADES (981) –0.089 0.06 –0.09 [–0.20; 0.02] 7.2% 7.7%
STABILITY (2951) –0.092 0.04 –0.09 [–0.16; –0.02] 19.5% 19.5%
STANLEY (344) –0.026 0.10 –0.03 [–0.23; 0.17] 2.3% 2.5%
STANLEY (300) –0.129 0.11 –0.13 [–0.34; 0.08] 2.1% 2.2%
VIS (901) –0.154 0.08 –0.15 [–0.31; 0.00] 3.8% 4.1%
Common effect model –0.11 [–0.14; –0.08] 100.0% --
Random effects model –0.11 [–0.14; –0.08] -- 100.0%
–0.4
–0.2
0
0.2
0.4
Heterogeneity: I^2 = 19%, τ^2 = 0.0001, p = 0.27

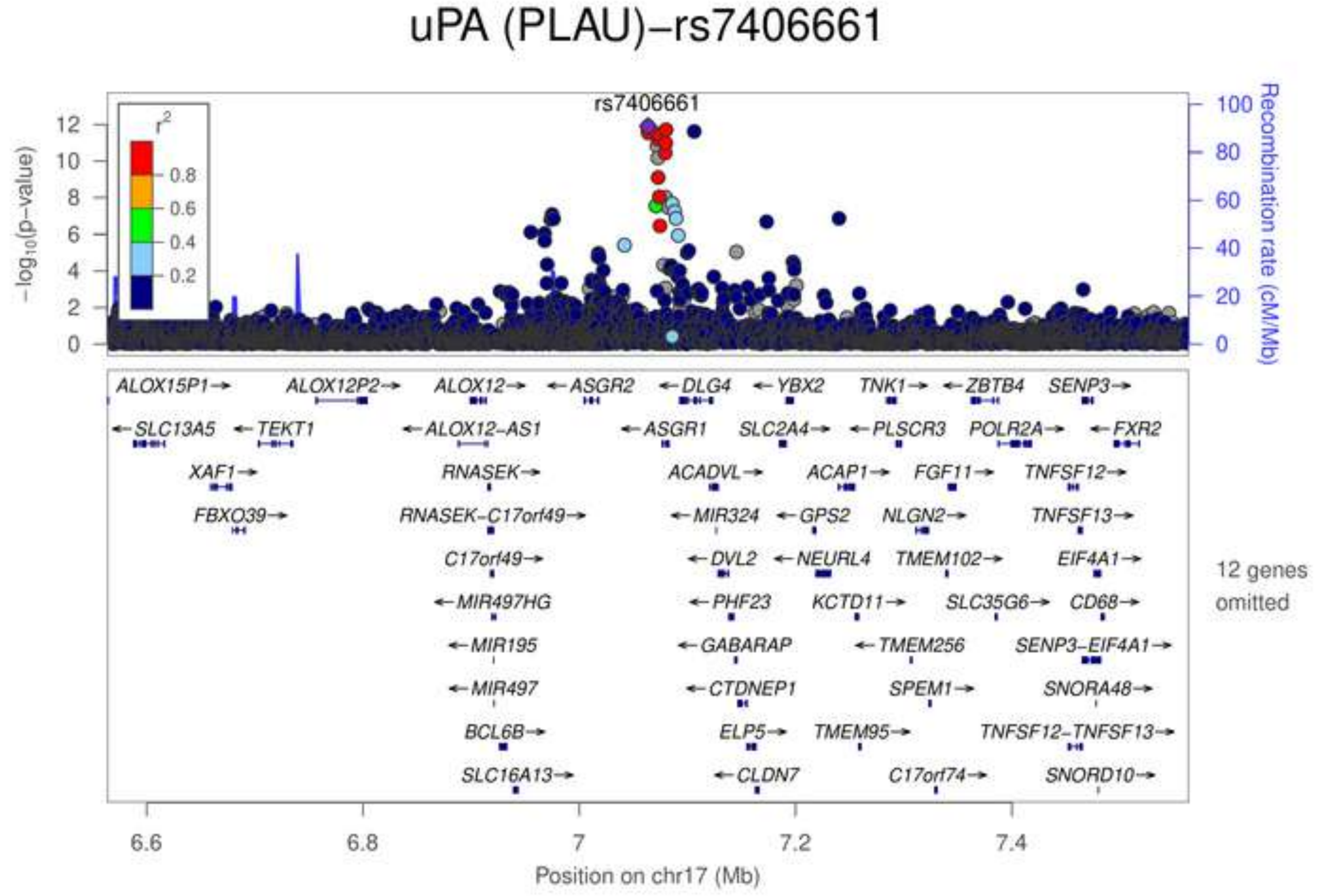
uPA (PLAU)–rs7406661
rs7406661
$-\log_{10}$(p-value)
Recombination rate (cM/Mb)
r^2
0.8
0.6
0.4
0.2
ALOX15P1→ ALOX12P2→ ALOX12→ ←ASGR2 ←DLG4 ←YBX2 TNK1→ ←ZBTB4 SENP3→
←SLC13A5 ←TEKT1 ←ALOX12-AS1 ←ASGR1 SLC2A4→ ←PLSCR3 POLR2A→ ←FXR2
XAF1→ RNASEK→ ACADVL→ ACAP1→ FGF11→ TNFSF12→
FBXO39→ RNASEK-C17orf49→ ←MIR324 ←GPS2 NLGN2→ TNFSF13→
C17orf49→ ←DVL2 ←NEURL4 TMEM102→ EIF4A1→
←MIR497HG ←PHF23 KCTD11→ SLC35G6→ CD68→
←MIR195 ←GABARAP ←TMEM256 SENP3-EIF4A1→
←MIR497 ←CTDNEP1 SPEM1→ SNORA48→
BCL6B→ ELP5→ TMEM95→ TNFSF12-TNFSF13→
SLC16A13→ ←CLDN7 C17orf74→ SNORD10→
12 genes omitted
6.6
6.8
7
7.2
7.4
Position on chr17 (Mb)

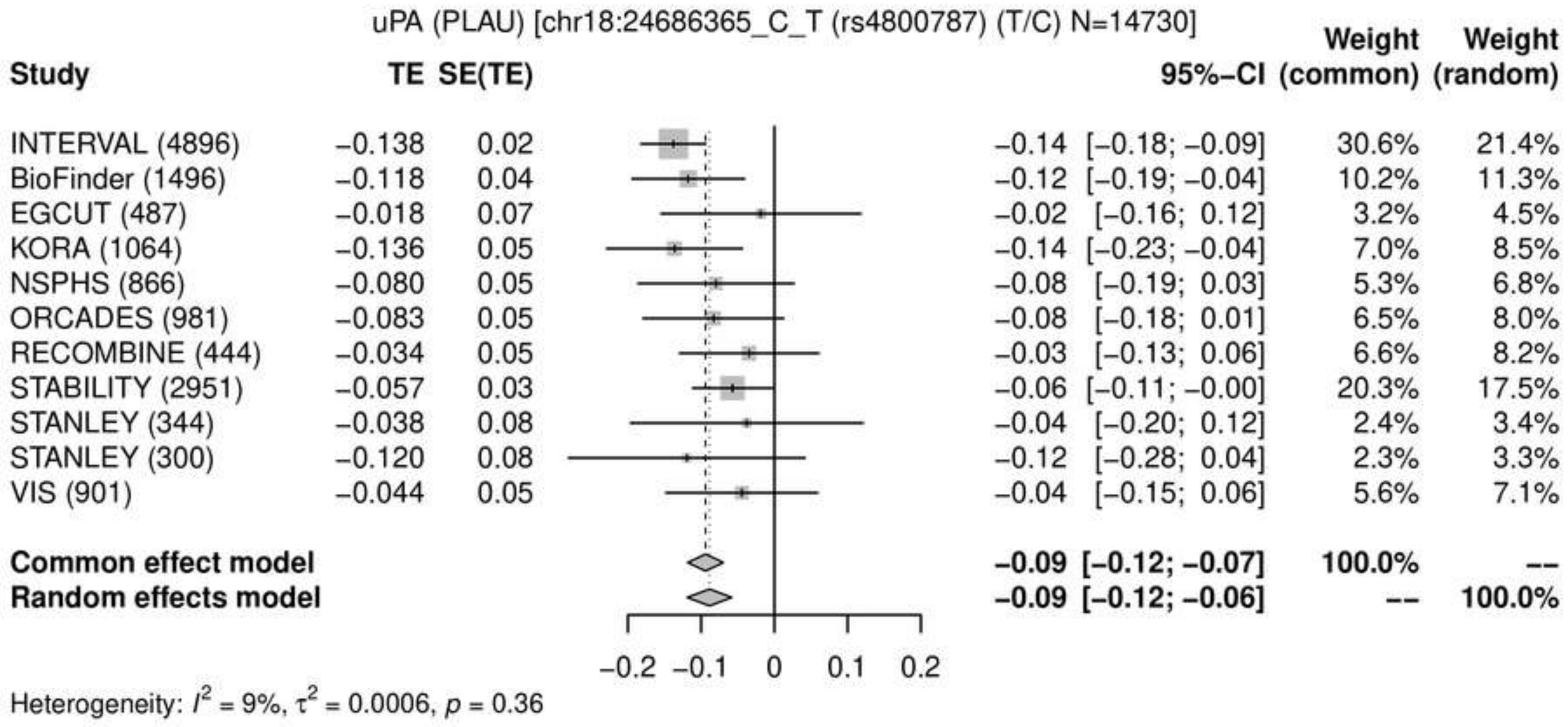
uPA (PLAU) [chr18:24686365_C_T (rs4800787) (T/C) N=14730]
Study
TE
SE(TE)
95%−CI
Weight (common)
Weight (random)
INTERVAL (4896) −0.138 0.02 −0.14 [−0.18; −0.09] 30.6% 21.4%
BioFinder (1496) −0.118 0.04 −0.12 [−0.19; −0.04] 10.2% 11.3%
EGCUT (487) −0.018 0.07 −0.02 [−0.16; 0.12] 3.2% 4.5%
KORA (1064) −0.136 0.05 −0.14 [−0.23; −0.04] 7.0% 8.5%
NSPHS (866) −0.080 0.05 −0.08 [−0.19; 0.03] 5.3% 6.8%
ORCADES (981) −0.083 0.05 −0.08 [−0.18; 0.01] 6.5% 8.0%
RECOMBINE (444) −0.034 0.05 −0.03 [−0.13; 0.06] 6.6% 8.2%
STABILITY (2951) −0.057 0.03 −0.06 [−0.11; −0.00] 20.3% 17.5%
STANLEY (344) −0.038 0.08 −0.04 [−0.20; 0.12] 2.4% 3.4%
STANLEY (300) −0.120 0.08 −0.12 [−0.28; 0.04] 2.3% 3.3%
VIS (901) −0.044 0.05 −0.04 [−0.15; 0.06] 5.6% 7.1%
Common effect model −0.09 [−0.12; −0.07] 100.0% --
Random effects model −0.09 [−0.12; −0.06] -- 100.0%
−0.2 −0.1 0 0.1 0.2
Heterogeneity: I^2 = 9%, τ^2 = 0.0006, p = 0.36

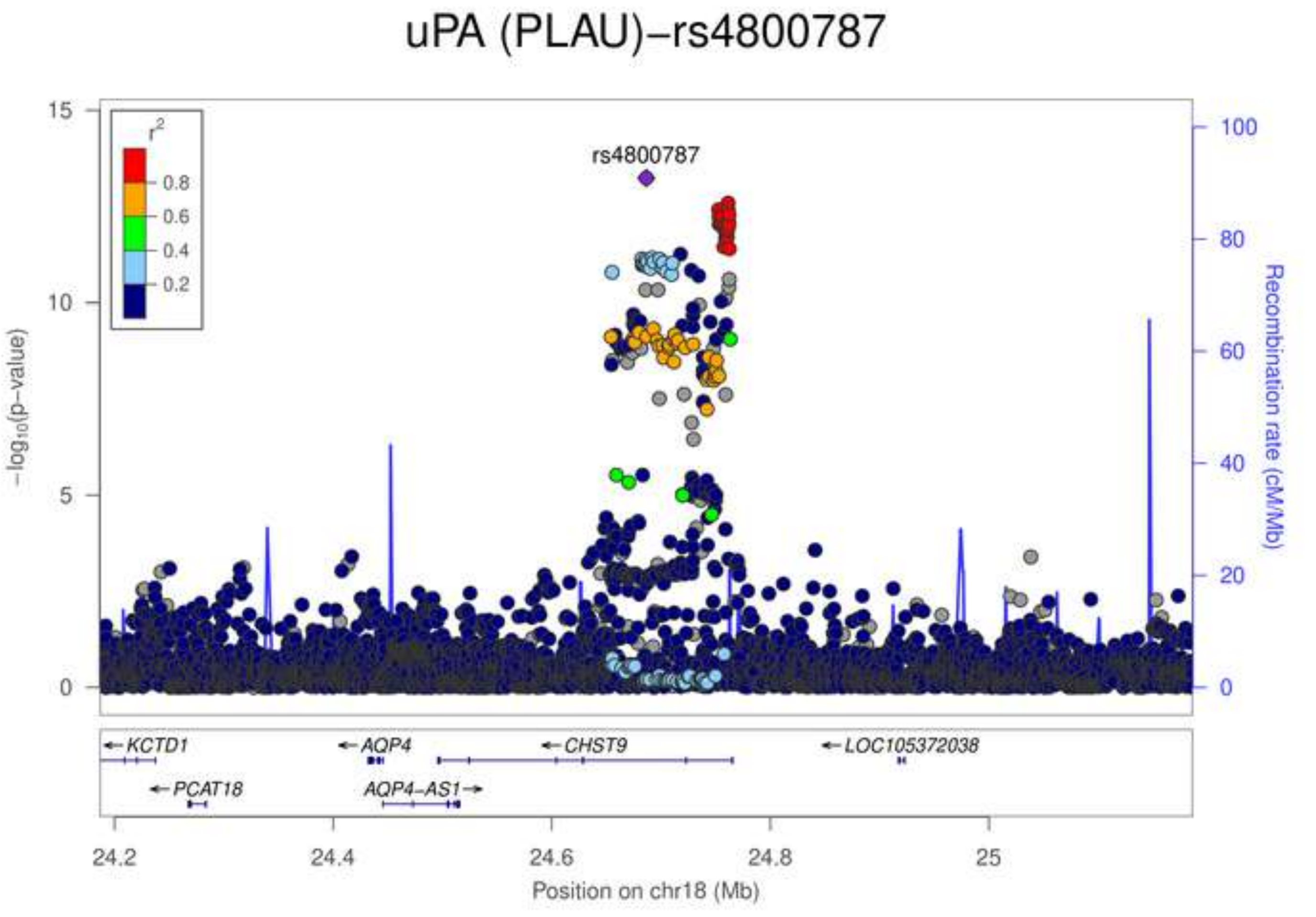
uPA (PLAU)−rs4800787
rs4800787
r^2
0.8
0.6
0.4
0.2
$-\log_{10}$(p−value)
Recombination rate (cM/Mb)
KCTD1
AQP4
CHST9
LOC105372038
PCAT18
AQP4−AS1
24.2 24.4 24.6 24.8 25
Position on chr18 (Mb)

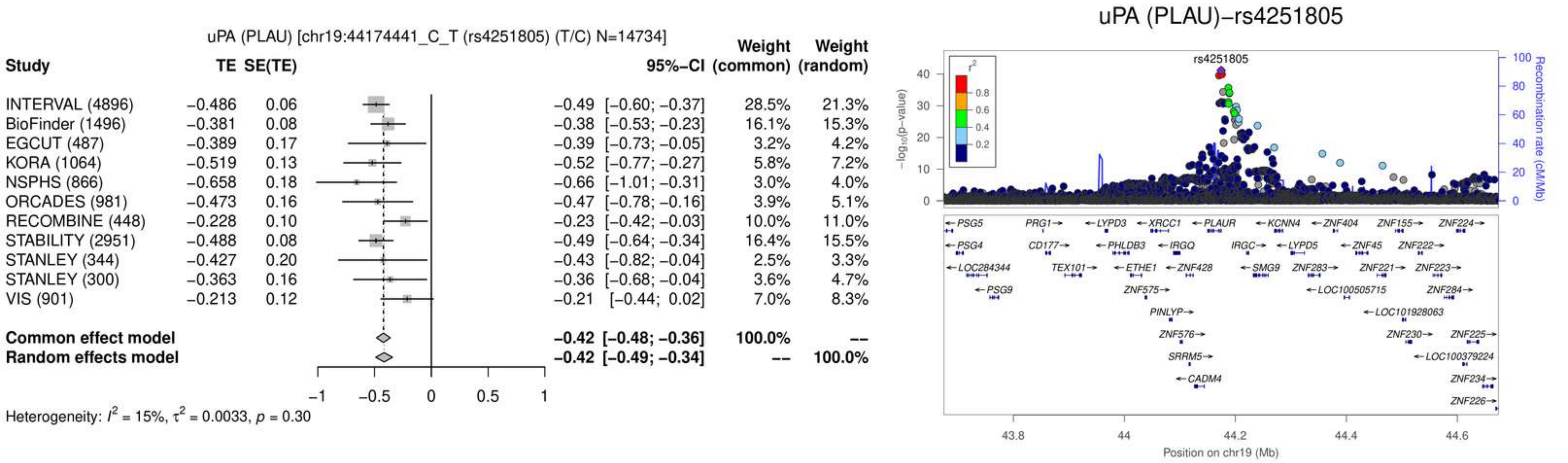

uPA (PLAU) [chr19:44174441_C_T (rs4251805) (T/C) N=14734]
Study
TE
SE(TE)
95%-CI
Weight (common)
Weight (random)
INTERVAL (4896) −0.486 0.06 −0.49 [−0.60; −0.37] 28.5% 21.3%
BioFinder (1496) −0.381 0.08 −0.38 [−0.53; −0.23] 16.1% 15.3%
EGCUT (487) −0.389 0.17 −0.39 [−0.73; −0.05] 3.2% 4.2%
KORA (1064) −0.519 0.13 −0.52 [−0.77; −0.27] 5.8% 7.2%
NSPHS (866) −0.658 0.18 −0.66 [−1.01; −0.31] 3.0% 4.0%
ORCADES (981) −0.473 0.16 −0.47 [−0.78; −0.16] 3.9% 5.1%
RECOMBINE (448) −0.228 0.10 −0.23 [−0.42; −0.03] 10.0% 11.0%
STABILITY (2951) −0.488 0.08 −0.49 [−0.64; −0.34] 16.4% 15.5%
STANLEY (344) −0.427 0.20 −0.43 [−0.82; −0.04] 2.5% 3.3%
STANLEY (300) −0.363 0.16 −0.36 [−0.68; −0.04] 3.6% 4.7%
VIS (901) −0.213 0.12 −0.21 [−0.44; 0.02] 7.0% 8.3%
Common effect model −0.42 [−0.48; −0.36] 100.0% --
Random effects model −0.42 [−0.49; −0.34] -- 100.0%
−1
−0.5
0
0.5
1
Heterogeneity: I^2 = 15%, τ^2 = 0.0033, p = 0.30
uPA (PLAU)−rs4251805
rs4251805
r^2
0.8
0.6
0.4
0.2
−log10(p−value)
Recombination rate (cM/Mb)
PSG5
PRG1
LYPD3
XRCC1
PLAUR
KCNN4
ZNF404
ZNF155
ZNF224
PSG4
CD177
PHLDB3
IRGQ
IRGC
LYPD5
ZNF45
ZNF222
LOC284344
TEX101
ETHE1
ZNF428
SMG9
ZNF283
ZNF221
ZNF223
PSG9
ZNF575
LOC100505715
ZNF284
PINLYP
LOC101928063
ZNF576
ZNF230
ZNF225
SRRM5
LOC100379224
CADM4
ZNF234
ZNF226
43.8
44
44.2
44.4
44.6
Position on chr19 (Mb)

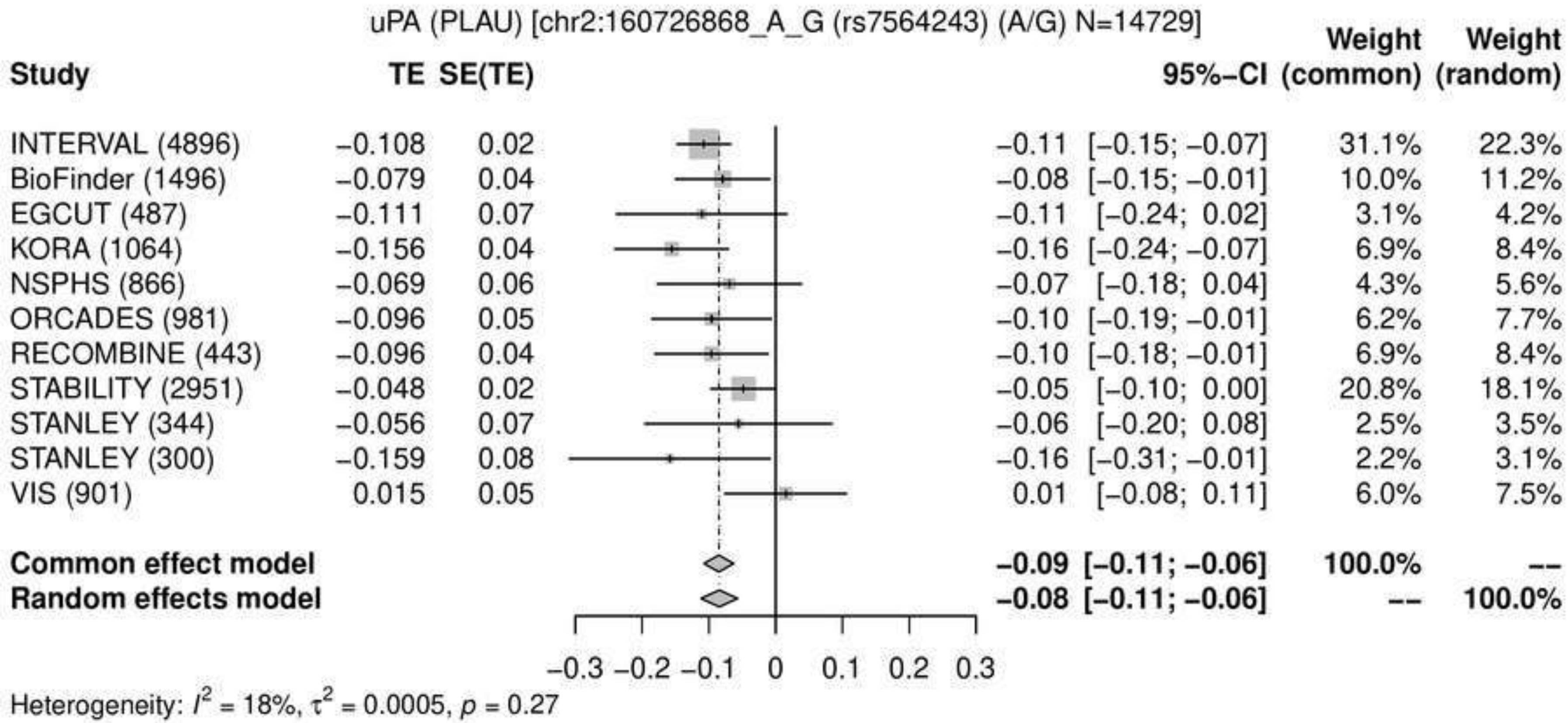
uPA (PLAU) [chr2:160726868_A_G (rs7564243) (A/G) N=14729]
Study
TE
SE(TE)
95%-CI
Weight (common)
Weight (random)
INTERVAL (4896) −0.108 0.02 −0.11 [−0.15; −0.07] 31.1% 22.3%
BioFinder (1496) −0.079 0.04 −0.08 [−0.15; −0.01] 10.0% 11.2%
EGCUT (487) −0.111 0.07 −0.11 [−0.24; 0.02] 3.1% 4.2%
KORA (1064) −0.156 0.04 −0.16 [−0.24; −0.07] 6.9% 8.4%
NSPHS (866) −0.069 0.06 −0.07 [−0.18; 0.04] 4.3% 5.6%
ORCADES (981) −0.096 0.05 −0.10 [−0.19; −0.01] 6.2% 7.7%
RECOMBINE (443) −0.096 0.04 −0.10 [−0.18; −0.01] 6.9% 8.4%
STABILITY (2951) −0.048 0.02 −0.05 [−0.10; 0.00] 20.8% 18.1%
STANLEY (344) −0.056 0.07 −0.06 [−0.20; 0.08] 2.5% 3.5%
STANLEY (300) −0.159 0.08 −0.16 [−0.31; −0.01] 2.2% 3.1%
VIS (901) 0.015 0.05 0.01 [−0.08; 0.11] 6.0% 7.5%
Common effect model −0.09 [−0.11; −0.06] 100.0% --
Random effects model −0.08 [−0.11; −0.06] -- 100.0%
−0.3 −0.2 −0.1 0 0.1 0.2 0.3
Heterogeneity: I² = 18%, τ² = 0.0005, p = 0.27

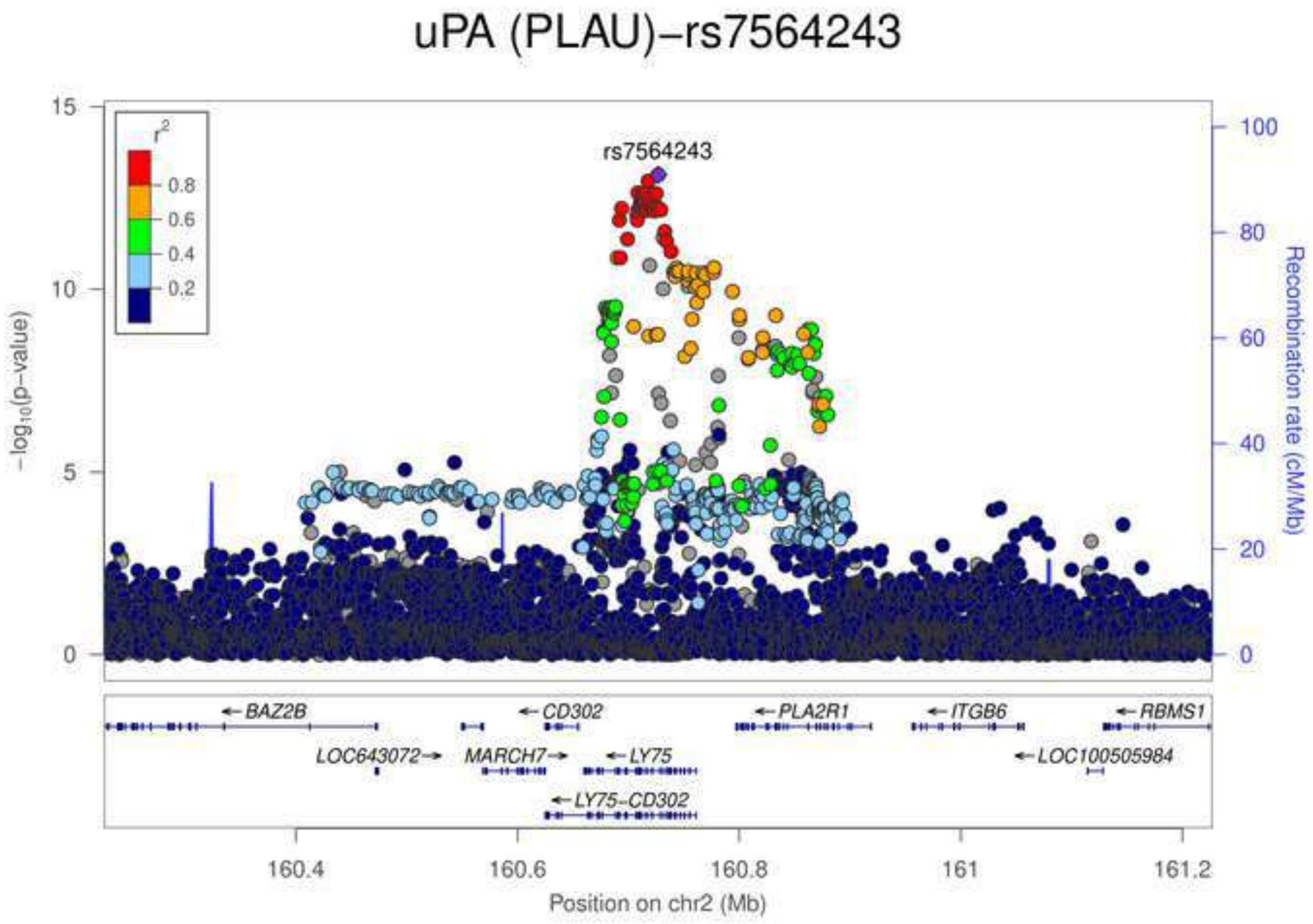
uPA (PLAU)−rs7564243
rs7564243
r²
0.8
0.6
0.4
0.2
−log10(p−value)
Recombination rate (cM/Mb)
15
10
5
0
100
80
60
40
20
0
BAZ2B
CD302
PLA2R1
ITGB6
RBMS1
LOC643072
MARCH7
LY75
LOC100505984
LY75−CD302
160.4
160.6
160.8
161
161.2
Position on chr2 (Mb)

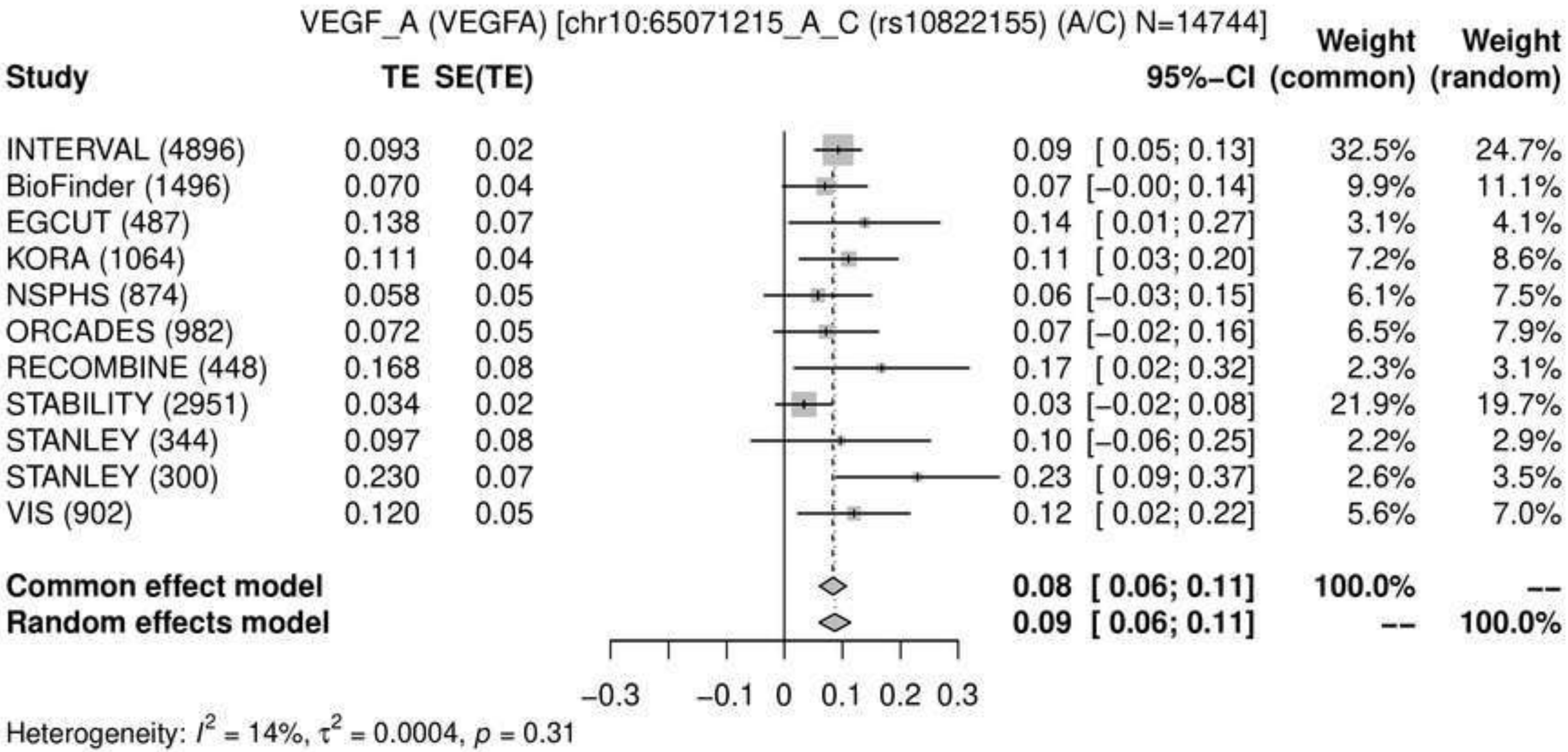
VEGF_A (VEGFA) [chr10:65071215_A_C (rs10822155) (A/C) N=14744]
Study
TE
SE(TE)
95%−CI
Weight (common)
Weight (random)
INTERVAL (4896) 0.093 0.02 0.09 [0.05; 0.13] 32.5% 24.7%
BioFinder (1496) 0.070 0.04 0.07 [−0.00; 0.14] 9.9% 11.1%
EGCUT (487) 0.138 0.07 0.14 [0.01; 0.27] 3.1% 4.1%
KORA (1064) 0.111 0.04 0.11 [0.03; 0.20] 7.2% 8.6%
NSPHS (874) 0.058 0.05 0.06 [−0.03; 0.15] 6.1% 7.5%
ORCADES (982) 0.072 0.05 0.07 [−0.02; 0.16] 6.5% 7.9%
RECOMBINE (448) 0.168 0.08 0.17 [0.02; 0.32] 2.3% 3.1%
STABILITY (2951) 0.034 0.02 0.03 [−0.02; 0.08] 21.9% 19.7%
STANLEY (344) 0.097 0.08 0.10 [−0.06; 0.25] 2.2% 2.9%
STANLEY (300) 0.230 0.07 0.23 [0.09; 0.37] 2.6% 3.5%
VIS (902) 0.120 0.05 0.12 [0.02; 0.22] 5.6% 7.0%
Common effect model 0.08 [0.06; 0.11] 100.0% --
Random effects model 0.09 [0.06; 0.11] -- 100.0%
−0.3 −0.1 0 0.1 0.2 0.3
Heterogeneity: I^2 = 14%, τ^2 = 0.0004, p = 0.31

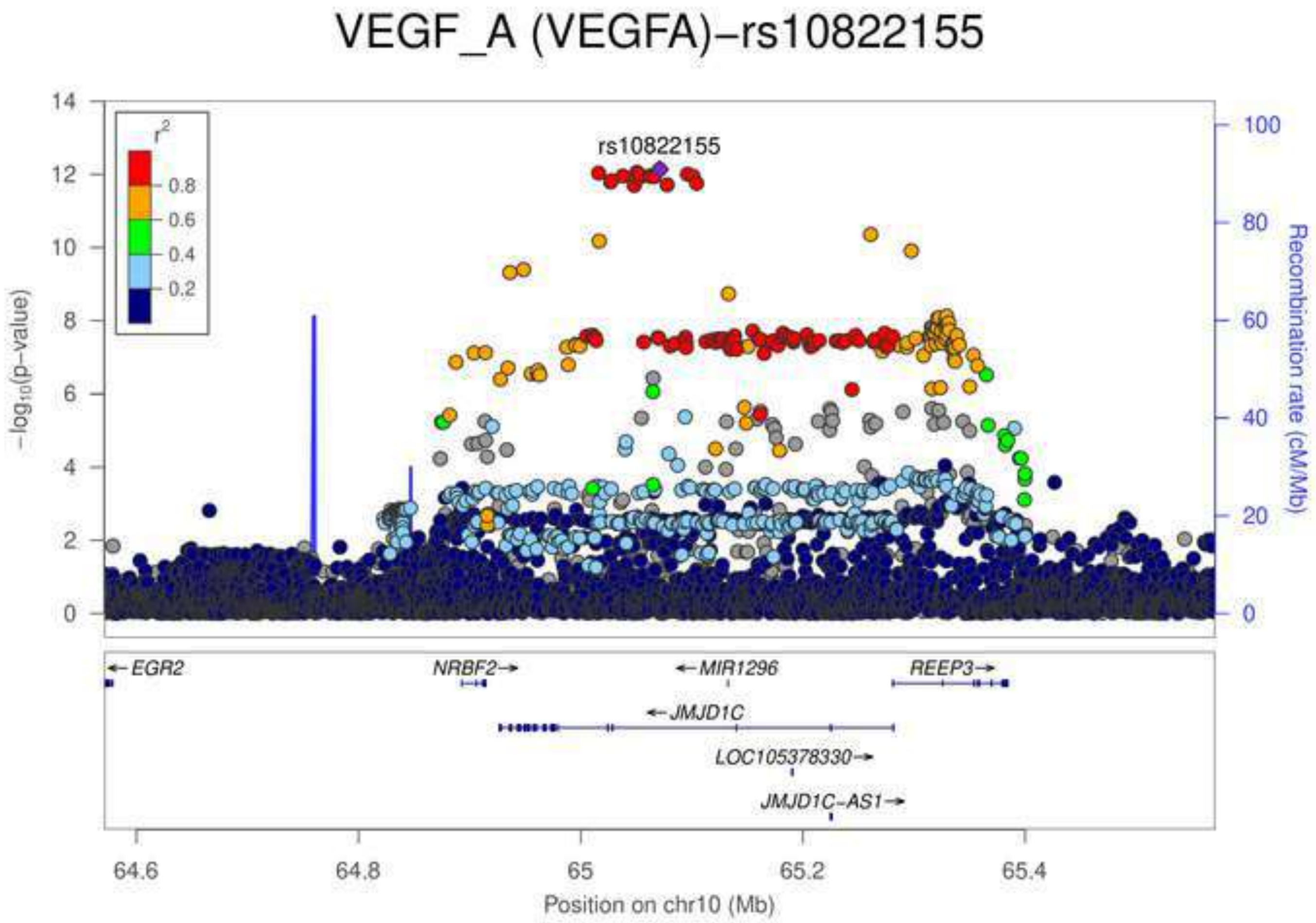
VEGF_A (VEGFA)−rs10822155
rs10822155
r^2
0.8
0.6
0.4
0.2
$-\log_{10}$(p−value)
Recombination rate (cM/Mb)
EGR2
NRBF2
MIR1296
REEP3
JMJD1C
LOC105378330
JMJD1C−AS1
64.6
64.8
65
65.2
65.4
Position on chr10 (Mb)

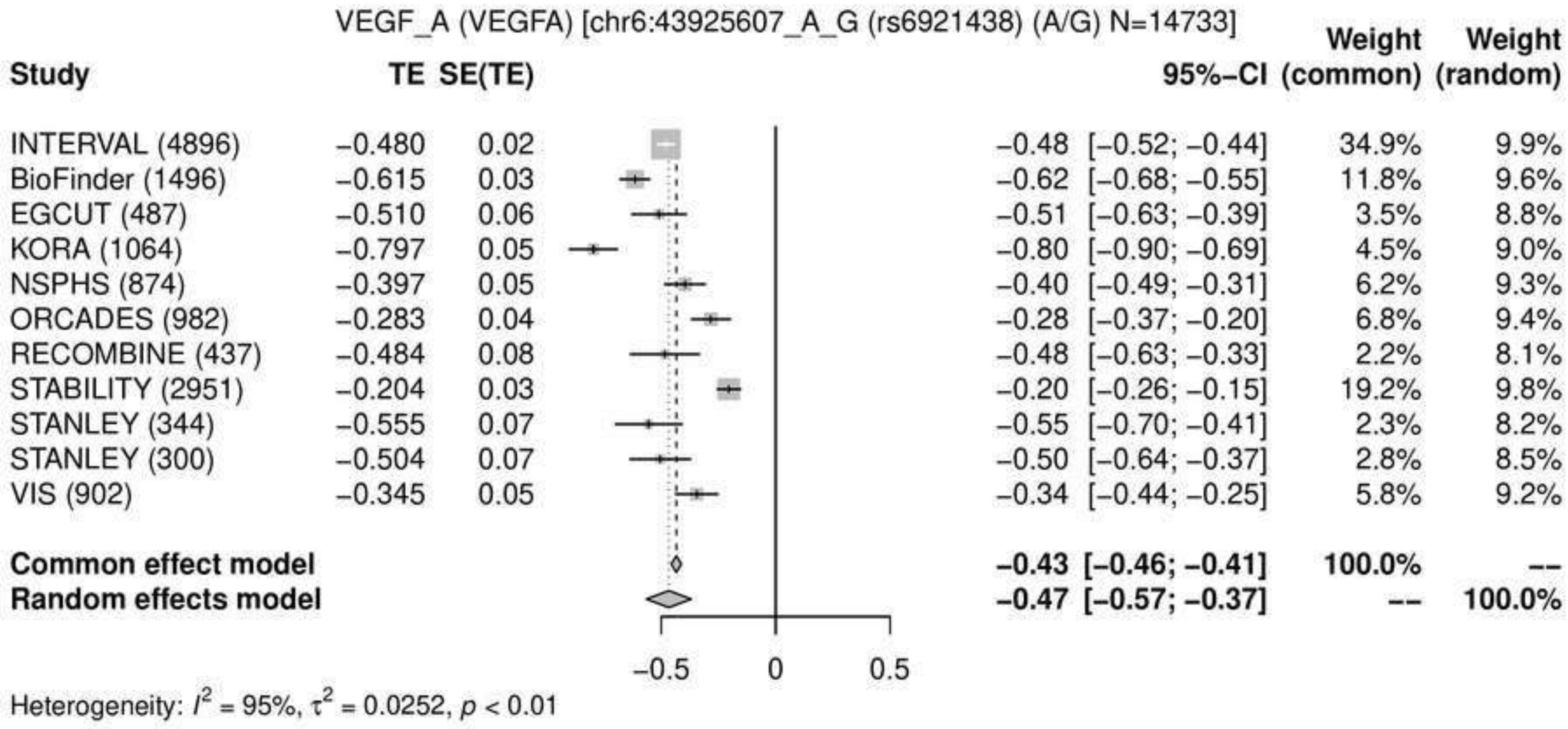
VEGF_A (VEGFA) [chr6:43925607_A_G (rs6921438) (A/G) N=14733]
Study
TE
SE(TE)
95%-CI
Weight (common)
Weight (random)
INTERVAL (4896) −0.480 0.02 −0.48 [−0.52; −0.44] 34.9% 9.9%
BioFinder (1496) −0.615 0.03 −0.62 [−0.68; −0.55] 11.8% 9.6%
EGCUT (487) −0.510 0.06 −0.51 [−0.63; −0.39] 3.5% 8.8%
KORA (1064) −0.797 0.05 −0.80 [−0.90; −0.69] 4.5% 9.0%
NSPHS (874) −0.397 0.05 −0.40 [−0.49; −0.31] 6.2% 9.3%
ORCADES (982) −0.283 0.04 −0.28 [−0.37; −0.20] 6.8% 9.4%
RECOMBINE (437) −0.484 0.08 −0.48 [−0.63; −0.33] 2.2% 8.1%
STABILITY (2951) −0.204 0.03 −0.20 [−0.26; −0.15] 19.2% 9.8%
STANLEY (344) −0.555 0.07 −0.55 [−0.70; −0.41] 2.3% 8.2%
STANLEY (300) −0.504 0.07 −0.50 [−0.64; −0.37] 2.8% 8.5%
VIS (902) −0.345 0.05 −0.34 [−0.44; −0.25] 5.8% 9.2%
Common effect model −0.43 [−0.46; −0.41] 100.0% --
Random effects model −0.47 [−0.57; −0.37] -- 100.0%
−0.5
0
0.5
Heterogeneity: I^2 = 95%, τ^2 = 0.0252, p < 0.01

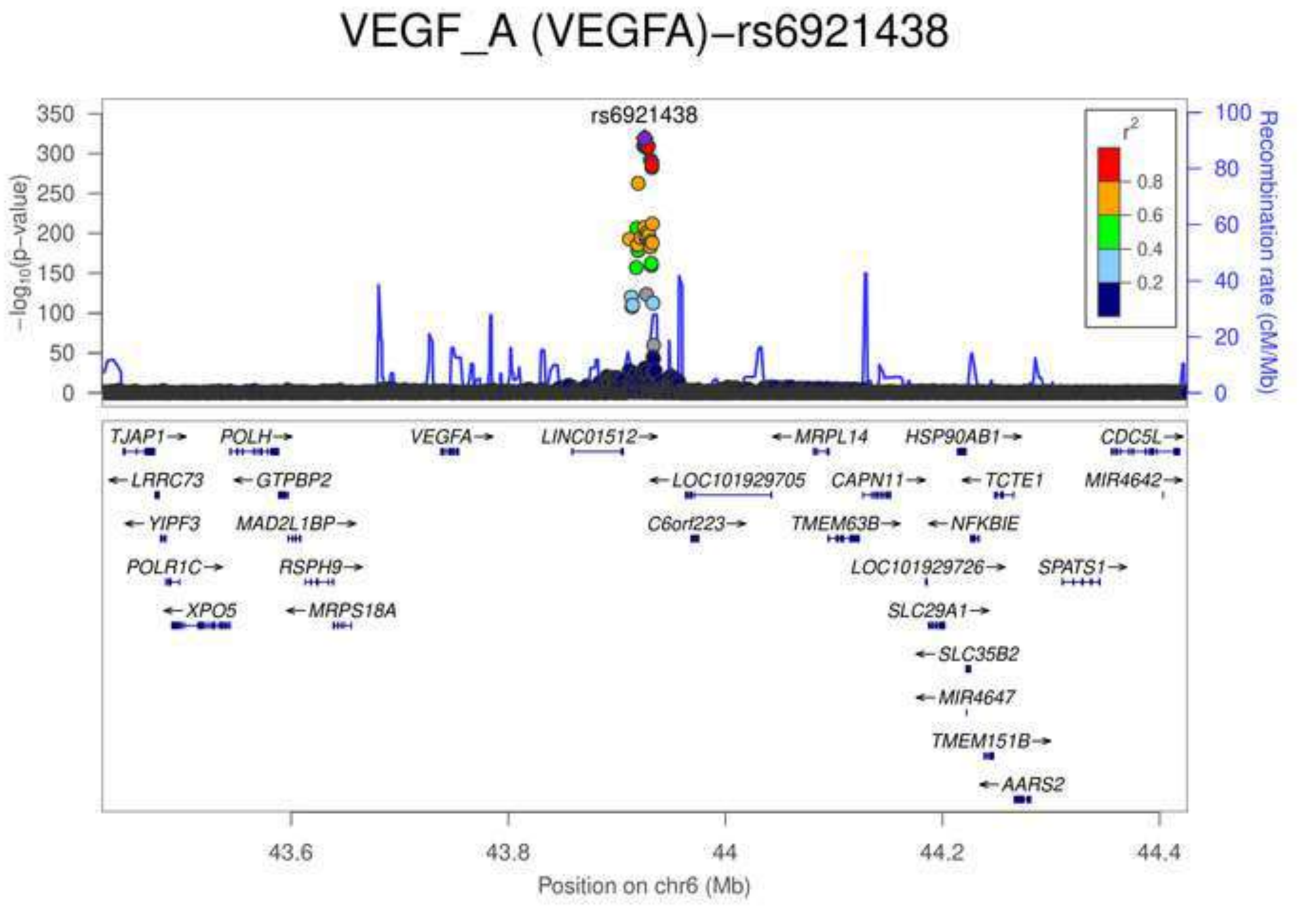
VEGF_A (VEGFA)−rs6921438
rs6921438
$-\log_{10}$(p−value)
Recombination rate (cM/Mb)
r^2
0.8
0.6
0.4
0.2
TJAP1
POLH
VEGFA
LINC01512
MRPL14
HSP90AB1
CDC5L
LRRC73
GTPBP2
LOC101929705
CAPN11
TCTE1
MIR4642
YIPF3
MAD2L1BP
C6orf223
TMEM63B
NFKBIE
POLR1C
RSPH9
LOC101929726
SPATS1
XPO5
MRPS18A
SLC29A1
SLC35B2
MIR4647
TMEM151B
AARS2
43.6
43.8
44
44.2
44.4
Position on chr6 (Mb)

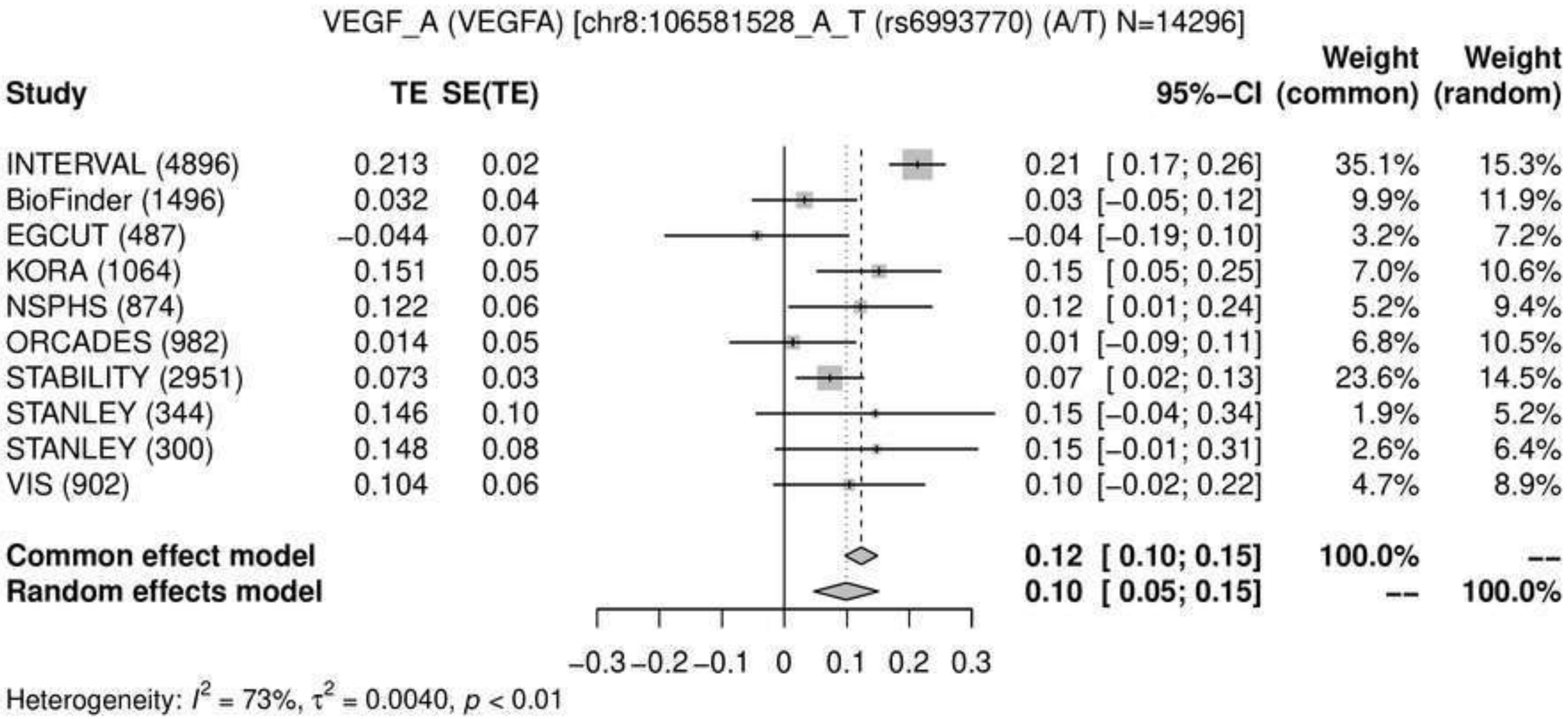
VEGF_A (VEGFA) [chr8:106581528_A_T (rs6993770) (A/T) N=14296]
Study
TE
SE(TE)
95%−CI
Weight (common)
Weight (random)
INTERVAL (4896) 0.213 0.02 0.21 [0.17; 0.26] 35.1% 15.3%
BioFinder (1496) 0.032 0.04 0.03 [−0.05; 0.12] 9.9% 11.9%
EGCUT (487) −0.044 0.07 −0.04 [−0.19; 0.10] 3.2% 7.2%
KORA (1064) 0.151 0.05 0.15 [0.05; 0.25] 7.0% 10.6%
NSPHS (874) 0.122 0.06 0.12 [0.01; 0.24] 5.2% 9.4%
ORCADES (982) 0.014 0.05 0.01 [−0.09; 0.11] 6.8% 10.5%
STABILITY (2951) 0.073 0.03 0.07 [0.02; 0.13] 23.6% 14.5%
STANLEY (344) 0.146 0.10 0.15 [−0.04; 0.34] 1.9% 5.2%
STANLEY (300) 0.148 0.08 0.15 [−0.01; 0.31] 2.6% 6.4%
VIS (902) 0.104 0.06 0.10 [−0.02; 0.22] 4.7% 8.9%
Common effect model 0.12 [0.10; 0.15] 100.0% --
Random effects model 0.10 [0.05; 0.15] -- 100.0%
−0.3 −0.2 −0.1 0 0.1 0.2 0.3
Heterogeneity: I^2 = 73%, τ^2 = 0.0040, p < 0.01

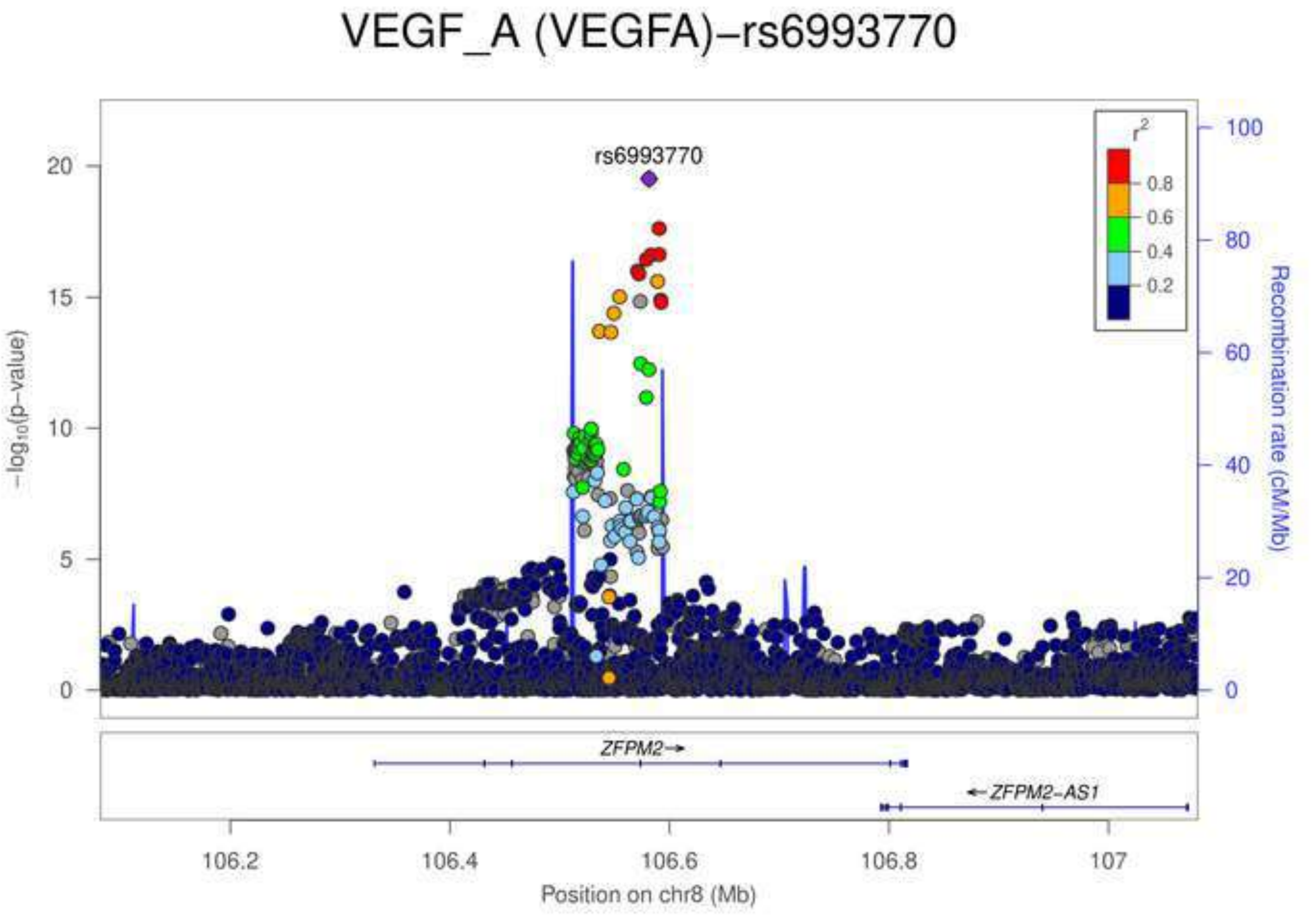
VEGF_A (VEGFA)−rs6993770
rs6993770
r^2
0.8
0.6
0.4
0.2
$-\log_{10}$(p−value)
Recombination rate (cM/Mb)
0
5
10
15
20
100
80
60
40
20
0
ZFPM2→
←ZFPM2−AS1
106.2
106.4
106.6
106.8
107
Position on chr8 (Mb)

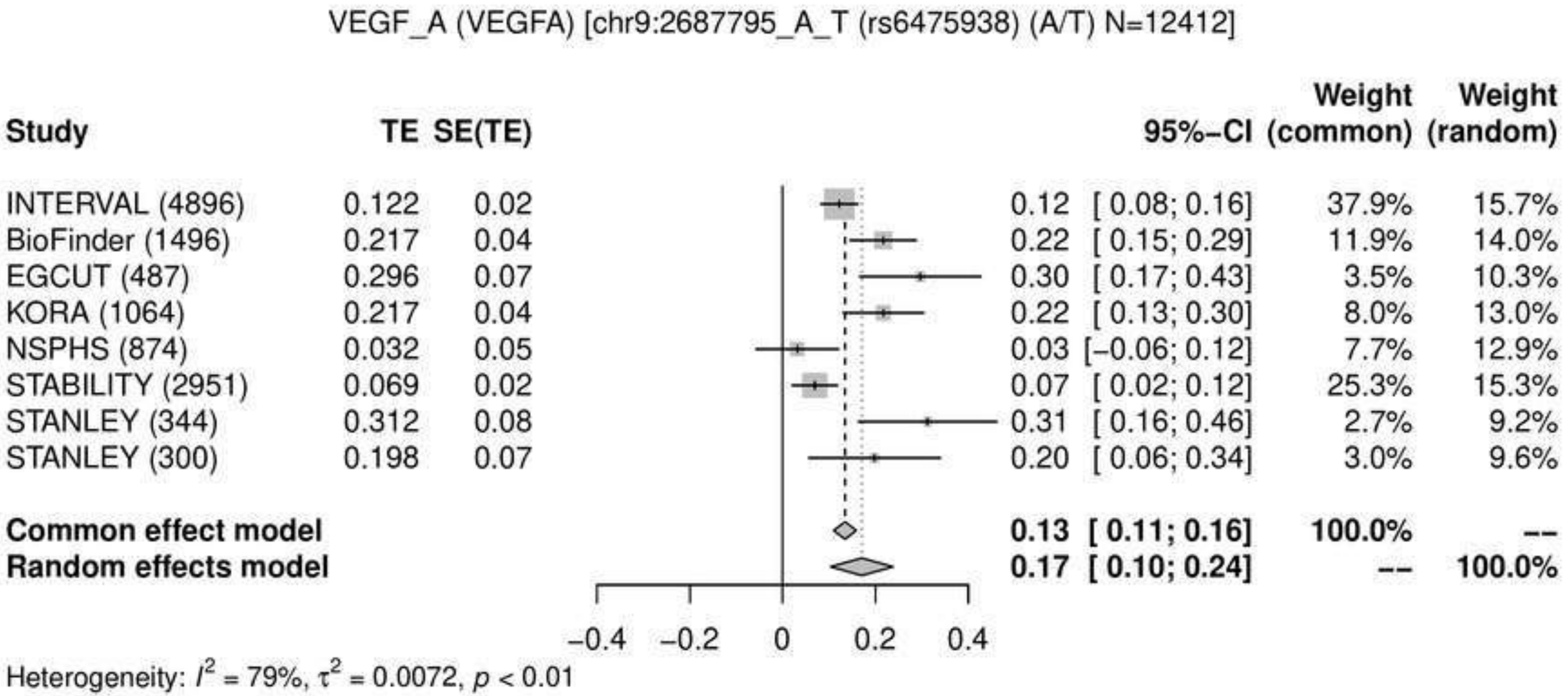
VEGF_A (VEGFA) [chr9:2687795_A_T (rs6475938) (A/T) N=12412]
Study TE SE(TE) 95%−CI Weight (common) Weight (random)
INTERVAL (4896) 0.122 0.02 0.12 [0.08; 0.16] 37.9% 15.7%
BioFinder (1496) 0.217 0.04 0.22 [0.15; 0.29] 11.9% 14.0%
EGCUT (487) 0.296 0.07 0.30 [0.17; 0.43] 3.5% 10.3%
KORA (1064) 0.217 0.04 0.22 [0.13; 0.30] 8.0% 13.0%
NSPHS (874) 0.032 0.05 0.03 [−0.06; 0.12] 7.7% 12.9%
STABILITY (2951) 0.069 0.02 0.07 [0.02; 0.12] 25.3% 15.3%
STANLEY (344) 0.312 0.08 0.31 [0.16; 0.46] 2.7% 9.2%
STANLEY (300) 0.198 0.07 0.20 [0.06; 0.34] 3.0% 9.6%
Common effect model 0.13 [0.11; 0.16] 100.0% --
Random effects model 0.17 [0.10; 0.24] -- 100.0%
−0.4 −0.2 0 0.2 0.4
Heterogeneity: I^2 = 79%, τ^2 = 0.0072, p < 0.01

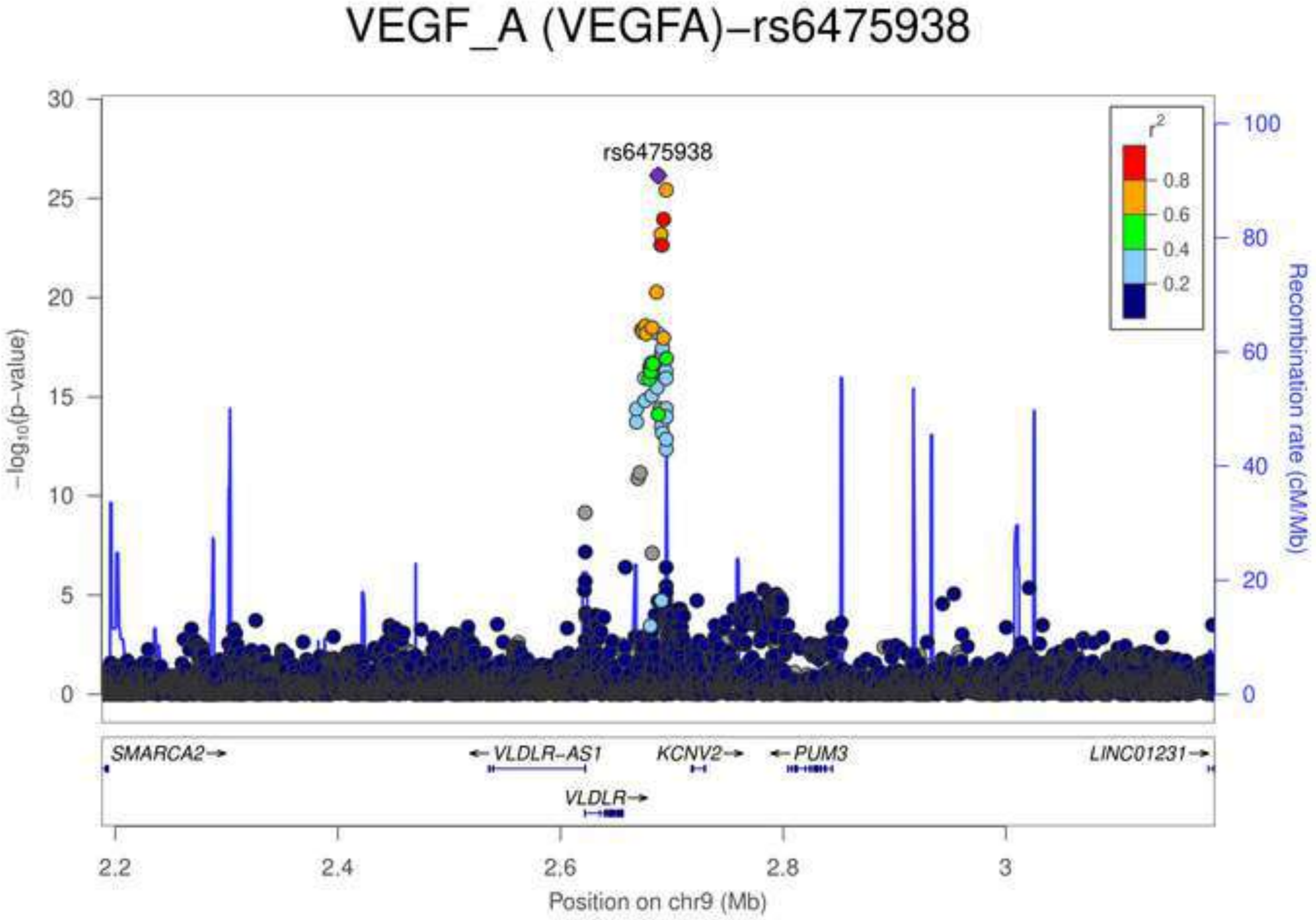
VEGF_A (VEGFA)−rs6475938
rs6475938
$-\log_{10}$(p−value)
Recombination rate (cM/Mb)
r^2
0.8
0.6
0.4
0.2
SMARCA2→
← VLDLR−AS1
KCNV2→
← PUM3
LINC01231→
VLDLR→
2.2
2.4
2.6
2.8
3
Position on chr9 (Mb)

Supplementary Figure 3. Regional association plots comparing plasma *cis*-pQTLs and blood *cis*-eQTLs for the gene encoding the corresponding protein. 'LocusZoom' regional association plots comparing the plasma *cis*-pQTL signals (lower panel) with the eQTL signals for the encoding gene in whole blood from the eQTLGen data (upper panel). The sentinel variant for the pQTL is coloured in purple. LD (r^2) to the sentinel pQTL is indicated by the colour key. Two-sided *P*-values are from meta-analysis of linear regression estimates.

eQTLGen: ADA (ADA)−rs11555566

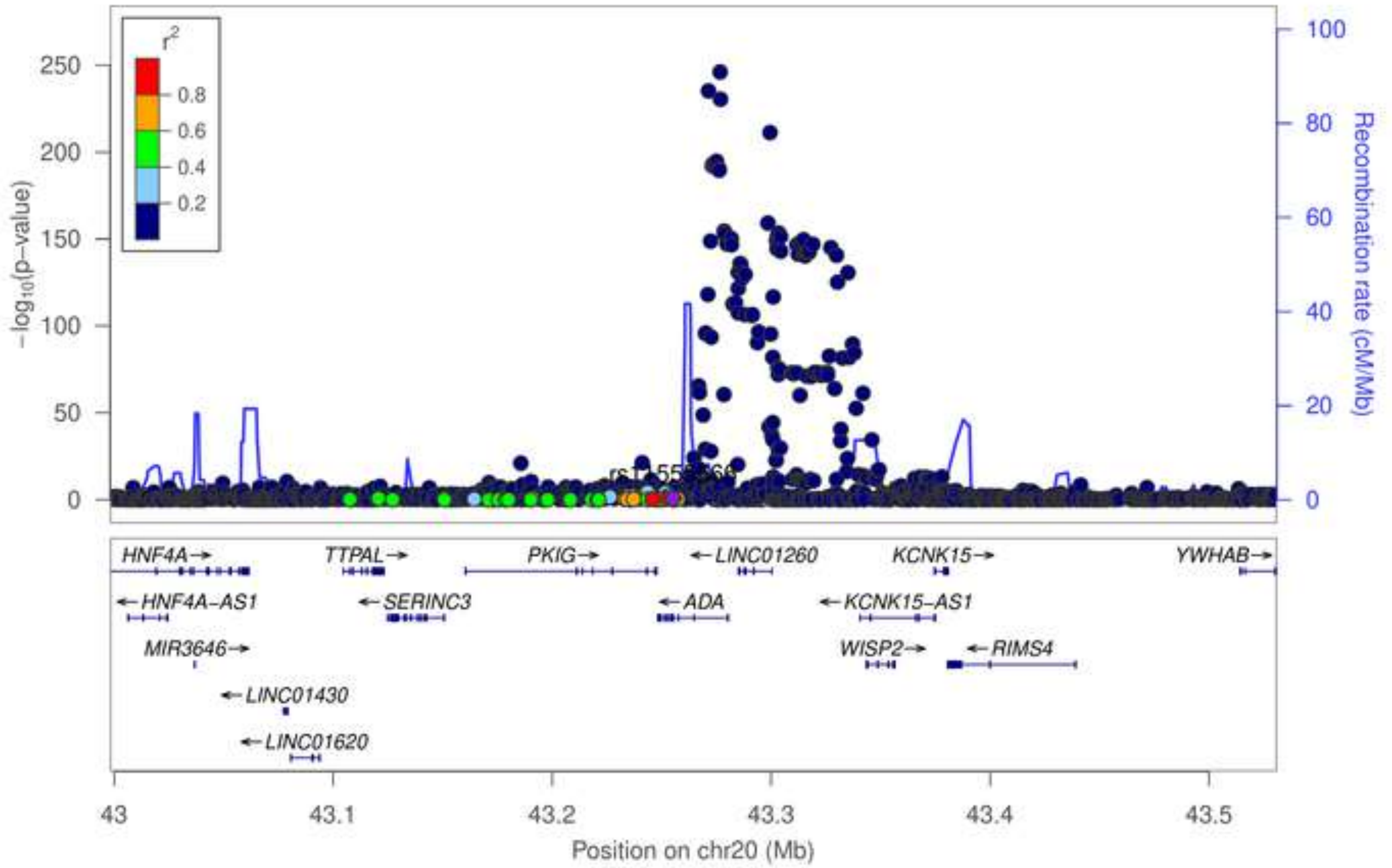

SCALLOP: ADA (ADA)−rs11555566

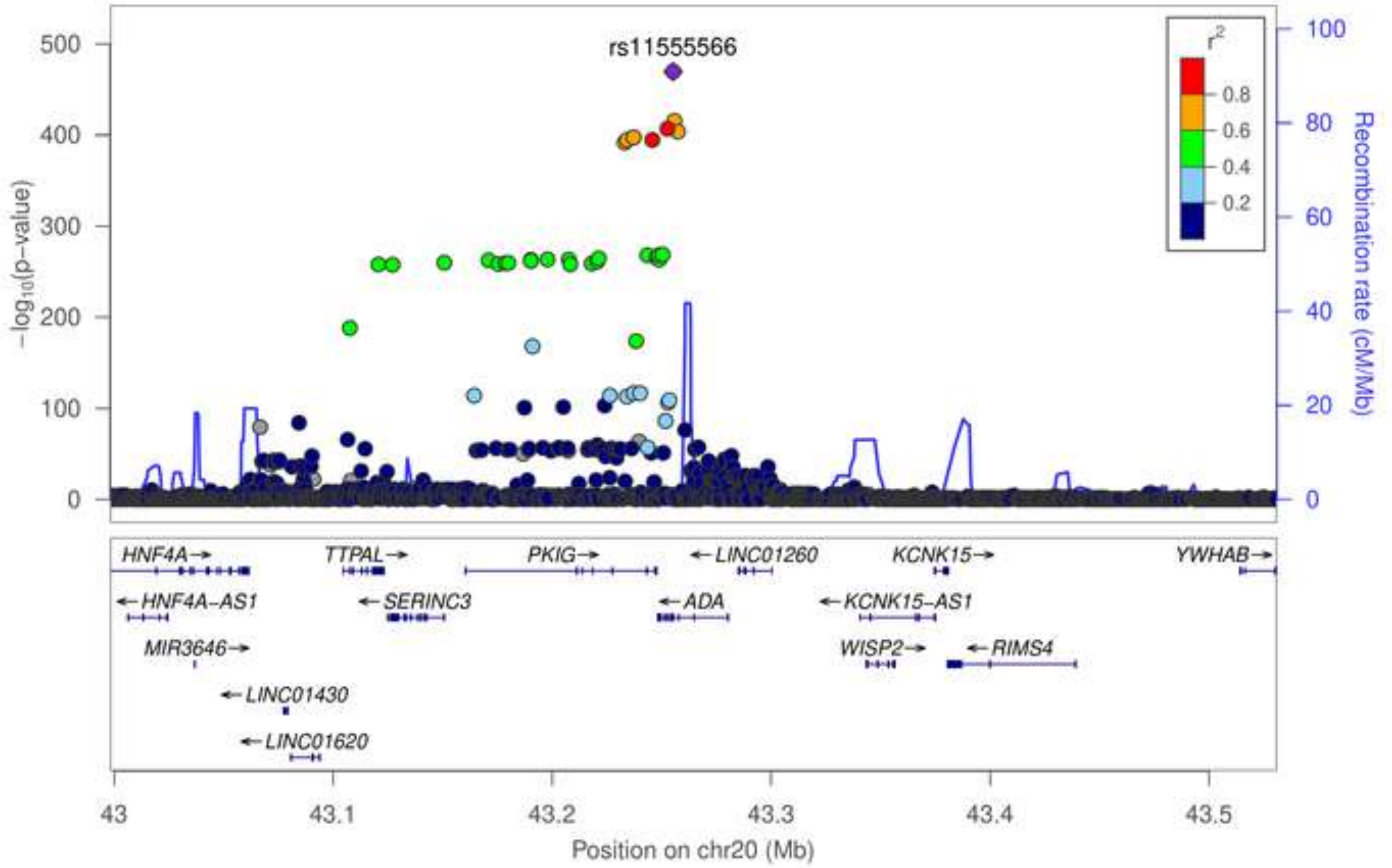

eQTLGen: CASP−8 (CASP8)−rs56328050

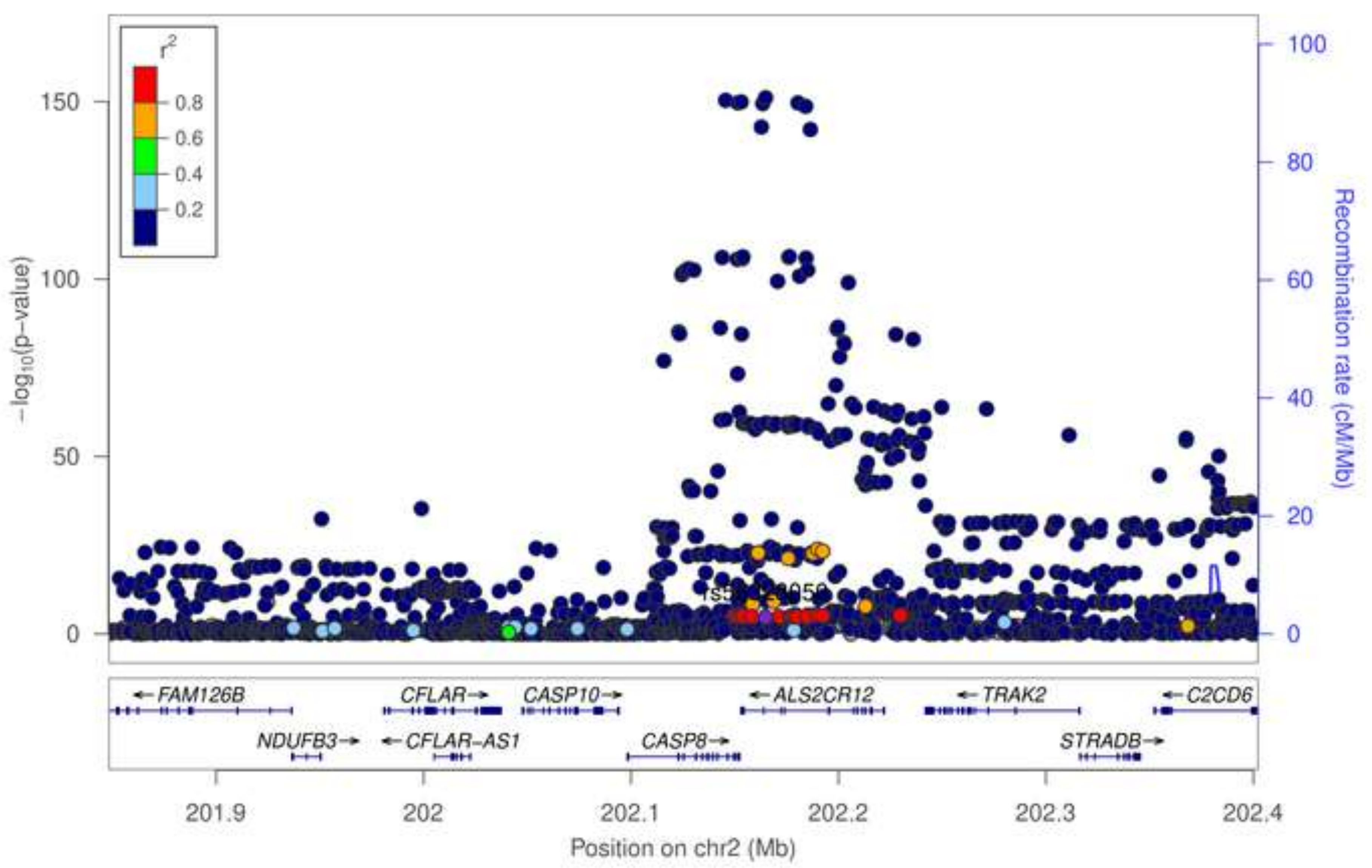

SCALLOP: CASP−8 (CASP8)−rs56328050

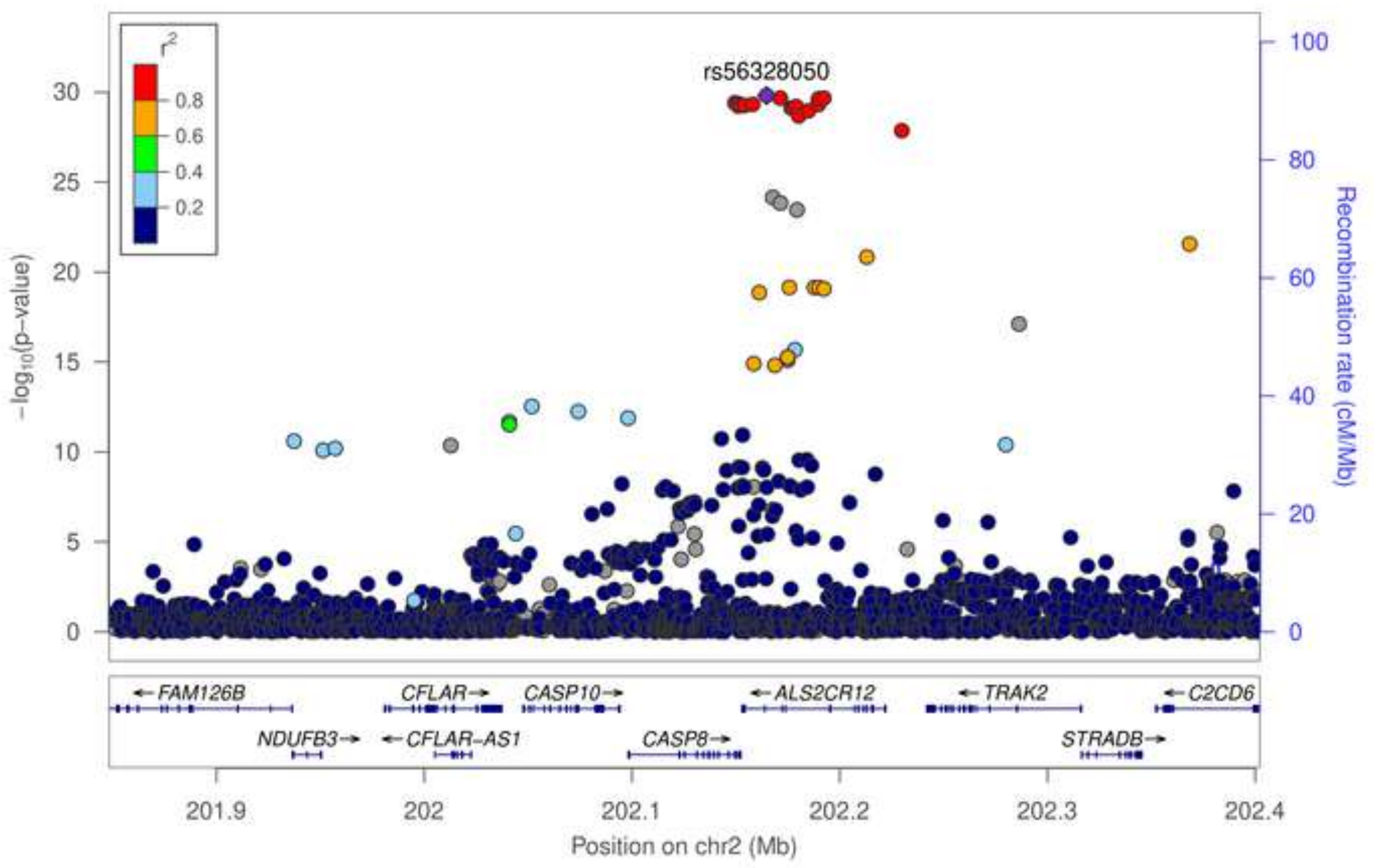

eQTLGen: CCL23 (CCL23)−rs712048

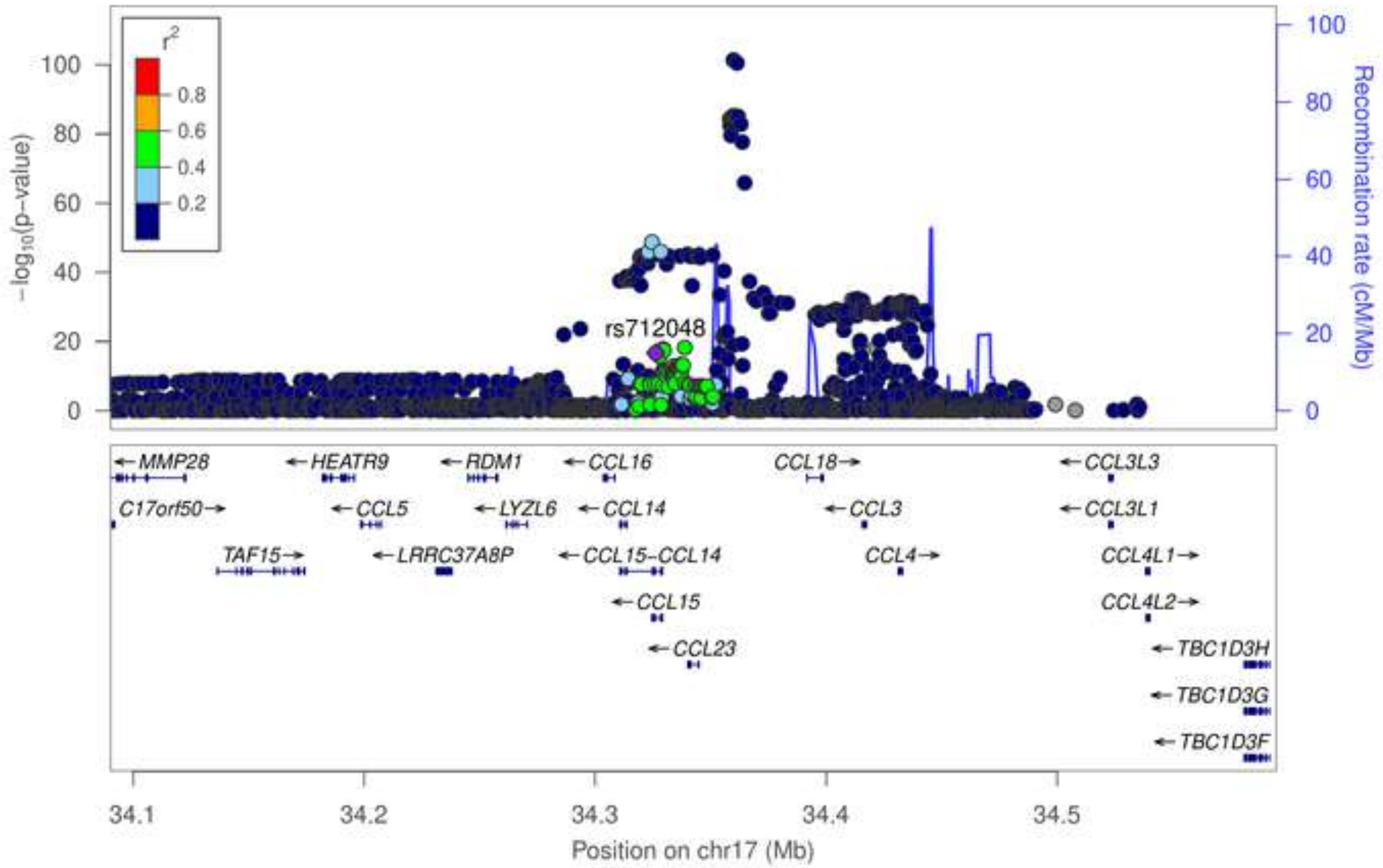

SCALLOP: CCL23 (CCL23)−rs712048

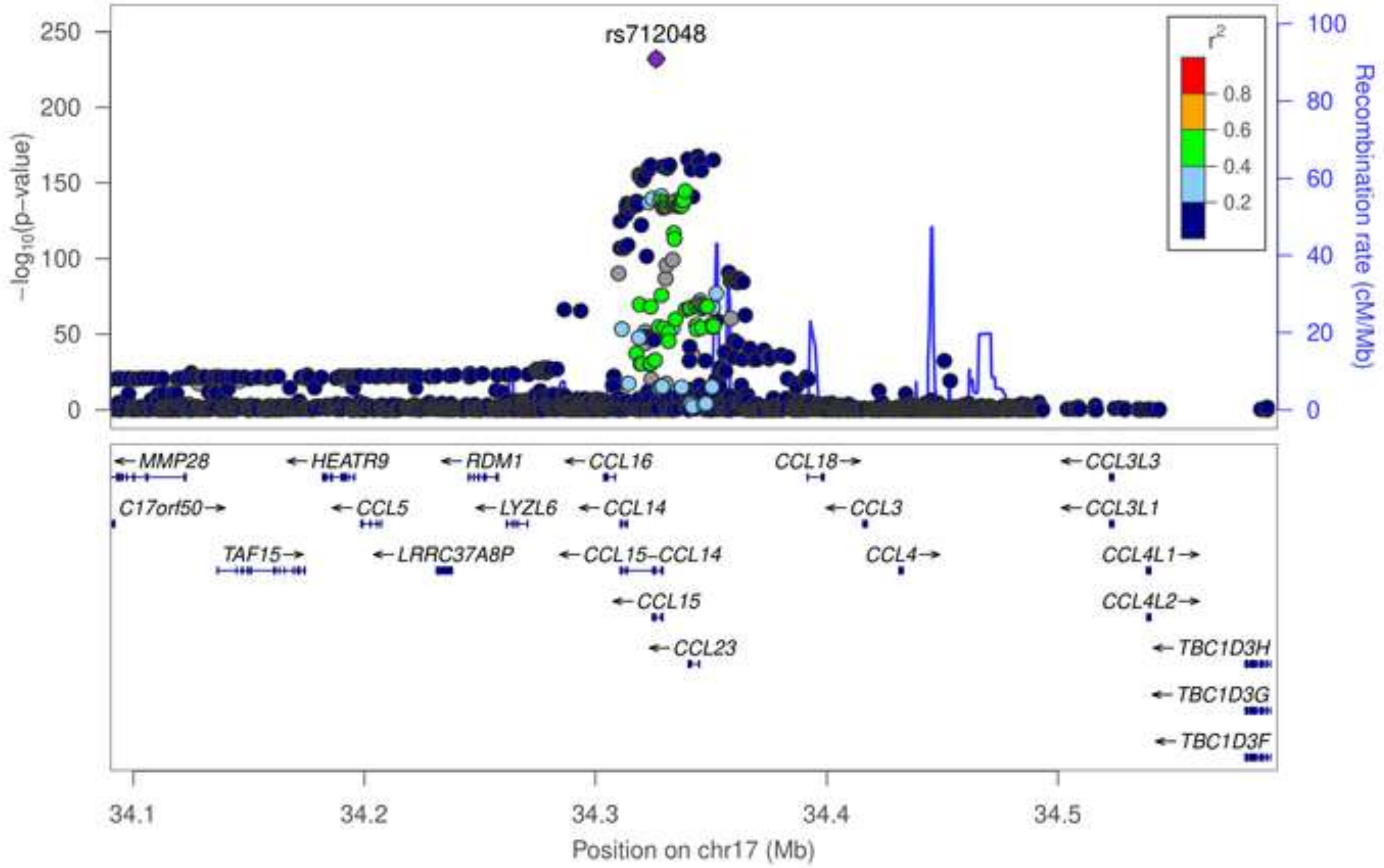

eQTLGen: CCL25 (CCL25)–rs2032887

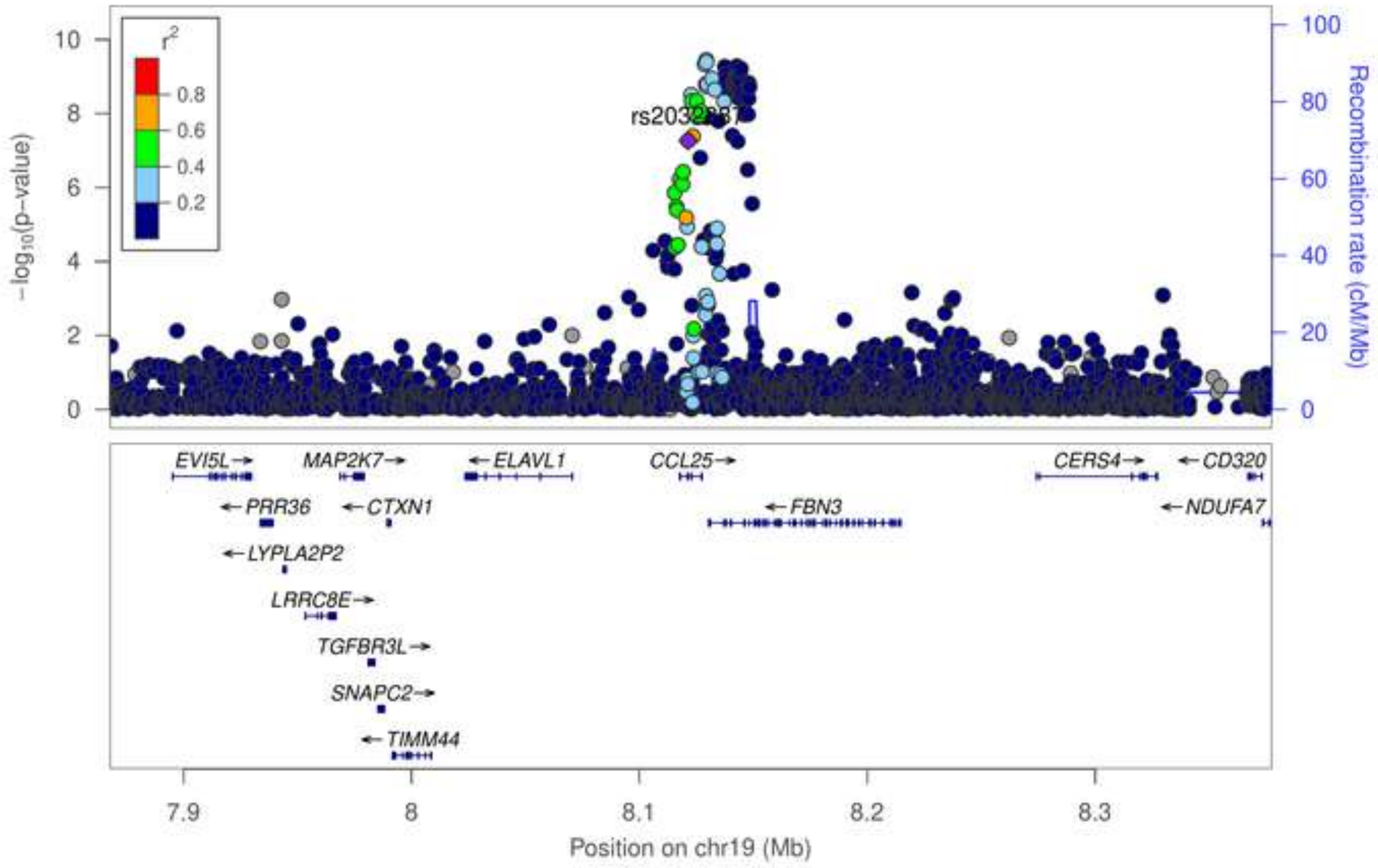

SCALLOP: CCL25 (CCL25)–rs2032887

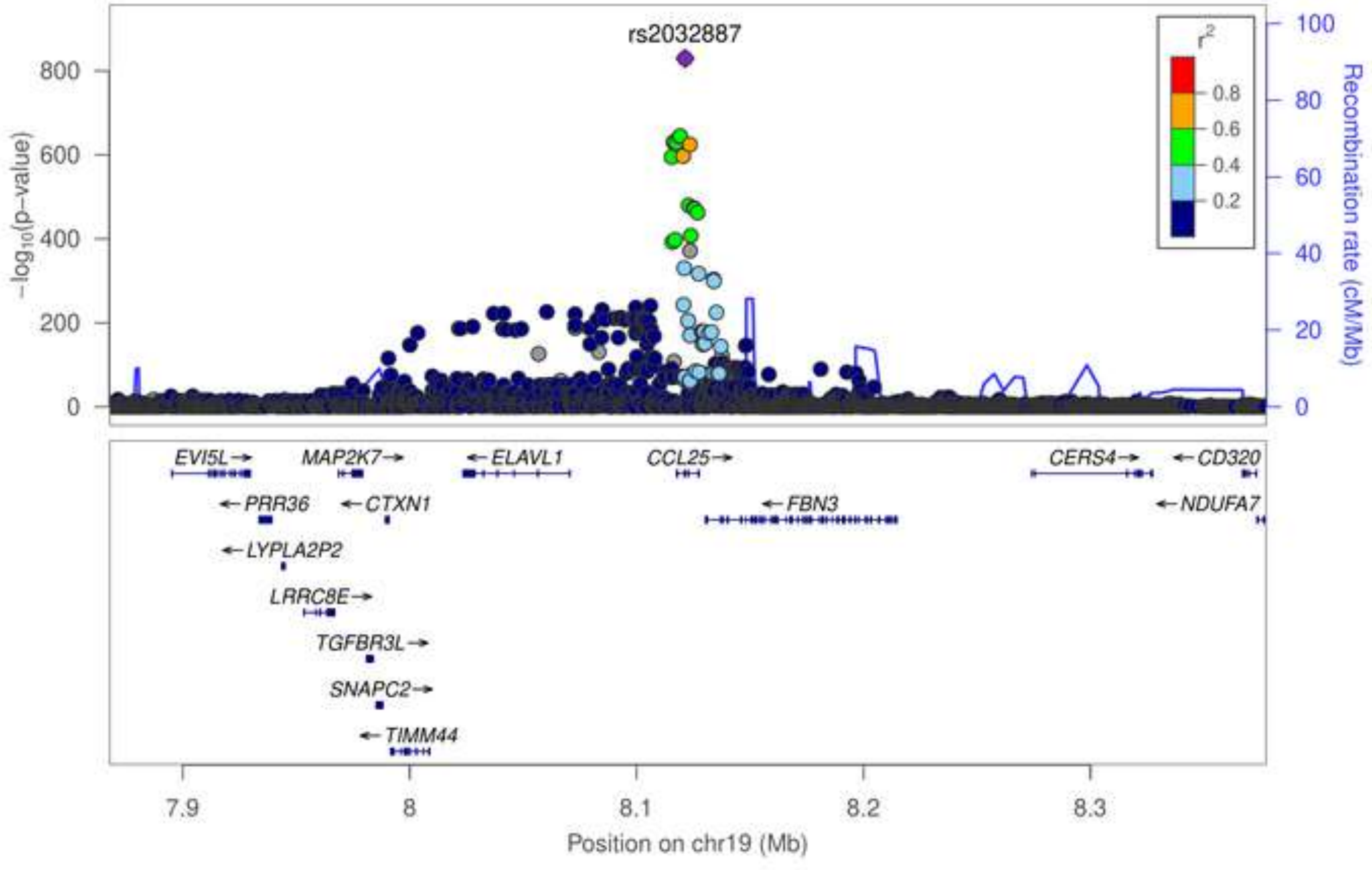

eQTLGen: CCL4 (CCL4)−rs8064426

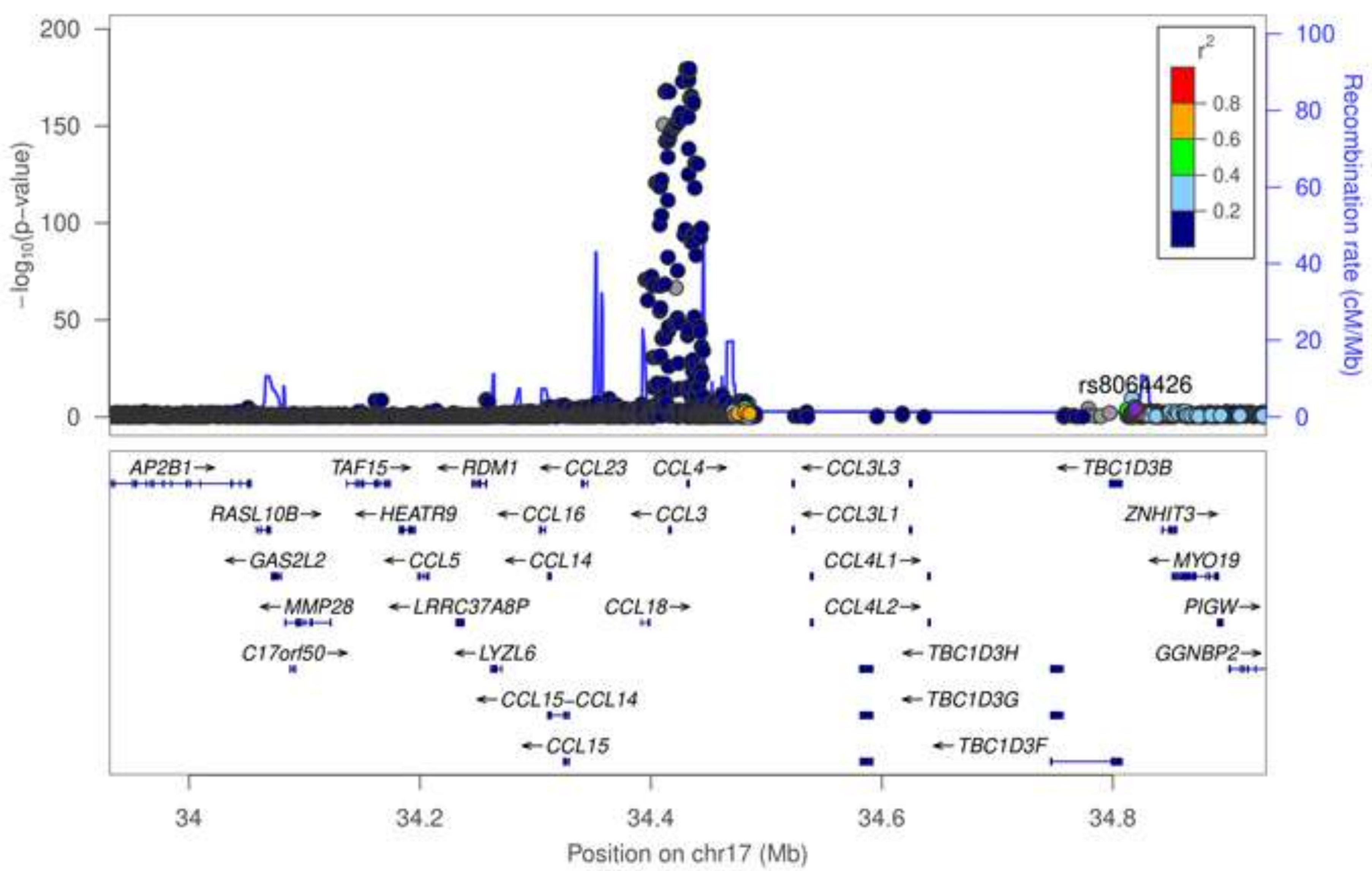

SCALLOP: CCL4 (CCL4)−rs8064426

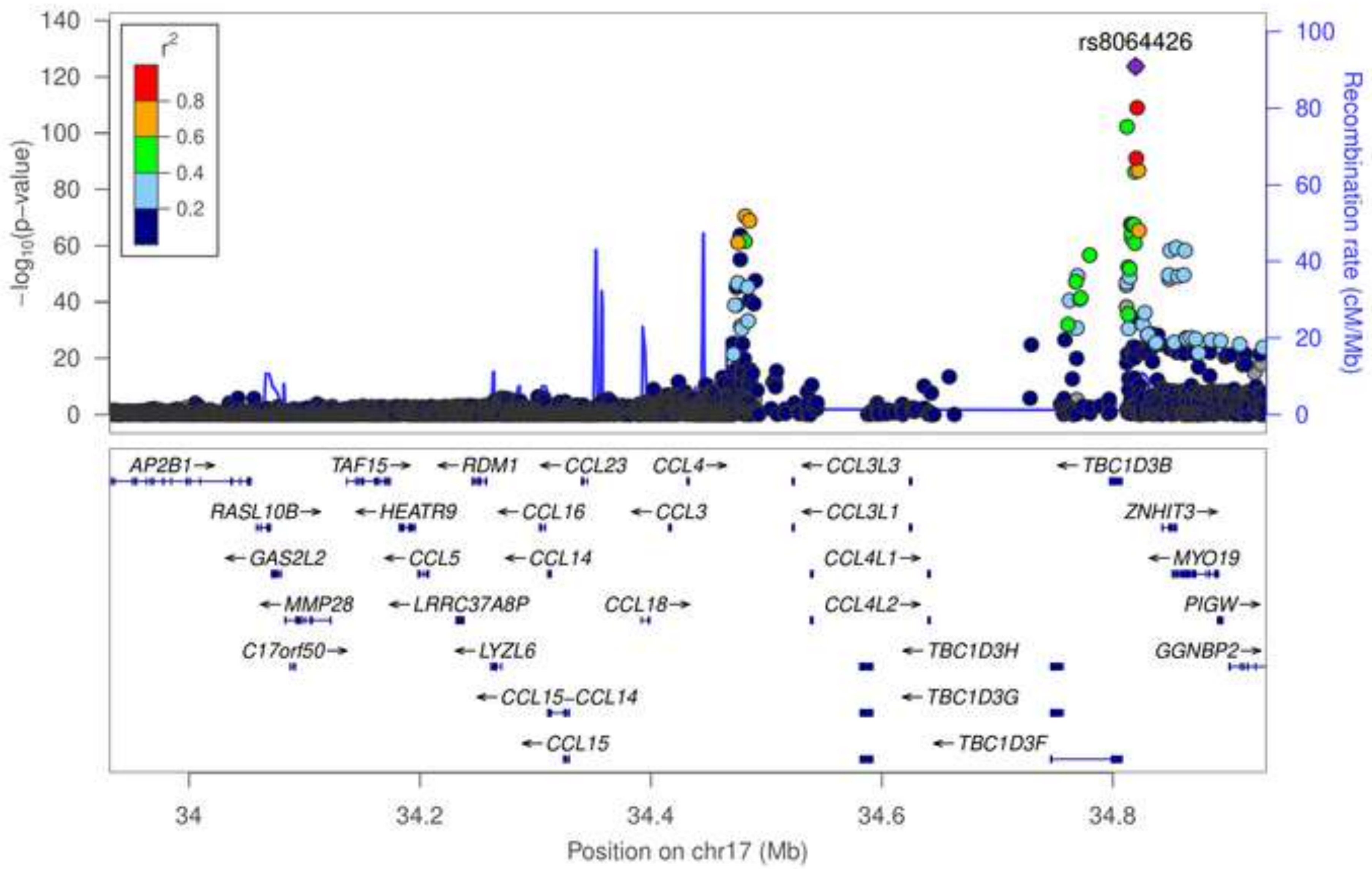

eQTLGen: CD244 (CD244)−rs11265493

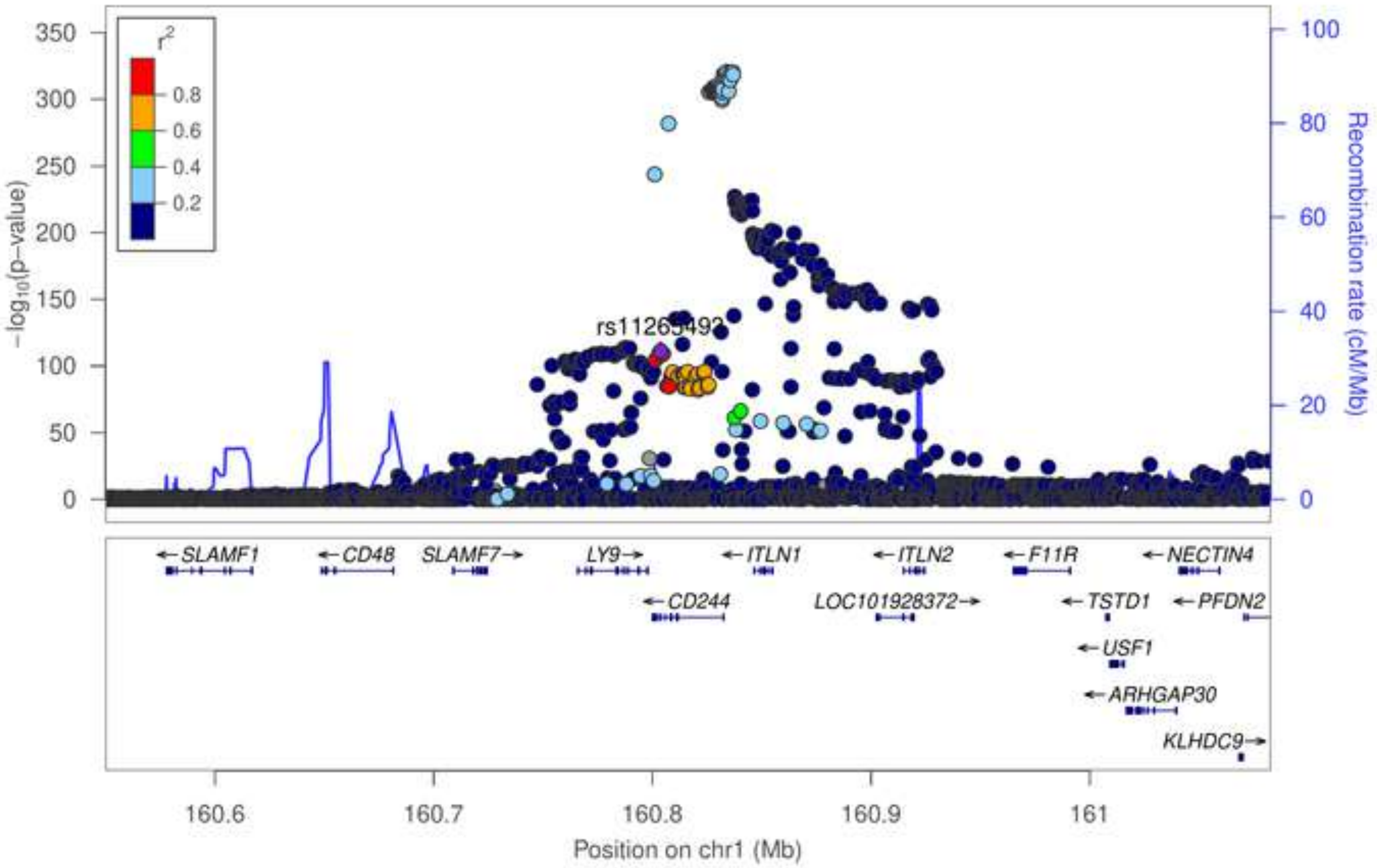

SCALLOP: CD244 (CD244)−rs11265493

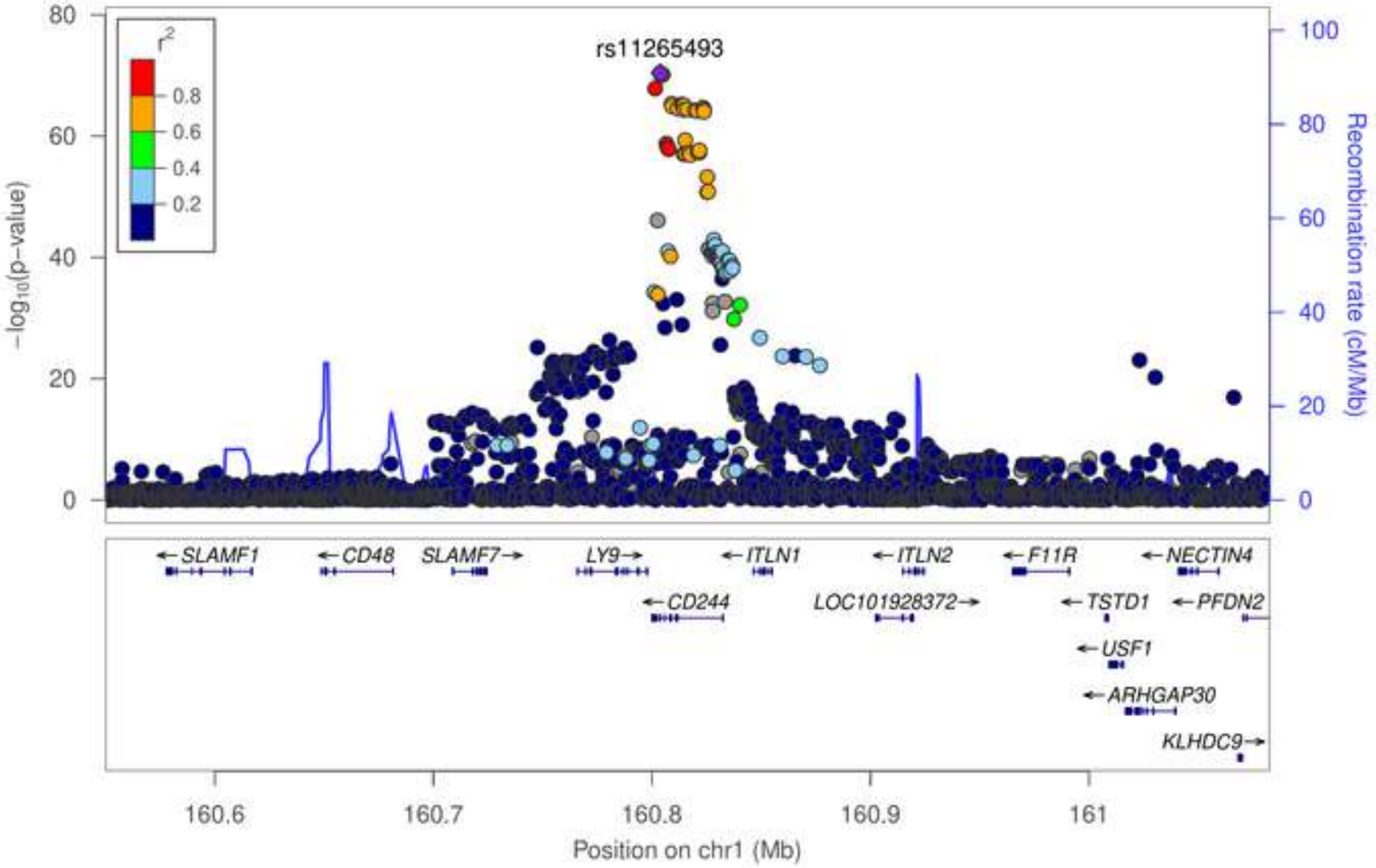

eQTLGen: CD40 (CD40)−rs1883832

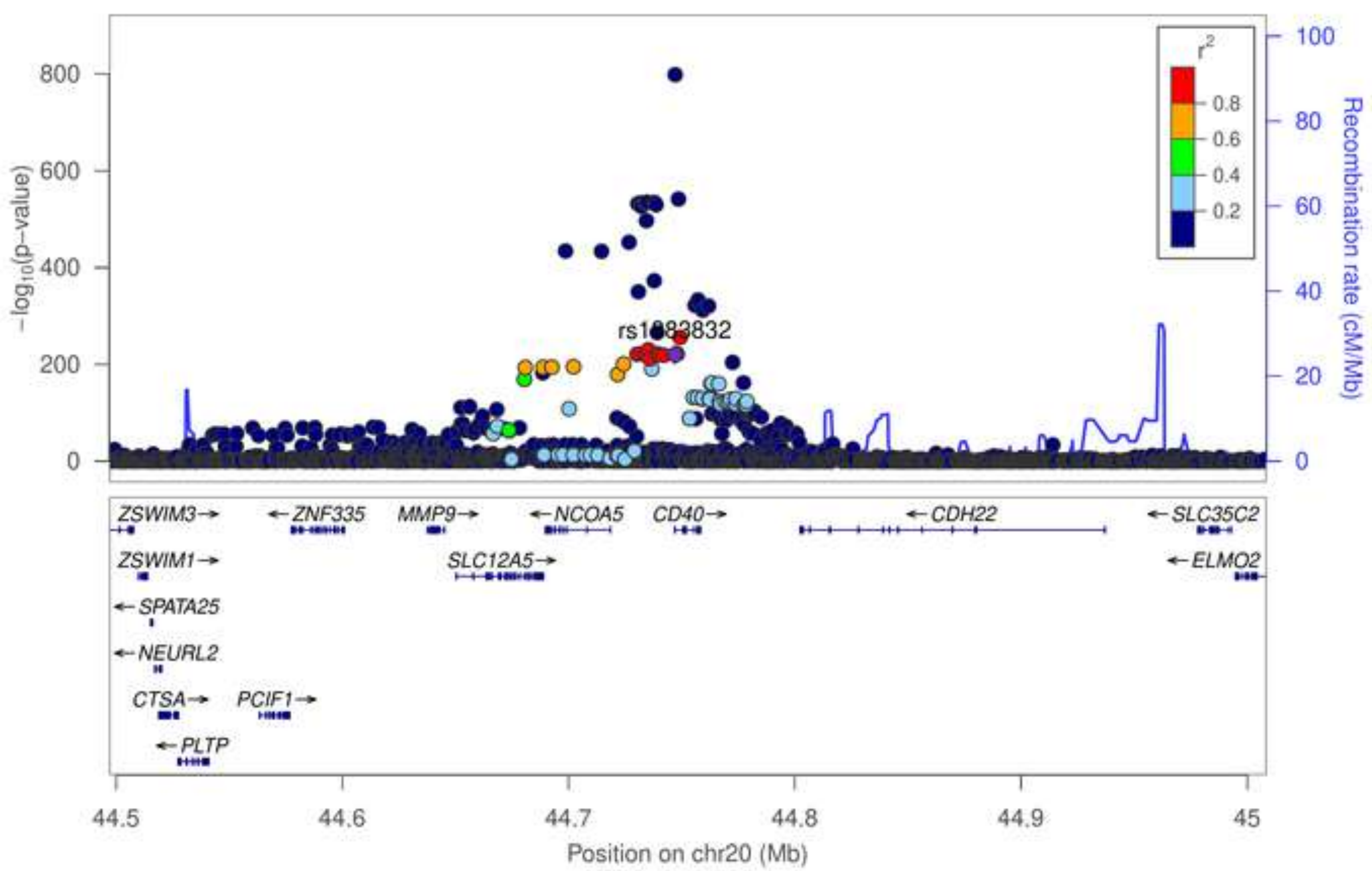

SCALLOP: CD40 (CD40)−rs1883832

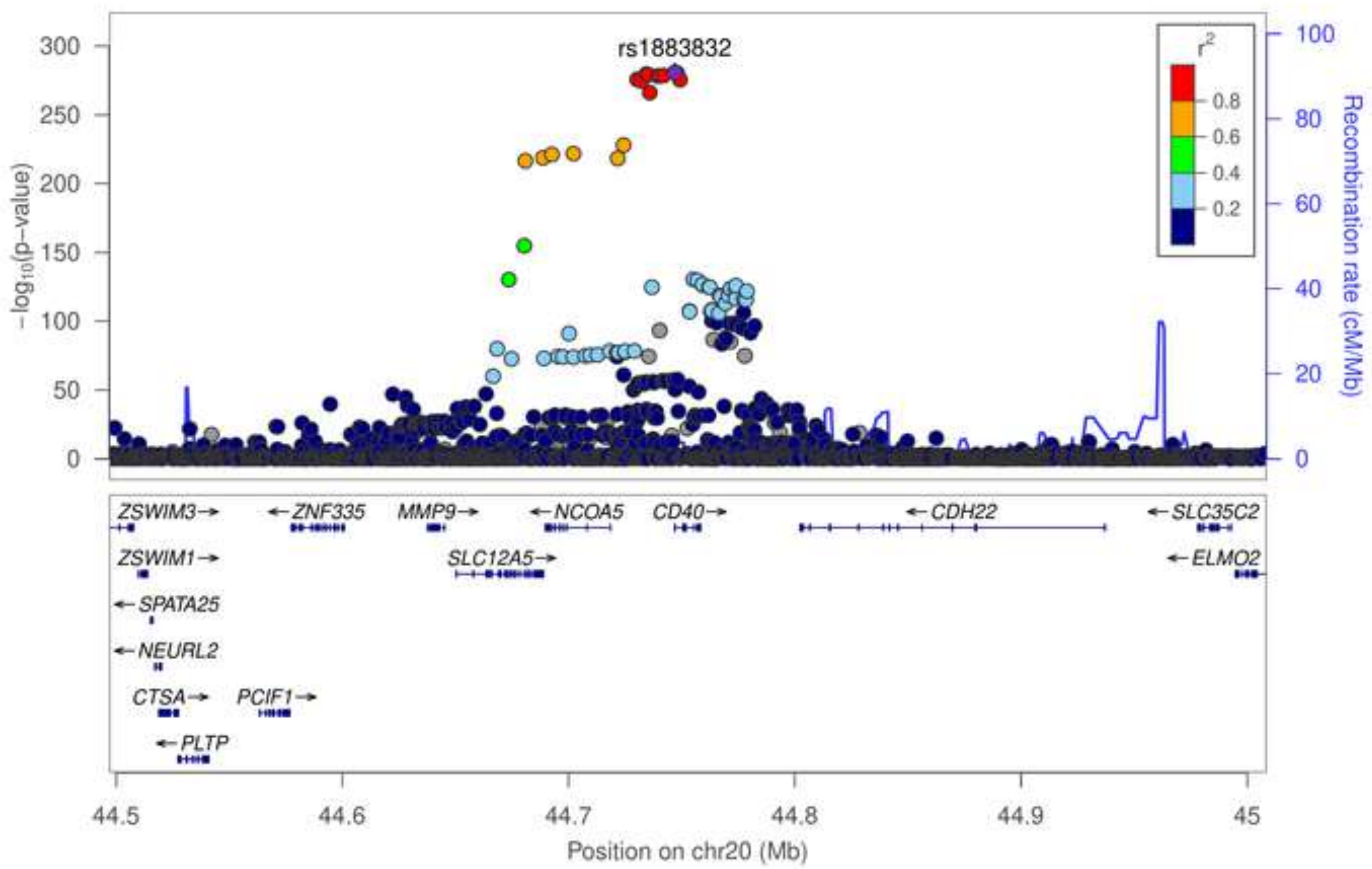

eQTLGen: CD5 (CD5)–rs674379

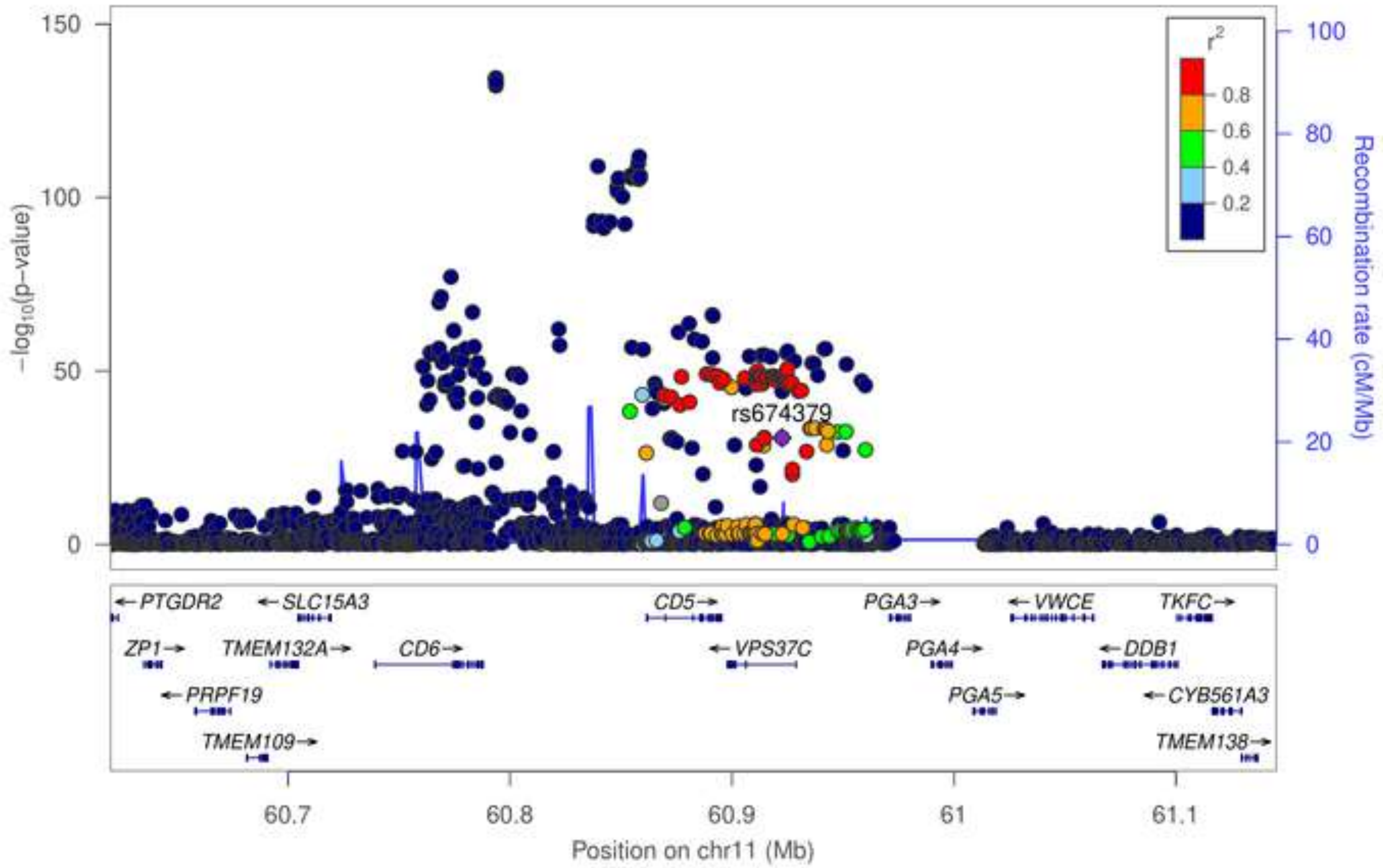

SCALLOP: CD5 (CD5)–rs674379

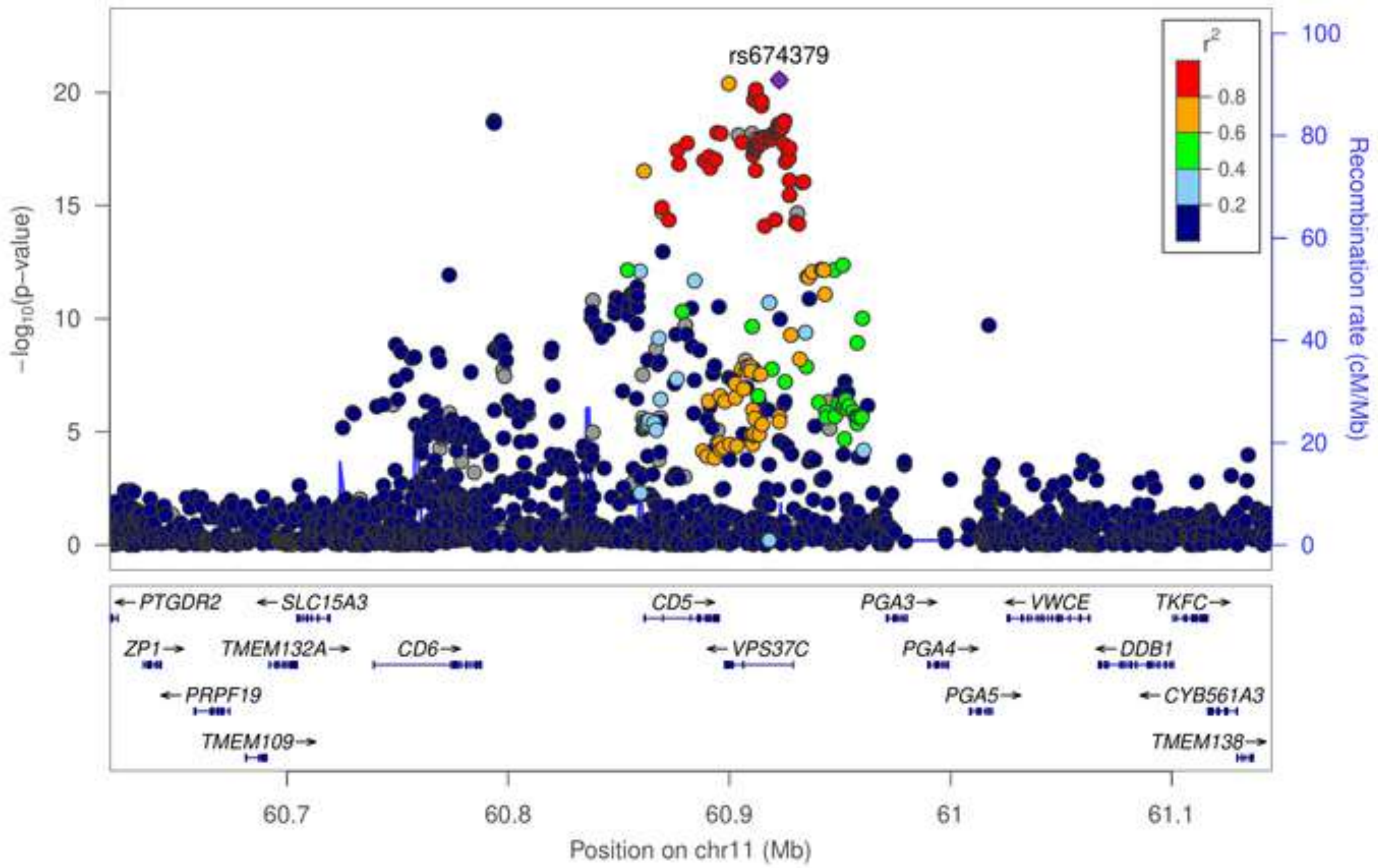

eQTLGen: CD6 (CD6)−rs2074227

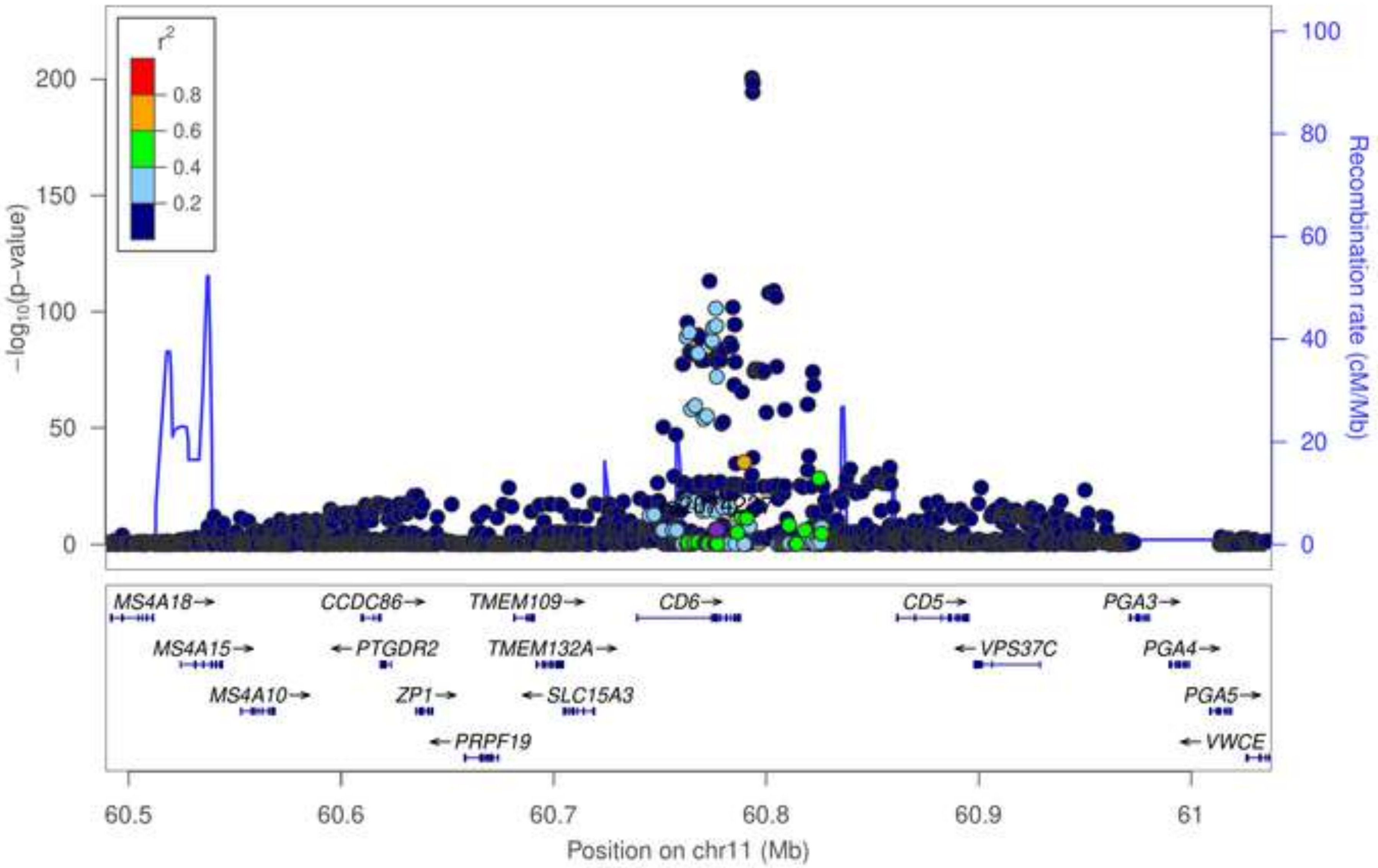

SCALLOP: CD6 (CD6)−rs2074227

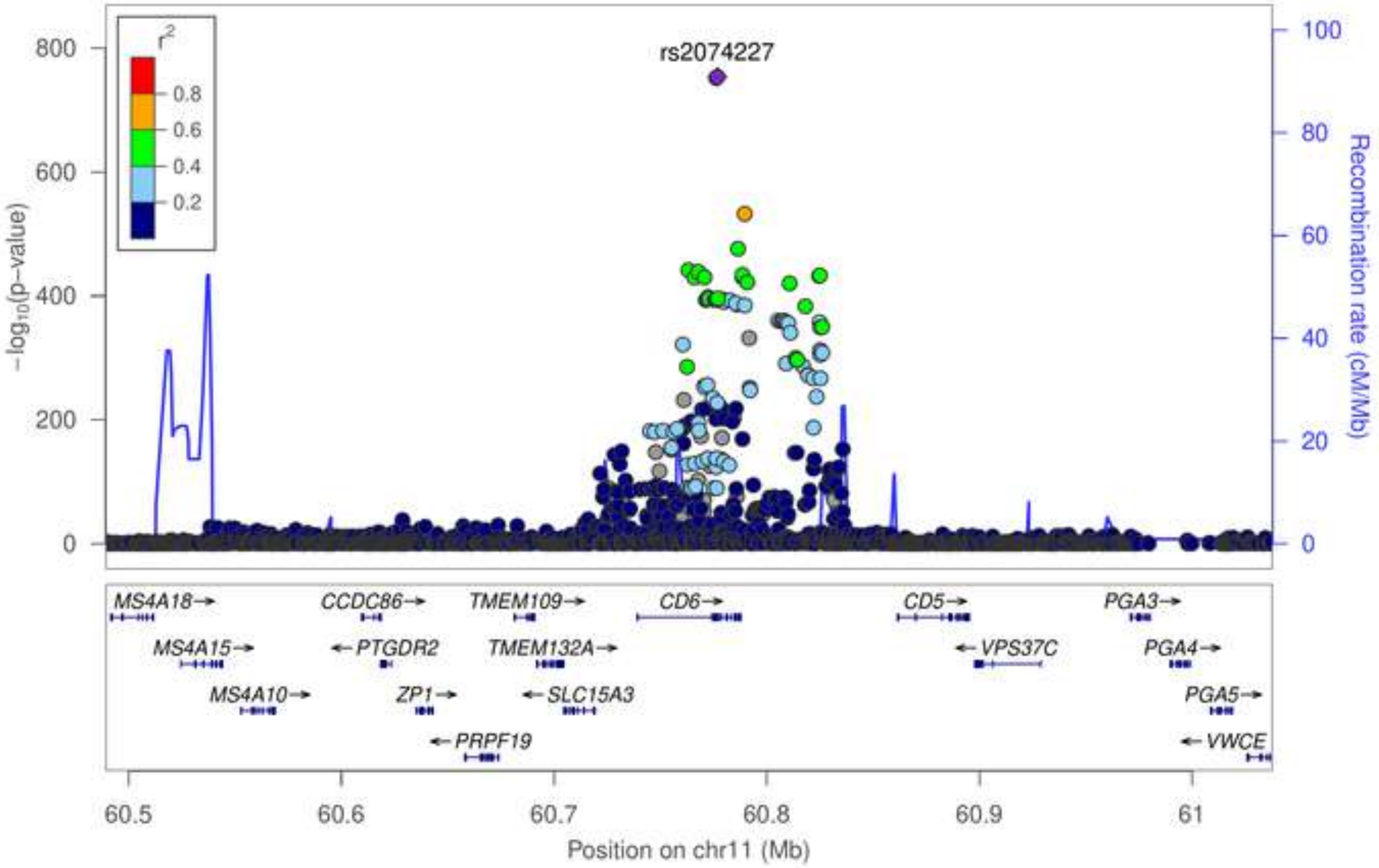

eQTLGen: CDCP1 (CDCP1)–rs2276862

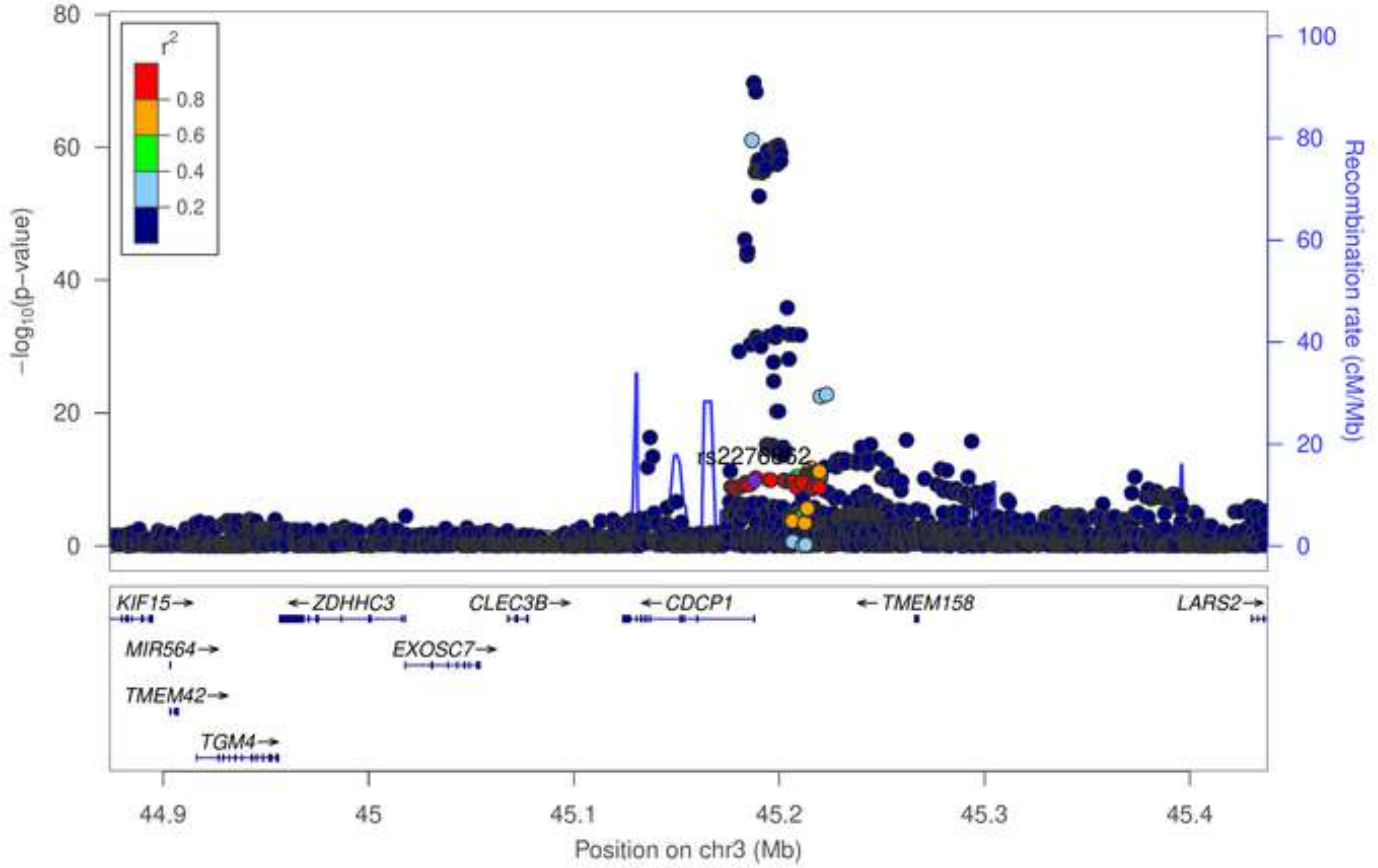

SCALLOP: CDCP1 (CDCP1)–rs2276862

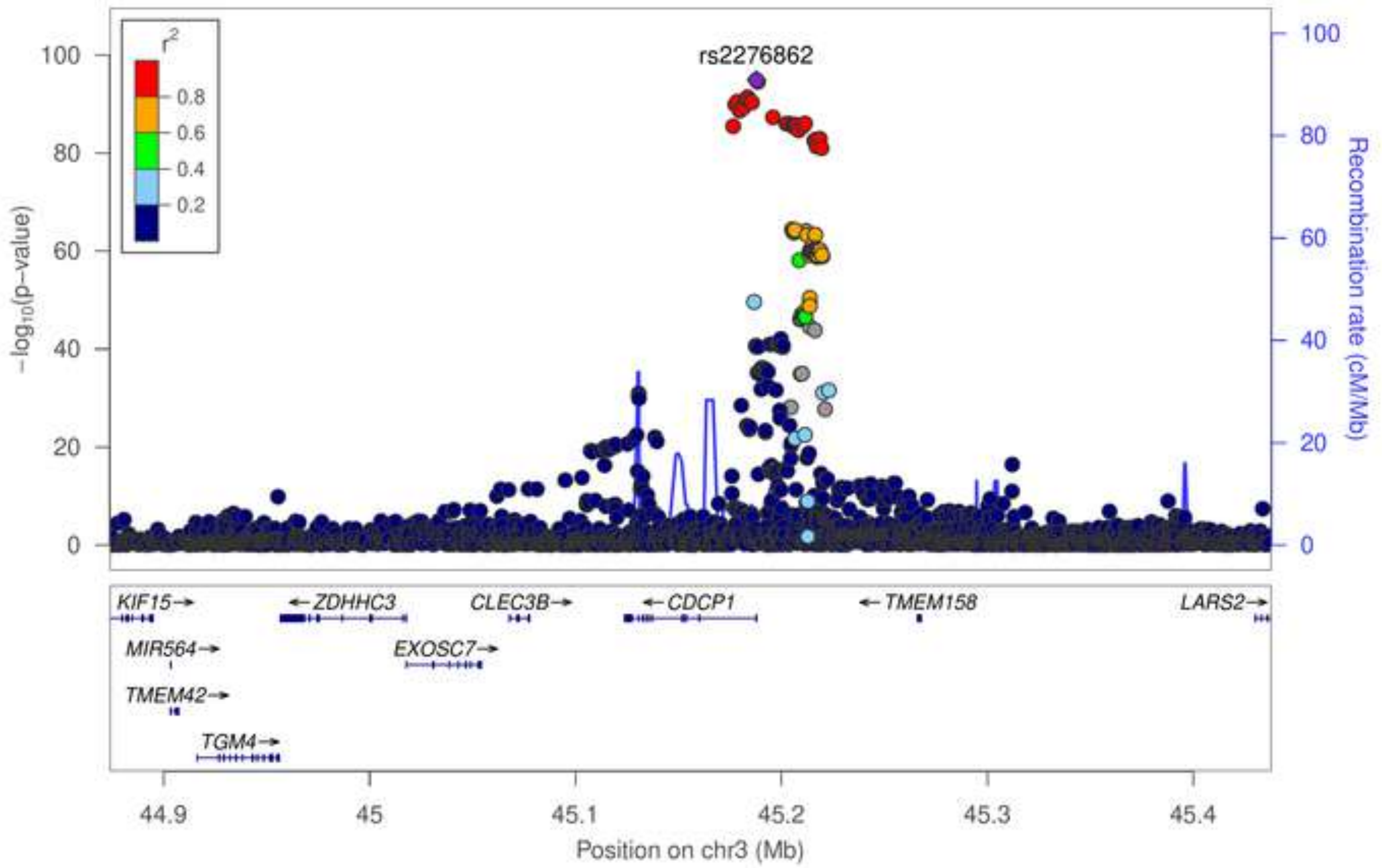

eQTLGen: CSF−1 (CSF1)−rs17610659

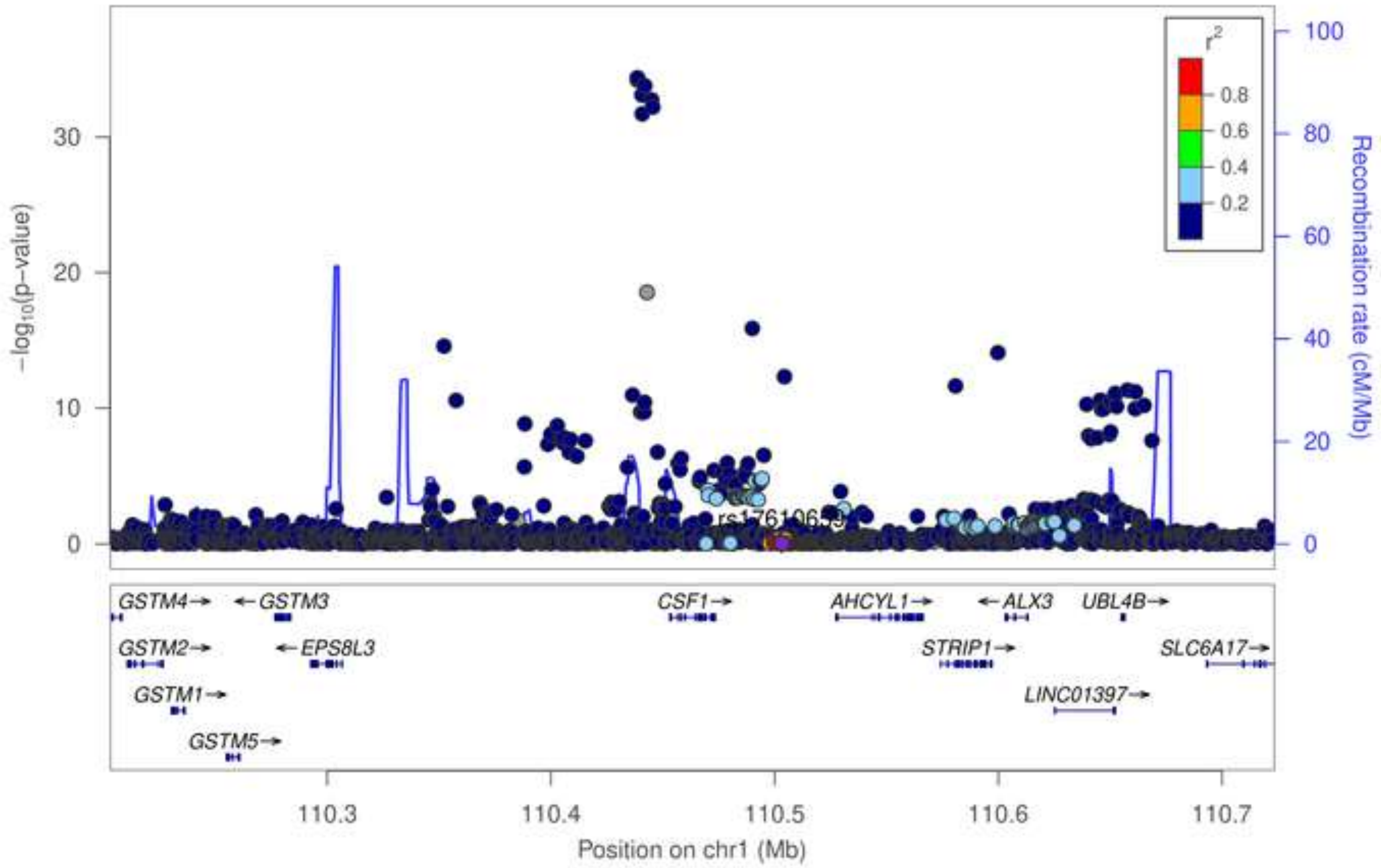

SCALLOP: CSF−1 (CSF1)−rs17610659

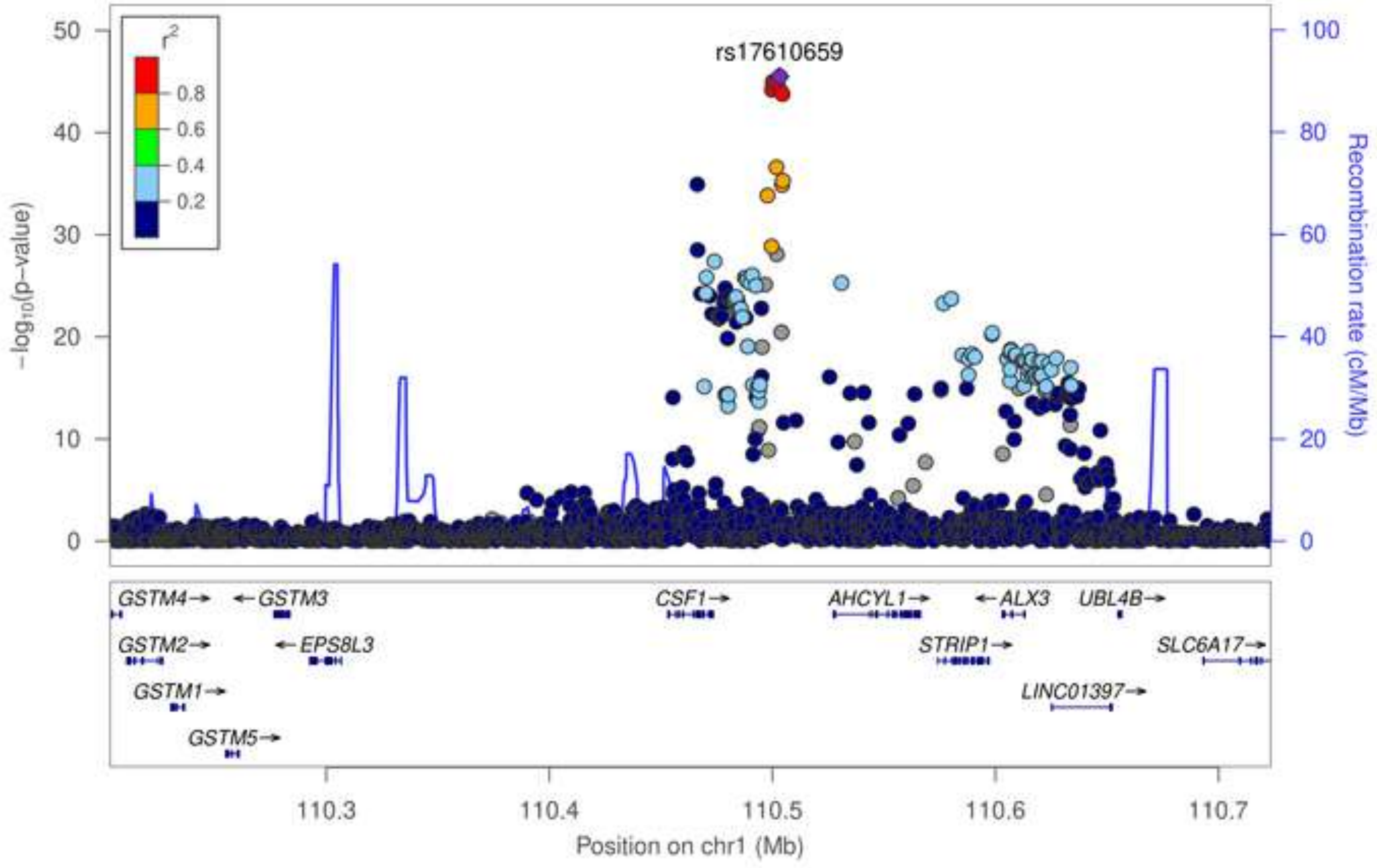

eQTLGen: CXCL10 (CXCL10)−rs11548618

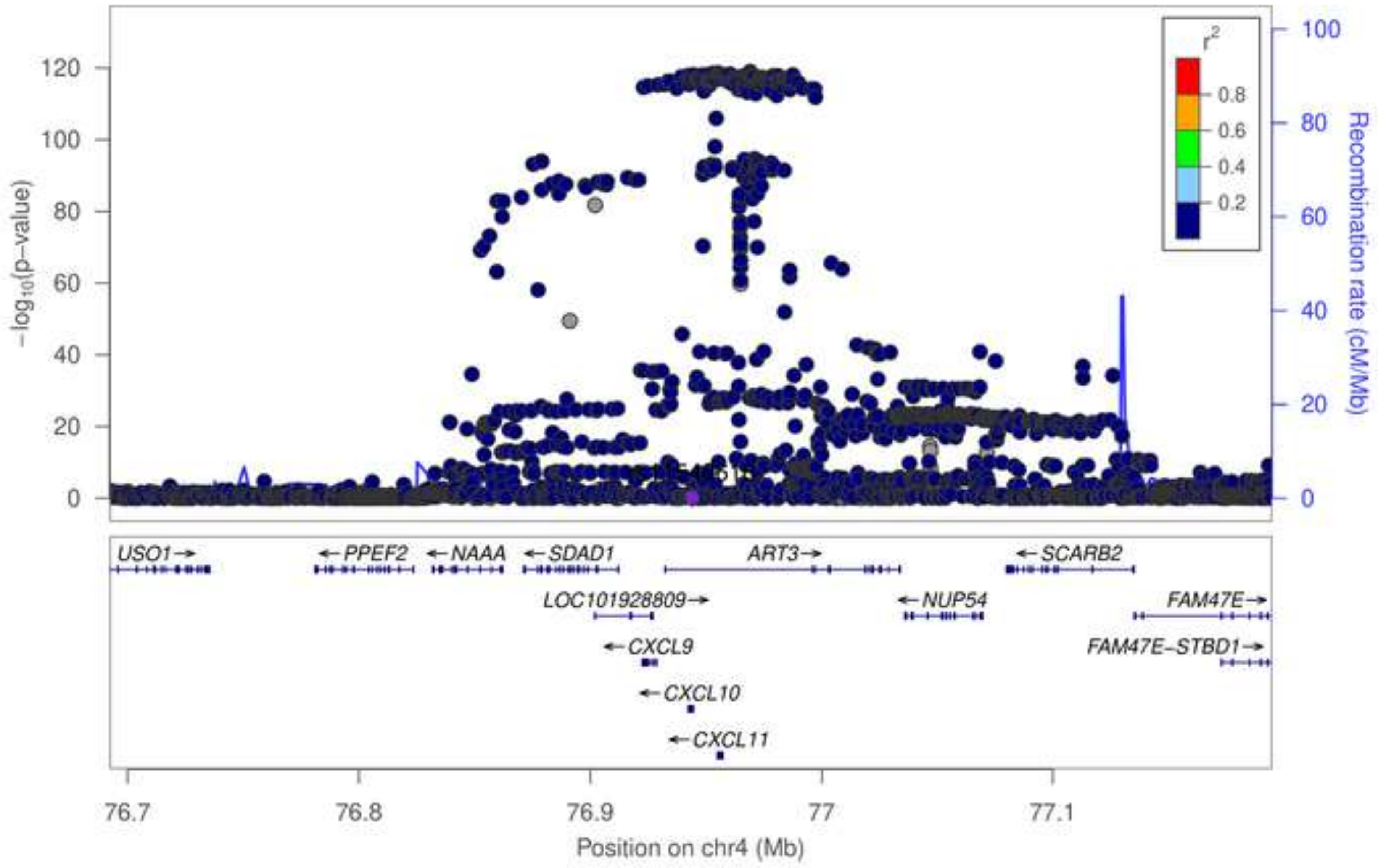

SCALLOP: CXCL10 (CXCL10)−rs11548618

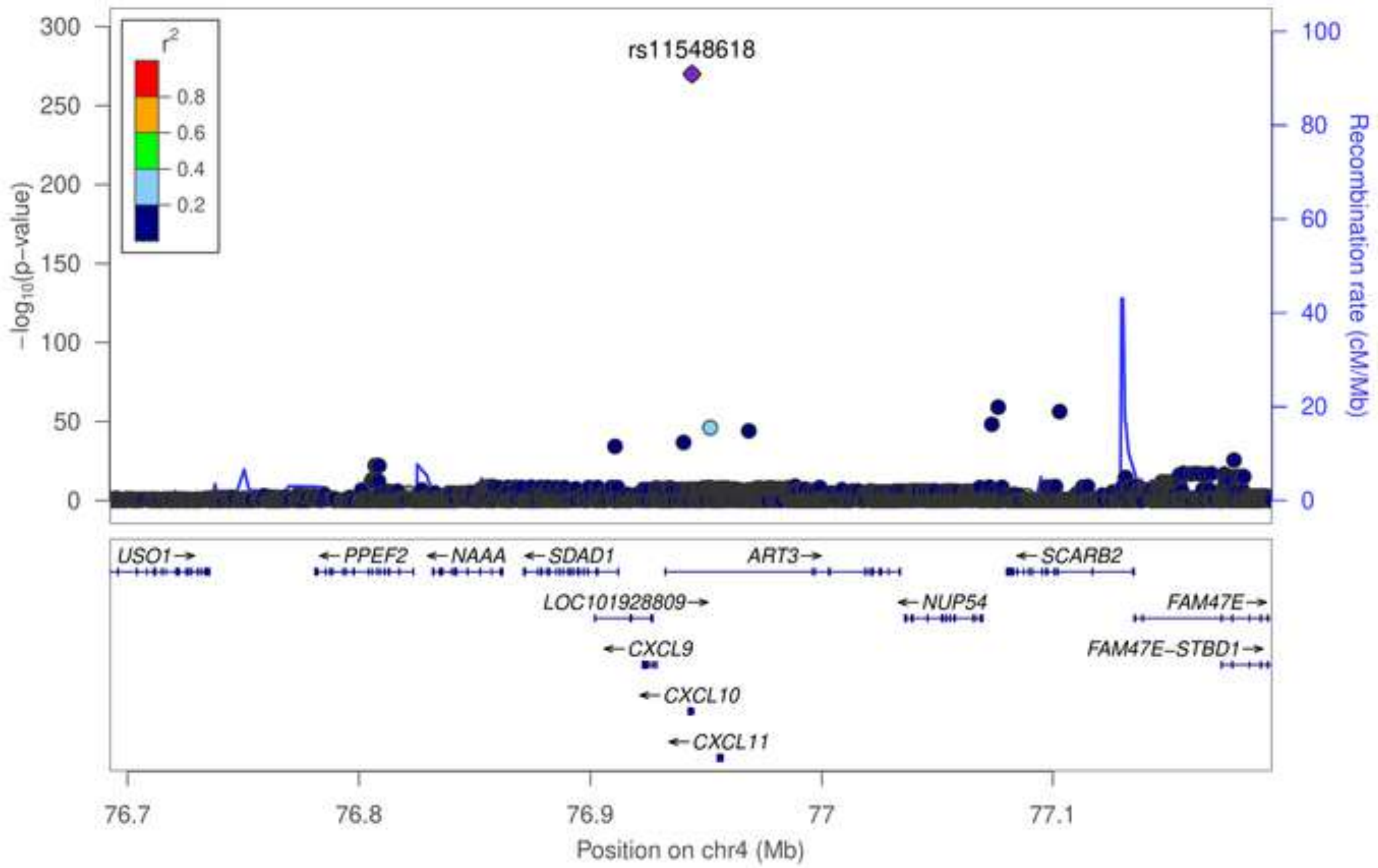

eQTLGen: CXCL1 (CXCL1)–rs1366949

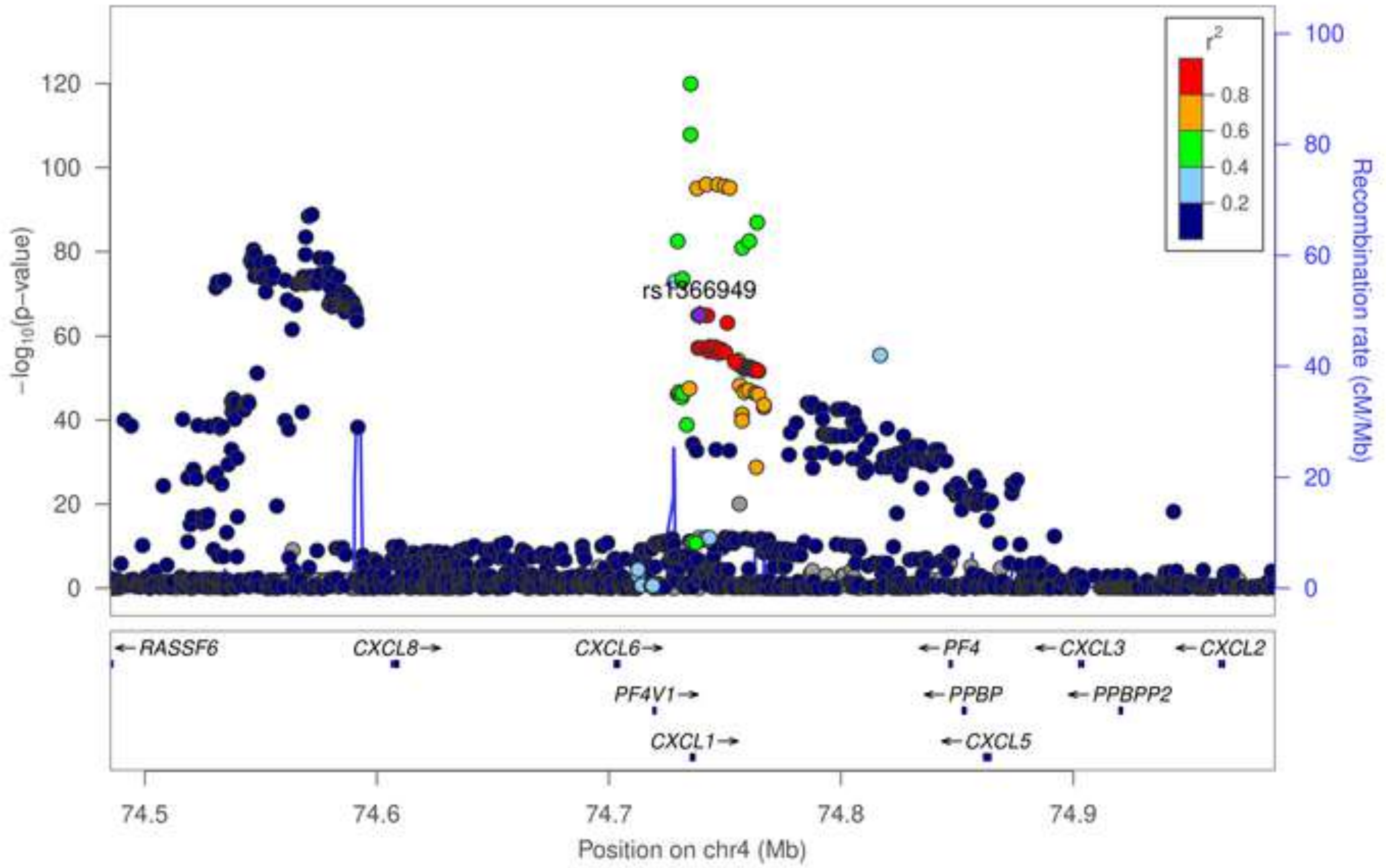

SCALLOP: CXCL1 (CXCL1)–rs1366949

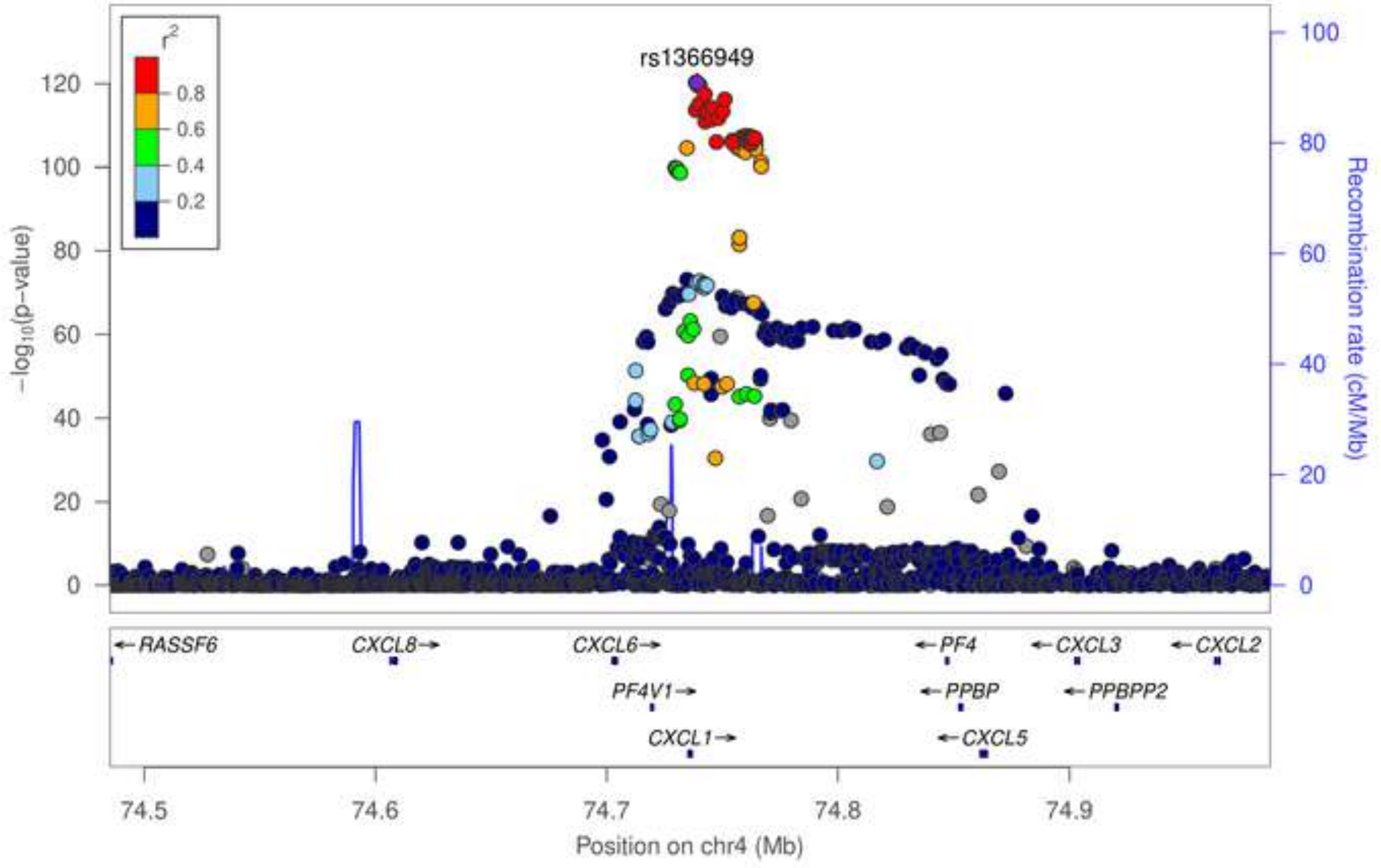

eQTLGen: CXCL5 (CXCL5)–rs450373

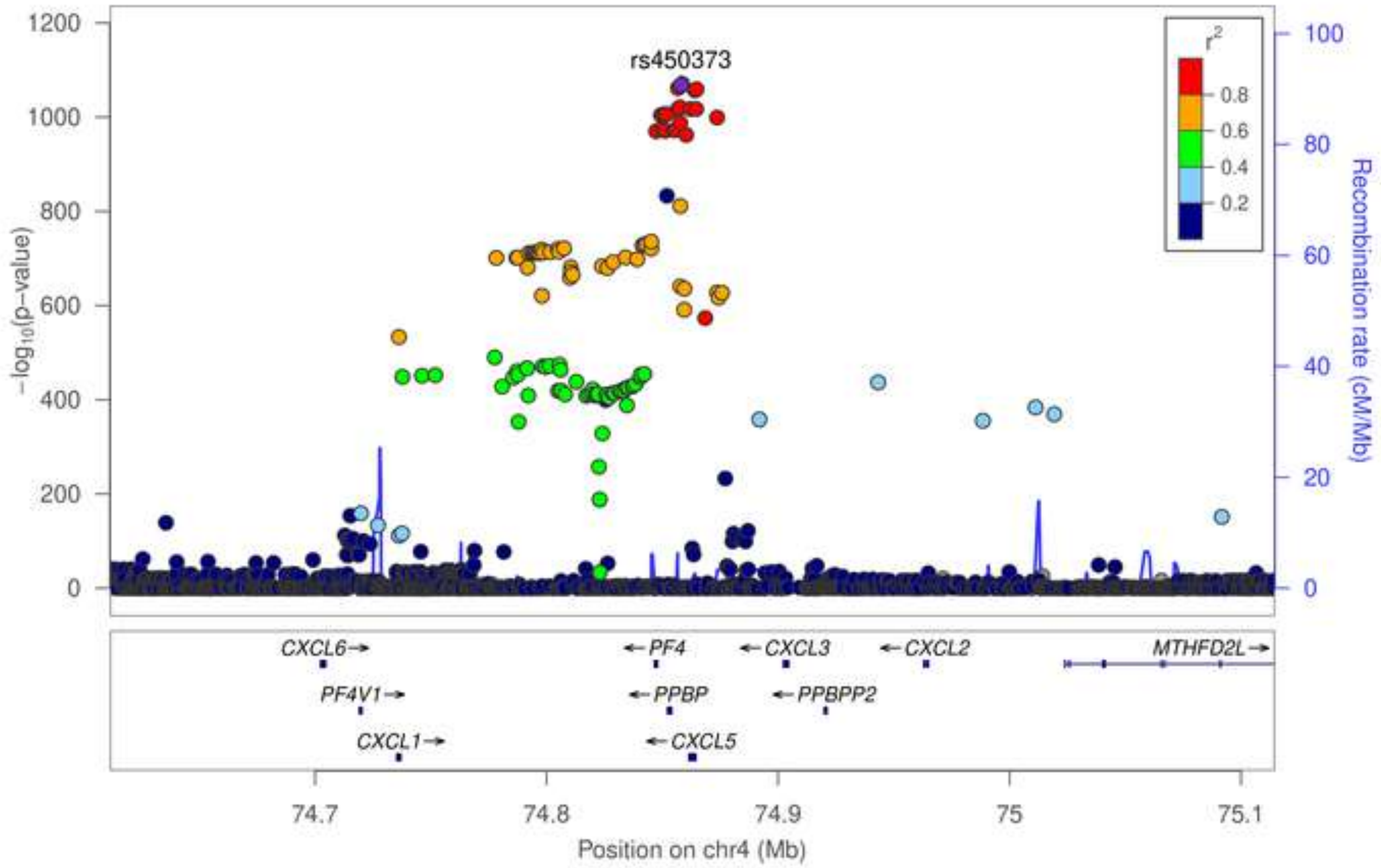

SCALLOP: CXCL5 (CXCL5)–rs450373

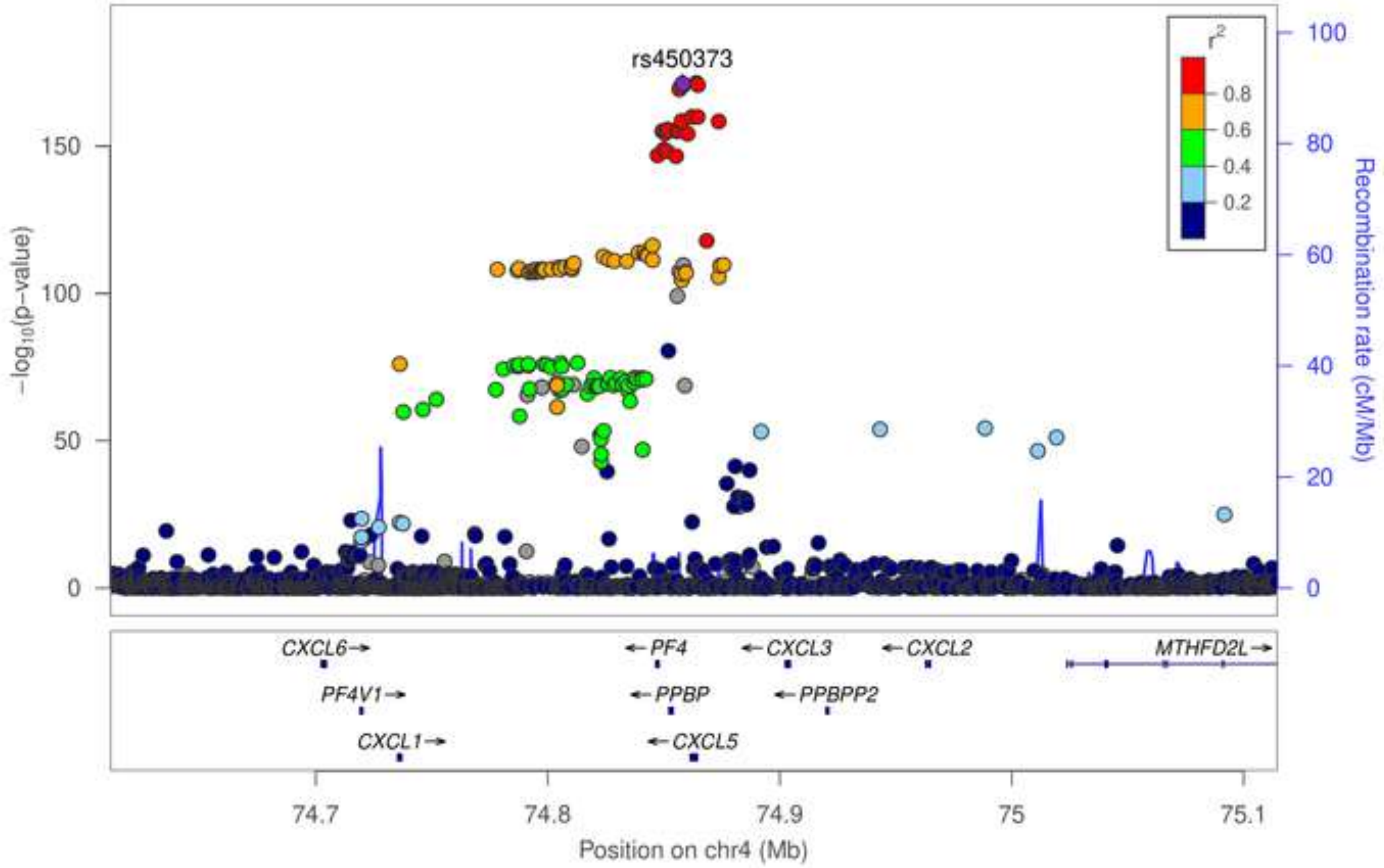

eQTLGen: CXCL6 (CXCL6)−rs16850073

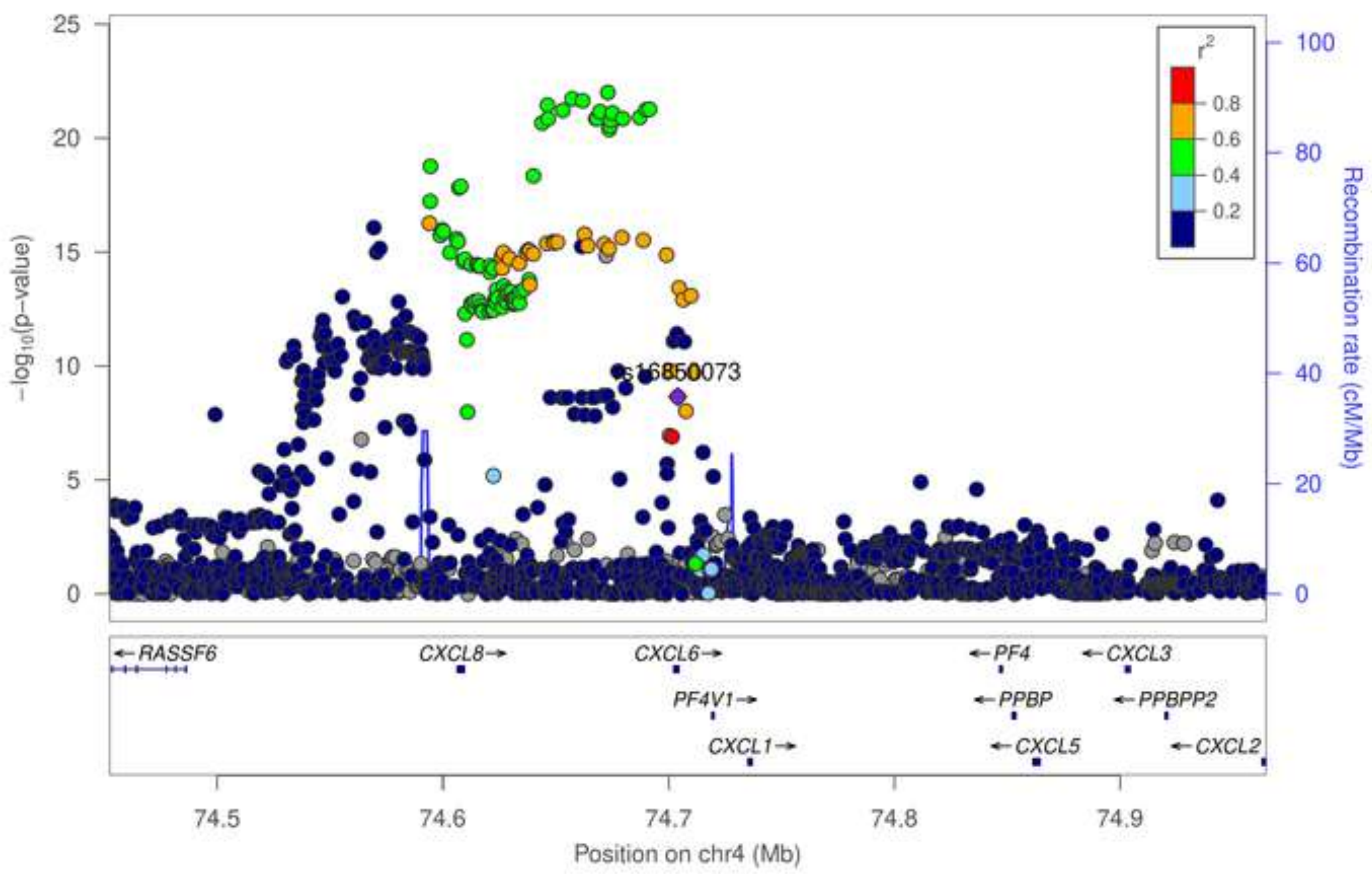

SCALLOP: CXCL6 (CXCL6)−rs16850073

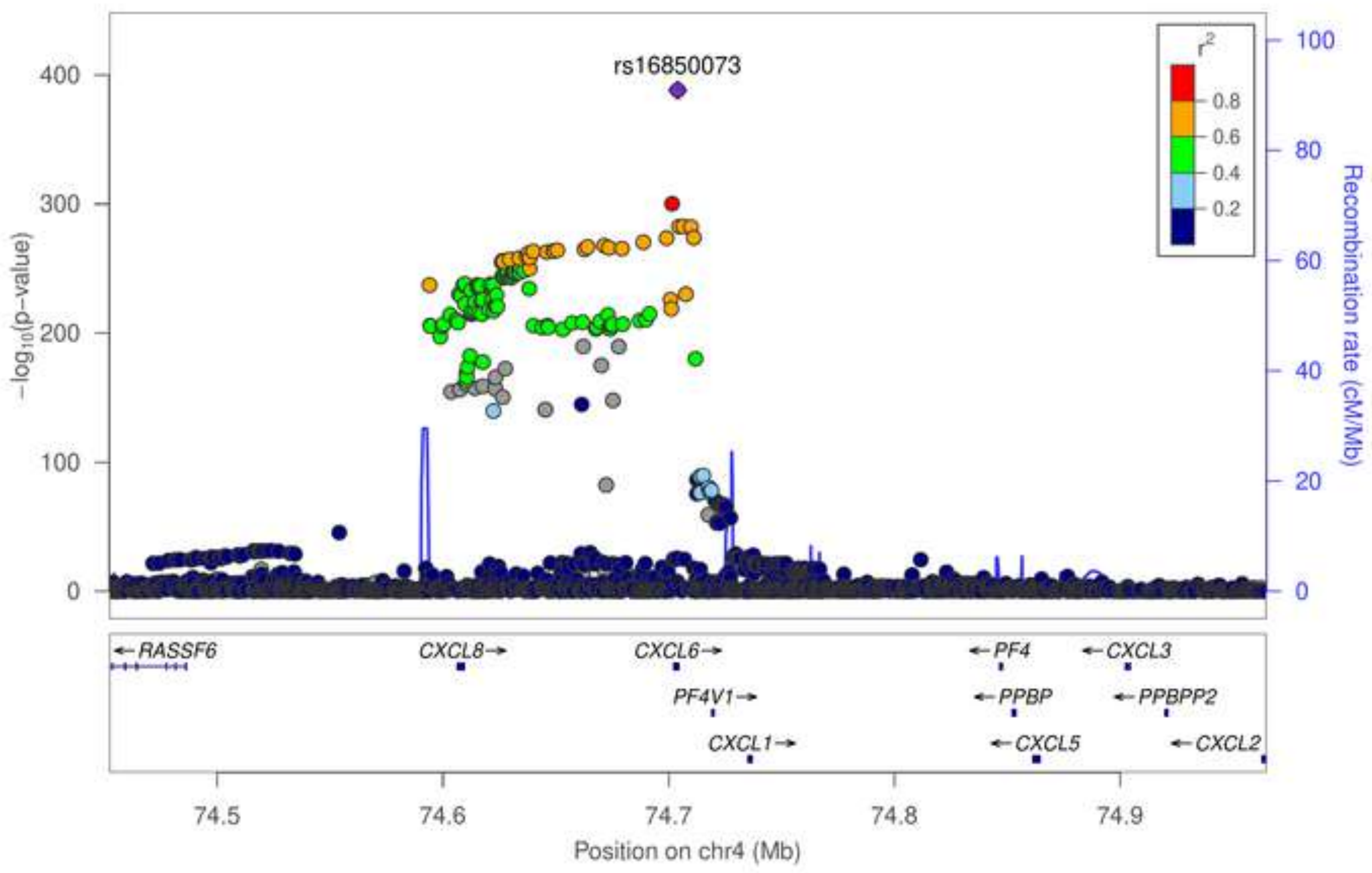

eQTLGen: CXCL9 (CXCL9)−rs4241577

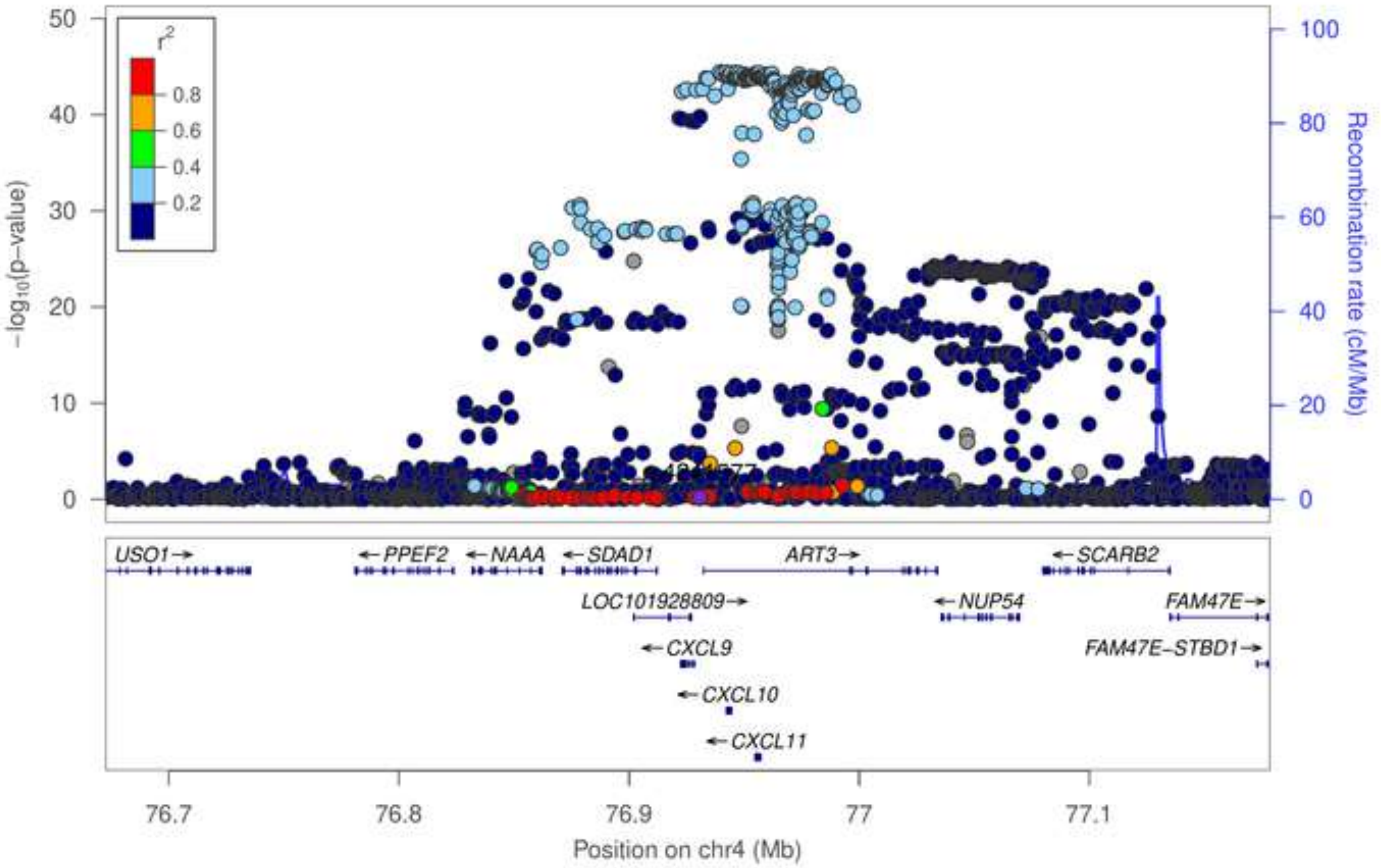

SCALLOP: CXCL9 (CXCL9)−rs4241577

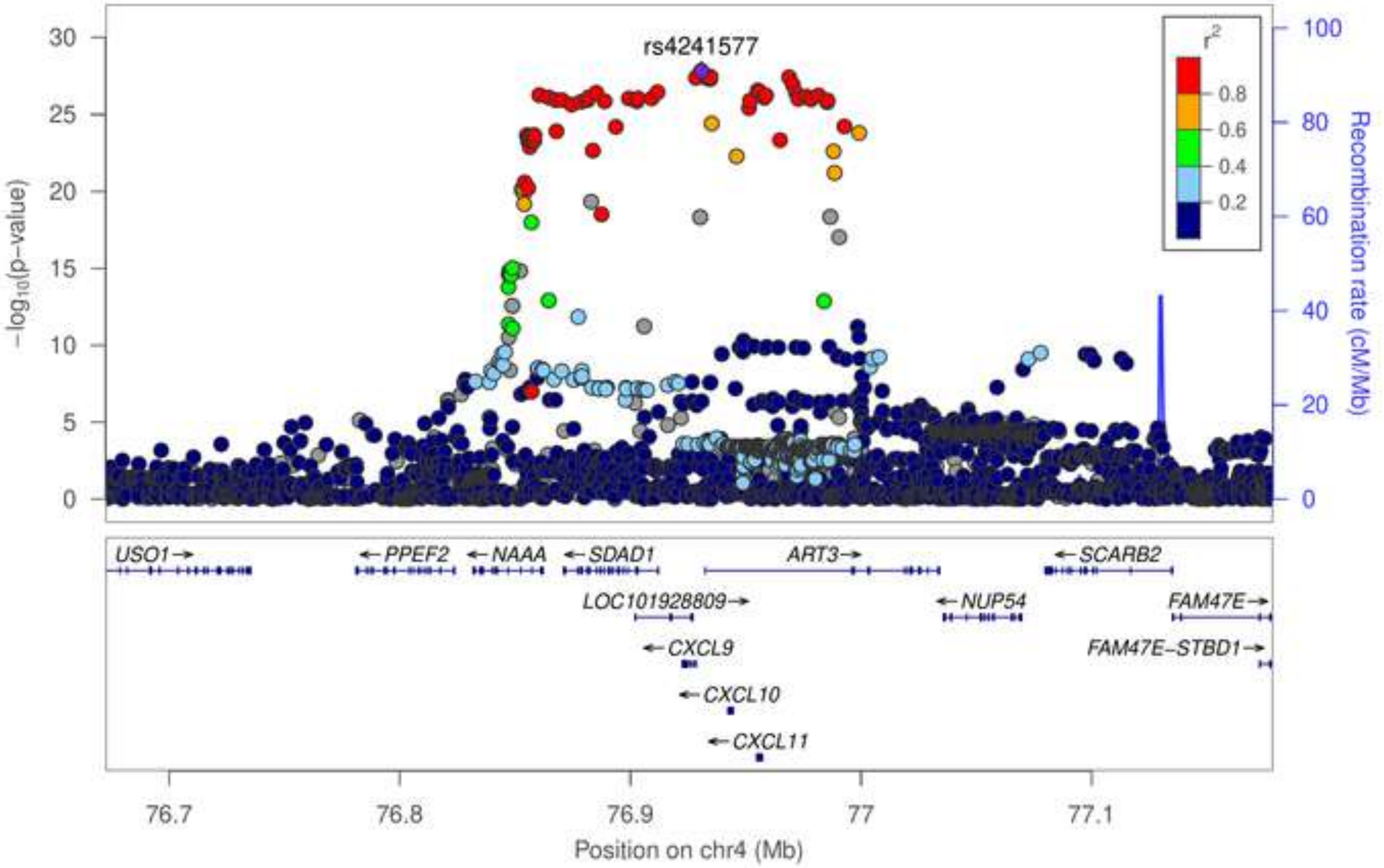

eQTLGen: EN−RAGE (S100A12)−rs3014874

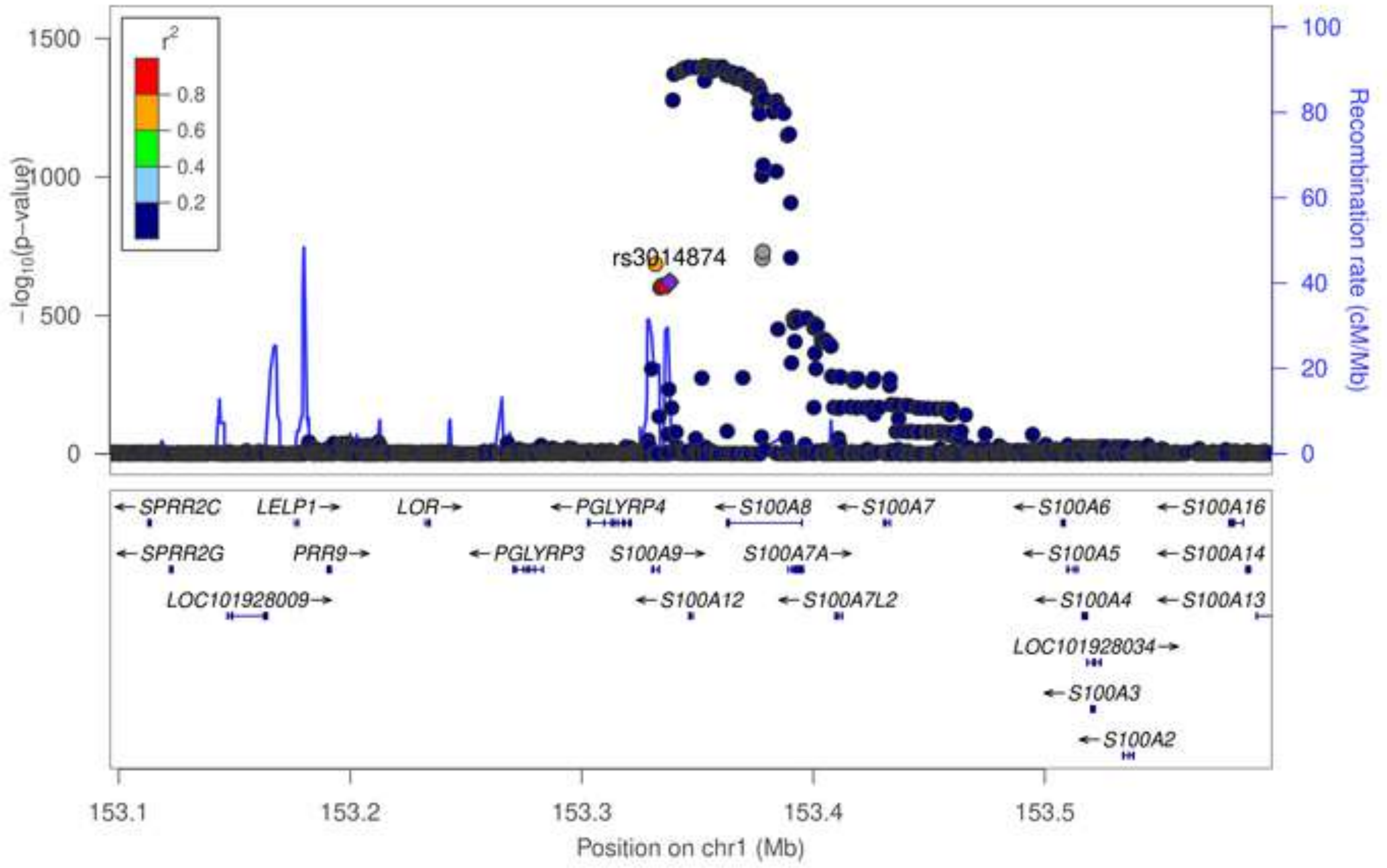

SCALLOP: EN−RAGE (S100A12)−rs3014874

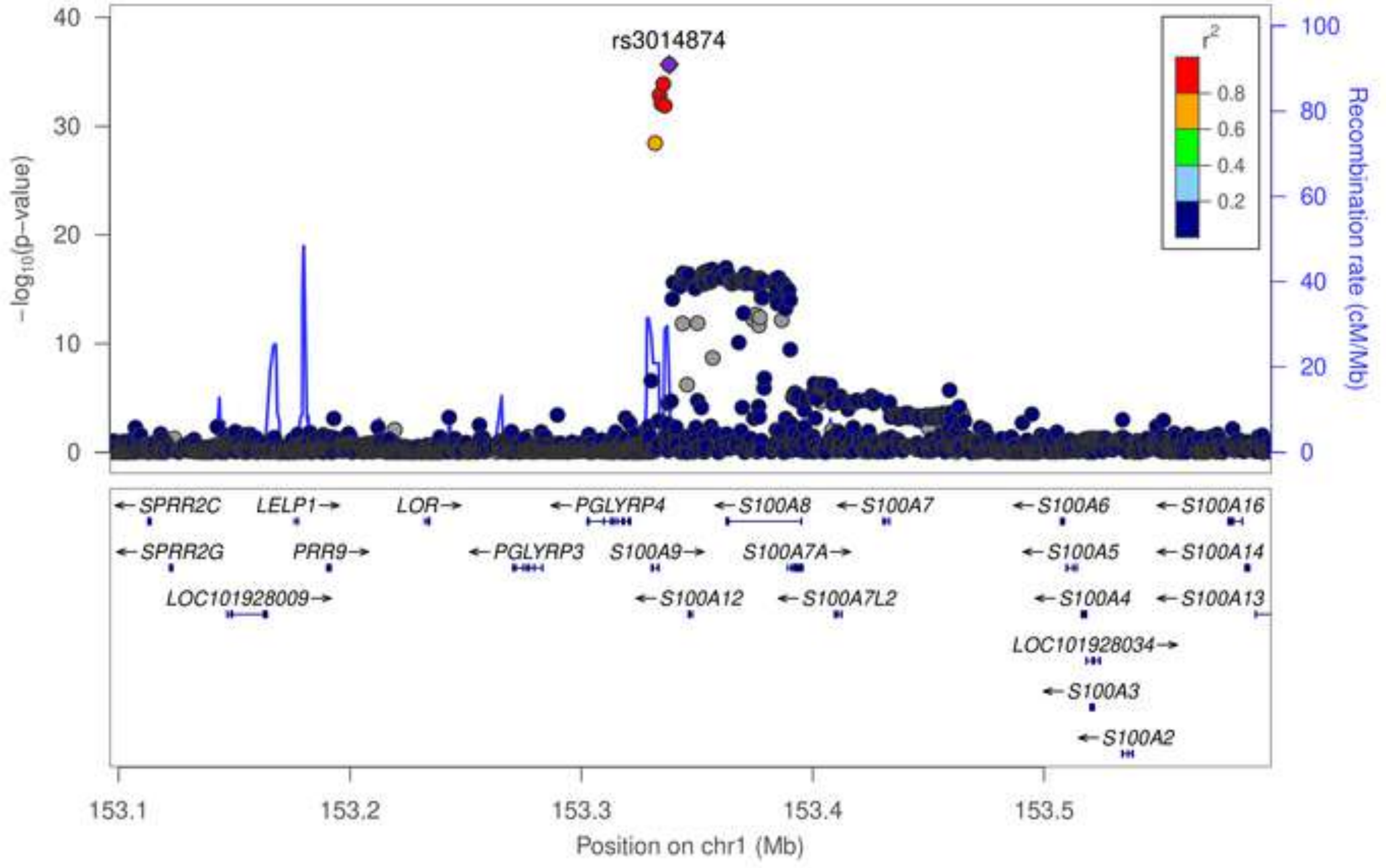

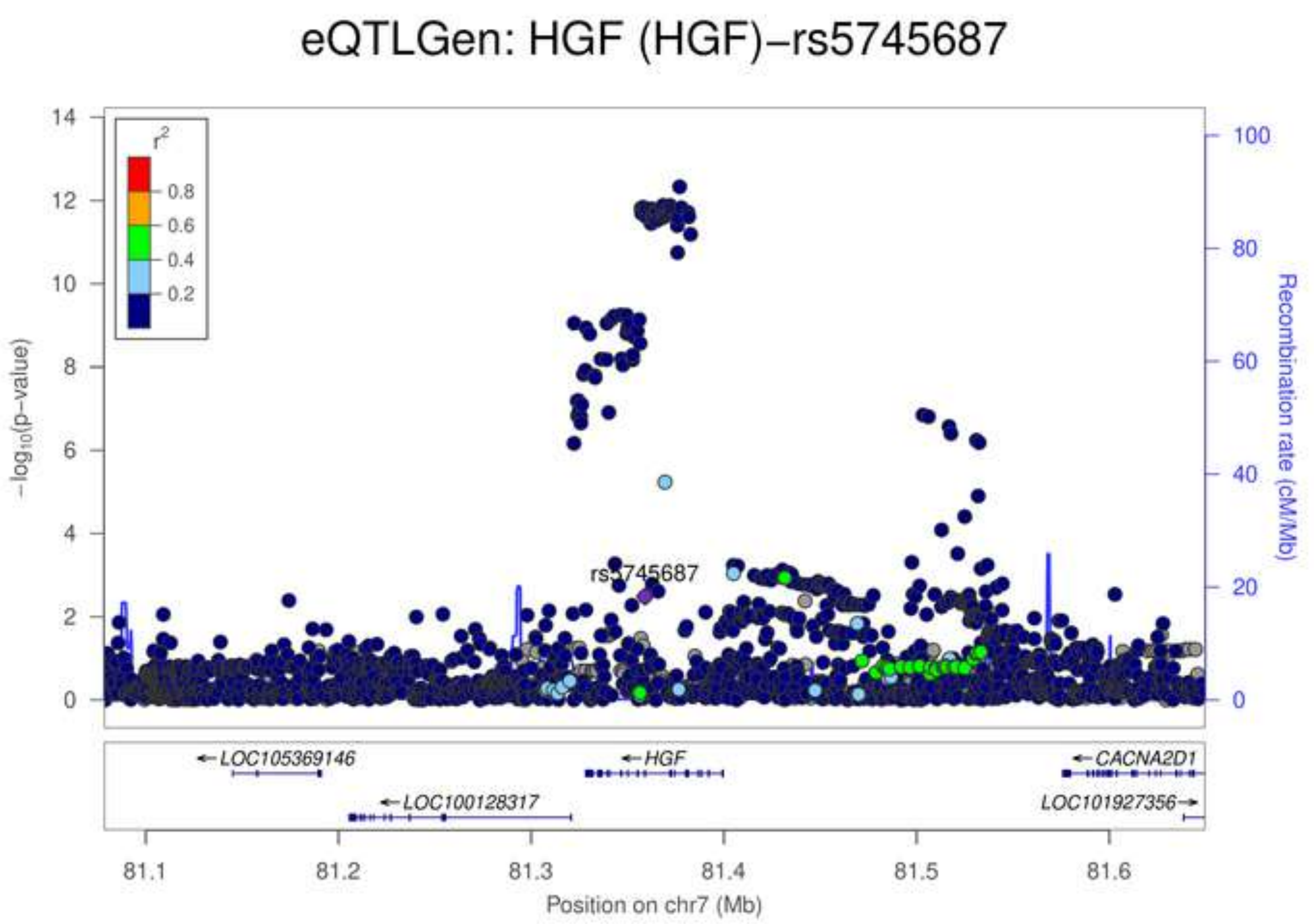
eQTLGen: HGF (HGF)−rs5745687
r2
0.8
0.6
0.4
0.2
rs5745687
−log10(p−value)
Recombination rate (cM/Mb)
LOC105369146
LOC100128317
HGF
CACNA2D1
LOC101927356
81.1
81.2
81.3
81.4
81.5
81.6
Position on chr7 (Mb)

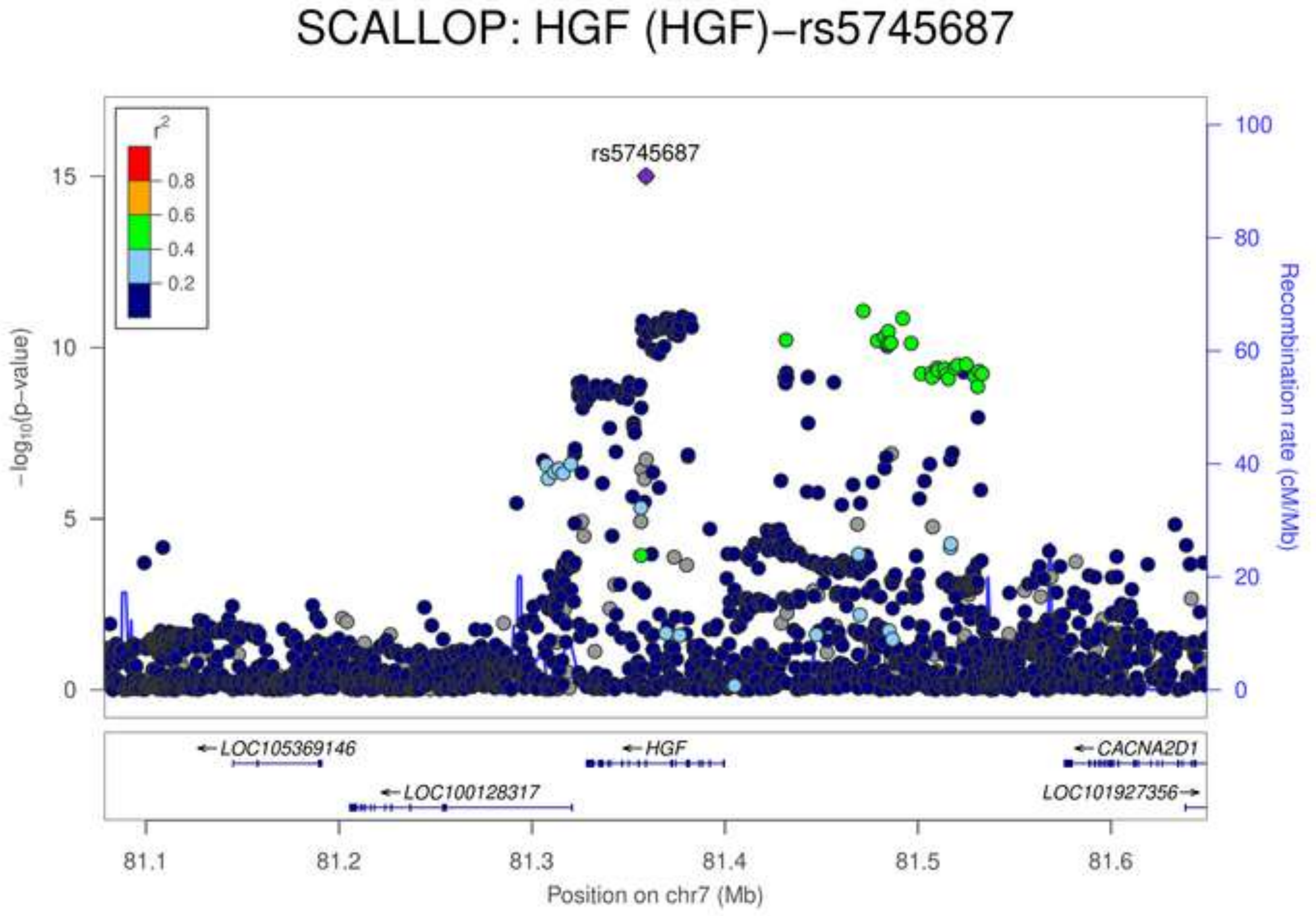
SCALLOP: HGF (HGF)−rs5745687
r2
0.8
0.6
0.4
0.2
rs5745687
−log10(p−value)
Recombination rate (cM/Mb)
LOC105369146
LOC100128317
HGF
CACNA2D1
LOC101927356
81.1
81.2
81.3
81.4
81.5
81.6
Position on chr7 (Mb)

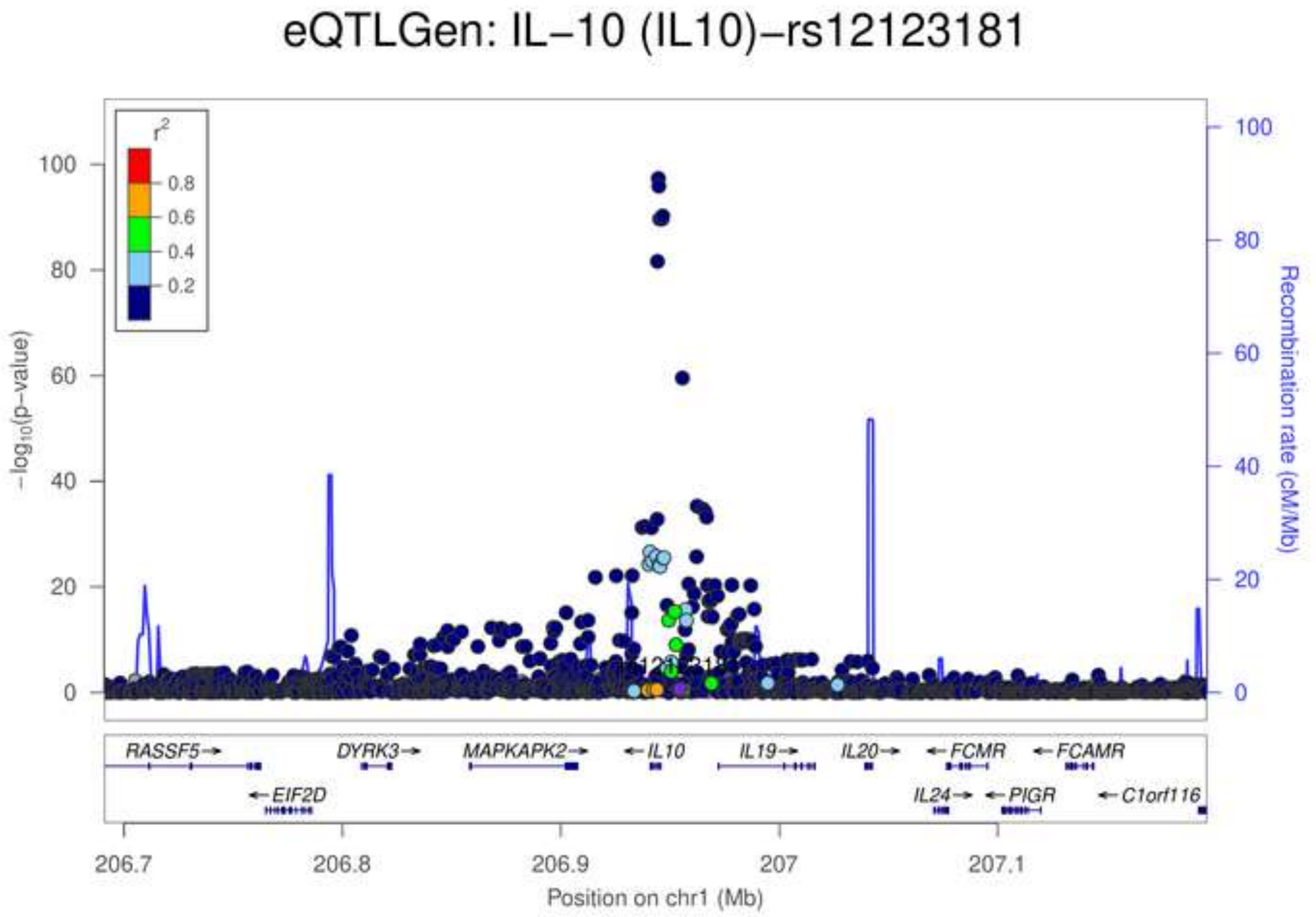
eQTLGen: IL−10 (IL10)−rs12123181
r²
0.8
0.6
0.4
0.2
−log10(p−value)
Recombination rate (cM/Mb)
RASSF5→
EIF2D
DYRK3→
MAPKAPK2→
IL10
IL19→
IL20→
FCMR
FCAMR
IL24→
PIGR
C1orf116
Position on chr1 (Mb)

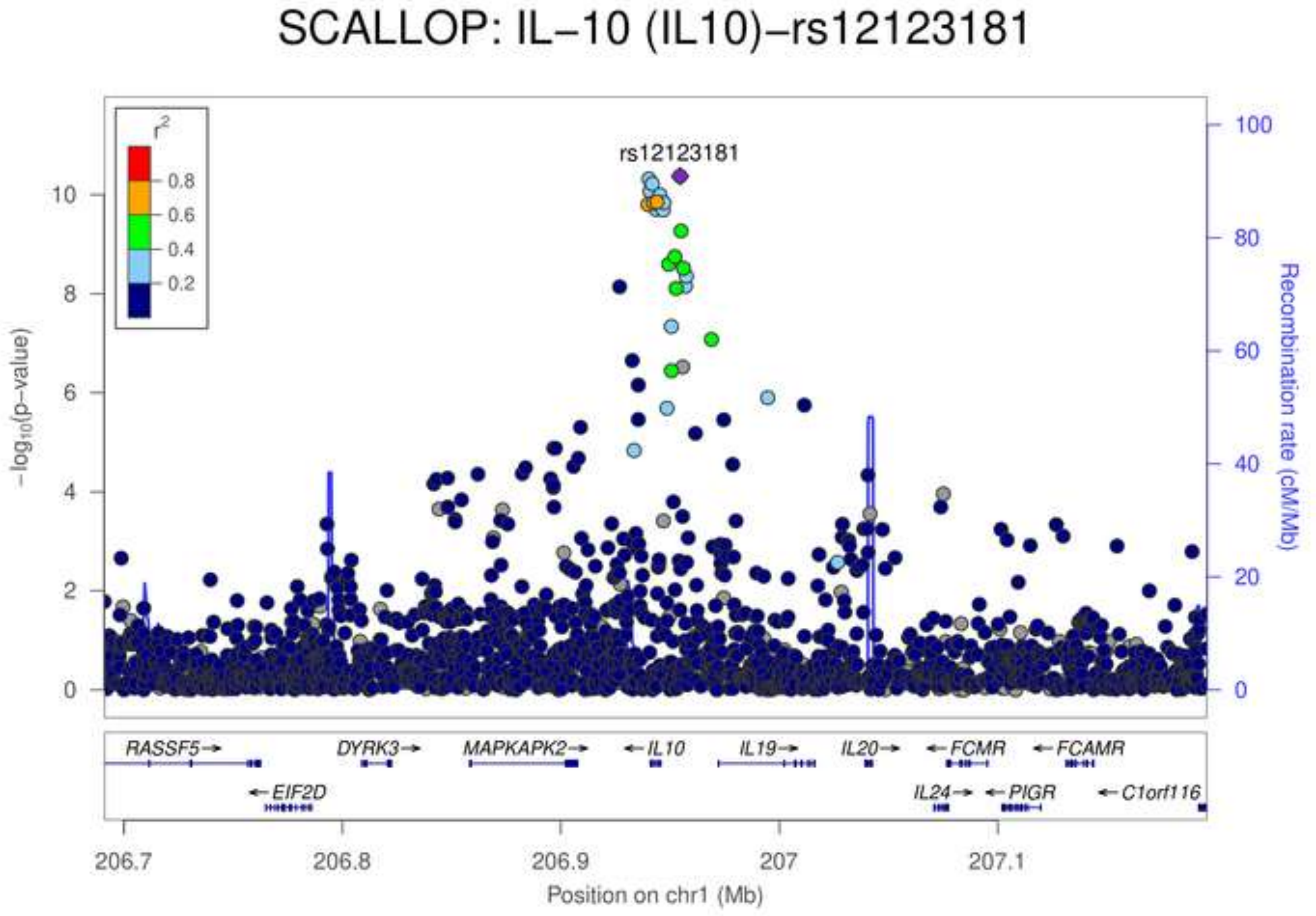
SCALLOP: IL−10 (IL10)−rs12123181
r²
0.8
0.6
0.4
0.2
rs12123181
−log10(p−value)
Recombination rate (cM/Mb)
RASSF5→
EIF2D
DYRK3→
MAPKAPK2→
IL10
IL19→
IL20→
FCMR
FCAMR
IL24→
PIGR
C1orf116
Position on chr1 (Mb)

eQTLGen: IL10RB (IL10RB)−rs2266590

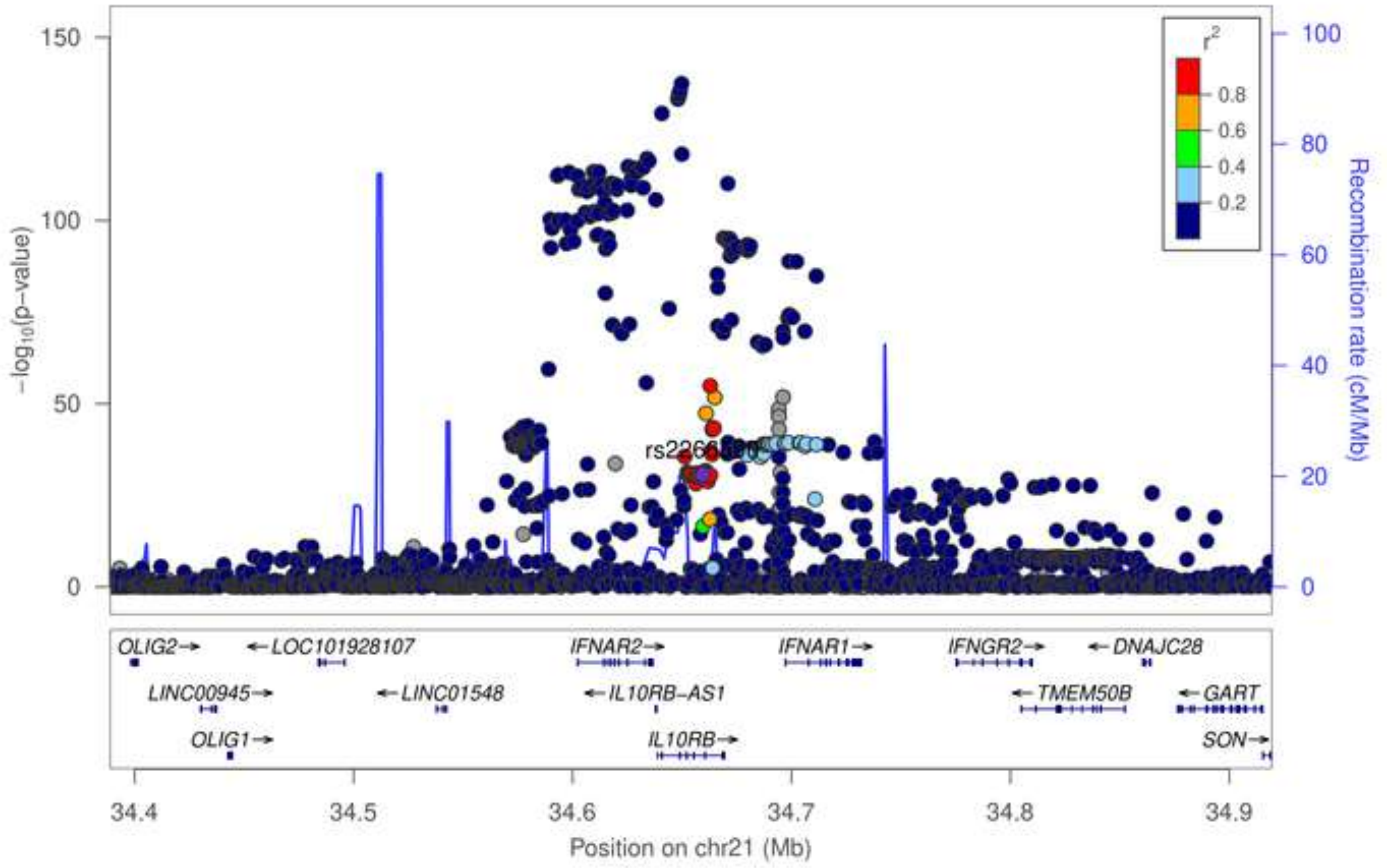

SCALLOP: IL10RB (IL10RB)−rs2266590

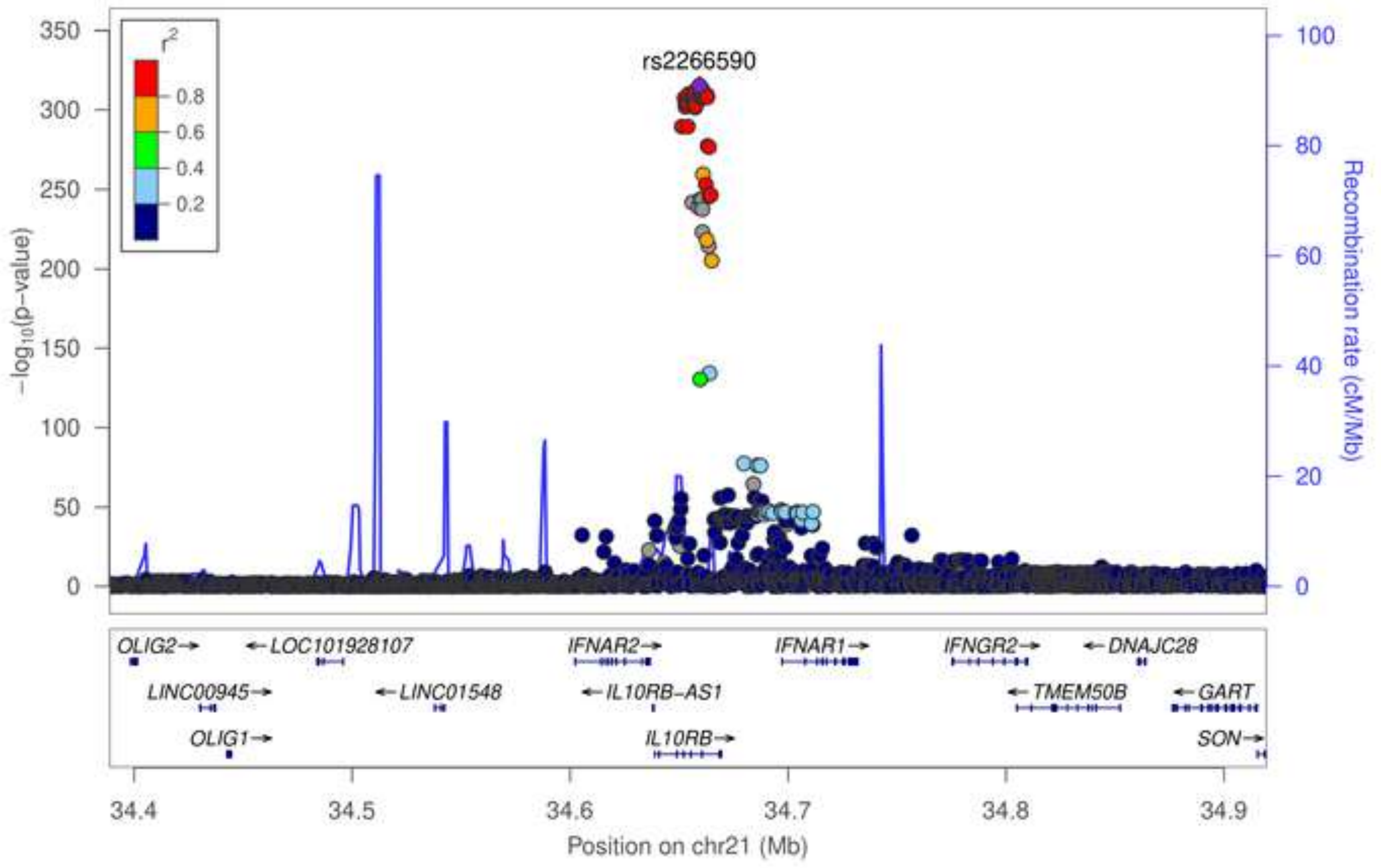

eQTLGen: IL−15RA (IL15RA)−rs2228059

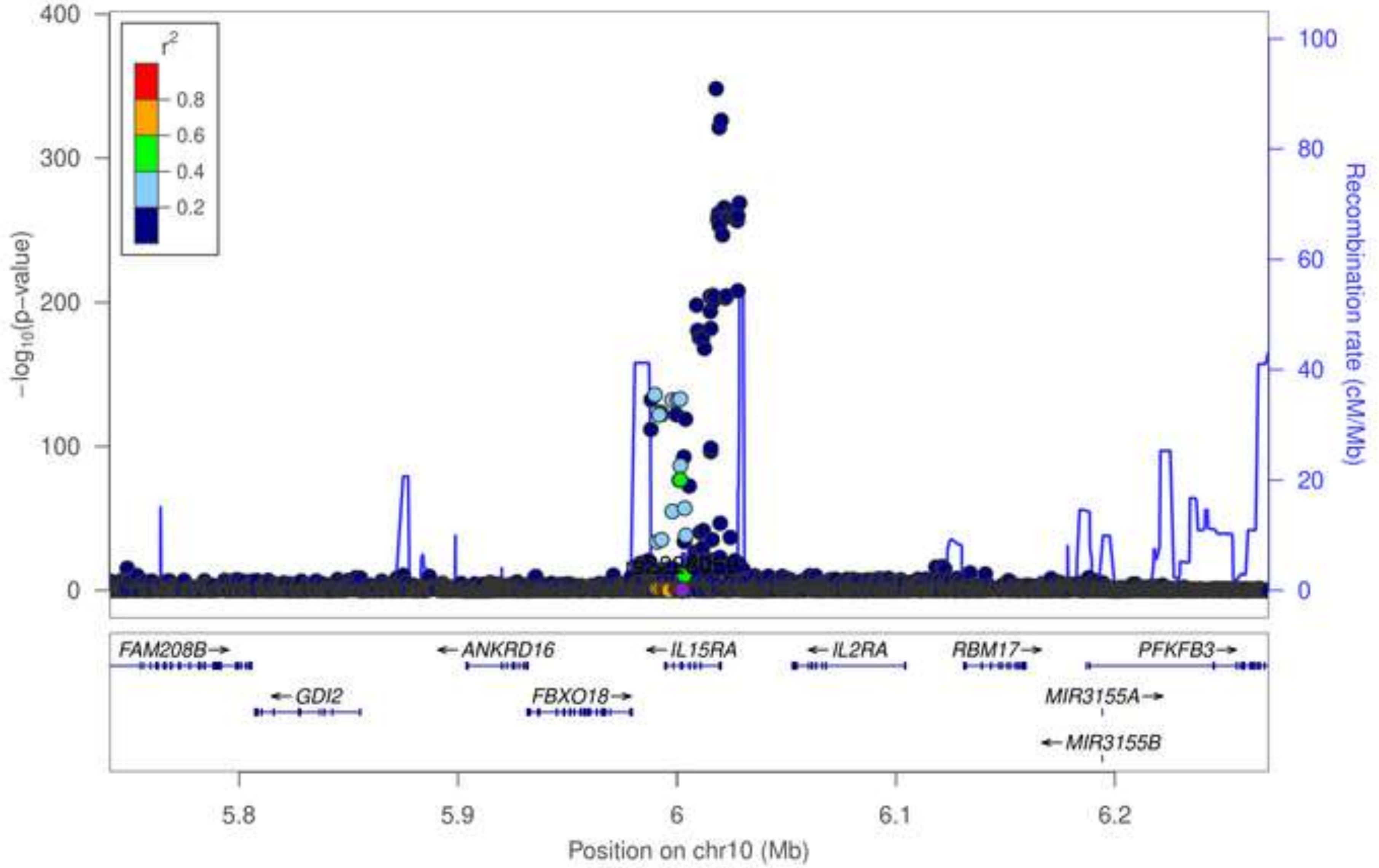

SCALLOP: IL−15RA (IL15RA)−rs2228059

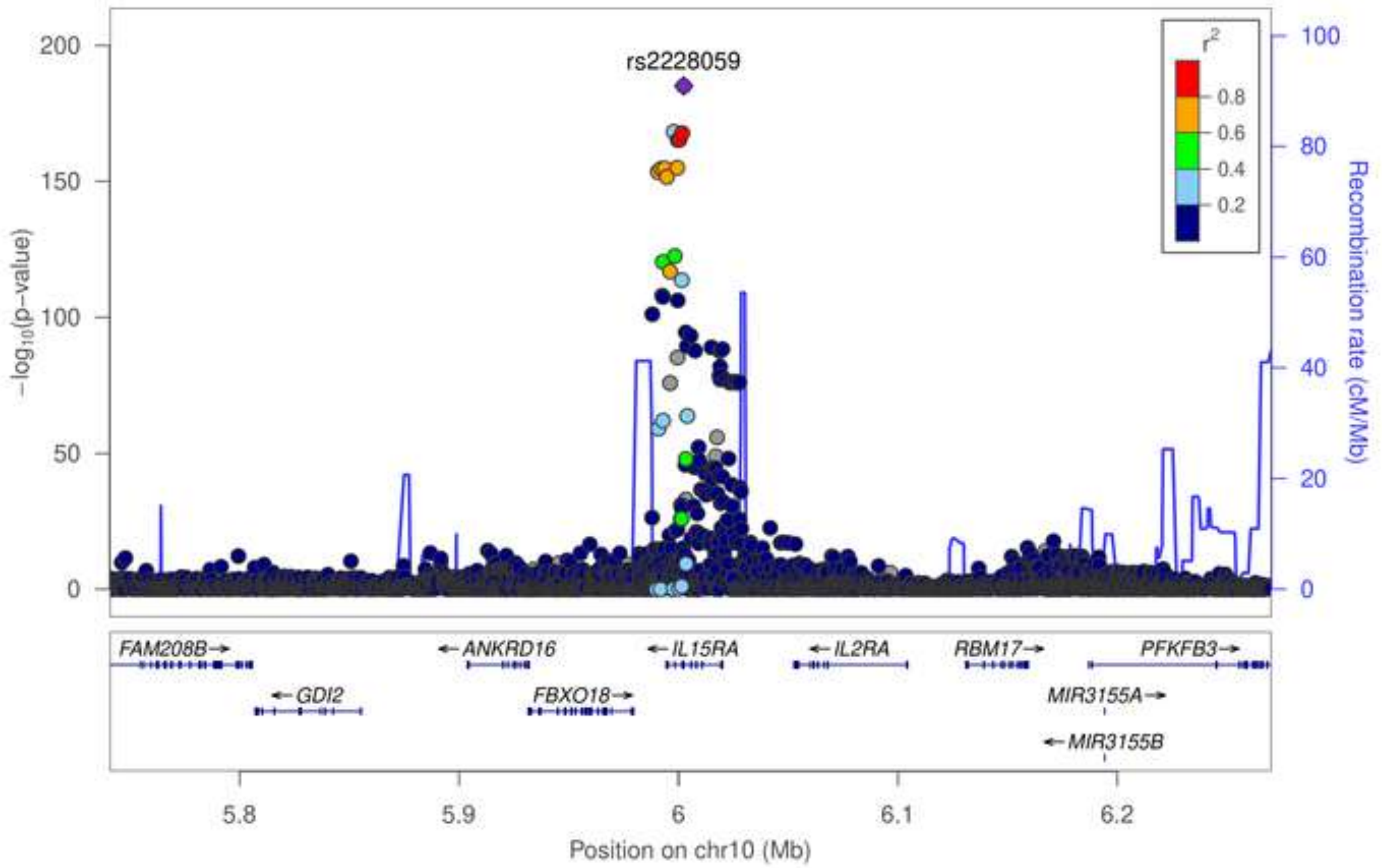

eQTLGen: IL−17C (IL17C)−rs17700884

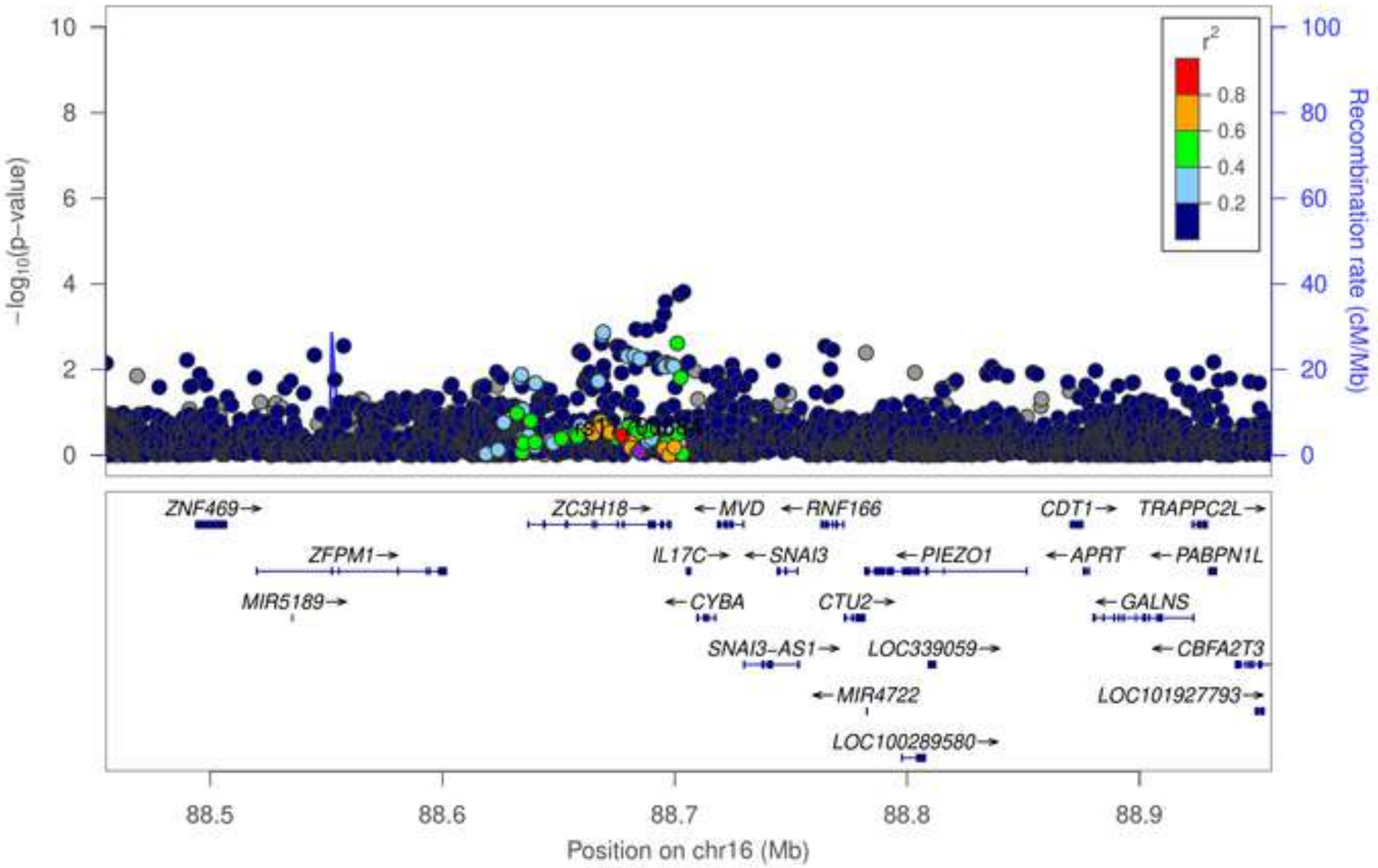

SCALLOP: IL−17C (IL17C)−rs17700884

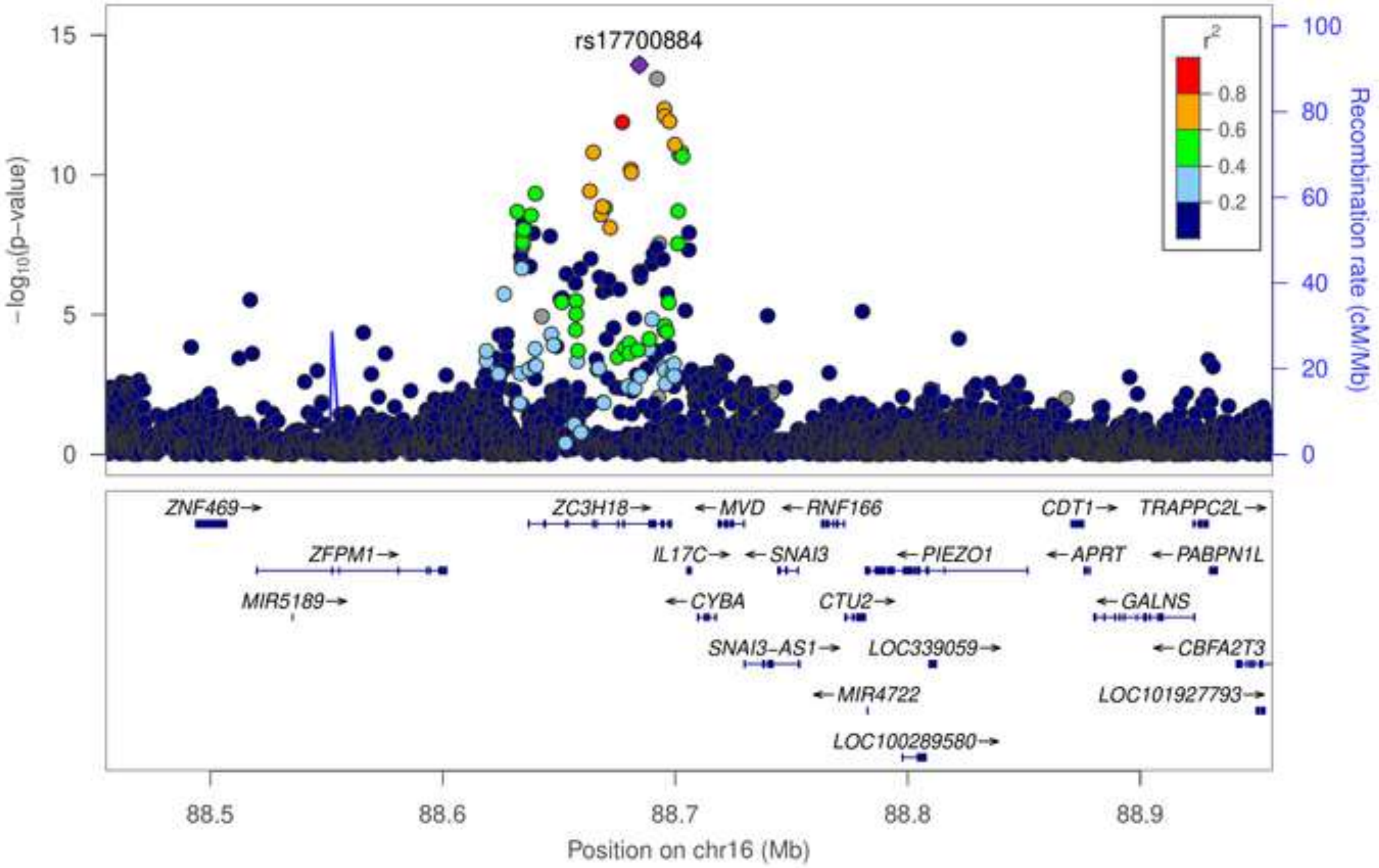

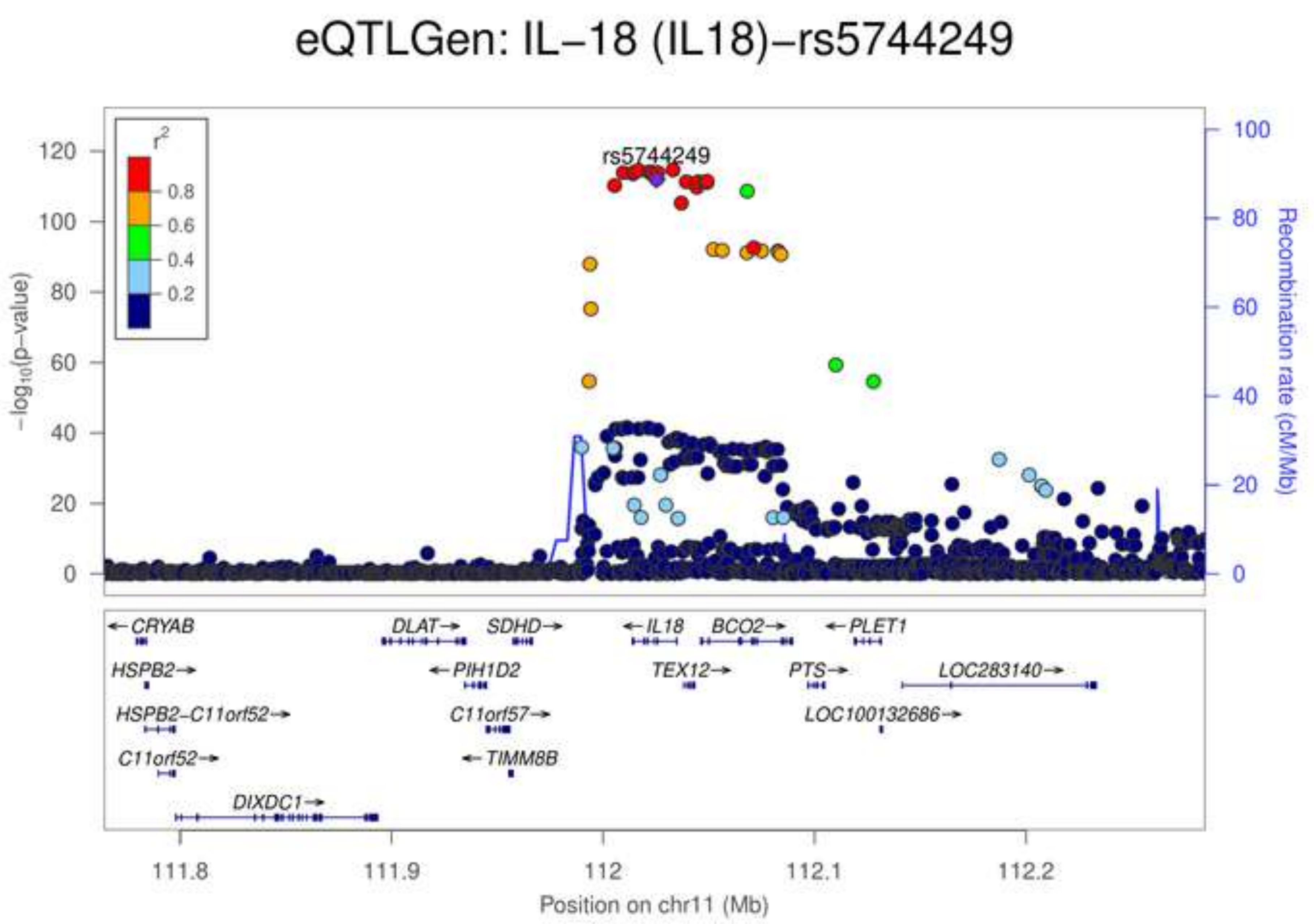
eQTLGen: IL−18 (IL18)−rs5744249
rs5744249
r2
0.8
0.6
0.4
0.2
−log10(p−value)
Recombination rate (cM/Mb)
CRYAB
HSPB2
HSPB2−C11orf52
C11orf52
DIXDC1
DLAT
PIH1D2
SDHD
C11orf57
TIMM8B
IL18
TEX12
BCO2
PTS
LOC100132686
PLET1
LOC283140
111.8
111.9
112
112.1
112.2
Position on chr11 (Mb)

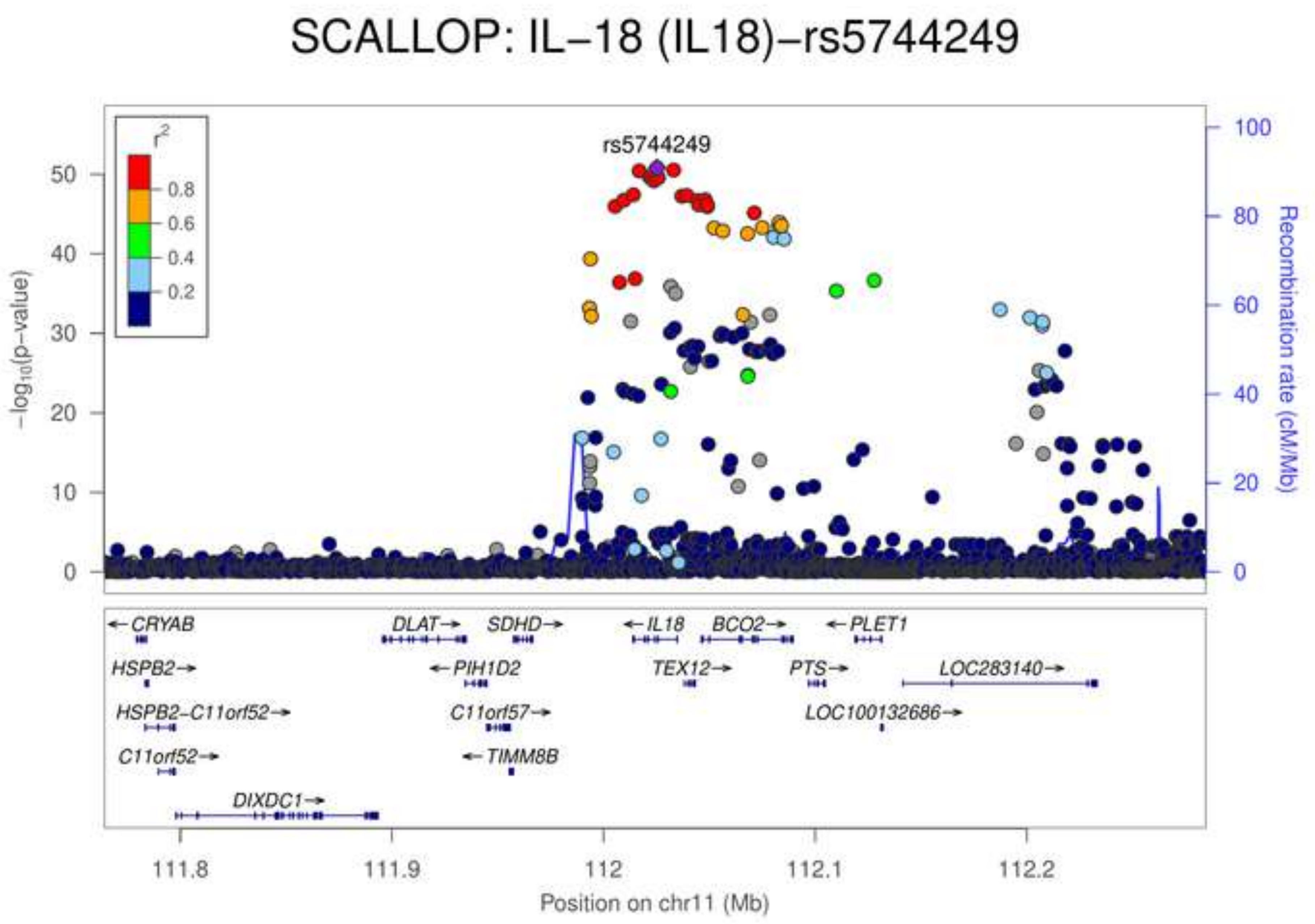
SCALLOP: IL−18 (IL18)−rs5744249
rs5744249
r2
0.8
0.6
0.4
0.2
−log10(p−value)
Recombination rate (cM/Mb)
CRYAB
HSPB2
HSPB2−C11orf52
C11orf52
DIXDC1
DLAT
PIH1D2
SDHD
C11orf57
TIMM8B
IL18
TEX12
BCO2
PTS
LOC100132686
PLET1
LOC283140
111.8
111.9
112
112.1
112.2
Position on chr11 (Mb)

eQTLGen: IL−18R1 (IL18R1)−rs2270297

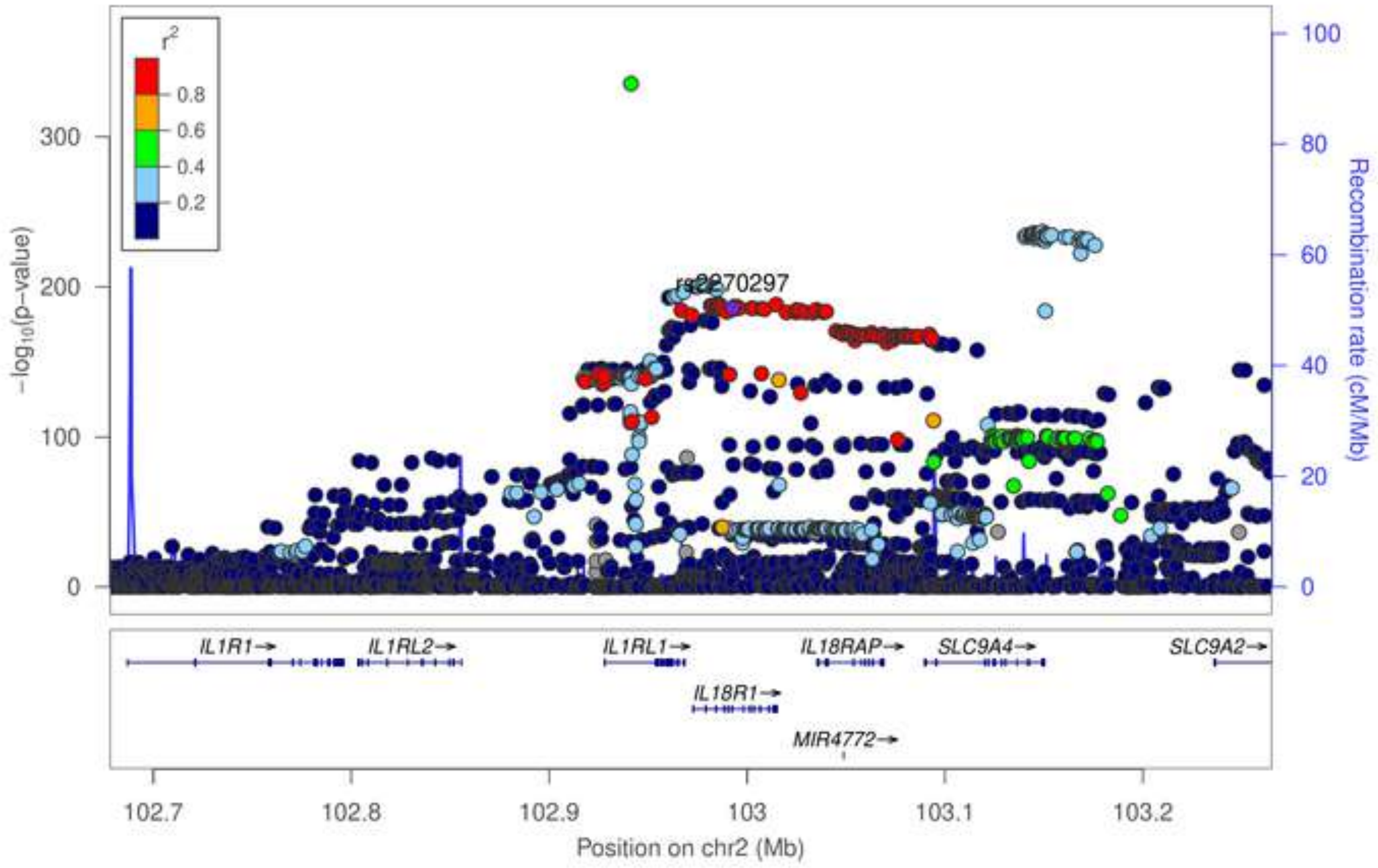

SCALLOP: IL−18R1 (IL18R1)−rs2270297

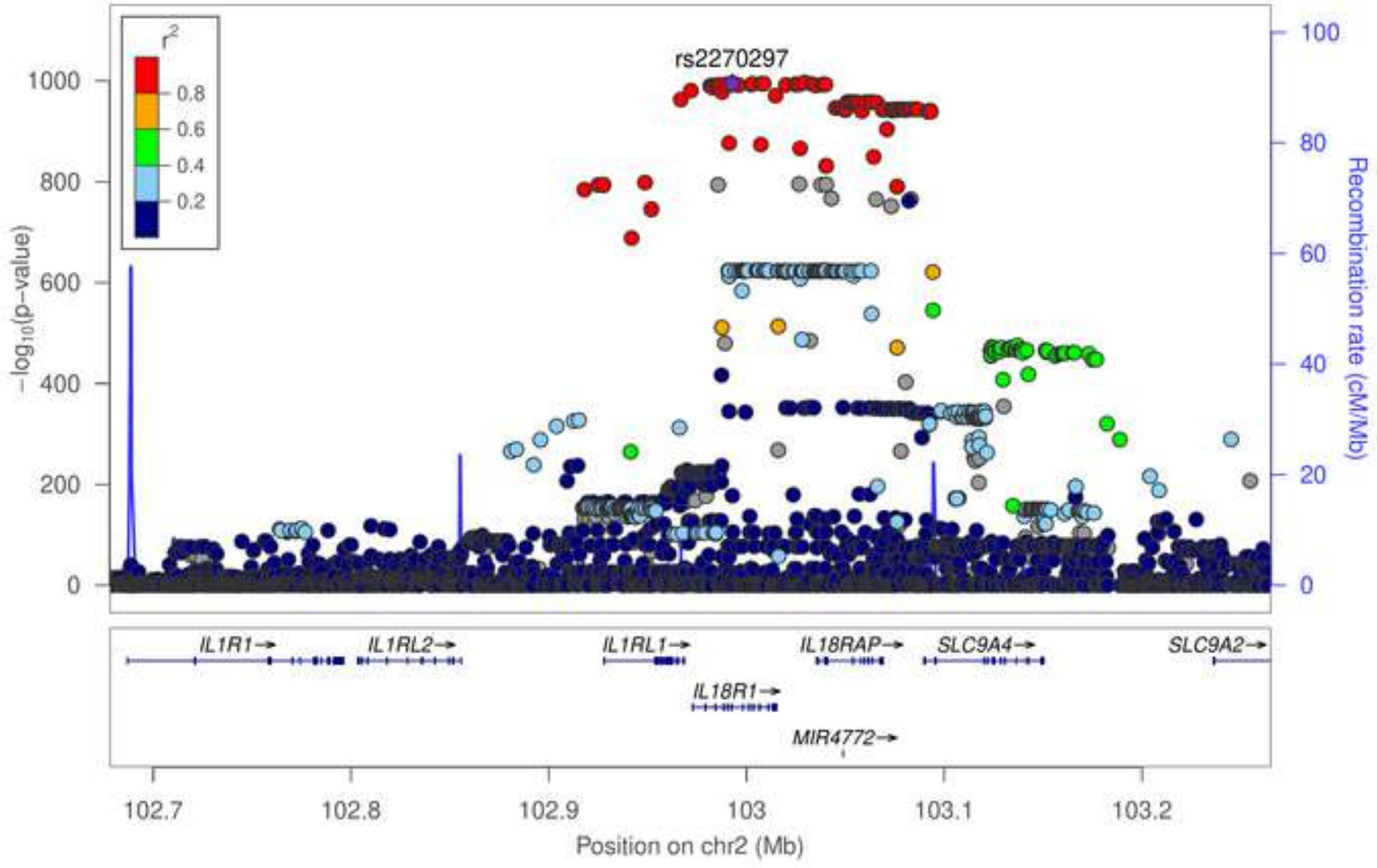

eQTLGen: IL−7 (IL7)−rs112359206

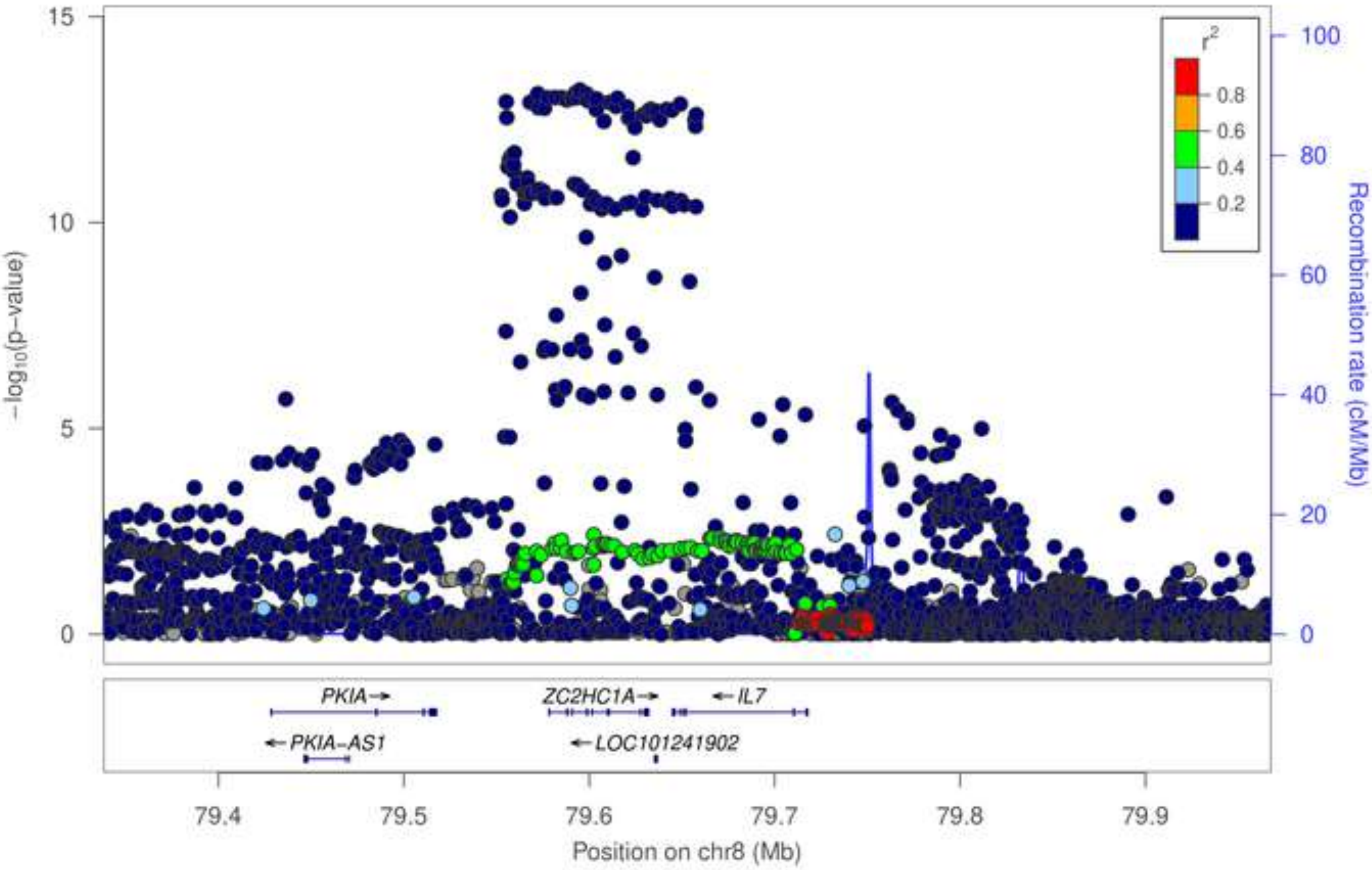

SCALLOP: IL−7 (IL7)−rs112359206

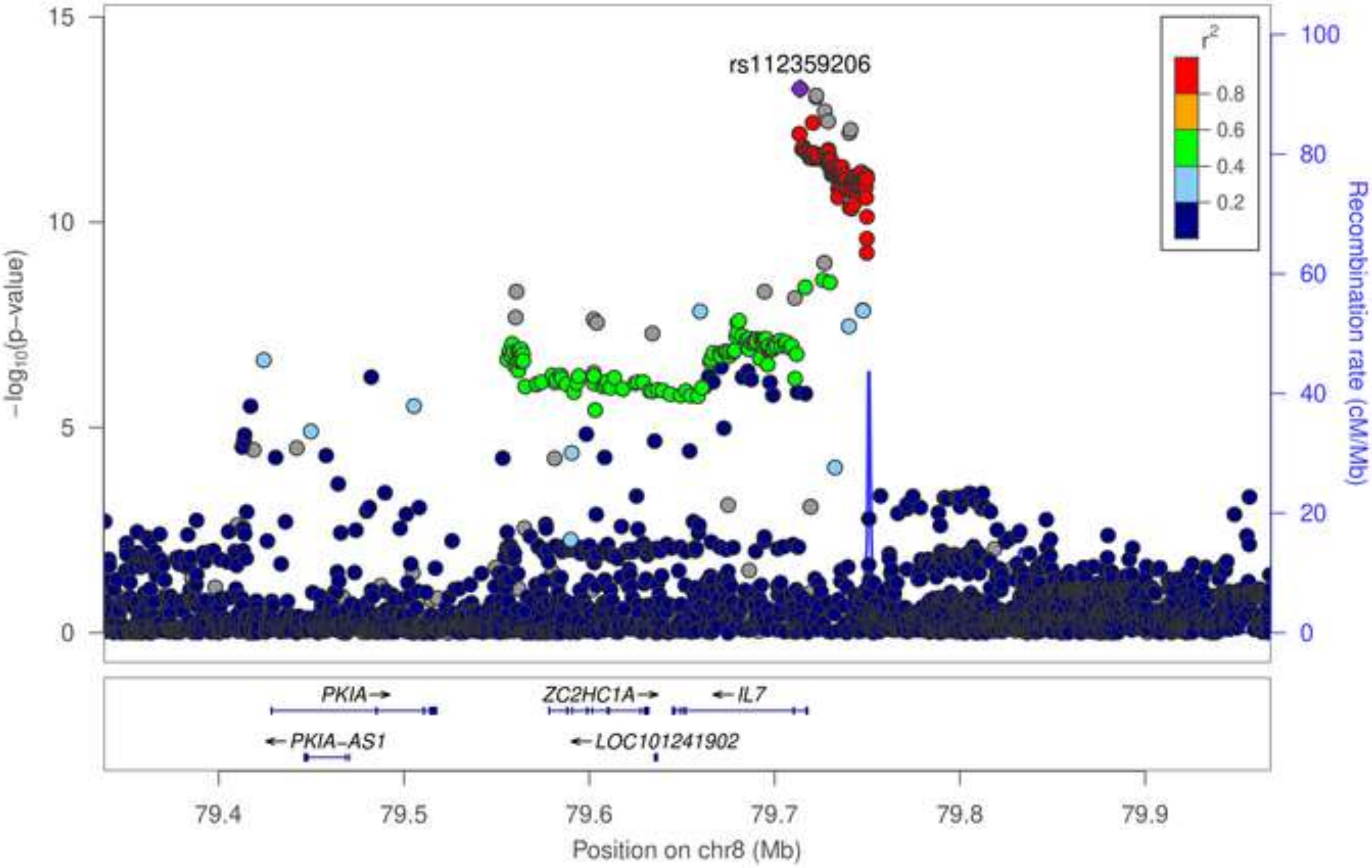

eQTLGen: IL−8 (IL8)−rs6446951

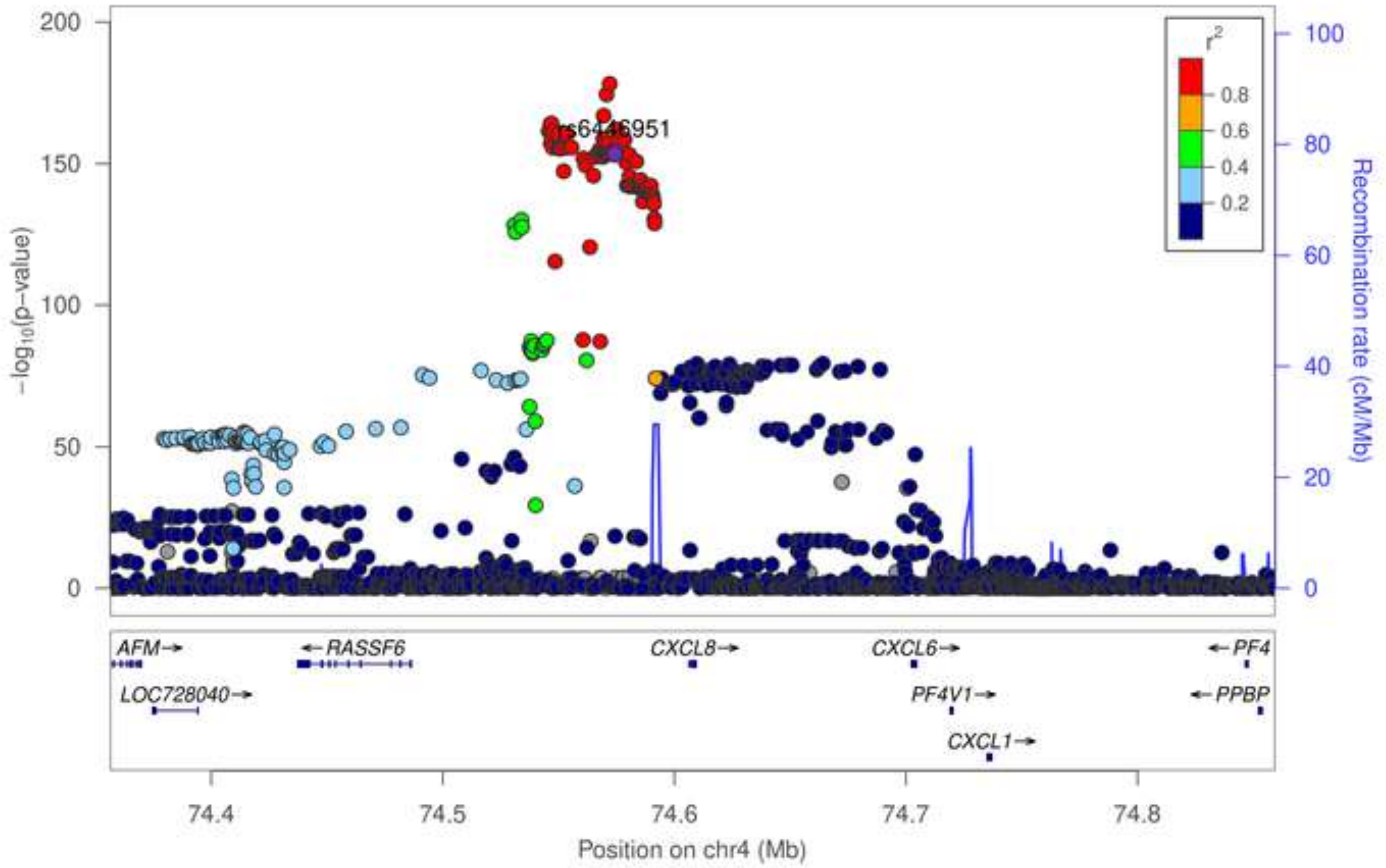

SCALLOP: IL−8 (IL8)−rs6446951

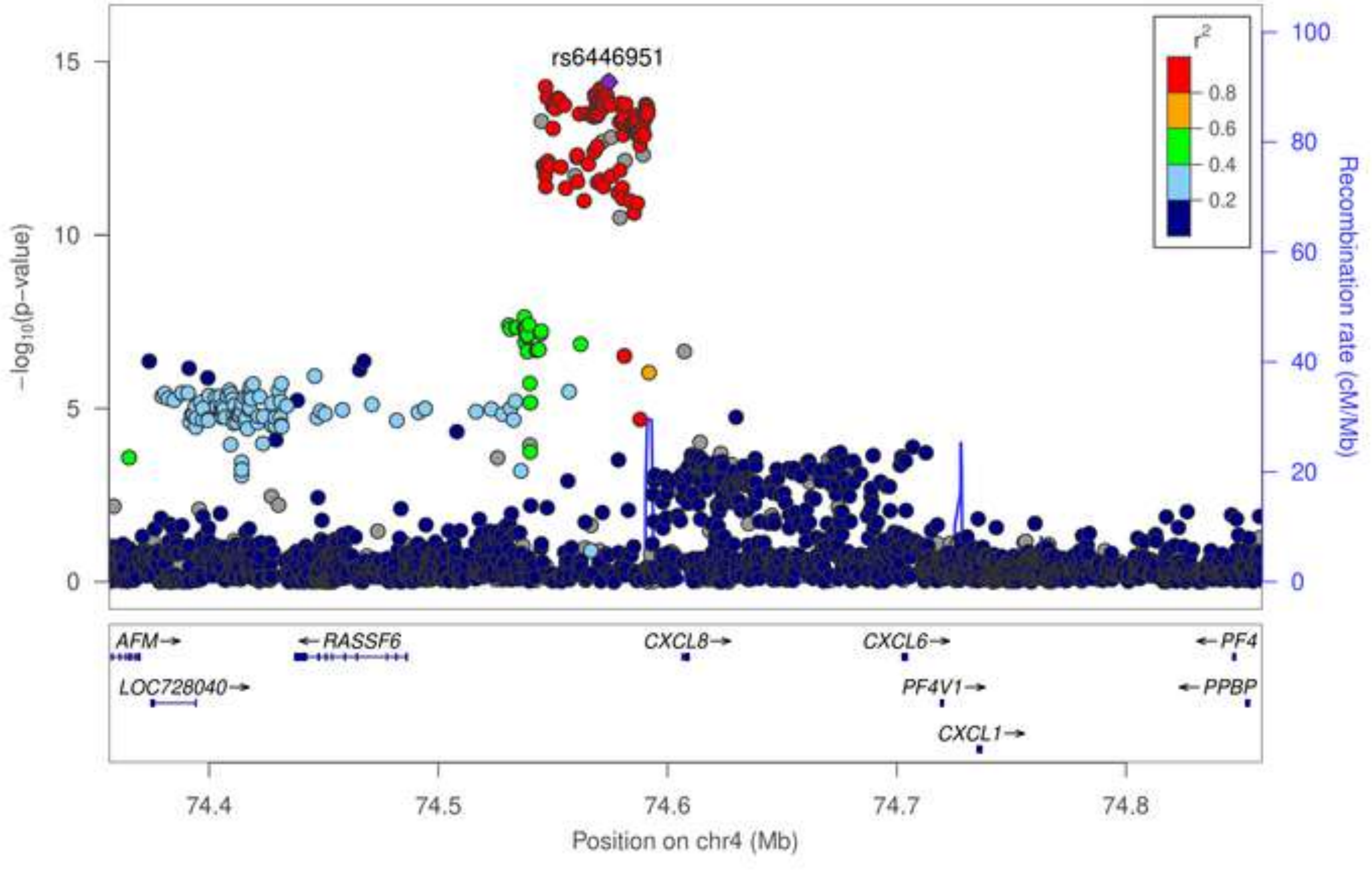

eQTLGen: LAP (TGFB1)−rs1800472

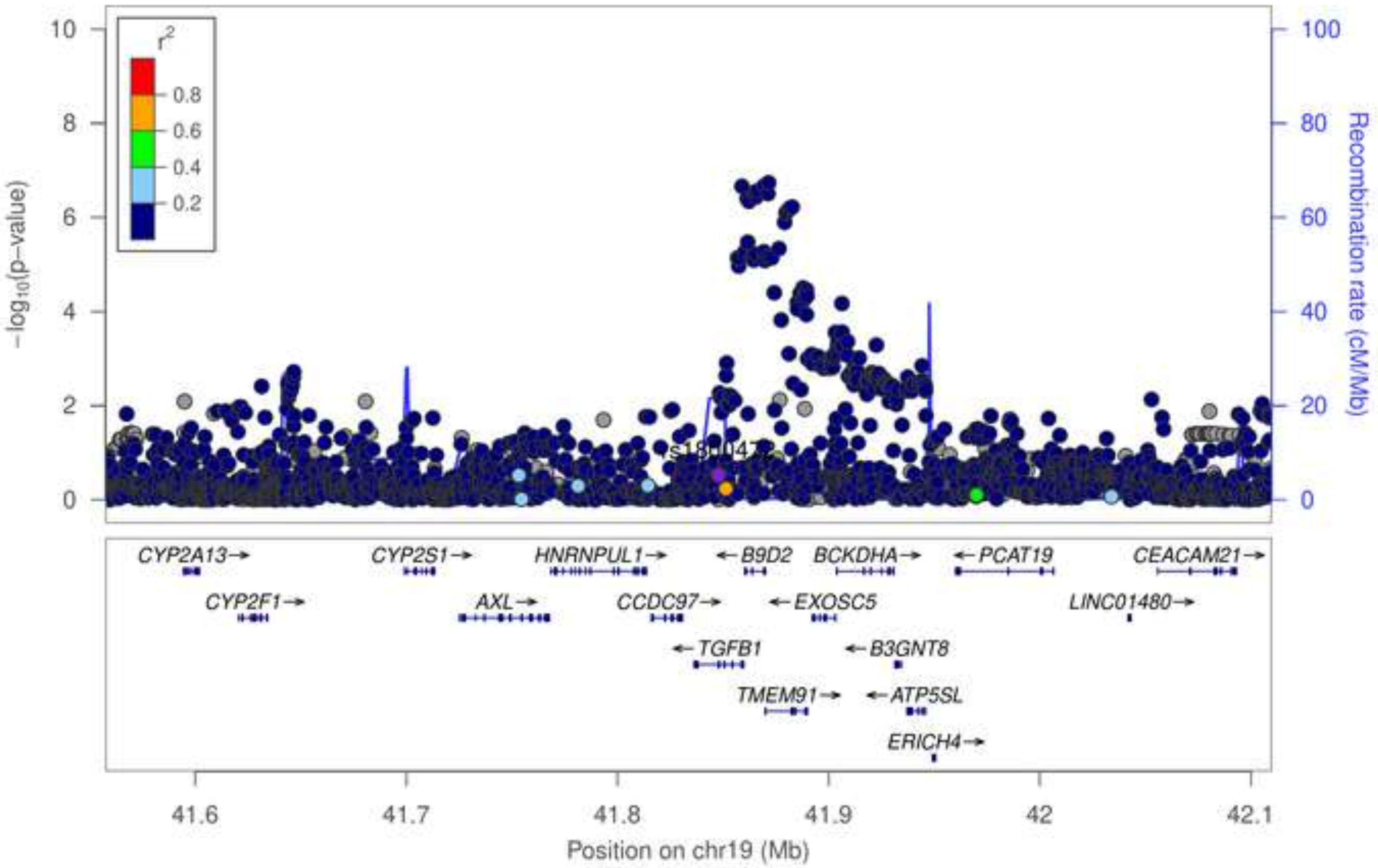

SCALLOP: LAP (TGFB1)−rs1800472

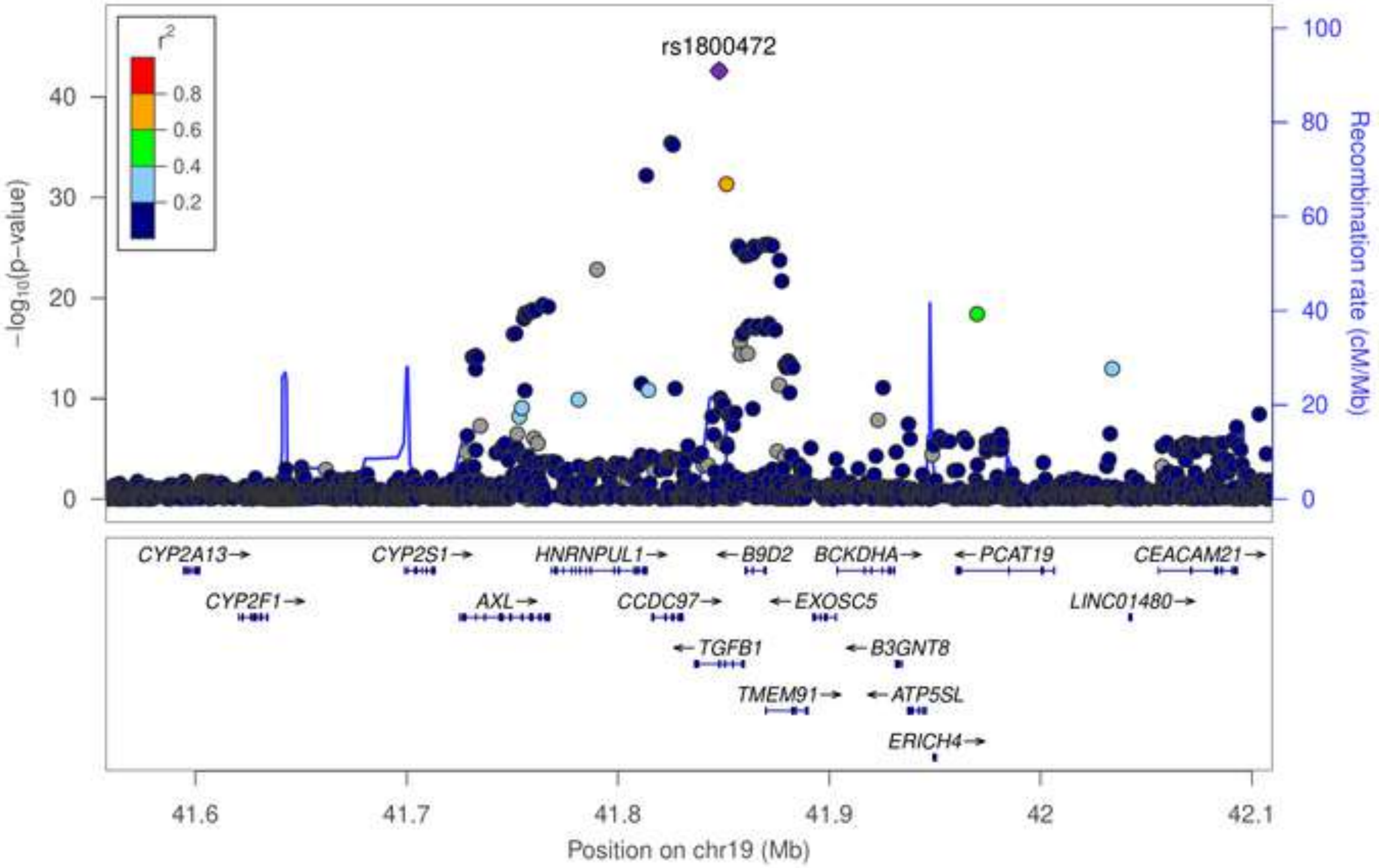

eQTLGen: MCP−2 (CCL8)−rs1133763

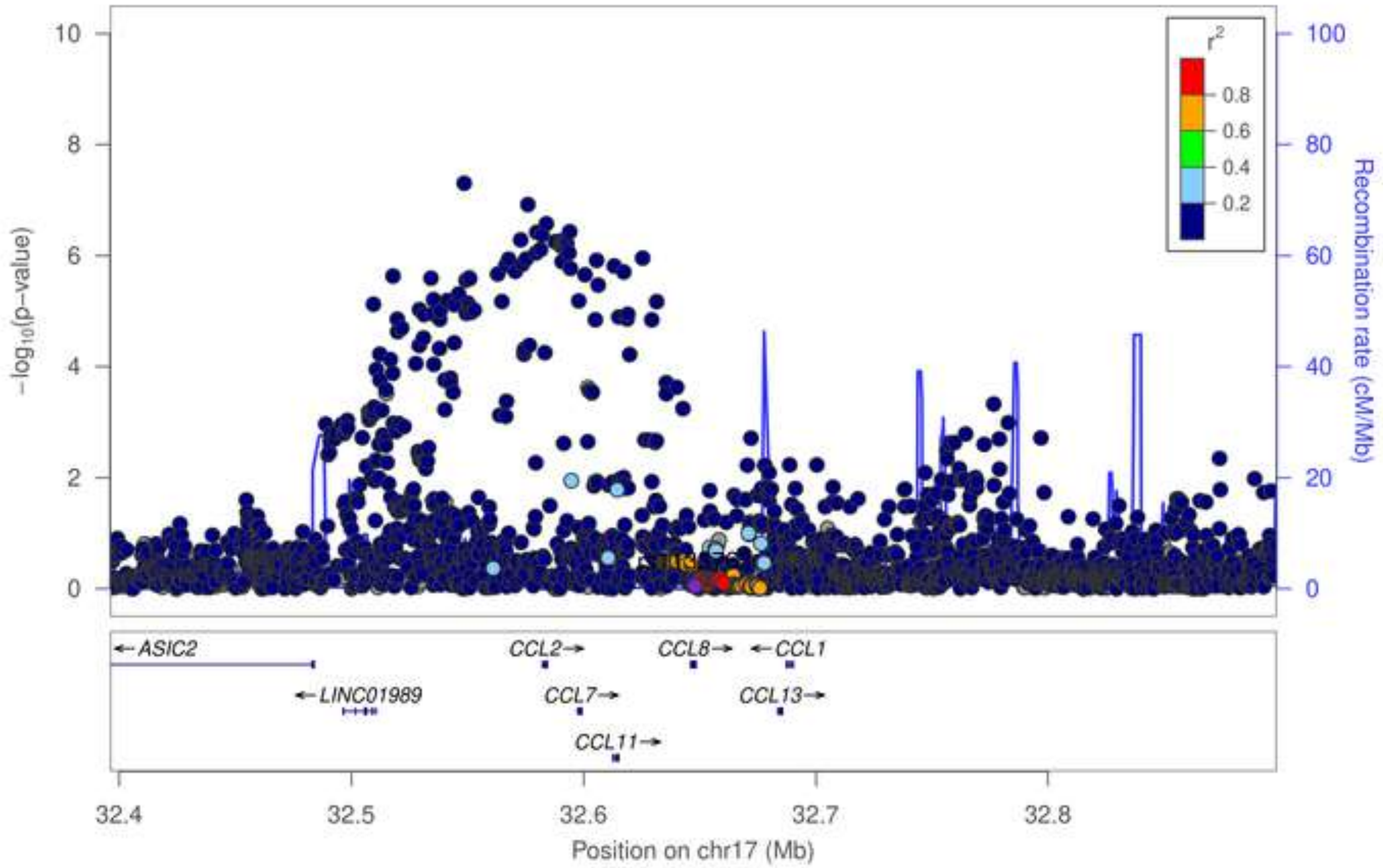

SCALLOP: MCP−2 (CCL8)−rs1133763

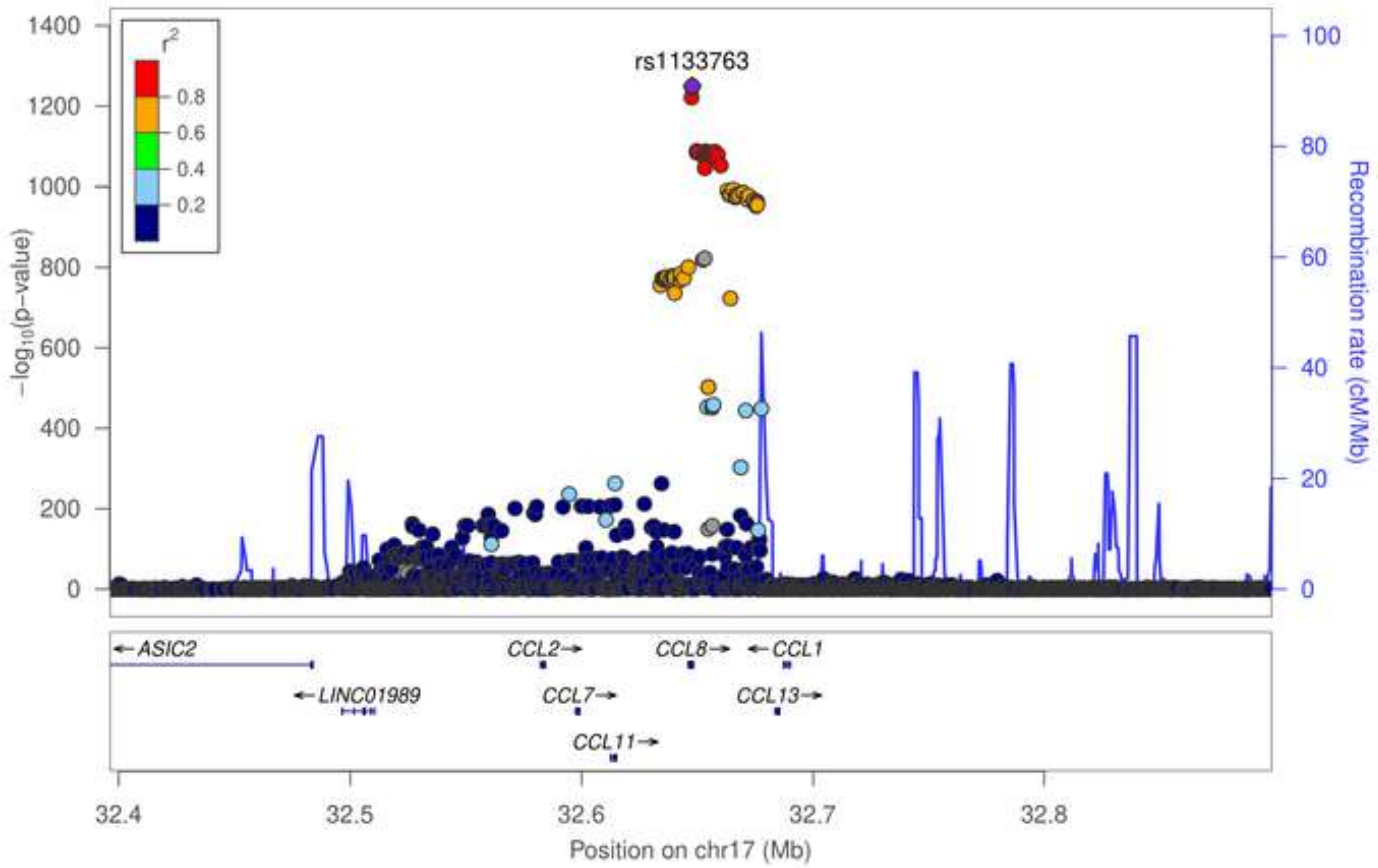

eQTLGen: MIP−1 (CCL3)−rs8951

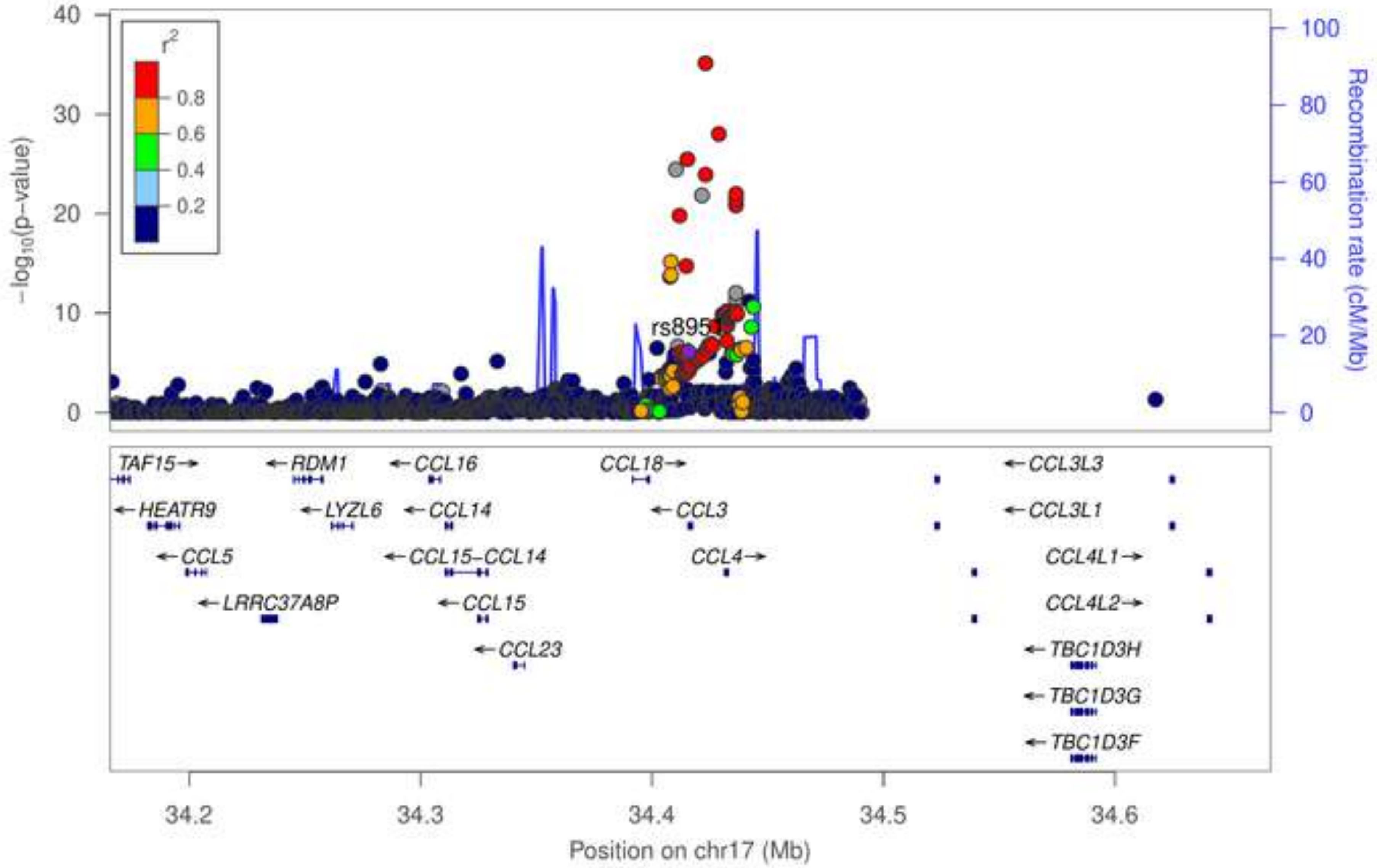

SCALLOP: MIP−1 (CCL3)−rs8951

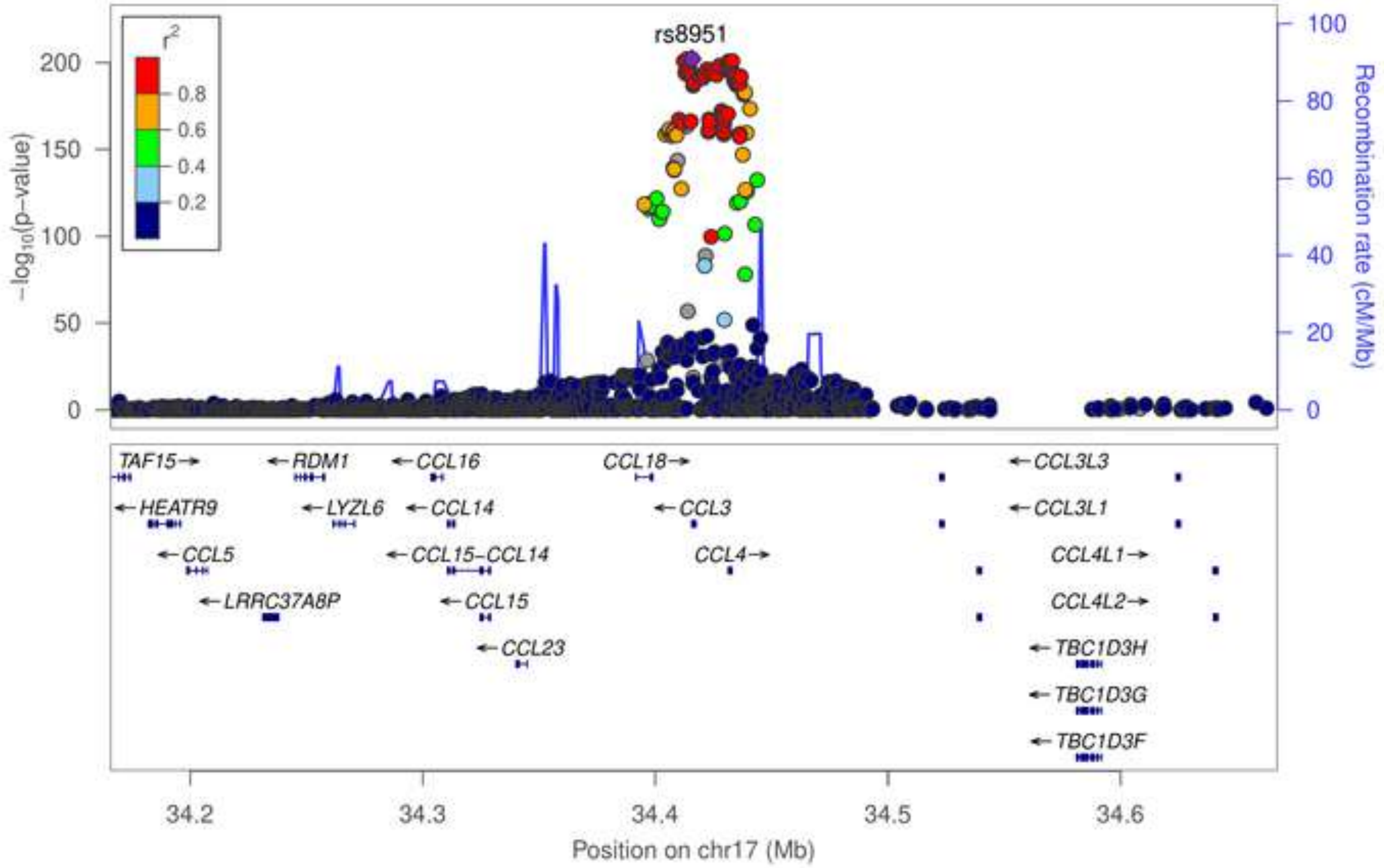

eQTLGen: MMP−1 (MMP1)−rs471994

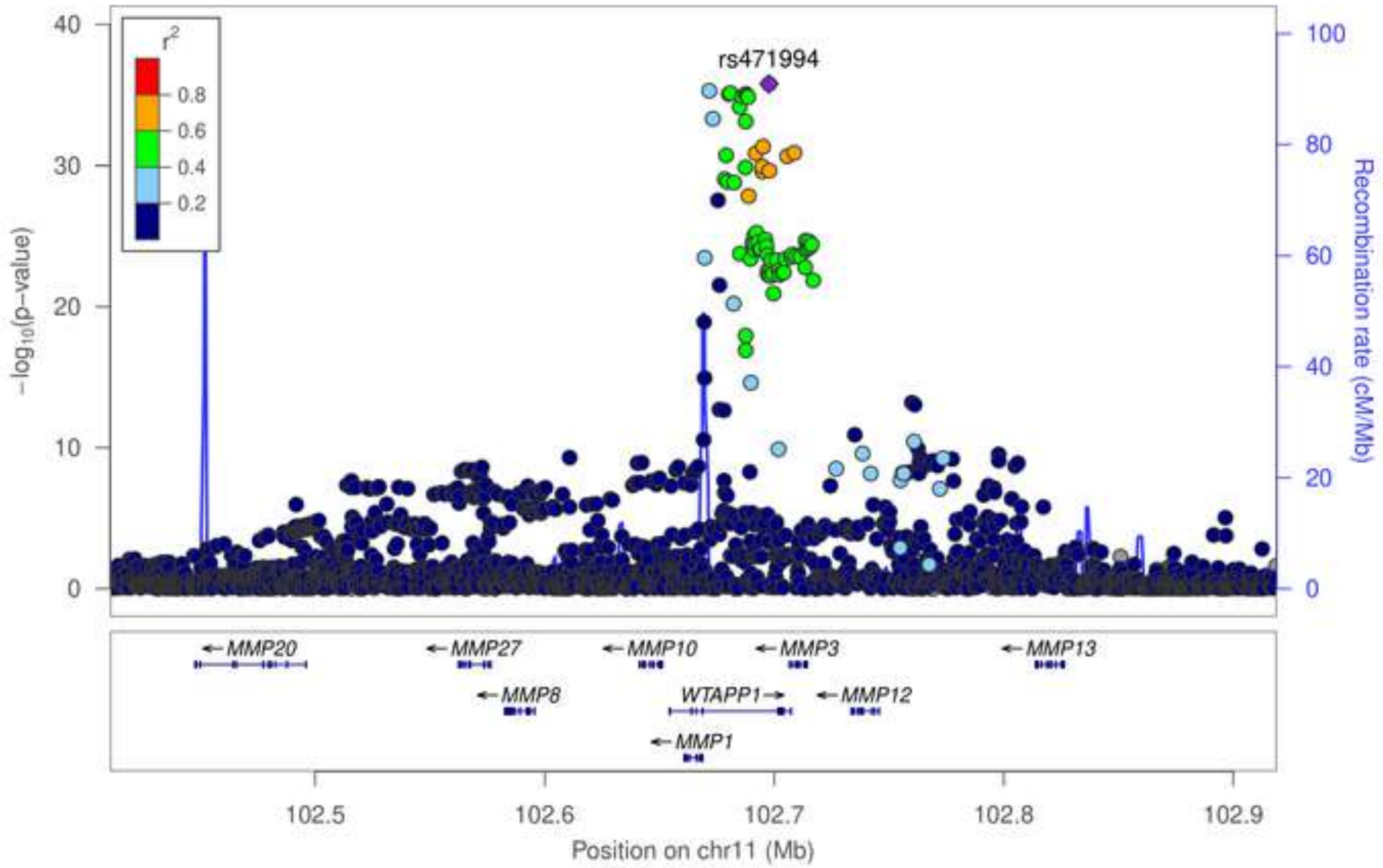

SCALLOP: MMP−1 (MMP1)−rs471994

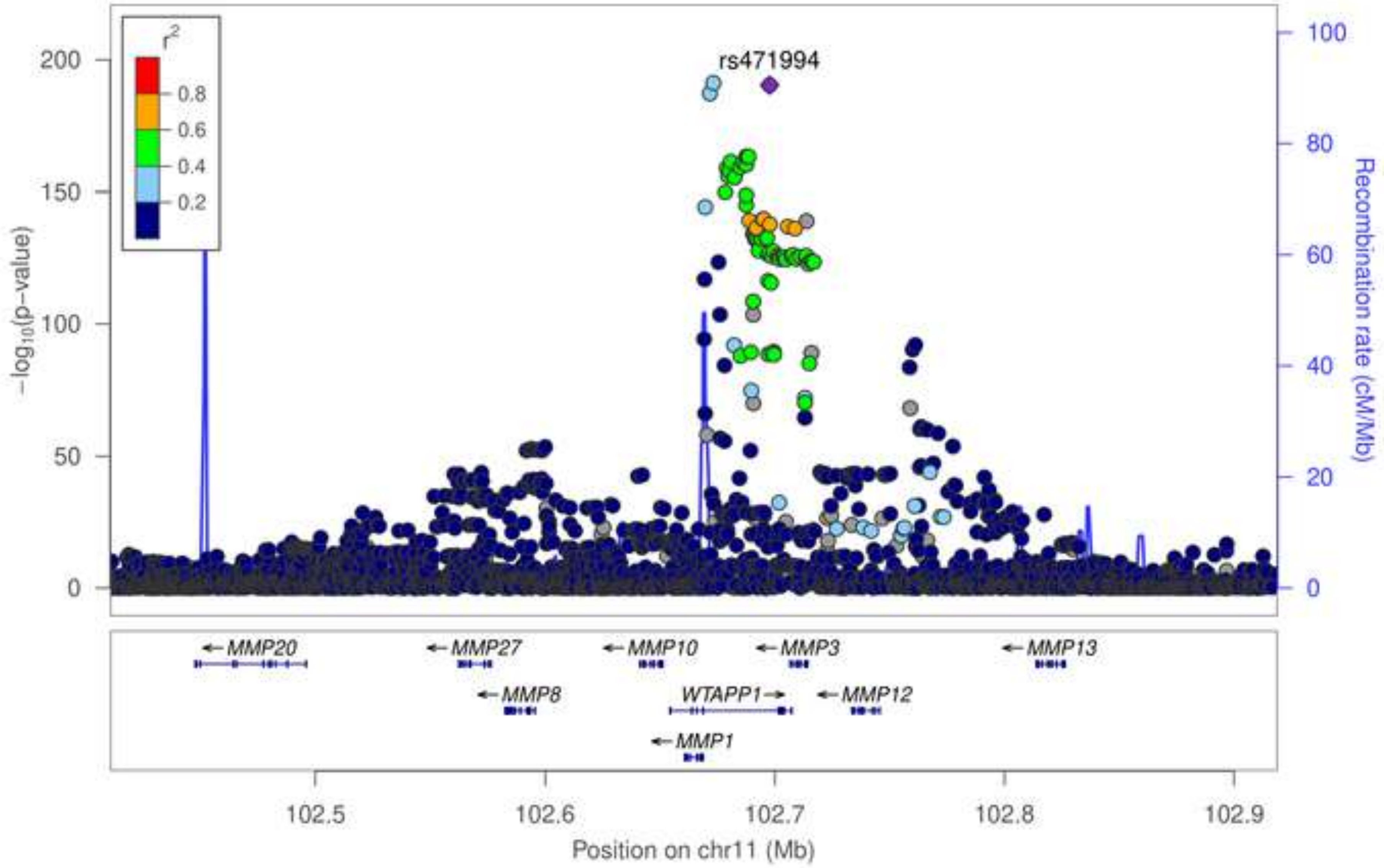

eQTLGen: PD−L1 (CD274)−rs822335

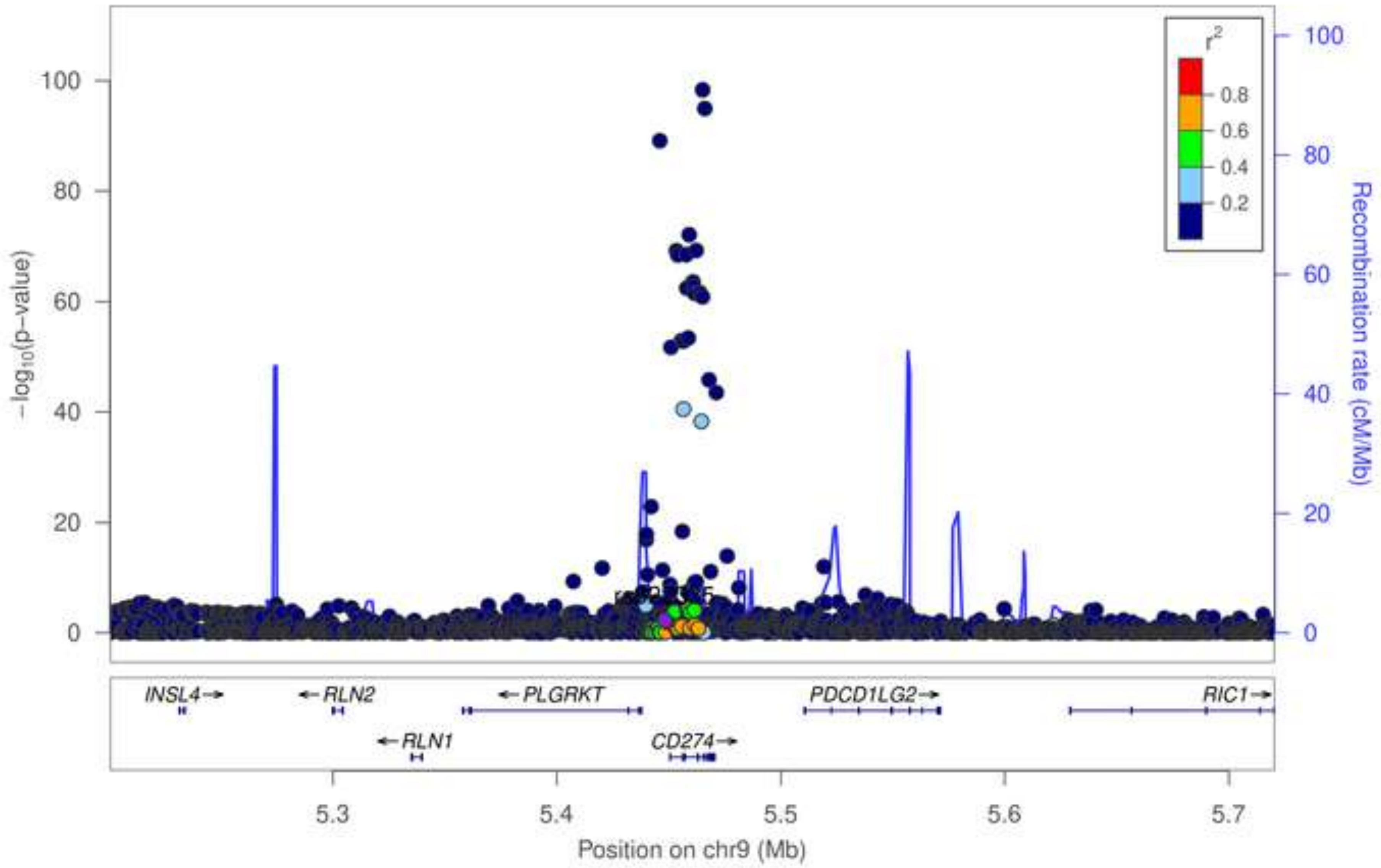

SCALLOP: PD−L1 (CD274)−rs822335

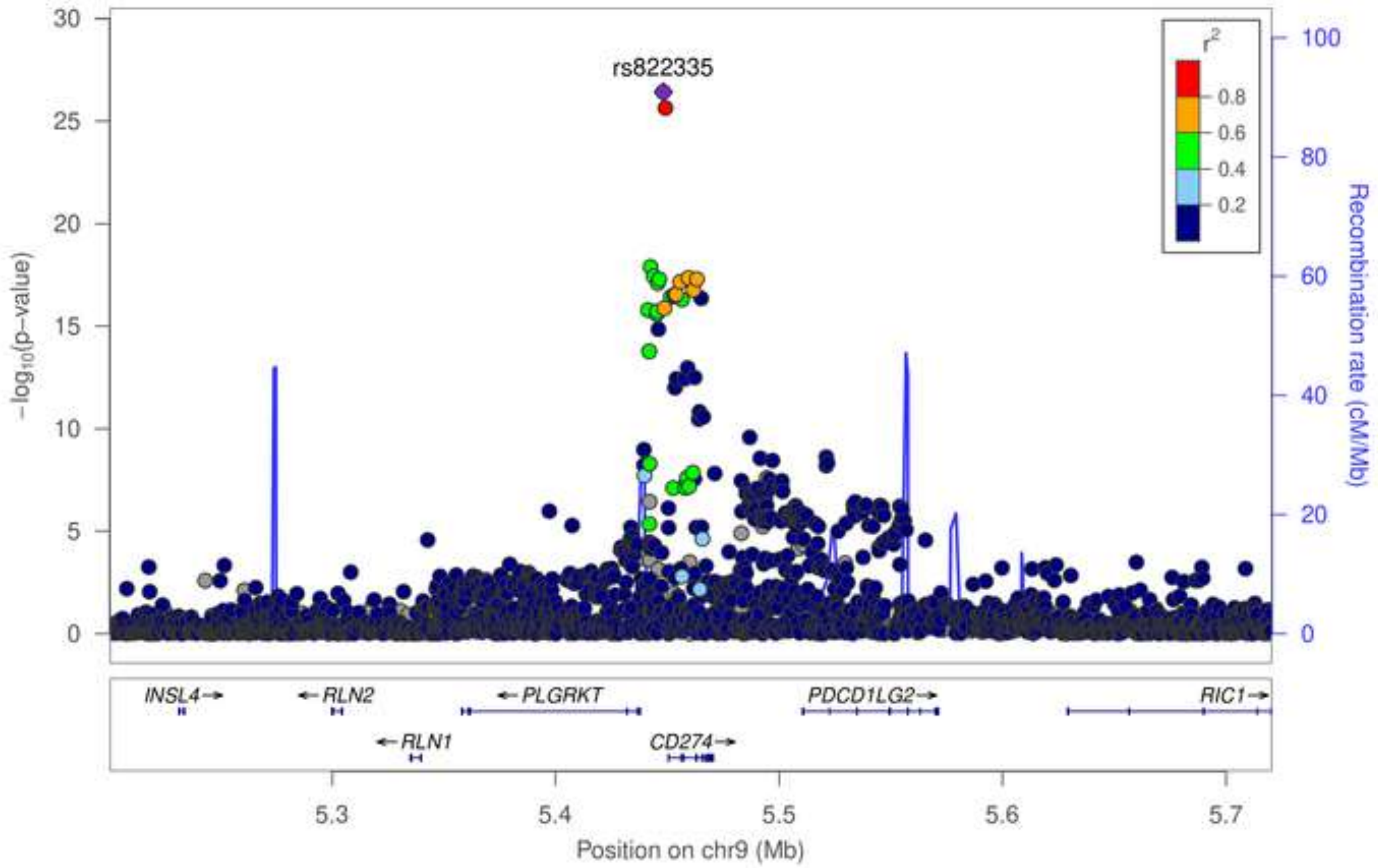

eQTLGen: SIRT2 (SIRT2)–rs144373891

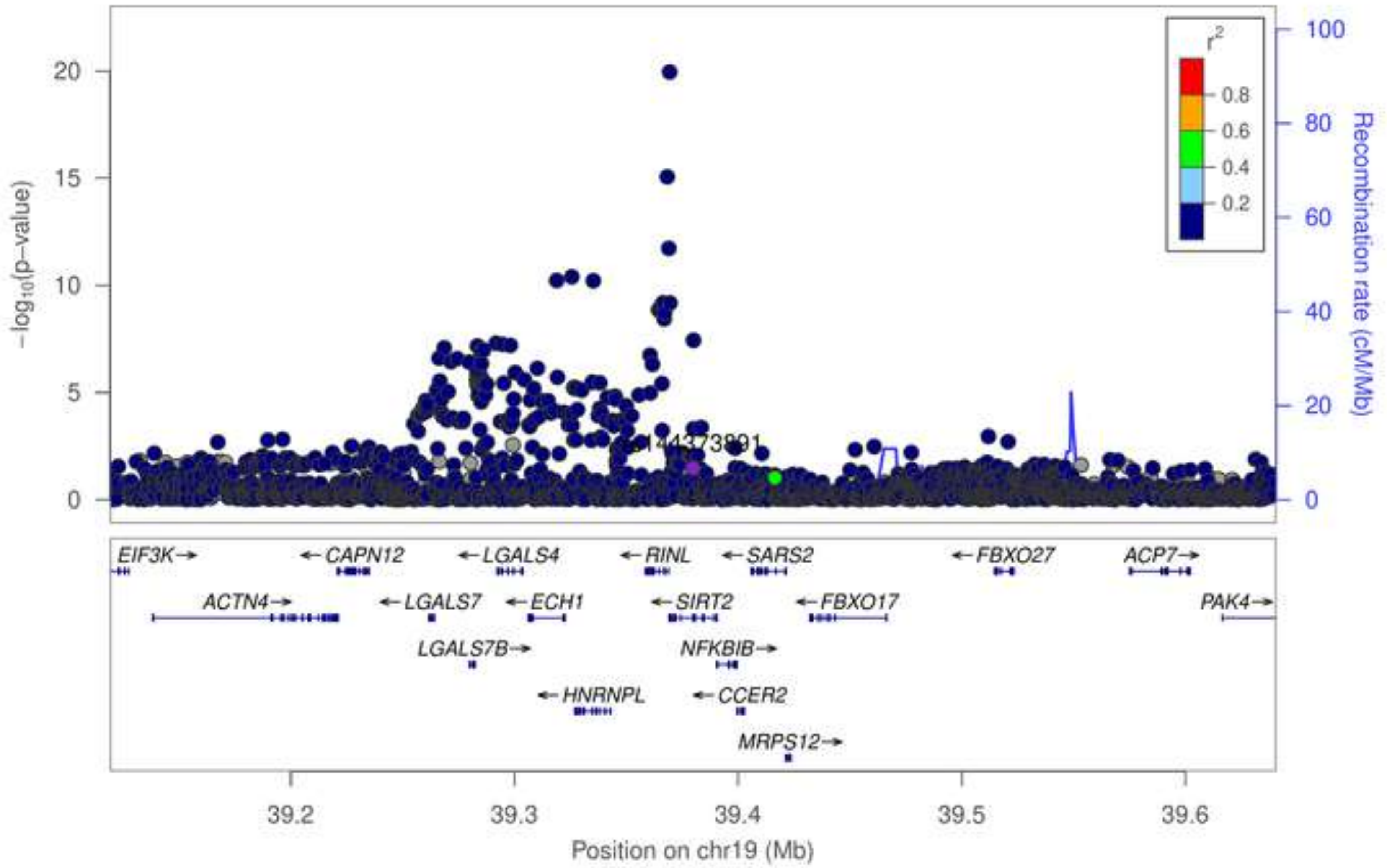

SCALLOP: SIRT2 (SIRT2)–rs144373891

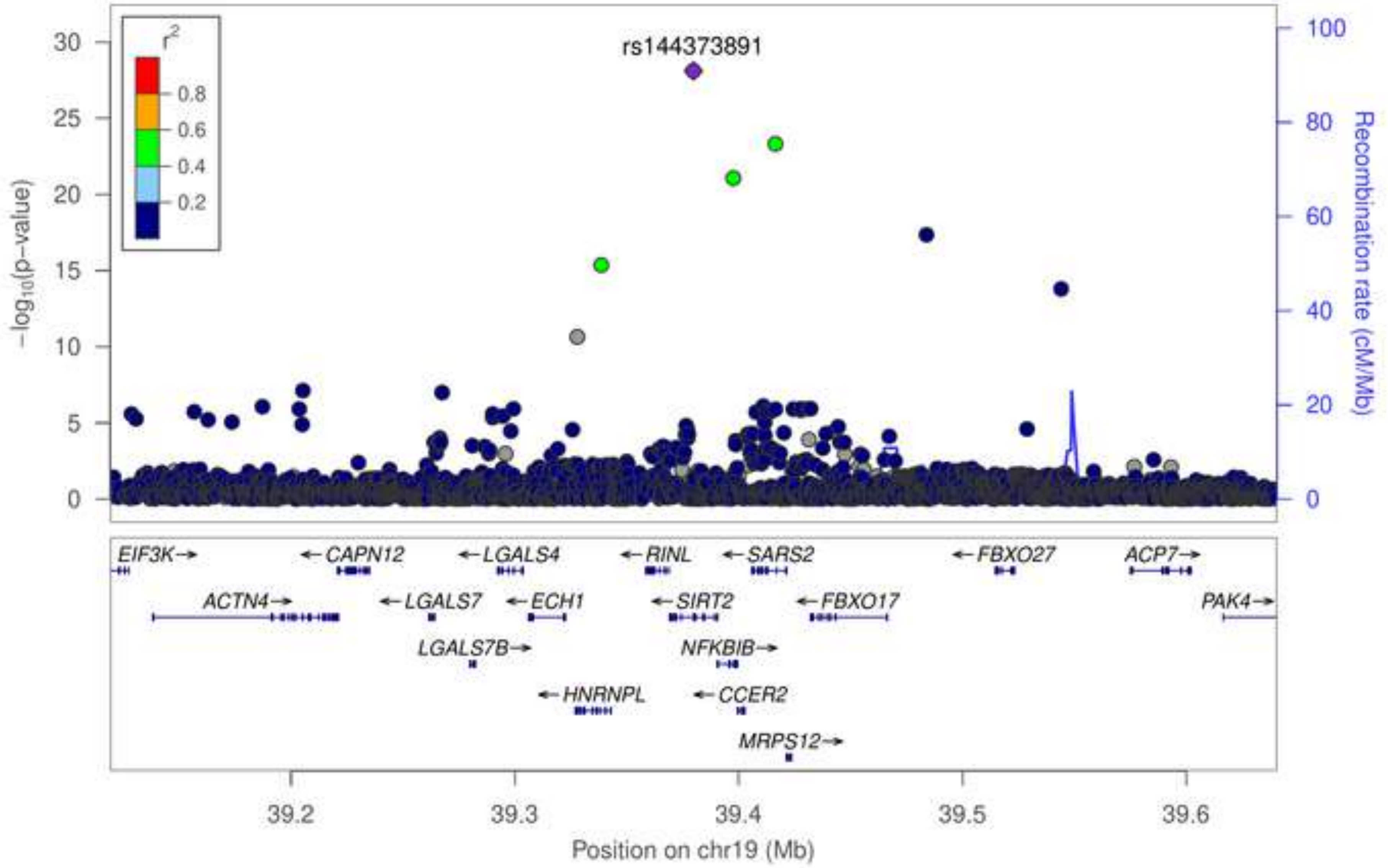

eQTLGen: SLAMF1 (SLAMF1)–rs60094514

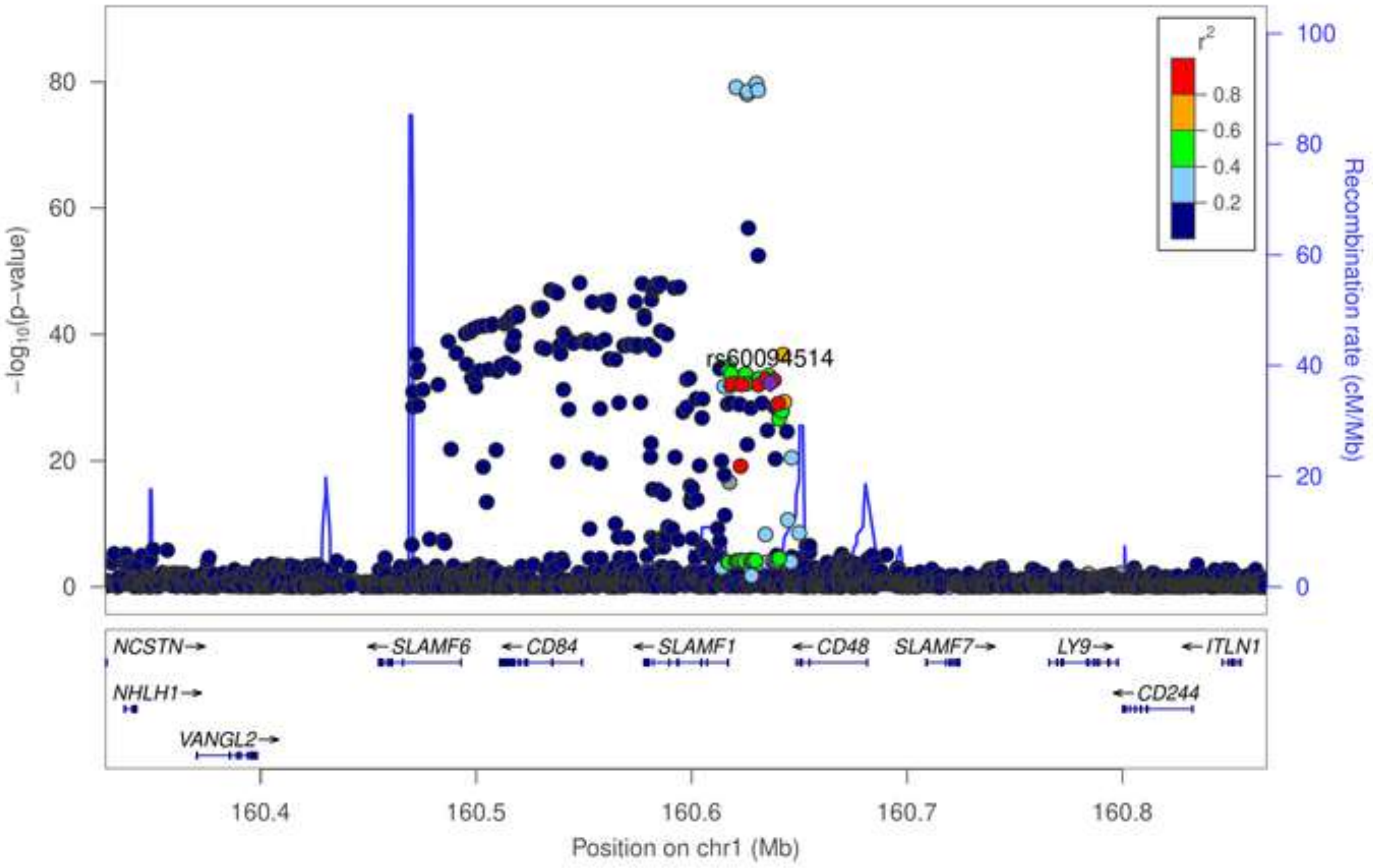

SCALLOP: SLAMF1 (SLAMF1)–rs60094514

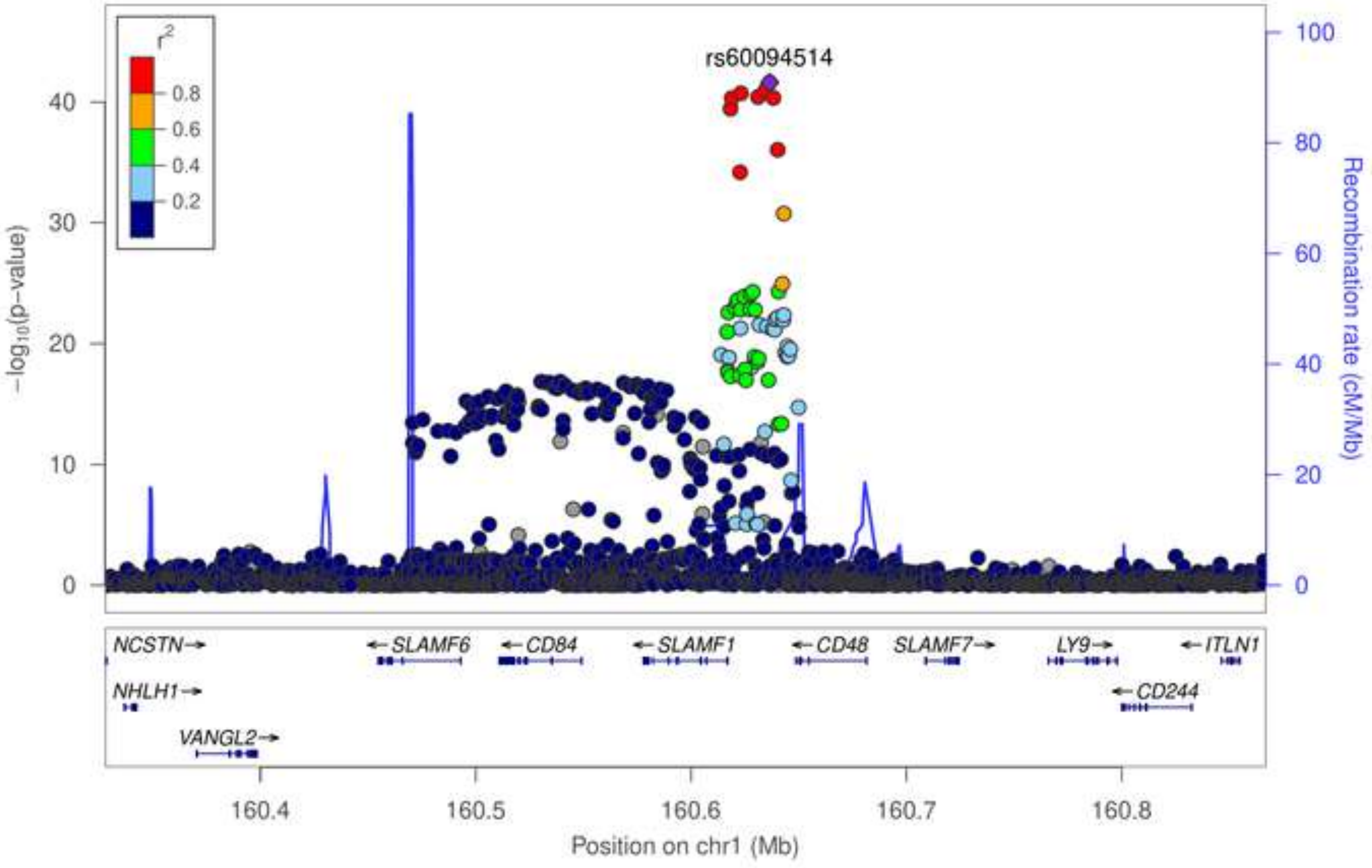

eQTLGen: ST1A1 (SULT1A1)–rs149278

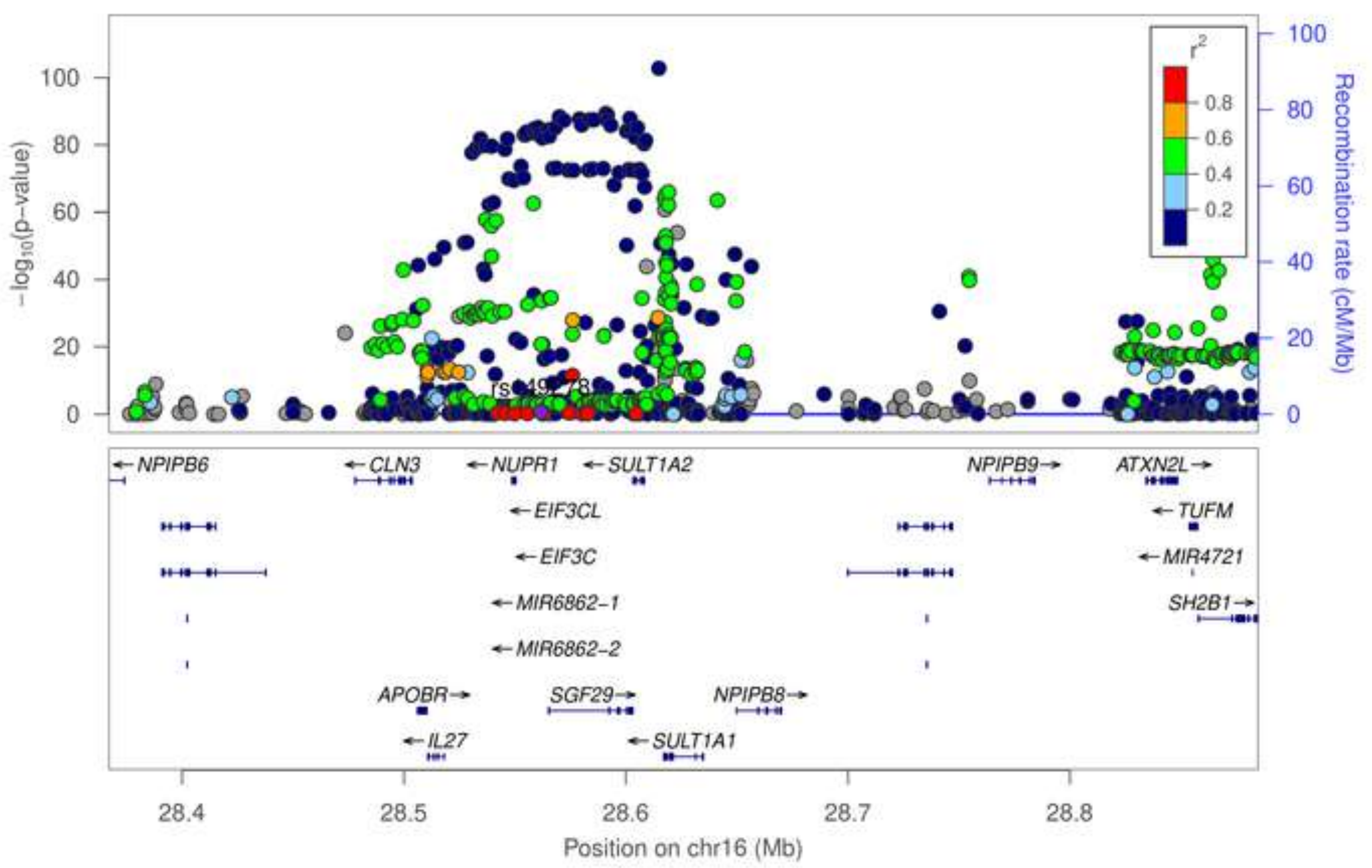

SCALLOP: ST1A1 (SULT1A1)–rs149278

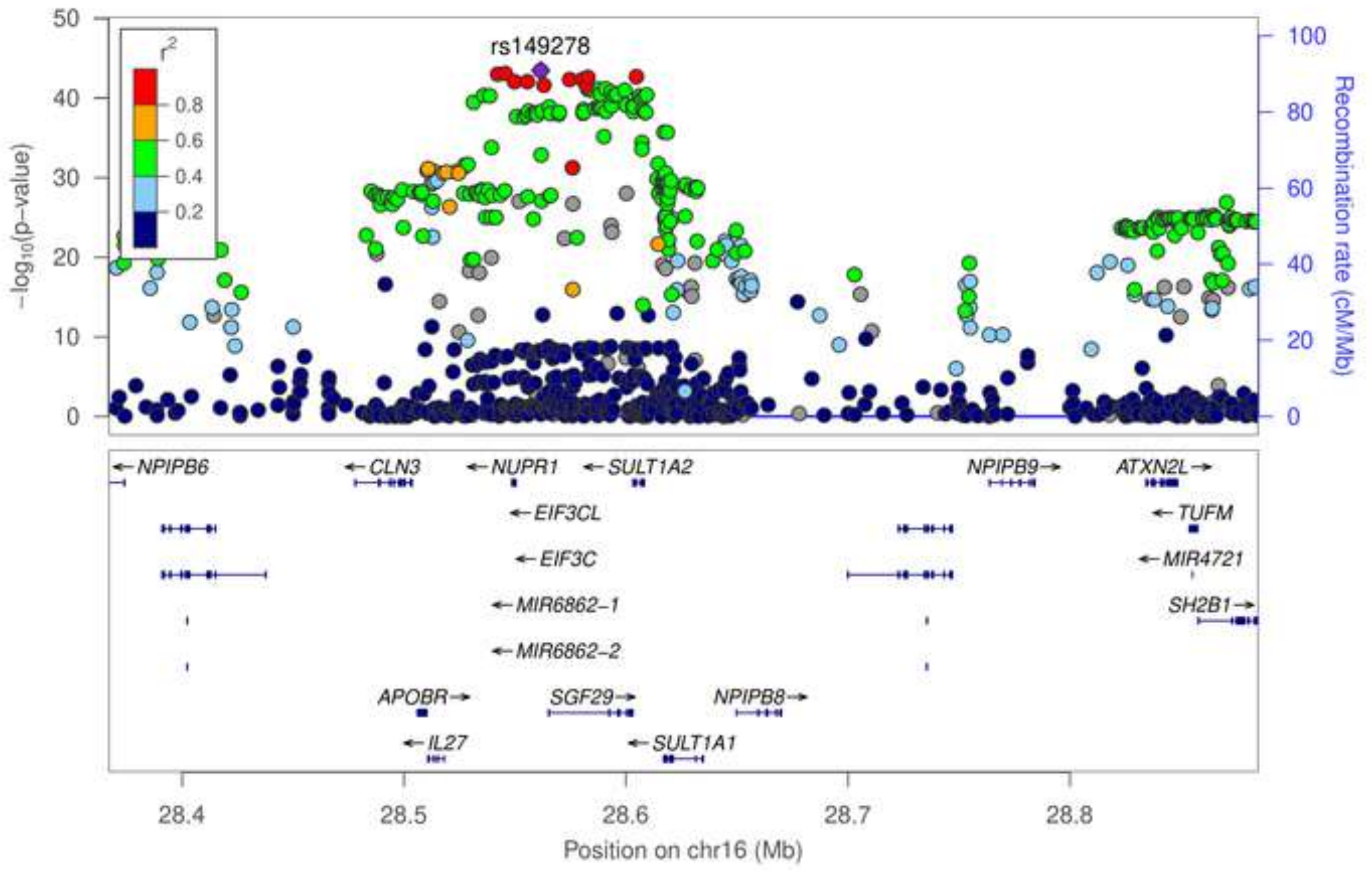

eQTLGen: TGF−alpha (TGFA)−rs72912115

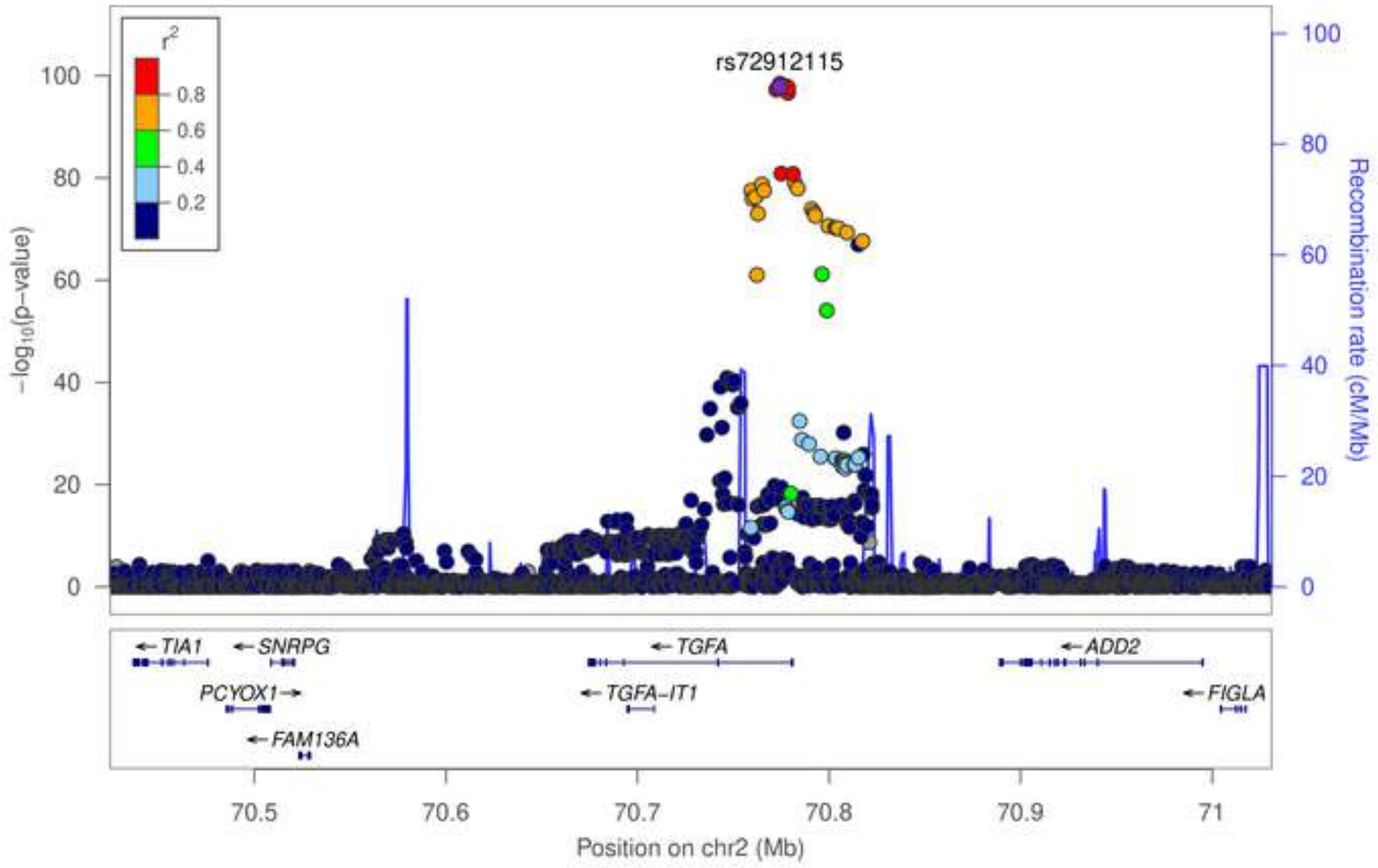

SCALLOP: TGF−alpha (TGFA)−rs72912115

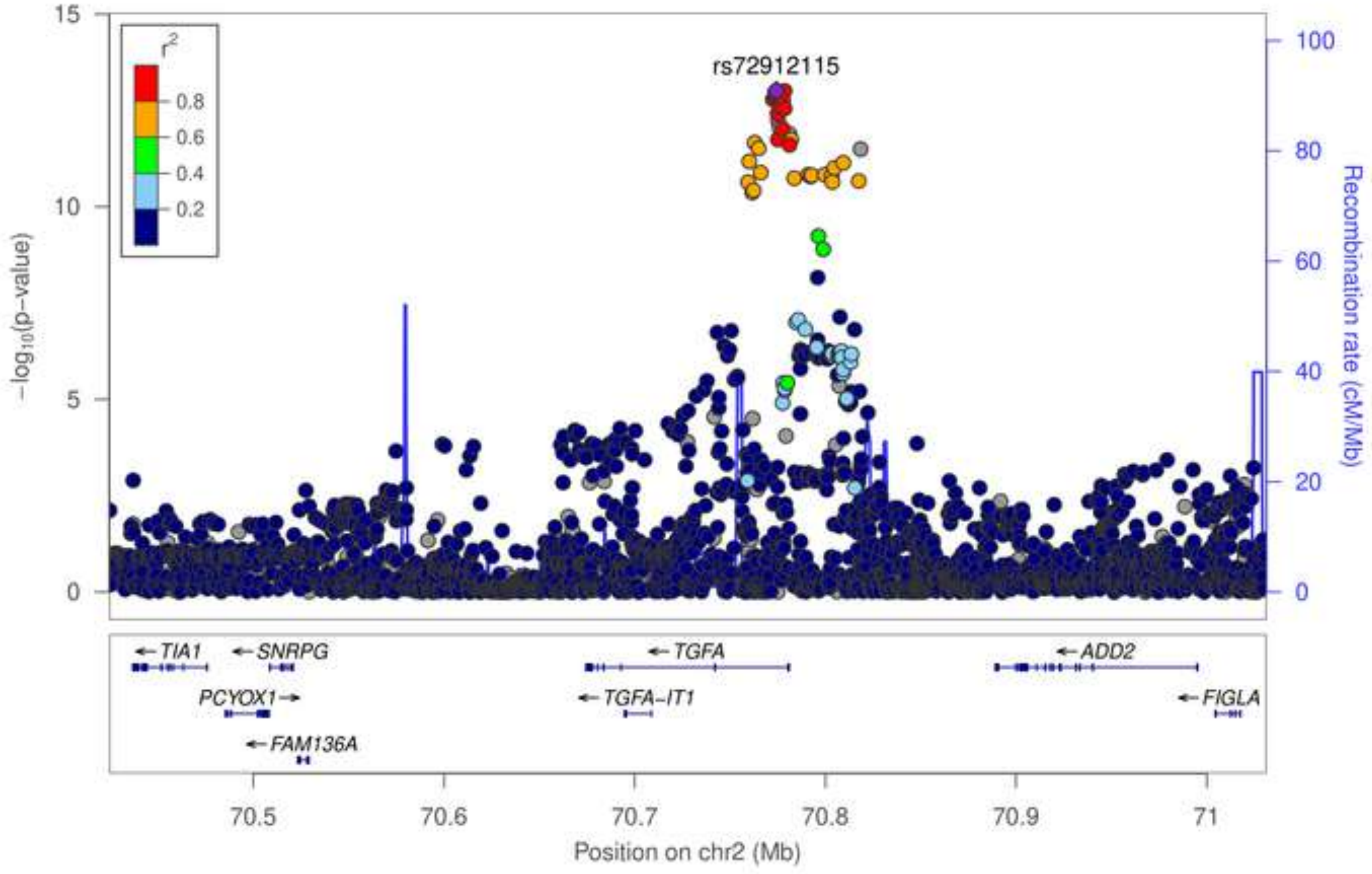

eQTLGen: TNFB (LTA)−rs2229092

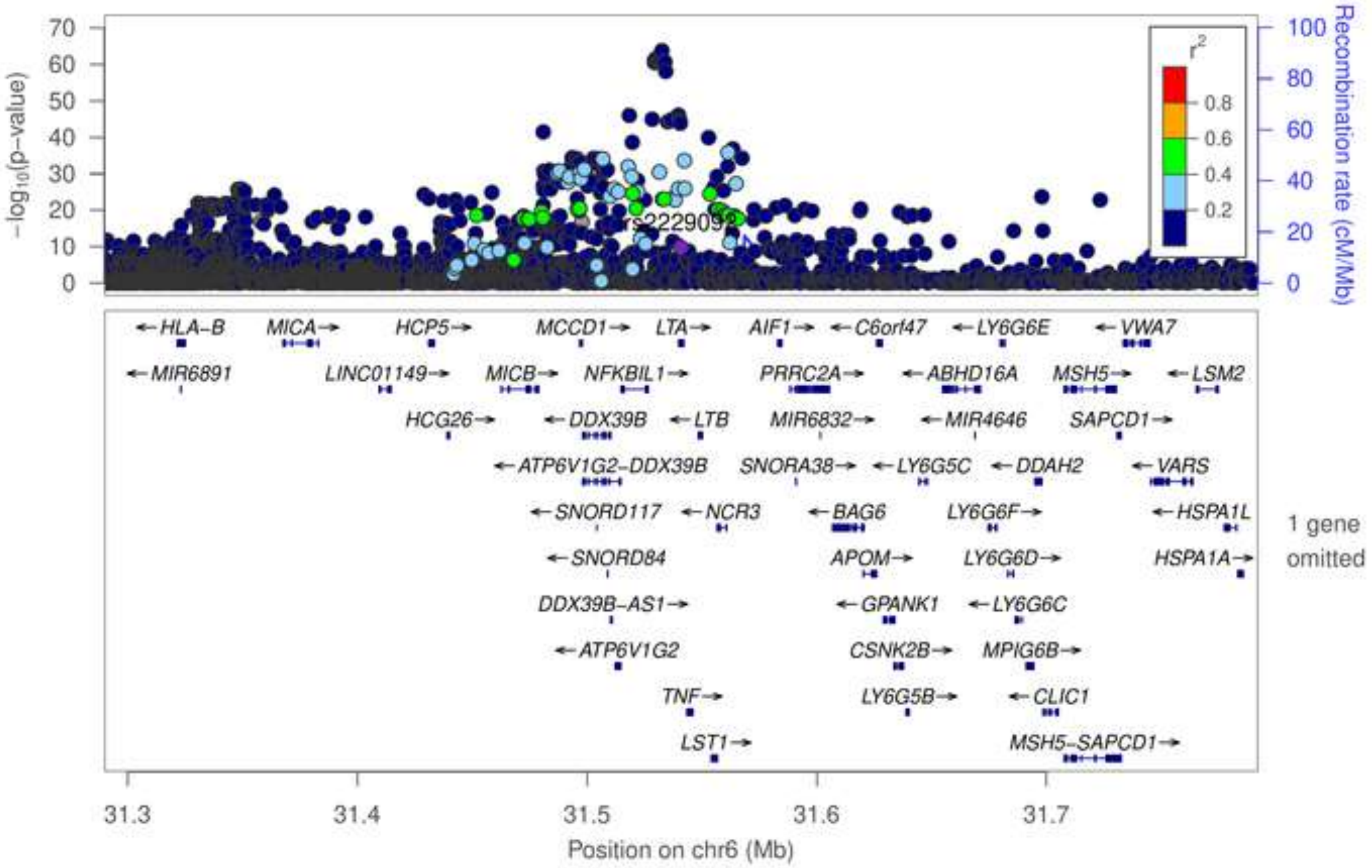

SCALLOP: TNFB (LTA)−rs2229092

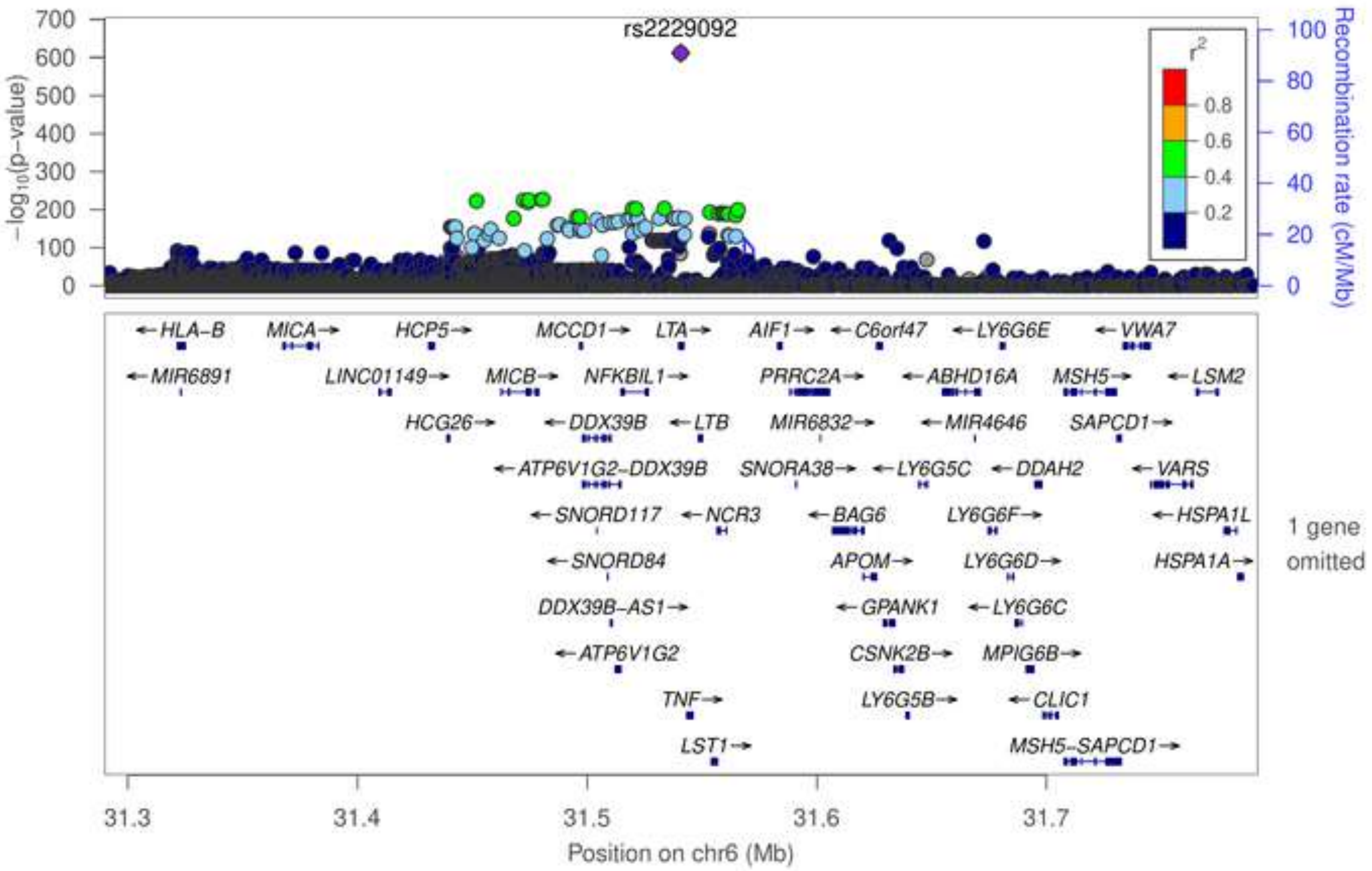

eQTLGen: TNFRSF9 (TNFRSF9)–rs1776354

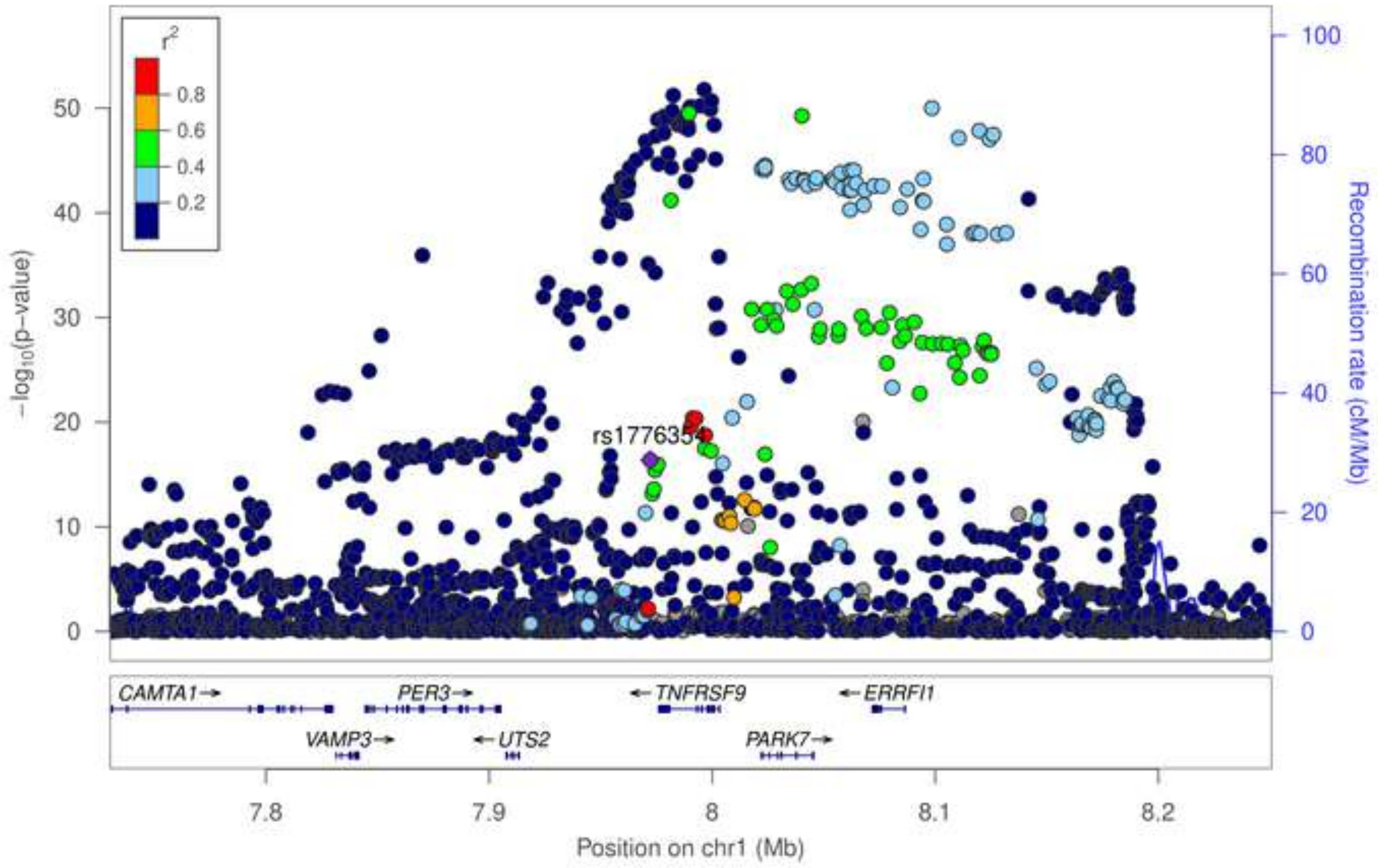

SCALLOP: TNFRSF9 (TNFRSF9)–rs1776354

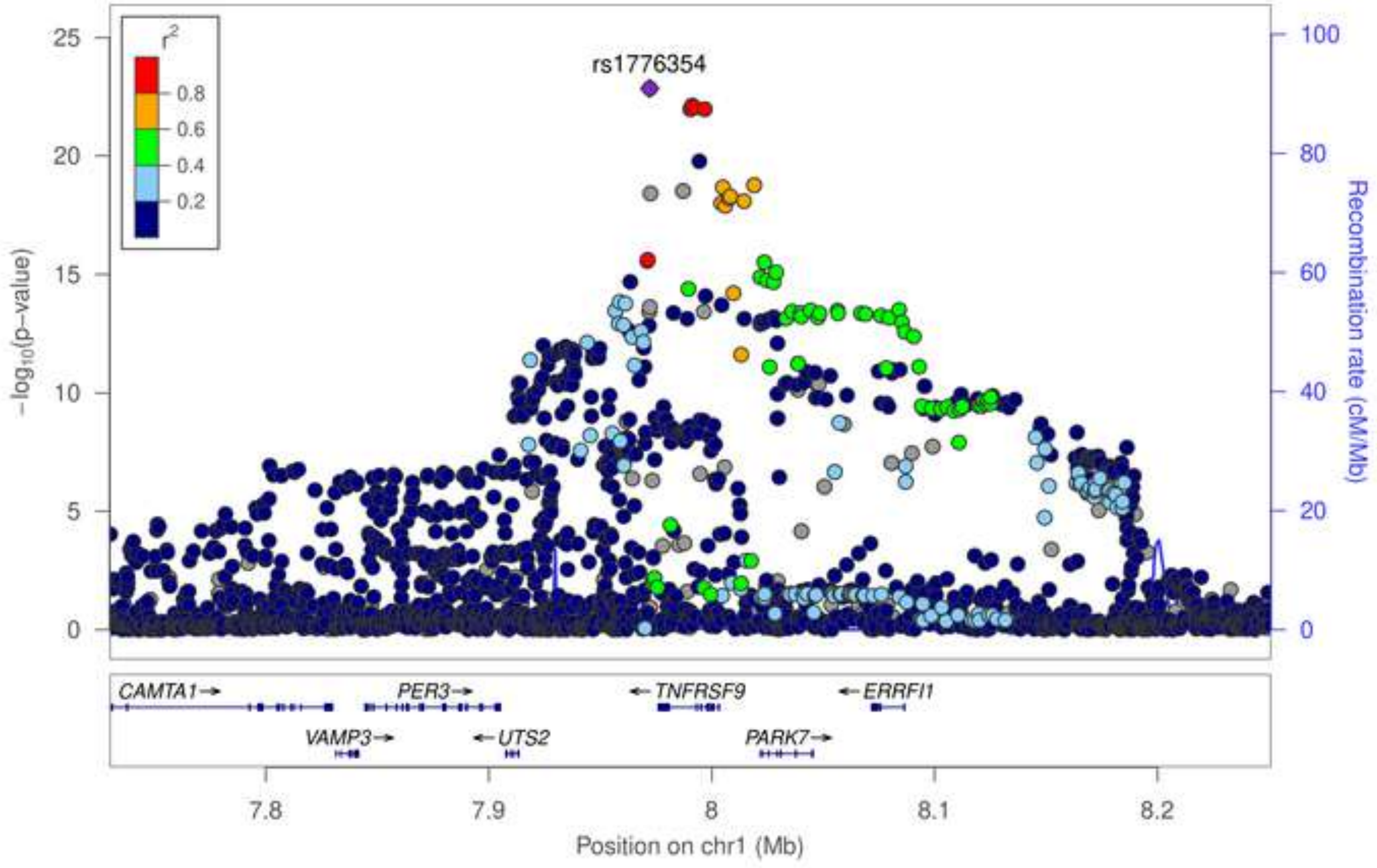

eQTLGen: TNFSF14 (TNFSF14)−rs344562

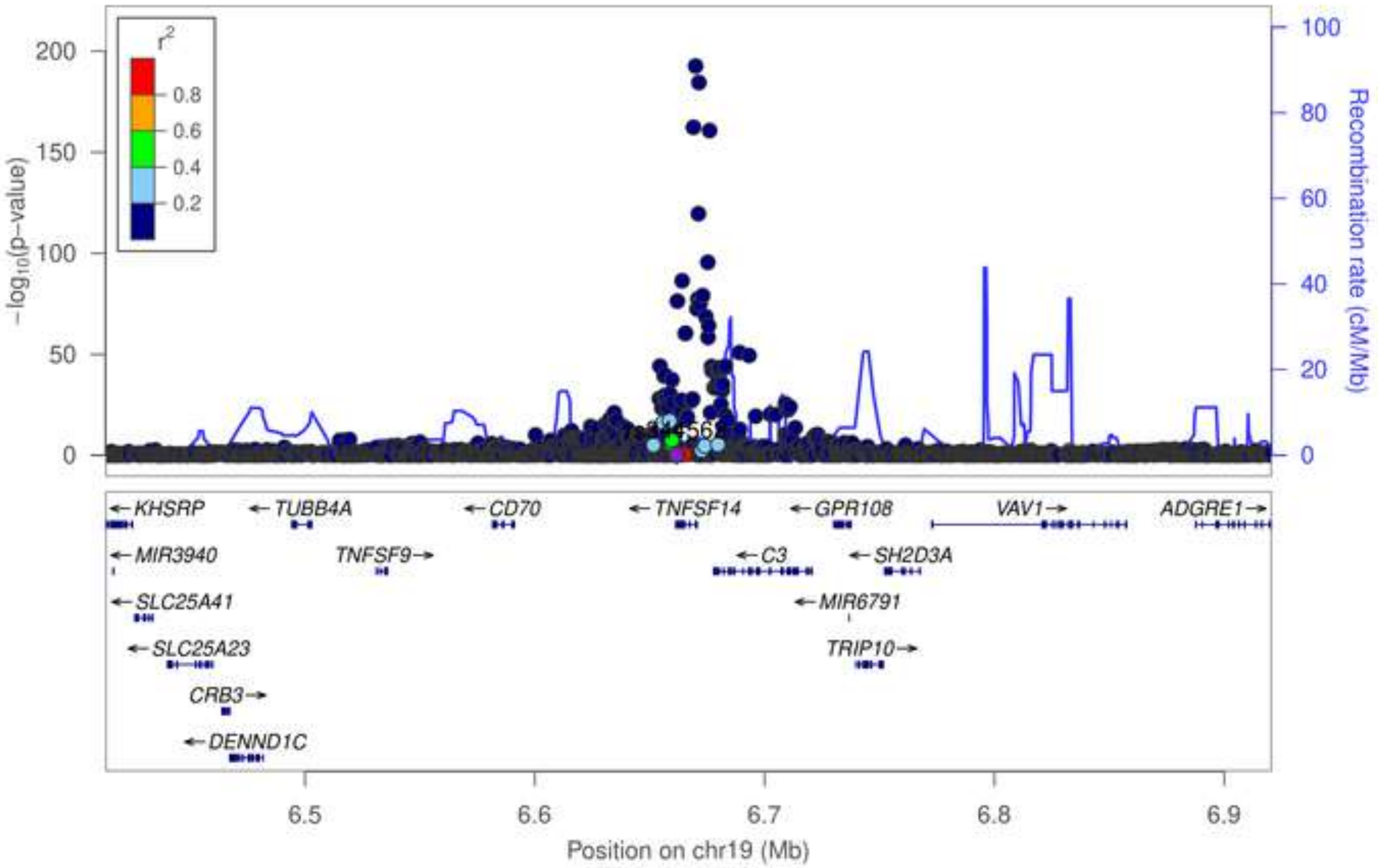

SCALLOP: TNFSF14 (TNFSF14)−rs344562

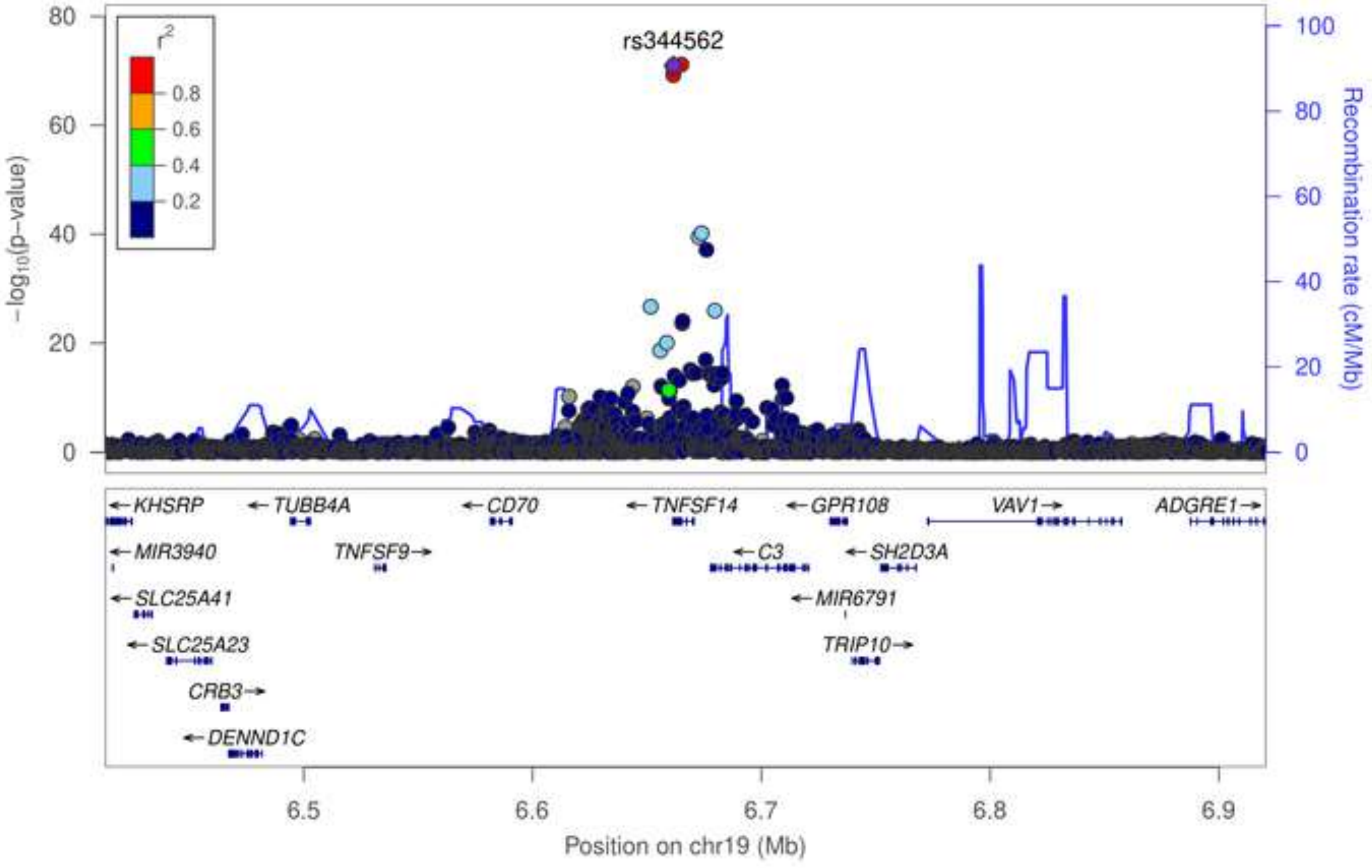

eQTLGen: TRAIL (TNFSF10)−rs574044675

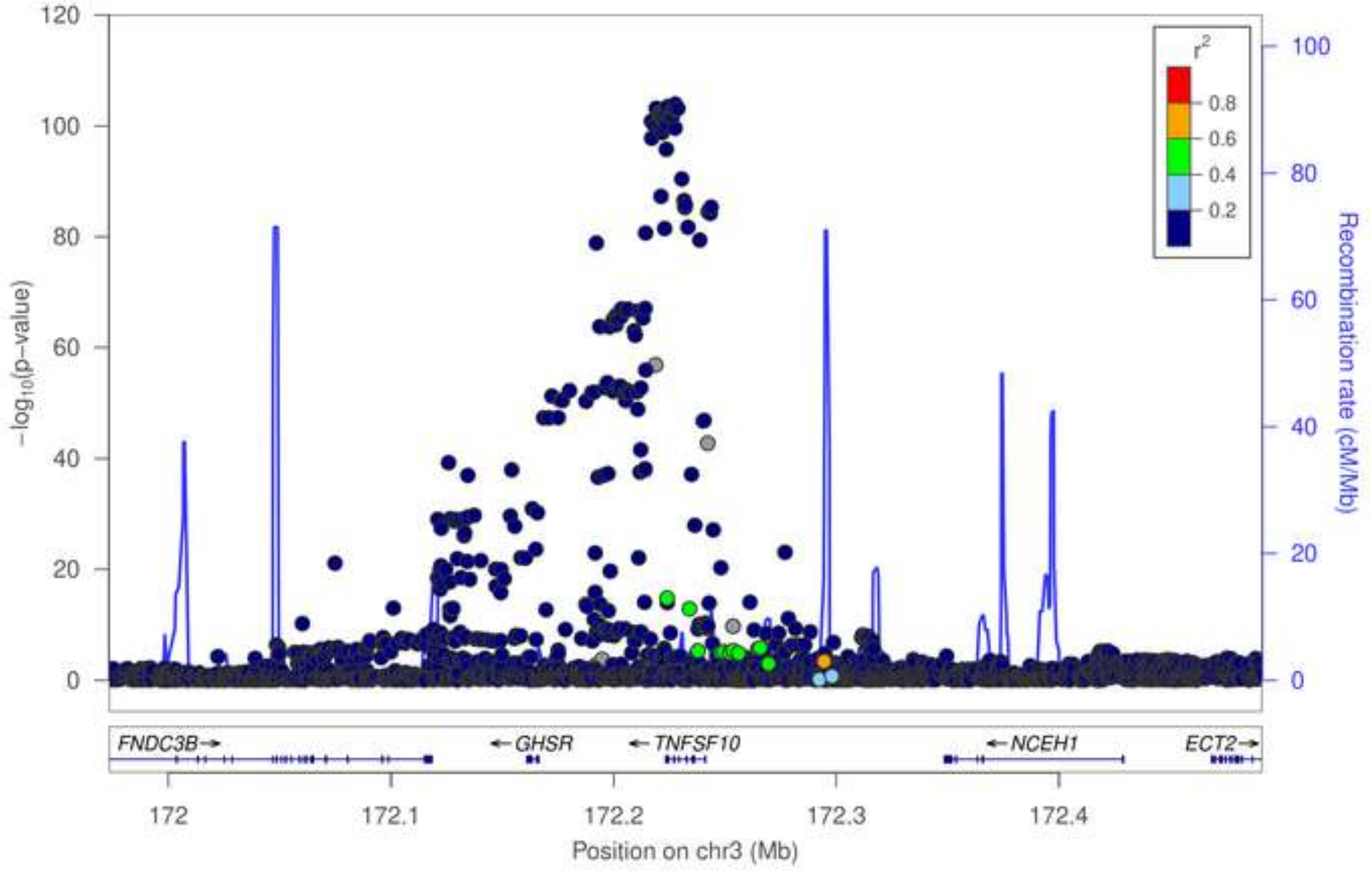

SCALLOP: TRAIL (TNFSF10)−rs574044675

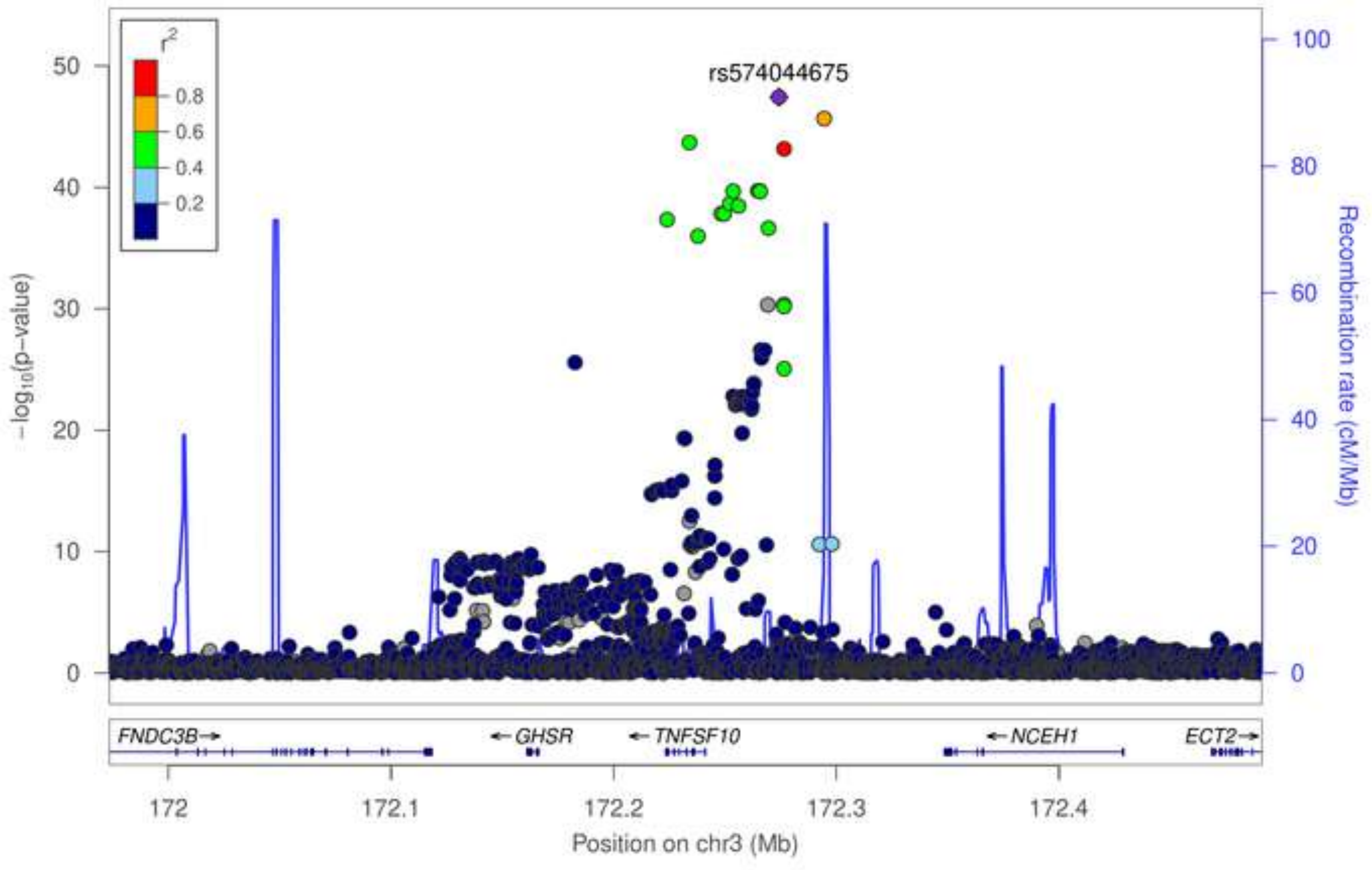

eQTLGen: TRANCE (TNFSF11)−rs4512994

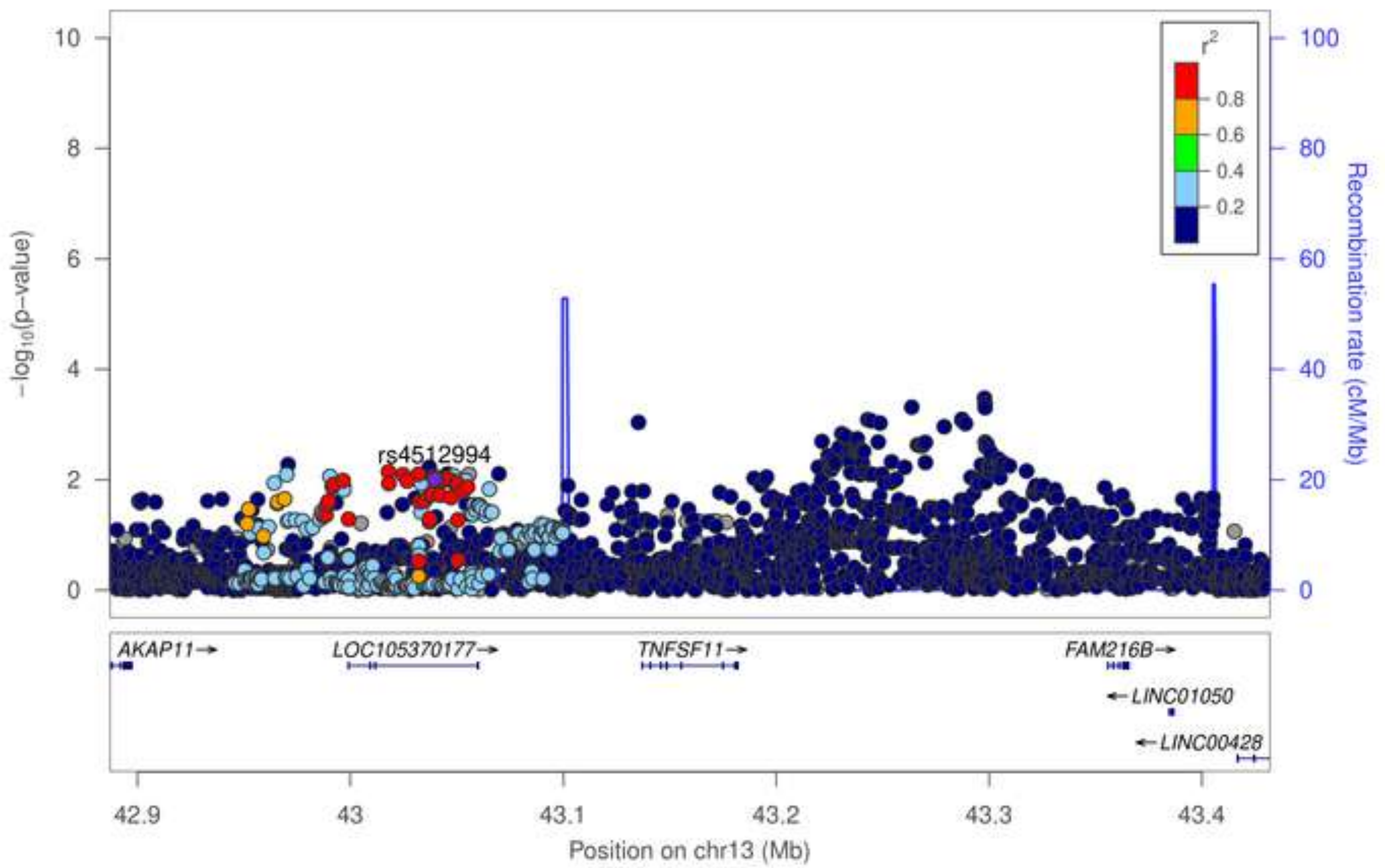

SCALLOP: TRANCE (TNFSF11)−rs4512994

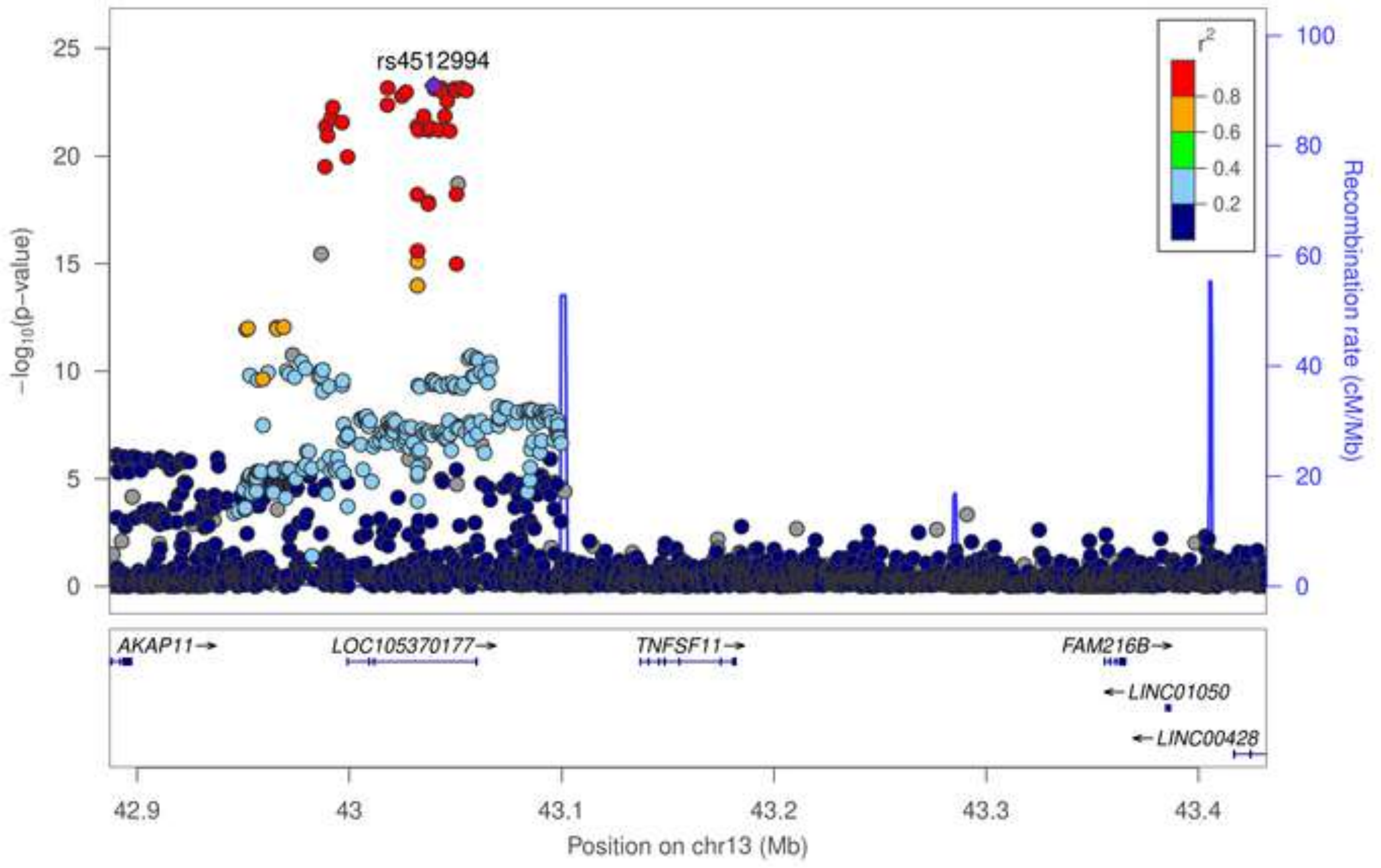

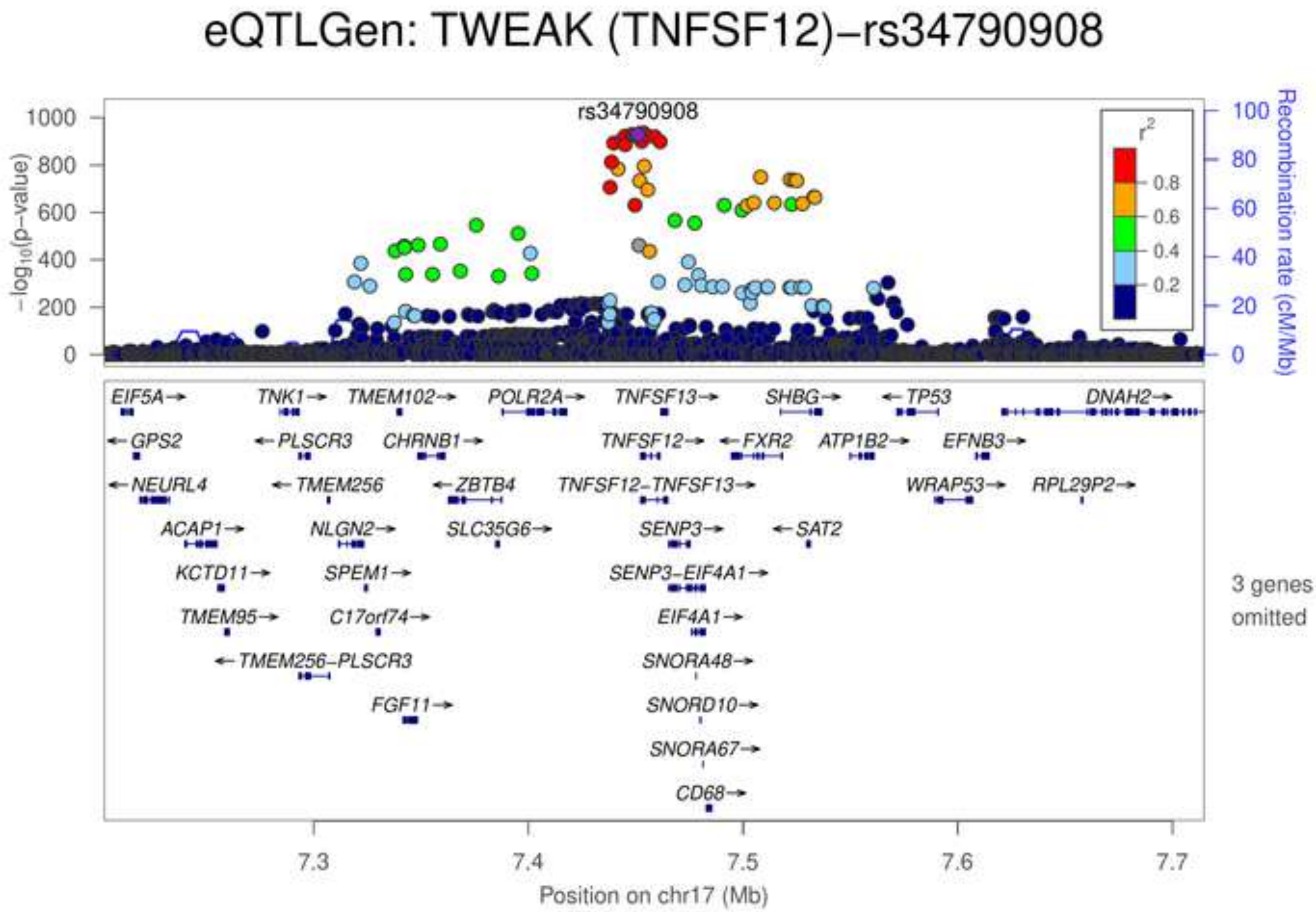
eQTLGen: TWEAK (TNFSF12)−rs34790908
rs34790908
−log10(p−value)
Recombination rate (cM/Mb)
r²
0.8
0.6
0.4
0.2
EIF5A→
TNK1→
TMEM102→
POLR2A→
TNFSF13→
SHBG→
←TP53
DNAH2→
←GPS2
←PLSCR3
CHRNB1→
TNFSF12→
←FXR2
ATP1B2→
EFNB3→
←NEURL4
←TMEM256
←ZBTB4
TNFSF12−TNFSF13→
WRAP53→
RPL29P2→
ACAP1→
NLGN2→
SLC35G6→
SENP3→
←SAT2
KCTD11→
SPEM1→
SENP3−EIF4A1→
3 genes omitted
TMEM95→
C17orf74→
EIF4A1→
←TMEM256−PLSCR3
SNORA48→
FGF11→
SNORD10→
SNORA67→
CD68→
Position on chr17 (Mb)

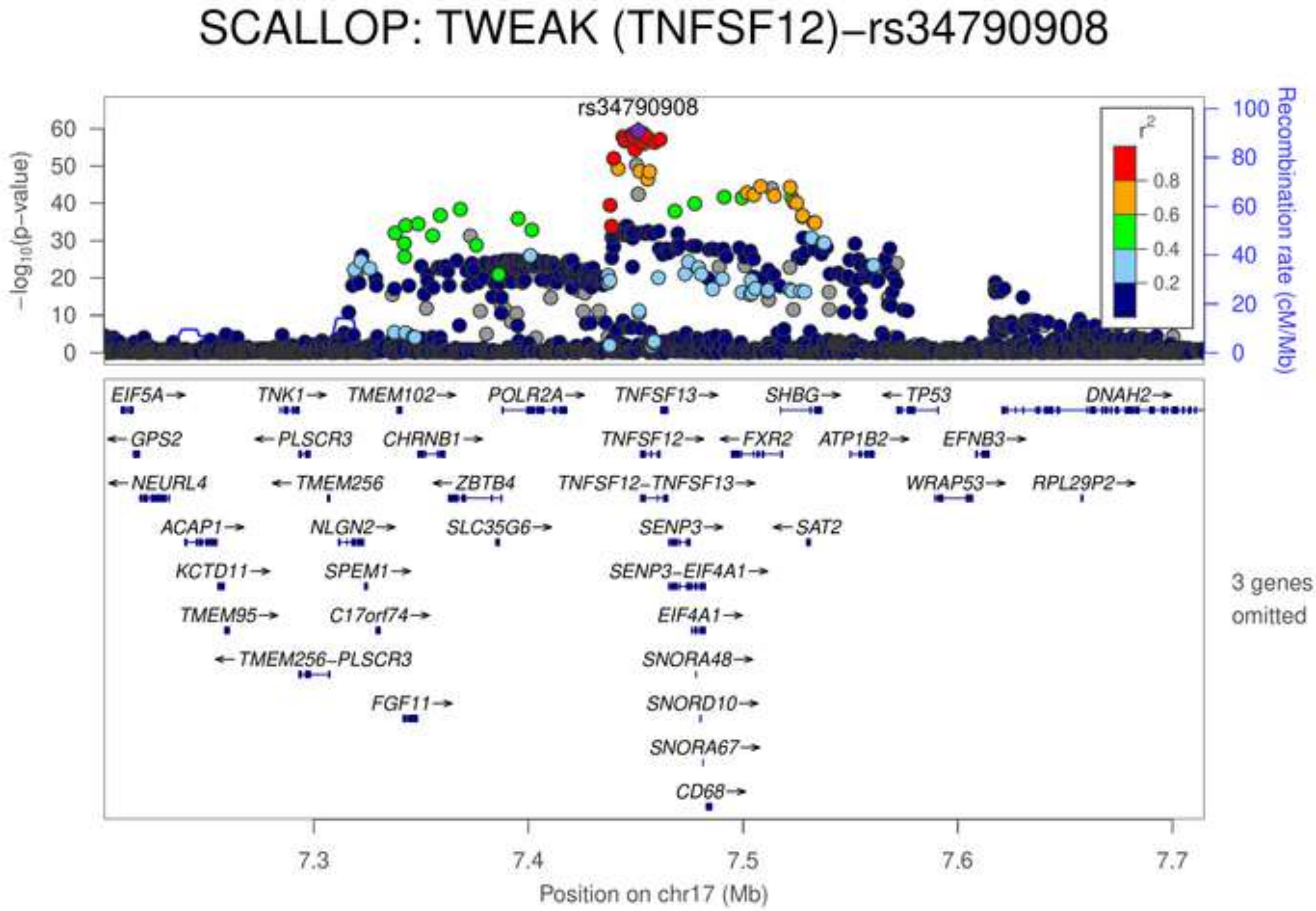
SCALLOP: TWEAK (TNFSF12)−rs34790908
rs34790908
−log10(p−value)
Recombination rate (cM/Mb)
r²
0.8
0.6
0.4
0.2
EIF5A→
TNK1→
TMEM102→
POLR2A→
TNFSF13→
SHBG→
←TP53
DNAH2→
←GPS2
←PLSCR3
CHRNB1→
TNFSF12→
←FXR2
ATP1B2→
EFNB3→
←NEURL4
←TMEM256
←ZBTB4
TNFSF12−TNFSF13→
WRAP53→
RPL29P2→
ACAP1→
NLGN2→
SLC35G6→
SENP3→
←SAT2
KCTD11→
SPEM1→
SENP3−EIF4A1→
3 genes omitted
TMEM95→
C17orf74→
EIF4A1→
←TMEM256−PLSCR3
SNORA48→
FGF11→
SNORD10→
SNORA67→
CD68→
Position on chr17 (Mb)

eQTLGen: uPA (PLAU)−rs55744193

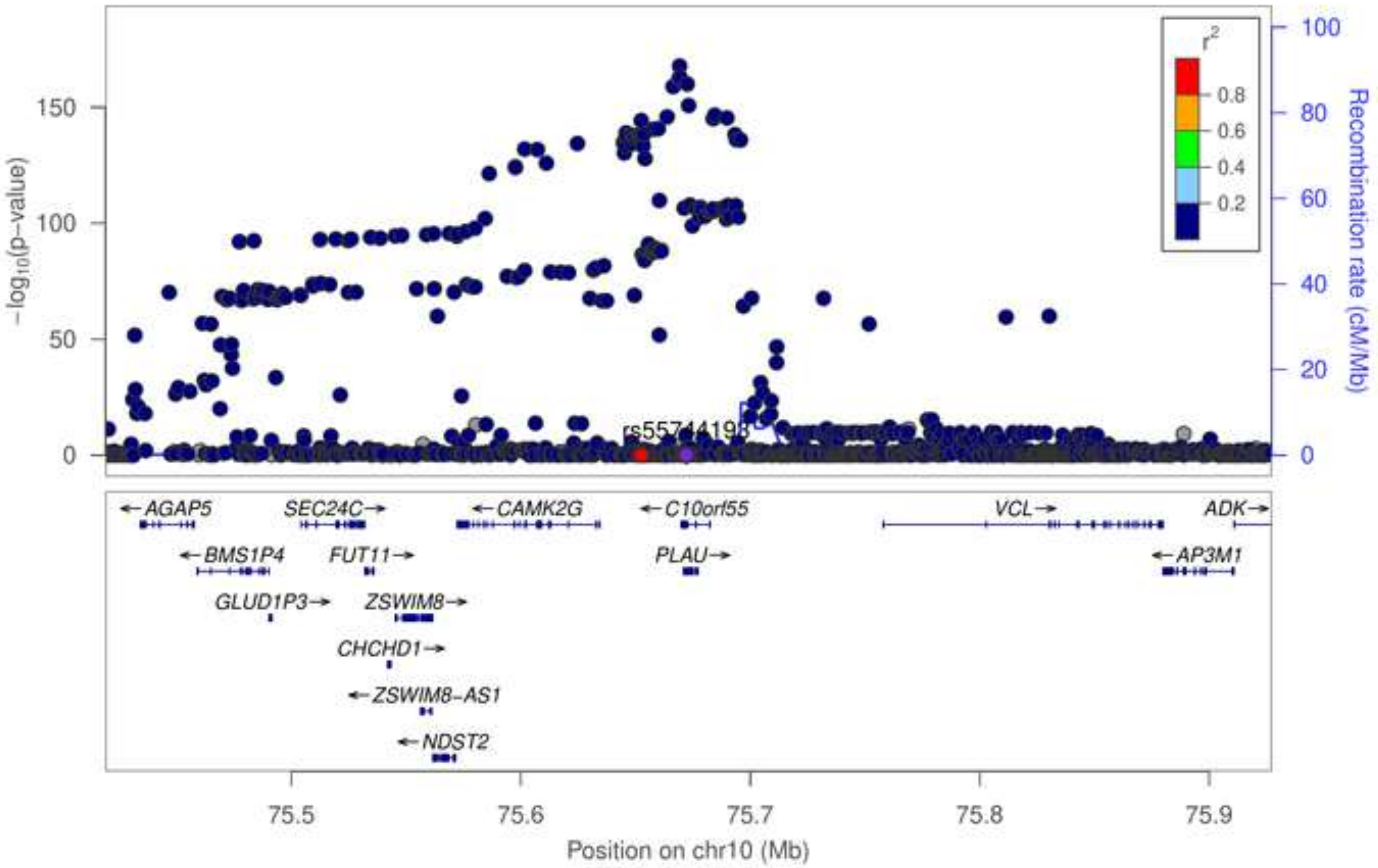

SCALLOP: uPA (PLAU)−rs55744193

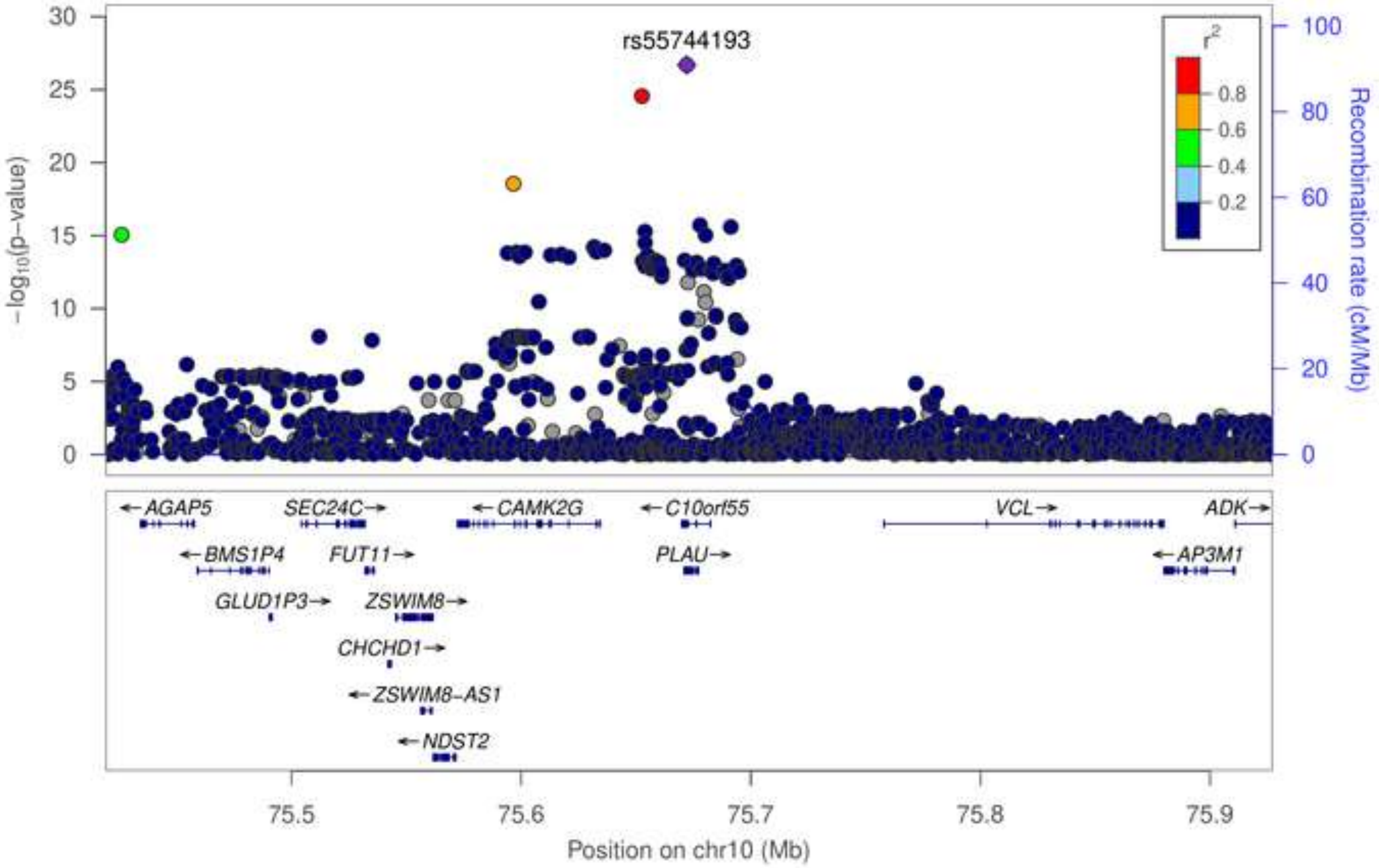

eQTLGen: VEGF_A (VEGFA)−rs6921438

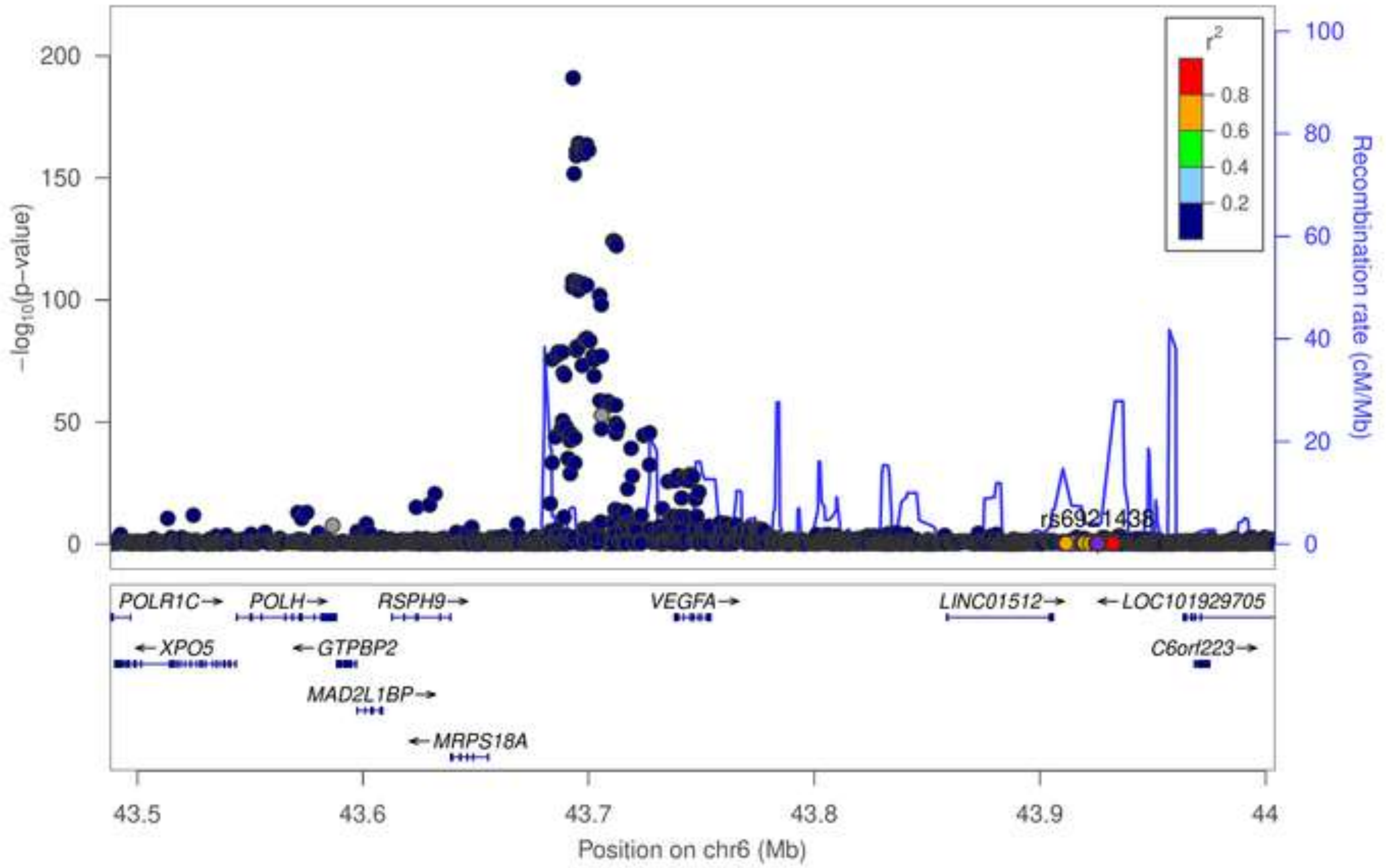

SCALLOP: VEGF_A (VEGFA)−rs6921438

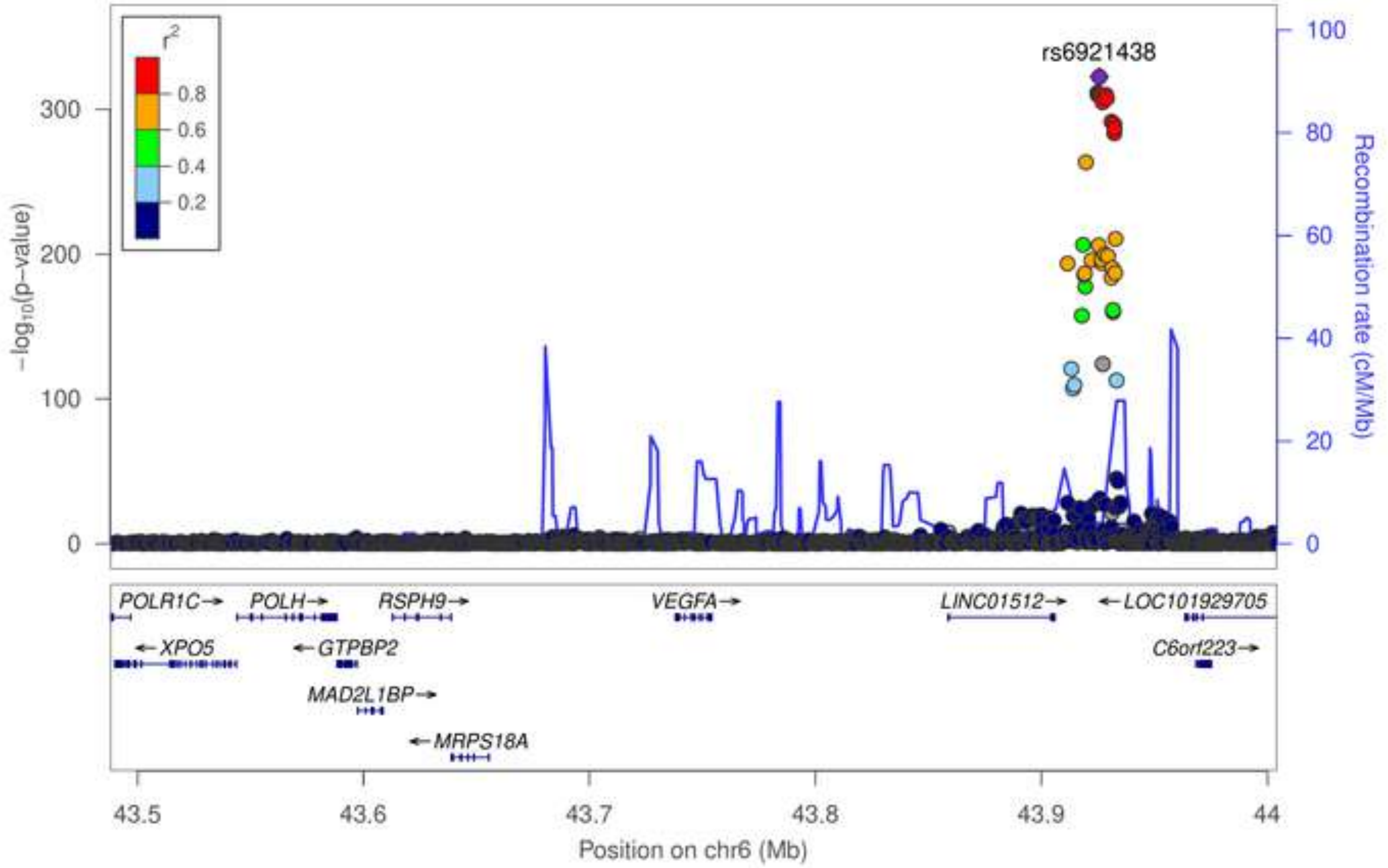

Supplementary Figure 4. Regional association plots comparing plasma *cis*-pQTLs and disease associations. 'LocusZoom' regional association plots comparing the plasma *cis*-pQTL signals (upper panel) with the relevant disease signals (lower panel) for the 12 protein-disease pairs with significant GSMR results following our initial filters prior to colocalisation testing (see Methods). The sentinel variant for the pQTL is coloured in purple. LD (r^2) to the sentinel pQTL is indicated by the colour key. For pQTLs, two-sided *P*-values are from meta-analysis of linear regression estimates. For diseases, P-values are from logistic regression.

SCALLOP: CD40−rs1883832

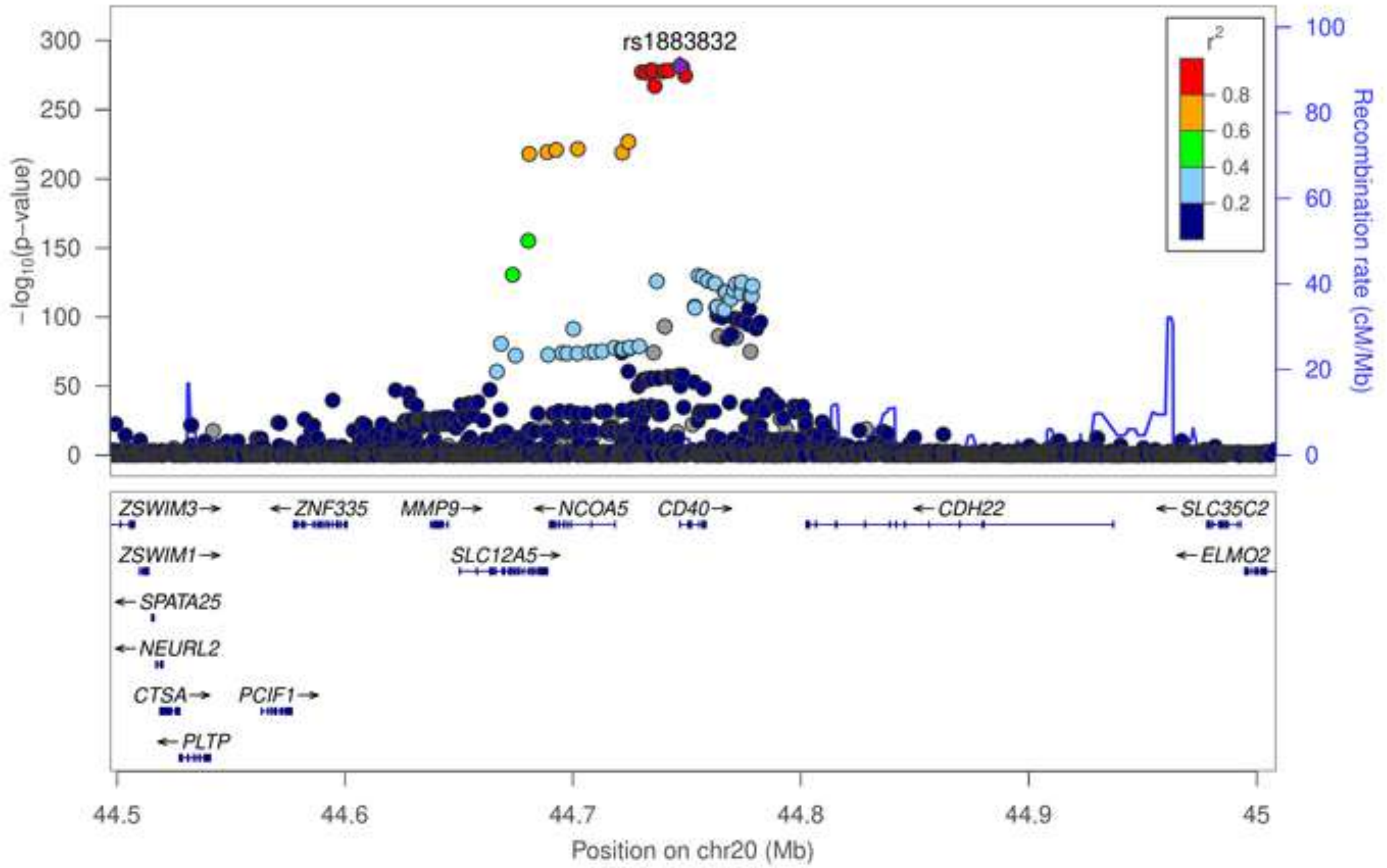

'Crohns disease'(ebi−a−GCST004132)−rs1883832

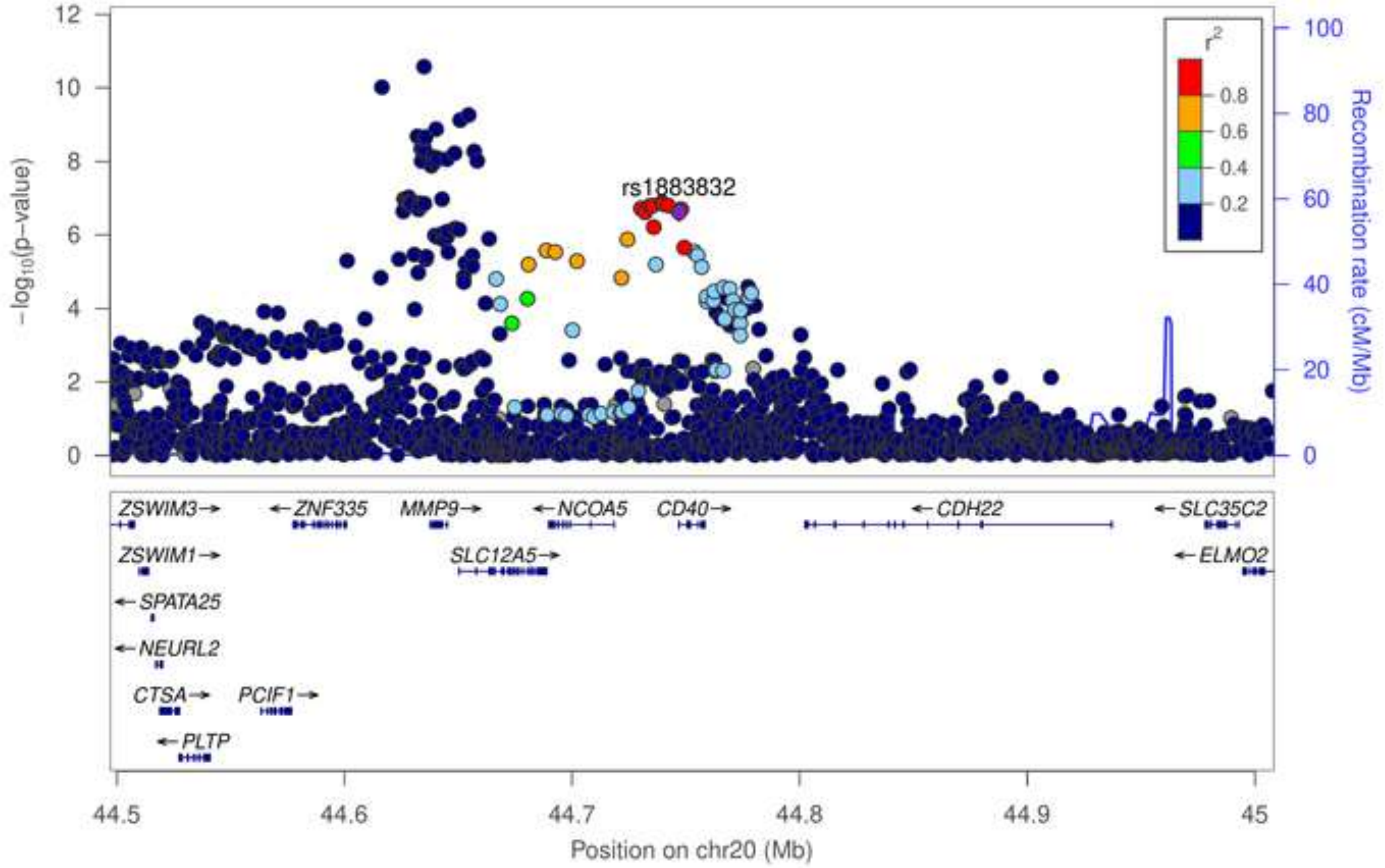

SCALLOP: CD40−rs1883832

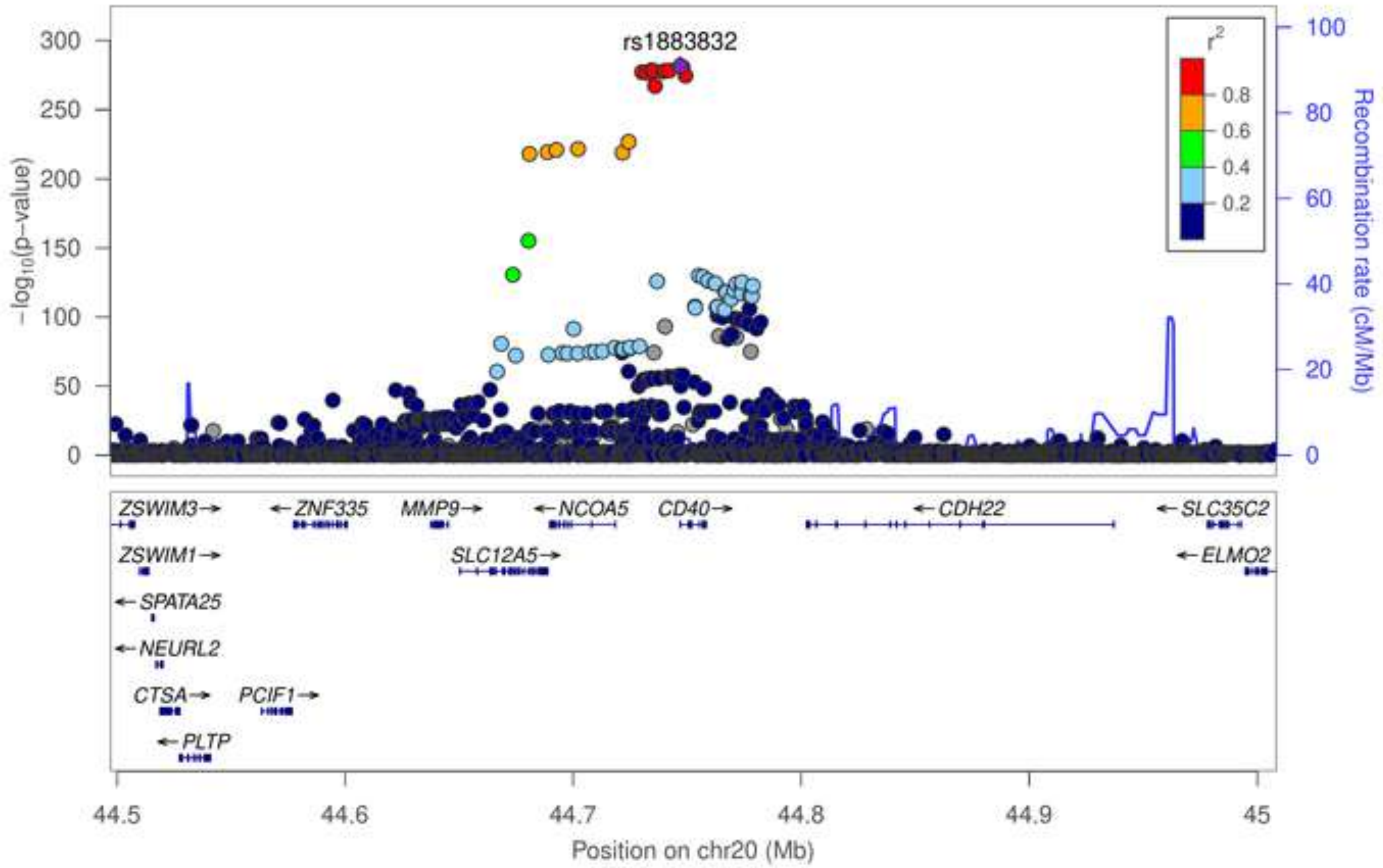

'Inflammatory bowel disease'(ebi−a−GCST004131)−rs1883832

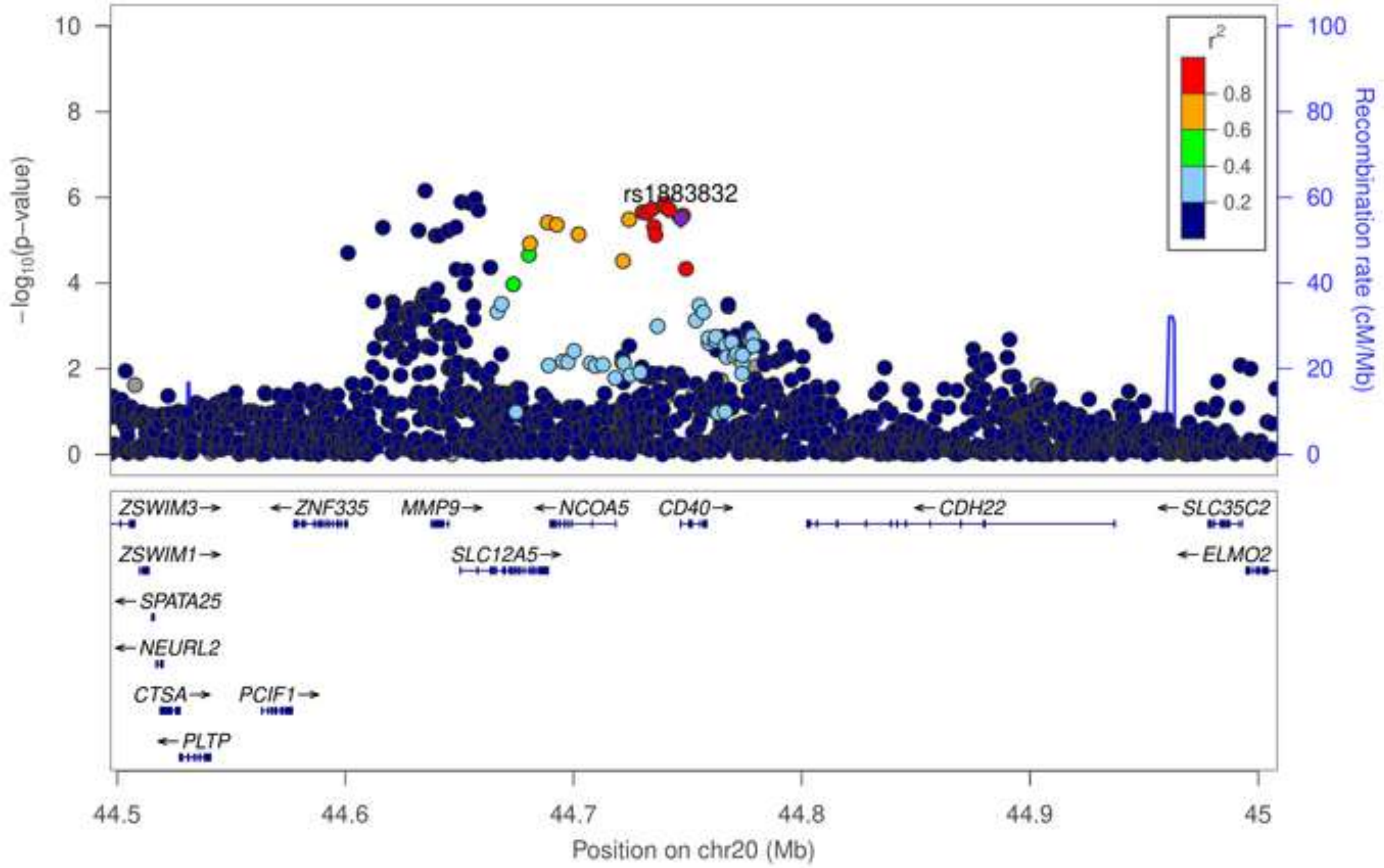

SCALLOP: CD40−rs1883832

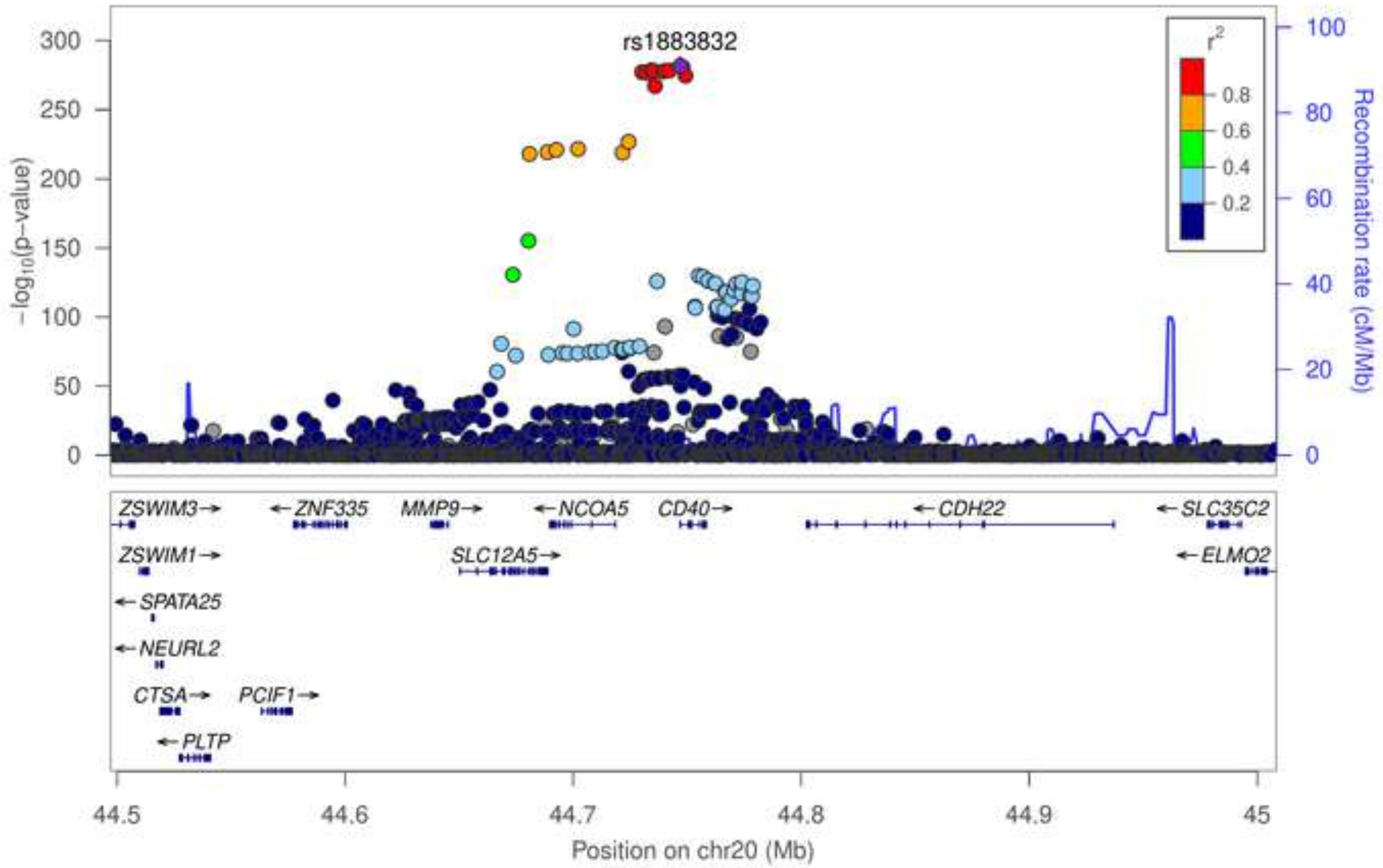

'Multiple sclerosis'(ieu−b−18)−rs1883832

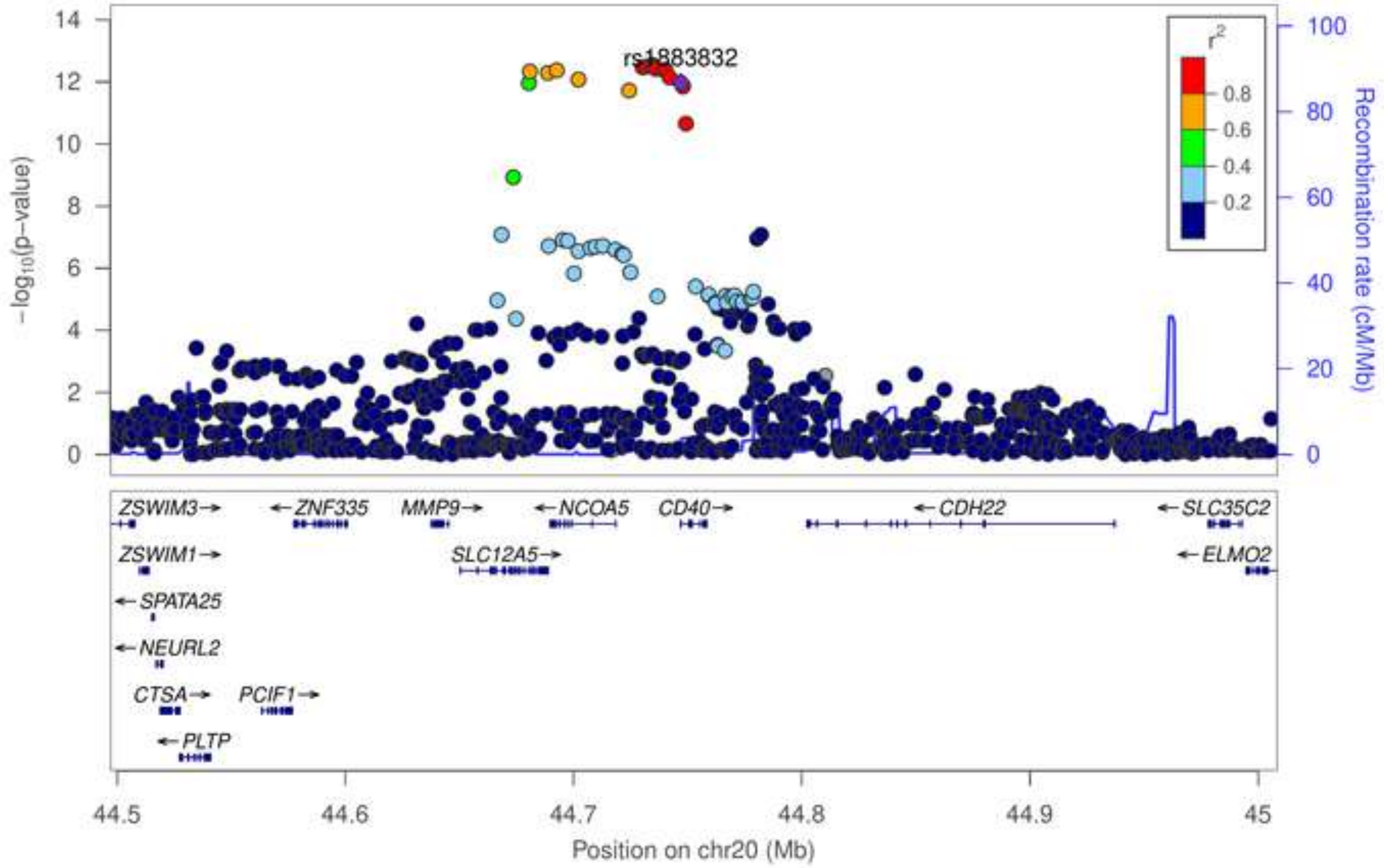

SCALLOP: CD40−rs1883832

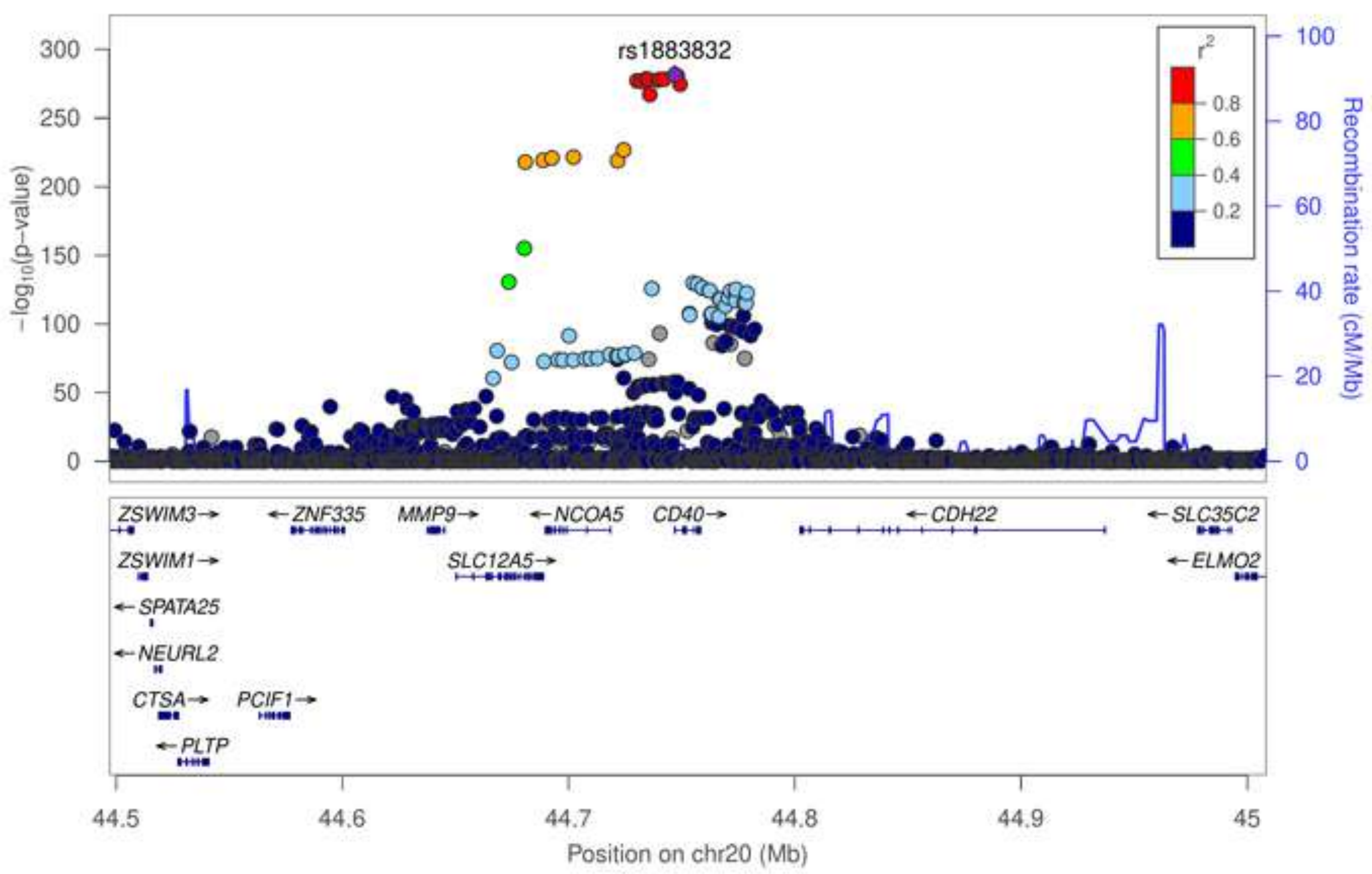

'Rheumatoid arthritis'(ieu−a−833)−rs1883832

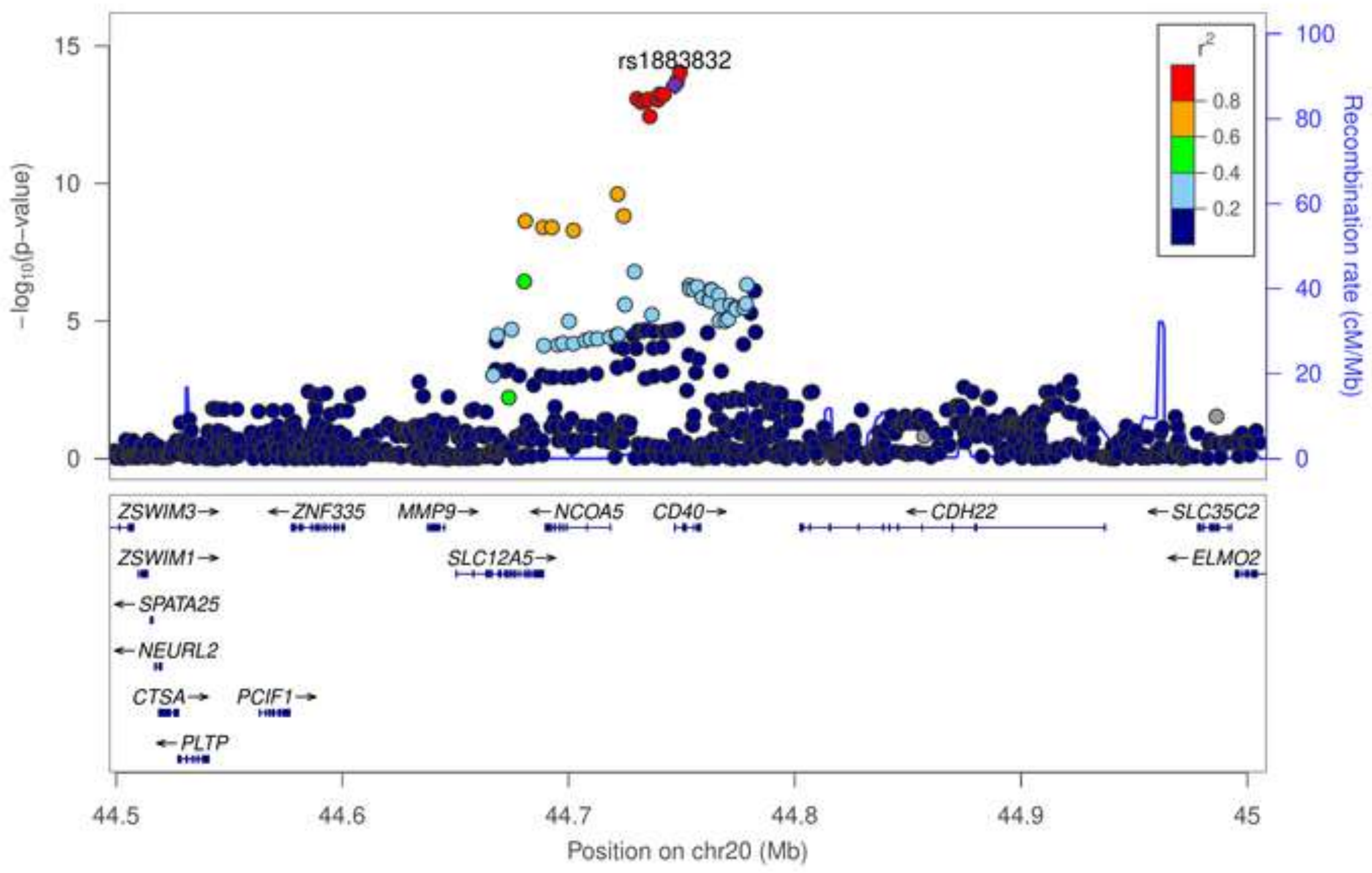

SCALLOP: CD5−rs674379

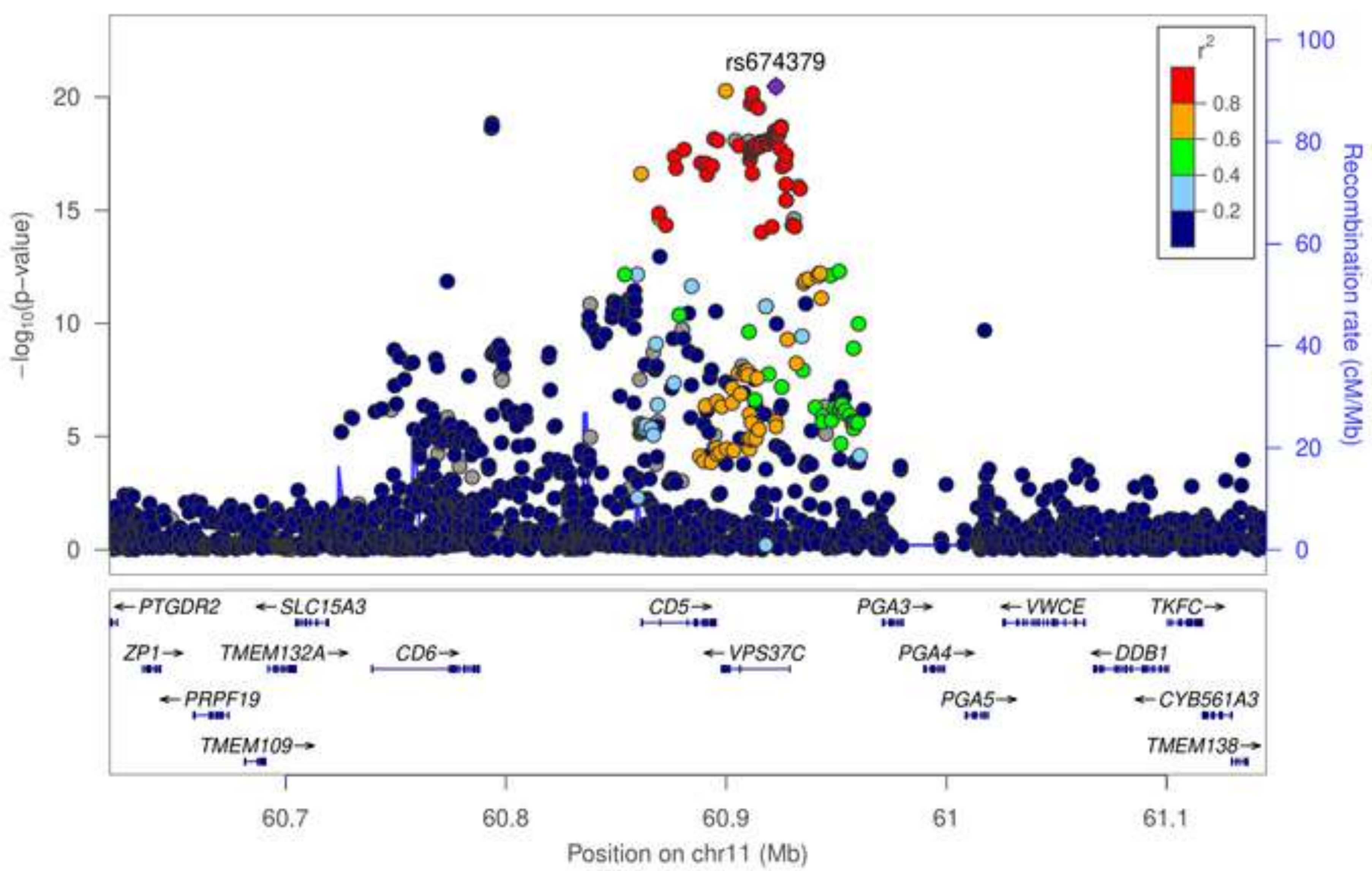

'Primary sclerosing cholangitis'(ieu−a−1112)−rs674379

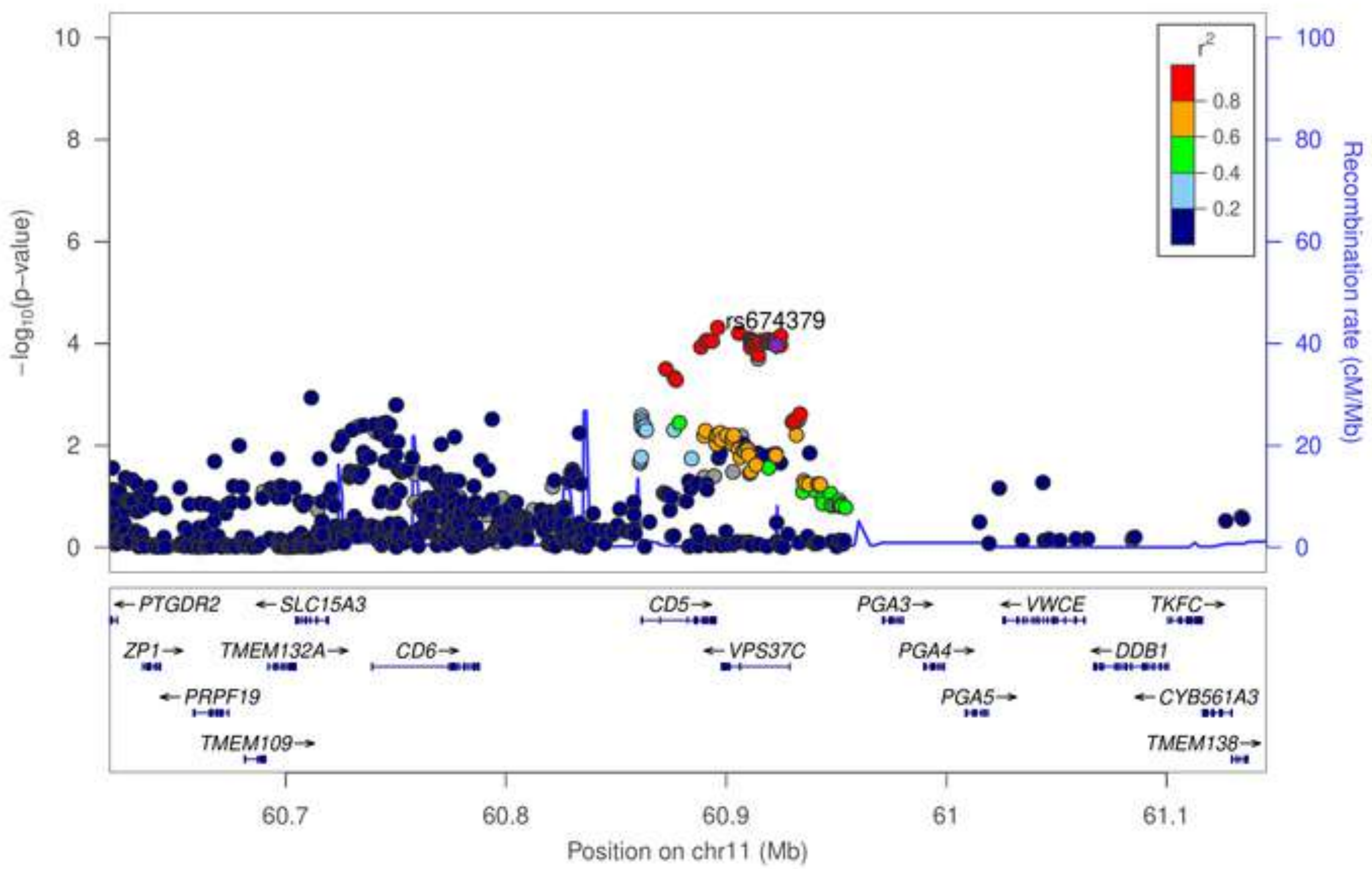

SCALLOP: CD6−rs2074227

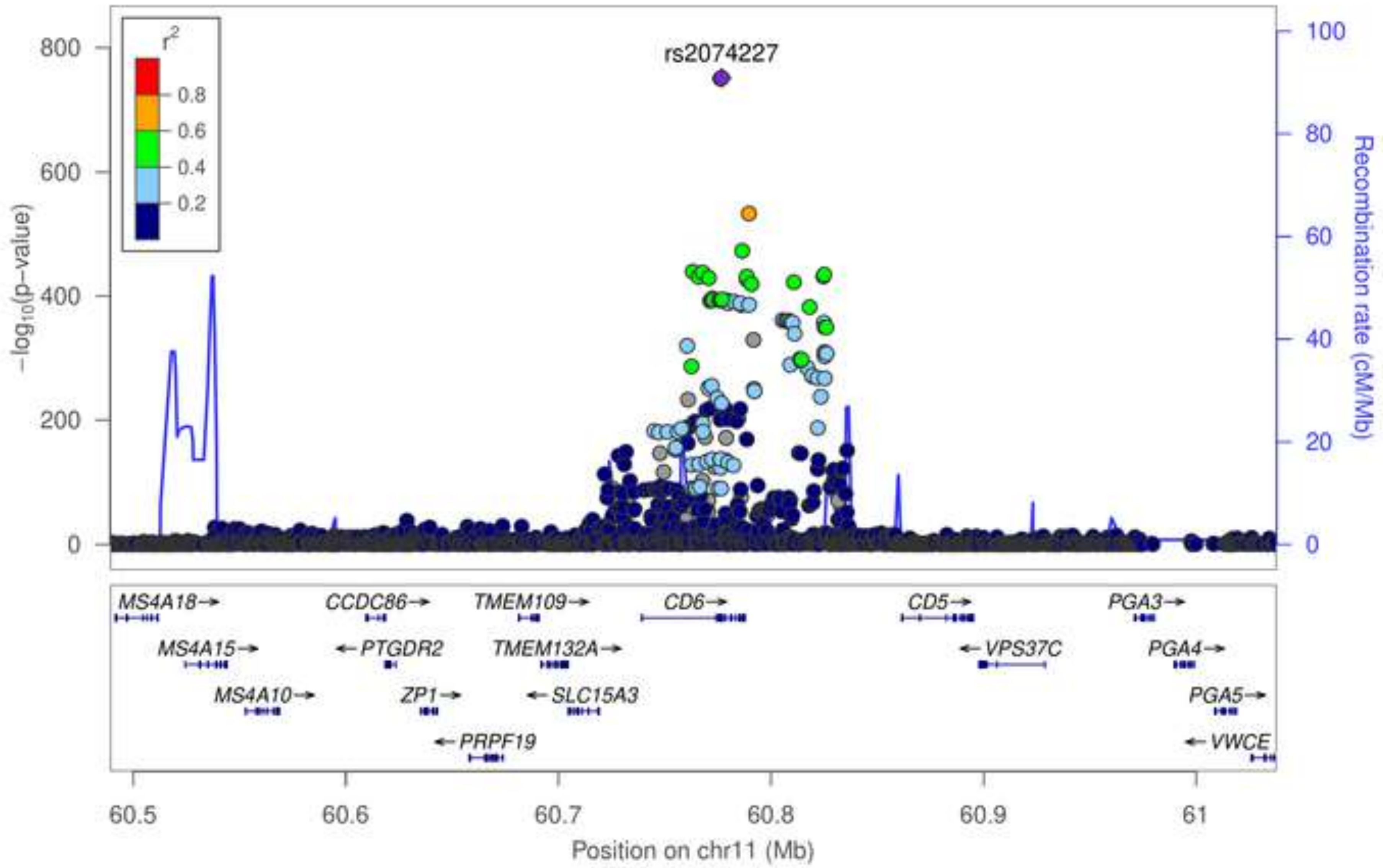

'Inflammatory bowel disease'(ebi−a−GCST004131)−rs2074227

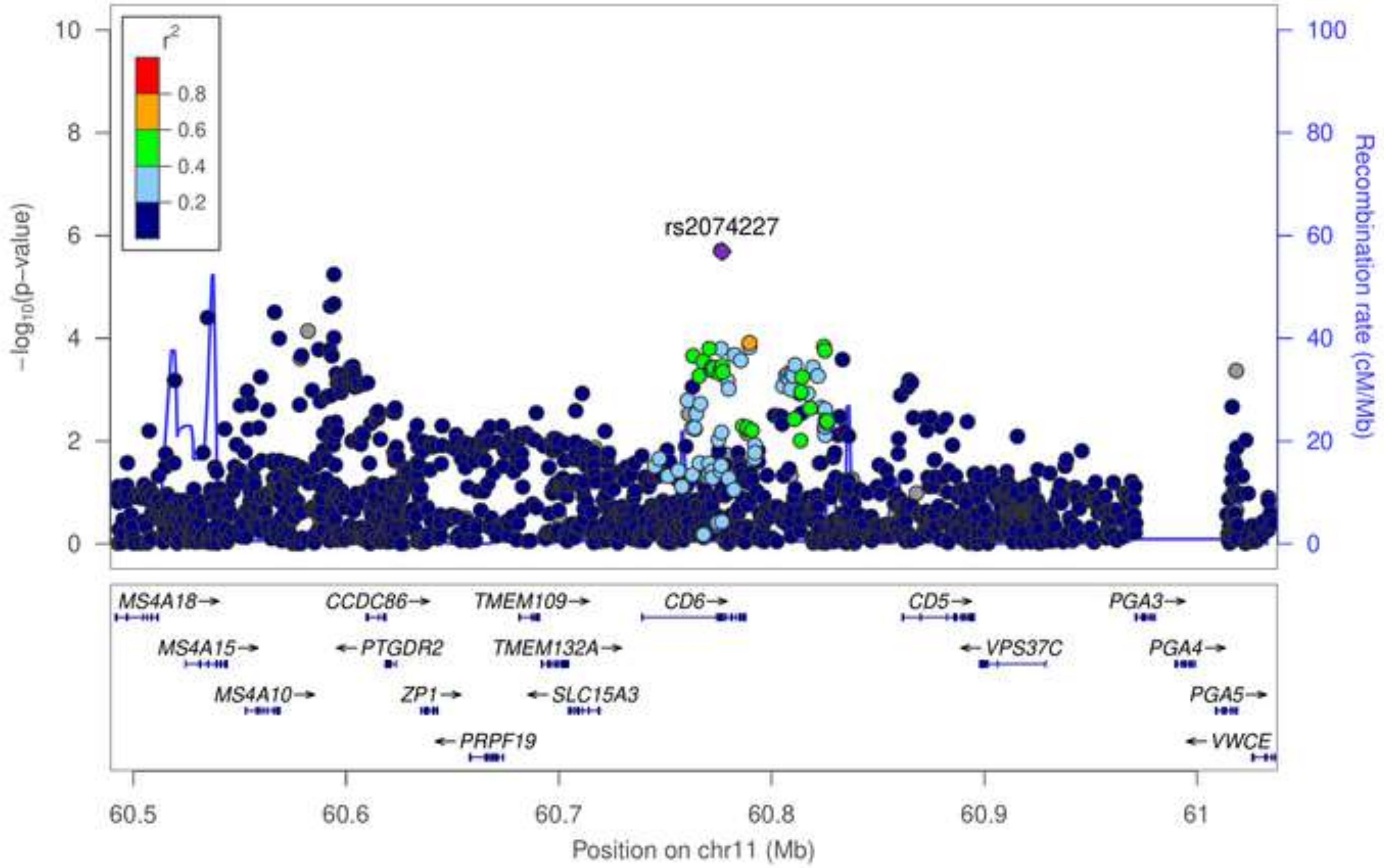

SCALLOP: CXCL5−rs450373

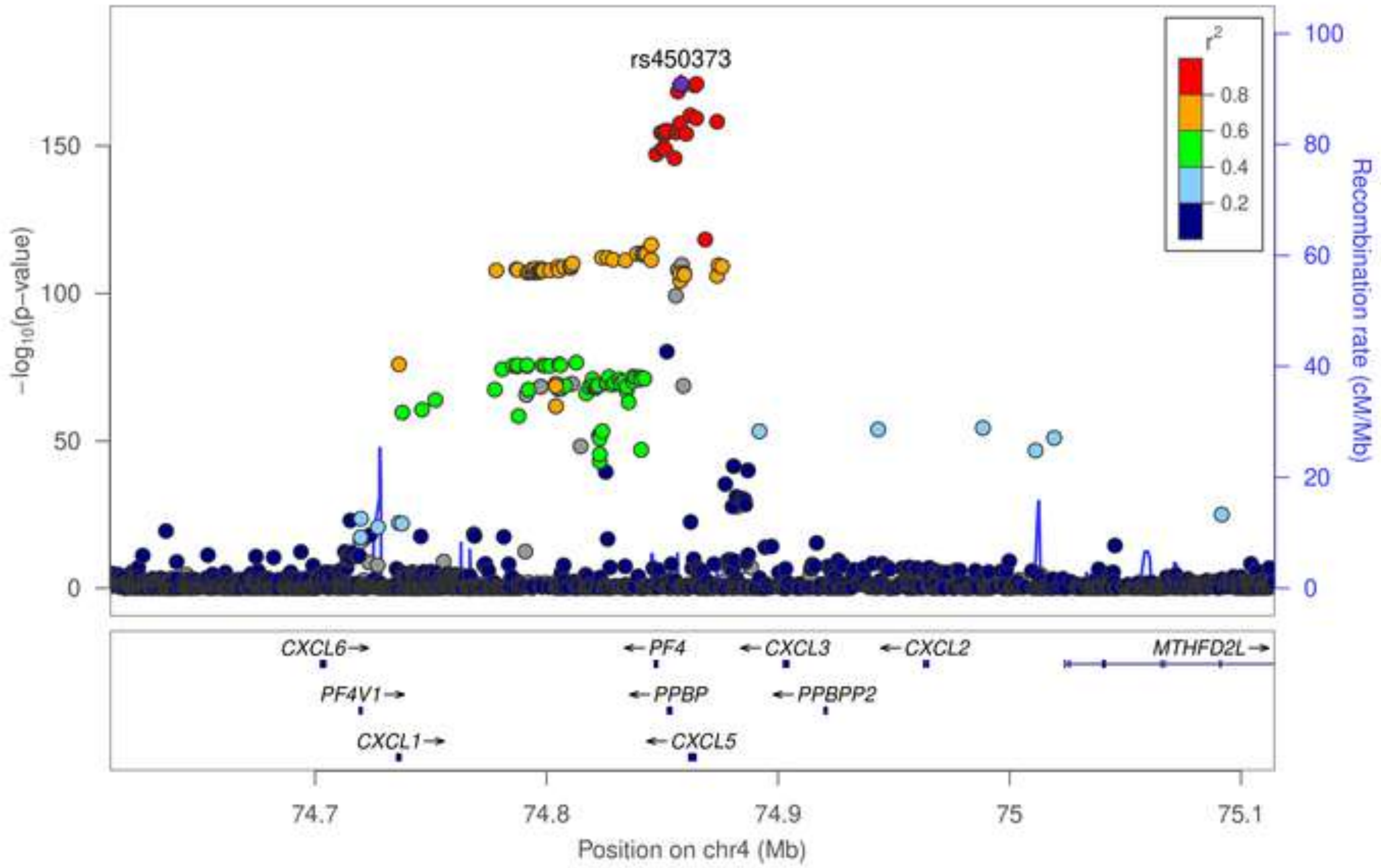

'Ulcerative colitis'(ebi−a−GCST004133)−rs450373

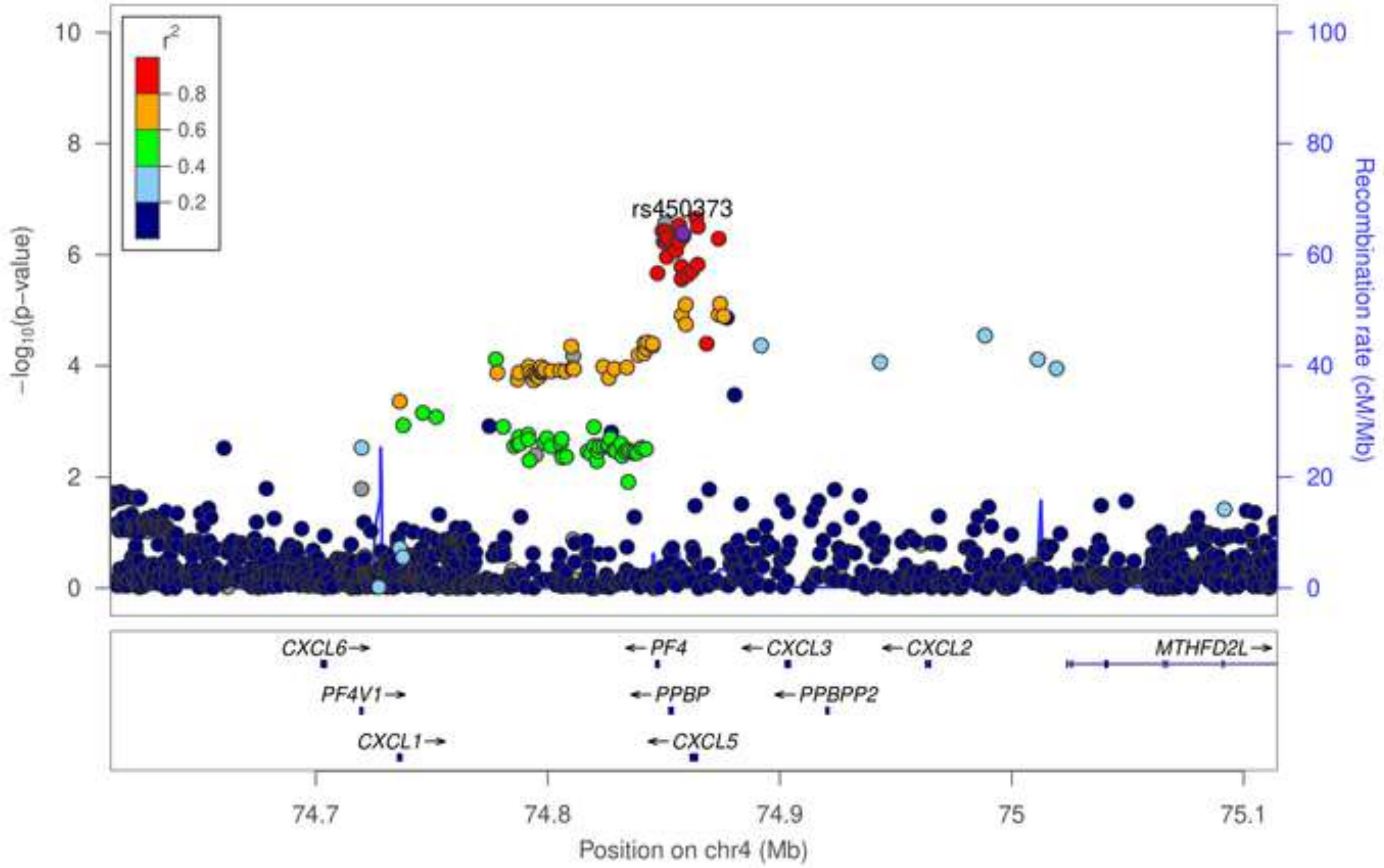

SCALLOP: IL12B−rs10076557

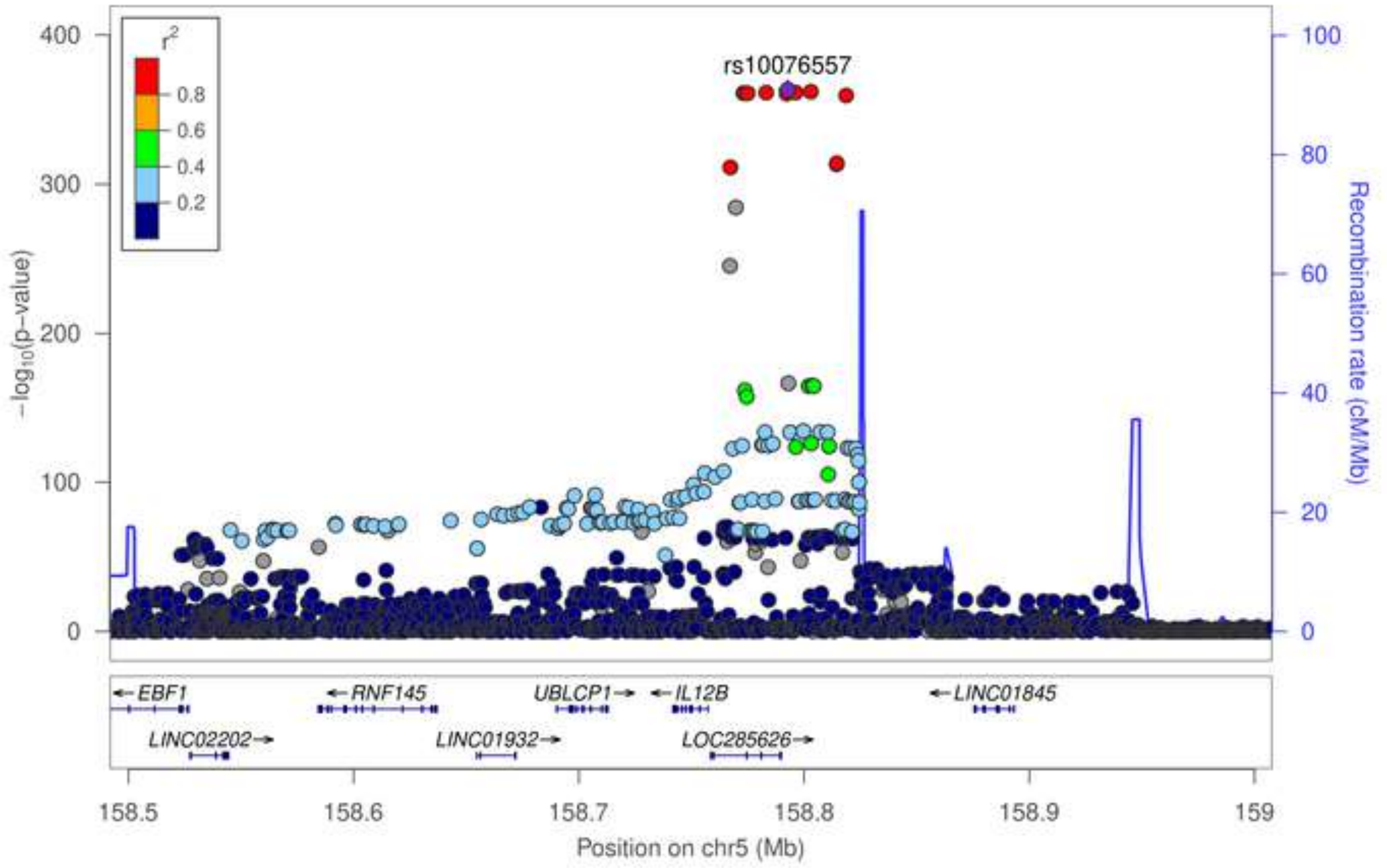

'Crohns disease'(ebi−a−GCST004132)−rs10076557

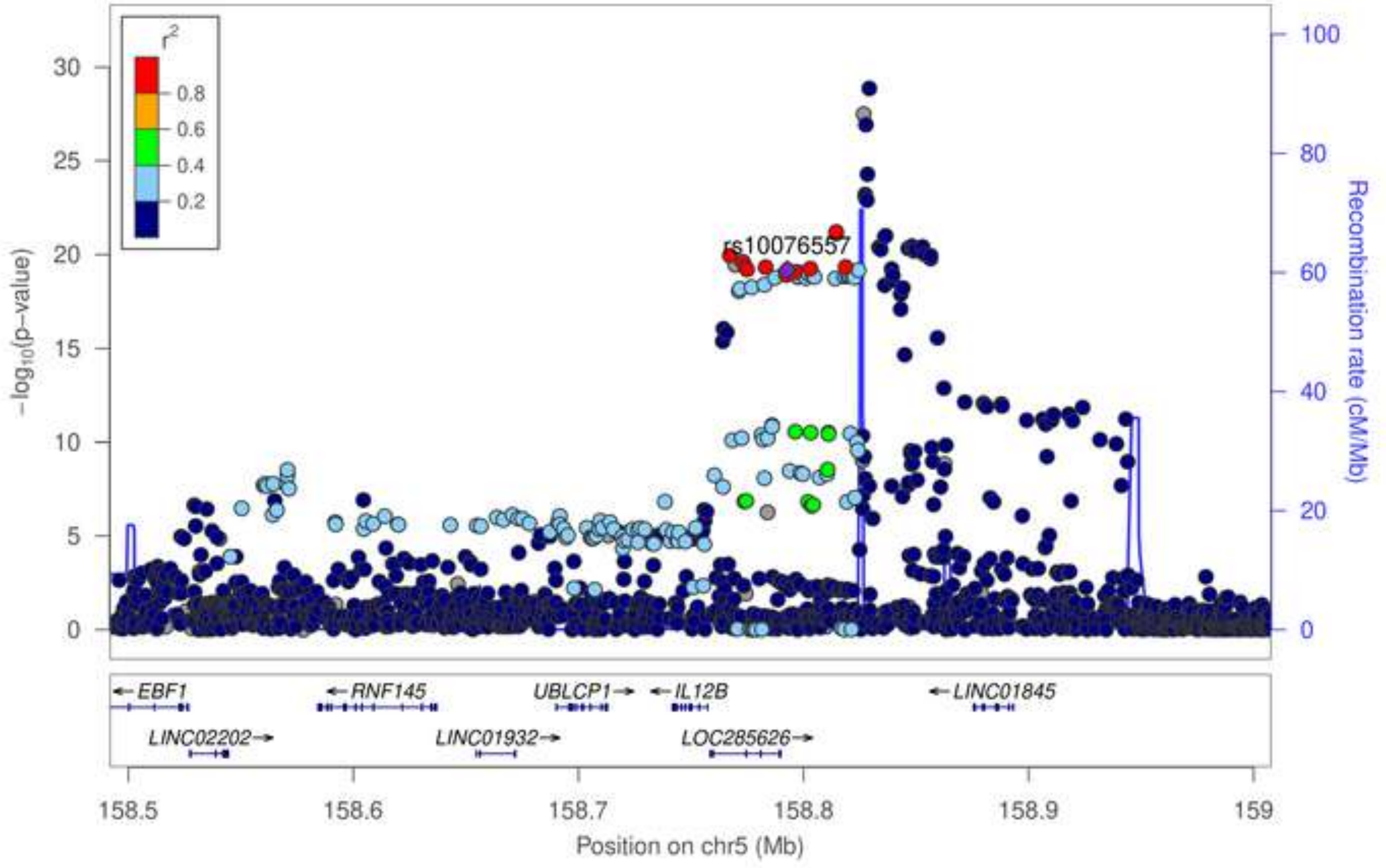

SCALLOP: IL12B−rs10076557

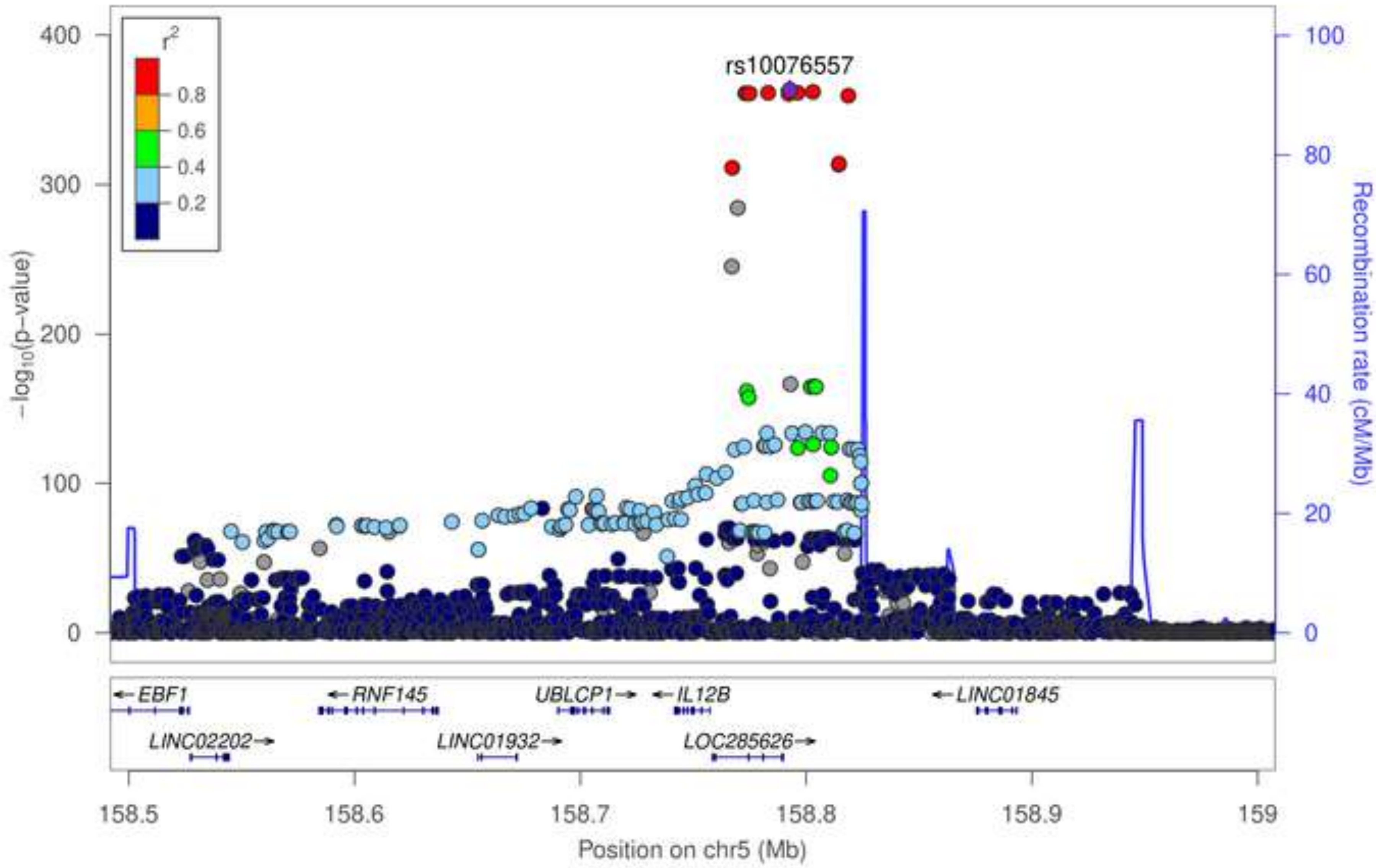

'Inflammatory bowel disease'(ebi−a−GCST004131)−rs10076557

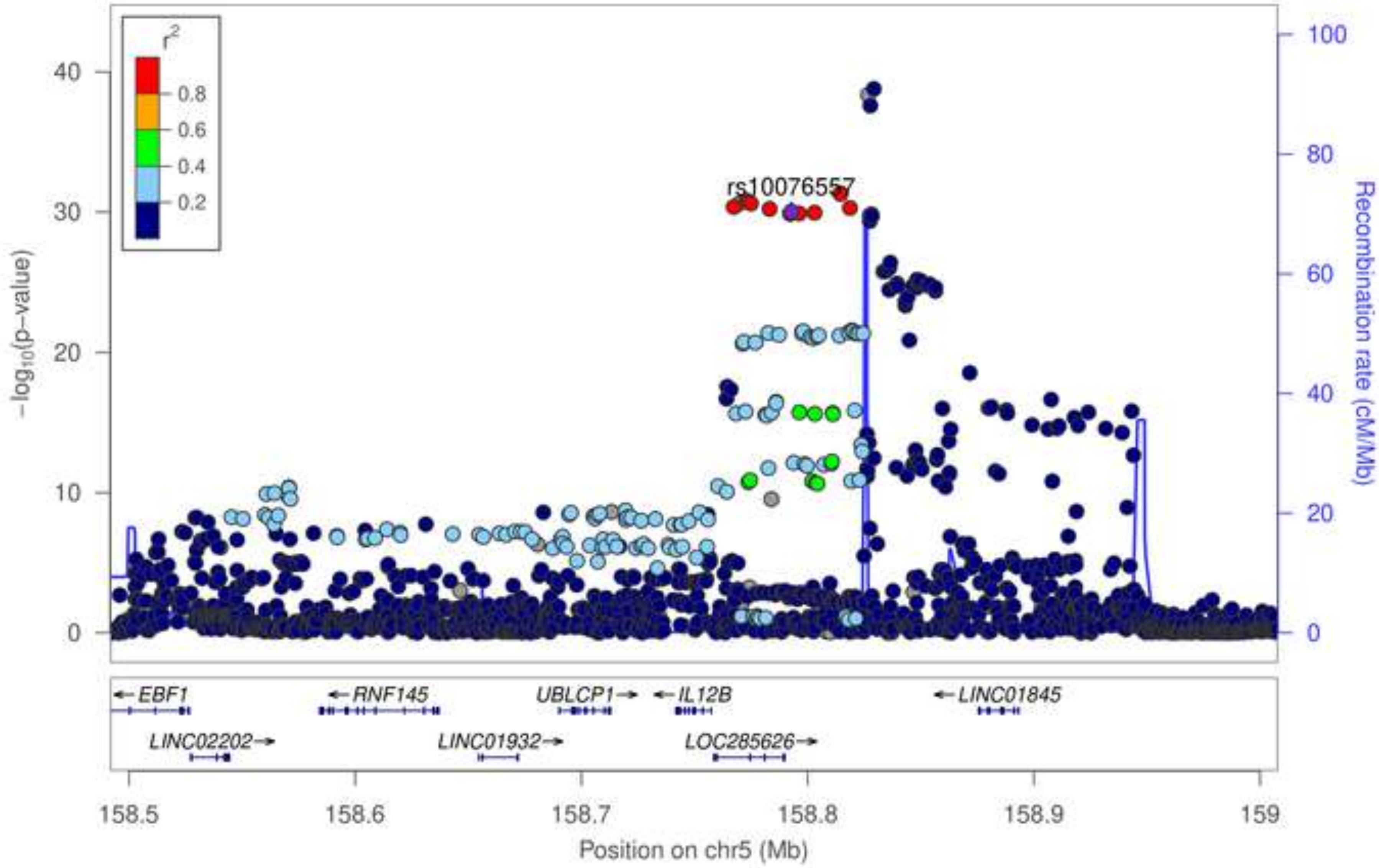

SCALLOP: IL12B−rs10076557

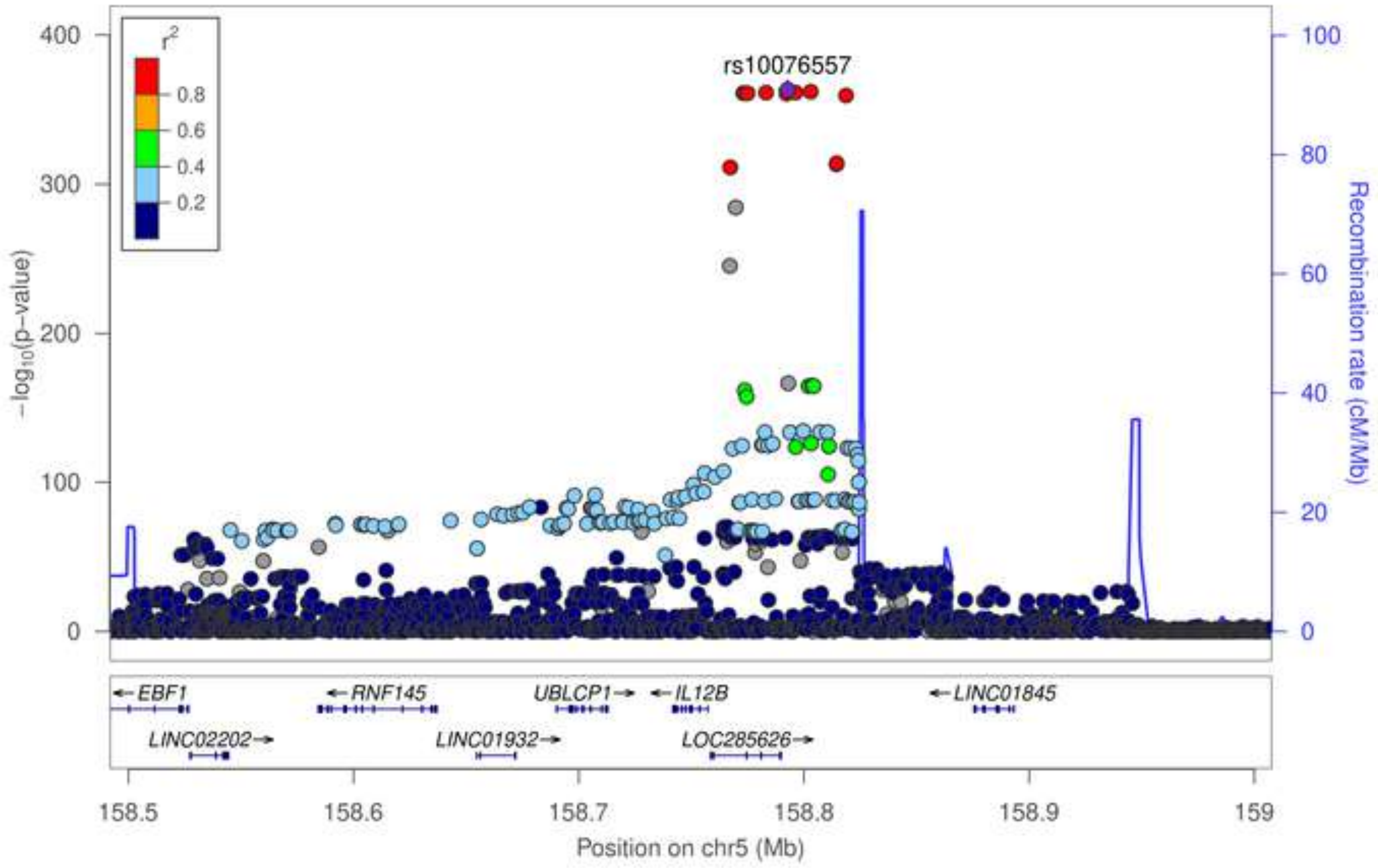

'Ulcerative colitis'(ebi−a−GCST004133)−rs10076557

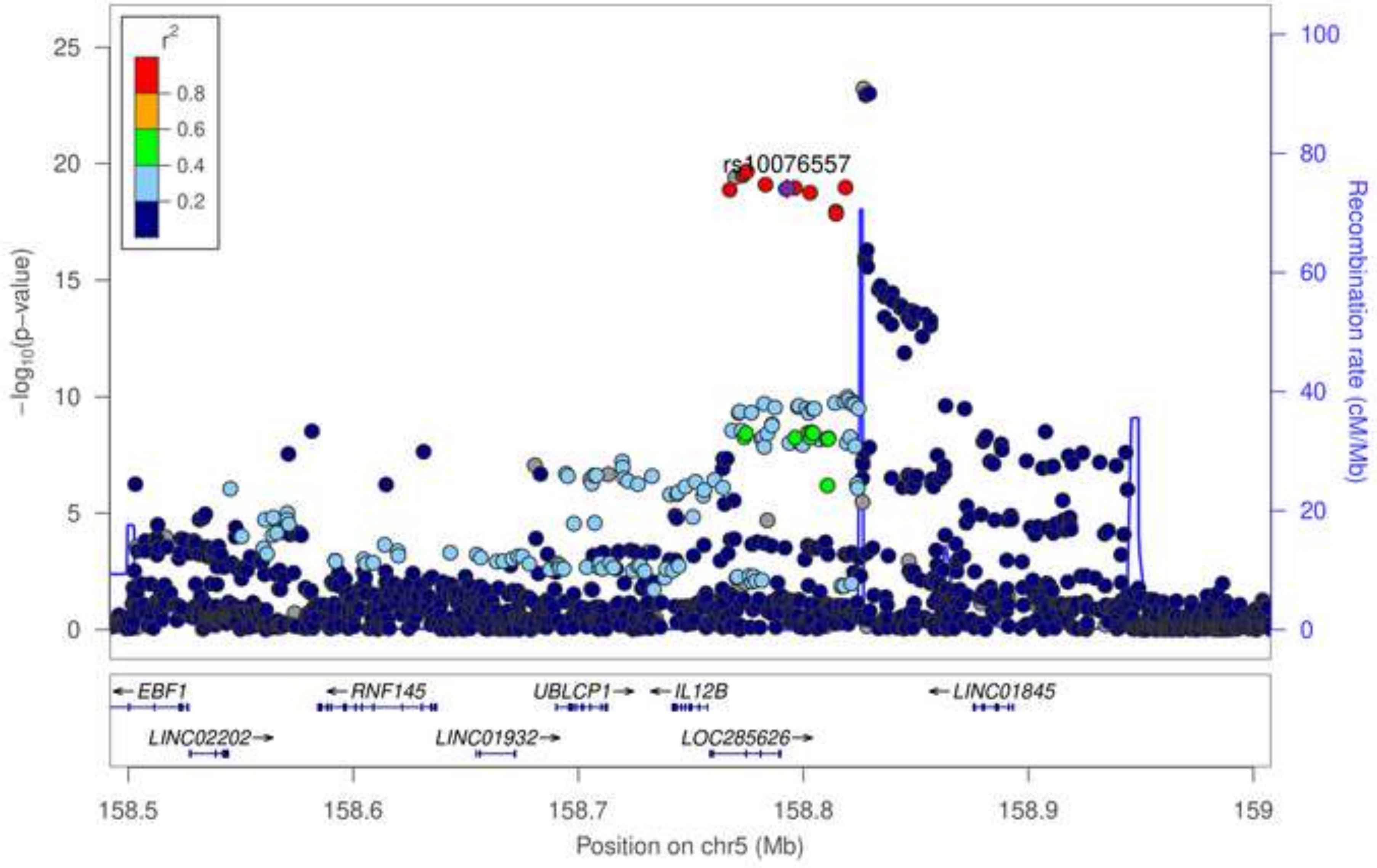

SCALLOP: IL18R1−rs2270297

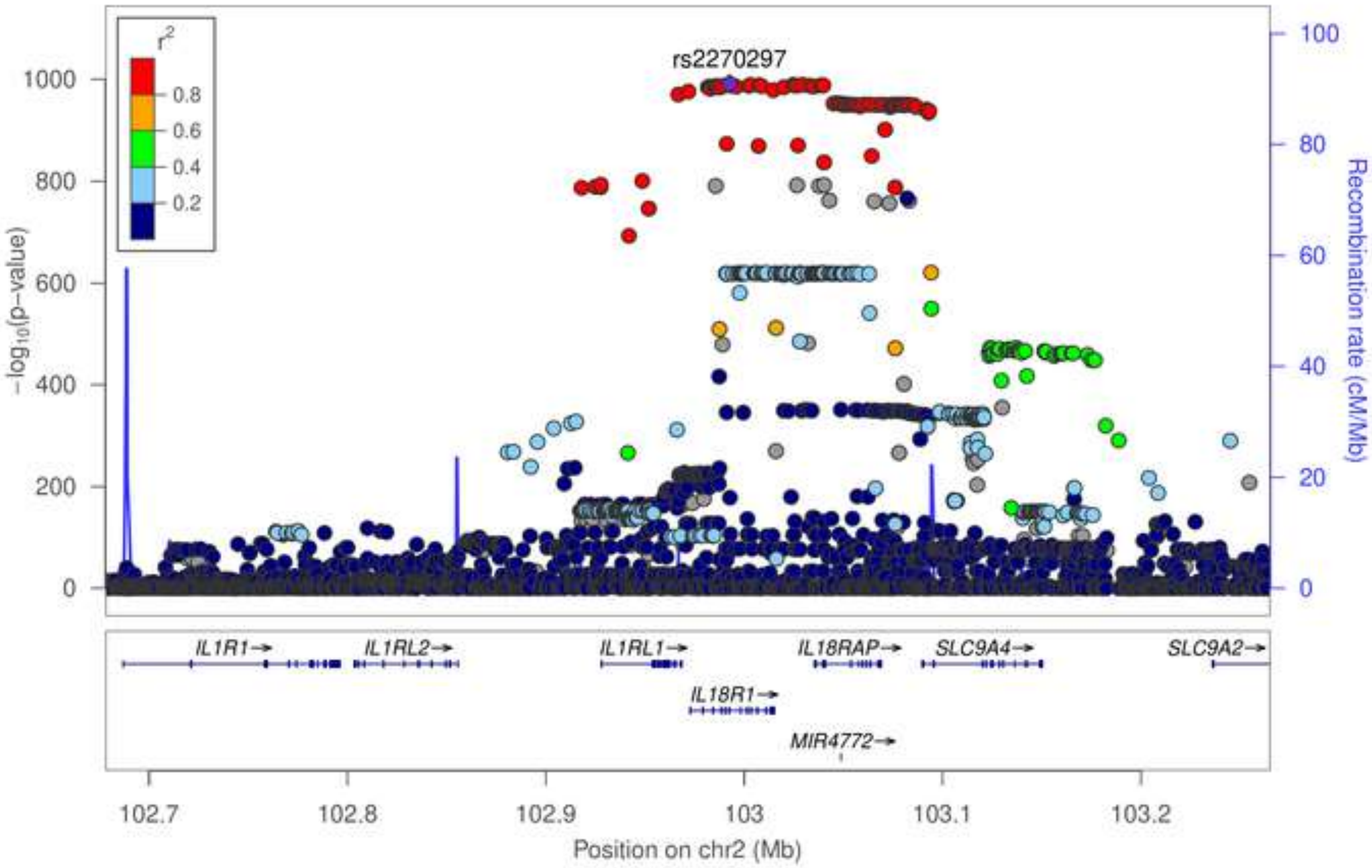

'Crohns disease'(ebi−a−GCST004132)−rs2270297

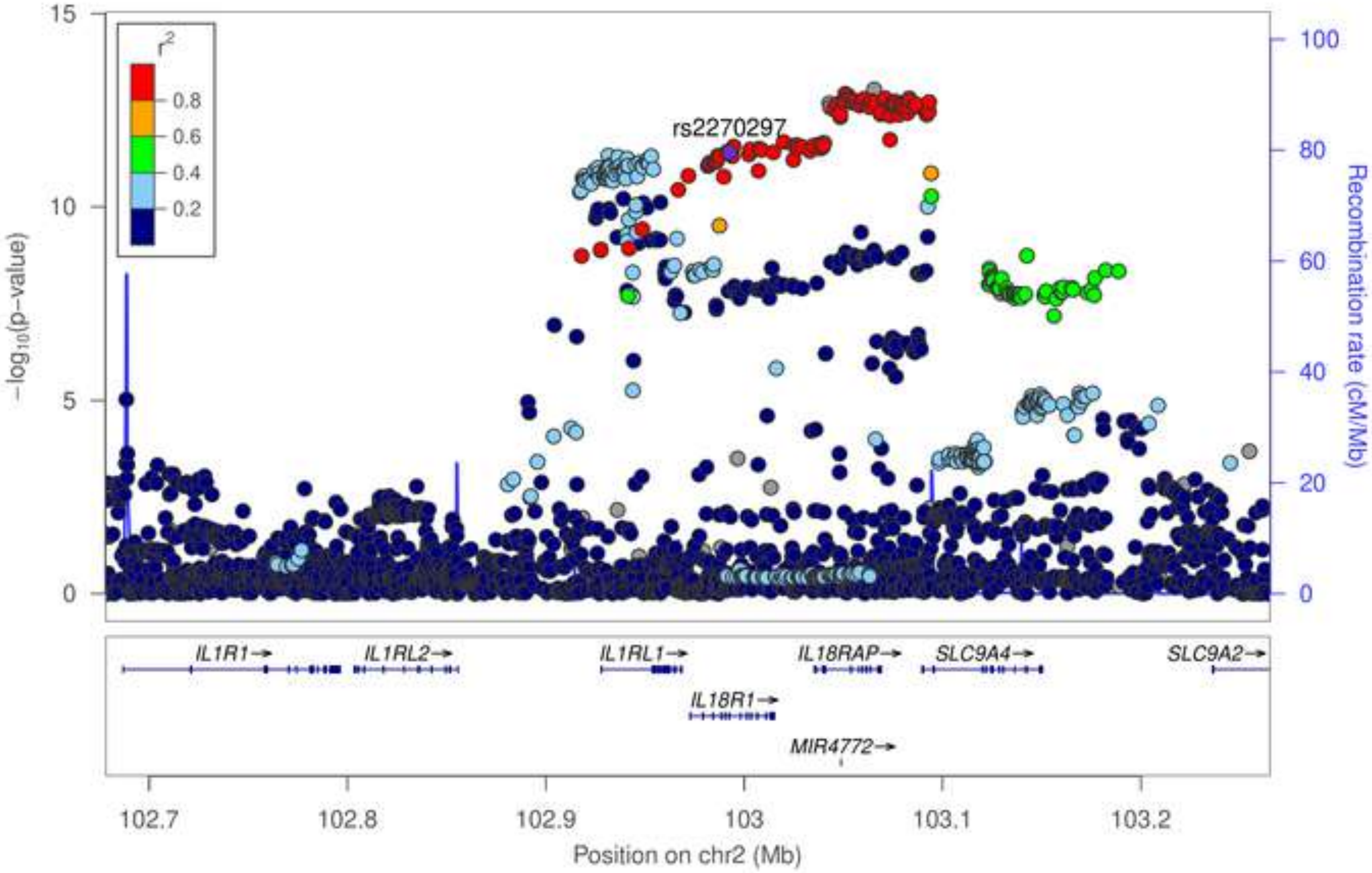

SCALLOP: IL18R1−rs2270297

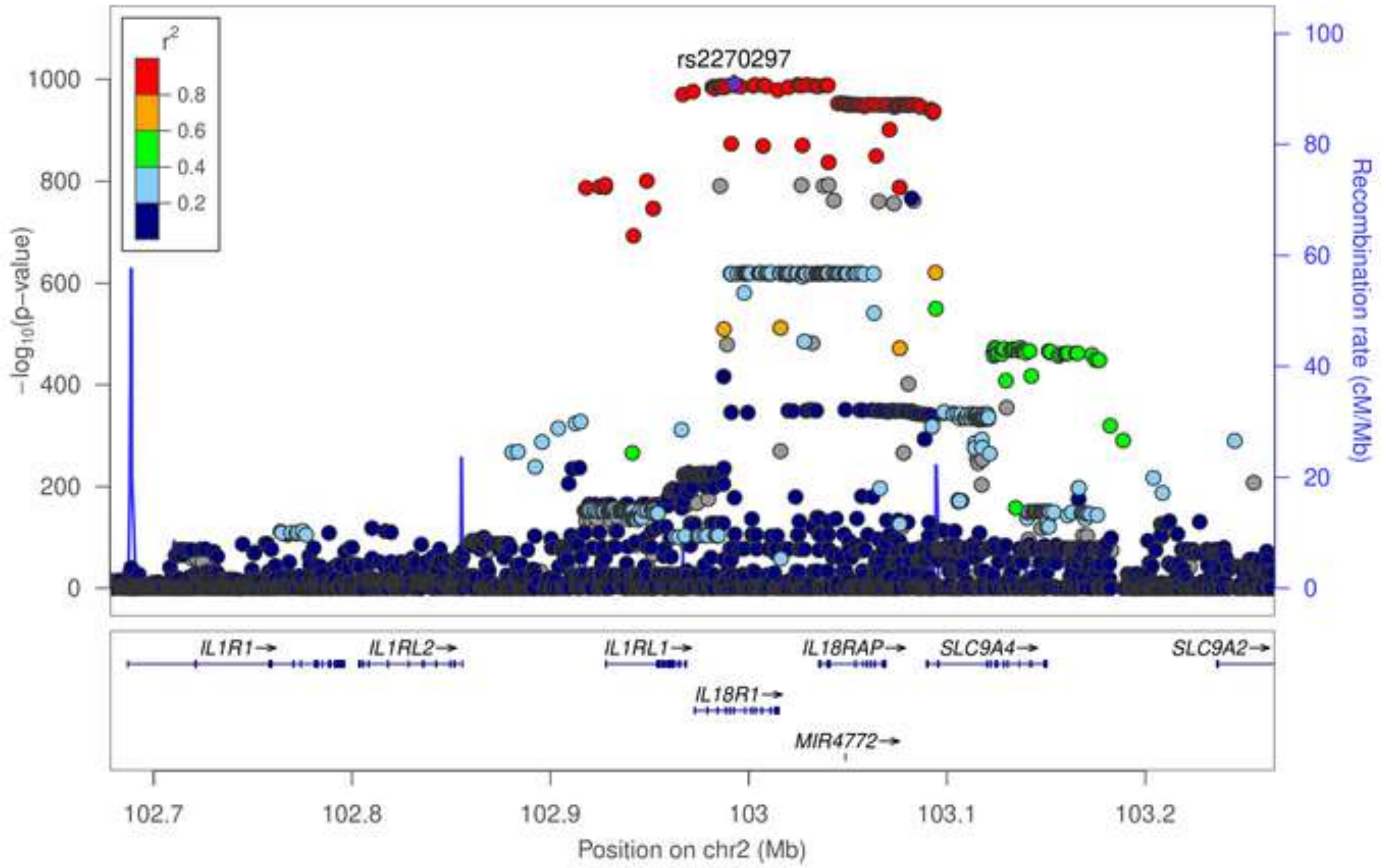

Eczema(ieu−a−996)−rs2270297

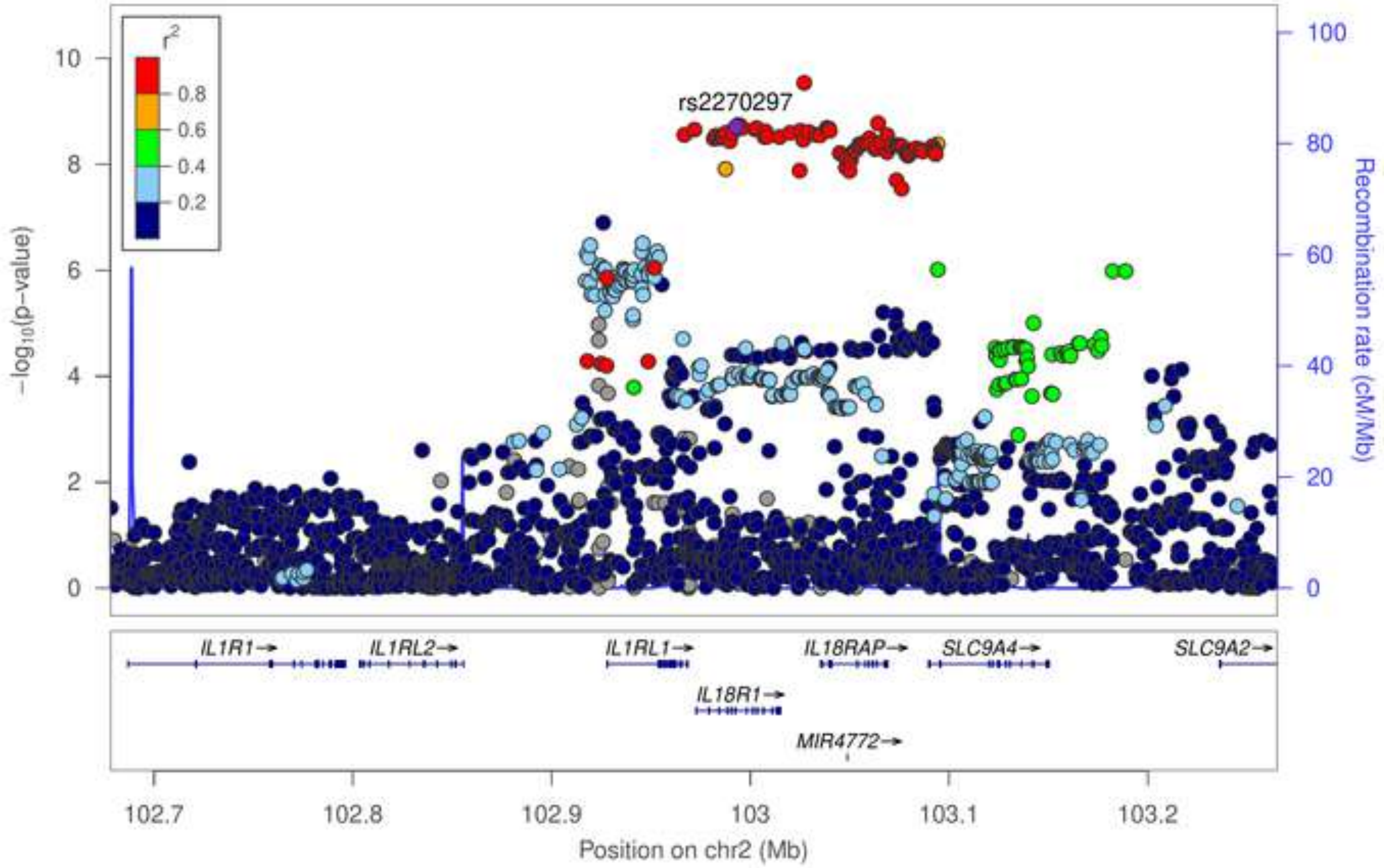

Supplement: Supplementary file 1 — Supplementary Note and Figs. 1–4. [file 41590_2023_1588_MOESM1_ESM.pdf]
