# Supplementary material for: Genetics of circulating inflammatory proteins identifies drivers of immune-mediated disease risk and therapeutic targets
Source: Nat Immunol. 2023 Aug 10;24(9):1540–51. doi: 10.1038/s41590-023-01588-w (PMC10457199; doi:10.1038/s41590-023-01588-w)
Supplement: Supplementary file 2 — Reporting Summary [file 41590_2023_1588_MOESM2_ESM.pdf]

nature portfolio

Corresponding author(s): James Peters

Last updated by author(s): Jul 6, 2023

Reporting Summary

Nature Portfolio wishes to improve the reproducibility of the work that we publish. This form provides structure for consistency and transparency in reporting. For further information on Nature Portfolio policies, see our Editorial Policies and the Editorial Policy Checklist.

Statistics

For all statistical analyses, confirm that the following items are present in the figure legend, table legend, main text, or Methods section.

n/a	Confirmed	
☐	☒	The exact sample size (*n*) for each experimental group/condition, given as a discrete number and unit of measurement
☐	☒	A statement on whether measurements were taken from distinct samples or whether the same sample was measured repeatedly
☐	☒	The statistical test(s) used AND whether they are one- or two-sided<br>*Only common tests should be described solely by name; describe more complex techniques in the Methods section.*
☐	☒	A description of all covariates tested
☐	☒	A description of any assumptions or corrections, such as tests of normality and adjustment for multiple comparisons
☐	☒	A full description of the statistical parameters including central tendency (e.g. means) or other basic estimates (e.g. regression coefficient) AND variation (e.g. standard deviation) or associated estimates of uncertainty (e.g. confidence intervals)
☐	☒	For null hypothesis testing, the test statistic (e.g. *F*, *t*, *r*) with confidence intervals, effect sizes, degrees of freedom and *P* value noted<br>*Give P values as exact values whenever suitable.*
☐	☒	For Bayesian analysis, information on the choice of priors and Markov chain Monte Carlo settings
☒	☐	For hierarchical and complex designs, identification of the appropriate level for tests and full reporting of outcomes
☐	☒	Estimates of effect sizes (e.g. Cohen's *d*, Pearson's *r*), indicating how they were calculated

Our web collection on statistics for biologists contains articles on many of the points above.

Software and code

Policy information about availability of computer code

Data collection	N/A
Data analysis	R packages: BiomaRt v2.52; qqman v0.1.4; QCGWAS v1.0-8; gap v1.2.3-6; rGREAT v2.0.0; KEGGREST v1.36, coloc v3.1; HyPrColoc v1.0; oligo v1.62.0; limma v3.54.0; DESeq2 v1.38.0<br>Other: METAL v28.8.2018; LocusZoom v1.4; bedtools v2.27.0; GCTA v1.93.0beta; PhenoScanner v2; Variant Effect Predictor v98.3; STRINGdb v2.8.4

For manuscripts utilizing custom algorithms or software that are central to the research but not yet described in published literature, software must be made available to editors and reviewers. We strongly encourage code deposition in a community repository (e.g. GitHub). See the Nature Portfolio guidelines for submitting code & software for further information.

Data

Policy information about availability of data

All manuscripts must include a data availability statement. This statement should provide the following information, where applicable:

- Accession codes, unique identifiers, or web links for publicly available datasets
- A description of any restrictions on data availability
- For clinical datasets or third party data, please ensure that the statement adheres to our policy

Full per-protein GWAS summary statistics are available for download at https://www.phpc.cam.ac.uk/ceu/proteins/ and the EBI GWAS Catalog https://

www.ebi.ac.uk/gwas/ (accession GCST90270765-GCST90270855). Individual-level genetic data cannot be shared in an open-access manner due to European and UK privacy (GDPR) legislation.

Human research participants

Policy information about studies involving human research participants and Sex and Gender in Research.

Reporting on sex and gender	We utilized data from population cohorts and case-control studies comprising both men and women, and did not perform any sex-specific analyses within this study. The findings from our study apply broadly to both men and women. Self-reported biological sex was used as a covariate in statistical models. The terms "gender mismatch" or "sex mismatch" were used in Supplementary Table 1 to indicate where the biological sex of participants did not match self-reported sex - this was one of the exclusion criteria we applied.
Population characteristics	We utilized data from 10 studies, primarily comprising participants of European ancestry and both men and women. All participants were adults (>=18). Some studies were case-control designs, and so our study included patients with neurodegenerative and neuropsychiatric conditions, rheumatoid arthritis, coronary artery disease, stroke, or atrial fibrillation cases. Details of the participants are summarised in the Supplementary Tables and the Supplementary Note.
Recruitment	Our study utilized summary data from pre-existing studies only, so no new recruitment was performed. As noted above, some studies comprised participants with chronic disease. Where possible, diagnostic categories were included as covariates to minimise confounding.
Ethics oversight	Details of ethics oversight for each of the contributing studies are included in the Supplementary Note.

Note that full information on the approval of the study protocol must also be provided in the manuscript.

Field-specific reporting

Please select the one below that is the best fit for your research. If you are not sure, read the appropriate sections before making your selection.

☒ Life sciences ☐ Behavioural & social sciences ☐ Ecological, evolutionary & environmental sciences

For a reference copy of the document with all sections, see nature.com/documents/nr-reporting-summary-flat.pdf

Life sciences study design

All studies must disclose on these points even when the disclosure is negative.

Sample size	This discovery study was a meta-analysis of pre-existing pQTL GWAS summary statistics, and so the aim was to generate as large a sample as possible given available resources at the time the analyses were conducted. As is typical for a GWAS meta-analysis, no power calculation was performed to inform sample size.
Data exclusions	Each contributing study had their own data exclusions, which included gender/sex mismatches, ethnic outliers, heterozygosity, cryptic relatedness, and low genotype call rates. Duplicates were also detected and removed. Details are provided in the Supplementary Tables.
Replication	We performed replication and validation of our meta-analysis results using an independent cohort of 1,585 participants with pQTL data from the ARISTOTLE study.
Randomization	This is an observational study, rather than a clinical trial, so no randomisation was performed.
Blinding	This was not a clinical trial so there was no requirement for blinding. The nature of pQTL and other GWAS analyses in general render investigators blind to genotypic groups.

Reporting for specific materials, systems and methods

We require information from authors about some types of materials, experimental systems and methods used in many studies. Here, indicate whether each material, system or method listed is relevant to your study. If you are not sure if a list item applies to your research, read the appropriate section before selecting a response.

Materials & experimental systems

n/a	Involved in the study
☒	☐ Antibodies
☒	☐ Eukaryotic cell lines
☒	☐ Palaeontology and archaeology
☒	☐ Animals and other organisms
☒	☐ Clinical data
☒	☐ Dual use research of concern

Methods

n/a	Involved in the study
☒	☐ ChIP-seq
☒	☐ Flow cytometry
☒	☐ MRI-based neuroimaging
